# Comprehensive analysis of correlations among codon usage bias, gene expression, and substitution rate in *Arachis duranensis* and *Arachis ipaënsis* orthologs

**DOI:** 10.1038/s41598-017-13981-1

**Published:** 2017-11-01

**Authors:** Hui Song, Hongjuan Gao, Jing Liu, Pei Tian, Zhibiao Nan

**Affiliations:** State Key Laboratory of Grassland Agro-ecosystems, College of Pastoral Agriculture Science and Technology, Lanzhou University, Lanzhou, 730000 China

**Keywords:** Molecular evolution, Plant evolution

## Abstract

The relationship between evolutionary rates and gene expression in model plant orthologs is well documented. However, little is known about the relationships between gene expression and evolutionary trends in *Arachis* orthologs. We identified 7,435 one-to-one orthologs, including 925 single-copy and 6,510 multiple-copy sequences in *Arachis duranensis* and *Arachis ipaënsis*. Codon usage was stronger for shorter polypeptides, which were encoded by codons with higher GC contents. Highly expressed coding sequences had higher codon usage bias, GC content, and expression breadth. Additionally, expression breadth was positively correlated with polypeptide length, but there was no correlation between gene expression and polypeptide length. Inferred selective pressure was also negatively correlated with both gene expression and expression breadth in all one-to-one orthologs, while positively but non-significantly correlated with gene expression in sequences with signatures of positive selection. Gene expression levels and expression breadth were significantly higher for single-copy genes than for multiple-copy genes. Similarly, the gene expression and expression breadth in sequences with signatures of purifying selection were higher than those of sequences with positive selective signatures. These results indicated that gene expression differed between single-copy and multiple-copy genes as well as sequences with signatures of positive and purifying selection.

## Introduction

Molecular biology and evolution research in the later 20^th^ century revealed that homologous genes can be divided into paralogs and orthologs^1^. Paralogous genes, or paralogs, are derived from sequence duplication events within a single lineage^2^. Paralogs are often free to evolve novel functions because the functional redundancy provided by gene duplicates frees one of the copies from the selective constraint maintaining its function prior to duplication^3^. In contrast, orthologous genes, or orthologs, are distributed among different species that diverged from a single ancestral gene at a speciation event^2^. Accordingly, orthologs typically perform equivalent functions across different species. Therefore, these genes can be used to construct phylogenetic relationships and provide insight into the processes of molecular evolution^2^. For example, data from β-tubulin and translation elongation factor sequences suggest that *Epichloë* species likely originated in Eurasia^4,5^. Similar types of molecular data from orthologs have been used to make comparisons of the evolutionary rates between gymnosperms and angiosperms, revealing lower evolutionary rates among gymnosperms^6^. Yue, *et al*.^7^ found that annual plant species (i.e., *Arabidopsis thaliana* and *Medicago truncatula*) have evolved at higher rates than perennial plant species (i.e., *Populus trichocarpa* and *Vitis vinifera*) using both nuclear and chloroplast genome loci.

Other evolutionary patterns have also revealed themselves in the study of orthologs. For example, highly expressed genes have potentially undergone stronger purifying selection based on their important functional roles that require high transcript levels^2^. The levels and patterns of expression are not only the major determinants that explain nonsynonymous rate variation among genes but also a crucial determinant of gene retention rates after duplication^8^. Recently, Grusz, *et al*.^9^ demonstrated that the faster evolutionary rates characteristic of vittarioid fern lineages are likely not driven by positive selection, nor are they limited to any particular type of nucleotide substitution. Hodgins, *et al*.^10^ found that genes with low expression levels have a lot of neutral substitutions, but rapidly diverging genes tend to have higher expression divergence in conifers. In brief, orthologs are well suited to revealing phylogenetic relationships and understanding the mechanisms shaping gene expression.

Generally, gene expression can be changed by many factors. For example, tissue-specific expression, DNA dosage, tRNA abundance and external environment factors can affect gene expression patterns^11–14^. A growing body of studies has revealed that several gene sequence architecture features, including synonymous codon usage, amino acid composition, coding sequences (CDSs) length, GC content, and intron size, are correlated with expression levels in prokaryotes and eukaryotes^15–17^. Similarly, highly expressed genes are biased towards using optimal codons in the *Chilodonella uncinata* genome^18^. Camiolo, *et al*.^19^ found short and GC-rich CDSs were positively correlated with expression and optimal usage bias in four monocot, fifteen dicot, and two moss species. In *Silene latifolia*, gene expression was positively correlated with third codon position GC content (GC3), but strongly and negatively correlated with intronic GC content^20^. Moreover, gene expression has been shown to be correlated with the size of gene families. Larger multiple-copy gene families exhibit both lower expression levels and breadth than genes in single-copy gene families^17^. In addition, tissue-specific expression was more often observed among genes in multiple-copy gene families than genes in single-copy gene families^17^.

Peanut is a major oil and protein crop. Cultivated peanut (*Arachis hypogaea*) is an allotetraploid species with an AABB genome^21^. Its ancestral species are most likely *Arachis duranensis* and *Arachis ipaënsis*, which contributed the A and B genomes, respectively^22–25^. Recently, the genome sequences of *A*. *duranensis* and *A*. *ipaënsis* have been sequenced, assembled, and released^26^. A comprehensive tissue-specific transcriptome of cultivated peanut has also been released^26,27^. Collectively, this research can be used to understand the relationship between evolutionary trends and gene expression. In this study, we identified orthologs from *A*. *duranensis* and *A*. *ipaënsis*, and used codon usage bias, polypeptide length, GC content, substitution rate, expression breadth, and gene expression level as explanatory variables to evaluate patterns among orthologs in *A*. *duranensis* and *A*. *ipaënsis*. Our study had two key aims: (1) to examine the codon usage pattern and relationship between codon usage bias, expression level, and substitution rate in these one-to-one orthologs and (2) to characterize differences in the evolutionary trends and expression patterns between genes in single-copy gene families and those in multiple-copy gene families from these orthologs. This study provides insight into the evolution and expression of gene families in *Arachis*.

## Results

### One-to-one orthologs

A total of 7,435 one-to-one ortholog pairs were used in this study after the filtering criteria (see Materials and Methods) were applied to the *A*. *duranensis* and *A*. *ipaënsis* genomes (Table S1). These selected gene sequences can be classified into two groups based on the number of genes in their gene families, single-copy gene families (with one gene) and multiple-copy gene families (with more than one gene). We identified 925 single-copy and 6,510 multiple-copy genes in both genomes. Bertioli, *et al*.^26^ found approximately 9,236 sequences belonged to multiple-copy gene families in *A*. *ipaënsis* or *A*. *duranensis*, while 6,357 and 7,253 sequences belonged to single-copy gene families in *A*. *ipaënsis* and *A*. *duranensis*, respectively. Many single-copy genes were detected in *A*. *duranensis* and *A*. *ipaënsis*, indicating these genes originated before the divergence of *A*. *duranensis* and *A*. *ipaënsis* approximately 2.16 million years ago^26^. Although wild-type peanut underwent a whole genome duplication (WGD) event^28^, these single-copy genes have exhibited no changes in number.

### Codon usage

The GC contents of each of the three codon positions were assessed across all identified CDSs as GC1, GC2, and GC3, which correspond to the GC content of the first, second, and third codon positions, respectively (Table S2). The average GC1 was 50.37%, followed by GC3 at 42.95% and GC2 at 40.44% in *A*. *duranensis* CDSs. Similarly, *A*. *ipaënsis* exhibited average GC1, GC3, and GC2 values of 50.37%, 43.01%, and 40.44%, respectively. The average GC contents across the three codons positions of CDSs were 44.59% in *A*. *duranensis* and 44.61% in *A*. *ipaënsis*, indicating that two wild peanuts have high a AT content (i.e., 55.41% in *A*. *duranensis* and 55.39% in *A*. *ipaënsis*) in CDSs. These results are consistent with previous research in eudicots, which found that GC1 content exceeded GC2 content, GC3 content exceeded GC2 content, and AT content exceeded GC content overall^29^. The average frequency of optimal codons (Fop) value was 0.39 in both *A*. *duranensis* and *A*. *ipaënsis*. Fop was negatively correlated with polypeptide length but positively correlated with GC content in *A*. *duranensis* and *A*. *ipaënsis* (Table 1). These results showed that codon usage bias in *A*. *duranensis* and *A*. *ipaënsis* one-to-one orthologs tended towards shorter polypeptides having higher GC contents.

**Table 1:** Codon usage bias in *Arachis duranensis* and *Arachis ipaënsis* one-to-one orthologs.

Fop	Polypeptide length	GC1 content	GC2 content	GC3 content	Overall GC content
*Arachis duranensis*^a^	−0.19**	0.25**	0.28**	0.61**	0.62**
*Arachis ipaënsis*^b^	−0.19**	0.25**	0.28**	0.61**	0.62**
Single-copy gene^c^	−0.72**	0.17**	0.18**	0.58**	0.54**
Multiple-copy gene^d^	−0.18**	0.26**	0.29**	0.62**	0.63**
Positive selection^e^	−0.15	0.34**	0.15**	0.56**	0.56**
Purifying selection^f^	−0.19**	0.25**	0.30**	0.63**	0.64**

^a^Frequency of optimal codons (Fop) in one-to-one orthologs from *Arachis duranensis*; ^b^Fop in one-to-one orthologs from *Arachis ipaënsis*; ^c^Fop in single-copy one-to-one orthologs; ^d^Fop in multiple-copy one-to-one orthologs; ^e^Fop in sequences that have experienced positive selection; ^f^Fop in sequences that have experienced purifying selection. **Indicates significance at *P* < 0.01.

### Gene expression

**Figure 1:**
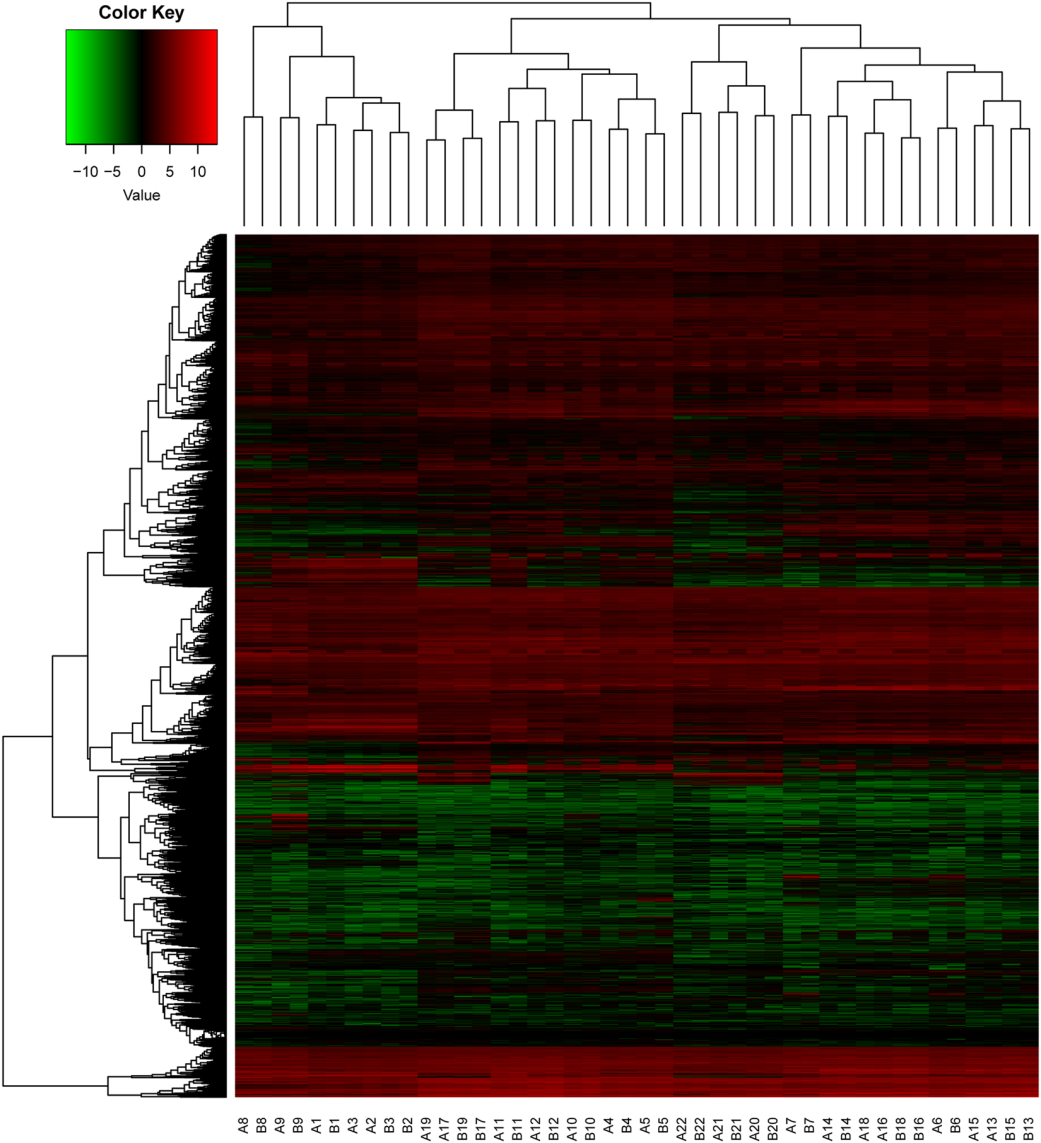
Gene expression level of one-to-one orthologs in 22 tissues in *Arachis duranensis* and *Arachis ipaënsis* based on RNA-seq data. (A1 and B1) seedling leaf 10 d post emergence; (A2 and B2) main stem leaf; (A3 and B3) lateral leaf; (A4 and B4) vegetative shoot tip from the main stem; (A5 and B5) reproductive shoot tip from the first lateral leaf; (A6 and B6) 10 d roots; (A7 and B7) 25 d nodules; (A8 and B8) perianth; (A9 and B9) gynoecium; (A10 and B10) androecium; (A11 and B11) aerial gynophore tip; (A12 and B12) subterranean gynophore tip; (A13 and B13) Pattee 1 pod; (A14 and B14) Pattee 1 stalk; (A15 and B15) Pattee 3 pod; (A16 and B16) Pattee 5 pericarp; (A17 and B17) Pattee 5 seed; (A18 and B18) Pattee 6 pericarp; (A19 and B19) Pattee 6 seed; (A20 and B20) Pattee 7 seed; (A21 and B21) Pattee 8 seed; (A22 and B22) Pattee 10 seed. The FPKM value for each gene in these various tissues was normalized using a log_2_-transformation.

Although gene expression patterns in most tissues were similar between *A*. *duranensis* and *A*. *ipaënsis* one-to-one orthologs, gene expression patterns in main stem leaf, lateral leaf, Pattee 1 (i.e., stage 1) pod, Pattee 3 pod, Pattee 5 pericarp, Pattee 6 pericarp, Pattee 5 seed, and Pattee 6 seed tissues were biased (Fig. 1 and Table S3). For example, gene expression patterns in *A*. *duranensis* and *A*. *ipaënsis* perianth tissue were similar, while gene expression patterns in *A*. *duranensis* main stem leaves and lateral leaves differed from those in *A*. *ipaënsis* (Fig. 1 and Table S3). In addition, expression breadth of 1,873 ortholog pairs differed between *A*. *duranensis* and *A*. *ipaënsis* (Table S4); for example, Aradu.NIH31 and Araip.I19HU were an ortholog pair, while their expression breadth values were 1 and 22, respectively. These results revealed biases in gene expression patterns and expression breadth between *A*. *duranensis* and *A*. *ipaënsis*. Moreover, these results are consistent with those described by Clevenger, *et al*.^27^, who found that 76.8–98.1% of expressed gene pairs exhibited balanced expression, but striking differences in expression bias were exhibited in a tissue-specific context in the two cultivated peanut subgenomes.

**Figure 2:**
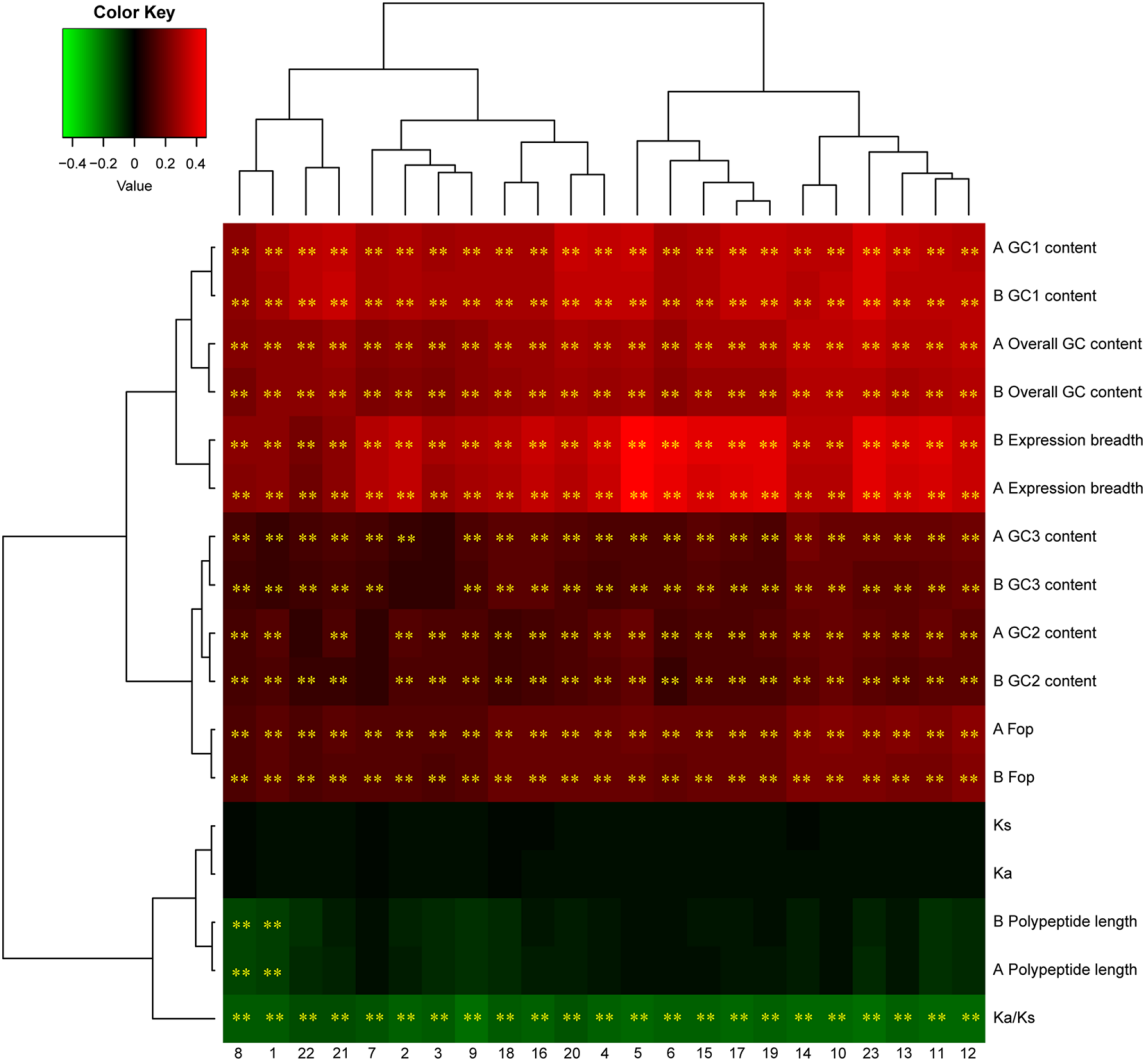
Correlation analyses of gene expression level and various attributes of *Arachis duranensis* and *Arachis ipaënsis* one-to-one orthologs. The analyzed sequence attributes include frequency of optimal codons (Fop), polypeptide length, GC1 content, GC2 content, GC3 content, overall GC content, expression breadth, *K*_*a*_, *K*_*s*_, and *K*_*a*_/*K*_*s*_. (**A** and **B**) *Arachis duranensis* and *Arachis ipaënsis*, respectively. (1) seedling leaf 10 d post emergence; (2) main stem leaf; (3) lateral leaf; (4) vegetative shoot tip from the main stem; (5) reproductive shoot tip from the first lateral leaf; (6) 10 d roots; (7) 25 d nodules; (8) perianth; (9) gynoecium; (10) androecium; (11) aerial gynophore tip; (12) subterranean gynophore tip; (13) Pattee 1 pod; (14) Pattee 1 stalk; (15) Pattee 3 pod; (16) Pattee 5 pericarp; (17) Pattee 5 seed; (18) Pattee 6 pericarp; (19) Pattee 6 seed; (20) Pattee 7 seed; (21) Pattee 8 seed; (22) Pattee 10 seed; (23) average gene expression level. The heat map was constructed based on Pearson correlation coefficients in Table S5. **Indicates significance at *P* < 0.01.

There was a positive correlation between Fop, GC content, expression breadth, and gene expression level, but no correlation between polypeptide length and gene expression level except for negative correlations between gene expression level and both seedling leaf 10 d post emergence and perianth tissues in *A*. *duranensis* and *A*. *ipaënsis* one-to-one orthologs (Fig. 2 and Table S5). Furthermore, we identified correlations between Fop, polypeptide length, GC content, expression breadth, and average gene expression levels among 22 tissues (Fig. 2 and Table S5). Average expression level was positively correlated with Fop, GC content, and expression breadth (Fig. 2 and Table S5). However, there was also no correlation between polypeptide length and average gene expression level. These results indicated that highly expressed one-to-one orthologs with higher GC content have codon usage bias (as demonstrated by Fop) and broad expression breadth in one-to-one orthologs. In addition, expression breadth was positively correlated with polypeptide length, GC1, GC2, and overall GC content in one-to-one orthologs (Table 2). A given gene with broader expression breadth tended to have longer polypeptide lengths and higher GC content, including GC1, GC2, and overall GC content in one-to-one orthologs.

**Table 2:** Correlation analyses of expression breadth and various attributes of *Arachis duranensis* and *Arachis ipaënsis* one-to-one orthologs.

Expression breadth	Fop	Polypeptide length	GC1 content	GC2 content	GC3 content	Overall GC content
*Arachis duranensis*^a^	0.10**	0.15**	0.22**	0.11**	0.07	0.18**
*Arachis ipaënsis*^b^	0.09	0.15**	0.22**	0.10**	0.06	0.17**
Positive selection^c^	0.19*	0.22**	0.08	0.02	0.20*	0.16
Purifying selection^d^	0.09	0.16**	0.23**	0.12**	0.06	0.17**
Single-copy gene^e^	0	0.11**	0.21**	0.07	0	0.11**
Multiple-copy gene^f^	0.10**	0.15**	0.21**	0.11**	0.08	0.18**

^a^expression breadth in one-to-one orthologs from *Arachis duranensis*; ^b^expression breadth in one-to-one orthologs from *Arachis ipaënsis*; ^c^expression breadth in sequences that have experienced positive selection; ^d^expression breadth in sequences that have experienced purifying selection; ^e^expression breadth in single-copy one-to-one orthologs; ^f^expression breadth in multiple-copy one-to-one orthologs. *Indicates significance at *P* < 0.05; **Indicates significance at *P* < 0.01.

### Substitution rate

For a total of 6,732 one-to-one orthologs, *K*_a_ (nonsynonymous per site substitution rate) and *K*_s_ (synonymous per site substitution rate) values were calculated and filtering criteria were applied (Table S6). The average *K*_a_, *K*_s_, and *K*_a_/*K*_s_ values were 0.05, 0.02, and 0.30, respectively. There were 6,598 *K*_a_/*K*_s_ ratio values (nonsynonymous to synonymous per site substitution rate ratios) less than 1, indicating these one-to-one orthologs underwent purifying selection. However, there were 134 *K*_a_/*K*_s_ values greater than 1 (Table S7), indicating positive selection shaped these one-to-one orthologs. The average synonymous substitution rate of the 6,732 one-to-one orthologs was 11.57 × 10^−9^ *K*_s_/year. The average synonymous substitution rate for *Arachis* genes was previously estimated at 8.12 × 10^−9^ *K*_s_/year^26^. The present results thus indicated that the synonymous substitution rate was elevated in 6,732 one-to-one orthologs. Moreover, *K*_a_/*K*_s_ values were negatively correlated with gene expression level (Fig. 2 and Table S5), expression breadth, GC1, GC3, and overall GC content (Table 3). Moreover, *K*_a_ and *K*_s_ values were not correlated with Fop, polypeptide length, GC content, gene expression level, and expression breadth (Fig. 2, Table 3, and Table S5).

**Table 3:** Correlation analyses of substitution rate and various attributes of *Arachis duranensis* and *Arachis ipaënsis* one-to-one orthologs.

	Fop	Polypeptide length	GC1 content	GC2 content	GC3 content	Overall GC content	Expression breadth
*One-to-one orthologs in Arachis duranensis* and *Arachis ipaënsis*							
*K*_*s*_	0	−0.05	−0.03	−0.04	0.03	−0.01	−0.09
*K*_*a*_	−0.01	−0.02	−0.03	−0.02	0	−0.02	−0.04
*K*_*a*_/*K*_*s*_	−0.05	−0.08	−0.16**	−0.05	−0.11**	−0.15**	−0.18**
*One-to-one orthologs in sequences experienced positive selection*							
*K*_*s*_	0	−0.18**	0.15**	−0.10**	−0.01	0.11**	0.20**
*K*_*a*_	0	−0.13**	0.12**	−0.08	0	0.08	0.16**
*K*_*a*_/*K*_*s*_	−0.26**	0.66**	0.26**	0.48**	0.15	−0.12**	−0.43**
*One-to-one orthologs in sequences experienced purifying selection*							
*K*_*s*_	0.01	−0.07	−0.03	−0.05	0.05	0	−0.15**
*K*_*a*_	−0.01	−0.04	−0.05	−0.03	0	−0.03	−0.12**
*K*_*a*_/*K*_*s*_	−0.06	−0.04	−0.17**	−0.06	−0.13**	−0.17**	−0.17**
*One-to-one orthologs in single-copy gene*							
*K*_*s*_	0.01	−0.02	−0.07	−0.05	0.04	−0.02	−0.18**
*K*_*a*_	−0.04	0.04	−0.03	−0.05	−0.04	−0.06	0.02
*K*_*a*_/*K*_*s*_	−0.01	0.01	−0.02	−0.11**	−0.01	−0.06	−0.02
*One-to-one orthologs in multiple-copy gene*							
*K*_*s*_	0	−0.05	−0.02	−0.03	0.03	−0.01	−0.08
*K*_*a*_	0	−0.02	−0.03	−0.02	0	−0.02	−0.04
*K*_*a*_/*K*_*s*_	−0.05	−0.08	−0.16**	−0.05	−0.10**	−0.15**	−0.18**

**Indicates significance at *P* < 0.01.

**Figure 3:**
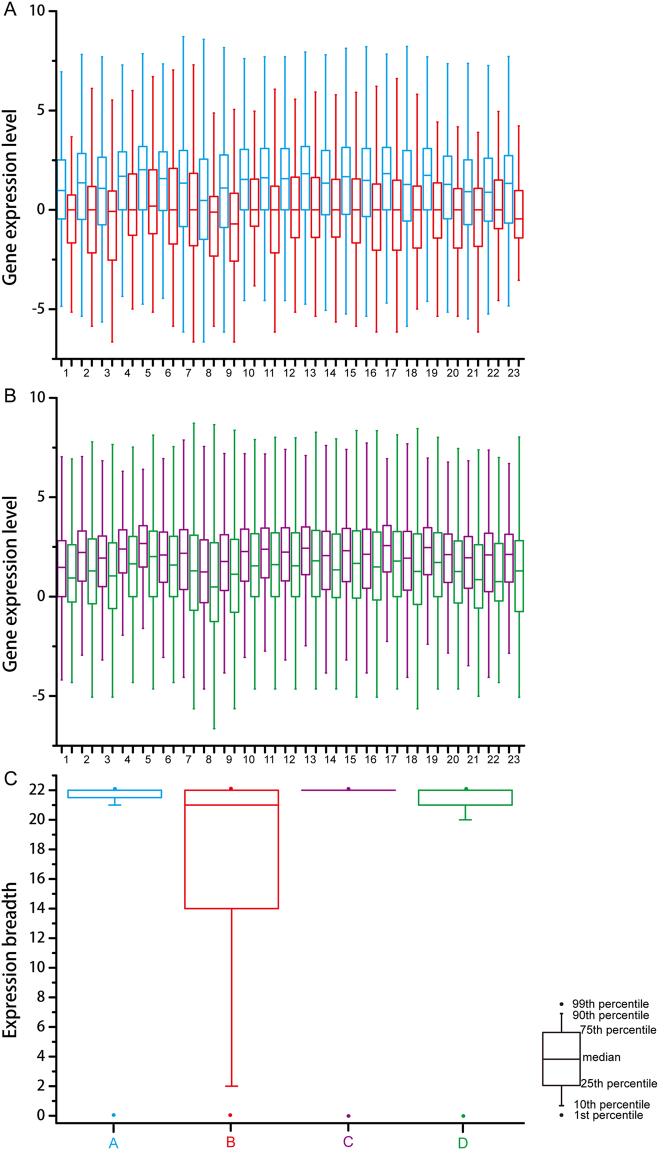
Comparative of gene expression level and expression breadth. (**A**) Gene expression level in sequences that have experienced purifying and positive selection. (**B**) Gene expression level in single-copy and multiple-copy genes. (**C**) Expression breadth in sequences that have experienced purifying and positive selection, and single-copy and multiple-copy genes. Blue (**A**), red (**B**), purple (**C**), and green (**C**) colors indicate sequences that have experienced purifying selection, sequences that have experienced positive selection, single-copy genes and multiple-copy genes, respectively. (1) seedling leaf 10 d post emergence; (2) main stem leaf; (3) lateral leaf; (4) vegetative shoot tip from the main stem; (5) reproductive shoot tip from the first lateral leaf; (6) 10 d root; (7) 25 d nodules; (8) perianth; (9) gynoecium; (10) androecium; (11) aerial gynophore tip; (12) subterranean gynophore tip; (13) Pattee 1 pod; (14) Pattee 1 stalk; (15) Pattee 3 pod; (16) Pattee 5 pericarp; (17) Pattee 5 seed; (18) Pattee 6 pericarp; (19) Pattee 6 seed; (20) Pattee 7 seed; (21) Pattee 8 seed; (22) Pattee 10 seed; (23) average gene expression level.

**Figure 4:**
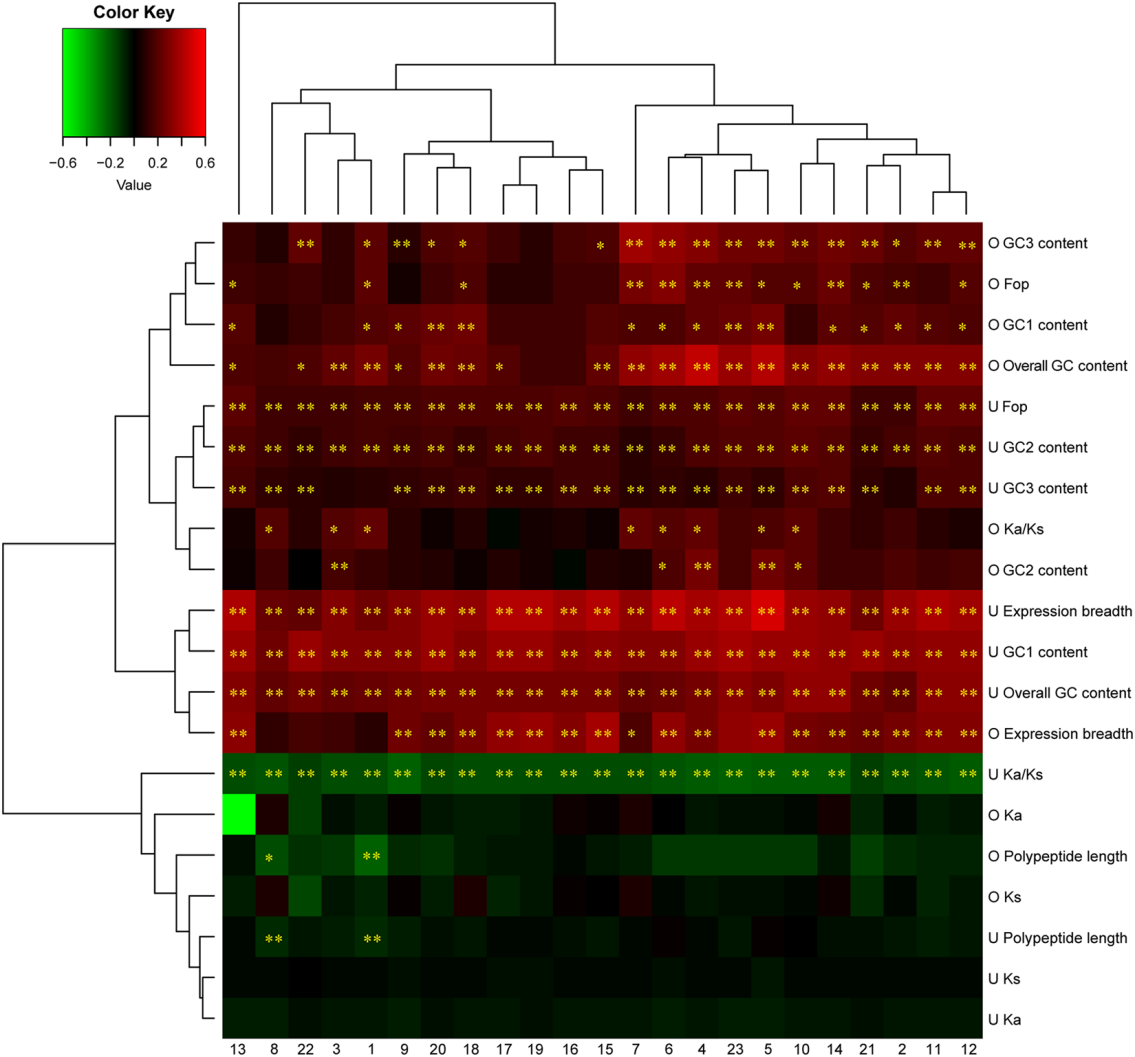
Correlation analyses of gene expression level and various attributes in sequences that have experienced positive and purifying selection. The analyzed sequence attributes include frequency of optimal codons (Fop), polypeptide length, GC1 content, GC2 content, GC3 content, overall GC content, expression breadth, *K*_*a*_, *K*_*s*_, and *K*_*a*_/*K*_*s*_. (U and O) Sequences that have experienced positive and purifying selection, respectively. (1) seedling leaf 10 d post emergence; (2) main stem leaf; (3) lateral leaf; (4) vegetative shoot tip from the main stem; (5) reproductive shoot tip from the first lateral leaf; (6) 10 d roots; (7) 25 d nodules; (8) perianth; (9) gynoecium; (10) androecium; (11) aerial gynophore tip; (12) subterranean gynophore tip; (13) Pattee 1 pod; (14) Pattee 1 stalk; (15) Pattee 3 pod; (16) Pattee 5 pericarp; (17) Pattee 5 seed; (18) Pattee 6 pericarp; (19) Pattee 6 seed; (20) Pattee 7 seed; (21) Pattee 8 seed; (22) Pattee 10 seed; (23) average gene expression level. The heat map was constructed based on Pearson correlation coefficients in Table S7. *Indicates significance at *P* < 0.05; **Indicates significance at *P* < 0.01.

Gene expression level and expression breadth of gene sequences that experienced purifying selection were significantly higher than those in gene sequences that have been shaped by positive selection (Mann–Whitney U test, *P* < 0.01; Fig. 3). In the two groups, Fop was negatively correlated with polypeptide length, but positively correlated with GC content (Table 1). However, there were inconsistent correlations between gene expression level from 22 tissues and variables including Fop, polypeptide length, GC content, expression breadth, *K*_a_, *K*_s_, and *K*_a_/*K*_s_; the average gene expression level was positively correlated with Fop, GC1 content, GC3 content, overall GC content, and expression breadth, while no correlation was observed between average gene expression level and both *K*_*a*_ and *K*_*s*_ values in the two groups (Fig. 4 and Table S8). It should be noted that *K*_a_/*K*_s_ was positively and non-significantly correlated with average gene expression level (*r* = 0.16, *P* > 0.05) in genes under positive selection, but negatively and significantly correlated with average gene expression level (*r* = −0.22, *P* < 0.01) in genes under purifying selection (Fig. 4 and Table S8). Moreover, expression breadth was positively correlated with polypeptide length in positive and negative groups (Table 2). However, expression breadth was positively correlated with Fop and GC3 content in genes under positive selection, and positively correlated with GC1 content, GC2 content, and overall GC content in genes under purifying selection (Table 2).

Among the genes under positive selection, *K*_a_/*K*_s_ values were negatively correlated with Fop, overall GC content, and expression breadth, but positively correlated with polypeptide length, GC1 content, and GC2 content (Table 3). However, *K*_a_/*K*_s_ values were negatively correlated with GC1 content, GC3 content, overall GC content, and expression breadth among genes under purifying selection (Table 3). The *K*_a_ and *K*_s_ values were negatively correlated with polypeptide length, but positively correlated with GC1 content and expression breadth in the positive selection group, respectively. Moreover, positive correlations were exhibited between the *K*_s_ value and GC1 content, overall GC content, and expression breadth among the genes under positive selection (Table 3). However, *K*_a_ and *K*_s_ values were only negatively correlated with expression breadth in the group of genes under purifying selection (Table 3). Overall, correlations differed both between genes under positive and purifying selection in a pattern that was potentially consistent with differences in synonymous and nonsynonymous substitution rates.

### Comparison of single-copy and multiple-copy gene families

To understand differences in gene expression levels and evolutionary trends between single-copy and multiple-copy gene families, we first compared gene expression levels and expression breadth between single-copy and multiple-copy gene families in *A*. *duranensis* and *A*. *ipaënsis*. We found that gene expression levels and expression breadth were significantly higher in single-copy gene families than those in multiple-copy gene families (Mann–Whitney U test, *P* < 0.01; Fig. 3). Further, Fop was negatively correlated with polypeptide length in multiple-copy gene families, and positively correlated with GC content in single-copy and multiple-copy gene families (Table 1).

**Figure 5:**
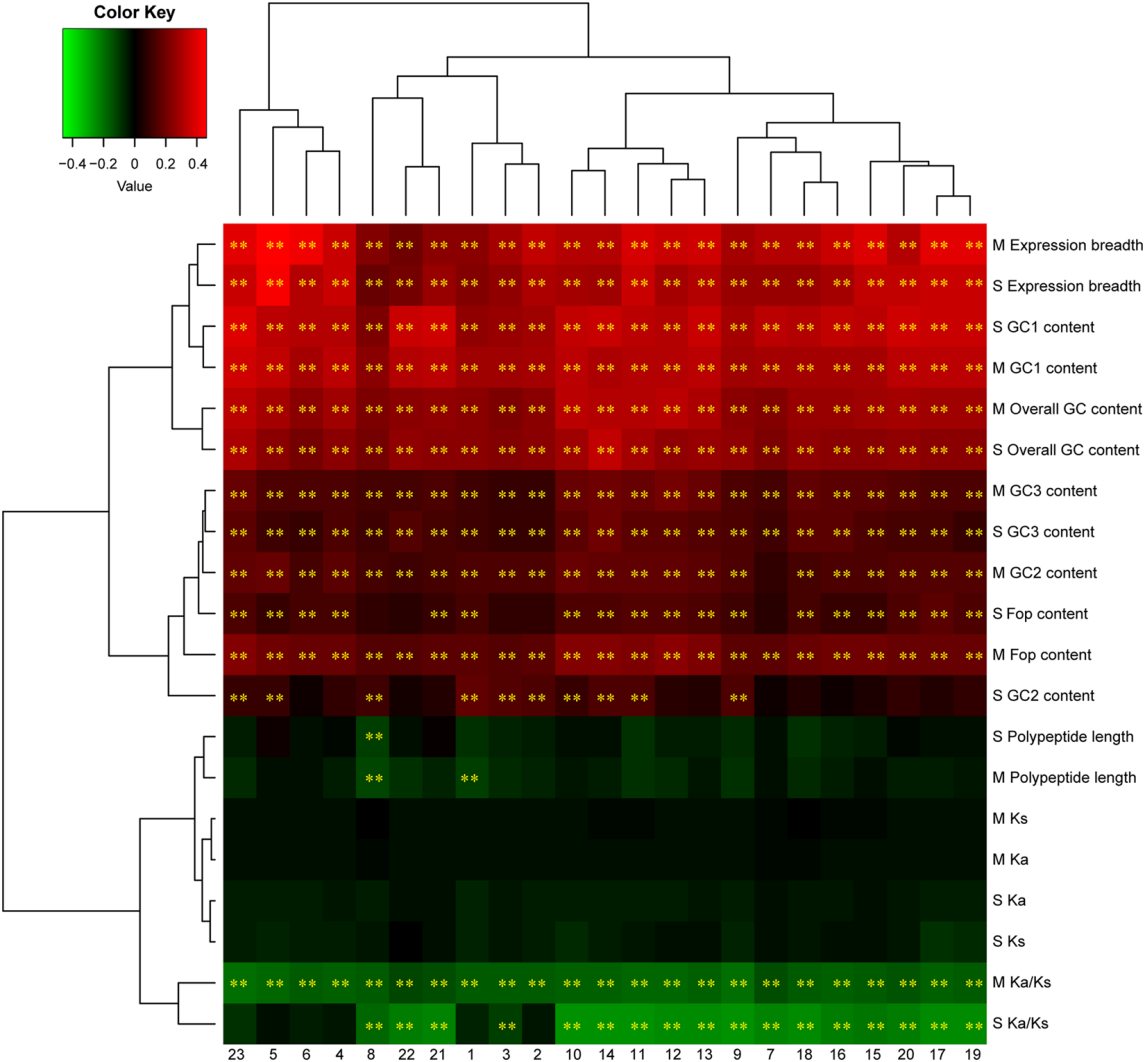
Correlation analyses of gene expression level and various attributes of single-copy and multiple-copy genes. The analyzed sequence attributes include frequency of optimal codons (Fop), polypeptide length, GC1 content, GC2 content, GC3 content, overall GC content, expression breadth, *K*_*a*_, *K*_*s*_, and *K*_*a*_/*K*_*s*_. (S and M) Single-copy and multiple-copy genes, respectively. (1) seedling leaf 10 d post emergence; (2) main stem leaf; (3) lateral leaf; (4) vegetative shoot tip from the main stem; (5) reproductive shoot tip from the first lateral leaf; (6) 10 d roots; (7) 25 d nodules; (8) perianth; (9) gynoecium; (10) androecium; (11) aerial gynophore tip; (12) subterranean gynophore tip; (13) Pattee 1 pod; (14) Pattee 1 stalk; (15) Pattee 3 pod; (16) Pattee 5 pericarp; (17) Pattee 5 seed; (18) Pattee 6 pericarp; (19) Pattee 6 seed; (20) Pattee 7 seed; (21) Pattee 8 seed; (22) Pattee 10 seed; (23) average gene expression level. The heat map was constructed based on Pearson correlation coefficients in Table S8. **Indicates significance at *P* < 0.01.

Although the gene expression level of some tissues was not correlated with Fop and GC content, average gene expression level was positively correlated with Fop and GC content in single-copy and multiple-copy gene families (Fig. 5 and Table S9). In addition, the gene expression level among 22 tissues was positively correlated with expression breadth in single-copy and multiple-copy gene families, but negatively correlated with *K*_a_/*K*_s_ value in multiple-copy gene families. There was no correlation between polypeptide length, *K*_a_, *K*_s_, and gene expression level in single-copy and multiple-copy gene families. Expression breadth was positively correlated with polypeptide length, overall GC content, and GC1 content among the single-copy and multiple-copy gene families (Table 2). However, expression breadth was positively correlated with Fop and GC2 content in multiple-copy gene families (Table 2). Moreover, *K*_s_ was negatively correlated with expression breadth, and *K*_a_/*K*_s_ was negatively correlated with GC2 content in single-copy gene families (Table 3). However, *K*_a_/*K*_s_ values were negatively correlated with GC1, GC3, overall GC content, and expression breadth in multiple-copy gene families (Table 3).

## Discussion

The *A*. *duranensis* and *A*. *ipaënsis* genome sequences were released in 2015. Bertioli, *et al*.^26^ found most genes had a one-to-one correspondence between the two species using both full-length and partial sequence alignment methods. In cultivated peanut, Clevenger, *et al*.^27^ identified 8,816 full-length homologs using reciprocal BLAST. In the present study, we report that 7,435 full-length sequences are one-to-one ortholog pairs in *A*. *duranensis* and *A*. *ipaënsis* using local BLAST. The number of one-to-one orthologs determined by a previous study was larger than that identified in our study because we excluded partial sequences and genes with unknown functions. Accordingly, many one-to-one ortholog pairs were possibly excluded in this study based on our screening strategies. Although it is popular to use OrthoMCL to identify orthologs, misinterpretation of the data is possible, and the number of orthologs can therefore be controlled by setting an inflation parameter^30,31^. Future studies may improve upon this method to identify more one-to-one orthologs.

Previous studies have shown that orthologs that experienced positive selective pressure are involved in abiotic or biotic stress resistance^17,32^. However, we found most one-to-one orthologs that experienced positive selective pressures play a role in binding, photosynthesis, and other pathways, rather than resistance to stresses (Table S7). Nevertheless, four *K*_a_/*K*_s_ values from toll/mammalian interleukin-1 receptor (TIR)–nucleotide-binding site–leucine-rich repeat (NBS–LRR) (*TNL*) genes exceeded 1. *TNL* belongs to the NBS–LRR gene family, which is associated with disease resistance^33^. Song, *et al*.^34^ determined that paralogous genes mainly underwent purifying selection in *A*. *duranensis* and *A*. *ipaënsis*. These results suggested that the biological function of NBS-LRR differed between *A*. *duranensis* and *A*. *ipaënsis*. Recently, Michelotto, *et al*.^35^ demonstrated that A-type wild peanut is more resistant to disease than B-type wild peanut. Similarly, Pandey, *et al*.^36^ found that A-type wild peanut had more resistance genes than B-type wild peanut based on the number of quantitative trait loci.

Previous studies have demonstrated that synonymous substitution rates in herbaceous lineages are higher than those in woody relatives^7,37^. One of the major factors underlying this difference is the shorter generation times of herbaceous lineages. Annual plants reach their first flowering more quickly than perennials, and thus they, on average, experience more frequent cell divisions per unit time prior to reproduction^7^. The average synonymous substitution rate in *Arachis* genes was similar to that in *Medicago*, though higher than that in *Lotus*, *Glycine*, and *Phaseolus*^26^. Here, the average synonymous substitution rate of 6,732 one-to-one orthologs (11.57 × 10^−9^ *K*_s_/year) was higher than that among *Arachis* genes (8.12 × 10^−9^ *K*_s_/year^26^), indicating one-to-one orthologs play a crucial role in sustaining biological functions. *Arachis* species originated in the high elevations of South America^26^, where UV radiation is relatively strong. Accordingly, plants under these conditions have higher synonymous substitution rates, consistent with an elevated rate of damage repair from pyrimidine dimers. Therefore, the elevated synonymous substitution rates of orthologs in *Arachis* may actually be an outcome of adaptive evolution. In addition, a higher substitution rate is considered a stronger and more important overall evolutionary force than positive selection in CDSs^38,39^.

Selection appeared to increase the efficiency and accuracy of transcription and translation, as supported by the positive correlation between codon usage bias (Fop) and gene expression level in *A*. *duranensis* and *A*. *ipaënsis* among one-to-one orthologs. This positive correlation between codon usage bias and gene expression has been reported in some plants previously, including *Populus tremula*^40^, *Silene latifolia*^20^, *Picea* spp.^17^, and *Cardamine* spp.^41^. On the other hand, codon usage bias (as demonstrated by Fop) was negatively correlated with polypeptide length, but positively correlated with GC content in this study. Similar results were derived from the genomes of four monocots, fifteen dicots, and two mosses described by Camiolo, *et al*.^19^, confirming that short and higher-GC DNA sequences exhibit relatively high levels of expression and optimal usage bias. Similarly, Ingvarsson^40^ showed Fop values were negatively correlated with protein lengths, but strongly and positively correlated with GC3 content in *Populus tremula*. In rice, Wang and Hickey^42^ found that codon usage bias was negatively correlated with gene length, and short genes contained high GC content compared to long genes.

In this study, highly expressed genes were subjected to stronger selective constraint than genes with low expression levels based on the negative correlation between selection pressure and both gene expression and expression breadth in *A*. *duranensis* and *A*. *ipaënsis* one-to-one orthologs. These results strongly support an expression-rate of sequence evolution anticorrelation model (E-R anticorrelation)^43^. This model can be explained by at least four hypotheses, including the expression cost hypothesis, the protein misfolding avoidance hypothesis, the protein misinteraction avoidance hypothesis, and the mRNA folding requirement hypothesis^44^. The expression cost hypothesis proposes that the optimal expression level of a gene corresponds to a trade-off between the benefits and costs associated with its expression^45,46^. The protein misfolding avoidance hypothesis asserts that protein misfolding is cytotoxic and thus reduces fitness^47^. However, Yang, *et al*.^48^ proposed that natural selection on traits other than misfolding avoidance against protein–protein misinteraction, which wastes functional molecules and is potentially toxic, constrains the evolution of surface residues. Park, *et al*.^49^ considered that selection for mRNA folding can impact the nonsynonymous-to-synonymous nucleotide substitution rate ratio, requiring a revision of the current interpretation of this ratio as a measure of protein-level selection. However, there is a positive but nonsignificant correlation between selective pressure and average gene expression in sequences experiencing positive pressure. Although the current study does not immediately suggest a reasonable explanation, the fitness of plants may increase more through the production of new biological functions than by avoiding misfolded proteins. Accordingly, subfunctionalization and neofunctionalization may lead to higher levels of gene expression that increase fitness.

The gene expression and expression breadth of single-copy gene family sequences were significantly higher than those in multiple-copy gene family sequences. The same result was observed in conifers^10,17^ and flowering plants^8,31^. Single-copy genes and their expression patterns have been shown to evolve more slowly than genes in multiple-copy genes^31,50^. These previous results were consistence with our result showing that gene expression levels and expression breadth in sequences that experienced purifying selection exceeded those in sequences experiencing positive selection.

Our results clarify the relationship between gene expression level and molecular evolution in *A*. *duranensis* and *A*. *ipaënsis* one-to-one orthologs using transcriptome data. The same codon usage bias was detected among all one-to-one orthologs. Additionally, gene expression and expression breadth were significantly higher in single-copy gene families than in multiple-copy gene families. Similarly, the gene expression and expression breadth in sequences that had experienced purifying selection were higher than those in sequences that had experienced positive selection. In addition, our results demonstrated that selective pressure was negatively correlated with gene expression and expression breadth in all one-to-one orthologs, while positively but not significantly correlated with gene expression in sequences that had experienced positive selection. This study provides the foundation for further research on gene expression and evolution in *Arachis*.

## Materials and Methods

### Sequence retrieval and expression data collection

The *A*. *duranensis* and *A*. *ipaënsis* CDSs were downloaded from PeanutBase (http://peanutbase.org/download)^26^. To avoid including partial sequences in these analyses, the following evaluation criteria were adopted: (1) CDSs were required to start with an ATG codon and end in TAA, TAG, or TGA codons and (2) CDSs were required to lack premature termination codons or ambiguous codons. Functional annotation of each gene was described by Bertioli, *et al*.^26^. Genes with unknown functions were excluded in the present study. Gene families were classified as either single-copy gene families (i.e., consisting of one gene) or multiple-copy gene families (i.e., consisting of more than one gene) based on gene number.

RNA-seq data derived from various tissues in cultivated peanut have also been released in PeanutBase^27^. We specifically collected RNA-seq data generated from leaf, shoot, root, nodule, perianth, gynoecium, androecium, gynophores, pod, pericarp, and seed tissues. All details of the sequencing, de novo transcriptome assembly, and expression level evaluation were described by Clevenger, *et al*.^27^. Briefly, cultivated peanuts (cultivar ‘Tifrunner’) were grown in a greenhouse (maintained at 24–30 °C). All tissues were harvested at 14:00 except for flower samples, which were collected at 8:30. Three biological replicates of each tissue were sampled from three different plants. The total RNA of each pooled tissue sample was extracted. TruSeq RNA Sample Preparation v2 kits were used for library construction, and paired-end 2 × 100 bp sequencing was conducted using an Illumina HiSeq. 2500 instrument with a total of 209 cycles of TruSeq Rapid SBS Kit v1 (Illumina, San Diego, CA, USA) chemistry. Second, an *in silico* amphidiploid genome was created by simply disregarding scaffolds and concatenating the *A*. *duranensis* genome assembly with the *A*. *ipaënsis* genome assembly and labeling each simply as corresponding to the “A” and “B” genomes, respectively. Once the reads were mapped, the SAM file was run through the genome-guided pipeline. Third, total reads were mapped to the transcript assembly from 58 libraries (consisting of samples from 22 distinct tissue types and developmental stages including vegetative and seed stages) using Bowtie, allowing two mismatches within any particular 25-bp seed. Fragments per kilobase per million reads mapped (FPKM) were estimated using RSEM^51^ for each library. When reads mapped to multiple transcripts, RSEM fractionates the read count among the transcripts so read counts are not integers. Transcripts that had less than 1 FPKM in all 58 libraries were filtered out using the Trinity package, because they were deemed to lack sufficient minimum read coverage. The FPKM values for each gene were distinguished for both the A (*A*. *duranensis*) and B (*A*. *ipaënsis*) genomes from cultivated peanut. The orthologs were identified between cultivated peanut and its diploid ancestors (*A*. *duranensis* [A genome] and *A*. *ipaënsis* [B genome] available from http://peanutbase.org/gene_expression/atlas). The FPKM value for each gene in these various tissues was normalized using a log_2_-transformation for both the A and B genomes. The heat maps in Figs 1, 2, 4 and 5 were generated in R using the heatmap.2 function available in the gplots CRAN library package. Expression breadth, defined as the number of tissues in which a gene is expressed (FPKM value > 0), was estimated in *A*. *duranensis* and *A*. *ipaënsis*, respectively.

### Identification of orthologs

We used *A*. *duranensis* CDSs as a query for comparison with *A*. *ipaënsis* CDSs using local BLAST, and vice versa. The following evaluation criteria were used as thresholds prior to inclusion of CDSs in subsequent analyses^27^: (1) alignment exceeding 80% of the length of the longer sequence, (2) identity > 80%, and (3) E-value ≤ 10^−10^. These pairs were also excluded if one CDS matched more than one hit or was annotated to have an unknown function.

MAFFT^52^ was used to align ortholog pairs. PAL2NAL^53^ was used to convert protein sequences into their corresponding nucleotide sequences. PAML 4.0 ^54^ was used to calculate the *K*_a_/*K*_s_ (nonsynonymous to synonymous per site substitution rates) ratio. Ortholog pairs with *K*_s_ < 0.01 were excluded because low sequence divergence could result in unreliable estimates. In addition, as *K*_a_ approaches 0, the *K*_a_/*K*_s_ value becomes essentially a constant. Hence, we excluded ortholog pairs with K_a_/K_s_ < 0.001. Generally, *K*_a_/*K*_s_ = 1, *K*_a_/*K*_s_ > 1, and *K*_a_/*K*_s_ < 1 indicated neutral, positive, and purifying selection, respectively. Absolute rates of substitution at synonymous sites, *u*, were calculated in pairwise comparisons using the formula *u* = *d*/2 T, where *d* is the synonymous substitution rate and T is 2.16 million years, corresponding to the estimated divergence time between *A*. *duranensis* and *A*. *ipaënsis*^26^.

### Calculation of codon usage bias

Codon bias, measured as the frequency of optimal codons (Fop), and polypeptide length were estimated using CodonW (version 1.4, http://codonw.sourceforge.net). For a gene with extreme codon bias, Fop equals 1, while for a gene with random codon usage, Fop equals 0 ^55^. GC content was also calculated using an in-house Perl script.

### Statistical analysis

A Mann–Whitney U test was used to make comparisons of all variables between single-copy and multiple-copy genes as well as between sequences in the positive and purifying groups. Correlations were assessed among all variables estimated from genes, including Fop, GC content, polypeptide length, substitution rate, gene expression level, and expression breadth. A one-way ANOVA test was performed, and *P*-values of less than 0.05 were considered significant in the correlation analyses. All analyses were conducted in JMP 9.0 (SAS Institute, Inc., Cary, NC, USA). Pearson correlation coefficients indicated the strength of the correlation between two variables, with the strongest relationships having the highest correlation coefficients. However, the strengths of these correlation coefficients are typically low in molecular biology data sets because of the high number of factors influencing these large data sets. In this study, we inferred there was no correlation if the correlation coefficient was less than 0.1 based on findings from previous studies^40,56^.

## Electronic supplementary material

Supplementary materials
