# Author Correction: Comprehensive analysis of correlations among codon usage bias, gene expression, and substitution rate in *Arachis duranensis* and *Arachis ipaënsis* orthologs

**DOI:** 10.1038/s41598-020-60582-6

**Published:** 2020-02-27

**Authors:** Hui Song, Hongjuan Gao, Jing Liu, Pei Tian, Zhibiao Nan

**Affiliations:** State Key Laboratory of Grassland Agro-ecosystems, College of Pastoral Agriculture Science and Technology, Lanzhou University, Lanzhou, 730000 China

Correction to: *Scientific Reports* 10.1038/s41598-017-13981-1, published online 01 November 2017

The original version of this Article contained a typographical error in the spelling of the author Hongjuan Gao, which was incorrectly given as Hongjun Gao.

In addition, the original version of this Article contained a typographical error in the Materials and Methods section under the subheading ‘Identification of orthologs’,

“Hence, we excluded ortholog pairs with *K*_a_/*K*_s_ > 0.001.”

now reads:

“Hence, we excluded ortholog pairs with K_a_/K_s_ < 0.001.”

These errors have now been corrected in the PDF and HTML versions of the Article, and in the accompanying Supplementary materials file.

