# Supplementary material for: Comprehensive analysis of correlations among codon usage bias, gene expression, and substitution rate in Arachis duranensis and Arachis ipaënsis orthologs
Source: Sci Rep. 2017 Nov 1;7:14853. doi: 10.1038/s41598-017-13981-1 (PMC5665869; doi:10.1038/s41598-017-13981-1)
Supplement: Supplementary file 1 — Supplementary materials [file 41598_2017_13981_MOESM1_ESM.pdf]

Comprehensive analysis of correlations among codon usage bias, gene expression, and substitution rate in *Arachis duranensis* and *Arachis ipaënsis* orthologs

Hui Song*, Hongjuan Gao, Jing Liu, Pei Tian, Zhibiao Nan*

State Key Laboratory of Grassland Agro-ecosystems, College of Pastoral Agriculture Science and Technology, Lanzhou University, Lanzhou, 730000, China

*Correspondence: biosonghui@outlook.com; zhibiao@lzu.edu.cn

Table S1 The one-to-one orthologs in *Arachis duranensis* and *Arachis ipaënsis*

Arachis duranensis	*Arachis ipaënsis*	Style of gene family	Annotation
Aradu.V5SB4	Araip.UJ9UQ	single-copy	(2R)-phospho-3-sulfolactatesynthaseComA
Aradu.PEP5T	Araip.RW6GJ	single-copy	(Dimethylallyl)adenosinetRNAmethylthiotransferaseMiaB
Aradu.M4JP1	Araip.IJ0FX	single-copy	15-cis-zeta-caroteneisomerase
Aradu.YSG0U	Araip.93JWP	single-copy	15kDaselenoprotein,putative
Aradu.5Y1RP	Araip.H7632	single-copy	1-phosphatidylinositolphosphodiesterase-likeprotein
Aradu.U80Z7	Araip.JTN8C	single-copy	2-dehydro-3-deoxyphosphooctonatealdolase
Aradu.AY7EP	Araip.8JP1D	single-copy	2Fe-2Siron-sulfurclusterbindingdomainproteinn=1Tax=Sp
Aradu.YPY6M	Araip.G0KQK	single-copy	2Fe-2Siron-sulfurcluster-bindingdomainprotein
Aradu.461JS	Araip.YN7KH	single-copy	2-hydroxyacyl-CoAlyase-like[Glycinemax]
Aradu.TU51C	Araip.L9MEB	single-copy	2-nitropropanedioxygenase-likeprotein
Aradu.NG2JA	Araip.UCT63	single-copy	2OG-Fe(II)oxygenasefamilyoxidoreductase
Aradu.E1B1W	Araip.51M35	single-copy	3'(2'),5'-bisphosphatenucleotidase
Aradu.WKA92	Araip.V59P2	single-copy	3'-5'exonucleasedomain-containingprotein
Aradu.KI06N	Araip.DRP4T	single-copy	3-deoxy-7-phosphoheptulonatesynthase(Phospho-2-deh
Aradu.R63W9	Araip.1R84J	single-copy	3-deoxy-D-manno-octulosonic-acidtransferase
Aradu.M707Y	Araip.IQE81	single-copy	3-hydroxyacyl-CoAdehydrogenaseNAD-bindingprotein,pu
Aradu.GX867	Araip.FB68F	single-copy	3-hydroxyisobutyratedehydrogenase
Aradu.MRW76	Araip.2MA0U	single-copy	3-oxoacyl-(acyl-carrier)reductase
Aradu.V220X	Araip.0HK7I	single-copy	3-oxo-delta(4,5)-steroid5-beta-reductase-likeprotein
Aradu.70V2V	Araip.98G5D	single-copy	3-phosphoinositide-dependentproteinkinase-1,putative
Aradu.EHC6P	Araip.S4QWE	single-copy	3-phosphoshikimate1-carboxyvinyltransferase
Aradu.FXP12	Araip.2U0RL	single-copy	4-hydroxyphenylpyruvatedioxygenase
Aradu.G40TW	Araip.0YN4A	single-copy	5'-3'exonucleasefamilyprotein
Aradu.93WPA	Araip.X0TV2	single-copy	5'-3'exoribonuclease3
Aradu.2B9FT	Araip.LL9X6	single-copy	5'-AMP-activatedproteinkinase-related
Aradu.KP13G	Araip.TNU5T	single-copy	5-formyltetrahydrofolatecyclo-ligase
Aradu.NKE3U	Araip.XM4LL	single-copy	5-methyltetrahydropteroyltriglutamate--homocysteinemet
Aradu.YDH5Z	Araip.8ZP6J	single-copy	5-methylthioadenosine/S-adenosylhomocysteinedeamina
Aradu.1M0CG	Araip.AL63T	single-copy	63kDainnermembranefamilyprotein
Aradu.HL6TS	Araip.EB319	single-copy	6-phosphofructo-2-kinase/fructose-2,6-bisphosphatase-li
Aradu.8A5JB	Araip.QU06Y	single-copy	6-phosphogluconatedehydrogenasefamilyprotein
Aradu.720N0	Araip.QP9W2	single-copy	7-dehydrocholesterolreductase-likeprotein
Aradu.X9ZB3	Araip.P5GSM	single-copy	8-amino-7-oxononanoatesynthase-likeprotein
Aradu.TL6UB	Araip.YJF57	single-copy	AAA-typeATPasefamilyprotein
Aradu.PRD5G	Araip.30QH3	single-copy	ABCCsubfamilyATP-bindingcassetteproteinn=4Tax=Vitis
Aradu.0Q16W	Araip.59D2H	single-copy	acclimationofphotosynthesistoenvironment
Aradu.JZ25F	Araip.23QAA	single-copy	aceousRNaseP2-like[Glycinemax]
Aradu.TW9EH	Araip.B0FC3	single-copy	actin-bindingcalponin-like(CH)domainprotein
Aradu.99GQI	Araip.PM79Q	single-copy	activatingsignalcointegrator1-like[Glycinemax]
Aradu.M2MY1	Araip.25NFE	single-copy	activatorof90kDaheatshockproteinATPasehomolog[Glyci

Aradu.3X2EP	Araip.EX8PA	single-copy	acyl-proteinthioesterase
Aradu.85GH9	Araip.L38X1	single-copy	Adaptor-relatedproteincomplex1,beta1subunitn=34Tax=A
Aradu.970IC	Araip.15B33	single-copy	adenosine/AMPdeaminase
Aradu.CJQ6L	Araip.B90DI	single-copy	adenosinekinase2
Aradu.AT7BU	Araip.6S3M7	single-copy	Adenosylmethioninedecarboxylasefamilyprotein
Aradu.W9VU7	Araip.VF78K	single-copy	adenylosuccinatelyase
Aradu.T9PKV	Araip.891PE	single-copy	adenylyl-sulfatekinase3-likeisoformX5[Glycinemax]
Aradu.3B2AS	Araip.F2WNQ	single-copy	adipocyteplasmamembrane-associated-likeprotein
Aradu.06JB2	Araip.BAZ0W	single-copy	Afadin/alpha-actinin-bindingprotein
Aradu.GHV9J	Araip.47BGG	single-copy	Agmatinedeiminasen=1Tax=Pseudomonassp.(strainM1)R
Aradu.9C32Y	Araip.GM4CV	single-copy	AIG2-like(avirulenceinducedgene)familyprotein
Aradu.ZE3IA	Araip.02P6R	single-copy	alanine:glyoxylateaminotransferase2
Aradu.RFT1Y	Araip.CN7HI	single-copy	AlkylhydroperoxidereductaseThiolspecificantioxidantMala
Aradu.ECK51	Araip.0NL51	single-copy	allantoateamidohydrolase
Aradu.4S94M	Araip.34D6V	single-copy	alpha-(1,4)-fucosyltransferase[Glycinemax]
Aradu.WB4HU	Araip.R7MQK	single-copy	alpha1,4-glycosyltransferasefamilyprotein
Aradu.Y3QBI	Araip.0W11Z	single-copy	alphadioxygenase
Aradu.4118A	Araip.X6A1T	single-copy	alpha-glucosidase
Aradu.D85KR	Araip.21M98	single-copy	alpha-L-fucosidase1
Aradu.ZUY8A	Araip.SI9K6	single-copy	alpha-solubleNSFattachmentprotein2
Aradu.X03Q4	Araip.PD52B	single-copy	ALUMINUMSENSITIVE3-like[Glycinemax]
Aradu.C5CLY	Araip.7F1V2	single-copy	ALWAYSEARLY3-likeisoformX3[Glycinemax]
Aradu.V33RZ	Araip.2W24M	single-copy	amidophosphoribosyltransferase1,chloroplastic-like[Glyci
Aradu.TB51Z	Araip.IAX0I	single-copy	aminoalcoholphosphotransferase1
Aradu.F2XPJ	Araip.6H93L	single-copy	Aminotransferase-like,plantmobiledomainfamilyprotein
Aradu.JC32L	Araip.UXF7P	single-copy	AMSH-likeubiquitinthioesterase1-like[Glycinemax]
Aradu.93R3L	Araip.W6HJ2	single-copy	anaerobicribonucleosidetriphosphatereductasen=1Tax=S
Aradu.T9MDP	Araip.1I30Q	single-copy	anion-transportingATPasen=1Tax=cyanobacteriumPCC7
Aradu.SJ7I8	Araip.26QE5	single-copy	anoctamin-likeproteinAt1g73020-likeisoformX1[Glycinem
Aradu.N7TR4	Araip.R1VPM	single-copy	anthocyanin5-aromaticacyltransferase-like[Glycinemax]
Aradu.A9U3A	Araip.6787A	single-copy	anticodon-bindingdomainprotein
Aradu.X0F96	Araip.3V9D5	single-copy	AP-5complexsubunitzeta-likeprotein
Aradu.48GKA	Araip.42YVV	single-copy	apoptoticchromatincondensationinducerinthenucleus-like
Aradu.SBU2D	Araip.8LI4K	single-copy	appr-1-pprocessingenzymefamilyprotein
Aradu.GNT02	Araip.Y5PBQ	single-copy	arabinogalactanprotein
Aradu.T2X3V	Araip.5JD7H	single-copy	arabinosekinase
Aradu.14D5A	Araip.1SI6Z	single-copy	Arginine-aspartate-richRNAbindingprotein-liken=6Tax=Br
Aradu.U5M8T	Araip.61LLE	single-copy	argininebiosynthesisproteinArgJfamily
Aradu.22ZEB	Araip.AP8LU	single-copy	argininedecarboxylase2
Aradu.RFQ9Z	Araip.U61TZ	single-copy	arginine--tRNAligase,cytoplasmic-like[Glycinemax]
Aradu.VXG8Q	Araip.XE7B2	single-copy	argininosuccinatelyase
Aradu.44VUH	Araip.MGZ00	single-copy	arginyl-tRNA-proteintransferase
Aradu.VDD5S	Araip.D7KCG	single-copy	armadillo/beta-cateninrepeatprotein
Aradu.8F49F	Araip.E0U35	single-copy	armadillorepeat-containingprotein6[Glycinemax]

Aradu.CJN5E	Araip.GVJ3M	single-copy	ARMREPEATPROTEININTERACTINGWITHABF2-likeis
Aradu.PIT85	Araip.VN33E	single-copy	ArseniteeffluxATP-bindingproteinArsAn=1Tax=Methanot
Aradu.TD814	Araip.HGD34	single-copy	asparagine-tRNAligase
Aradu.W88FU	Araip.LJ1YI	single-copy	aspartokinase-homoserinedehydrogenase
Aradu.88VQK	Araip.I55WQ	single-copy	aspartylaminopeptidase-like[Glycinemax]
Aradu.69H8E	Araip.D97MW	single-copy	ATPaseBadF/BadG/BcrA/BcrDtypen=3Tax=Paenibacillu
Aradu.L50NE	Araip.QP7G7	single-copy	ATPase-like,ParA/MinDn=2Tax=ChroococcalesRepID=K
Aradu.T1G99	Araip.YJC1H	single-copy	ATP-dependentpeptidases
Aradu.ZK0JG	Araip.J63ZS	single-copy	ATPsulfurylase1
Aradu.ET2UI	Araip.VJ1G3	single-copy	AT-richinteractivedomain-containingprotein1-likeisoformX
Aradu.T01MJ	Araip.14YQZ	single-copy	auxilin-relatedprotein1-likeisoformX1[Glycinemax]
Aradu.DE1N1	Araip.FRW69	single-copy	Avr9/Cf-9rapidlyelicitedprotein
Aradu.0DS3V	Araip.H7JSC	single-copy	baseexcisionDNArepairprotein,HhH-GPDfamilyprotein
Aradu.NQ3N9	Araip.96E8W	single-copy	basicleucinezipperandW2domain-containingprotein2-like
Aradu.ANJ8N	Araip.4X1DQ	single-copy	Beige/BEACHdomain
Aradu.79JEZ	Araip.M42LB	single-copy	BESTArabidopsisthalianaproteinmatchis:3'-5'exonucleas
Aradu.F52BW	Araip.57VD8	single-copy	beta-1,2-N-acetylglucosaminyltransferaseII
Aradu.K9UV7	Araip.Y1XIM	single-copy	beta-1,2-xylosyltransferase
Aradu.1T2VN	Araip.EX9I5	single-copy	beta-1,3-galactosyltransferase19-like[Glycinemax]
Aradu.V8J7V	Araip.1H31M	single-copy	beta-1,4-xylosyltransferase,putative
Aradu.E0Q62	Araip.9HV2M	single-copy	beta-amyrinsynthaseisoformX1[Glycinemax]
Aradu.A8KIV	Araip.N62I1	single-copy	beta-catenin-likeprotein
Aradu.90WZG	Araip.7VZ5Q	single-copy	beta-D-xylosidase4
Aradu.S4LWP	Araip.IXI9R	single-copy	Beta-propellerdomain-containingprotein,methanoldehydr
Aradu.9841E	Araip.58SA1	single-copy	bifunctional5,10-methylene-tetrahydrofolatedehydrogena
Aradu.ETD4B	Araip.9C8LR	single-copy	BifunctionalpolymyxinresistancearnAproteinn=2Tax=Pap
Aradu.13MQ9	Araip.VXU18	single-copy	bifunctionalpurinebiosynthesisproteinpurH-like[Glycinem
Aradu.TC25V	Araip.K2DDF	single-copy	BIM1-like[Glycinemax]
Aradu.6CL4K	Araip.58L9N	single-copy	binding
Aradu.P0J2M	Araip.CG0SE	single-copy	biotinsynthase-like[Glycinemax]
Aradu.EJE3Z	Araip.I81Z4	single-copy	BolA-likefamilyprotein
Aradu.M1AX1	Araip.Y81SY	single-copy	BPS1,chloroplastic-likeisoformX3[Glycinemax]
Aradu.C9WV0	Araip.1M7FH	single-copy	branchpoint-bridgingprotein-likeisoform1[Glycinemax]
Aradu.65JMT	Araip.N7GH3	single-copy	BRCTdomain-containingDNArepairprotein
Aradu.22C3R	Araip.MW4HG	single-copy	breastcancerassociatedRING1
Aradu.LP1C0	Araip.Y7GV4	single-copy	brefeldinA-inhibitedguaninenucleotide-exchangeprotein
Aradu.63N31	Araip.U5BCP	single-copy	BURPdomain-containingprotein
Aradu.D8A1M	Araip.S2SS4	single-copy	C3HCzincfinger-like
Aradu.C7P1W	Araip.V76V7	single-copy	caffeoyl-CoA3-O-methyltransferase
Aradu.BS31B	Araip.T7RFD	single-copy	Calcineurin-likemetallo-phosphoesterasesuperfamilyprot
Aradu.HNN21	Araip.2G27W	single-copy	calcium-dependentproteinkinase16
Aradu.CBZ5I	Araip.T2RIK	single-copy	calcium-dependentkinaseCPK1adapterprotein,putative
Aradu.11V2F	Araip.21TTJ	single-copy	Calcium-dependentphosphotriesterasesuperfamilyprotei
Aradu.L5Z6S	Araip.U0CS0	single-copy	calciumsensingreceptor

Aradu.BFS1P	Araip.LBX9K	single-copy	calcyclin-bindingprotein
Aradu.89ZRN	Araip.0Z5TL	single-copy	calponinhomologydomain-containingproteinDDB_G02724
Aradu.DAN0Y	Araip.LT8HC	single-copy	camphorresistanceCrcBfamilyprotein
Aradu.LK89V	Araip.W0145	single-copy	cAMP-regulatedphosphoprotein19-relatedprotein
Aradu.HI40M	Araip.AKP9Z	single-copy	cancer-relatednucleoside-triphosphatase-likeprotein
Aradu.648HW	Araip.W4NHQ	single-copy	canopy-likeprotein
Aradu.TQ3RZ	Araip.VVF66	single-copy	carboncataboliterepressorprotein4homolog5-likeisoformX
Aradu.P49BC	Araip.NU3WA	single-copy	carboxypeptidaseD,putative
Aradu.J4Z1M	Araip.2T4WR	single-copy	cardiolipinsynthase
Aradu.QDT9L	Araip.FRJ8B	single-copy	carotenoidcleavagedioxygenase1
Aradu.E4AIC	Araip.R12WQ	single-copy	carotenoidisomerase
Aradu.D588D	Araip.J8V1R	single-copy	CASPARALYDRAFT-likeprotein
Aradu.60HCE	Araip.2RJ39	single-copy	catalase2
Aradu.9H5DV	Araip.B2JYZ	single-copy	Catalytic,putativen=1Tax=RicinuscommunisRepID=B9T7
Aradu.J21EA	Araip.S909G	single-copy	cathepsinB-likecysteineprotease
Aradu.5W065	Araip.BN9Y3	single-copy	cation-chlorideco-transporter1
Aradu.MX2L0	Araip.K1JTN	single-copy	cationdiffusionfacilitatorfamilytransporter
Aradu.V2RP9	Araip.KFI7C	single-copy	Cationtransportdomaincontainingproteinn=1Tax=Acantha
Aradu.8LE5E	Araip.11SIA	single-copy	CCCH-typezincfingerfamilyproteinwithRNA-bindingdomai
Aradu.B14QA	Araip.NJ3UQ	single-copy	CDK-activatingkinaseassemblyfactorMAT1,putative
Aradu.KVT23	Araip.U985Z	single-copy	Cellcycleregulatedmicrotubuleassociatedprotein
Aradu.RUS0X	Araip.P01W2	single-copy	Celldifferentiation,Rcd1-likeprotein
Aradu.068JX	Araip.C0Z69	single-copy	cellnumberregulator6[Glycinemax]
Aradu.Q0MV7	Araip.ZDE4L	single-copy	cellwall/vacuolarinhibitoroffructosidase1-like[Glycinemax]
Aradu.GC05Y	Araip.FH1C1	single-copy	centromere/kinetochoreprotein,putative(ZW10)
Aradu.SJX08	Araip.8V9X0	single-copy	centromere-associatedproteinEisoformX2[Glycinemax]
Aradu.X9SEL	Araip.79WPQ	single-copy	ChaC-likefamilyprotein
Aradu.1M2X1	Araip.Y2HKR	single-copy	chlorophyllA/Bbindingprotein1
Aradu.P0CUQ	Araip.5U8GK	single-copy	chloroplastsensorkinase
Aradu.9MF3N	Araip.L2XTS	single-copy	chlororespiratoryreductionprotein
Aradu.ZTH93	Araip.ZU8P9	single-copy	cholinekinase1
Aradu.NYD5R	Araip.4VW3W	single-copy	cholinemonooxygenase
Aradu.WMN78	Araip.D85BU	single-copy	Chromatinremodelingcomplexsubunitn=1Tax=Sphaerulin
Aradu.G013M	Araip.Z633S	single-copy	chromodomain-containingproteinLHP1-likeisoformX2[Gly
Aradu.WW2SY	Araip.74PIQ	single-copy	chromosome-associatedkinesin-related
Aradu.9Y9CU	Araip.93U5S	single-copy	cirhin-likeisoform1[Glycinemax]
Aradu.CD8RE	Araip.JIQ05	single-copy	classIVaminotransferase
Aradu.Y6FFI	Araip.E0G5T	single-copy	Clathrin,heavychain
Aradu.3II03	Araip.ETT8S	single-copy	clathrinassemblyproteinAt5g57200-like[Glycinemax]
Aradu.K9JBW	Araip.P7F38	single-copy	clathrininteractorEPSIN2-likeisoformX2[Glycinemax]
Aradu.7VQ43	Araip.20HUP	single-copy	Clathrinlightchainprotein
Aradu.ZQ29G	Araip.45HI9	single-copy	CLAVATA3/ESR(CLE)-relatedprotein25-like[Glycinemax]
Aradu.MYL46	Araip.IN5DN	single-copy	cleftlipandpalatetransmembraneprotein
Aradu.5Y3NY	Araip.N90NP	single-copy	CLIP-associatedprotein

Aradu.BNJ62	Araip.JF5B7	single-copy	clusteredmitochondriaprotein-likeisoformX1[Glycinemax]
Aradu.J3SWW	Araip.92A9S	single-copy	Coatomerepsilonsubunit
Aradu.M167P	Araip.B25XR	single-copy	coatomergamma-2subunit,putative/gamma-2coatprotein,
Aradu.F8VUJ	Araip.01T7B	single-copy	Cobalamin(VitaminB12)biosynthesisCbiXproteinn=7Tax=
Aradu.MJT2G	Araip.VL605	single-copy	cobaltionbinding
Aradu.66UC2	Araip.4U431	single-copy	Co-chaperoneGrpEfamilyprotein
Aradu.WHS6B	Araip.RHZ53	single-copy	ColdacclimationproteinWCOR413family
Aradu.R7JCU	Araip.00FG1	single-copy	coldregulatedgene27
Aradu.IG7LI	Araip.F1QRY	single-copy	complex1protein,LYRfamilyprotein
Aradu.E8471	Araip.ZDT79	single-copy	Conservedhypotheticalintegralmembraneproteinn=1Tax=
Aradu.V9MVJ	Araip.WD50G	single-copy	COP1-interactingprotein7
Aradu.G0FGB	Araip.D59X4	single-copy	CoproporphyrinogenIIIoxidase
Aradu.XGN4F	Araip.CRU6Z	single-copy	Cottonfiberexpressedproteinn=1Tax=Medicagotruncatula
Aradu.X69MW	Araip.T8ZUZ	single-copy	CP12domain-containingprotein2
Aradu.BA406	Araip.R2L10	single-copy	CRAL/TRIOdomainprotein
Aradu.096I4	Araip.A4LGX	single-copy	CRIBdomain-containingproteinRIC4-like[Glycinemax]
Aradu.JY9SV	Araip.57ZZX	single-copy	CrookedneckpremRNAsplicingfactor1n=2Tax=Echinococ
Aradu.NE42A	Araip.175EE	single-copy	crossoverjunctionendonucleaseMUS81-likeprotein
Aradu.1347E	Araip.PTN9R	single-copy	CRS1/YhbY(CRM)domain-containingprotein
Aradu.3NG75	Araip.X9MCL	single-copy	CRS2-associatedfactor1,chloroplastic-like[Glycinemax]
Aradu.6I8N8	Araip.JR7JD	single-copy	CRT(chloroquine-resistancetransporter)-liketransporter3
Aradu.CH837	Araip.73ZM2	single-copy	CSLzincfingerdomain-containingprotein
Aradu.C65VQ	Araip.JY29C	single-copy	CTC-interactingdomain6
Aradu.BT3SQ	Araip.4YS5X	single-copy	CTDsmallphosphatase-likeprotein2-like[Glycinemax]
Aradu.64C9K	Araip.4L18R	single-copy	Cu/Zn-superoxidedismutasecopperchaperone
Aradu.I03LR	Araip.0PV7W	single-copy	cupinfamilyprotein
Aradu.FQ240	Araip.9F12T	single-copy	cupredoxinsuperfamilyprotein,putative
Aradu.8G7WX	Araip.C5C73	single-copy	CW7
Aradu.WLU86	Araip.F0BPT	single-copy	Cwf15/Cwc15cellcyclecontrolfamilyprotein
Aradu.45ENN	Araip.JV5S5	single-copy	CWF19-likeprotein2-likeisoformX3[Glycinemax]
Aradu.NE8U1	Araip.AW51S	single-copy	CW-typeZincFinger
Aradu.3V1LI	Araip.7C03S	single-copy	cyanobacterialandplantNDH-1subunitO
Aradu.L9M2B	Araip.71KEI	single-copy	Cyclasefamilyprotein
Aradu.HK0FS	Araip.2T5Q6	single-copy	Cyclicpyranopterinmonophosphatesynthaseaccessorypro
Aradu.I0JQ8	Araip.4N45L	single-copy	cycloeucalenolcycloisomerase
Aradu.D4D3B	Araip.LUF2N	single-copy	CYCLOIDEA-likeisoformX3[Glycinemax]
Aradu.ZFB7W	Araip.02MA2	single-copy	cyclopsprotein,putative
Aradu.1U59X	Araip.W0D6Y	single-copy	Cysteineandhistidine-richdomain-containingproteinRAR1
Aradu.FF6JW	Araip.70UIF	single-copy	Cysteine-typepeptidasen=2Tax=ArabidopsisthalianaRepI
Aradu.C3YLC	Araip.D2SBD	single-copy	cytidine/deoxycytidylatedeaminasefamilyprotein
Aradu.YI13V	Araip.2TN3Y	single-copy	cytochromecoxidaseassemblyproteinCtaG/Cox11family
Aradu.JV5EQ	Araip.DU3BQ	single-copy	cytochromeC-typebiogenesisproteinCcmE
Aradu.2Q7FP	Araip.AH9LJ	single-copy	cytochromeoxidasecomplexassemblyprotein
Aradu.H36S4	Araip.37WSW	single-copy	cytoplasmicmembraneprotein

Aradu.CV497	Araip.A7H6K	single-copy	cytoplasmictRNA2-thiolationprotein
Aradu.0XX6X	Araip.FJ13R	single-copy	cytosolicFe-SclusterassemblyfactorNUBP1-likeprotein
Aradu.PBY44	Araip.160V1	single-copy	DDB_G0282499-like[Glycinemax]
Aradu.Y7AUY	Araip.FPJ1M	single-copy	DDB1-CUL4associatedfactor1
Aradu.F4Y7W	Araip.X6TNY	single-copy	DDRGKdomain-containingprotein1-like[Glycinemax]
Aradu.UPU3E	Araip.8TY96	single-copy	DDTdomain-containingproteinDDB_G0282237-likeisofor
Aradu.YRW97	Araip.BG5WL	single-copy	debranchingenzyme1
Aradu.4Q3MP	Araip.K2XQ7	single-copy	defectsinmorphologyprotein1precursor
Aradu.DH5V9	Araip.0S5VX	single-copy	dehydration-responsiveproteinRD22
Aradu.E6T6V	Araip.0475C	single-copy	dehydrodolichyldiphosphatesynthase2-like[Glycinemax]
Aradu.9D49Q	Araip.B6W7Y	single-copy	deltasubunitofMtATPsynthase
Aradu.CML1P	Araip.VG726	single-copy	DENND6A-likeisoformX3[Glycinemax]
Aradu.JJ2FT	Araip.NC1F1	single-copy	deoxyhypusinehydroxylase
Aradu.P2TGB	Araip.G5M3E	single-copy	deoxyhypusinesynthase
Aradu.U51AH	Araip.AT5YU	single-copy	deoxyuridine5'-triphosphatenucleotidohydrolase-like[Glyc
Aradu.V7EH0	Araip.ZKD8M	single-copy	dessication-induced1VOCsuperfamilyprotein
Aradu.Q84QR	Araip.74MNE	single-copy	DGCR14-related
Aradu.DWS1F	Araip.D2NML	single-copy	DHBPsynthaseRibB-likealpha/betadomain
Aradu.6CQ40	Araip.D13W8	single-copy	Diacylglycerolkinasefamilyprotein
Aradu.46T9V	Araip.8H3SQ	single-copy	Diaminohydroxyphosphoribosylaminopyrimidinedeamina
Aradu.51556	Araip.MD8TF	single-copy	diaminopimelatedecarboxylase
Aradu.LF723	Araip.Q71DN	single-copy	dihydrolipoyldehydrogenase
Aradu.ZD05E	Araip.E0UG7	single-copy	Dihydroneopterinaldolase
Aradu.FWL77	Araip.D52D9	single-copy	dihydroorotase
Aradu.MNQ43	Araip.JNK1H	single-copy	Dihydropyrimidinedehydrogenase(NADP+)/dihydroorotat
Aradu.301B3	Araip.BK1UX	single-copy	dihydrosphingosine1-phosphatephosphataseC823.11-lik
Aradu.17HPD	Araip.W9HXX	single-copy	Dihydroxy-aciddehydratase,putativen=3Tax=Malpighiale
Aradu.MU261	Araip.S0RCS	single-copy	DIS3-likeexonuclease2-like[Glycinemax]
Aradu.8P3R8	Araip.PV4IE	single-copy	DNA-(apurinicorapyrimidinicsite)lyase2-like[Glycinemax]
Aradu.GN4F8	Araip.RYZ61	single-copy	DNA(cytosine-5-)-methyltransferasefamilyprotein
Aradu.INW8Z	Araip.R1BCW	single-copy	DNA/RNA-bindingproteinKin17,conservedregion
Aradu.0PL1F	Araip.PXW4K	single-copy	DNA-3-methyladenineglycosylase
Aradu.VV69K	Araip.XLP07	single-copy	DNAcross-linkrepairprotein
Aradu.S68YF	Araip.2A4VP	single-copy	DNAdamagecheckpointproteinn=1Tax=Chlamydomonas
Aradu.Z172U	Araip.60WTQ	single-copy	DNAdouble-strandbreakrepairandVJrecombinationXRCC
Aradu.9R07T	Araip.15KTJ	single-copy	DNAexcisionrepairproteinERCC-6-like[Glycinemax]
Aradu.3J7Z6	Araip.K1GZG	single-copy	DNAglycosylasesuperfamilyprotein
Aradu.R77I5	Araip.U2VM6	single-copy	chromodomain-helicase-DNA-bindingprotein1-likeisoform
Aradu.8UZ9S	Araip.RPL4T	single-copy	DNAligase1-likeisoformX2[Glycinemax]
Aradu.Y8XMT	Araip.AW9H3	single-copy	DNAmethyltransferase1-associatedprotein
Aradu.SSH0X	Araip.4WJ5B	single-copy	DNAtopoisomerase2-binding-likeprotein
Aradu.SF9ZV	Araip.BN5L7	single-copy	dolichol-phosphatemannosyltransferase-related
Aradu.MQK7K	Araip.C1GGI	single-copy	dolichyldiphosphatase1-likeisoform2[Glycinemax]
Aradu.RIZ04	Araip.K33N8	single-copy	dolichyl-phosphatebeta-glucosyltransferase

Aradu.K6SHY	Araip.QU01Y	single-copy	dolichylpyrophosphateGlc1Man9GlcNAc2alpha-1,3-gluco
Aradu.9L879	Araip.XH5UB	single-copy	dol-P-Man:Man(5)GlcNAc(2)-PP-Dolalpha-1,3-mannosylt
Aradu.PY313	Araip.GFH7L	single-copy	Dormancy/auxinassociatedfamilyprotein
Aradu.40DSM	Araip.PBD8R	single-copy	double-strandedDNA-bindingfamilyprotein
Aradu.83Q97	Araip.F02DS	single-copy	downstreamneighborofSon-likeprotein,putative
Aradu.8QI3B	Araip.54Y6W	single-copy	downsyndromecriticalregionprotein,putative
Aradu.Y4W78	Araip.TD7CE	single-copy	DSBAoxidoreductasefamilyprotein
Aradu.J8L3N	Araip.GTG68	single-copy	DTWdomain-containingprotein
Aradu.X8R94	Araip.3QG7R	single-copy	dualspecificityphosphatasedomainprotein
Aradu.96DV9	Araip.TCN35	single-copy	Dual-specificityRNAmethyltransferaseRlmNn=2Tax=Geo
Aradu.6N11A	Araip.06WA5	single-copy	DYADisoformX3[Glycinemax]
Aradu.S2K0Q	Araip.WN5VI	single-copy	dymeclin-likeisoformX1[Glycinemax]
Aradu.3IM1W	Araip.C4BMV	single-copy	E3SUMO-proteinligaseNSE2n=16Tax=EuarchontogliresR
Aradu.B4N91	Araip.VV015	single-copy	E3UFM1-proteinligase-likeprotein
Aradu.W4XF0	Araip.IX47H	single-copy	E6-likeisoformX2[Glycinemax]
Aradu.2E34B	Araip.4NC09	single-copy	EAF3chromatinmodificationrelatedproteinn=3Tax=Asperg
Aradu.DJ1H2	Araip.GF7L1	single-copy	EEIG1/EHBP1proteinamine-terminaldomainprotein
Aradu.YQ9K5	Araip.XM1AF	single-copy	EHdomain-containingprotein1-like[Glycinemax]
Aradu.NKZ2V	Araip.KQF28	single-copy	electroncarrier/proteindisulfideoxidoreductase
Aradu.WL92I	Araip.KA9ES	single-copy	elongationfactor1-beta
Aradu.GX5W4	Araip.2N7ZX	single-copy	elongationoffattyacidsproteinA-like[Glycinemax]
Aradu.8Y6AX	Araip.XJ4FW	single-copy	endo-1,3
Aradu.GH6V7	Araip.LM9A4	single-copy	endo-1,4-beta-glucanase
Aradu.BE884	Araip.6XF23	single-copy	endoplasmicreticulumauxinbindingprotein1
Aradu.VQ1N9	Araip.05E6K	single-copy	endoplasmicreticulummetallopeptidase-likeprotein
Aradu.ZS8UE	Araip.PD45N	single-copy	endoplasmicreticulumoxidoreductins2
Aradu.95B31	Araip.908FR	single-copy	enhancerofmRNA-decapping-likeprotein
Aradu.BB0Q9	Araip.E3LGC	single-copy	esophagealcancerassociatedprotein,putative
Aradu.W45TC	Araip.W7Q56	single-copy	EssentialproteinYae1,N-terminal
Aradu.W7HND	Araip.S195L	single-copy	Esterase/lipase/thioesterasefamilyprotein
Aradu.38M3H	Araip.9S8V8	single-copy	ethanolamine-phosphatecytidylyltransferase
Aradu.W801G	Araip.8K98A	single-copy	expressedproteinlocalizedtotheinnermembraneofthechlor
Aradu.K8H83	Araip.PK6CY	single-copy	EYESABSENThomolog
Aradu.QS5ZN	Araip.BW6Q6	single-copy	F8K7.25proteinn=1Tax=ArabidopsisthalianaRepID=Q9XH
Aradu.6F1MK	Araip.AXD2M	single-copy	F-actincappingproteinbetasubunit
Aradu.SPH1K	Araip.7D5HE	single-copy	FAM135B-likeisoformX3[Glycinemax]
Aradu.5P7M7	Araip.82VVP	single-copy	family31glycosylhydrolase,alpha-glucosidase
Aradu.J4C77	Araip.14VDU	single-copy	farnesyltransferase/geranylgeranyltransferasetype-1subu
Aradu.C7X7Y	Araip.QT81S	single-copy	FAR1-RELATEDSEQUENCE6-likeisoformX2[Glycinemax
Aradu.10BGT	Araip.I2X2E	single-copy	FAS-associatedfactor2-like[Glycinemax]
Aradu.A0MU0	Araip.3C7MV	single-copy	Fattyacid/sphingolipiddesaturase
Aradu.B7H15	Araip.Q37BC	single-copy	FBD-associatedF-boxprotein
Aradu.I99RK	Araip.QGK0J	single-copy	Fcf2pre-rRNAprocessingprotein
Aradu.T35QD	Araip.9J1EH	single-copy	ferric-chelatereductase1-like[Glycinemax]

Aradu.F06JI	Araip.DM29G	single-copy	ferrochelatase1
Aradu.R1E3C	Araip.M3RCE	single-copy	FeSassemblyproteinSufD
Aradu.PZ5G0	Araip.89MN9	single-copy	Fe-SmetabolismassociatedproteinSufE
Aradu.857YF	Araip.SD6FV	single-copy	Fe-Sproteinassemblyco-chaperoneHscB
Aradu.54BXB	Araip.G1DUK	single-copy	Fesuperoxidedismutase3
Aradu.UU910	Araip.AMI35	single-copy	FG-GAPrepeat-containingprotein
Aradu.QA9KZ	Araip.7U0RM	single-copy	fimbrin-likeprotein2
Aradu.Z6X40	Araip.BWV8I	single-copy	FK506-bindingprotein5-likeisoformX3[Glycinemax]
Aradu.DB65S	Araip.MH44P	single-copy	flapendonucleaseGEN-likeprotein
Aradu.Q5M0R	Araip.NIV47	single-copy	flavanone3-hydroxylase[Glycinemax]
Aradu.09HMV	Araip.21QMU	single-copy	FMN-bindingsplitbarreln=1Tax=PlasmoparaviticolaRepID
Aradu.V49C9	Araip.66MK2	single-copy	Folicacidbinding/transferasen=4Tax=CamelineaeRepID=
Aradu.2C28W	Araip.W0AVN	single-copy	Forkhead-associated(FHA)domain-containingprotein
Aradu.K05BK	Araip.71Z4W	single-copy	formamidopyrimidine-DNAglycosylase
Aradu.02ZTY	Araip.Z52VV	single-copy	formate--tetrahydrofolateligase-likeisoformX1[Glycinema
Aradu.VDX8A	Araip.08VK1	single-copy	formyltetrahydrofolatedeformylase,putative
Aradu.WI4BU	Araip.MWF2H	single-copy	frigida-LIKEprotein
Aradu.K18SI	Araip.SU28H	single-copy	fructokinase-like1
Aradu.YG6N5	Araip.Y0FEW	single-copy	furry-likeisoformX1[Glycinemax]
Aradu.CQ2HY	Araip.57MS8	single-copy	fusaricacidresistancefamilyprotein
Aradu.WS13Y	Araip.2K9WW	single-copy	gammacarbonicanhydrase1
Aradu.NCP7U	Araip.X02G0	single-copy	gamma-irradiationandmitomycininduced1
Aradu.8A403	Araip.S6JZ9	single-copy	gamma-solubleNSFattachmentprotein
Aradu.4B6K6	Araip.KNG8V	single-copy	gammasubunitofMtATPsynthase
Aradu.R479P	Araip.ISI3L	single-copy	gamma-tocopherolmethyltransferase
Aradu.7N2UH	Araip.9LQ1M	single-copy	ganglioside-induceddifferentiation-associated-likeprotein
Aradu.41I2U	Araip.A89IR	single-copy	GCN5-relatedN-acetyltransferasen=1Tax=Nostocsp.PCC
Aradu.PV79J	Araip.0WD88	single-copy	GC-richsequenceDNA-bindingfactor-likeprotein
Aradu.G21TT	Araip.UHJ6H	single-copy	GDP-Man:Man(3)GlcNAc(2)-PP-Dolalpha-1,2-mannosylt
Aradu.XN88F	Araip.635QE	single-copy	geranyldiphosphatesynthase1
Aradu.L6TW0	Araip.W5IYZ	single-copy	geranylgeranyldiphosphatereductase,chloroplastic-like[G
Aradu.EGN24	Araip.3J3Q4	single-copy	geranylgeranyltransferasetype-1subunitbeta-like[Glycine
Aradu.VIH8G	Araip.BS9NN	single-copy	GGLdomainprotein
Aradu.GX58B	Araip.RX4DL	single-copy	GHMPkinasefamilyprotein
Aradu.LS2KI	Araip.37BUF	single-copy	GLABRA3-like[Glycinemax]
Aradu.D8U6I	Araip.6P9JS	single-copy	glucomannan4-beta-mannosyltransferase2-like[Glycinem
Aradu.44JZ9	Araip.56MFZ	single-copy	Glucose-fructoseoxidoreductasedomain-containingprotei
Aradu.0PI99	Araip.L7WHL	single-copy	glutamate-ammonialigase-likeprotein
Aradu.RII5F	Araip.B5KKJ	single-copy	GlutamineamidotransferasesubunitpdxTn=3Tax=Papilion
Aradu.IRK1D	Araip.FV8HT	single-copy	glutamine-dependentNAD(+)synthetase,putative
Aradu.51WYZ	Araip.T9T6I	single-copy	glutaminesynthetase1
Aradu.B6T0L	Araip.3H4X4	single-copy	glutamine-tRNAligase,putative/glutaminyl-tRNAsynthetas
Aradu.F9182	Araip.9P9V2	single-copy	glutamyl-tRNA(Gln)amidotransferasesubunitA-likeprotein
Aradu.06HG0	Araip.IA04P	single-copy	GlycerophosphodiesterphosphodiesteraseGDE1n=2Tax=

Aradu.212BQ	Araip.E0W6R	single-copy	glycerophosphoryldiesterphosphodiesterase3-like[Glycine
Aradu.U7A5U	Araip.KF1D0	single-copy	GlycinecleavageT-proteinfamily
Aradu.RB83Y	Araip.R4K41	single-copy	Glycinedehydrogenasedecarboxylatingproteinn=3Tax=Ro
Aradu.AVN2W	Araip.0214S	single-copy	glycogen/starch/alpha-glucanphosphorylasefamilyprotein
Aradu.4C32F	Araip.89FXQ	single-copy	glycogenphosphorylase1-likeisoformX1[Glycinemax]
Aradu.H3P3G	Araip.8XZ82	single-copy	glycosidehydrolasefamily81protein
Aradu.7UX0U	Araip.G9ZI8	single-copy	glycylpeptideN-tetradecanoyltransferase
Aradu.T5087	Araip.2S2EB	single-copy	glycyl-tRNAsynthetase/glycine-tRNAligase
Aradu.RYQ8I	Araip.WUR54	single-copy	glyoxalase/bleomycinresistanceprotein/dioxygenase
Aradu.R4JE4	Araip.03SU2	single-copy	golgincandidate2
Aradu.8A153	Araip.2ZN3C	single-copy	GolgiSNAPreceptorcomplexmember1n=6Tax=Papilionoid
Aradu.20IMG	Araip.3F9PG	single-copy	GpatchdomainandKOWmotifs-containingproteinn=3Tax=
Aradu.L39ME	Araip.98DPV	single-copy	GPCR-typeGprotein2-likeisoformX3[Glycinemax]
Aradu.6A2D1	Araip.T5MHX	single-copy	GPIinositol-deacylasen=3Tax=AspergillusRepID=G7XGG
Aradu.T0VLB	Araip.VX608	single-copy	GPItransamidasecomponentGpi16subunitfamilyprotein
Aradu.GC6L4	Araip.13VTA	single-copy	GPN-loopGTPase3homologisoformX2[Glycinemax]
Aradu.S8XF6	Araip.ZDG59	single-copy	G-proteincoupledreceptors
Aradu.QK85I	Araip.T7YD7	single-copy	granuleboundstarchsynthase
Aradu.9XX4W	Araip.ZPK2M	single-copy	group1familyglycosyltransferase
Aradu.U012P	Araip.GHK6Z	single-copy	growthinhibitionanddifferentiation-likeprotein,putative
Aradu.WNV5J	Araip.2LE64	single-copy	GTPcyclohydrolaseI
Aradu.1ZQ3K	Araip.KET11	single-copy	G-typelectinS-receptor-likeSerine/Threonine-kinase
Aradu.G2YAZ	Araip.64VMF	single-copy	guanylylcyclase1
Aradu.Y5MLI	Araip.KTR75	single-copy	HASTY1-likeisoformX1[Glycinemax]
Aradu.GGH8Z	Araip.HF3EC	single-copy	HAUSaugmin-likecomplexsubunit-likeprotein
Aradu.QQF2S	Araip.J736G	single-copy	helicaseproteinMOM1-likeisoformX2[Glycinemax]
Aradu.FI298	Araip.I35QI	single-copy	heme-bindingprotein2[Glycinemax]
Aradu.M1UTK	Araip.EYE7R	single-copy	HemerythrinclassglutathioneS-transferasen=1Tax=Physc
Aradu.6KR5S	Araip.5C9RI	single-copy	hemoglobin3
Aradu.NC3JN	Araip.6A689	single-copy	hepatocellularcarcinoma-associatedantigen59
Aradu.3E5G3	Araip.R0W2T	single-copy	heterogeneousnuclearribonucleoproteinU-likeprotein1-like
Aradu.MSK3Z	Araip.UL14Y	single-copy	HhH-GPDbaseexcisionDNArepairfamilyprotein
Aradu.EL4CE	Araip.SX4W8	single-copy	HI0933familyflavoprotein
Aradu.2RS8H	Araip.Z7NW6	single-copy	high-affinitynickel-transportfamilyprotein
Aradu.TSW1P	Araip.354H4	single-copy	HIPL1protein-like[Glycinemax]
Aradu.UX9CD	Araip.LN55K	single-copy	hippocampusabundanttranscript-likeprotein
Aradu.105GZ	Araip.E92TL	single-copy	histidineproteinmethyltransferase1homologisoformX1[Gly
Aradu.3D2PD	Araip.JRQ56	single-copy	histidinoldehydrogenase
Aradu.DL5AX	Araip.F09QG	single-copy	histidinolphosphateaminotransferase1
Aradu.ZMN8D	Araip.ZSM5V	single-copy	Histidyl-tRNAsynthetase1
Aradu.JJY9M	Araip.564H9	single-copy	histoneacetyltransferaseoftheMYSTfamily1
Aradu.4XJ32	Araip.ZXF0A	single-copy	histoneacetyltransferasetypeBcatalyticsubunit,putative
Aradu.ZA91W	Araip.ING83	single-copy	holocarboxylasesynthetase
Aradu.V3C05	Araip.ZG914	single-copy	homeobox/lipid-bindingdomainprotein

Aradu.NC6JU	Araip.CQ2I9	single-copy	homogentisate1,2-dioxygenase
Aradu.70H43	Araip.DWY4Y	single-copy	homogentisateprenyltransferase
Aradu.T0YTZ	Araip.B73E0	single-copy	homologofDNAmismatchrepairproteinMSH3
Aradu.5R3CV	Araip.DS83W	single-copy	homologofnucleolarproteinNOP56
Aradu.HN2EG	Araip.BVG2F	single-copy	homologofseparase
Aradu.V8MJ9	Araip.1U5CY	single-copy	homologofSynechocystisYCF37
Aradu.CJT43	Araip.ZH07M	single-copy	hydrogenperoxideinducedprotein,putative
Aradu.57TMQ	Araip.KG8FH	single-copy	Hypoxia-responsivefamilyprotein
Aradu.E1TLU	Araip.XD01H	single-copy	impairedsucroseinductionprotein,putative
Aradu.Q67RD	Araip.VJ1WN	single-copy	Inorganicanionexchangern=1Tax=Rhodosporidiumtoruloi
Aradu.QMU6X	Araip.IQ4XK	single-copy	inorganicpyrophosphatase
Aradu.22C6J	Araip.ZZ5S6	single-copy	inosine-5'-monophosphatedehydrogenase
Aradu.7YA73	Araip.U3V28	single-copy	inositolhexakisphosphateanddiphosphoinositol-pentakisp
Aradu.KYQ8M	Araip.N3JDF	single-copy	Inositolmonophosphatasefamilyprotein
Aradu.41JSL	Araip.C1AWR	single-copy	Inositol-pentakisphosphate2-kinasefamilyprotein
Aradu.T9XRG	Araip.C8Q7I	single-copy	inositoltransporter1
Aradu.X7WYU	Araip.6V57K	single-copy	insulin-degradingenzyme
Aradu.5D3K5	Araip.VQ328	single-copy	interaptin-likeisoformX5[Glycinemax]
Aradu.U1A8I	Araip.GP1XI	single-copy	GTP-bindingproteinn=1Tax=Arabidopsislyratasubsp.lyrat
Aradu.T45BF	Araip.B162Z	single-copy	intermembranespaceimportandassemblyprotein
Aradu.ZFA07	Araip.6I56J	single-copy	internalalternativeNAD(P)H-ubiquinoneoxidoreductaseA1
Aradu.P82M7	Araip.R07YY	single-copy	intron-bindingproteinaquarius-like[Glycinemax]
Aradu.V2IK2	Araip.X33WA	single-copy	INVOLVEDIN:chromosomesegregation,celldivision
Aradu.KT81X	Araip.80ZQA	single-copy	INVOLVEDIN:positiveregulationofcatalyticactivity,proteinp
Aradu.0FI0R	Araip.V5LSC	single-copy	IonchannelDMI1n=23Tax=PapilionoideaeRepID=DMI1_M
Aradu.MW11Y	Araip.Q2QUM	single-copy	ionchannelPOLLUX-like2-likeisoformX2[Glycinemax]
Aradu.FD1ZK	Araip.Y1YK4	single-copy	ionchannelregulatoryproteinUNC-93
Aradu.BJ3DD	Araip.50ZEB	single-copy	irondonorproteinCyaY
Aradu.CQN7Q	Araip.BE5FQ	single-copy	isochorismatesynthase2
Aradu.0KG04	Araip.SVT7Q	single-copy	isocitratelyase
Aradu.93CHA	Araip.E99A6	single-copy	isoflavonereductasehomolog[Glycinemax]
Aradu.CG65D	Araip.9I2Z5	single-copy	Isoform3ofErlin-2n=1Tax=HomosapiensRepID=O94905-3
Aradu.P8M1S	Araip.SNW0B	single-copy	isopentenyl-diphosphatedelta-isomerase
Aradu.0RW41	Araip.KSH3B	single-copy	isoprenylcysteinealpha-carbonylmethylesteraseICMEprot
Aradu.RP5AZ	Araip.C6G0D	single-copy	isovaleryl-CoA-dehydrogenase
Aradu.12ETV	Araip.I4CFI	single-copy	jasmonicacidcarboxylmethyltransferase
Aradu.11XZB	Araip.W441F	single-copy	JHL23C09.7proteinn=1Tax=JatrophacurcasRepID=E6NU
Aradu.JQ0FN	Araip.DN0QK	single-copy	josephin-likeprotein-like[Glycinemax]
Aradu.APR5V	Araip.FJL9Y	single-copy	kataninp80WD40repeatsubunitB1-likeprotein
Aradu.PXZ8J	Araip.XS8K8	single-copy	kelchrepeatsuperfamilyprotein
Aradu.68XYR	Araip.7N26D	single-copy	kinetochoreNuf2-likeprotein
Aradu.CNT80	Araip.F11A4	single-copy	kish-A-likeprotein
Aradu.LH2UI	Araip.85AW8	single-copy	knotted1-bindingprotein
Aradu.GS29Q	Araip.31ZB6	single-copy	kunitztrypsininhibitor1

Aradu.J7JS6	Araip.87JV8	single-copy	lanC-likeprotein2-likeisoformX1[Glycinemax]
Aradu.254QE	Araip.Z3KJR	single-copy	largeproline-richproteinBAG6-likeisoformX3[Glycinemax]
Aradu.C2VRV	Araip.Y1811	single-copy	LARNA-bindingprotein
Aradu.FFY3F	Araip.D0YN7	single-copy	L-aspartateoxidase
Aradu.VW8XJ	Araip.989UZ	single-copy	LATERALROOTPRIMORDIUM1-likeisoformX1[Glycinem
Aradu.BZU3P	Araip.T09RD	single-copy	leafyhomolog2n=115Tax=PentapetalaeRepID=FL2_TOB
Aradu.XVQ80	Araip.06WGU	single-copy	legumintypeB-like[Glycinemax]
Aradu.DME0M	Araip.TU2HQ	single-copy	LETM1andEF-handdomain-containingprotein1,mitochond
Aradu.7GM9W	Araip.Q143D	single-copy	leukocytereceptorclustermember8homologisoformX3[Gly
Aradu.W3BNI	Araip.9J3MN	single-copy	L-galactono-1,4-lactonedehydrogenase
Aradu.Y8PUZ	Araip.BBV0C	single-copy	LHCPtranslocationdefectprotein,putative
Aradu.8L7DK	Araip.UYW0K	single-copy	LHW-like[Glycinemax]
Aradu.D7HT5	Araip.3A81Q	single-copy	light-regulatedprotein,putative
Aradu.T66QJ	Araip.BH6DK	single-copy	Light-sensorProteinkinasen=2Tax=CeratodonpurpureusR
Aradu.W07KG	Araip.NWR3L	single-copy	linoleate13S-lipoxygenase2-1,relatedprotein
Aradu.NI3N5	Araip.AAR28	single-copy	Lipinfamilyprotein
Aradu.K75LB	Araip.EP35E	single-copy	lipocalin-likedomainprotein
Aradu.14261	Araip.HH32S	single-copy	lisHdomainandHEATrepeatKIAA1468-likeprotein
Aradu.14U3F	Araip.D1KQ3	single-copy	lonprotease1
Aradu.A1C01	Araip.5N24I	single-copy	LowPSIIAccumulation3isoform1n=4Tax=Theobromacaca
Aradu.M5IDE	Araip.YTI0W	single-copy	LSD1zincfingerfamilyprotein
Aradu.W8K8D	Araip.9J3NW	single-copy	lycopenecyclase
Aradu.43TRE	Araip.WU624	single-copy	lysine-richarabinogalactanprotein18-like[Glycinemax]
Aradu.058CJ	Araip.9L82B	single-copy	lysine-tRNAligase-likeprotein
Aradu.Q4YIY	Araip.TLW99	single-copy	lysMandputativepeptidoglycan-bindingdomain-containing
Aradu.0TS71	Araip.D3HL4	single-copy	lysophosphatidylacyltransferase5
Aradu.W4W1A	Araip.89N01	single-copy	lysosomalalpha-mannosidase-like[Glycinemax]
Aradu.3C2YM	Araip.MV4VH	single-copy	lysosomalbetaglucosidase-likeisoformX4[Glycinemax]
Aradu.FT5QT	Araip.43I77	single-copy	lysosomalpro-Xcarboxypeptidase-likeprotein
Aradu.N7LX6	Araip.BK8GH	single-copy	M20peptidaseacetylornithinedeacetylase
Aradu.FC1CK	Araip.1J91U	single-copy	Maf-likeprotein
Aradu.76H6A	Araip.27JTJ	single-copy	magnesium(Mg)transporter10
Aradu.TL35C	Araip.23NEF	single-copy	magnesiumionbinding
Aradu.XRM9M	Araip.G25KD	single-copy	MAK16protein-related
Aradu.97TKQ	Araip.ATJ43	single-copy	malatesynthase
Aradu.XH3MQ	Araip.A1X3Q	single-copy	MalD1-associatedprotein
Aradu.X258N	Araip.LUU1Z	single-copy	Mandelateracemase/muconatelactonizingproteinn=2Tax=
Aradu.10VKJ	Araip.4N5EI	single-copy	mannosylglycoproteinendo-beta-mannosidase-like[Glycin
Aradu.74DEV	Araip.55L6R	single-copy	mannosyl-oligosaccharideglucosidase
Aradu.0DL1W	Araip.21V2Q	single-copy	MarinesedimentmetagenomeDNA,contig:S01H1_C00432
Aradu.EAE80	Araip.NH8TE	single-copy	MATHdomain-containingproteinAt5g43560-likeisoformX1
Aradu.Z5G5W	Araip.S3X4J	single-copy	MBOAT(membraneboundO-acyltransferase)familyprotein
Aradu.BHH9A	Araip.Y0HFI	single-copy	membrane-boundtranscriptionfactorsite-1protease[Glycin
Aradu.7MW89	Araip.1U5BB	single-copy	MembranefusionproteinUse1

Aradu.RY6N7	Araip.WC7UB	single-copy	membranesteroidbindingprotein1
Aradu.HRJ8J	Araip.K9XF4	single-copy	metacaspase1
Aradu.KM3QT	Araip.VRE44	single-copy	Metaltransportproteinn=1Tax=MedicagotruncatulaRepID=
Aradu.16Z19	Araip.UJ65V	single-copy	metaxin-related
Aradu.14CRM	Araip.39SY8	single-copy	methionineaminopeptidase1B
Aradu.02IGP	Araip.V3B0A	single-copy	methioninegamma-lyase
Aradu.LTN41	Araip.79B36	single-copy	methionine-tRNAligase,putative
Aradu.AE07K	Araip.UMR2E	single-copy	methylthioadenosinenucleosidase1
Aradu.V5ZS1	Araip.207MI	single-copy	methylthioribosekinase
Aradu.7W7QL	Araip.XMH4N	single-copy	methylthioribulose-1-phosphatedehydratase
Aradu.3EX3F	Araip.N3BQC	single-copy	microfibrillar-associatedprotein-related
Aradu.H9N2F	Araip.MHA7X	single-copy	microsomalglutathiones-transferase,putative
Aradu.DBJ1I	Araip.VX6NX	single-copy	microsomalsignalpeptidase12kDaprotein
Aradu.B3V05	Araip.J98CT	single-copy	microtubule-bindingproteinTANGLED-like[Glycinemax]
Aradu.7TI6W	Araip.59ZT3	single-copy	MID1-COMPLEMENTINGACTIVITY1-likeisoformX1[Glyc
Aradu.9CU19	Araip.7D071	single-copy	flavoproteinwrbA-like[Glycinemax]
Aradu.E7L5G	Araip.VV17U	single-copy	Miro(MitochondrialRho)protein(GTPase/calciumionbindin
Aradu.5VL3J	Araip.U65I2	single-copy	mitochondrial37SribosomalproteinS27-like[Glycinemax]
Aradu.96IA2	Araip.118R7	single-copy	mitochondrialchaperonebcs1-like[Glycinemax]
Aradu.8ET3H	Araip.UJT36	single-copy	MitochondrialDistributionandMorphologyn=1Tax=Scheffe
Aradu.LY8JJ	Araip.YR3S7	single-copy	mitochondrialprocessingpeptidasealphasubunit
Aradu.3LE29	Araip.2MY4B	single-copy	mitochondrialproteinAtMg00810-like[Glycinemax]
Aradu.LX2KK	Araip.0E3QE	single-copy	mitochondrialpyruvatecarrier1-likeisoformX3[Glycinemax]
Aradu.U6IZ8	Araip.DJ2ZW	single-copy	mitochondrialuncouplingprotein2-like[Glycinemax]
Aradu.RA9YT	Araip.4V6RH	single-copy	mitosisproteinDIM1
Aradu.2R2WH	Araip.7E7XY	single-copy	mitoticspindleassemblycheckpointprotein,putative
Aradu.E84ZM	Araip.C9CUB	single-copy	MitovirusRNA-dependentRNApolymerase
Aradu.IQ8X9	Araip.FU0RV	single-copy	MKI67FHAdomain-interactingnucleolarphosphoprotein-lik
Aradu.0JV1U	Araip.J80IJ	single-copy	MMS19nucleotideexcisionrepair-likeprotein
Aradu.0VP5J	Araip.ZVT8T	single-copy	Mo25familyprotein
Aradu.TM4JI	Araip.Q2JJX	single-copy	MON2homologisoformX3[Glycinemax]
Aradu.26N4W	Araip.0U4IT	single-copy	monodehydroascorbatereductase4
Aradu.P2TIC	Araip.TH9KW	single-copy	monosaccharidetransporter[Glycinemax]
Aradu.XH0YF	Araip.TM5WG	single-copy	MORN(MembraneOccupationandRecognitionNexus)repe
Aradu.3DV9B	Araip.04K1E	single-copy	MRNA,cds,clone:RAFL24-31-B07,putativen=1Tax=Theob
Aradu.I022X	Araip.KC2C1	single-copy	mRNA-decappingenzyme
Aradu.EUP1G	Araip.FN82I	single-copy	mRNAsplicingfactor,thioredoxin-likeU5snRNP
Aradu.90QAV	Araip.3V1RA	single-copy	MTD1n=2Tax=MedicagotruncatulaRepID=G7I932_MED
Aradu.9IB22	Araip.W1A0F	single-copy	multipleC2andtransmembranedomain-containingprotein2
Aradu.RY9YC	Araip.19TXN	single-copy	multiplemyelomatumor-associatedprotein2homolog[Glyci
Aradu.51KU5	Araip.H6QSZ	single-copy	muscleM-lineassemblyproteinunc-89-likeisoformX1[Glyci
Aradu.C2PQ3	Araip.HEL12	single-copy	n=1Tax=Oryzasativasubsp.japonicaRepID=Q7XKB1_OR
Aradu.5V20C	Araip.0IQ14	single-copy	n=3Tax=OryzasativaRepID=Q7XUY4_ORYSJ
Aradu.3C582	Araip.M7YXX	single-copy	N-acetyl-gamma-glutamyl-phosphatereductase

Aradu.JL0B3	Araip.ZI6SJ	single-copy	N-acetylglucosaminyl-phosphatidylinositol(GPI)biosynthe
Aradu.TUT46	Araip.Y7KTN	single-copy	N-acetyltransferaseESCO2-likeprotein
Aradu.2Y4H2	Araip.MY16C	single-copy	N-acylphosphatidylethanolaminesynthase-likeisoformX1[
Aradu.SAM0J	Araip.W7LWH	single-copy	NAD(H)kinase3
Aradu.JFA7C	Araip.JQ4T7	single-copy	NAD(P)Hdehydrogenase18
Aradu.ADH1A	Araip.3JF99	single-copy	NAD(P)-linkedoxidoreductase-likeprotein
Aradu.30TWS	Araip.SKT5W	single-copy	NAD+:PROTEIN(ADP-ribosyl)-transferase,ADPRTn=1Ta
Aradu.RT9KH	Araip.34S35	single-copy	NADH:cytochromeB5reductase1
Aradu.LSV4Q	Araip.H1403	single-copy	NADH:ubiquinoneoxidoreductaseintermediate-associated
Aradu.792W7	Araip.CZ98B	single-copy	NADH-quinoneoxidoreductasesubunitNn=7Tax=Rhizobiu
Aradu.7S6GM	Araip.I90PW	single-copy	NADPH:quinoneoxidoreductase
Aradu.ZSZ74	Araip.D92TL	single-copy	Naphthoatesynthasen=3Tax=CucumisRepID=E5GBI7_C
Aradu.16HC5	Araip.1I1JH	single-copy	nascentpolypeptide-associatedcomplexsubunitalpha-likep
Aradu.BM5FL	Araip.ZG9AN	single-copy	NCS1nucleosidetransporterfamilyproteinn=2Tax=Strepto
Aradu.MY7DS	Araip.C1XY2	single-copy	NEDD8-activatingenzymeE1regulatorysubunit-likeprotein
Aradu.91Y21	Araip.L4KJR	single-copy	negativecofactor2transcriptionalco-repressor,putative
Aradu.6X9I9	Araip.7S61E	single-copy	nestin-likeisoformX2[Glycinemax]
Aradu.45ASZ	Araip.JZ9QI	single-copy	neuroblastoma-amplifiedsequence-like[Glycinemax]
Aradu.GL7L0	Araip.3EZ7B	single-copy	neuroguidin-likeisoformX3[Glycinemax]
Aradu.8X20T	Araip.ZQ4WS	single-copy	neutral/alkalinenon-lysosomalceramidase
Aradu.EYN6M	Araip.V8DUY	single-copy	nicotianaminesynthase2
Aradu.I94L5	Araip.1CV02	single-copy	nicotianatabacumORFprotein
Aradu.ZHM8Y	Araip.M02FS	single-copy	Nicotinate-nucleotidepyrophosphorylase(Carboxylating)n
Aradu.XZP9V	Araip.Q3CU1	single-copy	nicotinatephosphoribosyltransferase1
Aradu.Y48CE	Araip.14FTC	single-copy	nine-cis-epoxycarotenoiddioxygenase4
Aradu.W2C81	Araip.YBR8J	single-copy	nitratereductase1
Aradu.R0BCA	Araip.BIA41	single-copy	nitricoxidesynthase-interactingprotein-like[Glycinemax]
Aradu.4S63Z	Araip.655BS	single-copy	nitritereductase1
Aradu.CZI9K	Araip.4S4ZY	single-copy	nitroreductasefamilyprotein
Aradu.F5XX7	Araip.WV79D	single-copy	NoduleCysteine-Rich(NCR)secretedpeptide
Aradu.9Q1AJ	Araip.RKK5U	single-copy	Non-lysosomalglucosylceramidase
Aradu.P7W5S	Araip.8I8HL	single-copy	Non-symbiotichemoglobin
Aradu.HU7Y9	Araip.61HR0	single-copy	NRDE2homologisoformX1[Glycinemax]
Aradu.3MG6J	Araip.EC0NK	single-copy	Nse4,componentofSmc5/6DNArepaircomplex
Aradu.X8R8H	Araip.KD41Z	single-copy	nuclearexportmediatorfactorNEMF-likeisoformX5[Glycine
Aradu.HG6NA	Araip.46604	single-copy	nuclearinhibitorofproteinphosphatase
Aradu.F2B57	Araip.V3I44	single-copy	NuclearporelocalisationproteinNPL4
Aradu.8025N	Araip.87MXF	single-copy	Nuclearproteinrequiredforactincytoskeletonorganization,p
Aradu.AA5JL	Araip.C5XGE	single-copy	nuclearribonucleaseZ
Aradu.3GC8H	Araip.K88SY	single-copy	nucleolaressentialprotein-related
Aradu.ZH4KD	Araip.L0F41	single-copy	nucleolarMIF4Gdomainprotein
Aradu.33XZT	Araip.AZ5LA	single-copy	nucleoredoxin2-likeisoform1[Glycinemax]
Aradu.6XE07	Araip.3A6UX	single-copy	nucleotideexchangefactorSIL1
Aradu.XDS84	Araip.LRG7E	single-copy	Nutrientreservoir,putativen=1Tax=RicinuscommunisRepI

Aradu.DC7TG	Araip.2WB4K	single-copy	O-acetyltransferasefamilyprotein
Aradu.UC09A	Araip.8508H	single-copy	OligopeptidaseB,Serinepeptidase,MEROPSfamilyS09An=
Aradu.THY46	Araip.5D9GX	single-copy	oligopeptidetransporter4
Aradu.X6AKD	Araip.AM4LP	single-copy	one-helixprotein2
Aradu.K1MHV	Araip.DY62Y	single-copy	Opticatrophy3protein(OPA3)
Aradu.86I8Y	Araip.U0G8M	single-copy	ORF64cn=1Tax=PinuskoraiensisRepID=UPI000017DDE
Aradu.LR2WR	Araip.HNC7T	single-copy	organellarsingle-strandedDNAbindingprotein3
Aradu.C31L4	Araip.DG2YH	single-copy	ornithinecyclodeaminase/mu-crystallin
Aradu.YU82F	Araip.0WM4W	single-copy	ornithine-delta-aminotransferase
Aradu.495R5	Araip.H55PK	single-copy	OSB1,mitochondrial-likeisoformX4[Glycinemax]
Aradu.EFX4S	Araip.E9N2X	single-copy	outerenvelopeporeprotein24,chloroplastic-like[Glycinema
Aradu.83UZ1	Araip.G3762	single-copy	Oxidativestress3n=1Tax=TheobromacacaoRepID=UPI00
Aradu.Q9Y83	Araip.S5AVW	single-copy	oxophytodienoate-reductase3
Aradu.J0PRI	Araip.Z08PZ	single-copy	oxoprolinase1
Aradu.9R7J6	Araip.171V2	single-copy	oxygen-independentcoproporphyrinogenIIIoxidase,putati
Aradu.PLL31	Araip.U184X	single-copy	PAB1-bindingprotein1-likeisoformX4[Glycinemax]
Aradu.C6S8Z	Araip.702H5	single-copy	palecressprotein(PAC)
Aradu.AC9ZE	Araip.F3TE1	single-copy	PAM68,chloroplastic[Glycinemax]
Aradu.P2KMP	Araip.PC2P2	single-copy	Pantoate--beta-alanineligasen=1Tax=LotusjaponicusRep
Aradu.Z3UBC	Araip.0FU6X	single-copy	PAR1protein
Aradu.0EM6K	Araip.S4JFP	single-copy	partnerofY14-MAGO
Aradu.23179	Araip.V0B0S	single-copy	PEBP(phosphatidylethanolamine-bindingprotein)familypr
Aradu.CB26G	Araip.AR2VK	single-copy	pelota-like[Glycinemax]
Aradu.P4KG5	Araip.LY7NT	single-copy	PeptidaseS24/S26A/S26B/S26Cfamilyprotein
Aradu.E1M4X	Araip.50JTJ	single-copy	peptidedeformylase1A
Aradu.N8VJ2	Araip.0MB9K	single-copy	peptidoglycan-bindingLysMdomain-containingprotein
Aradu.9DJ84	Araip.E7CDH	single-copy	Peptidyl-tRNAhydrolasefamilyprotein
Aradu.B24NU	Araip.69PE3	single-copy	periplasmicpolyamine-bindingprotein,putative
Aradu.K8XCN	Araip.V1PYY	single-copy	peroxin3
Aradu.54WNJ	Araip.H6WCB	single-copy	peroxisomal(S)-2-hydroxy-acidoxidaseGLO1
Aradu.DN8FJ	Araip.YA5WE	single-copy	peroxisomebiogenesisprotein2-likeisoformX2[Glycinema
Aradu.XGI6B	Araip.8W3B2	single-copy	pescadillohomolog[Glycinemax]
Aradu.2I4G0	Araip.B06YF	single-copy	PGR5-likeB
Aradu.62WGK	Araip.TTH1U	single-copy	phagecapsidscaffoldingprotein(GPO)serinepeptidase
Aradu.Y9QAN	Araip.IJ811	single-copy	phenylalanyl-tRNAsynthetase,putative/phenylalanine--tR
Aradu.X114S	Araip.N29YP	single-copy	phloemA10-likeprotein
Aradu.ZDD46	Araip.Z9JDD	single-copy	phosphatidatephosphataseLPIN3-like[Glycinemax]
Aradu.I83LC	Araip.PG6NQ	single-copy	phosphatidylinositol/phosphatidylcholinetransferproteinS
Aradu.E1NAR	Araip.DTH2E	single-copy	phosphatidylinositol-glycanbiosynthesisclassXprotein-like
Aradu.EX52R	Araip.T2824	single-copy	Phosphatidylinositolkinase(PIK-G1)n=7Tax=Phytophthor
Aradu.DK6DD	Araip.WZY1Y	single-copy	phosphatidylinositolN-acetylglucosaminyltransferasesubu
Aradu.42SQT	Araip.V7BYX	single-copy	phosphatidylserinesynthase
Aradu.DE8ET	Araip.Z22W3	single-copy	phosphoacetylglucosaminemutase-likeisoformX3[Glycine
Aradu.8NK92	Araip.ZJB2Y	single-copy	PhosphoesteraseDHHA1n=3Tax=AcidovoraxRepID=F0C

Aradu.XL4N2	Araip.VD3IG	single-copy	phosphoethanolamineN-methyltransferase
Aradu.RJA7P	Araip.S0TNW	single-copy	phosphoglucanphosphataseLSF1
Aradu.0UW7J	Araip.IB6M8	single-copy	Phosphoglyceratekinasefamilyprotein
Aradu.JM9X6	Araip.8FI20	single-copy	phospholipidN-methyltransferase
Aradu.4N6LD	Araip.B8Y5M	single-copy	phosphoribosylamine-glycineligase
Aradu.G3CH2	Araip.7PF1U	single-copy	phosphoribosylaminoimidazole-succinocarboxamidesynth
Aradu.81WR9	Araip.WT4L7	single-copy	phosphoribosylglycinamideformyltransferase
Aradu.2M3LR	Araip.6L6ZR	single-copy	Phosphorylasesuperfamilyprotein
Aradu.KCP9Z	Araip.YR35J	single-copy	phosphoserineaminotransferase
Aradu.1JP2B	Araip.61FFX	single-copy	phosphotransferases,alcoholgroupasacceptor
Aradu.B4I2A	Araip.GEE0K	single-copy	pH-responseregulatorproteinpalA/RIM20n=3Tax=Aspergi
Aradu.I118N	Araip.P1R6Y	single-copy	p-hydroxybenzoicacideffluxpumpsubunitaaeBn=1Tax=Me
Aradu.BL1S9	Araip.R0KMB	single-copy	phytochelatinsynthase2
Aradu.E3ZED	Araip.HY5UP	single-copy	phytochromeA
Aradu.TD3N5	Araip.Q55MD	single-copy	plant/F1M20-13protein
Aradu.86KS5	Araip.C841I	single-copy	plant/MNJ8-150protein
Aradu.5P6B7	Araip.L7VH4	single-copy	plant/T32A16-60protein
Aradu.V4203	Araip.Z0L35	single-copy	plant/T7N9-9protein
Aradu.K5U1F	Araip.UH7KP	single-copy	Plantcalmodulin-bindingprotein-related
Aradu.1EH3D	Araip.X14G7	single-copy	PlantnatriureticpeptideAn=1Tax=TheobromacacaoRepID
Aradu.HT46D	Araip.UB7JK	single-copy	plantUBXdomain-containingprotein2
Aradu.B78C7	Araip.V6D67	single-copy	plasma-membraneassociatedcation-bindingprotein1
Aradu.4BV7T	Araip.YCD8P	single-copy	plastidictypeisignalpeptidase1
Aradu.5H5FX	Araip.48FMM	single-copy	PLASTIDMOVEMENTIMPAIRED2-likeisoformX1[Glycine
Aradu.DK86D	Araip.91ECR	single-copy	PlastidribosomalproteinL1largeribosomalsubunitn=1Tax=
Aradu.5DE0L	Araip.IHF9W	single-copy	plastidtranscriptionallyactive14
Aradu.Q2AQJ	Araip.0R69M	single-copy	plectin-likeisoformX3[Glycinemax]
Aradu.0V3TN	Araip.89KY2	single-copy	pleiotropicdrugresistance12
Aradu.XZS36	Araip.85RP9	single-copy	poly(rC)-bindingprotein3-like[Glycinemax]
Aradu.5GH6D	Araip.I7JKU	single-copy	poly(U)-specificendoribonuclease-B-likeprotein
Aradu.UNB9U	Araip.JU13E	single-copy	polygalacturonaseQRT3-like[Glycinemax]
Aradu.D89KQ	Araip.7V77F	single-copy	polypyrimidinetract-bindingprotein1
Aradu.95FMB	Araip.YPU6N	single-copy	polyribonucleotidenucleotidyltransferase,putative
Aradu.WUH7T	Araip.FFV1Z	single-copy	post-GPIattachment-likefactor-protein
Aradu.F05R2	Araip.62ZJ7	single-copy	PotentialpeptideN-acetyltransferasen=1Tax=Pseudozyma
Aradu.KGT1D	Araip.ZDT9K	single-copy	PP2AregulatorysubunitTAP46-likeprotein
Aradu.5PB9J	Araip.5G7EV	single-copy	PPRsuperfamilyprotein
Aradu.S66GY	Araip.1S7CN	single-copy	PRA1(Prenylatedrabacceptor)familyprotein
Aradu.RYB7F	Araip.11Y3M	single-copy	PRD1-likeisoformX1[Glycinemax]
Aradu.NU5M2	Araip.QDA4J	single-copy	mRNAcleavagefactorcomplexcomponentPcf11n=4Tax=A
Aradu.7JW9M	Araip.H27NU	single-copy	prenylatedRABacceptor1.A1
Aradu.B3CRQ	Araip.THJ6R	single-copy	prenylyltransferasesuperfamily
Aradu.6GD1V	Araip.E8I2J	single-copy	PRKR-interactingprotein1-like[Glycinemax]
Aradu.N0QH3	Araip.K0390	single-copy	prohibitin3

Aradu.D8WVG	Araip.6ZX2I	single-copy	prolineiminopeptidase
Aradu.296RJ	Araip.G4AFR	single-copy	proline--tRNAligase-like[Glycinemax]
Aradu.6FW8M	Araip.NL6AM	single-copy	proteasomeassemblychaperone2-like[Glycinemax]
Aradu.GC5S7	Araip.RJB8C	single-copy	proteasomebetatype-3subunit
Aradu.S111G	Araip.N3P4J	single-copy	proteasomeinhibitor-related
Aradu.C4HNC	Araip.JV5C1	single-copy	ProteasomematurationfactorUMP1
Aradu.NG3QM	Araip.32MBN	single-copy	proteinFAR1-RELATEDSEQUENCE10-likeisoformX2[Gly
Aradu.RN1V3	Araip.608WW	single-copy	protein-l-isoaspartatemethyltransferase1
Aradu.C2F9N	Araip.YX3SF	single-copy	proteinn=1Tax=Oryzasativasubsp.japonicaRepID=C7J0D
Aradu.XL7AT	Araip.KA2EW	single-copy	proteinphosphatase2C59isoformX2[Glycinemax]
Aradu.W9GMQ	Araip.Q2M7H	single-copy	protein-proteininteractionregulatorfamilyprotein
Aradu.096TH	Araip.FCR1J	single-copy	proteinS-acyltransferase14-likeisoformX1[Glycinemax]
Aradu.34LNY	Araip.0W3FE	single-copy	proteoglycan4-likeisoformX2[Glycinemax]
Aradu.IGJ3I	Araip.2Y8GA	single-copy	protohemeIXfarnesyltransferase
Aradu.A8T4C	Araip.2RQ0L	single-copy	protoporphyrinogenIXoxidase
Aradu.JJW6C	Araip.X4N9F	single-copy	purin-richalpha1
Aradu.1C7BQ	Araip.Z7A9P	single-copy	pyridinenucleotide-disulfideoxidoreductasefamilyprotein
Aradu.UA8T0	Araip.RB200	single-copy	Pyridoxal-5'-phosphate-dependentenzymefamilyprotein
Aradu.Z3Z17	Araip.TTC98	single-copy	PyridoxalbiosynthesislyasePdxSn=5Tax=ClostridiumRep
Aradu.X0HV8	Araip.CW1SE	single-copy	Pyridoxamine5'-phosphateoxidase-relatedFMN-bindingpr
Aradu.IY69R	Araip.T5402	single-copy	pyridoxinebiosynthesis1.1
Aradu.HZ9T9	Araip.KY48S	single-copy	pyrrolidone-carboxylatepeptidase
Aradu.SHH9J	Araip.N2170	single-copy	pyruvate,phosphatedikinaseregulatoryprotein,putative
Aradu.GP04Q	Araip.N3HNT	single-copy	pyruvatedecarboxylase-2
Aradu.T8J76	Araip.ZSU41	single-copy	queuinetRNA-ribosyltransferase
Aradu.UM7TW	Araip.UIW35	single-copy	quinolinatesynthase
Aradu.D7HWB	Araip.DAZ2J	single-copy	R3H-associatedamine-terminaldomainprotein
Aradu.CN1WR	Araip.E8UKB	single-copy	RABgeranylgeranyltransferasealphasubunit1
Aradu.SH4VE	Araip.D5TXM	single-copy	Rad23UVexcisionrepairproteinfamily
Aradu.PUZ2B	Araip.6VK7W	single-copy	RAD3-likeDNA-bindinghelicaseprotein
Aradu.CI9FK	Araip.BJ5NT	single-copy	RADIALIS-like[Glycinemax]
Aradu.Q8JL3	Araip.LBU7J	single-copy	Raffinosesynthasefamilyprotein
Aradu.E3I48	Araip.RV8TN	single-copy	RanBP1domainprotein
Aradu.I9SXC	Araip.Y6U2J	single-copy	RAPannotationrelease2,NERDdomainprotein
Aradu.J7EAE	Araip.KG5RF	single-copy	ras-GTPase-activatingprotein-bindingprotein,putative
Aradu.KX0SA	Araip.03MS6	single-copy	RasproteinRab7,putative
Aradu.6P19N	Araip.7MX4C	single-copy	recQ-mediatedgenomeinstabilityprotein1-like[Glycinemax
Aradu.J7CRS	Araip.970Q7	single-copy	redchlorophyllcatabolitereductase,putative
Aradu.YP0LD	Araip.0IL8D	single-copy	regulationofnuclearpre-mRNAdomain-containingprotein1I
Aradu.35P83	Araip.6CP6H	single-copy	regulatorofnonsensetranscriptsUPF3-likeisoformX3[Glyci
Aradu.E8BQZ	Araip.X6QYG	single-copy	regulatoryprotein(NPR1)
Aradu.6LZ7W	Araip.R7BSY	single-copy	relatedtoubiquitin1
Aradu.0H4SB	Araip.FRL5W	single-copy	releasefactorglutaminemethyltransferase
Aradu.L5EJ3	Araip.EK4ZS	single-copy	resistancetophytophthora1

Aradu.5S25K	Araip.P1ARW	single-copy	Restrictionendonuclease,typeII-likesuperfamilyprotein
Aradu.RBQ3X	Araip.9HL1N	single-copy	retinoblastoma-related1
Aradu.CZ3TW	Araip.4KU76	single-copy	reversion-TO-ethyleneSENSITIVITY
Aradu.32A9T	Araip.L30PN	single-copy	RGPR-related
Aradu.Z07JX	Araip.L12RT	single-copy	RhodospirillumphotometricumDSM122draftgenomeseque
Aradu.Z6Z82	Araip.V7QJP	single-copy	Riboflavinkinase/FMNadenylyltransferasen=19Tax=Coryn
Aradu.S3DH3	Araip.GY1A9	single-copy	ribonucleoside-diphosphatereductasesmallchain-like[Glyc
Aradu.83I6G	Araip.X4PFH	single-copy	ribose-phosphatepyrophosphokinase
Aradu.FK0J4	Araip.Z370E	single-copy	ribosomalRNAprocessingprotein36homologisoformX1[Gly
Aradu.FNB9W	Araip.2A9KZ	single-copy	RibosomalsilencingfactorRsfSn=2Tax=BartonellaRepID=
Aradu.U3B0I	Araip.S2FNK	single-copy	ribosome-bindingfactorAfamilyprotein
Aradu.I1DRB	Araip.6ZN4Z	single-copy	ribosomeproductionfactor-likeprotein
Aradu.U70TW	Araip.7UJ60	single-copy	RIC1homologisoform1[Glycinemax]
Aradu.6MH23	Araip.IF4PZ	single-copy	RIK-likeisoformX1[Glycinemax]
Aradu.N5BSE	Araip.6M6IA	single-copy	RmlC-likecupinssuperfamilyprotein
Aradu.D464G	Araip.79PEZ	single-copy	RNA3-terminalphosphatecyclase-likeprotein,putative
Aradu.NS7I7	Araip.6D36H	single-copy	RNA-directedDNAmethylationprotein
Aradu.FU7NG	Araip.1Y0FU	single-copy	RNA-directedDNApolymerase(reversetranscriptase)
Aradu.IYZ29	Araip.V3SRF	single-copy	RNApseudouridinesynthase
Aradu.QA0D1	Araip.JQB3R	single-copy	RNasePRpr2/Rpp21subunitdomainprotein
Aradu.32EAS	Araip.JP7VM	single-copy	rootletin-likeisoformX3[Glycinemax]
Aradu.Q4VIY	Araip.L270U	single-copy	RRP12-likeprotein
Aradu.JRR3K	Araip.X2DNI	single-copy	Rubredoxin-likesuperfamilyprotein
Aradu.7BG9P	Araip.GX6C6	single-copy	RWDdomain-containingprotein
Aradu.MTE62	Araip.C53RL	single-copy	Saccharopinedehydrogenase
Aradu.8Z9JB	Araip.VH74R	single-copy	salicylicacidcarboxylmethyltransferase
Aradu.78ZXW	Araip.4LG3M	single-copy	SAM-dependentmethyltransferase,MraWmethylasefamily
Aradu.EZ4BH	Araip.3X1VK	single-copy	SBP(S-ribonucleasebindingprotein)familyprotein
Aradu.4MN5D	Araip.0NQ3E	single-copy	SCAIhomologisoformX1[Glycinemax]
Aradu.T2BI1	Araip.76R9S	single-copy	SCO1homolog2,mitochondrial-like[Glycinemax]
Aradu.M5LK1	Araip.6T0FQ	single-copy	SCP1-likesmallphosphatase5
Aradu.QE1YJ	Araip.3U41R	single-copy	scramblase-related
Aradu.T6EFY	Araip.B1ALG	single-copy	secondarythiamine-phosphatesynthaseenzyme
Aradu.EV49X	Araip.P5P82	single-copy	sedoheptulose-bisphosphatase
Aradu.HX36X	Araip.DK4JW	single-copy	seedbiotin-containingproteinSBP65[Glycinemax]
Aradu.0GK1P	Araip.VPK3J	single-copy	Sel1repeatprotein
Aradu.3AT2D	Araip.YTI05	single-copy	selenium-bindingprotein1
Aradu.XT8CD	Araip.Y7X9R	single-copy	selenoproteinO-like[Glycinemax]
Aradu.TJC58	Araip.P3X4Z	single-copy	selT-likeprotein-like[Glycinemax]
Aradu.LXN93	Araip.ZN0SC	single-copy	senescence-induciblechloroplaststay-greenprotein2[Glyci
Aradu.YS8K9	Araip.EB7GH	single-copy	septumsite-determiningprotein(MIND)
Aradu.G8908	Araip.ZJ8QE	single-copy	Serinc-domaincontainingserineandsphingolipidbiosynthes
Aradu.9R9X3	Araip.1117E	single-copy	serine-glyoxylateaminotransferase-likeprotein
Aradu.3J95M	Araip.G0AQ4	single-copy	serine-threoninekinasereceptor-associatedprotein-like[Gly

Aradu.YSN3X	Araip.DXU98	single-copy	serrateRNAeffectormolecule-likeprotein
Aradu.M530F	Araip.12PK8	single-copy	set1/Ash2histonemethyltransferasecomplexsubunitASH2
Aradu.MY1HL	Araip.2AE2F	single-copy	seventransmembranedomainprotein
Aradu.PS0E5	Araip.K2IF4	single-copy	SH3domain-containingprotein
Aradu.UQP4Y	Araip.NXK65	single-copy	shikimateO-hydroxycinnamoyltransferase[Glycinemax]
Aradu.Y178G	Araip.MF65Y	single-copy	shortageinchiasmata1
Aradu.T08NC	Araip.5J1JM	single-copy	SimilartoMaltoseexcessprotein1
Aradu.VVM35	Araip.IK2WW	single-copy	S-isoprenylcysteineO-methyltransferase
Aradu.36SKU	Araip.4NG5J	single-copy	smallmulti-drugexportprotein,putative
Aradu.TI81R	Araip.K6C0S	single-copy	smallubiquitin-likemodifier2
Aradu.TV1GT	Araip.85X1W	single-copy	SMG9-likeprotein
Aradu.B5JI0	Araip.TK8D6	single-copy	SNI1isoformX1[Glycinemax]
Aradu.IQK2Q	Araip.H09FU	single-copy	snurportin-1-like[Glycinemax]
Aradu.939VA	Araip.M76BN	single-copy	sodium/calciumexchangerfamilyprotein/calcium-bindingE
Aradu.IS9ZH	Araip.99NVB	single-copy	sodiumhydrogenexchanger2
Aradu.CXJ5P	Araip.4N7WF	single-copy	solanesyldiphosphatesynthase1
Aradu.EF798	Araip.A05V1	single-copy	Solublediacylglycerolacyltransferasen=2Tax=Arachishyp
Aradu.76BXR	Araip.NXE7U	single-copy	somethingaboutsilencingprotein10-likeisoformX2[Glycine
Aradu.82P95	Araip.4PT6K	single-copy	sortingandassemblymachinerycomponent50homolog[Gly
Aradu.DLS5S	Araip.7W817	single-copy	sortingnexin1
Aradu.FCK70	Araip.R004A	single-copy	Spc97/Spc98familyofspindlepolebody(SBP)component
Aradu.8E0AS	Araip.XP5YM	single-copy	specifictissueprotein
Aradu.5K9HV	Araip.KLV48	single-copy	sphingolipiddeltadesaturase
Aradu.9I2P6	Araip.V5ZSM	single-copy	Sphingomyelinsynthetasefamilyproteinisoform1n=1Tax=T
Aradu.SBS1U	Araip.4JZ1Y	single-copy	sphingosinekinase1
Aradu.FA889	Araip.KCY11	single-copy	spindleandkinetochore-associated-likeprotein
Aradu.XA5F9	Araip.Z8S2H	single-copy	SPla/RYanodinereceptor(SPRY)domain-containingprotei
Aradu.EV2Z9	Araip.TI2C9	single-copy	SPOCdomain/TranscriptionelongationfactorS-IIprotein
Aradu.1M9NC	Araip.04KZI	single-copy	sporulation-specificprotein15-likeisoformX3[Glycinemax]
Aradu.SG94C	Araip.7DQ34	single-copy	squalenemonooxygenase2
Aradu.J7DHV	Araip.I4GCK	single-copy	Starch-bindingdomain/4-alpha-glucanotransferasemulti-d
Aradu.K13LE	Araip.IH553	single-copy	starchbranchingenzymeI
Aradu.CW6KA	Araip.GG51M	single-copy	StAR-relatedlipidtransferprotein9,putativen=1Tax=Theob
Aradu.0Q24B	Araip.PIW91	single-copy	sterol3-beta-glucosyltransferaseUGT80A2-likeisoformX1
Aradu.ICY54	Araip.V7N0F	single-copy	Stigma-specificStig1familyprotein
Aradu.F7E59	Araip.4UB04	single-copy	stressenhancedprotein2,chloroplastic-like[Glycinemax]
Aradu.QU7BE	Araip.7W8RG	single-copy	stressresponseproteinNST1-like[Glycinemax]
Aradu.H3LPD	Araip.RWC6Y	single-copy	stromalcell-derivedfactor-likeprotein
Aradu.D0RIM	Araip.13HZD	single-copy	structuralconstituentofcellwallprotein,putative
Aradu.1Z4K3	Araip.R452I	single-copy	Structuralmolecule,putativen=1Tax=RicinuscommunisRe
Aradu.EVB44	Araip.638DS	single-copy	structure-specificendonucleasesubunitSLX1-likeprotein
Aradu.CP5FF	Araip.NLK3G	single-copy	Sua5/YciO/YrdC/YwlCfamilyproteinn=12Tax=Bacteroides
Aradu.XC8VW	Araip.Q3PHC	single-copy	substratecarrierfamilyprotein
Aradu.J16T3	Araip.E7852	single-copy	Succinyl-CoAligase,alphasubunit

Aradu.V0VX5	Araip.X4EHZ	single-copy	sucrosenonfermenting4-likeprotein-likeisoformX1[Glycine
Aradu.WZ2NF	Araip.U7E4D	single-copy	sulfiredoxin
Aradu.NFI11	Araip.X503X	single-copy	sulfotransferase2A
Aradu.J5HFQ	Araip.K9WKL	single-copy	SUNdomain-containingprotein1-likeisoformX3[Glycinema
Aradu.D7KSP	Araip.8Y60Z	single-copy	suppressorproteinSRP40-like[Glycinemax]
Aradu.1QD6E	Araip.1S76R	single-copy	Surfeitlocus1cytochromecoxidasebiogenesisprotein
Aradu.V1JTR	Araip.E6C5N	single-copy	SURPandG-patchdomain-containingprotein1-likeprotein-l
Aradu.7R439	Araip.1A9I8	single-copy	SWI3,ADA2,N-CoRandtfiiibDNA-bindingdomainprotein
Aradu.L0TFU	Araip.2GK61	single-copy	syringolide-inducedprotein14-1-1[Glycinemax]
Aradu.QQX3T	Araip.T1WWY	single-copy	TatDrelatedDNase
Aradu.R7YY4	Araip.40JWQ	single-copy	TelomeraseactivatingproteinEst1
Aradu.Y28R7	Araip.YA4GL	single-copy	temperature-inducedlipocalin
Aradu.VW9JZ	Araip.L6WEG	single-copy	tetraacyldisaccharide4'-kinasefamilyprotein
Aradu.LVM3Z	Araip.FKG2G	single-copy	TGA1-likeisoformX7[Glycinemax]
Aradu.AXU8L	Araip.14RNX	single-copy	TGF-beta-induciblenuclearprotein,putativen=7Tax=Entam
Aradu.YPS83	Araip.LR9VJ	single-copy	ThiaminepyrophosphateenzymeTPPbindingdomainprotei
Aradu.71LF7	Araip.5V540	single-copy	thiaminpyrophosphokinase2
Aradu.6V2BQ	Araip.B7NZU	single-copy	Thioesterasesuperfamilyprotein
Aradu.0ME59	Araip.U7HDR	single-copy	THOcomplex,subunit5
Aradu.ZBZ36	Araip.Z30L7	single-copy	threoninesynthase-likeprotein
Aradu.WIA05	Araip.JK7V6	single-copy	Thymidinekinase
Aradu.70A8U	Araip.FME0N	single-copy	Thymidylatesynthasen=2Tax=PseudomonasRepID=S6J7
Aradu.ZGD79	Araip.QKJ0T	single-copy	TIM-barrelsignaltransductionproteinn=6Tax=Bradyrhizobi
Aradu.C718S	Araip.G2LIW	single-copy	timelessfamilyprotein
Aradu.X689P	Araip.L11FI	single-copy	titan9,putative
Aradu.DLR8J	Araip.10A0F	single-copy	TMPIT-likeprotein
Aradu.HK1JB	Araip.Q75IR	single-copy	tocopherolcyclase
Aradu.02I1H	Araip.E67FB	single-copy	TONSOKU-likeisoformX2[Glycinemax]
Aradu.K7Z0H	Araip.7MT36	single-copy	TOPLESS-related1
Aradu.JK5YQ	Araip.D13ZN	single-copy	TPLATE-like[Glycinemax]
Aradu.U0DT9	Araip.I6NSD	single-copy	transaldolasefamilyprotein
Aradu.IAE5L	Araip.Z2B8F	single-copy	Transcripteionfactorn=1Tax=MedicagotruncatulaRepID=(
Aradu.61NAP	Araip.NM49E	single-copy	transcriptionactivators
Aradu.9X2TD	Araip.76TXX	single-copy	transcriptionalregulatorfamilyprotein
Aradu.IW8J6	Araip.12A3Y	single-copy	transcriptionregulators
Aradu.KEZ8L	Araip.0W0BL	single-copy	transformation/transcriptiondomainassociatedprotein
Aradu.IS5YT	Araip.QYZ6U	single-copy	Transketolase
Aradu.HQ5WT	Araip.PU5CI	single-copy	TranslationinitiationfactorSUI1familyprotein
Aradu.2P97F	Araip.W60W5	single-copy	translocaseofchloroplast132,chloroplastic-like[Glycinema
Aradu.82TF8	Araip.T2HWD	single-copy	Translocon-associatedprotein(TRAP),alphasubunit
Aradu.GP5WA	Araip.A01I6	single-copy	transloconatinnermembraneofchloroplasts21
Aradu.EH0QA	Araip.G3FGY	single-copy	transmembrane45B-likeprotein
Aradu.747H9	Araip.IID88	single-copy	transmembraneandcoiled-coildomain-containingprotein4-
Aradu.1SS2C	Araip.6B9LC	single-copy	transmembraneemp24domain-containingproteinp24beta2

Aradu.0YP72	Araip.FL5I2	single-copy	TransmembraneFragile-X-F-associatedprotein
Aradu.CMZ60	Araip.W8NVJ	single-copy	TRANSPARENTTESTA1-like[Glycinemax]
Aradu.B8U65	Araip.BJ25J	single-copy	transporterarsB-likeisoformX4[Glycinemax]
Aradu.27XB8	Araip.URJ02	single-copy	transportin1
Aradu.XIY30	Araip.RF6HA	single-copy	trehalase1
Aradu.85E8I	Araip.ZND2G	single-copy	trehalose-6-phosphatephosphatase
Aradu.A7Z3C	Araip.US4V4	single-copy	trehalose-6-phosphatesynthase
Aradu.JFD4U	Araip.QW4F4	single-copy	triggerfactor-likeprotein
Aradu.2QR5U	Araip.EC2BK	single-copy	trihelixtranscriptionfactor[Glycinemax]
Aradu.7I20U	Araip.W9YFB	single-copy	triosephosphateisomerase
Aradu.X44N9	Araip.Z3F8H	single-copy	tRNA(adenine(58)-N(1))-methyltransferasenon-catalyticsu
Aradu.E2AL2	Araip.1U1PJ	single-copy	tRNA(his)guanylyltransferase
Aradu.JFH3E	Araip.M52X2	single-copy	tRNAdimethylallyltransferase
Aradu.725QH	Araip.SS0DN	single-copy	tRNAguanosine-2'-O-methyltransferaseTRM13-likeprotei
Aradu.KB85J	Araip.K7EHW	single-copy	tRNApseudouridinesynthaseB
Aradu.L3K73	Araip.LA07L	single-copy	tRNA-specific2-thiouridylaseMnmA
Aradu.3Q7E3	Araip.42FFR	single-copy	tRNA-splicingendonucleasesubunitSen54-likeisoformX2[
Aradu.T346F	Araip.DY7BJ	single-copy	tRNAuridine5-carboxymethylaminomethylmodificationenz
Aradu.JY1UA	Araip.Y533V	single-copy	tryptophanaminotransferaserelated2
Aradu.M5WCG	Araip.U5NBJ	single-copy	TSPO(outermembranetryptophan-richsensoryprotein)-rel
Aradu.PM3IW	Araip.DQ2EG	single-copy	Tubulin-specificchaperoneAn=2Tax=MalvaceaeRepID=M
Aradu.PLM7U	Araip.ML7EU	single-copy	tubulin-tyrosineligasefamilyprotein
Aradu.TLI73	Araip.Q12S9	single-copy	TWINLOVprotein
Aradu.NBA8B	Araip.SX347	single-copy	twoporecalciumchannelprotein,putative
Aradu.3W0JX	Araip.PX9HF	single-copy	tyrosineaminotransferase3
Aradu.NK3YY	Araip.SG7HP	single-copy	Tyrosinephosphatasefamilyprotein
Aradu.04QLM	Araip.RX21A	single-copy	tyrosinesulfotransferase-likeprotein
Aradu.N76GN	Araip.X45KN	single-copy	U2smallnuclearribonucleoproteinB
Aradu.D9IA6	Araip.E13TP	single-copy	U2snRNPauxiliaryfactorsmallsubunit,putative
Aradu.08LLU	Araip.M4B3A	single-copy	U4/U6smallnuclearribonucleoproteinPRP4-likeprotein-like
Aradu.67UXU	Araip.VA0EM	single-copy	U4/U6XU5tri-snRNPcomplexsubunitPrp31n=1Tax=Schiz
Aradu.QV2N6	Araip.FB3XS	single-copy	u6snRNA-associated-like-Smprotein
Aradu.SD45B	Araip.7H6FH	single-copy	UbiAprenyltransferasefamilyprotein
Aradu.ZPB6A	Araip.9A6FH	single-copy	Ubiquinol-cytochromeCreductaseiron-sulfursubunit
Aradu.6S44C	Araip.KLH55	single-copy	ubiquinone/menaquinonebiosynthesismethyltransferase
Aradu.LA26X	Araip.T4R10	single-copy	ubiquinonebiosynthesismonooxygenaseCOQ6-likeprotei
Aradu.J6MLG	Araip.TL7VJ	single-copy	UbiquinonebiosynthesisO-methyltransferasen=2Tax=Ba
Aradu.UVJ1U	Araip.C8DTK	single-copy	ubiquitinactivatingenzyme2
Aradu.L2BKM	Araip.PG9PR	single-copy	ubiquitin-bindingWIYLDdomainprotein
Aradu.2N8AH	Araip.GS1GM	single-copy	ubiquitininteractionmotif-containingprotein
Aradu.XW1R1	Araip.D5WHX	single-copy	UbiquitinligaseSCFcomplexsubunitcullinn=1Tax=Chlamy
Aradu.CJQ5M	Araip.6M87Y	single-copy	ubiquitin-likedomainCTDphosphatase
Aradu.ER2GU	Araip.Y9ZBX	single-copy	ubiquitin-likemodifier-activatingenzyme
Aradu.JT96L	Araip.67K5R	single-copy	UB-likeprotease1A

Aradu.FF3VB	Araip.74IBX	single-copy	UDP-glucosepyrophosphorylase3
Aradu.VFN3G	Araip.EER3Y	single-copy	UDP-glucosyltransferasefamilyprotein
Aradu.PK7XR	Araip.Q8CS4	single-copy	UDP-N-acetylglucosaminepyrophosphorylasen=2Tax=Ps
Aradu.V1H1X	Araip.L03GB	single-copy	UDP-N-acetylmuramoyl-L-alanyl-D-glutamate--2,6-diamir
Aradu.E8W2B	Araip.8SS4C	single-copy	UNUSUALFLORALORGANS-like[Glycinemax]
Aradu.LWE3F	Araip.R46GQ	single-copy	uracildnaglycosylase
Aradu.1E0WP	Araip.PLC3S	single-copy	Ureaactivetransportproteinn=1Tax=Spathasporapassalida
Aradu.29QNA	Araip.E96SM	single-copy	ureaseaccessoryproteinG
Aradu.N1D5Q	Araip.D98D3	single-copy	ureidepermease1
Aradu.IXS5D	Araip.K8WAH	single-copy	ureidoglycineaminohydrolase
Aradu.KP7PE	Araip.UM9DW	single-copy	uricase-2isozyme2[Glycinemax]
Aradu.UM9US	Araip.Z77CR	single-copy	Uroporphyrinogendecarboxylase
Aradu.U7HHN	Araip.Q0CU4	single-copy	uroporphyrinogen-IIIC-methyltransferase
Aradu.810XL	Araip.NV5LW	single-copy	UTP-glucose-1-phosphateuridylyltransferase
Aradu.1ED5Z	Araip.TJ4SI	single-copy	uv-b-insensitive4
Aradu.X8KRI	Araip.VQH1V	single-copy	UvrB/uvrCdomainprotein
Aradu.P6GSC	Araip.6S3BR	single-copy	UV-stimulatedscaffoldA-likeprotein
Aradu.2RC64	Araip.I88R2	single-copy	vacuolarcation/protonexchanger3
Aradu.8R2K8	Araip.SV7HB	single-copy	vacuolarfusionproteinCCZ1homologB-likeisoformX3[Glyc
Aradu.DWF95	Araip.ZLP0T	single-copy	Vacuolarirontransporter(VIT)familyprotein
Aradu.EAG5M	Araip.B069Y	single-copy	VACUOLARSORTINGRECEPTOR2
Aradu.1FC14	Araip.1VK73	single-copy	vacuolemembrane-likeprotein
Aradu.802R8	Araip.7P115	single-copy	Vacuolarsortingprotein39
Aradu.T0ZKV	Araip.2M1DM	single-copy	vegetativecellwallproteingp1-like[Glycinemax]
Aradu.G2R4U	Araip.JH1AW	single-copy	VHSdomain-containingproteinAt3g16270-like[Glycinemax
Aradu.F433U	Araip.QU0FD	single-copy	vicilin-likeantimicrobialpeptides2-2-like[Glycinemax]
Aradu.XLR8N	Araip.J2QMS	single-copy	VIN3-likeprotein1-likeisoformX2[Glycinemax]
Aradu.U033V	Araip.6D6XW	single-copy	voltage-gatedpotassiumchannelsubunitbeta
Aradu.YPI85	Araip.3M1WU	single-copy	Vps52/Sac2family
Aradu.889PJ	Araip.WB46L	single-copy	Wernersyndrome-likeexonuclease
Aradu.NM2TY	Araip.E7MNJ	single-copy	WVD2-like1-likeisoformX1[Glycinemax]
Aradu.WB45H	Araip.99J68	single-copy	WWdomain-bindingprotein
Aradu.KWP32	Araip.MP5EW	single-copy	Xaa-proaminopeptidaseP
Aradu.RZ908	Araip.15C6I	single-copy	XH/XSdomain-containingprotein
Aradu.QJD0U	Araip.LNT1B	single-copy	xyloglucangalactosyltransferaseKATAMARI1homolog[Gly
Aradu.M3KDI	Araip.YE9C6	single-copy	xylulosekinase-2
Aradu.S6CPD	Araip.K4HRJ	single-copy	ycf20-likeprotein-like[Glycinemax]
Aradu.ZAK9S	Araip.B8RZP	single-copy	YEATSfamilyprotein
Aradu.747VP	Araip.4Z07Z	single-copy	Y-familyDNApolymeraseH
Aradu.QX6W8	Araip.JK2QJ	single-copy	YGGTfamilyprotein
Aradu.M4MQC	Araip.ZQQ7K	single-copy	YGL010w-likeprotein
Aradu.270HA	Araip.WJB0A	single-copy	YIF1B-likeisoformX3[Glycinemax]
Aradu.7X679	Araip.SD6PL	single-copy	yippee-likeisoform2[Glycinemax]
Aradu.XU97Z	Araip.EHI93	single-copy	Ypt/Rab-GAPdomainofgyp1psuperfamilyprotein

Aradu.EQE9W	Araip.ZW87C	single-copy	zinccarboxypeptidasefamilyprotein
Aradu.YZ24M	Araip.BJ6HU	single-copy	zincfingerwithUFM1-specificpeptidasedomainprotein
Aradu.QA44E	Araip.TL9EX	single-copy	Ziniontransmembranetransportern=2Tax=MedicagoRep
Aradu.LXL9C	Araip.7M8Z5	single-copy	Zincknucklefamilyproteinn=1Tax=Oryzasativasubsp.japo
Aradu.YV9QI	Araip.M6NPA	single-copy	Zn-dependenthydrolaseofthebeta-lactamasefoldprotein
Aradu.A22XE	Araip.13N2J	multiple-copy	1,4-alpha-glucan-branchingenzyme/amyloplasticprotein
Aradu.H0G14	Araip.17941	multiple-copy	12-oxophytodienoatereductase2
Aradu.V2AUT	Araip.C26XV	multiple-copy	1-acyl-sn-glycerol-3-phosphateacyltransferase
Aradu.WC1MX	Araip.337EV	multiple-copy	1-deoxy-D-xylulose5-phosphatesynthase1
Aradu.ED9QM	Araip.M733F	multiple-copy	3beta-hydroxysteroid-dehydrogenase/decarboxylaseisofo
Aradu.R42GY	Araip.M64Q1	multiple-copy	3-dehydroquinatedehydratasen=2Tax=StreptomycesRep
Aradu.H88LD	Araip.5ZJ1R	multiple-copy	3-hydroxy-3-methylglutaryl-coenzymeAreductase-likeprot
Aradu.07VYH	Araip.5MC2N	multiple-copy	3-hydroxyacyl-[acyl-carrier-protein]dehydrataseFabZn=2T
Aradu.2Q562	Araip.LWG2P	multiple-copy	3-isopropylmalatedehydratase,largesubunit
Aradu.WLL49	Araip.NZ168	multiple-copy	4-hydroxy-tetrahydrodipicolinatereductase2,chloroplastic-
Aradu.UTZ1D	Araip.CF2W6	multiple-copy	5-hydroxyisouratehydrolase
Aradu.ILV24	Araip.57S0G	multiple-copy	60Sacidicribosomalproteinfamily
Aradu.X51AF	Araip.7MI21	multiple-copy	7SKsnRNAmethylphosphatecappingenzymen=3Tax=Bor
Aradu.KX34B	Araip.R34I7	multiple-copy	aarFdomain-containingproteinkinase1[Glycinemax]
Aradu.C4MHC	Araip.3HN76	multiple-copy	ABIL1-like[Glycinemax]
Aradu.EG2K3	Araip.M1IPR	multiple-copy	abscisicacidresponsiveelements-bindingfactor3
Aradu.Z0G82	Araip.77JRH	multiple-copy	acetyl-CoAcarboxylase,carboxyltransferase,alphasubunit
Aradu.RS6VM	Araip.L3LJP	multiple-copy	Actin-relatedproteinArp2/3complex,subunitArp3n=6Tax=U
Aradu.JV5YL	Araip.GTR8C	multiple-copy	acylthioesterase-likeprotein
Aradu.MVW70	Araip.STT9U	multiple-copy	Adaptinear-bindingcoat-associatedprotein1NECAP-1
Aradu.W2FE7	Araip.Y1DIL	multiple-copy	adenylatecyclase
Aradu.74KVK	Araip.348XC	multiple-copy	adipose-regulatoryprotein(Seipin)
Aradu.A9RVD	Araip.K1YWU	multiple-copy	ADP,ATPcarrierprotein1,mitochondrial-like[Glycinemax]
Aradu.F3I3T	Araip.GEK49	multiple-copy	AFG1-likeATPasefamilyprotein
Aradu.D20L9	Araip.93XAK	multiple-copy	alanineaminotransferase2
Aradu.6W9YP	Araip.ZDZ6L	multiple-copy	alkalinephytoceramidase
Aradu.PZ18V	Araip.4J20T	multiple-copy	alkylatedDNArepairproteinalkBhomolog8-likeisoformX2[G
Aradu.H2RVW	Araip.673NH	multiple-copy	alleneoxidecyclase4
Aradu.72HG2	Araip.E0IYQ	multiple-copy	alleneoxidesynthase
Aradu.QVN0R	Araip.EMI4R	multiple-copy	alpha-1,4-glucan-proteinsynthase[UDP-forming]-likeprote
Aradu.E4PH8	Araip.MD98T	multiple-copy	alpha-amylase-like2
Aradu.AAT7R	Araip.I13PT	multiple-copy	alphacarbonicanhydrase7
Aradu.J8VZJ	Araip.RZ2UN	multiple-copy	alpha-L-arabinofuranosidase1
Aradu.EZ25P	Araip.N0TEM	multiple-copy	alpha-mannosidase1
Aradu.CZ128	Araip.YC2CD	multiple-copy	alternativeoxidase2
Aradu.AF0JE	Araip.TYL9N	multiple-copy	Aminoacidpermeasefamilyprotein
Aradu.IT4JF	Araip.ER2KN	multiple-copy	aminoacyl-tRNAsynthetase
Aradu.20FIR	Araip.BC51L	multiple-copy	AMPdeaminase-like[Glycinemax]
Aradu.NE9V5	Araip.21RG8	multiple-copy	ankyrinrepeatandBTB/POZdomainprotein

Aradu.Z4EVV	Araip.NBK0L	multiple-copy	apyrase2
Aradu.NQ7IA	Araip.H4L5F	multiple-copy	Aquaporin-likesuperfamilyprotein
Aradu.VBY1Y	Araip.9EA3M	multiple-copy	Arabidopsisphospholipase-likeprotein(PEARLI4)family
Aradu.N5BC9	Araip.543CV	multiple-copy	ARID/BRIGHTDNA-bindingdomain-containingprotein
Aradu.T3VDH	Araip.QP2XD	multiple-copy	ascorbateperoxidase3
Aradu.50C9M	Araip.M9DFJ	multiple-copy	asparaginesynthetase3
Aradu.RKM5L	Araip.J6CZK	multiple-copy	Aspartatekinase3isoform1n=2Tax=TheobromacacaoRep
Aradu.G9DQQ	Araip.96W3Q	multiple-copy	aspartateracemase
Aradu.ZUQ2N	Araip.Y1D91	multiple-copy	aspartyl/glutamyl-tRNA(Asn/Gln)amidotransferasesubuni
Aradu.K3FG8	Araip.A9FL4	multiple-copy	ataxin-10-like[Glycinemax]
Aradu.7NY4Q	Araip.6IS7T	multiple-copy	ATPase,V0/A0complex,subunitC/D
Aradu.CKU4P	Araip.ZS4AK	multiple-copy	ATP-citratelyaseA-1
Aradu.2LH1B	Araip.27Q72	multiple-copy	ATP-dependentchaperoneClpB
Aradu.188J4	Araip.XP707	multiple-copy	ATP-dependentDNAhelicase,putativen=7Tax=Trypanoso
Aradu.A7CW2	Araip.RL56E	multiple-copy	ATP-dependenthelicaseBRM-likeisoformX4[Glycinemax]
Aradu.VFG89	Araip.CP17Q	multiple-copy	AWPM-19-likefamilyprotein
Aradu.88QB9	Araip.AG87Q	multiple-copy	basic7Sglobulin[Glycinemax]
Aradu.C4XJQ	Araip.R44YW	multiple-copy	BEE1-like[Glycinemax]
Aradu.MQT1Y	Araip.B6RJA	multiple-copy	beta-caroteneisomeraseD27,chloroplastic-like[Glycinema
Aradu.L7XAF	Araip.10KZ6	multiple-copy	BOI-relatedE3ubiquitin-proteinligase3-like[Glycinemax]
Aradu.XJ3JE	Araip.FRI7H	multiple-copy	breastcarcinomaamplifiedsequence3protein
Aradu.49T9W	Araip.QK3HR	multiple-copy	bromo-adjacenthomology(BAH)domain-containingprotein
Aradu.RE8D2	Araip.DQ5V8	multiple-copy	BTBandTAZdomainprotein1
Aradu.I572Z	Araip.G99ZR	multiple-copy	C2andGRAMdomain-containingproteinAt1g03370-like[Gl
Aradu.J1JIJ	Araip.1G19U	multiple-copy	caffeoylshikimateesterase-likeisoformX1[Glycinemax]
Aradu.N3KHF	Araip.64IP1	multiple-copy	Calcium-bindingendonuclease/exonuclease/phosphatase
Aradu.XR3BY	Araip.7YJ6V	multiple-copy	calcium-bindingmitochondrialcarrierproteinSCaMC-1-like[
Aradu.503J9	Araip.36RIF	multiple-copy	callosesynthase5
Aradu.B7233	Araip.G8H3T	multiple-copy	carbamoyl-phosphatesynthaselargechain
Aradu.FU605	Araip.B1BGN	multiple-copy	CASP-likeproteinSb05g021340-like[Glycinemax]
Aradu.V7W75	Araip.A5QSA	multiple-copy	catalyticLigBsubunitofaromaticring-openingdioxygenasefa
Aradu.JQ8WR	Araip.026EM	multiple-copy	cationcalciumexchanger4
Aradu.YXG3J	Araip.X43U5	multiple-copy	Cationeffluxfamilyprotein
Aradu.304FM	Araip.K5A40	multiple-copy	CCR4-associatedfactor1homolog11-like[Glycinemax]
Aradu.MF07S	Araip.J2K2B	multiple-copy	Cellulase(glycosylhydrolasefamily5)protein
Aradu.E8PJN	Araip.E5RNZ	multiple-copy	centromereproteinS-likeisoformX3[Glycinemax]
Aradu.QE4AN	Araip.3R01Q	multiple-copy	chlorophyllideAoxygenase
Aradu.W3VUI	Araip.PPD7W	multiple-copy	chloroplasticgroupIIAintronsplicingfacilitatorCRS1,chlorop
Aradu.KV07Y	Araip.N9YA2	multiple-copy	Chloroplastoutermembraneprotein,putative,expressedn=3
Aradu.82T8W	Araip.JPK9F	multiple-copy	cholinetransporter-likeprotein2-like[Glycinemax]
Aradu.L2FW0	Araip.N76B5	multiple-copy	citrate-bindingprotein-like[Glycinemax]
Aradu.6WC85	Araip.TRT2Z	multiple-copy	citratesynthase3
Aradu.V3MUV	Araip.PJ6F5	multiple-copy	Clathrinadaptorcomplexesmediumsubunitfamilyprotein
Aradu.GT2DG	Araip.L7TNT	multiple-copy	clathrincoatassemblyproteinAP180-like[Glycinemax]

Aradu.7C3KS	Araip.Y00HL	multiple-copy	Coatomer,betasubunit
Aradu.DD5QZ	Araip.L0WI1	multiple-copy	cofactorassemblyofcomplexC
Aradu.FX5WG	Araip.7R33Q	multiple-copy	componentofhighaffinitynitratetransporter
Aradu.S7CRC	Araip.TK8K9	multiple-copy	condensin-2complexsubunitD3
Aradu.78C13	Araip.65HIY	multiple-copy	condensincomplexsubunit2
Aradu.KNK4F	Araip.PGV10	multiple-copy	copperionbinding
Aradu.0E5MF	Araip.YJC7I	multiple-copy	copper-transportingATPasePAA1,chloroplastic-like[Glycin
Aradu.ZU8HW	Araip.L0LZX	multiple-copy	C-terminaldomainphosphatase-like3
Aradu.9E8FC	Araip.LAW7P	multiple-copy	carboxy-terminalprocessingpeptidase-likeprotein
Aradu.NS580	Araip.QS8WY	multiple-copy	CTV.19n=1Tax=PoncirustrifoliataRepID=Q8H6Q9_PONT
Aradu.JQ7JG	Araip.P8SRT	multiple-copy	cystathioninebeta-lyase
Aradu.X6VZP	Araip.164TB	multiple-copy	cysteinedesulfurase-likeprotein
Aradu.A9CNL	Araip.E3K7S	multiple-copy	cysteine--tRNAligase,cytoplasmic-likeisoformX1[Glycinem
Aradu.D1XLL	Araip.G5VT6	multiple-copy	cystinosinhomologisoform1[Glycinemax]
Aradu.09F0B	Araip.1GQ6A	multiple-copy	cytochromecbiogenesisproteinfamily
Aradu.0U5ND	Araip.V5W4F	multiple-copy	cytochromeCoxidasesubunit5b
Aradu.B219P	Araip.K6VBU	multiple-copy	cytokininriboside5'-monophosphatephosphoribohydrolase
Aradu.AVD8P	Araip.3J27X	multiple-copy	cytoplasmic-likeaconitatehydratase
Aradu.7M1P4	Araip.AV0UY	multiple-copy	DA1-related1-likeisoformX4[Glycinemax]
Aradu.P9DB9	Araip.AM36D	multiple-copy	decapping5
Aradu.IF4XP	Araip.3269G	multiple-copy	Dehydrinfamilyprotein
Aradu.35HVS	Araip.CFP2Q	multiple-copy	Deoxyribodipyrimidinephoto-lyase(DNAphotolyase)(Photo
Aradu.DVL50	Araip.CF4RY	multiple-copy	DERLIN-1
Aradu.I3E1J	Araip.CF8RS	multiple-copy	desiccation-relatedproteinPCC13-62-like[Glycinemax]
Aradu.6I2MF	Araip.KVP0Y	multiple-copy	diacylglycerolacyltransferasefamily
Aradu.W9G7C	Araip.2528D	multiple-copy	diacylglycerolkinase2
Aradu.S1SNW	Araip.U9LFD	multiple-copy	Dihydrolipoyllysine-residuesuccinyltransferasecomponent
Aradu.NBA3F	Araip.QW087	multiple-copy	dihydroorotatedehydrogenase(quinone)
Aradu.05VAM	Araip.26Q67	multiple-copy	Dihydroxyacetonekinase
Aradu.89TY1	Araip.CI33Y	multiple-copy	diphosphate--fructose-6-phosphate1-phosphotransferase
Aradu.P28IF	Araip.JUS5G	multiple-copy	DNAGYRASEA
Aradu.L4C7Z	Araip.B8J3R	multiple-copy	Domainofunknownfunction(DUF23)
Aradu.F5KZV	Araip.6PA6C	multiple-copy	downstreamtargetofAGL15-4
Aradu.JN9TL	Araip.M301B	multiple-copy	DWNNdomain,aCCHC-typezincfinger
Aradu.DT0KY	Araip.AA1CY	multiple-copy	DYNAMIN-like1B
Aradu.2X0ZQ	Araip.JVY0J	multiple-copy	dynamin-like3
Aradu.GH306	Araip.Y472I	multiple-copy	earlylight-induced-likeprotein
Aradu.HG8ZF	Araip.35NZV	multiple-copy	EFhandcalcium-bindingfamilyprotein
Aradu.C1X9T	Araip.B52YR	multiple-copy	EIN3-bindingFboxprotein1
Aradu.P4R4T	Araip.P91HW	multiple-copy	ELF4-like1
Aradu.1I2B8	Araip.GVH79	multiple-copy	elongationfactorTuGTP-bindingdomainprotein
Aradu.6T5AB	Araip.MQ8B1	multiple-copy	elongatorcomplex-likeprotein
Aradu.0X9JH	Araip.K5R5A	multiple-copy	EMBRYONICFLOWER1-like[Glycinemax]
Aradu.KFD3G	Araip.0EV9Y	multiple-copy	embryo-specificprotein

Aradu.NNH8B	Araip.V9QLL	multiple-copy	endonucleaseorglycosylhydrolase
Aradu.MC16W	Araip.D3HJU	multiple-copy	enhancerofpolycombhomolog2-likeisoformX2[Glycinemax
Aradu.QWH3Y	Araip.GAR8H	multiple-copy	epsin-2-like[Glycinemax]
Aradu.JW08T	Araip.V9EKQ	multiple-copy	equilibrativenucleosidetransporter6
Aradu.U3SNU	Araip.B7KGV	multiple-copy	Ethyleneinsensitive3familyprotein
Aradu.1659P	Araip.B3H24	multiple-copy	ETO1-likeprotein1-likeisoformX1[Glycinemax]
Aradu.S8IEI	Araip.NLX79	multiple-copy	EXECUTER1,chloroplastic-like[Glycinemax]
Aradu.90TGE	Araip.IYZ3X	multiple-copy	externalalternativeNAD(P)H-ubiquinoneoxidoreductaseB
Aradu.V07RE	Araip.C4MN8	multiple-copy	FAD-dependentoxidoreductasefamilyprotein
Aradu.EG0P5	Araip.JAA8I	multiple-copy	Fasciclin-likearabinogalactanfamilyprotein
Aradu.RW69W	Araip.6LB90	multiple-copy	fattyacidamidehydrolase-like[Glycinemax]
Aradu.G1YNF	Araip.S3GXY	multiple-copy	fattyaciddesaturase2
Aradu.7HE2F	Araip.6R5A3	multiple-copy	fattyacidhydroxylase1
Aradu.3G1RX	Araip.J314H	multiple-copy	fission1protein
Aradu.68YSI	Araip.N0Z6R	multiple-copy	flavonolsynthase[Glycinemax]
Aradu.475M7	Araip.MEM8W	multiple-copy	floweringtimecontrolproteinFPA-like[Glycinemax]
Aradu.H6MX9	Araip.41DGV	multiple-copy	FMP32
Aradu.3GX35	Araip.S2XNF	multiple-copy	folate/biopterintransporter
Aradu.WS4CD	Araip.EM233	multiple-copy	formin8
Aradu.DZY4R	Araip.ET2IE	multiple-copy	forminhomolog6
Aradu.F6VM1	Araip.V3HZN	multiple-copy	FRIGIDA-like[Glycinemax]
Aradu.C5DXV	Araip.P4F2X	multiple-copy	fructose-bisphosphatealdolase2
Aradu.0FH1C	Araip.EK2ZC	multiple-copy	FUNCTIONSIN:molecular_functionunknown
Aradu.8S04V	Araip.71J30	multiple-copy	GAGA-bindingproteinisoformX3[Glycinemax]
Aradu.G4PX6	Araip.KNT6A	multiple-copy	galactinol--sucrosegalactosyltransferase2-likeisoformX1[
Aradu.EQ3HU	Araip.L3NWH	multiple-copy	galactinolsynthase1
Aradu.8Q0H7	Araip.5P589	multiple-copy	Galactose-bindingprotein
Aradu.H4IQD	Araip.7N7X7	multiple-copy	galactoside2-alpha-L-fucosyltransferase-likeprotein
Aradu.792L3	Araip.0HE2H	multiple-copy	gamete-likeprotein
Aradu.DVH8N	Araip.JHT8J	multiple-copy	gamma-glutamyltranspeptidase1
Aradu.ITR9J	Araip.HCQ42	multiple-copy	gammainterferoninduciblelysosomalthiolreductase
Aradu.VE1T0	Araip.3PJ33	multiple-copy	gar2-like[Glycinemax]
Aradu.1XB3Y	Araip.B118X	multiple-copy	GDP-L-galactosephosphorylase1-like[Glycinemax]
Aradu.Z7MCS	Araip.T2Z8Y	multiple-copy	geranylgeranylpyrophosphatesynthase1
Aradu.K84FP	Araip.A06C0	multiple-copy	gibberellin2-beta-dioxygenase8-like[Glycinemax]
Aradu.RF2LV	Araip.BG1FH	multiple-copy	globaltranscriptionfactorgroupprotein
Aradu.18DC6	Araip.4DN6E	multiple-copy	Glucose-1-phosphateadenylyltransferasefamilyprotein
Aradu.FZ0BJ	Araip.D19R8	multiple-copy	glucuronosyltransferasePGSIP6-likeisoformX1[Glycinem
Aradu.QYZ18	Araip.GFA2F	multiple-copy	glutamate-1-semialdehyde2,1-aminomutase2
Aradu.J9V4P	Araip.B1UDK	multiple-copy	glutamatedecarboxylase
Aradu.U8582	Araip.42EJ9	multiple-copy	Glutamyl-tRNAreductasefamilyprotein
Aradu.R659W	Araip.11YG0	multiple-copy	glutathionereductase
Aradu.T1G5I	Araip.J1I10	multiple-copy	glycine-richprotein
Aradu.78G42	Araip.0L8ZM	multiple-copy	Glycolipidtransferprotein(GLTP)familyprotein

Aradu.56PIZ	Araip.758EY	multiple-copy	glyoxaloxidase-relatedprotein
Aradu.MJZ2W	Araip.9Q1TC	multiple-copy	GolgiapparatusmembraneproteinTVP23homologBn=3Tax
Aradu.BTB7U	Araip.7N6M4	multiple-copy	Got1/Sft2-likevescicletransportproteinfamily
Aradu.N3TMI	Araip.4Z4V8	multiple-copy	GTE12-like[Glycinemax]
Aradu.16CAR	Araip.24N70	multiple-copy	GTPbinding
Aradu.WRA79	Araip.E4EEK	multiple-copy	Guanylate-bindingfamilyprotein
Aradu.Q9REZ	Araip.S1D0F	multiple-copy	H/ACAribonucleoproteincomplex,subunitGar1/Naf1protei
Aradu.PL5Z6	Araip.D0SBS	multiple-copy	Helicase-likeproteinn=1Tax=MedicagotruncatulaRepID=C
Aradu.1E0KB	Araip.DE8MF	multiple-copy	hemeoxygenase2
Aradu.7VL9B	Araip.9F87C	multiple-copy	heparan-alpha-glucosaminideN-acetyltransferase-likeprot
Aradu.T7BAA	Araip.ZQB6E	multiple-copy	HISTIDINETRIADNUCLEOTIDE-BINDING2
Aradu.E4N51	Araip.E0V6P	multiple-copy	histoneacetyltransferaseHAC1-likeisoformX2[Glycinemax
Aradu.J6DQU	Araip.E938G	multiple-copy	HMG-box(highmobilitygroup)DNA-bindingfamilyprotein
Aradu.19ZIS	Araip.G42NL	multiple-copy	Homeodomain-likesuperfamilyprotein
Aradu.X2HBL	Araip.AI3RV	multiple-copy	homoserinekinase
Aradu.RP1BS	Araip.0D136	multiple-copy	HR-likelesion-inducingprotein-related
Aradu.7KK7S	Araip.57HXG	multiple-copy	hydroxyacylglutathionehydrolase
Aradu.9A69L	Araip.E5HJE	multiple-copy	hydroxymethylglutaryl-CoAlyase
Aradu.UIC09	Araip.I90M3	multiple-copy	Importin(Ran-bindingprotein)n=1Tax=AnophelesdarlingiR
Aradu.3DC0K	Araip.2JH3B	multiple-copy	importinnermembranetranslocasesubunitTIM22
Aradu.04L8A	Araip.0Y7IG	multiple-copy	importinsubunitbeta-likeprotein
Aradu.Q95MC	Araip.24WNV	multiple-copy	inositol-tetrakisphosphate1-kinase1-likeisoformX1[Glycine
Aradu.H5EIL	Araip.PB4HL	multiple-copy	integratorcomplexsubunit3
Aradu.44R1C	Araip.LS077	multiple-copy	inter-alpha-trypsininhibitorheavychain-related
Aradu.4QT4U	Araip.UKK17	multiple-copy	isoflavonereductase-likeprotein-like[Glycinemax]
Aradu.54E80	Araip.I98ZE	multiple-copy	isopenicillinNepimerase-likeprotein
Aradu.4ME26	Araip.7RN1R	multiple-copy	isopentenyltransferase5
Aradu.BE7PX	Araip.J5910	multiple-copy	kinesin-3-likeisoformX2[Glycinemax]
Aradu.237HP	Araip.2Q2JE	multiple-copy	LAG1longevityassurancehomolog3
Aradu.PMG1D	Araip.6NT76	multiple-copy	lecithin:cholesterolacyltransferase3
Aradu.9S3Z5	Araip.7P2V7	multiple-copy	Leucinecarboxylmethyltransferase
Aradu.5T2RZ	Araip.ATR2W	multiple-copy	lipase1
Aradu.QKN8V	Araip.ARE8G	multiple-copy	lipid-bindingserumglycoproteinfamilyprotein
Aradu.35TX9	Araip.PP85N	multiple-copy	lipoylsynthase2,mitochondrial[Glycinemax]
Aradu.V8MCV	Araip.5N6GI	multiple-copy	LL-diaminopimelateaminotransferase
Aradu.1FM9H	Araip.XN7XL	multiple-copy	LMBR1domain-containingprotein2homologA-likeisoformX
Aradu.E32C9	Araip.5UJ5P	multiple-copy	long-chainacyl-CoAsynthetase2
Aradu.V5LSS	Araip.TY5FK	multiple-copy	long-chain-alcoholoxidaseFAO1
Aradu.CG33N	Araip.BS911	multiple-copy	lysineketoglutaratereductasetrans-splicing-likeprotein
Aradu.9QT6G	Araip.M4152	multiple-copy	lysine-specifichistonedemethylase1homolog2-like[Glycine
Aradu.7A2KY	Araip.7G39W	multiple-copy	MA3domain-containingprotein
Aradu.T7E55	Araip.UI4ZB	multiple-copy	magnesium-protoporphyrinIXmethyltransferase
Aradu.LD3HZ	Araip.1SH3C	multiple-copy	makorinRING-zinc-fingerprotein
Aradu.J5HIY	Araip.CW34G	multiple-copy	malatedehydrogenase

Aradu.4K089	Araip.32W9F	multiple-copy	MARbindingfilament-likeprotein1
Aradu.MEV99	Araip.8206I	multiple-copy	MEI2-like2-likeisoformX2[Glycinemax]
Aradu.GTI49	Araip.PK5IZ	multiple-copy	MetallopeptidaseM24n=1Tax=HeterobasidionirregulareT(
Aradu.RXW02	Araip.H8UEI	multiple-copy	MethionineS-adenosyltransferasen=1Tax=Detonulaconfe
Aradu.S4UH1	Araip.AD1AP	multiple-copy	methioninesulfoxidereductaseB2
Aradu.EUM6N	Araip.XG7T5	multiple-copy	Mevalonate/galactokinasefamilyprotein
Aradu.3SM7F	Araip.9PC2H	multiple-copy	microtubuleendbindingproteinEB1A
Aradu.G8W4L	Araip.7Y21E	multiple-copy	mitochondrialoutermembraneproteinporin1-like[Glycinem
Aradu.3L41J	Araip.0MK8M	multiple-copy	Mitochondrialtranscriptionterminationfactorfamilyprotein
Aradu.0SE2F	Araip.9UZ34	multiple-copy	mitoticcheckpointserine/threonine-proteinkinaseBUB1-like
Aradu.N25WS	Araip.T9R11	multiple-copy	Molybdenumcofactorsulfurasefamilyprotein
Aradu.82KWN	Araip.6IK4X	multiple-copy	monogalactosyldiacylglycerolsynthase2
Aradu.K59XP	Araip.5I1EE	multiple-copy	Mpsonebinderkinaseactivator-likeprotein1A
Aradu.T7J8U	Araip.C1Y5Y	multiple-copy	mRNAcappingenzymefamilyprotein
Aradu.W912S	Araip.4KH5L	multiple-copy	mucin-19-likeisoformX3[Glycinemax]
Aradu.Z9J76	Araip.PH9P5	multiple-copy	myo-inositoloxygenase2
Aradu.RLB9U	Araip.J11AY	multiple-copy	N2,N2-dimethylguanosinetRNAmethyltransferase
Aradu.H48T8	Araip.V9UEK	multiple-copy	NAD(P)H-quinoneoxidoreductasesubunitM
Aradu.U4RIV	Araip.306R5	multiple-copy	NADP-dependentalkenaldoublebondreductase
Aradu.7M26B	Araip.20V0X	multiple-copy	NADPH-dependentthioredoxinreductaseA
Aradu.B8ABT	Araip.TVG17	multiple-copy	N-alpha-acetyltransferase15,NatAauxiliarysubunit-like[Gly
Aradu.47LJL	Araip.T6887	multiple-copy	NB-ARCdomaindiseaseresistanceprotein
Aradu.2BE4F	Araip.CV72U	multiple-copy	NCdomain-containingprotein-related
Aradu.PRJ6R	Araip.8X38S	multiple-copy	NDH-dependentcyclicelectronflow1
Aradu.1CY96	Araip.4C08I	multiple-copy	NHLdomain-containingprotein
Aradu.AM8Z8	Araip.1G2UE	multiple-copy	NIPA-likeprotein2-likeisoformX1[Glycinemax]
Aradu.RCY11	Araip.WJ4BG	multiple-copy	nitrilase4
Aradu.5PW6G	Araip.G04W3	multiple-copy	Nodulin-like/MajorFacilitatorSuperfamilyprotein
Aradu.KQ4LB	Araip.W7ZN3	multiple-copy	novelplantsnare11
Aradu.5A0JT	Araip.84AZS	multiple-copy	N-terminalnucleophileaminohydrolases(Ntnhydrolases)su
Aradu.03ESY	Araip.D1KMK	multiple-copy	nuclearmovementfamilyprotein
Aradu.KU05P	Araip.7K2B7	multiple-copy	nucleolarcomplexprotein4homolog[Glycinemax]
Aradu.IFZ2Q	Araip.NU1G9	multiple-copy	NucleolarGTP-bindingprotein
Aradu.EPY5P	Araip.T88CG	multiple-copy	nucleolarprotein16-like[Glycinemax]
Aradu.MA23R	Araip.40836	multiple-copy	nucleosidediphosphatekinase3
Aradu.988BI	Araip.92VW5	multiple-copy	nucleosomeassemblyprotein1
Aradu.G5PJM	Araip.AA5NQ	multiple-copy	Nucleotide/sugartransporterfamilyprotein
Aradu.085LG	Araip.39IGX	multiple-copy	O-fucosyltransferasefamilyprotein
Aradu.81KMB	Araip.2TV5Y	multiple-copy	organiccation/carnitinetransporter2
Aradu.SI3S6	Araip.EDG2M	multiple-copy	organ-specificproteinS2-like[Glycinemax]
Aradu.U8VVP	Araip.56PXP	multiple-copy	ORMDLfamilyprotein
Aradu.Z1BP5	Araip.0L3VD	multiple-copy	Outerarmdyneinlightchain1protein
Aradu.9H3L9	Araip.S7WL6	multiple-copy	pantothenatekinase2
Aradu.V2Z0U	Araip.7JC4X	multiple-copy	Papainfamilycysteineprotease

Aradu.0NR0Q	Araip.8JW62	multiple-copy	PAT1homolog1-likeisoformX1[Glycinemax]
Aradu.C61GI	Araip.YLJ2W	multiple-copy	patellin-3-likeisoformX1[Glycinemax]
Aradu.IW3RC	Araip.K0Q5S	multiple-copy	PeptidaseM50familyprotein
Aradu.PU63R	Araip.Z7JB7	multiple-copy	peptide/nitratetransporter
Aradu.A5XMW	Araip.2HU43	multiple-copy	peptide-N(4)-(N-acetyl-beta-glucosaminyl)asparagineami
Aradu.U5BST	Araip.DSZ1Z	multiple-copy	peroxisomaladeninenucleotidecarrier1
Aradu.VK4DU	Araip.V5XRP	multiple-copy	peroxisomalbiogenesisfactor11familyprotein
Aradu.W4PG1	Araip.968KT	multiple-copy	Phosphatidatecytidylyltransferasefamilyprotein
Aradu.7H3UM	Araip.NH4AA	multiple-copy	phosphatidylinositol3,4,5-trisphosphate3-phosphataseanc
Aradu.TRI7U	Araip.CF7TW	multiple-copy	phosphatidylinositoltransferproteinCSR1-like[Glycinemax
Aradu.62PY8	Araip.A81A1	multiple-copy	phosphoenolpyruvatecarboxykinase1
Aradu.6VN02	Araip.Z5712	multiple-copy	phosphoglycerate/bisphosphoglyceratemutase
Aradu.4X75W	Araip.18K24	multiple-copy	phospholipid:diacylglycerolacyltransferase
Aradu.M4DGG	Araip.R828T	multiple-copy	phytoenedesaturase3
Aradu.QV5DJ	Araip.2LX3X	multiple-copy	PIF3-like[Glycinemax]
Aradu.SH4SS	Araip.K7F8Y	multiple-copy	PLAC8familyprotein
Aradu.T9RZI	Araip.2RY51	multiple-copy	plant/MEB5-likeprotein
Aradu.S8SZ6	Araip.48P6B	multiple-copy	Plasma-membranecholinetransporterfamilyprotein
Aradu.B3WTX	Araip.5VE9R	multiple-copy	plasmamembraneH+-ATPase
Aradu.8K8TN	Araip.2JP01	multiple-copy	plasmamembraneintrinsicprotein1
Aradu.93VCV	Araip.VX6P0	multiple-copy	plastiddivisionproteinPDV1-like[Glycinemax]
Aradu.LF3E5	Araip.Y1R8S	multiple-copy	plastid-lipid-associatedprotein12,chloroplastic-likeisoform
Aradu.92ZAJ	Araip.H70JX	multiple-copy	plastocyanin-likedomainprotein
Aradu.G4VEH	Araip.049JK	multiple-copy	polyadenylate-bindingprotein1
Aradu.SPS87	Araip.Z3KU8	multiple-copy	polynucleotide5'-hydroxyl-kinaseNOL9-likeisoformX2[Gly
Aradu.GG5EE	Araip.IA3XR	multiple-copy	PQ-looprepeatfamilyprotein/transmembranefamilyprotein
Aradu.Q3QE4	Araip.AFF6E	multiple-copy	prefoldin
Aradu.M035D	Araip.UI1ED	multiple-copy	prephenatedehydrogenasefamilyprotein
Aradu.BF9MN	Araip.Q865J	multiple-copy	profilin
Aradu.35Z4P	Araip.N2HP9	multiple-copy	prolinedehydrogenase
Aradu.MP8TG	Araip.26FIE	multiple-copy	prolyl4-hydroxylasesubunitalpha-1-like[Glycinemax]
Aradu.NAJ8T	Araip.X4UBD	multiple-copy	proteasomeactivatorcomplexsubunit-likeprotein
Aradu.CS27R	Araip.2GH89	multiple-copy	Proteinbinding/ubiquitin-proteinligase/zincionbindingn=1T
Aradu.82P2N	Araip.GBD9V	multiple-copy	protein-tyrosine-phosphataseMKP1-likeisoformX3[Glycine
Aradu.3Y26A	Araip.W0TVI	multiple-copy	protonpumpinteractor,putative
Aradu.D18PX	Araip.AZ7G0	multiple-copy	pterin-4-alpha-carbinolaminedehydratase
Aradu.J3P01	Araip.YIX8M	multiple-copy	PWWPdomain-containingprotein2A-like[Glycinemax]
Aradu.9CU4Z	Araip.F9W15	multiple-copy	Rab3GTPase-activatingproteinnon-catalyticsubunit
Aradu.0N518	Araip.5M5DL	multiple-copy	ralf-like34
Aradu.L1VEE	Araip.BAC4E	multiple-copy	rapidalkalinizationfactor1
Aradu.G229R	Araip.6TW9A	multiple-copy	Ras-relatedsmallGTP-bindingfamilyprotein
Aradu.T1ZHN	Araip.812WS	multiple-copy	retrotransposon-likeprotein1-like[Glycinemax]
Aradu.CI747	Araip.2M3GS	multiple-copy	riboflavinbiosynthesisprotein,putative
Aradu.L3WM9	Araip.18J4Y	multiple-copy	RibosomalRNAlargesubunitmethyltransferaseHn=4Tax=F

Aradu.TBT3N	Araip.J05HY	multiple-copy	RibosomematurationfactorRimMn=2Tax=Synechococcus
Aradu.Q51NH	Araip.XGD3E	multiple-copy	RNAhelicase,ATP-dependent,SK12/DOB1protein
Aradu.C9WAZ	Araip.2QM99	multiple-copy	RNAligase/cyclicnucleotidephosphodiesterasefamilyprote
Aradu.J1S8L	Araip.92A2E	multiple-copy	ROOTHAIRDEFECTIVE3homolog1-like[Glycinemax]
Aradu.AB724	Araip.IT82Y	multiple-copy	ROOTPRIMORDIUMDEFECTIVE1-likeisoformX2[Glycine
Aradu.UB2GG	Araip.BZ0SY	multiple-copy	RPM1interactingprotein4
Aradu.K8WQA	Araip.JN4EG	multiple-copy	rRNA-processingproteinFCF1homolog[Glycinemax]
Aradu.W5BH1	Araip.J80SY	multiple-copy	RWP-RKdomain-containingprotein
Aradu.F6HJK	Araip.64L2N	multiple-copy	SCARfamilyprotein
Aradu.E92C1	Araip.U3VBH	multiple-copy	Secretorycarriermembraneprotein(SCAMP)familyprotein
Aradu.S073D	Araip.HTP4Q	multiple-copy	senescence-associatedcarboxylesterase101-likeisoformX
Aradu.NDR67	Araip.WC0WL	multiple-copy	septum-promotingGTP-bindingprotein1-like[Glycinemax]
Aradu.QCD9Z	Araip.2S8VQ	multiple-copy	ser/thr-richproteinT10inDGCRregion-likeprotein
Aradu.623Y7	Araip.W2EJQ	multiple-copy	serineacetyltransferase1
Aradu.267PI	Araip.F91RT	multiple-copy	Serinepeptidase,clanSC,familyS9A-likeprotein,putativen=
Aradu.XZ2EW	Araip.0178A	multiple-copy	SGT1-1[Glycinemax]
Aradu.LJV84	Araip.0A4CX	multiple-copy	SHIRELATEDSEQUENCE1-like[Glycinemax]
Aradu.9TL9J	Araip.3W2E6	multiple-copy	shugoshin-1-likeisoformX1[Glycinemax]
Aradu.95REC	Araip.CW5YA	multiple-copy	sieveelementocclusionprotein
Aradu.2CK5F	Araip.34N7T	multiple-copy	Signalpeptidasecomplexcatalyticsubunitn=3Tax=Cycloph
Aradu.4Q608	Araip.81EVU	multiple-copy	signalpeptidasecomplexsubunit1-likeisoformX2[Glycinem
Aradu.SL2QQ	Araip.2HD3N	multiple-copy	SKP1-like21
Aradu.63ZUN	Araip.186SX	multiple-copy	SMAD/FHAdomain-containingprotein
Aradu.NNA2S	Araip.6QX3D	multiple-copy	Smallnuclearribonucleoproteinfamilyprotein
Aradu.JH5V3	Araip.4V07C	multiple-copy	SmallnuclearRNAactivatingcomplex(SNAPc),subunitSNA
Aradu.1R5WG	Araip.D3BJQ	multiple-copy	sn1-specificdiacylglycerollipasebeta-like[Glycinemax]
Aradu.612YH	Araip.84MED	multiple-copy	SNARE-interactingproteinKEULE-likeisoformX1[Glycinem
Aradu.V6ZCM	Araip.D7GGC	multiple-copy	solubleN-ethylmaleimide-sensitivefactoradaptorprotein33
Aradu.X685X	Araip.6G7UA	multiple-copy	solutecarrierfamily35protein
Aradu.A1KE0	Araip.81TFR	multiple-copy	spermidinesynthase1
Aradu.3AI2Z	Araip.6D2E5	multiple-copy	Spo11/DNAtopoisomeraseVI,subunitAprotein
Aradu.D0L18	Araip.76IDI	multiple-copy	SPT2homologisoformX5[Glycinemax]
Aradu.3X1QZ	Araip.0ZR5Y	multiple-copy	Staphylococcalnucleasehomologue
Aradu.C6ILJ	Araip.AJZ7U	multiple-copy	starchsynthase2
Aradu.M72UM	Araip.WI7LP	multiple-copy	sterolmethyltransferase1
Aradu.1R7R2	Araip.9634I	multiple-copy	StressresponsiveA/BBarrelDomain
Aradu.U1RBP	Araip.313P6	multiple-copy	strictosidinesynthase3
Aradu.YE5Q3	Araip.K0HCY	multiple-copy	sucrose-phosphatase1
Aradu.JW82A	Araip.PGB0K	multiple-copy	sucrosesynthase4
Aradu.VMK7V	Araip.E2XDB	multiple-copy	SulfiteexporterTauE/SafEfamilyprotein
Aradu.J4D1T	Araip.6I0UC	multiple-copy	survivalmotorneuroninteractingprotein
Aradu.1LM0G	Araip.S8ZYY	multiple-copy	synaptosomal-associatedproteinSNAP25-like29
Aradu.5T6PH	Araip.I7NVN	multiple-copy	taurinecatabolismdioxygenaseTauD/TfdA
Aradu.EG1H0	Araip.3D6BD	multiple-copy	thiol-disulfideoxidoreductaseDCC

Aradu.J60UE	Araip.J4ZFW	multiple-copy	thylakoidlumenal16.5kDaprotein,chloroplastic-likeisoform
Aradu.4P2F5	Araip.1JL7K	multiple-copy	thylakoidmembranephosphoprotein14kDaprotein
Aradu.4TY89	Araip.UJ1F9	multiple-copy	TIC20-IV,chloroplastic-likeisoformX2[Glycinemax]
Aradu.7C9V0	Araip.TD5YX	multiple-copy	Tic22-likefamilyprotein
Aradu.61JFB	Araip.L4X0W	multiple-copy	TIFY6B-likeisoformX1[Glycinemax]
Aradu.T5UQE	Araip.K6XKE	multiple-copy	TIMEFORCOFFEE-likeisoformX2[Glycinemax]
Aradu.KCC58	Araip.H2SSN	multiple-copy	tobamovirusmultiplication1
Aradu.P79WU	Araip.IMH7R	multiple-copy	tonoplastintrinsicprotein1
Aradu.B1CQK	Araip.JKM82	multiple-copy	topless-relatedprotein3-likeisoformX1[Glycinemax]
Aradu.CZ5NE	Araip.G26F0	multiple-copy	TraBfamilyprotein
Aradu.Y5UE5	Araip.237X1	multiple-copy	TRAF-likefamilyprotein
Aradu.JU9J9	Araip.9KL4T	multiple-copy	trans-2-enoyl-CoAreductase
Aradu.S5XS2	Araip.22Y09	multiple-copy	transcriptionalcorepressorLEUNIG-likeisoformX1[Glycine
Aradu.D24Y8	Araip.FDN31	multiple-copy	transcriptionterminationfactor,mitochondrial-like[Glycinem
Aradu.GT1TJ	Araip.C74GI	multiple-copy	transportSec61subunitalpha-like[Glycinemax]
Aradu.LAI2T	Araip.1K5KM	multiple-copy	triacylglycerollipase
Aradu.29IAA	Araip.JV3XS	multiple-copy	tripeptidylpeptidaseii
Aradu.0US4M	Araip.GL0UW	multiple-copy	tRNA/rRNAmethyltransferase(SpoU)familyprotein
Aradu.S9BZU	Araip.PY45A	multiple-copy	tRNA-specificadenosinedeaminase
Aradu.M206V	Araip.0T13B	multiple-copy	tryptophan-tRNAligase
Aradu.9S9RY	Araip.VDU5V	multiple-copy	tubby-likeF-boxprotein8-likeisoformX2[Glycinemax]
Aradu.K4GSH	Araip.4DS9Y	multiple-copy	tubulinalpha-6chain,putative
Aradu.8L8L4	Araip.1K0LY	multiple-copy	tubulinbetachain2
Aradu.18R1L	Araip.IX44P	multiple-copy	Signaltransductionhistidinekinase
Aradu.6C2JR	Araip.M4KG4	multiple-copy	tyrosinephosphatase
Aradu.F3VDW	Araip.2290W	multiple-copy	tyrosyl-DNAphosphodiesterase-related
Aradu.CDJ2J	Araip.842TG	multiple-copy	U3smallnucleolarRNA-associated-likeprotein,putative
Aradu.AY7XX	Araip.TK75I	multiple-copy	Ubiquitin-conjugatingenzymefamilyprotein
Aradu.ZT956	Araip.IMD5Y	multiple-copy	ubiquitinfusiondegradation1
Aradu.V2YDB	Araip.ZTF9F	multiple-copy	UDP-D-glucose/UDP-D-galactose4-epimerase1
Aradu.GGI7I	Araip.EV9VN	multiple-copy	UDP-galactosetransporter2
Aradu.F0FER	Araip.4P78F	multiple-copy	UDP-glucose6-dehydrogenasefamilyprotein
Aradu.XRA1G	Araip.4P4HG	multiple-copy	UDP-sugarpyrophosphorylase
Aradu.78VGB	Araip.5AX8I	multiple-copy	UNC93-likeprotein1-like[Glycinemax]
Aradu.B5WG1	Araip.52XKK	multiple-copy	UNE12[Glycinemax]
Aradu.D2I4Y	Araip.F1S7G	multiple-copy	Vacuolarimport/degradation,Vid27-relatedprotein
Aradu.IIT7E	Araip.S698B	multiple-copy	Vacuolarsortingprotein9(VPS9)domain
Aradu.4TM7E	Araip.I4Y3K	multiple-copy	vesicletransporterSec20,putative
Aradu.9ID7S	Araip.D2YEW	multiple-copy	violaxanthinde-epoxidase-related
Aradu.9EK2R	Araip.HEP3C	multiple-copy	viralmovementprotein
Aradu.S59W7	Araip.V3RAV	multiple-copy	VOZ1-likeisoformX2[Glycinemax]
Aradu.04EM8	Araip.TV5CN	multiple-copy	WEAKCHLOROPLASTMOVEMENTUNDERBLUELIGHT
Aradu.HH0H8	Araip.DT8XF	multiple-copy	XRI1-likeisoformX1[Glycinemax]
Aradu.IAV7G	Araip.7JC2C	multiple-copy	YABBYtranscriptionfactor

Aradu.M1JE3	Araip.D8IR5	multiple-copy	zinctransporterproteinDDB_G0291141isoform1[Glycinem
Aradu.5926B	Araip.4E9YI	multiple-copy	ZIPmetaliontransporterfamily
Aradu.99F66	Araip.7JJ4U	multiple-copy	2-aminoethanethioldioxygenase-like[Glycinemax]
Aradu.Z2J2V	Araip.5F3C7	multiple-copy	3-oxoacyl-[acyl-carrier-protein]synthase3n=2Tax=Synech
Aradu.07AAJ	Araip.1JP91	multiple-copy	Actincross-linkingprotein
Aradu.464FR	Araip.YGS65	multiple-copy	actindepolymerizingfactor10
Aradu.UM9AF	Araip.7BF1X	multiple-copy	acyl-CoAN-acyltransferase(NAT)superfamilyprotein
Aradu.CPH08	Araip.7RJ08	multiple-copy	acyl-CoAoxidase1
Aradu.ERC3E	Araip.4KS9Y	multiple-copy	acyl-CoAthioesterase,putative
Aradu.P6ZM2	Araip.2G1DX	multiple-copy	acyl-coenzymeAthioesterase-likeprotein
Aradu.JS0R2	Araip.6ZZ85	multiple-copy	adeninephosphoribosyltransferase5
Aradu.PW4PJ	Araip.DE4JR	multiple-copy	adiponectinreceptorprotein2-likeisoformX3[Glycinemax]
Aradu.E8NYC	Araip.C9FAB	multiple-copy	aldose1-epimerasefamilyprotein
Aradu.CZ7XQ	Araip.8M0ZD	multiple-copy	AluminiuminducedproteinwithYGLandLRDRmotifs
Aradu.49CW6	Araip.MU3QB	multiple-copy	anaphasepromotingcomplexsubunit2
Aradu.1G6QS	Araip.2A3ZP	multiple-copy	anthranilatephosphoribosyltransferase,putative
Aradu.YAN6P	Araip.11R1Y	multiple-copy	AP-1complexsubunitsigma-likeprotein
Aradu.Z33GV	Araip.2NY3H	multiple-copy	arginine/serine-richcoiledcoilprotein
Aradu.RF5XH	Araip.D30ND	multiple-copy	Argonautefamilyprotein
Aradu.8479S	Araip.EBH0J	multiple-copy	arogenatedehydratase1
Aradu.577TS	Araip.03RBH	multiple-copy	asparticproteinaseA1
Aradu.3I9MJ	Araip.FMA28	multiple-copy	ATPasefamilyAAAdomain-containingprotein1-like[Glycine
Aradu.47F3C	Araip.IW920	multiple-copy	ATP-dependentClpproteaseadapterproteinClpSn=2Tax=S
Aradu.G68BX	Araip.B227D	multiple-copy	BAGfamilymolecularchaperoneregulator1-like[Glycinema
Aradu.VKJ07	Araip.7827F	multiple-copy	Baxinhibitor-1familyprotein
Aradu.NG8V7	Araip.U6ER1	multiple-copy	BES1/BZR1homologprotein2-like[Glycinemax]
Aradu.B7Y20	Araip.1W5SS	multiple-copy	beta-1,4-N-acetylglucosaminyltransferasefamilyprotein
Aradu.BLA0I	Araip.VMH3J	multiple-copy	beta-xylosidase1
Aradu.C3FIF	Araip.028PN	multiple-copy	BON1-associated-likeprotein
Aradu.426TW	Araip.G5KAW	multiple-copy	branched-chain-amino-acidaminotransferase-likeprotein
Aradu.TM2EL	Araip.79M9T	multiple-copy	Brevisradix-like2-likeisoformX2[Glycinemax]
Aradu.CA6PJ	Araip.6L1FE	multiple-copy	BSDdomain-containingprotein
Aradu.6D72R	Araip.CG1RC	multiple-copy	BTB-POZandMATHdomain2
Aradu.VW946	Araip.3HJ42	multiple-copy	C4-dicarboxylatetransporter/malicacidtransportprotein
Aradu.73E3B	Araip.6W639	multiple-copy	CAAXprenylprotease1homolog[Glycinemax]
Aradu.HW921	Araip.92STN	multiple-copy	calcineurinB-like3
Aradu.59RNH	Araip.15F3V	multiple-copy	Calreticulin2,calcium-bindingproteinn=1Tax=Coccomyxas
Aradu.AKR7M	Araip.6C7DA	multiple-copy	CAP(Cysteine-richsecretoryproteins,Antigen5,andPathog
Aradu.JC6IN	Araip.38XYU	multiple-copy	carbohydrateesteraseAt4g34215-likeisoformX1[Glycinem
Aradu.12EER	Araip.H7369	multiple-copy	carbonicanhydrase2
Aradu.HQ2V8	Araip.5MK79	multiple-copy	caseinkinase1
Aradu.BK3J1	Araip.CL7TC	multiple-copy	Chalcone-flavanoneisomerasefamilyprotein
Aradu.FT8DK	Araip.I8X4Y	multiple-copy	chalconesynthase-like[Glycinemax]
Aradu.V6QFH	Araip.03SRG	multiple-copy	chargedmultivesicularbodyprotein

Aradu.FHW89	Araip.43JFQ	multiple-copy	Chitinase/Hevein/PR-4/Wheatwin2
Aradu.D5WZF	Araip.NM392	multiple-copy	chloridechannelA
Aradu.NY5CH	Araip.3R87M	multiple-copy	chorismatemutase1
Aradu.D2JYY	Araip.CDL83	multiple-copy	chromatinassemblyfactor1subunitFAS1-like[Glycinemax]
Aradu.80C88	Araip.E7AL7	multiple-copy	CLP1-similarprotein5
Aradu.2CB86	Araip.N94NJ	multiple-copy	COBRA-like[Glycinemax]
Aradu.N3NL4	Araip.2GN8J	multiple-copy	conservedoligomericGolgicomplexcomponent-related/CO
Aradu.JQ4B3	Araip.2H2XV	multiple-copy	Cornichonfamilyprotein
Aradu.0D7Q2	Araip.J7WAX	multiple-copy	cysteinesynthase26
Aradu.MN6GR	Araip.TQV21	multiple-copy	D-3-phosphoglyceratedehydrogenase
Aradu.16P0Z	Araip.785V6	multiple-copy	DCD(DevelopmentandCellDeath)domainprotein
Aradu.G3X0F	Araip.L9ELN	multiple-copy	defectiveincullinneddylationprotein,putative
Aradu.2P8EW	Araip.2W8EJ	multiple-copy	dehydroquinatedehydratase,putative/shikimatedehydroge
Aradu.E70M5	Araip.941M4	multiple-copy	DENN(AEX-3)domain-containingprotein
Aradu.V9U81	Araip.18474	multiple-copy	digalactosyldiacylglycerolsynthase1,chloroplastic-like[Gly
Aradu.XHP2L	Araip.I1C79	multiple-copy	dihydroflavonol4-reductase
Aradu.9AB3B	Araip.BDV5W	multiple-copy	dolichyl-diphosphooligosaccharide-proteinglycosyltransfe
Aradu.ST3KE	Araip.0AN9G	multiple-copy	DoubleClp-Nmotif-containingP-loopnucleosidetriphospha
Aradu.XP644	Araip.72X39	multiple-copy	EARLYFLOWERING3-likeisoformX2[Glycinemax]
Aradu.59CIU	Araip.G3NEL	multiple-copy	emp24/gp25L/p24family/GOLDfamilyprotein
Aradu.UTT3P	Araip.FU2FP	multiple-copy	EmsyNTerminus(ENT)/plantTudor-likedomains-containing
Aradu.3N57P	Araip.KUG5H	multiple-copy	ERlumenproteinretainingreceptorfamilyprotein
Aradu.Y9Y02	Araip.KR2LL	multiple-copy	ERmembraneDUF1077domainprotein,putativen=4Tax=A
Aradu.SYY8Y	Araip.9C7ZQ	multiple-copy	Erythronate-4-phosphatedehydrogenasefamilyprotein
Aradu.R6UC6	Araip.U8MRA	multiple-copy	exocystcomplexcomponent84B
Aradu.1IC89	Araip.M8TU7	multiple-copy	exportin1A
Aradu.CYF2K	Araip.GVS2Q	multiple-copy	extra-largeguaninenucleotide-bindingprotein1-like[Glycine
Aradu.V3ATA	Araip.500NU	multiple-copy	FAD/NAD(P)-bindingoxidoreductasefamilyprotein
Aradu.FX57K	Araip.T1F9U	multiple-copy	fattyacyl-CoAreductase2-like[Glycinemax]
Aradu.JT6Z2	Araip.4UD0W	multiple-copy	fiberproteinFb15
Aradu.JQY29	Araip.40GWN	multiple-copy	FLX-like3-likeisoformX1[Glycinemax]
Aradu.J0FTC	Araip.UI4QL	multiple-copy	Folicacidandderivativebiosyntheticprocess,putativen=1Ta
Aradu.8I79H	Araip.LA152	multiple-copy	fructose-1,6-bisphosphatase
Aradu.F7BLG	Araip.MW082	multiple-copy	gamma-tubulincomplexcomponent6-like[Glycinemax]
Aradu.N2X1B	Araip.EVL84	multiple-copy	GDA1/CD39nucleosidephosphatasefamilyprotein
Aradu.031YC	Araip.176AR	multiple-copy	GDSLesterase/lipaseplant-likeprotein
Aradu.3E4UZ	Araip.7L3C8	multiple-copy	germin-likeprotein2
Aradu.W7MG7	Araip.6C94R	multiple-copy	Glucose-methanol-choline(GMC)oxidoreductasefamilypro
Aradu.JI08P	Araip.Y4YUV	multiple-copy	glutamatereceptor2
Aradu.U75R0	Araip.A6KDQ	multiple-copy	glutaredoxin4
Aradu.6PP4L	Araip.M6JV5	multiple-copy	glycerol-3-phosphatedehydrogenase,putative
Aradu.A3D3N	Araip.P0LZ1	multiple-copy	glycerol-3-phosphatetransporter
Aradu.D9ISK	Araip.P2G41	multiple-copy	GlycoproteinmembraneprecursorGPI-anchored
Aradu.65NZB	Araip.0RS31	multiple-copy	GTPbindingElongationfactorTufamilyprotein

Aradu.3Q3ML	Araip.LG925	multiple-copy	HCP-likesuperfamilyprotein
Aradu.HU8ZS	Araip.VHP1S	multiple-copy	hexokinase1
Aradu.82FNR	Araip.PH8DZ	multiple-copy	histidine-containingphosphotransferprotein1-like[Glycinem
Aradu.9T44T	Araip.NI6UG	multiple-copy	HIT-typeZincfingerfamilyprotein
Aradu.D1CF5	Araip.P8QST	multiple-copy	homogentisatephytyltransferase1
Aradu.S54FQ	Araip.2J0CW	multiple-copy	Hydrolase,putativen=1Tax=RicinuscommunisRepID=B9R
Aradu.QT6LV	Araip.47CG9	multiple-copy	hydroxyproline-richglycoproteinfamilyprotein
Aradu.U9WH5	Araip.AP4U8	multiple-copy	IAA-aminoacidhydrolaseILR1-likeprotein
Aradu.1H4CB	Araip.JYQ4Z	multiple-copy	inactivepoly[ADP-ribose]polymeraseRCD1
Aradu.VM246	Araip.858RT	multiple-copy	inosine-uridinepreferringnucleosidehydrolasefamilyprotei
Aradu.6Z34Q	Araip.0ST2B	multiple-copy	isochorismatasehydrolasefamilyprotein
Aradu.FT1FE	Araip.RQ7L5	multiple-copy	KDELmotif-containingprotein1-likeisoformX2[Glycinemax]
Aradu.66KCD	Araip.P9NAH	multiple-copy	la-relatedprotein1isoformX2[Glycinemax]
Aradu.TW3FF	Araip.5R4LP	multiple-copy	LEM3(ligand-effectmodulator3)familyprotein/CDC50family
Aradu.MF6JY	Araip.63PMR	multiple-copy	LORELEI-LIKE-GPI-ANCHOREDPROTEIN1
Aradu.R07DC	Araip.BV0ZS	multiple-copy	L-typelectin-domaincontainingreceptorkinaseIX.1-like[Gly
Aradu.12CJQ	Araip.V8KRY	multiple-copy	LysMdomainGPI-anchoredprotein
Aradu.8Y5VQ	Araip.90BCU	multiple-copy	macrophagemigrationinhibitoryfactorhomolog[Glycinemax
Aradu.Q5DZL	Araip.2NV9I	multiple-copy	magnesiumchelatasei2
Aradu.EK8MW	Araip.9P3VC	multiple-copy	mannanendo-1,4-beta-mannosidase2-like[Glycinemax]
Aradu.3SD99	Araip.D0QDL	multiple-copy	maternaleffectembryoarrest60
Aradu.TZ1M5	Araip.YA4KW	multiple-copy	MD-2-relatedlipidrecognitiondomain-containingprotein
Aradu.317LS	Araip.149YW	multiple-copy	metalloendoproteinase1-like[Glycinemax]
Aradu.H6T1L	Araip.S5E2W	multiple-copy	methylesterase11
Aradu.W8SNB	Araip.7WS9N	multiple-copy	mitochondrialphosphatecarrierprotein3,mitochondrial-like
Aradu.YHD0I	Araip.EX3UF	multiple-copy	MLP-likeprotein423
Aradu.B34AL	Araip.D33IT	multiple-copy	myosinIBheavychain-like[Glycinemax]
Aradu.IHZ0W	Araip.W6NII	multiple-copy	NADH-ubiquinoneoxidoreductase24kDasubunit,putative
Aradu.JT1K4	Araip.D3EYV	multiple-copy	non-specificphospholipaseC2
Aradu.I609F	Araip.92GX5	multiple-copy	nuclearfactorY,subunitB3
Aradu.KX3YY	Araip.ACZ6E	multiple-copy	nuclearmatrixconstituentprotein1-likeprotein-likeisoformX
Aradu.GJB7M	Araip.EP5TR	multiple-copy	originrecognitioncomplexsubunit4
Aradu.PV43B	Araip.LR5ES	multiple-copy	ovatefamilyprotein14
Aradu.6RC9F	Araip.40P7B	multiple-copy	Peptidemethioninesulfoxidereductasefamilyprotein
Aradu.IU1HH	Araip.GM19P	multiple-copy	phenylalanineammonia-lyase2
Aradu.QK488	Araip.Q1NLX	multiple-copy	phloemprotein2-A9
Aradu.1J3FJ	Araip.92EP4	multiple-copy	Phosphatidylinositol3-and4-kinasefamilyprotein
Aradu.ZF53H	Araip.7C4C2	multiple-copy	Phosphoglucomutase/phosphomannomutase,alpha/beta/
Aradu.UC6E5	Araip.ZTR01	multiple-copy	Phosphoinositidephosphatasefamilyprotein
Aradu.N9R9J	Araip.03MNI	multiple-copy	phosphopantothenoylcysteinedecarboxylase-like[Glycine
Aradu.4E6H2	Araip.I7LKY	multiple-copy	phosphopyruvatehydratase
Aradu.F8T28	Araip.S8H7F	multiple-copy	Phox(PX)domain-containingprotein
Aradu.HUW75	Araip.C6UMI	multiple-copy	PI-PLCXdomain-containingproteinAt5g67130-like[Glycine
Aradu.1R3I6	Araip.T4LYM	multiple-copy	plantinvertase/pectinmethylesteraseinhibitor

Aradu.N3085	Araip.01BXJ	multiple-copy	plant-specificB3-DNA-bindingdomainprotein
Aradu.ZP2LZ	Araip.A9CVZ	multiple-copy	Plasmamembranemannitoltransportern=1Tax=Arachishy
Aradu.GT860	Araip.77NJ9	multiple-copy	plastiddevelopmentalproteinDAG,putative
Aradu.CX99Z	Araip.NHN1D	multiple-copy	poly(A)polymerase3
Aradu.329PQ	Araip.957AT	multiple-copy	polygalacturonasenon-catalyticprotein
Aradu.RAX6G	Araip.Z48R4	multiple-copy	pre-rRNA-processingesf1-likeprotein
Aradu.P833S	Araip.31SQ6	multiple-copy	Pyridoxalphosphate(PLP)-dependenttransferasessuperfa
Aradu.PD7X7	Araip.36SP7	multiple-copy	RABGDPdissociationinhibitor2
Aradu.WP07M	Araip.F4TTY	multiple-copy	rac-likeGTP-bindingprotein7-like[Glycinemax]
Aradu.MFG61	Araip.AE08Z	multiple-copy	RANbindingprotein1
Aradu.58BVX	Araip.VG1UA	multiple-copy	RELA/SPOThomolog2
Aradu.6MT8W	Araip.2R6PA	multiple-copy	replicationfactor-Acarboxy-terminaldomainprotein
Aradu.D3KJ3	Araip.3Z4V7	multiple-copy	Rhamnogalacturonatelyasefamilyprotein
Aradu.RWZ7N	Araip.FN9H2	multiple-copy	rhodanese-likedomain-containingprotein4,chloroplastic-li
Aradu.M2I9A	Araip.7IS7K	multiple-copy	rhodanese-relatedsulfurtransferase
Aradu.V9D23	Araip.94UKG	multiple-copy	rhoGTPase-activatingprotein1-like[Glycinemax]
Aradu.JV6V7	Araip.Y8ZSK	multiple-copy	ribosomebiogenesisproteinTSR3homologisoformX1[Glyc
Aradu.GDA41	Araip.06FC6	multiple-copy	ribulosebisphosphatecarboxylase/oxygenaseactivase
Aradu.523T3	Araip.F9PDZ	multiple-copy	ROP-interactiveCRIBmotif-containingprotein1
Aradu.EP05F	Araip.14ESI	multiple-copy	Rubberelongationfactorprotein(REF)
Aradu.Y328N	Araip.7HD9S	multiple-copy	ruBisCO-associatedprotein-like[Glycinemax]
Aradu.LDI07	Araip.1X2V2	multiple-copy	S1/P1nucleasefamilyprotein
Aradu.M0Q97	Araip.0S41I	multiple-copy	SAC3/GANP/Nin1/mts3/eIF-3p25family
Aradu.GGI7X	Araip.U8UTV	multiple-copy	SAWADEEHOMEODOMAINHOMOLOG1-likeisoformX2[
Aradu.ZFI13	Araip.B1TJC	multiple-copy	Senescence/dehydration-associatedprotein-related
Aradu.Q6P6Q	Araip.NQ67T	multiple-copy	shikimatekinase1
Aradu.FC1L9	Araip.11AMG	multiple-copy	solubleinorganicpyrophosphatase
Aradu.E6Z8G	Araip.3Q9LP	multiple-copy	SOULheme-bindingfamilyprotein
Aradu.HK5UD	Araip.HXW9F	multiple-copy	spermidinehydroxycinnamoyltransferase-like[Glycinemax
Aradu.7PG44	Araip.43FKD	multiple-copy	SPFH/Band7/PHBdomain-containingmembrane-associat
Aradu.TV4LZ	Araip.5Q25R	multiple-copy	SPIRAL1-like1
Aradu.WF6VN	Araip.UZ4WB	multiple-copy	SPXdomain-containingmembraneproteinAt4g22990-likeis
Aradu.LHL11	Araip.DHN9V	multiple-copy	structuralmaintenanceofchromosome3
Aradu.IC19J	Araip.I0CB1	multiple-copy	SUPPRESSOROFGENESILENCING3-likeisoformX1[Gly
Aradu.3ZE3D	Araip.45X5I	multiple-copy	SWAP(suppressor-of-white-APricot)/surpdomainprotein,p
Aradu.2NY77	Araip.5L4N3	multiple-copy	SWI/SNFcomplexcomponentSNF12homologisoformX2[G
Aradu.Y65N7	Araip.A4U29	multiple-copy	TMV-MP30bindingprotein2C,putative
Aradu.J7CY2	Araip.32X6B	multiple-copy	TPRXL-likeisoformX3[Glycinemax]
Aradu.FBB2P	Araip.GW6DK	multiple-copy	TRAM,LAG1andCLN8(TLC)lipid-sensingdomaincontainin
Aradu.58WGM	Araip.AR8PA	multiple-copy	TRANSPORTINHIBITORRESPONSE1-likeisoformX1[Gly
Aradu.N7UWQ	Araip.FMQ8F	multiple-copy	trichohyalin-likeisoformX3[Glycinemax]
Aradu.99FEF	Araip.W0MG3	multiple-copy	tRNA(guanine(37)-N1)-methyltransferase
Aradu.RJ2CI	Araip.FXA4L	multiple-copy	tRNA-dihydrouridine(47)synthase[NAD(P)(+)]-like[Glycine
Aradu.DT20E	Araip.P2Y88	multiple-copy	trypsinfamilyprotein

Aradu.Y966U	Araip.882KX	multiple-copy	U11/U12smallnuclearribonucleoprotein25kDaprotein-likei
Aradu.ZI9AT	Araip.0S8MN	multiple-copy	Ubiquitindomain-containingprotein
Aradu.MN7KS	Araip.9T0HK	multiple-copy	ubiquitinligaseSINAT3
Aradu.88K72	Araip.0ZS3I	multiple-copy	UBXdomain-containingprotein
Aradu.UJ5WJ	Araip.R83H8	multiple-copy	UDP-D-glucuronate4-epimerase2
Aradu.BAA8F	Araip.S12BL	multiple-copy	UDP-glucuronicaciddecarboxylase1
Aradu.J4JB7	Araip.02Z8U	multiple-copy	uridinekinase-like2
Aradu.483P6	Araip.IN0F4	multiple-copy	vacuolarH+-translocatinginorganicpyrophosphatase
Aradu.5D1Z0	Araip.GTY0B	multiple-copy	vacuolarmembraneproteinYML018C-likeisoformX2[Glycir
Aradu.S3AS8	Araip.WU7FZ	multiple-copy	Vacuolarprotein-sortingproteinbro1n=4Tax=Aspergillacea
Aradu.9TG4T	Araip.WX3WV	multiple-copy	xyloglucanglycosyltransferase12-like[Glycinemax]
Aradu.X96SI	Araip.U6BJT	multiple-copy	4-coumarate:CoAligase2
Aradu.UFC3R	Araip.87H0B	multiple-copy	acidphosphatase
Aradu.45CR1	Araip.3E8GX	multiple-copy	actin-bindingFH2(formin2)familyprotein
Aradu.569XL	Araip.41YFB	multiple-copy	AlbaDNA/RNA-bindingprotein
Aradu.B0MFP	Araip.S10I1	multiple-copy	amidase1
Aradu.08REY	Araip.4LL7A	multiple-copy	ammoniumtransporter1
Aradu.B9JN3	Araip.2Q9MF	multiple-copy	Anionexchangefamilyproteinn=2Tax=Populustrichocarpa
Aradu.EN58B	Araip.N6C0B	multiple-copy	AP2-likeethylene-responsivetranscriptionfactor
Aradu.149WS	Araip.HIL9C	multiple-copy	aspartateaminotransferase
Aradu.AP5ZW	Araip.VL9RM	multiple-copy	beta-amylase2
Aradu.7M5RR	Araip.DAS8U	multiple-copy	Biotin--[acetyl-CoA-carboxylase]ligase1n=1Tax=Dictyoste
Aradu.KS3VF	Araip.F2CNX	multiple-copy	CCR4-NOTtranscriptioncomplexfamilyproteinn=3Tax=ros
Aradu.RA8II	Araip.A81Z5	multiple-copy	chaperonin20
Aradu.QQ3BK	Araip.75DC1	multiple-copy	cinnamoylcoareductase
Aradu.S1UZ6	Araip.1H3PC	multiple-copy	Cysteine/Histidine-richC1domainfamilyprotein
Aradu.TY7A4	Araip.01ZK6	multiple-copy	cysteineproteinase1
Aradu.XES29	Araip.FL8FH	multiple-copy	D6proteinkinaselike2
Aradu.DN4RU	Araip.9J95X	multiple-copy	dCTPpyrophosphatase1-like[Glycinemax]
Aradu.QW806	Araip.90Q1T	multiple-copy	double-strandedRNA-bindingmotifprotein
Aradu.P0V8P	Araip.WMI4J	multiple-copy	E2F-associatedphosphoproteinisoformX1[Glycinemax]
Aradu.7Q4MS	Araip.QN9GG	multiple-copy	embryodefective1703n=1Tax=ArabidopsisthalianaRepID=
Aradu.AGS84	Araip.1Q8PX	multiple-copy	endonuclease/exonuclease/phosphatasefamilyprotein
Aradu.K5HNP	Araip.1ZG04	multiple-copy	enoyl-CoAhydratase/isomeraseA
Aradu.Z2HCJ	Araip.VC43M	multiple-copy	ENTH/VHS/GATfamilyprotein
Aradu.0B375	Araip.H48EG	multiple-copy	evolutionarilyconservedC-terminalregion2
Aradu.G0AVK	Araip.I5FVU	multiple-copy	Exonuclease,DNApolymeraseIII,epsilonsubunitn=1Tax=S
Aradu.64B3S	Araip.H3Y0N	multiple-copy	exosomecomplexcomponentRRP42-like[Glycinemax]
Aradu.95TME	Araip.NPM90	multiple-copy	exostosin-2-like[Glycinemax]
Aradu.ULI8B	Araip.14380	multiple-copy	ferredoxin3
Aradu.W4RCV	Araip.46XVA	multiple-copy	ferritin4
Aradu.L9VT7	Araip.6EG6G	multiple-copy	flocculationproteinFLO11-like[Glycinemax]
Aradu.9R08Q	Araip.2I50M	multiple-copy	glucan1,3-beta-glucosidaseA-like[Glycinemax]
Aradu.60DAC	Araip.NT0XC	multiple-copy	glutamatedehydrogenase1

Aradu.0HA70	Araip.I53JU	multiple-copy	glutathioneperoxidase2
Aradu.FD81T	Araip.MZ5SZ	multiple-copy	glycine-richRNA-binding,abscisicacid-inducibleprotein-like
Aradu.5EP67	Araip.65QZN	multiple-copy	GRFzincfingerprotein
Aradu.Z839U	Araip.0BN4Y	multiple-copy	heparanase-likeprotein1-likeisoformX2[Glycinemax]
Aradu.YB7BD	Araip.7B0U4	multiple-copy	highmobilitygroupB3
Aradu.GI6IZ	Araip.1FV4W	multiple-copy	importinsubunitalpha-1b
Aradu.677BP	Araip.TC9TY	multiple-copy	innermembraneproteasesubunit,putative
Aradu.L18YW	Araip.27R9P	multiple-copy	jasmonate-zim-domainprotein1
Aradu.SKW70	Araip.C53Q0	multiple-copy	kelchrepeat-containingprotein1-like[Glycinemax]
Aradu.0C0S7	Araip.B3ZXX	multiple-copy	Kinaseinteracting(KIP1-like)familyprotein
Aradu.GI6UB	Araip.576WD	multiple-copy	kinesinlightchain
Aradu.MAS03	Araip.081EX	multiple-copy	Lipase/lipooxygenase,PLAT/LH2familyprotein
Aradu.71RRV	Araip.1MM96	multiple-copy	lipidphosphatephosphatase2
Aradu.P6497	Araip.I17NZ	multiple-copy	mannose-1-phosphateguanyltransferase
Aradu.NP2GP	Araip.U8SW2	multiple-copy	metal-nicotianaminetransporterYSL3-likeisoformX1[Glycin
Aradu.D1ZPU	Araip.GLZ9X	multiple-copy	methyl-CPG-bindingdomain7
Aradu.C5W7K	Araip.246YY	multiple-copy	Mitochondrialglycoproteinfamilyprotein
Aradu.WJ2TG	Araip.N0DTV	multiple-copy	MLO-likeprotein1-like[Glycinemax]
Aradu.S6TIM	Araip.38345	multiple-copy	notumhomologisoformX1[Glycinemax]
Aradu.FV4D1	Araip.FLT6G	multiple-copy	NRAMPmetaliontransporter2
Aradu.WLH9X	Araip.5JH6G	multiple-copy	Nucleoporin,Nup133/Nup155-like
Aradu.SHC8B	Araip.0BB7B	multiple-copy	Nucleotide-sugartransporter/sugarportern=4Tax=Panicoid
Aradu.L2JLA	Araip.1KG8P	multiple-copy	O-acyltransferase(WSD1-like)familyprotein
Aradu.YXZ49	Araip.95TR1	multiple-copy	OBERON3-likeisoformX1[Glycinemax]
Aradu.7091K	Araip.AY0E9	multiple-copy	outermembraneOMP85familyprotein
Aradu.RB60U	Araip.8R9R2	multiple-copy	Outwardrectifyingpotassiumchannelprotein
Aradu.EL04J	Araip.92LQ2	multiple-copy	phosphatase2AregulatoryBsubunitfamily
Aradu.55LZT	Araip.75757	multiple-copy	PLANTCADMIUMRESISTANCE2
Aradu.46M2Y	Araip.38X68	multiple-copy	PlantregulatorRWP-RKfamilyprotein
Aradu.KMK70	Araip.GS4GH	multiple-copy	Pleckstrinhomology(PH)domain-containingprotein/lipid-bi
Aradu.YEP9N	Araip.55652	multiple-copy	Polyproteinn=5Tax=CitrusendogenouspararetrovirusRepl
Aradu.DZ5Y1	Araip.47DVE	multiple-copy	proline-richprotein4
Aradu.V4VHC	Araip.I10GN	multiple-copy	pumilio2
Aradu.N2G7A	Araip.NEM0P	multiple-copy	pyruvatedehydrogenaseE1beta
Aradu.X1LTA	Araip.31XHT	multiple-copy	Pyruvatekinasefamilyprotein
Aradu.49BKS	Araip.9CB1N	multiple-copy	QWRFmotif-containingprotein2-likeisoformX1[Glycinema
Aradu.WP30A	Araip.A5AHR	multiple-copy	respiratoryburstoxidasehomologA
Aradu.668QS	Araip.TVT35	multiple-copy	rhamnosebiosyntheticenzyme1-likeisoformX2[Glycinema
Aradu.B40DX	Araip.2GB7B	multiple-copy	ribosomalRNAsmallsubunitmethyltransferaseA
Aradu.1A2PM	Araip.KT98T	multiple-copy	sec-independentproteintranslocase
Aradu.WB7Y4	Araip.CSG3E	multiple-copy	seedmaturationPM36-likeprotein
Aradu.Z8G1Y	Araip.F4PJ9	multiple-copy	Sequence-specificDNAbindingtranscriptionfactorn=1Tax=
Aradu.1Y660	Araip.00X39	multiple-copy	serine/argininerepetitivematrixprotein2-like[Glycinemax]
Aradu.T693K	Araip.FP06E	multiple-copy	SETdomain-containingprotein

Aradu.AAN3U	Araip.S9EB1	multiple-copy	sigmafactorsigbregulationrsbq-likeprotein
Aradu.NEL01	Araip.7EC8V	multiple-copy	signalpeptidepeptidase
Aradu.79Z90	Araip.SJD2F	multiple-copy	signalrecognitionparticle14kDaprotein
Aradu.QY7MG	Araip.I28F8	multiple-copy	sisterchromatidcohesion1protein2-likeisoformX1[Glyciner
Aradu.F40AG	Araip.19CBQ	multiple-copy	SNAREassociatedGolgiproteinfamily
Aradu.92WMB	Araip.6JD6V	multiple-copy	Sterilealphamotif(SAM)domain-containingprotein
Aradu.M0QV5	Araip.KHC1F	multiple-copy	succinatedehydrogenase
Aradu.S8QXC	Araip.1BW4F	multiple-copy	Tesmin/TSO1-likeCXCdomain-containingprotein
Aradu.4V3YY	Araip.C9YWV	multiple-copy	Thaumatinsuperfamilyprotein
Aradu.XV49R	Araip.HCE40	multiple-copy	traffickingproteinparticlecomplexsubunit9-like[Glycinemax
Aradu.QII1E	Araip.D53S9	multiple-copy	transcriptionelongationfactor(TFIIS)familyprotein
Aradu.KRY7W	Araip.C6PK9	multiple-copy	trehalosephosphatesynthase
Aradu.M73X9	Araip.Q1514	multiple-copy	tryptophansynthasealphachain
Aradu.YQ4V9	Araip.H0886	multiple-copy	UbiquitinsystemcomponentCueprotein
Aradu.I0WQG	Araip.88RV1	multiple-copy	UPSTREAMOFFLC-likeisoformX1[Glycinemax]
Aradu.WIQ29	Araip.CBB88	multiple-copy	vacuolar(H+)-ATPaseGsubunit
Aradu.8G81K	Araip.0C8GZ	multiple-copy	winged-helixDNA-bindingtranscriptionfactorfamilyprotein
Aradu.640E1	Araip.3SL7S	multiple-copy	abscisicacidreceptor
Aradu.R9PSM	Araip.4YP5T	multiple-copy	actin-relatedprotein2/3complexsubunit2A-like[Glycinema
Aradu.9SL8H	Araip.220UP	multiple-copy	acyl-CoA-bindingdomain3
Aradu.I69M4	Araip.ER0KT	multiple-copy	adenylatekinase,putative
Aradu.53H6W	Araip.7KZ5D	multiple-copy	alkaline/neutralinvertase
Aradu.T5CVQ	Araip.1F56R	multiple-copy	aluminum-activated,malatetransporter12
Aradu.9BC7H	Araip.FX5SI	multiple-copy	annexin1
Aradu.NI3KM	Araip.P0CYG	multiple-copy	ATPsynthase,F1betasubunit
Aradu.YSU0C	Araip.6J0KF	multiple-copy	B3DNA-bindingdomainprotein
Aradu.UA4JV	Araip.44RH2	multiple-copy	BEL1-likehomeodomainprotein1-likeisoformX3[Glycinema
Aradu.K39P9	Araip.F1SXZ	multiple-copy	benzylalcoholO-benzoyltransferase[Glycinemax]
Aradu.KR6D1	Araip.MS1GW	multiple-copy	calcium-bindingproteinCML20[Glycinemax]
Aradu.RM7GK	Araip.22IQY	multiple-copy	casparianstripmembraneprotein
Aradu.GS1LC	Araip.FB76I	multiple-copy	cationicaminoacidtransporter2
Aradu.F77GY	Araip.TUB1K	multiple-copy	celldivisioncycle5-likeprotein-likeisoformX2[Glycinemax]
Aradu.H129B	Araip.135BH	multiple-copy	ClassIglutamineamidotransferase-likesuperfamilyprotein
Aradu.NCJ0H	Araip.26B5V	multiple-copy	Copperamineoxidasefamilyprotein
Aradu.M3S97	Araip.C3AMC	multiple-copy	Dyneinlightchaintype1familyprotein
Aradu.BW310	Araip.G5FLL	multiple-copy	EPIDERMALPATTERNINGFACTOR-likeprotein1-like[Gly
Aradu.4DA0K	Araip.4B2QS	multiple-copy	filament-likeplantprotein3-likeisoformX1[Glycinemax]
Aradu.Q4TAZ	Araip.5XX3F	multiple-copy	GATAtypezincfingertranscriptionfactorfamilyprotein
Aradu.J1AYY	Araip.4K0TW	multiple-copy	glyceraldehyde-3-phosphatedehydrogenaseC2
Aradu.2RV29	Araip.0G32G	multiple-copy	glycerol-3-phosphateacyltransferase2
Aradu.5X3H9	Araip.AM46T	multiple-copy	glycosylhydrolasefamilyprotein43
Aradu.MVB2G	Araip.FHM8U	multiple-copy	GRAMdomain-containingprotein/ABA-responsiveprotein-r
Aradu.75PY6	Araip.6YN77	multiple-copy	growth-regulatingfactor2
Aradu.R5ZHP	Araip.12IR2	multiple-copy	guaninenucleotide-bindingproteinalpha-2subunit-likeisofo

Aradu.SA3GU	Araip.KR2BY	multiple-copy	Ironionbinding/oxidoreductase/oxidoreductaseproteinn=1
Aradu.VJ0SY	Araip.7A7AE	multiple-copy	LONGIFOLIA1-likeisoformX2[Glycinemax]
Aradu.ZA2RQ	Araip.A2PE6	multiple-copy	Lungseventransmembranereceptorfamilyprotein
Aradu.VS4QJ	Araip.1ZK8J	multiple-copy	Mechanosensitiveionchannelfamilyprotein
Aradu.34ACD	Araip.07XD6	multiple-copy	melanoma-associatedantigen10-likeisoformX3[Glycinema
Aradu.234X6	Araip.2Q3AI	multiple-copy	N-acetyltransferaseHLS1-like[Glycinemax]
Aradu.WW65Q	Araip.6P6UG	multiple-copy	ninja-familyproteinAFP3-likeisoformX4[Glycinemax]
Aradu.3SS5K	Araip.S7U5R	multiple-copy	nuclearporecomplexproteinNup155-like[Glycinemax]
Aradu.67X2R	Araip.T3WCA	multiple-copy	nucleartranscriptionfactorYsubunitA-3-likeisoform3[Glycin
Aradu.74N2Q	Araip.03Q00	multiple-copy	nucleotidebinding
Aradu.2PT1E	Araip.953FR	multiple-copy	nucleotide-diphospho-sugartransferasedomainprotein
Aradu.54FG7	Araip.P1WT2	multiple-copy	octicosapeptide/Phox/Bem1p(PB1)domain-containingpro
Aradu.B353U	Araip.N6ZTJ	multiple-copy	oxygenevolvingcomplexofphotosystemIIn=5Tax=Sonnera
Aradu.T6XMF	Araip.D0D33	multiple-copy	Oxysterol-bindingfamilyprotein
Aradu.B7P36	Araip.M1IU9	multiple-copy	Peptidechainreleasefactor1
Aradu.WJ709	Araip.IWA7R	multiple-copy	phosphofructokinase2
Aradu.I4SX6	Araip.2NP4Y	multiple-copy	PLATZtranscriptionfactorfamilyprotein
Aradu.R5FQX	Araip.BVY6Z	multiple-copy	Polyketidecyclase/dehydraseandlipidtransportsuperfamily
Aradu.Z156R	Araip.78TK8	multiple-copy	responseregulator2
Aradu.Y54CT	Araip.WBT3S	multiple-copy	rhoGDP-dissociationinhibitor1[Glycinemax]
Aradu.D7PPK	Araip.80R5B	multiple-copy	RNAmethyltransferasefamilyprotein
Aradu.GJ1CE	Araip.7V9BH	multiple-copy	seedlinoleate9S-lipoxygenase
Aradu.HC4I0	Araip.9M15N	multiple-copy	serinehydroxymethyltransferase2
Aradu.N1IDK	Araip.JAR4Q	multiple-copy	splicingfactor3asubunit3,putative
Aradu.15R8P	Araip.33H23	multiple-copy	Structuralconstituentofribosome,putativen=1Tax=Ricinus
Aradu.33VFZ	Araip.0XT2W	multiple-copy	sugarphosphate/phosphatetranslocator[Glycinemax]
Aradu.B2STS	Araip.TDZ3S	multiple-copy	SWIB/MDM2domainprotein
Aradu.ND96S	Araip.DXJ40	multiple-copy	TCP-1/cpn60chaperoninfamilyprotein
Aradu.6K5DV	Araip.P8FX2	multiple-copy	telomererepeat-bindingfactor1-likeisoformX2[Glycinemax
Aradu.N7VLT	Araip.069K4	multiple-copy	TPX2(targetingproteinforXklp2)proteinfamily
Aradu.L5LVS	Araip.G8PK0	multiple-copy	transmembrane9superfamilymember3-like[Glycinemax]
Aradu.7H178	Araip.3K50N	multiple-copy	trihelixGT-2-like[Glycinemax]
Aradu.BB920	Araip.76E63	multiple-copy	tRNAwybutosine-synthesizingprotein1homolog[Glycinem
Aradu.2QS2R	Araip.UT6J6	multiple-copy	UDP-glucuronate:xylanalpha-glucuronosyltransferase1-lil
Aradu.YIH2Y	Araip.3S8EX	multiple-copy	Water-selectivetransportintrinsicmembraneprotein1n=1Ta
Aradu.475J2	Araip.167J0	multiple-copy	WPPdomainassociatedprotein
Aradu.2HD04	Araip.274S3	multiple-copy	WUSCHELrelatedhomeobox1
Aradu.YIG8H	Araip.64EI8	multiple-copy	zinc-bindingalcoholdehydrogenasefamilyprotein
Aradu.P759U	Araip.6U9BD	multiple-copy	alcoholdehydrogenase1
Aradu.4SA47	Araip.29FLU	multiple-copy	argininemethyltransferase10
Aradu.1RK1Y	Araip.0N0ZT	multiple-copy	ARMrepeatsuperfamilyprotein
Aradu.F8DL0	Araip.YJ9H2	multiple-copy	ATP-dependentzincmetalloproteaseFTSH10,mitochondria
Aradu.919C4	Araip.6IE3Z	multiple-copy	B-cellreceptor-associated31-like
Aradu.ILZ3Q	Araip.4B1V4	multiple-copy	Beta-1,3-N-Acetylglucosaminyltransferasefamilyprotein

Aradu.HVZ6R	Araip.A7M57	multiple-copy	calcium-transportingATPase,putative
Aradu.TZG12	Araip.Y99XU	multiple-copy	carboxylesterase11-like[Glycinemax]
Aradu.VUQ4V	Araip.BRP4U	multiple-copy	cinnamylalcoholdehydrogenase5
Aradu.76Y0H	Araip.0H44R	multiple-copy	cleavageandpolyadenylationspecificityfactor100
Aradu.KZ2JX	Araip.7TY5U	multiple-copy	cysteineproteinaseinhibitor[Glycinemax]
Aradu.543MU	Araip.5804B	multiple-copy	cytokininoxidase/dehydrogenase1
Aradu.YE0CC	Araip.6I2P8	multiple-copy	DHHC-typezincfingerfamilyprotein
Aradu.LFL4V	Araip.UE5VC	multiple-copy	dualspecificityproteinkinaseshkD-like[Glycinemax]
Aradu.V4LAJ	Araip.CS3DH	multiple-copy	epoxidehydrolase
Aradu.B8V9A	Araip.21REB	multiple-copy	ERD(early-responsivetodehydrationstress)familyprotein
Aradu.B81RW	Araip.N7CQ0	multiple-copy	FASCICLIN-likearabinogalactan-protein11
Aradu.2F2F8	Araip.00QX5	multiple-copy	generalregulatoryfactor12
Aradu.5N3RR	Araip.9Q1HG	multiple-copy	Histonesuperfamilyprotein
Aradu.ZIW9T	Araip.XN9LA	multiple-copy	hsp20/alphacrystallinfamilyprotein
Aradu.G4DCM	Araip.9U2AW	multiple-copy	HVA22homologueA
Aradu.U5CVT	Araip.UUG0Y	multiple-copy	integralmembranefamilyprotein
Aradu.N2D1U	Araip.9D7FV	multiple-copy	iron-sulfurclusterassemblyproteinIscA
Aradu.452B2	Araip.02N4R	multiple-copy	isocitratedehydrogenase
Aradu.Q4Y5D	Araip.6V0UJ	multiple-copy	lactoylglutathionelyase
Aradu.EZW4U	Araip.1UW0G	multiple-copy	leguminosingroup485secretedpeptide
Aradu.70NQH	Araip.M3B88	multiple-copy	leucine-richrepeat-containingproteinDDB_G0290503-likei
Aradu.HLK79	Araip.X9IXZ	multiple-copy	lysine-specificdemethylase5D-like[Glycinemax]
Aradu.N636R	Araip.TQJ7V	multiple-copy	membraneprotein,putative
Aradu.03ENG	Araip.0E4ZE	multiple-copy	Non-specificlipid-transferprotein,putative
Aradu.WB5Q0	Araip.5MD11	multiple-copy	Nucleartransportfactor2(NTF2)familyprotein
Aradu.U99RK	Araip.2E6XX	multiple-copy	OTU-likecysteineprotease
Aradu.N0CP9	Araip.8W43D	multiple-copy	pfkB-likecarbohydratekinasefamilyprotein
Aradu.D4CLJ	Araip.P78GJ	multiple-copy	proteasomesubunitalphatype-6-Aprotein
Aradu.6V13C	Araip.HLU05	multiple-copy	Quinonereductasefamilyprotein
Aradu.H49AM	Araip.M3BQV	multiple-copy	replicationproteinA32kDasubunit-likeprotein
Aradu.J3LFY	Araip.04TGY	multiple-copy	Rhodanese/Cellcyclecontrolphosphatasesuperfamilyprote
Aradu.CDK6C	Araip.3RS0Y	multiple-copy	SAUR-likeauxin-responsiveproteinfamily
Aradu.Z8VLV	Araip.216VR	multiple-copy	Sec23/Sec24proteintransportfamilyprotein
Aradu.4FK3V	Araip.49NYC	multiple-copy	Snf1-relatedkinaseinteractor1,putative
Aradu.M35ST	Araip.ZWY76	multiple-copy	squamosapromoter-binding-likeprotein6-likeisoformX1[Gl
Aradu.629Q2	Araip.KZ1TD	multiple-copy	sucrosetransporter2
Aradu.8PJ2J	Araip.89K67	multiple-copy	sugarporter(SP)familyMFStransporter
Aradu.90SID	Araip.Y03QP	multiple-copy	sulfatetransporter1
Aradu.23Z4K	Araip.W7597	multiple-copy	T-complexprotein11
Aradu.VY9WF	Araip.363DR	multiple-copy	typeIinositol-1,4,5-trisphosphate5-phosphatase
Aradu.88HY6	Araip.M0RYJ	multiple-copy	zincionbinding
Aradu.50IM9	Araip.66A5Z	multiple-copy	ACTdomain-containingprotein
Aradu.YZR0Z	Araip.0311L	multiple-copy	beta-fructofuranosidase
Aradu.S7NAJ	Araip.5Y6S2	multiple-copy	COP9signalosomecomplexsubunit1

Aradu.772NM	Araip.65SLH	multiple-copy	disulfideisomerase-like
Aradu.GL73Z	Araip.D9CPB	multiple-copy	DNAmismatchrepairMUTSfamilyprotein
Aradu.1TF3W	Araip.S1X2Y	multiple-copy	exocystsubunitexo70familyproteinA2
Aradu.IV8SJ	Araip.C6CF4	multiple-copy	expansinA1
Aradu.K3RPT	Araip.F3DK3	multiple-copy	FAD-bindingBerberinefamilyprotein
Aradu.FS4N2	Araip.1V67D	multiple-copy	Gibberellin-regulatedfamilyprotein
Aradu.B2Z42	Araip.4KB77	multiple-copy	GPI-anchoredprotein[Glycinemax]
Aradu.QCF5U	Araip.2D392	multiple-copy	histonedeacetylase
Aradu.5D1VA	Araip.CD2TQ	multiple-copy	interactorofconstitutiveactiveROPs2,chloroplastic-likeisof
Aradu.T09Z3	Araip.2D0HD	multiple-copy	MACPFdomain-containingproteinAt4g24290-likeisoformX
Aradu.HH10J	Araip.986AT	multiple-copy	majorintrinsicprotein(MIP)familytransporter
Aradu.62BQ5	Araip.4FJ07	multiple-copy	MembranetransporterD1n=3Tax=AndropogoneaeRepID=
Aradu.U7LA9	Araip.GM32M	multiple-copy	mitochondrialimportinnermembranetranslocasesubunittim
Aradu.G2GUD	Araip.C1PNV	multiple-copy	myosin1
Aradu.32310	Araip.JHV8K	multiple-copy	patatin-likephospholipasedomainprotein
Aradu.S2UYW	Araip.U91QP	multiple-copy	peptidyl-prolylcis-transisomeraseCYP40-likeisoformX1[G
Aradu.ZC0SW	Araip.QVW26	multiple-copy	phosphatetransporter1
Aradu.D7Y0Z	Araip.CX0EJ	multiple-copy	phosphatidylinositol-4-phosphate5-kinase1
Aradu.EQB6S	Araip.KHF35	multiple-copy	Phosphoglyceratemutase,2,3-bisphosphoglycerate-indep
Aradu.IPN47	Araip.D4SGT	multiple-copy	phospholipid-transportingATPase1-likeisoformX1[Glycine
Aradu.XF7S6	Araip.2Z41P	multiple-copy	Plastid-lipidassociatedproteinPAP/fibrillinfamilyprotein
Aradu.F9L0R	Araip.0I82K	multiple-copy	potassiumtransporter2
Aradu.T2QMK	Araip.6U899	multiple-copy	PPPDEputativethiolpeptidasefamilyprotein
Aradu.F0ADU	Araip.3GM3S	multiple-copy	Pseudouridinesynthasefamilyprotein
Aradu.FN1D7	Araip.Z4GS0	multiple-copy	purinepermease1
Aradu.6K05J	Araip.B6USA	multiple-copy	Rab-GAPdomainofgyp1psuperfamilyprotein
Aradu.H2G5C	Araip.2I21U	multiple-copy	RegulatorofVps4activityintheMVBpathwayprotein
Aradu.5I6E9	Araip.H02JY	multiple-copy	Remorinfamilyprotein
Aradu.9ZP9L	Araip.FP4H9	multiple-copy	ribonuclease1
Aradu.JM0ZT	Araip.EI4LW	multiple-copy	ROPguaninenucleotideexchangefactor5
Aradu.G9EXB	Araip.5Z5WT	multiple-copy	single-strandedDNA-bindingprotein
Aradu.9B6LA	Araip.N6UK3	multiple-copy	V-typeprotonATPase16kDaproteolipidsubunit-like[Glycine
Aradu.G957G	Araip.8NY60	multiple-copy	wall-associatedreceptorkinase3-like[Glycinemax]
Aradu.I27F7	Araip.263ND	multiple-copy	ZIPzinc/irontransportfamilyprotein
Aradu.IZ11Y	Araip.BNQ5K	multiple-copy	30Sribosomalprotein,putative
Aradu.5Q6ZX	Araip.M3SVD	multiple-copy	50SribosomalL24-likeprotein
Aradu.VPD9Z	Araip.JA8PY	multiple-copy	ABCtransporter(ATP-bindingprotein),putative
Aradu.L1M1M	Araip.6S389	multiple-copy	Acyl-[acyl-carrier-protein]desaturasen=2Tax=SolanumRe
Aradu.A0Z5X	Araip.NP9KT	multiple-copy	aldehydedehydrogenase12A1
Aradu.C2N0T	Araip.0G24M	multiple-copy	alpha/betafoldhydrolase
Aradu.FUK65	Araip.F5F3I	multiple-copy	betaglucosidase11
Aradu.TQ01E	Araip.11VE0	multiple-copy	Bifunctionalinhibitor/lipid-transferprotein/seedstorage2Sal
Aradu.X57SP	Araip.10PYM	multiple-copy	CHUP1,chloroplastic-like[Glycinemax]
Aradu.A6WCN	Araip.03WUR	multiple-copy	floweringlocusproteinT

Aradu.33DTZ	Araip.TV6SE	multiple-copy	galacturonosyltransferase10-like[Glycinemax]
Aradu.A1GM4	Araip.8T0TC	multiple-copy	GATAtranscriptionfactor11
Aradu.85KJF	Araip.1MS7W	multiple-copy	GTPbindingprotein,putativen=1Tax=RicinuscommunisRe
Aradu.J31ZQ	Araip.VH0NK	multiple-copy	histoneH1-3
Aradu.RMG12	Araip.T5YYS	multiple-copy	homeobox-leucinezipperprotein17
Aradu.EKZ3T	Araip.N5JHA	multiple-copy	LRRandNB-ARCdomaindiseaseresistanceprotein
Aradu.4IN7K	Araip.N2LA6	multiple-copy	LURP-one-likeprotein
Aradu.JK0W0	Araip.123NR	multiple-copy	Majorfacilitatorsuperfamilyprotein
Aradu.432N5	Araip.B2BPT	multiple-copy	NADH-ubiquinoneoxidoreductase39kDasubunit
Aradu.31IVL	Araip.H4E0W	multiple-copy	Nucleicacid-binding,OB-fold-likeprotein
Aradu.IMH3N	Araip.00WVB	multiple-copy	Peroxisomalmembrane22kDa(Mpv17/PMP22)familyprote
Aradu.70JJH	Araip.ZXB9C	multiple-copy	PHDandRINGfingerdomain-containingprotein1n=2Tax=T
Aradu.UH61L	Araip.1T9WY	multiple-copy	P-loopcontainingnucleosidetriphosphatehydrolasessupert
Aradu.R2VBI	Araip.BW8ER	multiple-copy	pre-mRNA-splicingfactor
Aradu.G44I1	Araip.28HGC	multiple-copy	RHOMBOID-like1
Aradu.FH3GQ	Araip.AYG40	multiple-copy	RNApolymeraseIIC-terminaldomainphosphatase-like1-lik
Aradu.50ZYK	Araip.KY4DP	multiple-copy	Signaltransductionhistidinekinase
Aradu.572L7	Araip.IL4VZ	multiple-copy	terpenesynthase02
Aradu.3Q3XZ	Araip.386YD	multiple-copy	tetraspanin-10-like[Glycinemax]
Aradu.92KA6	Araip.N6BQZ	multiple-copy	TPRrepeat-containingthioredoxinTDX-like[Glycinemax]
Aradu.ZW38I	Araip.YR061	multiple-copy	vesicle-associatedmembraneprotein711
Aradu.WLX6I	Araip.MJ1PB	multiple-copy	WEBfamilyproteinAt1g12150-like[Glycinemax]
Aradu.N3SPH	Araip.12MTY	multiple-copy	acyl-CoAsynthetase5
Aradu.TE378	Araip.1E0RS	multiple-copy	AThookmotifDNA-bindingfamilyprotein
Aradu.RF6XK	Araip.D37IR	multiple-copy	ATP-dependentRNAhelicase,mitochondrial,putative
Aradu.L0S59	Araip.73WZS	multiple-copy	betagalactosidase1
Aradu.T720W	Araip.8UW3Z	multiple-copy	CBSdomain-containingprotein
Aradu.7JU4H	Araip.0W082	multiple-copy	coiled-coildomain-containingprotein130-like[Glycinemax]
Aradu.RW8B0	Araip.HBQ1U	multiple-copy	cytochromeB561-1
Aradu.U14K2	Araip.HU9EV	multiple-copy	DNApolymerasealpha2
Aradu.L5T1V	Araip.116UB	multiple-copy	Galactoseoxidase/kelchrepeatsuperfamilyprotein
Aradu.WWA28	Araip.583LJ	multiple-copy	Glutaredoxinfamilyprotein
Aradu.LZ48I	Araip.C42Y7	multiple-copy	L-ascorbateoxidase[Glycinemax]
Aradu.TB0L3	Araip.3047C	multiple-copy	light-harvestingchlorophyllB-bindingprotein3
Aradu.767UQ	Araip.S0E50	multiple-copy	magnesiumtransporter2
Aradu.WM652	Araip.3Y8R0	multiple-copy	microtubule-associatedprotein65-4
Aradu.Z0E8S	Araip.8WA63	multiple-copy	nucleobase-ascorbatetransporter12
Aradu.SQ2QT	Araip.0Q5CU	multiple-copy	Pectatelyasefamilyprotein
Aradu.ZWN25	Araip.536TB	multiple-copy	transferringglycosylgrouptransferase
Aradu.F8JPN	Araip.60Q87	multiple-copy	Ubiquitin-likesuperfamilyprotein
Aradu.926HI	Araip.2CJ7I	multiple-copy	Ulp1proteasefamily,carboxy-terminaldomainprotein
Aradu.IZ7V8	Araip.XEF5B	multiple-copy	xyloglucanendotransglucosylase/hydrolase10
Aradu.E4IDB	Araip.H9JFA	multiple-copy	26Sproteaseregulatorysubunit6Bhomolog[Glycinemax]
Aradu.XSB36	Araip.UJ6B2	multiple-copy	3-ketoacyl-CoAsynthase1

Aradu.B04QQ	Araip.Q8Y00	multiple-copy	adeninenucleotidealphahydrolase-likedomainkinase
Aradu.JVL5D	Araip.44PA5	multiple-copy	aldo/ketoreductasefamilyoxidoreductase
Aradu.P9J09	Araip.U1RD3	multiple-copy	cellulosesynthase1
Aradu.0R9W1	Araip.01AT6	multiple-copy	cyclicnucleotide-gatedionchannelprotein,putative
Aradu.199XC	Araip.HJX61	multiple-copy	dentinsialophosphoprotein-like[Glycinemax]
Aradu.RF66L	Araip.0301B	multiple-copy	DNArepair(Rad51)familyprotein
Aradu.26IB4	Araip.CUS24	multiple-copy	DNAreplicationcomplexGINSprotein
Aradu.3YH97	Araip.3X74X	multiple-copy	Flavin-bindingmonooxygenasefamilyprotein
Aradu.QU90K	Araip.U6WSS	multiple-copy	Galactosyltransferasefamilyprotein
Aradu.D5GAK	Araip.1T874	multiple-copy	gibberellin20oxidase1-like[Glycinemax]
Aradu.VN8X3	Araip.HGV90	multiple-copy	glucose6-phosphate/phosphatetranslocator1
Aradu.577R9	Araip.390JY	multiple-copy	homeoboxproteinknotted-1-like10-likeisoformX3[Glycinem
Aradu.ZL6EF	Araip.R86PR	multiple-copy	NAD-dependentepimerase/dehydratasefamilyprotein
Aradu.JA2NX	Araip.K776G	multiple-copy	nitratetransporter1.5
Aradu.2P8HG	Araip.JQ4V7	multiple-copy	short-chaindehydrogenase/reductase
Aradu.22C31	Araip.GYE3T	multiple-copy	syntaxin,putative
Aradu.XV2LW	Araip.ARS4V	multiple-copy	two-componentresponseregulatorARR2-like[Glycinemax]
Aradu.U20ZR	Araip.6ME1P	multiple-copy	Auxineffluxcarrierfamilyprotein
Aradu.CW450	Araip.20CWB	multiple-copy	calcium-bindingEFhandfamilyprotein
Aradu.G4DWJ	Araip.0A5UL	multiple-copy	chromodomain-helicase-DNA-bindingprotein1-likeisoform
Aradu.257BX	Araip.1R8Y6	multiple-copy	Cyclophilin-likepeptidyl-prolylcis-transisomerasefamilypro
Aradu.JAV3X	Araip.J8RG7	multiple-copy	IQ-DOMAIN14-like[Glycinemax]
Aradu.T4HI5	Araip.2G506	multiple-copy	myosinheavychain-related
Aradu.EV17X	Araip.64GPS	multiple-copy	NAD(P)-bindingrossmann-foldprotein
Aradu.6Y8G9	Araip.NGD50	multiple-copy	nudixhydrolasehomolog17
Aradu.D8H5F	Araip.5582Q	multiple-copy	Regulatorofchromosomecondensation(RCC1)familyprote
Aradu.7AZ8Z	Araip.KZ8MV	multiple-copy	transcriptioninitiationfactorIIAsubunit2
Aradu.Q566Q	Araip.8M8IP	multiple-copy	chitinaseA
Aradu.D9DNB	Araip.AZY85	multiple-copy	earlynodulin-likeprotein10
Aradu.61W2G	Araip.TUL3I	multiple-copy	endoglucanase[Glycinemax]
Aradu.B26BB	Araip.P0KMD	multiple-copy	glycosyltransferaseAt3g07620-like[Glycinemax]
Aradu.U9IPR	Araip.2404G	multiple-copy	heatshocktranscriptionfactorA2
Aradu.5R97S	Araip.VG3KL	multiple-copy	purpleacidphosphatase15
Aradu.E8RZK	Araip.35416	multiple-copy	Reticulonfamilyprotein
Aradu.431XY	Araip.3W1KE	multiple-copy	autophagy3(APG3)protein
Aradu.D77VM	Araip.WT3SV	multiple-copy	B-zipDNAbindingproteinn=4Tax=CucumisRepID=E5GB6
Aradu.T8M37	Araip.V9NIV	multiple-copy	calciumdependentproteinkinase1
Aradu.FR1WN	Araip.65AV8	multiple-copy	Core-2/I-branchingbeta-1,6-N-acetylglucosaminyltransfer
Aradu.U6F8I	Araip.6SM52	multiple-copy	indole-3-aceticacid-amidosynthetaseGH3.9
Aradu.JZR6I	Araip.0K8ZX	multiple-copy	Lipidtransferprotein
Aradu.32X0N	Araip.560IW	multiple-copy	phospholipaseA1-Ibeta2,chloroplastic-like[Glycinemax]
Aradu.K64M1	Araip.2C3K4	multiple-copy	PollenOlee1allergenandextensinfamilyprotein
Aradu.AF9V9	Araip.0JY6V	multiple-copy	TGACG-sequence-specificDNA-bindingproteinTGA-1B-lik
Aradu.YPV42	Araip.6Q4X9	multiple-copy	transcriptionfactor

Aradu.I4X46	Araip.DW9H3	multiple-copy	vacuolar-processingenzyme-like[Glycinemax]
Aradu.ILB9Z	Araip.P6KBN	multiple-copy	1-aminocyclopropane-1-carboxylateoxidase
Aradu.C0NTR	Araip.2V326	multiple-copy	C2H2-likezincfingerprotein
Aradu.7T5LQ	Araip.1CJ82	multiple-copy	FKBP-likepeptidyl-prolylcis-transisomerasefamilyprotein
Aradu.A6HVN	Araip.M3BFQ	multiple-copy	histone-lysineN-methyltransferase
Aradu.J25NN	Araip.86EDZ	multiple-copy	laccase10
Aradu.UNY97	Araip.1FA5F	multiple-copy	O-methyltransferase1
Aradu.9EZ7Z	Araip.HDT92	multiple-copy	sugartransporter1
Aradu.N7YFC	Araip.7LD9B	multiple-copy	ubiquitincarboxyl-terminalhydrolase
Aradu.KS2FL	Araip.IIQ50	multiple-copy	UPF0369proteinC6orf57-likeisoformX2[Glycinemax]
Aradu.R1ZAI	Araip.AX32F	multiple-copy	ADP-ribosylationfactor1
Aradu.Q0XNV	Araip.6Q6V5	multiple-copy	Cysteineproteinasessuperfamilyprotein
Aradu.V9CJA	Araip.1Y485	multiple-copy	HXXXD-typeacyl-transferasefamilyprotein
Aradu.8KH6E	Araip.F0RXE	multiple-copy	MADS-boxfamilyprotein
Aradu.NJI26	Araip.54QEU	multiple-copy	mediatorofRNApolymeraseIItranscriptionsubunit11
Aradu.M0DM1	Araip.C60UZ	multiple-copy	oxidoreductase,2OG-Fe(II)oxygenasefamilyprotein
Aradu.58DAR	Araip.YC0K3	multiple-copy	photosystemII10kDaproteinPsbRprotein
Aradu.KNX76	Araip.50XHI	multiple-copy	calmodulin-bindingfamilyprotein
Aradu.KY185	Araip.E5LM9	multiple-copy	cation/H+exchanger18
Aradu.BM2UV	Araip.VEC9V	multiple-copy	cellwallproteinAWA1-likeisoformX2[Glycinemax]
Aradu.M0G9T	Araip.7I1D0	multiple-copy	Dof-typezincfingerDNA-bindingfamilyprotein
Aradu.2AU2Z	Araip.C9HSJ	multiple-copy	DUF21domainplantprotein
Aradu.2P1NS	Araip.4B6XP	multiple-copy	pathogenesis-likeprotein
Aradu.6K81G	Araip.K3V5A	multiple-copy	ubiquitin-conjugatingenzyme13
Aradu.G8ESS	Araip.T484U	multiple-copy	40SribosomalproteinS11[Glycinemax]
Aradu.X3TFJ	Araip.20IHP	multiple-copy	glutathioneS-transferase,amine-terminaldomainprotein
Aradu.F360A	Araip.4CZ22	multiple-copy	GTP-bindingnuclearproteinRan-3[Glycinemax]
Aradu.Z7SDW	Araip.VZ6R4	multiple-copy	HAD-familyhydrolaseIIA
Aradu.K10TZ	Araip.11N45	multiple-copy	peptidetransporter1
Aradu.3X77Q	Araip.62MQQ	multiple-copy	S-adenosyl-L-methionine-dependentmethyltransferasess
Aradu.N7IXY	Araip.N9QES	multiple-copy	SCARECROW-like[Glycinemax]
Aradu.Z922D	Araip.02EM5	multiple-copy	aspartylproteasefamilyprotein
Aradu.9V11P	Araip.81HNQ	multiple-copy	bluecopperprotein-like[Glycinemax]
Aradu.M6THB	Araip.03W6H	multiple-copy	Calcium-dependentlipid-binding(CaLBdomain)familyprote
Aradu.BBP4Z	Araip.00P1B	multiple-copy	MATEeffluxfamilyprotein
Aradu.2L1WI	Araip.3UE1W	multiple-copy	RING/FYVE/PHD-typezincfingerfamilyprotein
Aradu.7WJ9D	Araip.041RZ	multiple-copy	Sec14p-likephosphatidylinositoltransferfamilyprotein
Aradu.B0BA3	Araip.7P6A7	multiple-copy	serinecarboxypeptidase-like11
Aradu.J090Y	Araip.6C47W	multiple-copy	DEAD-boxATP-dependentRNAhelicase
Aradu.R6RRG	Araip.JFS3P	multiple-copy	polyamineoxidase4
Aradu.7Z2RH	Araip.DU7GL	multiple-copy	RING/U-boxproteinwithC6HC-typezincfinger
Aradu.WQI5W	Araip.85DUV	multiple-copy	subtilisin-likeprotease-like[Glycinemax]
Aradu.N64VX	Araip.A0AXY	multiple-copy	60SribosomalL12-likeprotein
Aradu.0YY6A	Araip.37KVD	multiple-copy	thioredoxin2

Aradu.Q507J	Araip.8B7U9	multiple-copy	ChaperoneDnaJ-domainsuperfamilyprotein
Aradu.UDE9J	Araip.H48JL	multiple-copy	pectinesterase/pectinesteraseinhibitor12-like[Glycinemax
Aradu.8T38A	Araip.177Y0	multiple-copy	nodulinMtN21/EamA-liketransporterfamilyprotein
Aradu.W9DC5	Araip.J92VB	multiple-copy	translationinitiationfactor1A-likeprotein
Aradu.235VA	Araip.54D8D	multiple-copy	YLS7-like[Glycinemax]
Aradu.DXP84	Araip.BYV33	multiple-copy	auxinresponsefactor10
Aradu.7Y1E8	Araip.HUA7A	multiple-copy	heatshockprotein
Aradu.H7VTY	Araip.60GBF	multiple-copy	Lateembryogenesisabundant(LEA)hydroxyproline-richgly
Aradu.U68KB	Araip.9C0A1	multiple-copy	LOBdomain-containingprotein1
Aradu.8UD68	Araip.3VW7G	multiple-copy	Mitochondrialsubstratecarrierfamilyprotein
Aradu.HHL1H	Araip.49HT7	multiple-copy	DNA-directedRNApolymerase
Aradu.JR8JR	Araip.4SN9W	multiple-copy	U-boxdomain-containingprotein13-like[Glycinemax]
Aradu.D8K1Y	Araip.NQ5HH	multiple-copy	DnaJ/Hsp40cysteine-richdomainsuperfamilyprotein
Aradu.70NLN	Araip.2539H	multiple-copy	tetratricopeptidedomainthioredoxin
Aradu.9M2XX	Araip.C4VS6	multiple-copy	cyclina2
Aradu.X3GS1	Araip.993UZ	multiple-copy	ethylene-responsivetranscriptionfactor12[Glycinemax]
Aradu.NB0W9	Araip.D723S	multiple-copy	alpha/betahydrolasedomain-containingprotein11-like[Glyc
Aradu.79FBE	Araip.45C6Q	multiple-copy	methyltransferaseAt1g22800-likeisoformX6[Glycinemax]
Aradu.RU8ZD	Araip.1P6L4	multiple-copy	transmembraneaminoacidtransporterfamilyprotein
Aradu.K1JIV	Araip.4F02G	multiple-copy	ankyrinrepeat-containingprotein[Glycinemax]
Aradu.IZ2A8	Araip.0TX9X	multiple-copy	phosphatase2Cfamily
Aradu.4T0IX	Araip.ENG6G	multiple-copy	RibosomalL22eproteinfamily
Aradu.UIA4U	Araip.0B6PB	multiple-copy	2-oxoglutarate(2OG)andFe(II)-dependentoxygenasesupe
Aradu.N90WJ	Araip.4T3QB	multiple-copy	BTB/POZdomain-containingprotein
Aradu.W56IJ	Araip.1V89E	multiple-copy	DNAbinding
Aradu.B2BUH	Araip.J67VV	multiple-copy	transmembraneprotein
Aradu.P432A	Araip.0P2W0	multiple-copy	E3ubiquitinligaseBIGBROTHER-likeisoformX4[Glycinema
Aradu.M414E	Araip.044E8	multiple-copy	Heavymetaltransport/detoxificationsuperfamilyprotein
Aradu.TP7W2	Araip.CVD1W	multiple-copy	FAR1DNA-bindingdomainprotein
Aradu.6X9VQ	Araip.BLV36	multiple-copy	UDP-glycosyltransferase74F1
Aradu.PM5NX	Araip.05XM7	multiple-copy	WRKYfamilytranscriptionfactor
Aradu.FU1XB	Araip.G3PYU	multiple-copy	GDSL-likelipase/acylhydrolase
Aradu.YFQ3P	Araip.8NR3H	multiple-copy	NACdomaincontainingprotein102
Aradu.7I7HI	Araip.64TPH	multiple-copy	glucanendo-1,3-beta-D-glucosidase-like[Glycinemax]
Aradu.UD5FH	Araip.P4GTJ	multiple-copy	RINGfingerandCHYzincfingerprotein
Aradu.12T0R	Araip.JSZ6P	multiple-copy	peroxidase2
Aradu.H1FFF	Araip.Q4KH3	multiple-copy	Serine/Threonine-kinase,putative
Aradu.S7Q9G	Araip.54FE9	multiple-copy	WD-40repeatfamilyprotein
Aradu.58KSZ	Araip.09N6L	multiple-copy	ATPbinding
Aradu.5CF9R	Araip.ERC12	multiple-copy	bHLH)DNA-bindingfamilyprotein
Aradu.65ZNS	Araip.K266S	multiple-copy	RNA-bindingprotein39-likeisoformX2[Glycinemax]
Aradu.4N3HV	Araip.64470	multiple-copy	diseaseresistanceprotein(TIR-NBS-LRRclass)
Aradu.L2PP0	Araip.FFF41	multiple-copy	zincfinger(C2H2type)familyprotein
Aradu.U2NUZ	Araip.KS63Q	multiple-copy	F-box/ankyrinrepeatproteinSKIP35-likeisoformX2[Glycine

Aradu.G3TTV	Araip.7H2KA	multiple-copy	Myb/SANT-likeDNA-bindingdomainprotein
Aradu.KNN0Y	Araip.FMC77	multiple-copy	cytochromeP450,family707,subfamilyA,polypeptide4
Aradu.C6P70	Araip.HK5CX	multiple-copy	Pentatricopeptiderepeat(PPR)superfamilyprotein
Aradu.2Q1S0	Araip.5YY4M	multiple-copy	receptorkinase1
Aradu.2J3VX	Araip.F86X8	multiple-copy	proteinkinase1
Aradu.6W1XZ	Araip.T0LEM	multiple-copy	1,4-alpha-glucan-branchingenzyme-like[Glycinemax]
Aradu.FV6YV	Araip.F1ZZD	multiple-copy	12-oxophytodienoatereductase2
Aradu.JF9VE	Araip.PFH2D	multiple-copy	1-acyl-sn-glycerol-3-phosphateacyltransferase-likeprotei
Aradu.CV82P	Araip.VW0QI	multiple-copy	1-aminocyclopropane-1-carboxylateoxidase
Aradu.8CB01	Araip.NL989	multiple-copy	1-aminocyclopropane-1-carboxylateoxidase1[Glycinema
Aradu.R3Z7V	Araip.Z6K8P	multiple-copy	1-aminocyclopropane-1-carboxylateoxidasehomolog12-li
Aradu.BK9AL	Araip.0I7VH	multiple-copy	1-aminocyclopropane-1-carboxylateoxidasehomolog1-lik
Aradu.2J2S3	Araip.BH9LK	multiple-copy	1-aminocyclopropane-1-carboxylateoxidasehomolog1-lik
Aradu.ACG3Y	Araip.I7WHX	multiple-copy	1-aminocyclopropane-1-carboxylateoxidasehomolog1-lik
Aradu.N8PYS	Araip.NWM0M	multiple-copy	1-aminocyclopropane-1-carboxylateoxidasehomolog1-lik
Aradu.X87GF	Araip.W3VW3	multiple-copy	1-aminocyclopropane-1-carboxylateoxidasehomolog1-lik
Aradu.DV28N	Araip.57AWT	multiple-copy	1-aminocyclopropane-1-carboxylatesynthase11
Aradu.EM688	Araip.FB4F0	multiple-copy	1-aminocyclopropane-1-carboxylatesynthase11
Aradu.Z0LGY	Araip.2S9Y0	multiple-copy	1-aminocyclopropane-1-carboxylatesynthase4
Aradu.55M8P	Araip.JB6HG	multiple-copy	1-aminocyclopropane-1-carboxylatesynthase9
Aradu.X763N	Araip.R98BP	multiple-copy	1-aminocyclopropane-1-carboxylatesynthase9
Aradu.ZI52D	Araip.K461S	multiple-copy	1-deoxy-D-xylulose5-phosphatesynthase1
Aradu.Q70DU	Araip.2F6VL	multiple-copy	26Sproteaseregulatorysubunit7-like[Glycinemax]
Aradu.384WQ	Araip.UJ6CM	multiple-copy	26Sproteaseregulatorysubunit7-like[Glycinemax]
Aradu.R8YHS	Araip.BN787	multiple-copy	26Sproteasomenon-ATPaseregulatorysubunit12homolog
Aradu.FJ32V	Araip.ULZ9V	multiple-copy	26Sproteasomenon-ATPaseregulatorysubunit3
Aradu.A5MHD	Araip.GD59T	multiple-copy	26Sproteasomenon-ATPaseregulatorysubunit7homologA
Aradu.TY4XK	Araip.6Y1SP	multiple-copy	26Sproteasomenon-ATPaseregulatorysubunit-likeprotein
Aradu.I8NTZ	Araip.ZEY7E	multiple-copy	26Sproteasomeregulatorycomplexcomponent
Aradu.J5WE7	Araip.84KVA	multiple-copy	26Sproteasomeregulatorysubunitn=8Tax=Sordariomycet
Aradu.6Y81Q	Araip.D9BFI	multiple-copy	26SproteasomeregulatorysubunitS21A
Aradu.Y4VNZ	Araip.C0YUJ	multiple-copy	2-aminoethanethioldioxygenase-like[Glycinemax]
Aradu.196ZM	Araip.J7PSL	multiple-copy	2-aminoethanethioldioxygenase-like[Glycinemax]
Aradu.QG2PF	Araip.1KN42	multiple-copy	2-oxoglutarate(2OG)andFe(II)-dependentoxygenasesupe
Aradu.2X4WG	Araip.2LP4Y	multiple-copy	2-oxoglutarate(2OG)andFe(II)-dependentoxygenasesupe
Aradu.Z6X71	Araip.3AQ0F	multiple-copy	2-oxoglutarate(2OG)andFe(II)-dependentoxygenasesupe
Aradu.353YW	Araip.3M5WT	multiple-copy	2-oxoglutarate(2OG)andFe(II)-dependentoxygenasesupe
Aradu.X81G8	Araip.42IVV	multiple-copy	2-oxoglutarate(2OG)andFe(II)-dependentoxygenasesupe
Aradu.U5UGY	Araip.5DJ69	multiple-copy	2-oxoglutarate(2OG)andFe(II)-dependentoxygenasesupe
Aradu.Z9DZQ	Araip.68F0J	multiple-copy	2-oxoglutarate(2OG)andFe(II)-dependentoxygenasesupe
Aradu.DU7VI	Araip.74F08	multiple-copy	2-oxoglutarate(2OG)andFe(II)-dependentoxygenasesupe
Aradu.2U1V2	Araip.86VQJ	multiple-copy	2-oxoglutarate(2OG)andFe(II)-dependentoxygenasesupe
Aradu.E43JR	Araip.9JP8R	multiple-copy	2-oxoglutarate(2OG)andFe(II)-dependentoxygenasesupe
Aradu.9MZ2X	Araip.EDE9C	multiple-copy	2-oxoglutarate(2OG)andFe(II)-dependentoxygenasesupe

Aradu.2H1GD	Araip.FBK18	multiple-copy	2-oxoglutarate(2OG)andFe(II)-dependentoxygenasesupe
Aradu.W6H6W	Araip.GP5B8	multiple-copy	2-oxoglutarate(2OG)andFe(II)-dependentoxygenasesupe
Aradu.ZN3FN	Araip.I128H	multiple-copy	2-oxoglutarate(2OG)andFe(II)-dependentoxygenasesupe
Aradu.ZZ3JW	Araip.LWJ5V	multiple-copy	2-oxoglutarate(2OG)andFe(II)-dependentoxygenasesupe
Aradu.P4XQA	Araip.N8G27	multiple-copy	2-oxoglutarate(2OG)andFe(II)-dependentoxygenasesupe
Aradu.TV3WI	Araip.R07X0	multiple-copy	2-oxoglutarate(2OG)andFe(II)-dependentoxygenasesupe
Aradu.LA0XT	Araip.TI5D7	multiple-copy	2-oxoglutarate(2OG)andFe(II)-dependentoxygenasesupe
Aradu.G0Z55	Araip.VA8G3	multiple-copy	2-oxoglutarate/Fe(II)-dependentdioxygenase-like[Glycine
Aradu.IEX64	Araip.X2IEW	multiple-copy	2-oxoglutarate(2OG)andFe(II)-dependentoxygenasesupe
Aradu.HE28Y	Araip.4F7TS	multiple-copy	2-oxoglutarate/Fe(II)-dependentdioxygenase[Glycinemax
Aradu.BF125	Araip.69K1T	multiple-copy	2-oxoglutarate(2OG)andFe(II)-dependentoxygenasesupe
Aradu.LJJ9P	Araip.6N7QF	multiple-copy	2-oxoglutarate/Fe(II)-dependentdioxygenase-like[Glycine
Aradu.5QH0B	Araip.78FT4	multiple-copy	2-oxoglutarate(2OG)andFe(II)-dependentoxygenasesupe
Aradu.SH6P7	Araip.8GH2C	multiple-copy	2-oxoglutarate/Fe(II)-dependentdioxygenase-like[Glycine
Aradu.H8Z7K	Araip.9GU4E	multiple-copy	2-oxoglutarate/Fe(II)-dependentdioxygenase-like[Glycine
Aradu.ID8H5	Araip.IDR0H	multiple-copy	2-oxoglutarate/Fe(II)-dependentdioxygenase-like[Glycine
Aradu.1UI8P	Araip.IJN8L	multiple-copy	2-oxoglutarate/Fe(II)-dependentdioxygenase-like[Glycine
Aradu.T6XQA	Araip.Q2MIL	multiple-copy	2-oxoglutarate/Fe(II)-dependentdioxygenase-like[Glycine
Aradu.RB3TY	Araip.ZHD9F	multiple-copy	2-oxoglutaratedehydrogenase,E1component
Aradu.INH3Y	Araip.3JN5Z	multiple-copy	2-oxoisovaleratedehydrogenasesubunitalpha
Aradu.S5T1J	Araip.RM73I	multiple-copy	2-phosphoglyceratekinase
Aradu.88CYL	Araip.DM3HR	multiple-copy	2-phosphoglycolatephosphatase1
Aradu.ZEB3I	Araip.P77AZ	multiple-copy	30SribosomalproteinS15
Aradu.15W25	Araip.I5RQQ	multiple-copy	30SribosomalproteinS18n=2Tax=OscillatoriophycideaeR
Aradu.40AX1	Araip.KF79G	multiple-copy	30SribosomalproteinS2
Aradu.II4Y3	Araip.0B3H2	multiple-copy	30SribosomalproteinS31,chloroplastic-like[Glycinemax]
Aradu.KV87W	Araip.DB9NP	multiple-copy	30SribosomalproteinS31,chloroplastic-like[Glycinemax]
Aradu.J3IKA	Araip.26IG1	multiple-copy	30SribosomalS16-likeprotein
Aradu.2K88G	Araip.R3Y0S	multiple-copy	30SribosomalS16-likeprotein
Aradu.IW9VR	Araip.781N3	multiple-copy	3-betahydroxysteroiddehydrogenasen=1Tax=Calothrixsp
Aradu.I9RLC	Araip.RVT5N	multiple-copy	3-dehydroquinatesynthase,putative
Aradu.XM9I6	Araip.BM7DX	multiple-copy	3-hydroxy-3-methylglutaryl-coenzymeAreductase-likeprot
Aradu.MSQ8X	Araip.CGA2T	multiple-copy	3-hydroxyacyl-[acyl-carrier-protein]dehydrataseFabZn=2T
Aradu.V8HSY	Araip.LP8AE	multiple-copy	3-isopropylmalatedehydratase,smallsubunit
Aradu.D6DD4	Araip.B5KUQ	multiple-copy	3-ketoacyl-CoAsynthase10
Aradu.3R84Q	Araip.NT8KP	multiple-copy	3-ketoacyl-CoAsynthase10
Aradu.0Y40Q	Araip.B7MLT	multiple-copy	3-ketoacyl-CoAsynthase11
Aradu.J6P55	Araip.ML7TB	multiple-copy	3-ketoacyl-CoAsynthase11
Aradu.MK4GU	Araip.ND08G	multiple-copy	3-ketoacyl-CoAsynthase12
Aradu.40JMZ	Araip.4QE8A	multiple-copy	3-ketoacyl-CoAsynthase19
Aradu.KD1XS	Araip.A8D0C	multiple-copy	3-ketoacyl-CoAsynthase2
Aradu.RBU21	Araip.YN0DS	multiple-copy	3-ketoacyl-CoAsynthase4
Aradu.J2YIY	Araip.A561Y	multiple-copy	3-ketoacyl-CoAsynthase6
Aradu.EYV3C	Araip.B7VJF	multiple-copy	3-oxoacyl-[acyl-carrier-protein]synthaseIn=7Tax=rosidsR

Aradu.AV6RW	Araip.E51S4	multiple-copy	3-oxoacyl-[acyl-carrier-protein]synthasen=4Tax=Phaseol
Aradu.E515S	Araip.0BH7D	multiple-copy	40SribosomalproteinS11-like[Glycinemax]
Aradu.79MUY	Araip.G0FLH	multiple-copy	40SribosomalproteinS13[Glycinemax]
Aradu.6V9D4	Araip.K74WI	multiple-copy	40SribosomalproteinS14-like[Glycinemax]
Aradu.MI9SZ	Araip.K8FHN	multiple-copy	40SribosomalproteinS14-like[Glycinemax]
Aradu.UX579	Araip.AP06R	multiple-copy	40SribosomalproteinS15-4
Aradu.LWF57	Araip.D1TXC	multiple-copy	40SribosomalproteinS15-4
Aradu.69TMW	Araip.K6C7R	multiple-copy	40SribosomalproteinS15-4
Aradu.X7VPA	Araip.4V5MM	multiple-copy	40SribosomalproteinS19-1
Aradu.AQ9N2	Araip.VB46U	multiple-copy	40SribosomalproteinS19-1
Aradu.DG44N	Araip.21DP5	multiple-copy	40SribosomalproteinS20-2
Aradu.7N01K	Araip.M35KP	multiple-copy	40SribosomalproteinS24-2
Aradu.H5NQ6	Araip.0U2QD	multiple-copy	40SribosomalproteinS26-2[Glycinemax]
Aradu.GS270	Araip.18N2T	multiple-copy	40SribosomalproteinS3a-1
Aradu.S59LW	Araip.GN6XE	multiple-copy	40SribosomalproteinS3a-1
Aradu.NND2F	Araip.81PGB	multiple-copy	40SribosomalproteinS8-like[Glycinemax]
Aradu.BHV10	Araip.ZDS2I	multiple-copy	40SribosomalS10-likeprotein
Aradu.CV6FA	Araip.F787E	multiple-copy	4-coumarate:CoAligase2
Aradu.99AQ5	Araip.G3TW3	multiple-copy	4-coumarate:CoAligase2
Aradu.WYR1J	Araip.C95X7	multiple-copy	4-coumarate:CoAligase3
Aradu.CMI46	Araip.VD7Y0	multiple-copy	4-hydroxy-tetrahydrodipicolinatereductase3,chloroplastic-
Aradu.P3RU0	Araip.V4W9N	multiple-copy	50SribosomalL30-likeprotein
Aradu.K7CLQ	Araip.2R8F4	multiple-copy	50SribosomalproteinL1,chloroplastic-likeisoformX1[Glycir
Aradu.P8YMU	Araip.09GVV	multiple-copy	50SribosomalproteinL14
Aradu.4TU5K	Araip.Q2MNW	multiple-copy	50SribosomalproteinL20
Aradu.Q5NNZ	Araip.UT0YW	multiple-copy	50SribosomalproteinL21,relatedprotein
Aradu.X5BAW	Araip.V2QG1	multiple-copy	50SribosomalproteinL21,relatedprotein
Aradu.270YY	Araip.L7AM8	multiple-copy	50SribosomalproteinL35
Aradu.Y0AR8	Araip.F8VVQ	multiple-copy	5-hydroxyisouratehydrolase
Aradu.DCZ6C	Araip.IZW7Y	multiple-copy	60SacidicribosomalproteinP0-1
Aradu.5ME2Z	Araip.W8189	multiple-copy	60SribosomalL12-likeprotein
Aradu.TC6LS	Araip.5P4LE	multiple-copy	60SribosomalL28-likeprotein
Aradu.Y4PLR	Araip.WA7Y0	multiple-copy	60SribosomalL28-likeprotein
Aradu.7GP5A	Araip.61SU1	multiple-copy	60SribosomalL35-likeprotein
Aradu.JM0WC	Araip.L6Z75	multiple-copy	60SribosomalL35-likeprotein
Aradu.Z3R4T	Araip.R5PNL	multiple-copy	60SribosomalproteinL11-like[Glycinemax]
Aradu.AC0Z7	Araip.5S1QP	multiple-copy	60SribosomalproteinL15-1-like[Glycinemax]
Aradu.NRN2N	Araip.U1KHK	multiple-copy	60SribosomalproteinL18-3
Aradu.FZ0BW	Araip.01H2N	multiple-copy	60SribosomalproteinL18A-1
Aradu.UHQ4E	Araip.6QE8H	multiple-copy	60SribosomalproteinL18A-1
Aradu.IJM7H	Araip.L6VN3	multiple-copy	60SribosomalproteinL18A-1
Aradu.Y92M0	Araip.S5ADV	multiple-copy	60SribosomalproteinL18A-1
Aradu.VY0ZL	Araip.2UA97	multiple-copy	60SribosomalproteinL23a-2
Aradu.Y1K8W	Araip.CI6Y7	multiple-copy	60SribosomalproteinL23a-2

Aradu.Q7MTE	Araip.47I29	multiple-copy	60SribosomalproteinL24-2
Aradu.5M2IF	Araip.MAE3X	multiple-copy	60SribosomalproteinL26-1-like[Glycinemax]
Aradu.Q0Z3H	Araip.AU5HS	multiple-copy	60SribosomalproteinL30-like[Glycinemax]
Aradu.P3N99	Araip.1L1V5	multiple-copy	60SribosomalproteinL32-1
Aradu.T5NFD	Araip.564FB	multiple-copy	60SribosomalproteinL36
Aradu.JXB2I	Araip.LC3UN	multiple-copy	60SribosomalproteinL7a-like[Glycinemax]
Aradu.H2I13	Araip.FK804	multiple-copy	7SKsnRNAmethylphosphatecappingenzymen=8Tax=Bor
Aradu.PRR6C	Araip.RQ6E9	multiple-copy	aarFdomain-containingproteinkinaseAt1g79600,chloropla
Aradu.NA9YP	Araip.RW9UY	multiple-copy	ABCtransporterfamilyprotein
Aradu.XJM2L	Araip.TW2ID	multiple-copy	ABCtransporterfamilyprotein
Aradu.EZU9P	Araip.LA28K	multiple-copy	ABCtransporterfamilyprotein(ATP-bindingcomponent)
Aradu.524PX	Araip.T8U1E	multiple-copy	ABCtransporterfamilyprotein(ATP-bindingcomponent)
Aradu.B80WC	Araip.WS6S0	multiple-copy	ABCtransporterfamilyprotein(ATP-bindingcomponent)
Aradu.VU792	Araip.YJ40C	multiple-copy	ABCtransporterfamilyprotein(ATP-bindingcomponent)
Aradu.2597P	Araip.Y64TL	multiple-copy	ABCtransporterGfamilymember22-likeisoformX2[Glycine
Aradu.N4IR4	Araip.TL94E	multiple-copy	ABIL2-likeisoformX3[Glycinemax]
Aradu.3X53E	Araip.5VX8G	multiple-copy	abscisicacidreceptor
Aradu.XBR9P	Araip.CT5HY	multiple-copy	abscisicacidreceptor
Aradu.PU6SR	Araip.SEZ0Y	multiple-copy	abscisicacidreceptor
Aradu.A24RB	Araip.T656T	multiple-copy	abscisicacidreceptor
Aradu.X0F3K	Araip.X3UTX	multiple-copy	abscisicacidresponsiveelement-bindingfactor1
Aradu.GHK20	Araip.ZAK9V	multiple-copy	acetyl-CoAcarboxylase1
Aradu.R4JGP	Araip.04GN0	multiple-copy	Acidphosphatase/vanadium-dependenthaloperoxidase-re
Aradu.SH3UZ	Araip.U8V9W	multiple-copy	Acidphosphatase/vanadium-dependenthaloperoxidase-re
Aradu.CSN5D	Araip.T1FEI	multiple-copy	acidphosphatase
Aradu.1JK2E	Araip.G9K0U	multiple-copy	ACTdomain-containingprotein
Aradu.WJZ15	Araip.PKN8E	multiple-copy	ACTdomain-containingprotein
Aradu.IY93L	Araip.21TG8	multiple-copy	ACTdomain-containingsmallsubunitofacetolactatesynthas
Aradu.9P432	Araip.04X9B	multiple-copy	ACTdomainrepeat1
Aradu.FEU0W	Araip.S2ICC	multiple-copy	ACTdomainrepeat3
Aradu.JMD12	Araip.U29VB	multiple-copy	ACTdomainrepeat8
Aradu.CD8IH	Araip.MC99C	multiple-copy	actin-bindingFH2(formin2)familyprotein
Aradu.Y4AJ0	Araip.DI99W	multiple-copy	Actin-bindingFH2(ForminHomology)protein
Aradu.MJ83N	Araip.REN75	multiple-copy	Actin-bindingFH2(ForminHomology)protein
Aradu.X89BI	Araip.5I8PV	multiple-copy	Actincross-linkingprotein
Aradu.Z4QQP	Araip.RAM8P	multiple-copy	Actincross-linkingprotein
Aradu.IDN32	Araip.DFY7G	multiple-copy	actindepolymerizingfactor5
Aradu.GHE18	Araip.Z2XC8	multiple-copy	actindepolymerizingfactor6
Aradu.C1ZP7	Araip.BW6MW	multiple-copy	actin-relatedprotein2/3complexsubunit4
Aradu.CL5YM	Araip.DW3Y4	multiple-copy	actin-relatedprotein4
Aradu.944BF	Araip.175SR	multiple-copy	actin-relatedprotein7
Aradu.92FHF	Araip.K5C2Q	multiple-copy	actin-relatedprotein8
Aradu.N7DXE	Araip.J0PEZ	multiple-copy	Actin-relatedproteinArp2/3complexsubunitARPC3n=2Tax
Aradu.XM2MR	Araip.RI4NB	multiple-copy	Acyl-[acyl-carrier-protein]desaturasen=2Tax=SolanumRe

Aradu.0EA4R	Araip.4509F	multiple-copy	Acyl-ACPthioesterase
Aradu.B3QR0	Araip.S1TFK	multiple-copy	Acyl-ACPthioesterase
Aradu.ZG6C0	Araip.SBA9Y	multiple-copy	Acyl-ACPthioesterase
Aradu.K42LN	Araip.BQ2U7	multiple-copy	acylcarrierprotein1
Aradu.B0TIL	Araip.M9QUH	multiple-copy	acylcarrierprotein1
Aradu.200CK	Araip.AQ14G	multiple-copy	acylcarrierprotein5
Aradu.1J1TW	Araip.GZL5I	multiple-copy	acyl-CoA-bindingdomain3
Aradu.8QB2V	Araip.J4RH5	multiple-copy	acyl-CoA-bindingdomain-containingprotein4-likeisoformX
Aradu.FN4CD	Araip.KU3NI	multiple-copy	acyl-CoA-bindingdomain-containingprotein4-likeisoformX
Aradu.M4DVD	Araip.2S7LP	multiple-copy	acyl-CoA-bindingdomain-containingprotein4-likeisoformX
Aradu.U5ZHH	Araip.A69R7	multiple-copy	Acyl-CoAN-acyltransferases(NAT)superfamilyprotein
Aradu.TH5KX	Araip.TUQ7N	multiple-copy	Acyl-CoAN-acyltransferases(NAT)superfamilyprotein
Aradu.6MW2E	Araip.7QP65	multiple-copy	acyl-CoAoxidase2
Aradu.URD4R	Araip.SV2QM	multiple-copy	acyl-CoAoxidase3
Aradu.JT1JK	Araip.22PIW	multiple-copy	acyl-CoAsynthetase5
Aradu.R403Z	Araip.2I7IS	multiple-copy	acyl-CoAsynthetase5
Aradu.P9PIK	Araip.5R5N0	multiple-copy	acyl-CoAsynthetase5
Aradu.JLT7Z	Araip.E86GN	multiple-copy	acyl-CoAsynthetase5
Aradu.V73WK	Araip.HV7J0	multiple-copy	acyl-CoAsynthetase5
Aradu.W7CRW	Araip.J8QA5	multiple-copy	acyl-CoAsynthetase5
Aradu.D1CUJ	Araip.UK85B	multiple-copy	acyl-CoAsynthetase5
Aradu.8C9LT	Araip.XF76V	multiple-copy	acyl-CoAsynthetase5
Aradu.TQ146	Araip.TK5D4	multiple-copy	Acyl-CoAthioesterasefamilyprotein
Aradu.QGW0X	Araip.YL8HA	multiple-copy	Acyl-CoAthioesterasefamilyprotein
Aradu.H5I2N	Araip.60749	multiple-copy	acyl-coenzymeAthioesterase-likeprotein
Aradu.0G2ML	Araip.Q98DA	multiple-copy	acyl-coenzymeAthioesterase-likeprotein
Aradu.B4Z82	Araip.GZ59D	multiple-copy	acylthioesterase-likeprotein
Aradu.8H45I	Araip.ZXN9C	multiple-copy	Adaptinear-bindingcoat-associatedprotein1NECAP-1
Aradu.E4NC1	Araip.167MA	multiple-copy	Adeninenucleotidealphahydrolases-likesuperfamilyprotei
Aradu.W23YF	Araip.1H048	multiple-copy	Adeninenucleotidealphahydrolases-likesuperfamilyprotei
Aradu.L9J3S	Araip.30SUW	multiple-copy	Adeninenucleotidealphahydrolases-likesuperfamilyprotei
Aradu.BLP4A	Araip.4U9WI	multiple-copy	Adeninenucleotidealphahydrolases-likesuperfamilyprotei
Aradu.R082J	Araip.CL35K	multiple-copy	Adeninenucleotidealphahydrolases-likesuperfamilyprotei
Aradu.S13AZ	Araip.GV2U7	multiple-copy	Adeninenucleotidealphahydrolases-likesuperfamilyprotei
Aradu.Q6DD2	Araip.S7E86	multiple-copy	Adeninenucleotidealphahydrolases-likesuperfamilyprotei
Aradu.J502L	Araip.T4LH3	multiple-copy	Adeninenucleotidealphahydrolases-likesuperfamilyprotei
Aradu.UM1Y9	Araip.U10LF	multiple-copy	Adeninenucleotidealphahydrolases-likesuperfamilyprotei
Aradu.VF877	Araip.HD8AG	multiple-copy	adeninephosphoribosyltransferase5
Aradu.IXF4G	Araip.U880P	multiple-copy	adeninephosphoribosyltransferase5
Aradu.HK079	Araip.ZN6UI	multiple-copy	adenylatecyclase
Aradu.B03MY	Araip.6P9G9	multiple-copy	adenylatekinasefamilyprotein
Aradu.M35ZU	Araip.KLU9X	multiple-copy	adenylatekinasefamilyprotein
Aradu.HU94F	Araip.SP2SD	multiple-copy	Adenylatekinasefamilyprotein
Aradu.JKK33	Araip.Y9WQJ	multiple-copy	adenylatekinasefamilyprotein

Aradu.EB4A5	Araip.EQ8I1	multiple-copy	adiponectinreceptorprotein2-likeisoformX3[Glycinemax]
Aradu.1DJ56	Araip.Q6AH4	multiple-copy	adiponectinreceptorprotein1-like[Glycinemax]
Aradu.H9GMI	Araip.EW35C	multiple-copy	adipose-regulatoryprotein(Seipin)
Aradu.0N1XE	Araip.H8TGQ	multiple-copy	ADP/ATPcarrier3
Aradu.UEG62	Araip.JAJ4W	multiple-copy	ADP-ribosylationfactor1
Aradu.1Q100	Araip.QR5JJ	multiple-copy	ADP-ribosylationfactor1
Aradu.H102I	Araip.T6H8P	multiple-copy	ADP-ribosylationfactor1
Aradu.SMR94	Araip.27R71	multiple-copy	ADP-ribosylationfactor3
Aradu.9DJ4L	Araip.2KU4C	multiple-copy	ADP-ribosylationfactor3
Aradu.DF4RH	Araip.59LK4	multiple-copy	ADP-ribosylationfactorGTPase-activatingproteinAGD10
Aradu.XA005	Araip.7MJ2M	multiple-copy	ADP-ribosylationfactorGTPase-activatingproteinAGD10
Aradu.R23DU	Araip.C57WH	multiple-copy	ADP-ribosylationfactorGTPase-activatingproteinAGD10
Aradu.SP6EC	Araip.1H6L7	multiple-copy	ADP-ribosylationfactorGTPase-activatingproteinAGD11-li
Aradu.UV8JW	Araip.IIT7G	multiple-copy	ADP-ribosylationfactorGTPase-activatingproteinAGD11-li
Aradu.X8MF9	Araip.4I4L7	multiple-copy	ADP-ribosylationfactorGTPase-activatingproteinAGD12-li
Aradu.9E2KY	Araip.HPE6X	multiple-copy	ADP-ribosylationfactorGTPase-activatingproteinAGD14-li
Aradu.WU1XZ	Araip.8YJ09	multiple-copy	ADP-ribosylationfactorGTPase-activatingproteinAGD3-lik
Aradu.W1519	Araip.9ER14	multiple-copy	ADP-ribosylationfactorGTPase-activatingproteinAGD3-lik
Aradu.Y1Z0R	Araip.Y3AYB	multiple-copy	AFG1-likeATPasefamilyprotein
Aradu.4K5XY	Araip.WHJ1H	multiple-copy	alanineaminotransferase2
Aradu.X5TRC	Araip.B9RM0	multiple-copy	AlbaDNA/RNA-bindingprotein
Aradu.707Z5	Araip.F963I	multiple-copy	AlbaDNA/RNA-bindingprotein
Aradu.R9T1V	Araip.Z4N27	multiple-copy	AlbaDNA/RNA-bindingprotein
Aradu.7YM1I	Araip.BYV00	multiple-copy	alcoholdehydrogenase1
Aradu.4ZD6C	Araip.C9VD3	multiple-copy	alcoholdehydrogenase1
Aradu.VAN9Z	Araip.GJ7LV	multiple-copy	alcoholdehydrogenase1
Aradu.6C4RF	Araip.NGU0E	multiple-copy	alcoholdehydrogenase1
Aradu.N39PG	Araip.QT4ZA	multiple-copy	alcoholdehydrogenase1
Aradu.288JJ	Araip.3485Y	multiple-copy	aldehydedehydrogenasefamily2memberC4-like[Glycinem
Aradu.I79F7	Araip.4V6B3	multiple-copy	aldehydedehydrogenasefamily2memberC4-like[Glycinem
Aradu.F2VIG	Araip.798H5	multiple-copy	aldehydedehydrogenasefamily2memberC4-like[Glycinem
Aradu.3DW3S	Araip.9SP7M	multiple-copy	aldehydedehydrogenasefamily2memberC4-like[Glycinem
Aradu.DAT06	Araip.B5YUT	multiple-copy	aldehydedehydrogenasefamily2memberC4-like[Glycinem
Aradu.83253	Araip.Y8KWK	multiple-copy	aldehydedehydrogenasefamily2memberC4-like[Glycinem
Aradu.ZIF6R	Araip.7EF18	multiple-copy	aldehydedehydrogenasefamily3memberH1-like[Glycinem
Aradu.VKB5P	Araip.8K7MC	multiple-copy	aldo/ketoreductasefamilyoxidoreductase
Aradu.U8ZNV	Araip.94SGJ	multiple-copy	aldo/ketoreductasefamilyoxidoreductase
Aradu.3SA2N	Araip.A28ZZ	multiple-copy	aldo/ketoreductasefamilyoxidoreductase
Aradu.I4L9J	Araip.DF82N	multiple-copy	aldo/ketoreductasefamilyoxidoreductase
Aradu.GWN14	Araip.F49MZ	multiple-copy	aldo/ketoreductasefamilyoxidoreductase
Aradu.83KXV	Araip.JE2NP	multiple-copy	aldo/ketoreductasefamilyoxidoreductase
Aradu.7065G	Araip.MKZ27	multiple-copy	aldo/ketoreductasefamilyoxidoreductase
Aradu.V45Z7	Araip.PP9AX	multiple-copy	aldo/ketoreductasefamilyoxidoreductase
Aradu.U53UJ	Araip.PR57R	multiple-copy	aldo/ketoreductasefamilyoxidoreductase

Aradu.9YK5E	Araip.MU9UP	multiple-copy	aldose1-epimerasefamilyprotein
Aradu.GTJ2E	Araip.AL2JW	multiple-copy	aldose1-epimerase-like[Glycinemax]
Aradu.200KX	Araip.EDC64	multiple-copy	alkaline/neutralinvertase
Aradu.HX0A2	Araip.JU5GB	multiple-copy	alkaline/neutralinvertase
Aradu.XI961	Araip.SEY9F	multiple-copy	alkaline/neutralinvertase
Aradu.M29FV	Araip.VSW8G	multiple-copy	alkaline/neutralinvertase
Aradu.94T7Q	Araip.7944J	multiple-copy	Alkalinephytoceramidase(aPHC)
Aradu.PFQ1E	Araip.H3DRL	multiple-copy	AlkylatedDNArepairproteinalkB-likeprotein8n=2Tax=Tritic
Aradu.65WG6	Araip.V48DY	multiple-copy	alleneoxidecyclase4
Aradu.8I12T	Araip.EZ4HJ	multiple-copy	alleneoxidesynthase
Aradu.V9EPJ	Araip.2HK29	multiple-copy	alpha/betafoldhydrolase
Aradu.Q27ZD	Araip.987R9	multiple-copy	alpha/betafoldhydrolase
Aradu.U1CK3	Araip.C26DA	multiple-copy	alpha/betafoldhydrolase
Aradu.69820	Araip.L5V11	multiple-copy	alpha/betafoldhydrolase
Aradu.M1DIQ	Araip.NMT69	multiple-copy	alpha/betafoldhydrolase
Aradu.0H9WK	Araip.VGR7G	multiple-copy	alpha/betafoldhydrolase
Aradu.F9BJN	Araip.Y5DXY	multiple-copy	alpha/betafoldhydrolase
Aradu.LBT1F	Araip.3WF3M	multiple-copy	alpha/betahydrolasefamilyprotein
Aradu.7C8N3	Araip.J4IZA	multiple-copy	alpha/betahydrolasefamilyprotein
Aradu.X3XEZ	Araip.FG09W	multiple-copy	Alpha/betahydrolaserelatedprotein
Aradu.D4CJV	Araip.0Z0CW	multiple-copy	alpha/beta-Hydrolasessuperfamilyprotein
Aradu.SL9AV	Araip.14067	multiple-copy	alpha/beta-Hydrolasessuperfamilyprotein
Aradu.J3FIC	Araip.37TH3	multiple-copy	alpha/beta-Hydrolasessuperfamilyprotein
Aradu.1A3FB	Araip.3YS8U	multiple-copy	alpha/beta-Hydrolasessuperfamilyprotein
Aradu.5R9LY	Araip.4E6BS	multiple-copy	alpha/beta-Hydrolasessuperfamilyprotein
Aradu.492LT	Araip.6MZ14	multiple-copy	alpha/beta-Hydrolasessuperfamilyprotein
Aradu.FSK9A	Araip.B9NXS	multiple-copy	alpha/beta-Hydrolasessuperfamilyprotein
Aradu.YH03P	Araip.C3SNG	multiple-copy	alpha/beta-Hydrolasessuperfamilyprotein
Aradu.8Z4L7	Araip.C4EXT	multiple-copy	alpha/beta-Hydrolasessuperfamilyprotein
Aradu.87LE7	Araip.EN4UY	multiple-copy	alpha/beta-Hydrolasessuperfamilyprotein
Aradu.BC6IG	Araip.I1K2T	multiple-copy	alpha/beta-Hydrolasessuperfamilyprotein
Aradu.89S1X	Araip.J2LD8	multiple-copy	alpha/beta-Hydrolasessuperfamilyprotein
Aradu.C3ARN	Araip.P15N1	multiple-copy	alpha/beta-Hydrolasessuperfamilyprotein
Aradu.R0Z7V	Araip.S9MFB	multiple-copy	alpha/beta-Hydrolasessuperfamilyprotein
Aradu.7P7FQ	Araip.SV5JG	multiple-copy	alpha/beta-Hydrolasessuperfamilyprotein
Aradu.1195M	Araip.U26LB	multiple-copy	alpha/beta-Hydrolasessuperfamilyprotein
Aradu.BV2ZB	Araip.W61UJ	multiple-copy	alpha/beta-Hydrolasessuperfamilyprotein
Aradu.5L8S5	Araip.W6S6X	multiple-copy	alpha/beta-Hydrolasessuperfamilyprotein
Aradu.T0LS0	Araip.XFW7H	multiple-copy	alpha/beta-Hydrolasessuperfamilyprotein
Aradu.4W183	Araip.57R3H	multiple-copy	alpha/beta-hydrolasesuperfamilyprotein
Aradu.R3U8P	Araip.A4TEB	multiple-copy	alpha/beta-hydrolasesuperfamilyprotein
Aradu.V0EF8	Araip.FHB4T	multiple-copy	alpha/beta-hydrolasesuperfamilyprotein
Aradu.M4ZYN	Araip.TSB8A	multiple-copy	alpha/betafoldhydrolase
Aradu.PZ2KX	Araip.R8FCB	multiple-copy	alpha/betasuperfamilyhydrolase

Aradu.AK4LW	Araip.MQI8S	multiple-copy	alpha-1,4-glucan-proteinsynthase[UDP-forming]-likeprote
Aradu.ERN5M	Araip.PI1LJ	multiple-copy	alpha-amylase-like3
Aradu.88UWV	Araip.RL9T9	multiple-copy	alphacarbonicanhydrase7
Aradu.NZ3TX	Araip.5WT0G	multiple-copy	alpha-L-arabinofuranosidase2
Aradu.CN01H	Araip.U4XHQ	multiple-copy	alpha-mannosidase2x-likeprotein
Aradu.UK4BE	Araip.7L4UM	multiple-copy	Alternativeoxidasefamilyprotein
Aradu.VE1AV	Araip.KA7I0	multiple-copy	AluminiuminducedproteinwithYGLandLRDRmotifs
Aradu.04RDY	Araip.ZX8HU	multiple-copy	AluminiuminducedproteinwithYGLandLRDRmotifs
Aradu.JJG2Q	Araip.J2KA8	multiple-copy	aluminum-activated,malatetransporter12
Aradu.EJA5A	Araip.I5C3J	multiple-copy	aluminum-activatedmalatetransporter1
Aradu.XDP02	Araip.K3DD8	multiple-copy	aluminum-activatedmalatetransporter1
Aradu.T52PW	Araip.A84G4	multiple-copy	aluminum-activatedmalatetransporter9
Aradu.Z84GQ	Araip.X0SWX	multiple-copy	amidase1-likeisoformX1[Glycinemax]
Aradu.Q0H7N	Araip.43WXS	multiple-copy	Amidasefamilyprotein
Aradu.G7BFP	Araip.CT78Y	multiple-copy	Amidasefamilyprotein
Aradu.R64XC	Araip.ZJJ3R	multiple-copy	Aminoacidpermeasefamilyprotein
Aradu.2V3YU	Araip.X2AQN	multiple-copy	aminoacyl-tRNAsynthetase
Aradu.KH25W	Araip.GL6UI	multiple-copy	ammoniumtransporter1
Aradu.VC6I6	Araip.28BYA	multiple-copy	ammoniumtransporter2
Aradu.M6DL9	Araip.N863A	multiple-copy	ammoniumtransporter2
Aradu.DZ2GN	Araip.IHS0X	multiple-copy	AMPdeaminase-like[Glycinemax]
Aradu.4XQ5D	Araip.TIN6U	multiple-copy	anaphase-promotingcomplexsubunit5
Aradu.NI37K	Araip.GM91W	multiple-copy	anaphase-promotingcomplexsubunit8
Aradu.ANS8T	Araip.PR0RR	multiple-copy	Anionexchangefamilyproteinn=2Tax=Populustrichocarpal
Aradu.R3QFW	Araip.02IFK	multiple-copy	Anionexchangerfamilyproteinn=1Tax=Medicagotruncatula
Aradu.I5BER	Araip.UNL4G	multiple-copy	Anionexchangerfamilyproteinn=1Tax=Medicagotruncatula
Aradu.H83MI	Araip.DY9AL	multiple-copy	ankyrinrepeat-containing2B
Aradu.7399Q	Araip.73ST3	multiple-copy	ankyrinrepeatfamilyprotein/AAA-typeATPasefamilyprotein
Aradu.2DM5J	Araip.CJ9D7	multiple-copy	ankyrinrepeat-containingprotein[Glycinemax]
Aradu.J08SD	Araip.K4G1H	multiple-copy	ankyrinrepeat-containingprotein[Glycinemax]
Aradu.63D6A	Araip.LWS1N	multiple-copy	ankyrinrepeat-containingprotein[Glycinemax]
Aradu.E9P6L	Araip.U15G0	multiple-copy	ankyrinrepeat-containingprotein[Glycinemax]
Aradu.PDJ58	Araip.Q76PE	multiple-copy	ankyrinrepeat-containingproteinAt3g12360-like[Glycinem
Aradu.BMJ8R	Araip.Z76YZ	multiple-copy	ankyrinrepeat-containingproteinAt3g12360-like[Glycinem
Aradu.NM8U4	Araip.5CF12	multiple-copy	ankyrinrepeat-containingproteinAt5g02620-likeisoformX3
Aradu.N8T7S	Araip.H2NMQ	multiple-copy	ankyrinrepeat-containingproteinAt5g02620-likeisoformX3
Aradu.PT6UP	Araip.EQQ7V	multiple-copy	ankyrinrepeat-containingproteinAt5g02620-likeisoformX2
Aradu.L49GZ	Araip.MJR9D	multiple-copy	ankyrinrepeat-containingproteinAt5g02620-likeisoformX2
Aradu.LBD98	Araip.Z968E	multiple-copy	ankyrinrepeat-containingproteinAt5g02620-likeisoformX3
Aradu.14WTD	Araip.2X2JD	multiple-copy	Ankyrinrepeatfamilyprotein
Aradu.6AZ0N	Araip.3BD5Q	multiple-copy	Ankyrinrepeatfamilyprotein
Aradu.V0UIC	Araip.3GT2G	multiple-copy	Ankyrinrepeatfamilyprotein
Aradu.UF8GX	Araip.3L11M	multiple-copy	Ankyrinrepeatfamilyprotein
Aradu.UJD9G	Araip.417P5	multiple-copy	Ankyrinrepeatfamilyprotein

Aradu.58Q87	Araip.91WQY	multiple-copy	ankyrinrepeat-containingprotein[Glycinemax]
Aradu.U7IHD	Araip.9E124	multiple-copy	Ankyrinrepeatfamilyprotein
Aradu.XRX70	Araip.A8INI	multiple-copy	Ankyrinrepeatfamilyprotein
Aradu.KY6W5	Araip.DBR9L	multiple-copy	Ankyrinrepeatfamilyprotein
Aradu.JSQ7J	Araip.FEQ3S	multiple-copy	Ankyrinrepeatfamilyprotein
Aradu.P9CN5	Araip.FL59H	multiple-copy	Ankyrinrepeatfamilyprotein
Aradu.C87QT	Araip.GDW3P	multiple-copy	ankyrinrepeatfamilyprotein
Aradu.209EV	Araip.Q76UC	multiple-copy	Ankyrinrepeatfamilyprotein
Aradu.A8RS9	Araip.QW0IT	multiple-copy	Ankyrinrepeatfamilyprotein
Aradu.1D3EG	Araip.RN11M	multiple-copy	Ankyrinrepeatfamilyprotein
Aradu.MVM9Y	Araip.RS9FT	multiple-copy	Ankyrinrepeatfamilyprotein
Aradu.YY4G2	Araip.W2Y6S	multiple-copy	Ankyrinrepeatfamilyprotein
Aradu.S16C5	Araip.Y8EDR	multiple-copy	annexin3
Aradu.WYZ5E	Araip.0MR1X	multiple-copy	annexin5
Aradu.3L5NK	Araip.HD6QL	multiple-copy	annexin5
Aradu.IZQ3Z	Araip.Z0Q6Q	multiple-copy	annexin8
Aradu.IR1JZ	Araip.0U07W	multiple-copy	anthranilatesynthase2
Aradu.ES65V	Araip.8074I	multiple-copy	anthranilatesynthase2
Aradu.XKU5K	Araip.494RG	multiple-copy	AP-1complexsubunitsigma-likeprotein
Aradu.109RJ	Araip.A97NN	multiple-copy	AP-1complexsubunitsigma-likeprotein
Aradu.0KB9D	Araip.1VN1Z	multiple-copy	AP2-likeethylene-responsivetranscriptionfactorAIL6-like[G
Aradu.S9CWT	Araip.60YE7	multiple-copy	AP2-likeethylene-responsivetranscriptionfactorANT-like[G
Aradu.VJK1R	Araip.AI81V	multiple-copy	AP2-likeethylene-responsivetranscriptionfactorANT-like[G
Aradu.1235N	Araip.46LQV	multiple-copy	apyrase7-likeisoformX1[Glycinemax]
Aradu.ERR96	Araip.UB1TB	multiple-copy	aquaporinPIP2-5-like[Glycinemax]
Aradu.M4EHD	Araip.SH4GT	multiple-copy	Arabidopsisphospholipase-likeprotein(PEARLI4)family
Aradu.LAK4N	Araip.0CF6W	multiple-copy	Arginine/serine-richsplicingfactor,putativen=5Tax=rosidsR
Aradu.L9SJ9	Araip.60QH4	multiple-copy	arginine/serine-rich-splicingfactorRSP31-likeisoformX2[G
Aradu.SNT27	Araip.3U4DB	multiple-copy	argininemethyltransferase10
Aradu.MGD3M	Araip.AYL5E	multiple-copy	argininemethyltransferase11
Aradu.5DL1E	Araip.HK3K8	multiple-copy	argininemethyltransferase3
Aradu.ZJM8H	Araip.RV7VH	multiple-copy	argininemethyltransferase6
Aradu.0PC6S	Araip.JKV7Y	multiple-copy	arginineN-methyltransferase-likeprotein
Aradu.W5GQC	Araip.DFU9Y	multiple-copy	Argonautefamilyprotein
Aradu.0YT2V	Araip.YRA5M	multiple-copy	Argonautefamilyprotein
Aradu.A13V2	Araip.JX6GK	multiple-copy	ARID/BRIGHTDNA-bindingdomain-containingprotein
Aradu.1R4IH	Araip.8E1SN	multiple-copy	ARMrepeatsuperfamilyprotein
Aradu.V4IDM	Araip.NVJ9Q	multiple-copy	ARMrepeatsuperfamilyprotein
Aradu.DF5I2	Araip.QL2G4	multiple-copy	ARMrepeatsuperfamilyprotein
Aradu.7X8FI	Araip.RL90M	multiple-copy	ARMrepeatsuperfamilyprotein
Aradu.H9ZXM	Araip.NF41G	multiple-copy	ARMrepeatsuperfamilyprotein,putativen=1Tax=Theobrom
Aradu.0XA87	Araip.1MT39	multiple-copy	arogenatedehydratase6
Aradu.54C3S	Araip.68IEK	multiple-copy	prephenatedehydrogenasefamilyprotein
Aradu.UHQ4T	Araip.S75SQ	multiple-copy	ascorbateperoxidase4

Aradu.UC2R1	Araip.2V2W4	multiple-copy	asparaginesynthetasedomain-containingprotein1-likeisofo
Aradu.T1J1B	Araip.XRY2X	multiple-copy	aspartateaminotransferase
Aradu.X1Y61	Araip.S985N	multiple-copy	aspartateaminotransferase1
Aradu.GKD3R	Araip.A03F3	multiple-copy	aspartateaminotransferase5
Aradu.PX1XB	Araip.2L6KD	multiple-copy	aspartatekinase-homoserinedehydrogenaseii
Aradu.R71CC	Araip.E9VAY	multiple-copy	aspartateracemase
Aradu.37C4I	Araip.485LJ	multiple-copy	asparticproteinaseA1
Aradu.H4MY7	Araip.T6DD9	multiple-copy	asparticproteinaseA1
Aradu.UJJ9I	Araip.YJ489	multiple-copy	aspartyl/glutamyl-tRNA(Asn/Gln)amidotransferasesubunit
Aradu.IWK4F	Araip.1C7B4	multiple-copy	aspartylproteasefamilyprotein
Aradu.4YQ11	Araip.206QL	multiple-copy	aspartylproteasefamilyprotein
Aradu.J43S7	Araip.2P2KT	multiple-copy	aspartylproteasefamilyprotein
Aradu.UB13R	Araip.6A16L	multiple-copy	aspartylproteasefamilyprotein
Aradu.Z9YF8	Araip.89AKE	multiple-copy	aspartylproteasefamilyprotein
Aradu.YR762	Araip.A4Y4Y	multiple-copy	aspartylproteasefamilyprotein
Aradu.64HJW	Araip.G8EGJ	multiple-copy	aspartylproteasefamilyprotein
Aradu.BA701	Araip.GP92E	multiple-copy	aspartylproteasefamilyprotein
Aradu.E79Y1	Araip.GS8E3	multiple-copy	aspartylproteasefamilyprotein
Aradu.A723B	Araip.H1AF4	multiple-copy	aspartylproteasefamilyprotein
Aradu.50TKD	Araip.IL01A	multiple-copy	aspartylproteasefamilyprotein
Aradu.R1DHB	Araip.K3A6V	multiple-copy	aspartylproteasefamilyprotein
Aradu.AE6VJ	Araip.L49IE	multiple-copy	aspartylproteasefamilyprotein
Aradu.63Q7N	Araip.PJ656	multiple-copy	aspartylproteasefamilyprotein
Aradu.YF7CH	Araip.QRI3R	multiple-copy	aspartylproteasefamilyprotein
Aradu.1C5Z6	Araip.S0S72	multiple-copy	aspartylproteasefamilyprotein
Aradu.ZYN4B	Araip.U9Z5C	multiple-copy	aspartylproteasefamilyprotein
Aradu.DS3R5	Araip.6L8TP	multiple-copy	ataxin-3homologisoformX1[Glycinemax]
Aradu.51ELU	Araip.33RQP	multiple-copy	AThookmotifDNA-bindingfamilyprotein
Aradu.H5END	Araip.679B1	multiple-copy	AThookmotifDNA-bindingfamilyprotein
Aradu.XN81L	Araip.74B2T	multiple-copy	AThookmotifDNA-bindingfamilyprotein
Aradu.L4KP0	Araip.IN0U2	multiple-copy	AThookmotifDNA-bindingfamilyprotein
Aradu.L5KF2	Araip.J00PK	multiple-copy	AThookmotifDNA-bindingfamilyprotein
Aradu.HR13D	Araip.V4XJQ	multiple-copy	AThookmotifDNA-bindingfamilyprotein
Aradu.TI2TK	Araip.YIG64	multiple-copy	AThookmotifDNA-bindingfamilyprotein
Aradu.44QR7	Araip.Z1KYK	multiple-copy	AThookmotifDNA-bindingfamilyprotein
Aradu.035RQ	Araip.8276P	multiple-copy	ATPase,V0complex,subunitE
Aradu.FL7HA	Araip.ZJE85	multiple-copy	ATPasefamilyAAAdomain-containingprotein1-like[Glycine
Aradu.E3GM7	Araip.1A4HT	multiple-copy	ATPasefamilyAAAdomain-containingprotein3-B-like[Glyci
Aradu.292D3	Araip.G5CUD	multiple-copy	ATPbinding
Aradu.QZS0L	Araip.MT4D8	multiple-copy	ATPbinding
Aradu.P98IW	Araip.0V3N6	multiple-copy	ATPbinding/kinase/proteinkinase/proteinserine/threoninek
Aradu.0MP9W	Araip.8PG2B	multiple-copy	ATPbinding/proteinserine/threoninekinase[Glycinemax]
Aradu.8567N	Araip.C4RSW	multiple-copy	ATPbinding/valine-tRNAligase/aminoacyl-tRNAligasen=4
Aradu.46Z90	Araip.291LW	multiple-copy	ATP-bindingABCtransporter

Aradu.9D9RN	Araip.2Y6XY	multiple-copy	ATP-bindingABCtransporter
Aradu.F5B1R	Araip.55A1G	multiple-copy	ATP-bindingABCtransporter
Aradu.CH84G	Araip.55F8V	multiple-copy	ATP-bindingABCtransporter
Aradu.XYY87	Araip.90JFZ	multiple-copy	ATP-bindingABCtransporter
Aradu.XQ5N2	Araip.AC7TH	multiple-copy	ATP-bindingABCtransporter
Aradu.5H0S7	Araip.CNB2V	multiple-copy	ATP-bindingABCtransporter
Aradu.PPM14	Araip.FBH15	multiple-copy	ABCtransporter(ATP-bindingprotein),putative
Aradu.2BI4W	Araip.FP1WW	multiple-copy	ATP-bindingABCtransporter
Aradu.M7GVG	Araip.G1HHU	multiple-copy	ATP-bindingABCtransporter
Aradu.TN9DS	Araip.GZ4IV	multiple-copy	ATP-bindingABCtransporter
Aradu.72QL5	Araip.HUC28	multiple-copy	ATP-bindingABCtransporter
Aradu.ZR4H7	Araip.IAU9S	multiple-copy	ATP-bindingABCtransporter
Aradu.YXJ1Q	Araip.J0VEU	multiple-copy	ATP-bindingABCtransporter
Aradu.2DG1V	Araip.JIW1A	multiple-copy	ATP-bindingABCtransporter
Aradu.YVU9U	Araip.MR96V	multiple-copy	ATP-bindingABCtransporter
Aradu.ASE53	Araip.QD22A	multiple-copy	ATP-bindingABCtransporter
Aradu.TBQ82	Araip.RDT17	multiple-copy	ATP-bindingABCtransporter
Aradu.J807R	Araip.T4UZH	multiple-copy	ATP-bindingABCtransporter
Aradu.EU9MI	Araip.V9IEZ	multiple-copy	ABCtransporterfamilyprotein
Aradu.E101D	Araip.WN11J	multiple-copy	ATP-bindingABCtransporter
Aradu.81GPB	Araip.XVV6S	multiple-copy	ATP-bindingABCtransporter
Aradu.694KT	Araip.Y3YQU	multiple-copy	ATP-bindingABCtransporter
Aradu.DES3N	Araip.LBS0B	multiple-copy	ATP-bindingcassetteproteinn=1Tax=LotusjaponicusRepI[illegible]
Aradu.RV9GW	Araip.M7L7S	multiple-copy	ATPbindingcassettesubfamilyA4
Aradu.3F84N	Araip.Z2ADB	multiple-copy	ATPbindingcassettesubfamilyB4isoform2n=2Tax=Theobr[illegible]
Aradu.BSR1D	Araip.Q10LZ	multiple-copy	ATP-bindingcassettetransportfamilyproteinn=1Tax=Popu[illegible]
Aradu.VX1FR	Araip.67SLS	multiple-copy	ATPbindingmicrotubulemotorfamilyprotein
Aradu.KK6CI	Araip.6WG88	multiple-copy	ATPbindingmicrotubulemotorfamilyprotein
Aradu.N0P3W	Araip.72USN	multiple-copy	ATPbindingmicrotubulemotorfamilyprotein
Aradu.ZFX80	Araip.7MD3E	multiple-copy	ATPbindingmicrotubulemotorfamilyprotein
Aradu.T2VBJ	Araip.BNK4F	multiple-copy	ATPbindingmicrotubulemotorfamilyprotein
Aradu.KB518	Araip.NR5KZ	multiple-copy	ATPbindingmicrotubulemotorfamilyprotein
Aradu.GT6LC	Araip.S13M1	multiple-copy	ATPbindingmicrotubulemotorfamilyprotein
Aradu.N0271	Araip.TDM9U	multiple-copy	ATPbindingmicrotubulemotorfamilyprotein
Aradu.8J5I0	Araip.Y987Q	multiple-copy	ATPbindingmicrotubulemotorfamilyprotein
Aradu.AF8HB	Araip.ZM608	multiple-copy	ATPbindingmicrotubulemotorfamilyprotein
Aradu.6R624	Araip.4JD4I	multiple-copy	ATP-bindingmicrotubulemotorfamilyprotein
Aradu.GH8FF	Araip.H1ZVY	multiple-copy	ATP-bindingmicrotubulemotorfamilyprotein
Aradu.S5UQ2	Araip.7EU1J	multiple-copy	ATPbindingmicrotubulemotorfamilyprotein,putativeisofor[illegible]
Aradu.S46KB	Araip.965G6	multiple-copy	ATPbindingmicrotubulemotorfamilyproteinisoform1n=2Ta[illegible]
Aradu.U77H9	Araip.P048V	multiple-copy	ATPbindingmicrotubulemotorfamilyproteinisoform1n=2Ta[illegible]
Aradu.M6HNT	Araip.W6TR0	multiple-copy	ATPbindingmicrotubulemotorfamilyproteinn=1Tax=Theob[illegible]
Aradu.G43SA	Araip.929NY	multiple-copy	ATPbindingprotein,putativeisoform1n=2Tax=Theobroma[illegible]
Aradu.94E63	Araip.G799L	multiple-copy	ATPbindingprotein,putativeisoform1n=2Tax=Theobroma[illegible]

Aradu.8A8WP	Araip.10QHS	multiple-copy	ATPbindingprotein,putativen=1Tax=RicinuscommunisRe
Aradu.XGK7G	Araip.77QS1	multiple-copy	ATPbindingprotein,putativen=1Tax=RicinuscommunisRe
Aradu.UMN0V	Araip.M4K9W	multiple-copy	ATPbindingprotein,putativen=1Tax=RicinuscommunisRe
Aradu.T35UC	Araip.S6815	multiple-copy	ATPbindingprotein,putativen=1Tax=RicinuscommunisRe
Aradu.HS65R	Araip.Y8CYZ	multiple-copy	ATP-citratelyaseA-3
Aradu.TBX7Z	Araip.9K0LI	multiple-copy	ATP-dependentchaperoneClpB
Aradu.6FR5V	Araip.I7UCD	multiple-copy	ATP-dependentclpproteaseATP-bindingsubunitclpxn=3T
Aradu.Y5XYT	Araip.W3ZIC	multiple-copy	ATP-dependentClpproteaseproteolyticprotein
Aradu.B6G6H	Araip.9E07Y	multiple-copy	ATP-dependentDNAhelicaseRecQ
Aradu.M7VT5	Araip.WHL9Y	multiple-copy	ATP-dependenthelicaseBRM-likeisoformX4[Glycinemax]
Aradu.V9PTU	Araip.3I7XZ	multiple-copy	ATP-dependentRNAhelicase,putative
Aradu.TYF13	Araip.B832X	multiple-copy	ATP-dependentRNAhelicase,putative
Aradu.61FU3	Araip.C7K4J	multiple-copy	ATP-dependentRNAhelicase,putative
Aradu.NQ2AE	Araip.CF3QY	multiple-copy	ATP-dependentRNAhelicase,putative
Aradu.P0D43	Araip.HTH9H	multiple-copy	ATP-dependentRNAhelicase,putative
Aradu.FGV5A	Araip.U5NKT	multiple-copy	ATP-dependentRNAhelicase,putative
Aradu.C42LD	Araip.VN7CM	multiple-copy	ATP-dependentRNAhelicase,putative
Aradu.R3T9A	Araip.DE5B2	multiple-copy	ATP-dependentRNAhelicaseDbp73Disoform1n=1Tax=Th
Aradu.8SL2K	Araip.13I5K	multiple-copy	ATP-dependentzincmetalloproteaseFtsH-like[Glycinema
Aradu.MX7X2	Araip.VJ64H	multiple-copy	ATP-dependentzincmetalloproteaseFtsH-like[Glycinema
Aradu.NII42	Araip.39SAQ	multiple-copy	ATP-dependentzincmetalloproteaseFTSHprotein
Aradu.R8ADM	Araip.ADD8R	multiple-copy	ATP-dependentzincmetalloproteaseFTSHprotein
Aradu.X2X3M	Araip.US77A	multiple-copy	ATP-dependentzincmetalloproteaseFtsH-like[Glycinema
Aradu.7I7Y0	Araip.X6YYU	multiple-copy	ATPsynthaseDchain,mitochondrial
Aradu.36UYV	Araip.P3BCU	multiple-copy	ATPsynthaseF1complexassemblyfactor2,putative
Aradu.03X4Q	Araip.1942F	multiple-copy	ATPsynthasegammachain1familyproteinn=3Tax=Populu
Aradu.M5V2I	Araip.DT2WX	multiple-copy	ATPsynthaseproteinI-related
Aradu.Y66CW	Araip.S4R2R	multiple-copy	AUTOPHAGY6
Aradu.Y99HX	Araip.9M4V3	multiple-copy	AUTOPHAGY8E
Aradu.CU60X	Araip.QF5PU	multiple-copy	AUTOPHAGY8E
Aradu.Z7FSM	Araip.SCT4C	multiple-copy	autophagyproteinApg5
Aradu.R0BLH	Araip.I3LCA	multiple-copy	autophagy-relatedprotein
Aradu.227K7	Araip.AHF5C	multiple-copy	Autophagy-relatedprotein13
Aradu.7K1LW	Araip.K6Z53	multiple-copy	autophagy-relatedprotein18a-like[Glycinemax]
Aradu.3RP7R	Araip.DYF51	multiple-copy	autophagy-relatedprotein18bisoformX2[Glycinemax]
Aradu.N4RHU	Araip.W05R7	multiple-copy	autophagy-relatedprotein18bisoformX2[Glycinemax]
Aradu.KAS7C	Araip.R5NIU	multiple-copy	autophagy-relatedprotein18c-likeisoformX4[Glycinemax]
Aradu.4H56Q	Araip.JAP1G	multiple-copy	autophagy-relatedprotein9-like[Glycinemax]
Aradu.CG16W	Araip.JYZ4H	multiple-copy	autophagy-relatedprotein9-like[Glycinemax]
Aradu.MHM9J	Araip.8Z8G7	multiple-copy	Auxineffluxcarrierfamilyprotein
Aradu.91876	Araip.E9XAH	multiple-copy	Auxineffluxcarrierfamilyprotein
Aradu.VN6S3	Araip.FTB4U	multiple-copy	Auxineffluxcarrierfamilyprotein
Aradu.34QFB	Araip.JH2G7	multiple-copy	Auxineffluxcarrierfamilyprotein
Aradu.CI3I2	Araip.JPZ0F	multiple-copy	Auxineffluxcarrierfamilyprotein

Aradu.T1478	Araip.K2TUF	multiple-copy	Auxineffluxcarrierfamilyprotein
Aradu.D0URU	Araip.L03M8	multiple-copy	Auxineffluxcarrierfamilyprotein
Aradu.M2EU8	Araip.LPX1M	multiple-copy	Auxineffluxcarrierfamilyprotein
Aradu.MIQ5P	Araip.MR59S	multiple-copy	Auxineffluxcarrierfamilyprotein
Aradu.296X5	Araip.YBJ1A	multiple-copy	Auxineffluxcarrierfamilyprotein
Aradu.VV430	Araip.6W0QH	multiple-copy	auxinresponsefactor16
Aradu.UX5R5	Araip.KD3S1	multiple-copy	auxinresponsefactor16
Aradu.M8B8F	Araip.LVJ4W	multiple-copy	auxinresponsefactor16
Aradu.05DT4	Araip.45RHI	multiple-copy	auxinresponsefactor23-like[Glycinemax]
Aradu.W01YL	Araip.0MR58	multiple-copy	auxinresponsefactor19
Aradu.4WT7F	Araip.B6JY4	multiple-copy	auxinresponsefactor2
Aradu.F0544	Araip.C1R8D	multiple-copy	auxinresponsefactor11
Aradu.HL1PG	Araip.K2L1V	multiple-copy	auxinresponsefactor2
Aradu.S3CLD	Araip.MU3T5	multiple-copy	auxinresponsefactor2
Aradu.CX437	Araip.B0EXC	multiple-copy	auxinresponsefactor3-like[Glycinemax]
Aradu.YC3RY	Araip.E2SK1	multiple-copy	auxinresponsefactor4
Aradu.XM1M6	Araip.M95YN	multiple-copy	auxinresponsefactor4
Aradu.W24J0	Araip.YR3WS	multiple-copy	auxinresponsefactor4
Aradu.IWK7R	Araip.5Z0QI	multiple-copy	auxinresponsefactor6
Aradu.FTT59	Araip.P08LA	multiple-copy	auxinresponsefactor6
Aradu.G05M1	Araip.NW1RI	multiple-copy	auxinresponsefactor9
Aradu.L4JLT	Araip.RUZ03	multiple-copy	auxinresponsefactor9
Aradu.KLS9W	Araip.H6SDM	multiple-copy	Auxin-responsivefamilyprotein
Aradu.MW13W	Araip.HP9ZJ	multiple-copy	Auxin-responsivefamilyprotein
Aradu.T52CY	Araip.IC9FD	multiple-copy	Auxin-responsivefamilyprotein
Aradu.Z6UQQ	Araip.IIU3H	multiple-copy	Auxin-responsivefamilyprotein
Aradu.52XVD	Araip.K00NE	multiple-copy	Auxin-responsivefamilyprotein
Aradu.GIN82	Araip.XVL9X	multiple-copy	Auxin-responsiveproteinn=2Tax=PopulusRepID=B9GWR
Aradu.F3KB2	Araip.SP2PF	multiple-copy	AWPM-19-likefamilyprotein
Aradu.001N3	Araip.P81AY	multiple-copy	B3domain-containingtranscriptionfactorABI3-likeisoformX
Aradu.EI7Y8	Araip.3K0PR	multiple-copy	B3domain-containingtranscriptionfactorFUS3-like[Glycine
Aradu.N7H5E	Araip.NL0PP	multiple-copy	B3domain-containingtranscriptionfactorVRN1-like[Glycine
Aradu.ZY2NN	Araip.ZF9TA	multiple-copy	B3domain-containingtranscriptionfactorVRN1-likeisoform
Aradu.PX3ED	Araip.X9Q77	multiple-copy	BAGfamilymolecularchaperoneregulator1-like[Glycinema
Aradu.Q8SMK	Araip.3KU1V	multiple-copy	BAGfamilymolecularchaperoneregulator3-like[Glycinema
Aradu.W51GD	Araip.XB3PS	multiple-copy	basic7Sglobulin[Glycinemax]
Aradu.I1DFV	Araip.SH3RA	multiple-copy	Baxinhibitor-1familyprotein
Aradu.G0QWJ	Araip.X10KQ	multiple-copy	Baxinhibitor-1familyprotein
Aradu.3CF99	Araip.C736X	multiple-copy	B-cellreceptor-associated31-like
Aradu.L9IYH	Araip.DJU8S	multiple-copy	B-cellreceptor-associated31-like
Aradu.38IMA	Araip.HU5JH	multiple-copy	B-cellreceptor-associated31-like
Aradu.63EET	Araip.WRX66	multiple-copy	B-cellreceptor-associated31-like
Aradu.ZE30N	Araip.69U4D	multiple-copy	B-cellreceptor-associatedprotein31-like
Aradu.47FME	Araip.NA6B3	multiple-copy	BEE3-like[Glycinemax]

Aradu.B0Q1E	Araip.6GF41	multiple-copy	BEL1-likehomeodomainprotein1-likeisoformX4[Glycinema
Aradu.88298	Araip.60IH6	multiple-copy	BEL1-likehomeodomainprotein3-likeisoformX2[Glycinema
Aradu.9ND07	Araip.S159U	multiple-copy	BEL1-likehomeodomainprotein3-likeisoformX2[Glycinema
Aradu.73HE8	Araip.1Z76L	multiple-copy	BEL1-likehomeodomainprotein8-like[Glycinemax]
Aradu.B6TJT	Araip.J0VZ0	multiple-copy	benzylalcoholO-benzoyltransferase-like[Glycinemax]
Aradu.KPJ13	Araip.KW34A	multiple-copy	benzylalcoholO-benzoyltransferase-like[Glycinemax]
Aradu.UT3S4	Araip.T87S4	multiple-copy	benzylalcoholO-benzoyltransferase-like[Glycinemax]
Aradu.KJ1B9	Araip.TCX3B	multiple-copy	benzylalcoholO-benzoyltransferase-like[Glycinemax]
Aradu.QU0CV	Araip.FB29M	multiple-copy	BES1/BZR1homologprotein4-like[Glycinemax]
Aradu.T7YJG	Araip.WT200	multiple-copy	BES1/BZR1homologprotein4-like[Glycinemax]
Aradu.ECS2U	Araip.DMN2L	multiple-copy	Beta-1,3-N-Acetylglucosaminyltransferasefamilyprotein
Aradu.ZYI0Y	Araip.NMI4A	multiple-copy	Beta-1,3-N-Acetylglucosaminyltransferasefamilyprotein
Aradu.G3TP5	Araip.SY4A0	multiple-copy	Beta-1,3-N-Acetylglucosaminyltransferasefamilyprotein
Aradu.Y2Z3L	Araip.T5R90	multiple-copy	Beta-1,3-N-Acetylglucosaminyltransferasefamilyprotein
Aradu.DGK47	Araip.Z8KSQ	multiple-copy	Beta-1,3-N-Acetylglucosaminyltransferasefamilyprotein
Aradu.JG113	Araip.37V98	multiple-copy	beta-1,4-N-acetylglucosaminyltransferasefamilyprotein
Aradu.QFV3H	Araip.95Q23	multiple-copy	beta-1,4-N-acetylglucosaminyltransferasefamilyprotein
Aradu.R2K02	Araip.ZQ78E	multiple-copy	beta-amylase3
Aradu.ZVZ4G	Araip.2WN0Z	multiple-copy	beta-amylase5
Aradu.R0WSE	Araip.5ZJ5X	multiple-copy	beta-amylase7
Aradu.FN25A	Araip.M2HHN	multiple-copy	beta-caroteneisomeraseD27,chloroplastic-likeisoformX1[
Aradu.RZZ9E	Araip.6800W	multiple-copy	beta-fructofuranosidase
Aradu.ET296	Araip.9LD3P	multiple-copy	beta-fructofuranosidase
Aradu.8MB2V	Araip.C8IBG	multiple-copy	beta-fructofuranosidase
Aradu.23E3L	Araip.DD0BF	multiple-copy	beta-fructofuranosidase
Aradu.W64DR	Araip.ET6IJ	multiple-copy	beta-fructofuranosidase
Aradu.7N2H0	Araip.28YBL	multiple-copy	beta-fructofuranosidase5
Aradu.22IYP	Araip.225U5	multiple-copy	beta-galactosidase11
Aradu.MY8MV	Araip.28CQG	multiple-copy	beta-galactosidase11
Aradu.97C3D	Araip.SM4VG	multiple-copy	beta-galactosidase11
Aradu.0ER06	Araip.B68MT	multiple-copy	beta-galactosidase3
Aradu.Q451G	Araip.R7LB3	multiple-copy	beta-galactosidase3
Aradu.3KC68	Araip.T49YB	multiple-copy	beta-galactosidase5
Aradu.DB6JJ	Araip.GVH0P	multiple-copy	beta-galactosidase8
Aradu.H306E	Araip.Q821H	multiple-copy	beta-galactosidase-like[Glycinemax]
Aradu.XVT29	Araip.VI7E7	multiple-copy	betaglucosidase12
Aradu.BLK03	Araip.XP1CY	multiple-copy	betaglucosidase17
Aradu.5UD3Q	Araip.0DK96	multiple-copy	betaglucosidase17
Aradu.L8BYH	Araip.21F8G	multiple-copy	betaglucosidase17
Aradu.KH972	Araip.D00MK	multiple-copy	betaglucosidase17
Aradu.D4D16	Araip.SDI9F	multiple-copy	betaglucosidase40
Aradu.W7CVG	Araip.LT700	multiple-copy	betaglucosidase46
Aradu.0KB9L	Araip.W6U6L	multiple-copy	beta-xylosidase2
Aradu.WYX50	Araip.JRH45	multiple-copy	beta-xylosidase3

Aradu.UB339	Araip.12TI6	multiple-copy	bHLH)DNA-bindingsuperfamilyprotein
Aradu.5Q1VY	Araip.13D8C	multiple-copy	bHLH)DNA-bindingsuperfamilyprotein
Aradu.VUX24	Araip.8JJ8B	multiple-copy	bHLH)DNA-bindingsuperfamilyprotein
Aradu.38JU0	Araip.8M056	multiple-copy	bHLH)DNA-bindingsuperfamilyprotein
Aradu.1QN19	Araip.9H3WY	multiple-copy	bHLH)DNA-bindingsuperfamilyprotein
Aradu.ATP30	Araip.AVK7Q	multiple-copy	bHLH)DNA-bindingsuperfamilyprotein
Aradu.X6KLL	Araip.B6R33	multiple-copy	bHLH)DNA-bindingsuperfamilyprotein
Aradu.HK2E0	Araip.J3ZJD	multiple-copy	bHLH)DNA-bindingsuperfamilyprotein
Aradu.DP2D5	Araip.LC4KN	multiple-copy	bHLH)DNA-bindingsuperfamilyprotein
Aradu.B7RDX	Araip.LGM59	multiple-copy	bHLH)DNA-bindingsuperfamilyprotein
Aradu.752ZV	Araip.LP6IV	multiple-copy	bHLH36-liketranscriptionfactor[Glycinemax]
Aradu.MB6LX	Araip.LV58R	multiple-copy	bHLH)DNA-bindingsuperfamilyprotein
Aradu.KJM0J	Araip.PUL8H	multiple-copy	bHLH)DNA-bindingsuperfamilyprotein
Aradu.S0KU9	Araip.PVV4Q	multiple-copy	bHLH)DNA-bindingsuperfamilyprotein
Aradu.K82B1	Araip.RAG9J	multiple-copy	bHLH)DNA-bindingsuperfamilyprotein
Aradu.GH2K1	Araip.RX20Z	multiple-copy	bHLH)DNA-bindingsuperfamilyprotein
Aradu.K00WP	Araip.UVF95	multiple-copy	helixloophelixDNA-bindingdomainprotein
Aradu.XA7KS	Araip.Z8IAR	multiple-copy	bHLH)DNA-bindingsuperfamilyprotein
Aradu.959KY	Araip.V1VB7	multiple-copy	transcriptionfactorbHLH106-like[Glycinemax]
Aradu.065GY	Araip.W1EXI	multiple-copy	transcriptionfactorbHLH111-likeisoformX2[Glycinemax]
Aradu.J80PY	Araip.807VL	multiple-copy	transcriptionfactorbHLH123-like[Glycinemax]
Aradu.9C8PR	Araip.A8YH6	multiple-copy	transcriptionfactorbHLH112-likeisoformX1[Glycinemax]
Aradu.I0YKW	Araip.8H1P2	multiple-copy	bHLH113-like[Glycinemax]
Aradu.F6ABZ	Araip.X394B	multiple-copy	bHLH113-likeisoformX3[Glycinemax]
Aradu.6E0QJ	Araip.JLE70	multiple-copy	bHLH122[Glycinemax]
Aradu.77XDI	Araip.MY2WL	multiple-copy	bHLH122[Glycinemax]
Aradu.45U0D	Araip.0HI1A	multiple-copy	bHLH123-likeisoformX2[Glycinemax]
Aradu.K087N	Araip.78HJ7	multiple-copy	bHLH130-like[Glycinemax]
Aradu.29A27	Araip.510K0	multiple-copy	bHLH131-like[Glycinemax]
Aradu.WC9V5	Araip.JIB5P	multiple-copy	bHLH25-like[Glycinemax]
Aradu.TN75P	Araip.GQB2P	multiple-copy	bHLH30-like[Glycinemax]
Aradu.LXV06	Araip.W4GJ9	multiple-copy	bHLH30-like[Glycinemax]
Aradu.33ULW	Araip.B52UH	multiple-copy	bHLH35-like[Glycinemax]
Aradu.JI62L	Araip.RM081	multiple-copy	bHLH35-like[Glycinemax]
Aradu.M566R	Araip.YT7PR	multiple-copy	bHLH35-like[Glycinemax]
Aradu.WNQ8E	Araip.W6M4V	multiple-copy	bHLH48-like[Glycinemax]
Aradu.4U54R	Araip.44BHE	multiple-copy	bHLH51[Glycinemax]
Aradu.AE9WN	Araip.MX2SJ	multiple-copy	bHLH51-like[Glycinemax]
Aradu.8U0A6	Araip.RMK33	multiple-copy	bHLH52-like[Glycinemax]
Aradu.25NPV	Araip.6K1VA	multiple-copy	bHLH63-like[Glycinemax]
Aradu.Z6I4Q	Araip.L8LRC	multiple-copy	bHLH63-like[Glycinemax]
Aradu.V6ZMF	Araip.FH6AP	multiple-copy	bHLH66-like[Glycinemax]
Aradu.S6EUF	Araip.N59L2	multiple-copy	bHLH68isoform1[Glycinemax]
Aradu.H9EKZ	Araip.L1316	multiple-copy	bHLH68-likeisoformX1[Glycinemax]

Aradu.573UI	Araip.8W8RT	multiple-copy	bHLH74-like[Glycinemax]
Aradu.4P1MR	Araip.E7Y1X	multiple-copy	bHLH74-like[Glycinemax]
Aradu.LW8Y2	Araip.M6R3N	multiple-copy	bHLH74-like[Glycinemax]
Aradu.01B4C	Araip.4C1AU	multiple-copy	bHLH93-like[Glycinemax]
Aradu.FPU47	Araip.4U4XR	multiple-copy	bHLH93-like[Glycinemax]
Aradu.T4PBI	Araip.778BR	multiple-copy	bHLH93-like[Glycinemax]
Aradu.N5F6J	Araip.DHX2R	multiple-copy	bHLH93-like[Glycinemax]
Aradu.WUW36	Araip.0B5Q5	multiple-copy	bHLHtranscriptionfactor
Aradu.RL2B3	Araip.6S5NP	multiple-copy	bHLHtranscriptionfactor
Aradu.6VX3K	Araip.AK8SS	multiple-copy	bHLHtranscriptionfactor
Aradu.R0BEJ	Araip.B9RXI	multiple-copy	Bifunctionalinhibitor/lipid-transferprotein/seedstorage2Sal
Aradu.1T3UD	Araip.NFP9Z	multiple-copy	Bifunctionalinhibitor/lipid-transferprotein/seedstorage2Sal
Aradu.U6RXK	Araip.P20M1	multiple-copy	Bifunctionalinhibitor/lipid-transferprotein/seedstorage2Sal
Aradu.HAF4V	Araip.T14K9	multiple-copy	Bifunctionalinhibitor/lipid-transferprotein/seedstorage2Sal
Aradu.A5IW3	Araip.U2EM6	multiple-copy	Bifunctionalinhibitor/lipid-transferprotein/seedstorage2Sal
Aradu.YX3TI	Araip.XF6FZ	multiple-copy	Bifunctionalinhibitor/lipid-transferprotein/seedstorage2Sal
Aradu.25DKA	Araip.Z4ATC	multiple-copy	Bifunctionalinhibitor/lipid-transferprotein/seedstorage2Sal
Aradu.CG4KR	Araip.4I8QR	multiple-copy	biotincarboxylcarrieracetyl-CoAcarboxylase
Aradu.CP1HR	Araip.I6HDR	multiple-copy	biotincarboxylcarrieracetyl-CoAcarboxylase
Aradu.0JT6M	Araip.LLR5T	multiple-copy	biotincarboxylcarrieracetyl-CoAcarboxylase
Aradu.D0SQ3	Araip.A63ZQ	multiple-copy	bluecopperprotein-like[Glycinemax]
Aradu.D1ZWK	Araip.AKH01	multiple-copy	bluecopperprotein-like[Glycinemax]
Aradu.LE6Z2	Araip.B9M77	multiple-copy	bluecopperprotein-like[Glycinemax]
Aradu.CI6AA	Araip.BU32W	multiple-copy	bluecopperprotein-like[Glycinemax]
Aradu.TFD03	Araip.C0P67	multiple-copy	bluecopperprotein-like[Glycinemax]
Aradu.B1FJV	Araip.DV8IK	multiple-copy	bluecopperprotein-like[Glycinemax]
Aradu.AP7WX	Araip.H5I9V	multiple-copy	bluecopperprotein-like[Glycinemax]
Aradu.WL4PN	Araip.H7PIN	multiple-copy	bluecopperprotein-like[Glycinemax]
Aradu.M7MKB	Araip.K11ZS	multiple-copy	bluecopperprotein-like[Glycinemax]
Aradu.KR2M4	Araip.P1EA5	multiple-copy	bluecopperprotein-like[Glycinemax]
Aradu.68MP6	Araip.P60JL	multiple-copy	bluecopperprotein-like[Glycinemax]
Aradu.5IN4K	Araip.P84QE	multiple-copy	bluecopperprotein-like[Glycinemax]
Aradu.4V3WP	Araip.PAL72	multiple-copy	bluecopperprotein-like[Glycinemax]
Aradu.CVT5N	Araip.Q1RER	multiple-copy	bluecopperprotein-like[Glycinemax]
Aradu.G85IC	Araip.Q3DRI	multiple-copy	bluecopperprotein-like[Glycinemax]
Aradu.SG4XX	Araip.SU3H2	multiple-copy	bluecopperprotein-like[Glycinemax]
Aradu.G9W58	Araip.YBU40	multiple-copy	bluecopperprotein-like[Glycinemax]
Aradu.GUU2R	Araip.461HE	multiple-copy	BOI-relatedE3ubiquitin-proteinligase3-like[Glycinemax]
Aradu.BX0IC	Araip.62GEG	multiple-copy	BON1-associated-likeprotein
Aradu.1W5FD	Araip.Q2JPR	multiple-copy	BON1-associated-likeprotein
Aradu.3Y7QG	Araip.54EQT	multiple-copy	branched-chainaminoacidtransaminase2
Aradu.XFR5L	Araip.I71NT	multiple-copy	branched-chainaminoacidtransaminase2
Aradu.3X54U	Araip.RKC4S	multiple-copy	breastcarcinomaamplifiedsequence3protein
Aradu.0XB6R	Araip.23MFU	multiple-copy	Brevisradix-like4-likeisoformX2[Glycinemax]

Aradu.HKL0U	Araip.TZP3M	multiple-copy	BREVISRADIX-likeisoformX3[Glycinemax]
Aradu.ZV3G2	Araip.Z6KJI	multiple-copy	bromoadjacent-likedomainprotein
Aradu.WBM9H	Araip.C0E46	multiple-copy	BSDdomain-containingprotein
Aradu.B240I	Araip.T2CTI	multiple-copy	BSDdomain-containingprotein
Aradu.23FX2	Araip.J4L5F	multiple-copy	BTB/POZdomain-containingprotein
Aradu.3VR2T	Araip.L9ZIB	multiple-copy	BTB/POZdomain-containingprotein
Aradu.MV2TG	Araip.S6UEZ	multiple-copy	BTB/POZdomain-containingprotein
Aradu.VX595	Araip.VH9FH	multiple-copy	BTB/POZdomain-containingprotein
Aradu.Q8N4A	Araip.YV08U	multiple-copy	BTB/POZdomain-containingprotein
Aradu.Z8W63	Araip.1F0E9	multiple-copy	BTB/POZdomain-containingprotein[Glycinemax]
Aradu.65H3Y	Araip.23JR9	multiple-copy	BTB/POZdomain-containingprotein[Glycinemax]
Aradu.3EB7Q	Araip.2I18H	multiple-copy	BTB/POZdomain-containingprotein[Glycinemax]
Aradu.KGX8R	Araip.BV0MU	multiple-copy	BTB/POZdomain-containingprotein[Glycinemax]
Aradu.NAE9S	Araip.E5XJJ	multiple-copy	BTB/POZdomain-containingprotein[Glycinemax]
Aradu.254Z6	Araip.EV6LQ	multiple-copy	BTB/POZdomain-containingprotein[Glycinemax]
Aradu.DEY30	Araip.F7M4C	multiple-copy	BTB/POZdomain-containingprotein[Glycinemax]
Aradu.UQ2CM	Araip.H7FNG	multiple-copy	BTB/POZdomain-containingprotein[Glycinemax]
Aradu.TYQ4J	Araip.J3YS9	multiple-copy	BTB/POZdomain-containingprotein[Glycinemax]
Aradu.GT5D9	Araip.K8SF0	multiple-copy	BTB/POZdomain-containingprotein[Glycinemax]
Aradu.HVD76	Araip.KL1K8	multiple-copy	BTB/POZdomain-containingprotein[Glycinemax]
Aradu.YSR48	Araip.MEW33	multiple-copy	BTB/POZdomain-containingprotein[Glycinemax]
Aradu.Q0GRU	Araip.MT85H	multiple-copy	BTB/POZdomain-containingprotein[Glycinemax]
Aradu.H3XI7	Araip.PUU5U	multiple-copy	BTB/POZdomain-containingprotein[Glycinemax]
Aradu.V3AZX	Araip.QI64Y	multiple-copy	BTB/POZdomain-containingprotein[Glycinemax]
Aradu.YLM4Q	Araip.QJT7Y	multiple-copy	BTB/POZdomain-containingprotein[Glycinemax]
Aradu.J67TY	Araip.QK072	multiple-copy	BTB/POZdomain-containingprotein[Glycinemax]
Aradu.AJ236	Araip.U7ZF0	multiple-copy	BTB/POZdomain-containingprotein[Glycinemax]
Aradu.H9NK1	Araip.X5C2D	multiple-copy	BTB/POZdomain-containingprotein[Glycinemax]
Aradu.U3UVH	Araip.Z65SS	multiple-copy	BTB/POZdomain-containingprotein[Glycinemax]
Aradu.PP1IT	Araip.ZS6RG	multiple-copy	BTB/POZdomain-containingprotein[Glycinemax]
Aradu.TEW52	Araip.BG5YS	multiple-copy	BTB/POZdomain-containingproteinAt3g49900-likeisoform
Aradu.DQQ0U	Araip.YZ8C7	multiple-copy	BTB/POZdomain-containingproteinAt5g47800-likeisoform
Aradu.JCH8S	Araip.BT7Z7	multiple-copy	BTB/POZdomain-containingproteinAt5g47800-likeisoform
Aradu.94UHF	Araip.TQI24	multiple-copy	BTB/POZdomain-containingproteinNPY2-likeisoformX4[G
Aradu.W7H9E	Araip.RE6G3	multiple-copy	BTB/POZdomainprotein
Aradu.B1W2E	Araip.DRF2S	multiple-copy	BTBandTAZdomainprotein4
Aradu.X7RSP	Araip.ZT9T9	multiple-copy	BTB-POZandMATHdomain2
Aradu.GVQ3S	Araip.2ES43	multiple-copy	BTB-POZandMATHdomain4
Aradu.R12NH	Araip.5T5JE	multiple-copy	bZIPfamilytranscriptionfactor
Aradu.VA5ME	Araip.713R1	multiple-copy	bZIPfamilytranscriptionfactor
Aradu.YM0TI	Araip.IA4XE	multiple-copy	bZIPfamilytranscriptionfactor
Aradu.1ES3P	Araip.JE9JZ	multiple-copy	bZIPfamilytranscriptionfactor
Aradu.B3YY0	Araip.R3LNH	multiple-copy	bZIPfamilytranscriptionfactor
Aradu.7VY22	Araip.83671	multiple-copy	BZIPtranscriptionfactor

Aradu.V37F6	Araip.A9GHS	multiple-copy	BZIPtranscriptionfactor
Aradu.80AKC	Araip.C4716	multiple-copy	bZIP)transcriptionfactorfamilyproteinBasic-leucinezipper(
Aradu.IIC03	Araip.TF5MZ	multiple-copy	bZIPtranscriptionfactor
Aradu.90RE6	Araip.64P0C	multiple-copy	bZIPtranscriptionfactorbZIP122isoformX7[Glycinemax]
Aradu.JJQ1T	Araip.62AFJ	multiple-copy	bZIPtranscriptionfactorfamilyprotein
Aradu.54E1H	Araip.LXV0U	multiple-copy	bZIPtranscriptionfactorfamilyprotein
Aradu.5X8VR	Araip.C88ZW	multiple-copy	C2calcium/lipid-bindingandGRAMdomaincontainingprotei
Aradu.Z4MY7	Araip.3Z2Y1	multiple-copy	C2H2-likezincfingerprotein
Aradu.55SXN	Araip.GZ79T	multiple-copy	C2H2-likezincfingerprotein
Aradu.HTX30	Araip.H8C7N	multiple-copy	C2H2-likezincfingerprotein
Aradu.VZQ8X	Araip.R6M68	multiple-copy	C2H2-likezincfingerprotein
Aradu.B3V7U	Araip.V7D04	multiple-copy	C2H2-likezincfingerprotein
Aradu.QH7UZ	Araip.2F21P	multiple-copy	C2-H2zincfingerprotein[Glycinemax]
Aradu.BWJ69	Araip.GI9KG	multiple-copy	C2-H2zincfingerprotein[Glycinemax]
Aradu.CW391	Araip.HCR6S	multiple-copy	C2-H2zincfingerprotein[Glycinemax]
Aradu.U8XKU	Araip.I2REN	multiple-copy	C2-H2zincfingerprotein[Glycinemax]
Aradu.JC2LL	Araip.NUV8F	multiple-copy	C2-H2zincfingerprotein[Glycinemax]
Aradu.40QFE	Araip.PRX0Z	multiple-copy	C2-H2zincfingerprotein[Glycinemax]
Aradu.QR8QC	Araip.TKX33	multiple-copy	C2-H2zincfingerprotein[Glycinemax]
Aradu.C2BM8	Araip.W0JLG	multiple-copy	C2-H2zincfingerprotein[Glycinemax]
Aradu.FE1BY	Araip.S8PCM	multiple-copy	C4-dicarboxylatetransporter/malicacidtransportprotein
Aradu.1D7KB	Araip.YWU2K	multiple-copy	C4-dicarboxylatetransporter/malicacidtransportprotein
Aradu.F2ZNU	Araip.X9SZ4	multiple-copy	CAAXprenylprotease1homolog[Glycinemax]
Aradu.E2TJL	Araip.L519U	multiple-copy	CAAXprenylprotease2n=2Tax=ArabidopsisRepID=FACE
Aradu.P2N1A	Araip.UMG0L	multiple-copy	caffeoylshikimateesterase-likeisoformX1[Glycinemax]
Aradu.6B67D	Araip.YF7P1	multiple-copy	calcineurinB-like3
Aradu.ZM9CR	Araip.PW5ES	multiple-copy	calcineurinB-likeprotein1
Aradu.L5P09	Araip.58VCE	multiple-copy	calcium-bindingEFhandfamilyprotein
Aradu.E4QK5	Araip.64I9W	multiple-copy	Calcium-bindingEFhandfamilyprotein
Aradu.2C8QX	Araip.95M6U	multiple-copy	calcium-bindingEFhandfamilyprotein
Aradu.GH35A	Araip.MZL3F	multiple-copy	Calcium-bindingEFhandfamilyprotein
Aradu.F2R8P	Araip.Q10CZ	multiple-copy	Calcium-bindingEFhandfamilyprotein
Aradu.F99CN	Araip.1Z65W	multiple-copy	calcium-bindingproteinCML41-like[Glycinemax]
Aradu.PJH5B	Araip.V8ASR	multiple-copy	Calcium-bindingEF-handfamilyprotein
Aradu.5CN8X	Araip.64DCF	multiple-copy	Calcium-bindingEF-handfamilyproteinn=1Tax=Theobrom
Aradu.R23EZ	Araip.IZ8TF	multiple-copy	Calcium-bindingEF-handfamilyproteinn=1Tax=Theobrom
Aradu.DI3D2	Araip.U291S	multiple-copy	Calcium-bindingEF-handfamilyproteinn=1Tax=Theobrom
Aradu.GJN8J	Araip.IPX0R	multiple-copy	Calcium-bindingendonuclease/exonuclease/phosphatase
Aradu.Z3YHX	Araip.U55ET	multiple-copy	calcium-bindingmitochondrialcarrierproteinSCaMC-2-like[
Aradu.82C4A	Araip.C6ZL6	multiple-copy	calcium-bindingproteinCML25-like[Glycinemax]
Aradu.XBR45	Araip.M2GYW	multiple-copy	calcium-bindingproteinCML25-like[Glycinemax]
Aradu.SB5CI	Araip.BF1SB	multiple-copy	calcium-bindingproteinCML41-like[Glycinemax]
Aradu.Q4G7J	Araip.SNJ74	multiple-copy	Calcium-bindingproteincnx1n=1Tax=Ophiostomapiceae(s
Aradu.H8P6Y	Araip.10EBD	multiple-copy	Calcium-dependentlipid-binding(CaLBdomain)familyprote

Aradu.L542Q	Araip.3MW2J	multiple-copy	Calcium-dependentlipid-binding(CaLBdomain)familyprote
Aradu.CJN7V	Araip.488BQ	multiple-copy	Calcium-dependentlipid-binding(CaLBdomain)familyprote
Aradu.VB99V	Araip.7E55M	multiple-copy	Calcium-dependentlipid-binding(CaLBdomain)familyprote
Aradu.2EU9J	Araip.B3EI3	multiple-copy	Calcium-dependentlipid-binding(CaLBdomain)familyprote
Aradu.9WH4P	Araip.FN28D	multiple-copy	Calcium-dependentlipid-binding(CaLBdomain)familyprote
Aradu.HY1A1	Araip.GV8BT	multiple-copy	Calcium-dependentlipid-binding(CaLBdomain)familyprote
Aradu.R6FI2	Araip.IE1UZ	multiple-copy	Calcium-dependentlipid-binding(CaLBdomain)familyprote
Aradu.H3050	Araip.JI00S	multiple-copy	Calcium-dependentlipid-binding(CaLBdomain)familyprote
Aradu.EQ5J6	Araip.KX5RC	multiple-copy	Calcium-dependentlipid-binding(CaLBdomain)familyprote
Aradu.12WE9	Araip.LS9DG	multiple-copy	Calcium-dependentlipid-binding(CaLBdomain)familyprote
Aradu.N906W	Araip.MKC7R	multiple-copy	Calcium-dependentlipid-binding(CaLBdomain)familyprote
Aradu.A6VDY	Araip.R34A5	multiple-copy	Calcium-dependentlipid-binding(CaLBdomain)familyprote
Aradu.L2TEA	Araip.S4ZEH	multiple-copy	Calcium-dependentlipid-binding(CaLBdomain)familyprote
Aradu.WB9GS	Araip.STR9D	multiple-copy	Calcium-dependentlipid-binding(CaLBdomain)familyprote
Aradu.069GY	Araip.V3YMP	multiple-copy	Calcium-dependentlipid-binding(CaLBdomain)familyprote
Aradu.5I3G5	Araip.YI68C	multiple-copy	Calcium-dependentlipid-binding(CaLBdomain)familyprote
Aradu.YV75B	Araip.DI7GJ	multiple-copy	calcium-dependentproteinkinase16
Aradu.D31R9	Araip.LG7R1	multiple-copy	calcium-dependentproteinkinase16
Aradu.I71PE	Araip.NH8GX	multiple-copy	calcium-dependentproteinkinase16
Aradu.ZZ215	Araip.79R74	multiple-copy	calcium-dependentproteinkinase2
Aradu.P0L8F	Araip.F3SVI	multiple-copy	calcium-dependentproteinkinase20
Aradu.ZFT0U	Araip.JGS8U	multiple-copy	calcium-dependentproteinkinase28
Aradu.3A2M2	Araip.UHZ8X	multiple-copy	calcium-dependentproteinkinase28
Aradu.4P8D3	Araip.KU06I	multiple-copy	calcium-dependentproteinkinase29
Aradu.383XS	Araip.3P5TL	multiple-copy	calcium-dependentproteinkinase32
Aradu.B4AI9	Araip.V50JD	multiple-copy	calcium-dependentproteinkinase33
Aradu.W2VYJ	Araip.KL8C5	multiple-copy	calcium-dependentproteinkinase6
Aradu.P4874	Araip.T1IDG	multiple-copy	Calcium-dependentproteinkinasefamilyprotein
Aradu.HY9UB	Araip.2B884	multiple-copy	calcium-transportingATPase2,plasmamembrane-typeprot
Aradu.46IYL	Araip.DXG9R	multiple-copy	calcium-transportingATPase2,plasmamembrane-typeprot
Aradu.T37GN	Araip.K7EZ3	multiple-copy	calcium-transportingATPase2,plasmamembrane-typeprot
Aradu.C1S3R	Araip.PCB4M	multiple-copy	calcium-transportingATPase8,plasmamembrane-typeprot
Aradu.4LY04	Araip.QIP2H	multiple-copy	calcium-transportingATPase8,plasmamembrane-typeprot
Aradu.2Y48Q	Araip.BH4UN	multiple-copy	callosesynthase5
Aradu.0CM87	Araip.D0KPG	multiple-copy	calmodulin-bindingfamilyprotein
Aradu.MS73B	Araip.Y8RPI	multiple-copy	calmodulin-bindingheat-shockprotein
Aradu.9V9BC	Araip.3M36F	multiple-copy	Calmodulin-bindingprotein
Aradu.RF6Z3	Araip.KQ1XS	multiple-copy	Calmodulin-bindingprotein
Aradu.G1Z6G	Araip.MJE46	multiple-copy	Calmodulin-bindingprotein
Aradu.X3GYY	Araip.U32YB	multiple-copy	Calmodulin-bindingprotein
Aradu.7N1F7	Araip.V1SVU	multiple-copy	Calmodulin-bindingprotein
Aradu.4IC4Q	Araip.ZJ8ZP	multiple-copy	Calmodulin-bindingprotein
Aradu.0Q24I	Araip.H8S62	multiple-copy	Calmodulinbindingprotein-like
Aradu.GD6AN	Araip.T58GE	multiple-copy	Calmodulinbindingprotein-like

Aradu.YWN5L	Araip.ZIC7C	multiple-copy	calmodulin-bindingprotein-related
Aradu.EA3WK	Araip.N7IIM	multiple-copy	calmodulin-bindingreceptor-likecytoplasmickinase1
Aradu.1LG94	Araip.4CU64	multiple-copy	calmodulin-bindingtranscriptionactivator
Aradu.KT95M	Araip.W4U3Q	multiple-copy	calmodulin-bindingtranscriptionactivator
Aradu.G8KNY	Araip.FL5PZ	multiple-copy	calmodulin-bindingtranscriptionactivator4isoformX2[Glyci
Aradu.XA41R	Araip.8CC6W	multiple-copy	Calreticulin2,calcium-bindingproteinn=1Tax=Coccomyxas
Aradu.V71C6	Araip.D5TXG	multiple-copy	calreticulin3
Aradu.YP8K9	Araip.E43K9	multiple-copy	CAP(Cysteine-richsecretoryproteins,Antigen5,andPathog
Aradu.GEA65	Araip.YRZ98	multiple-copy	CAP(Cysteine-richsecretoryproteins,Antigen5,andPathog
Aradu.D9QX6	Araip.Y6RSY	multiple-copy	Carbamoyl-phosphatesynthasesmallchainn=2Tax=Roseit
Aradu.6Q0CD	Araip.4Z79G	multiple-copy	carbohydrateesteraseAt4g34215-likeisoformX1[Glycinem
Aradu.M9CLB	Araip.BB6WZ	multiple-copy	carbohydrateesteraseAt4g34215-likeisoformX1[Glycinem
Aradu.K4APN	Araip.4F18W	multiple-copy	carbonicanhydrase1
Aradu.00MP0	Araip.T85A3	multiple-copy	carbonicanhydrase2
Aradu.4V0GE	Araip.Y8ZPE	multiple-copy	carboxylesterase13-like[Glycinemax]
Aradu.TX9D6	Araip.R02Z7	multiple-copy	carboxylesterase15-like[Glycinemax]
Aradu.QYX69	Araip.Q20Y3	multiple-copy	carboxylesterase17-like[Glycinemax]
Aradu.NJJ5I	Araip.6D3E7	multiple-copy	carboxylesterase18-like[Glycinemax]
Aradu.H46EZ	Araip.JTJ8V	multiple-copy	carboxylesterase9-like[Glycinemax]
Aradu.G3XBG	Araip.5GT5G	multiple-copy	caseinkinaseIIbetasubunit4
Aradu.8W1TJ	Araip.9K299	multiple-copy	caseinkinaseI-like5
Aradu.X3NJ9	Araip.53HJX	multiple-copy	casparianstripmembraneprotein
Aradu.CLE55	Araip.P8NLK	multiple-copy	casparianstripmembraneprotein
Aradu.P0D7S	Araip.HIX6A	multiple-copy	casparianstripmembraneprotein4-like[Glycinemax]
Aradu.KIY9T	Araip.JZF59	multiple-copy	casparianstripmembraneprotein5[Glycinemax]
Aradu.8GS2N	Araip.I1EY6	multiple-copy	CASP-likeproteinRCOM_0864260-like[Glycinemax]
Aradu.C04VQ	Araip.F0MKW	multiple-copy	catalyticLigBsubunitofaromaticring-openingdioxygenasefa
Aradu.Z5U1L	Araip.K5K1N	multiple-copy	cation/H+exchanger18
Aradu.RSV1J	Araip.Q0ZQ7	multiple-copy	cation/H+exchanger18
Aradu.E8YAE	Araip.R90BF	multiple-copy	cation/H+exchanger18
Aradu.96IDX	Araip.W8ITJ	multiple-copy	cation/H+exchanger18
Aradu.BX9V6	Araip.8VU19	multiple-copy	cation/H+exchanger19
Aradu.W4N58	Araip.B0TCT	multiple-copy	cation/H+exchanger19
Aradu.MP583	Araip.NV9EP	multiple-copy	cation/H+exchanger19
Aradu.T6HF1	Araip.T68FX	multiple-copy	cation/H(+)antiporter15-like[Glycinemax]
Aradu.2HT1T	Araip.PX6UV	multiple-copy	cation/H+exchanger2
Aradu.HP39D	Araip.3230M	multiple-copy	cation/H+exchanger20
Aradu.2L2SE	Araip.PN6DH	multiple-copy	cation/H+exchanger20
Aradu.0RQ1E	Araip.DB5EM	multiple-copy	cation/H+exchanger23
Aradu.GNV5R	Araip.Q4IHU	multiple-copy	cation/H+exchanger24
Aradu.GC3CE	Araip.QH6BR	multiple-copy	cation/H+exchanger3
Aradu.SN5BS	Araip.T42NE	multiple-copy	cation/H+exchanger4
Aradu.B049P	Araip.XI8EQ	multiple-copy	cationcalciumexchanger4
Aradu.9NR8A	Araip.39CBS	multiple-copy	cationeffluxprotein/zinctransporter

Aradu.P4HVI	Araip.3B9Y6	multiple-copy	cationicaminoacidtransporter5
Aradu.UI0XD	Araip.3N4EX	multiple-copy	cationicaminoacidtransporter5
Aradu.2Z821	Araip.B2EFG	multiple-copy	cationicaminoacidtransporter5
Aradu.4N3TL	Araip.WA8VW	multiple-copy	cationicaminoacidtransporter6
Aradu.0NY8Y	Araip.P2MP0	multiple-copy	CBSdomain-containingprotein
Aradu.2Y43U	Araip.493E4	multiple-copy	CBSdomain-containingproteinCBSCBSPB1-likeisoformX3
Aradu.1HN9G	Araip.B3CY6	multiple-copy	CBSdomain-containingproteinCBSCBSPB1-likeisoformX4
Aradu.RUT6G	Araip.P4TWL	multiple-copy	CBSdomain-containingproteinCBSCBSPB1-likeisoformX4
Aradu.16BXD	Araip.1309E	multiple-copy	CBSdomain-containingproteinCBSX1,chloroplastic[Glycin
Aradu.MD14G	Araip.9U01V	multiple-copy	CBSdomain-containingproteinwithadomainofunknownfunc
Aradu.C89XH	Araip.BT4VK	multiple-copy	CBSdomain-containingproteinwithadomainofunknownfunc
Aradu.8R77K	Araip.SX9F2	multiple-copy	CBSdomain-containingproteinwithadomainofunknownfunc
Aradu.R9XZF	Araip.R3TVX	multiple-copy	CCR4-associatedfactor1homolog11-like[Glycinemax]
Aradu.ZQK52	Araip.31UA1	multiple-copy	CCR4-NOTtranscriptioncomplexsubunit3-like[Glycinemax
Aradu.R4FBZ	Araip.DK1YP	multiple-copy	CCR4NOTtranscriptioncomplexsubunit4n=3Tax=Echinoc
Aradu.VBT9Y	Araip.HMS5U	multiple-copy	CCR4-NOTtranscriptioncomplexsubunit-likeprotein
Aradu.YIV0C	Araip.79FKI	multiple-copy	celldivisioncycleprotein27homologB-likeisoformX1[Glycin
Aradu.W6TAQ	Araip.5QY7Z	multiple-copy	celldivisioncycleprotein48homolog[Glycinemax]
Aradu.DV3N6	Araip.DU3VJ	multiple-copy	celldivisioncycleprotein48homolog[Glycinemax]
Aradu.C4EEM	Araip.G5KNS	multiple-copy	celldivisioncycleprotein48homolog[Glycinemax]
Aradu.YHW10	Araip.JME6F	multiple-copy	Cellulase(glycosylhydrolasefamily5)protein
Aradu.32ETH	Araip.GQ2VC	multiple-copy	cellulosesynthase6
Aradu.FF8NM	Araip.ZHH5I	multiple-copy	cellulosesynthase6
Aradu.IW3DF	Araip.M7HZK	multiple-copy	cellulosesynthase-likeD1
Aradu.YDW81	Araip.HTG9W	multiple-copy	cellulosesynthase-likeD3
Aradu.KU7WP	Araip.4397U	multiple-copy	cellulosesynthaselikeD4
Aradu.A050J	Araip.5Y8KI	multiple-copy	cellulosesynthase-likeD5
Aradu.M7K9G	Araip.CU3D7	multiple-copy	cellulosesynthaselikeE1
Aradu.WFC0U	Araip.HD6GL	multiple-copy	cellulosesynthaselikeE1
Aradu.77WYF	Araip.MZE77	multiple-copy	cellulosesynthaselikeG2
Aradu.78HGD	Araip.78H4F	multiple-copy	CellwallproteinExp1n=1Tax=MirabilisjalapaRepID=Q84L3
Aradu.DSS3T	Araip.IN0BK	multiple-copy	CellwallproteinExp1n=1Tax=MirabilisjalapaRepID=Q84L3
Aradu.V8XMS	Araip.T7KEI	multiple-copy	CellwallproteinExp1n=1Tax=MirabilisjalapaRepID=Q84L3
Aradu.IV1W2	Araip.6ZC3D	multiple-copy	CellwallproteinEXP2n=1Tax=MirabilisjalapaRepID=Q84L
Aradu.28WLZ	Araip.B56B4	multiple-copy	CellwallproteinEXP2n=1Tax=MirabilisjalapaRepID=Q84L
Aradu.EF2HR	Araip.KC908	multiple-copy	CellwallproteinEXP2n=1Tax=MirabilisjalapaRepID=Q84L
Aradu.Y1RZK	Araip.S4H30	multiple-copy	CellwallproteinEXP2n=1Tax=MirabilisjalapaRepID=Q84L
Aradu.KJE7Z	Araip.VF36H	multiple-copy	CellwallproteinEXP2n=1Tax=MirabilisjalapaRepID=Q84L
Aradu.JZT16	Araip.E30MW	multiple-copy	CellwallproteinExp4n=1Tax=MirabilisjalapaRepID=Q84L3
Aradu.E1MX8	Araip.UWK6L	multiple-copy	CellwallproteinExp4n=1Tax=MirabilisjalapaRepID=Q84L3
Aradu.RH8VE	Araip.EY77X	multiple-copy	CellwallproteinExp4n=1Tax=MirabilisjalapaRepID=Q84L3
Aradu.X3FXV	Araip.K5EKQ	multiple-copy	CellwallproteinExp4n=1Tax=StrigaasiaticaRepID=Q1W39
Aradu.VK4A9	Araip.5NJ9K	multiple-copy	cellwallproteinIFF6-likeisoformX2[Glycinemax]
Aradu.P9TWB	Araip.3Q94J	multiple-copy	Cellwallprotein-liken=3Tax=OryzaRepID=Q8H3Y9_ORYS

Aradu.T6VPT	Araip.QW1VD	multiple-copy	Cellwallprotein-liken=3Tax=OryzaRepID=Q8H3Y9_ORYS
Aradu.U96FI	Araip.C351P	multiple-copy	centromereproteinV-likeisoformX5[Glycinemax]
Aradu.F0W17	Araip.ML9SJ	multiple-copy	Chalcone-flavanoneisomerasefamilyprotein
Aradu.HR1QJ	Araip.V284D	multiple-copy	chalcone-flavanoneisomerasefamilyprotein
Aradu.WB4GB	Araip.JD11C	multiple-copy	chalconesynthase-like[Glycinemax]
Aradu.7F3TN	Araip.L8VR7	multiple-copy	chalconesynthase-like[Glycinemax]
Aradu.XX1YI	Araip.9B7I5	multiple-copy	ChaperoneDnaJ-domainsuperfamilyprotein
Aradu.412P9	Araip.C98N5	multiple-copy	ChaperoneDnaJ-domainsuperfamilyprotein
Aradu.WJU1S	Araip.I08KI	multiple-copy	ChaperoneDnaJ-domainsuperfamilyprotein
Aradu.1DT27	Araip.I1FHG	multiple-copy	ChaperoneDnaJ-domainsuperfamilyprotein
Aradu.U3EK7	Araip.IV1IH	multiple-copy	ChaperoneDnaJ-domainsuperfamilyprotein
Aradu.J3H3Z	Araip.K8LVP	multiple-copy	ChaperoneDnaJ-domainsuperfamilyprotein
Aradu.DM4CY	Araip.LLJ0S	multiple-copy	DnaJ-likeprotein[Glycinemax]
Aradu.QCH6Q	Araip.MGA4W	multiple-copy	ChaperoneDnaJ-domainsuperfamilyprotein
Aradu.SC9VF	Araip.Q0WU6	multiple-copy	ChaperoneDnaJ-domainsuperfamilyprotein
Aradu.1DA34	Araip.Q9V7K	multiple-copy	DNAJheatshockfamilyprotein
Aradu.N1YRP	Araip.SE9XW	multiple-copy	dnaJhomologsubfamilyBmember14-like[Glycinemax]
Aradu.J51N4	Araip.SJY4S	multiple-copy	ChaperoneDnaJ-domainsuperfamilyprotein
Aradu.MC57M	Araip.T280I	multiple-copy	ChaperoneDnaJ-domainsuperfamilyprotein
Aradu.A2MQ0	Araip.T4FCA	multiple-copy	ChaperoneDnaJ-domainsuperfamilyprotein
Aradu.4XP3R	Araip.T7FES	multiple-copy	ChaperoneDnaJ-domainsuperfamilyprotein
Aradu.352P0	Araip.TR5VC	multiple-copy	ChaperoneDnaJ-domainsuperfamilyprotein
Aradu.5HX5K	Araip.WWE5Q	multiple-copy	ChaperoneDnaJ-domainsuperfamilyprotein
Aradu.W56R3	Araip.XY63T	multiple-copy	ChaperoneDnaJ-domainsuperfamilyprotein
Aradu.ILS90	Araip.XZ2LF	multiple-copy	ChaperoneDnaJ-domainsuperfamilyprotein
Aradu.PRW5G	Araip.1Y2CP	multiple-copy	ChaperoneDnaJ-domainsuperfamilyprotein
Aradu.88DVA	Araip.U50TM	multiple-copy	ChaperoneDnaJ-domainsuperfamilyprotein
Aradu.8GW0S	Araip.0DM6L	multiple-copy	Chaperonin-likeRbcXprotein
Aradu.SCK30	Araip.37S9E	multiple-copy	Chaperonin-likeRbcXprotein
Aradu.CH4M9	Araip.842WX	multiple-copy	Chaperonin-likeRbcXprotein
Aradu.DCD5A	Araip.6NR98	multiple-copy	chargedmultivesicularbodyprotein
Aradu.UXI37	Araip.6RZ29	multiple-copy	chargedmultivesicularbodyprotein
Aradu.RDQ06	Araip.UY6ZE	multiple-copy	Chitinase/Hevein/PR-4/Wheatwin2
Aradu.98BJD	Araip.W4SPX	multiple-copy	Chitinase/Hevein/PR-4/Wheatwin2
Aradu.1BC5C	Araip.DN5WT	multiple-copy	chitinaseA
Aradu.1TX9B	Araip.RG2CK	multiple-copy	chitinaseA
Aradu.C0ZT2	Araip.DSQ1W	multiple-copy	chitinasedomain-containingprotein1isoformX1[Glycinema
Aradu.9AI6Q	Araip.20CID	multiple-copy	Chitinasefamilyprotein
Aradu.TW1EN	Araip.5J271	multiple-copy	Chitinasefamilyprotein
Aradu.JB051	Araip.HY7X8	multiple-copy	Chitinasefamilyprotein
Aradu.W9SJN	Araip.KCM6L	multiple-copy	Chitinasefamilyprotein
Aradu.77XML	Araip.KF8QF	multiple-copy	Chitinasefamilyprotein
Aradu.4196P	Araip.RMR7N	multiple-copy	Chitinasefamilyprotein
Aradu.E2KJV	Araip.I4NIK	multiple-copy	chitinase-likeprotein2

Aradu.R9NWV	Araip.W7R8X	multiple-copy	chitinase-likeproteinPB1E7.04c-likeisoformX3[Glycinema
Aradu.D7MSN	Araip.R0L1R	multiple-copy	chloridechannelE
Aradu.R6ZKE	Araip.X0E8P	multiple-copy	chloridechannelF
Aradu.LW197	Araip.J5SXF	multiple-copy	chlorophyllideAoxygenase
Aradu.7BV8W	Araip.14HQ9	multiple-copy	chloroplasticgroupIIAintronsplicingfacilitatorCRS1,chlorop
Aradu.56ZVJ	Araip.QZ033	multiple-copy	Chloroplastoutermembraneprotein,putative,expressedn=3
Aradu.N49M2	Araip.Q0MMZ	multiple-copy	cholinetransporter-likeprotein2-like[Glycinemax]
Aradu.IC2R8	Araip.V4QTU	multiple-copy	chorismatemutase1
Aradu.52DEQ	Araip.738JR	multiple-copy	chorismatemutase2
Aradu.E9CVQ	Araip.LQU55	multiple-copy	chromatinassemblyfactor1subunitFAS1-like[Glycinemax]
Aradu.SMW23	Araip.SE3MC	multiple-copy	chromatinassemblyfactor1subunitFAS2-likeisoformX1[Gl
Aradu.I6JFH	Araip.4BU2D	multiple-copy	chromodomain-helicase-DNA-bindingprotein1-likeisoform
Aradu.33XI8	Araip.545DL	multiple-copy	chromodomain-helicase-DNA-bindingprotein1-likeisoform
Aradu.LCP0L	Araip.5QZ4M	multiple-copy	chromodomain-helicase-DNA-bindingprotein1-likeisoform
Aradu.2V69M	Araip.8RS63	multiple-copy	chromodomain-helicase-DNA-bindingprotein1-likeisoform
Aradu.V4U2I	Araip.IT046	multiple-copy	chromodomain-helicase-DNA-bindingprotein1-likeisoform
Aradu.XP0GX	Araip.L3I3U	multiple-copy	chromodomain-helicase-DNA-bindingprotein1-likeisoform
Aradu.J4FDW	Araip.SMW5E	multiple-copy	chromodomain-helicase-DNA-bindingprotein1-likeisoform
Aradu.QL41Y	Araip.UR8F6	multiple-copy	chromodomain-helicase-DNA-bindingprotein1-likeisoform
Aradu.E58SH	Araip.VVZ0N	multiple-copy	chromodomain-helicase-DNA-bindingprotein1-likeisoform
Aradu.E79CC	Araip.ZS4TJ	multiple-copy	chromodomain-helicase-DNA-bindingprotein1-likeisoform
Aradu.02DA8	Araip.B00X5	multiple-copy	CHUP1,chloroplastic-like[Glycinemax]
Aradu.206DU	Araip.L9NXN	multiple-copy	CHUP1,chloroplastic-likeisoformX3[Glycinemax]
Aradu.VM8XK	Araip.X0ZRE	multiple-copy	CHUP1,chloroplastic-like[Glycinemax]
Aradu.ZR64Z	Araip.TWR85	multiple-copy	CHUP1,chloroplastic-likeisoformX1[Glycinemax]
Aradu.81LXC	Araip.00JXM	multiple-copy	CHUP1,chloroplastic-likeisoformX6[Glycinemax]
Aradu.K8DY2	Araip.DXM9R	multiple-copy	CHUP1,chloroplastic-likeisoformX6[Glycinemax]
Aradu.0V01P	Araip.ZP2M5	multiple-copy	CHUP1,chloroplastic-likeisoformX2[Glycinemax]
Aradu.FH10G	Araip.I1474	multiple-copy	cinnamoylcoareductase1
Aradu.GAW9B	Araip.Y03N6	multiple-copy	cinnamoylcoareductase
Aradu.L1U18	Araip.2LT0K	multiple-copy	cinnamoylcoareductase1
Aradu.LV8M3	Araip.K3RVL	multiple-copy	cinnamylalcoholdehydrogenase6
Aradu.GBM3Q	Araip.96G7D	multiple-copy	cinnamylalcoholdehydrogenase9
Aradu.GP6IB	Araip.IUY1V	multiple-copy	cinnamylalcoholdehydrogenase6
Aradu.MI2LX	Araip.WT1Z7	multiple-copy	cinnamylalcoholdehydrogenase9
Aradu.UFI9S	Araip.53WJE	multiple-copy	cinnamylalcoholdehydrogenasehomolog3
Aradu.Q5PIR	Araip.Z7C6V	multiple-copy	citrate-bindingprotein-like[Glycinemax]
Aradu.P5HY6	Araip.0W7EI	multiple-copy	Citratesynthasefamilyprotein
Aradu.3UN15	Araip.C32PD	multiple-copy	ClassIglutamineamidotransferase-likesuperfamilyprotein
Aradu.N0F3U	Araip.L7MAN	multiple-copy	ClassIglutamineamidotransferase-likesuperfamilyprotein
Aradu.FRD61	Araip.VC6AK	multiple-copy	ClassIglutamineamidotransferase-likesuperfamilyprotein
Aradu.33Q41	Araip.XD8CG	multiple-copy	ClassIglutamineamidotransferase-likesuperfamilyprotein
Aradu.ZM4HW	Araip.QLL2I	multiple-copy	clathrinadaptorcomplexesmediumsubunitfamilyprotein
Aradu.YMZ5A	Araip.T227J	multiple-copy	clathrincoatassemblyproteinAP180-like[Glycinemax]

Aradu.4IR6I	Araip.ETN8K	multiple-copy	cleavageandpolyadenylationspecificityfactor160
Aradu.U0KK7	Araip.E9ALR	multiple-copy	cleavageandpolyadenylationspecificityfactor5
Aradu.1UD93	Araip.S4KNL	multiple-copy	cleavageandpolyadenylationspecificityfactor5
Aradu.D3REG	Araip.0Y08C	multiple-copy	cleavageandpolyadenylationspecificityfactor73-I
Aradu.YUD54	Araip.2N3AS	multiple-copy	cleavageandpolyadenylationspecificityfactor73kDasubuni
Aradu.32V7X	Araip.V3PK4	multiple-copy	CLPproteaseproteolyticsubunit3
Aradu.LF76F	Araip.DQ9PJ	multiple-copy	CLPproteaseproteolyticsubunit6
Aradu.MW1NF	Araip.I2Q6L	multiple-copy	Coatomer,beta'subunit
Aradu.R2W4M	Araip.M0HKY	multiple-copy	COBRA-likeprotein4-like[Glycinemax]
Aradu.SUY1C	Araip.Z9UVM	multiple-copy	COBRA-likeprotein4-like[Glycinemax]
Aradu.NCD56	Araip.YFS8J	multiple-copy	cofactorassemblyofcomplexC
Aradu.BBF6Q	Araip.8LP4W	multiple-copy	coiled-coildomain-containingprotein174-like[Glycinemax]
Aradu.Q0NSJ	Araip.8395H	multiple-copy	Coiled-coildomain-containingprotein21,putativeisoform1n
Aradu.73Q6B	Araip.6328V	multiple-copy	coiled-coildomain-containingprotein22-likeisoformX2[Glyc
Aradu.K66VF	Araip.6035D	multiple-copy	Coiled-coildomain-containingprotein25n=17Tax=Eutheria
Aradu.Q54JY	Araip.W48V2	multiple-copy	Coiled-coildomain-containingprotein55(DUF2040)
Aradu.1W7NB	Araip.KPI1D	multiple-copy	Coiled-coildomain-containingprotein93n=75Tax=Eutheria
Aradu.MW6JM	Araip.YI047	multiple-copy	coiled-coildomain-containingprotein94homolog[Glycinema
Aradu.XDM1U	Araip.101RY	multiple-copy	coiled-coildomain-containingprotein97-like[Glycinemax]
Aradu.1X2PC	Araip.VD076	multiple-copy	componentofhighaffinitynitratetransporter
Aradu.RX5BN	Araip.TYP36	multiple-copy	condensin-2complexsubunitH2-like[Glycinemax]
Aradu.ZPL5X	Araip.S5QSK	multiple-copy	condensincomplexsubunit3-likeisoformX1[Glycinemax]
Aradu.CWT7S	Araip.TJ48Q	multiple-copy	conservedoligomericGolgicomplexsubunit4-likeisoformX2
Aradu.W51SD	Araip.EN37W	multiple-copy	conservedoligomericGolgicomplexsubunit6-like[Glycinem
Aradu.AF2FZ	Araip.Y1H47	multiple-copy	COP9signalosomecomplexsubunit2
Aradu.2HJ80	Araip.NU2XV	multiple-copy	COP9signalosomecomplexsubunit5
Aradu.11960	Araip.EIT18	multiple-copy	COP9signalosomecomplexsubunit8
Aradu.MTW86	Araip.G881G	multiple-copy	COP9signalosomecomplexsubunit-likeprotein
Aradu.A4DEK	Araip.RRZ2A	multiple-copy	COP9signalosomecomplexsubunit-likeprotein
Aradu.F3XDM	Araip.J47H3	multiple-copy	COP9signalosomesubunit6A
Aradu.142E0	Araip.P4FY7	multiple-copy	Copperamineoxidasefamilyprotein
Aradu.UF1FY	Araip.S4CS4	multiple-copy	copperamineoxidasefamilyprotein
Aradu.VUW7M	Araip.T371H	multiple-copy	Copperamineoxidasefamilyprotein
Aradu.A9E1A	Araip.V45YR	multiple-copy	Copperamineoxidasefamilyprotein
Aradu.755RJ	Araip.J1LUN	multiple-copy	copperion-bindingprotein
Aradu.2J9A4	Araip.8F4W7	multiple-copy	copper-transportingATPaseRAN1-like[Glycinemax]
Aradu.C0B8A	Araip.67BIA	multiple-copy	Core-2/I-branchingbeta-1,6-N-acetylglucosaminyltransfer
Aradu.ZN7FN	Araip.87I9S	multiple-copy	Core-2/I-branchingbeta-1,6-N-acetylglucosaminyltransfer
Aradu.I1U3H	Araip.CB4B3	multiple-copy	Core-2/I-branchingbeta-1,6-N-acetylglucosaminyltransfer
Aradu.UAH84	Araip.CLK0K	multiple-copy	Core-2/I-branchingbeta-1,6-N-acetylglucosaminyltransfer
Aradu.AJ5ZZ	Araip.FG0YW	multiple-copy	Core-2/I-branchingbeta-1,6-N-acetylglucosaminyltransfer
Aradu.NZS7L	Araip.IN81X	multiple-copy	Core-2/I-branchingbeta-1,6-N-acetylglucosaminyltransfer
Aradu.QAC2D	Araip.L0CPF	multiple-copy	Core-2/I-branchingbeta-1,6-N-acetylglucosaminyltransfer
Aradu.C114V	Araip.R25AT	multiple-copy	Core-2/I-branchingbeta-1,6-N-acetylglucosaminyltransfer

Aradu.Y1Z8I	Araip.RR6QJ	multiple-copy	Core-2/I-branchingbeta-1,6-N-acetylglucosaminyltransfer
Aradu.80U52	Araip.Z4GMI	multiple-copy	Core-2/I-branchingbeta-1,6-N-acetylglucosaminyltransfer
Aradu.HPG6B	Araip.Z9CFM	multiple-copy	Core-2/I-branchingbeta-1,6-N-acetylglucosaminyltransfer
Aradu.GQT7W	Araip.ZZ7ZP	multiple-copy	Core-2/I-branchingbeta-1,6-N-acetylglucosaminyltransfer
Aradu.72VHJ	Araip.78Q2V	multiple-copy	Cornichonfamilyprotein
Aradu.YM44Q	Araip.KNZ84	multiple-copy	Cornichonfamilyprotein
Aradu.P86W5	Araip.J1YCX	multiple-copy	C-terminaldomainphosphatase-like4
Aradu.8V7CS	Araip.ZV22A	multiple-copy	C-terminalprocessingpeptidasesubfamilyn=1Tax=Synech
Aradu.H0XA8	Araip.06FXT	multiple-copy	CTV.20n=1Tax=PoncirustrifoliataRepID=Q8H6Q8_PONT
Aradu.YA97R	Araip.F7X3N	multiple-copy	cyclicnucleotidegatedchannel19
Aradu.MI3Z8	Araip.1NJ8N	multiple-copy	cyclicnucleotide-gatedchanneln=1Tax=Populustrichocarp
Aradu.58FPQ	Araip.37R5L	multiple-copy	cyclicnucleotide-gatedionchannel15-likeisoformX1[Glycin
Aradu.EG6L6	Araip.1Z5U3	multiple-copy	cyclicnucleotide-gatedionchannel8-likeisoformX2[Glycine
Aradu.561J2	Araip.73UEX	multiple-copy	cyclicnucleotide-gatedionchannel-likeprotein
Aradu.ZJ7LY	Araip.A70P4	multiple-copy	cyclicnucleotide-gatedionchannel-likeprotein
Aradu.QZ44H	Araip.D5K21	multiple-copy	cyclicnucleotide-gatedionchannel-likeprotein
Aradu.U8CR2	Araip.E2AI3	multiple-copy	cyclicnucleotide-gatedionchannel-likeprotein
Aradu.QV0NG	Araip.LLP3C	multiple-copy	cyclicnucleotide-gatedchannel15
Aradu.88Z5T	Araip.KX771	multiple-copy	CyclinA2
Aradu.YV0Y1	Araip.M7KCZ	multiple-copy	CyclinA3
Aradu.QN5ZJ	Araip.L5ERK	multiple-copy	CyclinB1
Aradu.665TV	Araip.S2YNN	multiple-copy	CYCLINB1
Aradu.KW0UC	Araip.3NN3F	multiple-copy	CYCLIND1
Aradu.MEC8Q	Araip.5CH0R	multiple-copy	CYCLIND1
Aradu.Y9QIK	Araip.TE2GJ	multiple-copy	CyclinD2
Aradu.J5XVH	Araip.CI752	multiple-copy	CYCLIND3
Aradu.137AN	Araip.FZW6D	multiple-copy	CYCLIND3
Aradu.P9DZZ	Araip.ZBY9B	multiple-copy	CYCLIND4
Aradu.QYX7X	Araip.F7YE0	multiple-copy	cyclind5
Aradu.6KC8S	Araip.Y8FDH	multiple-copy	cyclin-D5-1-like[Glycinemax]
Aradu.VRG75	Araip.67BDE	multiple-copy	cyclin-D5-3-like[Glycinemax]
Aradu.K7AFI	Araip.HU8XL	multiple-copy	CyclinD6
Aradu.5GS43	Araip.3M2TW	multiple-copy	Cyclin-dependentkinaseinhibitorfamilyprotein
Aradu.IQS3D	Araip.I99X3	multiple-copy	Cyclin-dependentkinaseinhibitorfamilyprotein
Aradu.L3NRF	Araip.W3HL3	multiple-copy	Cyclin-dependentkinaseinhibitorfamilyprotein
Aradu.HN39D	Araip.58NSN	multiple-copy	Cyclin-dependentproteinkinasen=5Tax=AndropogoneaeF
Aradu.CC8V7	Araip.SWA4I	multiple-copy	Cyclin-dependentproteinkinasen=5Tax=AndropogoneaeF
Aradu.BEW9Q	Araip.2T05W	multiple-copy	Cyclinfamilyprotein
Aradu.ATW70	Araip.K02WH	multiple-copy	Cyclinfamilyprotein
Aradu.7B3CD	Araip.T9KCB	multiple-copy	Cyclinfamilyprotein
Aradu.7R8IR	Araip.UJ932	multiple-copy	Cyclinfamilyprotein
Aradu.L701F	Araip.UUS4S	multiple-copy	CyclinF-boxn=1Tax=TheobromacacaoRepID=UPI00042E
Aradu.DL83H	Araip.GY7IN	multiple-copy	cyclinp2
Aradu.10AS3	Araip.5I7AM	multiple-copy	cyclinp3

Aradu.L8EYA	Araip.75MM4	multiple-copy	Cyclophilin-likepeptidyl-prolylcis-transisomerasefamilypro
Aradu.041TI	Araip.BN5SV	multiple-copy	Cyclophilin-likepeptidyl-prolylcis-transisomerasefamilypro
Aradu.8AC2D	Araip.BQ8ZI	multiple-copy	Cyclophilin-likepeptidyl-prolylcis-transisomerasefamilypro
Aradu.CYS3J	Araip.C00SG	multiple-copy	Cyclophilin-likepeptidyl-prolylcis-transisomerasefamilypro
Aradu.S8E6L	Araip.IGH7A	multiple-copy	Cyclophilin-likepeptidyl-prolylcis-transisomerasefamilypro
Aradu.U4S49	Araip.MP6Y6	multiple-copy	Cyclophilin-likepeptidyl-prolylcis-transisomerasefamilypro
Aradu.01E53	Araip.NZG3T	multiple-copy	Cyclophilin-likepeptidyl-prolylcis-transisomerasefamilypro
Aradu.7W6T8	Araip.Q0ACF	multiple-copy	Cyclophilin-likepeptidyl-prolylcis-transisomerasefamilypro
Aradu.PJ1DH	Araip.WSE1J	multiple-copy	Cyclophilin-likepeptidyl-prolylcis-transisomerasefamilypro
Aradu.HG6CP	Araip.Y9XPY	multiple-copy	Cyclophilin-likepeptidyl-prolylcis-transisomerasefamilypro
Aradu.ZL9WR	Araip.R3S6C	multiple-copy	cystathioninebeta-synthase(CBS)familyprotein
Aradu.18BRD	Araip.81209	multiple-copy	Cysteine/Histidine-richC1domainfamilyprotein
Aradu.05WJ3	Araip.TD953	multiple-copy	Cysteine/Histidine-richC1domainfamilyprotein
Aradu.UF4PG	Araip.V4GNP	multiple-copy	Cysteine/Histidine-richC1domainfamilyprotein
Aradu.C674J	Araip.N2MW3	multiple-copy	cysteinedesulfurase-likeprotein
Aradu.08WSJ	Araip.48FDM	multiple-copy	cysteineproteinase1
Aradu.8KW68	Araip.F0TL2	multiple-copy	cysteineproteinase1
Aradu.V6900	Araip.NYR45	multiple-copy	cysteineproteinase1
Aradu.3L1J2	Araip.Y3GD9	multiple-copy	cysteineproteinaseinhibitor[Glycinemax]
Aradu.VX30A	Araip.JQ4PF	multiple-copy	cysteineproteinaseinhibitor1[Glycinemax]
Aradu.5Y3I5	Araip.1EQ95	multiple-copy	cysteineproteinaseinhibitor4-likeisoform1[Glycinemax]
Aradu.94J1A	Araip.W4FSU	multiple-copy	cysteineproteinaseinhibitor4-like[Glycinemax]
Aradu.X6KFI	Araip.ATF8R	multiple-copy	cysteineproteinaseinhibitor5-like[Glycinemax]
Aradu.HG68U	Araip.C5H4E	multiple-copy	Cysteineproteinasessuperfamilyprotein
Aradu.39755	Araip.D3HLX	multiple-copy	Cysteineproteinasessuperfamilyprotein
Aradu.A43U5	Araip.DI6YG	multiple-copy	Cysteineproteinasessuperfamilyprotein
Aradu.0924G	Araip.E2X73	multiple-copy	Cysteineproteinasessuperfamilyprotein
Aradu.XCA3M	Araip.J0W46	multiple-copy	Cysteineproteinasessuperfamilyprotein
Aradu.MDC65	Araip.SIY5C	multiple-copy	Cysteineproteinasessuperfamilyprotein
Aradu.S44IV	Araip.VKU4A	multiple-copy	Cysteineproteinasessuperfamilyprotein
Aradu.A16D8	Araip.X6F7N	multiple-copy	Cysteineproteinasessuperfamilyprotein
Aradu.6V6LL	Araip.1P0XB	multiple-copy	cysteine-richreceptor-likeproteinkinase10-like[Glycinemax
Aradu.2JQ32	Araip.EF1SW	multiple-copy	cysteine-richreceptor-likeproteinkinase10-like[Glycinemax
Aradu.IFW71	Araip.W6NP3	multiple-copy	cysteine-richreceptor-likeproteinkinase2-like[Glycinemax]
Aradu.UGC9M	Araip.XR0FV	multiple-copy	cysteine-richreceptor-likeproteinkinase25-like[Glycinemax
Aradu.XD25G	Araip.QL8ZY	multiple-copy	cysteine-richreceptor-likeproteinkinase29-like[Glycinemax
Aradu.62HJK	Araip.2SH4C	multiple-copy	cysteine-richrepeatsecretoryprotein3-like[Glycinemax]
Aradu.W0BBS	Araip.IA30F	multiple-copy	cysteinesynthaseC1
Aradu.R65GQ	Araip.22DFM	multiple-copy	cysteinesynthaseD2
Aradu.KH1Y4	Araip.S35TY	multiple-copy	cysteine--tRNAligase,cytoplasmic-likeisoformX1[Glycinem
Aradu.EWR3P	Araip.UPR46	multiple-copy	cystinosinhomologisoform2[Glycinemax]
Aradu.J0689	Araip.R8A8Y	multiple-copy	cytochromeB561-1
Aradu.8X6B9	Araip.VBB34	multiple-copy	cytochromeB561-1
Aradu.4A7CI	Araip.0RS02	multiple-copy	cytochromeb5-likeheme/steroid-bindingdomainprotein

Aradu.I5ETD	Araip.10KHS	multiple-copy	cytochromeb5-likeheme/steroid-bindingdomainprotein
Aradu.H77WY	Araip.J25TU	multiple-copy	cytochromeb5-likeheme/steroid-bindingdomainprotein
Aradu.L7RAQ	Araip.MTR7T	multiple-copy	cytochromeb5-likeheme/steroid-bindingdomainprotein
Aradu.26MHM	Araip.G2Y3C	multiple-copy	cytochromeb5reductase4-likeisoformX2[Glycinemax]
Aradu.UQR72	Araip.TD1JT	multiple-copy	cytochromeB-c1complexsubunit7
Aradu.T9WAY	Araip.T6HC3	multiple-copy	cytochromecbiogenesisproteinfamily
Aradu.49JIJ	Araip.D8MQT	multiple-copy	CytochromecoxidasesubunitVcfamilyprotein
Aradu.K6R51	Araip.9D5ER	multiple-copy	cytochromeP450,family710,subfamilyA,polypeptide1
Aradu.45I6B	Araip.M724S	multiple-copy	cytochromeP450,family711,subfamilyA,polypeptide1
Aradu.VK6NM	Araip.GC0LN	multiple-copy	cytochromeP450,family718
Aradu.D5VFR	Araip.MI1DI	multiple-copy	cytochromeP450,family718
Aradu.3KJ9A	Araip.WB5PP	multiple-copy	cytochromeP450,family718
Aradu.EZJ0T	Araip.Y8HLM	multiple-copy	cytochromeP450,family718
Aradu.61A42	Araip.7J81A	multiple-copy	cytochromeP450,family78,subfamilyA,polypeptide10
Aradu.268F1	Araip.F6KVE	multiple-copy	cytochromeP450,family78,subfamilyA,polypeptide7
Aradu.IL9UG	Araip.7E4DK	multiple-copy	cytochromeP450,family88,subfamilyA,polypeptide3
Aradu.LZ6SK	Araip.PL9PT	multiple-copy	CytochromeP450likeTBPn=1Tax=MedicagotruncatulaRe
Aradu.0Z8XN	Araip.0N6RB	multiple-copy	CytochromeP450superfamilyprotein
Aradu.TCQ2L	Araip.0P3RJ	multiple-copy	CytochromeP450superfamilyprotein
Aradu.X7ZD5	Araip.11WF7	multiple-copy	CytochromeP450superfamilyprotein
Aradu.PQW7I	Araip.13H0V	multiple-copy	CytochromeP450superfamilyprotein
Aradu.4T5J3	Araip.14ENN	multiple-copy	CytochromeP450superfamilyprotein
Aradu.D8HZZ	Araip.172DZ	multiple-copy	CytochromeP450superfamilyprotein
Aradu.4I5UW	Araip.18CW0	multiple-copy	CytochromeP450superfamilyprotein
Aradu.BYJ1W	Araip.19H18	multiple-copy	CytochromeP450superfamilyprotein
Aradu.R6QT2	Araip.21BTV	multiple-copy	CytochromeP450superfamilyprotein
Aradu.J9D9F	Araip.3J4UV	multiple-copy	CytochromeP450superfamilyprotein
Aradu.UP0S8	Araip.3S762	multiple-copy	CytochromeP450superfamilyprotein
Aradu.9M8FI	Araip.44Q3I	multiple-copy	CytochromeP450superfamilyprotein
Aradu.XA7IS	Araip.49GZ0	multiple-copy	CytochromeP450superfamilyprotein
Aradu.YDZ7F	Araip.4EU14	multiple-copy	CytochromeP450superfamilyprotein
Aradu.1Z30Z	Araip.4L730	multiple-copy	CytochromeP450superfamilyprotein
Aradu.3K9KE	Araip.51AE7	multiple-copy	CytochromeP450superfamilyprotein
Aradu.ZWA6F	Araip.642G1	multiple-copy	CytochromeP450superfamilyprotein
Aradu.3TT1Y	Araip.67S5I	multiple-copy	CytochromeP450superfamilyprotein
Aradu.FF4D5	Araip.6PJ8B	multiple-copy	CytochromeP450superfamilyprotein
Aradu.J296J	Araip.7B1XA	multiple-copy	CytochromeP450superfamilyprotein
Aradu.WJ5JK	Araip.7FJ61	multiple-copy	CytochromeP450superfamilyprotein
Aradu.32QV2	Araip.88UBN	multiple-copy	CytochromeP450superfamilyprotein
Aradu.SG97P	Araip.9KU8R	multiple-copy	CytochromeP450superfamilyprotein
Aradu.6HJ87	Araip.9P3Y0	multiple-copy	CytochromeP450superfamilyprotein
Aradu.Q9E1W	Araip.A4X5Z	multiple-copy	CytochromeP450superfamilyprotein
Aradu.5F6FU	Araip.B1BRC	multiple-copy	CytochromeP450superfamilyprotein
Aradu.0I3GY	Araip.B5SML	multiple-copy	CytochromeP450superfamilyprotein

Aradu.29ERF	Araip.BJ1LC	multiple-copy	CytochromeP450superfamilyprotein
Aradu.QH0IG	Araip.BS4D0	multiple-copy	CytochromeP450superfamilyprotein
Aradu.G7CZU	Araip.BT3ZZ	multiple-copy	CytochromeP450superfamilyprotein
Aradu.Q3FM7	Araip.BYM6A	multiple-copy	CytochromeP450superfamilyprotein
Aradu.L3X78	Araip.C0T71	multiple-copy	CytochromeP450superfamilyprotein
Aradu.QS20K	Araip.DB8NC	multiple-copy	CytochromeP450superfamilyprotein
Aradu.DXW6X	Araip.ET7IL	multiple-copy	CytochromeP450superfamilyprotein
Aradu.QY2T7	Araip.G0H3V	multiple-copy	CytochromeP450superfamilyprotein
Aradu.N7FJP	Araip.H625D	multiple-copy	CytochromeP450superfamilyprotein
Aradu.9JB4Z	Araip.HD4P6	multiple-copy	CytochromeP450superfamilyprotein
Aradu.I957I	Araip.HF56B	multiple-copy	CytochromeP450superfamilyprotein
Aradu.LSL3G	Araip.HWV62	multiple-copy	CytochromeP450superfamilyprotein
Aradu.40CKU	Araip.J51X4	multiple-copy	CytochromeP450superfamilyprotein
Aradu.K73MN	Araip.JB4PG	multiple-copy	CytochromeP450superfamilyprotein
Aradu.VV1WJ	Araip.JRA2H	multiple-copy	CytochromeP450superfamilyprotein
Aradu.N1Z7I	Araip.JSV6Q	multiple-copy	CytochromeP450superfamilyprotein
Aradu.UK6QI	Araip.K0ECG	multiple-copy	CytochromeP450superfamilyprotein
Aradu.J85DC	Araip.K6VWA	multiple-copy	CytochromeP450superfamilyprotein
Aradu.Y7I9D	Araip.KXX24	multiple-copy	CytochromeP450superfamilyprotein
Aradu.XT2G5	Araip.KZ32L	multiple-copy	CytochromeP450superfamilyprotein
Aradu.8J00F	Araip.L1D01	multiple-copy	CytochromeP450superfamilyprotein
Aradu.I2Z9Y	Araip.L96AH	multiple-copy	CytochromeP450superfamilyprotein
Aradu.IM9AT	Araip.M5K02	multiple-copy	CytochromeP450superfamilyprotein
Aradu.D1G1F	Araip.NGS4U	multiple-copy	CytochromeP450superfamilyprotein
Aradu.MZ49K	Araip.NPU4T	multiple-copy	CytochromeP450superfamilyprotein
Aradu.1B2LG	Araip.NY5B8	multiple-copy	CytochromeP450superfamilyprotein
Aradu.T1K5W	Araip.PE948	multiple-copy	CytochromeP450superfamilyprotein
Aradu.RI6SZ	Araip.PGI83	multiple-copy	CytochromeP450superfamilyprotein
Aradu.73MTJ	Araip.Q25ER	multiple-copy	CytochromeP450superfamilyprotein
Aradu.K6UHT	Araip.Q34LR	multiple-copy	CytochromeP450superfamilyprotein
Aradu.MRQ6G	Araip.QX9UN	multiple-copy	CytochromeP450superfamilyprotein
Aradu.K8EE3	Araip.R6JKD	multiple-copy	CytochromeP450superfamilyprotein
Aradu.TJ0ZU	Araip.RXK1S	multiple-copy	CytochromeP450superfamilyprotein
Aradu.LH845	Araip.S24CF	multiple-copy	CytochromeP450superfamilyprotein
Aradu.DB13D	Araip.S5BKS	multiple-copy	CytochromeP450superfamilyprotein
Aradu.A5EC7	Araip.S7EMP	multiple-copy	CytochromeP450superfamilyprotein
Aradu.BI3QN	Araip.SIS4H	multiple-copy	CytochromeP450superfamilyprotein
Aradu.Q8MCV	Araip.T1KRW	multiple-copy	CytochromeP450superfamilyprotein
Aradu.G2TPD	Araip.T6L7D	multiple-copy	CytochromeP450superfamilyprotein
Aradu.1X6G8	Araip.T9LT0	multiple-copy	CytochromeP450superfamilyprotein
Aradu.6AL72	Araip.TS2QL	multiple-copy	CytochromeP450superfamilyprotein
Aradu.JH3GC	Araip.UA42P	multiple-copy	CytochromeP450superfamilyprotein
Aradu.6C1Z1	Araip.UU39R	multiple-copy	CytochromeP450superfamilyprotein
Aradu.P51B9	Araip.V01DZ	multiple-copy	CytochromeP450superfamilyprotein

Aradu.H8Q03	Araip.V86U2	multiple-copy	CytochromeP450superfamilyprotein
Aradu.65CE7	Araip.VD1BS	multiple-copy	CytochromeP450superfamilyprotein
Aradu.75ICF	Araip.VL1E0	multiple-copy	CytochromeP450superfamilyprotein
Aradu.KJH8E	Araip.WI0M1	multiple-copy	CytochromeP450superfamilyprotein
Aradu.9NC59	Araip.WS1DN	multiple-copy	CytochromeP450superfamilyprotein
Aradu.49PAS	Araip.WX8L5	multiple-copy	CytochromeP450superfamilyprotein
Aradu.1WD61	Araip.XWP57	multiple-copy	CytochromeP450superfamilyprotein
Aradu.623GS	Araip.Z53WS	multiple-copy	CytochromeP450superfamilyprotein
Aradu.XW8AZ	Araip.ZD9NB	multiple-copy	CytochromeP450superfamilyprotein
Aradu.GA8EY	Araip.ZY8TR	multiple-copy	CytochromeP450superfamilyprotein
Aradu.09SAF	Araip.B1IVR	multiple-copy	cytokininoxidase/dehydrogenase1
Aradu.U999X	Araip.CJ1ZA	multiple-copy	cytokininoxidase/dehydrogenase1
Aradu.DVQ9A	Araip.DKI8Z	multiple-copy	cytokininoxidase/dehydrogenase1
Aradu.D11TE	Araip.SP6NR	multiple-copy	cytokininoxidase/dehydrogenase1
Aradu.SE3WE	Araip.RT6FK	multiple-copy	cytokininoxidase/dehydrogenase6
Aradu.DUE48	Araip.A3A9L	multiple-copy	cytokininriboside5'-monophosphatephosphoribohydrolase
Aradu.Z4QII	Araip.HF6E7	multiple-copy	cytoplasmic-likeaconitatehydratase
Aradu.UR0SD	Araip.U9YQ5	multiple-copy	D-3-phosphoglyceratedehydrogenase
Aradu.ZUG9I	Araip.UD1ML	multiple-copy	D-isomerspecific2-hydroxyaciddehydrogenaseNAD-bindi
Aradu.A32HC	Araip.FQ9GE	multiple-copy	D6proteinkinaselike2
Aradu.Z197N	Araip.H6F7C	multiple-copy	D6proteinkinaselike2
Aradu.UPE5A	Araip.X7QQD	multiple-copy	D6proteinkinaselike2
Aradu.L1KP9	Araip.L8E8C	multiple-copy	DA1-related2-likeisoformX1[Glycinemax]
Aradu.C96YL	Araip.P89N6	multiple-copy	DCD(DevelopmentandCellDeath)domainprotein
Aradu.LI04Q	Araip.WGQ93	multiple-copy	DCD(DevelopmentandCellDeath)domainprotein
Aradu.M0XVK	Araip.F1WJW	multiple-copy	dCTPpyrophosphatase1-like[Glycinemax]
Aradu.Y6F9Q	Araip.NBA78	multiple-copy	dCTPpyrophosphatase1-like[Glycinemax]
Aradu.K1BYW	Araip.WUV4J	multiple-copy	dCTPpyrophosphatase1-like[Glycinemax]
Aradu.D51RM	Araip.7X70M	multiple-copy	DEAD-boxATP-dependentRNAhelicase
Aradu.L0GAW	Araip.LUJ64	multiple-copy	DEAD-boxATP-dependentRNAhelicase
Aradu.8ND9A	Araip.N0NQI	multiple-copy	DEAD-boxATP-dependentRNAhelicase
Aradu.0NY83	Araip.T68Y6	multiple-copy	DEAD-boxATP-dependentRNAhelicase
Aradu.YQW51	Araip.UM7BZ	multiple-copy	DEAD-boxATP-dependentRNAhelicase
Aradu.0WA6T	Araip.W7GWX	multiple-copy	DEAD-boxATP-dependentRNAhelicase
Aradu.C7C1R	Araip.WW83V	multiple-copy	DEAD-boxATP-dependentRNAhelicase
Aradu.SC5U0	Araip.Y5AAV	multiple-copy	DEAD-boxATP-dependentRNAhelicase
Aradu.FW65S	Araip.ZZM8J	multiple-copy	DEAD-boxATP-dependentRNAhelicase
Aradu.7VP5C	Araip.AZ3UI	multiple-copy	DEAD-boxATP-dependentRNAhelicase-likeprotein
Aradu.73ZLX	Araip.CBX44	multiple-copy	DEAD-boxATP-dependentRNAhelicase-likeprotein
Aradu.SN12S	Araip.D1L9A	multiple-copy	DEAD-boxATP-dependentRNAhelicase-likeprotein
Aradu.AN6BF	Araip.JWK6Z	multiple-copy	DEAD-boxATP-dependentRNAhelicase-likeprotein
Aradu.NI3IK	Araip.NS167	multiple-copy	DEAD-boxATP-dependentRNAhelicase-likeprotein
Aradu.8203M	Araip.PTB9G	multiple-copy	DEAD-boxATP-dependentRNAhelicase-likeprotein
Aradu.H1ZYW	Araip.QB1CB	multiple-copy	DEAD-boxATP-dependentRNAhelicase-likeprotein

Aradu.V4E4H	Araip.U364I	multiple-copy	DEAD-boxATP-dependentRNAhelicase-likeprotein
Aradu.ZNZ7Q	Araip.UIM6D	multiple-copy	DEAD-boxATP-dependentRNAhelicase-likeprotein
Aradu.WDS9Z	Araip.X05NJ	multiple-copy	decapping5-likeprotein-like[Glycinemax]
Aradu.YWE4J	Araip.QVM9W	multiple-copy	defectiveincullinneddylationprotein,putative
Aradu.WL7ND	Araip.YNV3J	multiple-copy	defectiveincullinneddylationprotein,putative
Aradu.L7CNH	Araip.TR541	multiple-copy	Dehydrinfamilyprotein
Aradu.K65B5	Araip.7EN7C	multiple-copy	dehydroquinatedehydratase,putative/shikimatedehydroge
Aradu.V5JUH	Araip.UDC6V	multiple-copy	dehydroquinatedehydratase,putative/shikimatedehydroge
Aradu.IVE8F	Araip.M2UTN	multiple-copy	DENN(AEX-3)domain-containingprotein
Aradu.H49WY	Araip.Q7XEQ	multiple-copy	DENN(AEX-3)domain-containingprotein
Aradu.Q018E	Araip.3671W	multiple-copy	dentinsialophosphoprotein-likeisoformX1[Glycinemax]
Aradu.Z3NVH	Araip.DNI71	multiple-copy	dentinsialophosphoprotein-likeisoformX1[Glycinemax]
Aradu.UE6Z2	Araip.E8MZN	multiple-copy	dentinsialophosphoprotein-likeisoformX1[Glycinemax]
Aradu.NJ1GS	Araip.L1Q1P	multiple-copy	dentinsialophosphoprotein-like[Glycinemax]
Aradu.7HR5N	Araip.6W75R	multiple-copy	dentinsialophosphoprotein-likeisoformX2[Glycinemax]
Aradu.D400V	Araip.C6ELD	multiple-copy	dentinsialophosphoprotein-likeisoformX2[Glycinemax]
Aradu.7F8A1	Araip.S8WH0	multiple-copy	dentinsialophosphoprotein-likeisoformX2[Glycinemax]
Aradu.Q5FHV	Araip.T8ZMH	multiple-copy	dentinsialophosphoprotein-likeisoformX2[Glycinemax]
Aradu.6BK0V	Araip.SGS4X	multiple-copy	dentinsialophosphoprotein-likeisoformX3[Glycinemax]
Aradu.JF3DE	Araip.PJ16E	multiple-copy	Deoxyribodipyrimidinephoto-lyase(Single-strandedDNA-s
Aradu.GZZ2X	Araip.C2U1E	multiple-copy	DERLIN-2.2
Aradu.8M02Z	Araip.MZ6FM	multiple-copy	desiccation-relatedproteinPCC13-62-like[Glycinemax]
Aradu.T8L1U	Araip.7LQ31	multiple-copy	DHHC-typezincfingerfamilyprotein
Aradu.S0SM3	Araip.EVY0P	multiple-copy	DHHC-typezincfingerfamilyprotein
Aradu.3QR5Y	Araip.H8Y66	multiple-copy	DHHC-typezincfingerfamilyprotein
Aradu.CPF02	Araip.WN5LY	multiple-copy	DHHC-typezincfingerfamilyprotein
Aradu.94X53	Araip.4IT76	multiple-copy	DHHC-typezincfingerfamilyproteinisoform1n=1Tax=Theo
Aradu.Z4DIZ	Araip.TYL76	multiple-copy	diacylglycerolacyltransferasefamily
Aradu.V060T	Araip.Y13UG	multiple-copy	diacylglycerolkinase2
Aradu.NE5BN	Araip.72N6Y	multiple-copy	digalactosyldiacylglycerolsynthase1,chloroplastic-like[Gly
Aradu.T2XD3	Araip.VXN6M	multiple-copy	digalactosyldiacylglycerolsynthase2,chloroplastic[Glycine
Aradu.3KI43	Araip.M91DZ	multiple-copy	dihydroflavonol4-reductase
Aradu.632MM	Araip.M99F1	multiple-copy	dihydroflavonol4-reductase
Aradu.M0VKX	Araip.YIM92	multiple-copy	Dihydrolipoyllysine-residuesuccinyltransferasecomponent
Aradu.SGK85	Araip.W5V9C	multiple-copy	dihydroorotatedehydrogenase,putative
Aradu.EI99W	Araip.44UYF	multiple-copy	Dihydroxyacetonekinase
Aradu.SJK47	Araip.Q5RAQ	multiple-copy	diphosphate--fructose-6-phosphate1-phosphotransferase
Aradu.6UB5R	Araip.654N3	multiple-copy	diseaseresistancefamilyprotein/LRRfamilyprotein
Aradu.D3LC1	Araip.LKW41	multiple-copy	LRRandNB-ARCdomaindiseaseresistanceprotein
Aradu.014NJ	Araip.H43AY	multiple-copy	LRRandNB-ARCdomaindiseaseresistanceprotein
Aradu.B5ML3	Araip.IBP0A	multiple-copy	diseaseresistanceprotein
Aradu.G0IJA	Araip.JP4WR	multiple-copy	diseaseresistanceprotein
Aradu.J9ALL	Araip.S8LZD	multiple-copy	diseaseresistanceprotein
Aradu.SM8C1	Araip.TQ2BR	multiple-copy	diseaseresistanceprotein

Aradu.0NA24	Araip.U82F4	multiple-copy	diseaseresistanceprotein
Aradu.80TC2	Araip.Y9SXC	multiple-copy	diseaseresistanceprotein
Aradu.2V0TP	Araip.TL5BE	multiple-copy	Diseaseresistanceprotein(TIR-NBSclass)
Aradu.V5GXH	Araip.F4BSU	multiple-copy	diseaseresistanceprotein(TIR-NBS-LRRclass)
Aradu.KLS6P	Araip.S4GGT	multiple-copy	diseaseresistanceprotein(TIR-NBS-LRRclass)
Aradu.L3C1J	Araip.TJA70	multiple-copy	diseaseresistanceprotein(TIR-NBS-LRRclass)
Aradu.HQF0H	Araip.Y6LUD	multiple-copy	Diseaseresistanceprotein(TIR-NBS-LRRclass)family
Aradu.TP72Z	Araip.03C48	multiple-copy	diseaseresistanceprotein(TIR-NBS-LRRclass),putative
Aradu.6R3YG	Araip.224ZW	multiple-copy	diseaseresistanceprotein(TIR-NBS-LRRclass),putative
Aradu.BY4US	Araip.38QJB	multiple-copy	diseaseresistanceprotein(TIR-NBS-LRRclass),putative
Aradu.G4VT9	Araip.58W0E	multiple-copy	diseaseresistanceprotein(TIR-NBS-LRRclass),putative
Aradu.SR2IB	Araip.6MG4Z	multiple-copy	diseaseresistanceprotein(TIR-NBS-LRRclass),putative
Aradu.G78T6	Araip.83LYV	multiple-copy	diseaseresistanceprotein(TIR-NBS-LRRclass),putative
Aradu.L3RRG	Araip.C5YQ1	multiple-copy	diseaseresistanceprotein(TIR-NBS-LRRclass),putative
Aradu.C0GMC	Araip.GD26H	multiple-copy	diseaseresistanceprotein(TIR-NBS-LRRclass),putative
Aradu.ZLR3W	Araip.H1VEE	multiple-copy	diseaseresistanceprotein(TIR-NBS-LRRclass),putative
Aradu.HM4L0	Araip.H2P98	multiple-copy	diseaseresistanceprotein(TIR-NBS-LRRclass),putative
Aradu.JN158	Araip.I06X7	multiple-copy	diseaseresistanceprotein(TIR-NBS-LRRclass),putative
Aradu.4X7WG	Araip.I0NS8	multiple-copy	diseaseresistanceprotein(TIR-NBS-LRRclass),putative
Aradu.N09A5	Araip.K4VGI	multiple-copy	diseaseresistanceprotein(TIR-NBS-LRRclass),putative
Aradu.5W8P5	Araip.L6B1S	multiple-copy	diseaseresistanceprotein(TIR-NBS-LRRclass),putative
Aradu.2A8ZQ	Araip.MU8XA	multiple-copy	diseaseresistanceprotein(TIR-NBS-LRRclass),putative
Aradu.44QYZ	Araip.N2I6Z	multiple-copy	diseaseresistanceprotein(TIR-NBS-LRRclass),putative
Aradu.97AME	Araip.NRY2N	multiple-copy	diseaseresistanceprotein(TIR-NBS-LRRclass),putative
Aradu.R4V51	Araip.S5B0I	multiple-copy	diseaseresistanceprotein(TIR-NBS-LRRclass),putative
Aradu.ID7XN	Araip.U7EGE	multiple-copy	diseaseresistanceprotein(TIR-NBS-LRRclass),putative
Aradu.P5PIT	Araip.YI248	multiple-copy	diseaseresistanceprotein(TIR-NBS-LRRclass),putative
Aradu.3GT7T	Araip.ZUY8H	multiple-copy	diseaseresistanceprotein(TIR-NBS-LRRclass),putative
Aradu.GA7JT	Araip.ZVW3Y	multiple-copy	diseaseresistanceprotein(TIR-NBS-LRRclass),putative
Aradu.4DJ5M	Araip.0C951	multiple-copy	Diseaseresistanceprotein(TIR-NBS-LRRclass)family
Aradu.NSE79	Araip.0ER3Q	multiple-copy	diseaseresistanceprotein(TIR-NBS-LRRclass),putative
Aradu.51XM7	Araip.59D3Z	multiple-copy	Diseaseresistanceprotein(TIR-NBS-LRRclass)family
Aradu.YVT9V	Araip.8P06L	multiple-copy	Diseaseresistanceprotein(TIR-NBS-LRRclass)family
Aradu.HLR71	Araip.A249Q	multiple-copy	Diseaseresistanceprotein(TIR-NBS-LRRclass)family
Aradu.Y2WFH	Araip.C7JYL	multiple-copy	Diseaseresistanceprotein(TIR-NBS-LRRclass)family
Aradu.YU7JM	Araip.DBL0L	multiple-copy	diseaseresistanceprotein(TIR-NBS-LRRclass),putative
Aradu.P6DLH	Araip.FCU59	multiple-copy	Diseaseresistanceprotein(TIR-NBS-LRRclass)family
Aradu.MA8H9	Araip.FZ51R	multiple-copy	Diseaseresistanceprotein(TIR-NBS-LRRclass)family
Aradu.62QCX	Araip.R9899	multiple-copy	Diseaseresistanceprotein(TIR-NBS-LRRclass)family
Aradu.J4Y5T	Araip.V1FQK	multiple-copy	Diseaseresistanceprotein(TIR-NBS-LRRclass)family
Aradu.E0HSZ	Araip.X27S8	multiple-copy	Diseaseresistanceprotein(TIR-NBS-LRRclass)family
Aradu.LQH8Y	Araip.ZTF16	multiple-copy	Diseaseresistanceprotein(TIR-NBS-LRRclass)family
Aradu.ZSR47	Araip.C4YLK	multiple-copy	diseaseresistanceprotein[Glycinemax]
Aradu.2C9WA	Araip.GYU0T	multiple-copy	disease-resistanceresponseprotein

Aradu.9T1SG	Araip.IVG5H	multiple-copy	disease-resistanceresponseprotein
Aradu.32A1K	Araip.7N2TS	multiple-copy	Diseaseresistance-responsive(dirigent-likeprotein)familyp
Aradu.H92KN	Araip.90Y7E	multiple-copy	Diseaseresistance-responsive(dirigent-likeprotein)familyp
Aradu.HCB3B	Araip.J8I5I	multiple-copy	Diseaseresistance-responsive(dirigent-likeprotein)familyp
Aradu.YN1Y7	Araip.LW0C3	multiple-copy	Diseaseresistance-responsive(dirigent-likeprotein)familyp
Aradu.CLY5P	Araip.SQ4D9	multiple-copy	Diseaseresistance-responsive(dirigent-likeprotein)familyp
Aradu.0QA18	Araip.T5SG5	multiple-copy	Diseaseresistance-responsive(dirigent-likeprotein)familyp
Aradu.7673S	Araip.UTP9U	multiple-copy	Diseaseresistance-responsive(dirigent-likeprotein)familyp
Aradu.QV720	Araip.X6AXT	multiple-copy	Diseaseresistance-responsive(dirigent-likeprotein)familyp
Aradu.XJ6TW	Araip.9K298	multiple-copy	disulfideisomerase-likeprotein
Aradu.U7EBV	Araip.Q4MYK	multiple-copy	disulfideisomerase-like
Aradu.8P6BN	Araip.SB6R7	multiple-copy	disulfideisomerase-like
Aradu.MM6MH	Araip.V3N9B	multiple-copy	disulfideisomerase-like
Aradu.BH9NG	Araip.Q6KXW	multiple-copy	disulfideisomerase-like,putative
Aradu.443KM	Araip.K94XG	multiple-copy	disulfide-isomeraseSCO2-likeisoformX1[Glycinemax]
Aradu.B7486	Araip.EU8TM	multiple-copy	DNAbinding
Aradu.P477D	Araip.KK0GD	multiple-copy	DNAbinding
Aradu.73DHW	Araip.LYI86	multiple-copy	DNAbinding
Aradu.98BQH	Araip.U28W7	multiple-copy	DNAbinding
Aradu.03THN	Araip.Z85GJ	multiple-copy	DNAbinding
Aradu.L7120	Araip.4H3RV	multiple-copy	DNA-bindingbromodomain-containingprotein
Aradu.1Z587	Araip.64E4D	multiple-copy	DNA-bindingbromodomain-containingprotein
Aradu.C2HZQ	Araip.568TN	multiple-copy	DNA-bindingfamilyprotein
Aradu.JS2XW	Araip.FJG3C	multiple-copy	DNA-bindingfamilyprotein
Aradu.MA612	Araip.M32YC	multiple-copy	DNA-bindingfamilyproteinn=1Tax=PopulustrichocarpaRe
Aradu.2IG4B	Araip.AZH1U	multiple-copy	DNA-bindingprotein,putative
Aradu.3EF6P	Araip.H235T	multiple-copy	DNA-bindingprotein,putative
Aradu.5PD1B	Araip.LQJ0X	multiple-copy	DNA-bindingprotein,putative
Aradu.3Z6PL	Araip.UE7U5	multiple-copy	DNA-bindingprotein,putative
Aradu.86C6D	Araip.Z1EXY	multiple-copy	DNAbindingprotein,putativen=1Tax=RicinuscommunisRe
Aradu.F6LF0	Araip.2G28P	multiple-copy	DNAbindingprotein,putativen=1Tax=RicinuscommunisRe
Aradu.M0ZRG	Araip.PQ54V	multiple-copy	DNAbindingprotein,putativen=1Tax=RicinuscommunisRe
Aradu.VSF4G	Araip.UGC3H	multiple-copy	DNAbindingprotein,putativen=1Tax=RicinuscommunisRe
Aradu.JVP4N	Araip.1LL57	multiple-copy	DNA-bindingproteinESCAROLA-like[Glycinemax]
Aradu.N7NSR	Araip.1MI2Q	multiple-copy	DNA-bindingfamilyprotein
Aradu.G5F0C	Araip.T2RUM	multiple-copy	DNA-bindingproteinESCAROLA-like[Glycinemax]
Aradu.Z7FDS	Araip.0TX1W	multiple-copy	DNA-bindingproteinn=1Tax=CatharanthusroseusRepID=/
Aradu.PSF4U	Araip.CFK5T	multiple-copy	DNA-bindingproteinn=1Tax=CatharanthusroseusRepID=/
Aradu.MZX0S	Araip.H6DP8	multiple-copy	DNA-bindingproteinn=1Tax=DaucuscarotaRepID=Q4342
Aradu.FQ6XD	Araip.87M6M	multiple-copy	DNA-bindingproteinn=1Tax=CatharanthusroseusRepID=/
Aradu.6S06R	Araip.D6G4F	multiple-copy	DNA-bindingproteinn=1Tax=CatharanthusroseusRepID=/
Aradu.SEJ3V	Araip.VS7G2	multiple-copy	DNA-bindingproteinn=1Tax=DaucuscarotaRepID=Q4342
Aradu.FIU9K	Araip.30XFU	multiple-copy	DNA-bindingproteinn=1Tax=DaucuscarotaRepID=Q4342
Aradu.Z2BLJ	Araip.8505Z	multiple-copy	DNA-bindingproteinn=1Tax=DaucuscarotaRepID=Q4342

Aradu.IS6MU	Araip.3Z6CU	multiple-copy	DNA-bindingproteinRHL1,putative
Aradu.U808N	Araip.U1QS2	multiple-copy	DNA-bindingproteinSMUBP-2n=4Tax=UstilaginaceaeRep
Aradu.457AG	Araip.VT4Q1	multiple-copy	DNA-bindingstorekeeperprotein-relatedtranscriptionalregu
Aradu.FV8TW	Araip.AAM5W	multiple-copy	DNA-directedRNApolymerase
Aradu.EXQ60	Araip.NHV7F	multiple-copy	DNA-directedRNApolymerase
Aradu.V5V1W	Araip.2EA2I	multiple-copy	DNA-directedRNApolymerasefamilyprotein
Aradu.8Q4X6	Araip.AVR8T	multiple-copy	DNA-directedRNApolymerasefamilyprotein
Aradu.L6HBL	Araip.C2ULI	multiple-copy	DNA-directedRNApolymerasefamilyprotein
Aradu.DJF16	Araip.RSK88	multiple-copy	DNA-directedRNApolymerasefamilyprotein
Aradu.F9AQV	Araip.G8XIB	multiple-copy	DNA-directedRNApolymeraseI,II
Aradu.KZV99	Araip.J867Q	multiple-copy	DNA-directedRNApolymeraseI,II
Aradu.0FB9R	Araip.Q5FHC	multiple-copy	DNA-directedRNApolymeraseI,II
Aradu.I6I4A	Araip.13WQP	multiple-copy	DNA-directedRNApolymeraseII
Aradu.LT7X3	Araip.2H1ED	multiple-copy	DNA-directedRNApolymeraseII
Aradu.QI4L1	Araip.JM3P0	multiple-copy	DNA-directedRNApolymeraseII
Aradu.KEC4P	Araip.66GM5	multiple-copy	DNA-directedRNApolymeraseIIIprotein
Aradu.172F0	Araip.7Y0PA	multiple-copy	DNA-directedRNApolymeraseIIIsubunitRPC3-likeprotein
Aradu.8E8GF	Araip.AK5TZ	multiple-copy	DNA-directedRNApolymeraseIIIsubunitRPC5-likeprotein
Aradu.I9SPE	Araip.TJS96	multiple-copy	DNA-directedRNApolymeraseIIIsubunitRPC9-like[Glycine
Aradu.LDI0R	Araip.S5AR3	multiple-copy	DNA-directedRNApolymeraseIIsubunitRPB4n=82Tax=Eu
Aradu.U18XV	Araip.VXU4B	multiple-copy	DNA-directedRNApolymeraseIprotein
Aradu.4YA8F	Araip.Y9XZZ	multiple-copy	DNA-directedRNApolymeraseIsubunitrpa43-likeisoformX
Aradu.230WM	Araip.32U31	multiple-copy	DNA-directedRNApolymeraseIVsubunit1-likeisoformX1[G
Aradu.F1E13	Araip.C1EVE	multiple-copy	DNA-directedRNApolymerase-likeprotein
Aradu.8VP1A	Araip.4786J	multiple-copy	DNA-directedRNApolymerasesubunit
Aradu.NBQ7X	Araip.X6DID	multiple-copy	DNA-directedRNApolymerasesubunit
Aradu.C8SSW	Araip.PKK04	multiple-copy	DNA-directedRNApolymerasesubunitalpha
Aradu.1QU0K	Araip.T61X4	multiple-copy	DNAGYRASEB2
Aradu.R7CTG	Araip.3J01I	multiple-copy	DnaJ/Hsp40cysteine-richdomainsuperfamilyproteinisofor
Aradu.7YJ00	Araip.S9VNZ	multiple-copy	DnaJdomain
Aradu.97ZLB	Araip.68YDH	multiple-copy	DnaJheatshockamine-terminaldomainprotein
Aradu.SRY7A	Araip.Z6CDS	multiple-copy	DnaJheatshockamine-terminaldomainprotein
Aradu.MG836	Araip.Q2FZ0	multiple-copy	DNAJheatshockfamilyprotein
Aradu.DA0G4	Araip.6F52Y	multiple-copy	dnaJhomologsubfamilyBmember14-like[Glycinemax]
Aradu.870BG	Araip.L0ZBU	multiple-copy	dnaJhomologsubfamilyBmember14-like[Glycinemax]
Aradu.D05FL	Araip.F6TG6	multiple-copy	dnaJhomologsubfamilyBmember1-like[Glycinemax]
Aradu.QI7LX	Araip.L5QI4	multiple-copy	dnaJhomologsubfamilyBmember1-like[Glycinemax]
Aradu.W0FED	Araip.V4WLJ	multiple-copy	dnaJhomologsubfamilyBmember1-like[Glycinemax]
Aradu.J59GH	Araip.P3VNM	multiple-copy	DNAJheatshockN-terminaldomain-containingprotein
Aradu.N0WNK	Araip.P1PGD	multiple-copy	dnaJhomologsubfamilyCmember14-like[Glycinemax]
Aradu.4AK3M	Araip.E9N7G	multiple-copy	DNAJ-like20
Aradu.UY019	Araip.7NS74	multiple-copy	dnaJproteinhomolog2-like[Glycinemax]
Aradu.IL9WG	Araip.1UW8I	multiple-copy	DNAJhomologue3
Aradu.B0BG6	Araip.4UX45	multiple-copy	DNAJhomologue3

Aradu.YH413	Araip.AZN8Q	multiple-copy	DNAJhomologue3
Aradu.MA1DE	Araip.B0F5J	multiple-copy	DNAJhomologue3
Aradu.46TFL	Araip.GG6QM	multiple-copy	DNAJhomologue3
Aradu.BNM8A	Araip.Q4FLH	multiple-copy	DNAJ-like20
Aradu.C99J0	Araip.U1IEA	multiple-copy	DNAJhomologue3
Aradu.QF91Q	Araip.9B5MM	multiple-copy	DNAJ-like20
Aradu.Z194X	Araip.A74FX	multiple-copy	dnaJhomologsubfamilyCmember21-like[Glycinemax]
Aradu.N44D1	Araip.KI3IL	multiple-copy	DNAJ-like20
Aradu.R4H3N	Araip.4M6FA	multiple-copy	dnaJproteinhomolog1-like[Glycinemax]
Aradu.SUD1N	Araip.V76LN	multiple-copy	DNAmismatchrepairMUTSfamilyprotein
Aradu.QM4PI	Araip.9DP5A	multiple-copy	DNAmismatchrepairprotein,putative
Aradu.TVK0N	Araip.G7BAX	multiple-copy	DNAmismatchrepairproteinmsh6
Aradu.DRN6A	Araip.73385	multiple-copy	DNAmismatchrepairproteinMutS
Aradu.I567U	Araip.EU7DL	multiple-copy	DNAmismatchrepairproteinMutS
Aradu.CZD45	Araip.HQ7SW	multiple-copy	DNAmismatchrepairproteinMutS
Aradu.WVC4I	Araip.35KW5	multiple-copy	DNApolymerasedeltasubunit4
Aradu.VFE8Z	Araip.9S2X2	multiple-copy	DNApolymeraseIIIsubunitgamma/tau
Aradu.4I166	Araip.F53Z3	multiple-copy	DNApolymeraseIIIsubunitgamma/tau
Aradu.5M48C	Araip.F8718	multiple-copy	DNApolymeraseIIIsubunitgamma/tau
Aradu.G31YK	Araip.GVI8N	multiple-copy	DNApolymeraseI-likeprotein
Aradu.38ZXG	Araip.S7H0U	multiple-copy	DNApolymeraselambda-likeprotein
Aradu.83RRW	Araip.XL42X	multiple-copy	DNApolymerasesubunitCdc27
Aradu.7M4Z2	Araip.TMT3H	multiple-copy	DNApolymeraseVfamily
Aradu.6C6EU	Araip.678UJ	multiple-copy	DNArepair(Rad51)familyprotein
Aradu.8GK1W	Araip.0Y2KA	multiple-copy	DNArepairandrecombinationprotein
Aradu.MHS46	Araip.CGB26	multiple-copy	DNArepairandrecombinationRAD54-likeprotein
Aradu.FJQ80	Araip.S9NSP	multiple-copy	DNArepairendonucleaseUVH1-likeprotein
Aradu.Z83RP	Araip.Y7AG2	multiple-copy	DNArepairmetallo-beta-lactamasefamilyprotein
Aradu.VW4T4	Araip.UX0Q8	multiple-copy	DNArepairproteincomplementingxp-Ccells-likeprotein
Aradu.KL3C7	Araip.3BJ9Q	multiple-copy	DNArepairproteinREV1
Aradu.WT391	Araip.N1QWX	multiple-copy	DNArepairproteinXRCC1-liken=3Tax=OryzaRepID=Q5V
Aradu.A8KM2	Araip.M80H6	multiple-copy	DNArepairproteinXRCC2homologisoformX1[Glycinemax]
Aradu.0RA4R	Araip.JLZ8T	multiple-copy	DNAreplicationcomplexGINSproteinPSF1
Aradu.C00W8	Araip.HGE67	multiple-copy	DNAreplicationcomplexGINSSLD5-likeprotein
Aradu.R4MYV	Araip.ECF7H	multiple-copy	DNAreplicationfactorCcomplexsubunit1n=1Tax=Chlamyd
Aradu.A68AJ	Araip.0675S	multiple-copy	DNAreplicationfactorCDT1-likeprotein
Aradu.JF3WA	Araip.K79R5	multiple-copy	DNAreplicationlicensingfactorMCM2,putative
Aradu.X5WR6	Araip.Q6TYI	multiple-copy	DNAreplicationlicensingfactorMCM3homolog[Glycinema
Aradu.HY7QC	Araip.I4YCB	multiple-copy	DNAreplicationlicensingfactormcm5-A-like[Glycinemax]
Aradu.B3D9E	Araip.VJ6YA	multiple-copy	DNAreplicationlicensingfactormcm6[Glycinemax]
Aradu.56X7I	Araip.MEF1P	multiple-copy	DNAreplicationlicensingfactorMCM9n=19Tax=Phytophth
Aradu.K7TJG	Araip.7MS83	multiple-copy	Dof-typezincfingerDNA-bindingfamilyprotein
Aradu.PZ509	Araip.1P1MT	multiple-copy	DOFzincfingerprotein1
Aradu.ISN1L	Araip.1V5ZI	multiple-copy	DOFzincfingerprotein1

Aradu.J8H2F	Araip.EL37U	multiple-copy	DOFzincfingerprotein1
Aradu.1J1DY	Araip.H0H56	multiple-copy	DOFzincfingerprotein1
Aradu.W58GD	Araip.W28KY	multiple-copy	DOFzincfingerprotein1
Aradu.MS406	Araip.Z36KU	multiple-copy	DOFzincfingerprotein1
Aradu.08MEK	Araip.Z9VQ5	multiple-copy	DOFzincfingerprotein2
Aradu.YZ2FS	Araip.86DQ7	multiple-copy	DOFzincfingerprotein1
Aradu.DGR2N	Araip.RYZ75	multiple-copy	DOFzincfingerprotein2
Aradu.97XZI	Araip.8S6C1	multiple-copy	dofzincfingerproteinDOF3.2-like[Glycinemax]
Aradu.32ZUX	Araip.73IW9	multiple-copy	dofzincfingerproteinDOF5.1[Glycinemax]
Aradu.7E2JG	Araip.Z142E	multiple-copy	dofzincfingerproteinDOF5.6-like[Glycinemax]
Aradu.MRJ6D	Araip.A33WF	multiple-copy	dofzincfingerproteinDOF5.6[Glycinemax]
Aradu.BIZ8F	Araip.T3G5J	multiple-copy	dofzincfingerproteinDOF5.7-like[Glycinemax]
Aradu.4E354	Araip.K8DPE	multiple-copy	dolichyl-diphosphooligosaccharide--proteinglycosyltransfe
Aradu.250G2	Araip.FXZ9G	multiple-copy	dolichyl-diphosphooligosaccharide--proteinglycosyltransfe
Aradu.647P3	Araip.B71R1	multiple-copy	Domainofunknownfunction(DUF2431)
Aradu.VVR1U	Araip.EKA65	multiple-copy	DoubleClp-Nmotif-containingP-loopnucleosidetriphospha
Aradu.D8UDW	Araip.Y7NIY	multiple-copy	DoubleClp-Nmotif-containingP-loopnucleosidetriphospha
Aradu.E4IE7	Araip.BUW68	multiple-copy	double-strandedRNA-bindingmotifprotein
Aradu.WLS5Y	Araip.QT426	multiple-copy	double-strandedRNA-bindingprotein1-like[Glycinemax]
Aradu.MS08A	Araip.A18M2	multiple-copy	double-strandedRNA-specificadenosinedeaminase-likeis
Aradu.JV8RS	Araip.59ZNY	multiple-copy	downstreamtargetofAGL15-4protein,putative
Aradu.CWG39	Araip.GHT9B	multiple-copy	dualspecificityproteinphosphatase(DsPTP1)familyprotein
Aradu.CT2LH	Araip.1H1H0	multiple-copy	dualspecificityproteinphosphatase1
Aradu.75AY8	Araip.C1WRG	multiple-copy	dualspecificityproteinphosphatasefamilyprotein
Aradu.YLB4V	Araip.15K52	multiple-copy	dualspecificityproteinphosphatase-related
Aradu.15RGK	Araip.WG92W	multiple-copy	dualspecificityproteinphosphatase-related
Aradu.X49FH	Araip.MN0BK	multiple-copy	DUF21domainplantprotein
Aradu.YE41G	Araip.G3D9B	multiple-copy	DUF223domainprotein
Aradu.5R9X5	Araip.WU4P5	multiple-copy	DUF223domainprotein
Aradu.ND5YL	Araip.M8ZTC	multiple-copy	DUF2358familyprotein
Aradu.7I9FX	Araip.Q0TDL	multiple-copy	DUF247domainprotein
Aradu.UAL0U	Araip.UT9PH	multiple-copy	DUF247domainprotein
Aradu.Y43HK	Araip.WM0YD	multiple-copy	DUF247domainprotein
Aradu.13H1D	Araip.EU5DQ	multiple-copy	DUF309domainprotein
Aradu.E4G18	Araip.99548	multiple-copy	DUF3119familyprotein
Aradu.199G1	Araip.SJE6C	multiple-copy	DUF4228domainprotein
Aradu.XDC7C	Araip.6YP5U	multiple-copy	DUF4408domainprotein
Aradu.MZ9P5	Araip.BBI30	multiple-copy	DUF674familyprotein
Aradu.PP2S4	Araip.W5S9P	multiple-copy	DUF674familyprotein
Aradu.970W1	Araip.LQ4US	multiple-copy	DUF679domainmembraneprotein2
Aradu.63TDV	Araip.H81H9	multiple-copy	DUF740familyprotein
Aradu.Z030H	Araip.SQ1VV	multiple-copy	DWNNdomain,aCCHC-typezincfinger
Aradu.R0HWP	Araip.50K7X	multiple-copy	DYNAMIN-like1C
Aradu.6YX1J	Araip.D6GJ4	multiple-copy	Dynaminrelatedprotein5A

Aradu.KSK2P	Araip.HQ0LM	multiple-copy	Dyneinlightchaintype1familyprotein
Aradu.Q55UA	Araip.NG0LV	multiple-copy	Dyneinlightchaintype1familyprotein
Aradu.MSR4Q	Araip.VR494	multiple-copy	Dyneinlightchaintype1familyprotein
Aradu.V9VCU	Araip.WN95W	multiple-copy	Dyneinlightchaintype1familyprotein
Aradu.VW26U	Araip.091L0	multiple-copy	E2Ftranscriptionfactor3
Aradu.VSE1D	Araip.V48CN	multiple-copy	E2Ftranscriptionfactor3
Aradu.K3S1H	Araip.Y1XVN	multiple-copy	E2Ftranscriptionfactor3
Aradu.I8Q2P	Araip.8Z1AD	multiple-copy	E3Ubiquitinligasefamilyprotein
Aradu.EY5U3	Araip.FZ3D1	multiple-copy	E3ubiquitinligaseSCFcomplexsubunitSKP1/ASK1familypr
Aradu.JFR2C	Araip.F9RU1	multiple-copy	E3ubiquitin-proteinligase
Aradu.7AP79	Araip.09ESU	multiple-copy	E3ubiquitin-proteinligaseBAH1-likeprotein
Aradu.E0I63	Araip.G2F1A	multiple-copy	E3ubiquitin-proteinligaseBAH1-likeprotein,putative
Aradu.V2ISN	Araip.HNB3K	multiple-copy	E3ubiquitin-proteinligaseBOI-like[Glycinemax]
Aradu.IB4JW	Araip.7408P	multiple-copy	E3ubiquitin-proteinligaseBRE1-likeprotein
Aradu.LQK8F	Araip.D0AIB	multiple-copy	E3ubiquitin-proteinligaseCOP1-like[Glycinemax]
Aradu.XVQ98	Araip.G8VRW	multiple-copy	E3ubiquitin-proteinligaseCOP1-like[Glycinemax]
Aradu.X0EAG	Araip.P4BJL	multiple-copy	E3ubiquitin-proteinligaseCOP1-like[Glycinemax]
Aradu.749Z1	Araip.6AB1Y	multiple-copy	E3ubiquitinproteinligaseDRIP2-like[Glycinemax]
Aradu.FP0C1	Araip.GV7RQ	multiple-copy	E3ubiquitinproteinligaseDRIP2-like[Glycinemax]
Aradu.EQY7L	Araip.ZQ4EG	multiple-copy	E3ubiquitinproteinligaseDRIP2-like[Glycinemax]
Aradu.D4WQD	Araip.1Q1XN	multiple-copy	E3ubiquitin-proteinligaseLIN-1-likeisoformX4[Glycinemax
Aradu.C7AWI	Araip.7A4AR	multiple-copy	E3ubiquitin-proteinligaseLIN-1-likeisoformX3[Glycinemax
Aradu.1HQ4L	Araip.Q0N0W	multiple-copy	E3ubiquitin-proteinligaseLIN-likeisoformX2[Glycinemax]
Aradu.AQ0Z8	Araip.LB4BS	multiple-copy	E3ubiquitin-proteinligasen=5Tax=SolanumRepID=M1BD
Aradu.F2D3W	Araip.MSQ60	multiple-copy	E3ubiquitin-proteinligasen=5Tax=SolanumRepID=M1BD
Aradu.CV6Z5	Araip.P0NWF	multiple-copy	E3ubiquitin-proteinligasen=5Tax=SolanumRepID=M1BD
Aradu.67MY8	Araip.X1TUQ	multiple-copy	E3ubiquitin-proteinligasen=5Tax=SolanumRepID=M1BD
Aradu.M6IGU	Araip.8VU6U	multiple-copy	E3ubiquitin-proteinligasePUB23-like[Glycinemax]
Aradu.MA9YS	Araip.ADA7W	multiple-copy	E3ubiquitin-proteinligaseRF298-likeisoformX1[Glycinema
Aradu.2W21G	Araip.WF117	multiple-copy	E3ubiquitin-proteinligaseRF298-likeisoformX1[Glycinema
Aradu.E6ID0	Araip.977B6	multiple-copy	E3ubiquitin-proteinligaseRGLG2-likeisoformX2[Glycinem
Aradu.KR6YX	Araip.QL91M	multiple-copy	E3ubiquitin-proteinligaseRGLG2-likeisoformX6[Glycinem
Aradu.SY356	Araip.TU2YQ	multiple-copy	E3ubiquitin-proteinligaseRGLG2-likeisoformX1[Glycinem
Aradu.ZD4XA	Araip.XJC3R	multiple-copy	E3ubiquitin-proteinligaseRGLG2-likeisoformX4[Glycinem
Aradu.HU8I6	Araip.4C49Y	multiple-copy	E3ubiquitin-proteinligaseRHF2A-like[Glycinemax]
Aradu.K80ZI	Araip.43U91	multiple-copy	E3ubiquitinproteinligaseRIN2-likeisoformX3[Glycinemax]
Aradu.T1Q4B	Araip.HD55C	multiple-copy	E3ubiquitin-proteinligaseRING1-like[Glycinemax]
Aradu.I3I8S	Araip.125MX	multiple-copy	E3ubiquitin-proteinligasesynoviolin-likeisoformX1[Glycine
Aradu.L5NS4	Araip.9D8PC	multiple-copy	E3ubiquitin-proteinligaseUBR7isoformX3[Glycinemax]
Aradu.AU7A2	Araip.JVJ09	multiple-copy	E3ubiquitin-proteinligaseXBAT35-like[Glycinemax]
Aradu.Q5JK4	Araip.JYL2X	multiple-copy	EARLYFLOWERING3-likeisoformX2[Glycinemax]
Aradu.QC0V1	Araip.HEI1D	multiple-copy	EARLYFLOWERING3-likeisoformX3[Glycinemax]
Aradu.942ZP	Araip.Y4DBT	multiple-copy	earlylight-induced-likeprotein
Aradu.CRB6R	Araip.T3NCH	multiple-copy	earlynodulin-likeprotein13

Aradu.QED65	Araip.S2947	multiple-copy	earlynodulin-likeprotein2
Aradu.EWZ0P	Araip.T6R33	multiple-copy	earlynodulin-likeprotein2
Aradu.M0V1K	Araip.AL6IJ	multiple-copy	earlynodulin-likeprotein2-like[Glycinemax]
Aradu.JUW7B	Araip.K56MF	multiple-copy	earlynodulin-likeprotein2-like[Glycinemax]
Aradu.LC8HL	Araip.W0DHY	multiple-copy	earlynodulin-likeprotein3-like[Glycinemax]
Aradu.XN89N	Araip.K5Y49	multiple-copy	earlynodulin-likeprotein4
Aradu.M3LQ3	Araip.T7GHK	multiple-copy	earlynodulin-likeprotein9
Aradu.X9GQ3	Araip.1L9GW	multiple-copy	earlynodulin-related
Aradu.DV9UJ	Araip.52W96	multiple-copy	earlynodulin-related
Aradu.BV2Q9	Araip.CQ0DA	multiple-copy	earlynodulin-related
Aradu.1M38D	Araip.CW72W	multiple-copy	EFhandcalcium-bindingproteinfamily
Aradu.U1S9C	Araip.KKX96	multiple-copy	EIN3-bindingFboxprotein1
Aradu.XME24	Araip.H6J0Y	multiple-copy	ELF4-like4
Aradu.9FZ57	Araip.IEF2F	multiple-copy	elongationfactorTuGTP-bindingdomainprotein
Aradu.I977L	Araip.2R3UE	multiple-copy	elongatorprotein2
Aradu.G344I	Araip.32BSU	multiple-copy	embryodefective1923
Aradu.L3Q6E	Araip.V2691	multiple-copy	embryodefective2016
Aradu.V1PLQ	Araip.917K4	multiple-copy	embryodefective2735
Aradu.EW29C	Araip.3GQ6X	multiple-copy	EMBRYONICFLOWER1-likeisoformX2[Glycinemax]
Aradu.6M56A	Araip.ZZ3SQ	multiple-copy	embryo-specificprotein
Aradu.M0AKN	Araip.X6PEV	multiple-copy	emp24/gp25L/p24family/GOLDfamilyprotein
Aradu.HSJ3A	Araip.Y1ZJ1	multiple-copy	emp24/gp25L/p24family/GOLDfamilyprotein
Aradu.LL82A	Araip.KX93R	multiple-copy	EmsyNTerminus(ENT)/plantTudor-likedomains-containin[illegible]
Aradu.EX4VM	Araip.MY3WG	multiple-copy	EmsyNTerminus(ENT)/plantTudor-likedomains-containin[illegible]
Aradu.9G7GM	Araip.XM65N	multiple-copy	endoglucanase10-like[Glycinemax]
Aradu.FV1SH	Araip.32YK0	multiple-copy	endoglucanase11[Glycinemax]
Aradu.WLD4B	Araip.R3KEZ	multiple-copy	endoglucanase17[Glycinemax]
Aradu.SW5JR	Araip.K9VA5	multiple-copy	endoglucanase17-like[Glycinemax]
Aradu.EX9SJ	Araip.K24FA	multiple-copy	endoglucanase24-like[Glycinemax]
Aradu.1AM05	Araip.ZXR6U	multiple-copy	endoglucanase24-like[Glycinemax]
Aradu.IV87Z	Araip.B0DA9	multiple-copy	endoglucanase25-like[Glycinemax]
Aradu.69HMZ	Araip.GFC8K	multiple-copy	endoglucanase25-like[Glycinemax]
Aradu.FU781	Araip.Y92IB	multiple-copy	endoglucanase25-like[Glycinemax]
Aradu.L1447	Araip.L55CJ	multiple-copy	endoglucanase8-like[Glycinemax]
Aradu.DD097	Araip.YU7BP	multiple-copy	endoglucanase8-like[Glycinemax]
Aradu.LZ99D	Araip.FK4XP	multiple-copy	endonuclease/exonuclease/phosphatasefamilyprotein
Aradu.61Y68	Araip.HG5A7	multiple-copy	endonuclease/exonuclease/phosphatasefamilyprotein
Aradu.Z0FA2	Araip.Z254D	multiple-copy	endonuclease/exonuclease/phosphatasefamilyprotein
Aradu.ZFW51	Araip.VS416	multiple-copy	endonucleaseorglycosylhydrolase
Aradu.8II80	Araip.Z789E	multiple-copy	enhancerofpolycomb-liketranscriptionfactorprotein
Aradu.R8RVW	Araip.71KZF	multiple-copy	enoyl-CoAhydratase/isomeraseD
Aradu.3DX6E	Araip.BT3CW	multiple-copy	enoyl-CoAhydratase/isomeraseA
Aradu.JMZ0B	Araip.WUY3S	multiple-copy	enoyl-CoAhydratase/isomerasefamilyprotein
Aradu.G676K	Araip.W8N64	multiple-copy	ENTH/VHS/GATfamilyprotein

Aradu.01VAS	Araip.WE2GD	multiple-copy	ENTH/VHS/GATfamilyprotein
Aradu.NX4M4	Araip.X7P83	multiple-copy	ENTH/VHS/GATfamilyprotein
Aradu.HN7R4	Araip.B03U9	multiple-copy	EPIDERMALPATTERNINGFACTOR-likeprotein2-like[Gly
Aradu.2AQ5X	Araip.BYN6L	multiple-copy	EPIDERMALPATTERNINGFACTOR-likeprotein2-like[Gly
Aradu.F4S3N	Araip.Q083N	multiple-copy	EPIDERMALPATTERNINGFACTOR-likeprotein2-like[Gly
Aradu.87FCC	Araip.AS26T	multiple-copy	EPIDERMALPATTERNINGFACTOR-likeprotein4-like[Gly
Aradu.E26HT	Araip.E9DE0	multiple-copy	epoxidehydrolase
Aradu.LN5U5	Araip.EV4MA	multiple-copy	epoxidehydrolase
Aradu.PV1T4	Araip.G38PQ	multiple-copy	epoxidehydrolase
Aradu.V0C1K	Araip.J5QMQ	multiple-copy	epoxidehydrolase
Aradu.P3VBU	Araip.73614	multiple-copy	epoxidehydrolase-likeprotein
Aradu.UV1C8	Araip.N517Q	multiple-copy	epsin-3-like[Glycinemax]
Aradu.K3XG1	Araip.0DS8L	multiple-copy	equilibrativenucleosidetransporter7
Aradu.E01TZ	Araip.M7QQB	multiple-copy	ERD(early-responsivetodehydrationstress)familyprotein
Aradu.BF8QQ	Araip.Q47NS	multiple-copy	ERD(early-responsivetodehydrationstress)familyprotein
Aradu.QT3HF	Araip.UBX9E	multiple-copy	ERD(early-responsivetodehydrationstress)familyprotein
Aradu.PKA69	Araip.VA9R0	multiple-copy	ERD(early-responsivetodehydrationstress)familyprotein
Aradu.EA94B	Araip.YQA7J	multiple-copy	ERD(early-responsivetodehydrationstress)familyprotein
Aradu.Q97KS	Araip.LXC6U	multiple-copy	ERlumenproteinretainingreceptorfamilyprotein
Aradu.9MA52	Araip.RW9NX	multiple-copy	ERlumenproteinretainingreceptorfamilyprotein
Aradu.9E473	Araip.7V7BF	multiple-copy	ERmembraneproteincomplexsubunit6n=10Tax=Eutheria
Aradu.M1XBW	Araip.4GI7N	multiple-copy	ERmembraneproteincomplexsubunit-likeprotein
Aradu.SP6QR	Araip.FL27H	multiple-copy	Erythronate-4-phosphatedehydrogenasefamilyprotein
Aradu.46FZZ	Araip.WZ3EA	multiple-copy	Erythronate-4-phosphatedehydrogenasefamilyprotein
Aradu.MBB01	Araip.YX2BB	multiple-copy	Ethyleneinsensitive3familyprotein
Aradu.JJA0L	Araip.K3GK4	multiple-copy	ethylene-responsivetranscriptionfactorLEP-like[Glycinem
Aradu.9SR9S	Araip.6ZJ3N	multiple-copy	ethylene-responsivetranscriptionfactor13-like[Glycinema
Aradu.GB4U4	Araip.3JJ8N	multiple-copy	ethylene-responsivetranscriptionfactor1A-like[Glycinema
Aradu.LD7BF	Araip.B5DCI	multiple-copy	ethylene-responsivetranscriptionfactor1A-like[Glycinema
Aradu.I9TTZ	Araip.62IDN	multiple-copy	ethylene-responsivetranscriptionfactor1B
Aradu.00XL9	Araip.E2MIX	multiple-copy	ethylene-responsivetranscriptionfactor1B
Aradu.3UE4N	Araip.F3QBW	multiple-copy	ethylene-responsivetranscriptionfactor1B
Aradu.83F0C	Araip.KY0TY	multiple-copy	ethylene-responsivetranscriptionfactor1B
Aradu.NZ8CP	Araip.LJJ47	multiple-copy	ethylene-responsivetranscriptionfactor1B
Aradu.B90GQ	Araip.T3D3V	multiple-copy	ethylene-responsivetranscriptionfactor1B
Aradu.Y3BW3	Araip.ZCG46	multiple-copy	ethylene-responsivetranscriptionfactor1B
Aradu.3Q82R	Araip.IN0FT	multiple-copy	ethylene-responsivetranscriptionfactor3[Glycinemax]
Aradu.14Z9Y	Araip.KAK82	multiple-copy	ethylene-responsivetranscriptionfactor3-like[Glycinemax]
Aradu.MIW9U	Araip.S5ATW	multiple-copy	ethylene-responsivetranscriptionfactor3[Glycinemax]
Aradu.W4PMX	Araip.Y9RA8	multiple-copy	ethylene-responsivetranscriptionfactor7-like[Glycinemax]
Aradu.P6UBG	Araip.S3MIZ	multiple-copy	ethylene-responsivetranscriptionfactor4[Glycinemax]
Aradu.E2TII	Araip.AKR06	multiple-copy	ethylene-responsivetranscriptionfactor4-like[Glycinemax]
Aradu.88AFG	Araip.K8KF4	multiple-copy	ethylene-responsivetranscriptionfactor4-like[Glycinemax]
Aradu.IRG5E	Araip.9R7P2	multiple-copy	ethylene-responsivetranscriptionfactor5[Glycinemax]

Aradu.PBM8D	Araip.RQ5W2	multiple-copy	ethylene-responsivetranscriptionfactor1B
Aradu.470B5	Araip.N6IZA	multiple-copy	ethylene-responsivetranscriptionfactor5-like[Glycinemax]
Aradu.83Z01	Araip.P8G5K	multiple-copy	ethylene-responsivetranscriptionfactor5-like[Glycinemax]
Aradu.3P75R	Araip.B2ESJ	multiple-copy	ethylene-responsivetranscriptionfactor7-like[Glycinemax]
Aradu.D814J	Araip.XMM29	multiple-copy	ethylene-responsivetranscriptionfactor3-like[Glycinemax]
Aradu.SC4Y7	Araip.S1N8M	multiple-copy	ethylene-responsivetranscriptionfactor-likeproteinAt4g13[illegible]
Aradu.G5NTR	Araip.B5WG1	multiple-copy	ethylene-responsivetranscriptionfactorRAP2-7-likeisoform[illegible]
Aradu.ZIC37	Araip.G1V85	multiple-copy	ETO1-likeprotein1-likeisoformX6[Glycinemax]
Aradu.HR4SC	Araip.M1YMA	multiple-copy	evolutionarilyconservedC-terminalregion2
Aradu.C4Y83	Araip.VNV0X	multiple-copy	evolutionarilyconservedC-terminalregion5
Aradu.9VQ7B	Araip.G4JRD	multiple-copy	evolutionarilyconservedC-terminalregion7
Aradu.ATH33	Araip.TX4H4	multiple-copy	EXECUTER1,chloroplastic-like[Glycinemax]
Aradu.97X4A	Araip.DPR39	multiple-copy	exocystcomplexcomponentsec15A
Aradu.U0V69	Araip.P67TL	multiple-copy	ExocystcomplexcomponentSEC5
Aradu.I2DKG	Araip.H5Y6J	multiple-copy	exocystsubunitexo70familyproteinB1
Aradu.86BB6	Araip.ZP5QD	multiple-copy	exocystsubunitexo70familyproteinB1
Aradu.8V3QK	Araip.94DKY	multiple-copy	exocystsubunitexo70familyproteinC2
Aradu.X53KQ	Araip.L66A6	multiple-copy	exocystsubunitexo70familyproteinG1
Aradu.0VP21	Araip.Q56KQ	multiple-copy	exocystsubunitexo70familyproteinG1
Aradu.AU74Y	Araip.XS9S4	multiple-copy	exocystsubunitexo70familyproteinH7
Aradu.0N6WQ	Araip.ACR9K	multiple-copy	exonucleasedomain-containingproteinAt3g15140-likeiso[illegible]
Aradu.D2MQU	Araip.6983Z	multiple-copy	Exonucleasefamilyprotein
Aradu.GR7RL	Araip.STI4D	multiple-copy	Exonucleasefamilyprotein
Aradu.EI69K	Araip.6E9J8	multiple-copy	exosomecomplexexonucleaseRRP44
Aradu.WUP1N	Araip.3I7ZD	multiple-copy	ExosomecomplexexonucleaseRRP45n=3Tax=Papilionoi[illegible]
Aradu.G5WQA	Araip.DXE3Z	multiple-copy	exosomecomponent10-likeisoformX1[Glycinemax]
Aradu.PYG3I	Araip.7D3J7	multiple-copy	Exostosinfamilyprotein
Aradu.531ZK	Araip.FH86S	multiple-copy	exostosinfamilyprotein
Aradu.L7ZWM	Araip.Q0SP8	multiple-copy	Exostosinfamilyprotein
Aradu.CS1KJ	Araip.C0N7L	multiple-copy	expansinB3
Aradu.83MPA	Araip.CB643	multiple-copy	expansinB3
Aradu.MIM9N	Araip.CP6UC	multiple-copy	expansin-likeB1
Aradu.0QC7R	Araip.U67Y5	multiple-copy	expansin-likeB1
Aradu.KH5IZ	Araip.Y0GXG	multiple-copy	expansin-likeB1
Aradu.F00XS	Araip.TV66E	multiple-copy	expansin-likeB1-like[Glycinemax]
Aradu.G5CNQ	Araip.VW462	multiple-copy	exportin1A
Aradu.KVD8P	Araip.82I4N	multiple-copy	exportin-4protein,putative
Aradu.251QA	Araip.N3G6M	multiple-copy	externalalternativeNAD(P)H-ubiquinoneoxidoreductaseB[illegible]
Aradu.6896W	Araip.SX8DW	multiple-copy	extra-largeguaninenucleotide-bindingprotein1-like[Glycin[illegible]
Aradu.R4ZCN	Araip.XX28B	multiple-copy	extra-largeguaninenucleotide-bindingprotein1-like[Glycin[illegible]
Aradu.2FM0G	Araip.A674Q	multiple-copy	FAD/NAD(P)-bindingoxidoreductasefamilyprotein
Aradu.8T3G1	Araip.UUZ6A	multiple-copy	FAD/NAD(P)-bindingoxidoreductasefamilyprotein
Aradu.4X1GI	Araip.I4CPS	multiple-copy	FAD-bindingBerberinefamilyprotein
Aradu.UT1SE	Araip.RE8JQ	multiple-copy	FAD-bindingBerberinefamilyprotein

Aradu.642R3	Araip.UB5VN	multiple-copy	FAD-bindingBerberinefamilyprotein
Aradu.26N74	Araip.VB5WD	multiple-copy	FAD-bindingBerberinefamilyprotein
Aradu.G6CRU	Araip.ZD8Z8	multiple-copy	FAD-bindingBerberinefamilyprotein
Aradu.YPX3B	Araip.ZW2KS	multiple-copy	FAD-bindingBerberinefamilyprotein
Aradu.S0XAG	Araip.P5CS5	multiple-copy	FAD-dependentoxidoreductasefamilyprotein
Aradu.H33XQ	Araip.AV6R9	multiple-copy	FAR1-relatedsequence11
Aradu.W5JRX	Araip.GZM57	multiple-copy	FAR1-RELATEDSEQUENCE12-likeisoformX1[Glycinema
Aradu.KG94L	Araip.27EE4	multiple-copy	FAR1-RELATEDSEQUENCE2-likeisoformX3[Glycinemax
Aradu.8P3KF	Araip.S802V	multiple-copy	FAR1-RELATEDSEQUENCE2-likeisoformX3[Glycinema
Aradu.PA2UE	Araip.HB5N9	multiple-copy	FAR1-RELATEDSEQUENCE3-likeisoformX1[Glycinemax
Aradu.G1RFE	Araip.6AX31	multiple-copy	FAR1-RELATEDSEQUENCE3-likeisoformX1[Glycinema
Aradu.6DL9M	Araip.K5RKY	multiple-copy	FAR1-RELATEDSEQUENCE3-likeisoformX2[Glycinemax
Aradu.SF38I	Araip.L2AZX	multiple-copy	FAR1-RELATEDSEQUENCE3-likeisoformX2[Glycinema
Aradu.99PKX	Araip.T2XRD	multiple-copy	FAR1-RELATEDSEQUENCE3-likeisoformX2[Glycinemax
Aradu.J4SMU	Araip.ZEC5D	multiple-copy	FAR1-RELATEDSEQUENCE3-likeisoformX1[Glycinema
Aradu.1I5W4	Araip.M4D5S	multiple-copy	FAR1-RELATEDSEQUENCE4-likeisoformX1[Glycinemax
Aradu.EAF6N	Araip.43ZG0	multiple-copy	FAR1-RELATEDSEQUENCE5-like[Glycinemax]
Aradu.U1T2G	Araip.9JH6T	multiple-copy	FAR1-RELATEDSEQUENCE5-like[Glycinemax]
Aradu.JR4VV	Araip.H5UU3	multiple-copy	FAR1-RELATEDSEQUENCE5-like[Glycinemax]
Aradu.CG9KF	Araip.LCN5D	multiple-copy	FAR1-RELATEDSEQUENCE5-like[Glycinemax]
Aradu.K0FA0	Araip.MQ9VE	multiple-copy	FAR1-RELATEDSEQUENCE5-like[Glycinemax]
Aradu.QPZ5M	Araip.P3SXN	multiple-copy	FAR1-RELATEDSEQUENCE5-like[Glycinemax]
Aradu.19EUZ	Araip.C9I3T	multiple-copy	FAR1-RELATEDSEQUENCE5-likeisoformX2[Glycinema
Aradu.IUI2U	Araip.FW1RB	multiple-copy	FAR1-RELATEDSEQUENCE5-like[Glycinemax]
Aradu.WAT9N	Araip.MZJ7P	multiple-copy	FAR1-RELATEDSEQUENCE6-likeisoform1[Glycinemax]
Aradu.RW9W8	Araip.TY0LX	multiple-copy	FAR1-RELATEDSEQUENCE6-likeisoform1[Glycinemax]
Aradu.IHD20	Araip.3280G	multiple-copy	FAR1-RELATEDSEQUENCE6-likeisoformX2[Glycinema
Aradu.A984U	Araip.AU5HG	multiple-copy	FAR1-RELATEDSEQUENCE6-likeisoformX2[Glycinemax
Aradu.XYA4T	Araip.EDH29	multiple-copy	FAR1-RELATEDSEQUENCE6-likeisoformX2[Glycinema
Aradu.8WJ4P	Araip.HIE0T	multiple-copy	FAR1-RELATEDSEQUENCE6-likeisoformX2[Glycinema
Aradu.K9316	Araip.LN9X0	multiple-copy	FAR1-RELATEDSEQUENCE6-likeisoformX2[Glycinema
Aradu.JQ7DF	Araip.U25LN	multiple-copy	FAR1-RELATEDSEQUENCE6-likeisoformX2[Glycinema
Aradu.AFB92	Araip.H0PL5	multiple-copy	FAR-REDimpairedresponse-like
Aradu.6R4YC	Araip.B3PLA	multiple-copy	FAR1-RELATEDSEQUENCE5-like[Glycinemax]
Aradu.PIU2J	Araip.2B2WU	multiple-copy	FAR1-RELATEDSEQUENCE9-likeisoformX5[Glycinema
Aradu.13E7W	Araip.A6A17	multiple-copy	FAR1-RELATEDSEQUENCE9-likeisoformX5[Glycinema
Aradu.NC1XP	Araip.BK4FM	multiple-copy	FAR1-RELATEDSEQUENCE9-likeisoformX5[Glycinema
Aradu.MH3SJ	Araip.P5W3N	multiple-copy	FAR1-RELATEDSEQUENCE9-likeisoformX5[Glycinema
Aradu.M0JAP	Araip.S1S60	multiple-copy	FAR1-RELATEDSEQUENCE9-likeisoformX5[Glycinema
Aradu.94JTX	Araip.W1Z8X	multiple-copy	Fasciclin-likearabinogalactanfamilyprotein
Aradu.I4TMG	Araip.EG329	multiple-copy	FASCICLIN-likearabinogalactan-protein12
Aradu.RV5XK	Araip.X7Z10	multiple-copy	FASCICLIN-likearabinogalactanprotein15precursor
Aradu.ME00T	Araip.R0VZ6	multiple-copy	FASCICLIN-likearabinogalactanprotein16precursor
Aradu.RI35R	Araip.VQ8DT	multiple-copy	FASCICLIN-likearabinogalactanprotein16precursor

Aradu.14ANW	Araip.TP6JV	multiple-copy	fasciclin-likearabinogalactanprotein21-like[Glycinemax]
Aradu.GN16C	Araip.R99K0	multiple-copy	fattyacidamidehydrolase-like[Glycinemax]
Aradu.I67U0	Araip.CGW17	multiple-copy	fattyaciddesaturase8
Aradu.P7XEX	Araip.M4FLV	multiple-copy	Fattyacidhydroxylasesuperfamily
Aradu.UE0PI	Araip.K91M4	multiple-copy	fattyacyl-CoAreductase3-like[Glycinemax]
Aradu.AU8EP	Araip.QT6UY	multiple-copy	fattyacyl-CoAreductase3-like[Glycinemax]
Aradu.X8GPB	Araip.26EKA	multiple-copy	F-box/kelch-repeatplantprotein
Aradu.G0DW8	Araip.95R25	multiple-copy	F-box/kelch-repeatplantprotein
Aradu.WRB0S	Araip.Z9GTK	multiple-copy	F-box/kelch-repeatproteinAt1g51550-like[Glycinemax]
Aradu.JMQ30	Araip.J60GN	multiple-copy	F-box/LRRplantprotein
Aradu.MR5GS	Araip.G32SV	multiple-copy	F-box/LRRprotein
Aradu.GVV0Z	Araip.IN1U1	multiple-copy	F-box/LRRprotein
Aradu.64V56	Araip.JT9BC	multiple-copy	F-box/LRRprotein
Aradu.I05PE	Araip.TID01	multiple-copy	F-box/LRRprotein
Aradu.0M33I	Araip.0J2TC	multiple-copy	F-box/LRR-repeatprotein13-likeisoformX2[Glycinemax]
Aradu.1Z7PM	Araip.3JH87	multiple-copy	F-box/LRR-repeatprotein13-likeisoformX2[Glycinemax]
Aradu.HKL6E	Araip.1UK4V	multiple-copy	F-box/LRR-repeatprotein15-like[Glycinemax]
Aradu.4ER97	Araip.7G8YS	multiple-copy	F-box/LRR-repeatprotein17-like[Glycinemax]
Aradu.GRE2M	Araip.TJ7IP	multiple-copy	F-box/LRR-repeatproteinAt3g48880-likeisoformX3[Glycin
Aradu.GP6HQ	Araip.85R0G	multiple-copy	F-box/RNI-likesuperfamilyprotein
Aradu.7H8MA	Araip.30QVU	multiple-copy	F-box/RNI-likesuperfamilyprotein
Aradu.C665I	Araip.4J570	multiple-copy	F-box/RNI-likesuperfamilyprotein
Aradu.TR2FS	Araip.4NI21	multiple-copy	F-boxfamilyprotein
Aradu.N7MUQ	Araip.9RR7M	multiple-copy	F-box/RNI-likesuperfamilyprotein
Aradu.C824H	Araip.B68IR	multiple-copy	F-box/RNI-likesuperfamilyprotein
Aradu.8HB43	Araip.BXY3B	multiple-copy	F-box/RNI-likesuperfamilyprotein
Aradu.K4111	Araip.C2X2S	multiple-copy	F-box/RNI-likesuperfamilyprotein
Aradu.L8GUY	Araip.F7L5Y	multiple-copy	F-box/RNI-likesuperfamilyprotein
Aradu.HSB55	Araip.H11CF	multiple-copy	F-box/RNI-likesuperfamilyprotein
Aradu.HNV5C	Araip.LEK47	multiple-copy	F-box/RNI-likesuperfamilyprotein
Aradu.T9X3T	Araip.ST9EM	multiple-copy	F-box/RNI-likesuperfamilyprotein
Aradu.A4FGM	Araip.U68SV	multiple-copy	F-box/RNI-likesuperfamilyprotein
Aradu.HKL1A	Araip.WE8XB	multiple-copy	F-box/RNI-likesuperfamilyprotein
Aradu.P56V8	Araip.WZ7IP	multiple-copy	F-box/RNI-likesuperfamilyprotein
Aradu.U9CZ6	Araip.591ZY	multiple-copy	F-boxandassociatedinteractiondomains-containingprotein
Aradu.M9YMG	Araip.8XX1S	multiple-copy	F-boxandassociatedinteractiondomains-containingprotein
Aradu.6VU3X	Araip.B3R7B	multiple-copy	F-boxandassociatedinteractiondomains-containingprotein
Aradu.VM6CH	Araip.H9AVS	multiple-copy	F-boxandassociatedinteractiondomains-containingprotein
Aradu.7Z549	Araip.UTX5W	multiple-copy	F-boxandassociatedinteractiondomains-containingprotein
Aradu.B3Z15	Araip.6N7BK	multiple-copy	F-boxandJmjCdomainproteinn=3Tax=AspergillusRepID=
Aradu.P3YYJ	Araip.TGT3S	multiple-copy	F-boxassociatedubiquitinationeffector-likeprotein
Aradu.NQ8H0	Araip.0E8VY	multiple-copy	F-boxfamilyprotein
Aradu.YXH0F	Araip.0X2PV	multiple-copy	F-boxfamilyprotein
Aradu.G76IS	Araip.26B8A	multiple-copy	F-boxfamilyprotein

Aradu.RLZ61	Araip.3X35T	multiple-copy	F-boxfamilyprotein
Aradu.M49FB	Araip.41EGI	multiple-copy	F-boxfamilyprotein
Aradu.QT3H3	Araip.5JT26	multiple-copy	F-boxfamilyprotein
Aradu.HMN77	Araip.615VA	multiple-copy	F-boxproteininteractiondomainprotein
Aradu.902BX	Araip.645I6	multiple-copy	F-boxfamilyprotein
Aradu.QZD25	Araip.7D96E	multiple-copy	F-boxfamilyprotein
Aradu.7J78G	Araip.A7ZNN	multiple-copy	F-boxfamilyprotein
Aradu.62T99	Araip.AEP3U	multiple-copy	F-boxfamilyprotein
Aradu.TL8H2	Araip.D2LW3	multiple-copy	F-boxfamilyprotein
Aradu.6T9HL	Araip.DTM34	multiple-copy	F-boxfamilyprotein
Aradu.F3745	Araip.G3RW4	multiple-copy	F-boxfamilyprotein
Aradu.B2YUE	Araip.IZW7J	multiple-copy	F-boxfamilyprotein
Aradu.88EGA	Araip.JFW8D	multiple-copy	F-boxfamilyprotein
Aradu.HYT44	Araip.K5S58	multiple-copy	F-boxfamilyprotein
Aradu.BM68M	Araip.LL92K	multiple-copy	F-boxfamilyprotein
Aradu.SJ15T	Araip.NK3C5	multiple-copy	F-boxfamilyprotein
Aradu.6L9XG	Araip.PK0AD	multiple-copy	F-boxfamilyprotein
Aradu.F591F	Araip.W8SB4	multiple-copy	F-boxfamilyprotein
Aradu.3MT4E	Araip.X3LMP	multiple-copy	F-boxfamilyprotein
Aradu.EQ6UY	Araip.XVW43	multiple-copy	F-boxfamilyprotein
Aradu.30RBD	Araip.1HR72	multiple-copy	F-box-likeprotein
Aradu.KW737	Araip.J7Z63	multiple-copy	F-box-likeprotein
Aradu.CX1PI	Araip.D1J6I	multiple-copy	F-boxprotein
Aradu.39CFF	Araip.I8JAN	multiple-copy	F-boxprotein
Aradu.R2RMR	Araip.UM7ZN	multiple-copy	F-boxfamilyprotein
Aradu.8G40J	Araip.Y1R86	multiple-copy	F-boxprotein7
Aradu.VM1W8	Araip.EZS4N	multiple-copy	F-boxproteinAt1g10780-like[Glycinemax]
Aradu.V92BV	Araip.F6G6S	multiple-copy	F-boxproteinAt4g02760-likeisoformX1[Glycinemax]
Aradu.0Y99B	Araip.8Z53B	multiple-copy	F-boxproteininteractiondomainprotein
Aradu.W1X0Q	Araip.9TJ4Z	multiple-copy	F-boxproteininteractiondomainprotein
Aradu.FJ6K3	Araip.DK7U4	multiple-copy	F-boxproteininteractiondomainprotein
Aradu.NIH31	Araip.I19HU	multiple-copy	F-boxfamilyprotein
Aradu.R5Y58	Araip.IRU7H	multiple-copy	F-boxproteininteractiondomainprotein
Aradu.QH5RZ	Araip.IV7HZ	multiple-copy	F-boxproteininteractiondomainprotein
Aradu.CF5Z2	Araip.K3GN3	multiple-copy	F-boxandassociatedinteractiondomains-containingprotei
Aradu.AZF5J	Araip.SZQ74	multiple-copy	F-boxproteininteractiondomainprotein
Aradu.70QSY	Araip.DKH8U	multiple-copy	F-boxproteinPP2-A13
Aradu.4635I	Araip.ZP3NK	multiple-copy	F-boxproteinPP2-A13
Aradu.V8GHA	Araip.H7YH7	multiple-copy	F-boxproteinSKIP14-like[Glycinemax]
Aradu.MS621	Araip.I2I77	multiple-copy	F-boxproteinSKIP14-like[Glycinemax]
Aradu.PP2DC	Araip.L9YEX	multiple-copy	F-boxproteinSKIP16
Aradu.A1EFP	Araip.3MN07	multiple-copy	F-boxproteinSKIP2-like[Glycinemax]
Aradu.EZ7WI	Araip.23LH0	multiple-copy	F-boxproteinSKIP31-like[Glycinemax]
Aradu.Z2SQB	Araip.9W150	multiple-copy	ferredoxin3

Aradu.J8MWU	Araip.VR4Q2	multiple-copy	Ferredoxin-NADPreductasefamilyproteinn=2Tax=Populus
Aradu.29VJC	Araip.8AR8R	multiple-copy	ferredoxin-related
Aradu.92ILA	Araip.HN6Y6	multiple-copy	ferritin2
Aradu.HFI9K	Araip.LL8C3	multiple-copy	ferritin4
Aradu.N8FJN	Araip.RJ07Z	multiple-copy	ferritin4
Aradu.7I425	Araip.A22ZE	multiple-copy	fiberproteinFb34
Aradu.E3BEW	Araip.WK3TC	multiple-copy	fiberproteinFb34
Aradu.3TJ8Z	Araip.LCL9M	multiple-copy	filament-likeplantprotein4-likeisoformX2[Glycinemax]
Aradu.N9KIE	Araip.VZ9BG	multiple-copy	filament-likeplantprotein4-likeisoformX2[Glycinemax]
Aradu.MEY8C	Araip.SGQ01	multiple-copy	filament-likeplantprotein7-likeisoformX1[Glycinemax]
Aradu.BV4I8	Araip.FR6UM	multiple-copy	filament-likeplantprotein-likeisoformX3[Glycinemax]
Aradu.QZ3JB	Araip.T2U1L	multiple-copy	fission1protein
Aradu.R5E06	Araip.6L5T3	multiple-copy	FKBP-likepeptidyl-prolylcis-transisomerasefamilyprotein
Aradu.BR0NQ	Araip.CXD1I	multiple-copy	FKBP-likepeptidyl-prolylcis-transisomerasefamilyprotein
Aradu.K285D	Araip.EZ6WD	multiple-copy	FKBP-likepeptidyl-prolylcis-transisomerasefamilyprotein
Aradu.FD8VQ	Araip.F4F1R	multiple-copy	FKBP-likepeptidyl-prolylcis-transisomerasefamilyprotein
Aradu.33G7Z	Araip.GE4SS	multiple-copy	FKBP-likepeptidyl-prolylcis-transisomerasefamilyprotein
Aradu.GY0R3	Araip.GJ5QE	multiple-copy	FKBP-likepeptidyl-prolylcis-transisomerasefamilyprotein
Aradu.JU778	Araip.IIL5I	multiple-copy	FKBP-likepeptidyl-prolylcis-transisomerasefamilyprotein
Aradu.D66VA	Araip.J3KIF	multiple-copy	FKBP-likepeptidyl-prolylcis-transisomerasefamilyprotein
Aradu.Z1H9W	Araip.JA6AC	multiple-copy	FKBP-likepeptidyl-prolylcis-transisomerasefamilyprotein
Aradu.AN363	Araip.Q99H0	multiple-copy	FKBP-likepeptidyl-prolylcis-transisomerasefamilyprotein
Aradu.E37Q4	Araip.TCC2K	multiple-copy	FKBP-likepeptidyl-prolylcis-transisomerasefamilyprotein
Aradu.BUC40	Araip.X7VQF	multiple-copy	FKBP-likepeptidyl-prolylcis-transisomerasefamilyprotein
Aradu.PHE1E	Araip.ZE0AY	multiple-copy	FKBP-likepeptidyl-prolylcis-transisomerasefamilyprotein
Aradu.Q14N7	Araip.44WZX	multiple-copy	Flavin-bindingmonooxygenasefamilyprotein
Aradu.NYS45	Araip.C1JFS	multiple-copy	Flavin-bindingmonooxygenasefamilyprotein
Aradu.KF6LM	Araip.H3PA0	multiple-copy	Flavin-bindingmonooxygenasefamilyprotein
Aradu.D12DR	Araip.MQU8W	multiple-copy	Flavin-bindingmonooxygenasefamilyprotein
Aradu.H1RA6	Araip.NCY17	multiple-copy	Flavin-bindingmonooxygenasefamilyprotein
Aradu.M1252	Araip.R2UBA	multiple-copy	Flavin-bindingmonooxygenasefamilyprotein
Aradu.Z5LJB	Araip.VSP1L	multiple-copy	Flavin-bindingmonooxygenasefamilyprotein
Aradu.80EYC	Araip.YPF2X	multiple-copy	Flavin-bindingmonooxygenasefamilyprotein
Aradu.R2M0E	Araip.21220	multiple-copy	Flavincontainingamineoxidoreductasefamily
Aradu.HD203	Araip.VC101	multiple-copy	flavonolsynthase[Glycinemax]
Aradu.ZY67R	Araip.11CVG	multiple-copy	flocculationproteinFLO11-likeisoformX1[Glycinemax]
Aradu.634DQ	Araip.1D0FW	multiple-copy	flocculationproteinFLO11-likeisoformX3[Glycinemax]
Aradu.VA1N9	Araip.5DF3C	multiple-copy	flocculationproteinFLO11-likeisoformX3[Glycinemax]
Aradu.F950M	Araip.344D4	multiple-copy	floweringlocusproteinT
Aradu.P7QP3	Araip.A5PDN	multiple-copy	floweringlocusproteinT
Aradu.QIZ46	Araip.A8S33	multiple-copy	floweringlocusproteinT
Aradu.A9H9T	Araip.PC28F	multiple-copy	floweringlocusproteinT
Aradu.1I7E9	Araip.T1SIZ	multiple-copy	floweringlocusproteinT
Aradu.A4ISL	Araip.U9HL1	multiple-copy	floweringlocusproteinT

Aradu.Z8JSI	Araip.WF9GZ	multiple-copy	floweringlocusproteinT
Aradu.HY0D1	Araip.58I5B	multiple-copy	floweringtimecontrolproteinFPA-likeisoformX2[Glycinema
Aradu.T034F	Araip.B0LR2	multiple-copy	FLX-like3-likeisoformX1[Glycinemax]
Aradu.B2A4G	Araip.T7DB7	multiple-copy	FLX-like1-likeisoformX1[Glycinemax]
Aradu.1J0IW	Araip.HWJ4T	multiple-copy	FMP32
Aradu.0GS9T	Araip.IHP9I	multiple-copy	folate-biopterintransporter
Aradu.64LXI	Araip.J49VC	multiple-copy	Folicacidandderivativebiosyntheticprocessisoform1n=1Ta
Aradu.2088W	Araip.J5BBQ	multiple-copy	Folicacidandderivativebiosyntheticprocessisoform1n=1Ta
Aradu.W3KTW	Araip.R5DP2	multiple-copy	formin8
Aradu.RGH56	Araip.JS37L	multiple-copy	formin-likeprotein3-likeisoformX4[Glycinemax]
Aradu.3X0HY	Araip.1J2S6	multiple-copy	FRIGIDA-likeprotein
Aradu.N7F34	Araip.X8GX1	multiple-copy	fructose-1,6-bisphosphatase
Aradu.C7JM0	Araip.YB9GG	multiple-copy	fructose-1,6-bisphosphatase
Aradu.AH5QJ	Araip.XS0WA	multiple-copy	fructose-bisphosphatealdolase2
Aradu.P5PU5	Araip.IVL2L	multiple-copy	FUNCTIONSIN:molecular_functionunknown
Aradu.N21E2	Araip.ND6Q8	multiple-copy	GAGA-bindingproteinisoformX3[Glycinemax]
Aradu.BR5RX	Araip.57L7Q	multiple-copy	galactinol--sucrosegalactosyltransferase6-likeisoformX1[(
Aradu.P39C1	Araip.M48BY	multiple-copy	galactinolsynthase1
Aradu.XN3YF	Araip.64V16	multiple-copy	Galactose-bindingprotein
Aradu.8I7SF	Araip.6759X	multiple-copy	Galactoseoxidase/kelchrepeatsuperfamilyprotein
Aradu.D2EC5	Araip.9D1B8	multiple-copy	Galactoseoxidase/kelchrepeatsuperfamilyprotein
Aradu.XPA7G	Araip.AM8RU	multiple-copy	Galactoseoxidase/kelchrepeatsuperfamilyprotein
Aradu.1E1I2	Araip.I1B5X	multiple-copy	Galactoseoxidase/kelchrepeatsuperfamilyprotein
Aradu.S94H9	Araip.NVV81	multiple-copy	Galactoseoxidase/kelchrepeatsuperfamilyprotein
Aradu.KX3FZ	Araip.PDZ35	multiple-copy	Galactoseoxidase/kelchrepeatsuperfamilyprotein
Aradu.B45K6	Araip.QRQ13	multiple-copy	Galactoseoxidase/kelchrepeatsuperfamilyprotein
Aradu.F8MV2	Araip.VEY8S	multiple-copy	Galactoseoxidase/kelchrepeatsuperfamilyprotein
Aradu.EQT8P	Araip.R7UJS	multiple-copy	galactoside2-alpha-L-fucosyltransferase-likeprotein
Aradu.5G6X0	Araip.17ZYU	multiple-copy	Galactosyltransferasefamilyprotein
Aradu.MYL9N	Araip.567RF	multiple-copy	Galactosyltransferasefamilyprotein
Aradu.N62XI	Araip.78DPR	multiple-copy	Galactosyltransferasefamilyprotein
Aradu.00NYG	Araip.9D3W1	multiple-copy	Galactosyltransferasefamilyprotein
Aradu.EGG67	Araip.FA9IV	multiple-copy	Galactosyltransferasefamilyprotein
Aradu.F9XLT	Araip.GV8QE	multiple-copy	Galactosyltransferasefamilyprotein
Aradu.FI2RL	Araip.UUA6E	multiple-copy	Galactosyltransferasefamilyprotein
Aradu.AR630	Araip.W3RN5	multiple-copy	Galactosyltransferasefamilyprotein
Aradu.903PW	Araip.L0D82	multiple-copy	GalactosyltransferaseGMA12/MNN10familyprotein
Aradu.4C314	Araip.ZDD3V	multiple-copy	galacturonosyltransferase10-like[Glycinemax]
Aradu.2Z89F	Araip.864M6	multiple-copy	galacturonosyltransferase12-like[Glycinemax]
Aradu.4HQ1D	Araip.4L98G	multiple-copy	galacturonosyltransferase4-like[Glycinemax]
Aradu.1Z96C	Araip.97WJC	multiple-copy	galacturonosyltransferase4-like[Glycinemax]
Aradu.Y12MD	Araip.ST976	multiple-copy	galacturonosyltransferase4-like[Glycinemax]
Aradu.79NAD	Araip.NR5B5	multiple-copy	galacturonosyltransferase8-like[Glycinemax]
Aradu.AM0CF	Araip.J32EY	multiple-copy	galacturonosyltransferase-like4-like[Glycinemax]

Aradu.SAY01	Araip.5R1U0	multiple-copy	gamete-likeprotein
Aradu.UL23E	Araip.WJS9Q	multiple-copy	gamma-glutamyltranspeptidase4
Aradu.K2YQU	Araip.KQ0AG	multiple-copy	gammainterferoninduciblelysosomalthiolreductase
Aradu.W3YJN	Araip.69F5Y	multiple-copy	gamma-tubulincomplexprotein2
Aradu.KU4KX	Araip.9P2KQ	multiple-copy	GAMMA-TUBULINCOMPLEXPROTEIN4
Aradu.MD214	Araip.JY13W	multiple-copy	gar2-likeisoformX2[Glycinemax]
Aradu.YNN2E	Araip.YP44K	multiple-copy	GATAtranscriptionfactor12
Aradu.K9P9T	Araip.1196H	multiple-copy	GATAtranscriptionfactor15
Aradu.P7QDD	Araip.23X2M	multiple-copy	GATAtranscriptionfactor19
Aradu.LS0XC	Araip.AR1XP	multiple-copy	GATAtranscriptionfactor19
Aradu.SC6RY	Araip.QC09Z	multiple-copy	GATAtranscriptionfactor9
Aradu.TM66X	Araip.SN4PX	multiple-copy	GATAtranscriptionfactor9
Aradu.D252Q	Araip.UP1EH	multiple-copy	GATAtranscriptionfactor9
Aradu.YR0IE	Araip.A2CHT	multiple-copy	GATAtypezincfingertranscriptionfactorfamilyprotein
Aradu.G0S92	Araip.A841F	multiple-copy	GATAtypezincfingertranscriptionfactorfamilyprotein
Aradu.W0TSV	Araip.NID0Y	multiple-copy	GATAtypezincfingertranscriptionfactorfamilyprotein
Aradu.WU2UF	Araip.VYR7U	multiple-copy	GATAtypezincfingertranscriptionfactorfamilyprotein
Aradu.724NR	Araip.HSV9Y	multiple-copy	GDA1/CD39nucleosidephosphatasefamilyprotein
Aradu.M98W9	Araip.WKG50	multiple-copy	GDA1/CD39nucleosidephosphatasefamilyprotein
Aradu.P3SFW	Araip.UN61H	multiple-copy	GDP-L-galactosephosphorylase1-like[Glycinemax]
Aradu.8D6KF	Araip.7274A	multiple-copy	GDSLesterase/lipaseplant-likeprotein
Aradu.GVH4D	Araip.KU884	multiple-copy	GDSLesterase/lipaseplant-likeprotein
Aradu.ESL6S	Araip.PP1Y2	multiple-copy	GDSL-likeLipase/Acylhydrolasefamilyprotein
Aradu.KIS5R	Araip.0PX5T	multiple-copy	GDSL-likeLipase/Acylhydrolasesuperfamilyprotein
Aradu.ZX8WQ	Araip.1Y3MU	multiple-copy	GDSL-likeLipase/Acylhydrolasesuperfamilyprotein
Aradu.VT5GA	Araip.4K0XH	multiple-copy	GDSL-likeLipase/Acylhydrolasesuperfamilyprotein
Aradu.HZ16A	Araip.8C7AS	multiple-copy	GDSL-likeLipase/Acylhydrolasesuperfamilyprotein
Aradu.BS70J	Araip.8X7QI	multiple-copy	GDSL-likeLipase/Acylhydrolasesuperfamilyprotein
Aradu.Z6652	Araip.A2AJ6	multiple-copy	GDSL-likeLipase/Acylhydrolasesuperfamilyprotein
Aradu.RCQ39	Araip.AEM8T	multiple-copy	GDSL-likeLipase/Acylhydrolasesuperfamilyprotein
Aradu.ZD4TK	Araip.B24DH	multiple-copy	GDSL-likeLipase/Acylhydrolasesuperfamilyprotein
Aradu.13SFN	Araip.B6Q3S	multiple-copy	GDSL-likeLipase/Acylhydrolasesuperfamilyprotein
Aradu.D2U3D	Araip.BM4R7	multiple-copy	GDSL-likeLipase/Acylhydrolasesuperfamilyprotein
Aradu.AW1PY	Araip.CW8B2	multiple-copy	GDSL-likeLipase/Acylhydrolasesuperfamilyprotein
Aradu.QSW68	Araip.E9AXK	multiple-copy	GDSL-likeLipase/Acylhydrolasesuperfamilyprotein
Aradu.232FJ	Araip.EN1G0	multiple-copy	GDSL-likeLipase/Acylhydrolasesuperfamilyprotein
Aradu.L3677	Araip.EXQ89	multiple-copy	GDSL-likeLipase/Acylhydrolasesuperfamilyprotein
Aradu.LL477	Araip.G9LAW	multiple-copy	GDSL-likeLipase/Acylhydrolasesuperfamilyprotein
Aradu.69YXI	Araip.HWH2I	multiple-copy	GDSL-likeLipase/Acylhydrolasesuperfamilyprotein
Aradu.06CIQ	Araip.K7W5L	multiple-copy	GDSL-likeLipase/Acylhydrolasesuperfamilyprotein
Aradu.NFL6Z	Araip.KF0DD	multiple-copy	GDSL-likeLipase/Acylhydrolasesuperfamilyprotein
Aradu.M970R	Araip.LSW6W	multiple-copy	GDSL-likeLipase/Acylhydrolasesuperfamilyprotein
Aradu.9UB80	Araip.M0LAQ	multiple-copy	GDSL-likeLipase/Acylhydrolasesuperfamilyprotein
Aradu.EZ6B1	Araip.M87WJ	multiple-copy	GDSL-likeLipase/Acylhydrolasesuperfamilyprotein

Aradu.5B2EV	Araip.Q0F05	multiple-copy	GDSL-likeLipase/Acylhydrolasesuperfamilyprotein
Aradu.P709D	Araip.RVY5J	multiple-copy	GDSL-likeLipase/Acylhydrolasesuperfamilyprotein
Aradu.AQB31	Araip.T82XN	multiple-copy	GDSL-likeLipase/Acylhydrolasesuperfamilyprotein
Aradu.9RQ53	Araip.T97EY	multiple-copy	GDSL-likeLipase/Acylhydrolasesuperfamilyprotein
Aradu.S6F46	Araip.TME8J	multiple-copy	GDSL-likeLipase/Acylhydrolasesuperfamilyprotein
Aradu.QP7DQ	Araip.UIV2U	multiple-copy	GDSL-likeLipase/Acylhydrolasesuperfamilyprotein
Aradu.RQ3SQ	Araip.VL5WX	multiple-copy	GDSL-likeLipase/Acylhydrolasesuperfamilyprotein
Aradu.U260V	Araip.VN84X	multiple-copy	GDSL-likeLipase/Acylhydrolasesuperfamilyprotein
Aradu.0M9X8	Araip.VS99S	multiple-copy	GDSL-likeLipase/Acylhydrolasesuperfamilyprotein
Aradu.E0JEI	Araip.W7U7R	multiple-copy	GDSL-likeLipase/Acylhydrolasesuperfamilyprotein
Aradu.ZY82G	Araip.XU3BG	multiple-copy	GDSL-likeLipase/Acylhydrolasesuperfamilyprotein
Aradu.HG92V	Araip.Z54F6	multiple-copy	GDSL-likeLipase/Acylhydrolasesuperfamilyprotein
Aradu.Y4SSQ	Araip.ZDV0R	multiple-copy	GDSL-likeLipase/Acylhydrolasesuperfamilyprotein
Aradu.G7CKS	Araip.43P31	multiple-copy	generalregulatoryfactor2
Aradu.Q3RYP	Araip.QBG6Y	multiple-copy	generalregulatoryfactor7
Aradu.1M9DY	Araip.CVB1D	multiple-copy	generalregulatoryfactor8
Aradu.11KMG	Araip.647BE	multiple-copy	generalregulatoryfactor9
Aradu.C1UGC	Araip.Z68MJ	multiple-copy	generaltranscriptionfactorgroupE6
Aradu.BH653	Araip.UFD3P	multiple-copy	geranylgeranylpyrophosphatesynthase1
Aradu.90S6X	Araip.FD663	multiple-copy	germin-likeprotein10
Aradu.B1NGL	Araip.Q61XZ	multiple-copy	germin-likeprotein9-3-like[Glycinemax]
Aradu.D1QW8	Araip.8X7VT	multiple-copy	gibberellin20oxidase1-like[Glycinemax]
Aradu.GMU6R	Araip.A66EM	multiple-copy	gibberellin20oxidase1-like[Glycinemax]
Aradu.69GC2	Araip.JM070	multiple-copy	gibberellin20oxidase1-like[Glycinemax]
Aradu.Q0ZWM	Araip.SGA37	multiple-copy	gibberellin20oxidase1-like[Glycinemax]
Aradu.FI39R	Araip.5XD8H	multiple-copy	gibberellin20oxidase2-like[Glycinemax]
Aradu.GN3K0	Araip.E8TE0	multiple-copy	gibberellin20oxidase2-like[Glycinemax]
Aradu.7SN56	Araip.KVM2C	multiple-copy	gibberellin20oxidase2-like[Glycinemax]
Aradu.38DG2	Araip.L4RII	multiple-copy	gibberellin20oxidase2-like[Glycinemax]
Aradu.XZ0HK	Araip.YF8MJ	multiple-copy	gibberellin20oxidase2-like[Glycinemax]
Aradu.U838H	Araip.DQI4G	multiple-copy	gibberellin3-beta-dioxygenase1-like[Glycinemax]
Aradu.8V764	Araip.9I7A7	multiple-copy	Gibberellin-regulatedfamilyprotein
Aradu.C924Y	Araip.L5XNA	multiple-copy	Gibberellin-regulatedfamilyprotein
Aradu.1X6Z1	Araip.T84RU	multiple-copy	Gibberellin-regulatedfamilyprotein
Aradu.SZ9LD	Araip.V6F8I	multiple-copy	Gibberellin-regulatedfamilyprotein
Aradu.0E8DM	Araip.VQ4D8	multiple-copy	Gibberellin-regulatedfamilyprotein
Aradu.X22EB	Araip.ZQ9EU	multiple-copy	Gibberellin-regulatedfamilyprotein
Aradu.X1ANV	Araip.FU667	multiple-copy	globaltranscriptionfactorgroupprotein
Aradu.FA8EI	Araip.83IVK	multiple-copy	glucan1,3-beta-glucosidaseA-like[Glycinemax]
Aradu.61HSA	Araip.99RH1	multiple-copy	glucan1,3-beta-glucosidaseA-like[Glycinemax]
Aradu.WJZ76	Araip.EK3ME	multiple-copy	glucan1,3-beta-glucosidaseA-like[Glycinemax]
Aradu.8I8L4	Araip.GM86N	multiple-copy	glucanendo-1,3-beta-D-glucosidase-like[Glycinemax]
Aradu.D72GI	Araip.Q50JM	multiple-copy	glucanendo-1,3-beta-glucosidase11-like[Glycinemax]
Aradu.RUK3P	Araip.3F4LC	multiple-copy	glucanendo-1,3-beta-glucosidase12-like[Glycinemax]

Aradu.VK692	Araip.F9Y3B	multiple-copy	glucanendo-1,3-beta-glucosidase12-like[Glycinemax]
Aradu.8J98F	Araip.1WE6F	multiple-copy	glucanendo-1,3-beta-glucosidase3[Glycinemax]
Aradu.Q5EC8	Araip.3S4CE	multiple-copy	glucanendo-1,3-beta-glucosidase13-like[Glycinemax]
Aradu.VJ006	Araip.M5WW0	multiple-copy	glucanendo-1,3-beta-glucosidase13-like[Glycinemax]
Aradu.KDG8K	Araip.420T4	multiple-copy	glucanendo-1,3-beta-glucosidase14-like[Glycinemax]
Aradu.K0SUT	Araip.76PXL	multiple-copy	glucanendo-1,3-beta-glucosidase14-like[Glycinemax]
Aradu.FP3TI	Araip.CQY50	multiple-copy	glucanendo-1,3-beta-glucosidase14-like[Glycinemax]
Aradu.U8PRD	Araip.B6DZJ	multiple-copy	glucanendo-1,3-beta-glucosidase1-like[Glycinemax]
Aradu.UAQ2R	Araip.X6HL2	multiple-copy	glucanendo-1,3-beta-glucosidase1-like[Glycinemax]
Aradu.T8X2H	Araip.GL9AE	multiple-copy	glucanendo-1,3-beta-glucosidase2-like[Glycinemax]
Aradu.0LB5P	Araip.QP2H1	multiple-copy	glucanendo-1,3-beta-glucosidase3[Glycinemax]
Aradu.175L3	Araip.M2GWH	multiple-copy	glucanendo-1,3-beta-glucosidase3-like[Glycinemax]
Aradu.7Y3DJ	Araip.PBT3B	multiple-copy	glucanendo-1,3-beta-glucosidase3-like[Glycinemax]
Aradu.F0Y0D	Araip.PNY21	multiple-copy	glucanendo-1,3-beta-glucosidase2-like[Glycinemax]
Aradu.NG5IQ	Araip.E0Q5W	multiple-copy	glucanendo-1,3-beta-glucosidase4-like[Glycinemax]
Aradu.L46QG	Araip.61BVJ	multiple-copy	glucanendo-1,3-beta-glucosidase5-like[Glycinemax]
Aradu.ZP3W0	Araip.6DK8B	multiple-copy	glucanendo-1,3-beta-glucosidase5-like[Glycinemax]
Aradu.TK1NR	Araip.CW8LJ	multiple-copy	glucanendo-1,3-beta-glucosidase5-like[Glycinemax]
Aradu.MQ8BQ	Araip.S0PQ3	multiple-copy	glucanendo-1,3-beta-glucosidase5-like[Glycinemax]
Aradu.3V0DC	Araip.MW82X	multiple-copy	glucanendo-1,3-beta-glucosidase8-like[Glycinemax]
Aradu.JAT8P	Araip.T0MH3	multiple-copy	glucanendo-1,3-beta-glucosidase8-like[Glycinemax]
Aradu.B4P3F	Araip.VH6RA	multiple-copy	glucanendo-1,3-beta-glucosidase8-like[Glycinemax]
Aradu.12XXA	Araip.W3VCN	multiple-copy	glucanendo-1,3-beta-glucosidase8-like[Glycinemax]
Aradu.W9IJK	Araip.AB17F	multiple-copy	glucanendo-1,3-beta-glucosidase9-like[Glycinemax]
Aradu.J1G6I	Araip.4B1GU	multiple-copy	glucanendo-1,3-beta-glucosidaseA6-like[Glycinemax]
Aradu.7RC99	Araip.PZ7ZB	multiple-copy	glucanendo-1,3-beta-glucosidaseA6-like[Glycinemax]
Aradu.CAM8W	Araip.S0EN6	multiple-copy	glucanendo-1,3-beta-glucosidaseA6-like[Glycinemax]
Aradu.8Q6IV	Araip.BP0EH	multiple-copy	glucanendo-1,3-beta-glucosidase-like[Glycinemax]
Aradu.5474V	Araip.CP7MF	multiple-copy	glucanendo-1,3-beta-glucosidase-like[Glycinemax]
Aradu.SSI0J	Araip.E7C4C	multiple-copy	glucanendo-1,3-beta-glucosidase-like[Glycinemax]
Aradu.4R8IN	Araip.X067E	multiple-copy	glucanendo-1,3-beta-glucosidase-like[Glycinemax]
Aradu.HK2IG	Araip.G9SIR	multiple-copy	glucanendo-1,3-beta-glucosidase-likeprotein1-like[Glycin
Aradu.J4VEH	Araip.553HN	multiple-copy	glucanendo-1,3-beta-glucosidase-likeprotein2-like[Glycin
Aradu.1WV86	Araip.LCC0J	multiple-copy	glucanendo-1,3-beta-glucosidase-likeprotein2-like[Glycin
Aradu.SR17F	Araip.V2S44	multiple-copy	Glucose-1-phosphateadenylyltransferasefamilyprotein
Aradu.D3RA8	Araip.SR9I2	multiple-copy	glucose6-phosphate/phosphatetranslocator1
Aradu.K44L7	Araip.DML9Y	multiple-copy	glucose-6-phosphate/phosphatetranslocator2
Aradu.P0ZYD	Araip.VF4EA	multiple-copy	glucose-6-phosphate/phosphatetranslocator2
Aradu.HEE23	Araip.7KS0U	multiple-copy	Glucose-6-phosphate/phosphatetranslocator-related
Aradu.K3GE6	Araip.AA702	multiple-copy	Glucose-6-phosphate/phosphatetranslocator-related
Aradu.6M9LZ	Araip.G9XAZ	multiple-copy	Glucose-6-phosphate/phosphatetranslocator-related
Aradu.QRL86	Araip.QR9S0	multiple-copy	glucose-6-phosphate1-epimerase-likeisoformX4[Glycine
Aradu.90MFE	Araip.3A27H	multiple-copy	glucose-6-phosphatedehydrogenase2
Aradu.SHF2C	Araip.8K33E	multiple-copy	glucose-6-phosphatedehydrogenase4

Aradu.KRJ36	Araip.F4R2Z	multiple-copy	Glucose-methanol-choline(GMC)oxidoreductasefamilypro
Aradu.L8GY0	Araip.Z37FU	multiple-copy	Glucose-methanol-choline(GMC)oxidoreductasefamilypro
Aradu.JAD6J	Araip.I269H	multiple-copy	glucuronosyltransferasePGSIP6-likeisoformX2[Glycinema
Aradu.TN0QL	Araip.UF36S	multiple-copy	glutamate-1-semialdehyde2,1-aminomutase2
Aradu.2GH9Y	Araip.FRU70	multiple-copy	glutamatedecarboxylase
Aradu.ZC5IW	Araip.Q3F5T	multiple-copy	glutamatedehydrogenase1
Aradu.VTZ6D	Araip.WW8Y7	multiple-copy	glutamatedehydrogenase1
Aradu.57NVY	Araip.KAU0W	multiple-copy	glutamatedehydrogenase2
Aradu.0FG6K	Araip.W427S	multiple-copy	glutamatereceptor2.7
Aradu.QC919	Araip.IR8SW	multiple-copy	glutamatereceptor2.8
Aradu.RY2KR	Araip.8AA3H	multiple-copy	Glutamyl-tRNAreductasefamilyprotein
Aradu.21FR3	Araip.B72LY	multiple-copy	glutaredoxin4
Aradu.W48TA	Araip.X6683	multiple-copy	glutaredoxin4
Aradu.YFR3R	Araip.6J64A	multiple-copy	Glutaredoxinfamilyprotein
Aradu.910RR	Araip.E24Q0	multiple-copy	Glutaredoxinfamilyprotein
Aradu.20EC4	Araip.G6E5V	multiple-copy	Glutaredoxinfamilyprotein
Aradu.M1BLH	Araip.HEJ11	multiple-copy	Glutaredoxinfamilyprotein
Aradu.UIA0J	Araip.HR7AZ	multiple-copy	Glutaredoxinfamilyprotein
Aradu.H2W20	Araip.II7NF	multiple-copy	Glutaredoxinfamilyprotein
Aradu.F9KJM	Araip.U7F06	multiple-copy	Glutaredoxinfamilyprotein
Aradu.P2130	Araip.X36UP	multiple-copy	Glutaredoxinfamilyprotein
Aradu.M4TPQ	Araip.8AM62	multiple-copy	glutathioneperoxidase4
Aradu.4U4LS	Araip.PBK8S	multiple-copy	glutathioneperoxidase4
Aradu.8UL46	Araip.270IQ	multiple-copy	glutathioneperoxidase8
Aradu.K45PE	Araip.N346R	multiple-copy	glutathionereductase
Aradu.73PA3	Araip.0BL53	multiple-copy	GlutathioneS-transferasefamilyprotein
Aradu.K7JNV	Araip.2ES8K	multiple-copy	GlutathioneS-transferasefamilyprotein
Aradu.9Z02R	Araip.56C70	multiple-copy	GlutathioneS-transferasefamilyprotein
Aradu.ZPP4Z	Araip.5HK6K	multiple-copy	GlutathioneS-transferasefamilyprotein
Aradu.65TC6	Araip.916M9	multiple-copy	GlutathioneS-transferasefamilyprotein
Aradu.6PF06	Araip.BHX6F	multiple-copy	GlutathioneS-transferasefamilyprotein
Aradu.HA5JM	Araip.CA6VL	multiple-copy	GlutathioneS-transferasefamilyprotein
Aradu.RFH8Y	Araip.D6VSK	multiple-copy	GlutathioneS-transferasefamilyprotein
Aradu.48DYG	Araip.F8RVF	multiple-copy	GlutathioneS-transferasefamilyprotein
Aradu.DB78U	Araip.IBD1I	multiple-copy	GlutathioneS-transferasefamilyprotein
Aradu.KHJ4B	Araip.KVQ8U	multiple-copy	GlutathioneS-transferasefamilyprotein
Aradu.TZ9RJ	Araip.L2SQL	multiple-copy	GlutathioneS-transferasefamilyprotein
Aradu.86SPU	Araip.M9I94	multiple-copy	GlutathioneS-transferasefamilyprotein
Aradu.80TQM	Araip.N829R	multiple-copy	GlutathioneS-transferasefamilyprotein
Aradu.51L6N	Araip.U0SXH	multiple-copy	GlutathioneS-transferasefamilyprotein
Aradu.P7Y6N	Araip.Y4CF6	multiple-copy	GlutathioneS-transferasefamilyprotein
Aradu.A3N3V	Araip.4Z02U	multiple-copy	glyceraldehyde-3-phosphatedehydrogenaseC2
Aradu.Y017B	Araip.S0LKT	multiple-copy	glyceraldehyde-3-phosphatedehydrogenaseC2
Aradu.3S60E	Araip.U6VQA	multiple-copy	glyceraldehyde-3-phosphatedehydrogenaseC2

Aradu.7X3HU	Araip.W4V3G	multiple-copy	glyceraldehyde-3-phosphatedehydrogenaseC2
Aradu.GH8LH	Araip.8E1VL	multiple-copy	glycerol-3-phosphateacyltransferase3-likeisoformX1[Glyc
Aradu.KZX0F	Araip.IXQ5W	multiple-copy	glycerol-3-phosphateacyltransferase4
Aradu.X10AK	Araip.A87RC	multiple-copy	glycerol-3-phosphateacyltransferase5
Aradu.31H7A	Araip.99AMZ	multiple-copy	glycerol-3-phosphateacyltransferase6
Aradu.31VP0	Araip.33SF4	multiple-copy	glycerol-3-phosphatedehydrogenase[NAD(+)]GPDHC1,cy
Aradu.TT65B	Araip.XJ990	multiple-copy	glycerol-3-phosphatedehydrogenase[NAD(+)]GPDHC1,cy
Aradu.M62SP	Araip.G2WFV	multiple-copy	glycerol-3-phosphatetransporter1-likeisoformX1[Glycinem
Aradu.QK77V	Araip.CU4DG	multiple-copy	glycerol-3-phosphatetransporter4-likeisoformX1[Glycinem
Aradu.NS576	Araip.UHM0G	multiple-copy	glycine-richprotein
Aradu.NHR1S	Araip.14BL9	multiple-copy	glycine-richRNA-bindingprotein1
Aradu.5RE8S	Araip.R7UPP	multiple-copy	glycine-richRNA-bindingprotein3
Aradu.SC3I7	Araip.MA8U3	multiple-copy	glycine-richRNA-bindingprotein3
Aradu.HAD5C	Araip.4A1JG	multiple-copy	glycolipidtransferprotein1
Aradu.0LP8B	Araip.QPM11	multiple-copy	GlycoproteinmembraneprecursorGPI-anchored
Aradu.B4D1B	Araip.TWH1T	multiple-copy	GlycoproteinmembraneprecursorGPI-anchored
Aradu.F64Z1	Araip.914CH	multiple-copy	Glycosylhydrolasefamilyproteinwithchitinaseinsertiondom
Aradu.V0CGL	Araip.983RG	multiple-copy	Glycosylhydrolasefamilyproteinwithchitinaseinsertiondom
Aradu.BR4VK	Araip.KA397	multiple-copy	Glycosylhydrolasefamilyproteinwithchitinaseinsertiondom
Aradu.84XE2	Araip.1N6HI	multiple-copy	Glycosylhydrolasesuperfamilyprotein
Aradu.LEE20	Araip.PM669	multiple-copy	glycosyltransferaseAt3g07620-like[Glycinemax]
Aradu.YH2KM	Araip.51VIE	multiple-copy	glycosyltransferaseAt5g03795-like[Glycinemax]
Aradu.I73XP	Araip.569RT	multiple-copy	glycosyltransferaseAt5g03795-like[Glycinemax]
Aradu.79876	Araip.BFL4V	multiple-copy	glycosyltransferaseAt5g03795-like[Glycinemax]
Aradu.TN13P	Araip.Y0VZU	multiple-copy	glycosyltransferaseAt5g03795-like[Glycinemax]
Aradu.5JM2L	Araip.PX6LZ	multiple-copy	Glycosyltransferasefamily9n=1Tax=Nostocsp.PCC7107R
Aradu.U23UQ	Araip.JT07S	multiple-copy	glycosyltransferaseisoformX4[Glycinemax]
Aradu.ZF8NC	Araip.M9SP4	multiple-copy	glycosyltransferaseisoformX4[Glycinemax]
Aradu.N725Y	Araip.N6IWK	multiple-copy	glycosyltransferaseisoformX4[Glycinemax]
Aradu.1K1MI	Araip.PK50H	multiple-copy	glycosyltransferaseAt5g03795-like[Glycinemax]
Aradu.JAF9B	Araip.X26F2	multiple-copy	glycosyltransferaseisoformX4[Glycinemax]
Aradu.BV9FL	Araip.UR4F4	multiple-copy	glyoxaloxidase-relatedprotein
Aradu.77UYS	Araip.I1QS9	multiple-copy	Golgiapparatusmembraneproteintvp23n=3Tax=Aspergillu
Aradu.J355A	Araip.N9WJ3	multiple-copy	Got1/Sft2-likevescicletransportproteinfamily
Aradu.8DJ4R	Araip.580BK	multiple-copy	GPI-anchoredprotein[Glycinemax]
Aradu.CUQ8J	Araip.F0LWE	multiple-copy	GPI-anchoredprotein[Glycinemax]
Aradu.5SD5G	Araip.E377S	multiple-copy	GPI-anchoredprotein[Glycinemax]
Aradu.418KR	Araip.F83NR	multiple-copy	GPI-anchoredprotein[Glycinemax]
Aradu.C5KI2	Araip.XD91V	multiple-copy	GPI-anchoredprotein[Glycinemax]
Aradu.IBY74	Araip.5ZR88	multiple-copy	GPI-anchortransamidase-likeprotein
Aradu.VX0W5	Araip.JLU3W	multiple-copy	GRAMdomain-containingprotein/ABA-responsiveprotein-r
Aradu.02LQD	Araip.JP4ZN	multiple-copy	GRAMdomain-containingprotein/ABA-responsiveprotein-r
Aradu.DYF0M	Araip.P095Q	multiple-copy	GRAMdomain-containingprotein/ABA-responsiveprotein-r
Aradu.RQH55	Araip.J2LHI	multiple-copy	GRAMdomainfamilyprotein

Aradu.B4HJ4	Araip.ALT9X	multiple-copy	GRFzincfingerprotein
Aradu.70U4X	Araip.DB5YG	multiple-copy	GRFzincfingerprotein
Aradu.674AT	Araip.R87AF	multiple-copy	GRFzincfingerprotein
Aradu.ZF1LK	Araip.7PD4P	multiple-copy	growth-regulatingfactor4
Aradu.VXK5T	Araip.4Y3B5	multiple-copy	growth-regulatingfactor5
Aradu.Q77AH	Araip.J006E	multiple-copy	growth-regulatingfactor5
Aradu.86IQQ	Araip.YK7C2	multiple-copy	growth-regulatingfactor5
Aradu.Y1LT0	Araip.J8BZ6	multiple-copy	GTE8-likeisoformX2[Glycinemax]
Aradu.X0LHP	Araip.NPG6G	multiple-copy	GTPbinding
Aradu.DC5GT	Araip.6A4J6	multiple-copy	GTPbindingElongationfactorTufamilyprotein
Aradu.68E2R	Araip.L1E1K	multiple-copy	GTPbindingElongationfactorTufamilyprotein
Aradu.9SB6V	Araip.6F88V	multiple-copy	GTP-bindingnuclearproteinRan-3-like[Glycinemax]
Aradu.Z753H	Araip.7G11P	multiple-copy	GTP-bindingnuclearproteinRan-3[Glycinemax]
Aradu.PI5HS	Araip.8133V	multiple-copy	GTP-bindingnuclearproteinRan-3[Glycinemax]
Aradu.N4PGS	Araip.AX2SC	multiple-copy	GTP-bindingnuclearproteinRan-3[Glycinemax]
Aradu.VY6JK	Araip.F8CEG	multiple-copy	GTP-bindingnuclearproteinRan-3[Glycinemax]
Aradu.GU21D	Araip.I2GG6	multiple-copy	GTP-bindingnuclearproteinRan-3[Glycinemax]
Aradu.1PQ4F	Araip.T0L79	multiple-copy	GTP-bindingnuclearproteinRan-3-like[Glycinemax]
Aradu.S3UJF	Araip.W1QI3	multiple-copy	GTP-bindingnuclearproteinRan-3[Glycinemax]
Aradu.QE5XI	Araip.W5PJ7	multiple-copy	GTP-bindingnuclearproteinRan-3[Glycinemax]
Aradu.ZFF1D	Araip.2FX35	multiple-copy	GTP-bindingnuclearproteinRan-3-like[Glycinemax]
Aradu.ZJ194	Araip.73VEI	multiple-copy	GTP-bindingnuclearproteinRan-3-like[Glycinemax]
Aradu.EAQ74	Araip.DTN87	multiple-copy	GTP-bindingnuclearproteinRan-3[Glycinemax]
Aradu.G2VQL	Araip.PXU5X	multiple-copy	GTP-bindingnuclearproteinRan-3-like[Glycinemax]
Aradu.YJS53	Araip.YKX26	multiple-copy	GTP-bindingnuclearproteinRan-3-like[Glycinemax]
Aradu.F2KAM	Araip.J19JD	multiple-copy	GTP-bindingnuclearRan-likeprotein
Aradu.V9UDT	Araip.V71XV	multiple-copy	GTP-bindingnuclearRan-likeprotein
Aradu.GKG4B	Araip.N2GVW	multiple-copy	GTPbindingprotein,putativen=1Tax=RicinuscommunisRe
Aradu.1I5UA	Araip.RFS9X	multiple-copy	GTP-bindingproteinAt2g22870-likeisoformX3[Glycinema
Aradu.19W8X	Araip.V731N	multiple-copy	GTP-bindingproteinengAn=1Tax=MedicagotruncatulaRe
Aradu.KNP7I	Araip.KW46U	multiple-copy	GTP-bindingproteinERG-like[Glycinemax]
Aradu.2SV86	Araip.26UP8	multiple-copy	GTP-bindingproteinn=8Tax=BacillusRepID=A8FFF3_BA
Aradu.45HXA	Araip.U6HBJ	multiple-copy	GTP-bindingproteinObg/CgtA
Aradu.T337U	Araip.6NV0D	multiple-copy	GTP-bindingprotein-related
Aradu.AJZ98	Araip.K9ULC	multiple-copy	guaninenucleotide-bindingproteinalpha-2subunitisoformX
Aradu.WGT75	Araip.G2KFT	multiple-copy	guaninenucleotide-bindingproteinalpha-2subunitisoformX
Aradu.734NR	Araip.B7BWA	multiple-copy	guaninenucleotide-bindingproteinsubunitbeta-1[Glycinem
Aradu.G5W2I	Araip.UP566	multiple-copy	guaninenucleotide-bindingproteinsubunitbeta-1[Glycinem
Aradu.C8CZA	Araip.H639W	multiple-copy	Guanylate-bindingfamilyprotein
Aradu.K14NU	Araip.4J7U0	multiple-copy	H/ACAribonucleoproteincomplexsubunit2,putative
Aradu.WWM41	Araip.F2XI1	multiple-copy	HADsuperfamily,subfamilyIIIBacidphosphatase
Aradu.T25QT	Araip.P6G60	multiple-copy	HADsuperfamily,subfamilyIIIBacidphosphatase
Aradu.DZ6IG	Araip.WE1Z2	multiple-copy	HADsuperfamily,subfamilyIIIBacidphosphatase
Aradu.27F3R	Araip.217TR	multiple-copy	haloaciddehalogenase(HAD)superfamilyprotein

Aradu.GBG51	Araip.A0QGV	multiple-copy	haloaciddehalogenase-likehydrolase
Aradu.N7TNH	Araip.A1RD2	multiple-copy	haloaciddehalogenase-likehydrolase
Aradu.H76RB	Araip.II799	multiple-copy	haloaciddehalogenase-likehydrolase
Aradu.VK9S0	Araip.K5MNX	multiple-copy	haloaciddehalogenase-likehydrolase
Aradu.BX447	Araip.736QB	multiple-copy	Haloaciddehalogenase-likehydrolase(HAD)superfamilypr
Aradu.CAH9K	Araip.82QS5	multiple-copy	Haloaciddehalogenase-likehydrolase(HAD)superfamilypr
Aradu.J2HBF	Araip.PHT1P	multiple-copy	Haloaciddehalogenase-likehydrolase(HAD)superfamilypr
Aradu.VM94P	Araip.VD2UK	multiple-copy	Haloaciddehalogenase-likehydrolase(HAD)superfamilypr
Aradu.Z705N	Araip.25CYT	multiple-copy	Haloaciddehalogenase-likehydrolase,putativen=1Tax=Sy
Aradu.4A3GU	Araip.5Y2PN	multiple-copy	Haloaciddehalogenase-likehydrolase,putativen=1Tax=Sy
Aradu.AM9WK	Araip.EL2JP	multiple-copy	haloaciddehalogenase-likehydrolasedomainprotein
Aradu.D42IC	Araip.R0CLD	multiple-copy	haloaciddehalogenase-likehydrolasedomainprotein
Aradu.LP9ZI	Araip.NVV9S	multiple-copy	HCP-likesuperfamilyproteinwithMYND-typezincfinger
Aradu.R92J4	Araip.VE3V9	multiple-copy	HCP-likesuperfamilyproteinwithMYND-typezincfinger
Aradu.L4J2W	Araip.KV8CW	multiple-copy	heatshockprotein
Aradu.XQ3RE	Araip.PRK60	multiple-copy	heatshockprotein
Aradu.I2A4P	Araip.X1JSV	multiple-copy	heatshockprotein
Aradu.WL1AY	Araip.FT330	multiple-copy	heatshockprotein-like[Glycinemax]
Aradu.SJ2KY	Araip.SBC75	multiple-copy	heatshockprotein-like[Glycinemax]
Aradu.EM2UC	Araip.I39IS	multiple-copy	heatshockprotein-like[Glycinemax]
Aradu.724TL	Araip.3A86R	multiple-copy	heatshockprotein-like[Glycinemax]
Aradu.NL8HQ	Araip.1ML5W	multiple-copy	heatshockprotein21
Aradu.A3TK2	Araip.G7QFC	multiple-copy	heatshockprotein21
Aradu.MS98X	Araip.9XA0M	multiple-copy	heatshockprotein70
Aradu.SB9IZ	Araip.K6VSE	multiple-copy	heatshockprotein70
Aradu.TET8U	Araip.MFC9D	multiple-copy	heatshockprotein70
Aradu.Z1N61	Araip.VHZ5J	multiple-copy	heatshockprotein70
Aradu.G6ZA5	Araip.WYM3Y	multiple-copy	heatshockprotein70
Aradu.NE0BE	Araip.Z8IWW	multiple-copy	heatshockprotein70
Aradu.EYD2G	Araip.H6BYJ	multiple-copy	HEATSHOCKPROTEIN89.1
Aradu.H926S	Araip.YUI9C	multiple-copy	heatshockprotein91
Aradu.18HYX	Araip.AA0NE	multiple-copy	heatshockprotein-bindingprotein
Aradu.EK3X2	Araip.Y5NDQ	multiple-copy	heatshockfamilyprotein
Aradu.YU6QF	Araip.U1GUY	multiple-copy	Heatshockproteinbindingproteinn=1Tax=ZeamaysRepID=
Aradu.DU4CZ	Araip.G0TCZ	multiple-copy	HeatshockproteinHsp20n=2Tax=SulfurihydrogenibiumRe
Aradu.72KXP	Araip.CNH5B	multiple-copy	heatshockprotein-like[Glycinemax]
Aradu.73KGG	Araip.ZKH0K	multiple-copy	heatshockproteinSTI-likeisoformX1[Glycinemax]
Aradu.H8ZFN	Araip.4A18K	multiple-copy	heatshocktranscriptionfactorA2
Aradu.RY508	Araip.B0RQS	multiple-copy	heatshocktranscriptionfactorA2
Aradu.95F2Q	Araip.SMI4I	multiple-copy	heatshocktranscriptionfactorA2
Aradu.N3N49	Araip.U7I8R	multiple-copy	heatshocktranscriptionfactorA2
Aradu.RVF1V	Araip.2D8LN	multiple-copy	heatshocktranscriptionfactorA2
Aradu.N1DAK	Araip.3P0PJ	multiple-copy	heatshocktranscriptionfactorA8
Aradu.K2DQ9	Araip.3KN0A	multiple-copy	heatshocktranscriptionfactorB4

Aradu.S4DGV	Araip.90YM8	multiple-copy	heatshocktranscriptionfactorB4
Aradu.NTQ7W	Araip.K80UC	multiple-copy	heatshocktranscriptionfactorB4
Aradu.X3DNX	Araip.UGV6F	multiple-copy	heatshocktranscriptionfactorB4
Aradu.43K0P	Araip.FU4GL	multiple-copy	heatshocktranscriptionfactorC1
Aradu.FC7FL	Araip.23M3Y	multiple-copy	Heavymetaltransport/detoxificationsuperfamilyprotein
Aradu.56ZRQ	Araip.36PI6	multiple-copy	Heavymetaltransport/detoxificationsuperfamilyprotein
Aradu.V6UC8	Araip.3X84U	multiple-copy	Heavymetaltransport/detoxificationsuperfamilyprotein
Aradu.YZA37	Araip.4AW6Y	multiple-copy	Heavymetaltransport/detoxificationsuperfamilyprotein
Aradu.S0307	Araip.4FT5T	multiple-copy	Heavymetaltransport/detoxificationsuperfamilyprotein
Aradu.Q0NF8	Araip.65APQ	multiple-copy	Heavymetaltransport/detoxificationsuperfamilyprotein
Aradu.A87UA	Araip.86F6R	multiple-copy	Heavymetaltransport/detoxificationsuperfamilyprotein
Aradu.D46RH	Araip.B96WX	multiple-copy	Heavymetaltransport/detoxificationsuperfamilyprotein
Aradu.7Y76M	Araip.D6WDG	multiple-copy	Heavymetaltransport/detoxificationsuperfamilyprotein
Aradu.8UL0Z	Araip.FP6KG	multiple-copy	Heavymetaltransport/detoxificationsuperfamilyprotein
Aradu.YMR36	Araip.GK5JR	multiple-copy	Heavymetaltransport/detoxificationsuperfamilyprotein
Aradu.2I1UD	Araip.IYB9Y	multiple-copy	Heavymetaltransport/detoxificationsuperfamilyprotein
Aradu.T0IJJ	Araip.K2Q3T	multiple-copy	Heavymetaltransport/detoxificationsuperfamilyprotein
Aradu.G0P4Z	Araip.KM8WJ	multiple-copy	Heavymetaltransport/detoxificationsuperfamilyprotein
Aradu.G14M4	Araip.KW1YW	multiple-copy	Heavymetaltransport/detoxificationsuperfamilyprotein
Aradu.C7CT2	Araip.LWA5C	multiple-copy	Heavymetaltransport/detoxificationsuperfamilyprotein
Aradu.81XX0	Araip.MG0IA	multiple-copy	Heavymetaltransport/detoxificationsuperfamilyprotein
Aradu.Q2QD0	Araip.PIX7S	multiple-copy	Heavymetaltransport/detoxificationsuperfamilyprotein
Aradu.YD1VK	Araip.PK7HW	multiple-copy	Heavymetaltransport/detoxificationsuperfamilyprotein
Aradu.P156Z	Araip.Q9JAV	multiple-copy	Heavymetaltransport/detoxificationsuperfamilyprotein
Aradu.VQJ7V	Araip.R2GRG	multiple-copy	Heavymetaltransport/detoxificationsuperfamilyprotein
Aradu.I72RE	Araip.R5SLN	multiple-copy	Heavymetaltransport/detoxificationsuperfamilyprotein
Aradu.98FLM	Araip.RCT1Q	multiple-copy	Heavymetaltransport/detoxificationsuperfamilyprotein
Aradu.NKB4V	Araip.RKZ9H	multiple-copy	Heavymetaltransport/detoxificationsuperfamilyprotein
Aradu.7JU28	Araip.S3PA3	multiple-copy	Heavymetaltransport/detoxificationsuperfamilyprotein
Aradu.4IL2F	Araip.T34A8	multiple-copy	Heavymetaltransport/detoxificationsuperfamilyprotein
Aradu.65BY0	Araip.V64IQ	multiple-copy	Heavymetaltransport/detoxificationsuperfamilyprotein
Aradu.S9P8W	Araip.WG7BV	multiple-copy	Heavymetaltransport/detoxificationsuperfamilyprotein
Aradu.Q3KFX	Araip.WY6XF	multiple-copy	Heavymetaltransport/detoxificationsuperfamilyprotein
Aradu.48ATB	Araip.X1WDR	multiple-copy	Heavymetaltransport/detoxificationsuperfamilyprotein
Aradu.22KJ4	Araip.X2CY1	multiple-copy	Heavymetaltransport/detoxificationsuperfamilyprotein
Aradu.VM6WK	Araip.Z58RU	multiple-copy	Heavymetaltransport/detoxificationsuperfamilyprotein
Aradu.SCF1F	Araip.ZEX5T	multiple-copy	Heavymetaltransport/detoxificationsuperfamilyprotein
Aradu.VIL5A	Araip.3ZU66	multiple-copy	Helicase-likeproteinn=1Tax=MedicagotruncatulaRepID=G
Aradu.RV9UM	Araip.UQ6YY	multiple-copy	hemeoxygenase3
Aradu.KNH52	Araip.V5Y6Q	multiple-copy	heparan-alpha-glucosaminideN-acetyltransferase-likeprot
Aradu.4T8J3	Araip.U04P1	multiple-copy	heparanase-likeprotein1-likeisoformX2[Glycinemax]
Aradu.53YGT	Araip.H3GCU	multiple-copy	heparanase-likeprotein2-likeisoformX1[Glycinemax]
Aradu.ED8I5	Araip.LY1I1	multiple-copy	heparanase-likeprotein2-likeisoformX1[Glycinemax]
Aradu.DJW6E	Araip.LMP9N	multiple-copy	hexokinase2

Aradu.88KLW	Araip.Q9TXG	multiple-copy	hexokinase3
Aradu.QJ7B7	Araip.BC8KL	multiple-copy	highmobilitygroupBprotein15-likeisoformX7[Glycinemax]
Aradu.LAX0E	Araip.G7Z83	multiple-copy	highmobilitygroupBprotein15-likeisoformX7[Glycinemax]
Aradu.N0W4C	Araip.GVQ6N	multiple-copy	highmobilitygroupBprotein9-likeisoformX3[Glycinemax]
Aradu.YMT2S	Araip.FF9D7	multiple-copy	histidinephosphotransferprotein6
Aradu.NRF9P	Araip.N6TB9	multiple-copy	histidinephosphotransferprotein6
Aradu.7V00N	Araip.E5PIG	multiple-copy	histidinetriadnucleotide-binding4
Aradu.206YZ	Araip.S8FK3	multiple-copy	histoneacetyltransferaseHAC1-likeisoformX1[Glycinema[illegible]
Aradu.U9A7G	Araip.7RD3Q	multiple-copy	histonedeacetylase14
Aradu.K2UAZ	Araip.8U122	multiple-copy	histonedeacetylase15
Aradu.ND275	Araip.M0I6P	multiple-copy	histonedeacetylase2C
Aradu.F7Z5Y	Araip.TB7YD	multiple-copy	histonedeacetylase5
Aradu.MLQ47	Araip.N0SPZ	multiple-copy	histonedeacetylase8
Aradu.5A70G	Araip.8SM65	multiple-copy	histonedeacetylase9
Aradu.Z0GNU	Araip.721GK	multiple-copy	histoneH2A10
Aradu.Z31WB	Araip.RIF2S	multiple-copy	histoneH2A12
Aradu.DJ22Q	Araip.6WY95	multiple-copy	histoneH2A10
Aradu.EB16E	Araip.3Z0P1	multiple-copy	histoneH2A2
Aradu.7Y15X	Araip.R8SCL	multiple-copy	histoneH2A7
Aradu.M1IAT	Araip.C4K21	multiple-copy	histoneH2Aprotein9
Aradu.X2I4E	Araip.HPW81	multiple-copy	histoneH2Aprotein9
Aradu.FW5R5	Araip.MCH9G	multiple-copy	histone-lysineN-methyltransferase
Aradu.Q0YMS	Araip.H72KX	multiple-copy	histone-lysineN-methyltransferaseASHR2-likeisoformX3[[illegible]
Aradu.A4737	Araip.HL1JU	multiple-copy	histone-lysineN-methyltransferaseATX3-likeisoformX1[G[illegible]
Aradu.U7FRB	Araip.CG564	multiple-copy	histone-lysineN-methyltransferaseATX5-like[Glycinemax]
Aradu.0D317	Araip.GN51K	multiple-copy	histone-lysineN-methyltransferaseATXR2
Aradu.XK5C3	Araip.E02MH	multiple-copy	histone-lysineN-methyltransferaseATXR3-likeisoformX1[[illegible]
Aradu.AW9HW	Araip.16L7J	multiple-copy	histone-lysineN-methyltransferaseSUVR2-likeisoformX2[[illegible]
Aradu.P5027	Araip.YT9K8	multiple-copy	histone-lysineN-methyltransferaseSUVR2-likeisoformX2[[illegible]
Aradu.E9HU8	Araip.EBE31	multiple-copy	histone-lysineN-methyltransferaseSUVR2-likeisoformX2[[illegible]
Aradu.A5UR9	Araip.FNI26	multiple-copy	histone-lysineN-methyltransferaseSUVR2-likeisoformX2[[illegible]
Aradu.KL6JZ	Araip.SBB73	multiple-copy	histone-lysineN-methyltransferaseSUVR2-likeisoformX3[[illegible]
Aradu.0UC7C	Araip.7JE55	multiple-copy	histone-lysineN-methyltransferaseSUVR5-likeisoformX1[[illegible]
Aradu.KV0LC	Araip.XT6EH	multiple-copy	histone-lysineN-methyltransferaseSUVR5-likeisoformX2[[illegible]
Aradu.M5AZ6	Araip.D72J4	multiple-copy	Histonesuperfamilyprotein
Aradu.MM88P	Araip.G1BN4	multiple-copy	Histonesuperfamilyprotein
Aradu.6SC3T	Araip.PC9NR	multiple-copy	Histonesuperfamilyprotein
Aradu.W87GJ	Araip.R1LY9	multiple-copy	Histonesuperfamilyprotein
Aradu.I29MY	Araip.T5SL7	multiple-copy	Histonesuperfamilyprotein
Aradu.RP6HI	Araip.Y6RE5	multiple-copy	HIT-typeZincfingerfamilyprotein
Aradu.ZY1BM	Araip.M8CB8	multiple-copy	HITzincfinger
Aradu.IN3UA	Araip.KRL84	multiple-copy	HMG-Y-relatedproteinA-like[Glycinemax]
Aradu.KL6KV	Araip.F3CGD	multiple-copy	homeobox-leucinezipperprotein3
Aradu.4HB9F	Araip.APB7B	multiple-copy	homeobox-leucinezipperproteinANTHOCYANINLESS2-li[illegible]

Aradu.2T85U	Araip.0J7GI	multiple-copy	homeobox-leucinezipperproteinANTHOCYANINLESS2-li
Aradu.F2CY5	Araip.E6C1Q	multiple-copy	homeobox-leucinezipperproteinANTHOCYANINLESS2-li
Aradu.DA7MW	Araip.P0GNG	multiple-copy	homeobox-leucinezipperproteinANTHOCYANINLESS2-li
Aradu.NBA2I	Araip.V6XXN	multiple-copy	homeobox-leucinezipperprotein3
Aradu.TC0KD	Araip.Z68LQ	multiple-copy	Homeobox-leucinezipperproteinfamily
Aradu.A3E1X	Araip.K48LN	multiple-copy	homeoboxproteinknotted-1-like1-like[Glycinemax]
Aradu.S7JJM	Araip.953XL	multiple-copy	homeoboxproteinknotted-1-like2-like[Glycinemax]
Aradu.I940M	Araip.L12RP	multiple-copy	homeoboxproteinknotted-1-like2-like[Glycinemax]
Aradu.69PE5	Araip.GN10Z	multiple-copy	homeoboxproteinknotted-1-like3-likeisoformX4[Glycinema
Aradu.W71G4	Araip.11BKV	multiple-copy	homeoboxproteinknotted-1-like3-likeisoformX3[Glycinema
Aradu.D9Q5D	Araip.2CH00	multiple-copy	homeoboxproteinknotted-1-like3-likeisoformX4[Glycinema
Aradu.6LK51	Araip.SD3NU	multiple-copy	homeoboxproteinknotted-1-like3-likeisoformX2[Glycinema
Aradu.986KM	Araip.A9XWC	multiple-copy	homeoboxproteinknotted-1-like6-likeisoform2[Glycinemax
Aradu.S4P4Y	Araip.5MF6L	multiple-copy	homeoboxtranscriptionfactor
Aradu.D7VWN	Araip.22Y3L	multiple-copy	Homeodomain-liketranscriptionalregulator
Aradu.M3CK1	Araip.Q42SH	multiple-copy	homogentisatephytyltransferase1
Aradu.XR1NY	Araip.Y3ELL	multiple-copy	homogentisatephytyltransferase1
Aradu.DYP0A	Araip.LZ85S	multiple-copy	homoserinekinase
Aradu.I9N63	Araip.I5UTC	multiple-copy	HR-likelesion-inducingprotein-related
Aradu.U7UH3	Araip.3SG4B	multiple-copy	HSP20-likechaperonessuperfamilyprotein
Aradu.V0JK3	Araip.8KM9E	multiple-copy	HSP20-likechaperonessuperfamilyprotein
Aradu.10ZFH	Araip.IP580	multiple-copy	HSP20-likechaperonessuperfamilyprotein
Aradu.C7NWK	Araip.N40QT	multiple-copy	HSP20-likechaperonessuperfamilyprotein
Aradu.MD5SA	Araip.P97RG	multiple-copy	HSP20-likechaperonessuperfamilyprotein
Aradu.T8H92	Araip.AB5Q5	multiple-copy	HVA22homologueC
Aradu.7AK58	Araip.XZ72K	multiple-copy	HVA22-likeproteinG
Aradu.P8N7B	Araip.64WT2	multiple-copy	HVA22-likeproteinJ
Aradu.LH1JG	Araip.7L4MP	multiple-copy	HVA22-likeproteinJ
Aradu.KHV94	Araip.B8P7Q	multiple-copy	HVA22-likeproteinK
Aradu.GDX34	Araip.5J60P	multiple-copy	HXXXD-typeacyl-transferasefamilyprotein
Aradu.7XU9R	Araip.8Y65S	multiple-copy	HXXXD-typeacyl-transferasefamilyprotein
Aradu.5M687	Araip.A4CD7	multiple-copy	HXXXD-typeacyl-transferasefamilyprotein
Aradu.306G1	Araip.ASH11	multiple-copy	HXXXD-typeacyl-transferasefamilyprotein
Aradu.KD6QW	Araip.BJA2B	multiple-copy	HXXXD-typeacyl-transferasefamilyprotein
Aradu.X7B4K	Araip.FTC1S	multiple-copy	HXXXD-typeacyl-transferasefamilyprotein
Aradu.IS5GH	Araip.ISU30	multiple-copy	HXXXD-typeacyl-transferasefamilyprotein
Aradu.97PVW	Araip.MKZ4E	multiple-copy	HXXXD-typeacyl-transferasefamilyprotein
Aradu.9W64L	Araip.NY6BB	multiple-copy	HXXXD-typeacyl-transferasefamilyprotein
Aradu.TJM76	Araip.QVJ1V	multiple-copy	HXXXD-typeacyl-transferasefamilyprotein
Aradu.WSH4V	Araip.R1LM3	multiple-copy	HXXXD-typeacyl-transferasefamilyprotein
Aradu.E0NRM	Araip.U4SN7	multiple-copy	HXXXD-typeacyl-transferasefamilyprotein
Aradu.69JL5	Araip.X1F3R	multiple-copy	HXXXD-typeacyl-transferasefamilyprotein
Aradu.2JF44	Araip.ZR7N4	multiple-copy	HXXXD-typeacyl-transferasefamilyprotein
Aradu.M4V5T	Araip.E5BVS	multiple-copy	Hydrolase/zincionbindingproteinn=2Tax=Andropogoneae

Aradu.I4VKL	Araip.8DD0T	multiple-copy	hydrolasefamilyprotein/HAD-superfamilyprotein
Aradu.PA9WC	Araip.Y9NJG	multiple-copy	hydroxyacylglutathionehydrolase
Aradu.D0I1J	Araip.A515G	multiple-copy	hydroxymethylglutaryl-CoAsynthase-like[Glycinemax]
Aradu.9E2AM	Araip.EM2AJ	multiple-copy	hydroxyproline-richglycoproteinfamilyprotein
Aradu.HER1A	Araip.PWQ5I	multiple-copy	hydroxyproline-richglycoproteinfamilyprotein
Aradu.366M2	Araip.BYN0W	multiple-copy	IAA-aminoacidhydrolaseILR1-likeprotein
Aradu.ESU02	Araip.Q4NNC	multiple-copy	IAA-aminoacidhydrolaseILR1-likeprotein
Aradu.K952B	Araip.E510Q	multiple-copy	importin-9-like[Glycinemax]
Aradu.IM8LC	Araip.X7VZI	multiple-copy	importinnermembranetranslocasesubunitTIM44-2
Aradu.EJ09V	Araip.T2BAX	multiple-copy	importinsubunitalpha-1b
Aradu.LCC63	Araip.Z865I	multiple-copy	importinsubunitalpha-1b
Aradu.NXS8X	Araip.KQ3SG	multiple-copy	importinsubunitalpha-likeprotein
Aradu.G28T8	Araip.GE2VQ	multiple-copy	importinsubunitbeta-likeprotein
Aradu.D6JKJ	Araip.DBZ3D	multiple-copy	inactivepoly[ADP-ribose]polymeraseSRO5-likeisoformX1
Aradu.AKG36	Araip.K73B3	multiple-copy	inactivepoly[ADP-ribose]polymeraseSRO5-likeisoformX3
Aradu.R2082	Araip.DC3W4	multiple-copy	indole-3-aceticacid-amidosynthetaseGH3.9
Aradu.NGF0Q	Araip.IW8WM	multiple-copy	indole-3-aceticacid-amidosynthetaseGH3.9
Aradu.1LZ8T	Araip.PP5S8	multiple-copy	indole-3-aceticacid-amidosynthetaseGH3.9
Aradu.31IUC	Araip.TKL7Q	multiple-copy	indole-3-aceticacid-amidosynthetaseGH3.9
Aradu.PXH87	Araip.1ML5Q	multiple-copy	indole-3-aceticacidinducible14
Aradu.ANI3L	Araip.60Q0E	multiple-copy	indole-3-aceticacidinducible18
Aradu.EKX2U	Araip.AN5V8	multiple-copy	indole-3-aceticacidinducible2
Aradu.AA0QS	Araip.ED7B1	multiple-copy	indole-3-aceticacidinducible32
Aradu.M76UB	Araip.11643	multiple-copy	indole-3-aceticacidinducible9
Aradu.T65KX	Araip.47NV6	multiple-copy	indole-3-aceticacidinducible9
Aradu.JLY8Q	Araip.9V8AS	multiple-copy	indole-3-aceticacidinducible9
Aradu.K1R5H	Araip.G2N6N	multiple-copy	indole-3-aceticacidinducible9
Aradu.8PE87	Araip.38JBI	multiple-copy	innermembraneproteasesubunit-likeprotein
Aradu.LHT85	Araip.I12ZR	multiple-copy	innermembraneproteinOXA1-like
Aradu.G307L	Araip.C53V2	multiple-copy	InnermembraneproteinyicOn=2Tax=MagnaporthaceaeRe
Aradu.8Q9T1	Araip.CF54H	multiple-copy	inosine-uridinepreferringnucleosidehydrolasefamilyprotei
Aradu.N9RWR	Araip.WS5NM	multiple-copy	inosine-uridinepreferringnucleosidehydrolasefamilyprotei
Aradu.XTT89	Araip.AQR58	multiple-copy	inositol-tetrakisphosphate1-kinase1-likeisoformX1[Glycin
Aradu.DQM3T	Araip.C4SEV	multiple-copy	Integralmembranefamilyproteinn=1Tax=Populustrichocar
Aradu.RKH9D	Araip.ZG28U	multiple-copy	IntegralmembraneHPPfamilyprotein
Aradu.I5YUI	Araip.PS48V	multiple-copy	integralmembraneprotein,putative
Aradu.ZAY9P	Araip.Y23N0	multiple-copy	integralmembraneTerCfamilyprotein
Aradu.GX9A4	Araip.I1FW4	multiple-copy	IntegralmembraneYip1familyprotein
Aradu.2K3I2	Araip.A1F6W	multiple-copy	integratorcomplexsubunit4-likeisoformX1[Glycinemax]
Aradu.U795Q	Araip.W9BA5	multiple-copy	interactorofconstitutiveactiveROPs2,chloroplastic-likeisof
Aradu.W9TQV	Araip.CU8VC	multiple-copy	interactorofconstitutiveactiveROPs3isoformX6[Glycinema
Aradu.P7H9H	Araip.ATE50	multiple-copy	interactorofconstitutiveactiveROPs3-likeisoformX3[Glycin
Aradu.0AW7C	Araip.H38XD	multiple-copy	interactorofconstitutiveactiveROPs3-likeisoformX3[Glycin
Aradu.LT5WZ	Araip.T9749	multiple-copy	interactorofconstitutiveactiveROPs3-likeisoformX3[Glycin

Aradu.S0K8G	Araip.K67IB	multiple-copy	interactorofconstitutiveactiveROPs4-likeisoformX7[Glycin
Aradu.T9UI2	Araip.PAJ8W	multiple-copy	inter-alpha-trypsininhibitorheavychain-related
Aradu.0252U	Araip.HB95A	multiple-copy	IQ-DOMAIN14-likeisoformX1[Glycinemax]
Aradu.3D0ZZ	Araip.ZGL70	multiple-copy	IQ-DOMAIN14-likeisoformX1[Glycinemax]
Aradu.X9KA6	Araip.49S7T	multiple-copy	IQ-DOMAIN14-likeisoformX2[Glycinemax]
Aradu.88IFJ	Araip.QCG77	multiple-copy	IQ-DOMAIN14-likeisoformX2[Glycinemax]
Aradu.Z7K8X	Araip.V208D	multiple-copy	IQ-DOMAIN14-likeisoformX4[Glycinemax]
Aradu.6CW5S	Araip.2685R	multiple-copy	IQ-DOMAIN1-likeisoformX1[Glycinemax]
Aradu.J0ZDG	Araip.3W093	multiple-copy	IQ-DOMAIN1-likeisoformX1[Glycinemax]
Aradu.8C9N3	Araip.WZP2U	multiple-copy	IQ-DOMAIN1-likeisoformX2[Glycinemax]
Aradu.KVX0Z	Araip.BXV13	multiple-copy	IQ-DOMAIN31-likeisoformX1[Glycinemax]
Aradu.63RP0	Araip.IWK17	multiple-copy	IQ-DOMAIN31-likeisoformX9[Glycinemax]
Aradu.YX18B	Araip.S0ZCL	multiple-copy	Ironionbinding/oxidoreductase/oxidoreductaseproteinn=1
Aradu.5KS9C	Araip.WM4R3	multiple-copy	Ironionbinding/oxidoreductase/oxidoreductaseproteinn=1
Aradu.U35FJ	Araip.BEB3B	multiple-copy	Ironionbinding/oxidoreductase/oxidoreductaseproteinn=4
Aradu.00HGB	Araip.8H0EA	multiple-copy	Ironionbinding/oxidoreductasen=4Tax=CamelineaeRepID
Aradu.N60NI	Araip.N4PL2	multiple-copy	iron-sulfurclusterassemblyproteinIscA
Aradu.H3AX1	Araip.6FW03	multiple-copy	iron-sulfurclusterassemblyproteinIscU
Aradu.A0DL1	Araip.M8LL8	multiple-copy	Iron-sulfurclusterassemblyproteinn=1Tax=Coccomyxasub
Aradu.Q7KU7	Araip.YS2KW	multiple-copy	Iron-sulfurclusterassemblyproteinn=1Tax=Coccomyxasub
Aradu.02GMF	Araip.5JH12	multiple-copy	Iron-sulfurclusterassemblyproteinSufBn=4Tax=Methyloph
Aradu.MG1IB	Araip.2I1EB	multiple-copy	isochorismatasehydrolasefamilyprotein
Aradu.18V7U	Araip.G0BSA	multiple-copy	isochorismatasehydrolasefamilyprotein
Aradu.X5GZZ	Araip.KK7VF	multiple-copy	isocitratedehydrogenase
Aradu.9BY1S	Araip.A0TQ5	multiple-copy	isocitratedehydrogenase1
Aradu.SR3CA	Araip.HX4H1	multiple-copy	isocitratedehydrogenase1
Aradu.FP3UD	Araip.E335X	multiple-copy	isocitratedehydrogenasesubunit2
Aradu.A8B4S	Araip.RM8Z7	multiple-copy	isocitratedehydrogenaseV
Aradu.WQL62	Araip.X37CH	multiple-copy	isoflavonereductase-likeprotein-like[Glycinemax]
Aradu.I9SZF	Araip.YU4RI	multiple-copy	isopenicillinNepimerase-likeprotein
Aradu.Z1DQ9	Araip.PRI0M	multiple-copy	isopentenyltransferase5
Aradu.J4IUU	Araip.64KG6	multiple-copy	jasmonate-zim-domainprotein1
Aradu.VS34U	Araip.JS6GC	multiple-copy	jasmonate-zim-domainprotein12
Aradu.J3B7D	Araip.5SR4K	multiple-copy	jasmonate-zim-domainprotein6
Aradu.I5BGE	Araip.WF0FQ	multiple-copy	KDELmotif-containingprotein1-likeisoformX2[Glycinemax]
Aradu.ZG4JR	Araip.P10LD	multiple-copy	KDELmotif-containingprotein2-like[Glycinemax]
Aradu.1F5AZ	Araip.A0U1I	multiple-copy	kelchrepeatF-boxprotein
Aradu.50HDP	Araip.CI73X	multiple-copy	kelchrepeatF-boxprotein
Aradu.64PSN	Araip.LH6HR	multiple-copy	kelchrepeatF-boxprotein
Aradu.C9I9A	Araip.FI9WY	multiple-copy	Kinaseinteracting(KIP1-like)familyprotein
Aradu.DH7CT	Araip.X60H2	multiple-copy	Kinaseinteracting(KIP1-like)familyprotein
Aradu.GUH6T	Araip.Z72XG	multiple-copy	Kinaseinteracting(KIP1-like)familyprotein
Aradu.03IBL	Araip.R1CPL	multiple-copy	kinesin-4-likeisoformX4[Glycinemax]
Aradu.R9Y5X	Araip.IYS5H	multiple-copy	kinesinlightchain

Aradu.ZZU4A	Araip.XR2D0	multiple-copy	kinesinlightchain-likeisoformX2[Glycinemax]
Aradu.Y18FU	Araip.IC54M	multiple-copy	kinesinlightchain-likeisoformX1[Glycinemax]
Aradu.YCJ90	Araip.AT55H	multiple-copy	laccase10
Aradu.YCB13	Araip.Q69SP	multiple-copy	laccase10
Aradu.77ZLW	Araip.387J2	multiple-copy	laccase14
Aradu.H05SK	Araip.94M4C	multiple-copy	laccase14
Aradu.G39DJ	Araip.I0S4M	multiple-copy	laccase14
Aradu.VS07W	Araip.79RU1	multiple-copy	laccase17
Aradu.FN83T	Araip.E7ENS	multiple-copy	laccase17
Aradu.6NR0H	Araip.F26WX	multiple-copy	laccase17
Aradu.I7KI9	Araip.R7VSY	multiple-copy	laccase17
Aradu.CX05K	Araip.LT30A	multiple-copy	laccase3
Aradu.4V6Q9	Araip.VY0MP	multiple-copy	laccase3
Aradu.Z65EX	Araip.Z6UY4	multiple-copy	laccase6
Aradu.I23XS	Araip.KRV6R	multiple-copy	laccase,putative
Aradu.0C6YR	Araip.18S7T	multiple-copy	Lactoylglutathionelyase/glyoxalaseIfamilyprotein
Aradu.MW4MQ	Araip.E3P4S	multiple-copy	Lactoylglutathionelyase/glyoxalaseIfamilyprotein
Aradu.B21Z9	Araip.G25FJ	multiple-copy	Lactoylglutathionelyase/glyoxalaseIfamilyprotein
Aradu.TSZ7Q	Araip.N7CHL	multiple-copy	Lactoylglutathionelyase/glyoxalaseIfamilyprotein
Aradu.QVF0N	Araip.S11GW	multiple-copy	Lactoylglutathionelyase/glyoxalaseIfamilyprotein
Aradu.AMA0U	Araip.R0EFA	multiple-copy	LAG1longevityassurancehomolog3
Aradu.51BBB	Araip.IW1QB	multiple-copy	La-relatedprotein6isoform1n=1Tax=TheobromacacaoRe
Aradu.2F17T	Araip.W5ZI5	multiple-copy	la-relatedprotein-likeisoformX1[Glycinemax]
Aradu.413J1	Araip.GL07W	multiple-copy	L-ascorbateoxidase-like[Glycinemax]
Aradu.D3M3F	Araip.L7P2A	multiple-copy	L-ascorbateoxidase[Glycinemax]
Aradu.WM0X2	Araip.1S5XZ	multiple-copy	L-ascorbateoxidasehomolog[Glycinemax]
Aradu.NJ2WV	Araip.2P1HN	multiple-copy	L-ascorbateoxidasehomolog[Glycinemax]
Aradu.LN5YX	Araip.9V28V	multiple-copy	L-ascorbateoxidasehomolog[Glycinemax]
Aradu.M4YAD	Araip.EHL7Q	multiple-copy	L-ascorbateoxidasehomolog[Glycinemax]
Aradu.BU1C4	Araip.RNH2A	multiple-copy	L-ascorbateoxidasehomolog[Glycinemax]
Aradu.SK1HP	Araip.ZH461	multiple-copy	L-ascorbateoxidasehomolog[Glycinemax]
Aradu.AUX6R	Araip.666Q3	multiple-copy	Lateembryogenesisabundant(LEA)hydroxyproline-richgly
Aradu.883SN	Araip.GLT9E	multiple-copy	Lateembryogenesisabundant(LEA)hydroxyproline-richgly
Aradu.P7M49	Araip.J123M	multiple-copy	Lateembryogenesisabundant(LEA)hydroxyproline-richgly
Aradu.JRV68	Araip.S5KEZ	multiple-copy	Lateembryogenesisabundant(LEA)hydroxyproline-richgly
Aradu.1ZJ1C	Araip.WW8ZI	multiple-copy	Lateembryogenesisabundanthydroxyproline-richglycofam
Aradu.KTY6M	Araip.BHI10	multiple-copy	Lateembryogenesisabundant(LEA)protein
Aradu.60I66	Araip.1JG8Y	multiple-copy	Lateembryogenesisabundant(LEA)protein-related
Aradu.Z1DBI	Araip.9V1A2	multiple-copy	Lateembryogenesisabundant(LEA)protein-related
Aradu.8S28F	Araip.DF76F	multiple-copy	Lateembryogenesisabundant(LEA)protein-related
Aradu.0YE33	Araip.9A6T0	multiple-copy	Lateembryogenesisabundant3(LEA3)familyprotein
Aradu.PF378	Araip.L4IFZ	multiple-copy	Lateembryogenesisabundant(LEA)hydroxyproline-richgly
Aradu.7T62P	Araip.52Z96	multiple-copy	Lateembryogenesisabundanthydroxyproline-richglycopro
Aradu.HE8A8	Araip.0LC57	multiple-copy	Lateembryogenesisabundantprotein

Aradu.39QNU	Araip.24GT1	multiple-copy	lateembryogenesisabundantprotein
Aradu.JWG0R	Araip.HRN64	multiple-copy	lateembryogenesisabundantprotein
Aradu.VLM3K	Araip.JDU8H	multiple-copy	lateembryogenesisabundantprotein
Aradu.YG3U8	Araip.SU78D	multiple-copy	uncharacterizedprotein[Glycinemax]
Aradu.XJ1QF	Araip.UI1L0	multiple-copy	lateembryogenesisabundantprotein
Aradu.C9LG8	Araip.WRD18	multiple-copy	lateembryogenesisabundantprotein
Aradu.EH245	Araip.YZ0LP	multiple-copy	lateembryogenesisabundantprotein
Aradu.Y6LUX	Araip.84L6B	multiple-copy	Lateembryogenesisabundantprotein(LEA)familyprotein
Aradu.K9ZYN	Araip.J95X4	multiple-copy	Lateembryogenesisabundantprotein(LEA)familyprotein
Aradu.RC5C1	Araip.J6ZJ6	multiple-copy	Lateembryogenesisabundantprotein,group6
Aradu.WB5VJ	Araip.VC3BC	multiple-copy	Lecithin:cholesterolacyltransferasefamilyprotein
Aradu.71MQE	Araip.P03BP	multiple-copy	leguminosingroup485secretedpeptide
Aradu.IE711	Araip.76JTT	multiple-copy	leguminosingroup486secretedpeptide
Aradu.UH20T	Araip.GCD94	multiple-copy	leguminosingroup486secretedpeptide
Aradu.06NY9	Araip.NK3HL	multiple-copy	leguminosingroup486secretedpeptide
Aradu.KS96H	Araip.Y0ZC4	multiple-copy	leguminosingroup486secretedpeptide
Aradu.JK78S	Araip.F2E7A	multiple-copy	LEM3(ligand-effectmodulator3)familyprotein/CDC50famil
Aradu.C2W69	Araip.ITE41	multiple-copy	LEM3(ligand-effectmodulator3)familyprotein/CDC50famil
Aradu.EJ72Y	Araip.LZ9P5	multiple-copy	Leucinecarboxylmethyltransferase
Aradu.SUN9W	Araip.HY2YI	multiple-copy	leucine-richrepeatreceptor-likeproteinkinaseAt1g35710-li
Aradu.3S3UE	Araip.BB9A1	multiple-copy	Leucine-richrepeatreceptor-likeproteinkinasefamilyprotei
Aradu.V4L4B	Araip.S3E5L	multiple-copy	Leucine-richrepeatreceptor-likeproteinkinasefamilyprotei
Aradu.54DRJ	Araip.U5KWJ	multiple-copy	Leucine-richrepeatreceptor-likeproteinkinasefamilyprotei
Aradu.6FP0C	Araip.YU2P6	multiple-copy	Leucine-richrepeatreceptor-likeproteinkinasefamilyprotei
Aradu.EV8G8	Araip.5BR6I	multiple-copy	light-harvestingchlorophyllB-bindingprotein3
Aradu.KJ6HK	Araip.9C688	multiple-copy	light-harvestingchlorophyllB-bindingprotein3
Aradu.M64BQ	Araip.AW9T2	multiple-copy	light-harvestingchlorophyllB-bindingprotein3
Aradu.085I9	Araip.G8B8H	multiple-copy	light-harvestingchlorophyllB-bindingprotein3
Aradu.A2ZJG	Araip.H3LLI	multiple-copy	light-harvestingchlorophyllB-bindingprotein3
Aradu.SGR1V	Araip.JG35V	multiple-copy	light-harvestingchlorophyllB-bindingprotein3
Aradu.S4V52	Araip.N2TWA	multiple-copy	light-harvestingchlorophyllB-bindingprotein3
Aradu.ZX52Y	Araip.US2FW	multiple-copy	lightharvesting-likeprotein
Aradu.32DSM	Araip.88P7F	multiple-copy	Lipase/lipooxygenase,PLAT/LH2familyprotein
Aradu.RW91L	Araip.MC3E6	multiple-copy	Lipase/lipooxygenase,PLAT/LH2familyprotein
Aradu.M69JC	Araip.MSL5F	multiple-copy	Lipase/lipooxygenase,PLAT/LH2familyprotein
Aradu.RU466	Araip.DW1CY	multiple-copy	lipase1
Aradu.VA62W	Araip.Y67U3	multiple-copy	lipid-bindingserumglycoproteinfamilyprotein
Aradu.6NR6S	Araip.A4865	multiple-copy	lipidphosphatephosphatase2
Aradu.P7YVS	Araip.K18VU	multiple-copy	lipidphosphatephosphatase3
Aradu.M3B1E	Araip.Q09LN	multiple-copy	lipidphosphatephosphatase3
Aradu.Q6596	Araip.18UE9	multiple-copy	Lipidtransferprotein
Aradu.301P6	Araip.28HGD	multiple-copy	lipidtransferprotein
Aradu.C014T	Araip.JP66B	multiple-copy	Lipidtransferprotein
Aradu.6X61Q	Araip.K75B4	multiple-copy	Lipidtransferprotein

Aradu.117B0	Araip.KTA7Q	multiple-copy	Lipidtransferprotein
Aradu.TV5GQ	Araip.M2BYP	multiple-copy	Lipidtransferprotein
Aradu.VWQ48	Araip.MX9CS	multiple-copy	Lipidtransferprotein
Aradu.UK58V	Araip.TWB47	multiple-copy	Lipidtransferprotein
Aradu.JK25B	Araip.YD78C	multiple-copy	Lipidtransferprotein
Aradu.RV62R	Araip.YJ68E	multiple-copy	Lipidtransferprotein
Aradu.7PT52	Araip.WPA12	multiple-copy	lipidtransferprotein3
Aradu.8QE54	Araip.74FNF	multiple-copy	lipid-transferprotein13-like[Glycinemax]
Aradu.28PRF	Araip.RMX8U	multiple-copy	lipoylsynthase2,mitochondrial[Glycinemax]
Aradu.S8IWK	Araip.CD8S3	multiple-copy	LL-diaminopimelateaminotransferase
Aradu.24DGD	Araip.DE4L0	multiple-copy	LMBR1-likemembraneprotein
Aradu.R27T4	Araip.X44PT	multiple-copy	LOBdomain-containingprotein10
Aradu.HZI51	Araip.L2G8B	multiple-copy	LOBdomain-containingprotein1
Aradu.IV48P	Araip.G72RX	multiple-copy	LOBdomain-containingprotein14
Aradu.9L539	Araip.YB6JS	multiple-copy	LOBdomain-containingprotein18
Aradu.W1WQU	Araip.5R38D	multiple-copy	LOBdomain-containingprotein2
Aradu.PEH3C	Araip.8GW84	multiple-copy	LOBdomain-containingprotein20
Aradu.I83P6	Araip.I1LYJ	multiple-copy	LOBdomain-containingprotein20
Aradu.GFT3R	Araip.T1652	multiple-copy	LOBdomain-containingprotein20
Aradu.S2IZB	Araip.V9N10	multiple-copy	LOBdomain-containingprotein21
Aradu.X1PQU	Araip.T9QZJ	multiple-copy	LOBdomain-containingprotein22
Aradu.T5PPZ	Araip.YQ21T	multiple-copy	LOBdomain-containingprotein27
Aradu.SW9YM	Araip.DW1WN	multiple-copy	LOBdomain-containingprotein31
Aradu.7GX01	Araip.CF4LQ	multiple-copy	LOBdomain-containingprotein33
Aradu.0V2BM	Araip.MGF6M	multiple-copy	LOBdomain-containingprotein33
Aradu.07E2R	Araip.AF2L5	multiple-copy	LOBdomain-containingprotein38
Aradu.18TLF	Araip.TLB1K	multiple-copy	LOBdomain-containingprotein38
Aradu.F19SY	Araip.PD9M5	multiple-copy	LOBdomain-containingprotein39
Aradu.XI98N	Araip.VZF8R	multiple-copy	LOBdomain-containingprotein39
Aradu.H2K8Y	Araip.3S7QC	multiple-copy	LOBdomain-containingprotein4
Aradu.I2J7R	Araip.RJJ5V	multiple-copy	LOBdomain-containingprotein4
Aradu.X23JQ	Araip.2G7LE	multiple-copy	LOBdomain-containingprotein41
Aradu.D53YG	Araip.GK3PH	multiple-copy	LOBdomain-containingprotein41
Aradu.QH23N	Araip.90KYQ	multiple-copy	LOBdomain-containingprotein42
Aradu.I59PP	Araip.3X7VM	multiple-copy	longchainacyl-CoAsynthetase9
Aradu.W225Y	Araip.YX11Q	multiple-copy	long-chain-alcoholoxidaseFAO2-likeprotein
Aradu.J1M1P	Araip.UU70G	multiple-copy	LONGIFOLIA1-likeisoformX2[Glycinemax]
Aradu.N190Q	Araip.ADD0N	multiple-copy	LONGIFOLIA2-likeisoformX2[Glycinemax]
Aradu.SL3YH	Araip.ESV2B	multiple-copy	LONGIFOLIA2-likeisoformX2[Glycinemax]
Aradu.G31GN	Araip.W36DI	multiple-copy	LONGIFOLIA2-likeisoformX6[Glycinemax]
Aradu.GS1TU	Araip.JQ8WN	multiple-copy	LORELEI-LIKE-GPIANCHOREDPROTEIN3
Aradu.L30M6	Araip.S5WAI	multiple-copy	LORELEI-LIKE-GPIANCHOREDPROTEIN3
Aradu.9YL2E	Araip.TZQ98	multiple-copy	LRRreceptor-likekinase
Aradu.ZS0PF	Araip.Z17SR	multiple-copy	LRRreceptor-likekinase

Aradu.JS1QQ	Araip.ZY14U	multiple-copy	LRRreceptor-likekinasefamilyprotein
Aradu.I4P64	Araip.6RW05	multiple-copy	LRRreceptor-likekinasefamilyprotein
Aradu.UMP23	Araip.HL278	multiple-copy	LRRreceptor-likekinasefamilyprotein
Aradu.CN7V5	Araip.RVF4P	multiple-copy	LRRreceptor-likekinasefamilyprotein
Aradu.IJ0H9	Araip.8V7F0	multiple-copy	LRRreceptor-likeserine/threonine-proteinkinaseAt1g0684
Aradu.5U0XL	Araip.YKV9Q	multiple-copy	L-typelectin-domaincontainingreceptorkinaseIX.1-like[Gly
Aradu.7MF12	Araip.EJ1UY	multiple-copy	L-tyrosinedecarboxylase
Aradu.N289G	Araip.B6GK5	multiple-copy	Lungseventransmembranereceptorfamilyprotein
Aradu.22ZWX	Araip.IE5MN	multiple-copy	Lungseventransmembranereceptorfamilyprotein
Aradu.91M2N	Araip.MLW7G	multiple-copy	Lungseventransmembranereceptorfamilyprotein
Aradu.35WGP	Araip.NR5VL	multiple-copy	Lungseventransmembranereceptorfamilyprotein
Aradu.D4TC3	Araip.N4SC6	multiple-copy	LURP-one-related14-like[Glycinemax]
Aradu.T0R6F	Araip.FSA5T	multiple-copy	LURP-one-related15-like[Glycinemax]
Aradu.D1PNR	Araip.G8VKU	multiple-copy	LURP-one-related15-like[Glycinemax]
Aradu.S166E	Araip.L82PP	multiple-copy	LURP-one-related15-like[Glycinemax]
Aradu.QEK0G	Araip.X98AS	multiple-copy	LURP-one-related15-like[Glycinemax]
Aradu.C7QG4	Araip.ZZ77A	multiple-copy	LURP-one-related15-like[Glycinemax]
Aradu.883F2	Araip.287MP	multiple-copy	LURP-one-related4-like[Glycinemax]
Aradu.GR9T1	Araip.CN2DB	multiple-copy	lysineketoglutaratereductasetrans-splicing-likeprotein
Aradu.6U3S2	Araip.G2BMD	multiple-copy	lysine-specificdemethylaseJMJ14-likeisoformX2[Glycinem
Aradu.Q9F9Y	Araip.Z2DN9	multiple-copy	lysine-specificdemethylaseJMJ14-likeisoformX1[Glycinem
Aradu.J7LHV	Araip.69TNI	multiple-copy	lysine-specificdemethylaseJMJ14-likeisoformX4[Glycinem
Aradu.5C5XG	Araip.84IEQ	multiple-copy	lysine-specificdemethylaseJMJ14-likeisoformX4[Glycinem
Aradu.7K62W	Araip.N444E	multiple-copy	lysine-specificdemethylaseJMJ14-likeisoformX1[Glycinem
Aradu.V4R6L	Araip.36D69	multiple-copy	lysine-specificdemethylaseJMJ14-likeisoformX5[Glycinem
Aradu.ZV9WQ	Araip.F5V5K	multiple-copy	lysine-specifichistonedemethylase1homolog3-like[Glycine
Aradu.TKG0E	Araip.6J7QQ	multiple-copy	lysmdomainGPI-anchoredprotein1precursor
Aradu.MM40G	Araip.I8FLU	multiple-copy	lysmdomainGPI-anchoredprotein1precursor
Aradu.KUT1Q	Araip.G8BEX	multiple-copy	MA3domain-containingprotein
Aradu.P1TZQ	Araip.NS4X8	multiple-copy	MACPFdomain-containingproteinAt4g24290-likeisoformX
Aradu.M9TY2	Araip.CC8CA	multiple-copy	MACPFdomain-containingproteinAt4g24290-likeisoformX
Aradu.BS3D3	Araip.1IV9L	multiple-copy	MACPFdomainprotein
Aradu.W0IT8	Araip.H3SL4	multiple-copy	MACPFdomainprotein
Aradu.13TYF	Araip.NJ40E	multiple-copy	MACPFdomainprotein
Aradu.87IV3	Araip.RDY1Z	multiple-copy	MACPFdomainprotein
Aradu.1NK9R	Araip.M52V7	multiple-copy	macrophagemigrationinhibitoryfactorhomolog[Glycinemax
Aradu.WPA2I	Araip.Z6RXC	multiple-copy	macrophagemigrationinhibitoryfactorhomolog[Glycinemax
Aradu.8U64A	Araip.S4DRB	multiple-copy	MADS-boxtranscriptionfactor
Aradu.GUV6V	Araip.9M2DD	multiple-copy	MADS-boxtranscriptionfactor1[Glycinemax]
Aradu.KWJ2K	Araip.166TL	multiple-copy	MADS-boxtranscriptionfactor6[Glycinemax]
Aradu.X0I6L	Araip.38ZCB	multiple-copy	MADS-boxtranscriptionfactor6[Glycinemax]
Aradu.N9KWN	Araip.572XZ	multiple-copy	MADS-boxtranscriptionfactor6[Glycinemax]
Aradu.0Q5NS	Araip.JS7DG	multiple-copy	MADS-boxtranscriptionfactor6[Glycinemax]
Aradu.P04DI	Araip.RG64D	multiple-copy	MADS-boxtranscriptionfactor6[Glycinemax]

Aradu.NVB9V	Araip.Z7H22	multiple-copy	MADS-boxtranscriptionfactor6[Glycinemax]
Aradu.1M0CF	Araip.78XNL	multiple-copy	MADS-boxtranscriptionfactorfamilyprotein
Aradu.MY047	Araip.NR4UL	multiple-copy	MADS-boxtranscriptionfactorfamilyprotein
Aradu.UJ9WA	Araip.PB6S5	multiple-copy	MADS-boxtranscriptionfactorfamilyprotein
Aradu.22X1P	Araip.RE3SL	multiple-copy	MADS-boxtranscriptionfactorfamilyprotein
Aradu.WJ1IU	Araip.Z2S9D	multiple-copy	MADS-boxtranscriptionfactorfamilyprotein
Aradu.UG0RV	Araip.ZCR91	multiple-copy	MADS-boxtranscriptionfactorfamilyprotein
Aradu.DRU5H	Araip.I1ZW3	multiple-copy	magnesiumchelatasei2
Aradu.ZV73M	Araip.PJ399	multiple-copy	magnesiumchelatasesubunit[Glycinemax]
Aradu.XPZ1I	Araip.0V7N2	multiple-copy	magnesium-protoporphyrinIXmonomethylestercyclase
Aradu.22L4T	Araip.1LA66	multiple-copy	magnesiumtransporter3
Aradu.8C0WH	Araip.CV8RW	multiple-copy	magnesiumtransporter4
Aradu.3J25S	Araip.2JM6B	multiple-copy	magnesiumtransportermrs2n=3Tax=TriticeaeRepID=R7W
Aradu.IVB9H	Araip.706JC	multiple-copy	magnesiumtransporter7
Aradu.F70UL	Araip.5C457	multiple-copy	magnesiumtransporterNIPA2-likeisoformX1[Glycinemax]
Aradu.85WAE	Araip.6N0Z9	multiple-copy	magnesiumtransporterNIPA2-likeisoformX1[Glycinemax]
Aradu.W0WNS	Araip.H47JD	multiple-copy	magnesiumtransporterNIPA2-likeisoformX1[Glycinemax]
Aradu.VRG8M	Araip.N5RHE	multiple-copy	magnesiumtransporterNIPA2-likeisoformX1[Glycinemax]
Aradu.WGG5U	Araip.7WV1U	multiple-copy	Majorfacilitatorsuperfamilyprotein
Aradu.J5692	Araip.82HK7	multiple-copy	Majorfacilitatorsuperfamilyprotein
Aradu.6J2SN	Araip.BW1K8	multiple-copy	MajorFacilitatorSuperfamilyprotein
Aradu.24X5V	Araip.EY2N6	multiple-copy	Majorfacilitatorsuperfamilyprotein
Aradu.DN2D2	Araip.FH9AI	multiple-copy	Majorfacilitatorsuperfamilyprotein
Aradu.ZJ0E3	Araip.P8TR0	multiple-copy	Majorfacilitatorsuperfamilyprotein
Aradu.56GAP	Araip.XI1X3	multiple-copy	Majorfacilitatorsuperfamilyprotein
Aradu.034KP	Araip.GMD2H	multiple-copy	majorintrinsicprotein(MIP)familytransporter
Aradu.0NC5M	Araip.LMJ0Y	multiple-copy	majorintrinsicprotein(MIP)familytransporter
Aradu.VG4AF	Araip.M00I0	multiple-copy	majorintrinsicprotein(MIP)familytransporter
Aradu.VW5KP	Araip.RLY0Z	multiple-copy	majorintrinsicprotein(MIP)familytransporter
Aradu.0060C	Araip.U0Y4C	multiple-copy	majorintrinsicprotein(MIP)familytransporter
Aradu.R2ERN	Araip.Y41GL	multiple-copy	majorintrinsicprotein(MIP)familytransporter
Aradu.7Q1J6	Araip.9A3ZM	multiple-copy	makorinRING-zinc-fingerprotein
Aradu.N8RFP	Araip.KT3YI	multiple-copy	malatedehydrogenase
Aradu.EEF2D	Araip.2C5G8	multiple-copy	mannanendo-1,4-beta-mannosidase7-like[Glycinemax]
Aradu.HZ8ES	Araip.N7ZQV	multiple-copy	mannanendo-1,4-beta-mannosidase7-like[Glycinemax]
Aradu.3D1M6	Araip.VHD9K	multiple-copy	mannose-6-phosphateisomerase
Aradu.BUA5R	Araip.V1VLH	multiple-copy	Mannose-bindinglectinsuperfamilyprotein
Aradu.1QU40	Araip.SWI1N	multiple-copy	Mannose-P-dolicholutilizationdefect1protein
Aradu.9LK7Y	Araip.U8WPZ	multiple-copy	MARbindingfilament-likeprotein1
Aradu.FDG48	Araip.2AZ3I	multiple-copy	MATEeffluxfamilyprotein
Aradu.25XYL	Araip.3TN6J	multiple-copy	MATEeffluxfamilyprotein
Aradu.X9922	Araip.5J2NZ	multiple-copy	MATEeffluxfamilyprotein
Aradu.KY2F1	Araip.6DV57	multiple-copy	MATEeffluxfamilyprotein
Aradu.89624	Araip.6Y0I9	multiple-copy	MATEeffluxfamilyprotein

Aradu.9U2NA	Araip.83N9U	multiple-copy	MATEeffluxfamilyprotein
Aradu.L8QZK	Araip.AQN9K	multiple-copy	MATEeffluxfamilyprotein
Aradu.7L12D	Araip.BQB3L	multiple-copy	MATEeffluxfamilyprotein
Aradu.SQD67	Araip.F7NGT	multiple-copy	MATEeffluxfamilyprotein
Aradu.TE0XJ	Araip.HG9T7	multiple-copy	MATEeffluxfamilyprotein
Aradu.70AAJ	Araip.JF2JB	multiple-copy	MATEeffluxfamilyprotein
Aradu.L9ISE	Araip.N54TA	multiple-copy	MATEeffluxfamilyprotein
Aradu.2CJ0B	Araip.N63X6	multiple-copy	MATEeffluxfamilyprotein
Aradu.2X54C	Araip.R0RXM	multiple-copy	MATEeffluxfamilyprotein
Aradu.Z702T	Araip.T97LZ	multiple-copy	MATEeffluxfamilyprotein
Aradu.666C5	Araip.UC596	multiple-copy	MATEeffluxfamilyprotein
Aradu.1F4NN	Araip.ZI8DI	multiple-copy	MATEeffluxfamilyprotein
Aradu.W7LFI	Araip.W4DNX	multiple-copy	maternaleffectembryoarrest60
Aradu.N42M1	Araip.TY4ZB	multiple-copy	maternaleffectembryoarrest9
Aradu.FX47V	Araip.7A9UM	multiple-copy	MD-2-relatedlipidrecognitiondomain-containingprotein/ML
Aradu.JU5TS	Araip.VY1XN	multiple-copy	MD-2-relatedlipidrecognitiondomain-containingprotein/ML
Aradu.DH9GD	Araip.75JSE	multiple-copy	Mechanosensitiveionchannelfamilyprotein
Aradu.9B60Q	Araip.XZ9JQ	multiple-copy	Mechanosensitiveionchannelfamilyprotein
Aradu.M4U01	Araip.ZDE30	multiple-copy	mechanosensitiveionchannel-likeprotein
Aradu.A3HX3	Araip.7C73C	multiple-copy	Mechanosensitiveionchannelprotein
Aradu.4SA7Z	Araip.L5PVW	multiple-copy	mediatorofRNApolymeraseIItranscriptionsubunit12-likeisc
Aradu.A8886	Araip.89DFU	multiple-copy	mediatorofRNApolymeraseIItranscriptionsubunit15a-likeis
Aradu.A65JI	Araip.UF354	multiple-copy	mediatorofRNApolymeraseIItranscriptionsubunit15-likeisc
Aradu.R18J1	Araip.19N4V	multiple-copy	mediatorofRNApolymeraseIItranscriptionsubunit16
Aradu.9631L	Araip.122Z7	multiple-copy	mediatorofRNApolymeraseIItranscriptionsubunit20b
Aradu.N79JZ	Araip.M15ST	multiple-copy	mediatorofRNApolymeraseIItranscriptionsubunit21
Aradu.9H4V1	Araip.7I2ZN	multiple-copy	mediatorofRNApolymeraseIItranscriptionsubunit22
Aradu.VZ331	Araip.G7U46	multiple-copy	mediatorofRNApolymeraseIItranscriptionsubunit26c-likeis
Aradu.EDD1N	Araip.GY9AA	multiple-copy	mediatorofRNApolymeraseIItranscriptionsubunit28
Aradu.BR65G	Araip.A8VXL	multiple-copy	mediatorofRNApolymeraseIItranscriptionsubunit33A
Aradu.L1VA7	Araip.6C9XH	multiple-copy	mediatorofRNApolymeraseIItranscriptionsubunit36a-likeis
Aradu.4S7IS	Araip.FKP6C	multiple-copy	mediatorofRNApolymeraseIItranscriptionsubunit4-likeisof
Aradu.AH9XG	Araip.4GL4N	multiple-copy	mediatorofRNApolymeraseIItranscriptionsubunit6
Aradu.3D87V	Araip.FUG09	multiple-copy	mediatorofRNApolymeraseIItranscriptionsubunit7
Aradu.1P5GD	Araip.GAD8U	multiple-copy	MEI2-like2-likeisoformX2[Glycinemax]
Aradu.L71CJ	Araip.T16GQ	multiple-copy	membrane-associatedkinaseregulator1-like[Glycinemax]
Aradu.L6GSR	Araip.TJ04M	multiple-copy	membrane-associatedkinaseregulator5-like[Glycinemax]
Aradu.S3YJN	Araip.X2SJJ	multiple-copy	membrane-associatedkinaseregulator6-like[Glycinemax]
Aradu.5I03J	Araip.LF0TY	multiple-copy	membrane-associatedkinaseregulator1-like[Glycinemax]
Aradu.S514R	Araip.V8L3R	multiple-copy	membraneproteinAt1g16860-likeisoformX3[Glycinemax]
Aradu.P56I7	Araip.Z6W5C	multiple-copy	membraneproteinAt1g16860-likeisoformX4[Glycinemax]
Aradu.ZM868	Araip.H8E01	multiple-copy	membraneproteinAt1g16860-likeisoformX5[Glycinemax]
Aradu.WC37M	Araip.VS4IG	multiple-copy	membraneproteinAt1g16860-likeisoformX6[Glycinemax]
Aradu.LZ6ZA	Araip.J6HFZ	multiple-copy	membraneproteintypeI,putative

Aradu.JLM18	Araip.AK3ZS	multiple-copy	MembranetransporterD1n=3Tax=AndropogoneaeRepID=
Aradu.EXV8Z	Araip.CQA3P	multiple-copy	MembranetransporterD1n=3Tax=AndropogoneaeRepID=
Aradu.FUM3Y	Araip.MMB4Q	multiple-copy	MembranetransporterD1n=3Tax=AndropogoneaeRepID=
Aradu.7WG5V	Araip.NUB09	multiple-copy	MembranetransporterD1n=3Tax=AndropogoneaeRepID=
Aradu.FHT7B	Araip.XVQ2M	multiple-copy	MembranetransporterD1n=3Tax=AndropogoneaeRepID=
Aradu.S14WF	Araip.826U2	multiple-copy	Membranetransportprotein-liken=2Tax=Oryzasativasubsp
Aradu.E9AVR	Araip.KY8G4	multiple-copy	metalloendopeptidase/zincion-bindingprotein
Aradu.WXB2Z	Araip.L77XA	multiple-copy	metalloendoproteinase1-like[Glycinemax]
Aradu.XV773	Araip.T0YFD	multiple-copy	matrixmetalloproteinaseprecursor[Glycinemax]
Aradu.FJZ7Z	Araip.29BDM	multiple-copy	MetallopeptidaseM3An=1Tax=HeterobasidionirregulareT
Aradu.EX30Z	Araip.D7QWM	multiple-copy	metal-nicotianaminetransporterYSL3-likeisoformX1[Glycin
Aradu.GN6RI	Araip.LY6U4	multiple-copy	metal-nicotianaminetransporterYSL3-likeisoformX3[Glycin
Aradu.69EQ4	Araip.V2PM2	multiple-copy	metal-nicotianaminetransporterYSL3-likeisoformX3[Glycin
Aradu.X6LF1	Araip.XJS45	multiple-copy	MethionineS-adenosyltransferasen=1Tax=Detonulaconfe
Aradu.XQ1XQ	Araip.F41IP	multiple-copy	methioninesulfoxidereductaseB2
Aradu.50RNX	Araip.BUF0S	multiple-copy	methyl-CPG-bindingdomainprotein13
Aradu.R9HS1	Araip.FB0AM	multiple-copy	methyl-CpG-bindingdomain-containingprotein13-like[Glyc
Aradu.XR3QS	Araip.4G3PJ	multiple-copy	methyl-CpG-bindingdomain-containingprotein9-likeisoforn
Aradu.41J00	Araip.AC35D	multiple-copy	methylesterase17
Aradu.DM13Q	Araip.VUT3K	multiple-copy	methylesterase5
Aradu.115J9	Araip.671MH	multiple-copy	methyltransferasefamilyprotein
Aradu.P3C6F	Araip.AJ99C	multiple-copy	methyltransferasefamilyprotein
Aradu.A6BTS	Araip.H1AXV	multiple-copy	methyltransferasefamilyprotein
Aradu.40VD3	Araip.0431E	multiple-copy	Methyltransferasefamilyprotein
Aradu.FZ0TC	Araip.QN360	multiple-copy	methyltransferase-likeprotein13-likeisoformX1[Glycinema
Aradu.WA3AU	Araip.43W52	multiple-copy	methyltransferase-likeprotein1-likeisoformX3[Glycinemax
Aradu.X66TP	Araip.525WX	multiple-copy	Methyltransferase,putative,familyproteinn=7Tax=Mycoba
Aradu.Y8PPD	Araip.I4GZH	multiple-copy	methyltransferasePMT10-like[Glycinemax]
Aradu.9JQ87	Araip.BT1DS	multiple-copy	methyltransferasePMT11-like[Glycinemax]
Aradu.IBQ9G	Araip.I5637	multiple-copy	methyltransferasePMT13-like[Glycinemax]
Aradu.X9U06	Araip.VB5BT	multiple-copy	methyltransferasePMT13-like[Glycinemax]
Aradu.R9RNU	Araip.PFF61	multiple-copy	methyltransferasePMT16-like[Glycinemax]
Aradu.WAB2C	Araip.XF6W8	multiple-copy	methyltransferasePMT16-like[Glycinemax]
Aradu.VB76D	Araip.Y2F2L	multiple-copy	methyltransferasePMT16-like[Glycinemax]
Aradu.ZUH0W	Araip.YU5MB	multiple-copy	methyltransferasePMT16-like[Glycinemax]
Aradu.24I8J	Araip.3B8VN	multiple-copy	methyltransferasePMT23-like[Glycinemax]
Aradu.SS7YL	Araip.RV2U5	multiple-copy	methyltransferasePMT23-like[Glycinemax]
Aradu.1C8I8	Araip.43KWH	multiple-copy	methyltransferasePMT26-like[Glycinemax]
Aradu.7Y2HQ	Araip.87PEG	multiple-copy	methyltransferasePMT26-like[Glycinemax]
Aradu.Q2NGG	Araip.EGS0W	multiple-copy	methyltransferasePMT26-like[Glycinemax]
Aradu.Q7TCS	Araip.XKV3V	multiple-copy	methyltransferasePMT26-like[Glycinemax]
Aradu.SP7U9	Araip.34I8K	multiple-copy	methyltransferasePMT2-like[Glycinemax]
Aradu.47ARL	Araip.L4KXF	multiple-copy	methyltransferasePMT2-like[Glycinemax]
Aradu.T2QCP	Araip.Q00US	multiple-copy	methyltransferasePMT5-like[Glycinemax]

Aradu.M4PQ9	Araip.96LCB	multiple-copy	methyltransferasePMT5-like[Glycinemax]
Aradu.CJ510	Araip.YDF63	multiple-copy	methyltransferasePMT5-likeisoformX2[Glycinemax]
Aradu.28N0X	Araip.UDU9G	multiple-copy	methyltransferasetype11
Aradu.GPA6U	Araip.60EAB	multiple-copy	mevalonatekinase
Aradu.3589Z	Araip.8M1JQ	multiple-copy	microtubule-associatedproteinfutschisoformX9[Glycinema
Aradu.5D1Y3	Araip.YYL10	multiple-copy	microtubule-associatedproteinfutschisoformX8[Glycinema
Aradu.XE4CL	Araip.RZ75Q	multiple-copy	microtubule-associatedproteins65-1
Aradu.6Y1H7	Araip.0AR95	multiple-copy	microtubule-associatedproteins70-5
Aradu.E2VTX	Araip.XH9B3	multiple-copy	microtubule-associatedproteins70-5
Aradu.CMZ2S	Araip.333ZF	multiple-copy	microtubule-associatedproteinTORTIFOLIA1-likeisoformX
Aradu.T83RP	Araip.39QP6	multiple-copy	microtubule-associatedproteinTORTIFOLIA1-likeisoformX
Aradu.E3SP0	Araip.V9RCS	multiple-copy	microtubule-associatedproteinTORTIFOLIA1-likeisoformX
Aradu.H802Y	Araip.R7R05	multiple-copy	microtubuleendbindingproteinEB1A
Aradu.5B6UX	Araip.6FE68	multiple-copy	Mitochondrialglycoproteinfamilyprotein
Aradu.3JN5I	Araip.DQG9G	multiple-copy	Mitochondrialglycoproteinfamilyprotein
Aradu.0ZB6D	Araip.IX0TZ	multiple-copy	Mitochondrialglycoproteinfamilyprotein
Aradu.Q1F6L	Araip.BJ799	multiple-copy	MitochondrialimportinnermembranetranslocasesubunitTir
Aradu.7LR28	Araip.C16FL	multiple-copy	MitochondrialimportinnermembranetranslocasesubunitTir
Aradu.S7WZG	Araip.T7PRK	multiple-copy	MitochondrialimportinnermembranetranslocasesubunitTir
Aradu.G0ZCH	Araip.U6HL7	multiple-copy	MitochondrialimportinnermembranetranslocasesubunitTir
Aradu.VHJ0W	Araip.VE1A8	multiple-copy	mitochondrialimportinnermembranetranslocasesubunitTII
Aradu.669IL	Araip.S1XQK	multiple-copy	MitochondrialimportinnermembranetranslocasesubunitTII
Aradu.BE2IC	Araip.V2UYE	multiple-copy	mitochondrialoutermembraneproteinporin1-like[Glycinem
Aradu.SK8YF	Araip.CL7PQ	multiple-copy	mitochondrialphosphatecarrierprotein3,mitochondrial-like
Aradu.ZR9ZC	Araip.RV92P	multiple-copy	mitochondrialphosphatecarrierprotein3,mitochondrial-like
Aradu.WBJ0E	Araip.I0LNV	multiple-copy	Mitochondrialsubstratecarrierfamilyprotein
Aradu.RLX6T	Araip.QNY9B	multiple-copy	Mitochondrialsubstratecarrierfamilyprotein
Aradu.XU2Q6	Araip.V35VM	multiple-copy	Mitochondrialsubstratecarrierfamilyprotein
Aradu.TI9C9	Araip.W4E4J	multiple-copy	Mitochondrialsubstratecarrierfamilyprotein
Aradu.C4BMG	Araip.08H2V	multiple-copy	mitochondrialsubstratecarrierfamilyproteinB-like[Glycinem
Aradu.R2E4D	Araip.1G1M0	multiple-copy	mitochondrialsubstratecarrierfamilyproteinB-like[Glycinem
Aradu.UEG36	Araip.2Z354	multiple-copy	mitochondrialsubstratecarrierfamilyproteinB-like[Glycinem
Aradu.D48W8	Araip.DIA58	multiple-copy	mitochondrialsubstratecarrierfamilyproteinB-like[Glycinem
Aradu.E1VP1	Araip.EVL3M	multiple-copy	mitochondrialsubstratecarrierfamilyproteinB-like[Glycinem
Aradu.IN2S6	Araip.H6TT6	multiple-copy	mitochondrialsubstratecarrierfamilyproteinB-like[Glycinem
Aradu.F510W	Araip.IFK0L	multiple-copy	mitochondrialsubstratecarrierfamilyproteinB-like[Glycinem
Aradu.34FHG	Araip.KRU21	multiple-copy	mitochondrialsubstratecarrierfamilyproteinB-like[Glycinem
Aradu.PZ3X7	Araip.KSS1C	multiple-copy	mitochondrialsubstratecarrierfamilyproteinB-like[Glycinem
Aradu.BY27U	Araip.M8P59	multiple-copy	mitochondrialsubstratecarrierfamilyproteinB-like[Glycinem
Aradu.HC4GG	Araip.MLK6S	multiple-copy	mitochondrialsubstratecarrierfamilyproteinB-like[Glycinem
Aradu.T0NTP	Araip.Q8342	multiple-copy	mitochondrialsubstratecarrierfamilyproteinB-like[Glycinem
Aradu.9K72K	Araip.SM08A	multiple-copy	mitochondrialsubstratecarrierfamilyproteinB-like[Glycinem
Aradu.94Q3R	Araip.W932V	multiple-copy	mitochondrialsubstratecarrierfamilyproteinB-like[Glycinem
Aradu.W34NY	Araip.WYG4Z	multiple-copy	mitochondrialsubstratecarrierfamilyproteinB-like[Glycinem

Aradu.25F61	Araip.X8TMK	multiple-copy	mitochondrialsubstratecarrierfamilyproteinB-like[Glycinem
Aradu.24ZSJ	Araip.Y9V2V	multiple-copy	mitochondrialsubstratecarrierfamilyproteinB-like[Glycinem
Aradu.YWC0U	Araip.A1KWI	multiple-copy	mitochondrialsubstratecarrierfamilyproteinB-likeisoformX1
Aradu.145AN	Araip.613HG	multiple-copy	mitochondrialsubstratecarrierfamilyproteinC-like[Glycinem
Aradu.QVX65	Araip.86BCN	multiple-copy	Mitochondrialtranscriptionterminationfactorfamilyprotein
Aradu.BV67Y	Araip.U15FR	multiple-copy	mitoticcheckpointserine/threonine-proteinkinaseBUB1-like
Aradu.EU0BW	Araip.HRR0E	multiple-copy	MLO-likeprotein4-like[Glycinemax]
Aradu.27DYI	Araip.PW5DF	multiple-copy	MLO-likeprotein8-like[Glycinemax]
Aradu.0YR51	Araip.SD8AP	multiple-copy	MLOproteinhomolog1-like[Glycinemax]
Aradu.TZ5MC	Araip.ITT7M	multiple-copy	MLP-likeprotein423
Aradu.XAD2K	Araip.K0TZJ	multiple-copy	MLP-likeprotein43
Aradu.5E20L	Araip.AZ7RZ	multiple-copy	molybdenumcofactorsulfurase-like[Glycinemax]
Aradu.L86SE	Araip.81A8L	multiple-copy	monogalactosyldiacylglycerolsynthase2
Aradu.M5W9D	Araip.9NP15	multiple-copy	Mpsonebinderkinaseactivator-likeprotein1A
Aradu.V34QS	Araip.PA1WM	multiple-copy	mRNAcappingenzymefamilyprotein
Aradu.YE325	Araip.ZX87X	multiple-copy	mucin-2-like[Glycinemax]
Aradu.Y1KM6	Araip.FPC0F	multiple-copy	Myb/SANT-likeDNA-bindingdomainprotein
Aradu.C1C0K	Araip.I8L3V	multiple-copy	Myb/SANT-likeDNA-bindingdomainprotein
Aradu.LSK5Q	Araip.JKN5G	multiple-copy	Myb/SANT-likeDNA-bindingdomainprotein
Aradu.HB1CW	Araip.KM10P	multiple-copy	Myb/SANT-likeDNA-bindingdomainprotein
Aradu.VRW76	Araip.T00RM	multiple-copy	Myb/SANT-likeDNA-bindingdomainprotein
Aradu.7A20D	Araip.T2WNF	multiple-copy	Myb/SANT-likeDNA-bindingdomainprotein
Aradu.AT44H	Araip.D1N24	multiple-copy	mybfamilytranscriptionfactorAPL-likeisoformX1[Glycinem
Aradu.P4XEI	Araip.H36I7	multiple-copy	mybfamilytranscriptionfactorAPL-likeisoformX1[Glycinem
Aradu.WQ4BK	Araip.L6DF6	multiple-copy	mybfamilytranscriptionfactorAPL-likeisoformX1[Glycinem
Aradu.9Z8LC	Araip.YAF9X	multiple-copy	mybfamilytranscriptionfactorAPL-likeisoformX1[Glycinem
Aradu.WHH4F	Araip.04SWW	multiple-copy	mybfamilytranscriptionfactorAPL-likeisoformX3[Glycinem
Aradu.RN1PL	Araip.8YC75	multiple-copy	mybfamilytranscriptionfactorAPL-likeisoformX3[Glycinem
Aradu.HR3NA	Araip.CV4V1	multiple-copy	mybfamilytranscriptionfactorAPL-likeisoformX3[Glycinem
Aradu.Y7NUP	Araip.FD7VR	multiple-copy	mybfamilytranscriptionfactorAPL-likeisoformX3[Glycinem
Aradu.3ZC2Z	Araip.B91MN	multiple-copy	MybfamilytranscriptionfactorAt1g14600-likeisoformX2[Gly
Aradu.VP5WD	Araip.LA5WR	multiple-copy	MybfamilytranscriptionfactorAt1g14600-likeisoformX2[Gly
Aradu.2A606	Araip.MY1RF	multiple-copy	MybfamilytranscriptionfactorAt1g14600-likeisoformX2[Gly
Aradu.0BT33	Araip.ZD4VN	multiple-copy	MybfamilytranscriptionfactorAt1g14600-likeisoformX2[Gly
Aradu.5E0HP	Araip.1C050	multiple-copy	MyblikeDNA-bindingdomaincontainingproteinn=1Tax=Aca
Aradu.B78K2	Araip.GQ95T	multiple-copy	MyblikeDNA-bindingdomaincontainingproteinn=1Tax=Aca
Aradu.4707K	Araip.Q1JDY	multiple-copy	myb-likeDNA-bindingdomainprotein
Aradu.AZ535	Araip.5836D	multiple-copy	myb-likeproteinI-likeisoformX2[Glycinemax]
Aradu.C7JRZ	Araip.194YI	multiple-copy	myb-likeproteinX-like[Glycinemax]
Aradu.9P5M8	Araip.4YN76	multiple-copy	myb-likeproteinX-likeisoformX2[Glycinemax]
Aradu.RQK7Y	Araip.T9C3C	multiple-copy	myb-likeproteinX-likeisoformX2[Glycinemax]
Aradu.ZV8PI	Araip.U6RZ5	multiple-copy	mybtranscriptionfactor
Aradu.30RPU	Araip.07BW1	multiple-copy	mybtranscriptionfactor
Aradu.U7UZ6	Araip.18QQE	multiple-copy	mybtranscriptionfactor

Aradu.XW987	Araip.1Z8DE	multiple-copy	mybtranscriptionfactor
Aradu.AT974	Araip.24TKU	multiple-copy	mybtranscriptionfactor
Aradu.X7LBF	Araip.2H669	multiple-copy	mybtranscriptionfactor
Aradu.90QVH	Araip.2I7WK	multiple-copy	mybtranscriptionfactor
Aradu.K3GZ3	Araip.3E954	multiple-copy	mybtranscriptionfactor
Aradu.0Z09C	Araip.A49CU	multiple-copy	mybtranscriptionfactor
Aradu.T6HSI	Araip.C77KQ	multiple-copy	mybtranscriptionfactor
Aradu.G7UXI	Araip.EKK4C	multiple-copy	mybtranscriptionfactor
Aradu.IFP6S	Araip.EMV9L	multiple-copy	mybtranscriptionfactor
Aradu.40GCA	Araip.F8RKD	multiple-copy	mybtranscriptionfactor
Aradu.X0QAS	Araip.FZ3N9	multiple-copy	mybtranscriptionfactor
Aradu.BNM3L	Araip.GU31N	multiple-copy	mybtranscriptionfactor
Aradu.9Q62W	Araip.J7793	multiple-copy	mybtranscriptionfactor
Aradu.I4Z8N	Araip.K719G	multiple-copy	mybtranscriptionfactor
Aradu.Z1QR7	Araip.KHB2Z	multiple-copy	mybtranscriptionfactor
Aradu.862GA	Araip.KL088	multiple-copy	mybtranscriptionfactor
Aradu.X8I5Q	Araip.L0W9L	multiple-copy	mybtranscriptionfactor
Aradu.NSL0R	Araip.MJ3JY	multiple-copy	mybtranscriptionfactor
Aradu.EJ00N	Araip.N3NU2	multiple-copy	mybtranscriptionfactor
Aradu.V69Z9	Araip.NEN8H	multiple-copy	mybtranscriptionfactor
Aradu.CM6S6	Araip.P53WL	multiple-copy	mybtranscriptionfactor
Aradu.G26I2	Araip.PUS3S	multiple-copy	mybtranscriptionfactor
Aradu.A9NSU	Araip.RVR18	multiple-copy	mybtranscriptionfactor
Aradu.HJZ2Z	Araip.S31IS	multiple-copy	mybtranscriptionfactor
Aradu.ND06J	Araip.SUR5V	multiple-copy	mybtranscriptionfactor
Aradu.545W2	Araip.T0DQ5	multiple-copy	mybtranscriptionfactor
Aradu.2Z52F	Araip.TJ2TW	multiple-copy	mybtranscriptionfactor
Aradu.L25DZ	Araip.U6PZK	multiple-copy	mybtranscriptionfactor
Aradu.CW3NA	Araip.U94IX	multiple-copy	mybtranscriptionfactor
Aradu.9DD9N	Araip.V8F5Z	multiple-copy	mybtranscriptionfactor
Aradu.CT448	Araip.VH6HT	multiple-copy	mybtranscriptionfactor
Aradu.GFS4B	Araip.W5H5G	multiple-copy	mybtranscriptionfactor
Aradu.4D7HL	Araip.YZ7MJ	multiple-copy	mybtranscriptionfactor
Aradu.RJ0UE	Araip.ZP5MJ	multiple-copy	mybtranscriptionfactor
Aradu.74X9S	Araip.2XW30	multiple-copy	MYBtranscriptionfactorMYB109[Glycinemax]
Aradu.RB14Z	Araip.VE967	multiple-copy	MYBtranscriptionfactorMYB114isoformX2[Glycinemax]
Aradu.H5GIM	Araip.TF81A	multiple-copy	MYBtranscriptionfactorMYB118isoformX1[Glycinemax]
Aradu.K49J7	Araip.13WJ1	multiple-copy	MYBtranscriptionfactorMYB127[Glycinemax]
Aradu.K8V1Y	Araip.0AG3E	multiple-copy	MYBtranscriptionfactorMYB48[Glycinemax]
Aradu.D0JN5	Araip.DD2QX	multiple-copy	MYBtranscriptionfactorMYB48[Glycinemax]
Aradu.U6514	Araip.J0GUS	multiple-copy	MYBtranscriptionfactorMYB48[Glycinemax]
Aradu.07167	Araip.MY084	multiple-copy	MYBtranscriptionfactorMYB92[Glycinemax]
Aradu.K3TU7	Araip.Q811U	multiple-copy	MYBtranscriptionfactorMYB48[Glycinemax]
Aradu.J967R	Araip.TFG1J	multiple-copy	MYBtranscriptionfactorMYB48[Glycinemax]

Aradu.DW9XI	Araip.TSY02	multiple-copy	MYBtranscriptionfactorMYB48[Glycinemax]
Aradu.9MQ2E	Araip.9P5VU	multiple-copy	MYBtranscriptionfactorMYB51[Glycinemax]
Aradu.NI44V	Araip.1M3VN	multiple-copy	MYBtranscriptionfactorMYB57[Glycinemax]
Aradu.Z4AN4	Araip.M0P25	multiple-copy	MYBtranscriptionfactorMYB57[Glycinemax]
Aradu.C0E4H	Araip.J6KI9	multiple-copy	MYBtranscriptionfactorMYB60[Glycinemax]
Aradu.JGT0L	Araip.KEX5D	multiple-copy	MYBtranscriptionfactorMYB60[Glycinemax]
Aradu.NB41M	Araip.L417Q	multiple-copy	MYBtranscriptionfactorMYB62[Glycinemax]
Aradu.PJ07U	Araip.788L8	multiple-copy	MYBtranscriptionfactorMYB64[Glycinemax]
Aradu.IR2D4	Araip.97FYI	multiple-copy	MYBtranscriptionfactorMYB64[Glycinemax]
Aradu.QAZ4U	Araip.LSW71	multiple-copy	MYBtranscriptionfactorMYB64[Glycinemax]
Aradu.NN536	Araip.Q1LNW	multiple-copy	MYBtranscriptionfactorMYB64[Glycinemax]
Aradu.7H2PW	Araip.ZMP4R	multiple-copy	mybtranscriptionfactor
Aradu.IC1L6	Araip.45253	multiple-copy	MYBtranscriptionfactorMYB98[Glycinemax]
Aradu.0Z207	Araip.FL1CB	multiple-copy	myo-inositoloxygenase4
Aradu.UA393	Araip.6L8N8	multiple-copy	myosin-11-likeisoformX3[Glycinemax]
Aradu.I7LA7	Araip.CX9DW	multiple-copy	myosin2
Aradu.L4E7U	Araip.H61BH	multiple-copy	myosin2
Aradu.DE4QW	Araip.3S8CW	multiple-copy	myosin-4-likeisoformX2[Glycinemax]
Aradu.41DJI	Araip.ZE4M6	multiple-copy	myosin-5-like[Glycinemax]
Aradu.CC9MM	Araip.QL2Q5	multiple-copy	myosin-9-likeisoformX8[Glycinemax]
Aradu.09JHP	Araip.E0PG3	multiple-copy	myosinheavychain-related
Aradu.AW46C	Araip.I3PYA	multiple-copy	myosinheavychain-related
Aradu.SP3D0	Araip.LDM4X	multiple-copy	myosinheavychain-related
Aradu.T46DH	Araip.MMC70	multiple-copy	myosinheavychain-related
Aradu.AA7LT	Araip.S49L1	multiple-copy	myosinheavychain-related
Aradu.M2KYK	Araip.88EKE	multiple-copy	Myosinheavychain-relatedprotein
Aradu.FQ3N4	Araip.B7GXE	multiple-copy	Myosinheavychain-relatedprotein
Aradu.2447B	Araip.FX6KA	multiple-copy	Myosinheavychain-relatedprotein
Aradu.7JY58	Araip.PE4FX	multiple-copy	Myosinheavychain-relatedprotein
Aradu.NVM7U	Araip.YM3WQ	multiple-copy	Myosinheavychain-relatedprotein
Aradu.GB0MR	Araip.HQ3TB	multiple-copy	myosin-likeprotein
Aradu.47ZJ4	Araip.CCF9L	multiple-copy	myosin-related
Aradu.54IZY	Araip.Y0L6Q	multiple-copy	N2,N2-dimethylguanosinetRNAmethyltransferase
Aradu.1AJ4F	Araip.ZUP60	multiple-copy	NACdomaincontainingprotein15
Aradu.YIQ80	Araip.DL86S	multiple-copy	NACdomaincontainingprotein19
Aradu.46U1T	Araip.ZX5IX	multiple-copy	NACdomaincontainingprotein28
Aradu.Z5H58	Araip.333QY	multiple-copy	NACdomaincontainingprotein3
Aradu.13D06	Araip.U9RGH	multiple-copy	NACdomaincontainingprotein35
Aradu.5D5JN	Araip.2W5R5	multiple-copy	NACdomaincontainingprotein62
Aradu.BPK98	Araip.A6QWC	multiple-copy	NACdomaincontainingprotein62
Aradu.M7213	Araip.G3ZLR	multiple-copy	NACdomaincontainingprotein76
Aradu.EP425	Araip.71CS3	multiple-copy	NACdomaincontainingprotein82
Aradu.215DG	Araip.1N7IP	multiple-copy	NACdomain-containingprotein8-like[Glycinemax]
Aradu.F8VRL	Araip.1Z0SD	multiple-copy	NACdomain-containingprotein8-like[Glycinemax]

Aradu.XJF09	Araip.31EFM	multiple-copy	NACdomain-containingprotein8-like[Glycinemax]
Aradu.7NI41	Araip.9MG9F	multiple-copy	NACdomain-containingprotein8-like[Glycinemax]
Aradu.Z4K97	Araip.E0NQ0	multiple-copy	NACdomain-containingprotein8-like[Glycinemax]
Aradu.BS3JU	Araip.J93FI	multiple-copy	NACdomain-containingprotein8-like[Glycinemax]
Aradu.N8MU8	Araip.609WS	multiple-copy	NACdomainprotein,
Aradu.M9GL4	Araip.64GCN	multiple-copy	NACdomainprotein,
Aradu.USH95	Araip.78PTT	multiple-copy	NACdomainprotein,
Aradu.W3GLH	Araip.79TDF	multiple-copy	NACdomainprotein,
Aradu.6H4PP	Araip.92BTQ	multiple-copy	NACdomainprotein,
Aradu.KF8UQ	Araip.9BR1Z	multiple-copy	NACdomainprotein,
Aradu.U974Q	Araip.9W6SR	multiple-copy	NACdomainprotein,
Aradu.Z9Y3J	Araip.AVV74	multiple-copy	NACdomainprotein,
Aradu.F6Z4G	Araip.F5AGL	multiple-copy	NACdomainprotein,
Aradu.M8PFR	Araip.F8I62	multiple-copy	NACdomainprotein,
Aradu.Y1DM8	Araip.HYM8C	multiple-copy	NACdomainprotein,
Aradu.0MJ0X	Araip.J9WH5	multiple-copy	NACdomainprotein,
Aradu.RP61F	Araip.PNX61	multiple-copy	NACdomainprotein,
Aradu.F2DT2	Araip.Q3R6H	multiple-copy	NACdomainprotein,
Aradu.79PL2	Araip.R0657	multiple-copy	NACdomainprotein,
Aradu.L8SVN	Araip.T6ICI	multiple-copy	NACdomainprotein,
Aradu.22647	Araip.UA0W9	multiple-copy	NACdomainprotein,
Aradu.ETZ8K	Araip.X2KK1	multiple-copy	NACdomainprotein,
Aradu.L3QY1	Araip.XK9AB	multiple-copy	NACdomainprotein,
Aradu.ZT2TE	Araip.XQA0A	multiple-copy	NACdomainprotein,
Aradu.ILC9H	Araip.71XEY	multiple-copy	N-acetyltransferaseHLS1-like[Glycinemax]
Aradu.Z0DB5	Araip.7L870	multiple-copy	N-acetyltransferaseHLS1-like[Glycinemax]
Aradu.CD22H	Araip.CGA2X	multiple-copy	N-acetyltransferaseHLS1-like[Glycinemax]
Aradu.YC13W	Araip.Q2Z6R	multiple-copy	N-acetyltransferaseHLS1-like[Glycinemax]
Aradu.W0ILV	Araip.01CXT	multiple-copy	NAD(P)-bindingRossmann-foldsuperfamilyprotein
Aradu.F0EGY	Araip.BHW2G	multiple-copy	NAD(P)-bindingRossmann-foldsuperfamilyprotein
Aradu.I9PC5	Araip.BPS80	multiple-copy	NAD(P)-bindingRossmann-foldsuperfamilyprotein
Aradu.S0XYN	Araip.J8XTW	multiple-copy	NAD(P)-bindingRossmann-foldsuperfamilyprotein
Aradu.KG1H9	Araip.JK8BE	multiple-copy	NAD(P)-bindingRossmann-foldsuperfamilyprotein
Aradu.T19XF	Araip.N8HQ9	multiple-copy	NAD(P)-bindingRossmann-foldsuperfamilyprotein
Aradu.V6UVG	Araip.T0QWF	multiple-copy	NAD(P)-bindingRossmann-foldsuperfamilyprotein
Aradu.BP0GU	Araip.T5DLA	multiple-copy	NAD(P)-bindingRossmann-foldsuperfamilyprotein
Aradu.VI2RS	Araip.U0RZI	multiple-copy	NAD(P)-bindingRossmann-foldsuperfamilyprotein
Aradu.C881Z	Araip.UBP04	multiple-copy	NAD(P)-bindingRossmann-foldsuperfamilyprotein
Aradu.P0IKP	Araip.G27IP	multiple-copy	NAD(P)H-quinoneoxidoreductasesubunitNn=1Tax=Syne
Aradu.V1L1H	Araip.RB3WS	multiple-copy	NAD-dependentepimerase/dehydratasefamilyprotein
Aradu.SB3IS	Araip.GE5YY	multiple-copy	NAD-dependentepimerase/dehydratasen=1Tax=Calothri
Aradu.XR2K7	Araip.C95P2	multiple-copy	NAD(P)-bindingRossmann-foldsuperfamilyprotein
Aradu.XAX6Y	Araip.Y3JJL	multiple-copy	NAD-dependentglycerol-3-phosphatedehydrogenasefami
Aradu.P6639	Araip.G1WPG	multiple-copy	NAD-dependentmalicenzyme2

Aradu.S5K3Z	Araip.J5GX8	multiple-copy	NAD-dependentmalicenzyme1
Aradu.0C9TU	Araip.JZ063	multiple-copy	NAD-dependentmalicenzyme1
Aradu.1JK1L	Araip.Q3Y4B	multiple-copy	NAD-dependentmalicenzyme1
Aradu.S89VI	Araip.S9I8N	multiple-copy	NAD-dependentmalicenzyme2
Aradu.I24YI	Araip.2I89L	multiple-copy	NADHdehydrogenase(Ubiquinone)1alphasubcomplexsub
Aradu.5G7S4	Araip.LC3XS	multiple-copy	NADHdehydrogenase(ubiquinone)complexI,assemblyfac
Aradu.E9DCA	Araip.QE5ZB	multiple-copy	NADHdehydrogenase[ubiquinone]1alphasubcomplexass
Aradu.SZ07F	Araip.B1VRH	multiple-copy	NADHdehydrogenase[ubiquinone]1alphasubcomplexsub
Aradu.1N9W3	Araip.4U5VW	multiple-copy	NADHdehydrogenase[ubiquinone]iron-sulfurprotein
Aradu.6ZR5R	Araip.Y4SLP	multiple-copy	NADHdehydrogenase1alphasubcomplexsubunit13n=2Ta
Aradu.N2WYB	Araip.9F4Q1	multiple-copy	NADHdehydrogenase1alphasubcomplexsubunit5n=2Tax
Aradu.QBK5E	Araip.G34L7	multiple-copy	NADH-ubiquinoneoxidoreductase51kDasubunit
Aradu.R4RRW	Araip.WVB13	multiple-copy	NADH-ubiquinoneoxidoreductasechainn=1Tax=Medicag
Aradu.I5LRV	Araip.3A5RC	multiple-copy	NADP-dependentalkenaldoublebondreductaseP1
Aradu.U5A8Y	Araip.09CWU	multiple-copy	NADPH-dependentthioredoxinreductaseC
Aradu.R205B	Araip.X9FAS	multiple-copy	N-alpha-acetyltransferase35,NatCauxiliarysubunit
Aradu.1D399	Araip.QI1EB	multiple-copy	NBS1
Aradu.R8LXW	Araip.ED9LT	multiple-copy	NCdomain-containingprotein-related
Aradu.QM05Z	Araip.FL6S0	multiple-copy	NCdomain-containingprotein-related
Aradu.HG1BY	Araip.C64ZH	multiple-copy	NDHdependentflow6
Aradu.B0NST	Araip.G5X76	multiple-copy	NHLdomain-containingprotein
Aradu.94BIF	Araip.D15G9	multiple-copy	ninja-familyproteinmc410
Aradu.D8TZD	Araip.L5RT4	multiple-copy	ninja-familyproteinmc410
Aradu.J6GHV	Araip.PKX1N	multiple-copy	ninja-familyproteinmc410
Aradu.75AIB	Araip.Z0XNP	multiple-copy	ninja-familyproteinmc410
Aradu.LVP8M	Araip.WP2AA	multiple-copy	NIPA-likeprotein3-likeisoformX1[Glycinemax]
Aradu.L2RSA	Araip.WS2K0	multiple-copy	nitratetransporter1.5
Aradu.YRI3B	Araip.FT1CW	multiple-copy	nitratetransporter1.7
Aradu.P24XG	Araip.09AXD	multiple-copy	nitratetransporter1:2
Aradu.F297C	Araip.Q7NLU	multiple-copy	peptidetransporter2
Aradu.Y97H0	Araip.TJ4P5	multiple-copy	nitratetransporter1:2
Aradu.CD2E5	Araip.TJC4C	multiple-copy	nitratetransporter1:2
Aradu.L4IQ2	Araip.Y7S1I	multiple-copy	nitratetransporter1:2
Aradu.M2B10	Araip.NYT7M	multiple-copy	nitratetransporter2.2
Aradu.A7934	Araip.SS53P	multiple-copy	nitratetransporter2.4
Aradu.P59ZT	Araip.XKH8U	multiple-copy	nitrilase-likeprotein1
Aradu.H9FC2	Araip.Y6K7H	multiple-copy	Nodulin-like/MajorFacilitatorSuperfamilyprotein
Aradu.GE4T4	Araip.69H3W	multiple-copy	nodulinMtN21/EamA-liketransporterfamilyprotein
Aradu.W71HY	Araip.6S6W2	multiple-copy	nodulinMtN21/EamA-liketransporterfamilyprotein
Aradu.43YX5	Araip.8G316	multiple-copy	nodulinMtN21/EamA-liketransporterfamilyprotein
Aradu.1IK7L	Araip.9FT0W	multiple-copy	NodulinMtN21/EamA-liketransporterfamilyprotein
Aradu.570RS	Araip.AV3G9	multiple-copy	nodulinMtN21/EamA-liketransporterfamilyprotein
Aradu.AP9TH	Araip.DX4S4	multiple-copy	nodulinMtN21/EamA-liketransporterfamilyprotein
Aradu.1E1V6	Araip.F1PS1	multiple-copy	nodulinMtN21/EamA-liketransporterfamilyprotein

Aradu.FK3YK	Araip.GAB7J	multiple-copy	nodulinMtN21/EamA-liketransporterfamilyprotein
Aradu.TY9X1	Araip.HXZ5T	multiple-copy	nodulinMtN21/EamA-liketransporterfamilyprotein
Aradu.DTW5Z	Araip.LC2PG	multiple-copy	nodulinMtN21/EamA-liketransporterfamilyprotein
Aradu.PQ5HC	Araip.N4GPP	multiple-copy	nodulinMtN21/EamA-liketransporterfamilyprotein
Aradu.DCQ49	Araip.NHI3F	multiple-copy	nodulinMtN21/EamA-liketransporterfamilyprotein
Aradu.BUW4Z	Araip.P0TGF	multiple-copy	nodulinMtN21/EamA-liketransporterfamilyprotein
Aradu.7HS2D	Araip.PTL0N	multiple-copy	nodulinMtN21/EamA-liketransporterfamilyprotein
Aradu.0VN2L	Araip.RNE4C	multiple-copy	nodulinMtN21/EamA-liketransporterfamilyprotein
Aradu.W2IGQ	Araip.SB79H	multiple-copy	nodulinMtN21/EamA-liketransporterfamilyprotein
Aradu.G6GR7	Araip.TQY0D	multiple-copy	nodulinMtN21/EamA-liketransporterfamilyprotein
Aradu.0A17C	Araip.TVH0X	multiple-copy	nodulinMtN21/EamA-liketransporterfamilyprotein
Aradu.N3EHS	Araip.UVY31	multiple-copy	nodulinMtN21/EamA-liketransporterfamilyprotein
Aradu.E5ATX	Araip.W9LI3	multiple-copy	nodulinMtN21/EamA-liketransporterfamilyprotein
Aradu.ST5ZJ	Araip.X1ZFF	multiple-copy	nodulinMtN21/EamA-liketransporterfamilyprotein
Aradu.YY5C4	Araip.ZY9Q1	multiple-copy	nodulinMtN21/EamA-liketransporterfamilyprotein
Aradu.69JCH	Araip.6544P	multiple-copy	Non-specificlipid-transferprotein,putative
Aradu.IFF56	Araip.B5WS3	multiple-copy	Non-specificlipid-transferprotein,putative
Aradu.18FWJ	Araip.FIV2R	multiple-copy	Non-specificlipid-transferprotein,putative
Aradu.L66NF	Araip.PR3IV	multiple-copy	Non-specificlipid-transferprotein,putative
Aradu.RL2YG	Araip.XV7Z4	multiple-copy	Non-specificlipid-transferprotein,putative
Aradu.W2UK2	Araip.79E4D	multiple-copy	non-specificphospholipaseC3
Aradu.M2IMN	Araip.TF5RD	multiple-copy	non-specificphospholipaseC6
Aradu.G09L8	Araip.97TWR	multiple-copy	notumhomologisoformX1[Glycinemax]
Aradu.DKE60	Araip.ZYZ4W	multiple-copy	notumhomologisoformX1[Glycinemax]
Aradu.G47J2	Araip.PKQ0A	multiple-copy	notumhomologisoformX2[Glycinemax]
Aradu.L2YDJ	Araip.73QKW	multiple-copy	novelplantsnare13
Aradu.GB4V7	Araip.LM2NC	multiple-copy	NRAMPmetaliontransporter2
Aradu.IRQ7V	Araip.SYI2E	multiple-copy	NRAMPmetaliontransporter2
Aradu.74IXL	Araip.4SP4E	multiple-copy	NRAMPmetaliontransporter6
Aradu.H447Q	Araip.85BT6	multiple-copy	N-terminalnucleophileaminohydrolases(Ntnhydrolases)su
Aradu.0BJ82	Araip.A2GBD	multiple-copy	nuclearfactorY,subunitC4
Aradu.S0YHI	Araip.P48NJ	multiple-copy	nuclearfactorY,subunitC3
Aradu.AL6NG	Araip.9BI0F	multiple-copy	nuclearmatrixconstituentprotein-related
Aradu.GTZ9I	Araip.M1B8E	multiple-copy	nuclearmatrixconstituentprotein-related
Aradu.MBU55	Araip.D8NFL	multiple-copy	nuclearmovementfamilyprotein
Aradu.2YY9U	Araip.QHR4V	multiple-copy	nuclearporecomplexproteinNup160-likeisoformX1[Glycine
Aradu.98K4P	Araip.CMA8P	multiple-copy	nuclearporecomplexproteinNup98-Nup96-like[Glycinema
Aradu.6L7FC	Araip.FLC8C	multiple-copy	nuclearporecomplexproteinNup98-Nup96-likeisoformX2[G
Aradu.1EI01	Araip.FJ25G	multiple-copy	nuclearporecomplexprotein-related
Aradu.0KQ9H	Araip.YS13X	multiple-copy	nucleartranscriptionfactorYsubunitA-3-likeisoformX4[Glyc
Aradu.V66GG	Araip.JJ5F2	multiple-copy	nucleartranscriptionfactorYsubunitA-7-likeisoformX3[Glyc
Aradu.S37LQ	Araip.T9DPL	multiple-copy	nucleartranscriptionfactorYsubunitB-10-like[Glycinemax]
Aradu.M66BW	Araip.6F7K8	multiple-copy	nucleartranscriptionfactorYsubunitB-2[Glycinemax]
Aradu.U6V8H	Araip.9X806	multiple-copy	Nucleartransportfactor2(NTF2)familyprotein

Aradu.C4WL2	Araip.KP2HT	multiple-copy	Nucleartransportfactor2(NTF2)familyprotein
Aradu.LVR9T	Araip.P34Y4	multiple-copy	Nucleartransportfactor2(NTF2)familyprotein
Aradu.72C6M	Araip.W027Q	multiple-copy	Nucleartransportfactor2(NTF2)familyprotein
Aradu.AZC7U	Araip.QR2AA	multiple-copy	nucleartransportfactor2B
Aradu.2T909	Araip.KVQ91	multiple-copy	Nucleicacid-binding,OB-fold-likeprotein
Aradu.D17LB	Araip.U2B8S	multiple-copy	Nucleicacid-binding,OB-fold-likeprotein
Aradu.NH66H	Araip.VB4KC	multiple-copy	Nucleicacid-binding,OB-fold-likeprotein
Aradu.N5A68	Araip.R0KZE	multiple-copy	NucleicacidbindingandAminoacyl-tRNAsynthetasedomain
Aradu.52M2A	Araip.Y3D52	multiple-copy	nucleicacid-bindingprotein,putative
Aradu.XZ1FD	Araip.J4WHX	multiple-copy	Nucleicacidbindingprotein,putativen=1Tax=Ricinuscomm
Aradu.64RBA	Araip.Z40TR	multiple-copy	Nucleicacidbindingprotein,putativen=1Tax=Ricinuscomm
Aradu.366AT	Araip.VX6AQ	multiple-copy	nucleobase-ascorbatetransporter12
Aradu.5MH0W	Araip.3LR0U	multiple-copy	nucleobase-ascorbatetransporter7
Aradu.875VG	Araip.887KB	multiple-copy	nucleobase-ascorbatetransporter7
Aradu.Z7W7G	Araip.FC5PA	multiple-copy	nucleobase-ascorbatetransporter7
Aradu.YAN03	Araip.J1GUG	multiple-copy	nucleobase-ascorbatetransporter7
Aradu.AA5ZE	Araip.Q85BZ	multiple-copy	nucleobase-ascorbatetransporter7
Aradu.58JJ3	Araip.S8804	multiple-copy	nucleobase-ascorbatetransporter7
Aradu.P3XD1	Araip.X50KN	multiple-copy	nucleobase-ascorbatetransporter7
Aradu.ECB76	Araip.U2S9Q	multiple-copy	NucleolarGTP-bindingprotein
Aradu.U6RYQ	Araip.JM4UY	multiple-copy	nucleolarprotein6-likeisoformX2[Glycinemax]
Aradu.36VCP	Araip.6BC4J	multiple-copy	Nucleoporininteractingcomponent(Nup93/Nic96-like)famil
Aradu.F9JAF	Araip.SS61C	multiple-copy	nucleoporinNUP53-likeisoformX2[Glycinemax]
Aradu.CKD1V	Araip.Q4YAV	multiple-copy	nucleoporinseh1-likeprotein
Aradu.UR64R	Araip.IF6H8	multiple-copy	Nucleosidediphosphatekinasefamilyprotein
Aradu.RC9DQ	Araip.I11J5	multiple-copy	nucleosomeassemblyprotein1
Aradu.95DDC	Araip.WP65K	multiple-copy	Nucleotide/sugartransporterfamilyprotein
Aradu.J32IT	Araip.16EI2	multiple-copy	nucleotidebinding
Aradu.G44IN	Araip.L1TDI	multiple-copy	nucleotidebinding
Aradu.JG252	Araip.SCN43	multiple-copy	nucleotidebinding
Aradu.D9N4K	Araip.WQ00Y	multiple-copy	nucleotidebinding
Aradu.U564S	Araip.00G1J	multiple-copy	Nucleotide-diphospho-sugartransferasefamilyprotein
Aradu.QU5N8	Araip.MIY8R	multiple-copy	Nucleotide-diphospho-sugartransferasefamilyprotein
Aradu.Z2UA0	Araip.QTW3F	multiple-copy	Nucleotide-diphospho-sugartransferasefamilyprotein
Aradu.2TK0M	Araip.VTQ3M	multiple-copy	Nucleotide-diphospho-sugartransferasefamilyprotein
Aradu.1EC42	Araip.1S1ZZ	multiple-copy	Nucleotide-sugartransporterfamilyprotein
Aradu.R8CMR	Araip.RD2P3	multiple-copy	Nucleotide-sugartransporterfamilyprotein
Aradu.E4WB9	Araip.H5EJ2	multiple-copy	nucleotidesugartransporter-KT1
Aradu.9TU4F	Araip.YF63Z	multiple-copy	nudixhydrolasehomolog17
Aradu.NS8UC	Araip.B03QY	multiple-copy	nudixhydrolasehomolog2
Aradu.31RFE	Araip.E5NHA	multiple-copy	nudixhydrolasehomolog2
Aradu.40JGL	Araip.R4838	multiple-copy	nudixhydrolasehomolog2
Aradu.ADH9Y	Araip.9NY3L	multiple-copy	nudixhydrolasehomolog20
Aradu.X37CM	Araip.4UA5Y	multiple-copy	nudixhydrolasehomolog25

Aradu.ZC6SM	Araip.EQA46	multiple-copy	nudixhydrolasehomolog3
Aradu.M8UTW	Araip.M692U	multiple-copy	nudixhydrolasehomolog3
Aradu.ZTH8V	Araip.37TY6	multiple-copy	nudixhydrolasehomolog4
Aradu.U9SCT	Araip.0YS5Y	multiple-copy	nudixhydrolasehomolog8
Aradu.D43UE	Araip.7A0S5	multiple-copy	O-acyltransferase(WSD1-like)familyprotein
Aradu.NT54G	Araip.Z6NL1	multiple-copy	O-acyltransferase(WSD1-like)familyprotein
Aradu.74JTE	Araip.ZW937	multiple-copy	O-acyltransferase(WSD1-like)familyprotein
Aradu.YRE5K	Araip.F4XUG	multiple-copy	OBERON3-likeisoformX1[Glycinemax]
Aradu.8M51F	Araip.SA9TP	multiple-copy	OBERON4-like[Glycinemax]
Aradu.NL7A8	Araip.2H61M	multiple-copy	OBERON-likeprotein-likeisoformX6[Glycinemax]
Aradu.U8M4U	Araip.6YK9P	multiple-copy	Octicosapeptide/Phox/Bem1p(PB1)domain-containingpro
Aradu.A9V55	Araip.I6XYE	multiple-copy	Octicosapeptide/Phox/Bem1p(PB1)domain-containingpro
Aradu.50IFA	Araip.MR0T1	multiple-copy	Octicosapeptide/Phox/Bem1pfamilyprotein
Aradu.XX5Y6	Araip.SRP5E	multiple-copy	Octicosapeptide/Phox/Bem1pfamilyprotein
Aradu.15KNS	Araip.QCA7D	multiple-copy	O-fucosyltransferasefamilyprotein
Aradu.T2I1E	Araip.J3PJ2	multiple-copy	O-methyltransferase1
Aradu.LA1WX	Araip.Z8IVG	multiple-copy	O-methyltransferase1
Aradu.J1ZY0	Araip.09YU8	multiple-copy	O-methyltransferasefamilyprotein
Aradu.Y6TV9	Araip.91PPR	multiple-copy	O-methyltransferasefamilyprotein
Aradu.2QX0T	Araip.94GCY	multiple-copy	O-methyltransferasefamilyprotein
Aradu.38TXW	Araip.987DS	multiple-copy	O-methyltransferasefamilyprotein
Aradu.P5H21	Araip.BYS6X	multiple-copy	O-methyltransferasefamilyprotein
Aradu.P6RI2	Araip.EY5S9	multiple-copy	O-methyltransferasefamilyprotein
Aradu.K52EF	Araip.HY6IN	multiple-copy	O-methyltransferasefamilyprotein
Aradu.BM2KZ	Araip.P8FYF	multiple-copy	O-methyltransferasefamilyprotein
Aradu.F84AW	Araip.U1V6F	multiple-copy	O-methyltransferasefamilyprotein
Aradu.X3YIU	Araip.WDY6V	multiple-copy	O-methyltransferasefamilyprotein
Aradu.JB8ME	Araip.Z39M8	multiple-copy	O-methyltransferasefamilyprotein
Aradu.S0NBF	Araip.S398P	multiple-copy	organiccation/carnitinetransporter3
Aradu.8482Y	Araip.S8IS2	multiple-copy	organ-specificproteinS2-like[Glycinemax]
Aradu.SA9NZ	Araip.VR8J9	multiple-copy	originrecognitioncomplexprotein6
Aradu.K9R11	Araip.K222Y	multiple-copy	originrecognitioncomplexsubunit4
Aradu.GY8D2	Araip.W1HNL	multiple-copy	ORMDLfamilyprotein
Aradu.A2ZB2	Araip.F2LMB	multiple-copy	OTU-likecysteineproteasefamilyprotein
Aradu.LUI60	Araip.K5VKJ	multiple-copy	OTU-likecysteineprotease
Aradu.8GE4T	Araip.Z3FUF	multiple-copy	OTU-likecysteineproteasefamilyprotein
Aradu.W7CVC	Araip.8E97X	multiple-copy	OTU-likecysteineproteasefamilyprotein
Aradu.Q2SWK	Araip.H4PLS	multiple-copy	OTU-likecysteineproteasefamilyprotein
Aradu.SY0M5	Araip.HIR6V	multiple-copy	Outerarmdyneinlightchain1protein
Aradu.JGE04	Araip.QE1HM	multiple-copy	outermembraneOMP85familyprotein
Aradu.TJ47H	Araip.B0W5P	multiple-copy	Outermembraneprotein/protectiveantigenOMA87n=1Tax=
Aradu.3D1TM	Araip.RR6HX	multiple-copy	Outermembraneprotein/protectiveantigenOMA87n=1Tax=
Aradu.DWJ8H	Araip.I2FQ2	multiple-copy	Outwardrectifyingpotassiumchannelprotein
Aradu.DJ2MU	Araip.LFA0H	multiple-copy	Outwardrectifyingpotassiumchannelprotein

Aradu.Q0PGE	Araip.R9REP	multiple-copy	Outwardrectifyingpotassiumchannelprotein
Aradu.94ZD7	Araip.R5QTL	multiple-copy	ovatefamilyprotein3
Aradu.K81IQ	Araip.K7SM4	multiple-copy	ovatefamilyprotein5
Aradu.2GB7A	Araip.IS8T3	multiple-copy	oxidoreductase,2OG-Fe(II)oxygenasefamilyprotein
Aradu.F3FT3	Araip.JEN04	multiple-copy	oxidoreductase,2OG-Fe(II)oxygenasefamilyprotein
Aradu.10XKC	Araip.LWT8I	multiple-copy	oxidoreductase,2OG-Fe(II)oxygenasefamilyprotein
Aradu.NZM5S	Araip.RG980	multiple-copy	oxidoreductase,2OG-Fe(II)oxygenasefamilyprotein
Aradu.9VB1R	Araip.42ERT	multiple-copy	Oxidoreductase,zinc-bindingdehydrogenasefamilyprotein
Aradu.X4DG3	Araip.AEN35	multiple-copy	oxidoreductase,zinc-bindingdehydrogenasefamilyprotein
Aradu.DH828	Araip.P3SU7	multiple-copy	Oxidoreductase,zinc-bindingdehydrogenasefamilyprotein
Aradu.P0XL9	Araip.R00CW	multiple-copy	Oxidoreductase,zinc-bindingdehydrogenasefamilyprotein
Aradu.7U8XS	Araip.Y8LZ2	multiple-copy	Oxidoreductase,zinc-bindingdehydrogenasefamilyprotein
Aradu.L9JI8	Araip.6AJ5V	multiple-copy	oxidoreductase/transitionmetalion-bindingprotein
Aradu.9AT7R	Araip.ZN6CK	multiple-copy	oxidoreductasefamily,NAD-bindingrossmannfoldprotein
Aradu.F6CTF	Araip.5J63B	multiple-copy	Oxidoreductasefamilyprotein
Aradu.E2BAC	Araip.R3BYA	multiple-copy	Oxidoreductasefamilyprotein
Aradu.4H615	Araip.U8TWE	multiple-copy	Oxidoreductasefamilyprotein
Aradu.KJ041	Araip.62MB6	multiple-copy	oxygen-evolvingenhancerprotein
Aradu.SJ887	Araip.63HRP	multiple-copy	oxygen-evolvingenhancerprotein
Aradu.FL0YZ	Araip.M93U4	multiple-copy	oxygen-evolvingenhancerprotein
Aradu.Y8LHL	Araip.PR7LI	multiple-copy	oxygen-evolvingenhancerprotein
Aradu.VB0PQ	Araip.MJM6V	multiple-copy	Oxysterol-bindingfamilyprotein
Aradu.K16RE	Araip.NV86K	multiple-copy	Oxysterol-bindingfamilyprotein
Aradu.U3GYE	Araip.UB259	multiple-copy	Oxysterol-bindingfamilyprotein
Aradu.60S2U	Araip.ZB3XA	multiple-copy	Oxysterol-bindingfamilyprotein
Aradu.5Q0IP	Araip.X8LEZ	multiple-copy	pantothenatekinase2
Aradu.UEM5G	Araip.HCG81	multiple-copy	Papainfamilycysteineprotease
Aradu.XHT7Q	Araip.U4GJJ	multiple-copy	PAT1homolog1-likeisoformX1[Glycinemax]
Aradu.8X6ET	Araip.1C4CW	multiple-copy	PATATIN-likeprotein4
Aradu.0M8VI	Araip.72WPA	multiple-copy	PATATIN-likeprotein6
Aradu.B7XG0	Araip.95KUY	multiple-copy	patatin-likeprotein6
Aradu.YW2J0	Araip.U23Q6	multiple-copy	PATATIN-likeprotein6
Aradu.W162D	Araip.BU3C8	multiple-copy	PATATIN-likeprotein9
Aradu.5Y6Q8	Araip.W5EI6	multiple-copy	PATATIN-likeprotein9
Aradu.7NM14	Araip.IM1XC	multiple-copy	patellin-3-likeisoformX3[Glycinemax]
Aradu.A36AV	Araip.QB7B2	multiple-copy	pathogenesis-likeprotein
Aradu.ZP2GZ	Araip.W54L5	multiple-copy	pathogenesis-likeprotein
Aradu.0M5LW	Araip.85A5N	multiple-copy	pathogenesis-relatedhomeodomainprotein-likeisoformX2[
Aradu.4KD1L	Araip.4PY6A	multiple-copy	Pathogenesis-relatedthaumatinsuperfamilyprotein
Aradu.T1ZRM	Araip.6L6QM	multiple-copy	Pathogenesis-relatedthaumatinsuperfamilyprotein
Aradu.UTB08	Araip.899GP	multiple-copy	Pathogenesis-relatedthaumatinsuperfamilyprotein
Aradu.U66TG	Araip.BG3YM	multiple-copy	Pathogenesis-relatedthaumatinsuperfamilyprotein
Aradu.9G825	Araip.DN365	multiple-copy	Pathogenesis-relatedthaumatinsuperfamilyprotein
Aradu.E26DL	Araip.FLW58	multiple-copy	Pathogenesis-relatedthaumatinsuperfamilyprotein

Aradu.Z84L6	Araip.UQT8V	multiple-copy	Pathogenesis-relatedthaumatinsuperfamilyprotein
Aradu.0IR2T	Araip.VJ4I5	multiple-copy	Pathogenesis-relatedthaumatinsuperfamilyprotein
Aradu.UZ3UX	Araip.VX3FG	multiple-copy	Pathogenesis-relatedthaumatinsuperfamilyprotein
Aradu.N68GL	Araip.Y9BTG	multiple-copy	Pathogenesis-relatedthaumatinsuperfamilyprotein
Aradu.D6W9N	Araip.YJ85H	multiple-copy	Pathogenesis-relatedthaumatinsuperfamilyprotein
Aradu.W0R38	Araip.YBS5U	multiple-copy	pathogenictypeIIIeffectoravirulencefactorAvrAvrRpt-cleav
Aradu.Q78JW	Araip.0ZP55	multiple-copy	Pectatelyasefamilyprotein
Aradu.26288	Araip.27UQJ	multiple-copy	Pectatelyasefamilyprotein
Aradu.8CG1C	Araip.2X3C3	multiple-copy	Pectatelyasefamilyprotein
Aradu.TEF34	Araip.6ZH5D	multiple-copy	Pectatelyasefamilyprotein
Aradu.R0TCR	Araip.BNI9P	multiple-copy	Pectatelyasefamilyprotein
Aradu.W6JL4	Araip.F5941	multiple-copy	Pectatelyasefamilyprotein
Aradu.I1RXA	Araip.L7Z01	multiple-copy	Pectatelyasefamilyprotein
Aradu.0M3HI	Araip.N5J1U	multiple-copy	Pectatelyasefamilyprotein
Aradu.UDU6Q	Araip.5Z2JS	multiple-copy	pectinesterase/pectinesteraseinhibitor21-like[Glycinemax
Aradu.X5JSA	Araip.TZY7L	multiple-copy	pectinesterase/pectinesteraseinhibitor22[Glycinemax]
Aradu.945F4	Araip.J385S	multiple-copy	pectinesterase/pectinesteraseinhibitor24-like[Glycinemax
Aradu.346IW	Araip.ZGL25	multiple-copy	pectinesterase/pectinesteraseinhibitor24-like[Glycinemax
Aradu.TF2FR	Araip.EK6VJ	multiple-copy	pectinesterase/pectinesteraseinhibitor33-like[Glycinemax
Aradu.PU85I	Araip.F5HYI	multiple-copy	pectinesterase/pectinesteraseinhibitor34-like[Glycinemax
Aradu.3P5HB	Araip.PB3YQ	multiple-copy	pectinesterase/pectinesteraseinhibitor34-like[Glycinemax
Aradu.RDK4X	Araip.X6DZU	multiple-copy	pectinesterase/pectinesteraseinhibitor36-like[Glycinemax
Aradu.S28UE	Araip.6NP7W	multiple-copy	pectinesterase/pectinesteraseinhibitor41-like[Glycinemax
Aradu.VIF27	Araip.U4P9E	multiple-copy	pectinesterase/pectinesteraseinhibitor41-like[Glycinemax
Aradu.YE6PU	Araip.T8YPZ	multiple-copy	pectinesterase/pectinesteraseinhibitor6-like[Glycinemax]
Aradu.4U6IV	Araip.X62XD	multiple-copy	pectinesterase/pectinesteraseinhibitor7-like[Glycinemax]
Aradu.0S3RJ	Araip.S3E2A	multiple-copy	pectinesterase/pectinesteraseinhibitor-like[Glycinemax]
Aradu.8VI8X	Araip.1BL7S	multiple-copy	pectinesterase11
Aradu.BE5FD	Araip.3CP6N	multiple-copy	pectinesterase11
Aradu.ZZ3IQ	Araip.9Q46C	multiple-copy	pectinesterase11
Aradu.S226I	Araip.A0AYC	multiple-copy	pectinesterase11
Aradu.5Y4IB	Araip.AB04Q	multiple-copy	pectinesterase11
Aradu.B7FB5	Araip.GXP3H	multiple-copy	pectinesterase11
Aradu.6011J	Araip.JB5WF	multiple-copy	pectinesterase11
Aradu.K7HN9	Araip.XDY2R	multiple-copy	pectinesterase11
Aradu.90B63	Araip.0J6W3	multiple-copy	Pentatricopeptiderepeat(PPR)superfamilyprotein
Aradu.RAU4M	Araip.9L7ER	multiple-copy	Pentatricopeptiderepeat(PPR)superfamilyprotein
Aradu.NT394	Araip.9LU3E	multiple-copy	Pentatricopeptiderepeat(PPR)superfamilyprotein
Aradu.XGC1C	Araip.E0XSF	multiple-copy	Pentatricopeptiderepeat(PPR)superfamilyprotein
Aradu.Q6RE5	Araip.F73NM	multiple-copy	Pentatricopeptiderepeat(PPR)superfamilyprotein
Aradu.01YLW	Araip.J49D4	multiple-copy	Pentatricopeptiderepeat(PPR)superfamilyprotein
Aradu.Y7357	Araip.LGH1R	multiple-copy	Pentatricopeptiderepeat(PPR)superfamilyprotein
Aradu.GSM33	Araip.M4B28	multiple-copy	Pentatricopeptiderepeat(PPR)superfamilyprotein
Aradu.A77EC	Araip.MW8R0	multiple-copy	Pentatricopeptiderepeat(PPR)superfamilyprotein

Aradu.35NHM	Araip.N2G5P	multiple-copy	Pentatricopeptiderepeat(PPR)superfamilyprotein
Aradu.43TNC	Araip.N9GVK	multiple-copy	Pentatricopeptiderepeat(PPR)superfamilyprotein
Aradu.T7MUN	Araip.00Q3S	multiple-copy	Pentatricopeptiderepeat(PPR)superfamilyprotein
Aradu.3L1P7	Araip.0QI44	multiple-copy	Pentatricopeptiderepeat(PPR)superfamilyprotein
Aradu.A68HE	Araip.1X10D	multiple-copy	Pentatricopeptiderepeat(PPR)superfamilyprotein
Aradu.K9162	Araip.21S30	multiple-copy	Pentatricopeptiderepeat(PPR)superfamilyprotein
Aradu.HD7PF	Araip.2A5U1	multiple-copy	Pentatricopeptiderepeat(PPR)superfamilyprotein
Aradu.B4Y17	Araip.2RW57	multiple-copy	Pentatricopeptiderepeat(PPR)superfamilyprotein
Aradu.W5INW	Araip.357WQ	multiple-copy	Pentatricopeptiderepeat(PPR)superfamilyprotein
Aradu.517PS	Araip.3JG6V	multiple-copy	Pentatricopeptiderepeat(PPR)superfamilyprotein
Aradu.VI7D5	Araip.3W07G	multiple-copy	Pentatricopeptiderepeat(PPR)superfamilyprotein
Aradu.IDJ29	Araip.40FIW	multiple-copy	Pentatricopeptiderepeat(PPR)superfamilyprotein
Aradu.W2MG5	Araip.423MS	multiple-copy	Pentatricopeptiderepeat(PPR)superfamilyprotein
Aradu.E516Y	Araip.44DMF	multiple-copy	Pentatricopeptiderepeat(PPR)superfamilyprotein
Aradu.00CSZ	Araip.483YA	multiple-copy	Pentatricopeptiderepeat(PPR)superfamilyprotein
Aradu.T7EMW	Araip.4DP2E	multiple-copy	Pentatricopeptiderepeat(PPR)superfamilyprotein
Aradu.GB1JK	Araip.4E6MH	multiple-copy	Pentatricopeptiderepeat(PPR)superfamilyprotein
Aradu.DN87S	Araip.4L5CB	multiple-copy	Pentatricopeptiderepeat(PPR)superfamilyprotein
Aradu.M2JBM	Araip.4V1C3	multiple-copy	Pentatricopeptiderepeat(PPR)superfamilyprotein
Aradu.BWE80	Araip.51HAH	multiple-copy	Pentatricopeptiderepeat(PPR)superfamilyprotein
Aradu.Z34FG	Araip.69UD7	multiple-copy	Pentatricopeptiderepeat(PPR)superfamilyprotein
Aradu.772YU	Araip.6J4W8	multiple-copy	Pentatricopeptiderepeat(PPR)superfamilyprotein
Aradu.WWY9R	Araip.6SM8U	multiple-copy	Pentatricopeptiderepeat(PPR)superfamilyprotein
Aradu.JF030	Araip.7G62B	multiple-copy	Pentatricopeptiderepeat(PPR)superfamilyprotein
Aradu.H44CN	Araip.802QV	multiple-copy	Pentatricopeptiderepeat(PPR)superfamilyprotein
Aradu.CXC2W	Araip.80D4Q	multiple-copy	Pentatricopeptiderepeat(PPR)superfamilyprotein
Aradu.AA8NU	Araip.856EY	multiple-copy	Pentatricopeptiderepeat(PPR)superfamilyprotein
Aradu.TN7XF	Araip.89I2E	multiple-copy	Pentatricopeptiderepeat(PPR)superfamilyprotein
Aradu.X9YRF	Araip.8N9UM	multiple-copy	Pentatricopeptiderepeat(PPR)superfamilyprotein
Aradu.27WCK	Araip.8R0Z7	multiple-copy	Pentatricopeptiderepeat(PPR)superfamilyprotein
Aradu.02NM4	Araip.91RV8	multiple-copy	Pentatricopeptiderepeat(PPR)superfamilyprotein
Aradu.DXG5L	Araip.96GUU	multiple-copy	Pentatricopeptiderepeat(PPR)superfamilyprotein
Aradu.X4G0F	Araip.97W0E	multiple-copy	Pentatricopeptiderepeat(PPR)superfamilyprotein
Aradu.A6T5Y	Araip.98QEF	multiple-copy	Pentatricopeptiderepeat(PPR)superfamilyprotein
Aradu.V1TEZ	Araip.9K7D6	multiple-copy	Pentatricopeptiderepeat(PPR)superfamilyprotein
Aradu.Y478Z	Araip.A32IS	multiple-copy	Pentatricopeptiderepeat(PPR)superfamilyprotein
Aradu.R2TG0	Araip.A3GFE	multiple-copy	Pentatricopeptiderepeat(PPR)superfamilyprotein
Aradu.PK4QW	Araip.A9NYP	multiple-copy	Pentatricopeptiderepeat(PPR)superfamilyprotein
Aradu.YI7L8	Araip.AEB43	multiple-copy	Pentatricopeptiderepeat(PPR)superfamilyprotein
Aradu.IA5E9	Araip.AP2S4	multiple-copy	Pentatricopeptiderepeat(PPR)superfamilyprotein
Aradu.RR75T	Araip.BCQ7T	multiple-copy	Pentatricopeptiderepeat(PPR)superfamilyprotein
Aradu.B925F	Araip.BG0H4	multiple-copy	Pentatricopeptiderepeat(PPR)superfamilyprotein
Aradu.RZY5Q	Araip.BK6T6	multiple-copy	Pentatricopeptiderepeat(PPR)superfamilyprotein
Aradu.E1Q5N	Araip.BN4UN	multiple-copy	Pentatricopeptiderepeat(PPR)superfamilyprotein

Aradu.Q9JNX	Araip.BZS09	multiple-copy	Pentatricopeptiderepeat(PPR)superfamilyprotein
Aradu.LXU7Y	Araip.C8B17	multiple-copy	Pentatricopeptiderepeat(PPR)superfamilyprotein
Aradu.FK4M3	Araip.CJI78	multiple-copy	Pentatricopeptiderepeat(PPR)superfamilyprotein
Aradu.KH49T	Araip.CK6AG	multiple-copy	Pentatricopeptiderepeat(PPR)superfamilyprotein
Aradu.FNM4G	Araip.CY8BI	multiple-copy	Pentatricopeptiderepeat(PPR)superfamilyprotein
Aradu.HUV25	Araip.D59I5	multiple-copy	Pentatricopeptiderepeat(PPR)superfamilyprotein
Aradu.UT07K	Araip.E028Y	multiple-copy	Pentatricopeptiderepeat(PPR)superfamilyprotein
Aradu.T82FQ	Araip.E02KZ	multiple-copy	Pentatricopeptiderepeat(PPR)superfamilyprotein
Aradu.S2S1T	Araip.EH219	multiple-copy	Pentatricopeptiderepeat(PPR)superfamilyprotein
Aradu.GPN3U	Araip.F9LX7	multiple-copy	Pentatricopeptiderepeat(PPR)superfamilyprotein
Aradu.73S33	Araip.FW2CL	multiple-copy	Pentatricopeptiderepeat(PPR)superfamilyprotein
Aradu.0K11B	Araip.G4MI2	multiple-copy	Pentatricopeptiderepeat(PPR)superfamilyprotein
Aradu.MZ5HW	Araip.GXD6K	multiple-copy	Pentatricopeptiderepeat(PPR)superfamilyprotein
Aradu.1ZL4G	Araip.H1ACH	multiple-copy	Pentatricopeptiderepeat(PPR)superfamilyprotein
Aradu.YF3HA	Araip.HG4BM	multiple-copy	Pentatricopeptiderepeat(PPR)superfamilyprotein
Aradu.GZ5DT	Araip.HUB63	multiple-copy	Pentatricopeptiderepeat(PPR)superfamilyprotein
Aradu.U987Q	Araip.I4GQV	multiple-copy	Pentatricopeptiderepeat(PPR)superfamilyprotein
Aradu.98KS9	Araip.I51KZ	multiple-copy	Pentatricopeptiderepeat(PPR)superfamilyprotein
Aradu.BB9NS	Araip.IA5QC	multiple-copy	Pentatricopeptiderepeat(PPR)superfamilyprotein
Aradu.HP3G1	Araip.IN00Y	multiple-copy	Pentatricopeptiderepeat(PPR)superfamilyprotein
Aradu.XSZ7L	Araip.J3G2M	multiple-copy	Pentatricopeptiderepeat(PPR)superfamilyprotein
Aradu.M4TMQ	Araip.J9YKA	multiple-copy	Pentatricopeptiderepeat(PPR)superfamilyprotein
Aradu.LBJ9G	Araip.JA75T	multiple-copy	Pentatricopeptiderepeat(PPR)superfamilyprotein
Aradu.R4B3S	Araip.JQ9KH	multiple-copy	Pentatricopeptiderepeat(PPR)superfamilyprotein
Aradu.V66GY	Araip.K8YAU	multiple-copy	Pentatricopeptiderepeat(PPR)superfamilyprotein
Aradu.R3AGE	Araip.LBK80	multiple-copy	Pentatricopeptiderepeat(PPR)superfamilyprotein
Aradu.66MNC	Araip.LSP61	multiple-copy	Pentatricopeptiderepeat(PPR)superfamilyprotein
Aradu.T55NY	Araip.M1JS2	multiple-copy	Pentatricopeptiderepeat(PPR)superfamilyprotein
Aradu.P7UBS	Araip.N813Z	multiple-copy	Pentatricopeptiderepeat(PPR)superfamilyprotein
Aradu.A61EY	Araip.P4KQP	multiple-copy	Pentatricopeptiderepeat(PPR)superfamilyprotein
Aradu.TF0TM	Araip.PAE7Y	multiple-copy	Pentatricopeptiderepeat(PPR)superfamilyprotein
Aradu.JQD1M	Araip.PY0XX	multiple-copy	Pentatricopeptiderepeat(PPR)superfamilyprotein
Aradu.DU86V	Araip.Q821Q	multiple-copy	Pentatricopeptiderepeat(PPR)superfamilyprotein
Aradu.7MV2V	Araip.QE1NF	multiple-copy	Pentatricopeptiderepeat(PPR)superfamilyprotein
Aradu.ZI2XT	Araip.R0C6X	multiple-copy	Pentatricopeptiderepeat(PPR)superfamilyprotein
Aradu.LVF07	Araip.R19K2	multiple-copy	Pentatricopeptiderepeat(PPR)superfamilyprotein
Aradu.FA1DK	Araip.RPP7Y	multiple-copy	Pentatricopeptiderepeat(PPR)superfamilyprotein
Aradu.5JX5D	Araip.RX3N3	multiple-copy	Pentatricopeptiderepeat(PPR)superfamilyprotein
Aradu.C5AKB	Araip.S501C	multiple-copy	Pentatricopeptiderepeat(PPR)superfamilyprotein
Aradu.14TMT	Araip.S8ZP2	multiple-copy	Pentatricopeptiderepeat(PPR)superfamilyprotein
Aradu.H1R88	Araip.SAI7F	multiple-copy	Pentatricopeptiderepeat(PPR)superfamilyprotein
Aradu.R17B3	Araip.SP8PR	multiple-copy	Pentatricopeptiderepeat(PPR)superfamilyprotein
Aradu.YQ9IH	Araip.SSR3W	multiple-copy	Pentatricopeptiderepeat(PPR)superfamilyprotein
Aradu.1U41E	Araip.T165J	multiple-copy	Pentatricopeptiderepeat(PPR)superfamilyprotein

Aradu.R1S51	Araip.T9BHU	multiple-copy	Pentatricopeptiderepeat(PPR)superfamilyprotein
Aradu.E0QTY	Araip.T9ZJW	multiple-copy	Pentatricopeptiderepeat(PPR)superfamilyprotein
Aradu.4L72B	Araip.TG6R6	multiple-copy	Pentatricopeptiderepeat(PPR)superfamilyprotein
Aradu.1573F	Araip.TJ4MC	multiple-copy	Pentatricopeptiderepeat(PPR)superfamilyprotein
Aradu.TQ51J	Araip.TP6H4	multiple-copy	Pentatricopeptiderepeat(PPR)superfamilyprotein
Aradu.N1NH0	Araip.TUV2T	multiple-copy	Pentatricopeptiderepeat(PPR)superfamilyprotein
Aradu.ARZ2Q	Araip.U647S	multiple-copy	Pentatricopeptiderepeat(PPR)superfamilyprotein
Aradu.81UZ4	Araip.UE4FG	multiple-copy	Pentatricopeptiderepeat(PPR)superfamilyprotein
Aradu.I7X6T	Araip.V4857	multiple-copy	Pentatricopeptiderepeat(PPR)superfamilyprotein
Aradu.L3IFZ	Araip.VFY46	multiple-copy	Pentatricopeptiderepeat(PPR)superfamilyprotein
Aradu.98PQS	Araip.W17SH	multiple-copy	Pentatricopeptiderepeat(PPR)superfamilyprotein
Aradu.VE0KU	Araip.W4DPP	multiple-copy	Pentatricopeptiderepeat(PPR)superfamilyprotein
Aradu.3ZF9R	Araip.WJ4AY	multiple-copy	Pentatricopeptiderepeat(PPR)superfamilyprotein
Aradu.QNG0R	Araip.WJL2T	multiple-copy	Pentatricopeptiderepeat(PPR)superfamilyprotein
Aradu.F54T9	Araip.X6QP4	multiple-copy	Pentatricopeptiderepeat(PPR)superfamilyprotein
Aradu.K2M4L	Araip.X7MJQ	multiple-copy	Pentatricopeptiderepeat(PPR)superfamilyprotein
Aradu.KH0NV	Araip.Y3MEG	multiple-copy	Pentatricopeptiderepeat(PPR)superfamilyprotein
Aradu.ZAA79	Araip.Y8BE0	multiple-copy	Pentatricopeptiderepeat(PPR)superfamilyprotein
Aradu.DIB1B	Araip.YS3X1	multiple-copy	Pentatricopeptiderepeat(PPR)superfamilyprotein
Aradu.9F1L9	Araip.Z3JAA	multiple-copy	Pentatricopeptiderepeat(PPR)superfamilyprotein
Aradu.70YAF	Araip.Z53A4	multiple-copy	Pentatricopeptiderepeat(PPR)superfamilyprotein
Aradu.RG76L	Araip.Z6IWN	multiple-copy	Pentatricopeptiderepeat(PPR)superfamilyprotein
Aradu.RJ6JS	Araip.ZQ3Q0	multiple-copy	Pentatricopeptiderepeat(PPR)superfamilyprotein
Aradu.VQA02	Araip.ZW17J	multiple-copy	Pentatricopeptiderepeat(PPR)superfamilyprotein
Aradu.9SY6W	Araip.ZW511	multiple-copy	Pentatricopeptiderepeat(PPR)superfamilyprotein
Aradu.5LR1R	Araip.ZY4UN	multiple-copy	Pentatricopeptiderepeat(PPR)superfamilyprotein
Aradu.7I0FT	Araip.19HBE	multiple-copy	Pentatricopeptiderepeat(PPR-like)superfamilyprotein
Aradu.16YW2	Araip.43MGU	multiple-copy	Pentatricopeptiderepeat(PPR-like)superfamilyprotein
Aradu.5M8KA	Araip.6X4HD	multiple-copy	Pentatricopeptiderepeat(PPR-like)superfamilyprotein
Aradu.K6X31	Araip.8ER40	multiple-copy	Pentatricopeptiderepeat(PPR-like)superfamilyprotein
Aradu.J1S90	Araip.94W62	multiple-copy	Pentatricopeptiderepeat(PPR-like)superfamilyprotein
Aradu.8Z92W	Araip.9VV1K	multiple-copy	Pentatricopeptiderepeat(PPR-like)superfamilyprotein
Aradu.7GG37	Araip.BPU1I	multiple-copy	Pentatricopeptiderepeat(PPR-like)superfamilyprotein
Aradu.W1XFV	Araip.E2793	multiple-copy	Pentatricopeptiderepeat(PPR)superfamilyprotein
Aradu.5L39Q	Araip.E7N05	multiple-copy	Pentatricopeptiderepeat(PPR-like)superfamilyprotein
Aradu.5C8VM	Araip.G99TB	multiple-copy	Pentatricopeptiderepeat(PPR-like)superfamilyprotein
Aradu.ELH7L	Araip.GE0SE	multiple-copy	Pentatricopeptiderepeat(PPR-like)superfamilyprotein
Aradu.Q00GR	Araip.H1NU3	multiple-copy	Pentatricopeptiderepeat(PPR-like)superfamilyprotein
Aradu.14QL4	Araip.IHC2V	multiple-copy	Pentatricopeptiderepeat(PPR-like)superfamilyprotein
Aradu.HIV9Z	Araip.KQ8VJ	multiple-copy	Pentatricopeptiderepeat(PPR-like)superfamilyprotein
Aradu.P9YG3	Araip.NG0MG	multiple-copy	Pentatricopeptiderepeat(PPR-like)superfamilyprotein
Aradu.IG2QJ	Araip.V90TX	multiple-copy	Pentatricopeptiderepeat(PPR-like)superfamilyprotein
Aradu.93BUT	Araip.ZCE2V	multiple-copy	Pentatricopeptiderepeat(PPR-like)superfamilyprotein
Aradu.W4I1P	Araip.036V7	multiple-copy	PENTATRICOPEPTIDEREPEAT596

Aradu.YP0M1	Araip.Y9RQ5	multiple-copy	pentatricopeptiderepeat-containingproteinAt4g04790,mito
Aradu.L4AW6	Araip.UR9L3	multiple-copy	PeptidaseM50familyprotein
Aradu.HU3I3	Araip.HW618	multiple-copy	peptide/nitratetransporter[Glycinemax]
Aradu.0T5YP	Araip.WS0IW	multiple-copy	Peptidechainreleasefactor1
Aradu.ZU0H3	Araip.0P39S	multiple-copy	Peptidechainreleasefactor2
Aradu.22RTM	Araip.T4YQW	multiple-copy	Peptidechainreleasefactor2
Aradu.6E81Q	Araip.28KXR	multiple-copy	peptidechainreleasefactorsubunit1-3
Aradu.RQQ24	Araip.PF76P	multiple-copy	Peptidemethioninesulfoxidereductasefamilyprotein
Aradu.VQB2Q	Araip.V7U9F	multiple-copy	PeptidemethioninesulfoxidereductaseMsrBn=3Tax=Alcali
Aradu.M5I54	Araip.4G5MQ	multiple-copy	Peptide-N4-(N-acetyl-beta-glucosaminyl)asparagineamid
Aradu.63K76	Araip.653FM	multiple-copy	peptidetransporter1
Aradu.3IZ11	Araip.G3RDD	multiple-copy	peptidetransporter1
Aradu.TBP46	Araip.JH23Y	multiple-copy	peptidetransporter1
Aradu.S3HBK	Araip.Q7WA8	multiple-copy	peptidetransporter1
Aradu.V1MDS	Araip.S2MVY	multiple-copy	nitratetransporter1.7
Aradu.HJ002	Araip.T7I1X	multiple-copy	peptidetransporter1
Aradu.A64FA	Araip.W3I5K	multiple-copy	peptidetransporter1
Aradu.42JCX	Araip.8FA2Y	multiple-copy	peptidetransporter2
Aradu.W903S	Araip.8Y8HZ	multiple-copy	peptidetransporter3
Aradu.JP0ZJ	Araip.HUI6H	multiple-copy	peptidetransporter3
Aradu.VKC3S	Araip.NH2DF	multiple-copy	peptidetransporter3
Aradu.HQI6K	Araip.W8L59	multiple-copy	peptidetransporter3
Aradu.Q1VWH	Araip.76V1I	multiple-copy	peptidetransporter5
Aradu.W1BNF	Araip.BTU8P	multiple-copy	peptidetransporter5
Aradu.0KX1J	Araip.SKH2V	multiple-copy	peptidetransporter5
Aradu.K3I53	Araip.XG2Y6	multiple-copy	peptidetransporter1
Aradu.HGJ4G	Araip.0F3YM	multiple-copy	peptidyl-prolylcis-transisomeraseE-likeprotein
Aradu.Q85V2	Araip.7HD76	multiple-copy	peptidyl-prolylcis-transisomerase-like3-like[Glycinemax]
Aradu.714NZ	Araip.B6DJV	multiple-copy	peptidyl-prolylcis-transisomerases
Aradu.GB23E	Araip.KGX8X	multiple-copy	peptidyl-prolylcis-transisomerases
Aradu.EX904	Araip.SAL9A	multiple-copy	peptidyl-prolylcis-transisomerases
Aradu.ZJ4KX	Araip.T7TLE	multiple-copy	peptidyl-prolylcis-transisomerases
Aradu.XEA0F	Araip.PZ75Q	multiple-copy	peroxidase2
Aradu.8IQ81	Araip.SPY8U	multiple-copy	peroxidase2
Aradu.H03KA	Araip.15H8S	multiple-copy	Peroxidase48-like[Glycinemax]
Aradu.VF4U3	Araip.433VX	multiple-copy	Peroxidasefamilyprotein
Aradu.IG70C	Araip.09WK1	multiple-copy	Peroxidasesuperfamilyprotein
Aradu.61ES3	Araip.0QG56	multiple-copy	Peroxidasesuperfamilyprotein
Aradu.BG2Q4	Araip.1P50V	multiple-copy	Peroxidasesuperfamilyprotein
Aradu.6PX5N	Araip.1U8VF	multiple-copy	Peroxidasesuperfamilyprotein
Aradu.1D15U	Araip.5V6AL	multiple-copy	Peroxidasesuperfamilyprotein
Aradu.8I3T0	Araip.72YG4	multiple-copy	Peroxidasesuperfamilyprotein
Aradu.VS9VK	Araip.7P78Q	multiple-copy	Peroxidasesuperfamilyprotein
Aradu.V7F48	Araip.80WHH	multiple-copy	Peroxidasesuperfamilyprotein

Aradu.H7KNI	Araip.85TVG	multiple-copy	Peroxidasesuperfamilyprotein
Aradu.X4HEP	Araip.B9NBT	multiple-copy	Peroxidasesuperfamilyprotein
Aradu.074JV	Araip.BJ3QY	multiple-copy	Peroxidasesuperfamilyprotein
Aradu.2BI47	Araip.BKI6W	multiple-copy	Peroxidasesuperfamilyprotein
Aradu.QHA6Y	Araip.DER8T	multiple-copy	Peroxidasesuperfamilyprotein
Aradu.UJ1ZX	Araip.G30W8	multiple-copy	Peroxidasesuperfamilyprotein
Aradu.SH1J0	Araip.GIZ0F	multiple-copy	Peroxidasesuperfamilyprotein
Aradu.7ZK0R	Araip.H1RCV	multiple-copy	Peroxidasesuperfamilyprotein
Aradu.I66GQ	Araip.I0093	multiple-copy	Peroxidasesuperfamilyprotein
Aradu.6F2AZ	Araip.JC79F	multiple-copy	Peroxidasesuperfamilyprotein
Aradu.SQK9K	Araip.LXR9M	multiple-copy	Peroxidasesuperfamilyprotein
Aradu.Z08PN	Araip.LY9VA	multiple-copy	peroxidase5-like[Glycinemax]
Aradu.UD5NS	Araip.MM1BS	multiple-copy	Peroxidasesuperfamilyprotein
Aradu.YY51Q	Araip.N79SI	multiple-copy	Peroxidasesuperfamilyprotein
Aradu.N2X0I	Araip.N95WX	multiple-copy	Peroxidasesuperfamilyprotein
Aradu.00WGF	Araip.NLR8N	multiple-copy	Peroxidasesuperfamilyprotein
Aradu.947SP	Araip.PP1VA	multiple-copy	Peroxidasesuperfamilyprotein
Aradu.Y7A6L	Araip.SR41M	multiple-copy	Peroxidasesuperfamilyprotein
Aradu.2Z4NE	Araip.SR6HQ	multiple-copy	Peroxidasesuperfamilyprotein
Aradu.1LQ1Q	Araip.SWD4L	multiple-copy	Peroxidasesuperfamilyprotein
Aradu.CCC04	Araip.T2765	multiple-copy	Peroxidasesuperfamilyprotein
Aradu.U8NZD	Araip.U5BHC	multiple-copy	Peroxidasesuperfamilyprotein
Aradu.D4KTP	Araip.WQL72	multiple-copy	Peroxidasesuperfamilyprotein
Aradu.DDQ1E	Araip.X3VWY	multiple-copy	Peroxidasesuperfamilyprotein
Aradu.DAV01	Araip.Z1F3X	multiple-copy	Peroxidasesuperfamilyprotein
Aradu.U3RTW	Araip.ZEN1Y	multiple-copy	Peroxidasesuperfamilyprotein
Aradu.9PD7U	Araip.ZET1U	multiple-copy	Peroxidasesuperfamilyprotein
Aradu.EMN7F	Araip.SCS0N	multiple-copy	peroxisomaladeninenucleotidecarrier1
Aradu.B1ZZT	Araip.ZW85N	multiple-copy	peroxisomalbiogenesisfactor11familyprotein
Aradu.RQ7SQ	Araip.EDA7H	multiple-copy	Peroxisomalmembrane22kDa(Mpv17/PMP22)familyprote
Aradu.YZ4F8	Araip.L4AL0	multiple-copy	Peroxisomalmembrane22kDa(Mpv17/PMP22)familyprote
Aradu.B73B3	Araip.M16D8	multiple-copy	Peroxisomalmembrane22kDa(Mpv17/PMP22)familyprote
Aradu.MJ7TV	Araip.Q43KM	multiple-copy	Peroxisomalmembrane22kDa(Mpv17/PMP22)familyprote
Aradu.NW9B9	Araip.QT4UB	multiple-copy	Peroxisomalmembrane22kDa(Mpv17/PMP22)familyprote
Aradu.VV3RK	Araip.ZJ5LN	multiple-copy	Peroxisomalmembrane22kDa(Mpv17/PMP22)familyprote
Aradu.E9QIB	Araip.T9ZZ1	multiple-copy	PeroxisomalmembraneproteinPEX16n=2Tax=XenopusR
Aradu.2KV4N	Araip.G7W3J	multiple-copy	pfkB-likecarbohydratekinasefamilyprotein
Aradu.9RZ6U	Araip.MM388	multiple-copy	pfkB-likecarbohydratekinasefamilyprotein
Aradu.9HV89	Araip.X9ZFP	multiple-copy	pfkB-likecarbohydratekinasefamilyprotein
Aradu.SFF9P	Araip.YCH8N	multiple-copy	pfkB-likecarbohydratekinasefamilyprotein
Aradu.NH34T	Araip.ZD3SC	multiple-copy	pfkB-likecarbohydratekinasefamilyprotein
Aradu.J486B	Araip.LXT0U	multiple-copy	PHDfingerfamilyprotein
Aradu.P3PDP	Araip.K86BU	multiple-copy	PHDfingerfamilyprotein/bromo-adjacenthomology(BAH)d
Aradu.8VT0P	Araip.JZV3C	multiple-copy	PHD-fingerprotein

Aradu.V1NXI	Araip.3B3VN	multiple-copy	PHDfingerproteinALFIN-LIKE2-like[Glycinemax]
Aradu.1LI5S	Araip.I5MUZ	multiple-copy	PHDfingerproteinALFIN-LIKE2-like[Glycinemax]
Aradu.I3T1H	Araip.60LL8	multiple-copy	PHDfingerproteinALFIN-LIKE4-like[Glycinemax]
Aradu.4EB0K	Araip.5FM9T	multiple-copy	PHDfingerprotein-related
Aradu.U5NU2	Araip.JIL9K	multiple-copy	phenylalanineammonia-lyase2
Aradu.G7F8E	Araip.V9S7Z	multiple-copy	phenylalanineammonia-lyase2
Aradu.G1DZV	Araip.X6JCD	multiple-copy	phloemprotein2-B2
Aradu.0P8G3	Araip.AG3WT	multiple-copy	phloemprotein2-B5
Aradu.JP5P4	Araip.TG0L1	multiple-copy	phosphatase2AregulatoryBsubunitfamily
Aradu.NF928	Araip.WJD5Q	multiple-copy	phosphatase2AregulatoryBsubunitfamily
Aradu.H1X77	Araip.ZJ927	multiple-copy	phosphatase2AregulatoryBsubunitfamily
Aradu.V68II	Araip.1D5WV	multiple-copy	phosphatase2Cfamily
Aradu.88Z6K	Araip.2YE37	multiple-copy	phosphatase2Cfamily
Aradu.TYG98	Araip.45GIV	multiple-copy	phosphatase2Cfamily
Aradu.YH8NY	Araip.6KE2F	multiple-copy	phosphatase2Cfamily
Aradu.DB4LT	Araip.7IA97	multiple-copy	phosphatase2Cfamily
Aradu.0MS0A	Araip.7Y7KI	multiple-copy	phosphatase2Cfamily
Aradu.Q9LEZ	Araip.8VK7X	multiple-copy	phosphatase2Cfamily
Aradu.ME340	Araip.8YV7L	multiple-copy	phosphatase2Cfamily
Aradu.MLY6C	Araip.9J4X2	multiple-copy	phosphatase2Cfamily
Aradu.NA3JC	Araip.9K6YA	multiple-copy	phosphatase2Cfamily
Aradu.RC9UC	Araip.B0B1I	multiple-copy	phosphatase2Cfamily
Aradu.P2C14	Araip.CC2EP	multiple-copy	phosphatase2Cfamily
Aradu.SRM8N	Araip.D6MDF	multiple-copy	phosphatase2Cfamily
Aradu.A9FZ7	Araip.GC0FX	multiple-copy	phosphatase2Cfamily
Aradu.M7QN4	Araip.HQ6XR	multiple-copy	phosphatase2Cfamily
Aradu.5UI2Y	Araip.I6YVE	multiple-copy	phosphatase2Cfamily
Aradu.8005S	Araip.IHG6N	multiple-copy	phosphatase2Cfamily
Aradu.CT5EJ	Araip.K90VM	multiple-copy	phosphatasetype2C/serine/threoninephosphatasen=6Ta
Aradu.0EZ1S	Araip.KBB88	multiple-copy	phosphatase2Cfamily
Aradu.IT5KP	Araip.KBS8N	multiple-copy	phosphatase2Cfamily
Aradu.C7AZ0	Araip.L46F0	multiple-copy	phosphatase2Cfamily
Aradu.ZX8II	Araip.LUP74	multiple-copy	phosphatase2Cfamily
Aradu.S38FI	Araip.NJ5S3	multiple-copy	phosphatase2Cfamily
Aradu.PW3YK	Araip.SX5U5	multiple-copy	phosphatase2Cfamily
Aradu.Y64WA	Araip.TQP7Q	multiple-copy	phosphatase2Cfamily
Aradu.8S4TL	Araip.U4R4L	multiple-copy	phosphatase2Cfamily
Aradu.VZ4PC	Araip.UDU7A	multiple-copy	phosphatase2Cfamily
Aradu.BLA9Y	Araip.VBT3F	multiple-copy	phosphatase2Cfamily
Aradu.57C0B	Araip.Z0QLZ	multiple-copy	phosphatase2Cfamily
Aradu.289R9	Araip.ZJ40R	multiple-copy	phosphatase2Cfamily
Aradu.1Y87X	Araip.WMX3R	multiple-copy	phosphatetransporter1
Aradu.UA2HU	Araip.WR1Z1	multiple-copy	phosphatetransporter1
Aradu.A9U89	Araip.76SLC	multiple-copy	phosphatetransporter2

Aradu.40078	Araip.03WH7	multiple-copy	phosphatetransporter4
Aradu.VG3CJ	Araip.PZ3FG	multiple-copy	phosphatetransporter4
Aradu.81HAH	Araip.1KE3S	multiple-copy	phosphatetransporterPHO1homolog1-likeisoform1[Glycin
Aradu.7E3FL	Araip.W9K1P	multiple-copy	phosphatidatecytidylyltransferasefamilyprotein
Aradu.NKJ9K	Araip.WB0T6	multiple-copy	phosphatidylinositol3,4,5-trisphosphate3-phosphataseand
Aradu.CDX53	Araip.KU4D3	multiple-copy	Phosphatidylinositol3-and4-kinasefamilyprotein
Aradu.TWK59	Araip.YXG1J	multiple-copy	Phosphatidylinositol3-and4-kinasefamilyprotein
Aradu.WH2W0	Araip.FU10G	multiple-copy	phosphatidylinositol-4-phosphate5-kinase1
Aradu.LW7LA	Araip.HXN3H	multiple-copy	phosphatidylinositol-4-phosphate5-kinase1
Aradu.M96PR	Araip.TTR3L	multiple-copy	phosphatidylinositol-4-phosphate5-kinase1
Aradu.XQ1BV	Araip.1F19V	multiple-copy	phosphatidylinositol-4-phosphate5-kinase5
Aradu.QZ0ZC	Araip.6R0Q8	multiple-copy	Phosphatidylinositol-4-phosphate5-kinasefamilyprotein
Aradu.LKB1I	Araip.HD1YD	multiple-copy	phosphatidylinositol-4-phosphate5-kinasefamilyprotein
Aradu.9P6VM	Araip.SC1T4	multiple-copy	phosphatidylinositoltransferfamilyprotein
Aradu.TPI8X	Araip.BSJ6S	multiple-copy	phosphoenolpyruvatecarboxylase1
Aradu.DR5UL	Araip.60F3J	multiple-copy	phosphofructokinase3
Aradu.XK367	Araip.0D2JJ	multiple-copy	phosphofructokinase4
Aradu.M8RTT	Araip.AQV0I	multiple-copy	phosphofructokinase5
Aradu.SS76M	Araip.WK203	multiple-copy	phosphofructokinase5
Aradu.CZQ8X	Araip.71W8G	multiple-copy	Phosphoglucomutase/phosphomannomutase,alpha/beta/
Aradu.5U8HG	Araip.E514L	multiple-copy	Phosphoglucomutase/phosphomannomutasefamilyprotei
Aradu.QZ6DW	Araip.ZR8BC	multiple-copy	phosphoglycerate/bisphosphoglyceratemutase
Aradu.Y5B7U	Araip.18LXX	multiple-copy	Phosphoglyceratemutasefamilyprotein
Aradu.X7855	Araip.6Y4LC	multiple-copy	Phosphoglyceratemutasefamilyprotein
Aradu.DM3FR	Araip.RT1FB	multiple-copy	Phosphoglyceratemutasefamilyprotein
Aradu.22AJD	Araip.SI1NJ	multiple-copy	Phosphoglyceratemutasefamilyprotein
Aradu.V1TZX	Araip.TH0I1	multiple-copy	Phosphoglyceratemutasefamilyprotein
Aradu.3V07T	Araip.WSF31	multiple-copy	Phosphoglyceratemutasefamilyprotein
Aradu.36NL9	Araip.645JG	multiple-copy	phosphoinositidephospholipaseC2-like[Glycinemax]
Aradu.HP9IR	Araip.ZW55J	multiple-copy	phosphoinositidephospholipaseC6-like[Glycinemax]
Aradu.580FR	Araip.JP448	multiple-copy	phospholipaseA2
Aradu.64JV5	Araip.EE8FM	multiple-copy	PhospholipaseA2familyprotein
Aradu.J4XWG	Araip.HJ37G	multiple-copy	phospholipaseC2
Aradu.NR2Z3	Araip.3PC35	multiple-copy	phospholipaseDP1
Aradu.76V5M	Araip.ZU18G	multiple-copy	phospholipaseDP1
Aradu.7N75D	Araip.10YD3	multiple-copy	phospholipaseDP2
Aradu.X0Z7E	Araip.67DP4	multiple-copy	phospholipaseDP2
Aradu.NT5AR	Araip.CEE4L	multiple-copy	phospholipaseDP2
Aradu.FS7LG	Araip.MYU90	multiple-copy	phospholipaseDP2
Aradu.J7XF6	Araip.N118V	multiple-copy	phospholipaseDP2
Aradu.KSI0H	Araip.QK1BG	multiple-copy	phospholipaseDP2
Aradu.W0B0I	Araip.37594	multiple-copy	phospholipaseDY
Aradu.UA9D8	Araip.WVH6X	multiple-copy	phospholipid:diacylglycerolacyltransferase
Aradu.7Q4DG	Araip.TT5YQ	multiple-copy	phospholipid-transportingATPase1-likeisoformX3[Glycine

Aradu.G0BMM	Araip.V7N27	multiple-copy	phospholipid-transportingATPase8-likeisoformX1[Glycine
Aradu.4Z2QP	Araip.80E2A	multiple-copy	phospholipid-transportingATPase9-likeisoformX1[Glycine
Aradu.4M66F	Araip.EDG3L	multiple-copy	phospholipid-transportingATPase-likeprotein
Aradu.073NL	Araip.GS20D	multiple-copy	phospholipid-transportingATPase9-likeisoformX1[Glycine
Aradu.V9VEN	Araip.N4RJ5	multiple-copy	phospholipid-transportingATPase9-likeisoformX1[Glycine
Aradu.KNB1Y	Araip.D9UGE	multiple-copy	phosphopantothenoylcysteinedecarboxylase-like[Glycine
Aradu.K0HFM	Araip.DI07C	multiple-copy	phosphopantothenoylcysteinedecarboxylase-like[Glycine
Aradu.JVB3U	Araip.JX0VW	multiple-copy	phosphopyruvatehydratase
Aradu.07WQA	Araip.PQA2W	multiple-copy	Phosphopyruvatehydratasen=1Tax=Dictyosteliumfascicu
Aradu.L5CRG	Araip.YCD9D	multiple-copy	photosystemII22kDaprotein,chloroplastic-like[Glycinemax
Aradu.L9QRL	Araip.374K5	multiple-copy	PhotosystemIIoxygen-evolvingcomplex23Kproteinn=15Ta
Aradu.DG7AI	Araip.2EE1I	multiple-copy	PhotosystemIIoxygenevolvingcomplexproteinPsbP,23kD
Aradu.YTQ46	Araip.727WD	multiple-copy	PhotosystemIIoxygenevolvingcomplexproteinPsbP,23kD
Aradu.WQI06	Araip.P0TWG	multiple-copy	PhotosystemIIoxygenevolvingcomplexproteinPsbP,23kD
Aradu.63RCW	Araip.TA9E2	multiple-copy	PhotosystemIIoxygenevolvingcomplexproteinPsbP,23kD
Aradu.FLZ7V	Araip.U3HJ0	multiple-copy	PhotosystemIIoxygenevolvingcomplexproteinPsbPn=1Ta
Aradu.UZX8A	Araip.NPF88	multiple-copy	photosystemIIreactioncenterPSB28protein
Aradu.S3MQ8	Araip.5VP72	multiple-copy	PhotosystemIIreactioncenterPsbPfamilyprotein
Aradu.CI3JS	Araip.RSA74	multiple-copy	photosystemIreactioncentersubunitIII
Aradu.IPP1D	Araip.PQA29	multiple-copy	photosystemIreactioncentersubunitIVA
Aradu.B4GBB	Araip.S2EYP	multiple-copy	photosystemIreactioncentersubunitIVA
Aradu.09HBR	Araip.MX0X9	multiple-copy	photosystemIreactioncentersubunitVI
Aradu.VTB62	Araip.Y561F	multiple-copy	photosystemIreactioncentersubunitXI
Aradu.V3JVM	Araip.WLL04	multiple-copy	phox(PX)domainprotein
Aradu.9K9AQ	Araip.44M02	multiple-copy	Phox-associateddomain
Aradu.U57WW	Araip.40X13	multiple-copy	PHYTOENESYNTHASE
Aradu.YAX06	Araip.N5MMK	multiple-copy	PIF3-like[Glycinemax]
Aradu.I92X3	Araip.L4GEP	multiple-copy	PIF4-like[Glycinemax]
Aradu.VC25Y	Araip.D61AA	multiple-copy	PI-PLCXdomain-containingproteinAt5g67130-like[Glycine
Aradu.Y82ZL	Araip.TMG8Z	multiple-copy	PI-PLCXdomain-containingproteinAt5g67130-like[Glycine
Aradu.03GCY	Araip.YR17W	multiple-copy	PLAC8familyprotein
Aradu.H42AE	Araip.54D0C	multiple-copy	plant/MEB5-likeprotein
Aradu.Q1CYX	Araip.CS4YJ	multiple-copy	PLANTCADMIUMRESISTANCE2-like[Glycinemax]
Aradu.KA3UP	Araip.S3ICP	multiple-copy	PLANTCADMIUMRESISTANCE2-like[Glycinemax]
Aradu.PDS8K	Araip.YB0D8	multiple-copy	PLANTCADMIUMRESISTANCE2-like[Glycinemax]
Aradu.E5U8P	Araip.1E680	multiple-copy	Plantinvertase/pectinmethylesteraseinhibitorsuperfamilyp
Aradu.W8J78	Araip.X04AD	multiple-copy	Plantinvertase/pectinmethylesteraseinhibitorsuperfamilyp
Aradu.YRC2R	Araip.R44NW	multiple-copy	PlantregulatorRWP-RKfamilyprotein
Aradu.H6JXR	Araip.RLH2R	multiple-copy	PlantregulatorRWP-RKfamilyprotein
Aradu.T4VLF	Araip.Y4AFN	multiple-copy	PlantregulatorRWP-RKfamilyprotein
Aradu.LR6SL	Araip.PJ4IX	multiple-copy	plant-specificB3-DNA-bindingdomainprotein
Aradu.YYA59	Araip.RW92I	multiple-copy	plant-specificB3-DNA-bindingdomainprotein
Aradu.S6366	Araip.SI63P	multiple-copy	Plasma-membranecholinetransporterfamilyprotein
Aradu.0U1AX	Araip.M5PAK	multiple-copy	plasmamembraneH+-ATPase

Aradu.6AI81	Araip.Y94H3	multiple-copy	plasmamembraneintrinsicprotein2
Aradu.PJ0EM	Araip.Y0BV6	multiple-copy	Plasmamembranemannitoltransportern=1Tax=Arachishy
Aradu.T8C1S	Araip.Y6U3P	multiple-copy	Plasmamembranemannitoltransportern=1Tax=Arachishy
Aradu.H88U3	Araip.A7MMQ	multiple-copy	plastiddevelopmentalproteinDAG,putative
Aradu.E2YZD	Araip.N1KVL	multiple-copy	plastiddevelopmentalproteinDAG,putative
Aradu.XYY0T	Araip.TUV11	multiple-copy	plastiddivisionproteinPDV2,putative
Aradu.Z0JID	Araip.9HJ5A	multiple-copy	plastid-lipid-associatedprotein14,chloroplastic-likeisoform
Aradu.X3U5Y	Araip.84K6K	multiple-copy	Plastid-lipidassociatedproteinPAP/fibrillinfamilyprotein
Aradu.2R9BM	Araip.G1IA2	multiple-copy	Plastid-lipidassociatedproteinPAP/fibrillinfamilyprotein
Aradu.18DQZ	Araip.K8LIV	multiple-copy	Plastid-lipidassociatedproteinPAP/fibrillinfamilyprotein
Aradu.M2NRW	Araip.T2627	multiple-copy	Plastid-lipidassociatedproteinPAP/fibrillinfamilyprotein
Aradu.G44X8	Araip.UVI0L	multiple-copy	Plastid-lipidassociatedproteinPAP/fibrillinfamilyprotein
Aradu.5LG80	Araip.XR6PW	multiple-copy	Plastid-lipidassociatedproteinPAP/fibrillinfamilyprotein
Aradu.SK8K0	Araip.HE7HA	multiple-copy	plastocyanin-likedomainprotein
Aradu.YMC7K	Araip.4P39C	multiple-copy	PLATZtranscriptionfactorfamilyprotein
Aradu.7J9TI	Araip.F2177	multiple-copy	PLATZtranscriptionfactorfamilyprotein
Aradu.07NMY	Araip.G2AKQ	multiple-copy	PLATZtranscriptionfactorfamilyprotein
Aradu.46VDC	Araip.JG1MS	multiple-copy	PLATZtranscriptionfactorfamilyprotein
Aradu.1D83F	Araip.YY5JE	multiple-copy	Pleckstrinhomology(PH)domain-containingprotein/lipid-bi
Aradu.A7GFW	Araip.MSG33	multiple-copy	Pleckstrinhomology(PH)domainsuperfamilyprotein
Aradu.9TU3R	Araip.Z762D	multiple-copy	pleckstrin-like(PH)andlipid-bindingSTARTdomainprotein
Aradu.THV7P	Araip.66P9D	multiple-copy	P-loopcontainingnucleosidetriphosphatehydrolasessuper
Aradu.VQ1MT	Araip.BBT8F	multiple-copy	P-loopcontainingnucleosidetriphosphatehydrolasessuper
Aradu.1L0P8	Araip.C84JH	multiple-copy	P-loopcontainingnucleosidetriphosphatehydrolasessuper
Aradu.1IM7V	Araip.G9RMH	multiple-copy	P-loopcontainingnucleosidetriphosphatehydrolasessuper
Aradu.MZ5UW	Araip.HUQ8Q	multiple-copy	P-loopcontainingnucleosidetriphosphatehydrolasessuper
Aradu.J5AA1	Araip.I0EMG	multiple-copy	P-loopcontainingnucleosidetriphosphatehydrolasessuper
Aradu.FL84V	Araip.WVU2E	multiple-copy	P-loopcontainingnucleosidetriphosphatehydrolasessuper
Aradu.MI27L	Araip.39P36	multiple-copy	PollenOlee1allergenandextensinfamilyprotein
Aradu.GXJ7L	Araip.4RI8H	multiple-copy	PollenOlee1allergenandextensinfamilyprotein
Aradu.B33TG	Araip.8S5BI	multiple-copy	PollenOlee1allergenandextensinfamilyprotein
Aradu.13QYM	Araip.9ZM5J	multiple-copy	PollenOlee1allergenandextensinfamilyprotein
Aradu.3MD78	Araip.BP4Q3	multiple-copy	PollenOlee1allergenandextensinfamilyprotein
Aradu.RM26Y	Araip.D0TT7	multiple-copy	PollenOlee1allergenandextensinfamilyprotein
Aradu.EYQ2A	Araip.DN8BF	multiple-copy	PollenOlee1allergenandextensinfamilyprotein
Aradu.826D1	Araip.PT656	multiple-copy	PollenOlee1allergenandextensinfamilyprotein
Aradu.91ENH	Araip.W6PWH	multiple-copy	PollenOlee1allergenandextensinfamilyprotein
Aradu.QU8L1	Araip.WPV5M	multiple-copy	PollenOlee1allergenandextensinfamilyprotein
Aradu.AX4BJ	Araip.Y1B33	multiple-copy	PollenOlee1allergenandextensinfamilyprotein
Aradu.3GX6J	Araip.ZB5LG	multiple-copy	pollenproteinOleEI-likeprotein
Aradu.DG903	Araip.F9QDS	multiple-copy	poly(A)RNApolymerasecid11-likeisoformX2[Glycinemax]
Aradu.7GC8S	Araip.X3I39	multiple-copy	poly(A)RNApolymerasecid11-likeisoformX4[Glycinemax]
Aradu.C08W6	Araip.T4ATQ	multiple-copy	polyadenylate-bindingprotein7-likeisoformX2[Glycinemax
Aradu.6Z2TN	Araip.4U3W0	multiple-copy	polygalacturonase[Glycinemax]

Aradu.TVX63	Araip.GD8VH	multiple-copy	polygalacturonase[Glycinemax]
Aradu.LZC3L	Araip.1E43N	multiple-copy	polygalacturonase4
Aradu.6V1PP	Araip.6G6P9	multiple-copy	polygalacturonase4
Aradu.1GI8T	Araip.9U1PK	multiple-copy	polygalacturonase4
Aradu.S69T7	Araip.J0KBV	multiple-copy	polygalacturonase4
Aradu.65YXV	Araip.L9LN0	multiple-copy	polygalacturonase4
Aradu.ZR5CT	Araip.RZ98L	multiple-copy	polygalacturonase4
Aradu.X6DIS	Araip.T29CT	multiple-copy	polygalacturonase4
Aradu.JA3L7	Araip.X2NAH	multiple-copy	polygalacturonase4
Aradu.P0G7F	Araip.XFN00	multiple-copy	polygalacturonase4
Aradu.SH1QV	Araip.BL3I0	multiple-copy	polygalacturonase-like[Glycinemax]
Aradu.Z4D1S	Araip.NG6P6	multiple-copy	polygalacturonase[Glycinemax]
Aradu.P7M2S	Araip.GV2B3	multiple-copy	polygalacturonase-like[Glycinemax]
Aradu.RV6BG	Araip.IWB76	multiple-copy	polygalacturonase-like[Glycinemax]
Aradu.D4FDN	Araip.M4PVE	multiple-copy	polygalacturonase-like[Glycinemax]
Aradu.SCG5D	Araip.NXU71	multiple-copy	polygalacturonase-like[Glycinemax]
Aradu.70WHV	Araip.S9DS1	multiple-copy	polygalacturonase-like[Glycinemax]
Aradu.J1JQ8	Araip.AB8FX	multiple-copy	polygalacturonasenon-catalyticprotein
Aradu.TFK2R	Araip.H3C8N	multiple-copy	polygalacturonasenon-catalyticprotein
Aradu.B4FEF	Araip.CW7EC	multiple-copy	Polyketidecyclase/dehydraseandlipidtransportsuperfamily
Aradu.KR1YF	Araip.K5Y7B	multiple-copy	Polyketidecyclase/dehydraseandlipidtransportsuperfamily
Aradu.TVQ04	Araip.LN1Q8	multiple-copy	Polyketidecyclase/dehydraseandlipidtransportsuperfamily
Aradu.FWS4A	Araip.X2EME	multiple-copy	Polyketidecyclase/dehydraseandlipidtransportsuperfamily
Aradu.70NBC	Araip.AB1C2	multiple-copy	Polynucleotidyltransferase,ribonucleaseH-likesuperfamily
Aradu.S591T	Araip.D29UN	multiple-copy	Polyproteinn=1Tax=CitrusendogenouspararetrovirusRepl
Aradu.N1Q89	Araip.LFM83	multiple-copy	Polyproteinn=5Tax=CitrusendogenouspararetrovirusRepl
Aradu.8KB8X	Araip.N68W5	multiple-copy	Polyproteinn=5Tax=CitrusendogenouspararetrovirusRepl
Aradu.NQC4B	Araip.0G5JH	multiple-copy	Potassiumtransporterfamilyprotein
Aradu.8T3RP	Araip.J7NVV	multiple-copy	Potassiumtransporterfamilyprotein
Aradu.EG96G	Araip.MSE9J	multiple-copy	Potassiumtransporterfamilyprotein
Aradu.ZY590	Araip.NP2NQ	multiple-copy	Potassiumtransporterfamilyprotein
Aradu.0K49H	Araip.RZ87P	multiple-copy	Potassiumtransporterfamilyprotein
Aradu.PC4II	Araip.X9FTP	multiple-copy	Potassiumtransporterfamilyprotein
Aradu.69W3K	Araip.A3G0I	multiple-copy	PPPDEputativethiolpeptidasefamilyprotein
Aradu.N42W4	Araip.D2S2M	multiple-copy	PPPDEputativethiolpeptidasefamilyprotein
Aradu.W75H1	Araip.E1WBJ	multiple-copy	PPPDEputativethiolpeptidasefamilyprotein
Aradu.NZ4WV	Araip.LKP3W	multiple-copy	PPPDEputativethiolpeptidasefamilyprotein
Aradu.464FL	Araip.R8FCG	multiple-copy	PPPDEputativethiolpeptidasefamilyprotein
Aradu.71T87	Araip.YU9WM	multiple-copy	PPPDEputativethiolpeptidasefamilyprotein
Aradu.245YW	Araip.NN0H7	multiple-copy	PQ-looprepeatfamilyprotein/transmembranefamilyprotein
Aradu.W4S0X	Araip.X1U9E	multiple-copy	PREFOLDIN1
Aradu.1CN3T	Araip.9W9E2	multiple-copy	pre-mRNA-splicingfactor18-like[Glycinemax]
Aradu.Z9KCW	Araip.UF61R	multiple-copy	pre-mRNAsplicingfactor-related
Aradu.I2L2L	Araip.J8JYF	multiple-copy	pre-mRNA-splicingfactorSF2-likeisoformX2[Glycinemax]

Aradu.GV115	Araip.XVS2K	multiple-copy	pre-mRNA-splicingfactorSF2-likeisoformX4[Glycinemax]
Aradu.PG3K0	Araip.551RZ	multiple-copy	Pre-mRNA-splicingfactorSLU7-likeprotein
Aradu.GKD84	Araip.CTQ0H	multiple-copy	Pre-mRNA-splicingfactorSLU7-likeprotein
Aradu.7P38Z	Araip.EF2V0	multiple-copy	pre-mRNA-splicingfactorSPF27homolog[Glycinemax]
Aradu.P7PS3	Araip.P6A5Q	multiple-copy	prephenatedehydrogenasefamilyprotein
Aradu.3E55M	Araip.C6M4G	multiple-copy	pre-rRNA-processingproteinTSR2
Aradu.D2IBT	Araip.2T40G	multiple-copy	pre-rRNA-processingTSR1-likeprotein
Aradu.32TSS	Araip.QU4D9	multiple-copy	profilin4
Aradu.YK06D	Araip.W2DXP	multiple-copy	prolinedehydrogenase
Aradu.XD7VB	Araip.7RK50	multiple-copy	proline-richprotein4-like[Glycinemax]
Aradu.1B3IN	Araip.W1EIB	multiple-copy	proline-richprotein4-like[Glycinemax]
Aradu.MW8ED	Araip.T0KA2	multiple-copy	proline-richproteinPRCC-like[Glycinemax]
Aradu.UKP00	Araip.JE0JZ	multiple-copy	prolyl4-hydroxylasesubunitalpha-1-like[Glycinemax]
Aradu.B151U	Araip.L60S0	multiple-copy	proteasomealphasubunitF1
Aradu.MQ2DW	Araip.2178J	multiple-copy	proteasomesubunitalphatype-7-Aprotein
Aradu.3TE64	Araip.J0VA9	multiple-copy	proteasomesubunitalphatype-7-Aprotein
Aradu.LGS6Z	Araip.1T9DH	multiple-copy	proteasomesubunitbetatype-7-Aprotein
Aradu.HBT8X	Araip.H4G2Q	multiple-copy	Proteasomesubunitbetatypen=5Tax=PopulusRepID=B9H
Aradu.L9R8I	Araip.X09HZ	multiple-copy	proteasomesubunitbetatypeprotein,putative
Aradu.HNU76	Araip.Y5X21	multiple-copy	Proteinbinding/ubiquitin-proteinligase/zincionbindingn=1T
Aradu.1V1PL	Araip.EZI68	multiple-copy	Proteinkinasesuperfamilyprotein
Aradu.VK1JK	Araip.02HSD	multiple-copy	proteinkinase2A
Aradu.M9FCY	Araip.50DA0	multiple-copy	proteinkinase2B
Aradu.008V1	Araip.BJM1C	multiple-copy	Proteinkinasesuperfamilyprotein
Aradu.F1MD3	Araip.136S0	multiple-copy	Proteinkinasefamilyprotein
Aradu.J09BS	Araip.1C776	multiple-copy	proteinkinasefamilyprotein
Aradu.TG162	Araip.3V9HH	multiple-copy	proteinkinasefamilyprotein
Aradu.IJ1L1	Araip.72BD2	multiple-copy	proteinkinasefamilyprotein
Aradu.73JAV	Araip.7IS5A	multiple-copy	proteinkinasefamilyprotein
Aradu.1QV2U	Araip.7Z8D2	multiple-copy	proteinkinasefamilyprotein
Aradu.CM89H	Araip.A805A	multiple-copy	proteinkinasefamilyprotein
Aradu.JZ4PA	Araip.BR3MH	multiple-copy	proteinkinasefamilyprotein
Aradu.41EZW	Araip.DM985	multiple-copy	proteinkinasefamilyprotein
Aradu.L8GIZ	Araip.F3KB9	multiple-copy	Proteinkinasefamilyprotein
Aradu.J2BV7	Araip.F77E0	multiple-copy	proteinkinasefamilyprotein
Aradu.D0AKP	Araip.F82T0	multiple-copy	proteinkinasefamilyprotein
Aradu.JG7ZE	Araip.GN34D	multiple-copy	Proteinkinasefamilyprotein
Aradu.35B90	Araip.I46QR	multiple-copy	proteinkinasefamilyprotein
Aradu.SX4F9	Araip.I6C5W	multiple-copy	proteinkinasefamilyprotein
Aradu.6M72C	Araip.JR8N9	multiple-copy	proteinkinasefamilyprotein
Aradu.Z7XZ9	Araip.KS6V8	multiple-copy	proteinkinasefamilyprotein
Aradu.ZW165	Araip.P2X4L	multiple-copy	proteinkinasefamilyprotein
Aradu.6YC2Q	Araip.QXM6Z	multiple-copy	proteinkinasefamilyprotein
Aradu.23D9S	Araip.RB9NR	multiple-copy	proteinkinasefamilyprotein

Aradu.RWI1K	Araip.RK7QB	multiple-copy	Proteinkinasefamilyprotein
Aradu.Y31BQ	Araip.T1GN1	multiple-copy	proteinkinasefamilyprotein
Aradu.A8AWS	Araip.TA2YD	multiple-copy	proteinkinasefamilyprotein
Aradu.UIF60	Araip.UH3IE	multiple-copy	proteinkinasefamilyprotein
Aradu.5BW4X	Araip.V9BE8	multiple-copy	proteinkinasefamilyprotein
Aradu.IM707	Araip.V9W0G	multiple-copy	Proteinkinasefamilyprotein
Aradu.Q5XDE	Araip.W1LHP	multiple-copy	proteinkinasefamilyprotein
Aradu.VKS8C	Araip.W9PNA	multiple-copy	Proteinkinasefamilyprotein
Aradu.LKW4E	Araip.X0K65	multiple-copy	proteinkinasefamilyprotein
Aradu.9W61Z	Araip.X0WZQ	multiple-copy	proteinkinasefamilyprotein
Aradu.J6F5H	Araip.X8YYD	multiple-copy	Proteinkinasefamilyprotein
Aradu.162IL	Araip.Y74NR	multiple-copy	Proteinkinasefamilyprotein
Aradu.H15D7	Araip.Z2RR8	multiple-copy	proteinkinasefamilyprotein
Aradu.V638G	Araip.Z7THM	multiple-copy	proteinkinasefamilyprotein
Aradu.QKQ8N	Araip.01ZYD	multiple-copy	Proteinkinasesuperfamilyprotein
Aradu.D1N5P	Araip.04KBR	multiple-copy	Proteinkinasesuperfamilyprotein
Aradu.LRS2W	Araip.0I565	multiple-copy	Proteinkinasesuperfamilyprotein
Aradu.S0HMQ	Araip.0T4FD	multiple-copy	Proteinkinasesuperfamilyprotein
Aradu.2E3F6	Araip.0Y9HM	multiple-copy	Proteinkinasesuperfamilyprotein
Aradu.J4R4W	Araip.11KLU	multiple-copy	Proteinkinasesuperfamilyprotein
Aradu.DT3LR	Araip.17Z7K	multiple-copy	Proteinkinasesuperfamilyprotein
Aradu.D03W8	Araip.1897A	multiple-copy	Proteinkinasesuperfamilyprotein
Aradu.J0SGA	Araip.19T2H	multiple-copy	Proteinkinasesuperfamilyprotein
Aradu.QXQ1E	Araip.19YRX	multiple-copy	Proteinkinasesuperfamilyprotein
Aradu.P47CH	Araip.1G1KY	multiple-copy	Proteinkinasesuperfamilyprotein
Aradu.JZI09	Araip.1JL4U	multiple-copy	Proteinkinasesuperfamilyprotein
Aradu.R1FC6	Araip.1JR2Q	multiple-copy	Proteinkinasesuperfamilyprotein
Aradu.JN5HH	Araip.1KL0I	multiple-copy	Proteinkinasesuperfamilyprotein
Aradu.BC220	Araip.1N4XS	multiple-copy	Proteinkinasesuperfamilyprotein
Aradu.XAE56	Araip.1PU5F	multiple-copy	Proteinkinasesuperfamilyprotein
Aradu.3T4GH	Araip.1RB5V	multiple-copy	Proteinkinasesuperfamilyprotein
Aradu.XS31R	Araip.1V6N7	multiple-copy	Proteinkinasesuperfamilyprotein
Aradu.U8TGW	Araip.1ZJ86	multiple-copy	Proteinkinasesuperfamilyprotein
Aradu.GMN03	Araip.29BWP	multiple-copy	Proteinkinasesuperfamilyprotein
Aradu.T0VC2	Araip.2W29K	multiple-copy	Proteinkinasesuperfamilyprotein
Aradu.F6N2R	Araip.313YK	multiple-copy	Proteinkinasesuperfamilyprotein
Aradu.CGX1Z	Araip.32FLV	multiple-copy	Proteinkinasesuperfamilyprotein
Aradu.IZ4SH	Araip.33UB6	multiple-copy	Proteinkinasesuperfamilyprotein
Aradu.18NVF	Araip.36HY2	multiple-copy	Proteinkinasesuperfamilyprotein
Aradu.A1BA5	Araip.3C14J	multiple-copy	Proteinkinasesuperfamilyprotein
Aradu.4AW6R	Araip.3D0I5	multiple-copy	Proteinkinasesuperfamilyprotein
Aradu.CCT0A	Araip.3E86P	multiple-copy	Proteinkinasesuperfamilyprotein
Aradu.BDN4J	Araip.3NM2I	multiple-copy	Proteinkinasesuperfamilyprotein
Aradu.HVW3A	Araip.467WI	multiple-copy	Proteinkinasesuperfamilyprotein

Aradu.CDA12	Araip.4K162	multiple-copy	Proteinkinasesuperfamilyprotein
Aradu.Z4RIW	Araip.4N0QC	multiple-copy	Proteinkinasesuperfamilyprotein
Aradu.RGQ53	Araip.4R6AS	multiple-copy	Proteinkinasesuperfamilyprotein
Aradu.D15YQ	Araip.4SA3R	multiple-copy	Proteinkinasesuperfamilyprotein
Aradu.K34WH	Araip.4Y5NV	multiple-copy	Proteinkinasesuperfamilyprotein
Aradu.20BZ5	Araip.51T0U	multiple-copy	Proteinkinasesuperfamilyprotein
Aradu.M38D2	Araip.53EWD	multiple-copy	Proteinkinasesuperfamilyprotein
Aradu.70NX8	Araip.5435Q	multiple-copy	Proteinkinasesuperfamilyprotein
Aradu.DF397	Araip.555KP	multiple-copy	Proteinkinasesuperfamilyprotein
Aradu.0EG1Y	Araip.563L5	multiple-copy	Proteinkinasesuperfamilyprotein
Aradu.82ABY	Araip.57QUV	multiple-copy	Proteinkinasesuperfamilyprotein
Aradu.R9TCK	Araip.59E1I	multiple-copy	Proteinkinasesuperfamilyprotein
Aradu.S8Y3J	Araip.5CL0B	multiple-copy	Proteinkinasesuperfamilyprotein
Aradu.2717A	Araip.5RN6F	multiple-copy	Proteinkinasesuperfamilyprotein
Aradu.M9QM1	Araip.5T83W	multiple-copy	Proteinkinasesuperfamilyprotein
Aradu.LV4I2	Araip.5U7G7	multiple-copy	Proteinkinasesuperfamilyprotein
Aradu.WL13F	Araip.60K6G	multiple-copy	Proteinkinasesuperfamilyprotein
Aradu.QVT4C	Araip.61EW7	multiple-copy	Proteinkinasesuperfamilyprotein
Aradu.SBA69	Araip.626V8	multiple-copy	Proteinkinasesuperfamilyprotein
Aradu.TE4V4	Araip.63QU3	multiple-copy	Proteinkinasesuperfamilyprotein
Aradu.K7I1H	Araip.65AHE	multiple-copy	Proteinkinasesuperfamilyprotein
Aradu.I0IKB	Araip.65H6H	multiple-copy	Proteinkinasesuperfamilyprotein
Aradu.SK9II	Araip.68UU0	multiple-copy	Proteinkinasesuperfamilyprotein
Aradu.VK93Q	Araip.6IZ1V	multiple-copy	Proteinkinasesuperfamilyprotein
Aradu.VR7VT	Araip.6Q6H8	multiple-copy	Proteinkinasesuperfamilyprotein
Aradu.NVM07	Araip.6VJ9V	multiple-copy	Proteinkinasesuperfamilyprotein
Aradu.2H1TE	Araip.75CVM	multiple-copy	Proteinkinasesuperfamilyprotein
Aradu.G9QEY	Araip.79CH7	multiple-copy	Proteinkinasesuperfamilyprotein
Aradu.WE6GV	Araip.7KT8S	multiple-copy	Proteinkinasesuperfamilyprotein
Aradu.Y5WI6	Araip.82B50	multiple-copy	Proteinkinasesuperfamilyprotein
Aradu.WH701	Araip.86XAN	multiple-copy	Proteinkinasesuperfamilyprotein
Aradu.B4UNP	Araip.88TVT	multiple-copy	Proteinkinasesuperfamilyprotein
Aradu.AET7G	Araip.8J47Q	multiple-copy	Proteinkinasesuperfamilyprotein
Aradu.K7USP	Araip.8Q1DE	multiple-copy	Proteinkinasesuperfamilyprotein
Aradu.7H9F1	Araip.8Q65G	multiple-copy	Proteinkinasesuperfamilyprotein
Aradu.4M072	Araip.8Y41D	multiple-copy	Proteinkinasesuperfamilyprotein
Aradu.VUE49	Araip.99AZK	multiple-copy	Proteinkinasesuperfamilyprotein
Aradu.LX505	Araip.9D3HV	multiple-copy	Proteinkinasesuperfamilyprotein
Aradu.U0T6A	Araip.9E8AI	multiple-copy	Proteinkinasesuperfamilyprotein
Aradu.N24L5	Araip.9V3ZQ	multiple-copy	Proteinkinasesuperfamilyprotein
Aradu.212W8	Araip.A6VYR	multiple-copy	Proteinkinasesuperfamilyprotein
Aradu.4Y1KN	Araip.A77RF	multiple-copy	Proteinkinasesuperfamilyprotein
Aradu.AEH7T	Araip.A830F	multiple-copy	Proteinkinasesuperfamilyprotein
Aradu.MU7BZ	Araip.A9YXZ	multiple-copy	Proteinkinasesuperfamilyprotein

Aradu.MKD6V	Araip.ACE37	multiple-copy	Proteinkinasesuperfamilyprotein
Aradu.S70AZ	Araip.ADR3S	multiple-copy	Proteinkinasesuperfamilyprotein
Aradu.I0X0M	Araip.AH1WN	multiple-copy	Proteinkinasesuperfamilyprotein
Aradu.U4Z04	Araip.AJL31	multiple-copy	Proteinkinasesuperfamilyprotein
Aradu.Q0IL1	Araip.AT3RC	multiple-copy	Proteinkinasesuperfamilyprotein
Aradu.SI0X3	Araip.AV5UM	multiple-copy	Proteinkinasesuperfamilyprotein
Aradu.516WS	Araip.B3QST	multiple-copy	Proteinkinasesuperfamilyprotein
Aradu.T8I2T	Araip.B4RCV	multiple-copy	Proteinkinasesuperfamilyprotein
Aradu.D3JBM	Araip.B6G88	multiple-copy	Proteinkinasesuperfamilyprotein
Aradu.P8XB7	Araip.B7ULW	multiple-copy	Proteinkinasesuperfamilyprotein
Aradu.65T0K	Araip.BG1TQ	multiple-copy	Proteinkinasesuperfamilyprotein
Aradu.M9J7Q	Araip.BG7KI	multiple-copy	Proteinkinasesuperfamilyprotein
Aradu.K358E	Araip.BKJ61	multiple-copy	Proteinkinasesuperfamilyprotein
Aradu.DLI1Q	Araip.BR2EY	multiple-copy	Proteinkinasesuperfamilyprotein
Aradu.ZC9DU	Araip.C0Q51	multiple-copy	Proteinkinasesuperfamilyprotein
Aradu.C8Q2A	Araip.C66NM	multiple-copy	Proteinkinasesuperfamilyprotein
Aradu.C4J7U	Araip.C83J2	multiple-copy	Proteinkinasesuperfamilyprotein
Aradu.8T260	Araip.CB2CZ	multiple-copy	Proteinkinasesuperfamilyprotein
Aradu.CVS4L	Araip.CJ248	multiple-copy	Proteinkinasesuperfamilyprotein
Aradu.X0KMT	Araip.CSV1L	multiple-copy	Proteinkinasesuperfamilyprotein
Aradu.V5XVE	Araip.D0E4F	multiple-copy	Proteinkinasesuperfamilyprotein
Aradu.HS8FV	Araip.D1IVY	multiple-copy	Proteinkinasesuperfamilyprotein
Aradu.ER4SD	Araip.D4H7P	multiple-copy	Proteinkinasesuperfamilyprotein
Aradu.473E6	Araip.D9TAI	multiple-copy	Proteinkinasesuperfamilyprotein
Aradu.ZAF9Y	Araip.DC4BQ	multiple-copy	Proteinkinasesuperfamilyprotein
Aradu.Q3FMD	Araip.DYJ2G	multiple-copy	Proteinkinasesuperfamilyprotein
Aradu.ZJT5U	Araip.E26HH	multiple-copy	Proteinkinasesuperfamilyprotein
Aradu.ZR16S	Araip.E472Z	multiple-copy	Proteinkinasesuperfamilyprotein
Aradu.7KC0A	Araip.E8195	multiple-copy	Proteinkinasesuperfamilyprotein
Aradu.AA5CG	Araip.EN7EZ	multiple-copy	Proteinkinasesuperfamilyprotein
Aradu.463D4	Araip.EP8U9	multiple-copy	Proteinkinasesuperfamilyprotein
Aradu.BB6TM	Araip.EPL8Z	multiple-copy	Proteinkinasesuperfamilyprotein
Aradu.EC406	Araip.EWU0K	multiple-copy	Proteinkinasesuperfamilyprotein
Aradu.JJ88W	Araip.F0Y7Z	multiple-copy	Proteinkinasesuperfamilyprotein
Aradu.JTA11	Araip.F7384	multiple-copy	Proteinkinasesuperfamilyprotein
Aradu.AQJ6I	Araip.F92P4	multiple-copy	Proteinkinasesuperfamilyprotein
Aradu.0QN5D	Araip.FDR3N	multiple-copy	Proteinkinasesuperfamilyprotein
Aradu.98KHN	Araip.FM4II	multiple-copy	Proteinkinasesuperfamilyprotein
Aradu.PGH8Z	Araip.FM7NI	multiple-copy	Proteinkinasesuperfamilyprotein
Aradu.H7AQV	Araip.FXZ38	multiple-copy	Proteinkinasesuperfamilyprotein
Aradu.3MI4M	Araip.G2J9V	multiple-copy	Proteinkinasesuperfamilyprotein
Aradu.FTI5H	Araip.G5FJE	multiple-copy	Proteinkinasesuperfamilyprotein
Aradu.X6BWV	Araip.GGP20	multiple-copy	Proteinkinasesuperfamilyprotein
Aradu.SDR46	Araip.GIN3T	multiple-copy	Proteinkinasesuperfamilyprotein

Aradu.Q7508	Araip.GVN2J	multiple-copy	Proteinkinasesuperfamilyprotein
Aradu.WIS65	Araip.H4Q41	multiple-copy	Proteinkinasesuperfamilyprotein
Aradu.AR7XM	Araip.H663M	multiple-copy	Proteinkinasesuperfamilyprotein
Aradu.6FR0W	Araip.H9INU	multiple-copy	Proteinkinasesuperfamilyprotein
Aradu.L4D0H	Araip.HKZ7Q	multiple-copy	Proteinkinasesuperfamilyprotein
Aradu.20C0X	Araip.HTQ91	multiple-copy	Proteinkinasesuperfamilyprotein
Aradu.N2YDU	Araip.I1U0Y	multiple-copy	Proteinkinasesuperfamilyprotein
Aradu.NEM0A	Araip.I2EVD	multiple-copy	Proteinkinasesuperfamilyprotein
Aradu.3TD2D	Araip.I5BDL	multiple-copy	Proteinkinasesuperfamilyprotein
Aradu.PS45N	Araip.I5XHJ	multiple-copy	Proteinkinasesuperfamilyprotein
Aradu.IP5K7	Araip.IL3I2	multiple-copy	Proteinkinasesuperfamilyprotein
Aradu.UZJ7G	Araip.IPQ80	multiple-copy	Proteinkinasesuperfamilyprotein
Aradu.0SM3T	Araip.J4V1E	multiple-copy	Proteinkinasesuperfamilyprotein
Aradu.VCF9B	Araip.JEI3K	multiple-copy	Proteinkinasesuperfamilyprotein
Aradu.E1KPT	Araip.JL2EX	multiple-copy	Proteinkinasesuperfamilyprotein
Aradu.934TY	Araip.JMT0Y	multiple-copy	Proteinkinasesuperfamilyprotein
Aradu.DA1ZW	Araip.JSX23	multiple-copy	Proteinkinasesuperfamilyprotein
Aradu.305W9	Araip.JY0X0	multiple-copy	Proteinkinasesuperfamilyprotein
Aradu.EW7BI	Araip.K54B1	multiple-copy	Proteinkinasesuperfamilyprotein
Aradu.YQ4PR	Araip.K9KUX	multiple-copy	proteinkinase1B
Aradu.MP2U2	Araip.L02B7	multiple-copy	Proteinkinasesuperfamilyprotein
Aradu.JWM4N	Araip.L153Y	multiple-copy	Proteinkinasesuperfamilyprotein
Aradu.5K7DH	Araip.L8EL7	multiple-copy	Proteinkinasesuperfamilyprotein
Aradu.RCT4K	Araip.L9GIV	multiple-copy	Proteinkinasesuperfamilyprotein
Aradu.U4XDJ	Araip.LA6RB	multiple-copy	Proteinkinasesuperfamilyprotein
Aradu.6Z59M	Araip.LD20M	multiple-copy	Proteinkinasesuperfamilyprotein
Aradu.Q0T0K	Araip.LDR2S	multiple-copy	Proteinkinasesuperfamilyprotein
Aradu.VA4EQ	Araip.LM4PJ	multiple-copy	Proteinkinasesuperfamilyprotein
Aradu.A68MB	Araip.LMV71	multiple-copy	Proteinkinasesuperfamilyprotein
Aradu.SLM5J	Araip.LV17J	multiple-copy	Proteinkinasesuperfamilyprotein
Aradu.31PM4	Araip.M87GM	multiple-copy	Proteinkinasesuperfamilyprotein
Aradu.P2MN3	Araip.MC5NI	multiple-copy	Proteinkinasesuperfamilyprotein
Aradu.NIR19	Araip.MM2M0	multiple-copy	Proteinkinasesuperfamilyprotein
Aradu.YXX50	Araip.MXV6P	multiple-copy	Proteinkinasesuperfamilyprotein
Aradu.W5WIK	Araip.N8HHR	multiple-copy	Proteinkinasesuperfamilyprotein
Aradu.V43LA	Araip.NB0BG	multiple-copy	Proteinkinasesuperfamilyprotein
Aradu.BP7BM	Araip.NNQ15	multiple-copy	Proteinkinasesuperfamilyprotein
Aradu.L2IAX	Araip.NP5CT	multiple-copy	Proteinkinasesuperfamilyprotein
Aradu.YMP6X	Araip.NRD23	multiple-copy	Proteinkinasesuperfamilyprotein
Aradu.E1ZU5	Araip.P1N43	multiple-copy	Proteinkinasesuperfamilyprotein
Aradu.PF1HF	Araip.P4X64	multiple-copy	Proteinkinasesuperfamilyprotein
Aradu.EG5ES	Araip.PE2QK	multiple-copy	Proteinkinasesuperfamilyprotein
Aradu.AFL8M	Araip.PL90G	multiple-copy	Proteinkinasesuperfamilyprotein
Aradu.FL7H6	Araip.PM2EU	multiple-copy	Proteinkinasesuperfamilyprotein

Aradu.0Y2ZI	Araip.PP40A	multiple-copy	Proteinkinasesuperfamilyprotein
Aradu.ZCP8X	Araip.Q00WT	multiple-copy	Proteinkinasesuperfamilyprotein
Aradu.TP6EC	Araip.Q5EBR	multiple-copy	Proteinkinasesuperfamilyprotein
Aradu.LT240	Araip.Q779B	multiple-copy	Proteinkinasesuperfamilyprotein
Aradu.WX32R	Araip.QJ6DW	multiple-copy	Proteinkinasesuperfamilyprotein
Aradu.GZ0CM	Araip.QL2KR	multiple-copy	Proteinkinasesuperfamilyprotein
Aradu.P1KDA	Araip.RA1WW	multiple-copy	Proteinkinasesuperfamilyprotein
Aradu.31VER	Araip.RF1QP	multiple-copy	Proteinkinasesuperfamilyprotein
Aradu.22650	Araip.RL3D3	multiple-copy	Proteinkinasesuperfamilyprotein
Aradu.AAH6J	Araip.RM36F	multiple-copy	Proteinkinasesuperfamilyprotein
Aradu.Y7TD6	Araip.RMF9N	multiple-copy	Proteinkinasesuperfamilyprotein
Aradu.HG75V	Araip.RN6JS	multiple-copy	Proteinkinasesuperfamilyprotein
Aradu.L02C7	Araip.RZ2KY	multiple-copy	Proteinkinasesuperfamilyprotein
Aradu.NE81M	Araip.RZ5RS	multiple-copy	Proteinkinasesuperfamilyprotein
Aradu.E52Z4	Araip.RZ7V8	multiple-copy	Proteinkinasesuperfamilyprotein
Aradu.2UI08	Araip.S6BCW	multiple-copy	Proteinkinasesuperfamilyprotein
Aradu.2JA77	Araip.SD9JV	multiple-copy	Proteinkinasesuperfamilyprotein
Aradu.C19JT	Araip.SH2LK	multiple-copy	Proteinkinasesuperfamilyprotein
Aradu.FWZ0U	Araip.SQD8Z	multiple-copy	Proteinkinasesuperfamilyprotein
Aradu.UDC1Q	Araip.SS4HT	multiple-copy	Proteinkinasesuperfamilyprotein
Aradu.4FA0W	Araip.T0473	multiple-copy	Proteinkinasesuperfamilyprotein
Aradu.SM8ND	Araip.T2D4X	multiple-copy	Proteinkinasesuperfamilyprotein
Aradu.W3H15	Araip.T94UV	multiple-copy	Proteinkinasesuperfamilyprotein
Aradu.JG217	Araip.TL0AY	multiple-copy	Proteinkinasesuperfamilyprotein
Aradu.0Z7KI	Araip.TYZ14	multiple-copy	Proteinkinasesuperfamilyprotein
Aradu.93Y1F	Araip.U4Z8Q	multiple-copy	Proteinkinasesuperfamilyprotein
Aradu.T0K7A	Araip.UF0MQ	multiple-copy	Proteinkinasesuperfamilyprotein
Aradu.48GRH	Araip.UJ50A	multiple-copy	Proteinkinasesuperfamilyprotein
Aradu.G7DFD	Araip.ULK6W	multiple-copy	Proteinkinasesuperfamilyprotein
Aradu.HS2H7	Araip.V57W5	multiple-copy	Proteinkinasesuperfamilyprotein
Aradu.A42Y8	Araip.V7L8V	multiple-copy	Proteinkinasesuperfamilyprotein
Aradu.I67GN	Araip.VAX9L	multiple-copy	Proteinkinasesuperfamilyprotein
Aradu.8Y4W7	Araip.VLF9V	multiple-copy	Proteinkinasesuperfamilyprotein
Aradu.Y85YF	Araip.VS9N0	multiple-copy	Proteinkinasesuperfamilyprotein
Aradu.5XQ31	Araip.VV837	multiple-copy	Proteinkinasesuperfamilyprotein
Aradu.PSU1K	Araip.VW89Q	multiple-copy	Proteinkinasesuperfamilyprotein
Aradu.3GX0M	Araip.W7XT8	multiple-copy	Proteinkinasesuperfamilyprotein
Aradu.CBY05	Araip.WNQ27	multiple-copy	Proteinkinasesuperfamilyprotein
Aradu.BWC86	Araip.WP8Q7	multiple-copy	Proteinkinasesuperfamilyprotein
Aradu.B18E9	Araip.WVS7I	multiple-copy	proteinkinase1B
Aradu.4R7ZC	Araip.X52X0	multiple-copy	Proteinkinasesuperfamilyprotein
Aradu.EU5HZ	Araip.X69G6	multiple-copy	Proteinkinasesuperfamilyprotein
Aradu.DAC70	Araip.X7ERC	multiple-copy	Proteinkinasesuperfamilyprotein
Aradu.2123V	Araip.XJA18	multiple-copy	Proteinkinasesuperfamilyprotein

Aradu.0QJ0H	Araip.XLI18	multiple-copy	Proteinkinasesuperfamilyprotein
Aradu.C5UHL	Araip.Y8SXT	multiple-copy	Proteinkinasesuperfamilyprotein
Aradu.V3IQ0	Araip.Y9Y5W	multiple-copy	Proteinkinasesuperfamilyprotein
Aradu.4A33P	Araip.Z1JK3	multiple-copy	Proteinkinasesuperfamilyprotein
Aradu.DRK7I	Araip.Z3DP0	multiple-copy	Proteinkinasesuperfamilyprotein
Aradu.0GZ0B	Araip.ZD7NF	multiple-copy	Proteinkinasesuperfamilyprotein
Aradu.PV4QE	Araip.ZH99C	multiple-copy	Proteinkinasesuperfamilyprotein
Aradu.2W1AJ	Araip.ZR9LA	multiple-copy	Proteinkinasesuperfamilyprotein
Aradu.6JP4A	Araip.ZSM85	multiple-copy	Proteinkinasesuperfamilyprotein
Aradu.B3611	Araip.GQE2Q	multiple-copy	Protein-tyrosinephosphatasen=3Tax=ArabidopsisRepID=
Aradu.BZA24	Araip.FZ182	multiple-copy	protonpumpinteractor1
Aradu.2UZ0C	Araip.3TF2U	multiple-copy	Pseudouridinesynthasefamilyprotein
Aradu.81WTS	Araip.4I67D	multiple-copy	Pseudouridinesynthasefamilyprotein
Aradu.55H8J	Araip.7UJ37	multiple-copy	Pseudouridinesynthasefamilyprotein
Aradu.31ZWP	Araip.NA763	multiple-copy	Pseudouridinesynthasefamilyprotein
Aradu.B1CPC	Araip.Y5JHT	multiple-copy	Pseudouridinesynthasefamilyprotein
Aradu.YBC8E	Araip.YKJ2E	multiple-copy	Pseudouridinesynthasefamilyprotein
Aradu.5DD09	Araip.NS0VF	multiple-copy	pterin-4-alpha-carbinolaminedehydratase
Aradu.3LW1Z	Araip.51ELU	multiple-copy	pumilio-familyRNA-bindingrepeatprotein
Aradu.8C561	Araip.F5WNS	multiple-copy	pumilio-familyRNA-bindingrepeatprotein
Aradu.KWX5Q	Araip.GE4AA	multiple-copy	pumilio-familyRNA-bindingrepeatprotein
Aradu.LZ2RQ	Araip.B12DL	multiple-copy	purinepermease10
Aradu.T7TUD	Araip.IDM52	multiple-copy	purinepermease11
Aradu.8F945	Araip.N04DW	multiple-copy	purinepermease10
Aradu.M785W	Araip.VMV0J	multiple-copy	purinepermease11
Aradu.272PC	Araip.1C20S	multiple-copy	purinepermease1
Aradu.DL7C8	Araip.W3RGE	multiple-copy	purinepermease5
Aradu.5K7BE	Araip.ME59K	multiple-copy	purpleacidphosphatase17
Aradu.A2F1I	Araip.VV0DQ	multiple-copy	purpleacidphosphatase17
Aradu.E2DS0	Araip.422GS	multiple-copy	purpleacidphosphatase18
Aradu.3K2CT	Araip.T7TAP	multiple-copy	purpleacidphosphatase18
Aradu.GA66V	Araip.83S01	multiple-copy	purpleacidphosphatase22
Aradu.EEP0U	Araip.A48MR	multiple-copy	purpleacidphosphatase22
Aradu.PXT5K	Araip.DN1XY	multiple-copy	purpleacidphosphatase23
Aradu.4J3R3	Araip.NN141	multiple-copy	purpleacidphosphatase27
Aradu.PZB3C	Araip.XVM4V	multiple-copy	purpleacidphosphatase29
Aradu.6PG6R	Araip.G03BG	multiple-copy	purpleacidphosphatase3
Aradu.5Z6H3	Araip.KU01P	multiple-copy	purpleacidphosphatase9
Aradu.YF31D	Araip.24Q48	multiple-copy	PWWPdomainprotein
Aradu.BWM82	Araip.5MT98	multiple-copy	Pyridoxalphosphate(PLP)-dependenttransferasessuperfa
Aradu.6T4IB	Araip.7A9DT	multiple-copy	Pyridoxalphosphate(PLP)-dependenttransferasessuperfa
Aradu.WR10B	Araip.VR692	multiple-copy	pyruvatedehydrogenaseE1beta
Aradu.YJ46C	Araip.Z1FA8	multiple-copy	pyruvatedehydrogenaseE1componentsubunitbeta
Aradu.ZD7QJ	Araip.T0P1U	multiple-copy	pyruvatedehydrogenasekinase

Aradu.LF0Z4	Araip.BGK8H	multiple-copy	Pyruvatekinasefamilyprotein
Aradu.QE3CA	Araip.F9ULB	multiple-copy	Pyruvatekinasefamilyprotein
Aradu.8IN6N	Araip.L7NBZ	multiple-copy	Pyruvatekinasefamilyprotein
Aradu.SG5AP	Araip.JY8IW	multiple-copy	Quinonereductasefamilyprotein
Aradu.D0ZYM	Araip.L7HAD	multiple-copy	Quinonereductasefamilyprotein
Aradu.4206R	Araip.LE8QH	multiple-copy	Quinonereductasefamilyprotein
Aradu.G2CUK	Araip.PR8W2	multiple-copy	Quinonereductasefamilyprotein
Aradu.808LA	Araip.V72QV	multiple-copy	Quinonereductasefamilyprotein
Aradu.RDW4B	Araip.EGG9T	multiple-copy	QWRFmotif-containingprotein2-likeisoformX1[Glycinema
Aradu.T922Y	Araip.ULA1J	multiple-copy	QWRFmotif-containingprotein8-likeisoformX6[Glycinema
Aradu.AP5EP	Araip.G8MZ3	multiple-copy	QWRFmotif-containingprotein8-likeisoformX4[Glycinema
Aradu.72K0W	Araip.2M8UJ	multiple-copy	Rab5-interactingfamilyprotein
Aradu.RCS61	Araip.776JT	multiple-copy	RABGDPdissociationinhibitor2
Aradu.SJR7G	Araip.FU5J9	multiple-copy	RABGDPdissociationinhibitor2
Aradu.8Q40H	Araip.F3RJ4	multiple-copy	RabGTPaseactivator
Aradu.4XL4D	Araip.J9YK7	multiple-copy	RabGTPaseactivator
Aradu.Q123V	Araip.K1692	multiple-copy	RabGTPaseactivator
Aradu.ZF31Q	Araip.N3W3L	multiple-copy	RabGTPaseactivator
Aradu.U6HMB	Araip.BUM43	multiple-copy	RABGTPasehomolog7A
Aradu.8I2Q1	Araip.EKH4M	multiple-copy	RABGTPasehomolog7A
Aradu.87VJS	Araip.GRP0P	multiple-copy	rac-likeGTP-bindingprotein7-like[Glycinemax]
Aradu.W9Q7M	Araip.JYD7I	multiple-copy	rac-likeGTP-bindingprotein7-like[Glycinemax]
Aradu.M70VI	Araip.L8PD6	multiple-copy	ralf-like4
Aradu.HD6HL	Araip.445G9	multiple-copy	Ran-bindingprotein6n=72Tax=EutheriaRepID=RNBP6_H
Aradu.H5ED7	Araip.880AZ	multiple-copy	Ran-bindingprotein6n=72Tax=EutheriaRepID=RNBP6_H
Aradu.8SH4F	Araip.QC86V	multiple-copy	rapidalkalinizationfactor1
Aradu.C0HL8	Araip.RA9BV	multiple-copy	Ras-relatedsmallGTP-bindingfamilyprotein
Aradu.ZQ118	Araip.CU4A2	multiple-copy	receptorkinase1
Aradu.UR751	Araip.I6EYC	multiple-copy	receptorkinase1
Aradu.IZ0B7	Araip.W8764	multiple-copy	receptorkinase1
Aradu.12LHJ	Araip.137YQ	multiple-copy	receptorkinase2
Aradu.3N4PQ	Araip.69A15	multiple-copy	receptorkinase2
Aradu.B4US9	Araip.7H0TY	multiple-copy	receptorkinase2
Aradu.NJH70	Araip.A35MH	multiple-copy	receptorkinase2
Aradu.25H68	Araip.JW3M4	multiple-copy	receptorkinase2
Aradu.CT0FI	Araip.LD5UC	multiple-copy	receptorkinase2
Aradu.ZYE2B	Araip.MI9T9	multiple-copy	receptorkinase2
Aradu.LQA0K	Araip.N6W5R	multiple-copy	receptorkinase2
Aradu.UY6CA	Araip.PBR2C	multiple-copy	receptorkinase2
Aradu.M15HC	Araip.T81Z0	multiple-copy	receptorkinase3
Aradu.Z17B7	Araip.VK9DV	multiple-copy	receptorkinase2
Aradu.DHT3V	Araip.17GEB	multiple-copy	receptorkinase1
Aradu.WBB7S	Araip.AE3J6	multiple-copy	receptorlectinkinase
Aradu.7X64R	Araip.H796R	multiple-copy	receptorlectinkinase

Aradu.QD7TL	Araip.R9EZV	multiple-copy	receptorlectinkinase
Aradu.Y1B10	Araip.0WL30	multiple-copy	receptor-likekinase
Aradu.DD99U	Araip.26WUC	multiple-copy	receptor-likekinase
Aradu.K0D8U	Araip.29WB6	multiple-copy	receptor-likekinase
Aradu.ZB2RS	Araip.5ZK6T	multiple-copy	receptor-likekinase
Aradu.HZ14S	Araip.9ND7T	multiple-copy	receptor-likekinase
Aradu.ISN4P	Araip.IY0UZ	multiple-copy	receptor-likekinase
Aradu.M3FDP	Araip.K28CA	multiple-copy	receptor-likekinase
Aradu.U78VM	Araip.NRD2A	multiple-copy	receptor-likekinase
Aradu.Z101N	Araip.R2SZJ	multiple-copy	receptor-likekinase
Aradu.QR44H	Araip.VU9PS	multiple-copy	receptor-likekinase
Aradu.WLZ4Q	Araip.04VV6	multiple-copy	receptor-likekinase1
Aradu.MQ4WG	Araip.08Z17	multiple-copy	receptor-likekinase1
Aradu.2F99R	Araip.0J4UH	multiple-copy	receptor-likekinase1
Aradu.BS3NC	Araip.19369	multiple-copy	receptor-likekinase1
Aradu.42UET	Araip.4LL3W	multiple-copy	receptor-likekinase1
Aradu.53A4D	Araip.7E8G5	multiple-copy	receptor-likekinase1
Aradu.X9DDA	Araip.7F2XC	multiple-copy	receptor-likekinase1
Aradu.C6EHZ	Araip.7G9YB	multiple-copy	receptor-likekinase1
Aradu.550LU	Araip.8SB48	multiple-copy	receptor-likekinase1
Aradu.QL7M2	Araip.BTV23	multiple-copy	receptor-likekinase1
Aradu.I32AI	Araip.E5UFR	multiple-copy	receptor-likekinase1
Aradu.YL3ZN	Araip.GB07R	multiple-copy	receptor-likekinase1
Aradu.BE6XZ	Araip.H8VZN	multiple-copy	receptor-likekinase1
Aradu.3WJ36	Araip.HIF1W	multiple-copy	receptor-likekinase1
Aradu.RAS9Y	Araip.J3UIZ	multiple-copy	receptor-likekinase1
Aradu.14EED	Araip.JWP0N	multiple-copy	receptor-likekinase1
Aradu.8W40E	Araip.K50Y0	multiple-copy	receptor-likekinase1
Aradu.WT2VD	Araip.KX3VJ	multiple-copy	receptor-likekinase1
Aradu.HA4YC	Araip.S6A4N	multiple-copy	receptor-likekinase1
Aradu.I9JU3	Araip.TM0T5	multiple-copy	receptor-likekinase1
Aradu.HNS4U	Araip.WL53Y	multiple-copy	receptor-likekinase1
Aradu.Q26PA	Araip.XFD5U	multiple-copy	receptor-likekinase1
Aradu.JL69F	Araip.XLK0H	multiple-copy	receptor-likekinase902
Aradu.7Z02Q	Araip.Y0ZFX	multiple-copy	receptor-likekinase1
Aradu.T8IMJ	Araip.ZGM3R	multiple-copy	receptor-likekinase1
Aradu.X4KI4	Araip.10CZT	multiple-copy	receptor-likekinase1
Aradu.PA8BP	Araip.A2DFF	multiple-copy	receptor-likekinase902
Aradu.XX2R4	Araip.D1TZE	multiple-copy	receptor-likekinase1
Aradu.SG5CY	Araip.RU3FR	multiple-copy	receptor-likekinase1
Aradu.9GN4P	Araip.WJ0C8	multiple-copy	receptor-likekinase902
Aradu.JJU8Z	Araip.C9NM5	multiple-copy	receptorlikeprotein35
Aradu.ANT5S	Araip.W7ZUF	multiple-copy	receptor-likeproteinkinase2
Aradu.WU7D1	Araip.162FJ	multiple-copy	receptor-likeproteinkinase1

Aradu.58K6Q	Araip.2K79X	multiple-copy	receptor-likekinasefamilyprotein
Aradu.AI3EU	Araip.4263U	multiple-copy	receptor-likeproteinkinase1
Aradu.DM7P1	Araip.B4JB5	multiple-copy	receptor-likeproteinkinase1
Aradu.3T5V1	Araip.KI3H8	multiple-copy	receptor-likeproteinkinase1
Aradu.YZ9KD	Araip.X50TB	multiple-copy	receptor-likeproteinkinase1
Aradu.LRZ4G	Araip.YBI64	multiple-copy	receptor-likeproteinkinase1
Aradu.PXP20	Araip.06PW6	multiple-copy	receptor-likeproteinkinase2
Aradu.8GL4A	Araip.24ZZQ	multiple-copy	receptor-likeproteinkinase2
Aradu.G63Y1	Araip.3D13X	multiple-copy	receptor-likeproteinkinase2
Aradu.XDM9G	Araip.3G6B2	multiple-copy	receptor-likeproteinkinase2
Aradu.CC1R3	Araip.3K2R6	multiple-copy	receptor-likeproteinkinase2
Aradu.0SV2V	Araip.87PIS	multiple-copy	receptor-likeproteinkinase2
Aradu.CBT6M	Araip.A8G61	multiple-copy	receptor-likeproteinkinase2
Aradu.C64A0	Araip.BHZ61	multiple-copy	receptor-likeproteinkinase2
Aradu.CR9NG	Araip.CF1GV	multiple-copy	receptor-likeproteinkinase2
Aradu.S0DLC	Araip.D942R	multiple-copy	receptor-likekinase
Aradu.T12XR	Araip.E75HV	multiple-copy	receptor-likeproteinkinase2
Aradu.M89U9	Araip.EFP5Y	multiple-copy	receptor-likeproteinkinase2
Aradu.V824B	Araip.F2IB2	multiple-copy	receptor-likeproteinkinase2
Aradu.F623P	Araip.F3F2F	multiple-copy	receptor-likeproteinkinase1
Aradu.B99AB	Araip.FJB0B	multiple-copy	receptor-likeproteinkinase2
Aradu.H128Z	Araip.GMV49	multiple-copy	receptor-likeproteinkinase2
Aradu.9602J	Araip.HKC53	multiple-copy	receptor-likeproteinkinase2
Aradu.AH39E	Araip.HQ67P	multiple-copy	receptor-likeproteinkinase2
Aradu.N2VA8	Araip.JU6JI	multiple-copy	receptor-likeproteinkinase2
Aradu.HH2HB	Araip.K6G0G	multiple-copy	receptor-likeproteinkinase2
Aradu.N4HHX	Araip.KQ1P6	multiple-copy	receptor-likeproteinkinase2
Aradu.V05DH	Araip.KZ67T	multiple-copy	receptor-likeproteinkinase2
Aradu.27T3V	Araip.L8Z1N	multiple-copy	receptor-likeproteinkinase2
Aradu.398CK	Araip.MS7KA	multiple-copy	receptor-likeproteinkinase2
Aradu.DW9IA	Araip.MY88Z	multiple-copy	receptor-likeproteinkinase2
Aradu.C1KJF	Araip.PS43L	multiple-copy	receptor-likeproteinkinase2
Aradu.WH49R	Araip.S4S1Y	multiple-copy	receptor-likeproteinkinase2
Aradu.EL0FF	Araip.S9R4E	multiple-copy	receptor-likeproteinkinase2
Aradu.CU9HL	Araip.SP6K4	multiple-copy	receptor-likeproteinkinase2
Aradu.FL9HS	Araip.SS9V6	multiple-copy	receptor-likeproteinkinase2
Aradu.UI0N2	Araip.T9ZFK	multiple-copy	receptor-likeproteinkinase2
Aradu.XIP04	Araip.TPM3R	multiple-copy	receptor-likeproteinkinase2
Aradu.E0AA1	Araip.V1HRY	multiple-copy	receptor-likeproteinkinase2
Aradu.ZRM2P	Araip.WH0MC	multiple-copy	receptor-likeproteinkinase2
Aradu.M0ENQ	Araip.WT898	multiple-copy	receptor-likeproteinkinase2
Aradu.GG6GL	Araip.X5428	multiple-copy	receptor-likeproteinkinase2
Aradu.671CI	Araip.X7R9Y	multiple-copy	receptor-likeproteinkinase1
Aradu.L72JH	Araip.XF870	multiple-copy	receptor-likeproteinkinase2

Aradu.RTQ8K	Araip.XKH8G	multiple-copy	receptor-likeproteinkinase2
Aradu.8IX7E	Araip.Y67XK	multiple-copy	receptor-likeproteinkinase2
Aradu.55F3D	Araip.YFY4A	multiple-copy	receptor-likeproteinkinase2
Aradu.T5WNW	Araip.Z9LLX	multiple-copy	receptor-likeproteinkinase2
Aradu.STU80	Araip.0K3NF	multiple-copy	receptor-likeproteinkinase2-like[Glycinemax]
Aradu.DGW3H	Araip.18BT4	multiple-copy	receptor-likeproteinkinase4
Aradu.EE1XG	Araip.3M2RS	multiple-copy	receptor-likeproteinkinase4
Aradu.ZS4C4	Araip.4YR0R	multiple-copy	receptor-likeproteinkinase4
Aradu.K3YJZ	Araip.56WDA	multiple-copy	receptor-likeproteinkinase4
Aradu.A0XEY	Araip.5H0GE	multiple-copy	receptor-likeproteinkinase4
Aradu.60C6S	Araip.69WKH	multiple-copy	receptor-likeproteinkinase4
Aradu.CQ85M	Araip.840F4	multiple-copy	receptor-likeproteinkinase4
Aradu.28IY3	Araip.DBK4N	multiple-copy	receptor-likeproteinkinase4
Aradu.ZZ9EE	Araip.E291T	multiple-copy	receptor-likeproteinkinase4
Aradu.BJL71	Araip.ER6JN	multiple-copy	receptor-likeproteinkinase4
Aradu.54M2C	Araip.F2I2G	multiple-copy	receptor-likeproteinkinase4
Aradu.UEF4U	Araip.PS4N8	multiple-copy	receptor-likeproteinkinase4
Aradu.GJY15	Araip.QF4IK	multiple-copy	receptor-likeproteinkinase4
Aradu.712AL	Araip.V8K0J	multiple-copy	receptor-likeproteinkinase4
Aradu.X8LK6	Araip.XN478	multiple-copy	receptor-likeproteinkinase-like[Glycinemax]
Aradu.4HH0P	Araip.2V74Q	multiple-copy	receptor-likeserine/threoninekinase2
Aradu.L4EY2	Araip.3074P	multiple-copy	receptor-likeserine/threoninekinase2
Aradu.80RUZ	Araip.5E8U9	multiple-copy	receptor-likeserine/threoninekinase2
Aradu.9LI83	Araip.62J85	multiple-copy	receptor-likeserine/threoninekinase2
Aradu.YVF29	Araip.70K3K	multiple-copy	receptor-likeserine/threoninekinase2
Aradu.56PKD	Araip.8UK7H	multiple-copy	receptor-likeserine/threoninekinase2
Aradu.0SN1Y	Araip.900EH	multiple-copy	receptor-likeserine/threoninekinase2
Aradu.ACY83	Araip.95WQJ	multiple-copy	receptor-likeserine/threoninekinase2
Aradu.AX3Z0	Araip.97MHJ	multiple-copy	receptor-likeserine/threoninekinase2
Aradu.JTL52	Araip.HVP05	multiple-copy	receptor-likeserine/threoninekinase2
Aradu.YW23C	Araip.J34CB	multiple-copy	receptor-likeserine/threoninekinase2
Aradu.NI7KT	Araip.MU39B	multiple-copy	receptor-likeserine/threoninekinase2
Aradu.R8UZU	Araip.NTQ7I	multiple-copy	receptor-likeserine/threoninekinase2
Aradu.CED81	Araip.QR111	multiple-copy	receptor-likeserine/threoninekinase2
Aradu.D0NVI	Araip.S1I4L	multiple-copy	receptor-likeserine/threoninekinase2
Aradu.JLN1Z	Araip.4A7P4	multiple-copy	receptorproteinkinase-related
Aradu.WQP25	Araip.E60FJ	multiple-copy	receptorproteinkinase-related
Aradu.ZYW5P	Araip.N95NQ	multiple-copy	receptorproteinkinase-related
Aradu.J2H81	Araip.Q1Z2E	multiple-copy	receptorserine/threoninekinase,putative
Aradu.J5BIS	Araip.689G8	multiple-copy	Regulatorofchromosomecondensation(RCC1)familyprote
Aradu.89I67	Araip.8U4CD	multiple-copy	Regulatorofchromosomecondensation(RCC1)familyprote
Aradu.W9ER9	Araip.D91QG	multiple-copy	Regulatorofchromosomecondensation(RCC1)familyprote
Aradu.Z0X17	Araip.F1AX6	multiple-copy	Regulatorofchromosomecondensation(RCC1)familyprote
Aradu.YA6PI	Araip.GVK2U	multiple-copy	Regulatorofchromosomecondensation(RCC1)familyprote

Aradu.RQ0GF	Araip.SVN4N	multiple-copy	Regulatorofchromosomecondensation(RCC1)familyprote
Aradu.V2KKS	Araip.W3TWU	multiple-copy	Regulatorofchromosomecondensation(RCC1)familyprote
Aradu.X4JYD	Araip.YIH0B	multiple-copy	Regulatorofchromosomecondensation(RCC1)familyprote
Aradu.124FF	Araip.YQ272	multiple-copy	Regulatorofchromosomecondensation(RCC1)familyprote
Aradu.IYG99	Araip.ZSL68	multiple-copy	Regulatorofchromosomecondensation(RCC1)familyprote
Aradu.KC0GC	Araip.8W4UM	multiple-copy	RegulatorofVps4activityintheMVBpathwayprotein
Aradu.L3UB6	Araip.H1Y7A	multiple-copy	RegulatorofVps4activityintheMVBpathwayprotein
Aradu.R23AB	Araip.M2HVA	multiple-copy	RegulatorofVps4activityintheMVBpathwayprotein
Aradu.23ZKM	Araip.Q87QY	multiple-copy	RegulatorofVps4activityintheMVBpathwayprotein
Aradu.XH3ZX	Araip.R9PC3	multiple-copy	RegulatorofVps4activityintheMVBpathwayprotein
Aradu.102BT	Araip.UJ652	multiple-copy	RegulatorofVps4activityintheMVBpathwayprotein
Aradu.61Y7W	Araip.B9HDL	multiple-copy	RELA/SPOThomolog3
Aradu.A49KD	Araip.GD2Y5	multiple-copy	RELA/SPOThomolog3
Aradu.ANN7C	Araip.KN052	multiple-copy	Remorinfamilyprotein
Aradu.ZL56D	Araip.ZRU67	multiple-copy	Remorinfamilyprotein
Aradu.5N8I2	Araip.43TDN	multiple-copy	remorin-like[Glycinemax]
Aradu.BR6GB	Araip.5FP0F	multiple-copy	remorin-like[Glycinemax]
Aradu.L7ZR8	Araip.D5D4T	multiple-copy	remorin-like[Glycinemax]
Aradu.B98C6	Araip.LC8CF	multiple-copy	remorin-like[Glycinemax]
Aradu.H7GIV	Araip.4DY64	multiple-copy	replicationproteinA70kDaDNA-bindingsubunitD-like[Glyci
Aradu.HS2F0	Araip.BK7XJ	multiple-copy	replicationfactor-Acarboxy-terminaldomainprotein
Aradu.EZW7V	Araip.CER5U	multiple-copy	replicationproteinA70kDaDNA-bindingsubunitA-like[Glyci
Aradu.T3QX0	Araip.J86TR	multiple-copy	replicationproteinA70kDaDNA-bindingsubunitA-like[Glyci
Aradu.D4F55	Araip.0H2X8	multiple-copy	replicationproteinA70kDaDNA-bindingsubunitC-like[Glyci
Aradu.T9UN0	Araip.2A6VZ	multiple-copy	replicationproteinA70kDaDNA-bindingsubunitC-like[Glyci
Aradu.MX1D6	Araip.AI0JW	multiple-copy	Replicationfactor-Aprotein1-related
Aradu.J2RZP	Araip.RT5CP	multiple-copy	respiratoryburstoxidasehomologueD
Aradu.92NW5	Araip.C2I9V	multiple-copy	respiratoryburstoxidaseproteinF
Aradu.J7KPQ	Araip.HQ9VR	multiple-copy	respiratoryburstoxidaseproteinF
Aradu.W2RXK	Araip.08M0G	multiple-copy	responseregulator12
Aradu.2MS2X	Araip.Z2ASR	multiple-copy	responseregulator24
Aradu.764WC	Araip.2QS8R	multiple-copy	responseregulator5
Aradu.Z330P	Araip.E6IX0	multiple-copy	responseregulator9
Aradu.LK1UG	Araip.CAU9S	multiple-copy	Reticulonfamilyprotein
Aradu.HU672	Araip.FV4ZU	multiple-copy	Reticulonfamilyprotein
Aradu.N7C0U	Araip.FZ2I8	multiple-copy	Reticulonfamilyprotein
Aradu.0QQ3Z	Araip.KMZ8M	multiple-copy	Reticulonfamilyprotein
Aradu.003TN	Araip.LVH53	multiple-copy	Reticulonfamilyprotein
Aradu.46J5J	Araip.S2Y9M	multiple-copy	Reticulonfamilyprotein
Aradu.T7CCI	Araip.SV4TB	multiple-copy	Reticulonfamilyprotein
Aradu.A42TE	Araip.V3WGE	multiple-copy	Reticulonfamilyprotein
Aradu.X4CJ9	Araip.X4V4T	multiple-copy	Reticulonfamilyprotein
Aradu.BI5JF	Araip.YQ2F8	multiple-copy	Reticulonfamilyprotein
Aradu.QA1ML	Araip.UI3CA	multiple-copy	reticulon-likeproteinB2

Aradu.SCA2Z	Araip.A1530	multiple-copy	Retrotransposonprotein,putative,Ty1-copiasubclassn=1Ta
Aradu.MYV5A	Araip.QE4BD	multiple-copy	Rhamnogalacturonatelyasefamilyprotein
Aradu.88KAK	Araip.RBD33	multiple-copy	Rhamnogalacturonatelyasefamilyprotein
Aradu.17ZI4	Araip.7AQ3E	multiple-copy	rhamnosebiosyntheticenzyme1-likeisoformX3[Glycinema
Aradu.WT71X	Araip.DDF9G	multiple-copy	rhamnosebiosyntheticenzyme1-likeisoformX3[Glycinema
Aradu.9S16L	Araip.VI6GW	multiple-copy	rhamnosebiosyntheticenzyme1-likeisoformX3[Glycinema
Aradu.W8MVJ	Araip.6T35D	multiple-copy	Rhodanese/Cellcyclecontrolphosphatasesuperfamilyprote
Aradu.9M4ZC	Araip.K0UPW	multiple-copy	Rhodanese/Cellcyclecontrolphosphatasesuperfamilyprote
Aradu.5K5P7	Araip.QYK5M	multiple-copy	Rhodanese/Cellcyclecontrolphosphatasesuperfamilyprote
Aradu.VB3DF	Araip.RBA5R	multiple-copy	Rhodanese/Cellcyclecontrolphosphatasesuperfamilyprote
Aradu.AX5BM	Araip.U63G1	multiple-copy	rhodanese/cellcyclecontrolphosphatasesuperfamilyprotei
Aradu.35U3T	Araip.XVM77	multiple-copy	rhodanese-likedomain-containingprotein4,chloroplastic-lik
Aradu.TZS3T	Araip.SVT52	multiple-copy	rhodanese-likedomain-containingprotein4A,chloroplastic-
Aradu.YW2M0	Araip.C35A2	multiple-copy	rhodanese-relatedsulfurtransferase
Aradu.TM0DS	Araip.NS24Q	multiple-copy	rhodanese-relatedsulfurtransferase
Aradu.QUJ7R	Araip.53BLN	multiple-copy	rhoGDP-dissociationinhibitor1-like[Glycinemax]
Aradu.G9UB3	Araip.8Z7ED	multiple-copy	rhoGDP-dissociationinhibitor1-like[Glycinemax]
Aradu.7P5YA	Araip.VG2TJ	multiple-copy	rhoGDP-dissociationinhibitor1-like[Glycinemax]
Aradu.P9PXC	Araip.W1ASW	multiple-copy	rhoGDP-dissociationinhibitor1-like[Glycinemax]
Aradu.W1AGE	Araip.GNF5N	multiple-copy	rhoGTPase-activatingprotein2-like[Glycinemax]
Aradu.CKV49	Araip.W6JMB	multiple-copy	rhoGTPase-activatingprotein2-like[Glycinemax]
Aradu.5QJ48	Araip.7D99U	multiple-copy	RHOMBOID-like1
Aradu.Q1NTT	Araip.X2AYN	multiple-copy	RHOMBOID-like1
Aradu.R3US5	Araip.KQ9G3	multiple-copy	RHOMBOID-likeprotein10
Aradu.HM0P2	Araip.Q3W10	multiple-copy	RHOMBOID-likeprotein10
Aradu.B4CLV	Araip.PE5QA	multiple-copy	rhomboid-likeprotein11
Aradu.K0BKL	Araip.7942Z	multiple-copy	RHOMBOID-likeprotein13
Aradu.4UY6C	Araip.IK079	multiple-copy	RHOMBOID-likeprotein3
Aradu.QX9N9	Araip.84ICS	multiple-copy	riboflavinbiosynthesisprotein,putative
Aradu.N2UA7	Araip.Y79HX	multiple-copy	ribonuclease1
Aradu.356M1	Araip.3FK8B	multiple-copy	ribonuclease3
Aradu.EH8VM	Araip.7QT5G	multiple-copy	RibonucleaseEinhibitorRraA/Dimethylmenaquinonemethy
Aradu.B92XB	Araip.R6YM0	multiple-copy	ribonucleaseH2subunitB-like[Glycinemax]
Aradu.0G5T2	Araip.F5QUZ	multiple-copy	RibonucleaseH2subunitCn=5Tax=SalmoninaeRepID=B5
Aradu.S894K	Araip.JR3QJ	multiple-copy	ribonucleasePfamilyprotein
Aradu.UQQ1M	Araip.CCT6I	multiple-copy	RibosomalL29familyprotein
Aradu.Q1RYU	Araip.Q73AM	multiple-copy	ribosomalprotein1
Aradu.6E59T	Araip.HFF60	multiple-copy	RibosomalproteinL12familyprotein
Aradu.BNK96	Araip.RDA4Z	multiple-copy	RibosomalproteinL12familyprotein
Aradu.L2APB	Araip.41RUB	multiple-copy	RibosomalproteinL14
Aradu.JQC6T	Araip.9YL2S	multiple-copy	RibosomalproteinL17familyprotein
Aradu.7G9TQ	Araip.C0H6M	multiple-copy	RibosomalproteinL18aefamily
Aradu.RQ9JK	Araip.Z4EB4	multiple-copy	RibosomalproteinL19familyprotein
Aradu.N9CVY	Araip.4BX2B	multiple-copy	RibosomalproteinL1p/L10efamily

Aradu.AX7JB	Araip.A64V4	multiple-copy	RibosomalproteinL24efamilyprotein
Aradu.L5WLB	Araip.57WAC	multiple-copy	RibosomalproteinL25/Gln-tRNAsynthetase,anti-codon-bir
Aradu.0LC5Q	Araip.6TL19	multiple-copy	RibosomalproteinL27familyprotein
Aradu.2U2Q6	Araip.MF53E	multiple-copy	ribosomalproteinL34
Aradu.M01CD	Araip.529RL	multiple-copy	RibosomalproteinL35Aefamilyprotein
Aradu.SB62Q	Araip.LZ646	multiple-copy	RibosomalproteinL36
Aradu.J1Y0V	Araip.I7WTL	multiple-copy	RibosomalproteinL3familyprotein
Aradu.2503N	Araip.VG3DT	multiple-copy	RibosomalproteinL4/L1family
Aradu.W69K1	Araip.8G9ZI	multiple-copy	RibosomalproteinL6family
Aradu.P74XB	Araip.LKU3G	multiple-copy	RibosomalproteinL6family
Aradu.1WT6A	Araip.UKM5Q	multiple-copy	RibosomalproteinL6familyprotein
Aradu.16QCN	Araip.N7Q35	multiple-copy	RibosomalproteinL7Ae/L30e/S12e/Gadd45familyprotein
Aradu.FFW2J	Araip.DL6JR	multiple-copy	ribosomalproteinL9
Aradu.G8GYX	Araip.MA9F1	multiple-copy	ribosomalproteinS15A
Aradu.BGR5Z	Araip.U9QE5	multiple-copy	ribosomalproteinS19
Aradu.763L8	Araip.6VR42	multiple-copy	RibosomalproteinS21familyprotein
Aradu.CE0RB	Araip.G4EHF	multiple-copy	RibosomalproteinS5familyprotein
Aradu.16GQU	Araip.U0GR3	multiple-copy	RibosomalproteinS5familyprotein
Aradu.SX6G4	Araip.5CP68	multiple-copy	RibosomalproteinS7efamilyprotein
Aradu.H5024	Araip.DR5NH	multiple-copy	ribosomalproteinS9
Aradu.04DD4	Araip.USK4J	multiple-copy	ribosomalproteinS9
Aradu.XHT76	Araip.37P5S	multiple-copy	RibosomalRNAlargesubunitmethyltransferaseNn=2Tax=R
Aradu.FK3YI	Araip.UE1LF	multiple-copy	ribosomalRNAsmallsubunitmethyltransferaseE
Aradu.XY3GQ	Araip.SNP97	multiple-copy	ribosomalRNAsmallsubunitmethyltransferaseG
Aradu.C8SIT	Araip.QP82L	multiple-copy	RibosomalRNAsmallsubunitmethyltransferaseNEP1n=4T
Aradu.WBK0P	Araip.AJ7AK	multiple-copy	ribosomebiogenesisproteinWDR12homolog[Glycinemax]
Aradu.0X9JV	Araip.Q4VW4	multiple-copy	ribosomebiogenesisregulatoryfamilyprotein
Aradu.04GWS	Araip.VZN05	multiple-copy	ribosomematurationproteinSBDS-like[Glycinemax]
Aradu.5IY98	Araip.Y58G9	multiple-copy	ribulosebisphosphatecarboxylase/oxygenaseactivase
Aradu.I4MAR	Araip.BQL2U	multiple-copy	ribulosebisphosphatecarboxylaselargechain
Aradu.4VZ9K	Araip.506UP	multiple-copy	RING/FYVE/PHD-typezincfingerfamilyprotein
Aradu.0HM6U	Araip.3UC9S	multiple-copy	RING/FYVE/PHDzincfingersuperfamilyprotein
Aradu.716Q8	Araip.5HV78	multiple-copy	RING/FYVE/PHDzincfingersuperfamilyprotein
Aradu.P5VH9	Araip.83FL0	multiple-copy	RING/FYVE/PHDzincfingersuperfamilyprotein
Aradu.IF5Y6	Araip.A1R8U	multiple-copy	RING/FYVE/PHDzincfingersuperfamilyprotein
Aradu.G04HZ	Araip.F6UPB	multiple-copy	RING/FYVE/PHDzincfingersuperfamilyprotein
Aradu.L3I19	Araip.L03B5	multiple-copy	RING/FYVE/PHDzincfingersuperfamilyprotein
Aradu.P535S	Araip.LW4LX	multiple-copy	RING/FYVE/PHDzincfingersuperfamilyprotein
Aradu.47JD2	Araip.MC3BU	multiple-copy	RING/FYVE/PHDzincfingersuperfamilyprotein
Aradu.8FB72	Araip.N7N6Z	multiple-copy	RING/FYVE/PHDzincfingersuperfamilyprotein
Aradu.S20WA	Araip.PCN2L	multiple-copy	RING/FYVE/PHDzincfingersuperfamilyprotein
Aradu.V94FJ	Araip.PI5NS	multiple-copy	RING/FYVE/PHDzincfingersuperfamilyprotein
Aradu.UU4C2	Araip.QG6DX	multiple-copy	RING/FYVE/PHDzincfingersuperfamilyprotein
Aradu.3P3LL	Araip.W5NDF	multiple-copy	RING/FYVE/PHDzincfingersuperfamilyprotein

Aradu.36LCY	Araip.WH0LB	multiple-copy	RING/FYVE/PHDzincfingersuperfamilyprotein
Aradu.U61F3	Araip.X69LT	multiple-copy	RING/FYVE/PHDzincfingersuperfamilyprotein
Aradu.5G8Q4	Araip.V2R6Y	multiple-copy	RING/FYVE/PHDzincfingersuperfamilyprotein
Aradu.BU5Q7	Araip.21F2H	multiple-copy	RING/U-boxsuperfamilyprotein
Aradu.R2YBN	Araip.47EMD	multiple-copy	RING/U-boxsuperfamilyprotein
Aradu.V019K	Araip.51SDF	multiple-copy	RING/U-boxsuperfamilyprotein
Aradu.92CPX	Araip.6NN2A	multiple-copy	RING/U-boxsuperfamilyprotein
Aradu.QS9PE	Araip.AS5XR	multiple-copy	RING/U-boxsuperfamilyprotein
Aradu.H40D4	Araip.CXX9S	multiple-copy	RING/U-boxsuperfamilyprotein
Aradu.CBB8Q	Araip.D3NXV	multiple-copy	RING/U-boxsuperfamilyprotein
Aradu.J79ME	Araip.FM1J9	multiple-copy	RING/U-boxsuperfamilyprotein
Aradu.P81TA	Araip.JM5TE	multiple-copy	RING/U-boxsuperfamilyprotein
Aradu.W3ELN	Araip.L3N8P	multiple-copy	RING/U-boxsuperfamilyprotein
Aradu.ES74T	Araip.LDB2J	multiple-copy	RING/U-boxsuperfamilyprotein
Aradu.8W59U	Araip.MYF07	multiple-copy	RING/U-boxsuperfamilyprotein
Aradu.P7Z3S	Araip.NEP9J	multiple-copy	RING/U-boxsuperfamilyprotein
Aradu.71EUZ	Araip.R8R5W	multiple-copy	RING/U-boxsuperfamilyprotein
Aradu.W7KRJ	Araip.TC7E2	multiple-copy	RING/U-boxsuperfamilyprotein
Aradu.ED9IS	Araip.V16E1	multiple-copy	RING/U-boxsuperfamilyprotein
Aradu.CI09B	Araip.VC2I3	multiple-copy	RING/U-boxsuperfamilyprotein
Aradu.GF1M7	Araip.VCN0P	multiple-copy	RING/U-boxsuperfamilyprotein
Aradu.YLV00	Araip.X7NAU	multiple-copy	RINGfingerandCHYzincfingerprotein
Aradu.78IK4	Araip.3266N	multiple-copy	RINGfingerprotein126-A-like[Glycinemax]
Aradu.5T4PS	Araip.V3A9S	multiple-copy	RINGfingerprotein126-A-like[Glycinemax]
Aradu.47VKV	Araip.BX0PW	multiple-copy	RINGfingerprotein38-like[Glycinemax]
Aradu.XM1WL	Araip.Y2BA4	multiple-copy	RINGfingerprotein38-like[Glycinemax]
Aradu.4L5WB	Araip.33YRY	multiple-copy	RINGfingerprotein38-likeisoformX2[Glycinemax]
Aradu.3C7HF	Araip.45CYE	multiple-copy	RINGfingerprotein38-likeisoformX2[Glycinemax]
Aradu.VL23N	Araip.D5A51	multiple-copy	RINGfingerprotein38-like[Glycinemax]
Aradu.T3K2L	Araip.QHS45	multiple-copy	RINGfingerprotein38-likeisoformX2[Glycinemax]
Aradu.WSK5V	Araip.IN8QT	multiple-copy	RINGfingerprotein44-like[Glycinemax]
Aradu.88E60	Araip.ZF8FB	multiple-copy	RINGfingerprotein44-like[Glycinemax]
Aradu.HL95H	Araip.R4IFK	multiple-copy	RINGfingerprotein5isoform2[Glycinemax]
Aradu.27VHF	Araip.UF3L0	multiple-copy	RINGfingerprotein5-like[Glycinemax]
Aradu.ZJM47	Araip.DDX3A	multiple-copy	RING-H2fingerprotein[Glycinemax]
Aradu.IFI3Q	Araip.00K86	multiple-copy	RING-H2fingerprotein2B
Aradu.771JD	Araip.05I70	multiple-copy	RING-H2fingerprotein2B
Aradu.4I70C	Araip.0B2S6	multiple-copy	RING-H2fingerprotein2B
Aradu.DHI4B	Araip.15N6Y	multiple-copy	RING-H2fingerprotein2B
Aradu.C3IHK	Araip.4S7ZN	multiple-copy	RING-H2fingerprotein2B
Aradu.657DW	Araip.5S35P	multiple-copy	RING-H2fingerprotein2B
Aradu.I5BSN	Araip.5XR69	multiple-copy	RING-H2fingerprotein2B
Aradu.EH857	Araip.6TB90	multiple-copy	RING-H2fingerprotein2B
Aradu.9AW6M	Araip.ANL75	multiple-copy	RING-H2fingerprotein2B

Aradu.UJ8BX	Araip.EK901	multiple-copy	RING-H2fingerprotein2B
Aradu.5F8QQ	Araip.J1VUP	multiple-copy	RING-H2fingerprotein2B
Aradu.L0YDF	Araip.QLX2I	multiple-copy	RING-H2fingerprotein2B
Aradu.N69D4	Araip.R4HD6	multiple-copy	RING-H2fingerprotein2B
Aradu.UZ754	Araip.R86UC	multiple-copy	RING-H2fingerprotein2B
Aradu.4D16J	Araip.SS0GG	multiple-copy	RING-H2fingerprotein2B
Aradu.N3CWH	Araip.YQI1Y	multiple-copy	RING-H2fingerprotein2B
Aradu.24SCU	Araip.U33C2	multiple-copy	RING-H2fingerproteinATL18-like[Glycinemax]
Aradu.K119F	Araip.4WS44	multiple-copy	RING-H2zincfingerprotein
Aradu.Z2H09	Araip.B2B7T	multiple-copy	RING-H2zincfingerprotein
Aradu.VXS15	Araip.Q9692	multiple-copy	RING/U-boxsuperfamilyprotein
Aradu.0D9UK	Araip.3C7F6	multiple-copy	RINGfingerprotein38-likeisoformX2[Glycinemax]
Aradu.LX0F3	Araip.1YM3E	multiple-copy	RINGzincfingerprotein,putative
Aradu.39CB1	Araip.50B92	multiple-copy	RINGzincfingerprotein,putative
Aradu.W3DTP	Araip.N7IGQ	multiple-copy	RNAbinding(RRM/RBD/RNPmotifs)familyprotein
Aradu.92KLI	Araip.12RXW	multiple-copy	RNA-binding(RRM/RBD/RNPmotifs)familyprotein
Aradu.YA89L	Araip.BP2SC	multiple-copy	RNA-binding(RRM/RBD/RNPmotifs)familyprotein
Aradu.EM2BW	Araip.IV0J9	multiple-copy	RNA-binding(RRM/RBD/RNPmotifs)familyprotein
Aradu.AXH5G	Araip.JB1PE	multiple-copy	RNA-bindingprotein1-like[Glycinemax]
Aradu.RDM6K	Araip.MS1SV	multiple-copy	RNA-binding(RRM/RBD/RNPmotifs)familyprotein
Aradu.K6YI3	Araip.WW0EW	multiple-copy	RNA-binding(RRM/RBD/RNPmotifs)familyprotein
Aradu.CP8NA	Araip.ZSP2K	multiple-copy	RNA-binding(RRM/RBD/RNPmotifs)familyprotein
Aradu.W0GZN	Araip.QB1DK	multiple-copy	RNA-bindingCRS1/YhbY(CRM)domain-containingprotein
Aradu.Y5WFV	Araip.ZUI4X	multiple-copy	RNA-bindingCRS1/YhbY(CRM)domain-containingprotein
Aradu.88Z5A	Araip.1J28K	multiple-copy	RNA-bindingdomainCCCH-typezincfingerprotein
Aradu.Z67WQ	Araip.2K8HT	multiple-copy	RNA-bindingdomainCCCH-typezincfingerprotein
Aradu.DI7FZ	Araip.9D0ML	multiple-copy	RNA-bindingdomainCCCH-typezincfingerprotein
Aradu.R8LP2	Araip.M0GMD	multiple-copy	RNA-bindingdomainCCCH-typezincfingerprotein
Aradu.X0SMT	Araip.K3RKP	multiple-copy	RNA-bindingdomain-containingproteinn=1Tax=Acantham
Aradu.3L4RS	Araip.PAD1A	multiple-copy	RNA-bindingfamilyproteinn=1Tax=PopulustrichocarpaRe
Aradu.13Y5Y	Araip.2BU9K	multiple-copy	RNA-bindingKHdomain-containingprotein
Aradu.MS2JZ	Araip.601K9	multiple-copy	RNA-bindingKHdomain-containingprotein
Aradu.LZR7H	Araip.63YUF	multiple-copy	RNA-bindingKHdomain-containingprotein
Aradu.1FQ73	Araip.HL18M	multiple-copy	RNA-bindingLUC7-likeprotein
Aradu.4H70X	Araip.V7QPL	multiple-copy	RNAbindingmethyltransferaseFtsJliken=1Tax=Halanaero
Aradu.EG6FJ	Araip.8G8VL	multiple-copy	RNA-bindingprotein,putative
Aradu.06C7F	Araip.KPA97	multiple-copy	RNA-bindingprotein1
Aradu.ZM5JL	Araip.IH5NI	multiple-copy	RNA-bindingprotein1-like[Glycinemax]
Aradu.WQ0V2	Araip.V3UEW	multiple-copy	RNA-bindingprotein1-like[Glycinemax]
Aradu.T8SW7	Araip.1ZW9V	multiple-copy	RNA-bindingprotein24-A[Glycinemax]
Aradu.AH9TD	Araip.05VR1	multiple-copy	RNA-bindingprotein24-A-like[Glycinemax]
Aradu.VZ2PZ	Araip.3B7EF	multiple-copy	RNA-binding(RRM/RBD/RNPmotifs)familyprotein
Aradu.MM9KG	Araip.GJS6W	multiple-copy	RNA-bindingprotein24-A-like[Glycinemax]
Aradu.J364A	Araip.GZD9C	multiple-copy	RNA-bindingprotein24-A-like[Glycinemax]

Aradu.KLJ74	Araip.5J7CQ	multiple-copy	RNA-binding(RRM/RBD/RNPmotifs)familyprotein
Aradu.T8TQR	Araip.SN4N7	multiple-copy	RNA-bindingprotein24-like[Glycinemax]
Aradu.EAZ6K	Araip.U75BK	multiple-copy	RNA-bindingprotein24-like[Glycinemax]
Aradu.S1HRU	Araip.FD42N	multiple-copy	RNA-bindingprotein24-likeisoformX2[Glycinemax]
Aradu.LQY7B	Araip.6Q97S	multiple-copy	RNA-bindingprotein25-likeisoformX2[Glycinemax]
Aradu.67PUF	Araip.PVQ2W	multiple-copy	RNA-bindingprotein28-likeisoformX2[Glycinemax]
Aradu.60GK5	Araip.W10FE	multiple-copy	RNA-bindingprotein38-like[Glycinemax]
Aradu.N8S8A	Araip.24FNG	multiple-copy	RNA-bindingprotein38-likeisoformX2[Glycinemax]
Aradu.B9T25	Araip.9E5ZT	multiple-copy	RNA-bindingprotein38-likeisoformX2[Glycinemax]
Aradu.53Y6H	Araip.60SDM	multiple-copy	RNA-bindingprotein38-likeisoformX3[Glycinemax]
Aradu.Z3EIC	Araip.D1CR7	multiple-copy	RNA-bindingprotein38-likeisoformX3[Glycinemax]
Aradu.2448N	Araip.71Z7T	multiple-copy	RNA-bindingprotein1-like[Glycinemax]
Aradu.ZS4VI	Araip.L7KTT	multiple-copy	RNA-bindingprotein39-like[Glycinemax]
Aradu.TTW29	Araip.P7WV8	multiple-copy	RNA-bindingprotein39-like[Glycinemax]
Aradu.AN6JJ	Araip.S7GYW	multiple-copy	RNA-bindingprotein39-like[Glycinemax]
Aradu.8K2VK	Araip.J8DCC	multiple-copy	RNA-bindingprotein39-likeisoformX2[Glycinemax]
Aradu.I4E8B	Araip.5V8J3	multiple-copy	RNA-bindingprotein42-like[Glycinemax]
Aradu.BJ55N	Araip.S52ED	multiple-copy	RNA-bindingprotein42-like[Glycinemax]
Aradu.576P8	Araip.UZ11J	multiple-copy	RNA-bindingprotein42-like[Glycinemax]
Aradu.8X203	Araip.Q1XVU	multiple-copy	RNA-bindingprotein48isoformX3[Glycinemax]
Aradu.ZS9DU	Araip.87K0L	multiple-copy	RNA-bindingprotein8A-like[Glycinemax]
Aradu.4D9C8	Araip.PS258	multiple-copy	RNAbindingproteinn=1Tax=ZeamaysRepID=B6SGY5_M
Aradu.GI0SK	Araip.58R6B	multiple-copy	RNA-bindingproteinn=3Tax=CucumisRepID=E5GB57_C
Aradu.509WJ	Araip.L2L5B	multiple-copy	RNA-bindingproteinNOB1
Aradu.0NE9W	Araip.AL2VM	multiple-copy	RNA-bindingregionRNP-1(RNArecognitionmotif)
Aradu.SJ6MW	Araip.1F7P8	multiple-copy	RNA-bindingregionRNP-1protein
Aradu.EA9CU	Araip.IH5JS	multiple-copy	RNA-bindingS4domain-containingprotein
Aradu.UZS9C	Araip.TZC8T	multiple-copy	RNAhelicaseSDE3-likeisoformX3[Glycinemax]
Aradu.G4M3I	Araip.JLS7G	multiple-copy	RNAligase/cyclicnucleotidephosphodiesterasefamilyprote
Aradu.L273D	Araip.CC7UH	multiple-copy	RNAmethyltransferase-likeproteinn=1Tax=Medicagotrunc
Aradu.3F83L	Araip.3F57L	multiple-copy	RNAmethyltransferasen=1Tax=Paenibacillussp.A9RepID
Aradu.NUY4D	Araip.QF21H	multiple-copy	RNAmethyltransferasesmalldomainprotein
Aradu.7P55W	Araip.PYU91	multiple-copy	RNAmethyltransferasen=4Tax=StreptomycesRepID=M3
Aradu.3IY7B	Araip.L1GQW	multiple-copy	RNApolymeraseIIC-terminaldomainphosphatase-like1-lik
Aradu.CSH6U	Araip.3Y5DK	multiple-copy	RNApolymeraseIItranscriptionalcoactivator
Aradu.PI1VW	Araip.JF7QM	multiple-copy	RNApolymeraseIItranscriptionmediators
Aradu.L0BQ6	Araip.K7ZBG	multiple-copy	RNApolymeraseIspecifictranscriptioninitiationfactorRRN3
Aradu.YF1F6	Araip.HV00F	multiple-copy	RNApolymerasesigmafactor
Aradu.W20TC	Araip.KLX3M	multiple-copy	RNApolymerasesigmafactor
Aradu.S0S2R	Araip.Y28R2	multiple-copy	RNApolymerasesigmafactor
Aradu.V4S8W	Araip.S85MS	multiple-copy	ROOTHAIRDEFECTIVE3homolog1-like[Glycinemax]
Aradu.QV7H5	Araip.HP461	multiple-copy	ROOTPRIMORDIUMDEFECTIVE1-likeisoformX4[Glycin
Aradu.AM9MC	Araip.G85GD	multiple-copy	ROPguaninenucleotideexchangefactor5
Aradu.S4XSK	Araip.HP1ES	multiple-copy	ROPguaninenucleotideexchangefactor5

Aradu.QVD5S	Araip.ID15H	multiple-copy	ROPguaninenucleotideexchangefactor5
Aradu.P9JVV	Araip.T9719	multiple-copy	ROPguaninenucleotideexchangefactor5
Aradu.73L3W	Araip.TSM4K	multiple-copy	ROPguaninenucleotideexchangefactor5
Aradu.L210Z	Araip.UHC90	multiple-copy	ROPguaninenucleotideexchangefactor5
Aradu.C8KRG	Araip.PN8UI	multiple-copy	ROP-interactiveCRIBmotif-containingprotein5
Aradu.E8VVL	Araip.XF60E	multiple-copy	ROP-interactiveCRIBmotif-containingprotein6
Aradu.7K02I	Araip.Q2FNA	multiple-copy	RPM1-interactingprotein4-like[Glycinemax]
Aradu.RM1RY	Araip.QUZ7L	multiple-copy	rRNA-processingproteinPINdomainprotein
Aradu.AKB12	Araip.1E7ZD	multiple-copy	Rubberelongationfactorprotein(REF)
Aradu.24YRC	Araip.FYI8V	multiple-copy	Rubberelongationfactorprotein(REF)
Aradu.P2MZP	Araip.MI54T	multiple-copy	ruBisCO-associatedprotein-like[Glycinemax]
Aradu.EW82S	Araip.NED9I	multiple-copy	ruBisCO-associatedprotein-like[Glycinemax]
Aradu.0768L	Araip.VC558	multiple-copy	RWP-RKdomain-containingprotein
Aradu.U79FN	Araip.8TI0S	multiple-copy	S1/P1nucleasefamilyprotein
Aradu.25L3E	Araip.154H4	multiple-copy	S1RNAbindingdomainproteinn=4Tax=rootRepID=B0MWI
Aradu.AR9AM	Araip.FV4T0	multiple-copy	SAC51-like[Glycinemax]
Aradu.EZ30H	Araip.XZ3ZL	multiple-copy	SAC51-like[Glycinemax]
Aradu.E91AT	Araip.7H21U	multiple-copy	S-adenosyl-L-methionine-dependentmethyltransferasess
Aradu.K9Z81	Araip.9I9CS	multiple-copy	S-adenosyl-L-methionine-dependentmethyltransferasess
Aradu.IVC5J	Araip.B9DG2	multiple-copy	S-adenosyl-L-methionine-dependentmethyltransferasess
Aradu.B9ZHB	Araip.E4D1J	multiple-copy	S-adenosyl-L-methionine-dependentmethyltransferasess
Aradu.TKG8T	Araip.EA9WI	multiple-copy	S-adenosyl-L-methionine-dependentmethyltransferasess
Aradu.H50Y7	Araip.F0YLK	multiple-copy	S-adenosyl-L-methionine-dependentmethyltransferasess
Aradu.YTF7E	Araip.FZQ92	multiple-copy	S-adenosyl-L-methionine-dependentmethyltransferasess
Aradu.0B0D2	Araip.J9R36	multiple-copy	S-adenosyl-L-methionine-dependentmethyltransferasess
Aradu.WW0P3	Araip.M4X9J	multiple-copy	S-adenosyl-L-methionine-dependentmethyltransferasess
Aradu.I1T9D	Araip.V2GHQ	multiple-copy	S-adenosyl-L-methionine-dependentmethyltransferasess
Aradu.A5HRL	Araip.6VN5N	multiple-copy	S-adenosylmethioninedecarboxylase
Aradu.UQA28	Araip.3H7G0	multiple-copy	S-adenosylmethionine-dependentmethyltransferase
Aradu.RI1GB	Araip.HDU9M	multiple-copy	S-adenosylmethionine-dependentmethyltransferase
Aradu.X9447	Araip.N5EXR	multiple-copy	S-adenosylmethionine-dependentmethyltransferase
Aradu.YQ9M6	Araip.77H8K	multiple-copy	S-adenosylmethionine-dependentmethyltransferase,puta
Aradu.D3TM0	Araip.U33VJ	multiple-copy	S-adenosylmethionine-dependentmethyltransferase,puta
Aradu.7Q9WK	Araip.4V3JQ	multiple-copy	SAUR-likeauxin-responsiveproteinfamily
Aradu.F38CM	Araip.A8ULT	multiple-copy	SAUR-likeauxin-responsiveproteinfamily
Aradu.7J6XP	Araip.EB6ED	multiple-copy	SAUR-likeauxin-responsiveproteinfamily
Aradu.DKR1D	Araip.ECU5E	multiple-copy	SAUR-likeauxin-responsiveproteinfamily
Aradu.A0FDR	Araip.G06Y7	multiple-copy	SAUR-likeauxin-responsiveproteinfamily
Aradu.EAI63	Araip.8KF2K	multiple-copy	SAWADEEHOMEODOMAINHOMOLOG2-likeisoformX1[
Aradu.VJ2JF	Araip.4S2KN	multiple-copy	SAWADEEHOMEODOMAINHOMOLOG2-likeisoformX2[
Aradu.R95HC	Araip.YAQ0L	multiple-copy	SCARECROW-like[Glycinemax]
Aradu.10YFM	Araip.N5Q83	multiple-copy	scarecrow-likeprotein14-like[Glycinemax]
Aradu.Y5WGV	Araip.XL0W8	multiple-copy	scarecrow-likeprotein1-like[Glycinemax]
Aradu.FG20F	Araip.KB0T7	multiple-copy	scarecrow-likeprotein32-like[Glycinemax]

Aradu.3B8NW	Araip.TD6FV	multiple-copy	scarecrow-likeprotein3-like[Glycinemax]
Aradu.V6MI1	Araip.Y26KW	multiple-copy	scarecrow-likeprotein3-like[Glycinemax]
Aradu.88125	Araip.2U0KZ	multiple-copy	Scarecrow-liketranscriptionfactor11,putativeisoform1n=1T
Aradu.1A2XY	Araip.6I3DS	multiple-copy	scarecrow-liketranscriptionfactorPAT1-like[Glycinemax]
Aradu.2X0TU	Araip.C9B5T	multiple-copy	scarecrow-liketranscriptionfactorPAT1-like[Glycinemax]
Aradu.G5P3T	Araip.CZ0IQ	multiple-copy	scarecrow-liketranscriptionfactorPAT1-like[Glycinemax]
Aradu.RYS1S	Araip.DNQ5K	multiple-copy	scarecrow-liketranscriptionfactorPAT1-like[Glycinemax]
Aradu.YMD6U	Araip.E00UL	multiple-copy	scarecrow-liketranscriptionfactorPAT1-like[Glycinemax]
Aradu.1Y370	Araip.ED1H8	multiple-copy	scarecrow-liketranscriptionfactorPAT1-like[Glycinemax]
Aradu.PHB5M	Araip.G0MKK	multiple-copy	scarecrow-liketranscriptionfactorPAT1-like[Glycinemax]
Aradu.B11ZS	Araip.W23GC	multiple-copy	scarecrow-liketranscriptionfactorPAT1-like[Glycinemax]
Aradu.FB7VW	Araip.WB4KA	multiple-copy	scarecrow-liketranscriptionfactorPAT1-like[Glycinemax]
Aradu.R75XI	Araip.RY956	multiple-copy	SCARhomolog2
Aradu.54J14	Araip.0NG6C	multiple-copy	Sec14p-likephosphatidylinositoltransferfamilyprotein
Aradu.3B9N0	Araip.1I49F	multiple-copy	Sec14p-likephosphatidylinositoltransferfamilyprotein
Aradu.UXS7E	Araip.2KR54	multiple-copy	Sec14p-likephosphatidylinositoltransferfamilyprotein
Aradu.1864T	Araip.33CRB	multiple-copy	Sec14p-likephosphatidylinositoltransferfamilyprotein
Aradu.ZW5X6	Araip.83CVJ	multiple-copy	Sec14p-likephosphatidylinositoltransferfamilyprotein
Aradu.Y8YHS	Araip.9ML2X	multiple-copy	Sec14p-likephosphatidylinositoltransferfamilyprotein
Aradu.MC6VZ	Araip.A87CL	multiple-copy	Sec14p-likephosphatidylinositoltransferfamilyprotein
Aradu.B6TUW	Araip.HU0D3	multiple-copy	Sec14p-likephosphatidylinositoltransferfamilyprotein
Aradu.IC00B	Araip.K22F0	multiple-copy	Sec14p-likephosphatidylinositoltransferfamilyprotein
Aradu.X2LCY	Araip.KC5KI	multiple-copy	Sec14p-likephosphatidylinositoltransferfamilyprotein
Aradu.X6P8N	Araip.M21JP	multiple-copy	Sec14p-likephosphatidylinositoltransferfamilyprotein
Aradu.CD4CM	Araip.N65BG	multiple-copy	Sec14p-likephosphatidylinositoltransferfamilyprotein
Aradu.VA9CU	Araip.N65ZU	multiple-copy	Sec14p-likephosphatidylinositoltransferfamilyprotein
Aradu.3N6NA	Araip.Q655H	multiple-copy	Sec14p-likephosphatidylinositoltransferfamilyprotein
Aradu.AXB4D	Araip.RRZ3Z	multiple-copy	Sec14p-likephosphatidylinositoltransferfamilyprotein
Aradu.I0353	Araip.UV12P	multiple-copy	Sec14p-likephosphatidylinositoltransferfamilyprotein
Aradu.Y37CK	Araip.C817Q	multiple-copy	SEC1familytransportproteinSLY1-like[Glycinemax]
Aradu.DWZ8T	Araip.33PYF	multiple-copy	Sec23/Sec24proteintransportfamilyprotein
Aradu.ZX42L	Araip.GC2RS	multiple-copy	Sec23/Sec24proteintransportfamilyprotein
Aradu.T1JCL	Araip.LQ3KM	multiple-copy	Sec23/Sec24proteintransportfamilyprotein
Aradu.87SDL	Araip.VU8GK	multiple-copy	Sec23/Sec24proteintransportfamilyprotein
Aradu.CJP5L	Araip.1842Y	multiple-copy	sec23/sec24transportfamilyprotein
Aradu.7X826	Araip.5FG6Z	multiple-copy	Sec-independentproteintranslocaseproteinTatAn=1Tax=S
Aradu.P6XBQ	Araip.I2DE4	multiple-copy	sec-independentproteintranslocaseTatB-likeprotein,putati
Aradu.JBU5E	Araip.4P1DQ	multiple-copy	Sec-independentproteintranslocaseTatC
Aradu.CA5TC	Araip.X2AP6	multiple-copy	Secretorycarriermembraneprotein(SCAMP)familyprotein
Aradu.FM0YX	Araip.GV48H	multiple-copy	seedlinoleate9S-lipoxygenase
Aradu.TJL9X	Araip.K56RN	multiple-copy	seedlinoleate9S-lipoxygenase
Aradu.951UC	Araip.Q8LFT	multiple-copy	seedlinoleate9S-lipoxygenase
Aradu.G99LQ	Araip.W6TLM	multiple-copy	seedlinoleate9S-lipoxygenase
Aradu.2K04Y	Araip.7HP1R	multiple-copy	seedmaturationprotein

Aradu.3G22S	Araip.JTL3L	multiple-copy	Seedmaturationprotein
Aradu.FPC2C	Araip.U0WFW	multiple-copy	Seedmaturationprotein
Aradu.CA6YX	Araip.RUD0N	multiple-copy	Senescence/dehydration-associatedprotein-related
Aradu.B80V6	Araip.V6UVY	multiple-copy	Senescence/dehydration-associatedprotein-related
Aradu.GR4IB	Araip.3S1SV	multiple-copy	senescence-associatedprotein
Aradu.SL05Q	Araip.ZWB49	multiple-copy	septum-promotingGTP-bindingprotein1-like[Glycinemax]
Aradu.SYI1K	Araip.98Q8H	multiple-copy	sequence-specificDNAbindingtranscriptionfactors
Aradu.UAJ8F	Araip.BEJ3Y	multiple-copy	sequence-specificDNAbindingtranscriptionfactors
Aradu.VZ497	Araip.L4V14	multiple-copy	sequence-specificDNAbindingtranscriptionfactors
Aradu.LCM6E	Araip.ZPS65	multiple-copy	ser/thr-richproteinT10inDGCRregion-likeprotein
Aradu.N5B8E	Araip.ZMH2R	multiple-copy	serine/argininerepetitivematrixprotein2-like[Glycinemax]
Aradu.IH0N2	Araip.82GLE	multiple-copy	serine/argininerepetitivematrixprotein2-likeisoformX2[Gly
Aradu.C6I2X	Araip.RWR8N	multiple-copy	serine/arginine-richsplicingfactor33isoform1[Glycinemax]
Aradu.GFE68	Araip.Y8HCT	multiple-copy	Serine/Threonine-kinaseATR-likeprotein
Aradu.Z1Y2A	Araip.2HX98	multiple-copy	Serine/Threoninekinasefamilyprotein
Aradu.76BI5	Araip.Y683M	multiple-copy	Serine/Threonine-kinasehaspin
Aradu.8Z0S4	Araip.1N36Z	multiple-copy	serine/threoninekinases
Aradu.I7IYL	Araip.XS400	multiple-copy	kinasefamily
Aradu.YC6ZD	Araip.H1BC4	multiple-copy	serine/threonineproteinkinase1
Aradu.HD19V	Araip.IHD8T	multiple-copy	serine/threonine-proteinkinaseATG1-likeisoformX4[Glyci
Aradu.A9F8B	Araip.2I121	multiple-copy	serine/threonineproteinphosphatase2A
Aradu.KT4MN	Araip.64GAL	multiple-copy	serine/threonineproteinphosphatase2A
Aradu.W2A66	Araip.AW13W	multiple-copy	serine/threonineproteinphosphatase2A
Aradu.AU35U	Araip.FB5WA	multiple-copy	serine/threonineproteinphosphatase2A
Aradu.T6KM7	Araip.K77RU	multiple-copy	serine/threonineproteinphosphatase2A
Aradu.PS85R	Araip.K9ABC	multiple-copy	serine/threonineproteinphosphatase2A
Aradu.RAZ0G	Araip.SI5FX	multiple-copy	Serine/threonine-proteinphosphatase2A55kDaregulatory
Aradu.CTE87	Araip.VES4J	multiple-copy	Serine/threonine-proteinphosphatase2A55kDaregulatory
Aradu.4RN5Y	Araip.C9WGS	multiple-copy	serine/threonineproteinphosphatase2AregulatorysubunitB
Aradu.131HS	Araip.22U9L	multiple-copy	serine/threonine-proteinphosphatase4regulatorysubunit3-
Aradu.092KC	Araip.09VK1	multiple-copy	serine/threonine-proteinphosphatase7longformhomolog[G
Aradu.H1BIK	Araip.0C096	multiple-copy	serine/threonine-proteinphosphatase7longformhomolog[G
Aradu.HL03T	Araip.0I39J	multiple-copy	serine/threonine-proteinphosphatase7longformhomolog[G
Aradu.64Z0M	Araip.0WR1B	multiple-copy	serine/threonine-proteinphosphatase7longformhomolog[G
Aradu.B9U3D	Araip.2Q11V	multiple-copy	serine/threonine-proteinphosphatase7longformhomolog[G
Aradu.VM38K	Araip.308FD	multiple-copy	serine/threonine-proteinphosphatase7longformhomolog[G
Aradu.PT2CX	Araip.3PM87	multiple-copy	serine/threonine-proteinphosphatase7longformhomolog[G
Aradu.K85GV	Araip.787IL	multiple-copy	serine/threonine-proteinphosphatase7longformhomolog[G
Aradu.ZDZ6K	Araip.97H71	multiple-copy	serine/threonine-proteinphosphatase7longformhomolog[G
Aradu.U9G17	Araip.AFI6W	multiple-copy	serine/threonine-proteinphosphatase7longformhomolog[G
Aradu.CZW32	Araip.BET6I	multiple-copy	serine/threonine-proteinphosphatase7longformhomolog[G
Aradu.D6N4B	Araip.BP5EC	multiple-copy	serine/threonine-proteinphosphatase7longformhomolog[G
Aradu.FU7AU	Araip.C17N5	multiple-copy	serine/threonine-proteinphosphatase7longformhomolog[G
Aradu.L6R4W	Araip.DU9DL	multiple-copy	serine/threonine-proteinphosphatase7longformhomolog[G

Aradu.SXB8E	Araip.GTM84	multiple-copy	serine/threonine-proteinphosphatase7longformhomolog[G
Aradu.QIJ36	Araip.H6D5I	multiple-copy	serine/threonine-proteinphosphatase7longformhomolog[G
Aradu.L7FSS	Araip.I6KEH	multiple-copy	serine/threonine-proteinphosphatase7longformhomolog[G
Aradu.RA95M	Araip.N0K65	multiple-copy	serine/threonine-proteinphosphatase7longformhomolog[G
Aradu.W6T3N	Araip.RB35J	multiple-copy	serine/threonine-proteinphosphatase7longformhomolog[G
Aradu.DGG35	Araip.UV61I	multiple-copy	serine/threonine-proteinphosphatase7longformhomolog[G
Aradu.JL9ML	Araip.V2L0E	multiple-copy	serine/threonine-proteinphosphatase7longformhomolog[G
Aradu.RRT07	Araip.V4DIY	multiple-copy	serine/threonine-proteinphosphatase7longformhomolog[G
Aradu.D45R1	Araip.Y0TCW	multiple-copy	serine/threonine-proteinphosphatase7longformhomolog[G
Aradu.N1ASM	Araip.PE2BT	multiple-copy	serine/threonine-proteinphosphatase7longformhomologis
Aradu.AT3ZW	Araip.3FA1A	multiple-copy	Serine/threonineproteinphosphatasefamilyprotein
Aradu.M5RIF	Araip.H7U4C	multiple-copy	Serine/threonineproteinphosphatasefamilyprotein
Aradu.HP9LA	Araip.Z6T03	multiple-copy	Serine/threonineproteinphosphatasefamilyprotein
Aradu.51DJW	Araip.JN8SQ	multiple-copy	serineacetyltransferase3
Aradu.K0SK7	Araip.BR9B7	multiple-copy	serinecarboxypeptidase-like18
Aradu.P9IKS	Araip.Z1VW2	multiple-copy	serinecarboxypeptidase-like19
Aradu.YR8RM	Araip.YW5U3	multiple-copy	serinecarboxypeptidase-like2
Aradu.F7D9L	Araip.QZ6Y2	multiple-copy	serinecarboxypeptidase-like20
Aradu.4XE1V	Araip.V86YG	multiple-copy	serinecarboxypeptidase-like20
Aradu.QDB5N	Araip.E1EX9	multiple-copy	serinecarboxypeptidase-like25
Aradu.R1SGR	Araip.233C9	multiple-copy	serinecarboxypeptidase-like27
Aradu.44KEY	Araip.KJ84C	multiple-copy	serinecarboxypeptidase-like29
Aradu.RY6HH	Araip.S0JW5	multiple-copy	serinecarboxypeptidase-like31
Aradu.8A22P	Araip.R511F	multiple-copy	serinecarboxypeptidase-like33
Aradu.VZ8XR	Araip.T1BG2	multiple-copy	serinecarboxypeptidase-like33
Aradu.XE6VM	Araip.98KTC	multiple-copy	serinecarboxypeptidase-like40
Aradu.D2UEN	Araip.Z7SA4	multiple-copy	serinecarboxypeptidase-like40
Aradu.SDL7M	Araip.Z7025	multiple-copy	serinecarboxypeptidase-like42
Aradu.10HN0	Araip.62ZD7	multiple-copy	serinecarboxypeptidase-like45
Aradu.AHG3H	Araip.B4FBZ	multiple-copy	serinecarboxypeptidase-like45
Aradu.1N8DM	Araip.TWL2H	multiple-copy	SERINECARBOXYPEPTIDASE-LIKE49
Aradu.MUM0J	Araip.JTL29	multiple-copy	serinehydroxymethyltransferase2
Aradu.BAW60	Araip.UF6J5	multiple-copy	serinehydroxymethyltransferase4
Aradu.MRR38	Araip.XPK3R	multiple-copy	serinehydroxymethyltransferase6
Aradu.JZ8MY	Araip.N74EU	multiple-copy	serinehydroxymethyltransferase7
Aradu.G9RLQ	Araip.Q3U94	multiple-copy	serine-richprotein-related
Aradu.SKC35	Araip.3LL8X	multiple-copy	Serine-typeendopeptidaseisoform2n=2Tax=Galdieriasulp
Aradu.8WJ1L	Araip.KS112	multiple-copy	Serine-typeendopeptidaseisoform2n=2Tax=Galdieriasulp
Aradu.XBM41	Araip.64B54	multiple-copy	Serine-typeendopeptidasen=2Tax=CucumisRepID=E5G(
Aradu.B8HUR	Araip.RB3EK	multiple-copy	Serine-typeendopeptidasen=2Tax=CucumisRepID=E5G(
Aradu.CP7C1	Araip.L90LE	multiple-copy	SETdomain-containingprotein
Aradu.L6FF7	Araip.MFD08	multiple-copy	SETdomain-containingprotein
Aradu.88CDZ	Araip.RNC6M	multiple-copy	SETdomaingroup40
Aradu.IR41I	Araip.LE6XA	multiple-copy	SGT1-2[Glycinemax]

Aradu.KZ4HC	Araip.VW2EE	multiple-copy	shikimatekinase1
Aradu.SV33Z	Araip.6T3P4	multiple-copy	shikimatekinaselike2
Aradu.HJ2Y0	Araip.EF3DD	multiple-copy	SHIRELATEDSEQUENCE5-like[Glycinemax]
Aradu.K955R	Araip.IX4R9	multiple-copy	short-chaindehydrogenasereductase2a-like[Glycinemax]
Aradu.V5NSB	Araip.N7X0P	multiple-copy	short-chaindehydrogenasereductase2a-like[Glycinemax]
Aradu.P9ETF	Araip.AZ41E	multiple-copy	short-chaindehydrogenase-reductaseB
Aradu.15QSN	Araip.E3UJF	multiple-copy	short-chaindehydrogenase-reductaseB
Aradu.IZX1C	Araip.F8GND	multiple-copy	short-chaindehydrogenase-reductaseB
Aradu.64BNM	Araip.H76K9	multiple-copy	short-chaindehydrogenase-reductaseB
Aradu.C37MP	Araip.TL0ID	multiple-copy	short-chaindehydrogenase-reductaseB
Aradu.P54FB	Araip.UPW6L	multiple-copy	short-chaindehydrogenase-reductaseB
Aradu.X33LT	Araip.Y0RK1	multiple-copy	short-chaindehydrogenasereductase3b-like[Glycinemax]
Aradu.EQ8WP	Araip.53NP1	multiple-copy	shugoshin-1-likeisoformX1[Glycinemax]
Aradu.586VX	Araip.WD7HC	multiple-copy	sieveelementocclusionprotein
Aradu.49WR7	Araip.50H5N	multiple-copy	sigmafactorsigbregulationrsbq-likeprotein
Aradu.TS004	Araip.77AM3	multiple-copy	sigmafactorsigbregulationrsbq-likeprotein
Aradu.9TY2N	Araip.G2WXB	multiple-copy	sigmafactorsigbregulationrsbq-likeprotein
Aradu.T698T	Araip.X25NR	multiple-copy	signalpeptidasecomplexcatalyticsubunitSEC11C-likeisofo
Aradu.49VWN	Araip.CCV5U	multiple-copy	Signalpeptidasesubunit
Aradu.IR00B	Araip.U4GR2	multiple-copy	SIGNALPEPTIDEPEPTIDASE-LIKE1
Aradu.Q3XX4	Araip.AH9LW	multiple-copy	SIGNALPEPTIDEPEPTIDASE-LIKE2
Aradu.K2B5B	Araip.VBB27	multiple-copy	SIGNALPEPTIDEPEPTIDASE-LIKE2
Aradu.4NJ9R	Araip.WT5FG	multiple-copy	signalrecognitionparticle19kDaprotein
Aradu.LCE5M	Araip.BT890	multiple-copy	signalrecognitionparticle9kDaprotein
Aradu.G99GB	Araip.W5GXX	multiple-copy	signalrecognitionparticlereceptoralphasubunitfamilyprotei
Aradu.S9NLD	Araip.L0EG5	multiple-copy	Signaltransductionhistidinekinase
Aradu.UYZ93	Araip.R77E0	multiple-copy	Signaltransductionhistidinekinase
Aradu.R3YL8	Araip.Z4BH6	multiple-copy	Signaltransductionhistidinekinase
Aradu.TTI87	Araip.07W64	multiple-copy	Signaltransductionhistidinekinase,hybrid-type,ethylenese
Aradu.RU1MG	Araip.9H8RU	multiple-copy	Signaltransductionhistidinekinase,hybrid-type,ethylenese
Aradu.AS0KA	Araip.T94IA	multiple-copy	Signaltransductionhistidinekinase,hybrid-type,ethylenese
Aradu.PZ2PD	Araip.Y45KL	multiple-copy	Signaltransductionhistidinekinase,hybrid-type,ethylenese
Aradu.S0871	Araip.Q2VVS	multiple-copy	single-strandedDNA-bindingprotein
Aradu.D81FK	Araip.8WF5E	multiple-copy	single-strandedDNA-bindingproteinWHY1,chloroplastic-li
Aradu.ZM6VG	Araip.JY10U	multiple-copy	single-strandedDNA-bindingproteinWHY3
Aradu.HM1JQ	Araip.YD0N6	multiple-copy	Single-strandednucleicacidbindingR3Hdomainproteinn=2
Aradu.316F6	Araip.8VP2X	multiple-copy	Single-strandednucleicacidbindingR3Hprotein
Aradu.7KU2A	Araip.IA1HS	multiple-copy	Single-strandednucleicacidbindingR3Hprotein
Aradu.20QKH	Araip.AYN5N	multiple-copy	sisterchromatidcohesion1protein1-likeisoformX2[Glyciner
Aradu.Y32L7	Araip.9J27P	multiple-copy	sisterchromatidcohesionproteinDCC1
Aradu.AS44H	Araip.8K50I	multiple-copy	sister-chromatidecohesionprotein
Aradu.D80L9	Araip.Q99GN	multiple-copy	SKP1-like21
Aradu.R0TXA	Araip.235AB	multiple-copy	SMAD/FHAdomain-containingprotein
Aradu.WZ6G2	Araip.80KYM	multiple-copy	Smallnuclearribonucleoproteinfamilyprotein

Aradu.GXP8K	Araip.Y5NRI	multiple-copy	SmallnuclearRNAactivatingcomplex(SNAPc),subunitSNA
Aradu.K38DA	Araip.Y7LP9	multiple-copy	sn1-specificdiacylglycerollipasebeta-like[Glycinemax]
Aradu.81RLF	Araip.3UW0X	multiple-copy	SNAREassociatedGolgiproteinfamily
Aradu.Z0DJ4	Araip.MR96W	multiple-copy	SNAREassociatedGolgiproteinfamily
Aradu.NP782	Araip.QNB65	multiple-copy	SNAREassociatedGolgiproteinfamily
Aradu.76UYG	Araip.AC28S	multiple-copy	SNARE-interactingproteinKEULE-likeisoformX1[Glycinem
Aradu.L3GA1	Araip.HRU9Y	multiple-copy	SNF1-relatedkinaseregulatorysubunitbeta-2
Aradu.D3HZI	Araip.N3LVC	multiple-copy	SNF1-relatedkinaseregulatorysubunitbeta-2
Aradu.90H62	Araip.X4YKT	multiple-copy	SNF1-relatedkinaseregulatorysubunitbeta-2
Aradu.24LGE	Araip.TVQ9L	multiple-copy	SNF1-relatedkinaseregulatorysubunitgamma1
Aradu.EMM34	Araip.67QDW	multiple-copy	SNF1-relatedproteinkinaseregulatorysubunitgamma1
Aradu.X2Y57	Araip.Q1BR2	multiple-copy	solubleinorganicpyrophosphatase
Aradu.V1INC	Araip.T13Y8	multiple-copy	solubleinorganicpyrophosphatase
Aradu.ZZD2I	Araip.HL73L	multiple-copy	solubleN-ethylmaleimide-sensitivefactoradaptorprotein33
Aradu.WEZ9F	Araip.JYT9K	multiple-copy	solutecarrierfamily35protein
Aradu.KDH0U	Araip.4R8ZS	multiple-copy	SOULheme-bindingfamilyprotein
Aradu.MMG1D	Araip.A8L1C	multiple-copy	SOULheme-bindingfamilyprotein
Aradu.X3A7J	Araip.JGK52	multiple-copy	spermidinehydroxycinnamoyltransferase-like[Glycinemax
Aradu.2QG6G	Araip.ZI6GM	multiple-copy	spermidinehydroxycinnamoyltransferase-like[Glycinemax
Aradu.E5KCW	Araip.L7Y2B	multiple-copy	spermidinesynthase3
Aradu.L1FUQ	Araip.G1ISM	multiple-copy	SPFH/Band7/PHBdomain-containingmembrane-associat
Aradu.C51SS	Araip.I3EE6	multiple-copy	SPFH/Band7/PHBdomain-containingmembrane-associat
Aradu.286YF	Araip.H5KZZ	multiple-copy	SPIRAL1-like1
Aradu.6Y48C	Araip.5XB02	multiple-copy	SPIRAL1-like2
Aradu.71U9H	Araip.E8ET3	multiple-copy	splicingfactor3Bsubunit2-like[Glycinemax]
Aradu.483RE	Araip.D5YHX	multiple-copy	splicingfactor3Bsubunit3-likeisoformX2[Glycinemax]
Aradu.GGF3N	Araip.M6ZJI	multiple-copy	splicingfactor3Bsubunit-likeprotein
Aradu.DK5WH	Araip.CR6RJ	multiple-copy	splicingfactorU2afsmallsubunitA-likeprotein
Aradu.Y5HX4	Araip.8HM3Z	multiple-copy	Spo11/DNAtopoisomeraseVI,subunitAprotein
Aradu.ZT0IB	Araip.81IM1	multiple-copy	SPT2homologisoformX1[Glycinemax]
Aradu.A97TU	Araip.B1QY2	multiple-copy	SPXdomaingene1
Aradu.UK481	Araip.LWR36	multiple-copy	SPXdomaingene1
Aradu.LNZ6T	Araip.QT8AK	multiple-copy	squamosapromoter-bindingprotein-like12
Aradu.W5KRK	Araip.RD4BX	multiple-copy	squamosapromoterbindingprotein-like14
Aradu.NXM6F	Araip.NP0MM	multiple-copy	squamosapromoterbindingprotein-like4
Aradu.UUC9E	Araip.N9DIE	multiple-copy	squamosapromoterbindingprotein-like5
Aradu.CGD8Q	Araip.W5573	multiple-copy	squamosapromoterbindingprotein-like9
Aradu.LJH4E	Araip.98AKT	multiple-copy	Staphylococcalnucleasehomologue
Aradu.8G93R	Araip.DDD6V	multiple-copy	starchsynthase3
Aradu.Z2UHU	Araip.M86M0	multiple-copy	Sterilealphamotif(SAM)domain-containingprotein
Aradu.ZG8VV	Araip.SKR9D	multiple-copy	Sterilealphamotif(SAM)domain-containingprotein
Aradu.TJ8NB	Araip.TNG4W	multiple-copy	Sterilealphamotif(SAM)domain-containingprotein
Aradu.RA5XM	Araip.QF1TG	multiple-copy	sterolmethyltransferase2
Aradu.JI3DJ	Araip.E5BJJ	multiple-copy	StressresponsiveA/BBarrelDomain

Aradu.PU1U6	Araip.9BQ78	multiple-copy	strictosidinesynthase-like3
Aradu.95964	Araip.ZNX0X	multiple-copy	Structuralconstituentofribosome,putativen=1Tax=Ricinus
Aradu.XDC1M	Araip.9M0VE	multiple-copy	Structuralconstituentofribosome,putativen=1Tax=Ricinus
Aradu.II5C0	Araip.8T4WW	multiple-copy	Structuralconstituentofribosome,putativen=4Tax=Filobasi
Aradu.4L5V2	Araip.CX1A1	multiple-copy	structuralconstituentofribosomeprotein
Aradu.82VY6	Araip.14A0C	multiple-copy	structuralmaintenanceofchromosomes2
Aradu.S63UK	Araip.7F16U	multiple-copy	structuralmaintenanceofchromosomes2
Aradu.D7ZWV	Araip.FP7ST	multiple-copy	subtilisin-likeserineprotease
Aradu.Y3TY0	Araip.00HCH	multiple-copy	subtilisin-likeserineprotease2
Aradu.EI4DF	Araip.2Y3EX	multiple-copy	subtilisin-likeserineprotease2
Aradu.N1UVT	Araip.332V3	multiple-copy	subtilisin-likeserineprotease2
Aradu.4W4JU	Araip.B0RRS	multiple-copy	subtilisin-likeserineprotease2
Aradu.B71RC	Araip.CYK8B	multiple-copy	subtilisin-likeserineprotease2
Aradu.D7NJ0	Araip.GZF1H	multiple-copy	subtilisin-likeserineprotease2
Aradu.68AHZ	Araip.IBG9Y	multiple-copy	subtilisin-likeserineprotease2
Aradu.1G3YF	Araip.JQ9KB	multiple-copy	subtilisin-likeserineprotease2
Aradu.52U3G	Araip.K797H	multiple-copy	subtilisin-likeserineprotease2
Aradu.M9A72	Araip.NM5LZ	multiple-copy	subtilisin-likeserineprotease2
Aradu.N98FX	Araip.PYI9U	multiple-copy	subtilisin-likeserineprotease2
Aradu.N47AG	Araip.S85UY	multiple-copy	subtilisin-likeserineprotease2
Aradu.80WBV	Araip.X40X6	multiple-copy	subtilisin-likeserineprotease2
Aradu.U5BRX	Araip.Y1ZA6	multiple-copy	subtilisin-likeserineprotease2
Aradu.VI09T	Araip.YT3RJ	multiple-copy	subtilisin-likeserineprotease2
Aradu.6UQ9U	Araip.60HFL	multiple-copy	subtilisin-likeserineprotease3
Aradu.LP8UK	Araip.NZT7X	multiple-copy	subtilisin-likeserineprotease3
Aradu.FMP57	Araip.N59F9	multiple-copy	succinatedehydrogenase[ubiquinone]iron-sulfursubunit
Aradu.W4ZB9	Araip.67ZY4	multiple-copy	succinatedehydrogenase1-1
Aradu.Z93ZE	Araip.NVE0S	multiple-copy	Succinatedehydrogenaseassemblyfactor2n=6Tax=Came
Aradu.S7P2G	Araip.TIT4D	multiple-copy	sucrosephosphatesynthase1F
Aradu.PD37S	Araip.51VQE	multiple-copy	sucrosesynthase6
Aradu.Q59PD	Araip.FBY21	multiple-copy	sucrose-protonsymporter7
Aradu.QA3D9	Araip.FR0CD	multiple-copy	sucrosetransporter4
Aradu.ZN5ZV	Araip.NX2DL	multiple-copy	sucrosetransporter4
Aradu.751D4	Araip.SJI2G	multiple-copy	sucrosetransporter4
Aradu.95EP3	Araip.XF8RP	multiple-copy	sucrosetransporter4
Aradu.4RZ6U	Araip.4LV9F	multiple-copy	sugarphosphate/phosphatetranslocator[Glycinemax]
Aradu.50KFD	Araip.RS4E7	multiple-copy	sugarphosphate/phosphatetranslocator[Glycinemax]
Aradu.V56CC	Araip.UKG53	multiple-copy	sugarphosphate/phosphatetranslocator[Glycinemax]
Aradu.7QE0L	Araip.V7V2P	multiple-copy	sugarphosphate/phosphatetranslocator[Glycinemax]
Aradu.Z8VFD	Araip.8R4SA	multiple-copy	sugarporter(SP)familyMFStransporter
Aradu.08Y2X	Araip.GL06B	multiple-copy	sugarporter(SP)familyMFStransporter
Aradu.CR12A	Araip.ID0PF	multiple-copy	sugartransporter14
Aradu.VD3WH	Araip.LX0AL	multiple-copy	sugarporter(SP)familyMFStransporter
Aradu.L5IHI	Araip.X4GNJ	multiple-copy	sugarporter(SP)familyMFStransporter

Aradu.6BH4S	Araip.MS5EY	multiple-copy	sugartransporter1
Aradu.K08LS	Araip.JRK98	multiple-copy	sugartransporter11
Aradu.81PPC	Araip.83U1D	multiple-copy	sugartransporter14
Aradu.YRM8T	Araip.G26YQ	multiple-copy	sugarporter(SP)familyMFStransporter
Aradu.3CD7I	Araip.RBV5T	multiple-copy	sugartransporterprotein7
Aradu.H4VY0	Araip.2E2K8	multiple-copy	SugartransporterSWEETn=2Tax=CitrusRepID=V4SX91_
Aradu.0C27S	Araip.REH1B	multiple-copy	SugartransporterSWEETn=2Tax=PooideaeRepID=I1HKV
Aradu.IX4YQ	Araip.JQ4TL	multiple-copy	SugartransporterSWEETn=2Tax=TriticumRepID=M7ZHC
Aradu.U6YR6	Araip.X0SC5	multiple-copy	SugartransporterSWEETn=3Tax=CitrusRepID=V4TK53_
Aradu.28KIR	Araip.LSW2G	multiple-copy	SugartransporterSWEETn=3Tax=PhaseoleaeRepID=I1M
Aradu.JS1AI	Araip.BV9QU	multiple-copy	SugartransporterSWEETn=3Tax=SolanumRepID=K4BJH
Aradu.K52XK	Araip.S4KSY	multiple-copy	SugartransporterSWEETn=5Tax=PhaseoleaeRepID=I1K
Aradu.L61KF	Araip.CN5UC	multiple-copy	sugartransportprotein5-like[Glycinemax]
Aradu.T5RWU	Araip.Y9H0E	multiple-copy	sulfatetransporter1
Aradu.AU4WK	Araip.N8F74	multiple-copy	sulfatetransporter3
Aradu.GN2JM	Araip.Q2C6W	multiple-copy	sulfatetransporter1
Aradu.S94TG	Araip.QE1LN	multiple-copy	sulfatetransporter3
Aradu.JU8EK	Araip.9W1BE	multiple-copy	sulfatetransporter-likeprotein
Aradu.EU3KX	Araip.VGU5W	multiple-copy	SulfiteexporterTauE/SafEfamilyprotein
Aradu.L5Q0P	Araip.5YV6C	multiple-copy	SUPPRESSOROFGENESILENCING3-likeisoformX3[Gly
Aradu.MBF39	Araip.7L5BF	multiple-copy	SUPPRESSOROFGENESILENCING3-likeisoformX3[Gly
Aradu.5E5UI	Araip.TLQ46	multiple-copy	survivalproteinSurE-likephosphatase/nucleotidase
Aradu.JYC3Z	Araip.8K2W2	multiple-copy	SWAP(Suppressor-of-White-APricot)/surpRNA-bindingdo
Aradu.47F2N	Araip.XGP93	multiple-copy	SWAP(Suppressor-of-White-APricot)/surpRNA-bindingdo
Aradu.71DP5	Araip.HDJ4D	multiple-copy	SWI/SNFcomplexcomponentSNF12homologisoformX2[G
Aradu.65QW9	Araip.T587R	multiple-copy	SWI/SNFcomplexsubunitSWI3C-like[Glycinemax]
Aradu.I9U5I	Araip.627JR	multiple-copy	SWIB/MDM2domainsuperfamilyprotein
Aradu.4M8RF	Araip.82XGB	multiple-copy	SWIB/MDM2domainsuperfamilyprotein
Aradu.M3Q0G	Araip.87I2H	multiple-copy	SWIB/MDM2domainsuperfamilyprotein
Aradu.24BNI	Araip.CBT0Y	multiple-copy	SWIB/MDM2domainsuperfamilyprotein
Aradu.MI8WP	Araip.9V0W5	multiple-copy	synaptotagmin-5-like[Glycinemax]
Aradu.TC75Q	Araip.RW9KD	multiple-copy	syntaxin,putative
Aradu.I4YNH	Araip.09EYJ	multiple-copy	Syntaxin/t-SNAREfamilyprotein
Aradu.SM6NC	Araip.TG6SS	multiple-copy	syntaxin/T-SNAREfamilyprotein
Aradu.N8P27	Araip.YG292	multiple-copy	syntaxin-32-like[Glycinemax]
Aradu.0P9WS	Araip.QK4T6	multiple-copy	syntaxin-71-like[Glycinemax]
Aradu.4Q38F	Araip.JP2KL	multiple-copy	syntaxinofplants124
Aradu.K26VM	Araip.WYW75	multiple-copy	syntaxin-121-like[Glycinemax]
Aradu.Z06PE	Araip.DT1LC	multiple-copy	syntaxinofplants131
Aradu.A0SRT	Araip.GV3PW	multiple-copy	syntaxinofplants52
Aradu.XBE20	Araip.IG5LD	multiple-copy	taurinecatabolismdioxygenaseTauD/TfdA
Aradu.IU0EI	Araip.KG3H5	multiple-copy	T-complexprotein1alphasubunit
Aradu.40X7J	Araip.A5SHC	multiple-copy	T-complexprotein1subunitepsilon-like[Glycinemax]
Aradu.Y62C6	Araip.RT1VC	multiple-copy	T-complexprotein1subuniteta-like[Glycinemax]

Aradu.B3U8E	Araip.TY86Z	multiple-copy	T-complexprotein1subunitgamma-like[Glycinemax]
Aradu.UWX45	Araip.W7JXY	multiple-copy	T-complexprotein1subunitzeta-like[Glycinemax]
Aradu.C73IQ	Araip.J0WZR	multiple-copy	TCP-1/cpn60chaperoninfamilyprotein
Aradu.JKM1G	Araip.X255Z	multiple-copy	TCPfamilytranscriptionfactor
Aradu.6M9JS	Araip.Y5APX	multiple-copy	TCPfamilytranscriptionfactor
Aradu.J06JT	Araip.Z17TF	multiple-copy	TCPfamilytranscriptionfactor4
Aradu.Z5EFE	Araip.CD6XJ	multiple-copy	telomererepeat-bindingfactor2-likeisoformX2[Glycinemax
Aradu.DPF9Q	Araip.CL2J8	multiple-copy	telomererepeat-bindingprotein1-likeisoformX1[Glycinema
Aradu.B4YLF	Araip.7P4U9	multiple-copy	telomererepeat-bindingprotein3-likeisoformX2[Glycinema
Aradu.01TNR	Araip.RX5RL	multiple-copy	telomererepeat-bindingprotein5-likeisoformX3[Glycinema
Aradu.A8ITS	Araip.QP80U	multiple-copy	terpenesynthase10
Aradu.5CY6X	Araip.ARQ95	multiple-copy	terpenesynthase14
Aradu.V7ZTF	Araip.LM2JS	multiple-copy	terpenesynthase14
Aradu.PU456	Araip.76CRM	multiple-copy	terpenesynthase21
Aradu.9J6QD	Araip.C41LK	multiple-copy	terpenesynthase21
Aradu.Y2ZCM	Araip.H04N0	multiple-copy	terpenesynthase21
Aradu.8542F	Araip.NWK8S	multiple-copy	terpenesynthasefamily,metal-bindingdomainprotein
Aradu.L75IP	Araip.DC4LL	multiple-copy	Tesmin/TSO1-likeCXCdomain-containingprotein
Aradu.4S1TT	Araip.M800H	multiple-copy	Tesmin/TSO1-likeCXCdomain-containingprotein
Aradu.L6U41	Araip.YNW3R	multiple-copy	Tesmin/TSO1-likeCXCdomain-containingprotein
Aradu.SE8E9	Araip.0JU1Y	multiple-copy	tetraspanin-11[Glycinemax]
Aradu.P0TWG	Araip.H444F	multiple-copy	tetraspanin-19-likeisoform1[Glycinemax]
Aradu.Q71BR	Araip.71CN8	multiple-copy	tetraspanin-2[Glycinemax]
Aradu.QU3KT	Araip.F18V2	multiple-copy	tetraspanin-2[Glycinemax]
Aradu.NRG58	Araip.GV4V3	multiple-copy	tetraspanin-2[Glycinemax]
Aradu.1H2UR	Araip.M07D2	multiple-copy	tetraspanin-3[Glycinemax]
Aradu.8W1FQ	Araip.JM8Z9	multiple-copy	Tetraspaninfamilyprotein
Aradu.F66UW	Araip.X1Q90	multiple-copy	tetratricopeptiderepeat(TPR)-containingprotein
Aradu.KUN1X	Araip.3R0IT	multiple-copy	Tetratricopeptiderepeat(TPR)-likesuperfamilyprotein
Aradu.PVS6M	Araip.3W28R	multiple-copy	Tetratricopeptiderepeat(TPR)-likesuperfamilyprotein
Aradu.DK67P	Araip.4F1IC	multiple-copy	Tetratricopeptiderepeat(TPR)-likesuperfamilyprotein
Aradu.3L3H2	Araip.57TGT	multiple-copy	Tetratricopeptiderepeat(TPR)-likesuperfamilyprotein
Aradu.ZJ042	Araip.5NU5L	multiple-copy	Tetratricopeptiderepeat(TPR)-likesuperfamilyprotein
Aradu.S6JX1	Araip.5Y1QQ	multiple-copy	Tetratricopeptiderepeat(TPR)-likesuperfamilyprotein
Aradu.1G4QF	Araip.7D543	multiple-copy	Tetratricopeptiderepeat(TPR)-likesuperfamilyprotein
Aradu.1EH7R	Araip.7N70L	multiple-copy	Tetratricopeptiderepeat(TPR)-likesuperfamilyprotein
Aradu.Q86YJ	Araip.DH2VM	multiple-copy	Tetratricopeptiderepeat(TPR)-likesuperfamilyprotein
Aradu.U1FJC	Araip.FW0V7	multiple-copy	Tetratricopeptiderepeat(TPR)-likesuperfamilyprotein
Aradu.Z84W9	Araip.L2LE7	multiple-copy	Tetratricopeptiderepeat(TPR)-likesuperfamilyprotein
Aradu.H8AL3	Araip.N54GH	multiple-copy	Tetratricopeptiderepeat(TPR)-likesuperfamilyprotein
Aradu.LBR87	Araip.Q54JE	multiple-copy	Tetratricopeptiderepeat(TPR)-likesuperfamilyprotein
Aradu.75JKD	Araip.QGQ22	multiple-copy	Tetratricopeptiderepeat(TPR)-likesuperfamilyprotein
Aradu.V2YPR	Araip.YPA63	multiple-copy	Tetratricopeptiderepeat(TPR)-likesuperfamilyprotein
Aradu.P82NP	Araip.E05X3	multiple-copy	tetratricopeptiderepeatprotein14-like[Glycinemax]

Aradu.9CT7A	Araip.19472	multiple-copy	tetratricopeptiderepeatprotein1-likeisoformX1[Glycinema
Aradu.1L0UP	Araip.D7K2H	multiple-copy	tetratricopeptiderepeatprotein13-like[Glycinemax]
Aradu.2LE9C	Araip.D7U08	multiple-copy	tetratricopeptiderepeatprotein1-likeisoformX1[Glycinema
Aradu.E6N9S	Araip.L1T00	multiple-copy	tetratricopeptiderepeatprotein5-likeisoformX1[Glycinema
Aradu.I1SW1	Araip.R7SWT	multiple-copy	tetratricopeptiderepeatprotein7A-likeisoformX1[Glycinem
Aradu.SL6TQ	Araip.Y3EN4	multiple-copy	tetratricopeptiderepeatprotein7A-likeisoformX1[Glycinem
Aradu.83CAT	Araip.E0GHH	multiple-copy	tetratricopeptiderepeatprotein7B-likeisoformX2[Glycinem
Aradu.NJ1ET	Araip.KE2SI	multiple-copy	Tetratricopeptiderepeatproteinn=1Tax=Synechococcuss
Aradu.BCZ93	Araip.0W448	multiple-copy	TGACG-sequence-specificDNA-bindingproteinTGA-1B-li
Aradu.315HW	Araip.6L19K	multiple-copy	TGACG-sequence-specificDNA-bindingproteinTGA-1B-li
Aradu.KB6LK	Araip.ETI9M	multiple-copy	TGACG-sequence-specificDNA-bindingproteinTGA-1B-li
Aradu.Q3HQI	Araip.I5D0R	multiple-copy	TGACG-sequence-specificDNA-bindingproteinTGA-1B-li
Aradu.MF9WN	Araip.ML4Q2	multiple-copy	TGACG-sequence-specificDNA-bindingproteinTGA-1B-li
Aradu.898PR	Araip.TQF5F	multiple-copy	TGACG-sequence-specificDNA-bindingproteinTGA-1B-li
Aradu.X6FMF	Araip.I5L57	multiple-copy	TGACG-sequence-specificDNA-bindingproteinTGA-1Bn=
Aradu.M9L15	Araip.NZ32C	multiple-copy	TGACG-sequence-specificDNA-bindingproteinTGA-2.1-li
Aradu.GUH8G	Araip.D5QVM	multiple-copy	TGACG-sequence-specificDNA-bindingproteinTGA-2.1-li
Aradu.U5DG8	Araip.W8KP6	multiple-copy	TGACG-sequence-specificDNA-bindingproteinTGA-2.1-li
Aradu.C716W	Araip.X0N4G	multiple-copy	TGACG-sequence-specificDNA-bindingproteinTGA-2.1-li
Aradu.BQ4IU	Araip.W3SNY	multiple-copy	TGACG-sequence-specificDNA-bindingproteinTGA-2.1-li
Aradu.8F3EB	Araip.GEV84	multiple-copy	thaumatin-likeprotein3
Aradu.ETS8A	Araip.ZQ0IN	multiple-copy	thaumatin-likeprotein3
Aradu.61CW5	Araip.WP004	multiple-copy	thaumatin-likeprotein-like[Glycinemax]
Aradu.BYZ1A	Araip.4G5WD	multiple-copy	thiol-disulfideoxidoreductaseDCC
Aradu.4V2X0	Araip.7BB5W	multiple-copy	thioredoxin2
Aradu.IHP1V	Araip.EHM8Y	multiple-copy	thioredoxin2
Aradu.71JL3	Araip.Z394L	multiple-copy	thioredoxin2
Aradu.CK902	Araip.80FVV	multiple-copy	thioredoxin3
Aradu.6GP3J	Araip.XJ5ED	multiple-copy	thioredoxin-dependentperoxidase1
Aradu.34MNK	Araip.280PC	multiple-copy	thioredoxindomain-containingprotein9homolog
Aradu.X4GW8	Araip.L5NAQ	multiple-copy	thioredoxinF2
Aradu.I0VP0	Araip.GP5GN	multiple-copy	thioredoxinfamilyTrp26protein
Aradu.GA8V3	Araip.0Y4CE	multiple-copy	Thioredoxinfold,putativeisoform1n=1Tax=Theobromacac
Aradu.N0VBS	Araip.3QQ7J	multiple-copy	thioredoxinO1
Aradu.24PXZ	Araip.IMW9T	multiple-copy	Thioredoxinsuperfamilyprotein
Aradu.0QF6H	Araip.03GF9	multiple-copy	Thioredoxinsuperfamilyprotein
Aradu.4YZ2K	Araip.0CL7X	multiple-copy	Thioredoxinsuperfamilyprotein
Aradu.SG5SM	Araip.0D7QS	multiple-copy	Thioredoxinsuperfamilyprotein
Aradu.PT44X	Araip.1SL1G	multiple-copy	Thioredoxinsuperfamilyprotein
Aradu.UA6JC	Araip.GU4P9	multiple-copy	Thioredoxinsuperfamilyprotein
Aradu.TES1U	Araip.H56DJ	multiple-copy	Thioredoxinsuperfamilyprotein
Aradu.04VAI	Araip.U7YDL	multiple-copy	Thioredoxinsuperfamilyprotein
Aradu.EWB3L	Araip.UFN92	multiple-copy	Thioredoxinsuperfamilyprotein
Aradu.LVQ6D	Araip.35TV0	multiple-copy	Thioredoxinz

Aradu.DB14S	Araip.Y8L0P	multiple-copy	thylakoidlumenal19kDaprotein
Aradu.7GQ9E	Araip.SX1UB	multiple-copy	thylakoidmembranephosphoprotein14kDaprotein
Aradu.YC3GJ	Araip.GI5GJ	multiple-copy	TIC20-v,chloroplastic-like[Glycinemax]
Aradu.H9EEY	Araip.FXS1L	multiple-copy	TIC62,chloroplastic-likeisoformX2[Glycinemax]
Aradu.33VZ6	Araip.H94QL	multiple-copy	TIFYdomain/DivergentCCTmotiffamilyprotein
Aradu.H976G	Araip.V3GZS	multiple-copy	TIMEFORCOFFEE-likeisoformX2[Glycinemax]
Aradu.GZ5P7	Araip.8Z142	multiple-copy	TMVresistanceproteinN-like[Glycinemax]
Aradu.NP275	Araip.LY9Q9	multiple-copy	TMVresistanceproteinN-like[Glycinemax]
Aradu.L8JCD	Araip.QF0UL	multiple-copy	tobamovirusmultiplication2B
Aradu.617EV	Araip.Y6XIR	multiple-copy	tonoplastintrinsicprotein1
Aradu.H365I	Araip.Z5U4R	multiple-copy	topless-relatedprotein3-likeisoformX1[Glycinemax]
Aradu.7G8QU	Araip.SZR1J	multiple-copy	TPRrepeat-containingthioredoxinTTL1[Glycinemax]
Aradu.GIY15	Araip.93AYN	multiple-copy	TPRrepeat-containingthioredoxinTTL1-like[Glycinemax]
Aradu.Z639Y	Araip.J3QMD	multiple-copy	TPRrepeat-containingthioredoxinTTL1-like[Glycinemax]
Aradu.IS3BT	Araip.Q0RIX	multiple-copy	TPRrepeat-containingthioredoxinTTL1-like[Glycinemax]
Aradu.VT3C0	Araip.W7YQ0	multiple-copy	TPRrepeat-containingthioredoxinTTL1-like[Glycinemax]
Aradu.EG568	Araip.HF59E	multiple-copy	TPRrepeatprotein
Aradu.S6195	Araip.KVK3X	multiple-copy	TPRrepeatprotein
Aradu.87YLX	Araip.8T3QY	multiple-copy	TPRXL-likeisoformX2[Glycinemax]
Aradu.65NT6	Araip.5FB8R	multiple-copy	TPRXL-likeisoformX3[Glycinemax]
Aradu.AKP9L	Araip.F92FW	multiple-copy	TPX2(targetingproteinforXklp2)proteinfamily
Aradu.D5Z0P	Araip.M4UKA	multiple-copy	TPX2(targetingproteinforXklp2)proteinfamily
Aradu.T3ZAX	Araip.X95V4	multiple-copy	TPX2(targetingproteinforXklp2)proteinfamily
Aradu.LF9C0	Araip.67C1Y	multiple-copy	TPX2-likeisoformX3[Glycinemax]
Aradu.UD2Q8	Araip.ZV5ML	multiple-copy	TraBfamilyprotein
Aradu.U16MC	Araip.18PWY	multiple-copy	traffickingproteinparticlecomplexsubunit-likeprotein
Aradu.8N3KD	Araip.E3Z66	multiple-copy	traffickingproteinparticlecomplexsubunit-likeprotein
Aradu.U4UF8	Araip.NAY3U	multiple-copy	traffickingproteinparticlecomplexsubunit-likeprotein
Aradu.M0XAT	Araip.M3EG0	multiple-copy	TRAF-likesuperfamilyprotein
Aradu.QU580	Araip.HP125	multiple-copy	TRAM,LAG1andCLN8(TLC)lipid-sensingdomaincontainin
Aradu.FSY43	Araip.I9EB2	multiple-copy	TRAM,LAG1andCLN8(TLC)lipid-sensingdomaincontainin
Aradu.IWR1X	Araip.AE0WJ	multiple-copy	trans-2-enoyl-CoAreductase,mitochondrial-like[Glycinema
Aradu.Z8HLS	Araip.09BNR	multiple-copy	transcriptionalcorepressorSEUSS-likeisoformX2[Glyciner
Aradu.0BM64	Araip.C8HFV	multiple-copy	transcriptionelongationfactorB
Aradu.FWD57	Araip.Q2LZS	multiple-copy	transcriptionelongationfactorSPT4-likeprotein
Aradu.4094W	Araip.J0JMG	multiple-copy	TranscriptionelongationfactorSpt5
Aradu.D69CU	Araip.7B3CC	multiple-copy	transcriptionfactor
Aradu.4NW8B	Araip.VCZ4R	multiple-copy	transcriptionfactor
Aradu.0572C	Araip.Z1VRT	multiple-copy	transcriptionfactor
Aradu.L6791	Araip.32X2R	multiple-copy	transcriptionfactor,putative
Aradu.P05N1	Araip.T5WNM	multiple-copy	transcriptionfactor25-like[Glycinemax]
Aradu.6IB2A	Araip.T4V1N	multiple-copy	generaltranscriptionfactorIIH2
Aradu.898CQ	Araip.M9JCH	multiple-copy	transcriptionfactorIIIA
Aradu.C3QXP	Araip.38J51	multiple-copy	transcriptionfactorjumonji(jmjC)domain-containingprotein

Aradu.0291B	Araip.FW7GM	multiple-copy	Transcriptionfactorjumonji(jmjC)domain-containingprotein
Aradu.QQY2K	Araip.G1GCS	multiple-copy	Transcriptionfactorjumonji(jmjC)domain-containingprotein
Aradu.9L2RT	Araip.A16W6	multiple-copy	transcriptionfactorjumonjidomainprotein
Aradu.908DD	Araip.PMK11	multiple-copy	transcriptionfactor-related
Aradu.GA7ZA	Araip.RVB68	multiple-copy	TranscriptioninitiationfactorIIF,betasubunit
Aradu.NGG06	Araip.PE9PU	multiple-copy	transcriptioninitiationfactorIIFsubunitalpha
Aradu.6Q6K7	Araip.WCB4Z	multiple-copy	transcriptioninitiationfactorTFIID,subunitTAF11
Aradu.FW1J1	Araip.E8JYR	multiple-copy	transcriptioninitiationfactorTFIIDsubunit4b-likeisoformX1[(
Aradu.FNH9F	Araip.VQ5T4	multiple-copy	transcriptioninitiationfactorTFIIDsubunit5-likeisoformX1[G
Aradu.22BDB	Araip.X17SE	multiple-copy	mediatorofRNApolymeraseIItranscriptionsubunit15a-likeis
Aradu.JR9Q4	Araip.E6H2Y	multiple-copy	transcriptioninitiationfactorTFIIDsubunit9-like[Glycinemax
Aradu.00SPE	Araip.PG7W4	multiple-copy	TranscriptioninitiationfactorTFIIDsubunitA
Aradu.SE6Y8	Araip.8IH70	multiple-copy	TranscriptioninitiationfactorTFIIE,betasubunit
Aradu.E9QI4	Araip.XRM4G	multiple-copy	TranscriptioninitiationfactorTFIIE,betasubunit
Aradu.ZD5DD	Araip.ST1UP	multiple-copy	transcriptionterminationfactor,mitochondrial-like[Glycinem
Aradu.085T6	Araip.852K4	multiple-copy	transferringglycosylgrouptransferase
Aradu.6V4TL	Araip.99AN8	multiple-copy	transferringglycosylgrouptransferase
Aradu.CQ0KB	Araip.GWH2N	multiple-copy	transferringglycosylgrouptransferase
Aradu.ZEU97	Araip.PH1BR	multiple-copy	transferringglycosylgrouptransferase
Aradu.PQF90	Araip.R4PLR	multiple-copy	transferringglycosylgrouptransferase
Aradu.19YSM	Araip.XY52C	multiple-copy	transferringglycosylgrouptransferase
Aradu.S0JY2	Araip.YP690	multiple-copy	transferringglycosylgrouptransferase
Aradu.U3Z1S	Araip.U82NT	multiple-copy	transferringglycosylgrouptransferase,putative
Aradu.7FW6D	Araip.QJ62N	multiple-copy	translationinitiationfactor2
Aradu.BD9UN	Araip.JR03F	multiple-copy	Translationinitiationfactor2,smallGTP-bindingprotein
Aradu.7B9HI	Araip.NWV62	multiple-copy	translationinitiationfactor2alphasubunit
Aradu.EI543	Araip.A2EQF	multiple-copy	translationinitiationfactor3(IF-3)familyprotein
Aradu.E90C6	Araip.16F3N	multiple-copy	translationinitiationfactor3B-2
Aradu.GM25T	Araip.Y359Z	multiple-copy	translationinitiationfactor3K
Aradu.56DPU	Araip.NX9LF	multiple-copy	translationinitiationfactor3subunit7(eIF-3)
Aradu.CU20J	Araip.K6B0A	multiple-copy	translationinitiationfactor3subunitI
Aradu.3127Y	Araip.V6XPZ	multiple-copy	translationinitiationfactor3subunitL-like[Glycinemax]
Aradu.DY43A	Araip.G9AT5	multiple-copy	translationinitiationfactor4B1
Aradu.07V9Z	Araip.00H39	multiple-copy	translationinitiationfactor4E
Aradu.A252K	Araip.LK0RZ	multiple-copy	translationinitiationfactor4E
Aradu.U8FLG	Araip.09VK0	multiple-copy	translationinitiationfactor4G
Aradu.U3ACN	Araip.0XQ95	multiple-copy	translationinitiationfactor4G
Aradu.A2SS4	Araip.9P4NA	multiple-copy	translationinitiationfactor4G
Aradu.UD5RI	Araip.HF1C0	multiple-copy	translationinitiationfactor4G
Aradu.L933I	Araip.ZLC7F	multiple-copy	translationinitiationfactor6-likeprotein
Aradu.4M2H4	Araip.7P4BA	multiple-copy	translationinitiationfactoreIF-2Bdeltasubunit
Aradu.IW0ES	Araip.W6LGW	multiple-copy	translationinitiationfactoreIF-2Bdeltasubunit
Aradu.JJ0LZ	Araip.G085N	multiple-copy	translationinitiationfactoreIF-2Bsubunitalpha-like[Glycinem
Aradu.0WP1T	Araip.KU7H8	multiple-copy	translationinitiationfactoreIF-2Bsubunitalpha-like[Glycinem

Aradu.B74MT	Araip.B2Q36	multiple-copy	translationinitiationfactoreIF-2Bsubunitepsilon-like[Glycine
Aradu.X88X0	Araip.T7WB5	multiple-copy	transmembrane9superfamilymember3-like[Glycinemax]
Aradu.ISD6F	Araip.SCJ6B	multiple-copy	transmembrane9superfamilymember4-like[Glycinemax]
Aradu.8L9C6	Araip.UK2C9	multiple-copy	transmembrane9superfamilymember4-like[Glycinemax]
Aradu.6WI58	Araip.XEB47	multiple-copy	transmembrane9superfamilymember4-like[Glycinemax]
Aradu.J1D71	Araip.26W3E	multiple-copy	transmembraneaminoacidtransporterfamilyprotein
Aradu.H1YN5	Araip.5IP7M	multiple-copy	Transmembraneaminoacidtransporterfamilyprotein
Aradu.D0XFS	Araip.71U6H	multiple-copy	Transmembraneaminoacidtransporterfamilyprotein
Aradu.LT37C	Araip.7ER0N	multiple-copy	Transmembraneaminoacidtransporterfamilyprotein
Aradu.S2HHA	Araip.893D0	multiple-copy	Transmembraneaminoacidtransporterfamilyprotein
Aradu.1HX6J	Araip.8A3C5	multiple-copy	Transmembraneaminoacidtransporterfamilyprotein
Aradu.KD75D	Araip.8M6IT	multiple-copy	transmembraneaminoacidtransporterfamilyprotein
Aradu.BG56S	Araip.AVA7B	multiple-copy	Transmembraneaminoacidtransporterfamilyprotein
Aradu.20KIH	Araip.BQC59	multiple-copy	transmembraneaminoacidtransporterfamilyprotein
Aradu.UD1IS	Araip.BTG7R	multiple-copy	Transmembraneaminoacidtransporterfamilyprotein
Aradu.FA88A	Araip.F8FRB	multiple-copy	Transmembraneaminoacidtransporterfamilyprotein
Aradu.295V7	Araip.FC9LL	multiple-copy	Transmembraneaminoacidtransporterfamilyprotein
Aradu.805L3	Araip.GT9SI	multiple-copy	Transmembraneaminoacidtransporterfamilyprotein
Aradu.78FH9	Araip.I5LSS	multiple-copy	transmembraneaminoacidtransporterfamilyprotein
Aradu.UWB5V	Araip.IFL1M	multiple-copy	Transmembraneaminoacidtransporterfamilyprotein
Aradu.0AW6N	Araip.K08CD	multiple-copy	Transmembraneaminoacidtransporterfamilyprotein
Aradu.I88HR	Araip.PN7VH	multiple-copy	transmembraneaminoacidtransporterfamilyprotein
Aradu.B83GZ	Araip.Q6R0G	multiple-copy	Transmembraneaminoacidtransporterfamilyprotein
Aradu.TJK3R	Araip.R22ES	multiple-copy	Transmembraneaminoacidtransporterfamilyprotein
Aradu.I6GSC	Araip.RB0W1	multiple-copy	Transmembraneaminoacidtransporterfamilyprotein
Aradu.KBD84	Araip.T22BQ	multiple-copy	Transmembraneaminoacidtransporterfamilyprotein
Aradu.G6PP5	Araip.U49S5	multiple-copy	transmembraneaminoacidtransporterfamilyprotein
Aradu.D2NW0	Araip.UZ3PZ	multiple-copy	Transmembraneaminoacidtransporterfamilyprotein
Aradu.2W9TG	Araip.W0U7G	multiple-copy	Transmembraneaminoacidtransporterfamilyprotein
Aradu.CG1AI	Araip.WJJ4Z	multiple-copy	transmembraneaminoacidtransporterfamilyprotein
Aradu.6JB9C	Araip.YSG6K	multiple-copy	transmembraneaminoacidtransporterfamilyprotein
Aradu.G1DD8	Araip.ZWQ00	multiple-copy	Transmembraneaminoacidtransporterfamilyprotein
Aradu.V83DR	Araip.KZF5J	multiple-copy	transmembraneprotein
Aradu.MP2DM	Araip.0K6MU	multiple-copy	transmembraneprotein,putative
Aradu.WJ6G7	Araip.0U7A4	multiple-copy	transmembraneprotein,putative
Aradu.E34IK	Araip.12ZFL	multiple-copy	transmembraneprotein,putative
Aradu.UVG4U	Araip.5FY0D	multiple-copy	transmembraneprotein,putative
Aradu.7N548	Araip.6PA9N	multiple-copy	transmembraneprotein,putative
Aradu.CV157	Araip.8LE7X	multiple-copy	transmembraneprotein,putative
Aradu.6S4B3	Araip.BV4C5	multiple-copy	transmembraneprotein,putative
Aradu.RUD1R	Araip.G5J45	multiple-copy	transmembraneprotein,putative
Aradu.07ZWB	Araip.I4558	multiple-copy	transmembraneprotein,putative
Aradu.IF1JM	Araip.IEW6X	multiple-copy	transmembraneprotein,putative
Aradu.RAP3E	Araip.JNX15	multiple-copy	transmembraneprotein,putative

Aradu.D5C91	Araip.P61QJ	multiple-copy	transmembraneprotein,putative
Aradu.DE67Z	Araip.QI669	multiple-copy	transmembraneprotein,putative
Aradu.8Y02T	Araip.RR9FR	multiple-copy	transmembraneprotein,putative
Aradu.TK9TC	Araip.YJ2ZI	multiple-copy	transmembraneprotein,putative
Aradu.6JU5H	Araip.I2XBN	multiple-copy	transmembraneprotein184A-like[Glycinemax]
Aradu.TF9ZM	Araip.B188M	multiple-copy	transmembraneprotein184C-likeisoformX2[Glycinemax]
Aradu.MQQ90	Araip.QDL0D	multiple-copy	transmembraneprotein184C-likeisoformX2[Glycinemax]
Aradu.SK7RE	Araip.ISZ7W	multiple-copy	transmembraneprotein230-likeisoformX4[Glycinemax]
Aradu.44DMI	Araip.290GK	multiple-copy	transmembraneprotein230-likeisoformX5[Glycinemax]
Aradu.1ZR6L	Araip.CFW6I	multiple-copy	transmembraneprotein45B-like[Glycinemax]
Aradu.X3LUW	Araip.D2PEY	multiple-copy	transmembraneprotein45B-like[Glycinemax]
Aradu.98WRB	Araip.PPQ7L	multiple-copy	transmembraneprotein45B-like[Glycinemax]
Aradu.TV03P	Araip.68E16	multiple-copy	Transmembraneprotein97,predicted
Aradu.WF5M4	Araip.D814V	multiple-copy	transmembraneproteinadipocyte-associated1homolog[Gl
Aradu.16E7C	Araip.JG5RP	multiple-copy	transmembraneproteinadipocyte-associated1homolog[Gl
Aradu.4BB0R	Araip.B82I6	multiple-copy	TransmembraneproteinC20orf108n=2Tax=Medicagotrunc
Aradu.5TM0N	Araip.JBC57	multiple-copy	Transmembraneproteinn=1Tax=MedicagotruncatulaRepI
Aradu.F6LZ6	Araip.RM2KP	multiple-copy	Transmembraneproteins14C
Aradu.YPK4Q	Araip.V8CIS	multiple-copy	Transmembraneproteins14C
Aradu.YC3N7	Araip.YBY9J	multiple-copy	Transmembraneproteins14C
Aradu.P2KV0	Araip.774UX	multiple-copy	transportinhibitorresponse1-likeprotein-like[Glycinemax]
Aradu.N9F03	Araip.Y1FMZ	multiple-copy	transportinhibitorresponse1-likeprotein-like[Glycinemax]
Aradu.A90PK	Araip.EL8BY	multiple-copy	transportSec61subunitalpha-like[Glycinemax]
Aradu.JZT13	Araip.GGW4P	multiple-copy	trehalosephosphatesynthase
Aradu.TVX9T	Araip.J98GW	multiple-copy	trehalosephosphatesynthase
Aradu.CHD0U	Araip.UK5PE	multiple-copy	trehalosephosphatesynthase
Aradu.WN017	Araip.8VB8P	multiple-copy	triacylglycerollipase-like1
Aradu.D77GP	Araip.K9GJK	multiple-copy	trichohyalin-likeisoformX3[Glycinemax]
Aradu.I625R	Araip.RSM1K	multiple-copy	trichohyalin-likeisoformX4[Glycinemax]
Aradu.7FN1T	Araip.X1WI1	multiple-copy	trihelixGT-2-like[Glycinemax]
Aradu.XU3IM	Araip.AC5JZ	multiple-copy	trihelixGT-3b-like[Glycinemax]
Aradu.9YL2F	Araip.IRM4P	multiple-copy	trihelixGT-3b-like[Glycinemax]
Aradu.ZS6PI	Araip.RA8N4	multiple-copy	trihelixGTL1-like[Glycinemax]
Aradu.752JK	Araip.L3SN7	multiple-copy	tripeptidylpeptidaseii
Aradu.1I015	Araip.6E6I2	multiple-copy	tRNA(guanine(37)-N1)-methyltransferase,putative
Aradu.XF0PF	Araip.37ILI	multiple-copy	tRNA(guanine-N(7)-)-methyltransferasenon-catalyticsubu
Aradu.P778G	Araip.Q83B6	multiple-copy	tRNA/rRNAmethyltransferase(SpoU)familyprotein
Aradu.Z8XIW	Araip.KCK8F	multiple-copy	tRNA-dihydrouridinesynthase
Aradu.HVG4L	Araip.Z9C8C	multiple-copy	tRNA-dihydrouridinesynthase-likeprotein
Aradu.ZYI4E	Araip.ZD7CP	multiple-copy	tRNA-specificadenosinedeaminase
Aradu.D3RAM	Araip.KW6YR	multiple-copy	tRNAwybutosine-synthesizingprotein1homolog[Glycinem
Aradu.K5W59	Araip.7513V	multiple-copy	tRNAwybutosine-synthesizingprotein1homologisoform2[G
Aradu.Z2TGH	Araip.LNC3W	multiple-copy	tRNAwybutosine-synthesizingprotein1homologisoform2[G
Aradu.RQ5UT	Araip.V99DX	multiple-copy	tRNAwybutosine-synthesizingprotein2/3/4-like[Glycinema

Aradu.M3YXV	Araip.MJ6R4	multiple-copy	trypsin-likeserineprotease
Aradu.W4XL4	Araip.R9908	multiple-copy	trypsin-likeserineprotease
Aradu.PIJ3J	Araip.1A0QT	multiple-copy	tryptophansynthasebetachain
Aradu.A336V	Araip.8F6MX	multiple-copy	tryptophansynthasebetachain
Aradu.F4EXD	Araip.K7EKQ	multiple-copy	tryptophansynthasebetachain
Aradu.68CEE	Araip.R3N8M	multiple-copy	tryptophan-tRNAligase
Aradu.L7DZT	Araip.BT19G	multiple-copy	tubbylikeprotein3
Aradu.INV9V	Araip.F5D2P	multiple-copy	tubulinalpha-6chain,putative
Aradu.AG70H	Araip.EL2NI	multiple-copy	tubulinbetachain4
Aradu.YD7VI	Araip.EC30Y	multiple-copy	two-componentresponseregulatorARR2-like[Glycinemax]
Aradu.9DS5H	Araip.M6U8Q	multiple-copy	two-componentresponseregulatorARR2-like[Glycinemax]
Aradu.QNE8S	Araip.XLI8D	multiple-copy	two-componentresponseregulatorARR2-like[Glycinemax]
Aradu.76EJ2	Araip.819C3	multiple-copy	two-componentresponseregulatorARR2-likeisoformX2[Gl
Aradu.YH6P6	Araip.Y2EMI	multiple-copy	two-componentresponseregulatorARR2-likeisoformX2[Gl
Aradu.ER3NJ	Araip.L5CJ5	multiple-copy	two-componentresponseregulator-likeAPRR2-likeisoform
Aradu.DF8UA	Araip.AU0RS	multiple-copy	two-componentresponseregulator-likeAPRR2-likeisoform
Aradu.0SC6H	Araip.VL6YY	multiple-copy	two-componentresponseregulator-likeAPRR2-likeisoform
Aradu.KLU2C	Araip.1M393	multiple-copy	two-componentresponseregulator-likeAPRR2-likeisoform
Aradu.R3EFV	Araip.V7EVF	multiple-copy	Twocomponentsignaltransductionsystemhybridhistidineki
Aradu.R9TKV	Araip.Y8GBE	multiple-copy	typeIinositol1,4,5-trisphosphate5-phosphatase2-like[Glyc
Aradu.00U6W	Araip.A82X5	multiple-copy	typeIinositol1,4,5-trisphosphate5-phosphataseCVP2-like[
Aradu.81LQN	Araip.B861E	multiple-copy	typeIinositol1,4,5-trisphosphate5-phosphataseCVP2-like[
Aradu.1E9H5	Araip.Y83VE	multiple-copy	typeIinositol1,4,5-trisphosphate5-phosphataseCVP2-like[
Aradu.25DC4	Araip.6749X	multiple-copy	typeIinositol1,4,5-trisphosphate5-phosphataseCVP2-like[
Aradu.5M1Q4	Araip.A7TGT	multiple-copy	tyrosinephosphatase1
Aradu.AT0C1	Araip.G1J68	multiple-copy	tyrosyl-DNAphosphodiesterase-related
Aradu.WNH12	Araip.C4L8E	multiple-copy	U11/U12smallnuclearribonucleoprotein25kDaprotein-likei
Aradu.DR03T	Araip.LE75L	multiple-copy	U11/U12smallnuclearribonucleoprotein25kDaprotein-likei
Aradu.DNJ9W	Araip.X44C7	multiple-copy	U3smallnucleolarRNA-associatedprotein6homolog[Glycin
Aradu.SY6IP	Araip.8PW0Z	multiple-copy	ubiquitincarboxyl-terminalhydrolase
Aradu.C8KKZ	Araip.C9LX5	multiple-copy	ubiquitincarboxyl-terminalhydrolase
Aradu.V3HYN	Araip.NC47B	multiple-copy	ubiquitincarboxyl-terminalhydrolase
Aradu.RBF27	Araip.R66IE	multiple-copy	ubiquitincarboxyl-terminalhydrolase
Aradu.VK984	Araip.X1YIB	multiple-copy	ubiquitincarboxyl-terminalhydrolase
Aradu.N3IWP	Araip.9WM3A	multiple-copy	ubiquitincarboxyl-terminalhydrolase12-like[Glycinemax]
Aradu.E710C	Araip.YN270	multiple-copy	ubiquitincarboxyl-terminalhydrolase16-likeisoformX2[Glyc
Aradu.J6I78	Araip.ZK9JP	multiple-copy	ubiquitincarboxyl-terminalhydrolase17-like[Glycinemax]
Aradu.R9VGI	Araip.RDY0K	multiple-copy	ubiquitincarboxyl-terminalhydrolase18-likeisoformX2[Glyc
Aradu.YTJ16	Araip.E9P76	multiple-copy	ubiquitincarboxyl-terminalhydrolasefamilyprotein
Aradu.SXB7Z	Araip.KV8TN	multiple-copy	ubiquitincarboxyl-terminalhydrolase-likeprotein
Aradu.4DR9N	Araip.N7CRY	multiple-copy	ubiquitincarboxyl-terminalhydrolase-likeprotein
Aradu.9J6N2	Araip.6Q5LU	multiple-copy	Ubiquitincarboxyl-terminalhydrolase-relatedprotein
Aradu.MA01V	Araip.L50X7	multiple-copy	ubiquitin-conjugatingenzyme20
Aradu.JBH10	Araip.MP4SQ	multiple-copy	ubiquitin-conjugatingenzyme22

Aradu.G2EQN	Araip.09PBC	multiple-copy	Ubiquitin-conjugatingenzymefamilyprotein
Aradu.MJ2SD	Araip.GR4Q0	multiple-copy	ubiquitin-conjugatingenzyme23
Aradu.LQC6W	Araip.3F2A6	multiple-copy	ubiquitin-conjugatingenzyme27
Aradu.TB877	Araip.GDQ5V	multiple-copy	ubiquitin-conjugatingenzyme28
Aradu.Q49PC	Araip.S7ANW	multiple-copy	ubiquitin-conjugatingenzyme28
Aradu.RBV6Z	Araip.66GXB	multiple-copy	ubiquitin-conjugatingenzyme3
Aradu.GY08R	Araip.72FUN	multiple-copy	ubiquitin-conjugatingenzyme32
Aradu.999EQ	Araip.G7PU7	multiple-copy	ubiquitin-conjugatingenzyme34
Aradu.B3VS2	Araip.K23QX	multiple-copy	ubiquitin-conjugatingenzyme36
Aradu.Q5Q2B	Araip.BI95U	multiple-copy	ubiquitin-conjugatingenzyme5
Aradu.LS42S	Araip.P5I6R	multiple-copy	ubiquitin-conjugatingenzyme5
Aradu.66DLZ	Araip.W7TM3	multiple-copy	Ubiquitin-conjugatingenzymefamilyprotein
Aradu.JKI99	Araip.EZE10	multiple-copy	ubiquitinfamilyprotein
Aradu.QCH8L	Araip.UI2R0	multiple-copy	ubiquitin1,relatedprotein
Aradu.ZVP8C	Araip.ZK7F1	multiple-copy	ubiquitinfusiondegradation1
Aradu.QJ7XC	Araip.HX3C4	multiple-copy	ubiquitinligaseSINAT3
Aradu.CP61L	Araip.LP8FF	multiple-copy	ubiquitinligaseSINAT3
Aradu.PIA7H	Araip.FUS30	multiple-copy	Ubiquitin-likesuperfamilyprotein
Aradu.DDG3L	Araip.UD0YL	multiple-copy	Ubiquitin-proteinligase,PUB52n=1Tax=Selaginellamoelle
Aradu.8ZT7E	Araip.60Y07	multiple-copy	Ubiquitin-proteinligase,PUB59n=2Tax=Selaginellamoelle
Aradu.NS08D	Araip.D6MSX	multiple-copy	ubiquitin-proteinligase,putative
Aradu.QS26M	Araip.D5S7B	multiple-copy	ubiquitin-proteinligase4
Aradu.X0CJD	Araip.N3ZNP	multiple-copy	ubiquitin-proteinligase4
Aradu.50FPJ	Araip.Q3GAG	multiple-copy	ubiquitinproteinligase5
Aradu.U2UEV	Araip.65HZ4	multiple-copy	ubiquitinproteinligase6
Aradu.M18SC	Araip.V4I5J	multiple-copy	UbiquitinsystemcomponentCueprotein
Aradu.55W9R	Araip.VL132	multiple-copy	UbiquitinsystemcomponentCueprotein
Aradu.FB522	Araip.ZL723	multiple-copy	UbiquitinsystemcomponentCueprotein
Aradu.BDH44	Araip.658MF	multiple-copy	U-boxdomain-containingprotein13-like[Glycinemax]
Aradu.A2VXS	Araip.PM53A	multiple-copy	U-boxdomain-containingprotein14-like[Glycinemax]
Aradu.3N314	Araip.P55C3	multiple-copy	U-boxdomain-containingprotein17-like[Glycinemax]
Aradu.VI8FD	Araip.4CP0D	multiple-copy	U-boxdomain-containingprotein21-like[Glycinemax]
Aradu.AZ3DS	Araip.W9EY0	multiple-copy	U-boxdomain-containingprotein30-like[Glycinemax]
Aradu.M2A53	Araip.8VG3L	multiple-copy	U-boxdomain-containingprotein35-like[Glycinemax]
Aradu.Y8Q46	Araip.A6Q3K	multiple-copy	U-boxdomain-containingprotein33-like[Glycinemax]
Aradu.VQ2JX	Araip.E13P0	multiple-copy	U-boxdomain-containingprotein3-likeisoformX3[Glycinem
Aradu.QZS0Y	Araip.ATZ24	multiple-copy	U-boxdomain-containingprotein4[Glycinemax]
Aradu.U8C21	Araip.TWG69	multiple-copy	U-boxdomain-containingprotein4[Glycinemax]
Aradu.HX9PW	Araip.MLC5R	multiple-copy	U-boxdomain-containingprotein43-likeisoformX2[Glycine
Aradu.Y0VZV	Araip.LCG1X	multiple-copy	U-boxdomain-containingprotein44-likeisoformX1[Glycine
Aradu.JC4ID	Araip.C58L0	multiple-copy	U-boxdomain-containingprotein4-like[Glycinemax]
Aradu.15MGK	Araip.G1GMX	multiple-copy	U-boxdomain-containingprotein4-like[Glycinemax]
Aradu.94ETY	Araip.K9U7W	multiple-copy	U-boxdomain-containingprotein4-like[Glycinemax]
Aradu.Q1S4K	Araip.P1GI9	multiple-copy	U-boxdomain-containingprotein4-like[Glycinemax]

Aradu.1J279	Araip.VAZ0T	multiple-copy	U-boxdomain-containingprotein4-like[Glycinemax]
Aradu.FRY2U	Araip.Y25J6	multiple-copy	U-boxdomain-containingprotein4-like[Glycinemax]
Aradu.CTQ6E	Araip.G18BD	multiple-copy	U-boxdomain-containingprotein5-like[Glycinemax]
Aradu.E36HP	Araip.9T4E8	multiple-copy	U-boxdomain-containingprotein5-likeisoformX2[Glycinem
Aradu.B7QXE	Araip.9P3RH	multiple-copy	U-boxdomain-containingprotein62-likeisoformX1[Glyciner
Aradu.U5Z1W	Araip.W00GH	multiple-copy	U-boxdomain-containingprotein6-like[Glycinemax]
Aradu.1FD3D	Araip.V9RIN	multiple-copy	U-boxdomain-containingprotein7-like[Glycinemax]
Aradu.BGJ4E	Araip.YN1XP	multiple-copy	U-boxdomain-containingprotein4-like[Glycinemax]
Aradu.U55D3	Araip.M6EG0	multiple-copy	UBXdomain-containingprotein
Aradu.G4U2S	Araip.UU2Q4	multiple-copy	UBXdomain-containingprotein
Aradu.W7JB7	Araip.2SG63	multiple-copy	UDP-D-glucose/UDP-D-galactose4-epimerase5
Aradu.7W9JR	Araip.EV8CZ	multiple-copy	UDP-D-glucuronate4-epimerase3
Aradu.AR3UR	Araip.6K53R	multiple-copy	UDP-D-glucuronate4-epimerase6
Aradu.T8M8U	Araip.YFS36	multiple-copy	UDP-galactosetransporter5
Aradu.2Q2R1	Araip.FD3D0	multiple-copy	UDP-glucose6-dehydrogenasefamilyprotein
Aradu.NF6XD	Araip.CQ7YT	multiple-copy	UDP-glucuronate:xylanalpha-glucuronosyltransferase2-lil
Aradu.H0BW9	Araip.E7PK4	multiple-copy	UDP-glucuronate:xylanalpha-glucuronosyltransferase2-lil
Aradu.0KH4T	Araip.65II6	multiple-copy	UDP-glucuronate:xylanalpha-glucuronosyltransferase3-lil
Aradu.K2SIM	Araip.W9TLJ	multiple-copy	UDP-glucuronate:xylanalpha-glucuronosyltransferase4-lil
Aradu.W3UAF	Araip.UGL36	multiple-copy	UDP-glucuronicaciddecarboxylase1
Aradu.X23MZ	Araip.QUX0P	multiple-copy	UDP-glucuronicaciddecarboxylase6-like[Glycinemax]
Aradu.PXM5A	Araip.IF23W	multiple-copy	UDP-glycosyltransferase74F1
Aradu.RE1JT	Araip.0P3QP	multiple-copy	UDP-Glycosyltransferasesuperfamilyprotein
Aradu.C0GKW	Araip.37ZE6	multiple-copy	UDP-Glycosyltransferasesuperfamilyprotein
Aradu.TF4C3	Araip.79KSY	multiple-copy	UDP-Glycosyltransferasesuperfamilyprotein
Aradu.UI9VM	Araip.7QY88	multiple-copy	UDP-Glycosyltransferasesuperfamilyprotein
Aradu.L7WWU	Araip.91EFH	multiple-copy	UDP-Glycosyltransferasesuperfamilyprotein
Aradu.MD0ZW	Araip.9E6PV	multiple-copy	UDP-Glycosyltransferasesuperfamilyprotein
Aradu.YC6XN	Araip.AMY99	multiple-copy	UDP-Glycosyltransferasesuperfamilyprotein
Aradu.J5AII	Araip.B6VA3	multiple-copy	UDP-Glycosyltransferasesuperfamilyprotein
Aradu.P8Y29	Araip.C4A9H	multiple-copy	UDP-Glycosyltransferasesuperfamilyprotein
Aradu.Z7FZQ	Araip.CSX4W	multiple-copy	UDP-Glycosyltransferasesuperfamilyprotein
Aradu.V32T3	Araip.D5YYK	multiple-copy	UDP-Glycosyltransferasesuperfamilyprotein
Aradu.D24R3	Araip.EK557	multiple-copy	UDP-Glycosyltransferasesuperfamilyprotein
Aradu.5ZT49	Araip.EK9PX	multiple-copy	UDP-Glycosyltransferasesuperfamilyprotein
Aradu.EZK5X	Araip.EMY47	multiple-copy	UDP-Glycosyltransferasesuperfamilyprotein
Aradu.TH1E7	Araip.EQA8W	multiple-copy	UDP-Glycosyltransferasesuperfamilyprotein
Aradu.070N1	Araip.EX0TX	multiple-copy	UDP-Glycosyltransferasesuperfamilyprotein
Aradu.24BEK	Araip.G37T0	multiple-copy	UDP-Glycosyltransferasesuperfamilyprotein
Aradu.Y5G1G	Araip.GP5MB	multiple-copy	UDP-Glycosyltransferasesuperfamilyprotein
Aradu.K93AE	Araip.H41HP	multiple-copy	UDP-Glycosyltransferasesuperfamilyprotein
Aradu.SBB2J	Araip.IE5FR	multiple-copy	UDP-Glycosyltransferasesuperfamilyprotein
Aradu.AM65N	Araip.IY8RA	multiple-copy	UDP-Glycosyltransferasesuperfamilyprotein
Aradu.JG94E	Araip.JN8FM	multiple-copy	UDP-Glycosyltransferasesuperfamilyprotein

Aradu.N89Q4	Araip.KW3Z9	multiple-copy	UDP-Glycosyltransferasesuperfamilyprotein
Aradu.LF5EZ	Araip.LTD41	multiple-copy	UDP-Glycosyltransferasesuperfamilyprotein
Aradu.WM4V4	Araip.M83DH	multiple-copy	UDP-Glycosyltransferasesuperfamilyprotein
Aradu.MX9DT	Araip.SH80B	multiple-copy	UDP-Glycosyltransferasesuperfamilyprotein
Aradu.A1MN7	Araip.UA25L	multiple-copy	UDP-Glycosyltransferasesuperfamilyprotein
Aradu.JFD76	Araip.V2JWX	multiple-copy	UDP-Glycosyltransferasesuperfamilyprotein
Aradu.V5FRM	Araip.X4RTI	multiple-copy	UDP-Glycosyltransferasesuperfamilyprotein
Aradu.598J8	Araip.XL1Z1	multiple-copy	UDP-Glycosyltransferasesuperfamilyprotein
Aradu.9Y73E	Araip.Y8SSF	multiple-copy	UDP-Glycosyltransferasesuperfamilyprotein
Aradu.Z8K87	Araip.YN5F9	multiple-copy	UDP-Glycosyltransferasesuperfamilyprotein
Aradu.GM5S2	Araip.ZZI8I	multiple-copy	UDP-Glycosyltransferasesuperfamilyprotein
Aradu.E9IFL	Araip.KL3B6	multiple-copy	UDP-sulfoquinovosesynthase
Aradu.K7STL	Araip.6T61F	multiple-copy	Ulp1proteasefamily,carboxy-terminaldomainprotein
Aradu.114L5	Araip.C8BSP	multiple-copy	Ulp1proteasefamily,carboxy-terminaldomainprotein
Aradu.8T892	Araip.JLC9M	multiple-copy	Ulp1proteasefamily,carboxy-terminaldomainprotein
Aradu.BBW0R	Araip.LQ7SX	multiple-copy	Ulp1proteasefamily,carboxy-terminaldomainprotein
Aradu.I03VB	Araip.N6CRE	multiple-copy	Ulp1proteasefamilyC-terminalcatalyticdomaincontainingp
Aradu.4KE9F	Araip.PRY9G	multiple-copy	Ulp1proteasefamily,carboxy-terminaldomainprotein
Aradu.A2UTQ	Araip.W5PYV	multiple-copy	Ulp1proteasefamily,carboxy-terminaldomainprotein
Aradu.TE7Z6	Araip.YBU7P	multiple-copy	Ulp1proteasefamily,carboxy-terminaldomainprotein
Aradu.Z9WRN	Araip.9D138	multiple-copy	UNC93-likeprotein1-like[Glycinemax]
Aradu.FJ441	Araip.86J2T	multiple-copy	UNE12-like[Glycinemax]
Aradu.I38Z6	Araip.XTW2R	multiple-copy	UPF0415proteinC7orf25homologisoformX2[Glycinemax]
Aradu.BK33F	Araip.XIH72	multiple-copy	UPF0420proteinC16orf58homolog[Glycinemax]
Aradu.8B0HV	Araip.NH7YS	multiple-copy	UPF0451C17orf61-likeprotein
Aradu.LNC81	Araip.NPK9J	multiple-copy	UPF0481protein[Glycinemax]
Aradu.JMT41	Araip.RLC71	multiple-copy	UPF0481protein[Glycinemax]
Aradu.5TE5X	Araip.U7K5S	multiple-copy	UPF0481protein[Glycinemax]
Aradu.0ZG2X	Araip.52WME	multiple-copy	UPF0481proteinAt3g47200-like[Glycinemax]
Aradu.V2BW0	Araip.6TJ7D	multiple-copy	UPF0481proteinAt3g47200-like[Glycinemax]
Aradu.TC1A2	Araip.D4JH9	multiple-copy	UPF0481protein[Glycinemax]
Aradu.10I3J	Araip.M15N8	multiple-copy	UPF0481proteinAt3g47200-like[Glycinemax]
Aradu.BD2SQ	Araip.PT961	multiple-copy	UPF0481proteinAt3g47200-like[Glycinemax]
Aradu.27Y2W	Araip.71FLX	multiple-copy	UPF0496proteinAt1g20180-like[Glycinemax]
Aradu.ZK7ZU	Araip.U324B	multiple-copy	UPF0678fattyacid-bindingprotein-likeprotein
Aradu.RWZ7G	Araip.8C9BN	multiple-copy	UPSTREAMOFFLC-likeisoformX2[Glycinemax]
Aradu.0514U	Araip.541ME	multiple-copy	UPSTREAMOFFLC-likeisoformX3[Glycinemax]
Aradu.4PC58	Araip.Q8T53	multiple-copy	UPSTREAMOFFLC-likeisoformX3[Glycinemax]
Aradu.JY8JV	Araip.RP235	multiple-copy	uridinekinase-like2
Aradu.5S2PY	Araip.3630D	multiple-copy	uridinekinase-like3
Aradu.C6SJS	Araip.DP7XY	multiple-copy	vacuolar(H+)-ATPaseGsubunit
Aradu.L5GQQ	Araip.LA7GW	multiple-copy	vacuolar(H+)-ATPaseGsubunit
Aradu.EJW3I	Araip.A36V0	multiple-copy	vacuolarATPsynthasecatalyticsubunit-related/V-ATPase-
Aradu.PJ77P	Araip.V26H1	multiple-copy	vacuolarH+-translocatinginorganicpyrophosphatase

Aradu.JR9SL	Araip.XJP9J	multiple-copy	vacuolarH+-translocatinginorganicpyrophosphatase
Aradu.VT0DP	Araip.J9D4H	multiple-copy	Vacuolarimport/degradation,Vid27-relatedprotein
Aradu.K0L5G	Araip.PN2Q6	multiple-copy	vacuolarmembraneproteinYML018C-likeisoformX1[Glycin
Aradu.Q17ZW	Araip.2L5W7	multiple-copy	vacuolarmembraneproteinYML018C-likeisoformX2[Glycin
Aradu.Z8A7E	Araip.73UW5	multiple-copy	vacuolarproteinsorting11
Aradu.X1DZR	Araip.4R2IW	multiple-copy	vacuolarproteinsorting26B
Aradu.0G8DQ	Araip.TSB39	multiple-copy	vacuolarproteinsorting45
Aradu.HAL7W	Araip.L9BTW	multiple-copy	Vacuolarproteinsorting55(VPS55)familyprotein
Aradu.97VNG	Araip.X0AN4	multiple-copy	Vacuolarproteinsorting55(VPS55)familyprotein
Aradu.89XIU	Araip.D20VZ	multiple-copy	vacuolarproteinsorting-associatedprotein20.2
Aradu.I5672	Araip.8K74T	multiple-copy	vacuolarproteinsorting-associatedprotein27-likeisoformX1
Aradu.R8T0J	Araip.13XDD	multiple-copy	vacuolarproteinsorting-associatedprotein28homolog1
Aradu.425TL	Araip.12XQ6	multiple-copy	vacuolarproteinsorting-associatedprotein4-like[Glycinema
Aradu.70QGC	Araip.6E7KQ	multiple-copy	vacuolarproteinsorting-associatedprotein4-like[Glycinema
Aradu.4HV8T	Araip.M0K3R	multiple-copy	vacuolarproteinsorting-associatedproteinVTA1homolog[G
Aradu.X3R2Q	Araip.SX5JP	multiple-copy	vacuolarproteinsortingprotein,putative
Aradu.JPJ0M	Araip.H3L98	multiple-copy	Vacuolarprotein-sortingproteinbro1n=4Tax=Aspergillacea
Aradu.6LS7N	Araip.CPP3Y	multiple-copy	vacuolarsorting-associated-likeprotein
Aradu.1DV9M	Araip.1G7Y9	multiple-copy	vacuolarsortingproteinSNF8
Aradu.PJM2P	Araip.L434T	multiple-copy	vesicle-associatedmembraneprotein713
Aradu.MQ1LE	Araip.X04IN	multiple-copy	vesicle-associatedmembraneprotein714
Aradu.V72BH	Araip.0BL8B	multiple-copy	vesicle-associatedmembraneprotein725
Aradu.3XT84	Araip.5735B	multiple-copy	vesicle-associatedmembraneprotein725
Aradu.Q6WYU	Araip.5U4XM	multiple-copy	vesicle-associatedmembraneprotein726
Aradu.LW83G	Araip.15UQN	multiple-copy	vesicleassociatedprotein
Aradu.M4GP4	Araip.L92QM	multiple-copy	vesicle-associatedprotein2-2-likeisoformX2[Glycinemax]
Aradu.WB83L	Araip.J0SL5	multiple-copy	vesicletransportproteinSFT2B[Glycinemax]
Aradu.75D3M	Araip.YJ8QA	multiple-copy	violaxanthinde-epoxidase-related
Aradu.U1I3J	Araip.IUW8W	multiple-copy	viralmovementprotein
Aradu.A1XBQ	Araip.712IZ	multiple-copy	VOZ1-likeisoformX3[Glycinemax]
Aradu.JMV7E	Araip.P7ZIQ	multiple-copy	V-typeprotonATPase16kDaproteolipidsubunit-like[Glycine
Aradu.9T7BM	Araip.2EA83	multiple-copy	V-typeprotonATPasesubunitB1-likeisoformX2[Glycinemax
Aradu.H0NY1	Araip.EAG6M	multiple-copy	V-typeprotonATPasesubunitD-like[Glycinemax]
Aradu.GJ5P4	Araip.P31AE	multiple-copy	V-typeprotonATPasesubunitE2-likeisoformX1[Glycinemax
Aradu.J5HSK	Araip.WKJ1H	multiple-copy	V-typeprotonATPasesubunitE-likeisoformX1[Glycinemax]
Aradu.JV2C7	Araip.CP6YA	multiple-copy	V-typeprotonATPasesubunitF-like[Glycinemax]
Aradu.52L7X	Araip.9JY32	multiple-copy	wall-associatedreceptorkinase3-like[Glycinemax]
Aradu.G8EKB	Araip.BM4V8	multiple-copy	wall-associatedreceptorkinasegalacturonan-bindingprotei
Aradu.X7WGL	Araip.9H56X	multiple-copy	wall-associatedreceptorkinase-like15-like[Glycinemax]
Aradu.02NZN	Araip.F8JDN	multiple-copy	wall-associatedreceptorkinase-like15-like[Glycinemax]
Aradu.GC1Z5	Araip.Q58BJ	multiple-copy	wall-associatedreceptorkinase-like15-like[Glycinemax]
Aradu.Q9ZWS	Araip.XM7CZ	multiple-copy	wall-associatedreceptorkinase-like15-like[Glycinemax]
Aradu.R0KJF	Araip.6ZS67	multiple-copy	Water-selectivetransportintrinsicmembraneprotein1n=1Ta
Aradu.DWL7L	Araip.LJX8Z	multiple-copy	Water-selectivetransportintrinsicmembraneprotein1n=1Ta

Aradu.7MF1E	Araip.U5J78	multiple-copy	Water-selectivetransportintrinsicmembraneprotein1n=1Ta
Aradu.901R7	Araip.XJU6V	multiple-copy	Water-selectivetransportintrinsicmembraneprotein1n=1Ta
Aradu.5NS8M	Araip.G8PU5	multiple-copy	WD-40repeatfamilyprotein
Aradu.IM5S3	Araip.0FB59	multiple-copy	WD-40repeatprotein
Aradu.DI7YA	Araip.0TB3B	multiple-copy	WD-40repeatprotein
Aradu.7XA36	Araip.0V0EF	multiple-copy	WD-40repeatprotein
Aradu.4U7AL	Araip.3JS6L	multiple-copy	WD-40repeatprotein
Aradu.S2VPA	Araip.4B8IV	multiple-copy	WD-40repeatprotein
Aradu.TXN9A	Araip.7PD3K	multiple-copy	WD-40repeatprotein
Aradu.1VB35	Araip.F3XKF	multiple-copy	WD-40repeatprotein
Aradu.I01VW	Araip.H55D8	multiple-copy	WD-40repeatprotein
Aradu.7LZ09	Araip.HA6TQ	multiple-copy	WD-40repeatprotein
Aradu.HXK3I	Araip.J3620	multiple-copy	WD-40repeatprotein
Aradu.QH2VI	Araip.KJS5P	multiple-copy	WD-40repeatprotein
Aradu.E6FD3	Araip.L9EW3	multiple-copy	WD-40repeatprotein
Aradu.SP99D	Araip.S7CAX	multiple-copy	WD-40repeatprotein
Aradu.0IN20	Araip.VP2HF	multiple-copy	WD-40repeatprotein
Aradu.00WD1	Araip.WI73I	multiple-copy	WD-40repeatprotein
Aradu.3VD4A	Araip.XDK4R	multiple-copy	WD-40repeatprotein
Aradu.TR8YP	Araip.Y74Z9	multiple-copy	WD-40repeatprotein
Aradu.8QL4Z	Araip.ZW9IZ	multiple-copy	WD-40repeatprotein
Aradu.7DV9M	Araip.V99IN	multiple-copy	WD-40repeatprotein
Aradu.KYE4X	Araip.E0FPV	multiple-copy	WD-repeatcellcycleregulatoryprotein[Glycinemax]
Aradu.FW3IU	Araip.L8NZD	multiple-copy	WD-repeatcellcycleregulatoryprotein[Glycinemax]
Aradu.8LY9S	Araip.LYD5Q	multiple-copy	WD-repeatcellcycleregulatoryprotein[Glycinemax]
Aradu.DA6I6	Araip.IT6Z9	multiple-copy	WDrepeat-containingprotein3-like[Glycinemax]
Aradu.BIW4R	Araip.L9809	multiple-copy	WDrepeat-containingprotein3-like[Glycinemax]
Aradu.KUP4F	Araip.1L67X	multiple-copy	WDrepeat-containingprotein3-likeisoformX1[Glycinemax]
Aradu.X2T4X	Araip.R4XYF	multiple-copy	WDrepeat-containingprotein3-likeisoformX1[Glycinemax]
Aradu.MV1EC	Araip.5G9GQ	multiple-copy	WDrepeat-containingprotein3-likeisoformX1[Glycinemax]
Aradu.R2B96	Araip.JS7IQ	multiple-copy	WDrepeat-containingprotein3-likeisoformX1[Glycinemax]
Aradu.10YFJ	Araip.256VQ	multiple-copy	WDrepeat-containingprotein44-like[Glycinemax]
Aradu.Q9YKL	Araip.AJD1E	multiple-copy	Transducin/WD40repeat-likesuperfamilyprotein
Aradu.JL8FJ	Araip.DGW0S	multiple-copy	WDrepeat-containingprotein44-like[Glycinemax]
Aradu.C4M03	Araip.0448C	multiple-copy	WDrepeat-containingprotein5-like[Glycinemax]
Aradu.ZTY0U	Araip.1T581	multiple-copy	WDrepeat-containingprotein5-like[Glycinemax]
Aradu.5BX1F	Araip.53MRH	multiple-copy	WDrepeat-containingprotein5-like[Glycinemax]
Aradu.3MG08	Araip.B49UZ	multiple-copy	WDrepeat-containingprotein5-like[Glycinemax]
Aradu.EAT9T	Araip.G992G	multiple-copy	WDrepeat-containingprotein5-like[Glycinemax]
Aradu.8EU70	Araip.IUR9I	multiple-copy	WDrepeat-containingprotein5-like[Glycinemax]
Aradu.IM4U5	Araip.KGP2Q	multiple-copy	WDrepeat-containingprotein5-like[Glycinemax]
Aradu.B5E0M	Araip.N4R7Y	multiple-copy	WDrepeat-containingprotein5-like[Glycinemax]
Aradu.H56YA	Araip.VE6EG	multiple-copy	WDrepeat-containingprotein3-likeisoformX1[Glycinemax]
Aradu.EFI11	Araip.W6QDR	multiple-copy	WDrepeat-containingprotein61-likeisoform1[Glycinemax]

Aradu.7WF2V	Araip.ZQ3CB	multiple-copy	Transducin/WD40repeat-likesuperfamilyprotein
Aradu.V8FCP	Araip.XV3D7	multiple-copy	WDrepeat-containingprotein61-like[Glycinemax]
Aradu.2IP37	Araip.KWB60	multiple-copy	WDrepeat-containingprotein61-likeisoform1[Glycinemax]
Aradu.T3LBA	Araip.H2PQ2	multiple-copy	Transducin/WD40repeat-likesuperfamilyprotein
Aradu.FA8DG	Araip.MF1KR	multiple-copy	WEAKmovementUNDERBLUELIGHT-likeprotein
Aradu.L6IIL	Araip.Q7D64	multiple-copy	WEBfamilyproteinAt1g12150-like[Glycinemax]
Aradu.50AY2	Araip.7E6JE	multiple-copy	WEBfamilyproteinAt1g75720-likeisoformX1[Glycinemax]
Aradu.0WU0I	Araip.AF9FL	multiple-copy	WEBfamilyproteinAt1g75720-likeisoformX1[Glycinemax]
Aradu.YQ4IR	Araip.B0NA7	multiple-copy	WEBfamilyproteinAt1g75720-likeisoformX2[Glycinemax]
Aradu.QS8M2	Araip.M8E98	multiple-copy	WEBfamilyproteinAt2g38370-like[Glycinemax]
Aradu.1SD0Q	Araip.V1BCZ	multiple-copy	WEBfamilyproteinAt2g38370-like[Glycinemax]
Aradu.320AL	Araip.I1KV0	multiple-copy	WEBfamilyproteinAt5g16730,chloroplastic-like[Glycinema
Aradu.2YF2R	Araip.BV8FB	multiple-copy	winged-helixDNA-bindingtranscriptionfactorfamilyprotein
Aradu.S9LIL	Araip.1Q3SQ	multiple-copy	wound-inducedprotein1-like[Glycinemax]
Aradu.JV441	Araip.1Y87C	multiple-copy	Wound-responsivefamilyprotein
Aradu.CMH1Z	Araip.5G3Z8	multiple-copy	WPPdomain-associatedprotein-like[Glycinemax]
Aradu.1WP35	Araip.Y32G0	multiple-copy	WPPdomaininteractingprotein,putative
Aradu.D7B0D	Araip.0QI46	multiple-copy	WPPdomain-interactingtail-anchoredprotein1-likeisoform
Aradu.G07IW	Araip.PRB9P	multiple-copy	WPPdomain-interactingtail-anchoredprotein2-likeisoform
Aradu.M1GCR	Araip.15EJY	multiple-copy	WRKYfamilytranscriptionfactor
Aradu.KG41H	Araip.1E1WQ	multiple-copy	WRKYfamilytranscriptionfactor
Aradu.KD2FK	Araip.20FU5	multiple-copy	WRKYfamilytranscriptionfactor
Aradu.A205B	Araip.3A1VT	multiple-copy	WRKYfamilytranscriptionfactor
Aradu.BB4JP	Araip.4L5AH	multiple-copy	WRKYfamilytranscriptionfactor
Aradu.VSA8A	Araip.5X628	multiple-copy	WRKYfamilytranscriptionfactor
Aradu.93TCK	Araip.60WGZ	multiple-copy	WRKYfamilytranscriptionfactor
Aradu.2V4EQ	Araip.AR7FU	multiple-copy	WRKYfamilytranscriptionfactor
Aradu.I8GKQ	Araip.B07N1	multiple-copy	WRKYfamilytranscriptionfactor
Aradu.B1C6F	Araip.D0ST6	multiple-copy	WRKYfamilytranscriptionfactor
Aradu.PW6QG	Araip.E23GM	multiple-copy	WRKYfamilytranscriptionfactor
Aradu.T9MSY	Araip.G2UJA	multiple-copy	WRKYfamilytranscriptionfactor
Aradu.U1JXN	Araip.HGN3R	multiple-copy	WRKYfamilytranscriptionfactor
Aradu.VL3IZ	Araip.KXH96	multiple-copy	WRKYfamilytranscriptionfactor
Aradu.PK283	Araip.M90ED	multiple-copy	WRKYfamilytranscriptionfactor
Aradu.CDI4M	Araip.N4R5C	multiple-copy	WRKYfamilytranscriptionfactor
Aradu.C8ZMR	Araip.NX7E8	multiple-copy	WRKYfamilytranscriptionfactorfamilyprotein
Aradu.NH44H	Araip.PY0W6	multiple-copy	WRKYfamilytranscriptionfactor
Aradu.20TYF	Araip.V76CX	multiple-copy	WRKYfamilytranscriptionfactor
Aradu.C90G3	Araip.WEZ1Z	multiple-copy	WRKYfamilytranscriptionfactor
Aradu.VE705	Araip.WHJ14	multiple-copy	WRKYfamilytranscriptionfactor
Aradu.LAA50	Araip.X9JQZ	multiple-copy	WRKYfamilytranscriptionfactor
Aradu.R83G6	Araip.2412K	multiple-copy	WRKYfamilytranscriptionfactorfamilyprotein
Aradu.TF48T	Araip.4KW8X	multiple-copy	WRKYfamilytranscriptionfactorfamilyprotein
Aradu.WLB92	Araip.G0Q9N	multiple-copy	WRKYfamilytranscriptionfactorfamilyprotein

Aradu.CST5R	Araip.MAR0Q	multiple-copy	WRKYfamilytranscriptionfactorfamilyprotein
Aradu.AW21H	Araip.NCA4A	multiple-copy	WRKYfamilytranscriptionfactorfamilyprotein
Aradu.76148	Araip.R3I3B	multiple-copy	WRKYfamilytranscriptionfactorfamilyprotein
Aradu.S7YD6	Araip.RC4R7	multiple-copy	WRKYfamilytranscriptionfactorfamilyprotein
Aradu.CW4J0	Araip.X395U	multiple-copy	WRKYfamilytranscriptionfactorfamilyprotein
Aradu.NTD67	Araip.X4GRJ	multiple-copy	WRKYfamilytranscriptionfactorfamilyprotein
Aradu.2S5CB	Araip.UGG4B	multiple-copy	WRKYtranscriptionfactor33[Glycinemax]
Aradu.Z5BX3	Araip.WH7IR	multiple-copy	WRKYtranscriptionfactor48[Glycinemax]
Aradu.S9TS8	Araip.RQ3D1	multiple-copy	WRKYtranscriptionfactor49-like[Glycinemax]
Aradu.SXL5T	Araip.SJY96	multiple-copy	WUSCHELrelatedhomeobox1
Aradu.VDW04	Araip.54YKW	multiple-copy	WUSCHELrelatedhomeobox2
Aradu.YN99B	Araip.F59A9	multiple-copy	WUSCHELrelatedhomeobox5
Aradu.3BA5T	Araip.8BB4D	multiple-copy	WUSCHELrelatedhomeobox8
Aradu.YQU8F	Araip.R870C	multiple-copy	XRI1-likeisoformX2[Glycinemax]
Aradu.54URP	Araip.3F9W2	multiple-copy	xyloglucanendotransglucosylase/hydrolase16
Aradu.27SYD	Araip.WY1NK	multiple-copy	Xyloglucanendotransglucosylase/hydrolasefamilyprotein
Aradu.HRR2H	Araip.JZJ9I	multiple-copy	xyloglucanendotransglucosylase/hydrolase26
Aradu.RA8CW	Araip.LX6XT	multiple-copy	xyloglucanendotransglucosylase/hydrolase26
Aradu.Y1TID	Araip.66N5R	multiple-copy	xyloglucanendotransglucosylase/hydrolase32
Aradu.YW5TG	Araip.C7AJI	multiple-copy	xyloglucanendotransglucosylase/hydrolase4
Aradu.AA5UH	Araip.2H071	multiple-copy	xyloglucanendotransglucosylase/hydrolase5
Aradu.EV762	Araip.7JN11	multiple-copy	xyloglucanendotransglucosylase/hydrolase8
Aradu.44Z6R	Araip.DTK89	multiple-copy	xyloglucanglycosyltransferase4-like[Glycinemax]
Aradu.P6GL4	Araip.6IN8N	multiple-copy	xyloglucanglycosyltransferase5-like[Glycinemax]
Aradu.B5DUD	Araip.9N4E5	multiple-copy	YABBYtranscriptionfactor
Aradu.N1NLT	Araip.6FE6S	multiple-copy	YLS7-like[Glycinemax]
Aradu.X3XXG	Araip.76HFA	multiple-copy	YLS7-like[Glycinemax]
Aradu.MIZ18	Araip.7QP8H	multiple-copy	YLS7-like[Glycinemax]
Aradu.T8IUY	Araip.AHX0C	multiple-copy	YLS7-like[Glycinemax]
Aradu.94WDB	Araip.D9NIG	multiple-copy	YLS7-like[Glycinemax]
Aradu.W98YX	Araip.F66CA	multiple-copy	YLS7-like[Glycinemax]
Aradu.P3XDF	Araip.GH9X1	multiple-copy	YLS7-like[Glycinemax]
Aradu.UD253	Araip.LRH88	multiple-copy	YLS7-like[Glycinemax]
Aradu.Q9UFT	Araip.LTC7U	multiple-copy	YLS7-like[Glycinemax]
Aradu.298AN	Araip.PYR7K	multiple-copy	YLS7-like[Glycinemax]
Aradu.1T3XU	Araip.R06WH	multiple-copy	YLS7-like[Glycinemax]
Aradu.NHB6E	Araip.SK1X8	multiple-copy	YLS7-like[Glycinemax]
Aradu.95XXR	Araip.SR040	multiple-copy	YLS7-like[Glycinemax]
Aradu.84U8V	Araip.WNH0S	multiple-copy	YLS7-like[Glycinemax]
Aradu.EI8LI	Araip.X7A0B	multiple-copy	YLS7-like[Glycinemax]
Aradu.9VB4E	Araip.Y3NGW	multiple-copy	YLS7-like[Glycinemax]
Aradu.F4UZI	Araip.YL5Y5	multiple-copy	YLS7-like[Glycinemax]
Aradu.B6CZV	Araip.Z8DH4	multiple-copy	YLS7-like[Glycinemax]
Aradu.54T4X	Araip.ZYB37	multiple-copy	YLS7-like[Glycinemax]

Aradu.KR62J	Araip.KT1QG	multiple-copy	YLS9[Glycinemax]
Aradu.36PEF	Araip.NA12S	multiple-copy	YLS9[Glycinemax]
Aradu.N1KEX	Araip.XD82V	multiple-copy	YLS9-like[Glycinemax]
Aradu.M6LMC	Araip.KD7KV	multiple-copy	zinc-bindingalcoholdehydrogenasefamilyprotein
Aradu.X1MH8	Araip.R4JRM	multiple-copy	zinc-bindingalcoholdehydrogenasefamilyprotein
Aradu.SCR9A	Araip.RHZ7C	multiple-copy	zinc-bindingalcoholdehydrogenasefamilyprotein
Aradu.XWZ7Z	Araip.P6LSC	multiple-copy	Zinc-bindingintegralperoxisomalmembraneproteinPEX12
Aradu.16U3K	Araip.RP87W	multiple-copy	zincfinger(C2H2type)familyprotein
Aradu.YMP6T	Araip.111QN	multiple-copy	zincfinger(C3HC4-typeRINGfinger)familyprotein
Aradu.AL954	Araip.8H28J	multiple-copy	Zincfinger(C3HC4-typeRINGfinger)familyprotein
Aradu.G6N6R	Araip.DP3JP	multiple-copy	Zincfinger(C3HC4-typeRINGfinger)familyprotein
Aradu.TJ219	Araip.K4YB2	multiple-copy	Zincfinger(C3HC4-typeRINGfinger)familyprotein
Aradu.553J0	Araip.NE3HD	multiple-copy	Zincfinger(C3HC4-typeRINGfinger)familyprotein
Aradu.5B3LS	Araip.NFH73	multiple-copy	Zincfinger(C3HC4-typeRINGfinger)familyprotein
Aradu.1TB02	Araip.SVX3P	multiple-copy	Zincfinger(C3HC4-typeRINGfinger)familyprotein
Aradu.93KPX	Araip.V6JZU	multiple-copy	zincfinger(C3HC4-typeRINGfinger)familyprotein
Aradu.8LR63	Araip.JH5QN	multiple-copy	zincfinger(CCCH-type)familyprotein
Aradu.L3NZR	Araip.7869H	multiple-copy	zincfinger(Ran-binding)familyprotein
Aradu.MCA4R	Araip.E58R7	multiple-copy	zincfinger(Ran-binding)familyprotein
Aradu.JK14X	Araip.F7XDB	multiple-copy	zincfinger(Ran-binding)familyprotein
Aradu.934D7	Araip.T3DX1	multiple-copy	zincfinger(Ran-binding)familyprotein
Aradu.Y2BEF	Araip.2L0M6	multiple-copy	zincfinger,C3HC4type(RINGfinger)protein
Aradu.I9X8G	Araip.KTN2B	multiple-copy	zincfinger,C3HC4type(RINGfinger)protein
Aradu.M0KDX	Araip.F83BZ	multiple-copy	zincfinger,C3HC4type(RINGfinger)protein,putative
Aradu.ZP7JG	Araip.4R6C8	multiple-copy	zincfingerA20andAN1domainstress-associatedprotein
Aradu.GL6NL	Araip.K8PTD	multiple-copy	zincfingerA20andAN1domainstress-associatedprotein
Aradu.C6W6J	Araip.YJ4K8	multiple-copy	zincfingerA20andAN1domainstress-associatedprotein
Aradu.2ZT9L	Araip.ID1ZZ	multiple-copy	zincfingerCCCHdomain-containingprotein17-like[Glyciner
Aradu.6W7E4	Araip.BAN8Q	multiple-copy	zincfingerCCCHdomain-containingprotein31-like[Glyciner
Aradu.X5630	Araip.ZI2S3	multiple-copy	zincfingerCCCHdomain-containingprotein38-likeisoformX
Aradu.QQB0D	Araip.DFK5Q	multiple-copy	zincfingerCCCHdomain-containingprotein48-likeisoformX
Aradu.TA161	Araip.G4AXZ	multiple-copy	zincfingerCCCHdomain-containingprotein48-likeisoformX
Aradu.BY7GE	Araip.15RTX	multiple-copy	zincfingerCCCHdomain-containingprotein48-likeisoformX
Aradu.QC0XR	Araip.I6LMJ	multiple-copy	zincfingerCCCHdomain-containingprotein48-likeisoformX
Aradu.4S6WR	Araip.JY0CR	multiple-copy	zincfingerCCCHdomain-containingprotein6-like[Glycinem
Aradu.JM1K2	Araip.06TYZ	multiple-copy	zincfingerCCCHdomainprotein
Aradu.M8FIQ	Araip.20WLX	multiple-copy	zincfingerCCCHdomainprotein
Aradu.UDE5G	Araip.6WV2C	multiple-copy	zincfingerCCCHdomainprotein
Aradu.59NQ7	Araip.A437V	multiple-copy	zincfingerCCCHdomainprotein
Aradu.1P8DE	Araip.EY89K	multiple-copy	zincfingerCCCHdomainprotein
Aradu.MVP73	Araip.YT0BK	multiple-copy	zincfingerCCCHdomainprotein
Aradu.50M8D	Araip.DB6DZ	multiple-copy	zincfingerCCCHdomainprotein,putative
Aradu.YP97N	Araip.YGP1F	multiple-copy	zincfingerCCCHdomainprotein,putative
Aradu.I7M78	Araip.0Z6G1	multiple-copy	ZincfingerC-x8-C-x5-C-x3-Htypefamilyprotein

Aradu.N5GDJ	Araip.WZG9Z	multiple-copy	ZincfingerC-x8-C-x5-C-x3-Htypefamilyprotein
Aradu.J72N3	Araip.83T6W	multiple-copy	zincfingerMYM-typeprotein1-like[Glycinemax]
Aradu.L2S26	Araip.CKV7I	multiple-copy	zincfingerMYM-typeprotein1-like[Glycinemax]
Aradu.WFH0K	Araip.G0JJM	multiple-copy	zincfingerMYM-typeprotein1-like[Glycinemax]
Aradu.W06MF	Araip.BYF4T	multiple-copy	zinc-fingerprotein
Aradu.68MCI	Araip.S69I6	multiple-copy	zinc-fingerprotein
Aradu.42P16	Araip.HF5I3	multiple-copy	zinc-fingerprotein
Aradu.E6FTH	Araip.6B24B	multiple-copy	zincfingerprotein,putative
Aradu.S056J	Araip.L9LM7	multiple-copy	zincfingerprotein4-like[Glycinemax]
Aradu.DS35R	Araip.6NZ59	multiple-copy	zincfingerprotein598-likeisoformX1[Glycinemax]
Aradu.YCI2Q	Araip.6H4KV	multiple-copy	Zincfingerprotein622
Aradu.2L1YZ	Araip.NDM7B	multiple-copy	zincfingerprotein6-like[Glycinemax]
Aradu.X9YQG	Araip.V6DM9	multiple-copy	zincfingerprotein6-like[Glycinemax]
Aradu.ZT2KF	Araip.YU18D	multiple-copy	zincfingerproteinCONSTANS-LIKE12-like[Glycinemax]
Aradu.J9KV2	Araip.IK2R0	multiple-copy	zincfingerproteinCONSTANS-LIKE16-like[Glycinemax]
Aradu.WJ2ZP	Araip.JXV3W	multiple-copy	zincfingerproteinCONSTANS-LIKE16-like[Glycinemax]
Aradu.53CQM	Araip.BHE2G	multiple-copy	zincfingerproteinCONSTANS-LIKE1-like[Glycinemax]
Aradu.W44WH	Araip.I0PWR	multiple-copy	zincfingerproteinCONSTANS-LIKE1-like[Glycinemax]
Aradu.F08JS	Araip.E27FI	multiple-copy	zincfingerproteinCONSTANS-LIKE2-like[Glycinemax]
Aradu.7ZG0H	Araip.P77MW	multiple-copy	zincfingerproteinCONSTANS-LIKE2-like[Glycinemax]
Aradu.S156I	Araip.PEC40	multiple-copy	zincfingerproteinCONSTANS-LIKE2-like[Glycinemax]
Aradu.5RF5F	Araip.0L5SE	multiple-copy	zincfingerproteinCONSTANS-LIKE4-like[Glycinemax]
Aradu.7V6T8	Araip.8LI6X	multiple-copy	zincfingerproteinCONSTANS-LIKE5-like[Glycinemax]
Aradu.V9D7S	Araip.E4G9U	multiple-copy	zincfingerproteinCONSTANS-LIKE5-like[Glycinemax]
Aradu.3ZR52	Araip.WH6UQ	multiple-copy	zincfingerproteinCONSTANS-LIKE2-like[Glycinemax]
Aradu.BV95P	Araip.U0W6U	multiple-copy	zincfingerproteinCONSTANS-LIKE9-likeisoformX4[Glycin
Aradu.28KTI	Araip.HCG04	multiple-copy	zincfingerproteinCONSTANS-likeisoformX2[Glycinemax]
Aradu.ZM93S	Araip.14NBL	multiple-copy	zincfingerproteinMAGPIE-like[Glycinemax]
Aradu.72K9J	Araip.AJC62	multiple-copy	zincfingerproteinMAGPIE-like[Glycinemax]
Aradu.H0HJA	Araip.HB8ML	multiple-copy	zincfingerproteinMAGPIE-like[Glycinemax]
Aradu.05H52	Araip.R4F69	multiple-copy	zincfingerproteinMAGPIE-like[Glycinemax]
Aradu.9C9UT	Araip.T7RN5	multiple-copy	zincfingerproteinMAGPIE-like[Glycinemax]
Aradu.2119N	Araip.69YUJ	multiple-copy	zincfingerprotein-related
Aradu.T9YMT	Araip.V40G1	multiple-copy	zincfingerdomain-containingprotein1-likeisoformX2[Glycir
Aradu.V5HPY	Araip.P6VI6	multiple-copy	zincionbinding
Aradu.EB27U	Araip.PH2FC	multiple-copy	zincionbinding
Aradu.AU181	Araip.TW0D4	multiple-copy	zincionbinding
Aradu.99LXR	Araip.YI4UQ	multiple-copy	zincionbinding
Aradu.G6ZH0	Araip.Q4K8M	multiple-copy	zincion-bindingprotein
Aradu.YR4CG	Araip.X7S59	multiple-copy	zinctransporterproteinDDB_G0291141isoform1[Glycinem
Aradu.YJH4A	Araip.H38UN	multiple-copy	ZIPmetaliontransporterfamily
Aradu.ULX7G	Araip.A98H2	multiple-copy	ZIPzinc/irontransportfamilyprotein
Aradu.F0D6Z	Araip.CAX6Y	multiple-copy	ZIPzinc/irontransportfamilyprotein
Aradu.9T8AF	Araip.IT4EA	multiple-copy	ZIPzinc/irontransportfamilyprotein

Aradu.CX8YW	Araip.J8RHM	multiple-copy	ZIPzinc/irontransportfamilyprotein
Aradu.1P1D6	Araip.V9JLA	multiple-copy	ZIPzinc/irontransportfamilyprotein
Aradu.ZN9Y3	Araip.YRI89	multiple-copy	ZIPzinc/irontransportfamilyprotein

Table S2 Fop, amino acid length, GC content in *Arachis duranensis* and *Arachis ipaënsis* one-to-one orthologs

Arachis duranensis	Fop	Polypeptide length	GC1 content	GC2 content	GC3 content	Arachis ipaënsis	Fop
Aradu.001N3	0.347	644	50.698	46.512	35.039	Araip.P81AY	0.339
Aradu.003TN	0.43	255	47.266	34.375	51.172	Araip.LVH53	0.434
Aradu.0060C	0.302	285	48.252	48.252	33.217	Araip.U0Y4C	0.336
Aradu.008V1	0.291	297	47.987	38.926	31.879	Araip.BJM1C	0.3
Aradu.00CSZ	0.374	669	48.955	37.761	43.582	Araip.483YA	0.375
Aradu.00HGB	0.348	238	51.046	41.004	34.31	Araip.8H0EA	0.339
Aradu.00MP0	0.371	254	51.765	39.608	49.02	Araip.T85A3	0.371
Aradu.00NYG	0.396	652	51.914	39.816	57.887	Araip.9D3W1	0.408
Aradu.00SPE	0.377	391	59.184	51.786	35.969	Araip.PG7W4	0.384
Aradu.00U6W	0.384	504	43.168	37.624	40.198	Araip.A82X5	0.376
Aradu.00WD1	0.418	459	48.913	37.826	49.565	Araip.WI73I	0.417
Aradu.00WGF	0.316	328	46.505	43.161	31.915	Araip.NLR8N	0.311
Aradu.00XL9	0.496	374	56.267	44.533	49.333	Araip.E2MIX	0.492
Aradu.014NJ	0.365	1401	47.29	35.093	41.013	Araip.H43AY	0.365
Aradu.01B4C	0.302	191	41.146	34.375	44.271	Araip.4C1AU	0.296
Aradu.01E53	0.428	194	50.769	41.538	45.128	Araip.NZG3T	0.454
Aradu.01TNR	0.363	595	49.329	40.772	37.919	Araip.RX5RL	0.374
Aradu.01VAS	0.414	405	57.635	36.7	49.261	Araip.WE2GD	0.402
Aradu.01YLW	0.36	654	47.786	38.626	42.137	Araip.J49D4	0.369
Aradu.0252U	0.342	452	47.461	52.539	42.605	Araip.HB95A	0.353
Aradu.0291B	0.408	1017	47.348	37.525	42.436	Araip.FW7GM	0.411
Aradu.02DA8	0.334	431	46.528	28.009	34.954	Araip.B00X5	0.331
Aradu.02GMF	0.424	550	48.82	40.472	54.265	Araip.5JH12	0.421
Aradu.02I1H	0.392	1332	49.887	36.384	40.81	Araip.E67FB	0.395
Aradu.02IGP	0.452	426	53.162	43.091	57.611	Araip.V3B0A	0.442
Aradu.02LQD	0.391	216	36.406	33.641	32.258	Araip.JP4ZN	0.396
Aradu.02NM4	0.386	377	52.116	42.593	52.646	Araip.91RV8	0.383
Aradu.02NZN	0.375	313	40.127	48.089	37.898	Araip.F8JDN	0.367
Aradu.02ZTY	0.405	586	54.174	42.419	35.605	Araip.Z52VV	0.4
Aradu.031YC	0.365	342	51.02	41.108	37.901	Araip.176AR	0.364
Aradu.034KP	0.455	249	49.6	45.2	51.6	Araip.GMD2H	0.466
Aradu.035RQ	0.33	106	43.925	42.056	45.794	Araip.8276P	0.33
Aradu.03ENG	0.411	116	48.718	54.701	46.154	Araip.0E4ZE	0.447
Aradu.03ESY	0.397	158	54.088	35.849	45.912	Araip.D1KMK	0.404

Aradu.03GCY	0.349	486	41.684	35.318	34.497	Araip.YR17W	0.351
Aradu.03IBL	0.449	150	52.318	37.748	39.073	Araip.R1CPL	0.463
Aradu.03THN	0.376	1628	48.987	42.296	37.139	Araip.Z85GJ	0.376
Aradu.03X4Q	0.405	375	51.862	38.564	41.489	Araip.1942F	0.4
Aradu.041TI	0.376	334	51.343	44.179	40.597	Araip.BN5SV	0.378
Aradu.04DD4	0.399	424	55.294	39.765	42.353	Araip.USK4J	0.403
Aradu.04EM8	0.385	906	57.442	38.699	33.407	Araip.TV5CN	0.392
Aradu.04GWS	0.39	361	52.21	34.807	47.79	Araip.VZN05	0.375
Aradu.04L8A	0.365	871	53.67	36.697	36.927	Araip.0Y7IG	0.354
Aradu.04QLM	0.356	501	48.805	37.649	38.845	Araip.RX21A	0.364
Aradu.04RDY	0.429	228	55.459	46.725	50.218	Araip.ZX8HU	0.427
Aradu.04VAI	0.441	109	50.909	30.909	49.091	Araip.U7YDL	0.404
Aradu.0514U	0.446	258	54.826	48.649	50.965	Araip.541ME	0.426
Aradu.0572C	0.429	262	61.217	47.909	59.316	Araip.Z1VRT	0.43
Aradu.058CJ	0.396	611	52.288	38.072	42.81	Araip.9L82B	0.396
Aradu.05DT4	0.424	343	52.326	40.698	47.965	Araip.45RHI	0.424
Aradu.05H52	0.355	488	42.127	45.194	38.037	Araip.R4F69	0.36
Aradu.05VAM	0.37	237	57.563	50	31.513	Araip.26Q67	0.396
Aradu.05WJ3	0.494	356	45.378	42.577	54.622	Araip.TD953	0.499
Aradu.065GY	0.326	479	42.708	40	33.958	Araip.W1EXI	0.327
Aradu.068JX	0.397	248	52.61	45.783	48.594	Araip.C0Z69	0.392
Aradu.069GY	0.416	385	48.964	38.083	44.819	Araip.V3YMP	0.408
Aradu.06C7F	0.483	280	58.719	46.619	54.448	Araip.KPA97	0.49
Aradu.06CIQ	0.359	378	48.285	42.744	35.62	Araip.K7W5L	0.362
Aradu.06HG0	0.368	413	49.758	30.676	42.754	Araip.IA04P	0.372
Aradu.06JB2	0.364	379	52.632	36.053	45.526	Araip.BAZ0W	0.372
Aradu.06NY9	0.406	148	44.295	37.584	32.886	Araip.NK3HL	0.419
Aradu.070N1	0.361	125	45.238	38.889	42.857	Araip.EX0TX	0.353
Aradu.07167	0.411	323	46.605	42.284	42.593	Araip.MY084	0.41
Aradu.073NL	0.363	1202	46.55	37.24	43.724	Araip.GS20D	0.374
Aradu.074JV	0.427	336	46.588	42.73	43.62	Araip.BJ3QY	0.418
Aradu.0768L	0.356	329	49.697	31.515	45.758	Araip.VC558	0.356
Aradu.07AAJ	0.339	301	47.351	39.404	42.053	Araip.1JP91	0.343
Aradu.07E2R	0.519	195	54.082	48.469	62.755	Araip.AF2L5	0.485
Aradu.07NMY	0.397	212	46.009	38.967	44.601	Araip.G2AKQ	0.399
Aradu.07V9Z	0.479	231	47.414	40.948	55.172	Araip.00H39	0.479
Aradu.07VYH	0.416	232	49.356	43.777	48.927	Araip.5MC2N	0.427
Aradu.07WQA	0.384	490	57.841	43.177	42.974	Araip.PQA2W	0.397
Aradu.07ZWB	0.366	176	45.763	42.938	40.113	Araip.I4558	0.36
Aradu.085I9	0.453	258	57.915	50.193	40.541	Araip.G8B8H	0.461
Aradu.085LG	0.365	489	47.959	40	38.776	Araip.39IGX	0.369
Aradu.085T6	0.432	552	45.389	41.049	43.4	Araip.852K4	0.438
Aradu.08LLU	0.38	585	57.85	47.099	42.321	Araip.M4B3A	0.387
Aradu.08MEK	0.488	176	42.938	46.893	44.633	Araip.Z9VQ5	0.54

Aradu.08REY	0.469	497	52.61	48.594	64.257	Araip.4LL7A	0.454
Aradu.08WSJ	0.432	443	50.45	45.495	51.351	Araip.48FDM	0.445
Aradu.08Y2X	0.371	488	51.125	44.581	60.123	Araip.GL06B	0.367
Aradu.0924G	0.347	317	48.428	38.679	38.994	Araip.E2X73	0.35
Aradu.092KC	0.359	315	49.684	42.405	43.354	Araip.09VK1	0.329
Aradu.096I4	0.359	205	49.515	43.204	30.097	Araip.A4LGX	0.359
Aradu.096TH	0.365	267	44.776	34.328	37.313	Araip.FCR1J	0.375
Aradu.09F0B	0.369	566	49.03	40.212	38.272	Araip.1GQ6A	0.372
Aradu.09HBR	0.394	144	48.966	47.586	42.069	Araip.MX0X9	0.394
Aradu.09HMV	0.413	203	49.02	41.176	45.588	Araip.21QMU	0.408
Aradu.09JHP	0.426	750	49.134	26.365	54.727	Araip.E0PG3	0.426
Aradu.09SAF	0.451	522	57.17	40.153	57.744	Araip.B1IVR	0.455
Aradu.0A17C	0.31	345	42.775	37.861	39.017	Araip.TVH0X	0.31
Aradu.0AW6N	0.327	155	42.949	42.949	32.051	Araip.K08CD	0.333
Aradu.0AW7C	0.394	572	51.483	34.729	42.059	Araip.H38XD	0.405
Aradu.0B0D2	0.422	422	49.173	36.643	50.827	Araip.J9R36	0.42
Aradu.0B375	0.404	653	48.93	42.813	39.297	Araip.H48EG	0.401
Aradu.0BJ82	0.486	118	52.101	31.092	52.941	Araip.A2GBD	0.459
Aradu.0BM64	0.467	111	49.107	34.821	49.107	Araip.C8HFV	0.476
Aradu.0BT33	0.362	147	46.622	32.432	34.459	Araip.ZD4VN	0.376
Aradu.0C0S7	0.327	268	52.045	27.509	43.866	Araip.B3ZXX	0.343
Aradu.0C27S	0.335	273	46.715	37.591	43.066	Araip.REH1B	0.335
Aradu.0C6YR	0.36	116	58.12	40.171	49.573	Araip.18S7T	0.342
Aradu.0C9TU	0.415	604	54.38	39.008	42.314	Araip.JZ063	0.411
Aradu.0CM87	0.372	530	48.776	44.068	38.795	Araip.D0KPG	0.373
Aradu.0D317	0.383	419	47.619	40.238	35.476	Araip.GN51K	0.393
Aradu.0D7Q2	0.44	375	51.33	44.149	38.298	Araip.J7WAX	0.443
Aradu.0D9UK	0.358	436	49.199	47.14	37.3	Araip.3C7F6	0.359
Aradu.0DL1W	0.35	416	55.875	41.247	43.405	Araip.21V2Q	0.344
Aradu.0DS3V	0.368	939	48.085	35.106	35.851	Araip.H7JSC	0.366
Aradu.0E5MF	0.518	149	53.333	45.333	50.667	Araip.YJC7I	0.511
Aradu.0E8DM	0.333	198	50.251	58.794	38.693	Araip.VQ4D8	0.342
Aradu.0EA4R	0.386	355	39.888	36.517	31.742	Araip.4509F	0.378
Aradu.0EG1Y	0.468	459	56.304	41.304	68.696	Araip.563L5	0.468
Aradu.0EM6K	0.402	264	54.717	36.981	37.358	Araip.S4JFP	0.384
Aradu.0ER06	0.384	845	48.109	43.853	41.017	Araip.B68MT	0.392
Aradu.0EZ1S	0.413	803	56.716	38.93	28.856	Araip.KBB88	0.43
Aradu.0FB9R	0.318	162	50.307	34.969	34.356	Araip.Q5FHC	0.28
Aradu.0FG6K	0.374	530	44.444	36.535	38.983	Araip.W427S	0.379
Aradu.0FH1C	0.424	300	51.495	46.844	39.203	Araip.EK2ZC	0.429
Aradu.0FI0R	0.351	869	52.874	39.195	39.195	Araip.V5LSC	0.351
Aradu.0G2ML	0.413	155	55.769	41.667	52.564	Araip.Q98DA	0.387
Aradu.0G5T2	0.427	146	57.823	44.218	47.619	Araip.F5QUZ	0.448
Aradu.0G8DQ	0.368	568	53.954	31.986	44.815	Araip.TSB39	0.37

Aradu.0GK1P	0.42	274	57.818	47.273	53.091	Araip.VPK3J	0.416
Aradu.0GS9T	0.361	442	46.501	41.309	41.761	Araip.IHP9I	0.361
Aradu.0GZ0B	0.359	756	52.576	41.347	37.252	Araip.ZD7NF	0.354
Aradu.0H4SB	0.364	372	51.743	41.019	38.874	Araip.FRL5W	0.362
Aradu.0H9WK	0.421	371	53.763	41.398	40.86	Araip.VGR7G	0.421
Aradu.0HA70	0.413	193	45.876	32.474	45.876	Araip.I53JU	0.418
Aradu.0HM6U	0.393	515	51.163	49.031	43.023	Araip.3UC9S	0.399
Aradu.0I3GY	0.341	507	45.276	35.039	36.024	Araip.B5SML	0.334
Aradu.0IN20	0.458	514	51.456	41.942	40.971	Araip.VP2HF	0.458
Aradu.0IR2T	0.481	418	49.165	62.053	46.778	Araip.VJ4I5	0.486
Aradu.0JT6M	0.371	293	55.442	49.32	36.054	Araip.LLR5T	0.387
Aradu.0JV1U	0.341	977	47.444	35.787	34.151	Araip.J80IJ	0.337
Aradu.0K11B	0.376	1531	46.606	40.535	38.838	Araip.G4MI2	0.377
Aradu.0K49H	0.345	836	47.431	37.276	40.263	Araip.RZ87P	0.355
Aradu.0KB9D	0.48	459	53.261	48.478	58.913	Araip.1VN1Z	0.485
Aradu.0KB9L	0.369	773	56.589	45.09	50.646	Araip.W6U6L	0.356
Aradu.0KG04	0.434	576	52.86	43.674	51.993	Araip.SVT7Q	0.432
Aradu.0KH4T	0.389	644	49.302	37.209	40	Araip.65II6	0.389
Aradu.0KQ9H	0.411	278	51.971	46.953	32.616	Araip.YS13X	0.419
Aradu.0KX1J	0.408	570	50.088	38.879	52.715	Araip.SKH2V	0.402
Aradu.0LB5P	0.46	393	49.746	42.893	43.401	Araip.QP2H1	0.458
Aradu.0LC5Q	0.441	188	55.556	47.619	51.852	Araip.6TL19	0.446
Aradu.0LP8B	0.378	196	42.64	40.102	33.503	Araip.QPM11	0.373
Aradu.0M33I	0.425	595	47.651	37.584	44.463	Araip.0J2TC	0.423
Aradu.0M3HI	0.407	408	48.411	45.721	47.188	Araip.N5J1U	0.414
Aradu.0M5LW	0.404	714	48.112	38.881	37.622	Araip.85A5N	0.404
Aradu.0M8VI	0.48	471	56.78	45.551	65.466	Araip.72WPA	0.489
Aradu.0M9X8	0.371	323	47.531	41.667	36.728	Araip.VS99S	0.347
Aradu.0ME59	0.387	807	53.837	35.891	42.203	Araip.U7HDR	0.382
Aradu.0MJ0X	0.386	384	52.987	40.779	53.247	Araip.J9WH5	0.385
Aradu.0MP9W	0.385	1094	48.128	38.356	42.283	Araip.8PG2B	0.377
Aradu.0MS0A	0.363	439	53.182	44.091	42.955	Araip.7Y7KI	0.365
Aradu.0N1XE	0.483	371	52.688	43.548	40.591	Araip.H8TGQ	0.485
Aradu.0N518	0.526	139	51.429	50.714	56.429	Araip.5M5DL	0.526
Aradu.0N6WQ	0.348	317	49.371	36.792	47.799	Araip.ACR9K	0.361
Aradu.0NA24	0.36	842	48.873	34.994	38.316	Araip.U82F4	0.369
Aradu.0NC5M	0.385	263	50.758	40.909	43.182	Araip.LMJ0Y	0.395
Aradu.0NE9W	0.398	606	48.27	39.539	36.573	Araip.AL2VM	0.398
Aradu.0NR0Q	0.366	885	55.982	42.438	32.054	Araip.8JW62	0.368
Aradu.0NY83	0.382	413	57.488	34.541	42.271	Araip.T68Y6	0.379
Aradu.0NY8Y	0.409	425	48.592	45.54	40.376	Araip.P2MP0	0.415
Aradu.0P8G3	0.41	224	55.111	40.889	47.111	Araip.AG3WT	0.406
Aradu.0P9WS	0.417	264	53.962	36.226	48.302	Araip.QK4T6	0.42
Aradu.0PC6S	0.426	327	59.756	37.5	50.305	Araip.JKV7Y	0.442

Aradu.0PI99	0.393	835	50.598	40.311	37.679	Araip.L7WHL	0.395
Aradu.0PL1F	0.433	260	54.789	47.893	40.23	Araip.PXW4K	0.418
Aradu.0Q16W	0.377	288	50.865	43.945	35.294	Araip.59D2H	0.372
Aradu.0Q24B	0.324	491	57.52	38.211	32.317	Araip.PIW91	0.32
Aradu.0Q24I	0.363	512	46.784	40.741	43.08	Araip.H8S62	0.371
Aradu.0Q5NS	0.332	236	49.789	32.911	40.084	Araip.JS7DG	0.346
Aradu.0QA18	0.436	192	49.223	42.487	63.212	Araip.T5SG5	0.426
Aradu.0QC7R	0.393	248	49.398	44.578	38.153	Araip.U67Y5	0.389
Aradu.0QF6H	0.328	142	45.455	34.965	36.364	Araip.03GF9	0.307
Aradu.0QJ0H	0.39	769	50.26	45.455	47.662	Araip.XLI18	0.388
Aradu.0QN5D	0.362	861	48.492	44.432	31.787	Araip.FDR3N	0.361
Aradu.0QQ3Z	0.398	267	52.612	39.925	51.866	Araip.KMZ8M	0.408
Aradu.0R9W1	0.405	706	50.354	44.272	53.607	Araip.01AT6	0.417
Aradu.0RA4R	0.405	198	52.764	31.156	44.724	Araip.JLZ8T	0.395
Aradu.0RQ1E	0.327	843	44.905	34.123	41.943	Araip.DB5EM	0.326
Aradu.0RW41	0.374	364	51.507	41.37	35.89	Araip.KSH3B	0.347
Aradu.0S3RJ	0.484	611	49.183	40.686	64.379	Araip.S3E2A	0.48
Aradu.0SC6H	0.407	380	55.906	45.144	47.507	Araip.VL6YY	0.398
Aradu.0SE2F	0.372	566	51.499	36.332	41.27	Araip.9UZ34	0.361
Aradu.0SM3T	0.341	424	49.176	38.824	41.412	Araip.J4V1E	0.347
Aradu.0SN1Y	0.387	1035	44.112	41.216	40.637	Araip.900EH	0.384
Aradu.0SV2V	0.355	487	44.262	36.27	35.861	Araip.87PIS	0.363
Aradu.0T5YP	0.394	337	54.734	36.686	34.615	Araip.WS0IW	0.388
Aradu.0TS71	0.394	451	45.354	36.504	43.142	Araip.D3HL4	0.418
Aradu.0U1AX	0.372	941	51.592	39.49	41.614	Araip.M5PAK	0.383
Aradu.0U5ND	0.354	163	62.195	48.78	42.073	Araip.V5W4F	0.35
Aradu.0UC7C	0.354	1484	47.946	39.798	34.478	Araip.7JE55	0.355
Aradu.0US4M	0.42	321	49.068	43.168	49.689	Araip.GL0UW	0.413
Aradu.0UW7J	0.433	488	58.078	44.99	50.102	Araip.IB6M8	0.433
Aradu.0V01P	0.397	947	55.063	38.08	42.3	Araip.ZP2M5	0.395
Aradu.0V2BM	0.276	211	48.585	40.094	28.774	Araip.MGF6M	0.279
Aradu.0V3TN	0.342	1313	47.26	37.671	33.333	Araip.89KY2	0.34
Aradu.0VN2L	0.387	344	47.536	40.58	38.841	Araip.RNE4C	0.353
Aradu.0VP21	0.346	727	48.626	35.165	44.231	Araip.Q56KQ	0.352
Aradu.0VP5J	0.419	340	49.853	32.551	42.229	Araip.ZVT8T	0.413
Aradu.0WA6T	0.457	431	59.028	36.111	44.213	Araip.W7GWX	0.448
Aradu.0WP1T	0.448	393	53.299	43.401	53.807	Araip.KU7H8	0.454
Aradu.0WU0I	0.353	214	53.953	34.419	46.512	Araip.AF9FL	0.369
Aradu.0X9JH	0.381	1288	49.341	40.807	32.661	Araip.K5R5A	0.373
Aradu.0X9JV	0.425	331	53.012	37.048	44.277	Araip.Q4VW4	0.422
Aradu.0XA87	0.481	426	61.593	45.433	65.808	Araip.1MT39	0.478
Aradu.0XB6R	0.396	167	50	47.024	44.643	Araip.23MFU	0.39
Aradu.0XX6X	0.372	366	56.131	40.599	37.602	Araip.FJ13R	0.352
Aradu.0Y2ZI	0.357	427	52.103	39.019	35.981	Araip.PP40A	0.35

Aradu.0Y40Q	0.388	528	47.637	37.24	55.955	Araip.B7MLT	0.375
Aradu.0Y99B	0.407	415	43.75	40.625	45.192	Araip.8Z53B	0.416
Aradu.0YE33	0.474	100	61.386	53.465	46.535	Araip.9A6T0	0.474
Aradu.0YP72	0.33	467	48.504	40.812	40.385	Araip.FL5I2	0.331
Aradu.0YR51	0.346	561	43.95	36.299	42.349	Araip.SD8AP	0.344
Aradu.0YT2V	0.386	1027	50.584	40.759	39.883	Araip.YRA5M	0.386
Aradu.0YY6A	0.422	124	55.2	34.4	46.4	Araip.37KVD	0.414
Aradu.0Z09C	0.407	221	43.243	36.937	36.036	Araip.A49CU	0.398
Aradu.0Z207	0.336	308	51.133	28.479	34.951	Araip.FL1CB	0.329
Aradu.0Z7KI	0.361	432	47.806	39.03	33.487	Araip.TYZ14	0.371
Aradu.0Z8XN	0.411	518	51.638	40.655	51.252	Araip.0N6RB	0.396
Aradu.0ZB6D	0.443	231	52.155	35.776	47.845	Araip.IX0TZ	0.453
Aradu.0ZG2X	0.393	470	41.401	29.087	37.792	Araip.52WME	0.388
Aradu.102BT	0.368	606	45.14	40.362	35.75	Araip.UJ652	0.373
Aradu.105GZ	0.334	346	53.89	43.228	37.176	Araip.E92TL	0.328
Aradu.109RJ	0.338	161	49.383	32.099	39.506	Araip.A97NN	0.344
Aradu.10AS3	0.336	152	39.216	35.294	43.791	Araip.5I7AM	0.342
Aradu.10BGT	0.368	588	59.423	43.124	34.465	Araip.I2X2E	0.373
Aradu.10HN0	0.382	474	49.474	37.684	38.947	Araip.62ZD7	0.388
Aradu.10I3J	0.422	510	41.292	30.528	46.967	Araip.M15N8	0.417
Aradu.10VKJ	0.38	975	50.615	38.422	36.066	Araip.4N5EI	0.379
Aradu.10XKC	0.37	501	52.988	44.622	39.641	Araip.LWT8I	0.365
Aradu.10YFJ	0.428	1023	49.902	43.945	47.363	Araip.256VQ	0.414
Aradu.10YFM	0.345	702	48.791	41.821	46.23	Araip.N5Q83	0.349
Aradu.10ZFH	0.385	424	51.294	38.824	39.059	Araip.IP580	0.38
Aradu.114L5	0.377	138	46.763	32.374	41.007	Araip.C8BSP	0.398
Aradu.115J9	0.391	323	54.012	37.654	35.185	Araip.671MH	0.385
Aradu.117B0	0.43	198	43.216	59.799	29.648	Araip.KTA7Q	0.435
Aradu.1195M	0.342	441	53.167	39.367	46.38	Araip.U26LB	0.335
Aradu.11960	0.414	197	54.04	34.343	45.455	Araip.EIT18	0.419
Aradu.11KMG	0.403	263	52.652	33.333	47.348	Araip.647BE	0.382
Aradu.11V2F	0.412	331	54.518	40.06	51.506	Araip.21TTJ	0.415
Aradu.11XZB	0.528	160	44.099	35.404	39.13	Araip.W441F	0.392
Aradu.1235N	0.359	719	47.917	40.972	44.306	Araip.46LQV	0.371
Aradu.124FF	0.312	878	51.308	46.758	28.896	Araip.YQ272	0.307
Aradu.12CJQ	0.492	370	47.439	47.17	59.03	Araip.V8KRY	0.476
Aradu.12EER	0.392	297	46.309	40.604	33.221	Araip.H7369	0.385
Aradu.12ETV	0.451	360	42.936	35.18	48.199	Araip.I4CFI	0.462
Aradu.12LHJ	0.406	391	50.255	41.837	42.347	Araip.137YQ	0.406
Aradu.12T0R	0.414	335	43.75	44.94	40.179	Araip.JSZ6P	0.419
Aradu.12WE9	0.377	127	53.125	38.281	30.469	Araip.LS9DG	0.38
Aradu.12XXA	0.374	932	47.803	36.013	37.406	Araip.W3VCN	0.372
Aradu.131HS	0.342	825	50	32.688	37.53	Araip.22U9L	0.353
Aradu.1347E	0.308	467	49.145	32.692	36.966	Araip.PTN9R	0.305

Aradu.137AN	0.443	382	50.392	34.987	46.214	Araip.FZW6D	0.441
Aradu.13D06	0.473	396	55.164	42.065	56.927	Araip.U9RGH	0.449
Aradu.13E7W	0.371	365	50	36.885	42.35	Araip.A6A17	0.399
Aradu.13H1D	0.387	245	54.065	35.772	41.463	Araip.EU5DQ	0.387
Aradu.13MQ9	0.385	594	55.294	42.857	35.126	Araip.VXU18	0.386
Aradu.13QYM	0.457	144	56.552	46.207	46.897	Araip.9ZM5J	0.449
Aradu.13SFN	0.398	363	50.549	40.385	38.462	Araip.B6Q3S	0.398
Aradu.13TYF	0.367	616	51.378	42.301	41.653	Araip.NJ40E	0.367
Aradu.13Y5Y	0.384	406	52.334	43.735	42.26	Araip.2BU9K	0.394
Aradu.14261	0.374	1151	53.646	35.243	35.938	Araip.HH32S	0.379
Aradu.142E0	0.42	1254	48.845	39.363	48.287	Araip.P4FY7	0.406
Aradu.145AN	0.374	331	51.205	47.892	40.06	Araip.613HG	0.386
Aradu.149WS	0.387	479	52.917	47.083	36.042	Araip.HIL9C	0.392
Aradu.14ANW	0.466	365	51.913	44.536	64.754	Araip.TP6JV	0.467
Aradu.14CRM	0.359	307	51.948	45.13	35.065	Araip.39SY8	0.355
Aradu.14D5A	0.387	346	58.213	40.922	43.228	Araip.1SI6Z	0.378
Aradu.14EED	0.316	591	46.284	34.291	36.486	Araip.JWP0N	0.32
Aradu.14QL4	0.372	675	43.491	37.13	39.497	Araip.IHC2V	0.38
Aradu.14TMT	0.386	574	44.696	37.217	35.304	Araip.S8ZP2	0.39
Aradu.14U3F	0.387	995	56.225	38.153	39.558	Araip.D1KQ3	0.384
Aradu.14WTD	0.419	675	48.521	44.083	44.527	Araip.2X2JD	0.41
Aradu.14Z9Y	0.332	205	50	38.835	35.922	Araip.KAK82	0.34
Aradu.1573F	0.365	649	41.846	35.538	38.923	Araip.TJ4MC	0.373
Aradu.15KNS	0.444	548	50.82	36.612	48.27	Araip.QCA7D	0.435
Aradu.15MGK	0.353	833	53.357	43.885	36.571	Araip.G1GMX	0.357
Aradu.15QSN	0.393	255	54.688	42.188	33.984	Araip.E3UJF	0.393
Aradu.15R8P	0.45	193	48.969	44.845	56.186	Araip.33H23	0.46
Aradu.15RGK	0.403	129	46.154	41.538	39.231	Araip.WG92W	0.429
Aradu.15W25	0.314	177	40.449	37.64	27.528	Araip.I5RQQ	0.314
Aradu.162IL	0.342	635	47.799	40.252	36.478	Araip.Y74NR	0.346
Aradu.1659P	0.365	971	47.737	38.992	41.255	Araip.B3H24	0.357
Aradu.16BXD	0.417	230	55.844	45.022	42.857	Araip.1309E	0.439
Aradu.16CAR	0.394	544	55.229	43.303	44.404	Araip.24N70	0.39
Aradu.16E7C	0.378	305	47.059	36.601	50.654	Araip.JG5RP	0.385
Aradu.16GQU	0.457	274	57.455	47.273	49.091	Araip.U0GR3	0.455
Aradu.16HC5	0.386	184	51.351	38.919	43.243	Araip.1I1JH	0.381
Aradu.16P0Z	0.367	427	46.262	37.383	35.514	Araip.785V6	0.379
Aradu.16QCN	0.405	158	45.912	42.138	39.623	Araip.N7Q35	0.392
Aradu.16U3K	0.377	386	58.915	49.354	39.793	Araip.RP87W	0.371
Aradu.16YW2	0.386	605	48.515	39.439	45.215	Araip.43MGU	0.397
Aradu.16Z19	0.363	330	49.547	39.879	45.015	Araip.UJ65V	0.373
Aradu.172F0	0.361	509	53.137	34.706	39.608	Araip.7Y0PA	0.354
Aradu.175L3	0.467	524	47.238	41.143	59.619	Araip.M2GWH	0.469
Aradu.17HPD	0.442	606	52.554	44.152	49.423	Araip.W9HXX	0.435

Aradu.17ZI4	0.488	301	49.669	39.073	74.834	Araip.7AQ3E	0.498
Aradu.1864T	0.345	585	46.928	35.836	36.007	Araip.33CRB	0.352
Aradu.188J4	0.403	465	57.511	38.197	57.94	Araip.XP707	0.41
Aradu.18BRD	0.38	264	45.283	43.396	55.849	Araip.81209	0.38
Aradu.18DC6	0.38	524	51.238	39.619	38.095	Araip.4DN6E	0.39
Aradu.18DQZ	0.415	247	51.21	41.935	41.532	Araip.K8LIV	0.419
Aradu.18FWJ	0.455	143	45.833	42.361	60.417	Araip.FIV2R	0.414
Aradu.18HYX	0.384	441	47.285	47.738	36.425	Araip.AA0NE	0.389
Aradu.18NVF	0.429	610	49.1	42.553	41.735	Araip.36HY2	0.409
Aradu.18R1L	0.358	1033	51.064	37.718	42.263	Araip.IX44P	0.353
Aradu.18TLF	0.427	156	53.503	48.408	56.051	Araip.TLB1K	0.439
Aradu.18V7U	0.438	197	51.515	39.899	72.727	Araip.G0BSA	0.449
Aradu.196ZM	0.353	267	51.493	44.776	39.925	Araip.J7PSL	0.348
Aradu.199G1	0.374	105	44.34	34.906	33.019	Araip.SJE6C	0.337
Aradu.199XC	0.422	591	45.777	41.554	39.189	Araip.HJX61	0.437
Aradu.19EUZ	0.387	620	52.335	42.351	47.987	Araip.C9I3T	0.387
Aradu.19W8X	0.393	913	52.516	37.637	36.98	Araip.V731N	0.394
Aradu.19YSM	0.356	500	47.305	39.321	43.713	Araip.XY52C	0.333
Aradu.19ZIS	0.407	427	48.131	42.991	43.692	Araip.G42NL	0.412
Aradu.1A2PM	0.435	198	54.271	50.251	46.734	Araip.KT98T	0.457
Aradu.1A2XY	0.369	586	48.382	40.716	42.078	Araip.6I3DS	0.379
Aradu.1A3FB	0.363	411	52.913	41.99	41.262	Araip.3YS8U	0.362
Aradu.1AJ4F	0.411	350	53.276	35.897	46.154	Araip.ZUP60	0.408
Aradu.1AM05	0.419	493	51.822	42.51	49.798	Araip.ZXR6U	0.414
Aradu.1B2LG	0.384	532	48.405	38.086	42.964	Araip.NY5B8	0.375
Aradu.1B3IN	0.316	179	47.778	31.667	28.333	Araip.W1EIB	0.322
Aradu.1BC5C	0.371	304	48.525	37.705	36.393	Araip.DN5WT	0.41
Aradu.1C5Z6	0.416	488	46.626	46.83	43.558	Araip.S0S72	0.425
Aradu.1C7BQ	0.374	358	51.253	38.162	37.047	Araip.Z7A9P	0.363
Aradu.1C8I8	0.408	127	35.938	32.813	33.594	Araip.43KWH	0.358
Aradu.1CN3T	0.455	449	54	39.556	64.444	Araip.9W9E2	0.457
Aradu.1CY96	0.394	502	53.082	46.918	38.966	Araip.4C08I	0.407
Aradu.1D15U	0.355	371	47.849	38.172	40.591	Araip.5V6AL	0.343
Aradu.1D399	0.384	555	46.583	37.95	30.396	Araip.QI1EB	0.393
Aradu.1D3EG	0.454	172	59.538	46.821	47.977	Araip.RN11M	0.454
Aradu.1D7KB	0.435	359	44.722	39.722	54.167	Araip.YWU2K	0.424
Aradu.1D83F	0.313	724	49.793	37.931	35.586	Araip.YY5JE	0.314
Aradu.1DA34	0.368	445	51.345	44.395	35.874	Araip.Q9V7K	0.37
Aradu.1DJ56	0.382	357	50.559	44.693	48.045	Araip.Q6AH4	0.386
Aradu.1DT27	0.416	223	49.554	41.518	40.625	Araip.I1FHG	0.408
Aradu.1DV9M	0.403	250	58.566	40.239	37.849	Araip.1G7Y9	0.399
Aradu.1E0KB	0.477	290	46.735	43.986	52.577	Araip.DE8MF	0.483
Aradu.1E0WP	0.393	698	49.356	43.491	37.911	Araip.PLC3S	0.391
Aradu.1E1I2	0.356	669	51.194	37.91	35.821	Araip.I1B5X	0.349

Aradu.1E1V6	0.372	544	51.743	40.183	37.431	Araip.F1PS1	0.375
Aradu.1E9H5	0.398	610	45.827	42.226	43.372	Araip.Y83VE	0.394
Aradu.1EC42	0.391	322	45.82	39.319	43.653	Araip.1S1ZZ	0.416
Aradu.1ED5Z	0.431	244	59.592	55.102	46.939	Araip.TJ4SI	0.431
Aradu.1EH3D	0.421	176	44.633	44.633	45.198	Araip.X14G7	0.468
Aradu.1EH7R	0.498	234	47.234	44.681	48.511	Araip.7N70L	0.475
Aradu.1EI01	0.395	771	53.238	42.098	37.824	Araip.FJ25G	0.381
Aradu.1ES3P	0.377	431	54.63	44.907	38.426	Araip.JE9JZ	0.37
Aradu.1F4NN	0.339	501	46.016	38.845	34.064	Araip.ZI8DI	0.349
Aradu.1F5AZ	0.357	400	47.382	42.643	45.137	Araip.A0U1I	0.367
Aradu.1FC14	0.36	426	46.604	38.876	36.768	Araip.1VK73	0.362
Aradu.1FD3D	0.335	447	44.196	39.286	45.536	Araip.V9RIN	0.343
Aradu.1FM9H	0.375	688	42.38	39.478	38.171	Araip.XN7XL	0.369
Aradu.1FQ73	0.352	407	60.049	39.216	43.873	Araip.HL18M	0.354
Aradu.1G3YF	0.452	816	53.488	47.858	57.038	Araip.JQ9KB	0.457
Aradu.1G4QF	0.453	477	55.649	42.05	38.494	Araip.7D543	0.465
Aradu.1G6QS	0.386	610	58.429	44.517	39.771	Araip.2A3ZP	0.401
Aradu.1GI8T	0.373	121	45.902	39.344	37.705	Araip.9U1PK	0.359
Aradu.1H2UR	0.423	252	52.964	47.036	56.126	Araip.M07D2	0.479
Aradu.1H4CB	0.376	559	52.143	37.679	36.071	Araip.JYQ4Z	0.381
Aradu.1HN9G	0.347	205	49.515	35.437	40.291	Araip.B3CY6	0.347
Aradu.1HQ4L	0.371	1002	46.959	38.784	38.684	Araip.Q0N0W	0.365
Aradu.1HX6J	0.403	436	45.538	42.563	42.334	Araip.8A3C5	0.397
Aradu.1I015	0.368	532	56.098	36.585	49.719	Araip.6E6I2	0.369
Aradu.1I2B8	0.428	771	55.311	39.119	41.062	Araip.GVH79	0.415
Aradu.1I5UA	0.401	283	48.239	41.901	50.704	Araip.RFS9X	0.397
Aradu.1I5W4	0.375	461	46.97	40.26	32.9	Araip.M4D5S	0.368
Aradu.1I7E9	0.359	177	53.371	44.944	38.202	Araip.T1SIZ	0.359
Aradu.1IC89	0.35	989	50.101	32.929	41.01	Araip.M8TU7	0.347
Aradu.1IK7L	0.398	354	45.07	39.437	43.944	Araip.9FT0W	0.407
Aradu.1IM7V	0.388	812	50.308	37.638	36.531	Araip.G9RMH	0.392
Aradu.1J0IW	0.335	233	49.573	39.744	42.735	Araip.HWJ4T	0.345
Aradu.1J1DY	0.343	306	43.974	40.391	35.831	Araip.H0H56	0.331
Aradu.1J1TW	0.427	336	59.05	45.697	54.599	Araip.GZL5I	0.435
Aradu.1J279	0.408	384	55.844	43.377	42.338	Araip.VAZ0T	0.384
Aradu.1J3FJ	0.325	567	53.873	37.148	41.373	Araip.92EP4	0.33
Aradu.1JK1L	0.345	622	57.143	39.005	40.61	Araip.Q3Y4B	0.34
Aradu.1JK2E	0.382	420	50.594	38.005	44.656	Araip.G9K0U	0.387
Aradu.1JP2B	0.379	2419	57.521	39.504	37.355	Araip.61FFX	0.381
Aradu.1K1MI	0.364	341	49.123	36.55	44.152	Araip.PK50H	0.361
Aradu.1L0P8	0.363	942	43.372	34.252	34.464	Araip.C84JH	0.36
Aradu.1L0UP	0.421	323	53.086	37.037	37.654	Araip.D7K2H	0.441
Aradu.1LG94	0.354	908	50.495	37.624	38.614	Araip.4CU64	0.352
Aradu.1LI5S	0.447	237	52.521	44.958	49.58	Araip.I5MUZ	0.452

Aradu.1LM0G	0.382	296	53.199	41.414	38.384	Araip.S8ZYY	0.378
Aradu.1LQ1Q	0.339	343	46.221	43.023	44.477	Araip.SWD4L	0.355
Aradu.1LZ8T	0.477	597	52.174	40.468	60.033	Araip.PP5S8	0.47
Aradu.1M0CF	0.421	494	51.111	35.96	47.273	Araip.78XNL	0.415
Aradu.1M0CG	0.378	316	54.574	41.009	29.338	Araip.AL63T	0.361
Aradu.1M2X1	0.466	265	58.647	44.361	51.88	Araip.Y2HKR	0.47
Aradu.1M38D	0.345	154	52.258	23.871	59.355	Araip.CW72W	0.4
Aradu.1M9DY	0.381	263	48.106	37.121	45.455	Araip.CVB1D	0.401
Aradu.1M9NC	0.402	1819	52.967	30.769	35.275	Araip.04KZI	0.396
Aradu.1N8DM	0.431	509	53.333	37.843	45.294	Araip.TWL2H	0.425
Aradu.1N9W3	0.444	222	56.054	41.256	38.117	Araip.4U5VW	0.444
Aradu.1NK9R	0.426	113	37.719	38.596	45.614	Araip.M52V7	0.426
Aradu.1P1D6	0.356	426	53.396	40.515	35.597	Araip.V9JLA	0.342
Aradu.1P5GD	0.347	808	49.32	43.14	34.734	Araip.GAD8U	0.349
Aradu.1P8DE	0.359	366	48.501	48.229	45.232	Araip.EY89K	0.359
Aradu.1PQ4F	0.425	277	47.122	33.813	50	Araip.T0L79	0.399
Aradu.1Q100	0.441	231	51.293	35.776	46.552	Araip.QR5JJ	0.441
Aradu.1QD6E	0.42	330	47.734	48.338	35.65	Araip.1S76R	0.407
Aradu.1QN19	0.348	379	45.526	36.842	30.263	Araip.9H3WY	0.345
Aradu.1QU0K	0.354	707	51.836	39.124	32.91	Araip.T61X4	0.35
Aradu.1QU40	0.433	240	45.643	39.004	50.207	Araip.SWI1N	0.44
Aradu.1QV2U	0.409	284	52.281	36.491	45.263	Araip.7Z8D2	0.392
Aradu.1R3I6	0.514	213	49.065	40.187	63.084	Araip.T4LYM	0.527
Aradu.1R4IH	0.351	601	47.508	42.193	38.704	Araip.8E1SN	0.343
Aradu.1R5WG	0.398	601	52.159	44.352	43.854	Araip.D3BJQ	0.391
Aradu.1R7R2	0.376	106	51.402	30.841	41.121	Araip.9634I	0.386
Aradu.1RK1Y	0.369	513	51.556	34.63	34.63	Araip.0N0ZT	0.369
Aradu.1SD0Q	0.382	626	49.761	36.204	42.584	Araip.V1BCZ	0.368
Aradu.1SS2C	0.443	212	55.869	29.577	46.009	Araip.6B9LC	0.443
Aradu.1T2VN	0.41	658	52.2	41.275	49.621	Araip.EX9I5	0.418
Aradu.1T3UD	0.414	194	62.051	48.205	62.564	Araip.NFP9Z	0.444
Aradu.1T3XU	0.33	618	48.142	43.619	39.418	Araip.R06WH	0.337
Aradu.1TB02	0.41	733	54.496	46.049	41.417	Araip.SVX3P	0.405
Aradu.1TF3W	0.394	636	49.922	35.793	43.799	Araip.S1X2Y	0.405
Aradu.1TX9B	0.375	414	45.783	40.723	37.831	Araip.RG2CK	0.364
Aradu.1U41E	0.387	854	45.146	32.398	41.287	Araip.T165J	0.391
Aradu.1U59X	0.367	277	50.36	43.885	39.568	Araip.W0D6Y	0.384
Aradu.1UD93	0.443	204	48.78	35.61	52.195	Araip.S4KNL	0.443
Aradu.1UI8P	0.377	345	45.954	36.994	34.682	Araip.IJN8L	0.377
Aradu.1V1PL	0.388	410	51.825	45.499	46.959	Araip.EZI68	0.389
Aradu.1VB35	0.398	532	51.407	43.902	37.711	Araip.F3XKF	0.41
Aradu.1W5FD	0.357	357	44.413	40.223	47.207	Araip.Q2JPR	0.362
Aradu.1W7NB	0.405	266	52.06	30.712	38.951	Araip.KPI1D	0.403
Aradu.1WD61	0.299	433	44.24	31.797	27.88	Araip.XWP57	0.297

Aradu.1WP35	0.329	155	47.436	30.769	37.821	Araip.Y32G0	0.331
Aradu.1WT6A	0.397	192	50.259	36.269	54.922	Araip.UKM5Q	0.407
Aradu.1WV86	0.344	132	49.624	58.647	41.353	Araip.LCC0J	0.321
Aradu.1X2PC	0.493	206	45.894	42.029	59.903	Araip.VD076	0.502
Aradu.1X6G8	0.413	517	55.598	37.452	56.371	Araip.T9LT0	0.408
Aradu.1X6Z1	0.446	103	48.077	51.923	48.077	Araip.T84RU	0.455
Aradu.1XB3Y	0.366	430	57.309	36.891	44.548	Araip.B118X	0.354
Aradu.1Y370	0.299	426	43.794	36.534	34.426	Araip.ED1H8	0.3
Aradu.1Y660	0.376	628	49.921	35.294	42.607	Araip.00X39	0.37
Aradu.1Y87X	0.477	530	46.704	41.055	64.972	Araip.WMX3R	0.486
Aradu.1Z30Z	0.331	550	45.735	34.483	37.387	Araip.4L730	0.33
Aradu.1Z4K3	0.398	254	56.078	42.353	41.961	Araip.R452I	0.385
Aradu.1Z587	0.388	483	49.174	45.248	48.347	Araip.64E4D	0.381
Aradu.1Z7PM	0.333	587	43.367	39.626	39.286	Araip.3JH87	0.338
Aradu.1Z96C	0.396	663	51.506	35.843	41.867	Araip.97WJC	0.393
Aradu.1ZJ1C	0.419	177	55.618	37.079	60.112	Araip.WW8ZI	0.41
Aradu.1ZL4G	0.367	681	46.481	36.07	41.202	Araip.H1ACH	0.369
Aradu.1ZQ3K	0.398	510	42.27	37.182	39.726	Araip.KET11	0.394
Aradu.1ZR6L	0.395	295	48.649	37.838	39.527	Araip.CFW6I	0.395
Aradu.200CK	0.441	130	56.489	35.115	57.252	Araip.AQ14G	0.449
Aradu.200KX	0.354	687	49.855	40.407	36.919	Araip.EDC64	0.347
Aradu.206DU	0.356	507	48.031	35.433	34.843	Araip.L9NXN	0.36
Aradu.206YZ	0.374	1730	51.531	39.919	35.009	Araip.S8FK3	0.373
Aradu.2088W	0.365	183	52.174	34.239	46.196	Araip.J5BBQ	0.382
Aradu.209EV	0.364	756	50.859	38.441	41.48	Araip.Q76UC	0.365
Aradu.20BZ5	0.348	1398	54.825	38.885	33.024	Araip.51T0U	0.355
Aradu.20C0X	0.487	277	48.921	44.604	50.36	Araip.HTQ91	0.484
Aradu.20EC4	0.316	102	52.427	43.689	58.252	Araip.G6E5V	0.316
Aradu.20FIR	0.369	851	53.521	36.854	38.498	Araip.BC51L	0.373
Aradu.20IMG	0.391	470	57.962	38.429	54.565	Araip.3F9PG	0.4
Aradu.20KIH	0.326	452	41.28	40.397	31.567	Araip.BQC59	0.314
Aradu.20QKH	0.359	1142	57.568	36.308	32.721	Araip.AYN5N	0.364
Aradu.20TYF	0.379	284	46.667	43.509	47.719	Araip.V76CX	0.361
Aradu.2119N	0.408	933	43.576	34.154	40.578	Araip.69YUJ	0.42
Aradu.2123V	0.406	380	56.43	42.52	43.57	Araip.XJA18	0.401
Aradu.212BQ	0.412	760	48.226	41.261	36.662	Araip.E0W6R	0.411
Aradu.212W8	0.492	672	48.143	41.01	47.4	Araip.A6VYR	0.483
Aradu.215DG	0.322	322	49.536	34.985	38.7	Araip.1N7IP	0.339
Aradu.21FR3	0.412	491	55.081	37.602	43.496	Araip.B72LY	0.408
Aradu.22647	0.366	274	43.636	37.091	41.818	Araip.UA0W9	0.363
Aradu.22650	0.375	395	54.04	41.919	37.879	Araip.RL3D3	0.36
Aradu.227K7	0.342	602	49.254	46.6	38.806	Araip.AHF5C	0.345
Aradu.22AJD	0.421	325	54.601	38.957	53.681	Araip.SI1NJ	0.418
Aradu.22BDB	0.389	503	53.968	41.667	40.873	Araip.X17SE	0.381

Aradu.22C31	0.372	334	54.328	39.403	41.791	Araip.GYE3T	0.387
Aradu.22C3R	0.36	682	48.902	42.46	37.335	Araip.MW4HG	0.364
Aradu.22C6J	0.5	504	55.842	40	59.406	Araip.ZZ5S6	0.493
Aradu.22IYP	0.443	853	50.117	40.515	57.26	Araip.225U5	0.44
Aradu.22KJ4	0.37	240	47.303	38.589	49.793	Araip.X2CY1	0.361
Aradu.22L4T	0.377	416	48.201	39.089	34.532	Araip.1LA66	0.387
Aradu.22RTM	0.449	460	55.748	39.046	39.046	Araip.T4YQW	0.452
Aradu.22X1P	0.427	227	48.684	32.895	52.193	Araip.RE3SL	0.417
Aradu.22ZEB	0.479	721	58.864	41.274	54.709	Araip.AP8LU	0.466
Aradu.22ZWX	0.43	507	48.622	40.354	42.52	Araip.IE5MN	0.428
Aradu.230WM	0.439	219	53.182	36.364	58.636	Araip.32U31	0.443
Aradu.23179	0.348	169	58.235	40	44.706	Araip.V0B0S	0.373
Aradu.232FJ	0.358	330	45.317	38.671	42.296	Araip.EN1G0	0.358
Aradu.234X6	0.482	407	51.716	43.382	57.353	Araip.2Q3AI	0.477
Aradu.235VA	0.398	432	40.878	36.721	40.416	Araip.54D8D	0.391
Aradu.237HP	0.392	313	46.178	35.669	40.127	Araip.2Q2JE	0.399
Aradu.23D9S	0.377	411	49.515	40.291	34.709	Araip.RB9NR	0.357
Aradu.23E3L	0.331	513	45.914	36.576	35.409	Araip.DD0BF	0.326
Aradu.23FX2	0.44	586	51.959	43.271	55.026	Araip.J4L5F	0.432
Aradu.23Z4K	0.367	1146	52.746	42.11	40.279	Araip.W7597	0.364
Aradu.23ZKM	0.405	297	52.013	40.268	35.906	Araip.Q87QY	0.399
Aradu.2447B	0.403	891	51.009	30.942	39.686	Araip.FX6KA	0.403
Aradu.2448N	0.369	359	54.444	36.389	40.833	Araip.71Z7T	0.364
Aradu.245YW	0.371	354	45.634	38.028	34.93	Araip.NN0H7	0.347
Aradu.24BEK	0.353	493	50.405	37.045	33.401	Araip.G37T0	0.359
Aradu.24BNI	0.457	356	56.863	33.053	42.857	Araip.CBT0Y	0.439
Aradu.24DGD	0.419	509	50.784	38.039	46.078	Araip.DE4L0	0.412
Aradu.24I8J	0.37	606	50.741	37.562	44.152	Araip.3B8VN	0.365
Aradu.24LGE	0.426	421	54.502	40.758	56.872	Araip.TVQ9L	0.425
Aradu.24PXZ	0.34	259	50.769	38.462	34.231	Araip.IMW9T	0.356
Aradu.24SCU	0.363	244	44.898	37.551	41.224	Araip.U33C2	0.363
Aradu.24X5V	0.375	486	45.585	43.326	34.292	Araip.EY2N6	0.375
Aradu.24YRC	0.433	210	56.398	40.284	61.611	Araip.FYI8V	0.428
Aradu.24ZSJ	0.351	344	50.145	45.507	34.493	Araip.Y9V2V	0.357
Aradu.2503N	0.469	406	56.511	45.7	67.322	Araip.VG3DT	0.477
Aradu.250G2	0.381	436	54.005	36.842	34.096	Araip.FXZ9G	0.376
Aradu.251QA	0.414	588	51.104	37.182	43.633	Araip.N3G6M	0.42
Aradu.254QE	0.379	923	57.143	49.134	32.251	Araip.Z3KJR	0.379
Aradu.254Z6	0.383	625	47.923	36.422	36.741	Araip.EV6LQ	0.38
Aradu.257BX	0.368	160	48.447	41.615	40.373	Araip.1R8Y6	0.368
Aradu.2597P	0.415	128	41.085	52.713	51.938	Araip.Y64TL	0.402
Aradu.25DC4	0.347	549	46.182	39.273	38.909	Araip.6749X	0.331
Aradu.25DKA	0.237	121	40.164	56.557	33.607	Araip.Z4ATC	0.231
Aradu.25F61	0.376	313	50.318	47.134	38.217	Araip.X8TMK	0.369

Aradu.25H68	0.417	666	47.226	43.628	46.327	Araip.JW3M4	0.426
Aradu.25L3E	0.376	965	50	35.3	33.851	Araip.154H4	0.375
Aradu.25NPV	0.42	412	47.458	46.489	39.467	Araip.6K1VA	0.418
Aradu.25XYL	0.268	547	43.613	40.511	34.124	Araip.3TN6J	0.269
Aradu.26288	0.38	451	44.248	40.265	34.735	Araip.27UQJ	0.38
Aradu.267PI	0.384	780	49.552	40.205	36.748	Araip.F91RT	0.404
Aradu.268F1	0.375	544	53.761	40	49.541	Araip.F6KVE	0.384
Aradu.26IB4	0.344	185	44.624	42.473	36.022	Araip.CUS24	0.35
Aradu.26MHM	0.414	239	47.083	40	35.417	Araip.G2Y3C	0.405
Aradu.26N4W	0.414	480	54.262	43.659	34.304	Araip.0U4IT	0.403
Aradu.26N74	0.423	559	47.857	38.393	45.714	Araip.VB5WD	0.421
Aradu.270HA	0.362	271	46.691	39.338	43.75	Araip.WJB0A	0.366
Aradu.270YY	0.42	146	43.537	48.98	51.701	Araip.L7AM8	0.42
Aradu.2717A	0.372	510	52.25	43.249	45.401	Araip.5RN6F	0.369
Aradu.272PC	0.458	377	50.529	38.36	62.963	Araip.1C20S	0.464
Aradu.27DYI	0.387	569	48.421	39.474	41.228	Araip.PW5DF	0.385
Aradu.27F3R	0.347	224	51.111	40.444	37.333	Araip.217TR	0.338
Aradu.27SYD	0.404	300	43.189	40.532	39.867	Araip.WY1NK	0.394
Aradu.27T3V	0.404	1061	49.435	39.736	43.691	Araip.L8Z1N	0.409
Aradu.27VHF	0.379	231	52.155	45.69	43.534	Araip.UF3L0	0.375
Aradu.27WCK	0.393	465	50.644	39.7	45.494	Araip.8R0Z7	0.384
Aradu.27XB8	0.356	922	56.338	37.486	36.511	Araip.URJ02	0.354
Aradu.27Y2W	0.377	411	45.631	37.864	40.534	Araip.71FLX	0.387
Aradu.286YF	0.438	129	65.385	58.462	32.308	Araip.H5KZZ	0.43
Aradu.288JJ	0.388	597	53.512	39.298	36.957	Araip.3485Y	0.387
Aradu.289R9	0.367	370	51.752	40.162	39.353	Araip.ZJ40R	0.37
Aradu.28IY3	0.364	657	41.033	39.514	38.906	Araip.DBK4N	0.368
Aradu.28KIR	0.319	235	39.407	36.864	43.22	Araip.LSW2G	0.319
Aradu.28KTI	0.453	293	46.599	50.34	61.224	Araip.HCG04	0.438
Aradu.28N0X	0.321	275	46.739	38.406	44.928	Araip.UDU9G	0.324
Aradu.28PRF	0.406	382	52.219	46.214	48.825	Araip.RMX8U	0.392
Aradu.28WLZ	0.516	260	46.743	52.49	60.153	Araip.B56B4	0.529
Aradu.292D3	0.362	1311	49.619	34.07	41.463	Araip.G5CUD	0.367
Aradu.295V7	0.337	433	40.783	38.94	39.631	Araip.FC9LL	0.358
Aradu.296RJ	0.387	547	52.737	42.153	41.606	Araip.G4AFR	0.392
Aradu.296X5	0.415	616	48.784	42.788	54.133	Araip.YBJ1A	0.403
Aradu.298AN	0.314	433	41.475	36.406	41.244	Araip.PYR7K	0.32
Aradu.29A27	0.344	222	43.498	38.565	37.668	Araip.510K0	0.34
Aradu.29ERF	0.406	501	51.594	37.052	44.821	Araip.BJ1LC	0.398
Aradu.29IAA	0.363	1318	53.45	39.272	35.027	Araip.JV3XS	0.362
Aradu.29QNA	0.406	278	59.857	41.577	39.427	Araip.E96SM	0.387
Aradu.29VJC	0.447	231	50.431	48.276	43.103	Araip.8AR8R	0.451
Aradu.2A606	0.331	121	48.361	36.066	31.967	Araip.MY1RF	0.339
Aradu.2A8ZQ	0.336	1005	45.03	32.207	35.189	Araip.MU8XA	0.348

Aradu.2AQ5X	0.293	121	38.525	40.164	44.262	Araip.BYN6L	0.31
Aradu.2AU2Z	0.368	473	48.312	35.654	37.764	Araip.C9HSJ	0.364
Aradu.2B9FT	0.404	300	48.505	38.87	37.542	Araip.LL9X6	0.4
Aradu.2BE4F	0.398	267	55.224	44.776	36.194	Araip.CV72U	0.409
Aradu.2BI47	0.42	325	51.227	42.945	44.172	Araip.BKI6W	0.426
Aradu.2BI4W	0.366	1465	46.862	36.494	39.086	Araip.FP1WW	0.364
Aradu.2C28W	0.367	826	53.204	39.782	29.383	Araip.W0AVN	0.371
Aradu.2C8QX	0.4	329	60.909	31.515	47.273	Araip.95M6U	0.4
Aradu.2C9WA	0.392	160	50.311	34.161	44.099	Araip.GYU0T	0.386
Aradu.2CB86	0.399	452	44.371	45.695	42.163	Araip.N94NJ	0.396
Aradu.2CJ0B	0.362	530	48.964	41.431	31.073	Araip.N63X6	0.35
Aradu.2CK5F	0.377	165	53.614	36.747	48.193	Araip.34N7T	0.355
Aradu.2DG1V	0.365	612	51.387	37.52	40.457	Araip.JIW1A	0.349
Aradu.2DM5J	0.391	473	58.017	42.827	54.219	Araip.CJ9D7	0.393
Aradu.2E34B	0.396	294	48.814	29.492	39.322	Araip.4NC09	0.395
Aradu.2E3F6	0.346	400	52.618	41.646	39.152	Araip.0Y9HM	0.354
Aradu.2EU9J	0.389	170	47.368	32.749	45.029	Araip.B3EI3	0.393
Aradu.2F17T	0.405	397	55.528	46.482	42.462	Araip.W5ZI5	0.389
Aradu.2F2F8	0.401	261	50.763	34.351	39.695	Araip.00QX5	0.401
Aradu.2F99R	0.399	692	52.237	40.693	41.847	Araip.0J4UH	0.408
Aradu.2FM0G	0.346	414	51.807	39.518	43.133	Araip.A674Q	0.344
Aradu.2GB7A	0.393	471	51.907	43.22	48.305	Araip.IS8T3	0.388
Aradu.2GH9Y	0.417	500	54.691	34.93	56.687	Araip.FRU70	0.43
Aradu.2H1GD	0.335	351	54.261	36.932	46.307	Araip.FBK18	0.356
Aradu.2H1TE	0.376	955	52.197	40.586	45.921	Araip.75CVM	0.381
Aradu.2HD04	0.391	215	44.907	41.204	40.278	Araip.274S3	0.401
Aradu.2HJ80	0.449	373	54.545	40.642	46.257	Araip.NU2XV	0.46
Aradu.2HT1T	0.357	782	47.637	35.632	40.23	Araip.PX6UV	0.358
Aradu.2I1UD	0.283	113	50	25.439	38.596	Araip.IYB9Y	0.292
Aradu.2I4G0	0.425	296	47.811	51.178	47.475	Araip.B06YF	0.416
Aradu.2IG4B	0.374	556	44.165	42.908	39.677	Araip.AZH1U	0.391
Aradu.2IP37	0.436	351	49.716	39.773	41.477	Araip.KWB60	0.432
Aradu.2J2S3	0.346	298	50.167	32.107	36.12	Araip.BH9LK	0.331
Aradu.2J3VX	0.349	778	49.807	40.822	39.153	Araip.F86X8	0.35
Aradu.2J9A4	0.402	1021	55.871	41.977	40.411	Araip.8F4W7	0.399
Aradu.2JA77	0.335	350	51.282	43.59	41.026	Araip.SD9JV	0.32
Aradu.2JF44	0.363	440	53.515	36.508	47.392	Araip.ZR7N4	0.371
Aradu.2JQ32	0.484	326	47.401	43.119	65.443	Araip.EF1SW	0.52
Aradu.2K04Y	0.434	136	56.934	50.365	54.015	Araip.7HP1R	0.43
Aradu.2K3I2	0.37	1056	50.331	37.275	44.182	Araip.A1F6W	0.371
Aradu.2K88G	0.425	130	60.305	51.145	39.695	Araip.R3Y0S	0.433
Aradu.2KV4N	0.406	330	58.308	42.598	62.538	Araip.G7W3J	0.416
Aradu.2L1WI	0.397	541	48.893	45.387	35.055	Araip.3UE1W	0.404
Aradu.2L1YZ	0.445	230	57.576	50.216	49.784	Araip.NDM7B	0.466

Aradu.2L2SE	0.392	858	55.763	41.327	63.795	Araip.PN6DH	0.395
Aradu.2LE9C	0.374	324	48.615	37.231	41.231	Araip.D7U08	0.373
Aradu.2LH1B	0.365	910	61.361	38.749	44.457	Araip.27Q72	0.369
Aradu.2M3LR	0.333	407	45.343	39.216	39.706	Araip.6L6ZR	0.327
Aradu.2MS2X	0.319	127	53.906	30.469	36.719	Araip.Z2ASR	0.319
Aradu.2N8AH	0.363	627	55.573	43.631	43.79	Araip.GS1GM	0.355
Aradu.2NY77	0.371	550	52.087	40.472	40.29	Araip.5L4N3	0.373
Aradu.2P1NS	0.409	244	50.204	37.551	38.367	Araip.4B6XP	0.405
Aradu.2P8EW	0.381	527	51.894	41.477	38.068	Araip.2W8EJ	0.38
Aradu.2P8HG	0.362	526	48.956	43.454	42.884	Araip.JQ4V7	0.367
Aradu.2P97F	0.373	1206	57.995	39.685	32.56	Araip.W60W5	0.366
Aradu.2PT1E	0.356	523	47.519	38.74	37.023	Araip.953FR	0.346
Aradu.2Q1S0	0.377	420	48.931	37.767	39.667	Araip.5YY4M	0.391
Aradu.2Q2R1	0.325	480	49.48	38.669	51.559	Araip.FD3D0	0.325
Aradu.2Q562	0.408	508	49.509	45.187	35.167	Araip.LWG2P	0.413
Aradu.2Q7FP	0.395	197	47.98	47.98	38.384	Araip.AH9LJ	0.395
Aradu.2QG6G	0.37	453	50.22	42.291	49.78	Araip.ZI6GM	0.386
Aradu.2QR5U	0.357	513	49.611	34.825	40.467	Araip.EC2BK	0.345
Aradu.2QS2R	0.386	640	48.05	39.47	44.618	Araip.UT6J6	0.388
Aradu.2QX0T	0.373	377	45.767	29.894	29.894	Araip.94GCY	0.363
Aradu.2R2WH	0.391	682	51.977	33.675	39.531	Araip.7E7XY	0.388
Aradu.2R9BM	0.38	282	43.11	37.809	46.29	Araip.G1IA2	0.39
Aradu.2RC64	0.377	428	50.117	36.364	46.387	Araip.I88R2	0.388
Aradu.2RS8H	0.415	377	52.116	50.794	37.831	Araip.Z7NW6	0.42
Aradu.2RV29	0.427	544	47.706	35.78	57.248	Araip.0G32G	0.433
Aradu.2S5CB	0.383	325	39.877	37.117	41.104	Araip.UGG4B	0.389
Aradu.2SV86	0.394	550	55.172	41.016	41.016	Araip.26UP8	0.392
Aradu.2T85U	0.36	794	52.579	43.396	49.182	Araip.0J7GI	0.357
Aradu.2T909	0.507	145	52.74	36.986	61.644	Araip.KVQ91	0.514
Aradu.2TK0M	0.386	367	39.946	35.87	45.652	Araip.VTQ3M	0.376
Aradu.2U1V2	0.325	395	48.737	35.354	37.121	Araip.86VQJ	0.336
Aradu.2U2Q6	0.403	120	46.281	43.802	57.025	Araip.MF53E	0.403
Aradu.2UI08	0.381	436	51.945	43.478	46.682	Araip.S6BCW	0.38
Aradu.2UZ0C	0.404	443	48.649	36.486	37.613	Araip.3TF2U	0.404
Aradu.2V0TP	0.408	181	50	38.462	58.791	Araip.TL5BE	0.399
Aradu.2V3YU	0.389	726	56.534	38.514	41.265	Araip.X2AQN	0.381
Aradu.2V4EQ	0.361	313	41.72	35.987	32.803	Araip.AR7FU	0.362
Aradu.2V69M	0.358	2359	51.568	41.78	35.72	Araip.8RS63	0.36
Aradu.2W1AJ	0.395	600	44.592	38.769	35.774	Araip.ZR9LA	0.401
Aradu.2W21G	0.376	862	48.899	40.324	38.355	Araip.WF117	0.378
Aradu.2W9TG	0.364	403	41.337	38.614	39.109	Araip.W0U7G	0.365
Aradu.2X0TU	0.377	587	50.68	43.197	49.49	Araip.C9B5T	0.388
Aradu.2X0ZQ	0.389	919	56.087	45.109	34.674	Araip.JVY0J	0.392
Aradu.2X4WG	0.438	355	52.247	33.427	47.191	Araip.2LP4Y	0.423

Aradu.2X54C	0.342	503	50.794	41.468	34.921	Araip.R0RXM	0.35
Aradu.2Y43U	0.348	536	53.445	43.575	37.058	Araip.493E4	0.362
Aradu.2Y48Q	0.399	1665	45.318	34.094	48.92	Araip.BH4UN	0.403
Aradu.2Y4H2	0.355	281	52.482	45.39	42.908	Araip.MY16C	0.358
Aradu.2YF2R	0.436	198	50.754	53.266	54.271	Araip.BV8FB	0.423
Aradu.2YY9U	0.365	1454	49.966	38.419	36.082	Araip.QHR4V	0.366
Aradu.2Z4NE	0.385	603	46.854	41.225	36.258	Araip.SR6HQ	0.386
Aradu.2Z52F	0.325	293	43.537	41.497	35.374	Araip.TJ2TW	0.324
Aradu.2Z821	0.422	563	49.113	40.603	54.787	Araip.B2EFG	0.422
Aradu.2Z89F	0.364	534	50.093	36.075	43.925	Araip.864M6	0.346
Aradu.2ZT9L	0.343	662	56.712	42.836	34.389	Araip.ID1ZZ	0.35
Aradu.301B3	0.368	403	50.99	38.861	37.871	Araip.BK1UX	0.375
Aradu.301P6	0.365	200	48.259	41.791	52.736	Araip.28HGD	0.38
Aradu.304FM	0.433	323	56.173	35.494	50.617	Araip.K5A40	0.434
Aradu.305W9	0.398	928	52.745	41.981	44.779	Araip.JY0X0	0.394
Aradu.306G1	0.373	467	56.197	39.957	51.923	Araip.ASH11	0.376
Aradu.30RBD	0.399	293	50.68	36.054	43.878	Araip.1HR72	0.402
Aradu.30RPU	0.33	365	40.71	41.257	39.344	Araip.07BW1	0.336
Aradu.30TWS	0.39	986	47.822	37.893	39.818	Araip.SKT5W	0.382
Aradu.3127Y	0.427	524	52.381	30.095	58.476	Araip.V6XPZ	0.427
Aradu.315HW	0.399	440	52.154	50.567	34.694	Araip.6L19K	0.385
Aradu.316F6	0.414	352	50.708	42.493	40.51	Araip.8VP2X	0.404
Aradu.317LS	0.429	360	48.476	45.706	55.402	Araip.149YW	0.426
Aradu.31H7A	0.338	498	44.689	39.88	39.279	Araip.99AMZ	0.344
Aradu.31IUC	0.355	609	50.984	38.361	52.623	Araip.TKL7Q	0.35
Aradu.31IVL	0.311	107	55.556	35.185	37.963	Araip.H4E0W	0.301
Aradu.31PM4	0.355	681	53.519	36.217	36.657	Araip.M87GM	0.364
Aradu.31RFE	0.327	173	59.77	34.483	32.184	Araip.E5NHA	0.333
Aradu.31VER	0.411	435	54.817	39.679	47.018	Araip.RF1QP	0.406
Aradu.31VP0	0.326	469	51.489	40	40.851	Araip.33SF4	0.324
Aradu.31ZWP	0.417	356	53.782	43.978	38.655	Araip.NA763	0.411
Aradu.320AL	0.341	563	45.213	33.333	43.617	Araip.I1KV0	0.344
Aradu.32310	0.418	397	57.286	48.995	42.462	Araip.JHV8K	0.382
Aradu.329PQ	0.508	642	42.924	46.812	61.431	Araip.957AT	0.525
Aradu.32A1K	0.434	193	45.876	36.598	32.474	Araip.7N2TS	0.429
Aradu.32A9T	0.369	1363	52.419	46.188	34.311	Araip.L30PN	0.374
Aradu.32DSM	0.503	177	55.056	48.315	62.36	Araip.88P7F	0.503
Aradu.32EAS	0.373	530	51.036	37.665	34.652	Araip.JP7VM	0.37
Aradu.32ETH	0.342	1073	50	38.547	40.41	Araip.GQ2VC	0.35
Aradu.32QV2	0.313	476	48.008	33.753	39.832	Araip.88UBN	0.313
Aradu.32TSS	0.484	133	58.955	40.299	57.463	Araip.QU4D9	0.484
Aradu.32V7X	0.426	326	49.541	44.343	39.45	Araip.V3PK4	0.418
Aradu.32X0N	0.407	537	52.974	43.866	60.409	Araip.560IW	0.396
Aradu.32ZUX	0.425	363	45.604	51.923	41.758	Araip.73IW9	0.428

Aradu.33DTZ	0.351	539	44.815	33.889	43.333	Araip.TV6SE	0.358
Aradu.33G7Z	0.413	401	52.736	34.577	37.313	Araip.GE4SS	0.395
Aradu.33Q41	0.427	387	60.825	43.299	42.784	Araip.XD8CG	0.411
Aradu.33ULW	0.346	156	52.229	33.121	36.306	Araip.B52UH	0.342
Aradu.33VFZ	0.384	339	43.529	36.176	44.118	Araip.0XT2W	0.371
Aradu.33VZ6	0.377	208	46.89	44.019	34.45	Araip.H94QL	0.372
Aradu.33XI8	0.375	1456	51.75	33.974	36.925	Araip.545DL	0.367
Aradu.33XZT	0.406	427	53.972	36.682	46.495	Araip.AZ5LA	0.406
Aradu.346IW	0.509	582	49.4	43.911	73.585	Araip.ZGL25	0.496
Aradu.34ACD	0.435	287	51.042	33.333	38.889	Araip.07XD6	0.416
Aradu.34FHG	0.389	366	49.591	42.507	34.877	Araip.KRU21	0.395
Aradu.34LNY	0.377	579	44.138	44.828	46.207	Araip.0W3FE	0.387
Aradu.34MNK	0.395	210	56.398	34.123	56.872	Araip.280PC	0.41
Aradu.34QFB	0.344	421	48.815	37.441	37.441	Araip.JH2G7	0.349
Aradu.352P0	0.361	138	45.324	34.532	43.165	Araip.TR5VC	0.361
Aradu.353YW	0.403	278	53.763	34.767	41.577	Araip.3M5WT	0.396
Aradu.356M1	0.357	223	43.75	39.286	44.196	Araip.3FK8B	0.353
Aradu.3589Z	0.386	552	43.219	42.857	36.347	Araip.8M1JQ	0.374
Aradu.35B90	0.34	727	49.725	38.187	35.577	Araip.I46QR	0.345
Aradu.35HVS	0.361	732	54.843	44.611	37.381	Araip.CFP2Q	0.352
Aradu.35NHM	0.352	920	47.123	38.111	34.962	Araip.N2G5P	0.365
Aradu.35P83	0.445	554	52.252	44.865	33.514	Araip.6CP6H	0.446
Aradu.35TX9	0.44	380	53.806	46.457	45.407	Araip.PP85N	0.44
Aradu.35U3T	0.366	509	54.706	48.039	32.157	Araip.XVM77	0.366
Aradu.35WGP	0.334	479	47.5	39.167	37.917	Araip.NR5VL	0.371
Aradu.35Z4P	0.354	494	52.929	42.626	52.323	Araip.N2HP9	0.351
Aradu.366AT	0.409	698	54.363	46.924	44.063	Araip.VX6AQ	0.411
Aradu.366M2	0.376	440	57.143	36.961	40.136	Araip.BYN0W	0.381
Aradu.36LCY	0.359	870	48.794	40.873	36.51	Araip.WH0LB	0.353
Aradu.36NL9	0.383	577	48.789	35.467	37.716	Araip.645JG	0.388
Aradu.36PEF	0.386	195	48.469	36.735	44.898	Araip.NA12S	0.381
Aradu.36SKU	0.418	328	50.456	43.465	39.514	Araip.4NG5J	0.405
Aradu.36UYV	0.379	251	56.349	38.095	43.254	Araip.P3BCU	0.379
Aradu.36VCP	0.375	858	54.482	40.396	37.485	Araip.6BC4J	0.372
Aradu.37C4I	0.337	505	48.221	39.526	34.19	Araip.485LJ	0.34
Aradu.383XS	0.37	538	55.473	35.065	48.237	Araip.3P5TL	0.37
Aradu.384WQ	0.397	412	55.932	33.656	47.215	Araip.UJ6CM	0.395
Aradu.38DG2	0.365	382	46.214	37.859	48.042	Araip.L4RII	0.371
Aradu.38IMA	0.252	129	42.308	33.077	30.769	Araip.HU5JH	0.252
Aradu.38JU0	0.363	336	48.071	34.718	41.84	Araip.8M056	0.38
Aradu.38M3H	0.4	422	52.719	38.771	41.135	Araip.9S8V8	0.418
Aradu.38TXW	0.375	361	43.646	35.083	38.398	Araip.987DS	0.372
Aradu.38ZXG	0.354	549	50.545	36.727	38.182	Araip.S7H0U	0.358
Aradu.39755	0.479	349	48	38	36.286	Araip.D3HLX	0.488

Aradu.398CK	0.381	1137	46.221	39.631	45.87	Araip.MS7KA	0.385
Aradu.39CB1	0.392	160	43.478	42.236	52.795	Araip.50B92	0.386
Aradu.39CFF	0.332	417	41.388	38.038	40.431	Araip.I8JAN	0.322
Aradu.39QNU	0.49	102	43.689	45.631	45.631	Araip.24GT1	0.469
Aradu.3A2M2	0.411	523	54.771	36.832	44.275	Araip.UHZ8X	0.384
Aradu.3AI2Z	0.425	417	49.522	36.842	61.962	Araip.6D2E5	0.439
Aradu.3AT2D	0.351	494	54.141	41.212	37.374	Araip.YTI05	0.35
Aradu.3B2AS	0.436	144	50.345	40.69	42.759	Araip.F2WNQ	0.436
Aradu.3B8NW	0.357	446	49.217	36.913	41.611	Araip.TD6FV	0.353
Aradu.3B9N0	0.399	659	48.788	35.606	47.879	Araip.1I49F	0.398
Aradu.3BA5T	0.445	368	50.136	48.78	53.117	Araip.8BB4D	0.448
Aradu.3C2YM	0.374	662	52.036	38.612	39.367	Araip.MV4VH	0.368
Aradu.3C582	0.374	353	54.802	37.571	37.571	Araip.M7YXX	0.409
Aradu.3C7HF	0.414	355	48.315	45.225	48.876	Araip.45CYE	0.413
Aradu.3CD7I	0.438	507	46.26	41.339	47.835	Araip.RBV5T	0.445
Aradu.3CF99	0.401	214	57.209	33.488	52.558	Araip.C736X	0.406
Aradu.3D0ZZ	0.344	433	43.779	50.461	39.631	Araip.ZGL70	0.338
Aradu.3D1M6	0.359	436	56.522	37.757	44.165	Araip.VHD9K	0.354
Aradu.3D1TM	0.404	808	55.377	44.87	44.994	Araip.RR6HX	0.405
Aradu.3D2PD	0.388	437	59.361	40.868	33.562	Araip.JRQ56	0.388
Aradu.3D87V	0.311	168	55.621	37.87	38.462	Araip.FUG09	0.305
Aradu.3DC0K	0.409	168	56.805	48.521	37.278	Araip.2JH3B	0.409
Aradu.3DV9B	0.434	250	53.785	43.028	37.849	Araip.04K1E	0.43
Aradu.3DW3S	0.425	496	50.905	41.449	39.437	Araip.9SP7M	0.423
Aradu.3DX6E	0.481	272	56.044	40.293	58.974	Araip.BT3CW	0.472
Aradu.3E4UZ	0.424	219	55.909	40	37.273	Araip.7L3C8	0.438
Aradu.3E55M	0.408	232	56.223	42.06	45.923	Araip.C6M4G	0.421
Aradu.3E5G3	0.366	607	52.467	40.296	35.691	Araip.R0W2T	0.36
Aradu.3EB7Q	0.329	533	43.446	35.393	34.082	Araip.2I18H	0.302
Aradu.3EF6P	0.384	578	43.005	40.069	35.579	Araip.H235T	0.385
Aradu.3EX3F	0.393	438	56.264	35.991	43.28	Araip.N3BQC	0.386
Aradu.3F83L	0.42	262	53.992	44.867	32.319	Araip.3F57L	0.421
Aradu.3F84N	0.395	1277	50.939	40.454	36.307	Araip.Z2ADB	0.39
Aradu.3G1RX	0.422	167	57.143	42.857	46.429	Araip.J314H	0.416
Aradu.3G22S	0.406	264	60	47.925	52.453	Araip.JTL3L	0.401
Aradu.3GC8H	0.345	259	53.077	33.846	31.923	Araip.K88SY	0.354
Aradu.3GT7T	0.367	1176	45.54	34.834	34.664	Araip.ZUY8H	0.376
Aradu.3GX0M	0.385	1638	48.322	34.106	38.133	Araip.W7XT8	0.384
Aradu.3GX35	0.416	505	50.395	40.711	46.245	Araip.S2XNF	0.411
Aradu.3GX6J	0.299	1297	58.089	60.709	31.433	Araip.ZB5LG	0.31
Aradu.3I9MJ	0.354	1106	54.833	42.005	33.785	Araip.FMA28	0.355
Aradu.3II03	0.359	591	55.236	35.135	37.669	Araip.ETT8S	0.358
Aradu.3IM1W	0.385	207	52.885	30.769	36.538	Araip.C4BMV	0.351
Aradu.3IY7B	0.35	962	55.971	41.121	34.787	Araip.L1GQW	0.351

Aradu.3IZ11	0.396	575	48.611	41.84	48.785	Araip.G3RDD	0.404
Aradu.3J25S	0.399	371	55.914	38.172	42.742	Araip.2JM6B	0.392
Aradu.3J7Z6	0.446	282	54.064	45.23	61.484	Araip.K1GZG	0.446
Aradu.3J95M	0.353	350	54.131	46.154	36.752	Araip.G0AQ4	0.353
Aradu.3JN5I	0.435	252	54.941	36.364	47.036	Araip.DQG9G	0.435
Aradu.3K2CT	0.44	450	52.55	40.355	48.337	Araip.T7TAP	0.434
Aradu.3K9KE	0.34	502	52.684	35.785	45.129	Araip.51AE7	0.354
Aradu.3KC68	0.363	723	46.823	42.541	33.287	Araip.T49YB	0.366
Aradu.3KI43	0.404	396	48.866	43.829	37.531	Araip.M91DZ	0.367
Aradu.3KJ9A	0.356	473	48.734	37.975	48.945	Araip.WB5PP	0.368
Aradu.3L1J2	0.41	201	60.396	30.693	45.05	Araip.Y3GD9	0.395
Aradu.3L1P7	0.397	374	44.267	34.933	44.8	Araip.0QI44	0.4
Aradu.3L3H2	0.357	282	49.117	32.862	39.576	Araip.57TGT	0.347
Aradu.3L41J	0.402	650	50.077	32.104	45.161	Araip.0MK8M	0.412
Aradu.3L4RS	0.381	476	54.507	40.461	53.249	Araip.PAD1A	0.381
Aradu.3L5NK	0.358	315	52.215	37.975	41.139	Araip.HD6QL	0.354
Aradu.3LE29	0.286	135	38.235	44.118	22.059	Araip.2MY4B	0.309
Aradu.3LW1Z	0.363	976	50.972	39.406	35.926	Araip.51ELU	0.364
Aradu.3MD78	0.441	157	47.468	33.544	51.266	Araip.BP4Q3	0.438
Aradu.3MG08	0.403	313	50.637	40.127	38.535	Araip.B49UZ	0.399
Aradu.3MG6J	0.464	390	54.476	38.107	47.826	Araip.EC0NK	0.449
Aradu.3MI4M	0.416	744	51.678	34.765	42.013	Araip.G2J9V	0.414
Aradu.3MT4E	0.362	474	47.368	45.053	52	Araip.X3LMP	0.364
Aradu.3N314	0.347	714	53.147	43.497	45.315	Araip.P55C3	0.344
Aradu.3N4PQ	0.364	1034	43.478	40.097	35.942	Araip.69A15	0.365
Aradu.3N57P	0.357	277	48.201	40.288	39.209	Araip.KUG5H	0.345
Aradu.3N6NA	0.348	296	50.168	32.997	39.394	Araip.Q655H	0.351
Aradu.3NG75	0.373	684	49.489	43.942	44.672	Araip.X9MCL	0.383
Aradu.3P3LL	0.387	1349	46.519	40.593	36.074	Araip.W5NDF	0.389
Aradu.3P5HB	0.492	610	50.573	47.791	62.848	Araip.PB3YQ	0.489
Aradu.3P75R	0.437	228	58.515	58.079	62.882	Araip.B2ESJ	0.441
Aradu.3Q3ML	0.382	711	56.32	40.169	52.949	Araip.LG925	0.388
Aradu.3Q3XZ	0.425	265	39.474	39.85	40.977	Araip.386YD	0.421
Aradu.3Q7E3	0.384	264	48.302	39.623	42.264	Araip.42FFR	0.376
Aradu.3Q82R	0.418	357	43.296	44.972	35.475	Araip.IN0FT	0.426
Aradu.3QR5Y	0.427	290	51.89	41.924	51.89	Araip.H8Y66	0.423
Aradu.3R84Q	0.445	538	49.165	39.889	61.596	Araip.NT8KP	0.445
Aradu.3RP7R	0.386	371	45.699	44.624	33.333	Araip.DYF51	0.39
Aradu.3S3UE	0.497	367	45.924	38.315	60.326	Araip.BB9A1	0.486
Aradu.3S60E	0.508	391	54.082	42.602	59.439	Araip.U6VQA	0.514
Aradu.3SA2N	0.371	366	57.493	44.959	36.785	Araip.A28ZZ	0.361
Aradu.3SD99	0.524	195	53.571	40.306	72.959	Araip.D0QDL	0.529
Aradu.3SM7F	0.411	276	50.542	34.657	41.516	Araip.9PC2H	0.415
Aradu.3SS5K	0.354	1500	54.164	45.57	32.179	Araip.S7U5R	0.354

Aradu.3T4GH	0.397	510	51.272	42.27	43.249	Araip.1RB5V	0.397
Aradu.3T5V1	0.373	822	44.714	37.546	37.789	Araip.KI3H8	0.357
Aradu.3TD2D	0.474	573	47.561	44.077	46.69	Araip.I5BDL	0.48
Aradu.3TE64	0.449	243	52.459	41.803	48.77	Araip.J0VA9	0.398
Aradu.3TJ8Z	0.409	1078	52.456	37.442	38.276	Araip.LCL9M	0.406
Aradu.3TT1Y	0.342	537	47.212	36.989	40.52	Araip.67S5I	0.337
Aradu.3UE4N	0.483	340	55.132	44.868	56.305	Araip.F3QBW	0.479
Aradu.3UN15	0.398	387	56.186	42.526	40.206	Araip.C32PD	0.396
Aradu.3V07T	0.438	303	51.316	41.118	45.066	Araip.WSF31	0.405
Aradu.3V0DC	0.355	487	44.877	32.992	36.475	Araip.MW82X	0.35
Aradu.3V1LI	0.42	159	48.125	41.875	41.875	Araip.7C03S	0.43
Aradu.3VD4A	0.399	633	47.161	42.587	37.224	Araip.XDK4R	0.4
Aradu.3VR2T	0.387	413	52.174	42.271	49.517	Araip.L9ZIB	0.389
Aradu.3W0JX	0.39	431	50.463	40.046	42.13	Araip.PX9HF	0.404
Aradu.3WJ36	0.465	628	55.962	43.243	64.229	Araip.HIF1W	0.469
Aradu.3X0HY	0.397	559	57.5	38.571	38.214	Araip.1J2S6	0.397
Aradu.3X1QZ	0.397	322	52.941	40.867	33.127	Araip.0ZR5Y	0.382
Aradu.3X2EP	0.387	257	51.163	50.775	32.946	Araip.EX8PA	0.391
Aradu.3X53E	0.533	174	47.429	37.714	57.143	Araip.5VX8G	0.506
Aradu.3X54U	0.381	942	50.053	44.008	34.252	Araip.RKC4S	0.396
Aradu.3X77Q	0.396	224	47.556	36.889	42.667	Araip.62MQQ	0.383
Aradu.3XT84	0.469	219	49.545	31.818	52.727	Araip.5735B	0.488
Aradu.3Y26A	0.417	361	43.646	30.11	40.884	Araip.W0TVI	0.4
Aradu.3Y7QG	0.381	350	51.282	41.595	44.16	Araip.54EQT	0.384
Aradu.3YH97	0.375	511	43.359	36.523	38.281	Araip.3X74X	0.372
Aradu.3Z6PL	0.366	695	53.592	36.351	37.069	Araip.UE7U5	0.367
Aradu.3ZC2Z	0.357	301	45.033	40.066	34.768	Araip.B91MN	0.361
Aradu.3ZE3D	0.397	889	55.393	41.011	40.562	Araip.45X5I	0.397
Aradu.3ZF9R	0.401	587	47.449	34.524	51.361	Araip.WJ4AY	0.381
Aradu.3ZR52	0.322	189	56.842	42.632	37.368	Araip.WH6UQ	0.313
Aradu.40078	0.424	630	52.932	46.593	46.434	Araip.03WH7	0.425
Aradu.4094W	0.45	1497	51.001	52.47	35.113	Araip.J0JMG	0.451
Aradu.40AX1	0.355	468	45.842	42.004	33.049	Araip.KF79G	0.353
Aradu.40CKU	0.429	507	49.016	35.236	54.134	Araip.J51X4	0.415
Aradu.40DSM	0.426	128	61.24	35.659	44.961	Araip.PBD8R	0.418
Aradu.40GCA	0.398	288	49.135	40.484	49.135	Araip.F8RKD	0.405
Aradu.40JGL	0.342	338	48.378	40.413	35.398	Araip.R4838	0.362
Aradu.40JMZ	0.364	455	43.421	37.281	47.368	Araip.4QE8A	0.361
Aradu.40QFE	0.461	318	48.903	44.514	49.843	Araip.PRX0Z	0.461
Aradu.40VD3	0.345	588	47.199	40.068	40.238	Araip.0431E	0.348
Aradu.40X7J	0.438	535	54.851	36.194	44.776	Araip.A5SHC	0.434
Aradu.4118A	0.41	919	49.674	40.761	47.174	Araip.X6A1T	0.415
Aradu.412P9	0.379	157	46.203	41.139	32.911	Araip.C98N5	0.379
Aradu.413J1	0.348	595	42.617	39.43	34.564	Araip.GL07W	0.367

Aradu.418KR	0.358	436	44.394	43.478	38.673	Araip.F83NR	0.353
Aradu.4196P	0.415	264	46.792	46.792	46.792	Araip.RMR7N	0.415
Aradu.41DJI	0.351	138	58.273	56.835	48.201	Araip.ZE4M6	0.371
Aradu.41EZW	0.402	806	52.045	41.884	42.627	Araip.DM985	0.407
Aradu.41I2U	0.414	250	52.59	41.036	49.004	Araip.A89IR	0.407
Aradu.41J00	0.356	272	54.212	37.729	40.659	Araip.AC35D	0.355
Aradu.41JSL	0.376	455	49.561	36.404	37.5	Araip.C1AWR	0.376
Aradu.4206R	0.439	265	60.15	50.376	63.534	Araip.LE8QH	0.437
Aradu.425TL	0.379	401	49.502	37.065	43.284	Araip.12XQ6	0.393
Aradu.426TW	0.328	345	53.179	39.017	40.462	Araip.G5KAW	0.336
Aradu.42JCX	0.366	614	47.805	40.65	37.073	Araip.8FA2Y	0.356
Aradu.42P16	0.299	148	40.268	42.282	42.282	Araip.HF5I3	0.271
Aradu.42SQT	0.33	449	48.667	38.444	37.333	Araip.V7BYX	0.335
Aradu.42UET	0.399	638	49.452	41.002	41.315	Araip.4LL3W	0.391
Aradu.431XY	0.395	348	53.295	36.39	38.109	Araip.3W1KE	0.394
Aradu.432N5	0.401	394	53.165	41.266	38.987	Araip.B2BPT	0.386
Aradu.43K0P	0.434	353	53.107	39.831	62.429	Araip.FU4GL	0.43
Aradu.43TNC	0.382	754	46.358	37.086	41.457	Araip.N9GVK	0.378
Aradu.43TRE	0.449	267	59.328	69.776	41.418	Araip.WU624	0.447
Aradu.43YX5	0.373	352	45.892	36.827	41.076	Araip.8G316	0.37
Aradu.443KM	0.436	163	54.878	50	55.488	Araip.K94XG	0.44
Aradu.44DMI	0.327	117	54.237	44.915	33.898	Araip.290GK	0.345
Aradu.44JZ9	0.391	100	41.584	36.634	57.426	Araip.56MFZ	0.402
Aradu.44KEY	0.375	466	51.606	40.043	39.4	Araip.KJ84C	0.38
Aradu.44QR7	0.374	333	55.09	55.689	40.12	Araip.Z1KYK	0.386
Aradu.44QYZ	0.36	1197	46.244	35.392	39.816	Araip.N2I6Z	0.354
Aradu.44R1C	0.393	762	49.541	38.925	44.037	Araip.LS077	0.387
Aradu.44VUH	0.382	624	48.32	41.12	37.12	Araip.MGZ00	0.381
Aradu.44Z6R	0.382	661	45.468	33.988	47.281	Araip.DTK89	0.378
Aradu.452B2	0.388	411	50.243	37.864	43.204	Araip.02N4R	0.39
Aradu.457AG	0.438	307	51.623	35.065	49.026	Araip.VT4Q1	0.435
Aradu.45ASZ	0.385	115	51.724	37.931	59.483	Araip.JZ9QI	0.398
Aradu.45CR1	0.344	1728	49.624	41.932	34.818	Araip.3E8GX	0.342
Aradu.45ENN	0.365	783	50.638	40.689	35.714	Araip.JV5S5	0.36
Aradu.45HXA	0.401	509	58.039	44.51	31.569	Araip.U6HBJ	0.395
Aradu.45I6B	0.362	528	48.771	38.941	44.423	Araip.M724S	0.372
Aradu.45U0D	0.417	317	45.912	48.742	40.881	Araip.0HI1A	0.413
Aradu.461JS	0.339	115	53.448	37.931	38.793	Araip.YN7KH	0.379
Aradu.4635I	0.299	291	45.548	40.411	41.438	Araip.ZP3NK	0.313
Aradu.463D4	0.408	339	52.353	38.824	45.882	Araip.EP8U9	0.414
Aradu.464FL	0.354	248	41.767	43.373	37.349	Araip.R8FCG	0.354
Aradu.464FR	0.402	138	45.324	36.691	46.043	Araip.YGS65	0.386
Aradu.46FZZ	0.364	309	51.935	41.613	37.742	Araip.WZ3EA	0.369
Aradu.46IYL	0.385	1019	51.961	37.843	36.275	Araip.DXG9R	0.382

Aradu.46J5J	0.333	219	42.727	28.182	37.727	Araip.S2Y9M	0.329
Aradu.46M2Y	0.421	813	53.194	43.366	63.636	Araip.38X68	0.431
Aradu.46T9V	0.38	597	54.682	45.485	38.963	Araip.8H3SQ	0.378
Aradu.46TFL	0.352	290	49.828	29.897	50.172	Araip.GG6QM	0.352
Aradu.46U1T	0.506	251	51.984	40.873	55.952	Araip.ZX5IX	0.476
Aradu.46VDC	0.42	244	42.857	40	35.102	Araip.JG1MS	0.412
Aradu.46Z90	0.329	1476	45.701	38.727	35.003	Araip.291LW	0.328
Aradu.4707K	0.359	446	48.546	47.427	33.11	Araip.Q1JDY	0.373
Aradu.470B5	0.458	257	50.775	46.512	55.814	Araip.N6IZA	0.452
Aradu.473E6	0.344	409	51.463	36.098	35.366	Araip.D9TAI	0.347
Aradu.475J2	0.369	866	49.942	30.104	37.486	Araip.167J0	0.371
Aradu.475M7	0.38	1030	51.891	45.878	38.506	Araip.MEM8W	0.388
Aradu.47ARL	0.407	516	48.162	39.845	42.553	Araip.L4KXF	0.389
Aradu.47F2N	0.342	816	59.608	43.819	36.353	Araip.XGP93	0.343
Aradu.47F3C	0.382	159	53.125	43.75	41.25	Araip.IW920	0.395
Aradu.47FME	0.394	265	42.857	37.97	36.09	Araip.NA6B3	0.39
Aradu.47JD2	0.42	716	55.509	43.933	48.675	Araip.MC3BU	0.419
Aradu.47LJL	0.317	254	48.235	38.039	37.255	Araip.T6887	0.343
Aradu.47VKV	0.433	212	51.174	46.479	32.394	Araip.BX0PW	0.433
Aradu.47ZJ4	0.409	483	48.14	39.876	47.314	Araip.CCF9L	0.394
Aradu.483P6	0.433	765	52.872	44.256	45.039	Araip.IN0F4	0.436
Aradu.483RE	0.358	1365	49.048	41.362	33.382	Araip.D5YHX	0.358
Aradu.48ATB	0.372	153	49.351	38.961	53.247	Araip.X1WDR	0.378
Aradu.48DYG	0.338	228	53.275	34.061	38.865	Araip.F8RVF	0.347
Aradu.48GKA	0.365	731	57.65	40.301	39.754	Araip.42YVV	0.361
Aradu.48GRH	0.352	482	53.416	39.545	38.509	Araip.UJ50A	0.364
Aradu.492LT	0.471	330	50.453	39.577	61.631	Araip.6MZ14	0.483
Aradu.495R5	0.416	290	46.735	40.206	42.268	Araip.H55PK	0.43
Aradu.49BKS	0.427	616	50.243	56.564	52.026	Araip.9CB1N	0.418
Aradu.49CW6	0.418	188	51.852	36.508	58.201	Araip.MU3QB	0.402
Aradu.49JIJ	0.353	121	53.279	40.984	40.164	Araip.D8MQT	0.394
Aradu.49PAS	0.422	504	50.297	36.238	55.842	Araip.WX8L5	0.417
Aradu.49T9W	0.348	525	47.909	38.023	39.354	Araip.QK3HR	0.368
Aradu.49VWN	0.442	168	41.42	36.095	50.296	Araip.CCV5U	0.448
Aradu.49WR7	0.488	270	55.72	36.9	54.244	Araip.50H5N	0.483
Aradu.4A33P	0.385	726	50.894	36.589	35.901	Araip.Z1JK3	0.39
Aradu.4A3GU	0.373	272	52.381	38.095	43.59	Araip.5Y2PN	0.357
Aradu.4A7CI	0.496	131	50	34.848	56.818	Araip.0RS02	0.496
Aradu.4AK3M	0.4	144	48.276	35.862	42.759	Araip.E9N7G	0.396
Aradu.4AW6R	0.377	492	54.97	37.728	50.101	Araip.3D0I5	0.379
Aradu.4B6K6	0.414	283	48.592	39.437	46.127	Araip.KNG8V	0.394
Aradu.4BB0R	0.401	192	46.114	45.078	40.415	Araip.B82I6	0.417
Aradu.4BV7T	0.442	335	51.488	42.56	49.107	Araip.YCD8P	0.429
Aradu.4C314	0.371	502	47.714	35.189	41.948	Araip.ZDD3V	0.364

Aradu.4C32F	0.392	1021	51.566	37.378	41.781	Araip.89FXQ	0.393
Aradu.4D16J	0.53	206	57.971	44.928	60.386	Araip.SS0GG	0.53
Aradu.4D7HL	0.387	568	42.531	46.573	35.852	Araip.YZ7MJ	0.386
Aradu.4D9C8	0.489	183	53.261	45.652	50.543	Araip.PS258	0.5
Aradu.4DA0K	0.338	724	51.31	33.379	35.862	Araip.4B2QS	0.345
Aradu.4DJ5M	0.337	1286	49.65	35.742	38.384	Araip.0C951	0.347
Aradu.4DR9N	0.371	911	51.316	39.145	39.803	Araip.N7CRY	0.372
Aradu.4E354	0.369	740	50.877	36.842	40.756	Araip.K8DPE	0.38
Aradu.4E6H2	0.417	445	55.605	38.341	40.807	Araip.I7LKY	0.41
Aradu.4EB0K	0.313	272	53.846	36.996	40.293	Araip.5FM9T	0.302
Aradu.4ER97	0.411	595	51.342	44.966	45.805	Araip.7G8YS	0.415
Aradu.4FA0W	0.408	417	48.565	41.148	40.191	Araip.T0473	0.423
Aradu.4FK3V	0.346	218	57.078	37.9	45.205	Araip.49NYC	0.346
Aradu.4H56Q	0.356	686	46.87	38.719	37.555	Araip.JAP1G	0.364
Aradu.4H615	0.397	364	56.164	40	45.753	Araip.U8TWE	0.402
Aradu.4H70X	0.398	304	52.787	37.377	34.098	Araip.V7QPL	0.401
Aradu.4HB9F	0.373	527	54.356	39.583	37.5	Araip.APB7B	0.376
Aradu.4HH0P	0.358	1103	41.395	38.496	38.496	Araip.2V74Q	0.35
Aradu.4HQ1D	0.396	661	48.792	36.254	39.728	Araip.4L98G	0.399
Aradu.4HV8T	0.339	456	55.361	44.42	33.479	Araip.M0K3R	0.339
Aradu.4I166	0.372	1258	47.498	46.624	40.826	Araip.F53Z3	0.369
Aradu.4I5UW	0.371	514	45.243	37.864	44.466	Araip.18CW0	0.375
Aradu.4I70C	0.374	360	51.524	46.26	44.321	Araip.0B2S6	0.371
Aradu.4IC4Q	0.387	624	56.64	38.72	41.92	Araip.ZJ8ZP	0.388
Aradu.4IL2F	0.408	186	43.316	36.364	50.267	Araip.T34A8	0.393
Aradu.4IN7K	0.408	218	54.338	38.813	46.575	Araip.N2LA6	0.493
Aradu.4IR6I	0.381	1458	52.981	42.563	34.339	Araip.ETN8K	0.381
Aradu.4J3R3	0.376	627	46.497	41.561	39.49	Araip.NN141	0.374
Aradu.4K089	0.415	737	50.813	31.572	45.122	Araip.32W9F	0.41
Aradu.4K5XY	0.404	481	54.564	38.589	36.929	Araip.WHJ1H	0.406
Aradu.4KD1L	0.399	286	43.206	49.129	44.948	Araip.4PY6A	0.395
Aradu.4KE9F	0.343	564	48.319	37.345	38.761	Araip.PRY9G	0.356
Aradu.4L5V2	0.375	114	53.913	40.87	46.087	Araip.CX1A1	0.364
Aradu.4L5WB	0.382	316	46.057	42.271	41.009	Araip.33YRY	0.395
Aradu.4L72B	0.382	619	52.903	37.419	45.968	Araip.TG6R6	0.375
Aradu.4LY04	0.377	908	47.085	34.543	38.614	Araip.QIP2H	0.371
Aradu.4M072	0.411	275	53.986	42.029	49.638	Araip.8Y41D	0.415
Aradu.4M2H4	0.379	675	58.136	46.006	39.053	Araip.7P4BA	0.383
Aradu.4M66F	0.373	1171	47.44	36.263	39.42	Araip.EDG3L	0.362
Aradu.4M8RF	0.462	361	62.707	37.845	47.238	Araip.82XGB	0.456
Aradu.4ME26	0.355	293	49.66	32.313	39.456	Araip.7RN1R	0.357
Aradu.4MN5D	0.362	555	49.281	39.568	38.669	Araip.0NQ3E	0.359
Aradu.4N3HV	0.367	813	44.84	33.907	41.892	Araip.64470	0.371
Aradu.4N3TL	0.342	593	45.455	38.889	39.899	Araip.WA8VW	0.338

Aradu.4N6LD	0.404	528	56.9	44.234	35.728	Araip.B8Y5M	0.396
Aradu.4NJ9R	0.371	139	45	42.143	46.429	Araip.WT5FG	0.386
Aradu.4NW8B	0.369	586	42.419	41.738	36.116	Araip.VCZ4R	0.384
Aradu.4P1MR	0.378	393	49.239	42.64	34.01	Araip.E7Y1X	0.374
Aradu.4P2F5	0.427	156	54.777	52.229	42.675	Araip.1JL7K	0.45
Aradu.4P8D3	0.41	763	50.393	37.304	56.937	Araip.KU06I	0.41
Aradu.4PC58	0.412	418	44.153	42.243	39.379	Araip.Q8T53	0.406
Aradu.4Q38F	0.446	266	54.682	35.955	68.539	Araip.JP2KL	0.429
Aradu.4Q3MP	0.385	438	49.203	40.547	40.091	Araip.K2XQ7	0.39
Aradu.4Q608	0.365	106	50.467	35.514	46.729	Araip.81EVU	0.371
Aradu.4QT4U	0.457	309	50.645	34.839	40.645	Araip.UKK17	0.464
Aradu.4R7ZC	0.334	754	44.636	37.748	36.026	Araip.X52X0	0.33
Aradu.4R8IN	0.385	376	50.663	38.462	45.358	Araip.X067E	0.394
Aradu.4RN5Y	0.428	476	45.912	29.56	46.751	Araip.C9WGS	0.431
Aradu.4RZ6U	0.344	378	46.966	36.412	40.897	Araip.4LV9F	0.339
Aradu.4S1TT	0.356	397	52.764	46.985	40.452	Araip.M800H	0.358
Aradu.4S63Z	0.4	568	53.076	39.543	47.979	Araip.655BS	0.396
Aradu.4S6WR	0.317	417	49.282	45.694	30.144	Araip.JY0CR	0.309
Aradu.4S7IS	0.339	400	62.095	44.389	36.16	Araip.FKP6C	0.37
Aradu.4S94M	0.394	301	47.682	42.715	43.046	Araip.34D6V	0.394
Aradu.4SA47	0.376	646	53.632	42.04	35.703	Araip.29FLU	0.376
Aradu.4SA7Z	0.367	2221	52.79	44.104	34.383	Araip.L5PVW	0.366
Aradu.4T0IX	0.459	125	46.032	38.095	68.254	Araip.ENG6G	0.451
Aradu.4T5J3	0.314	507	47.835	36.417	35.63	Araip.14ENN	0.322
Aradu.4T8J3	0.368	569	47.719	41.579	33.86	Araip.U04P1	0.367
Aradu.4TM7E	0.383	135	48.529	33.088	35.294	Araip.I4Y3K	0.375
Aradu.4TU5K	0.313	121	43.443	34.426	53.279	Araip.Q2MNW	0.313
Aradu.4TY89	0.333	324	46.154	43.077	37.846	Araip.UJ1F9	0.342
Aradu.4U4LS	0.369	170	45.614	38.596	43.86	Araip.PBK8S	0.375
Aradu.4U54R	0.368	256	46.693	40.856	33.852	Araip.44BHE	0.372
Aradu.4U6IV	0.38	578	41.278	39.206	32.988	Araip.X62XD	0.388
Aradu.4U7AL	0.447	417	50.478	40.909	48.804	Araip.3JS6L	0.452
Aradu.4UY6C	0.412	342	47.23	41.399	36.443	Araip.IK079	0.404
Aradu.4V0GE	0.432	305	52.614	41.83	47.712	Araip.Y8ZPE	0.439
Aradu.4V2X0	0.376	132	46.617	33.835	40.602	Araip.7BB5W	0.376
Aradu.4V3WP	0.388	124	50.4	44	44	Araip.PAL72	0.371
Aradu.4V3YY	0.411	314	42.857	54.603	54.603	Araip.C9YWV	0.413
Aradu.4V6Q9	0.394	575	51.736	46.528	47.222	Araip.VY0MP	0.392
Aradu.4VZ9K	0.41	488	50.716	46.83	37.832	Araip.506UP	0.404
Aradu.4W183	0.344	348	51.289	42.407	51.289	Araip.57R3H	0.346
Aradu.4W4JU	0.391	770	49.027	46.044	41.764	Araip.B0RRS	0.386
Aradu.4WT7F	0.411	869	53.793	44.943	42.299	Araip.B6JY4	0.395
Aradu.4X1GI	0.421	534	47.103	41.495	48.224	Araip.I4CPS	0.435
Aradu.4X75W	0.332	609	49.18	40.984	29.672	Araip.18K24	0.328

Aradu.4X7WG	0.373	1000	46.354	34.965	38.262	Araip.I0NS8	0.378
Aradu.4XE1V	0.36	497	47.39	36.546	32.329	Araip.V86YG	0.355
Aradu.4XJ32	0.413	474	54.105	33.684	40.632	Araip.ZXF0A	0.401
Aradu.4XL4D	0.373	395	51.263	35.606	42.929	Araip.J9YK7	0.362
Aradu.4XP3R	0.429	208	44.498	38.756	47.368	Araip.T7FES	0.462
Aradu.4XQ5D	0.342	938	51.225	38.445	37.274	Araip.TIN6U	0.342
Aradu.4Y1KN	0.399	568	57.821	36.731	40.598	Araip.A77RF	0.404
Aradu.4YA8F	0.361	234	47.234	31.064	40.851	Araip.Y9XZZ	0.375
Aradu.4YQ11	0.469	450	47.672	49.446	60.089	Araip.206QL	0.466
Aradu.4YZ2K	0.359	195	47.959	35.204	46.429	Araip.0CL7X	0.359
Aradu.4Z2QP	0.378	1186	46.925	35.973	41.533	Araip.80E2A	0.376
Aradu.4ZD6C	0.345	202	53.695	36.453	31.034	Araip.C9VD3	0.34
Aradu.503J9	0.383	1787	48.826	36.633	47.427	Araip.36RIF	0.382
Aradu.509WJ	0.386	614	56.585	41.463	38.699	Araip.L2L5B	0.395
Aradu.50AY2	0.33	213	53.271	33.645	39.252	Araip.7E6JE	0.348
Aradu.50C9M	0.409	567	48.592	41.373	41.021	Araip.M9DFJ	0.382
Aradu.50FPJ	0.404	1031	51.163	38.953	47.19	Araip.Q3GAG	0.386
Aradu.50HDP	0.387	361	48.066	46.961	35.359	Araip.CI73X	0.387
Aradu.50IFA	0.494	373	57.487	46.791	55.615	Araip.MR0T1	0.505
Aradu.50IM9	0.341	408	49.633	37.653	40.831	Araip.66A5Z	0.354
Aradu.50KFD	0.352	306	43.974	39.739	47.557	Araip.RS4E7	0.348
Aradu.50M8D	0.42	701	53.704	49.003	42.877	Araip.DB6DZ	0.428
Aradu.50RNX	0.423	527	50.189	44.508	39.015	Araip.BUF0S	0.423
Aradu.50TKD	0.417	421	47.393	49.526	46.919	Araip.IL01A	0.398
Aradu.50ZYK	0.369	1251	47.684	38.259	38.019	Araip.KY4DP	0.37
Aradu.51556	0.391	487	52.459	36.885	43.033	Araip.MD8TF	0.387
Aradu.516WS	0.384	338	45.133	43.363	36.283	Araip.B3QST	0.401
Aradu.517PS	0.344	580	46.816	37.005	40.792	Araip.3JG6V	0.35
Aradu.51BBB	0.445	132	52.632	53.383	44.361	Araip.IW1QB	0.445
Aradu.51DJW	0.352	319	52.812	38.75	28.438	Araip.JN8SQ	0.345
Aradu.51ELU	0.499	393	50.761	52.03	46.447	Araip.33RQP	0.503
Aradu.51KU5	0.454	366	48.229	44.959	40.872	Araip.H6QSZ	0.456
Aradu.51L6N	0.314	220	51.584	31.674	42.986	Araip.U0SXH	0.324
Aradu.51WYZ	0.438	358	54.875	44.847	45.404	Araip.T9T6I	0.445
Aradu.51XM7	0.386	216	47.465	35.945	47.005	Araip.59D3Z	0.407
Aradu.523T3	0.351	174	46.286	45.714	38.857	Araip.F9PDZ	0.351
Aradu.524PX	0.348	1349	52.148	40.222	37.259	Araip.T8U1E	0.352
Aradu.52DEQ	0.398	283	49.648	33.803	42.254	Araip.738JR	0.398
Aradu.52L7X	0.36	320	44.548	47.352	38.318	Araip.9JY32	0.373
Aradu.52M2A	0.383	364	49.315	37.808	41.096	Araip.Y3D52	0.38
Aradu.52U3G	0.369	758	45.718	44.532	35.968	Araip.K797H	0.383
Aradu.52XVD	0.366	398	48.371	45.113	46.115	Araip.K00NE	0.371
Aradu.531ZK	0.391	920	50.054	40.608	38.979	Araip.FH86S	0.4
Aradu.53A4D	0.407	493	51.417	35.425	50.607	Araip.7E8G5	0.407

Aradu.53CQM	0.386	283	49.648	35.915	44.366	Araip.BHE2G	0.391
Aradu.53H6W	0.408	666	48.276	39.88	48.126	Araip.7KZ5D	0.415
Aradu.53Y6H	0.394	478	60.125	39.248	34.447	Araip.60SDM	0.406
Aradu.53YGT	0.309	505	45.059	37.352	28.854	Araip.H3GCU	0.321
Aradu.543MU	0.357	517	53.282	36.68	53.089	Araip.5804B	0.363
Aradu.545W2	0.365	281	41.844	33.333	40.78	Araip.T0DQ5	0.383
Aradu.5474V	0.329	329	49.091	36.061	34.545	Araip.CP7MF	0.325
Aradu.54BXB	0.461	237	57.563	38.655	56.303	Araip.G1DUK	0.461
Aradu.54C3S	0.344	272	47.253	31.868	50.183	Araip.68IEK	0.338
Aradu.54DRJ	0.415	362	47.658	38.017	42.424	Araip.U5KWJ	0.424
Aradu.54E1H	0.411	433	49.309	47.005	37.788	Araip.LXV0U	0.409
Aradu.54E80	0.446	451	56.637	40.708	63.938	Araip.I98ZE	0.464
Aradu.54FG7	0.531	486	63.86	47.844	58.727	Araip.P1WT2	0.536
Aradu.54IZY	0.414	657	51.824	41.337	34.195	Araip.Y0L6Q	0.408
Aradu.54J14	0.376	628	49.444	38.792	41.176	Araip.0NG6C	0.376
Aradu.54M2C	0.39	623	49.038	36.378	42.147	Araip.F2I2G	0.384
Aradu.54T4X	0.364	567	47.183	43.838	38.908	Araip.ZYB37	0.36
Aradu.54URP	0.366	277	46.763	40.288	38.489	Araip.3F9W2	0.369
Aradu.54WNJ	0.367	446	52.573	42.953	39.374	Araip.H6WCB	0.381
Aradu.550LU	0.419	900	51.609	43.063	42.397	Araip.8SB48	0.414
Aradu.553J0	0.414	586	53.492	45.145	58.433	Araip.NE3HD	0.419
Aradu.55F3D	0.352	923	43.723	36.039	34.74	Araip.YFY4A	0.353
Aradu.55H8J	0.39	403	55.446	41.089	36.881	Araip.7UJ37	0.397
Aradu.55LZT	0.414	153	44.805	52.597	34.416	Araip.75757	0.421
Aradu.55M8P	0.475	467	47.863	40.171	54.274	Araip.JB6HG	0.478
Aradu.55SXN	0.378	278	53.405	42.652	58.065	Araip.GZ79T	0.359
Aradu.55W9R	0.404	266	55.431	42.697	46.442	Araip.VL132	0.4
Aradu.561J2	0.392	744	49.396	42.148	44.43	Araip.73UEX	0.397
Aradu.569XL	0.46	131	51.515	33.333	47.727	Araip.41YFB	0.468
Aradu.56DPU	0.458	520	57.198	37.428	61.228	Araip.NX9LF	0.475
Aradu.56GAP	0.439	510	50.489	43.053	62.622	Araip.XI1X3	0.434
Aradu.56PIZ	0.444	649	51.231	41.077	62.769	Araip.758EY	0.441
Aradu.56PKD	0.374	782	46.232	38.059	38.314	Araip.8UK7H	0.371
Aradu.56X7I	0.406	408	52.812	37.897	44.254	Araip.MEF1P	0.394
Aradu.56ZRQ	0.41	279	50	33.214	61.429	Araip.36PI6	0.407
Aradu.56ZVJ	0.364	365	47.541	37.705	41.257	Araip.QZ033	0.371
Aradu.570RS	0.345	387	46.392	39.948	40.722	Araip.AV3G9	0.346
Aradu.572L7	0.364	602	47.595	34.163	42.62	Araip.IL4VZ	0.357
Aradu.573UI	0.329	520	55.278	45.298	39.923	Araip.8W8RT	0.331
Aradu.576P8	0.438	434	58.391	42.759	50.345	Araip.UZ11J	0.437
Aradu.577R9	0.367	315	49.367	37.658	42.405	Araip.390JY	0.387
Aradu.577TS	0.357	487	47.951	37.91	31.762	Araip.03RBH	0.35
Aradu.57C0B	0.401	350	49.858	39.601	37.892	Araip.Z0QLZ	0.401
Aradu.57NVY	0.376	1386	51.839	43.908	36.554	Araip.KAU0W	0.372

Aradu.57TMQ	0.408	104	53.333	44.762	35.238	Araip.KG8FH	0.429
Aradu.580FR	0.318	245	46.748	45.122	41.87	Araip.JP448	0.31
Aradu.586VX	0.378	656	49.467	33.486	41.4	Araip.WD7HC	0.388
Aradu.58BVX	0.387	590	56.684	38.24	47.039	Araip.VG1UA	0.363
Aradu.58DAR	0.418	139	50	51.429	44.286	Araip.YC0K3	0.425
Aradu.58FPQ	0.418	823	50.485	36.65	52.306	Araip.37R5L	0.42
Aradu.58JJ3	0.407	536	52.328	46.182	36.872	Araip.S8804	0.416
Aradu.58K6Q	0.35	1176	46.644	36.194	38.403	Araip.2K79X	0.35
Aradu.58KSZ	0.374	1120	50.312	38.537	43.176	Araip.09N6L	0.38
Aradu.58Q87	0.351	627	48.248	36.943	34.395	Araip.91WQY	0.348
Aradu.58WGM	0.394	566	54.145	39.33	53.968	Araip.AR8PA	0.395
Aradu.5926B	0.378	275	52.174	39.493	34.42	Araip.4E9YI	0.374
Aradu.598J8	0.366	487	47.746	36.066	42.008	Araip.XL1Z1	0.365
Aradu.59CIU	0.365	211	44.811	39.623	54.717	Araip.G3NEL	0.35
Aradu.59NQ7	0.401	746	50.736	44.444	40.83	Araip.A437V	0.395
Aradu.59RNH	0.41	417	55.502	33.493	49.761	Araip.15F3V	0.412
Aradu.5A0JT	0.5	326	59.939	45.26	62.997	Araip.84AZS	0.497
Aradu.5A70G	0.406	429	54.651	33.023	37.674	Araip.8SM65	0.403
Aradu.5B2EV	0.36	382	46.997	42.037	35.248	Araip.Q0F05	0.36
Aradu.5B3LS	0.419	713	52.381	48.179	45.378	Araip.NFH73	0.422
Aradu.5B6UX	0.443	226	48.018	35.242	45.374	Araip.6FE68	0.435
Aradu.5BW4X	0.379	907	52.974	34.471	36.233	Araip.V9BE8	0.376
Aradu.5BX1F	0.404	913	49.891	42.56	42.341	Araip.53MRH	0.393
Aradu.5C5XG	0.362	1259	50.079	41.429	33.889	Araip.84IEQ	0.368
Aradu.5C8VM	0.405	517	49.614	38.803	47.683	Araip.G99TB	0.411
Aradu.5CF9R	0.436	173	50	39.08	55.172	Araip.ERC12	0.412
Aradu.5CN8X	0.361	216	46.083	31.797	40.092	Araip.64DCF	0.367
Aradu.5CY6X	0.391	580	48.193	35.456	40.103	Araip.ARQ95	0.406
Aradu.5D1VA	0.412	846	54.782	32.349	41.44	Araip.CD2TQ	0.41
Aradu.5D1Y3	0.39	515	48.643	44.186	36.434	Araip.YYL10	0.402
Aradu.5D1Z0	0.358	394	51.646	41.013	38.228	Araip.GTY0B	0.347
Aradu.5D3K5	0.384	1818	52.501	32.216	41.451	Araip.VQ328	0.383
Aradu.5D5JN	0.403	592	49.241	38.954	44.182	Araip.2W5R5	0.399
Aradu.5DD09	0.377	214	51.628	41.395	42.791	Araip.NS0VF	0.382
Aradu.5DE0L	0.35	483	51.24	39.05	39.876	Araip.IHF9W	0.348
Aradu.5DL1E	0.42	637	53.918	37.461	36.52	Araip.HK3K8	0.407
Aradu.5E0HP	0.389	979	48.673	42.653	33.673	Araip.1C050	0.389
Aradu.5E20L	0.397	357	46.927	37.989	44.134	Araip.AZ7RZ	0.399
Aradu.5E5UI	0.435	354	51.268	45.07	41.127	Araip.TLQ46	0.431
Aradu.5EP67	0.388	108	49.541	54.128	38.532	Araip.65QZN	0.402
Aradu.5F6FU	0.406	505	44.664	38.538	49.802	Araip.B1BRC	0.403
Aradu.5F8QQ	0.364	193	54.124	41.237	39.175	Araip.J1VUP	0.351
Aradu.5G6X0	0.414	583	47.26	38.356	35.445	Araip.17ZYU	0.421
Aradu.5G7S4	0.429	524	54.476	47.238	48.19	Araip.LC3XS	0.407

Aradu.5G8Q4	0.38	1113	47.935	45.961	38.6	Araip.V2R6Y	0.38
Aradu.5GH6D	0.442	446	54.81	38.031	43.177	Araip.I7JKU	0.415
Aradu.5GS43	0.464	253	53.15	48.819	50	Araip.3M2TW	0.472
Aradu.5H0S7	0.382	630	46.751	36.767	35.975	Araip.CNB2V	0.38
Aradu.5H5FX	0.376	622	47.191	36.437	40.931	Araip.48FMM	0.389
Aradu.5HX5K	0.494	164	52.727	58.788	67.273	Araip.WWE5Q	0.494
Aradu.5I03J	0.468	160	39.752	49.689	56.522	Araip.LF0TY	0.485
Aradu.5I3G5	0.36	323	54.938	41.975	35.802	Araip.YI68C	0.368
Aradu.5I6E9	0.366	176	51.412	37.288	40.113	Araip.H02JY	0.355
Aradu.5IN4K	0.345	160	35.404	31.677	40.373	Araip.P84QE	0.372
Aradu.5IY98	0.393	478	50.104	39.666	45.929	Araip.Y58G9	0.401
Aradu.5JM2L	0.287	476	48.008	41.09	35.43	Araip.PX6LZ	0.289
Aradu.5JX5D	0.378	837	47.613	39.021	42.005	Araip.RX3N3	0.365
Aradu.5K5P7	0.362	220	49.774	41.176	38.914	Araip.QYK5M	0.348
Aradu.5K7BE	0.373	327	49.085	34.756	32.927	Araip.ME59K	0.386
Aradu.5K7DH	0.394	387	52.835	38.402	45.361	Araip.L8EL7	0.395
Aradu.5K9HV	0.449	314	49.206	34.921	53.016	Araip.KLV48	0.453
Aradu.5KS9C	0.398	285	47.203	37.063	37.063	Araip.WM4R3	0.398
Aradu.5L39Q	0.315	658	46.434	34.75	37.026	Araip.E7N05	0.308
Aradu.5L8S5	0.337	351	53.977	41.761	33.523	Araip.W6S6X	0.352
Aradu.5LG80	0.462	217	56.422	46.789	57.798	Araip.XR6PW	0.464
Aradu.5LR1R	0.35	638	49.922	36.463	38.185	Araip.ZY4UN	0.343
Aradu.5M1Q4	0.41	375	47.606	43.617	43.617	Araip.A7TGT	0.41
Aradu.5M2IF	0.546	146	54.422	42.177	68.027	Araip.MAE3X	0.532
Aradu.5M48C	0.359	942	46.448	42.842	35.843	Araip.F8718	0.364
Aradu.5M687	0.393	414	45.542	39.277	40.964	Araip.A4CD7	0.398
Aradu.5M8KA	0.389	535	47.201	39.179	36.194	Araip.6X4HD	0.387
Aradu.5ME2Z	0.42	166	59.281	40.12	73.054	Araip.W8189	0.414
Aradu.5MH0W	0.343	533	52.996	44.569	37.266	Araip.3LR0U	0.368
Aradu.5N3RR	0.455	136	57.664	46.715	45.255	Araip.9Q1HG	0.47
Aradu.5N8I2	0.38	487	49.59	46.926	36.27	Araip.43TDN	0.378
Aradu.5NS8M	0.349	473	48.312	47.046	36.287	Araip.G8PU5	0.35
Aradu.5P6B7	0.403	226	52.423	38.326	40.969	Araip.L7VH4	0.407
Aradu.5P7M7	0.383	748	46.195	38.051	39.252	Araip.82VVP	0.391
Aradu.5PB9J	0.333	198	47.739	30.653	35.176	Araip.5G7EV	0.346
Aradu.5PD1B	0.376	533	43.071	40.824	36.704	Araip.LQJ0X	0.39
Aradu.5PW6G	0.433	545	53.114	44.322	49.451	Araip.G04W3	0.429
Aradu.5Q0IP	0.343	871	52.638	38.532	36.697	Araip.X8LEZ	0.354
Aradu.5Q1VY	0.394	335	51.488	35.417	54.464	Araip.13D8C	0.388
Aradu.5Q6ZX	0.383	192	48.705	36.269	47.668	Araip.M3SVD	0.404
Aradu.5QH0B	0.319	298	48.495	30.769	31.773	Araip.78FT4	0.353
Aradu.5QJ48	0.377	403	48.267	44.059	42.327	Araip.7D99U	0.402
Aradu.5R3CV	0.397	540	51.941	33.826	40.85	Araip.DS83W	0.394
Aradu.5R97S	0.374	547	49.27	42.518	35.584	Araip.VG3KL	0.389

Aradu.5R9LY	0.408	268	48.699	33.457	37.918	Araip.4E6BS	0.396
Aradu.5R9X5	0.396	175	42.045	30.114	42.045	Araip.WU4P5	0.383
Aradu.5RE8S	0.402	240	61.826	58.921	33.61	Araip.R7UPP	0.397
Aradu.5RF5F	0.495	327	55.488	48.171	70.427	Araip.0L5SE	0.5
Aradu.5S25K	0.416	319	47.813	46.563	48.438	Araip.P1ARW	0.402
Aradu.5S2PY	0.364	670	48.286	35.171	36.066	Araip.3630D	0.354
Aradu.5SD5G	0.395	481	49.585	42.946	38.174	Araip.E377S	0.403
Aradu.5T2RZ	0.359	413	49.758	40.338	38.647	Araip.ATR2W	0.355
Aradu.5T4PS	0.35	525	53.422	44.677	38.593	Araip.V3A9S	0.358
Aradu.5T6PH	0.454	315	57.911	38.608	60.127	Araip.I7NVN	0.447
Aradu.5TE5X	0.387	474	50.316	35.368	53.474	Araip.U7K5S	0.4
Aradu.5TM0N	0.399	678	51.546	45.95	40.501	Araip.JBC57	0.389
Aradu.5U0XL	0.445	287	51.389	43.75	46.875	Araip.YKV9Q	0.459
Aradu.5U8HG	0.399	562	56.306	41.385	33.57	Araip.E514L	0.4
Aradu.5UD3Q	0.364	520	46.065	34.357	38.004	Araip.0DK96	0.374
Aradu.5UI2Y	0.395	432	51.963	40.647	44.111	Araip.I6YVE	0.394
Aradu.5V20C	0.437	262	47.148	43.346	47.148	Araip.0IQ14	0.435
Aradu.5VL3J	0.485	105	60.377	41.509	64.151	Araip.U65I2	0.485
Aradu.5W065	0.367	1047	52.195	40.84	37.5	Araip.BN9Y3	0.371
Aradu.5W8P5	0.36	1270	46.656	35.563	34.933	Araip.L6B1S	0.357
Aradu.5X3H9	0.334	471	51.059	42.797	39.831	Araip.AM46T	0.347
Aradu.5X8VR	0.414	1019	56.471	41.373	54.216	Araip.C88ZW	0.422
Aradu.5XQ31	0.376	929	50.43	38.28	38.28	Araip.VV837	0.374
Aradu.5Y1RP	0.38	318	47.649	33.856	38.871	Araip.H7632	0.364
Aradu.5Y3I5	0.388	120	52.066	33.058	71.074	Araip.1EQ95	0.397
Aradu.5Y3NY	0.367	1442	52.114	41.719	34.858	Araip.N90NP	0.369
Aradu.5Y4IB	0.324	335	39.583	38.988	31.845	Araip.AB04Q	0.321
Aradu.5Y6Q8	0.509	385	55.959	44.819	67.876	Araip.W5EI6	0.509
Aradu.5Z6H3	0.396	528	53.308	43.1	48.393	Araip.KU01P	0.386
Aradu.5ZT49	0.393	415	50.962	36.298	40.144	Araip.EK9PX	0.404
Aradu.6011J	0.348	313	42.038	43.312	36.624	Araip.JB5WF	0.338
Aradu.60C6S	0.387	621	47.106	36.334	36.977	Araip.69WKH	0.39
Aradu.60DAC	0.368	409	54.878	41.22	36.585	Araip.NT0XC	0.349
Aradu.60GK5	0.393	285	53.497	51.399	36.713	Araip.W10FE	0.42
Aradu.60HCE	0.45	492	53.753	37.931	48.884	Araip.2RJ39	0.428
Aradu.60I66	0.427	429	46.744	40	47.209	Araip.1JG8Y	0.409
Aradu.60S2U	0.392	430	51.044	40.371	44.084	Araip.ZB3XA	0.405
Aradu.612YH	0.34	720	47.434	35.784	38.696	Araip.84MED	0.36
Aradu.617EV	0.336	252	52.964	47.826	37.945	Araip.Y6XIR	0.336
Aradu.61A42	0.377	528	52.174	37.996	41.588	Araip.7J81A	0.387
Aradu.61CW5	0.411	289	44.138	50	45.172	Araip.WP004	0.414
Aradu.61ES3	0.358	333	50.599	38.922	37.725	Araip.0QG56	0.367
Aradu.61FU3	0.377	1693	51.712	39.492	42.208	Araip.C7K4J	0.381
Aradu.61HSA	0.356	500	48.902	38.922	38.523	Araip.99RH1	0.354

Aradu.61JFB	0.434	339	43.235	50	37.941	Araip.L4X0W	0.437
Aradu.61NAP	0.348	1311	49.771	37.652	35.823	Araip.NM49E	0.347
Aradu.61W2G	0.423	496	46.076	45.674	44.869	Araip.TUL3I	0.426
Aradu.61Y68	0.428	453	48.238	39.427	49.339	Araip.HG5A7	0.429
Aradu.61Y7W	0.416	735	51.766	43.342	49.728	Araip.B9HDL	0.415
Aradu.623GS	0.347	592	46.037	33.895	33.052	Araip.Z53WS	0.352
Aradu.623Y7	0.428	298	60.535	49.498	58.863	Araip.W2EJQ	0.401
Aradu.629Q2	0.36	513	52.14	43.385	33.074	Araip.KZ1TD	0.376
Aradu.62BQ5	0.421	582	46.998	43.739	40.309	Araip.4FJ07	0.413
Aradu.62HJK	0.395	292	46.758	49.147	51.195	Araip.2SH4C	0.399
Aradu.62PY8	0.438	663	50.301	40.813	49.548	Araip.A81A1	0.432
Aradu.62QCX	0.352	980	48.624	34.76	39.551	Araip.R9899	0.353
Aradu.62T99	0.354	390	46.803	38.875	42.455	Araip.AEP3U	0.364
Aradu.62WGK	0.444	230	51.948	37.229	37.229	Araip.TTH1U	0.446
Aradu.632MM	0.368	341	49.415	39.474	32.456	Araip.M99F1	0.368
Aradu.634DQ	0.375	132	50.376	44.361	26.316	Araip.1D0FW	0.398
Aradu.63D6A	0.338	512	52.437	50.097	37.622	Araip.LWS1N	0.33
Aradu.63EET	0.408	216	57.604	33.18	44.24	Araip.WRX66	0.413
Aradu.63K76	0.386	612	48.287	40.457	51.876	Araip.653FM	0.404
Aradu.63N31	0.375	295	51.351	36.486	42.568	Araip.U5BCP	0.354
Aradu.63Q7N	0.442	431	49.537	42.361	41.667	Araip.PJ656	0.434
Aradu.63RCW	0.465	248	51.406	40.161	47.791	Araip.TA9E2	0.461
Aradu.63RP0	0.334	430	47.1	43.619	29.234	Araip.IWK17	0.336
Aradu.63TDV	0.484	221	49.099	58.559	70.27	Araip.H81H9	0.488
Aradu.63ZUN	0.39	883	55.882	40.271	34.615	Araip.186SX	0.39
Aradu.640E1	0.459	191	54.167	41.667	41.146	Araip.3SL7S	0.451
Aradu.642R3	0.41	531	48.496	38.158	42.293	Araip.UB5VN	0.412
Aradu.647P3	0.395	426	45.902	38.173	38.407	Araip.B71R1	0.38
Aradu.648HW	0.315	192	53.886	33.679	45.078	Araip.W4NHQ	0.326
Aradu.64B3S	0.396	286	56.794	43.554	32.753	Araip.H3Y0N	0.404
Aradu.64BNM	0.34	263	55.303	41.667	49.242	Araip.H76K9	0.339
Aradu.64C9K	0.483	249	53.6	41.6	40.8	Araip.4L18R	0.479
Aradu.64HJW	0.376	400	45.885	40.15	36.16	Araip.G8EGJ	0.39
Aradu.64JV5	0.403	135	53.676	36.765	55.147	Araip.EE8FM	0.382
Aradu.64LXI	0.375	285	47.552	38.462	40.559	Araip.J49VC	0.36
Aradu.64PSN	0.353	403	52.475	41.832	47.525	Araip.LH6HR	0.371
Aradu.64RBA	0.395	263	54.167	35.227	47.348	Araip.Z40TR	0.418
Aradu.64V56	0.37	361	46.685	37.293	38.674	Araip.JT9BC	0.373
Aradu.64Z0M	0.444	769	63.766	50.39	60	Araip.0WR1B	0.446
Aradu.657DW	0.343	183	42.935	45.652	42.391	Araip.5S35P	0.345
Aradu.65BY0	0.451	149	46.667	37.333	48	Araip.V64IQ	0.444
Aradu.65CE7	0.328	504	50.099	33.069	37.624	Araip.VD1BS	0.327
Aradu.65H3Y	0.401	634	47.717	39.37	44.094	Araip.23JR9	0.392
Aradu.65JMT	0.403	536	48.045	41.713	35.94	Araip.N7GH3	0.399

Aradu.65NT6	0.356	496	45.473	48.893	33.602	Araip.5FB8R	0.355
Aradu.65NZB	0.396	731	52.049	38.934	39.071	Araip.0RS31	0.391
Aradu.65QW9	0.393	795	55.653	43.844	41.457	Araip.T587R	0.393
Aradu.65T0K	0.38	683	49.708	44.152	38.45	Araip.BG1TQ	0.372
Aradu.65TC6	0.327	219	47.727	29.091	38.182	Araip.916M9	0.337
Aradu.65WG6	0.442	303	40.132	43.092	38.158	Araip.V48DY	0.44
Aradu.65YXV	0.478	398	48.12	46.366	57.143	Araip.L9LN0	0.463
Aradu.65ZNS	0.363	262	50.57	39.544	39.544	Araip.K266S	0.35
Aradu.665TV	0.377	406	48.894	33.415	37.592	Araip.S2YNN	0.381
Aradu.666C5	0.448	620	49.919	40.419	54.911	Araip.UC596	0.439
Aradu.668QS	0.362	649	45.846	34.154	34.923	Araip.TVT35	0.354
Aradu.669IL	0.391	118	50.42	38.655	38.655	Araip.S1XQK	0.391
Aradu.66DLZ	0.393	714	49.93	39.86	36.923	Araip.W7TM3	0.391
Aradu.66KCD	0.407	505	59.684	50	35.771	Araip.P9NAH	0.403
Aradu.66MNC	0.356	439	50.909	38.864	47.273	Araip.LSP61	0.354
Aradu.66UC2	0.394	292	57.338	35.154	40.273	Araip.4U431	0.39
Aradu.671CI	0.323	1202	41.895	36.243	36.741	Araip.X7R9Y	0.331
Aradu.674AT	0.33	110	48.649	54.955	36.036	Araip.R87AF	0.409
Aradu.677BP	0.352	169	46.471	42.353	35.882	Araip.TC9TY	0.377
Aradu.67MY8	0.394	226	52.863	32.159	47.577	Araip.X1TUQ	0.421
Aradu.67PUF	0.412	552	52.622	33.996	33.635	Araip.PVQ2W	0.41
Aradu.67UXU	0.391	485	57.407	40.329	38.477	Araip.VA0EM	0.391
Aradu.67X2R	0.382	224	54.222	44.444	40.889	Araip.T3WCA	0.377
Aradu.6896W	0.33	308	46.278	45.631	31.068	Araip.SX8DW	0.336
Aradu.68AHZ	0.367	803	48.134	43.781	35.323	Araip.IBG9Y	0.351
Aradu.68CEE	0.392	412	56.174	37.046	44.31	Araip.R3N8M	0.389
Aradu.68E2R	0.404	514	56.311	34.563	32.233	Araip.L1E1K	0.391
Aradu.68MCI	0.417	343	51.163	44.477	45.058	Araip.S69I6	0.412
Aradu.68MP6	0.385	176	43.503	49.153	39.548	Araip.P60JL	0.387
Aradu.68XYR	0.442	452	52.097	29.36	45.254	Araip.7N26D	0.449
Aradu.68YSI	0.329	323	52.16	31.79	49.383	Araip.N0Z6R	0.325
Aradu.694KT	0.389	722	52.144	35.823	49.516	Araip.Y3YQU	0.388
Aradu.69820	0.349	339	48.529	39.118	39.412	Araip.L5V11	0.343
Aradu.69EQ4	0.355	773	47.028	39.018	43.023	Araip.V2PM2	0.359
Aradu.69GC2	0.428	331	51.807	41.265	50.301	Araip.JM070	0.431
Aradu.69H8E	0.354	354	58.31	47.887	32.676	Araip.D97MW	0.365
Aradu.69HMZ	0.341	512	45.029	40.156	32.554	Araip.GFC8K	0.333
Aradu.69JCH	0.426	120	49.587	52.066	63.636	Araip.6544P	0.461
Aradu.69JL5	0.46	442	57.336	42.438	62.754	Araip.X1F3R	0.457
Aradu.69PE5	0.44	294	57.627	38.644	50.508	Araip.GN10Z	0.436
Aradu.69TMW	0.462	151	51.974	41.447	53.947	Araip.K6C7R	0.46
Aradu.69W3K	0.337	278	51.613	46.953	38.351	Araip.A3G0I	0.326
Aradu.69YXI	0.349	691	50.434	41.329	36.272	Araip.HWH2I	0.358
Aradu.6A2D1	0.377	1170	49.189	42.955	37.062	Araip.T5MHX	0.372

Aradu.6AI81	0.457	289	53.103	44.483	52.414	Araip.Y94H3	0.475
Aradu.6AL72	0.378	520	54.319	40.307	42.61	Araip.TS2QL	0.377
Aradu.6AZ0N	0.363	544	51.56	37.248	43.486	Araip.3BD5Q	0.361
Aradu.6B67D	0.33	226	51.982	28.634	43.612	Araip.YF7P1	0.339
Aradu.6BH4S	0.427	510	45.793	42.466	48.141	Araip.MS5EY	0.437
Aradu.6BK0V	0.388	1182	47.422	50.465	33.981	Araip.SGS4X	0.388
Aradu.6C1Z1	0.326	520	43.186	36.084	41.075	Araip.UU39R	0.323
Aradu.6C2JR	0.365	284	50.877	44.912	42.105	Araip.M4KG4	0.372
Aradu.6C4RF	0.469	169	45.882	32.941	42.941	Araip.NGU0E	0.451
Aradu.6C6EU	0.368	365	52.732	40.984	35.792	Araip.678UJ	0.362
Aradu.6CL4K	0.354	275	53.623	40.217	41.667	Araip.58L9N	0.345
Aradu.6CQ40	0.382	358	49.582	43.454	32.869	Araip.D13W8	0.402
Aradu.6CW5S	0.376	427	57.243	45.794	32.477	Araip.2685R	0.365
Aradu.6D72R	0.37	410	50.365	42.336	49.392	Araip.CG1RC	0.375
Aradu.6DL9M	0.354	192	53.886	31.088	52.332	Araip.K5RKY	0.34
Aradu.6E0QJ	0.436	448	48.107	44.321	42.539	Araip.JLE70	0.435
Aradu.6E59T	0.358	191	56.25	35.938	36.458	Araip.HFF60	0.36
Aradu.6E81Q	0.386	437	49.315	34.932	43.379	Araip.28KXR	0.386
Aradu.6F1MK	0.389	255	51.953	39.844	42.578	Araip.AXD2M	0.385
Aradu.6F2AZ	0.44	321	45.963	41.304	40.062	Araip.JC79F	0.434
Aradu.6FP0C	0.383	573	56.446	53.31	38.85	Araip.YU2P6	0.386
Aradu.6FR0W	0.411	390	51.662	38.875	41.688	Araip.H9INU	0.392
Aradu.6FR5V	0.408	513	53.891	40.661	42.412	Araip.I7UCD	0.41
Aradu.6FW8M	0.367	279	53.214	38.214	36.429	Araip.NL6AM	0.352
Aradu.6GD1V	0.439	170	56.725	39.766	50.292	Araip.E8I2J	0.439
Aradu.6GP3J	0.396	162	54.601	36.81	55.828	Araip.XJ5ED	0.396
Aradu.6H4PP	0.374	230	45.022	36.797	39.827	Araip.92BTQ	0.384
Aradu.6HJ87	0.367	510	50.685	37.182	52.25	Araip.9P3Y0	0.365
Aradu.6I2MF	0.393	334	49.552	40	44.179	Araip.KVP0Y	0.383
Aradu.6I8N8	0.353	457	54.148	46.725	43.45	Araip.JR7JD	0.339
Aradu.6IB2A	0.377	422	55.083	44.681	31.678	Araip.T4V1N	0.374
Aradu.6J2SN	0.333	526	41.176	39.089	35.674	Araip.BW1K8	0.342
Aradu.6JB9C	0.329	424	44.235	40.235	37.882	Araip.YSG6K	0.325
Aradu.6JP4A	0.392	361	54.42	38.122	39.779	Araip.ZSM85	0.389
Aradu.6JU5H	0.408	427	47.897	38.084	40.888	Araip.I2XBN	0.414
Aradu.6K05J	0.392	608	50.903	43.021	42.529	Araip.B6USA	0.393
Aradu.6K5DV	0.351	295	53.041	45.946	34.459	Araip.P8FX2	0.356
Aradu.6K81G	0.36	159	50.625	38.75	39.375	Araip.K3V5A	0.353
Aradu.6KC8S	0.353	266	41.573	32.21	38.577	Araip.Y8FDH	0.357
Aradu.6KR5S	0.439	165	54.217	37.952	44.578	Araip.5C9RI	0.452
Aradu.6L7FC	0.369	1024	51.707	35.22	34.927	Araip.FLC8C	0.369
Aradu.6L9XG	0.376	295	53.378	42.23	52.703	Araip.PK0AD	0.372
Aradu.6LK51	0.48	373	58.556	39.572	57.219	Araip.SD3NU	0.484
Aradu.6LS7N	0.364	754	51.656	37.748	35.364	Araip.CPP3Y	0.363

Aradu.6LZ7W	0.47	151	55.921	34.211	54.605	Araip.R7BSY	0.459
Aradu.6M56A	0.521	171	40.698	48.256	51.744	Araip.ZZ3SQ	0.533
Aradu.6M72C	0.333	961	44.283	40.541	34.2	Araip.JR8N9	0.331
Aradu.6M9JS	0.549	456	60.394	55.142	56.455	Araip.Y5APX	0.552
Aradu.6M9LZ	0.416	405	50.739	42.118	57.389	Araip.G9XAZ	0.411
Aradu.6MH23	0.391	651	56.135	48.62	33.436	Araip.IF4PZ	0.388
Aradu.6MT8W	0.415	123	45.161	37.097	33.065	Araip.2R6PA	0.432
Aradu.6MW2E	0.396	646	55.178	39.567	46.059	Araip.7QP65	0.385
Aradu.6N11A	0.391	449	52	36	43.556	Araip.06WA5	0.395
Aradu.6NR0H	0.431	581	51.031	42.44	54.639	Araip.F26WX	0.44
Aradu.6NR6S	0.363	361	50.276	39.779	36.74	Araip.A4865	0.364
Aradu.6P19N	0.37	629	55.556	43.333	40.476	Araip.7MX4C	0.375
Aradu.6PF06	0.421	221	47.297	35.586	57.658	Araip.BHX6F	0.411
Aradu.6PG6R	0.345	344	52.174	33.623	43.768	Araip.G03BG	0.338
Aradu.6PP4L	0.419	629	58.889	45.714	42.857	Araip.M6JV5	0.417
Aradu.6PX5N	0.378	260	49.425	44.444	42.529	Araip.1U8VF	0.372
Aradu.6Q0CD	0.342	281	51.418	39.362	39.716	Araip.4Z79G	0.357
Aradu.6Q6K7	0.426	234	53.191	43.83	43.83	Araip.WCB4Z	0.431
Aradu.6R3YG	0.371	1381	47.902	36.541	39.219	Araip.224ZW	0.361
Aradu.6R4YC	0.389	200	54.726	42.786	57.214	Araip.B3PLA	0.459
Aradu.6R624	0.399	1064	50.986	38.779	39.343	Araip.4JD4I	0.401
Aradu.6RC9F	0.492	190	56.545	41.361	50.262	Araip.40P7B	0.471
Aradu.6S06R	0.31	251	47.222	38.492	42.46	Araip.D6G4F	0.305
Aradu.6S44C	0.375	279	53.214	35	40.357	Araip.KLH55	0.355
Aradu.6S4B3	0.217	133	38.806	31.343	39.552	Araip.BV4C5	0.233
Aradu.6SC3T	0.507	139	45	40	56.429	Araip.PC9NR	0.507
Aradu.6T4IB	0.351	417	47.368	37.799	33.971	Araip.7A9DT	0.345
Aradu.6T5AB	0.439	260	52.874	31.801	43.295	Araip.MQ8B1	0.425
Aradu.6T9HL	0.352	359	46.111	35.278	36.944	Araip.DTM34	0.352
Aradu.6U3S2	0.381	1340	49.441	42.356	36.018	Araip.G2BMD	0.374
Aradu.6UB5R	0.37	1279	47.969	34.688	41.797	Araip.654N3	0.383
Aradu.6UQ9U	0.392	831	50.24	46.154	38.582	Araip.60HFL	0.391
Aradu.6V13C	0.463	258	56.371	44.015	53.668	Araip.HLU05	0.472
Aradu.6V1PP	0.449	393	44.416	44.67	44.416	Araip.6G6P9	0.439
Aradu.6V2BQ	0.351	153	50	38.312	38.312	Araip.B7NZU	0.338
Aradu.6V4TL	0.39	539	51.111	44.074	39.074	Araip.99AN8	0.386
Aradu.6V6LL	0.42	294	47.458	44.746	37.966	Araip.1P0XB	0.422
Aradu.6V9D4	0.432	181	54.396	51.099	37.912	Araip.K74WI	0.452
Aradu.6VN02	0.428	327	45.732	43.293	42.073	Araip.Z5712	0.429
Aradu.6VU3X	0.39	439	45	35.227	40.682	Araip.B3R7B	0.392
Aradu.6VX3K	0.365	687	45.93	36.919	38.808	Araip.AK8SS	0.355
Aradu.6W1XZ	0.396	843	51.303	38.626	36.848	Araip.T0LEM	0.378
Aradu.6W7E4	0.434	295	54.73	51.014	40.878	Araip.BAN8Q	0.427
Aradu.6W9YP	0.397	255	42.188	37.109	42.188	Araip.ZDZ6L	0.389

Aradu.6WC85	0.353	511	53.906	42.188	39.648	Araip.TRT2Z	0.349
Aradu.6WI58	0.406	602	43.284	33.665	46.103	Araip.XEB47	0.417
Aradu.6X61Q	0.286	107	39.815	43.519	47.222	Araip.K75B4	0.327
Aradu.6X9I9	0.338	369	51.892	30.811	32.162	Araip.7S61E	0.351
Aradu.6X9VQ	0.357	469	45.319	33.617	38.936	Araip.BLV36	0.35
Aradu.6XE07	0.365	369	55.946	32.432	45.676	Araip.3A6UX	0.36
Aradu.6Y1H7	0.358	573	50.348	33.275	40.244	Araip.0AR95	0.348
Aradu.6Y48C	0.415	124	63.2	53.6	32	Araip.5XB02	0.423
Aradu.6Y81Q	0.354	938	55.591	37.806	39.084	Araip.D9BFI	0.365
Aradu.6Y8G9	0.409	167	49.405	36.905	41.667	Araip.NGD50	0.394
Aradu.6YC2Q	0.35	658	48.71	33.232	42.489	Araip.QXM6Z	0.362
Aradu.6YX1J	0.414	806	55.638	43.494	42.875	Araip.D6GJ4	0.422
Aradu.6Z2TN	0.373	494	54.545	43.03	47.071	Araip.4U3W0	0.378
Aradu.6Z34Q	0.405	245	54.472	36.179	46.748	Araip.0ST2B	0.387
Aradu.6Z59M	0.376	985	56.694	43.509	34.888	Araip.LD20M	0.374
Aradu.6ZR5R	0.481	143	58.333	45.139	61.111	Araip.Y4SLP	0.458
Aradu.7065G	0.435	313	56.688	36.306	62.102	Araip.MKZ27	0.442
Aradu.707Z5	0.489	136	53.285	33.577	50.365	Araip.F963I	0.481
Aradu.7091K	0.428	663	53.313	46.084	45.482	Araip.AY0E9	0.421
Aradu.70A8U	0.451	244	49.796	43.673	45.714	Araip.FME0N	0.46
Aradu.70AAJ	0.313	128	50.388	38.76	51.163	Araip.JF2JB	0.396
Aradu.70H43	0.352	406	42.506	43.489	35.381	Araip.DWY4Y	0.354
Aradu.70JJH	0.452	390	51.407	49.872	46.292	Araip.ZXB9C	0.452
Aradu.70NBC	0.423	507	50.591	43.504	41.339	Araip.AB1C2	0.409
Aradu.70NLN	0.402	103	53.846	32.692	45.192	Araip.2539H	0.402
Aradu.70NQH	0.396	1352	55.95	29.047	37.546	Araip.M3B88	0.394
Aradu.70NX8	0.422	540	52.495	41.405	49.723	Araip.5435Q	0.43
Aradu.70QGC	0.387	437	55.936	40.183	49.543	Araip.6E7KQ	0.387
Aradu.70QSY	0.454	320	44.86	44.86	43.925	Araip.DKH8U	0.438
Aradu.70U4X	0.333	166	43.114	37.725	37.725	Araip.DB5YG	0.308
Aradu.70V2V	0.392	354	48.732	39.155	33.239	Araip.98G5D	0.392
Aradu.70WHV	0.388	485	47.942	43.827	33.745	Araip.S9DS1	0.397
Aradu.70YAF	0.369	650	46.697	38.402	39.939	Araip.Z53A4	0.384
Aradu.712AL	0.413	588	48.048	37.182	40.068	Araip.V8K0J	0.42
Aradu.714NZ	0.386	689	51.739	42.464	42.899	Araip.B6DJV	0.392
Aradu.716Q8	0.393	583	55.651	51.541	44.521	Araip.5HV78	0.398
Aradu.71DP5	0.375	558	53.131	44.902	44.365	Araip.HDJ4D	0.367
Aradu.71EUZ	0.425	281	46.809	35.816	44.681	Araip.R8R5W	0.406
Aradu.71JL3	0.347	131	46.97	34.091	33.333	Araip.Z394L	0.347
Aradu.71LF7	0.43	261	51.527	37.786	40.84	Araip.5V540	0.43
Aradu.71MQE	0.574	164	68.485	62.424	43.03	Araip.P03BP	0.568
Aradu.71RRV	0.353	250	46.215	35.458	35.458	Araip.1MM96	0.357
Aradu.71T87	0.341	226	47.137	41.85	32.159	Araip.YU9WM	0.355
Aradu.71U9H	0.435	593	59.428	35.017	45.286	Araip.E8ET3	0.438

Aradu.720N0	0.369	444	45.393	41.798	46.067	Araip.QP9W2	0.373
Aradu.724NR	0.358	563	44.681	42.73	36.348	Araip.HSV9Y	0.36
Aradu.724TL	0.317	217	50	32.11	46.33	Araip.3A86R	0.322
Aradu.725QH	0.407	507	47.835	38.583	40.551	Araip.SS0DN	0.405
Aradu.72C6M	0.371	301	49.007	43.046	53.974	Araip.W027Q	0.361
Aradu.72HG2	0.449	455	52.412	37.281	57.237	Araip.E0IYQ	0.435
Aradu.72K0W	0.364	127	50.781	35.156	41.406	Araip.2M8UJ	0.388
Aradu.72K9J	0.491	593	49.832	47.643	47.811	Araip.AJC62	0.484
Aradu.72KXP	0.314	194	47.179	38.974	33.333	Araip.CNH5B	0.305
Aradu.72QL5	0.373	1205	49.751	40.713	38.391	Araip.HUC28	0.379
Aradu.72VHJ	0.456	133	43.284	25.373	58.209	Araip.78Q2V	0.448
Aradu.734NR	0.441	377	48.942	48.942	48.148	Araip.B7BWA	0.449
Aradu.7399Q	0.391	481	55.809	37.137	38.589	Araip.73ST3	0.374
Aradu.73DHW	0.353	1729	47.341	40.983	37.225	Araip.LYI86	0.356
Aradu.73E3B	0.373	386	49.354	31.525	42.377	Araip.6W639	0.368
Aradu.73HE8	0.343	359	43.889	44.167	28.611	Araip.1Z76L	0.321
Aradu.73JAV	0.369	441	51.81	32.805	39.819	Araip.7IS5A	0.38
Aradu.73KGG	0.436	585	56.314	37.884	54.949	Araip.ZKH0K	0.434
Aradu.73L3W	0.346	494	46.667	40.606	35.556	Araip.TSM4K	0.348
Aradu.73MTJ	0.353	535	48.881	33.769	44.776	Araip.Q25ER	0.354
Aradu.73PA3	0.401	237	51.681	36.975	39.916	Araip.0BL53	0.388
Aradu.73Q6B	0.38	334	51.94	32.239	34.03	Araip.6328V	0.38
Aradu.73S33	0.391	611	47.386	38.889	41.993	Araip.FW2CL	0.384
Aradu.73ZLX	0.426	468	52.452	34.542	48.827	Araip.CBX44	0.417
Aradu.747H9	0.351	296	53.535	40.741	35.69	Araip.IID88	0.355
Aradu.747VP	0.39	647	47.685	40.895	38.735	Araip.4Z07Z	0.395
Aradu.749Z1	0.366	369	49.189	45.135	41.351	Araip.6AB1Y	0.349
Aradu.74DEV	0.352	856	51.342	39.673	39.907	Araip.55L6R	0.355
Aradu.74IXL	0.321	535	47.761	40.299	37.313	Araip.4SP4E	0.319
Aradu.74JTE	0.336	469	43.191	37.872	40	Araip.ZW937	0.343
Aradu.74KVK	0.386	515	49.225	43.798	44.186	Araip.348XC	0.384
Aradu.74N2Q	0.379	577	54.325	40.311	40.138	Araip.03Q00	0.386
Aradu.74X9S	0.318	311	50.962	46.795	27.885	Araip.2XW30	0.312
Aradu.751D4	0.478	515	56.589	47.481	63.372	Araip.SJI2G	0.487
Aradu.752JK	0.376	1365	52.269	39.678	36.31	Araip.L3SN7	0.362
Aradu.752ZV	0.315	275	46.739	38.043	35.145	Araip.LP6IV	0.327
Aradu.755RJ	0.259	146	53.741	49.66	42.177	Araip.J1LUN	0.252
Aradu.75AIB	0.306	236	42.616	41.772	39.241	Araip.Z0XNP	0.3
Aradu.75AY8	0.37	966	48.811	41.986	35.677	Araip.C1WRG	0.372
Aradu.75D3M	0.374	469	46.383	37.234	33.617	Araip.YJ8QA	0.383
Aradu.75ICF	0.347	527	42.614	34.659	43.371	Araip.VL1E0	0.352
Aradu.75JKD	0.356	460	49.458	37.31	44.252	Araip.QGQ22	0.353
Aradu.75PY6	0.359	562	41.918	42.806	40.497	Araip.6YN77	0.351
Aradu.76148	0.403	566	52.734	49.559	35.979	Araip.R3I3B	0.408

Aradu.763L8	0.383	125	46.825	34.921	50.794	Araip.6VR42	0.381
Aradu.764WC	0.308	111	48.214	33.929	39.286	Araip.2QS8R	0.308
Aradu.7673S	0.494	191	51.042	39.063	46.354	Araip.UTP9U	0.497
Aradu.767UQ	0.326	443	53.378	39.865	37.162	Araip.S0E50	0.329
Aradu.76BI5	0.393	603	50.828	40.232	39.57	Araip.Y683M	0.395
Aradu.76BXR	0.382	585	55.29	33.106	36.689	Araip.NXE7U	0.393
Aradu.76EJ2	0.469	285	64.685	45.804	70.629	Araip.819C3	0.483
Aradu.76H6A	0.383	465	58.369	41.416	42.06	Araip.27JTJ	0.383
Aradu.76UYG	0.345	655	47.256	37.5	38.11	Araip.AC28S	0.343
Aradu.76V5M	0.328	677	48.673	37.758	37.758	Araip.ZU18G	0.331
Aradu.76Y0H	0.354	728	55.693	35.802	34.019	Araip.0H44R	0.354
Aradu.771JD	0.375	168	43.787	41.42	36.686	Araip.05I70	0.379
Aradu.772NM	0.431	356	48.459	33.053	37.535	Araip.65SLH	0.428
Aradu.772YU	0.34	550	45.009	39.201	44.102	Araip.6J4W8	0.34
Aradu.77UYS	0.335	183	46.196	36.413	42.391	Araip.I1QS9	0.347
Aradu.77WYF	0.367	740	45.479	37.652	41.43	Araip.MZE77	0.371
Aradu.77XDI	0.411	401	50	41.791	45.025	Araip.MY2WL	0.409
Aradu.77XML	0.37	268	43.123	47.584	39.033	Araip.KF8QF	0.385
Aradu.77ZLW	0.338	570	49.212	40.105	36.602	Araip.387J2	0.327
Aradu.78C13	0.389	645	52.477	38.39	40.093	Araip.65HIY	0.398
Aradu.78FH9	0.444	469	47.66	41.915	48.085	Araip.I5LSS	0.436
Aradu.78G42	0.463	226	54.185	42.291	48.018	Araip.0L8ZM	0.468
Aradu.78HGD	0.416	256	40.467	46.693	40.856	Araip.78H4F	0.401
Aradu.78IK4	0.405	189	55.789	43.684	31.579	Araip.3266N	0.395
Aradu.78VGB	0.479	469	46.383	41.702	52.979	Araip.5AX8I	0.471
Aradu.78ZXW	0.395	227	50.877	41.667	39.474	Araip.4LG3M	0.382
Aradu.792L3	0.381	597	47.492	33.779	39.967	Araip.0HE2H	0.375
Aradu.792W7	0.396	470	48.832	51.38	42.675	Araip.CZ98B	0.398
Aradu.79876	0.351	556	49.91	40.575	44.704	Araip.BFL4V	0.356
Aradu.79FBE	0.39	345	49.422	44.22	36.705	Araip.45C6Q	0.393
Aradu.79JEZ	0.378	261	49.237	52.29	41.221	Araip.M42LB	0.382
Aradu.79MUY	0.415	151	46.053	41.447	58.553	Araip.G0FLH	0.429
Aradu.79NAD	0.375	544	47.523	33.028	51.376	Araip.NR5B5	0.348
Aradu.79PL2	0.413	211	37.736	45.755	41.981	Araip.R0657	0.398
Aradu.79Z90	0.438	135	43.382	44.118	45.588	Araip.SJD2F	0.487
Aradu.7A20D	0.333	288	47.751	35.294	25.952	Araip.T2WNF	0.36
Aradu.7A2KY	0.369	705	58.499	37.677	43.484	Araip.7G39W	0.371
Aradu.7AK58	0.333	276	51.625	47.292	32.491	Araip.XZ72K	0.348
Aradu.7AP79	0.382	298	46.154	35.117	39.799	Araip.09ESU	0.374
Aradu.7AZ8Z	0.35	106	46.729	33.645	47.664	Araip.KZ8MV	0.38
Aradu.7B3CD	0.374	457	53.493	37.991	43.013	Araip.T9KCB	0.372
Aradu.7B9HI	0.438	338	59.292	32.743	53.982	Araip.NWV62	0.428
Aradu.7BG9P	0.375	359	53.889	39.444	40	Araip.GX6C6	0.377
Aradu.7BV8W	0.342	518	45.665	38.343	42.389	Araip.14HQ9	0.332

Aradu.7C3KS	0.387	951	50.945	35.924	50.21	Araip.Y00HL	0.385
Aradu.7C8N3	0.491	223	55.357	42.411	56.25	Araip.J4IZA	0.491
Aradu.7C9V0	0.429	275	56.522	39.855	55.435	Araip.TD5YX	0.436
Aradu.7DV9M	0.369	1447	47.859	44.13	37.155	Araip.V99IN	0.367
Aradu.7E2JG	0.353	359	36.111	45	41.944	Araip.Z142E	0.347
Aradu.7E3FL	0.349	546	48.812	41.865	40.951	Araip.W9K1P	0.363
Aradu.7F3TN	0.344	391	53.571	39.031	44.133	Araip.L8VR7	0.333
Aradu.7F8A1	0.394	710	47.117	37.975	38.678	Araip.S8WH0	0.403
Aradu.7FN1T	0.388	310	51.768	37.299	50.804	Araip.X1WI1	0.411
Aradu.7FW6D	0.429	288	54.325	37.37	44.291	Araip.QJ62N	0.421
Aradu.7G8QU	0.444	615	57.143	44.968	44.156	Araip.SZR1J	0.438
Aradu.7G9TQ	0.277	144	51.034	41.379	32.414	Araip.C0H6M	0.262
Aradu.7GC8S	0.367	443	48.198	39.865	38.739	Araip.X3I39	0.355
Aradu.7GG37	0.336	355	43.539	35.674	41.573	Araip.BPU1I	0.347
Aradu.7GM9W	0.37	974	48.103	45.026	33.949	Araip.Q143D	0.372
Aradu.7GP5A	0.366	124	47.2	33.6	63.2	Araip.61SU1	0.385
Aradu.7GQ9E	0.367	171	48.256	47.093	31.977	Araip.SX1UB	0.362
Aradu.7GX01	0.363	220	45.249	34.842	38.462	Araip.CF4LQ	0.373
Aradu.7H178	0.402	474	50.737	36	59.789	Araip.3K50N	0.388
Aradu.7H2PW	0.437	461	48.701	42.424	53.03	Araip.ZMP4R	0.43
Aradu.7H3UM	0.343	407	47.304	38.235	37.255	Araip.NH4AA	0.335
Aradu.7H8MA	0.407	474	46.737	35.158	57.263	Araip.30QVU	0.402
Aradu.7H9F1	0.387	454	56.923	39.341	45.714	Araip.8Q65G	0.394
Aradu.7HE2F	0.425	234	51.915	37.447	57.447	Araip.6R5A3	0.416
Aradu.7HR5N	0.397	869	46.092	40.575	42.529	Araip.6W75R	0.392
Aradu.7HS2D	0.379	371	48.387	43.011	41.935	Araip.PTL0N	0.379
Aradu.7I0FT	0.398	605	50.33	38.449	51.815	Araip.19HBE	0.378
Aradu.7I20U	0.447	253	56.693	40.551	37.795	Araip.W9YFB	0.461
Aradu.7I425	0.517	185	45.699	43.011	53.226	Araip.A22ZE	0.517
Aradu.7I7HI	0.442	111	41.071	44.643	41.071	Araip.64TPH	0.442
Aradu.7I7Y0	0.381	168	49.704	27.811	57.988	Araip.X6YYU	0.406
Aradu.7I9FX	0.353	471	48.941	33.475	46.822	Araip.Q0TDL	0.347
Aradu.7J6XP	0.534	139	60	20	60	Araip.EB6ED	0.528
Aradu.7J78G	0.337	386	45.22	37.209	40.31	Araip.A7ZNN	0.353
Aradu.7J9TI	0.405	258	41.313	42.471	50.193	Araip.F2177	0.423
Aradu.7JU28	0.375	188	58.201	43.915	43.386	Araip.S3PA3	0.368
Aradu.7JU4H	0.416	312	48.243	41.534	40.895	Araip.0W082	0.407
Aradu.7JW9M	0.416	209	50	49.048	40.952	Araip.H27NU	0.419
Aradu.7JY58	0.396	1919	50.99	31.615	38.49	Araip.PE4FX	0.395
Aradu.7K02I	0.44	326	56.269	51.988	39.755	Araip.Q2FNA	0.437
Aradu.7K1LW	0.472	400	54.115	44.389	59.352	Araip.K6Z53	0.469
Aradu.7K62W	0.377	484	43.918	36.289	34.227	Araip.N444E	0.383
Aradu.7KC0A	0.345	420	50.831	37.055	43.705	Araip.E8195	0.338
Aradu.7KK7S	0.381	327	48.476	40.854	43.902	Araip.57HXG	0.384

Aradu.7KU2A	0.374	475	56.933	46.218	40.966	Araip.IA1HS	0.376
Aradu.7L12D	0.389	492	47.87	39.554	47.059	Araip.BQB3L	0.388
Aradu.7LR28	0.456	130	53.435	51.908	41.221	Araip.C16FL	0.464
Aradu.7LZ09	0.404	789	47.089	49.494	36.456	Araip.HA6TQ	0.41
Aradu.7M1P4	0.337	519	51.923	41.154	35.577	Araip.AV0UY	0.337
Aradu.7M26B	0.464	370	54.987	46.092	68.464	Araip.20V0X	0.467
Aradu.7M4Z2	0.41	1265	53.239	37.441	39.258	Araip.TMT3H	0.413
Aradu.7M5RR	0.382	370	46.631	36.658	39.892	Araip.DAS8U	0.387
Aradu.7MF12	0.353	519	45	37.115	32.5	Araip.EJ1UY	0.355
Aradu.7MF1E	0.432	248	55.422	48.594	38.956	Araip.U5J78	0.444
Aradu.7MV2V	0.382	525	44.106	35.932	39.734	Araip.QE1NF	0.388
Aradu.7MW89	0.376	241	52.066	39.256	40.083	Araip.1U5BB	0.385
Aradu.7N01K	0.358	137	42.754	36.957	49.275	Araip.M35KP	0.348
Aradu.7N1F7	0.36	580	46.299	41.136	35.112	Araip.V1SVU	0.367
Aradu.7N2H0	0.427	662	48.869	43.288	47.36	Araip.28YBL	0.421
Aradu.7N2UH	0.427	205	53.883	34.466	62.136	Araip.9LQ1M	0.432
Aradu.7N548	0.397	243	50	46.721	41.803	Araip.6PA9N	0.39
Aradu.7N75D	0.335	1103	52.899	44.112	33.243	Araip.10YD3	0.336
Aradu.7NI41	0.34	286	50.523	35.54	41.115	Araip.9MG9F	0.36
Aradu.7NM14	0.376	473	49.789	31.857	45.57	Araip.IM1XC	0.375
Aradu.7NY4Q	0.455	351	50.852	32.67	49.432	Araip.6IS7T	0.458
Aradu.7P38Z	0.407	254	54.118	40	43.137	Araip.EF2V0	0.421
Aradu.7P55W	0.37	600	48.419	43.428	35.441	Araip.PYU91	0.358
Aradu.7P5YA	0.38	229	50.435	40.87	42.609	Araip.VG2TJ	0.371
Aradu.7P7FQ	0.409	498	49.299	38.878	44.89	Araip.SV5JG	0.407
Aradu.7PG44	0.365	284	60.351	36.14	40	Araip.43FKD	0.365
Aradu.7PT52	0.429	115	43.103	53.448	32.759	Araip.WPA12	0.432
Aradu.7Q1J6	0.347	358	49.861	46.24	31.198	Araip.9A3ZM	0.344
Aradu.7Q4DG	0.415	1142	50.131	38.67	48.294	Araip.TT5YQ	0.403
Aradu.7Q4MS	0.373	1221	50.245	33.552	37.643	Araip.QN9GG	0.371
Aradu.7Q9WK	0.525	148	59.06	46.309	59.732	Araip.4V3JQ	0.518
Aradu.7QE0L	0.499	366	45.232	39.782	65.668	Araip.V7V2P	0.501
Aradu.7R439	0.369	377	44.709	30.423	44.18	Araip.1A9I8	0.366
Aradu.7R8IR	0.349	194	41.026	35.385	43.077	Araip.UJ932	0.363
Aradu.7RC99	0.483	464	47.097	43.011	61.505	Araip.PZ7ZB	0.479
Aradu.7S6GM	0.39	198	55.779	40.201	43.216	Araip.I90PW	0.402
Aradu.7SN56	0.38	337	45.266	39.645	52.959	Araip.KVM2C	0.382
Aradu.7T5LQ	0.467	112	61.062	46.018	53.097	Araip.1CJ82	0.467
Aradu.7T62P	0.437	249	56	43.6	59.2	Araip.52Z96	0.429
Aradu.7TI6W	0.337	434	48.736	35.862	41.839	Araip.59ZT3	0.348
Aradu.7U8XS	0.377	318	57.367	39.185	37.931	Araip.Y8LZ2	0.386
Aradu.7UX0U	0.388	434	52.414	36.322	40.92	Araip.G9ZI8	0.391
Aradu.7V00N	0.403	124	51.2	36.8	44.8	Araip.E5PIG	0.403
Aradu.7V6T8	0.414	216	44.24	46.544	41.935	Araip.8LI6X	0.414

Aradu.7VL9B	0.343	495	48.79	36.492	38.508	Araip.9F87C	0.339
Aradu.7VP5C	0.358	546	46.984	41.316	36.929	Araip.AZ3UI	0.361
Aradu.7VQ43	0.439	318	58.934	42.633	51.724	Araip.20HUP	0.436
Aradu.7VY22	0.371	503	48.214	44.444	36.508	Araip.83671	0.382
Aradu.7W6T8	0.434	164	47.273	44.242	40	Araip.Q0ACF	0.434
Aradu.7W7QL	0.374	522	54.493	40.153	39.006	Araip.XMH4N	0.386
Aradu.7W9JR	0.465	443	54.054	45.495	67.342	Araip.EV8CZ	0.464
Aradu.7WF2V	0.365	498	42.285	46.493	35.471	Araip.ZQ3CB	0.365
Aradu.7WG5V	0.416	588	49.576	42.275	48.217	Araip.NUB09	0.415
Aradu.7WJ9D	0.393	629	63.333	35.238	61.905	Araip.041RZ	0.404
Aradu.7X3HU	0.462	347	50.575	40.517	46.264	Araip.W4V3G	0.474
Aradu.7X64R	0.369	690	45.876	35.456	40.955	Araip.H796R	0.368
Aradu.7X679	0.416	158	52.201	38.994	44.025	Araip.SD6PL	0.423
Aradu.7X826	0.404	152	51.634	48.366	59.477	Araip.5FG6Z	0.395
Aradu.7X8FI	0.352	768	48.114	36.931	43.693	Araip.RL90M	0.35
Aradu.7XA36	0.368	752	47.676	40.106	41.965	Araip.0V0EF	0.373
Aradu.7XU9R	0.389	355	56.742	40.169	49.157	Araip.8Y65S	0.421
Aradu.7Y15X	0.51	157	51.899	50	64.557	Araip.R8SCL	0.516
Aradu.7Y1E8	0.412	145	56.164	34.932	68.493	Araip.HUA7A	0.441
Aradu.7Y2HQ	0.375	787	50.888	39.34	35.406	Araip.87PEG	0.378
Aradu.7Y3DJ	0.41	498	50.701	43.086	35.471	Araip.PBT3B	0.406
Aradu.7Y76M	0.413	495	62.903	40.121	49.194	Araip.D6WDG	0.422
Aradu.7YA73	0.37	1085	54.88	37.937	35.359	Araip.U3V28	0.37
Aradu.7YJ00	0.386	645	53.406	38.39	39.009	Araip.S9VNZ	0.389
Aradu.7YM1I	0.39	104	39.048	40	31.429	Araip.BYV00	0.38
Aradu.7Z02Q	0.367	712	55.119	42.917	44.741	Araip.Y0ZFX	0.37
Aradu.7Z2RH	0.426	485	53.086	38.066	50.617	Araip.DU7GL	0.414
Aradu.7Z549	0.379	519	45.385	35.769	43.462	Araip.UTX5W	0.4
Aradu.7ZG0H	0.421	244	55.918	41.633	33.878	Araip.P77MW	0.429
Aradu.7ZK0R	0.374	358	46.518	42.061	38.997	Araip.H1RCV	0.382
Aradu.8005S	0.373	422	52.009	45.863	37.116	Araip.IHG6N	0.365
Aradu.8025N	0.392	803	54.353	36.318	43.905	Araip.87MXF	0.385
Aradu.802R8	0.326	995	48.795	35.141	32.631	Araip.7P115	0.336
Aradu.805L3	0.399	433	46.083	42.166	39.401	Araip.GT9SI	0.393
Aradu.808LA	0.413	205	58.738	43.689	47.087	Araip.V72QV	0.425
Aradu.80AKC	0.45	577	52.768	45.156	51.557	Araip.C4716	0.454
Aradu.80C88	0.398	424	55.294	42.353	39.529	Araip.E7AL7	0.393
Aradu.80EYC	0.431	459	58.261	41.739	51.087	Araip.YPF2X	0.444
Aradu.80RUZ	0.314	772	44.761	40.75	34.411	Araip.5E8U9	0.32
Aradu.80TC2	0.346	1143	49.65	34.79	40.822	Araip.Y9SXC	0.347
Aradu.80TQM	0.341	228	53.275	29.694	45.415	Araip.N829R	0.35
Aradu.80U52	0.356	434	46.897	40.92	37.011	Araip.Z4GMI	0.342
Aradu.80WBV	0.395	767	50.391	43.62	44.141	Araip.X40X6	0.413
Aradu.810XL	0.401	473	51.899	34.599	45.57	Araip.NV5LW	0.407

Aradu.81GPB	0.373	1443	49.1	40.512	38.435	Araip.XVV6S	0.375
Aradu.81HAH	0.375	746	45.248	33.601	38.688	Araip.1KE3S	0.381
Aradu.81KMB	0.393	552	47.016	43.58	45.75	Araip.2TV5Y	0.396
Aradu.81LQN	0.347	594	46.218	44.538	36.303	Araip.B861E	0.353
Aradu.81LXC	0.382	606	53.213	41.021	43.328	Araip.00JXM	0.392
Aradu.81PPC	0.37	706	50.354	44.413	40.17	Araip.83U1D	0.363
Aradu.81RLF	0.393	348	52.149	43.84	44.126	Araip.3UW0X	0.414
Aradu.81UZ4	0.339	282	40.283	31.095	33.569	Araip.UE4FG	0.343
Aradu.81WR9	0.365	291	56.164	39.384	42.123	Araip.WT4L7	0.381
Aradu.81WTS	0.417	320	54.829	38.318	40.187	Araip.4I67D	0.417
Aradu.81XX0	0.397	147	43.919	37.838	33.108	Araip.MG0IA	0.39
Aradu.8203M	0.388	773	53.747	47.933	33.721	Araip.PTB9G	0.39
Aradu.826D1	0.392	179	36.667	43.889	39.444	Araip.PT656	0.394
Aradu.82ABY	0.43	353	52.825	39.831	63.277	Araip.57QUV	0.423
Aradu.82C4A	0.42	151	56.579	23.684	38.158	Araip.C6ZL6	0.42
Aradu.82FNR	0.42	151	50.658	31.579	41.447	Araip.PH8DZ	0.449
Aradu.82KWN	0.379	451	52.655	39.381	45.575	Araip.6IK4X	0.384
Aradu.82P2N	0.37	846	46.281	46.163	42.385	Araip.GBD9V	0.372
Aradu.82P95	0.382	468	52.452	46.482	44.35	Araip.4PT6K	0.377
Aradu.82T8W	0.378	684	45.839	39.562	39.416	Araip.JPK9F	0.383
Aradu.82TF8	0.451	262	55.133	39.544	36.502	Araip.T2HWD	0.455
Aradu.82VY6	0.389	1174	51.66	31.404	37.021	Araip.14A0C	0.389
Aradu.83253	0.354	528	51.796	38.374	37.24	Araip.Y8KWK	0.348
Aradu.83CAT	0.35	603	57.616	40.563	36.921	Araip.E0GHH	0.366
Aradu.83F0C	0.459	342	51.895	47.522	50.729	Araip.KY0TY	0.462
Aradu.83I6G	0.357	407	57.108	43.627	37.5	Araip.X4PFH	0.351
Aradu.83KXV	0.331	302	49.175	36.634	41.254	Araip.JE2NP	0.329
Aradu.83MPA	0.444	263	53.03	50.758	56.061	Araip.CB643	0.438
Aradu.83Q97	0.397	652	48.239	47.779	33.844	Araip.F02DS	0.388
Aradu.83RRW	0.381	518	49.711	42.004	35.067	Araip.XL42X	0.393
Aradu.83UZ1	0.372	190	34.555	49.215	35.602	Araip.G3762	0.396
Aradu.83Z01	0.5	281	55.674	51.418	52.128	Araip.P8G5K	0.513
Aradu.8479S	0.355	401	56.468	41.791	34.577	Araip.EBH0J	0.363
Aradu.8482Y	0.32	114	46.957	26.957	36.522	Araip.S8IS2	0.32
Aradu.84U8V	0.374	483	43.802	40.909	46.488	Araip.WNH0S	0.38
Aradu.84XE2	0.436	116	36.752	34.188	52.137	Araip.1N6HI	0.427
Aradu.8542F	0.349	560	45.455	31.194	30.838	Araip.NWK8S	0.329
Aradu.8567N	0.355	868	54.315	37.745	33.947	Araip.C4RSW	0.378
Aradu.857YF	0.356	301	42.715	42.384	38.079	Araip.SD6FV	0.388
Aradu.85E8I	0.391	378	45.119	35.62	39.842	Araip.ZND2G	0.382
Aradu.85GH9	0.384	845	53.31	39.835	37.234	Araip.L38X1	0.379
Aradu.85KJF	0.389	475	54.412	41.597	53.571	Araip.1MS7W	0.391
Aradu.85WAE	0.351	343	48.837	42.442	42.151	Araip.6N0Z9	0.351
Aradu.862GA	0.477	319	44.688	49.688	40.938	Araip.KL088	0.464

Aradu.86BB6	0.373	632	45.024	33.333	46.445	Araip.ZP5QD	0.366
Aradu.86C6D	0.371	837	54.773	44.272	41.169	Araip.Z1EXY	0.372
Aradu.86I8Y	0.43	116	54.701	58.974	50.427	Araip.U0G8M	0.439
Aradu.86IQQ	0.418	407	42.157	42.157	40.441	Araip.YK7C2	0.419
Aradu.86KS5	0.427	302	52.145	44.224	42.904	Araip.C841I	0.41
Aradu.86SPU	0.399	433	49.539	44.7	53.917	Araip.M9I94	0.41
Aradu.870BG	0.386	705	49.008	43.768	36.686	Araip.L0ZBU	0.383
Aradu.875VG	0.382	529	49.245	46.226	37.925	Araip.887KB	0.383
Aradu.87FCC	0.323	104	49.524	51.429	40.952	Araip.AS26T	0.343
Aradu.87IV3	0.388	568	51.494	40.773	39.543	Araip.RDY1Z	0.394
Aradu.87LE7	0.444	381	53.141	44.241	52.356	Araip.EN4UY	0.438
Aradu.87SDL	0.347	1011	56.225	47.53	35.277	Araip.VU8GK	0.35
Aradu.87VJS	0.351	197	48.485	39.394	47.98	Araip.GRP0P	0.345
Aradu.87YLX	0.31	294	42.712	36.949	32.203	Araip.8T3QY	0.303
Aradu.88125	0.415	176	49.153	41.808	30.508	Araip.2U0KZ	0.409
Aradu.88298	0.386	460	50.976	38.612	36.226	Araip.60IH6	0.39
Aradu.883F2	0.371	222	43.498	37.668	43.498	Araip.287MP	0.386
Aradu.883SN	0.348	184	44.865	40.541	38.919	Araip.GLT9E	0.328
Aradu.889PJ	0.389	216	46.083	37.788	40.092	Araip.WB46L	0.379
Aradu.88AFG	0.434	364	49.863	45.479	45.479	Araip.K8KF4	0.44
Aradu.88CDZ	0.352	519	45.769	39.615	37.5	Araip.RNC6M	0.371
Aradu.88CYL	0.401	368	48.78	39.295	36.314	Araip.DM3HR	0.403
Aradu.88DVA	0.35	418	52.267	49.881	36.277	Araip.U50TM	0.347
Aradu.88E60	0.526	280	55.872	46.619	67.26	Araip.ZF8FB	0.518
Aradu.88EGA	0.332	386	42.636	44.703	37.984	Araip.JFW8D	0.321
Aradu.88HY6	0.363	884	51.299	40.565	36.836	Araip.M0RYJ	0.354
Aradu.88IFJ	0.32	383	47.135	51.563	49.479	Araip.QCG77	0.332
Aradu.88K72	0.359	393	53.046	39.594	42.132	Araip.0ZS3I	0.352
Aradu.88KAK	0.357	572	51.134	38.045	38.045	Araip.RBD33	0.357
Aradu.88KLW	0.354	490	54.582	42.363	43.788	Araip.Q9TXG	0.35
Aradu.88QB9	0.401	414	46.506	42.169	43.855	Araip.AG87Q	0.398
Aradu.88UWV	0.343	283	46.479	35.563	38.38	Araip.RL9T9	0.369
Aradu.88VQK	0.419	287	52.778	38.889	30.208	Araip.I55WQ	0.426
Aradu.88Z5A	0.38	670	51.416	42.325	40.984	Araip.1J28K	0.376
Aradu.88Z5T	0.399	470	49.257	40.127	39.703	Araip.KX771	0.39
Aradu.88Z6K	0.389	491	50.61	41.26	36.179	Araip.2YE37	0.398
Aradu.89624	0.406	493	47.773	39.474	45.749	Araip.6Y0I9	0.4
Aradu.898CQ	0.395	415	44.952	37.26	37.981	Araip.M9JCH	0.388
Aradu.898PR	0.413	163	42.073	39.024	55.488	Araip.TQF5F	0.397
Aradu.89I67	0.369	508	50.295	44.204	38.114	Araip.8U4CD	0.367
Aradu.89S1X	0.4	325	50	40.491	58.282	Araip.J2LD8	0.394
Aradu.89TY1	0.403	652	54.518	40.582	40.276	Araip.CI33Y	0.399
Aradu.89XIU	0.362	214	60	29.302	39.535	Araip.D20VZ	0.37
Aradu.89ZRN	0.378	658	54.78	35.66	33.991	Araip.0Z5TL	0.385

Aradu.8A153	0.363	255	48.438	43.359	42.969	Araip.2ZN3C	0.363
Aradu.8A22P	0.328	488	45.808	38.037	33.947	Araip.R511F	0.339
Aradu.8A403	0.436	296	53.872	40.741	37.71	Araip.S6JZ9	0.42
Aradu.8A5JB	0.377	325	51.84	38.65	39.264	Araip.QU06Y	0.367
Aradu.8A8WP	0.394	763	49.084	38.22	44.634	Araip.10QHS	0.405
Aradu.8AC2D	0.395	242	51.852	43.21	40.329	Araip.BQ8ZI	0.414
Aradu.8B0HV	0.411	111	50	48.214	34.821	Araip.NH7YS	0.411
Aradu.8C0WH	0.442	486	62.218	36.756	59.343	Araip.CV8RW	0.435
Aradu.8C561	0.341	742	50.067	39.031	33.782	Araip.F5WNS	0.337
Aradu.8C9LT	0.371	661	50.302	40.785	35.952	Araip.XF76V	0.381
Aradu.8C9N3	0.407	391	43.112	45.918	31.378	Araip.WZP2U	0.414
Aradu.8CB01	0.403	311	45.513	32.372	42.308	Araip.NL989	0.407
Aradu.8CG1C	0.429	332	52.252	43.544	42.643	Araip.2X3C3	0.429
Aradu.8D6KF	0.393	404	50.864	42.963	38.272	Araip.7274A	0.408
Aradu.8DJ4R	0.486	296	52.862	45.455	64.983	Araip.580BK	0.483
Aradu.8E0AS	0.421	185	55.376	25.806	33.871	Araip.XP5YM	0.393
Aradu.8E8GF	0.421	595	52.013	38.423	36.577	Araip.AK5TZ	0.412
Aradu.8ET3H	0.423	109	40.909	36.364	42.727	Araip.UJT36	0.423
Aradu.8EU70	0.403	485	49.383	50	44.65	Araip.IUR9I	0.408
Aradu.8F3EB	0.419	509	45.49	53.137	41.569	Araip.GEV84	0.427
Aradu.8F49F	0.365	436	54.233	39.588	40.503	Araip.E0U35	0.377
Aradu.8F945	0.433	354	40.563	37.746	47.887	Araip.N04DW	0.436
Aradu.8FB72	0.376	929	52.258	39.462	41.613	Araip.N7N6Z	0.376
Aradu.8G40J	0.298	329	50	43.333	37.879	Araip.Y1R86	0.298
Aradu.8G7WX	0.402	374	51.733	42.4	36.8	Araip.C5C73	0.4
Aradu.8G81K	0.361	358	53.76	53.482	55.71	Araip.0C8GZ	0.364
Aradu.8G93R	0.361	1096	51.869	37.375	34.184	Araip.DDD6V	0.355
Aradu.8GE4T	0.363	302	45.215	43.564	31.353	Araip.Z3FUF	0.361
Aradu.8GK1W	0.393	427	53.738	40.888	35.28	Araip.0Y2KA	0.394
Aradu.8GL4A	0.345	884	42.486	35.706	38.079	Araip.24ZZQ	0.347
Aradu.8GS2N	0.39	196	51.269	37.056	47.716	Araip.I1EY6	0.387
Aradu.8GW0S	0.445	176	43.503	39.548	41.808	Araip.0DM6L	0.451
Aradu.8H45I	0.435	281	58.511	49.645	56.738	Araip.ZXN9C	0.446
Aradu.8HB43	0.342	421	48.815	36.256	41.943	Araip.BXY3B	0.345
Aradu.8I12T	0.511	484	52.371	38.557	65.773	Araip.EZ4HJ	0.504
Aradu.8I2Q1	0.393	350	50.997	47.578	35.328	Araip.EKH4M	0.401
Aradu.8I3T0	0.439	318	51.411	42.006	47.962	Araip.72YG4	0.438
Aradu.8I79H	0.409	337	51.183	38.757	42.604	Araip.LA152	0.409
Aradu.8I7SF	0.331	544	47.89	44.587	34.495	Araip.6759X	0.346
Aradu.8I8L4	0.469	466	53.747	47.109	56.103	Araip.GM86N	0.475
Aradu.8II80	0.355	771	48.964	43.135	43.394	Araip.Z789E	0.352
Aradu.8IN6N	0.413	574	51.826	40.348	40.174	Araip.L7NBZ	0.411
Aradu.8IQ81	0.486	316	49.211	46.688	48.58	Araip.SPY8U	0.488
Aradu.8IX7E	0.391	620	49.436	39.452	44.283	Araip.Y67XK	0.386

Aradu.8J00F	0.459	500	48.503	39.92	57.285	Araip.L1D01	0.447
Aradu.8J5I0	0.372	1032	50.823	38.045	42.11	Araip.Y987Q	0.366
Aradu.8J98F	0.349	362	48.485	58.127	39.669	Araip.1WE6F	0.346
Aradu.8K2VK	0.428	248	61.446	54.217	43.775	Araip.J8DCC	0.436
Aradu.8K8TN	0.495	289	51.724	46.207	55.172	Araip.2JP01	0.5
Aradu.8KB8X	0.354	349	54.857	34.571	28.857	Araip.N68W5	0.359
Aradu.8KH6E	0.33	239	47.917	32.083	49.167	Araip.F0RXE	0.339
Aradu.8KW68	0.42	370	48.518	41.779	52.291	Araip.F0TL2	0.428
Aradu.8L7DK	0.372	662	46.606	38.763	40.573	Araip.UYW0K	0.372
Aradu.8L8L4	0.409	446	52.796	39.15	54.586	Araip.1K0LY	0.4
Aradu.8L9C6	0.42	639	46.875	33.594	45.469	Araip.UK2C9	0.421
Aradu.8LE5E	0.385	494	59.192	46.263	37.576	Araip.11SIA	0.382
Aradu.8LR63	0.446	855	54.322	42.757	40.07	Araip.JH5QN	0.447
Aradu.8LY9S	0.378	465	48.712	48.283	41.631	Araip.LYD5Q	0.378
Aradu.8M02Z	0.285	311	57.051	41.026	31.09	Araip.MZ6FM	0.307
Aradu.8M51F	0.413	470	50.106	40.764	40.34	Araip.SA9TP	0.396
Aradu.8MB2V	0.355	571	45.629	37.762	36.538	Araip.C8IBG	0.36
Aradu.8N3KD	0.441	186	55.08	31.016	43.85	Araip.E3Z66	0.442
Aradu.8ND9A	0.409	492	53.347	44.219	37.728	Araip.N0NQI	0.415
Aradu.8NK92	0.379	321	53.106	34.472	42.547	Araip.ZJB2Y	0.381
Aradu.8P3KF	0.381	559	49.821	39.107	35.536	Araip.S802V	0.384
Aradu.8P3R8	0.42	603	48.841	42.219	37.583	Araip.PV4IE	0.422
Aradu.8P6BN	0.407	585	53.584	33.447	49.317	Araip.SB6R7	0.401
Aradu.8PE87	0.392	128	48.062	42.636	33.333	Araip.38JBI	0.392
Aradu.8PJ2J	0.349	529	48.302	42.075	42.453	Araip.89K67	0.35
Aradu.8Q0H7	0.431	542	48.987	36.096	39.042	Araip.5P589	0.435
Aradu.8Q40H	0.403	859	53.256	40.814	35.698	Araip.F3RJ4	0.403
Aradu.8Q4X6	0.41	1194	51.883	37.573	46.527	Araip.AVR8T	0.41
Aradu.8Q6IV	0.436	351	49.716	38.636	51.136	Araip.BP0EH	0.444
Aradu.8Q9T1	0.367	887	49.437	37.725	38.739	Araip.CF54H	0.368
Aradu.8QB2V	0.333	648	51.002	42.373	40.37	Araip.J4RH5	0.334
Aradu.8QE54	0.371	109	40.909	36.364	40.909	Araip.74FNF	0.362
Aradu.8QI3B	0.404	339	47.941	40.294	40	Araip.54Y6W	0.399
Aradu.8QL4Z	0.416	345	50.867	39.595	55.491	Araip.ZW9IZ	0.428
Aradu.8R2K8	0.403	515	52.132	39.341	42.248	Araip.SV7HB	0.408
Aradu.8R77K	0.342	483	51.446	38.223	38.43	Araip.SX9F2	0.346
Aradu.8S04V	0.373	303	49.671	43.092	41.447	Araip.71J30	0.385
Aradu.8S28F	0.443	305	45.752	42.81	46.078	Araip.DF76F	0.458
Aradu.8S4TL	0.357	381	54.45	40.314	46.073	Araip.U4R4L	0.359
Aradu.8SH4F	0.35	112	37.168	40.708	50.442	Araip.QC86V	0.375
Aradu.8SL2K	0.356	718	54.103	39.499	37.135	Araip.13I5K	0.355
Aradu.8T260	0.409	558	54.383	44.723	39.356	Araip.CB2CZ	0.409
Aradu.8T38A	0.371	379	47.895	38.158	40.263	Araip.177Y0	0.388
Aradu.8T3G1	0.343	256	50.584	37.354	43.191	Araip.UUZ6A	0.328

Aradu.8T3RP	0.36	1518	49.309	38.512	41.277	Araip.J7NVV	0.368
Aradu.8T892	0.419	408	51.834	41.809	53.301	Araip.JLC9M	0.374
Aradu.8U0A6	0.327	328	48.632	34.954	32.523	Araip.RMK33	0.346
Aradu.8U64A	0.341	193	53.093	35.052	42.268	Araip.S4DRB	0.332
Aradu.8UD68	0.41	462	50.756	49.676	36.285	Araip.3VW7G	0.404
Aradu.8UL0Z	0.415	290	43.643	49.141	34.021	Araip.FP6KG	0.414
Aradu.8UL46	0.377	171	47.093	31.977	43.023	Araip.270IQ	0.383
Aradu.8UZ9S	0.367	158	41.509	30.818	47.799	Araip.RPL4T	0.325
Aradu.8V3QK	0.448	667	54.79	35.928	65.868	Araip.94DKY	0.452
Aradu.8V764	0.413	109	40.909	47.273	55.455	Araip.9I7A7	0.413
Aradu.8V7CS	0.347	533	48.502	45.318	35.019	Araip.ZV22A	0.347
Aradu.8VI8X	0.413	386	50.129	43.669	48.062	Araip.1BL7S	0.412
Aradu.8VP1A	0.427	244	50.612	40.408	46.939	Araip.4786J	0.441
Aradu.8VT0P	0.366	762	50.197	46.789	34.207	Araip.JZV3C	0.367
Aradu.8W1FQ	0.376	165	46.988	37.349	34.94	Araip.JM8Z9	0.359
Aradu.8W1TJ	0.402	695	54.885	44.397	37.644	Araip.9K299	0.405
Aradu.8W40E	0.382	951	45.168	38.761	40.231	Araip.K50Y0	0.377
Aradu.8W59U	0.336	346	45.533	37.752	34.582	Araip.MYF07	0.323
Aradu.8WJ1L	0.396	566	55.026	40.564	40.035	Araip.KS112	0.396
Aradu.8WJ4P	0.399	436	49.199	38.902	39.359	Araip.HIE0T	0.407
Aradu.8X203	0.309	225	49.558	42.92	38.053	Araip.Q1XVU	0.318
Aradu.8X20T	0.342	778	52.118	41.207	36.585	Araip.ZQ4WS	0.348
Aradu.8X6B9	0.423	212	49.296	36.62	47.887	Araip.VBB34	0.418
Aradu.8X6ET	0.362	409	48.293	37.805	34.634	Araip.1C4CW	0.359
Aradu.8Y02T	0.462	191	52.083	39.063	59.896	Araip.RR9FR	0.457
Aradu.8Y4W7	0.326	633	55.521	36.751	38.959	Araip.VLF9V	0.324
Aradu.8Y5VQ	0.426	151	51.974	42.763	52.632	Araip.90BCU	0.432
Aradu.8Y6AX	0.392	238	55.649	41.841	31.381	Araip.XJ4FW	0.392
Aradu.8Z0S4	0.363	1283	48.52	38.785	39.174	Araip.1N36Z	0.363
Aradu.8Z4L7	0.402	311	52.564	35.897	62.179	Araip.C4EXT	0.402
Aradu.8Z92W	0.422	403	49.01	36.634	63.366	Araip.9VV1K	0.422
Aradu.8Z9JB	0.39	258	45.56	35.907	46.332	Araip.VH74R	0.398
Aradu.8ZT7E	0.402	524	50.857	44	36.762	Araip.60Y07	0.398
Aradu.901R7	0.473	248	55.823	47.791	48.594	Araip.XJU6V	0.473
Aradu.902BX	0.321	161	47.531	40.741	38.889	Araip.645I6	0.321
Aradu.903PW	0.446	465	50.215	40.558	69.099	Araip.L0D82	0.454
Aradu.908DD	0.366	451	48.673	34.735	41.15	Araip.PMK11	0.363
Aradu.90B63	0.335	660	48.109	37.519	41.604	Araip.0J6W3	0.332
Aradu.90H62	0.313	117	51.695	42.373	33.898	Araip.X4YKT	0.313
Aradu.90MFE	0.397	603	52.649	39.901	40.563	Araip.3A27H	0.392
Aradu.90QAV	0.414	242	47.325	52.675	47.737	Araip.3V1RA	0.429
Aradu.90QVH	0.513	197	51.515	40.404	47.98	Araip.2I7WK	0.497
Aradu.90RE6	0.419	271	55.147	45.221	45.956	Araip.64P0C	0.415
Aradu.90S6X	0.386	264	49.811	44.906	45.66	Araip.FD663	0.386

Aradu.90SID	0.338	138	43.885	30.935	33.094	Araip.Y03QP	0.382
Aradu.90TGE	0.402	591	51.014	38.514	44.257	Araip.IYZ3X	0.409
Aradu.90WZG	0.349	786	51.461	40.915	36.722	Araip.7VZ5Q	0.347
Aradu.910RR	0.575	255	55.859	52.734	72.266	Araip.E24Q0	0.579
Aradu.91876	0.423	152	49.673	43.137	41.176	Araip.E9XAH	0.423
Aradu.919C4	0.448	222	54.26	34.081	61.883	Araip.6IE3Z	0.455
Aradu.91ENH	0.416	173	51.724	38.506	45.977	Araip.W6PWH	0.416
Aradu.91M2N	0.487	361	49.724	36.464	71.823	Araip.MLW7G	0.484
Aradu.91Y21	0.405	182	56.284	42.077	38.798	Araip.L4KJR	0.399
Aradu.926HI	0.389	616	54.943	45.705	56.564	Araip.2CJ7I	0.389
Aradu.92CPX	0.387	543	48.346	37.316	39.338	Araip.6NN2A	0.37
Aradu.92FHF	0.4	489	52.245	44.082	40.204	Araip.K5C2Q	0.405
Aradu.92ILA	0.451	262	58.175	34.601	52.852	Araip.HN6Y6	0.447
Aradu.92KA6	0.407	400	60.599	39.9	45.387	Araip.N6BQZ	0.41
Aradu.92KLI	0.422	725	64.187	53.168	39.256	Araip.12RXW	0.417
Aradu.92NW5	0.371	858	47.381	37.718	36.205	Araip.C2I9V	0.368
Aradu.92WMB	0.305	207	57.212	40.865	39.423	Araip.6JD6V	0.3
Aradu.92ZAJ	0.436	178	40.782	41.899	51.397	Araip.H70JX	0.482
Aradu.934D7	0.39	471	46.61	38.559	44.703	Araip.T3DX1	0.404
Aradu.934TY	0.432	383	58.854	42.448	52.604	Araip.JMT0Y	0.443
Aradu.939VA	0.32	594	45.882	38.319	35.63	Araip.M76BN	0.321
Aradu.93BUT	0.352	559	46.964	38.393	45	Araip.ZCE2V	0.347
Aradu.93CHA	0.367	311	54.167	33.654	42.308	Araip.E99A6	0.358
Aradu.93KPX	0.439	421	50.948	36.73	45.498	Araip.V6JZU	0.443
Aradu.93R3L	0.401	365	56.011	43.716	42.077	Araip.W6HJ2	0.41
Aradu.93TCK	0.376	354	44.789	40.282	41.972	Araip.60WGZ	0.397
Aradu.93VCV	0.452	279	52.857	36.786	45.357	Araip.VX6P0	0.456
Aradu.93WPA	0.355	1075	55.576	40.428	35.874	Araip.X0TV2	0.354
Aradu.93Y1F	0.299	600	52.745	39.268	40.266	Araip.U4Z8Q	0.299
Aradu.942ZP	0.389	192	50.777	49.741	39.896	Araip.Y4DBT	0.399
Aradu.944BF	0.348	362	56.198	39.669	34.711	Araip.175SR	0.354
Aradu.945F4	0.382	574	43.826	41.565	39.304	Araip.J385S	0.383
Aradu.947SP	0.432	325	53.374	45.399	42.331	Araip.PP1VA	0.445
Aradu.94BIF	0.365	376	48.276	47.48	45.358	Araip.D15G9	0.362
Aradu.94E63	0.354	1506	50.1	41.606	36.297	Araip.G799L	0.352
Aradu.94ETY	0.377	293	55.102	36.735	41.156	Araip.K9U7W	0.375
Aradu.94J1A	0.393	141	52.817	38.028	68.31	Araip.W4FSU	0.381
Aradu.94JTX	0.35	166	43.713	37.725	32.335	Araip.W1Z8X	0.353
Aradu.94Q3R	0.428	442	51.242	43.792	40.632	Araip.W932V	0.427
Aradu.94T7Q	0.435	296	48.485	34.68	43.098	Araip.7944J	0.442
Aradu.94UHF	0.382	624	47.68	38.56	41.12	Araip.TQI24	0.375
Aradu.94WDB	0.381	414	43.614	37.349	49.88	Araip.D9NIG	0.377
Aradu.94X53	0.372	561	49.11	45.73	41.103	Araip.4IT76	0.367
Aradu.94ZD7	0.41	328	38.298	42.249	41.945	Araip.R5QTL	0.407

Aradu.951UC	0.406	867	50.461	38.825	43.548	Araip.Q8LFT	0.409
Aradu.95964	0.315	224	56.444	36	37.333	Araip.ZNX0X	0.306
Aradu.959KY	0.489	238	58.159	42.259	55.23	Araip.V1VB7	0.481
Aradu.95B31	0.373	1442	52.391	44.699	36.244	Araip.908FR	0.382
Aradu.95DDC	0.326	359	47.5	39.444	39.722	Araip.WP65K	0.339
Aradu.95EP3	0.489	232	60.515	46.781	63.09	Araip.XF8RP	0.461
Aradu.95F2Q	0.379	453	55.727	41.41	39.427	Araip.SMI4I	0.387
Aradu.95FMB	0.411	976	56.397	42.682	36.029	Araip.YPU6N	0.411
Aradu.95REC	0.402	694	50.216	34.101	52.806	Araip.CW5YA	0.398
Aradu.95TME	0.315	331	45.181	43.976	42.47	Araip.NPM90	0.318
Aradu.95XXR	0.454	624	44.96	43.36	46.56	Araip.SR040	0.454
Aradu.9602J	0.335	1063	43.797	36.748	35.62	Araip.HKC53	0.336
Aradu.9631L	0.418	218	48.402	34.703	47.489	Araip.122Z7	0.409
Aradu.96DV9	0.394	447	49.33	43.304	43.527	Araip.TCN35	0.399
Aradu.96IA2	0.361	524	44.381	36.762	52.381	Araip.118R7	0.368
Aradu.96IDX	0.36	851	50.469	37.911	42.723	Araip.W8ITJ	0.351
Aradu.970IC	0.402	216	51.613	38.249	35.484	Araip.15B33	0.406
Aradu.970W1	0.5	188	48.677	41.799	65.608	Araip.LQ4US	0.505
Aradu.97AME	0.359	972	46.043	34.224	33.196	Araip.NRY2N	0.387
Aradu.97C3D	0.381	828	48.733	40.531	41.013	Araip.SM4VG	0.382
Aradu.97PVW	0.361	417	47.608	38.038	34.928	Araip.MKZ4E	0.366
Aradu.97TKQ	0.406	567	56.866	39.789	50.176	Araip.ATJ43	0.427
Aradu.97VNG	0.294	129	40	40.769	42.308	Araip.X0AN4	0.301
Aradu.97X4A	0.372	815	49.755	31.618	42.402	Araip.DPR39	0.385
Aradu.97XZI	0.348	296	43.434	48.148	30.976	Araip.8S6C1	0.345
Aradu.97ZLB	0.447	549	50.727	44.727	54.727	Araip.68YDH	0.457
Aradu.9841E	0.375	362	55.647	38.292	31.129	Araip.58SA1	0.373
Aradu.986KM	0.477	298	51.171	40.803	61.204	Araip.A9XWC	0.46
Aradu.988BI	0.43	261	50.763	28.244	41.985	Araip.92VW5	0.424
Aradu.98BJD	0.441	151	50	45.395	51.316	Araip.W4SPX	0.436
Aradu.98BQH	0.374	1702	47.211	36.171	41.339	Araip.U28W7	0.38
Aradu.98FLM	0.382	130	42.748	29.771	40.458	Araip.RCT1Q	0.382
Aradu.98K4P	0.416	1084	49.217	58.986	35.115	Araip.CMA8P	0.415
Aradu.98KHN	0.35	551	55.435	46.196	38.587	Araip.FM4II	0.363
Aradu.98KS9	0.379	606	49.259	37.727	43.328	Araip.I51KZ	0.401
Aradu.98PQS	0.358	486	45.175	36.55	40.862	Araip.W17SH	0.355
Aradu.98WRB	0.465	296	43.771	32.997	51.178	Araip.PPQ7L	0.461
Aradu.999EQ	0.363	241	46.694	40.909	43.802	Araip.G7PU7	0.372
Aradu.99AQ5	0.428	562	56.483	43.517	53.641	Araip.G3TW3	0.426
Aradu.99F66	0.359	292	49.488	36.177	40.956	Araip.7JJ4U	0.36
Aradu.99FEF	0.366	473	47.257	36.498	34.81	Araip.W0MG3	0.366
Aradu.99GQI	0.37	348	49.857	46.132	38.109	Araip.PM79Q	0.367
Aradu.99LXR	0.381	848	51.708	37.22	34.158	Araip.YI4UQ	0.378
Aradu.99PKX	0.325	295	45.946	41.216	37.162	Araip.T2XRD	0.327

Aradu.9A69L	0.423	426	50.351	45.667	40.515	Araip.E5HJE	0.418
Aradu.9AB3B	0.443	425	49.296	38.028	44.131	Araip.BDV5W	0.455
Aradu.9AI6Q	0.385	325	46.319	53.988	38.344	Araip.20CID	0.385
Aradu.9AT7R	0.398	259	51.154	42.308	36.154	Araip.ZN6CK	0.407
Aradu.9AW6M	0.392	515	45.736	46.318	39.535	Araip.ANL75	0.387
Aradu.9B60Q	0.381	754	44.768	35.364	43.179	Araip.XZ9JQ	0.381
Aradu.9B6LA	0.519	164	56.97	47.879	44.242	Araip.N6UK3	0.532
Aradu.9BC7H	0.418	342	52.77	38.484	48.688	Araip.FX5SI	0.406
Aradu.9BY1S	0.428	368	59.621	39.837	71.003	Araip.A0TQ5	0.425
Aradu.9C32Y	0.428	170	64.912	36.842	63.158	Araip.GM4CV	0.404
Aradu.9C8PR	0.336	447	40.848	46.652	29.241	Araip.A8YH6	0.34
Aradu.9C9UT	0.43	459	55.435	53.696	53.913	Araip.T7RN5	0.438
Aradu.9CT7A	0.474	501	51.195	54.781	46.813	Araip.19472	0.48
Aradu.9CU19	0.434	150	55.629	49.669	47.682	Araip.7D071	0.451
Aradu.9CU4Z	0.351	347	47.701	43.966	36.494	Araip.F9W15	0.372
Aradu.9D49Q	0.364	245	50	36.179	46.341	Araip.B6W7Y	0.364
Aradu.9D9RN	0.33	614	48.618	38.862	35.772	Araip.2Y6XY	0.332
Aradu.9DD9N	0.386	358	41.783	43.733	44.568	Araip.V8F5Z	0.403
Aradu.9DJ4L	0.412	188	49.735	33.862	41.799	Araip.2KU4C	0.42
Aradu.9DJ84	0.414	256	49.416	42.802	44.747	Araip.E7CDH	0.418
Aradu.9DS5H	0.386	680	53.304	38.326	42.731	Araip.M6U8Q	0.388
Aradu.9E2AM	0.413	517	50.579	55.985	31.66	Araip.EM2AJ	0.415
Aradu.9E2KY	0.353	627	48.089	46.815	27.707	Araip.HPE6X	0.345
Aradu.9E473	0.397	124	43.2	35.2	43.2	Araip.7V7BF	0.405
Aradu.9E8FC	0.373	511	51.758	41.211	40.43	Araip.LAW7P	0.372
Aradu.9EK2R	0.26	150	34.437	32.45	15.894	Araip.HEP3C	0.296
Aradu.9EZ7Z	0.442	518	48.94	38.536	57.418	Araip.HDT92	0.438
Aradu.9F1L9	0.402	625	46.805	38.978	40.415	Araip.Z3JAA	0.382
Aradu.9FZ57	0.415	751	57.713	41.223	39.096	Araip.IEF2F	0.412
Aradu.9G7GM	0.393	488	47.444	44.172	38.037	Araip.XM65N	0.404
Aradu.9G825	0.577	226	45.374	55.066	65.639	Araip.DN365	0.564
Aradu.9GN4P	0.432	674	49.926	43.111	48	Araip.WJ0C8	0.424
Aradu.9H3L9	0.346	413	48.551	37.923	36.715	Araip.S7WL6	0.348
Aradu.9H4V1	0.412	153	57.792	42.857	35.714	Araip.7I2ZN	0.407
Aradu.9H5DV	0.378	408	54.279	43.765	40.587	Araip.B2JYZ	0.378
Aradu.9HV89	0.356	280	48.754	45.196	33.096	Araip.X9ZFP	0.324
Aradu.9I2P6	0.379	433	55.76	44.931	49.078	Araip.V5ZSM	0.379
Aradu.9IB22	0.356	792	51.072	39.47	43.001	Araip.W1A0F	0.365
Aradu.9ID7S	0.365	529	53.019	40.377	35.472	Araip.D2YEW	0.342
Aradu.9J6N2	0.36	561	44.128	41.815	43.06	Araip.6Q5LU	0.358
Aradu.9J6QD	0.362	578	46.287	29.361	31.261	Araip.C41LK	0.332
Aradu.9JB4Z	0.309	557	41.577	35.305	27.778	Araip.HD4P6	0.31
Aradu.9JQ87	0.416	664	49.774	42.556	43.609	Araip.BT1DS	0.416
Aradu.9K72K	0.368	321	54.348	45.031	50.311	Araip.SM08A	0.368

Aradu.9K9AQ	0.337	965	49.586	39.234	35.093	Araip.44M02	0.336
Aradu.9L2RT	0.359	867	50.691	39.862	42.166	Araip.A16W6	0.355
Aradu.9L539	0.364	217	51.835	42.661	40.826	Araip.YB6JS	0.372
Aradu.9L879	0.388	453	46.916	37.885	45.374	Araip.XH5UB	0.373
Aradu.9LI83	0.333	810	46.116	40.197	37.731	Araip.62J85	0.335
Aradu.9LK7Y	0.405	706	51.061	29.986	43.564	Araip.U8WPZ	0.403
Aradu.9M2XX	0.364	389	46.154	35.385	36.154	Araip.C4VS6	0.379
Aradu.9M4ZC	0.336	438	47.38	40.319	35.763	Araip.K0UPW	0.341
Aradu.9M8FI	0.374	501	45.02	35.657	40.04	Araip.44Q3I	0.392
Aradu.9MA52	0.396	215	43.519	33.796	60.648	Araip.RW9NX	0.401
Aradu.9MF3N	0.384	152	50.98	39.869	35.948	Araip.L2XTS	0.37
Aradu.9MQ2E	0.389	320	47.352	40.81	39.564	Araip.9P5VU	0.381
Aradu.9MZ2X	0.287	342	47.813	35.277	29.446	Araip.EDE9C	0.29
Aradu.9NC59	0.414	515	51.55	37.984	46.124	Araip.WS1DN	0.418
Aradu.9ND07	0.387	628	50.556	40.382	38.951	Araip.S159U	0.388
Aradu.9NR8A	0.327	372	53.351	35.925	36.461	Araip.39CBS	0.338
Aradu.9P432	0.401	491	53.252	41.87	57.927	Araip.04X9B	0.412
Aradu.9P5M8	0.417	577	53.114	35.467	36.678	Araip.4YN76	0.424
Aradu.9P6VM	0.449	329	49.697	32.727	52.121	Araip.SC1T4	0.43
Aradu.9PD7U	0.392	322	47.368	41.486	45.201	Araip.ZET1U	0.39
Aradu.9Q1AJ	0.364	938	50.799	42.492	32.268	Araip.RKK5U	0.362
Aradu.9Q62W	0.302	192	43.523	41.969	41.969	Araip.J7793	0.333
Aradu.9QT6G	0.372	746	52.075	40.295	41.633	Araip.M4152	0.372
Aradu.9R07T	0.371	1090	50.321	38.955	39.963	Araip.15KTJ	0.368
Aradu.9R08Q	0.429	434	45.287	41.609	43.678	Araip.2I50M	0.414
Aradu.9R7J6	0.343	480	47.609	38.462	44.699	Araip.171V2	0.359
Aradu.9R9X3	0.416	401	52.736	40.299	43.284	Araip.1117E	0.423
Aradu.9RQ53	0.419	348	41.834	35.53	40.688	Araip.T97EY	0.406
Aradu.9RZ6U	0.391	383	55.208	42.448	34.375	Araip.MM388	0.382
Aradu.9S16L	0.417	669	47.164	37.015	46.716	Araip.VI6GW	0.419
Aradu.9S3Z5	0.359	336	52.226	38.279	36.202	Araip.7P2V7	0.343
Aradu.9S9RY	0.403	431	49.074	47.685	50.694	Araip.VDU5V	0.404
Aradu.9SB6V	0.409	207	49.038	38.942	37.019	Araip.6F88V	0.414
Aradu.9SL8H	0.328	279	57.143	33.571	36.429	Araip.220UP	0.333
Aradu.9SR9S	0.383	407	50.98	41.667	42.157	Araip.6ZJ3N	0.378
Aradu.9SY6W	0.393	1131	49.47	39.841	38.781	Araip.ZW511	0.39
Aradu.9T1SG	0.447	153	55.844	37.013	43.506	Araip.IVG5H	0.441
Aradu.9T44T	0.361	384	47.792	32.208	32.468	Araip.NI6UG	0.366
Aradu.9T7BM	0.368	488	55.419	39.059	36.605	Araip.2EA83	0.366
Aradu.9T8AF	0.377	388	49.614	38.817	35.99	Araip.IT4EA	0.364
Aradu.9TG4T	0.389	704	50.638	36.028	51.206	Araip.WX3WV	0.388
Aradu.9TL9J	0.441	418	51.313	40.573	31.026	Araip.3W2E6	0.44
Aradu.9TU3R	0.373	744	49.664	42.55	37.45	Araip.Z762D	0.366
Aradu.9TU4F	0.433	163	53.049	37.805	41.463	Araip.YF63Z	0.456

Aradu.9TY2N	0.43	273	55.109	33.577	47.445	Araip.G2WXB	0.45
Aradu.9U2NA	0.316	504	46.337	36.634	39.406	Araip.83N9U	0.318
Aradu.9UB80	0.377	351	48.58	35.227	44.034	Araip.M0LAQ	0.377
Aradu.9V11P	0.563	346	48.991	61.96	54.179	Araip.81HNQ	0.56
Aradu.9V9BC	0.381	546	50.823	42.23	46.069	Araip.3M36F	0.376
Aradu.9VB1R	0.373	651	54.141	37.73	36.656	Araip.42ERT	0.357
Aradu.9VB4E	0.359	458	47.059	45.098	39.216	Araip.Y3NGW	0.364
Aradu.9VQ7B	0.364	692	46.465	39.827	35.354	Araip.G4JRD	0.369
Aradu.9W61Z	0.382	461	50.866	36.797	38.961	Araip.X0WZQ	0.375
Aradu.9W64L	0.362	383	46.875	37.5	46.094	Araip.NY6BB	0.348
Aradu.9WH4P	0.414	175	50.568	30.114	32.955	Araip.FN28D	0.414
Aradu.9X2TD	0.373	455	58.333	41.667	40.57	Araip.76TXX	0.38
Aradu.9XX4W	0.345	687	47.238	39.244	38.953	Araip.ZPK2M	0.357
Aradu.9Y73E	0.342	501	58.964	47.012	54.98	Araip.Y8SSF	0.363
Aradu.9Y9CU	0.368	788	50.063	42.966	39.924	Araip.93U5S	0.361
Aradu.9YK5E	0.325	305	44.771	39.542	37.908	Araip.MU9UP	0.305
Aradu.9YL2E	0.339	1016	42.576	35.792	36.775	Araip.TZQ98	0.334
Aradu.9YL2F	0.308	294	48.814	30.508	42.034	Araip.IRM4P	0.319
Aradu.9Z02R	0.377	215	47.685	31.944	37.037	Araip.56C70	0.372
Aradu.9Z8LC	0.358	434	48.046	38.621	38.391	Araip.YAF9X	0.356
Aradu.9ZP9L	0.392	225	42.92	42.035	40.708	Araip.FP4H9	0.383
Aradu.A050J	0.385	1177	50.17	45.246	45.671	Araip.5Y8KI	0.392
Aradu.A0DL1	0.315	223	55.804	39.732	41.071	Araip.M8LL8	0.302
Aradu.A0FDR	0.33	102	55.34	29.126	39.806	Araip.G06Y7	0.31
Aradu.A0MU0	0.456	417	47.847	39.474	51.435	Araip.3C7MV	0.479
Aradu.A0SRT	0.37	233	47.863	37.179	42.735	Araip.GV3PW	0.377
Aradu.A0XEY	0.374	562	45.826	38.188	41.208	Araip.5H0GE	0.372
Aradu.A0Z5X	0.386	446	54.362	39.15	38.702	Araip.NP9KT	0.386
Aradu.A13V2	0.343	827	54.71	42.995	34.058	Araip.JX6GK	0.34
Aradu.A16D8	0.405	344	46.087	41.159	29.275	Araip.X6F7N	0.394
Aradu.A1BA5	0.399	707	53.955	40.395	43.22	Araip.3C14J	0.391
Aradu.A1C01	0.418	388	49.1	43.959	40.874	Araip.5N24I	0.423
Aradu.A1EFP	0.406	521	53.065	42.337	53.257	Araip.3MN07	0.395
Aradu.A1GM4	0.459	348	51.862	48.997	57.88	Araip.8T0TC	0.453
Aradu.A1KE0	0.372	324	53.538	38.154	37.538	Araip.81TFR	0.367
Aradu.A1MN7	0.376	486	51.54	40.041	43.121	Araip.UA25L	0.369
Aradu.A1XBQ	0.39	477	56.695	41.841	42.678	Araip.712IZ	0.393
Aradu.A205B	0.361	174	46.286	45.143	33.714	Araip.3A1VT	0.367
Aradu.A22XE	0.388	925	50.216	38.013	38.769	Araip.13N2J	0.393
Aradu.A24RB	0.557	224	60.444	48.444	68.444	Araip.T656T	0.557
Aradu.A252K	0.421	224	51.111	39.556	39.556	Araip.LK0RZ	0.416
Aradu.A2F1I	0.392	327	50	37.805	41.768	Araip.VV0DQ	0.392
Aradu.A2MQ0	0.382	412	50.121	39.467	38.983	Araip.T4FCA	0.38
Aradu.A2SS4	0.377	1792	51.868	45.789	33.017	Araip.9P4NA	0.383

Aradu.A2UTQ	0.378	560	48.307	43.137	50.089	Araip.W5PYV	0.384
Aradu.A2VXS	0.377	635	54.56	38.208	39.465	Araip.PM53A	0.382
Aradu.A2ZB2	0.347	158	49.057	37.736	38.365	Araip.F2LMB	0.353
Aradu.A2ZJG	0.368	272	55.678	46.154	50.183	Araip.H3LLI	0.372
Aradu.A32HC	0.368	604	50.579	45.124	39.339	Araip.FQ9GE	0.364
Aradu.A336V	0.384	409	61.22	42.439	47.317	Araip.8F6MX	0.382
Aradu.A36AV	0.406	206	49.275	35.749	40.58	Araip.QB7B2	0.385
Aradu.A3D3N	0.373	498	51.703	44.289	39.88	Araip.P0LZ1	0.359
Aradu.A3E1X	0.444	117	47.458	43.22	54.237	Araip.K48LN	0.43
Aradu.A3HX3	0.393	537	49.442	43.68	33.271	Araip.7C73C	0.393
Aradu.A3N3V	0.402	452	54.305	43.488	44.812	Araip.4Z02U	0.421
Aradu.A3TK2	0.463	237	51.261	42.857	51.681	Araip.G7QFC	0.47
Aradu.A42TE	0.372	626	48.963	39.234	37.321	Araip.V3WGE	0.376
Aradu.A42Y8	0.402	396	57.431	38.287	41.562	Araip.V7L8V	0.409
Aradu.A43U5	0.34	457	46.943	42.795	36.9	Araip.DI6YG	0.339
Aradu.A4737	0.359	852	50.41	44.08	36.928	Araip.HL1JU	0.364
Aradu.A49KD	0.39	853	52.342	39.696	38.29	Araip.GD2Y5	0.392
Aradu.A4DEK	0.35	226	55.507	33.04	51.542	Araip.RRZ2A	0.35
Aradu.A4FGM	0.436	400	44.389	40.15	59.102	Araip.U68SV	0.44
Aradu.A4ISL	0.359	180	49.171	40.884	37.569	Araip.U9HL1	0.37
Aradu.A5EC7	0.447	517	50.965	38.031	59.266	Araip.S7EMP	0.44
Aradu.A5HRL	0.465	355	48.876	42.978	44.101	Araip.6VN5N	0.47
Aradu.A5IW3	0.441	103	44.231	45.192	39.423	Araip.U2EM6	0.419
Aradu.A5MHD	0.361	276	51.625	31.047	48.014	Araip.GD59T	0.363
Aradu.A5UR9	0.338	752	45.817	36.653	28.685	Araip.FNI26	0.348
Aradu.A5XMW	0.38	714	51.469	38.601	40.699	Araip.2HU43	0.39
Aradu.A61EY	0.342	664	46.015	37.143	37.594	Araip.P4KQP	0.353
Aradu.A64FA	0.437	657	49.088	42.097	56.991	Araip.W3I5K	0.434
Aradu.A65JI	0.376	1309	53.053	43.13	37.023	Araip.UF354	0.376
Aradu.A68AJ	0.357	530	48.211	39.736	37.1	Araip.0675S	0.367
Aradu.A68HE	0.389	563	52.837	38.83	52.128	Araip.1X10D	0.393
Aradu.A68MB	0.343	607	52.961	38.158	36.184	Araip.LMV71	0.354
Aradu.A6BTS	0.39	257	57.752	39.147	41.86	Araip.H1AXV	0.389
Aradu.A6HVN	0.367	659	51.364	42.424	49.545	Araip.M3BFQ	0.388
Aradu.A6T5Y	0.379	515	49.806	37.791	50.581	Araip.98QEF	0.377
Aradu.A6VDY	0.413	450	43.237	35.698	36.142	Araip.R34A5	0.409
Aradu.A6WCN	0.376	162	46.626	41.104	42.331	Araip.03WUR	0.392
Aradu.A723B	0.37	479	47.917	42.292	38.333	Araip.H1AF4	0.366
Aradu.A77EC	0.385	712	55.259	37.868	52.595	Araip.MW8R0	0.385
Aradu.A7934	0.416	557	48.029	46.595	50.717	Araip.SS53P	0.406
Aradu.A7CW2	0.351	1078	52.363	37.349	38.091	Araip.RL56E	0.35
Aradu.A7GFW	0.546	161	55.556	44.444	67.901	Araip.MSG33	0.548
Aradu.A7Z3C	0.408	927	56.573	39.332	35.453	Araip.US4V4	0.411
Aradu.A87UA	0.365	185	53.226	29.032	56.452	Araip.86F6R	0.381

Aradu.A8886	0.397	1295	55.401	38.58	37.886	Araip.89DFU	0.395
Aradu.A8AWS	0.389	769	52.208	38.312	36.623	Araip.TA2YD	0.379
Aradu.A8B4S	0.397	384	53.247	40.779	43.117	Araip.RM8Z7	0.396
Aradu.A8ITS	0.322	586	43.271	32.538	35.605	Araip.QP80U	0.323
Aradu.A8KIV	0.39	567	57.57	29.401	57.218	Araip.N62I1	0.389
Aradu.A8KM2	0.38	339	50	35.882	43.529	Araip.M80H6	0.391
Aradu.A8RS9	0.396	464	49.032	40.645	49.892	Araip.QW0IT	0.402
Aradu.A8T4C	0.392	504	57.822	45.545	43.168	Araip.2RQ0L	0.417
Aradu.A90PK	0.372	458	46.841	39.216	39.869	Araip.EL8BY	0.359
Aradu.A97TU	0.293	241	44.215	26.033	47.934	Araip.B1QY2	0.296
Aradu.A984U	0.394	481	47.51	40.041	43.983	Araip.AU5HG	0.406
Aradu.A9CNL	0.436	580	49.053	38.382	41.308	Araip.E3K7S	0.43
Aradu.A9E1A	0.418	737	50.949	36.585	50.813	Araip.V45YR	0.415
Aradu.A9F8B	0.324	539	53.333	36.111	32.963	Araip.2I121	0.317
Aradu.A9FZ7	0.385	281	56.028	39.362	42.908	Araip.GC0FX	0.378
Aradu.A9H9T	0.377	180	50.276	43.646	33.149	Araip.PC28F	0.366
Aradu.A9NSU	0.4	266	42.697	36.704	38.951	Araip.RVR18	0.417
Aradu.A9RVD	0.458	389	52.051	45.128	46.667	Araip.K1YWU	0.448
Aradu.A9U3A	0.472	180	61.878	43.646	60.221	Araip.6787A	0.472
Aradu.A9U89	0.405	576	53.38	48.527	38.648	Araip.76SLC	0.411
Aradu.A9V55	0.341	732	51.296	35.334	39.154	Araip.I6XYE	0.344
Aradu.AA0QS	0.372	237	38.235	37.815	39.076	Araip.ED7B1	0.372
Aradu.AA5CG	0.405	953	52.201	41.614	48.847	Araip.EN7EZ	0.406
Aradu.AA5JL	0.41	356	50.98	36.134	41.457	Araip.C5XGE	0.41
Aradu.AA5UH	0.46	573	43.728	41.289	44.599	Araip.2H071	0.463
Aradu.AA5ZE	0.388	509	49.02	43.137	44.706	Araip.Q85BZ	0.381
Aradu.AA7LT	0.47	189	57.368	31.053	51.579	Araip.S49L1	0.467
Aradu.AA8NU	0.39	770	46.433	36.706	39.04	Araip.856EY	0.379
Aradu.AAH6J	0.356	790	45.259	39.317	39.444	Araip.RM36F	0.354
Aradu.AAN3U	0.368	281	48.582	36.879	33.688	Araip.S9EB1	0.359
Aradu.AAT7R	0.385	282	49.117	34.629	49.117	Araip.I13PT	0.39
Aradu.AB724	0.384	428	48.019	36.83	48.485	Araip.IT82Y	0.391
Aradu.AC0Z7	0.469	204	52.683	46.829	55.122	Araip.5S1QP	0.454
Aradu.AC9ZE	0.271	165	40.964	42.771	42.771	Araip.F3TE1	0.276
Aradu.ACG3Y	0.281	371	47.849	35.215	33.065	Araip.I7WHX	0.286
Aradu.ACY83	0.388	848	45.701	40.165	48.057	Araip.95WQJ	0.4
Aradu.ADH1A	0.474	194	48.718	49.744	49.231	Araip.3JF99	0.474
Aradu.ADH9Y	0.418	339	49.706	36.471	43.529	Araip.9NY3L	0.426
Aradu.AE07K	0.432	264	56.604	40	38.113	Araip.UMR2E	0.436
Aradu.AE6VJ	0.461	465	49.142	44.85	59.227	Araip.L49IE	0.469
Aradu.AE9WN	0.366	235	50	41.525	37.712	Araip.MX2SJ	0.371
Aradu.AEH7T	0.404	473	49.367	43.038	36.92	Araip.A830F	0.394
Aradu.AET7G	0.36	465	51.502	45.494	47.21	Araip.8J47Q	0.369
Aradu.AF0JE	0.329	469	45.319	38.511	40.638	Araip.TYL9N	0.325

Aradu.AF2FZ	0.381	438	47.38	29.613	41.686	Araip.Y1H47	0.384
Aradu.AF8HB	0.351	896	54.627	39.242	35.897	Araip.ZM608	0.364
Aradu.AF9V9	0.406	146	58.503	41.497	68.027	Araip.0JY6V	0.406
Aradu.AFB92	0.39	265	46.241	42.481	43.233	Araip.H0PL5	0.399
Aradu.AFL8M	0.341	687	49.709	42.006	34.012	Araip.PL90G	0.343
Aradu.AG70H	0.41	438	54.897	39.408	53.759	Araip.EL2NI	0.417
Aradu.AGS84	0.368	486	48.46	37.166	45.791	Araip.1Q8PX	0.37
Aradu.AH39E	0.359	1167	45.805	35.788	39.64	Araip.HQ67P	0.351
Aradu.AH5QJ	0.403	314	57.778	42.222	54.603	Araip.XS0WA	0.396
Aradu.AH9TD	0.371	363	55.769	48.626	40.11	Araip.05VR1	0.379
Aradu.AH9XG	0.38	256	59.922	43.58	42.802	Araip.4GL4N	0.363
Aradu.AHG3H	0.317	466	49.893	36.831	29.336	Araip.B4FBZ	0.314
Aradu.AI3EU	0.391	1010	45.895	38.081	45.697	Araip.4263U	0.394
Aradu.AJ236	0.407	640	45.554	42.59	45.554	Araip.U7ZF0	0.405
Aradu.AJ5ZZ	0.401	419	47.857	38.81	43.333	Araip.FG0YW	0.398
Aradu.AJZ98	0.373	863	52.546	39.468	41.088	Araip.K9ULC	0.368
Aradu.AK4LW	0.363	394	48.861	39.494	48.354	Araip.MQI8S	0.366
Aradu.AKB12	0.362	232	56.223	38.627	44.206	Araip.1E7ZD	0.345
Aradu.AKG36	0.459	329	51.818	44.242	50.909	Araip.K73B3	0.473
Aradu.AKP9L	0.373	503	49.008	41.865	47.222	Araip.F92FW	0.373
Aradu.AKR7M	0.472	167	52.976	45.833	70.833	Araip.6C7DA	0.473
Aradu.AL6NG	0.387	1206	54.267	33.554	39.685	Araip.9BI0F	0.389
Aradu.AL954	0.451	714	53.566	47.832	59.441	Araip.8H28J	0.445
Aradu.AM0CF	0.413	356	47.899	37.255	53.782	Araip.J32EY	0.397
Aradu.AM65N	0.376	488	50.716	35.992	41.718	Araip.IY8RA	0.373
Aradu.AM8Z8	0.384	349	51.429	44	39.714	Araip.1G2UE	0.371
Aradu.AM9MC	0.397	531	49.624	37.406	41.353	Araip.G85GD	0.393
Aradu.AM9WK	0.395	266	50.187	41.573	38.202	Araip.EL2JP	0.403
Aradu.AMA0U	0.39	311	45.192	33.333	42.628	Araip.R0EFA	0.343
Aradu.AN363	0.284	145	54.11	47.26	39.041	Araip.Q99H0	0.315
Aradu.AN6BF	0.375	612	57.586	48.613	37.684	Araip.JWK6Z	0.375
Aradu.AN6JJ	0.412	249	50.8	44.4	44	Araip.S7GYW	0.4
Aradu.ANI3L	0.366	343	50.581	39.826	31.105	Araip.60Q0E	0.387
Aradu.ANJ8N	0.363	3555	51.828	38.808	34.899	Araip.4X1DQ	0.362
Aradu.ANN7C	0.405	320	48.287	44.237	38.318	Araip.KN052	0.408
Aradu.ANS8T	0.36	722	49.378	42.047	38.589	Araip.PR0RR	0.362
Aradu.ANT5S	0.348	1095	43.066	36.405	36.588	Araip.W7ZUF	0.352
Aradu.AP5EP	0.374	548	41.348	42.623	41.53	Araip.G8MZ3	0.385
Aradu.AP5ZW	0.413	649	54.923	42.462	42.308	Araip.VL9RM	0.405
Aradu.AP7WX	0.433	158	48.428	37.736	46.541	Araip.H5I9V	0.435
Aradu.AP9TH	0.315	364	41.37	40.822	41.644	Araip.DX4S4	0.312
Aradu.APR5V	0.372	808	50.803	40.42	35.476	Araip.FJL9Y	0.376
Aradu.AQ0Z8	0.386	383	48.177	39.323	39.844	Araip.LB4BS	0.397
Aradu.AQ9N2	0.362	143	49.306	47.222	46.528	Araip.VB46U	0.355

Aradu.AQB31	0.359	370	43.396	36.119	32.884	Araip.T82XN	0.377
Aradu.AQJ6I	0.43	644	46.667	42.946	45.426	Araip.F92P4	0.429
Aradu.AR3UR	0.479	460	53.579	41.649	67.896	Araip.6K53R	0.488
Aradu.AR630	0.384	642	48.834	37.636	38.569	Araip.W3RN5	0.383
Aradu.AR7XM	0.394	1193	50.084	38.61	35.343	Araip.H663M	0.389
Aradu.AR9AM	0.375	341	52.339	40.936	33.041	Araip.FV4T0	0.381
Aradu.ARZ2Q	0.327	698	46.209	36.195	35.336	Araip.U647S	0.337
Aradu.AS0KA	0.348	765	51.436	34.073	48.172	Araip.T94IA	0.35
Aradu.AS44H	0.379	1103	52.899	35.326	37.047	Araip.8K50I	0.379
Aradu.ASE53	0.372	621	49.518	38.264	35.37	Araip.QD22A	0.37
Aradu.AT0C1	0.381	1102	50.227	42.43	40.888	Araip.G1J68	0.377
Aradu.AT3ZW	0.373	1002	62.313	50.15	34.197	Araip.3FA1A	0.37
Aradu.AT44H	0.382	371	48.925	49.462	43.28	Araip.D1N24	0.393
Aradu.AT7BU	0.506	344	48.696	42.609	63.768	Araip.6S3M7	0.497
Aradu.AT974	0.372	260	48.659	42.146	41.762	Araip.24TKU	0.344
Aradu.ATH33	0.401	625	53.674	39.617	38.498	Araip.TX4H4	0.393
Aradu.ATP30	0.357	487	50	37.5	41.189	Araip.AVK7Q	0.353
Aradu.ATW70	0.405	253	48.819	29.921	44.882	Araip.K02WH	0.405
Aradu.AU181	0.392	464	48.602	34.409	38.925	Araip.TW0D4	0.382
Aradu.AU35U	0.365	482	52.174	33.54	33.954	Araip.FB5WA	0.357
Aradu.AU4WK	0.39	661	47.583	40.03	41.541	Araip.N8F74	0.398
Aradu.AU74Y	0.483	645	49.845	41.331	62.539	Araip.XS9S4	0.497
Aradu.AU7A2	0.365	459	59.565	46.957	38.696	Araip.JVJ09	0.362
Aradu.AU8EP	0.356	495	46.774	31.653	39.919	Araip.QT6UY	0.386
Aradu.AUX6R	0.39	229	48.261	37.826	49.13	Araip.666Q3	0.378
Aradu.AV6RW	0.393	478	53.653	49.269	32.15	Araip.E51S4	0.393
Aradu.AVD8P	0.417	991	54.839	43.145	39.415	Araip.3J27X	0.416
Aradu.AVN2W	0.388	850	53.349	38.19	39.013	Araip.0214S	0.391
Aradu.AW1PY	0.346	372	47.453	39.41	37.265	Araip.CW8B2	0.369
Aradu.AW21H	0.377	608	52.874	47.947	35.468	Araip.NCA4A	0.374
Aradu.AW46C	0.356	283	47.535	29.225	48.239	Araip.I3PYA	0.362
Aradu.AW9HW	0.392	797	51.629	46.241	32.08	Araip.16L7J	0.387
Aradu.AX3Z0	0.346	839	45	41.31	36.905	Araip.97MHJ	0.345
Aradu.AX4BJ	0.515	168	51.479	36.095	55.621	Araip.Y1B33	0.49
Aradu.AX5BM	0.344	613	50.163	42.997	35.016	Araip.U63G1	0.339
Aradu.AX7JB	0.321	169	42.353	36.471	52.353	Araip.A64V4	0.321
Aradu.AXB4D	0.348	648	48.536	37.904	39.291	Araip.RRZ3Z	0.339
Aradu.AXH5G	0.425	479	48.75	52.708	34.583	Araip.JB1PE	0.421
Aradu.AXU8L	0.446	260	52.107	39.08	43.678	Araip.14RNX	0.45
Aradu.AY7EP	0.55	164	56.364	45.455	66.061	Araip.8JP1D	0.544
Aradu.AY7XX	0.376	177	51.685	51.124	46.067	Araip.TK75I	0.395
Aradu.AZ3DS	0.423	454	50.11	38.681	59.121	Araip.W9EY0	0.423
Aradu.AZ535	0.442	115	52.586	35.345	29.31	Araip.5836D	0.466
Aradu.AZC7U	0.588	123	52.419	38.71	79.839	Araip.QR2AA	0.569

Aradu.AZF5J	0.378	293	45.918	35.034	43.197	Araip.SZQ74	0.381
Aradu.B03MY	0.368	554	50.27	40.541	36.396	Araip.6P9G9	0.389
Aradu.B049P	0.381	586	44.804	38.501	44.293	Araip.XI8EQ	0.384
Aradu.B04QQ	0.35	289	43.793	35.172	33.448	Araip.Q8Y00	0.343
Aradu.B0BA3	0.32	453	46.696	40.529	37.004	Araip.7P6A7	0.331
Aradu.B0BG6	0.396	369	50.811	41.622	52.703	Araip.4UX45	0.391
Aradu.B0MFP	0.37	494	52.121	44.242	37.576	Araip.S10I1	0.372
Aradu.B0NST	0.369	511	52.734	42.383	42.969	Araip.G5X76	0.369
Aradu.B0Q1E	0.439	727	54.533	42.995	51.374	Araip.6GF41	0.433
Aradu.B0TIL	0.437	124	58.4	32.8	56.8	Araip.M9QUH	0.445
Aradu.B11ZS	0.375	492	48.682	38.54	37.525	Araip.W23GC	0.364
Aradu.B14QA	0.331	188	56.614	43.386	33.862	Araip.NJ3UQ	0.343
Aradu.B151U	0.467	279	60	42.857	49.643	Araip.L60S0	0.471
Aradu.B18E9	0.394	389	47.949	41.795	38.974	Araip.WVS7I	0.407
Aradu.B1C6F	0.395	156	38.217	34.395	43.949	Araip.D0ST6	0.401
Aradu.B1CPC	0.389	453	53.965	46.035	38.326	Araip.Y5JHT	0.393
Aradu.B1CQK	0.4	1088	52.709	43.434	36.731	Araip.JKM82	0.391
Aradu.B1FJV	0.472	210	45.972	54.028	42.18	Araip.DV8IK	0.475
Aradu.B1NGL	0.456	388	50.129	40.103	44.216	Araip.Q61XZ	0.443
Aradu.B1W2E	0.35	386	48.579	40.827	39.276	Araip.DRF2S	0.37
Aradu.B1ZZT	0.362	235	49.153	40.678	44.068	Araip.ZW85N	0.367
Aradu.B219P	0.346	226	56.388	36.123	30.396	Araip.K6VBU	0.341
Aradu.B21Z9	0.386	138	43.165	30.216	51.079	Araip.G25FJ	0.386
Aradu.B240I	0.383	411	56.796	36.65	38.107	Araip.T2CTI	0.377
Aradu.B24NU	0.395	544	54.312	42.569	40.55	Araip.69PE3	0.391
Aradu.B26BB	0.334	480	45.53	38.254	39.501	Araip.P0KMD	0.317
Aradu.B2A4G	0.391	282	57.244	41.343	39.929	Araip.T7DB7	0.393
Aradu.B2BUH	0.341	453	51.322	49.339	40.088	Araip.J67VV	0.356
Aradu.B2STS	0.465	152	42.484	45.752	56.863	Araip.TDZ3S	0.458
Aradu.B2YUE	0.307	653	49.847	40.061	39.144	Araip.IZW7J	0.306
Aradu.B2Z42	0.506	324	53.846	49.231	64.615	Araip.4KB77	0.492
Aradu.B33TG	0.356	337	50.888	51.183	30.769	Araip.8S5BI	0.359
Aradu.B34AL	0.385	186	52.406	36.364	49.198	Araip.D33IT	0.374
Aradu.B353U	0.422	259	50.769	44.231	42.308	Araip.N6ZTJ	0.435
Aradu.B3611	0.377	260	45.211	41.762	43.295	Araip.GQE2Q	0.373
Aradu.B3CRQ	0.368	302	53.135	38.284	46.205	Araip.THJ6R	0.363
Aradu.B3D9E	0.397	835	54.904	38.636	39.833	Araip.VJ6YA	0.394
Aradu.B3QR0	0.341	424	48.471	38.588	36.235	Araip.S1TFK	0.346
Aradu.B3U8E	0.389	588	53.141	38.54	36.163	Araip.TY86Z	0.384
Aradu.B3V05	0.317	397	41.96	49.246	36.432	Araip.J98CT	0.329
Aradu.B3V7U	0.358	630	52.139	40.095	32.964	Araip.V7D04	0.361
Aradu.B3VS2	0.377	153	52.597	42.208	40.26	Araip.K23QX	0.39
Aradu.B3WTX	0.381	917	54.248	39.325	35.621	Araip.5VE9R	0.381
Aradu.B3YY0	0.397	380	54.856	42.782	32.546	Araip.R3LNH	0.38

Aradu.B3Z15	0.38	522	45.889	37.094	41.874	Araip.6N7BK	0.39
Aradu.B40DX	0.422	320	50.779	39.564	44.237	Araip.2GB7B	0.404
Aradu.B45K6	0.398	428	51.515	44.289	43.357	Araip.QRQ13	0.4
Aradu.B4AI9	0.383	536	50.279	39.479	39.292	Araip.V50JD	0.38
Aradu.B4CLV	0.443	213	50	35.981	41.121	Araip.PE5QA	0.453
Aradu.B4D1B	0.49	201	49.505	38.614	46.04	Araip.TWH1T	0.497
Aradu.B4FEF	0.371	440	46.259	46.259	38.095	Araip.CW7EC	0.362
Aradu.B4GBB	0.531	151	51.316	51.974	36.184	Araip.S2EYP	0.517
Aradu.B4HJ4	0.308	138	41.007	44.604	32.374	Araip.ALT9X	0.325
Aradu.B4I2A	0.405	867	56.336	40.899	47.811	Araip.GEE0K	0.403
Aradu.B4N91	0.391	820	52.984	36.784	39.951	Araip.VV015	0.395
Aradu.B4P3F	0.375	495	48.79	36.492	40.524	Araip.VH6RA	0.373
Aradu.B4UNP	0.35	480	53.846	38.046	46.57	Araip.88TVT	0.352
Aradu.B4US9	0.357	648	48.228	37.134	35.901	Araip.7H0TY	0.351
Aradu.B4Y17	0.406	787	47.97	36.929	48.096	Araip.2RW57	0.417
Aradu.B4YLF	0.387	676	50.074	43.427	38.552	Araip.7P4U9	0.39
Aradu.B4Z82	0.378	260	52.874	51.724	41.379	Araip.GZ59D	0.382
Aradu.B5DUD	0.452	193	49.485	48.969	52.577	Araip.9N4E5	0.438
Aradu.B5E0M	0.384	431	51.852	46.528	38.426	Araip.N4R7Y	0.39
Aradu.B5JI0	0.427	459	47.609	36.522	38.043	Araip.TK8D6	0.422
Aradu.B5ML3	0.379	925	47.948	32.721	42.549	Araip.IBP0A	0.379
Aradu.B5WG1	0.401	316	58.044	38.801	34.7	Araip.52XKK	0.389
Aradu.B6CZV	0.293	387	44.845	34.794	32.732	Araip.Z8DH4	0.284
Aradu.B6G6H	0.406	865	49.423	36.49	34.988	Araip.9E07Y	0.414
Aradu.B6T0L	0.349	775	53.608	36.34	38.66	Araip.3H4X4	0.355
Aradu.B6TJT	0.379	464	53.763	43.226	38.28	Araip.J0VZ0	0.386
Aradu.B6TUW	0.348	473	46.624	37.975	38.186	Araip.HU0D3	0.359
Aradu.B71RC	0.39	773	49.225	45.607	46.253	Araip.CYK8B	0.393
Aradu.B7233	0.382	1086	52.346	39.742	41.122	Araip.G8H3T	0.374
Aradu.B73B3	0.397	246	50.607	42.105	38.462	Araip.M16D8	0.4
Aradu.B7486	0.358	1931	50	39.959	33.23	Araip.EU8TM	0.36
Aradu.B74MT	0.435	723	52.348	34.53	43.646	Araip.B2Q36	0.432
Aradu.B78C7	0.436	237	55.042	39.496	28.992	Araip.V6D67	0.43
Aradu.B78K2	0.37	405	47.783	40.394	38.424	Araip.GQ95T	0.378
Aradu.B7FB5	0.395	317	50	45.283	45.912	Araip.GXP3H	0.395
Aradu.B7H15	0.336	544	43.119	32.11	36.697	Araip.Q37BC	0.303
Aradu.B7P36	0.45	418	54.654	39.379	38.902	Araip.M1IU9	0.443
Aradu.B7QXE	0.396	623	57.051	50.321	42.308	Araip.9P3RH	0.394
Aradu.B7RDX	0.343	256	45.525	34.241	49.805	Araip.LGM59	0.336
Aradu.B7XG0	0.465	451	55.531	44.69	56.858	Araip.95KUY	0.463
Aradu.B7Y20	0.387	391	45.153	36.735	48.724	Araip.1W5SS	0.39
Aradu.B80V6	0.416	381	51.832	42.932	40.838	Araip.V6UVY	0.418
Aradu.B80WC	0.365	2612	52.354	41.676	34.941	Araip.WS6S0	0.362
Aradu.B81RW	0.394	252	48.221	43.478	43.083	Araip.N7CQ0	0.391

Aradu.B83GZ	0.397	451	48.451	41.593	46.903	Araip.Q6R0G	0.404
Aradu.B8ABT	0.386	901	51.996	35.255	38.137	Araip.TVG17	0.393
Aradu.B8HUR	0.366	760	51.774	36.925	37.714	Araip.RB3EK	0.366
Aradu.B8U65	0.332	538	49.536	40.074	35.25	Araip.BJ25J	0.321
Aradu.B8V9A	0.338	744	46.04	38.255	40.268	Araip.21REB	0.334
Aradu.B90GQ	0.478	149	51.333	44	52.667	Araip.T3D3V	0.451
Aradu.B925F	0.405	166	37.725	28.144	40.12	Araip.BG0H4	0.345
Aradu.B92XB	0.333	279	48.214	34.286	38.571	Araip.R6YM0	0.341
Aradu.B98C6	0.436	624	49.6	53.6	44.8	Araip.LC8CF	0.438
Aradu.B99AB	0.354	968	43.55	35.294	39.216	Araip.FJB0B	0.35
Aradu.B9JN3	0.34	686	47.307	42.795	38.719	Araip.2Q9MF	0.355
Aradu.B9T25	0.38	779	53.974	42.692	32.179	Araip.9E5ZT	0.383
Aradu.B9U3D	0.351	296	45.118	42.424	43.434	Araip.2Q11V	0.353
Aradu.B9ZHB	0.401	428	52.448	39.161	44.988	Araip.E4D1J	0.406
Aradu.BA406	0.362	491	48.171	40.041	35.976	Araip.R2L10	0.36
Aradu.BA701	0.49	421	50.711	45.972	63.27	Araip.GP92E	0.508
Aradu.BAA8F	0.388	430	55.22	38.979	50.116	Araip.S12BL	0.386
Aradu.BAW60	0.457	471	53.39	39.195	65.678	Araip.UF6J5	0.457
Aradu.BB0Q9	0.367	927	49.569	35.129	37.177	Araip.E3LGC	0.374
Aradu.BB4JP	0.387	369	50.541	49.459	37.297	Araip.4L5AH	0.378
Aradu.BB6TM	0.352	312	56.869	37.7	53.994	Araip.EPL8Z	0.349
Aradu.BB920	0.412	596	48.911	38.526	46.231	Araip.76E63	0.417
Aradu.BB9NS	0.354	522	45.889	34.799	38.432	Araip.IA5QC	0.356
Aradu.BBF6Q	0.41	257	60.853	35.659	50	Araip.8LP4W	0.414
Aradu.BBP4Z	0.41	500	50.1	37.525	49.301	Araip.00P1B	0.388
Aradu.BBW0R	0.374	280	44.484	35.587	34.52	Araip.LQ7SX	0.338
Aradu.BC220	0.363	759	53.026	42.895	34.342	Araip.1N4XS	0.365
Aradu.BC6IG	0.386	609	52.131	44.098	41.967	Araip.I1K2T	0.4
Aradu.BCZ93	0.504	126	43.307	35.433	59.843	Araip.0W448	0.493
Aradu.BD2SQ	0.409	502	50.298	35.388	48.31	Araip.PT961	0.414
Aradu.BD9UN	0.347	976	56.704	41.146	37.155	Araip.JR03F	0.345
Aradu.BDH44	0.358	674	49.333	37.926	38.222	Araip.658MF	0.363
Aradu.BDN4J	0.334	619	47.258	34.677	38.871	Araip.3NM2I	0.334
Aradu.BE2IC	0.462	276	48.375	43.682	42.599	Araip.V2UYE	0.454
Aradu.BE5FD	0.42	317	47.17	41.509	38.05	Araip.3CP6N	0.432
Aradu.BE6XZ	0.46	271	53.309	41.912	70.588	Araip.H8VZN	0.475
Aradu.BE7PX	0.344	136	50.365	37.956	48.905	Araip.J5910	0.328
Aradu.BE884	0.332	193	51.031	37.113	35.052	Araip.6XF23	0.332
Aradu.BEW9Q	0.432	597	47.157	39.13	49.331	Araip.2T05W	0.428
Aradu.BF125	0.342	359	50	35.556	36.389	Araip.69K1T	0.335
Aradu.BF8QQ	0.343	698	45.351	36.624	38.484	Araip.Q47NS	0.34
Aradu.BF9MN	0.446	147	45.946	43.919	40.541	Araip.Q865J	0.446
Aradu.BFS1P	0.365	218	48.402	36.986	49.315	Araip.LBX9K	0.361
Aradu.BG2Q4	0.43	320	55.452	44.548	44.237	Araip.1P50V	0.436

Aradu.BG56S	0.349	513	46.304	43.58	49.805	Araip.AVA7B	0.354
Aradu.BGJ4E	0.425	476	52.621	42.977	53.459	Araip.YN1XP	0.384
Aradu.BGR5Z	0.46	130	38.931	39.695	61.069	Araip.U9QE5	0.444
Aradu.BH653	0.352	313	52.866	40.446	44.268	Araip.UFD3P	0.344
Aradu.BH9NG	0.434	436	53.318	32.037	38.215	Araip.Q6KXW	0.429
Aradu.BHH9A	0.364	1060	50.707	42.413	40.245	Araip.Y0HFI	0.362
Aradu.BHV10	0.449	211	58.962	51.415	55.66	Araip.ZDS2I	0.439
Aradu.BI3QN	0.371	506	44.379	33.728	35.306	Araip.SIS4H	0.362
Aradu.BI5JF	0.38	231	47.414	34.483	46.121	Araip.YQ2F8	0.384
Aradu.BIW4R	0.396	992	53.072	39.678	41.591	Araip.L9809	0.396
Aradu.BIZ8F	0.494	184	41.622	43.784	45.946	Araip.T3G5J	0.48
Aradu.BJ3DD	0.429	193	50	40.722	39.175	Araip.50ZEB	0.431
Aradu.BJ55N	0.364	436	57.437	45.08	34.554	Araip.S52ED	0.361
Aradu.BJL71	0.384	426	45.902	37.705	42.155	Araip.ER6JN	0.38
Aradu.BK33F	0.371	519	46.538	38.462	39.615	Araip.XIH72	0.365
Aradu.BK3J1	0.449	287	50.347	41.319	55.208	Araip.CL7TC	0.451
Aradu.BK9AL	0.31	401	47.512	34.328	30.1	Araip.0I7VH	0.335
Aradu.BL1S9	0.409	491	50.407	41.26	38.211	Araip.R0KMB	0.406
Aradu.BLA0I	0.378	789	54.304	46.329	48.734	Araip.VMH3J	0.383
Aradu.BLA9Y	0.401	383	54.688	41.146	32.292	Araip.VBT3F	0.399
Aradu.BLK03	0.348	439	44.091	35.455	29.773	Araip.XP1CY	0.345
Aradu.BLP4A	0.4	225	62.832	41.15	63.274	Araip.4U9WI	0.42
Aradu.BM2KZ	0.39	365	45.082	34.426	42.077	Araip.P8FYF	0.387
Aradu.BM2UV	0.385	846	52.066	46.989	38.371	Araip.VEC9V	0.387
Aradu.BM5FL	0.334	466	45.61	43.683	26.552	Araip.ZG9AN	0.326
Aradu.BM68M	0.389	376	43.501	40.318	39.523	Araip.LL92K	0.38
Aradu.BMJ8R	0.393	676	50.517	40.03	37.518	Araip.Z76YZ	0.395
Aradu.BNJ62	0.378	1829	54.208	40.874	35.738	Araip.JF5B7	0.369
Aradu.BNK96	0.405	203	53.922	37.745	65.686	Araip.RDA4Z	0.415
Aradu.BNM3L	0.398	122	43.089	44.715	45.528	Araip.GU31N	0.398
Aradu.BNM8A	0.414	365	49.727	36.612	49.454	Araip.Q4FLH	0.408
Aradu.BP0GU	0.426	321	50	45.031	54.658	Araip.T5DLA	0.424
Aradu.BP7BM	0.359	807	47.153	39.48	37.376	Araip.NNQ15	0.366
Aradu.BPK98	0.362	463	46.983	40.733	34.483	Araip.A6QWC	0.349
Aradu.BQ4IU	0.344	538	51.948	44.527	38.033	Araip.W3SNY	0.344
Aradu.BR0NQ	0.362	591	56.757	33.108	39.696	Araip.CXD1I	0.357
Aradu.BR4VK	0.408	380	44.619	45.407	44.882	Araip.KA397	0.398
Aradu.BR5RX	0.457	859	54.302	41.86	62.093	Araip.57L7Q	0.454
Aradu.BR65G	0.384	1312	50.038	42.422	38.233	Araip.A8VXL	0.386
Aradu.BR6GB	0.388	476	47.589	43.396	35.01	Araip.5FP0F	0.385
Aradu.BS31B	0.362	1034	47.246	36.812	39.42	Araip.T7RFD	0.359
Aradu.BS3D3	0.374	553	51.083	37.726	41.516	Araip.1IV9L	0.361
Aradu.BS3JU	0.397	300	49.502	36.877	39.867	Araip.J93FI	0.403
Aradu.BS3NC	0.346	367	49.457	34.239	43.75	Araip.19369	0.349

Aradu.BS70J	0.415	393	47.208	41.117	39.086	Araip.8X7QI	0.408
Aradu.BSR1D	0.35	1406	45.629	39.588	35.323	Araip.Q10LZ	0.357
Aradu.BT3SQ	0.367	520	52.015	35.893	35.125	Araip.4YS5X	0.369
Aradu.BTB7U	0.452	227	42.982	47.368	48.684	Araip.7N6M4	0.461
Aradu.BU1C4	0.386	548	47.905	44.991	41.53	Araip.RNH2A	0.394
Aradu.BU5Q7	0.396	209	51.905	46.667	39.048	Araip.21F2H	0.386
Aradu.BUA5R	0.355	555	48.741	42.266	32.014	Araip.V1VLH	0.358
Aradu.BUC40	0.307	195	55.612	40.306	33.163	Araip.X7VQF	0.307
Aradu.BUW4Z	0.31	317	41.509	39.623	33.962	Araip.P0TGF	0.3
Aradu.BV2Q9	0.433	109	53.636	51.818	41.818	Araip.CQ0DA	0.433
Aradu.BV2ZB	0.362	401	53.483	41.294	34.577	Araip.W61UJ	0.372
Aradu.BV4I8	0.373	586	53.322	33.049	39.693	Araip.FR6UM	0.365
Aradu.BV67Y	0.406	366	50.681	41.689	43.869	Araip.U15FR	0.403
Aradu.BV95P	0.399	415	48.798	50.721	37.26	Araip.U0W6U	0.407
Aradu.BV9FL	0.374	619	47.742	39.355	41.774	Araip.UR4F4	0.379
Aradu.BW310	0.516	129	42.308	41.538	47.692	Araip.G5FLL	0.516
Aradu.BWC86	0.361	392	46.565	35.623	43.257	Araip.WP8Q7	0.36
Aradu.BWE80	0.421	448	48.33	36.303	58.352	Araip.51HAH	0.414
Aradu.BWJ69	0.386	487	45.492	45.902	46.721	Araip.GI9KG	0.376
Aradu.BWM82	0.359	466	49.465	37.045	39.829	Araip.5MT98	0.352
Aradu.BX0IC	0.3	168	42.012	39.645	45.562	Araip.62GEG	0.294
Aradu.BX447	0.42	291	51.027	43.493	44.863	Araip.736QB	0.394
Aradu.BX9V6	0.438	811	53.695	40.764	49.754	Araip.8VU19	0.443
Aradu.BY27U	0.391	418	54.415	45.346	43.675	Araip.M8P59	0.381
Aradu.BY4US	0.39	1207	46.937	38.328	35.513	Araip.38QJB	0.397
Aradu.BY7GE	0.365	523	46.183	45.038	39.885	Araip.15RTX	0.359
Aradu.BYJ1W	0.331	1025	48.246	34.503	30.312	Araip.19H18	0.324
Aradu.BYZ1A	0.362	187	50.532	39.362	32.979	Araip.4G5WD	0.371
Aradu.BZA24	0.388	618	56.866	34.41	40.872	Araip.FZ182	0.38
Aradu.BZU3P	0.429	421	59.005	43.365	54.976	Araip.T09RD	0.45
Aradu.C00W8	0.419	272	48.718	34.066	46.886	Araip.HGE67	0.411
Aradu.C014T	0.416	155	42.949	54.487	48.718	Araip.JP66B	0.416
Aradu.C04VQ	0.39	315	48.734	44.62	47.152	Araip.F0MKW	0.401
Aradu.C08W6	0.358	308	53.074	46.926	36.57	Araip.T4ATQ	0.354
Aradu.C0B8A	0.404	459	49.783	42.174	47.826	Araip.67BIA	0.412
Aradu.C0E4H	0.387	230	48.485	35.498	47.619	Araip.J6KI9	0.382
Aradu.C0GKW	0.412	472	55.603	38.689	41.015	Araip.37ZE6	0.41
Aradu.C0GMC	0.355	1178	47.243	34.945	38.253	Araip.GD26H	0.356
Aradu.C0HL8	0.329	216	49.77	30.876	46.083	Araip.RA9BV	0.335
Aradu.C0NTR	0.433	454	45.275	45.275	45.055	Araip.2V326	0.424
Aradu.C0ZT2	0.389	403	49.505	43.812	42.079	Araip.DSQ1W	0.393
Aradu.C114V	0.405	409	47.561	36.829	49.756	Araip.R25AT	0.409
Aradu.C19JT	0.33	424	47.294	38.118	37.882	Araip.SH2LK	0.331
Aradu.C1C0K	0.38	307	45.455	35.065	21.753	Araip.I8L3V	0.38

Aradu.C1KJF	0.353	974	46.564	35.897	39.897	Araip.PS43L	0.357
Aradu.C1S3R	0.385	771	54.016	38.86	38.083	Araip.PCB4M	0.386
Aradu.C1UGC	0.431	346	49.856	28.818	40.058	Araip.Z68MJ	0.431
Aradu.C1X9T	0.393	640	48.83	43.682	44.618	Araip.B52YR	0.39
Aradu.C1ZP7	0.385	139	46.429	34.286	52.857	Araip.BW6MW	0.364
Aradu.C2BM8	0.383	249	41.6	40	40.8	Araip.W0JLG	0.399
Aradu.C2F9N	0.382	156	51.592	56.051	56.688	Araip.YX3SF	0.424
Aradu.C2HZQ	0.41	568	52.724	42.179	44.815	Araip.568TN	0.414
Aradu.C2N0T	0.349	390	46.547	43.734	34.015	Araip.0G24M	0.341
Aradu.C2PQ3	0.395	273	48.54	33.942	44.526	Araip.HEL12	0.377
Aradu.C2VRV	0.365	556	52.244	53.501	36.804	Araip.Y1811	0.369
Aradu.C2W69	0.407	341	44.737	37.719	46.199	Araip.ITE41	0.4
Aradu.C31L4	0.386	329	49.394	43.939	43.333	Araip.DG2YH	0.389
Aradu.C37MP	0.408	297	57.047	44.295	40.268	Araip.TL0ID	0.401
Aradu.C3ARN	0.359	119	51.667	31.667	33.333	Araip.P15N1	0.368
Aradu.C3FIF	0.455	179	54.444	46.667	61.111	Araip.028PN	0.429
Aradu.C3IHK	0.445	172	43.931	41.04	49.133	Araip.4S7ZN	0.436
Aradu.C3QXP	0.365	822	49.453	38.275	34.386	Araip.38J51	0.366
Aradu.C3YLC	0.426	230	51.948	41.991	45.022	Araip.D2SBD	0.435
Aradu.C42LD	0.396	1175	50.255	39.796	36.48	Araip.VN7CM	0.389
Aradu.C4BMG	0.398	386	51.421	43.928	43.152	Araip.08H2V	0.39
Aradu.C4EEM	0.412	806	56.258	39.281	42.007	Araip.G5KNS	0.407
Aradu.C4HNC	0.363	142	59.441	37.762	45.455	Araip.JV5C1	0.363
Aradu.C4J7U	0.37	300	47.508	34.551	47.176	Araip.C83J2	0.369
Aradu.C4M03	0.319	725	53.444	47.383	33.471	Araip.0448C	0.309
Aradu.C4MHC	0.392	238	50.209	37.238	35.983	Araip.3HN76	0.399
Aradu.C4WL2	0.362	254	43.529	37.647	33.333	Araip.KP2HT	0.35
Aradu.C4XJQ	0.36	249	46.4	36.4	32.8	Araip.R44YW	0.353
Aradu.C4Y83	0.374	631	50.791	46.044	32.437	Araip.VNV0X	0.381
Aradu.C51SS	0.397	402	52.605	43.176	41.439	Araip.I3EE6	0.406
Aradu.C5AKB	0.363	801	49.501	35.91	35.91	Araip.S501C	0.387
Aradu.C5CLY	0.391	1143	49.738	40.035	34.178	Araip.7F1V2	0.397
Aradu.C5DXV	0.423	394	53.418	46.582	47.595	Araip.P4F2X	0.428
Aradu.C5KI2	0.385	395	43.434	43.182	33.586	Araip.XD91V	0.395
Aradu.C5UHL	0.338	1355	50.885	38.201	36.947	Araip.Y8SXT	0.335
Aradu.C5W7K	0.342	241	45.041	37.603	40.496	Araip.246YY	0.328
Aradu.C61GI	0.398	472	51.374	33.404	45.666	Araip.YLJ2W	0.399
Aradu.C64A0	0.356	1066	44.611	36.832	37.488	Araip.BHZ61	0.357
Aradu.C65VQ	0.431	186	54.011	42.781	33.155	Araip.JY29C	0.436
Aradu.C665I	0.374	456	49.672	36.324	42.451	Araip.4J570	0.392
Aradu.C674J	0.406	412	54.722	43.584	53.995	Araip.N2MW3	0.399
Aradu.C6EHZ	0.429	670	60.656	41.878	63.636	Araip.7G9YB	0.429
Aradu.C6I2X	0.379	247	62.903	45.565	33.871	Araip.RWR8N	0.387
Aradu.C6ILJ	0.37	646	55.023	42.504	35.394	Araip.AJZ7U	0.367

Aradu.C6P70	0.434	263	49.621	44.697	43.561	Araip.HK5CX	0.468
Aradu.C6S8Z	0.323	240	61.411	33.195	36.515	Araip.702H5	0.332
Aradu.C6SJS	0.372	141	52.113	33.099	30.986	Araip.DP7XY	0.358
Aradu.C6W6J	0.478	163	57.927	50.61	70.732	Araip.YJ4K8	0.5
Aradu.C716W	0.333	476	54.507	39.203	44.235	Araip.X0N4G	0.348
Aradu.C718S	0.384	1252	50.918	30.886	37.749	Araip.G2LIW	0.386
Aradu.C73IQ	0.394	584	57.607	42.051	45.299	Araip.J0WZR	0.398
Aradu.C7AWI	0.372	1323	46.677	37.085	37.311	Araip.7A4AR	0.388
Aradu.C7AZ0	0.448	346	56.196	47.262	53.314	Araip.L46F0	0.444
Aradu.C7C1R	0.404	406	52.826	37.101	44.717	Araip.WW83V	0.396
Aradu.C7CT2	0.394	136	43.796	42.336	45.985	Araip.LWA5C	0.364
Aradu.C7JM0	0.417	342	52.187	37.318	37.318	Araip.YB9GG	0.413
Aradu.C7JRZ	0.357	254	47.843	26.667	40.784	Araip.194YI	0.373
Aradu.C7NWK	0.451	151	55.263	42.763	35.526	Araip.N40QT	0.444
Aradu.C7P1W	0.372	229	51.304	34.348	45.217	Araip.V76V7	0.368
Aradu.C7QG4	0.391	166	46.108	45.509	40.719	Araip.ZZ77A	0.43
Aradu.C7X7Y	0.376	285	48.951	36.014	41.958	Araip.QT81S	0.367
Aradu.C824H	0.389	463	51.724	35.345	40.733	Araip.B68IR	0.388
Aradu.C87QT	0.46	584	58.12	45.641	62.564	Araip.GDW3P	0.465
Aradu.C881Z	0.376	296	49.832	37.71	30.976	Araip.UBP04	0.359
Aradu.C89XH	0.341	425	52.113	35.211	45.305	Araip.BT4VK	0.342
Aradu.C8CZA	0.38	1009	53.564	37.129	39.307	Araip.H639W	0.369
Aradu.C8KKZ	0.333	848	48.057	36.514	35.453	Araip.C9LX5	0.337
Aradu.C8KRG	0.338	138	47.482	47.482	40.288	Araip.PN8UI	0.346
Aradu.C8Q2A	0.334	360	52.909	41.551	53.74	Araip.C66NM	0.337
Aradu.C8SIT	0.407	278	55.556	36.201	46.237	Araip.QP82L	0.415
Aradu.C8SSW	0.337	317	42.138	29.245	26.101	Araip.PKK04	0.345
Aradu.C8ZMR	0.343	184	40.541	37.838	42.162	Araip.NX7E8	0.347
Aradu.C90G3	0.498	323	54.321	52.778	56.173	Araip.WEZ1Z	0.508
Aradu.C924Y	0.476	110	47.748	50.45	54.955	Araip.L5XNA	0.476
Aradu.C96YL	0.399	309	47.419	39.032	42.581	Araip.P89N6	0.411
Aradu.C99J0	0.406	598	48.08	42.237	39.733	Araip.U1IEA	0.398
Aradu.C9I9A	0.351	568	48.33	26.011	41.125	Araip.FI9WY	0.361
Aradu.C9LG8	0.374	324	54.154	30.769	45.231	Araip.WRD18	0.372
Aradu.C9WAZ	0.507	137	59.42	40.58	62.319	Araip.2QM99	0.507
Aradu.C9WV0	0.336	787	54.949	50	29.315	Araip.1M7FH	0.334
Aradu.CA5TC	0.388	311	50.321	41.987	37.821	Araip.X2AP6	0.388
Aradu.CA6PJ	0.406	472	57.717	41.226	50.74	Araip.6L1FE	0.397
Aradu.CA6YX	0.4	433	56.912	41.014	55.991	Araip.RUD0N	0.398
Aradu.CAH9K	0.377	365	54.098	37.158	40.164	Araip.82QS5	0.368
Aradu.CAM8W	0.438	462	45.356	42.333	49.676	Araip.S0EN6	0.443
Aradu.CB26G	0.455	585	51.195	36.177	60.751	Araip.AR2VK	0.468
Aradu.CBB8Q	0.427	541	45.756	37.454	51.292	Araip.D3NXV	0.432
Aradu.CBT6M	0.33	549	37.455	37.455	38.182	Araip.A8G61	0.335

Aradu.CBY05	0.403	424	52.471	45.176	51.294	Araip.WNQ27	0.402
Aradu.CBZ5I	0.38	423	51.415	35.849	41.745	Araip.T2RIK	0.373
Aradu.CC1R3	0.325	1007	45.933	38.889	37.996	Araip.3K2R6	0.322
Aradu.CC8V7	0.387	581	56.701	39.003	40.378	Araip.SWA4I	0.381
Aradu.CC9MM	0.369	950	48.265	37.645	36.698	Araip.QL2Q5	0.368
Aradu.CCC04	0.417	308	43.689	46.278	37.864	Araip.T2765	0.41
Aradu.CCT0A	0.405	474	51.579	41.474	38.737	Araip.3E86P	0.415
Aradu.CD22H	0.386	389	43.59	38.205	43.333	Araip.CGA2X	0.407
Aradu.CD2E5	0.398	607	49.836	37.829	44.737	Araip.TJC4C	0.387
Aradu.CD4CM	0.413	583	47.089	34.932	41.267	Araip.N65BG	0.407
Aradu.CD8IH	0.368	1142	53.018	43.132	40.157	Araip.MC99C	0.367
Aradu.CD8RE	0.316	389	48.205	40.256	32.051	Araip.JIQ05	0.299
Aradu.CDA12	0.394	408	52.323	38.875	36.43	Araip.4K162	0.352
Aradu.CDI4M	0.353	278	44.086	40.502	36.201	Araip.N4R5C	0.362
Aradu.CDJ2J	0.409	536	52.886	36.872	40.41	Araip.842TG	0.407
Aradu.CDK6C	0.266	112	48.673	23.009	30.088	Araip.3RS0Y	0.272
Aradu.CDX53	0.385	595	53.188	37.919	45.134	Araip.KU4D3	0.37
Aradu.CE0RB	0.481	273	58.029	46.715	58.394	Araip.G4EHF	0.481
Aradu.CED81	0.353	812	45.264	39.975	38.376	Araip.QR111	0.359
Aradu.CF5Z2	0.389	365	44.536	31.967	43.989	Araip.K3GN3	0.385
Aradu.CG16W	0.381	873	48.398	39.245	40.618	Araip.JYZ4H	0.384
Aradu.CG1AI	0.362	475	44.958	41.176	42.857	Araip.WJJ4Z	0.351
Aradu.CG33N	0.322	418	49.165	40.811	31.504	Araip.BS911	0.319
Aradu.CG4KR	0.356	290	56.357	50.859	36.426	Araip.4I8QR	0.337
Aradu.CG65D	0.367	364	52.329	33.151	43.562	Araip.9I2Z5	0.356
Aradu.CG9KF	0.39	260	50.958	48.659	47.126	Araip.LCN5D	0.418
Aradu.CGD8Q	0.396	302	50.825	52.145	42.244	Araip.W5573	0.388
Aradu.CGX1Z	0.369	371	52.957	36.559	35.753	Araip.32FLV	0.358
Aradu.CH4M9	0.327	209	48.095	35.714	47.143	Araip.842WX	0.332
Aradu.CH837	0.445	243	43.852	43.852	48.361	Araip.73ZM2	0.441
Aradu.CH84G	0.365	817	47.922	40.22	43.276	Araip.55F8V	0.363
Aradu.CHD0U	0.374	855	51.869	39.136	42.173	Araip.UK5PE	0.37
Aradu.CI09B	0.451	231	54.741	55.603	62.069	Araip.VC2I3	0.469
Aradu.CI3I2	0.339	380	44.357	40.157	40.157	Araip.JPZ0F	0.344
Aradu.CI3JS	0.487	233	49.573	47.863	74.359	Araip.RSA74	0.496
Aradu.CI6AA	0.409	122	52.033	50.407	60.163	Araip.BU32W	0.426
Aradu.CI747	0.388	536	55.307	43.203	33.52	Araip.2M3GS	0.385
Aradu.CI9FK	0.392	100	46.535	39.604	33.663	Araip.BJ5NT	0.381
Aradu.CJ510	0.371	430	48.956	43.387	42.459	Araip.YDF63	0.37
Aradu.CJN5E	0.378	679	56.324	36.912	42.647	Araip.GVJ3M	0.378
Aradu.CJN7V	0.39	567	51.056	33.979	44.19	Araip.488BQ	0.386
Aradu.CJP5L	0.4	824	55.515	43.273	41.576	Araip.1842Y	0.39
Aradu.CJQ5M	0.431	336	52.226	34.421	58.457	Araip.6M87Y	0.431
Aradu.CJQ6L	0.396	343	51.744	37.791	42.733	Araip.B90DI	0.399

Aradu.CJT43	0.397	125	57.937	54.762	61.111	Araip.ZH07M	0.397
Aradu.CK902	0.421	227	42.544	42.544	46.93	Araip.80FVV	0.423
Aradu.CKD1V	0.461	326	52.905	44.954	40.367	Araip.Q4YAV	0.474
Aradu.CKU4P	0.354	407	45.098	36.765	42.892	Araip.ZS4AK	0.35
Aradu.CKV49	0.408	475	53.151	41.176	48.109	Araip.W6JMB	0.398
Aradu.CL5YM	0.399	445	51.57	41.256	37.22	Araip.DW3Y4	0.399
Aradu.CLE55	0.381	414	46.024	41.687	35.904	Araip.P8NLK	0.366
Aradu.CLY5P	0.386	310	57.235	44.695	43.73	Araip.SQ4D9	0.392
Aradu.CM6S6	0.354	220	40.271	48.416	33.032	Araip.P53WL	0.352
Aradu.CM89H	0.385	497	52.008	38.353	39.96	Araip.A805A	0.376
Aradu.CMH1Z	0.373	977	48.16	33.129	39.059	Araip.5G3Z8	0.376
Aradu.CMI46	0.363	330	50.755	38.066	38.369	Araip.VD7Y0	0.351
Aradu.CML1P	0.343	674	51.852	42.519	34.667	Araip.VG726	0.333
Aradu.CMZ2S	0.386	734	54.966	46.531	38.639	Araip.333ZF	0.399
Aradu.CMZ60	0.345	318	47.022	45.455	42.006	Araip.W8NVJ	0.34
Aradu.CN01H	0.393	1156	50.389	38.894	40.882	Araip.U4XHQ	0.398
Aradu.CN1WR	0.358	700	49.501	36.377	35.949	Araip.E8UKB	0.357
Aradu.CN7V5	0.329	1020	40.842	35.553	35.162	Araip.RVF4P	0.322
Aradu.CNT80	0.351	114	44.348	39.13	34.783	Araip.F11A4	0.345
Aradu.CP1HR	0.37	222	43.049	44.843	38.565	Araip.I6HDR	0.354
Aradu.CP5FF	0.418	304	51.148	42.295	35.41	Araip.NLK3G	0.414
Aradu.CP61L	0.364	340	50.147	39.883	47.214	Araip.LP8FF	0.362
Aradu.CP7C1	0.372	564	47.08	33.982	37.876	Araip.L90LE	0.376
Aradu.CP8NA	0.424	307	49.675	46.429	41.558	Araip.ZSP2K	0.418
Aradu.CPF02	0.326	333	45.21	38.922	34.431	Araip.WN5LY	0.322
Aradu.CPH08	0.385	749	54.133	42.533	36.4	Araip.7RJ08	0.397
Aradu.CQ0KB	0.376	442	51.016	41.986	40.181	Araip.GWH2N	0.384
Aradu.CQ2HY	0.352	817	44.621	40.098	43.154	Araip.57MS8	0.358
Aradu.CQ85M	0.387	605	45.215	35.644	36.799	Araip.840F4	0.401
Aradu.CQN7Q	0.385	554	47.568	43.423	42.703	Araip.BE5FQ	0.375
Aradu.CR12A	0.36	511	49.805	37.305	42.578	Araip.ID0PF	0.368
Aradu.CR9NG	0.354	1113	46.409	38.869	38.869	Araip.CF1GV	0.357
Aradu.CRB6R	0.366	193	54.639	49.485	37.629	Araip.T3NCH	0.362
Aradu.CS1KJ	0.404	268	51.301	51.673	53.16	Araip.C0N7L	0.404
Aradu.CS27R	0.386	276	54.152	45.487	37.545	Araip.2GH89	0.399
Aradu.CSH6U	0.43	132	54.887	33.835	63.91	Araip.3Y5DK	0.445
Aradu.CSN5D	0.386	304	46.23	37.049	39.672	Araip.T1FEI	0.391
Aradu.CST5R	0.43	534	55.514	47.29	44.86	Araip.MAR0Q	0.42
Aradu.CT0FI	0.452	690	48.48	39.797	57.453	Araip.LD5UC	0.455
Aradu.CT2LH	0.489	188	50.794	35.45	39.153	Araip.1H1H0	0.466
Aradu.CT448	0.32	316	41.64	34.069	36.278	Araip.VH6HT	0.314
Aradu.CT5EJ	0.476	386	54.522	51.163	44.961	Araip.K90VM	0.481
Aradu.CTE87	0.398	507	48.622	42.52	34.449	Araip.VES4J	0.398
Aradu.CTQ6E	0.381	743	47.984	35.887	35.215	Araip.G18BD	0.377

Aradu.CU20J	0.419	374	48.267	42.933	50.4	Araip.K6B0A	0.421
Aradu.CU60X	0.388	118	52.101	35.294	43.697	Araip.QF5PU	0.391
Aradu.CU9HL	0.381	624	50.56	39.2	40.16	Araip.SP6K4	0.384
Aradu.CUQ8J	0.447	305	48.039	45.098	56.536	Araip.F0LWE	0.446
Aradu.CV157	0.32	271	47.794	47.794	32.353	Araip.8LE7X	0.332
Aradu.CV497	0.395	458	48.366	42.702	40.741	Araip.A7H6K	0.388
Aradu.CV6FA	0.39	560	48.485	37.611	39.929	Araip.F787E	0.391
Aradu.CV6Z5	0.413	407	47.304	32.843	45.098	Araip.P0NWF	0.409
Aradu.CV82P	0.387	320	51.402	32.087	52.025	Araip.VW0QI	0.37
Aradu.CVS4L	0.359	365	54.372	44.536	37.158	Araip.CJ248	0.367
Aradu.CVT5N	0.36	170	46.784	45.029	35.673	Araip.Q1RER	0.387
Aradu.CW391	0.397	362	41.047	40.496	41.598	Araip.HCR6S	0.419
Aradu.CW3NA	0.306	249	47.6	36	28	Araip.U94IX	0.296
Aradu.CW450	0.401	478	51.357	37.578	40.292	Araip.20CWB	0.406
Aradu.CW4J0	0.4	504	54.653	43.168	28.119	Araip.X395U	0.4
Aradu.CW6KA	0.434	106	57.944	49.533	52.336	Araip.GG51M	0.424
Aradu.CWG39	0.327	284	47.018	39.298	40.351	Araip.GHT9B	0.33
Aradu.CWT7S	0.418	764	54.771	36.993	56.863	Araip.TJ48Q	0.416
Aradu.CX05K	0.334	571	51.224	46.154	41.084	Araip.LT30A	0.33
Aradu.CX1PI	0.419	378	43.272	40.106	40.106	Araip.D1J6I	0.416
Aradu.CX437	0.439	442	55.079	44.695	54.402	Araip.B0EXC	0.439
Aradu.CX8YW	0.357	293	46.599	40.476	44.218	Araip.J8RHM	0.38
Aradu.CX99Z	0.383	498	48.697	38.477	43.687	Araip.NHN1D	0.381
Aradu.CXC2W	0.341	537	42.193	35.688	39.219	Araip.80D4Q	0.34
Aradu.CXJ5P	0.365	384	51.169	39.221	38.701	Araip.4N7WF	0.363
Aradu.CYF2K	0.38	808	51.916	44.994	35.352	Araip.GVS2Q	0.376
Aradu.CYS3J	0.37	508	54.813	40.275	39.882	Araip.C00SG	0.381
Aradu.CZ128	0.394	342	51.895	40.816	50.146	Araip.YC2CD	0.389
Aradu.CZ3TW	0.347	235	43.22	38.559	44.492	Araip.4KU76	0.361
Aradu.CZ5NE	0.428	305	58.497	42.157	53.595	Araip.G26F0	0.438
Aradu.CZ7XQ	0.353	247	47.581	39.516	41.532	Araip.8M0ZD	0.362
Aradu.CZD45	0.363	803	49.502	33.209	34.577	Araip.HQ7SW	0.358
Aradu.CZI9K	0.339	638	53.678	41.784	43.192	Araip.4S4ZY	0.346
Aradu.CZQ8X	0.4	304	54.754	49.18	42.295	Araip.71W8G	0.415
Aradu.CZW32	0.356	590	49.746	32.995	43.147	Araip.BET6I	0.361
Aradu.D03W8	0.403	553	56.137	36.101	58.303	Araip.1897A	0.399
Aradu.D05FL	0.329	426	41.452	39.344	36.3	Araip.F6TG6	0.338
Aradu.D0AKP	0.375	851	47.418	34.624	34.624	Araip.F82T0	0.38
Aradu.D0I1J	0.424	458	49.455	40.741	42.484	Araip.A515G	0.426
Aradu.D0JN5	0.361	371	42.204	41.935	41.667	Araip.DD2QX	0.367
Aradu.D0L18	0.318	431	50.694	42.824	36.806	Araip.76IDI	0.322
Aradu.D0NVI	0.356	837	44.63	38.663	37.47	Araip.S1I4L	0.342
Aradu.D0RIM	0.405	559	59.286	47.5	44.107	Araip.13HZD	0.403
Aradu.D0SQ3	0.423	150	43.709	41.722	37.748	Araip.A63ZQ	0.372

Aradu.D0URU	0.343	569	48.246	42.632	42.105	Araip.L03M8	0.351
Aradu.D0XFS	0.459	451	47.566	39.381	48.451	Araip.71U6H	0.442
Aradu.D0ZYM	0.363	203	57.353	45.098	45.588	Araip.L7HAD	0.385
Aradu.D11TE	0.456	521	56.705	42.146	54.023	Araip.SP6NR	0.456
Aradu.D12DR	0.408	423	53.066	39.623	43.396	Araip.MQU8W	0.412
Aradu.D15YQ	0.361	978	48.008	39.428	38.509	Araip.4SA3R	0.359
Aradu.D17LB	0.486	150	53.642	43.709	50.993	Araip.U2B8S	0.472
Aradu.D18PX	0.446	175	55.114	38.068	44.886	Araip.AZ7G0	0.44
Aradu.D1CF5	0.347	453	37.004	35.683	34.802	Araip.P8QST	0.342
Aradu.D1CUJ	0.425	589	54.407	40.508	58.983	Araip.UK85B	0.439
Aradu.D1G1F	0.332	497	50.602	35.944	40.361	Araip.NGS4U	0.335
Aradu.D1N5P	0.361	628	47.854	36.725	37.52	Araip.04KBR	0.363
Aradu.D1PNR	0.431	211	49.528	33.019	46.698	Araip.G8VKU	0.441
Aradu.D1QW8	0.375	342	49.854	40.816	52.478	Araip.8X7VT	0.382
Aradu.D1XLL	0.398	270	45.756	35.424	37.269	Araip.G5VT6	0.398
Aradu.D1ZPU	0.425	257	50.775	46.124	39.535	Araip.GLZ9X	0.43
Aradu.D1ZWK	0.438	128	43.411	49.612	41.085	Araip.AKH01	0.463
Aradu.D20L9	0.426	553	52.527	40.614	43.141	Araip.93XAK	0.426
Aradu.D24R3	0.441	467	58.761	46.154	53.632	Araip.EK557	0.412
Aradu.D24Y8	0.362	387	42.268	34.794	52.835	Araip.FDN31	0.365
Aradu.D252Q	0.393	309	45.484	43.548	45.806	Araip.UP1EH	0.403
Aradu.D2EC5	0.48	366	57.766	49.046	68.937	Araip.9D1B8	0.481
Aradu.D2I4Y	0.477	639	51.406	39.531	55.469	Araip.F1S7G	0.482
Aradu.D2IBT	0.357	788	54.753	39.163	35.234	Araip.2T40G	0.359
Aradu.D2JYY	0.386	864	49.48	36.763	37.572	Araip.CDL83	0.386
Aradu.D2MQU	0.378	272	52.015	35.897	38.462	Araip.6983Z	0.382
Aradu.D2NW0	0.379	463	48.922	40.086	47.629	Araip.UZ3PZ	0.39
Aradu.D2U3D	0.349	311	45.513	37.179	28.205	Araip.BM4R7	0.365
Aradu.D2UEN	0.322	472	46.723	37.421	32.135	Araip.Z7SA4	0.326
Aradu.D31R9	0.362	605	55.776	43.399	48.35	Araip.LG7R1	0.362
Aradu.D3HZI	0.383	301	55.298	42.053	36.093	Araip.N3LVC	0.382
Aradu.D3JBM	0.383	758	52.437	45.982	41.765	Araip.B6G88	0.38
Aradu.D3KJ3	0.358	661	47.885	38.369	38.671	Araip.3Z4V7	0.362
Aradu.D3LC1	0.332	1246	45.79	36.167	42.342	Araip.LKW41	0.347
Aradu.D3M3F	0.409	566	49.912	40.741	43.739	Araip.L7P2A	0.403
Aradu.D3RA8	0.37	401	46.517	44.527	44.279	Araip.SR9I2	0.365
Aradu.D3RAM	0.4	567	49.472	38.028	45.599	Araip.KW6YR	0.396
Aradu.D3REG	0.414	701	53.561	37.892	50	Araip.0Y08C	0.41
Aradu.D3TM0	0.317	335	53.274	41.071	42.857	Araip.U33VJ	0.322
Aradu.D400V	0.432	580	49.914	33.735	40.103	Araip.C6ELD	0.443
Aradu.D42IC	0.379	253	53.543	40.945	50.787	Araip.R0CLD	0.379
Aradu.D43UE	0.457	443	50.901	41.441	56.532	Araip.7A0S5	0.441
Aradu.D45R1	0.33	606	56.178	43.493	40.198	Araip.Y0TCW	0.361
Aradu.D464G	0.412	378	53.034	41.425	41.425	Araip.79PEZ	0.412

Aradu.D46RH	0.448	319	52.812	47.188	44.063	Araip.B96WX	0.449
Aradu.D48W8	0.361	409	51.951	43.171	37.561	Araip.DIA58	0.352
Aradu.D4CJV	0.352	496	51.71	41.046	41.449	Araip.0Z0CW	0.368
Aradu.D4CLJ	0.412	249	57.6	41.6	40.8	Araip.P78GJ	0.412
Aradu.D4D16	0.368	502	47.913	39.165	36.382	Araip.SDI9F	0.365
Aradu.D4D3B	0.446	429	45.814	45.116	50	Araip.LUF2N	0.443
Aradu.D4F55	0.394	515	48.837	34.109	41.473	Araip.0H2X8	0.408
Aradu.D4FDN	0.385	459	47.826	44.783	37.174	Araip.M4PVE	0.396
Aradu.D4KTP	0.409	328	49.24	41.337	45.897	Araip.WQL72	0.4
Aradu.D4TC3	0.374	208	46.89	31.1	42.584	Araip.N4SC6	0.372
Aradu.D4WQD	0.39	1170	50.043	38.429	39.71	Araip.1Q1XN	0.385
Aradu.D51RM	0.349	456	51.204	40.044	39.387	Araip.7X70M	0.345
Aradu.D53YG	0.423	294	53.22	47.458	62.034	Araip.GK3PH	0.43
Aradu.D588D	0.353	189	44.211	42.632	37.895	Araip.J8V1R	0.357
Aradu.D5C91	0.383	338	53.982	41.593	58.702	Araip.P61QJ	0.383
Aradu.D5GAK	0.429	331	49.398	38.253	62.349	Araip.1T874	0.423
Aradu.D5VFR	0.313	481	44.398	33.61	40.456	Araip.MI1DI	0.329
Aradu.D5WZF	0.385	782	52.618	39.08	47.893	Araip.NM392	0.387
Aradu.D5Z0P	0.412	421	44.313	40.995	38.626	Araip.M4UKA	0.426
Aradu.D66VA	0.373	248	55.422	45.382	32.129	Araip.J3KIF	0.375
Aradu.D69CU	0.38	279	49.643	45.357	33.214	Araip.7B3CC	0.38
Aradu.D6DD4	0.388	498	46.493	38.277	44.89	Araip.B5KUQ	0.4
Aradu.D6JKJ	0.402	351	48.011	40.625	42.614	Araip.DBZ3D	0.403
Aradu.D6N4B	0.358	415	54.808	43.75	49.76	Araip.BP5EC	0.369
Aradu.D6W9N	0.48	315	50.949	56.329	60.443	Araip.YJ85H	0.493
Aradu.D72GI	0.475	404	45.432	41.975	55.309	Araip.Q50JM	0.472
Aradu.D77GP	0.38	701	51.425	35.755	40.313	Araip.K9GJK	0.374
Aradu.D77VM	0.386	260	52.49	44.444	44.061	Araip.WT3SV	0.366
Aradu.D7B0D	0.374	572	50.087	30.89	41.536	Araip.0QI46	0.381
Aradu.D7HT5	0.448	139	55	45	42.143	Araip.3A81Q	0.448
Aradu.D7HWB	0.378	246	49.393	39.271	40.891	Araip.DAZ2J	0.379
Aradu.D7KSP	0.432	136	44.526	42.336	40.146	Araip.8Y60Z	0.443
Aradu.D7MSN	0.396	719	53.056	45.833	37.361	Araip.R0L1R	0.398
Aradu.D7NJ0	0.43	802	54.172	49.938	52.428	Araip.GZF1H	0.424
Aradu.D7PPK	0.382	557	53.763	37.455	41.577	Araip.80R5B	0.37
Aradu.D7VWN	0.352	1072	49.394	38.956	35.415	Araip.22Y3L	0.354
Aradu.D7Y0Z	0.449	422	53.428	43.262	49.645	Araip.CX0EJ	0.444
Aradu.D7ZWV	0.43	110	60.36	45.045	50.45	Araip.FP7ST	0.439
Aradu.D80L9	0.336	410	50.122	35.766	37.47	Araip.Q99GN	0.328
Aradu.D814J	0.447	241	48.76	47.521	50	Araip.XMM29	0.455
Aradu.D81FK	0.449	253	46.85	46.063	44.488	Araip.8WF5E	0.465
Aradu.D85KR	0.333	490	47.862	40.733	37.882	Araip.21M98	0.333
Aradu.D89KQ	0.336	261	59.924	50.763	32.824	Araip.7V77F	0.328
Aradu.D8A1M	0.337	641	49.688	42.679	33.489	Araip.S2SS4	0.332

Aradu.D8H5F	0.388	1088	53.535	44.169	37.466	Araip.5582Q	0.385
Aradu.D8HZZ	0.335	1015	46.555	36.811	36.22	Araip.172DZ	0.331
Aradu.D8K1Y	0.439	175	43.182	50	45.455	Araip.NQ5HH	0.439
Aradu.D8TZD	0.426	493	52.632	48.583	41.498	Araip.L5RT4	0.418
Aradu.D8U6I	0.408	539	47.222	35.926	46.111	Araip.6P9JS	0.434
Aradu.D8UDW	0.397	1043	49.234	36.494	44.253	Araip.Y7NIY	0.404
Aradu.D8WVG	0.374	406	50.614	40.049	38.084	Araip.6ZX2I	0.38
Aradu.D9DNB	0.451	195	51.531	40.306	46.939	Araip.AZY85	0.44
Aradu.D9IA6	0.375	626	50.877	37.64	38.596	Araip.E13TP	0.379
Aradu.D9ISK	0.438	198	46.734	39.698	45.226	Araip.P2G41	0.433
Aradu.D9N4K	0.42	156	48.408	43.949	43.949	Araip.WQ00Y	0.393
Aradu.D9Q5D	0.484	453	53.744	42.291	57.048	Araip.2CH00	0.487
Aradu.D9QX6	0.399	441	51.131	43.891	35.973	Araip.Y6RSY	0.41
Aradu.DA0G4	0.296	280	38.79	39.502	32.384	Araip.6F52Y	0.304
Aradu.DA1ZW	0.345	826	43.652	39.782	35.429	Araip.JSX23	0.348
Aradu.DA6I6	0.523	431	47.454	42.593	70.139	Araip.IT6Z9	0.523
Aradu.DA7MW	0.345	667	44.311	39.97	34.132	Araip.P0GNG	0.347
Aradu.DAC70	0.405	537	47.77	44.61	43.123	Araip.X7ERC	0.405
Aradu.DAN0Y	0.396	457	48.035	45.852	40.393	Araip.LT8HC	0.411
Aradu.DAT06	0.393	618	53.15	41.357	36.834	Araip.B5YUT	0.389
Aradu.DAV01	0.5	335	51.786	38.095	57.738	Araip.Z1F3X	0.491
Aradu.DB13D	0.371	588	45.501	32.767	46.01	Araip.S5BKS	0.363
Aradu.DB14S	0.436	248	47.39	46.586	53.012	Araip.Y8L0P	0.398
Aradu.DB4LT	0.379	390	53.708	40.409	41.432	Araip.7IA97	0.377
Aradu.DB65S	0.371	556	47.576	36.266	36.804	Araip.MH44P	0.371
Aradu.DB6JJ	0.385	858	47.846	45.402	38.65	Araip.GVH0P	0.383
Aradu.DB78U	0.402	267	47.761	33.209	45.149	Araip.IBD1I	0.395
Aradu.DBJ1I	0.424	108	43.119	40.367	44.037	Araip.VX6NX	0.404
Aradu.DC5GT	0.41	997	56.212	37.675	42.786	Araip.6A4J6	0.411
Aradu.DC7TG	0.408	545	42.308	36.264	42.308	Araip.2WB4K	0.424
Aradu.DCD5A	0.337	223	51.786	31.696	41.071	Araip.6NR98	0.361
Aradu.DCQ49	0.368	357	44.134	42.179	39.385	Araip.NHI3F	0.362
Aradu.DCZ6C	0.423	174	45.143	37.714	57.143	Araip.IZW7Y	0.429
Aradu.DD097	0.433	510	54.403	46.38	59.883	Araip.YU7BP	0.424
Aradu.DD5QZ	0.433	306	52.769	47.231	41.368	Araip.L0WI1	0.418
Aradu.DD99U	0.36	671	50.149	44.196	33.482	Araip.26WUC	0.347
Aradu.DDG3L	0.354	788	49.937	36.375	45.501	Araip.UD0YL	0.344
Aradu.DDQ1E	0.447	320	49.844	41.121	61.994	Araip.X3VWY	0.447
Aradu.DE1N1	0.467	190	49.738	32.461	67.016	Araip.FRW69	0.47
Aradu.DE4QW	0.351	353	54.237	32.486	42.938	Araip.3S8CW	0.354
Aradu.DE67Z	0.288	122	45.528	43.089	26.016	Araip.QI669	0.28
Aradu.DE8ET	0.399	580	54.733	40.275	42.169	Araip.Z22W3	0.406
Aradu.DES3N	0.363	1289	52.481	40.775	39.38	Araip.LBS0B	0.364
Aradu.DEY30	0.359	648	53.005	40.062	40.37	Araip.F7M4C	0.361

Aradu.DF397	0.348	407	52.206	40.686	40.686	Araip.555KP	0.353
Aradu.DF4RH	0.393	358	48.189	46.518	34.54	Araip.59LK4	0.412
Aradu.DF5I2	0.355	948	48.683	33.298	38.145	Araip.QL2G4	0.357
Aradu.DF8UA	0.406	404	52.346	47.901	37.778	Araip.AU0RS	0.395
Aradu.DG44N	0.449	122	50.407	39.837	59.35	Araip.21DP5	0.458
Aradu.DG7AI	0.407	241	49.174	39.669	37.603	Araip.2EE1I	0.411
Aradu.DG903	0.359	614	48.78	41.789	27.642	Araip.F9QDS	0.362
Aradu.DGG35	0.384	889	54.944	41.573	40.112	Araip.UV61I	0.376
Aradu.DGK47	0.437	122	42.276	39.837	40.65	Araip.Z8KSQ	0.445
Aradu.DGR2N	0.397	453	51.322	48.678	39.427	Araip.RYZ75	0.401
Aradu.DGW3H	0.367	411	44.175	34.466	35.68	Araip.18BT4	0.36
Aradu.DH5V9	0.419	328	51.672	38.906	44.985	Araip.0S5VX	0.427
Aradu.DH7CT	0.373	995	50.602	34.639	41.064	Araip.X60H2	0.372
Aradu.DH828	0.414	385	57.772	44.301	40.933	Araip.P3SU7	0.414
Aradu.DH9GD	0.395	957	48.121	38.1	52.192	Araip.75JSE	0.395
Aradu.DHI4B	0.384	381	43.717	46.073	37.173	Araip.15N6Y	0.359
Aradu.DHT3V	0.391	677	48.378	38.938	42.625	Araip.17GEB	0.392
Aradu.DI3D2	0.284	223	50	29.911	33.929	Araip.U291S	0.294
Aradu.DI7FZ	0.414	663	51.506	41.717	43.072	Araip.9D0ML	0.423
Aradu.DI7YA	0.378	479	53.542	38.125	39.792	Araip.0TB3B	0.378
Aradu.DIB1B	0.39	949	46.526	41.579	43.263	Araip.YS3X1	0.404
Aradu.DJ1H2	0.366	1184	52.236	40.338	40.084	Araip.GF7L1	0.375
Aradu.DJ22Q	0.448	145	55.479	47.26	57.534	Araip.6WY95	0.42
Aradu.DJ2MU	0.459	385	46.891	36.528	48.964	Araip.LFA0H	0.459
Aradu.DJF16	0.364	1084	50.323	39.263	34.378	Araip.RSK88	0.354
Aradu.DJW6E	0.359	495	57.258	40.323	40.524	Araip.LMP9N	0.373
Aradu.DK5WH	0.463	321	51.553	44.72	65.528	Araip.CR6RJ	0.473
Aradu.DK67P	0.447	250	60.956	31.873	54.98	Araip.4F1IC	0.464
Aradu.DK6DD	0.461	161	45.679	44.444	47.531	Araip.WZY1Y	0.467
Aradu.DK86D	0.415	365	52.732	44.262	46.448	Araip.91ECR	0.407
Aradu.DKE60	0.359	419	47.143	44.286	41.667	Araip.ZYZ4W	0.361
Aradu.DKR1D	0.358	111	44.643	36.607	33.929	Araip.ECU5E	0.358
Aradu.DL5AX	0.344	430	53.828	36.659	39.907	Araip.F09QG	0.347
Aradu.DL7C8	0.393	352	47.025	43.626	39.943	Araip.W3RGE	0.414
Aradu.DL83H	0.38	249	50.8	32.4	50.4	Araip.GY7IN	0.376
Aradu.DLI1Q	0.367	574	49.217	43.304	35.826	Araip.BR2EY	0.375
Aradu.DLR8J	0.387	364	53.973	38.082	46.849	Araip.10A0F	0.384
Aradu.DLS5S	0.381	373	52.674	35.561	43.316	Araip.7W817	0.371
Aradu.DM13Q	0.333	262	50.19	35.741	49.049	Araip.VUT3K	0.335
Aradu.DM3FR	0.403	280	54.804	42.349	58.363	Araip.RT1FB	0.407
Aradu.DM4CY	0.436	160	55.28	44.099	60.87	Araip.LLJ0S	0.44
Aradu.DM7P1	0.351	596	52.931	37.353	38.023	Araip.B4JB5	0.363
Aradu.DME0M	0.395	757	53.298	40.369	44.855	Araip.TU2HQ	0.399
Aradu.DN2D2	0.482	558	52.057	44.007	67.263	Araip.FH9AI	0.487

Aradu.DN4RU	0.372	117	55.085	35.593	33.051	Araip.9J95X	0.363
Aradu.DN87S	0.358	765	46.475	35.64	38.773	Araip.4L5CB	0.36
Aradu.DN8FJ	0.363	352	49.292	49.858	38.244	Araip.YA5WE	0.369
Aradu.DNJ9W	0.37	651	51.38	34.969	43.558	Araip.X44C7	0.375
Aradu.DP2D5	0.41	266	47.566	39.7	55.805	Araip.LC4KN	0.409
Aradu.DPF9Q	0.357	471	49.364	40.678	33.051	Araip.CL2J8	0.364
Aradu.DQM3T	0.348	469	50.851	44.255	35.532	Araip.C4SEV	0.35
Aradu.DQQ0U	0.359	611	49.51	38.235	44.771	Araip.YZ8C7	0.356
Aradu.DR03T	0.366	273	44.891	40.146	44.526	Araip.LE75L	0.375
Aradu.DR5UL	0.405	542	53.039	39.595	37.753	Araip.60F3J	0.402
Aradu.DRK7I	0.38	1340	54.437	43.475	40.343	Araip.Z3DP0	0.389
Aradu.DRN6A	0.352	821	47.932	35.645	39.051	Araip.73385	0.357
Aradu.DRU5H	0.412	731	59.29	43.033	35.109	Araip.I1ZW3	0.412
Aradu.DS35R	0.376	836	50.777	45.998	37.634	Araip.6NZ59	0.376
Aradu.DS3R5	0.383	344	56.232	37.101	37.971	Araip.6L8TP	0.392
Aradu.DSS3T	0.396	254	41.569	50.98	47.451	Araip.IN0BK	0.383
Aradu.DT0KY	0.368	588	56.367	38.031	38.031	Araip.AA1CY	0.368
Aradu.DT20E	0.377	498	56.313	43.086	39.279	Araip.P2Y88	0.38
Aradu.DT3LR	0.369	334	49.552	35.224	44.179	Araip.17Z7K	0.352
Aradu.DTW5Z	0.378	351	44.318	43.466	43.466	Araip.LC2PG	0.384
Aradu.DU4CZ	0.393	268	50.558	50.186	34.572	Araip.G0TCZ	0.397
Aradu.DU7VI	0.422	417	54.067	41.388	52.392	Araip.74F08	0.417
Aradu.DU86V	0.354	1132	45.543	35.393	40.688	Araip.Q821Q	0.357
Aradu.DUE48	0.34	207	55.769	37.019	38.942	Araip.A3A9L	0.345
Aradu.DV28N	0.425	448	48.552	40.312	48.998	Araip.57AWT	0.425
Aradu.DV3N6	0.369	1005	52.584	38.668	40.557	Araip.DU3VJ	0.364
Aradu.DV9UJ	0.486	116	49.573	50.427	41.88	Araip.52W96	0.514
Aradu.DVH8N	0.343	572	58.29	41.361	39.267	Araip.JHT8J	0.337
Aradu.DVL50	0.323	249	50.4	43.2	40	Araip.CF4RY	0.328
Aradu.DVQ9A	0.376	542	48.066	36.648	45.12	Araip.DKI8Z	0.382
Aradu.DW9IA	0.368	1079	44.352	39.074	44.63	Araip.MY88Z	0.364
Aradu.DW9XI	0.36	314	45.714	39.048	43.492	Araip.TSY02	0.359
Aradu.DWF95	0.43	907	52.203	39.648	30.066	Araip.ZLP0T	0.422
Aradu.DWJ8H	0.396	349	50	33.714	38.857	Araip.I2FQ2	0.402
Aradu.DWL7L	0.38	255	57.422	47.266	43.359	Araip.LJX8Z	0.357
Aradu.DWS1F	0.364	275	57.609	48.551	35.507	Araip.D2NML	0.362
Aradu.DWZ8T	0.436	778	54.814	43.132	47.497	Araip.33PYF	0.438
Aradu.DXG5L	0.28	258	43.243	32.432	49.035	Araip.96GUU	0.29
Aradu.DXP84	0.393	725	51.24	47.107	46.97	Araip.BYV33	0.392
Aradu.DXW6X	0.328	531	48.684	36.278	43.985	Araip.ET7IL	0.324
Aradu.DY43A	0.478	374	55.733	53.6	55.467	Araip.G9AT5	0.482
Aradu.DYF0M	0.402	275	48.551	48.551	43.478	Araip.P095Q	0.402
Aradu.DYP0A	0.439	129	59.231	50	64.615	Araip.LZ85S	0.466
Aradu.DZ2GN	0.385	845	51.537	37.943	41.371	Araip.IHS0X	0.395

Aradu.DZ5Y1	0.269	481	58.299	47.925	29.876	Araip.47DVE	0.251
Aradu.DZ6IG	0.333	262	41.445	39.163	44.487	Araip.WE1Z2	0.329
Aradu.DZY4R	0.437	849	51.412	45.294	49.412	Araip.ET2IE	0.433
Aradu.E01TZ	0.35	706	49.081	36.209	40.594	Araip.M7QQB	0.35
Aradu.E0AA1	0.358	614	44.39	40.813	41.301	Araip.V1HRY	0.361
Aradu.E0HSZ	0.348	1425	47.125	33.731	35.063	Araip.X27S8	0.35
Aradu.E0I63	0.398	340	45.455	36.95	42.229	Araip.G2F1A	0.384
Aradu.E0JEI	0.395	409	48.537	40	35.61	Araip.W7U7R	0.387
Aradu.E0NRM	0.35	474	48.421	37.053	35.789	Araip.U4SN7	0.342
Aradu.E0Q62	0.385	759	55.263	40.789	55.263	Araip.9HV2M	0.383
Aradu.E0QTY	0.335	812	47.232	35.424	34.44	Araip.T9ZJW	0.335
Aradu.E101D	0.344	1260	45.995	40.285	35.052	Araip.WN11J	0.342
Aradu.E1B1W	0.385	204	48.293	45.854	39.512	Araip.51M35	0.38
Aradu.E1KPT	0.366	489	53.673	36.939	44.49	Araip.JL2EX	0.371
Aradu.E1M4X	0.396	192	59.067	37.824	43.005	Araip.50JTJ	0.401
Aradu.E1MX8	0.442	271	52.206	50	54.412	Araip.UWK6L	0.438
Aradu.E1NAR	0.346	305	48.039	35.294	30.065	Araip.DTH2E	0.361
Aradu.E1Q5N	0.37	878	45.734	37.543	44.71	Araip.BN4UN	0.371
Aradu.E1TLU	0.348	304	57.049	39.672	45.246	Araip.XD01H	0.345
Aradu.E1VP1	0.352	414	51.084	42.651	39.518	Araip.EVL3M	0.365
Aradu.E1ZU5	0.329	669	50.896	37.91	38.507	Araip.P1N43	0.324
Aradu.E26DL	0.489	338	48.673	55.752	59.292	Araip.FLW58	0.48
Aradu.E26HT	0.505	228	55.459	40.175	58.952	Araip.E9DE0	0.493
Aradu.E2AL2	0.348	589	43.559	34.915	41.695	Araip.1U1PJ	0.331
Aradu.E2BAC	0.456	361	56.906	40.608	56.077	Araip.R3BYA	0.443
Aradu.E2DS0	0.338	441	52.715	41.176	36.652	Araip.422GS	0.355
Aradu.E2KJV	0.381	324	50.769	42.462	49.846	Araip.I4NIK	0.375
Aradu.E2TII	0.563	237	60.924	50.84	66.807	Araip.AKR06	0.563
Aradu.E2TJL	0.343	319	49.688	41.25	44.688	Araip.L519U	0.343
Aradu.E2VTX	0.372	572	50.262	33.333	39.267	Araip.XH9B3	0.371
Aradu.E2YZD	0.439	195	60.714	49.49	64.286	Araip.N1KVL	0.457
Aradu.E32C9	0.364	663	51.506	36.898	36.747	Araip.5UJ5P	0.378
Aradu.E34IK	0.318	157	43.038	32.911	39.873	Araip.12ZFL	0.295
Aradu.E36HP	0.337	267	45.896	30.224	27.612	Araip.9T4E8	0.336
Aradu.E37Q4	0.569	127	55.469	46.875	56.25	Araip.TCC2K	0.541
Aradu.E3BEW	0.431	191	43.229	47.917	46.875	Araip.WK3TC	0.42
Aradu.E3GM7	0.365	601	57.475	42.525	36.877	Araip.1A4HT	0.371
Aradu.E3I48	0.398	382	48.564	44.125	28.721	Araip.RV8TN	0.404
Aradu.E3SP0	0.412	631	50.791	46.361	54.589	Araip.V9RCS	0.413
Aradu.E3ZED	0.379	1105	51.175	38.517	42.134	Araip.HY5UP	0.377
Aradu.E43JR	0.422	425	55.399	34.038	44.366	Araip.9JP8R	0.397
Aradu.E4AIC	0.358	612	50.734	38.989	38.499	Araip.R12WQ	0.37
Aradu.E4G18	0.327	234	50.638	41.702	37.021	Araip.99548	0.355
Aradu.E4IDB	0.421	422	58.629	37.352	45.39	Araip.H9JFA	0.421

Aradu.E4IE7	0.361	750	47.403	35.153	36.218	Araip.BUW68	0.365
Aradu.E4N51	0.343	290	51.203	43.643	37.457	Araip.E0V6P	0.338
Aradu.E4NC1	0.369	167	55.952	37.5	48.214	Araip.167MA	0.35
Aradu.E4PH8	0.43	413	49.758	42.995	47.101	Araip.MD98T	0.427
Aradu.E4QK5	0.378	1166	51.842	49.272	35.818	Araip.64I9W	0.385
Aradu.E4WB9	0.394	333	44.91	39.521	38.024	Araip.H5EJ2	0.387
Aradu.E515S	0.456	139	42.857	42.857	52.857	Araip.0BH7D	0.444
Aradu.E516Y	0.37	666	46.777	38.381	39.13	Araip.44DMF	0.369
Aradu.E52Z4	0.407	418	55.847	39.379	42.005	Araip.RZ7V8	0.41
Aradu.E58SH	0.39	866	51.672	35.063	39.1	Araip.VVZ0N	0.394
Aradu.E5ATX	0.381	363	45.879	42.033	42.857	Araip.W9LI3	0.372
Aradu.E5KCW	0.38	398	55.388	38.346	37.093	Araip.L7Y2B	0.39
Aradu.E5U8P	0.346	192	47.15	39.378	35.233	Araip.1E680	0.353
Aradu.E6FD3	0.396	507	54.528	38.78	40.945	Araip.L9EW3	0.398
Aradu.E6FTH	0.373	2510	53.724	41.139	36.798	Araip.6B24B	0.371
Aradu.E6ID0	0.358	461	50.216	43.939	31.602	Araip.977B6	0.368
Aradu.E6N9S	0.369	465	51.931	36.695	37.983	Araip.L1T00	0.38
Aradu.E6T6V	0.4	207	42.308	36.538	45.192	Araip.0475C	0.378
Aradu.E6Z8G	0.414	297	47.651	42.617	42.953	Araip.3Q9LP	0.416
Aradu.E70M5	0.343	870	48.794	37.199	35.821	Araip.941M4	0.353
Aradu.E710C	0.398	1095	46.077	46.624	36.679	Araip.YN270	0.388
Aradu.E79CC	0.39	828	48.251	41.013	34.741	Araip.ZS4TJ	0.386
Aradu.E79Y1	0.356	555	48.201	40.288	40.468	Araip.GS8E3	0.35
Aradu.E7L5G	0.335	651	51.38	40.951	34.816	Araip.VV17U	0.34
Aradu.E8471	0.349	354	48.169	46.479	34.648	Araip.ZDT79	0.348
Aradu.E84ZM	0.376	722	51.867	44.813	44.26	Araip.C9CUB	0.381
Aradu.E8BQZ	0.378	613	50.814	37.948	43.811	Araip.X6QYG	0.379
Aradu.E8NYC	0.404	313	49.682	40.764	35.987	Araip.C9FAB	0.407
Aradu.E8PJN	0.421	135	48.529	41.176	47.794	Araip.E5RNZ	0.458
Aradu.E8RZK	0.402	258	49.807	37.066	39.768	Araip.35416	0.421
Aradu.E8VVL	0.384	240	43.983	42.324	31.535	Araip.XF60E	0.396
Aradu.E8W2B	0.47	435	44.954	46.33	54.358	Araip.8SS4C	0.466
Aradu.E8YAE	0.318	837	52.267	37.947	38.305	Araip.R90BF	0.315
Aradu.E90C6	0.382	534	48.224	44.486	41.121	Araip.16F3N	0.384
Aradu.E91AT	0.403	319	49.375	41.875	35.938	Araip.7H21U	0.386
Aradu.E92C1	0.384	268	53.532	38.29	34.201	Araip.U3VBH	0.382
Aradu.E9AVR	0.402	856	53.559	48.658	37.69	Araip.KY8G4	0.4
Aradu.E9CVQ	0.323	417	51.914	35.167	36.364	Araip.LQU55	0.305
Aradu.E9DCA	0.379	171	52.326	40.698	40.116	Araip.QE5ZB	0.381
Aradu.E9HU8	0.377	1991	50.502	41.265	35.944	Araip.EBE31	0.375
Aradu.E9IFL	0.415	476	50.734	40.671	38.994	Araip.KL3B6	0.411
Aradu.E9P6L	0.405	430	53.364	48.26	41.531	Araip.U15G0	0.4
Aradu.E9QI4	0.37	290	49.141	37.113	47.766	Araip.XRM4G	0.356
Aradu.E9QIB	0.332	361	47.514	43.094	36.188	Araip.T9ZZ1	0.346

Aradu.EA3WK	0.412	751	46.543	38.564	40.957	Araip.N7IIM	0.4
Aradu.EA94B	0.343	713	48.459	38.095	35.854	Araip.YQA7J	0.343
Aradu.EA9CU	0.366	328	51.672	44.985	30.091	Araip.IH5JS	0.354
Aradu.EAE80	0.374	1122	52.093	42.921	37.667	Araip.NH8TE	0.373
Aradu.EAF6N	0.372	619	47.258	38.387	47.258	Araip.43ZG0	0.364
Aradu.EAG5M	0.464	630	51.03	42.472	52.773	Araip.B069Y	0.463
Aradu.EAI63	0.364	235	50	38.559	40.254	Araip.8KF2K	0.379
Aradu.EAQ74	0.356	200	49.751	39.801	38.806	Araip.DTN87	0.361
Aradu.EAT9T	0.394	274	54.182	46.545	37.818	Araip.G992G	0.402
Aradu.EAZ6K	0.29	277	42.446	47.482	25.18	Araip.U75BK	0.28
Aradu.EB16E	0.417	140	58.156	46.099	53.901	Araip.3Z0P1	0.432
Aradu.EB27U	0.371	1397	52.003	44.349	37.339	Araip.PH2FC	0.365
Aradu.EB4A5	0.405	327	51.829	43.598	49.695	Araip.EQ8I1	0.412
Aradu.EC406	0.366	709	55.07	44.93	39.296	Araip.EWU0K	0.362
Aradu.ECB76	0.418	676	49.926	37.518	47.563	Araip.U2S9Q	0.423
Aradu.ECK51	0.356	458	55.338	40.305	35.73	Araip.0NL51	0.354
Aradu.ECS2U	0.425	123	45.968	41.129	37.097	Araip.DMN2L	0.437
Aradu.ED8I5	0.334	492	44.219	37.931	31.237	Araip.LY1I1	0.321
Aradu.ED9IS	0.408	578	50.604	44.041	42.142	Araip.V16E1	0.407
Aradu.ED9QM	0.366	588	46.35	38.031	40.238	Araip.M733F	0.359
Aradu.EDD1N	0.419	144	59.31	30.345	40.69	Araip.GY9AA	0.419
Aradu.EE1XG	0.36	624	56.64	39.04	37.76	Araip.3M2RS	0.376
Aradu.EEF2D	0.403	442	46.727	41.084	48.307	Araip.2C5G8	0.394
Aradu.EEP0U	0.446	504	45.149	42.178	48.911	Araip.A48MR	0.443
Aradu.EF2HR	0.488	259	46.538	53.077	63.077	Araip.KC908	0.496
Aradu.EF798	0.355	125	50.794	37.302	58.73	Araip.A05V1	0.429
Aradu.EFI11	0.446	476	48.008	50.943	48.637	Araip.W6QDR	0.441
Aradu.EFX4S	0.447	214	45.581	41.395	62.326	Araip.E9N2X	0.432
Aradu.EG0P5	0.367	194	49.231	39.487	43.077	Araip.JAA8I	0.362
Aradu.EG1H0	0.415	176	50.847	37.853	36.158	Araip.3D6BD	0.427
Aradu.EG2K3	0.397	445	51.121	46.188	44.395	Araip.M1IPR	0.387
Aradu.EG568	0.438	331	49.398	46.988	43.072	Araip.HF59E	0.438
Aradu.EG5ES	0.356	641	51.246	42.523	35.67	Araip.PE2QK	0.354
Aradu.EG6FJ	0.354	1095	55.474	48.358	33.303	Araip.8G8VL	0.366
Aradu.EG6L6	0.376	623	45.673	33.654	40.705	Araip.1Z5U3	0.382
Aradu.EG96G	0.34	708	44.993	33.992	35.543	Araip.MSE9J	0.337
Aradu.EGG67	0.38	383	48.698	39.844	38.021	Araip.FA9IV	0.384
Aradu.EGN24	0.395	357	47.486	39.106	39.385	Araip.3J3Q4	0.383
Aradu.EH0QA	0.363	279	50.357	35.714	47.5	Araip.G3FGY	0.356
Aradu.EH245	0.333	222	47.085	34.529	50.673	Araip.YZ0LP	0.365
Aradu.EH857	0.393	430	46.172	45.708	38.051	Araip.6TB90	0.396
Aradu.EH8VM	0.379	166	55.689	42.515	46.707	Araip.7QT5G	0.385
Aradu.EHC6P	0.406	526	55.598	44.782	40.607	Araip.S4QWE	0.407
Aradu.EI4DF	0.4	763	50	44.11	46.466	Araip.2Y3EX	0.41

Aradu.EI543	0.38	461	50.433	44.156	34.848	Araip.A2EQF	0.379
Aradu.EI69K	0.368	938	53.568	39.723	37.274	Araip.6E9J8	0.367
Aradu.EI7Y8	0.413	378	50.132	42.216	40.369	Araip.3K0PR	0.404
Aradu.EI8LI	0.414	396	47.355	43.073	47.355	Araip.X7A0B	0.412
Aradu.EI99W	0.378	574	60.348	47.652	33.565	Araip.44UYF	0.364
Aradu.EJ00N	0.35	337	45.858	36.391	34.024	Araip.N3NU2	0.352
Aradu.EJ09V	0.383	554	57.838	38.919	43.604	Araip.T2BAX	0.39
Aradu.EJ72Y	0.343	335	51.488	36.31	40.774	Araip.LZ9P5	0.324
Aradu.EJA5A	0.396	457	49.563	38.428	35.371	Araip.I5C3J	0.39
Aradu.EJE3Z	0.399	165	56.024	51.205	39.157	Araip.I81Z4	0.411
Aradu.EJW3I	0.421	623	56.731	41.667	35.417	Araip.A36V0	0.419
Aradu.EK3X2	0.347	439	53.182	41.364	35.455	Araip.Y5NDQ	0.353
Aradu.EK8MW	0.391	430	43.387	35.267	43.155	Araip.9P3VC	0.393
Aradu.EKX2U	0.333	194	46.667	35.897	37.436	Araip.AN5V8	0.331
Aradu.EKZ3T	0.352	1216	47.247	36.483	42.564	Araip.N5JHA	0.347
Aradu.EL04J	0.392	489	52.041	33.878	48.98	Araip.92LQ2	0.392
Aradu.EL0FF	0.408	834	46.467	37.365	42.515	Araip.S9R4E	0.405
Aradu.EL4CE	0.397	459	49.783	41.304	35.87	Araip.SX4W8	0.397
Aradu.ELH7L	0.374	653	46.024	34.098	34.098	Araip.GE0SE	0.372
Aradu.EM2BW	0.436	146	57.143	51.701	53.741	Araip.IV0J9	0.414
Aradu.EM2UC	0.366	237	56.303	41.597	34.874	Araip.I39IS	0.377
Aradu.EM688	0.448	485	50.206	38.683	55.761	Araip.FB4F0	0.45
Aradu.EMM34	0.334	425	53.756	39.671	40.61	Araip.67QDW	0.332
Aradu.EMN7F	0.412	286	43.206	38.328	43.554	Araip.SCS0N	0.422
Aradu.EN58B	0.432	344	44.348	44.348	42.029	Araip.N6C0B	0.429
Aradu.EP05F	0.45	212	61.502	38.028	72.3	Araip.14ESI	0.44
Aradu.EP425	0.386	367	54.348	42.12	38.859	Araip.71CS3	0.376
Aradu.EPY5P	0.409	187	53.191	39.894	56.383	Araip.T88CG	0.403
Aradu.EQ3HU	0.424	328	51.672	33.739	47.416	Araip.L3NWH	0.419
Aradu.EQ5J6	0.401	165	55.422	31.928	43.373	Araip.KX5RC	0.414
Aradu.EQ6UY	0.349	254	45.49	40.784	40.784	Araip.XVW43	0.386
Aradu.EQ8WP	0.385	250	45.817	37.45	34.661	Araip.53NP1	0.376
Aradu.EQB6S	0.365	530	61.205	38.23	36.535	Araip.KHF35	0.375
Aradu.EQE9W	0.335	458	46.623	37.473	38.998	Araip.ZW87C	0.329
Aradu.EQT8P	0.407	502	50.497	39.761	41.153	Araip.R7UJS	0.413
Aradu.EQY7L	0.315	370	48.787	48.248	36.388	Araip.ZQ4EG	0.309
Aradu.ER2GU	0.386	427	56.542	39.486	34.346	Araip.Y9ZBX	0.381
Aradu.ER3NJ	0.432	431	50.231	45.139	40.046	Araip.L5CJ5	0.431
Aradu.ER4SD	0.403	395	51.768	35.606	42.929	Araip.D4H7P	0.41
Aradu.ERC3E	0.422	444	52.135	42.022	46.292	Araip.4KS9Y	0.423
Aradu.ERN5M	0.394	928	48.547	40.797	34.123	Araip.PI1LJ	0.376
Aradu.ERR96	0.301	312	46.006	40.575	31.629	Araip.UB1TB	0.311
Aradu.ES65V	0.377	544	56.514	41.101	43.486	Araip.8074I	0.374
Aradu.ES74T	0.373	536	51.769	46.182	34.823	Araip.LDB2J	0.378

Aradu.ESL6S	0.325	295	45.27	36.824	40.541	Araip.PP1Y2	0.321
Aradu.ESU02	0.403	483	54.132	40.702	41.529	Araip.Q4NNC	0.393
Aradu.ET296	0.34	556	45.601	36.086	28.725	Araip.9LD3P	0.343
Aradu.ET2UI	0.344	512	47.173	43.275	39.766	Araip.VJ1G3	0.346
Aradu.ETD4B	0.417	382	50.653	38.381	52.48	Araip.9C8LR	0.428
Aradu.ETS8A	0.582	250	50.199	57.769	60.159	Araip.ZQ0IN	0.59
Aradu.ETZ8K	0.376	356	47.339	43.137	40.616	Araip.X2KK1	0.383
Aradu.EU0BW	0.382	532	42.214	38.086	39.212	Araip.HRR0E	0.379
Aradu.EU3KX	0.371	469	47.447	39.574	41.277	Araip.VGU5W	0.384
Aradu.EU5HZ	0.385	738	46.279	38.972	40.731	Araip.X69G6	0.386
Aradu.EU9MI	0.338	667	47.754	39.371	38.323	Araip.V9IEZ	0.35
Aradu.EUM6N	0.416	497	53.213	37.952	41.165	Araip.XG7T5	0.416
Aradu.EUP1G	0.377	151	50.658	30.263	44.079	Araip.FN82I	0.377
Aradu.EV17X	0.38	359	50.278	43.889	36.667	Araip.64GPS	0.389
Aradu.EV2Z9	0.384	1135	52.289	42.43	34.243	Araip.TI2C9	0.382
Aradu.EV49X	0.393	367	48.641	44.293	39.13	Araip.P5P82	0.396
Aradu.EV762	0.401	296	45.791	40.067	55.892	Araip.7JN11	0.394
Aradu.EV8G8	0.383	286	56.098	45.645	53.31	Araip.5BR6I	0.384
Aradu.EVB44	0.491	168	42.012	51.479	44.97	Araip.638DS	0.479
Aradu.EW29C	0.398	255	47.656	39.063	37.891	Araip.3GQ6X	0.391
Aradu.EW7BI	0.374	729	49.726	42.74	36.301	Araip.K54B1	0.376
Aradu.EW82S	0.395	263	45.076	32.955	51.515	Araip.NED9I	0.379
Aradu.EWB3L	0.461	184	51.892	52.973	62.162	Araip.UFN92	0.461
Aradu.EWR3P	0.41	283	41.197	31.338	38.38	Araip.UPR46	0.403
Aradu.EWZ0P	0.404	192	58.031	44.041	44.041	Araip.T6R33	0.399
Aradu.EX30Z	0.326	624	47.36	40.32	40.8	Araip.D7QWM	0.331
Aradu.EX4VM	0.374	251	51.984	39.286	36.508	Araip.MY3WG	0.369
Aradu.EX52R	0.336	1105	50.542	38.788	38.065	Araip.T2824	0.336
Aradu.EX904	0.385	466	50.107	41.756	38.116	Araip.SAL9A	0.383
Aradu.EX9SJ	0.431	498	55.11	43.487	54.108	Araip.K24FA	0.431
Aradu.EXQ60	0.386	1455	48.008	39.148	34.066	Araip.NHV7F	0.383
Aradu.EXV8Z	0.444	476	50.524	42.977	59.958	Araip.CQA3P	0.43
Aradu.EY5U3	0.399	394	43.038	32.152	38.481	Araip.FZ3D1	0.411
Aradu.EYD2G	0.404	786	50.445	37.865	42.186	Araip.H6BYJ	0.409
Aradu.EYN6M	0.395	284	49.825	32.281	51.228	Araip.V8DUY	0.395
Aradu.EYQ2A	0.318	396	52.897	52.897	30.227	Araip.DN8BF	0.316
Aradu.EYV3C	0.436	453	53.965	46.035	46.916	Araip.B7VJF	0.429
Aradu.EZ25P	0.379	627	49.682	40.446	39.65	Araip.N0TEM	0.383
Aradu.EZ30H	0.355	428	49.65	39.86	35.431	Araip.XZ3ZL	0.357
Aradu.EZ4BH	0.421	365	50.273	44.809	51.366	Araip.3X1VK	0.415
Aradu.EZ6B1	0.412	385	48.187	41.969	51.554	Araip.M87WJ	0.401
Aradu.EZ7WI	0.344	328	51.368	40.426	43.465	Araip.23LH0	0.341
Aradu.EZJ0T	0.373	473	44.937	34.599	36.709	Araip.Y8HLM	0.366
Aradu.EZK5X	0.397	476	49.057	36.897	45.283	Araip.EMY47	0.397

Aradu.EZU9P	0.358	1256	50.676	39.857	36.913	Araip.LA28K	0.366
Aradu.EZW4U	0.513	115	75	57.759	41.379	Araip.1UW0G	0.545
Aradu.EZW7V	0.393	532	49.719	38.274	39.4	Araip.CER5U	0.406
Aradu.F00XS	0.44	250	50.598	46.614	38.247	Araip.TV66E	0.432
Aradu.F0544	0.401	686	51.237	45.56	36.827	Araip.C1R8D	0.405
Aradu.F05R2	0.406	200	53.731	36.816	43.781	Araip.62ZJ7	0.439
Aradu.F06JI	0.401	482	51.553	42.236	39.752	Araip.DM29G	0.406
Aradu.F08JS	0.416	155	51.923	44.872	30.769	Araip.E27FI	0.405
Aradu.F0ADU	0.336	537	49.628	38.476	41.45	Araip.3GM3S	0.34
Aradu.F0D6Z	0.382	349	51.429	42.857	41.714	Araip.CAX6Y	0.384
Aradu.F0EGY	0.346	307	50.325	39.61	35.39	Araip.BHW2G	0.342
Aradu.F0FER	0.393	486	51.951	38.398	45.791	Araip.4P78F	0.387
Aradu.F0W17	0.383	280	44.128	43.772	33.452	Araip.ML9SJ	0.386
Aradu.F0Y0D	0.436	520	46.065	40.115	47.601	Araip.PNY21	0.437
Aradu.F19SY	0.451	236	59.072	52.321	64.979	Araip.PD9M5	0.467
Aradu.F1E13	0.366	985	51.623	38.641	42.799	Araip.C1EVE	0.365
Aradu.F1MD3	0.367	605	49.835	43.399	38.614	Araip.136S0	0.363
Aradu.F297C	0.423	544	48.073	38.349	53.945	Araip.Q7NLU	0.433
Aradu.F2B57	0.414	415	57.692	35.337	58.413	Araip.V3I44	0.414
Aradu.F2CY5	0.383	853	50.468	39.813	44.028	Araip.E6C1Q	0.379
Aradu.F2D3W	0.435	390	45.269	30.435	41.944	Araip.MSQ60	0.418
Aradu.F2DT2	0.422	360	44.044	40.72	40.997	Araip.Q3R6H	0.413
Aradu.F2KAM	0.418	247	49.194	43.548	41.129	Araip.J19JD	0.414
Aradu.F2R8P	0.388	1020	57.689	46.915	36.337	Araip.Q10CZ	0.388
Aradu.F2VIG	0.423	538	55.659	41.929	39.332	Araip.798H5	0.418
Aradu.F2XPJ	0.388	230	49.351	45.022	33.333	Araip.6H93L	0.409
Aradu.F2ZNU	0.382	424	48	31.059	43.059	Araip.X9SZ4	0.367
Aradu.F360A	0.485	206	51.208	38.647	38.647	Araip.4CZ22	0.475
Aradu.F3745	0.401	406	48.403	43.489	54.545	Araip.G3RW4	0.43
Aradu.F38CM	0.376	129	43.846	34.615	40.769	Araip.A8ULT	0.381
Aradu.F3FT3	0.37	598	56.928	45.91	38.898	Araip.JEN04	0.37
Aradu.F3I3T	0.376	603	51.821	38.079	41.556	Araip.GEK49	0.374
Aradu.F3KB2	0.453	181	47.253	48.352	50.549	Araip.SP2PF	0.424
Aradu.F3VDW	0.389	651	50.613	42.638	38.65	Araip.2290W	0.378
Aradu.F3XDM	0.485	307	49.026	37.987	54.545	Araip.J47H3	0.488
Aradu.F40AG	0.379	339	47.059	41.176	38.824	Araip.19CBQ	0.38
Aradu.F433U	0.362	479	52.083	35.833	46.042	Araip.QU0FD	0.362
Aradu.F4EXD	0.387	501	53.785	43.028	38.446	Araip.K7EKQ	0.381
Aradu.F4S3N	0.336	134	44.444	42.963	32.593	Araip.Q083N	0.336
Aradu.F4UZI	0.38	549	44.909	36.727	41.636	Araip.YL5Y5	0.378
Aradu.F4Y7W	0.471	298	61.873	39.465	42.14	Araip.X6TNY	0.457
Aradu.F510W	0.396	297	62.081	50.336	36.242	Araip.IFK0L	0.399
Aradu.F52BW	0.379	413	49.517	37.681	43.72	Araip.57VD8	0.374
Aradu.F54T9	0.383	698	46.781	38.77	43.634	Araip.X6QP4	0.378

Aradu.F591F	0.445	336	53.412	39.763	47.181	Araip.W8SB4	0.427
Aradu.F5B1R	0.369	693	43.804	37.32	41.21	Araip.55A1G	0.374
Aradu.F5KZV	0.387	329	52.727	37.576	49.091	Araip.6PA6C	0.396
Aradu.F5XX7	0.48	133	45.522	46.269	46.269	Araip.WV79D	0.472
Aradu.F623P	0.375	995	48.896	36.546	43.474	Araip.F3F2F	0.376
Aradu.F64Z1	0.439	387	46.392	47.423	52.062	Araip.914CH	0.439
Aradu.F66UW	0.414	248	55.422	45.783	55.422	Araip.X1Q90	0.406
Aradu.F6ABZ	0.364	246	53.036	40.891	46.964	Araip.X394B	0.377
Aradu.F6CTF	0.398	370	56.334	34.232	36.658	Araip.5J63B	0.403
Aradu.F6HJK	0.383	1284	51.673	41.712	34.864	Araip.64L2N	0.381
Aradu.F6LF0	0.409	209	53.81	36.19	49.048	Araip.2G28P	0.404
Aradu.F6LZ6	0.478	119	50.833	43.333	53.333	Araip.RM2KP	0.478
Aradu.F6N2R	0.356	1542	56.837	44.977	38.367	Araip.313YK	0.356
Aradu.F6VM1	0.414	622	53.612	44.141	47.833	Araip.V3HZN	0.417
Aradu.F6Z4G	0.443	330	46.526	43.505	58.912	Araip.F5AGL	0.449
Aradu.F70UL	0.387	344	48.116	41.739	42.029	Araip.5C457	0.372
Aradu.F77GY	0.388	1123	56.851	42.082	42.527	Araip.TUB1K	0.386
Aradu.F7BLG	0.366	1010	48.071	36.894	38.872	Araip.MW082	0.374
Aradu.F7D9L	0.351	221	50	43.243	43.694	Araip.QZ6Y2	0.393
Aradu.F7E59	0.487	165	53.614	45.783	59.639	Araip.4UB04	0.5
Aradu.F7Z5Y	0.406	239	42.5	39.167	36.25	Araip.TB7YD	0.404
Aradu.F84AW	0.435	331	53.313	39.759	50.301	Araip.U1V6F	0.438
Aradu.F8DL0	0.315	307	47.403	39.935	38.636	Araip.YJ9H2	0.331
Aradu.F8JPN	0.382	280	49.11	29.537	41.993	Araip.60Q87	0.402
Aradu.F8MV2	0.359	404	53.827	39.506	40	Araip.VEY8S	0.365
Aradu.F8T28	0.382	738	52.909	38.295	39.783	Araip.S8H7F	0.384
Aradu.F8VRL	0.402	382	50.392	43.342	50.392	Araip.1Z0SD	0.413
Aradu.F8VUJ	0.326	196	50.761	44.162	37.563	Araip.01T7B	0.328
Aradu.F9182	0.402	361	55.249	46.133	35.912	Araip.9P9V2	0.402
Aradu.F950M	0.464	147	50	46.622	39.189	Araip.344D4	0.438
Aradu.F99CN	0.478	190	46.597	38.22	51.309	Araip.1Z65W	0.441
Aradu.F9AQV	0.397	144	65.517	33.793	51.034	Araip.G8XIB	0.375
Aradu.F9BJN	0.377	391	50.765	41.071	38.265	Araip.Y5DXY	0.37
Aradu.F9JAF	0.372	331	52.108	48.193	37.349	Araip.SS61C	0.378
Aradu.F9KJM	0.5	108	53.211	44.037	48.624	Araip.U7F06	0.519
Aradu.F9L0R	0.373	798	45.932	38.673	46.308	Araip.0I82K	0.37
Aradu.F9XLT	0.42	340	49.853	44.282	46.334	Araip.GV8QE	0.417
Aradu.FA1DK	0.344	445	48.879	41.256	46.861	Araip.RPP7Y	0.342
Aradu.FA889	0.391	291	51.027	38.699	43.493	Araip.KCY11	0.398
Aradu.FA88A	0.341	416	49.4	40.767	38.849	Araip.F8FRB	0.341
Aradu.FA8DG	0.344	227	50	30.263	41.667	Araip.MF1KR	0.333
Aradu.FA8EI	0.335	505	47.628	40.711	43.478	Araip.83IVK	0.326
Aradu.FB522	0.415	260	57.854	36.782	38.314	Araip.ZL723	0.419
Aradu.FB7VW	0.387	551	49.638	42.029	44.384	Araip.WB4KA	0.391

Aradu.FBB2P	0.437	256	41.634	36.965	40.856	Araip.GW6DK	0.445
Aradu.FC1CK	0.388	216	53.917	37.788	36.866	Araip.1J91U	0.386
Aradu.FC1L9	0.412	277	53.957	34.892	43.165	Araip.11AMG	0.407
Aradu.FC7FL	0.398	135	47.059	26.471	44.118	Araip.23M3Y	0.383
Aradu.FCK70	0.373	139	44.286	32.857	37.857	Araip.R004A	0.377
Aradu.FD1ZK	0.379	446	50.336	44.966	42.058	Araip.Y1YK4	0.386
Aradu.FD81T	0.468	139	56.429	55.714	37.143	Araip.MZ5SZ	0.451
Aradu.FD8VQ	0.37	607	51.151	33.059	43.75	Araip.F4F1R	0.372
Aradu.FDG48	0.412	508	55.599	41.65	58.743	Araip.2AZ3I	0.41
Aradu.FE1BY	0.381	387	46.392	40.206	58.247	Araip.S8PCM	0.376
Aradu.FEU0W	0.4	445	49.103	40.135	48.879	Araip.S2ICC	0.405
Aradu.FF3VB	0.394	881	50.454	42.177	42.517	Araip.74IBX	0.393
Aradu.FF4D5	0.395	562	52.575	42.629	53.286	Araip.6PJ8B	0.39
Aradu.FF6JW	0.328	287	44.444	39.236	41.319	Araip.70UIF	0.349
Aradu.FF8NM	0.351	1046	47.851	39.637	36.008	Araip.ZHH5I	0.343
Aradu.FFW2J	0.41	197	52.02	34.343	39.394	Araip.DL6JR	0.41
Aradu.FFY3F	0.391	673	53.116	41.543	39.763	Araip.D0YN7	0.378
Aradu.FG20F	0.446	429	53.023	40.465	54.651	Araip.KB0T7	0.45
Aradu.FGV5A	0.344	1208	53.681	39.041	40.943	Araip.U5NKT	0.341
Aradu.FH10G	0.409	330	57.704	39.275	53.172	Araip.I1474	0.412
Aradu.FH3GQ	0.373	838	55.185	41.359	42.908	Araip.AYG40	0.381
Aradu.FHT7B	0.427	571	50.524	43.706	49.301	Araip.XVQ2M	0.427
Aradu.FHW89	0.398	211	48.113	54.717	51.887	Araip.43JFQ	0.378
Aradu.FI298	0.425	234	52.34	41.702	44.681	Araip.I35QI	0.394
Aradu.FI2RL	0.419	347	48.563	41.379	36.207	Araip.UUA6E	0.419
Aradu.FI39R	0.409	379	50.263	38.421	46.053	Araip.5XD8H	0.417
Aradu.FIU9K	0.309	258	45.174	41.699	35.521	Araip.30XFU	0.315
Aradu.FJ32V	0.414	487	52.254	38.934	41.598	Araip.ULZ9V	0.406
Aradu.FJ441	0.34	204	59.024	43.415	41.463	Araip.86J2T	0.34
Aradu.FJ6K3	0.379	360	44.875	40.443	41.551	Araip.DK7U4	0.379
Aradu.FJQ80	0.391	1024	51.512	36.098	45.854	Araip.S9NSP	0.383
Aradu.FJZ7Z	0.348	711	51.124	34.551	39.185	Araip.29BDM	0.343
Aradu.FK0J4	0.388	255	47.656	35.938	39.063	Araip.Z370E	0.391
Aradu.FK3YI	0.385	276	56.318	40.794	36.823	Araip.UE1LF	0.372
Aradu.FK3YK	0.366	378	48.285	43.536	41.953	Araip.GAB7J	0.368
Aradu.FK4M3	0.409	772	47.995	36.999	42.044	Araip.CJI78	0.413
Aradu.FL0YZ	0.313	186	41.176	35.829	40.107	Araip.M93U4	0.318
Aradu.FL7H6	0.396	581	47.766	37.973	40.722	Araip.PM2EU	0.401
Aradu.FL7HA	0.365	386	56.331	38.501	40.052	Araip.ZJE85	0.357
Aradu.FL84V	0.332	341	42.105	34.211	45.906	Araip.WVU2E	0.354
Aradu.FL9HS	0.322	1096	41.203	35.643	34.913	Araip.SS9V6	0.341
Aradu.FLZ7V	0.401	242	46.091	39.095	34.568	Araip.U3HJ0	0.384
Aradu.FM0YX	0.384	865	49.192	38.222	40.878	Araip.GV48H	0.383
Aradu.FMP57	0.472	277	45.683	45.683	70.863	Araip.N59F9	0.457

Aradu.FN1D7	0.432	368	46.612	36.585	52.575	Araip.Z4GS0	0.438
Aradu.FN25A	0.374	273	50.73	40.876	48.905	Araip.M2HHN	0.355
Aradu.FN4CD	0.424	589	51.186	43.898	34.237	Araip.KU3NI	0.438
Aradu.FN83T	0.393	568	48.858	40.422	43.234	Araip.E7ENS	0.388
Aradu.FNB9W	0.361	181	53.846	41.758	39.56	Araip.2A9KZ	0.375
Aradu.FNH9F	0.39	663	50.602	42.62	39.608	Araip.VQ5T4	0.39
Aradu.FNM4G	0.393	757	46.966	38.654	48.021	Araip.CY8BI	0.4
Aradu.FP0C1	0.34	434	47.586	45.747	41.609	Araip.GV7RQ	0.337
Aradu.FP3TI	0.379	388	48.329	40.103	44.216	Araip.CQY50	0.372
Aradu.FP3UD	0.433	367	59.783	41.033	59.239	Araip.E335X	0.433
Aradu.FPC2C	0.524	256	68.872	50.973	61.089	Araip.U0WFW	0.516
Aradu.FPU47	0.364	341	52.047	38.012	38.304	Araip.4U4XR	0.366
Aradu.FQ240	0.41	236	50.211	48.101	40.084	Araip.9F12T	0.418
Aradu.FQ3N4	0.384	911	50.768	30.482	38.268	Araip.B7GXE	0.382
Aradu.FQ6XD	0.338	278	47.67	33.692	39.068	Araip.87M6M	0.343
Aradu.FR1WN	0.379	387	46.134	41.495	48.196	Araip.65AV8	0.388
Aradu.FRD61	0.401	393	61.421	41.371	40.609	Araip.VC6AK	0.406
Aradu.FRY2U	0.35	794	49.182	37.484	34.969	Araip.Y25J6	0.353
Aradu.FS4N2	0.446	102	51.456	45.631	42.718	Araip.1V67D	0.436
Aradu.FS7LG	0.388	706	53.89	38.614	44.272	Araip.MYU90	0.379
Aradu.FSK9A	0.355	433	56.912	43.318	30.645	Araip.B9NXS	0.349
Aradu.FSY43	0.392	274	40	33.455	43.273	Araip.I9EB2	0.373
Aradu.FT1FE	0.337	448	42.984	39.421	40.98	Araip.RQ7L5	0.34
Aradu.FT5QT	0.343	491	42.073	40.244	31.707	Araip.43I77	0.347
Aradu.FT8DK	0.37	388	51.671	40.36	48.843	Araip.I8X4Y	0.37
Aradu.FTI5H	0.366	663	49.699	37.048	38.102	Araip.G5FJE	0.372
Aradu.FTT59	0.395	855	56.075	45.327	41.121	Araip.P08LA	0.39
Aradu.FU1XB	0.383	141	50.704	35.915	37.324	Araip.G3PYU	0.383
Aradu.FU605	0.422	175	50	36.932	46.023	Araip.B1BGN	0.413
Aradu.FU781	0.447	601	50.664	42.027	50.498	Araip.Y92IB	0.443
Aradu.FU7AU	0.399	475	55.042	43.487	46.849	Araip.C17N5	0.392
Aradu.FU7NG	0.362	806	44.362	37.546	38.042	Araip.1Y0FU	0.365
Aradu.FUK65	0.326	509	47.255	35.49	39.804	Araip.F5F3I	0.34
Aradu.FUM3Y	0.399	573	48.606	45.296	43.031	Araip.MMB4Q	0.403
Aradu.FV1SH	0.371	547	48.905	37.591	41.971	Araip.32YK0	0.375
Aradu.FV4D1	0.326	545	49.817	38.645	41.209	Araip.FLT6G	0.319
Aradu.FV6YV	0.365	380	53.543	40.945	45.144	Araip.F1ZZD	0.377
Aradu.FV8TW	0.378	1838	48.668	44.589	39.424	Araip.AAM5W	0.379
Aradu.FW1J1	0.374	996	56.169	41.926	34.504	Araip.E8JYR	0.38
Aradu.FW3IU	0.426	455	50.219	47.368	50.219	Araip.L8NZD	0.421
Aradu.FW5R5	0.375	813	48.28	41.278	35.012	Araip.MCH9G	0.373
Aradu.FW65S	0.411	576	56.672	41.941	35.702	Araip.ZZM8J	0.416
Aradu.FWD57	0.418	115	55.172	46.552	38.793	Araip.Q2LZS	0.418
Aradu.FWL77	0.382	401	50.249	38.806	36.567	Araip.D52D9	0.375

Aradu.FWS4A	0.395	155	51.923	36.538	50	Araip.X2EME	0.408
Aradu.FWZ0U	0.411	220	63.801	39.819	36.199	Araip.SQD8Z	0.395
Aradu.FX47V	0.396	160	47.205	40.373	39.13	Araip.7A9UM	0.392
Aradu.FX57K	0.334	605	45.875	37.129	34.323	Araip.T1F9U	0.336
Aradu.FX5WG	0.41	206	42.995	41.546	49.758	Araip.7R33Q	0.401
Aradu.FXP12	0.497	385	57.513	41.71	61.399	Araip.2U0RL	0.488
Aradu.FZ0BJ	0.349	557	45.878	38.889	36.559	Araip.D19R8	0.346
Aradu.FZ0BW	0.406	134	51.111	50.37	48.148	Araip.01H2N	0.402
Aradu.FZ0TC	0.416	237	46.218	37.395	41.597	Araip.QN360	0.439
Aradu.G013M	0.395	346	59.078	44.957	46.686	Araip.Z633S	0.401
Aradu.G04HZ	0.361	1306	46.978	41.622	38.256	Araip.F6UPB	0.359
Aradu.G05M1	0.4	683	52.193	41.52	41.52	Araip.NW1RI	0.394
Aradu.G07IW	0.397	632	51.185	27.646	40.442	Araip.PRB9P	0.407
Aradu.G09L8	0.356	424	47.294	44.941	37.412	Araip.97TWR	0.366
Aradu.G0AVK	0.39	276	46.931	44.404	49.458	Araip.I5FVU	0.395
Aradu.G0BMM	0.375	1145	47.295	36.387	39.703	Araip.V7N27	0.375
Aradu.G0DW8	0.47	405	45.567	41.379	49.015	Araip.95R25	0.475
Aradu.G0FGB	0.45	393	54.061	47.208	51.523	Araip.D59X4	0.449
Aradu.G0IJA	0.347	1101	46.824	34.392	39.655	Araip.JP4WR	0.346
Aradu.G0P4Z	0.35	249	48.8	40	41.2	Araip.KM8WJ	0.356
Aradu.G0QWJ	0.494	249	46.8	39.2	58.4	Araip.X10KQ	0.469
Aradu.G0S92	0.473	229	48.261	40.87	51.739	Araip.A841F	0.464
Aradu.G0Z55	0.364	292	46.416	31.399	39.249	Araip.VA8G3	0.375
Aradu.G0ZCH	0.345	126	51.181	44.882	42.52	Araip.U6HL7	0.378
Aradu.G14M4	0.387	175	45.455	42.045	46.591	Araip.KW1YW	0.368
Aradu.G1DD8	0.384	438	51.481	39.18	50.797	Araip.ZWQ00	0.384
Aradu.G1DZV	0.42	327	52.134	43.598	53.659	Araip.X6JCD	0.425
Aradu.G1RFE	0.318	437	51.142	43.151	33.105	Araip.6AX31	0.327
Aradu.G1YNF	0.385	444	46.517	39.551	42.472	Araip.S3GXY	0.372
Aradu.G1Z6G	0.372	618	54.766	39.418	36.672	Araip.MJE46	0.367
Aradu.G21TT	0.354	477	52.51	43.305	39.958	Araip.UHJ6H	0.381
Aradu.G229R	0.402	193	53.608	32.99	52.577	Araip.6TW9A	0.402
Aradu.G26I2	0.423	253	48.425	40.157	52.756	Araip.PUS3S	0.434
Aradu.G28T8	0.354	870	54.076	37.199	39.265	Araip.GE2VQ	0.361
Aradu.G2CUK	0.375	202	55.172	45.813	37.931	Araip.PR8W2	0.399
Aradu.G2EQN	0.39	461	49.784	39.61	38.095	Araip.09PBC	0.385
Aradu.G2GUD	0.364	1483	49.798	38.208	33.76	Araip.C1PNV	0.358
Aradu.G2R4U	0.327	701	50.855	41.88	33.903	Araip.JH1AW	0.323
Aradu.G2TPD	0.336	468	44.136	33.049	39.872	Araip.T6L7D	0.333
Aradu.G2VQL	0.528	217	53.67	45.413	48.165	Araip.PXU5X	0.507
Aradu.G2YAZ	0.339	291	45.205	34.932	35.274	Araip.64VMF	0.338
Aradu.G307L	0.368	547	47.993	44.708	38.504	Araip.C53V2	0.376
Aradu.G31GN	0.38	656	47.184	39.726	35.008	Araip.W36DI	0.375
Aradu.G31YK	0.362	368	49.322	38.211	34.688	Araip.GVI8N	0.365

Aradu.G344I	0.416	332	51.351	45.646	40.541	Araip.32BSU	0.414
Aradu.G39DJ	0.375	567	47.711	38.556	35.739	Araip.I0S4M	0.389
Aradu.G3CH2	0.41	401	50.498	41.294	42.04	Araip.7PF1U	0.4
Aradu.G3TP5	0.403	125	39.683	38.889	36.508	Araip.SY4A0	0.408
Aradu.G3TTV	0.454	381	54.974	37.958	49.215	Araip.7H2KA	0.458
Aradu.G3X0F	0.416	260	50.192	32.95	42.146	Araip.L9ELN	0.403
Aradu.G3XBG	0.371	237	51.261	41.176	37.815	Araip.5GT5G	0.411
Aradu.G40TW	0.382	582	47.341	36.192	33.791	Araip.0YN4A	0.383
Aradu.G43SA	0.359	1471	50.476	41.44	37.772	Araip.929NY	0.351
Aradu.G44I1	0.418	383	50.26	42.708	44.01	Araip.28HGC	0.43
Aradu.G44IN	0.386	534	51.402	38.318	39.065	Araip.L1TDI	0.389
Aradu.G44X8	0.383	241	46.281	40.083	36.777	Araip.UVI0L	0.386
Aradu.G47J2	0.397	411	49.272	40.777	37.864	Araip.PKQ0A	0.403
Aradu.G4DCM	0.407	171	48.256	34.884	42.442	Araip.9U2AW	0.414
Aradu.G4DWJ	0.388	753	52.918	35.676	44.297	Araip.0A5UL	0.395
Aradu.G4M3I	0.474	180	54.696	40.331	56.354	Araip.JLS7G	0.468
Aradu.G4PX6	0.349	751	51.064	37.899	36.17	Araip.KNT6A	0.351
Aradu.G4U2S	0.414	458	55.773	44.227	38.126	Araip.UU2Q4	0.408
Aradu.G4VEH	0.346	352	53.541	37.96	40.227	Araip.049JK	0.364
Aradu.G4VT9	0.349	1118	46.559	35.121	34.406	Araip.58W0E	0.347
Aradu.G5CNQ	0.315	1029	48.835	32.33	40.777	Araip.VW462	0.31
Aradu.G5F0C	0.463	411	59.709	50.971	54.126	Araip.T2RUM	0.452
Aradu.G5NTR	0.349	179	48.333	46.111	39.444	Araip.B5WG1	0.349
Aradu.G5P3T	0.398	487	50	39.139	43.033	Araip.CZ0IQ	0.393
Aradu.G5PJM	0.36	184	44.865	35.135	36.757	Araip.AA5NQ	0.361
Aradu.G5W2I	0.388	747	49.465	48.93	37.567	Araip.UP566	0.386
Aradu.G5WQA	0.406	928	53.498	38.859	45.318	Araip.DXE3Z	0.4
Aradu.G63Y1	0.403	485	48.971	40.123	51.646	Araip.3D13X	0.424
Aradu.G676K	0.352	744	55.973	46.711	38.658	Araip.W8N64	0.357
Aradu.G68BX	0.385	246	44.939	34.413	38.057	Araip.B227D	0.368
Aradu.G6CRU	0.311	513	43.774	38.327	33.268	Araip.ZD8Z8	0.325
Aradu.G6GR7	0.351	371	40.86	39.516	35.484	Araip.TQY0D	0.359
Aradu.G6N6R	0.398	706	54.031	46.676	41.443	Araip.DP3JP	0.39
Aradu.G6PP5	0.393	462	45.572	40.821	42.117	Araip.U49S5	0.391
Aradu.G6ZA5	0.393	623	52.083	34.615	45.994	Araip.WYM3Y	0.389
Aradu.G6ZH0	0.401	909	54.286	49.56	30	Araip.Q4K8M	0.41
Aradu.G76IS	0.447	179	60	40.556	69.444	Araip.26B8A	0.441
Aradu.G78T6	0.392	1265	49.921	35.782	43.444	Araip.83LYV	0.397
Aradu.G7BFP	0.306	520	49.904	42.035	34.165	Araip.CT78Y	0.316
Aradu.G7CKS	0.508	259	58.077	37.692	66.923	Araip.43P31	0.508
Aradu.G7CZU	0.36	1022	50.538	35.973	43.695	Araip.BT3ZZ	0.361
Aradu.G7DFD	0.411	687	51.163	42.297	40.116	Araip.ULK6W	0.409
Aradu.G7F8E	0.385	711	52.528	39.185	55.618	Araip.V9S7Z	0.39
Aradu.G7UXI	0.446	295	56.081	46.959	62.5	Araip.EKK4C	0.436

Aradu.G85IC	0.449	173	40.805	46.552	31.034	Araip.Q3DRI	0.441
Aradu.G8908	0.388	412	47.942	44.068	40.678	Araip.ZJ8QE	0.383
Aradu.G8EKB	0.422	282	49.117	44.876	46.643	Araip.BM4V8	0.4
Aradu.G8ESS	0.299	147	42.568	39.865	36.486	Araip.T484U	0.299
Aradu.G8GYX	0.47	157	51.899	37.975	48.734	Araip.MA9F1	0.461
Aradu.G8KNY	0.391	993	52.515	44.064	36.419	Araip.FL5PZ	0.398
Aradu.G8W4L	0.381	244	48.571	40	37.551	Araip.7Y21E	0.393
Aradu.G957G	0.457	292	42.321	41.297	50.171	Araip.8NY60	0.45
Aradu.G99GB	0.401	638	55.869	38.811	47.574	Araip.W5GXX	0.401
Aradu.G99LQ	0.336	862	50.637	35.805	34.762	Araip.W6TLM	0.331
Aradu.G9DQQ	0.337	326	50.459	40.061	43.731	Araip.96W3Q	0.33
Aradu.G9EXB	0.333	219	51.818	37.273	37.273	Araip.5Z5WT	0.302
Aradu.G9QEY	0.349	1242	50.282	41.271	34.191	Araip.79CH7	0.35
Aradu.G9RLQ	0.515	206	48.792	60.87	65.7	Araip.Q3U94	0.522
Aradu.G9UB3	0.426	244	50.204	35.918	35.918	Araip.8Z7ED	0.435
Aradu.G9W58	0.439	178	39.665	55.307	38.547	Araip.YBU40	0.429
Aradu.GA66V	0.401	450	50.333	37.916	42.129	Araip.83S01	0.402
Aradu.GA7JT	0.343	1181	48.054	36.971	37.563	Araip.ZVW3Y	0.343
Aradu.GA7ZA	0.381	297	47.987	36.577	43.96	Araip.RVB68	0.403
Aradu.GA8EY	0.427	527	52.083	39.962	61.174	Araip.ZY8TR	0.43
Aradu.GA8V3	0.357	1117	50.626	35.868	35.778	Araip.0Y4CE	0.355
Aradu.GAW9B	0.404	296	51.178	38.721	52.525	Araip.Y03N6	0.396
Aradu.GB0MR	0.441	286	69.338	44.251	57.491	Araip.HQ3TB	0.437
Aradu.GB1JK	0.351	634	47.244	39.37	38.268	Araip.4E6MH	0.379
Aradu.GB23E	0.388	1264	52.016	43.241	37.708	Araip.KGX8X	0.382
Aradu.GB4U4	0.379	491	48.78	34.146	35.976	Araip.3JJ8N	0.393
Aradu.GB4V7	0.377	505	54.941	39.723	47.826	Araip.LM2NC	0.368
Aradu.GBG51	0.484	259	55.385	39.615	56.923	Araip.A0QGV	0.484
Aradu.GBM3Q	0.373	715	54.19	39.944	39.944	Araip.96G7D	0.374
Aradu.GC05Y	0.372	826	52.842	33.615	46.433	Araip.FH1C1	0.365
Aradu.GC1Z5	0.443	305	48.039	46.732	59.15	Araip.Q58BJ	0.44
Aradu.GC3CE	0.362	775	46.907	36.211	44.072	Araip.QH6BR	0.358
Aradu.GC5S7	0.423	197	53.03	39.394	46.97	Araip.RJB8C	0.429
Aradu.GC6L4	0.38	278	53.405	27.599	40.86	Araip.13VTA	0.383
Aradu.GD6AN	0.377	464	49.462	38.495	47.527	Araip.T58GE	0.384
Aradu.GDA41	0.346	410	50.365	36.983	42.336	Araip.06FC6	0.351
Aradu.GDX34	0.35	474	51.579	44.211	47.158	Araip.5J60P	0.346
Aradu.GE4T4	0.395	339	46.471	39.706	46.471	Araip.69H3W	0.386
Aradu.GEA65	0.364	147	50	36.486	33.784	Araip.YRZ98	0.377
Aradu.GF1M7	0.394	353	56.215	43.22	46.045	Araip.VCN0P	0.381
Aradu.GFE68	0.382	2747	51.237	38.646	37.191	Araip.Y8HCT	0.378
Aradu.GFS4B	0.325	253	39.764	34.646	42.52	Araip.W5H5G	0.303
Aradu.GFT3R	0.345	258	55.598	40.154	45.174	Araip.T1652	0.369
Aradu.GG5EE	0.37	353	47.458	42.938	45.48	Araip.IA3XR	0.366

Aradu.GG6GL	0.35	1215	40.543	36.678	38.816	Araip.X5428	0.348
Aradu.GGF3N	0.389	1236	54.406	39.208	39.208	Araip.M6ZJI	0.392
Aradu.GGH8Z	0.335	612	54.16	35.563	41.436	Araip.HF3EC	0.332
Aradu.GGI7I	0.374	366	44.687	37.875	40.327	Araip.EV9VN	0.372
Aradu.GGI7X	0.383	257	50.388	39.147	36.822	Araip.U8UTV	0.371
Aradu.GH2K1	0.359	202	48.768	33.99	38.916	Araip.RX20Z	0.349
Aradu.GH306	0.398	180	49.171	48.619	45.856	Araip.Y472I	0.398
Aradu.GH35A	0.473	364	60	31.781	61.918	Araip.MZL3F	0.474
Aradu.GH6V7	0.443	128	50.388	48.837	42.636	Araip.LM9A4	0.459
Aradu.GH8FF	0.341	876	49.715	41.619	37.856	Araip.H1ZVY	0.336
Aradu.GH8LH	0.43	408	53.545	39.609	49.144	Araip.8E1VL	0.435
Aradu.GHE18	0.417	145	51.37	39.041	50.685	Araip.Z2XC8	0.41
Aradu.GHK20	0.388	2276	55.16	39.789	36.276	Araip.ZAK9V	0.384
Aradu.GHV9J	0.357	378	52.77	42.48	31.662	Araip.47BGG	0.345
Aradu.GI0SK	0.367	694	48.777	38.417	32.662	Araip.58R6B	0.37
Aradu.GI6IZ	0.374	555	58.094	41.367	41.906	Araip.1FV4W	0.369
Aradu.GI6UB	0.405	586	57.922	42.249	52.3	Araip.576WD	0.412
Aradu.GIN82	0.335	191	44.792	34.375	49.479	Araip.XVL9X	0.313
Aradu.GIY15	0.372	664	46.165	40.451	45.263	Araip.93AYN	0.374
Aradu.GJ1CE	0.341	803	44.03	38.308	36.194	Araip.7V9BH	0.33
Aradu.GJ5P4	0.363	219	51.818	30	47.273	Araip.P31AE	0.371
Aradu.GJB7M	0.358	863	51.852	39.583	38.542	Araip.EP5TR	0.357
Aradu.GJN8J	0.378	447	50.223	34.821	39.732	Araip.IPX0R	0.368
Aradu.GJY15	0.435	632	42.812	40.758	43.128	Araip.QF4IK	0.437
Aradu.GKD3R	0.334	464	50.968	41.505	37.419	Araip.A03F3	0.325
Aradu.GKD84	0.352	535	55.784	38.06	41.045	Araip.CTQ0H	0.357
Aradu.GKG4B	0.325	390	49.616	43.223	29.923	Araip.N2GVW	0.33
Aradu.GL6NL	0.491	172	50.289	43.931	49.711	Araip.K8PTD	0.491
Aradu.GL73Z	0.388	910	50.055	40.505	39.846	Araip.D9CPB	0.381
Aradu.GL7L0	0.34	326	50.153	34.557	39.144	Araip.3EZ7B	0.339
Aradu.GM25T	0.354	233	58.12	32.051	46.581	Araip.Y359Z	0.356
Aradu.GM5S2	0.33	474	52.421	39.579	35.368	Araip.ZZI8I	0.319
Aradu.GMN03	0.332	345	52.023	40.751	40.751	Araip.29BWP	0.334
Aradu.GMU6R	0.432	375	55.585	41.755	58.511	Araip.A66EM	0.454
Aradu.GN16C	0.34	606	52.389	45.47	35.255	Araip.R99K0	0.346
Aradu.GN2JM	0.354	236	48.101	35.021	44.304	Araip.Q2C6W	0.35
Aradu.GN3K0	0.358	379	50.526	35.263	35.526	Araip.E8TE0	0.356
Aradu.GN4F8	0.382	1572	49.523	40.242	34.139	Araip.RYZ61	0.38
Aradu.GN6RI	0.395	671	46.726	42.857	48.363	Araip.LY6U4	0.392
Aradu.GNT02	0.382	208	53.589	34.928	40.67	Araip.Y5PBQ	0.372
Aradu.GNV5R	0.344	813	48.28	34.521	34.398	Araip.Q4IHU	0.339
Aradu.GP04Q	0.444	574	55.826	42.435	51.304	Araip.N3HNT	0.453
Aradu.GP5WA	0.416	292	51.877	51.536	47.099	Araip.A01I6	0.427
Aradu.GP6HQ	0.407	402	46.402	38.462	43.921	Araip.85R0G	0.395

Aradu.GP6IB	0.372	413	51.449	42.512	45.894	Araip.IUY1V	0.364
Aradu.GPA6U	0.477	410	49.635	42.822	45.255	Araip.60EAB	0.476
Aradu.GPN3U	0.458	928	47.47	38.644	43.703	Araip.F9LX7	0.449
Aradu.GQT7W	0.357	378	43.536	40.369	41.425	Araip.ZZ7ZP	0.345
Aradu.GR4IB	0.391	587	42.517	34.014	43.367	Araip.3S1SV	0.381
Aradu.GR7RL	0.344	308	45.631	43.042	37.864	Araip.STI4D	0.344
Aradu.GR9T1	0.297	387	50.773	38.144	29.639	Araip.CN2DB	0.294
Aradu.GRE2M	0.4	303	42.105	33.224	42.763	Araip.TJ7IP	0.396
Aradu.GS1LC	0.44	477	49.372	40.795	61.297	Araip.FB76I	0.44
Aradu.GS1TU	0.443	171	43.605	44.767	41.279	Araip.JQ8WN	0.449
Aradu.GS270	0.355	261	45.802	37.405	49.618	Araip.18N2T	0.347
Aradu.GS29Q	0.306	416	44.844	39.089	32.854	Araip.31ZB6	0.299
Aradu.GSM33	0.343	514	54.175	36.311	48.155	Araip.M4B28	0.345
Aradu.GT1TJ	0.422	476	52.201	38.994	44.864	Araip.C74GI	0.424
Aradu.GT2DG	0.363	578	54.059	40.242	43.351	Araip.L7TNT	0.367
Aradu.GT5D9	0.377	454	50.769	41.319	49.011	Araip.K8SF0	0.376
Aradu.GT6LC	0.4	995	52.711	41.265	36.948	Araip.S13M1	0.404
Aradu.GT860	0.434	233	58.547	46.581	58.12	Araip.77NJ9	0.436
Aradu.GTI49	0.405	336	52.819	37.092	41.84	Araip.PK5IZ	0.391
Aradu.GTJ2E	0.424	355	50	35.112	44.101	Araip.AL2JW	0.429
Aradu.GTZ9I	0.386	1219	55.492	33.852	42.541	Araip.M1B8E	0.389
Aradu.GU21D	0.381	216	48.848	43.318	37.327	Araip.I2GG6	0.395
Aradu.GUH6T	0.415	860	52.033	32.056	43.206	Araip.Z72XG	0.421
Aradu.GUH8G	0.394	331	59.036	43.072	39.458	Araip.D5QVM	0.394
Aradu.GUU2R	0.411	293	49.66	37.075	44.218	Araip.461HE	0.391
Aradu.GUV6V	0.304	303	49.013	35.855	38.158	Araip.9M2DD	0.307
Aradu.GV115	0.384	141	57.042	47.183	33.803	Araip.XVS2K	0.377
Aradu.GVH4D	0.421	363	57.418	40.659	42.033	Araip.KU884	0.407
Aradu.GVQ3S	0.399	461	49.134	41.991	48.052	Araip.2ES43	0.392
Aradu.GVV0Z	0.415	594	53.109	41.849	48.067	Araip.IN1U1	0.413
Aradu.GWN14	0.369	323	52.16	37.037	43.519	Araip.F49MZ	0.377
Aradu.GX58B	0.385	352	56.941	35.127	41.076	Araip.RX4DL	0.385
Aradu.GX5W4	0.492	334	40	37.91	66.567	Araip.2N7ZX	0.491
Aradu.GX867	0.45	354	49.014	45.634	32.394	Araip.FB68F	0.452
Aradu.GX9A4	0.355	269	46.296	41.111	39.259	Araip.I1FW4	0.352
Aradu.GXJ7L	0.412	164	49.697	42.424	40	Araip.4RI8H	0.406
Aradu.GXP8K	0.381	219	54.545	27.727	35.909	Araip.Y5NRI	0.384
Aradu.GY08R	0.362	312	55.272	39.936	40.256	Araip.72FUN	0.359
Aradu.GY0R3	0.362	215	54.63	50.926	38.426	Araip.GJ5QE	0.345
Aradu.GY8D2	0.448	157	46.835	36.709	61.392	Araip.W1HNL	0.46
Aradu.GZ0CM	0.373	347	55.46	42.241	36.207	Araip.QL2KR	0.369
Aradu.GZ5DT	0.355	900	49.168	35.183	36.737	Araip.HUB63	0.36
Aradu.GZ5P7	0.397	268	45.353	36.431	38.662	Araip.8Z142	0.394
Aradu.GZZ2X	0.395	244	49.388	37.551	45.306	Araip.C2U1E	0.399

Aradu.H03KA	0.453	405	51.724	40.64	51.232	Araip.15H8S	0.457
Aradu.H05SK	0.314	145	46.575	36.986	34.247	Araip.94M4C	0.317
Aradu.H0BW9	0.343	607	44.079	34.211	43.75	Araip.E7PK4	0.344
Aradu.H0G14	0.361	367	53.804	40.217	33.967	Araip.17941	0.377
Aradu.H0HJA	0.407	517	46.332	46.525	50.965	Araip.HB8ML	0.415
Aradu.H0NY1	0.381	262	53.992	33.08	57.414	Araip.EAG6M	0.395
Aradu.H0XA8	0.3	133	33.582	35.821	23.881	Araip.06FXT	0.304
Aradu.H102I	0.354	184	48.649	37.297	43.243	Araip.T6H8P	0.349
Aradu.H128Z	0.38	975	43.852	38.115	40.984	Araip.GMV49	0.381
Aradu.H129B	0.285	257	51.938	32.946	36.434	Araip.135BH	0.293
Aradu.H15D7	0.4	562	56.306	44.227	46.181	Araip.Z2RR8	0.406
Aradu.H1BIK	0.329	545	51.099	41.941	42.125	Araip.0C096	0.341
Aradu.H1FFF	0.403	266	51.685	34.082	43.82	Araip.Q4KH3	0.399
Aradu.H1R88	0.492	200	47.264	39.801	50.249	Araip.SAI7F	0.482
Aradu.H1RA6	0.313	458	50.327	36.166	42.048	Araip.NCY17	0.309
Aradu.H1X77	0.394	515	49.031	37.209	47.481	Araip.ZJ927	0.395
Aradu.H1YN5	0.308	411	41.262	40.534	33.738	Araip.5IP7M	0.332
Aradu.H1ZYW	0.378	344	52.464	31.884	37.101	Araip.QB1CB	0.37
Aradu.H2G5C	0.337	414	43.373	41.205	32.771	Araip.2I21U	0.322
Aradu.H2I13	0.365	297	48.993	37.248	36.577	Araip.FK804	0.357
Aradu.H2K8Y	0.391	171	56.395	37.791	44.186	Araip.3S7QC	0.391
Aradu.H2RVW	0.503	201	57.426	38.119	52.97	Araip.673NH	0.51
Aradu.H2W20	0.393	125	54.762	38.889	37.302	Araip.II7NF	0.41
Aradu.H3050	0.357	567	52.641	31.338	45.599	Araip.JI00S	0.361
Aradu.H306E	0.356	1066	49.11	39.925	33.271	Araip.Q821H	0.373
Aradu.H33XQ	0.345	149	46	42.667	37.333	Araip.AV6R9	0.305
Aradu.H365I	0.401	992	48.338	42.699	41.994	Araip.Z5U4R	0.396
Aradu.H36S4	0.361	184	52.973	45.946	37.838	Araip.37WSW	0.367
Aradu.H3AX1	0.488	171	56.395	44.186	53.488	Araip.6FW03	0.47
Aradu.H3LPD	0.444	222	52.915	46.188	45.74	Araip.RWC6Y	0.433
Aradu.H3P3G	0.403	694	46.619	39.568	35.54	Araip.8XZ82	0.374
Aradu.H3XI7	0.432	670	57.526	42.623	60.06	Araip.PUU5U	0.428
Aradu.H40D4	0.438	368	44.173	46.07	52.033	Araip.CXX9S	0.426
Aradu.H42AE	0.347	823	45.51	42.476	31.675	Araip.54D0C	0.348
Aradu.H447Q	0.441	326	59.633	45.872	54.74	Araip.85BT6	0.431
Aradu.H44CN	0.423	522	44.933	35.182	41.109	Araip.802QV	0.416
Aradu.H46EZ	0.405	318	51.097	37.931	44.514	Araip.JTJ8V	0.399
Aradu.H48T8	0.385	220	50.679	38.462	54.299	Araip.V9UEK	0.388
Aradu.H49AM	0.347	178	48.603	33.52	35.754	Araip.M3BQV	0.349
Aradu.H49WY	0.363	803	51.741	39.179	34.204	Araip.Q7XEQ	0.365
Aradu.H4IQD	0.347	575	46.528	37.326	37.153	Araip.7N7X7	0.357
Aradu.H4MY7	0.401	493	48.381	40.283	41.296	Araip.T6DD9	0.402
Aradu.H4VY0	0.47	248	47.39	41.767	59.036	Araip.2E2K8	0.46
Aradu.H5024	0.488	207	57.212	50.481	45.673	Araip.DR5NH	0.498

Aradu.H50Y7	0.378	319	50.938	37.188	36.25	Araip.F0YLK	0.372
Aradu.H56YA	0.474	532	48.405	44.09	62.852	Araip.VE6EG	0.482
Aradu.H5ED7	0.365	1061	58.004	38.606	47.552	Araip.880AZ	0.375
Aradu.H5EIL	0.322	487	44.672	31.762	42.213	Araip.PB4HL	0.319
Aradu.H5END	0.407	342	54.81	53.061	42.857	Araip.679B1	0.412
Aradu.H5GIM	0.417	298	51.839	44.816	45.151	Araip.TF81A	0.414
Aradu.H5I2N	0.431	164	58.182	40.606	44.848	Araip.60749	0.431
Aradu.H5NQ6	0.451	146	57.143	49.66	44.898	Araip.0U2QD	0.465
Aradu.H6JXR	0.385	870	47.417	40.758	35.476	Araip.RLH2R	0.382
Aradu.H6MX9	0.409	170	47.368	40.936	42.105	Araip.41DGV	0.425
Aradu.H6T1L	0.407	378	52.243	40.633	38.522	Araip.S5E2W	0.404
Aradu.H76RB	0.402	693	55.331	41.066	41.066	Araip.II799	0.399
Aradu.H77WY	0.404	140	52.482	36.17	42.553	Araip.J25TU	0.39
Aradu.H7AQV	0.383	661	52.87	41.843	39.879	Araip.FXZ38	0.383
Aradu.H7GIV	0.395	430	48.724	42.227	46.404	Araip.4DY64	0.412
Aradu.H7KNI	0.375	337	52.367	41.42	42.012	Araip.85TVG	0.372
Aradu.H7VTY	0.437	249	56	46	58	Araip.60GBF	0.424
Aradu.H802Y	0.419	321	51.553	41.925	47.826	Araip.R7R05	0.426
Aradu.H83MI	0.407	375	55.585	40.691	34.574	Araip.DY9AL	0.414
Aradu.H88LD	0.43	581	55.498	45.017	52.062	Araip.5ZJ1R	0.434
Aradu.H88U3	0.431	257	55.039	49.225	46.124	Araip.A7MMQ	0.407
Aradu.H8AL3	0.381	354	50.704	35.493	45.07	Araip.N54GH	0.391
Aradu.H8P6Y	0.383	568	55.36	41.301	35.852	Araip.10EBD	0.379
Aradu.H8Q03	0.346	496	44.869	33.4	36.419	Araip.V86U2	0.338
Aradu.H8Z7K	0.36	308	49.838	32.686	38.188	Araip.9GU4E	0.364
Aradu.H8ZFN	0.349	398	48.872	36.842	35.088	Araip.4A18K	0.369
Aradu.H926S	0.352	865	56.005	39.261	37.298	Araip.YUI9C	0.355
Aradu.H92KN	0.385	248	52.61	47.39	45.382	Araip.90Y7E	0.356
Aradu.H976G	0.394	1151	49.566	46.701	37.413	Araip.V3GZS	0.394
Aradu.H9EEY	0.421	522	54.302	43.212	45.889	Araip.FXS1L	0.429
Aradu.H9EKZ	0.383	194	45.128	40.513	39.487	Araip.L1316	0.356
Aradu.H9FC2	0.403	571	51.923	43.881	44.755	Araip.Y6K7H	0.403
Aradu.H9GMI	0.36	422	48.7	38.534	46.336	Araip.EW35C	0.371
Aradu.H9N2F	0.407	123	45.968	33.871	47.581	Araip.MHA7X	0.378
Aradu.H9NK1	0.364	617	49.191	38.997	41.909	Araip.X5C2D	0.367
Aradu.H9ZXM	0.388	1869	51.711	38.235	36.791	Araip.NF41G	0.389
Aradu.HA4YC	0.344	697	51.433	39.828	44.126	Araip.S6A4N	0.336
Aradu.HA5JM	0.362	230	50.216	38.961	48.485	Araip.CA6VL	0.358
Aradu.HAD5C	0.338	207	52.404	39.423	51.923	Araip.4A1JG	0.338
Aradu.HAF4V	0.433	102	55.34	51.456	43.689	Araip.T14K9	0.464
Aradu.HAL7W	0.376	122	47.154	39.837	39.837	Araip.L9BTW	0.382
Aradu.HB1CW	0.34	331	46.386	35.843	29.819	Araip.KM10P	0.358
Aradu.HBT8X	0.467	204	54.634	40	57.073	Araip.H4G2Q	0.475
Aradu.HC4GG	0.399	304	52.787	44.59	39.344	Araip.MLK6S	0.392

Aradu.HC4I0	0.384	510	52.25	37.965	40.313	Araip.9M15N	0.387
Aradu.HCB3B	0.332	260	47.51	36.398	30.268	Araip.J8I5I	0.333
Aradu.HD19V	0.369	267	52.239	43.284	38.06	Araip.IHD8T	0.373
Aradu.HD203	0.382	333	52.695	32.934	45.21	Araip.VC101	0.372
Aradu.HD6HL	0.393	1086	54.922	39.006	47.838	Araip.445G9	0.396
Aradu.HD7PF	0.32	578	44.56	36.442	38.169	Araip.2A5U1	0.309
Aradu.HE28Y	0.374	349	55.429	38.571	47.429	Araip.4F7TS	0.385
Aradu.HE8A8	0.332	194	50.256	36.923	50.256	Araip.0LC57	0.337
Aradu.HEE23	0.429	415	45.913	41.346	42.067	Araip.7KS0U	0.417
Aradu.HER1A	0.373	481	49.17	51.245	31.743	Araip.PWQ5I	0.37
Aradu.HFI9K	0.413	262	55.133	36.122	51.331	Araip.LL8C3	0.409
Aradu.HG1BY	0.4	182	46.995	39.344	48.634	Araip.C64ZH	0.396
Aradu.HG68U	0.433	343	50.581	41.279	39.826	Araip.C5H4E	0.429
Aradu.HG6CP	0.348	630	52.615	36.767	36.292	Araip.Y9XPY	0.35
Aradu.HG6NA	0.409	430	58.701	46.404	36.659	Araip.46604	0.387
Aradu.HG75V	0.35	529	51.132	34.34	36.038	Araip.RN6JS	0.343
Aradu.HG8ZF	0.418	255	51.172	30.078	41.797	Araip.35NZV	0.418
Aradu.HG92V	0.394	365	49.454	42.35	46.995	Araip.Z54F6	0.397
Aradu.HGJ4G	0.438	170	67.251	38.012	48.538	Araip.0F3YM	0.444
Aradu.HH0H8	0.381	320	51.713	39.875	41.121	Araip.DT8XF	0.395
Aradu.HH10J	0.431	278	49.821	44.444	45.161	Araip.986AT	0.43
Aradu.HH2HB	0.445	678	47.128	46.392	56.554	Araip.K6G0G	0.445
Aradu.HHL1H	0.374	1476	50.643	36.087	39.675	Araip.49HT7	0.37
Aradu.HI40M	0.442	194	51.795	43.59	42.051	Araip.AKP9Z	0.432
Aradu.HIV9Z	0.368	676	49.483	39.882	38.257	Araip.KQ8VJ	0.363
Aradu.HJ002	0.389	619	44.355	37.742	43.387	Araip.T7I1X	0.393
Aradu.HJ2Y0	0.524	334	55.522	49.254	51.343	Araip.EF3DD	0.51
Aradu.HJZ2Z	0.343	281	48.936	39.007	40.426	Araip.S31IS	0.347
Aradu.HK079	0.327	202	51.724	34.483	51.232	Araip.ZN6UI	0.321
Aradu.HK0FS	0.428	227	46.053	42.544	42.105	Araip.2T5Q6	0.419
Aradu.HK1JB	0.411	451	53.761	48.009	41.15	Araip.Q75IR	0.393
Aradu.HK2E0	0.399	338	48.083	36.578	34.808	Araip.J3ZJD	0.399
Aradu.HK2IG	0.436	191	44.792	52.604	56.25	Araip.G9SIR	0.449
Aradu.HK5UD	0.316	447	44.42	32.813	29.464	Araip.HXW9F	0.339
Aradu.HKL0U	0.388	341	48.83	50.292	45.614	Araip.TZP3M	0.394
Aradu.HKL1A	0.436	444	42.247	37.303	42.472	Araip.WE8XB	0.436
Aradu.HKL6E	0.398	993	49.396	42.656	40.644	Araip.1UK4V	0.384
Aradu.HL03T	0.34	642	57.854	43.546	44.79	Araip.0I39J	0.339
Aradu.HL1PG	0.404	843	55.213	46.09	40.64	Araip.K2L1V	0.405
Aradu.HL6TS	0.371	751	56.117	41.356	39.495	Araip.EB319	0.376
Aradu.HL95H	0.401	358	49.861	45.961	36.212	Araip.R4IFK	0.389
Aradu.HLK79	0.392	1577	51.077	40.938	41.381	Araip.X9IXZ	0.393
Aradu.HLR71	0.414	431	49.074	36.111	39.815	Araip.A249Q	0.422
Aradu.HM0P2	0.358	482	47.205	39.13	33.54	Araip.Q3W10	0.351

Aradu.HM1JQ	0.36	397	56.533	39.447	35.678	Araip.YD0N6	0.359
Aradu.HM4L0	0.336	931	44.421	34.013	34.979	Araip.H2P98	0.323
Aradu.HMN77	0.335	382	44.125	32.637	31.593	Araip.615VA	0.34
Aradu.HN2EG	0.374	2190	46.006	37.198	36.924	Araip.BVG2F	0.375
Aradu.HN39D	0.34	581	54.467	39.519	36.082	Araip.58NSN	0.329
Aradu.HN7R4	0.382	106	46.729	43.925	50.467	Araip.B03U9	0.366
Aradu.HNN21	0.334	594	52.437	42.185	38.992	Araip.2G27W	0.337
Aradu.HNS4U	0.366	862	49.479	41.02	40.672	Araip.WL53Y	0.362
Aradu.HNU76	0.323	275	56.884	44.565	37.319	Araip.Y5X21	0.327
Aradu.HNV5C	0.43	456	47.046	35.011	46.827	Araip.LEK47	0.417
Aradu.HP39D	0.386	643	53.727	39.286	56.677	Araip.3230M	0.395
Aradu.HP3G1	0.455	484	56.701	40.206	55.464	Araip.IN00Y	0.451
Aradu.HP9IR	0.377	601	48.007	33.389	39.037	Araip.ZW55J	0.382
Aradu.HP9LA	0.396	972	61.459	49.435	37.102	Araip.Z6T03	0.403
Aradu.HPG6B	0.458	364	49.589	41.644	64.11	Araip.Z9CFM	0.468
Aradu.HQ2V8	0.351	709	52.958	45.211	37.042	Araip.5MK79	0.348
Aradu.HQ5WT	0.434	114	51.304	31.304	44.348	Araip.PU5CI	0.434
Aradu.HQF0H	0.361	1007	45.734	36.607	37.004	Araip.Y6LUD	0.376
Aradu.HQI6K	0.337	600	45.092	37.77	42.429	Araip.W8L59	0.327
Aradu.HR13D	0.438	363	53.297	57.692	38.462	Araip.V4XJQ	0.421
Aradu.HR1QJ	0.423	226	52.423	40.969	56.388	Araip.V284D	0.414
Aradu.HR3NA	0.402	408	51.589	41.076	44.254	Araip.CV4V1	0.397
Aradu.HR4SC	0.401	697	52.149	43.696	40.974	Araip.M1YMA	0.399
Aradu.HRJ8J	0.447	367	55.163	47.826	48.37	Araip.K9XF4	0.454
Aradu.HRR2H	0.357	291	40.753	39.726	36.986	Araip.JZJ9I	0.36
Aradu.HS2F0	0.34	379	49.737	32.632	26.316	Araip.BK7XJ	0.347
Aradu.HS2H7	0.409	953	51.468	40.985	45.597	Araip.V57W5	0.404
Aradu.HS65R	0.4	423	53.066	40.566	45.283	Araip.Y8CYZ	0.401
Aradu.HS8FV	0.384	521	50.192	45.019	42.337	Araip.D1IVY	0.377
Aradu.HSB55	0.377	421	47.156	34.123	44.313	Araip.H11CF	0.377
Aradu.HSJ3A	0.411	228	47.162	36.245	47.162	Araip.Y1ZJ1	0.42
Aradu.HT46D	0.395	454	50.989	41.099	44.396	Araip.UB7JK	0.403
Aradu.HTX30	0.355	265	44.361	36.466	35.338	Araip.H8C7N	0.351
Aradu.HU3I3	0.348	552	41.953	40.145	42.676	Araip.HW618	0.349
Aradu.HU672	0.393	254	50.196	35.294	56.863	Araip.FV4ZU	0.393
Aradu.HU7Y9	0.401	1180	51.566	40.39	38.95	Araip.61HR0	0.402
Aradu.HU8I6	0.418	396	51.385	54.156	34.005	Araip.4C49Y	0.41
Aradu.HU8ZS	0.376	546	55.393	41.682	42.596	Araip.VHP1S	0.385
Aradu.HU94F	0.449	262	59.696	39.924	53.992	Araip.SP2SD	0.452
Aradu.HUV25	0.389	665	48.949	36.787	45.646	Araip.D59I5	0.364
Aradu.HUW75	0.448	430	45.94	46.636	44.548	Araip.C6UMI	0.462
Aradu.HVD76	0.441	678	55.523	44.477	59.205	Araip.KL1K8	0.448
Aradu.HVG4L	0.364	431	54.398	38.426	45.602	Araip.Z9C8C	0.366
Aradu.HVW3A	0.373	411	50.971	38.107	40.291	Araip.467WI	0.368

Aradu.HVZ6R	0.403	1062	51.834	39.605	48.636	Araip.A7M57	0.404
Aradu.HW921	0.325	248	48.193	33.735	39.357	Araip.92STN	0.329
Aradu.HX0A2	0.374	647	47.377	40.741	43.827	Araip.JU5GB	0.373
Aradu.HX36X	0.443	535	63.06	45.336	44.403	Araip.DK4JW	0.447
Aradu.HX9PW	0.379	1179	51.78	34.153	43.729	Araip.MLC5R	0.37
Aradu.HXK3I	0.402	392	47.074	42.748	41.985	Araip.J3620	0.399
Aradu.HY0D1	0.383	970	58.29	45.211	35.633	Araip.58I5B	0.379
Aradu.HY1A1	0.358	165	48.795	34.94	49.398	Araip.GV8BT	0.35
Aradu.HY7QC	0.41	730	54.172	41.04	40.492	Araip.I4YCB	0.409
Aradu.HY9UB	0.381	997	52.705	37.976	42.685	Araip.2B884	0.377
Aradu.HYT44	0.395	309	49.677	42.903	52.581	Araip.K5S58	0.389
Aradu.HZ14S	0.413	333	43.413	40.719	54.491	Araip.9ND7T	0.396
Aradu.HZ16A	0.434	355	49.438	39.045	60.393	Araip.8C7AS	0.426
Aradu.HZ8ES	0.369	400	46.135	36.908	34.414	Araip.N7ZQV	0.36
Aradu.HZ9T9	0.38	223	49.554	41.518	46.875	Araip.KY48S	0.375
Aradu.HZI51	0.382	209	50.952	45.238	44.286	Araip.L2G8B	0.413
Aradu.I01VW	0.384	449	55.111	41.778	38.444	Araip.H55D8	0.397
Aradu.I022X	0.34	322	47.678	39.628	36.223	Araip.KC2C1	0.34
Aradu.I0353	0.381	624	47.68	38.4	41.6	Araip.UV12P	0.381
Aradu.I03LR	0.524	190	58.639	43.455	60.209	Araip.0PV7W	0.524
Aradu.I03VB	0.338	225	42.92	36.283	43.805	Araip.N6CRE	0.382
Aradu.I05PE	0.386	636	52.119	40.345	39.874	Araip.TID01	0.398
Aradu.I0IKB	0.358	808	55.624	39.802	38.566	Araip.65H6H	0.362
Aradu.I0JQ8	0.374	290	45.704	40.893	42.268	Araip.4N45L	0.379
Aradu.I0VP0	0.418	204	51.22	39.024	37.073	Araip.GP5GN	0.403
Aradu.I0WQG	0.41	222	46.188	44.843	44.395	Araip.88RV1	0.408
Aradu.I0X0M	0.368	361	56.077	41.713	35.083	Araip.AH1WN	0.363
Aradu.I0YKW	0.392	146	48.98	45.578	44.898	Araip.8H1P2	0.39
Aradu.I118N	0.334	802	46.7	38.605	42.839	Araip.P1R6Y	0.339
Aradu.I1DFV	0.475	251	50	37.302	61.905	Araip.SH3RA	0.471
Aradu.I1DRB	0.404	370	48.518	36.388	46.092	Araip.6ZN4Z	0.394
Aradu.I1RXA	0.399	431	50	42.361	43.75	Araip.L7Z01	0.389
Aradu.I1SW1	0.4	714	53.147	38.601	34.685	Araip.R7SWT	0.403
Aradu.I1T9D	0.448	326	51.682	44.343	49.235	Araip.V2GHQ	0.432
Aradu.I1U3H	0.325	330	44.713	39.275	41.994	Araip.CB4B3	0.359
Aradu.I23XS	0.382	538	52.505	39.147	41.002	Araip.KRV6R	0.378
Aradu.I24YI	0.455	106	52.336	37.383	56.075	Araip.2I89L	0.446
Aradu.I27F7	0.439	362	50.689	36.915	48.209	Araip.263ND	0.433
Aradu.I29MY	0.44	136	53.285	48.175	62.044	Araip.T5SL7	0.455
Aradu.I2A4P	0.373	137	52.899	41.304	40.58	Araip.X1JSV	0.373
Aradu.I2DKG	0.438	636	55.887	37.677	59.341	Araip.H5Y6J	0.436
Aradu.I2J7R	0.284	173	58.046	43.678	41.954	Araip.RJJ5V	0.284
Aradu.I2L2L	0.37	307	53.571	53.896	35.39	Araip.J8JYF	0.378
Aradu.I2Z9Y	0.311	529	42.075	36.792	35.849	Araip.L96AH	0.308

Aradu.I32AI	0.369	633	50.946	40.063	40.221	Araip.E5UFR	0.361
Aradu.I38Z6	0.401	590	49.069	42.978	37.733	Araip.XTW2R	0.4
Aradu.I3E1J	0.329	304	53.77	40	30.82	Araip.CF8RS	0.315
Aradu.I3I8S	0.354	552	51.175	39.783	39.421	Araip.125MX	0.354
Aradu.I3T1H	0.431	240	50.622	42.739	41.079	Araip.60LL8	0.418
Aradu.I4E8B	0.428	291	52.397	43.151	44.178	Araip.5V8J3	0.437
Aradu.I4L9J	0.399	393	50.761	40.102	35.279	Araip.DF82N	0.403
Aradu.I4MAR	0.439	100	49.505	66.337	58.416	Araip.BQL2U	0.469
Aradu.I4P64	0.331	981	46.029	36.965	36.66	Araip.6RW05	0.327
Aradu.I4SX6	0.353	177	41.011	47.191	42.135	Araip.2NP4Y	0.341
Aradu.I4TMG	0.462	258	53.668	47.876	45.946	Araip.EG329	0.451
Aradu.I4VKL	0.416	430	51.972	40.835	38.979	Araip.8DD0T	0.412
Aradu.I4X46	0.388	487	50.82	40.574	41.393	Araip.DW9H3	0.38
Aradu.I4YNH	0.393	325	51.227	43.252	37.423	Araip.09EYJ	0.384
Aradu.I4Z8N	0.393	321	45.342	37.578	40.373	Araip.K719G	0.392
Aradu.I5672	0.395	600	61.73	45.258	37.77	Araip.8K74T	0.398
Aradu.I567U	0.362	943	50.636	37.288	36.123	Araip.EU7DL	0.364
Aradu.I572Z	0.385	1023	49.609	37.598	41.016	Araip.G99ZR	0.385
Aradu.I59PP	0.406	724	52.276	39.862	33.793	Araip.3X7VM	0.412
Aradu.I5BER	0.332	662	49.623	36.048	36.048	Araip.UNL4G	0.341
Aradu.I5BGE	0.311	381	45.812	36.387	37.173	Araip.WF0FQ	0.346
Aradu.I5BSN	0.405	130	42.748	39.695	50.382	Araip.5XR69	0.41
Aradu.I5ETD	0.399	142	44.056	39.86	37.762	Araip.10KHS	0.381
Aradu.I5LRV	0.383	347	50	34.483	33.621	Araip.3A5RC	0.389
Aradu.I5YUI	0.351	279	50	45	37.857	Araip.PS48V	0.347
Aradu.I609F	0.464	177	53.933	44.944	62.921	Araip.92GX5	0.482
Aradu.I625R	0.449	126	55.906	29.921	56.693	Araip.RSM1K	0.466
Aradu.I66GQ	0.397	324	49.538	42.462	40.615	Araip.I0093	0.396
Aradu.I67GN	0.381	562	53.464	39.432	38.366	Araip.VAX9L	0.364
Aradu.I67U0	0.326	383	47.917	35.417	38.542	Araip.CGW17	0.321
Aradu.I69M4	0.347	177	52.809	35.955	42.135	Araip.ER0KT	0.335
Aradu.I6GSC	0.388	398	47.118	37.845	41.855	Araip.RB0W1	0.385
Aradu.I6I4A	0.465	220	52.489	27.149	48.416	Araip.13WQP	0.47
Aradu.I6JFH	0.356	837	52.745	35.084	36.635	Araip.4BU2D	0.373
Aradu.I71PE	0.35	588	53.48	41.766	43.463	Araip.NH8GX	0.357
Aradu.I72RE	0.467	234	48.936	40.851	40.851	Araip.R5SLN	0.479
Aradu.I73XP	0.411	423	48.585	36.557	47.406	Araip.569RT	0.419
Aradu.I79F7	0.382	496	52.113	40.845	38.229	Araip.4V6B3	0.39
Aradu.I7IYL	0.39	277	51.079	33.094	45.324	Araip.XS400	0.383
Aradu.I7KI9	0.391	580	48.881	41.48	42.857	Araip.R7VSY	0.394
Aradu.I7LA7	0.379	1163	48.024	37.371	39.261	Araip.CX9DW	0.381
Aradu.I7M78	0.375	780	48.912	42.638	33.035	Araip.0Z6G1	0.364
Aradu.I7X6T	0.447	413	45.411	38.647	49.275	Araip.V4857	0.427
Aradu.I83LC	0.341	336	48.961	35.015	41.84	Araip.PG6NQ	0.348

Aradu.I83P6	0.3	219	45	40.455	38.636	Araip.I1LYJ	0.319
Aradu.I88HR	0.373	477	45.397	40.167	41.004	Araip.PN7VH	0.353
Aradu.I8GKQ	0.392	365	48.087	41.257	43.443	Araip.B07N1	0.394
Aradu.I8NTZ	0.382	980	54.944	41.386	39.755	Araip.ZEY7E	0.387
Aradu.I8Q2P	0.393	308	56.958	43.042	37.217	Araip.8Z1AD	0.393
Aradu.I92X3	0.347	552	47.74	42.134	31.646	Araip.L4GEP	0.341
Aradu.I940M	0.421	332	50.15	36.637	47.147	Araip.L12RP	0.408
Aradu.I94L5	0.383	428	58.275	40.559	47.319	Araip.1CV02	0.388
Aradu.I957I	0.374	519	45.192	37.308	41.731	Araip.HF56B	0.377
Aradu.I977L	0.38	838	50.536	43.504	35.995	Araip.2R3UE	0.371
Aradu.I99RK	0.31	204	46.829	41.463	36.098	Araip.QGK0J	0.328
Aradu.I9JU3	0.405	688	48.766	40.058	41.655	Araip.TM0T5	0.404
Aradu.I9N63	0.307	157	43.671	35.443	46.835	Araip.I5UTC	0.301
Aradu.I9PC5	0.415	503	50	43.651	38.69	Araip.BPS80	0.423
Aradu.I9RLC	0.358	425	54.225	42.488	42.019	Araip.RVT5N	0.355
Aradu.I9SPE	0.406	145	52.74	36.986	36.301	Araip.TJS96	0.399
Aradu.I9SXC	0.391	324	50.154	38.154	39.385	Araip.Y6U2J	0.391
Aradu.I9SZF	0.407	419	52.143	46.905	55.476	Araip.YU4RI	0.45
Aradu.I9TTZ	0.413	302	50.495	41.254	35.314	Araip.62IDN	0.393
Aradu.I9U5I	0.448	100	51.485	31.683	52.475	Araip.627JR	0.448
Aradu.I9X8G	0.399	307	47.078	39.286	43.831	Araip.KTN2B	0.394
Aradu.IA5E9	0.374	598	48.915	36.394	42.905	Araip.AP2S4	0.372
Aradu.IAE5L	0.367	389	44.103	38.205	37.436	Araip.Z2B8F	0.368
Aradu.IAV7G	0.435	183	47.283	42.391	51.63	Araip.7JC2C	0.468
Aradu.IB4JW	0.409	880	50.965	34.052	36.436	Araip.7408P	0.409
Aradu.IBQ9G	0.419	578	53.195	42.66	45.769	Araip.I5637	0.412
Aradu.IBY74	0.361	418	47.017	35.322	37.709	Araip.5ZR88	0.374
Aradu.IC00B	0.38	293	50.68	38.435	45.238	Araip.K22F0	0.372
Aradu.IC19J	0.374	640	54.29	37.441	39.002	Araip.I0CB1	0.375
Aradu.IC1L6	0.39	258	49.807	37.066	47.876	Araip.45253	0.38
Aradu.IC2R8	0.34	323	49.383	37.346	36.728	Araip.V4QTU	0.333
Aradu.ICY54	0.466	120	46.281	48.76	54.545	Araip.V7N0F	0.483
Aradu.ID7XN	0.345	897	47.105	34.967	38.085	Araip.U7EGE	0.346
Aradu.ID8H5	0.377	347	52.011	33.046	53.161	Araip.IDR0H	0.387
Aradu.IDJ29	0.362	808	45.983	36.959	35.97	Araip.40FIW	0.357
Aradu.IDN32	0.353	143	46.528	40.278	54.861	Araip.DFY7G	0.348
Aradu.IE711	0.357	137	46.377	39.855	37.681	Araip.76JTT	0.377
Aradu.IEX64	0.354	317	47.17	32.39	39.308	Araip.X2IEW	0.342
Aradu.IF1JM	0.377	1232	56.285	39.74	32.766	Araip.IEW6X	0.381
Aradu.IF4XP	0.405	192	62.176	33.679	57.513	Araip.3269G	0.39
Aradu.IF5Y6	0.335	293	51.361	40.136	35.034	Araip.A1R8U	0.321
Aradu.IFF56	0.448	127	44.531	47.656	57.813	Araip.B5WS3	0.432
Aradu.IFI3Q	0.343	382	49.086	45.431	36.815	Araip.00K86	0.34
Aradu.IFP6S	0.393	380	40.682	42.257	42.52	Araip.EMV9L	0.403

Aradu.IFW71	0.401	301	47.351	39.404	39.073	Araip.W6NP3	0.419
Aradu.IFZ2Q	0.349	472	47.146	37.844	35.729	Araip.NU1G9	0.346
Aradu.IG2QJ	0.423	400	46.384	40.898	57.357	Araip.V90TX	0.432
Aradu.IG70C	0.393	334	51.642	36.119	45.373	Araip.09WK1	0.413
Aradu.IG7LI	0.462	107	58.333	38.889	61.111	Araip.F1QRY	0.462
Aradu.IGJ3I	0.392	410	50.852	48.662	42.579	Araip.2Y8GA	0.392
Aradu.IH0N2	0.416	747	47.861	50.668	42.246	Araip.82GLE	0.419
Aradu.IHD20	0.363	420	42.755	38.48	41.093	Araip.3280G	0.379
Aradu.IHP1V	0.415	122	58.537	37.398	57.724	Araip.EHM8Y	0.408
Aradu.IHZ0W	0.39	251	54.365	42.857	43.254	Araip.W6NII	0.368
Aradu.II4Y3	0.473	131	46.212	55.303	41.667	Araip.0B3H2	0.488
Aradu.II5C0	0.406	206	52.174	37.681	58.937	Araip.8T4WW	0.406
Aradu.IIC03	0.375	320	57.009	44.237	42.991	Araip.TF5MZ	0.365
Aradu.IIT7E	0.377	370	47.439	36.388	40.97	Araip.S698B	0.382
Aradu.IJ0H9	0.361	1353	55.391	40.103	40.694	Araip.8V7F0	0.361
Aradu.IJ1L1	0.36	911	51.316	36.623	35.197	Araip.72BD2	0.357
Aradu.IJM7H	0.439	174	39.429	35.429	66.857	Araip.L6VN3	0.452
Aradu.IL9UG	0.353	454	43.736	35.385	40.879	Araip.7E4DK	0.363
Aradu.IL9WG	0.356	350	53.846	34.758	45.014	Araip.1UW8I	0.358
Aradu.ILB9Z	0.383	312	51.118	30.671	51.438	Araip.P6KBN	0.38
Aradu.ILC9H	0.349	397	48.241	38.693	38.945	Araip.71XEY	0.348
Aradu.ILS90	0.47	134	51.852	45.185	34.074	Araip.XZ2LF	0.447
Aradu.ILV24	0.391	113	64.912	43.86	46.491	Araip.57S0G	0.409
Aradu.ILZ3Q	0.366	125	48.413	42.063	30.159	Araip.4B1V4	0.396
Aradu.IM4U5	0.39	342	47.813	43.44	44.898	Araip.KGP2Q	0.389
Aradu.IM5S3	0.429	391	47.194	45.153	39.541	Araip.0FB59	0.426
Aradu.IM707	0.351	751	48.936	38.032	38.032	Araip.V9W0G	0.354
Aradu.IM8LC	0.396	478	50.313	36.326	40.919	Araip.X7VZI	0.389
Aradu.IM9AT	0.43	505	53.755	34.387	65.415	Araip.M5K02	0.428
Aradu.IMH3N	0.374	185	44.086	32.796	43.011	Araip.00WVB	0.374
Aradu.IN2S6	0.326	325	50.307	46.626	36.81	Araip.H6TT6	0.33
Aradu.IN3UA	0.337	173	53.448	55.747	55.172	Araip.KRL84	0.337
Aradu.INH3Y	0.4	476	50.943	40.881	39.413	Araip.3JN5Z	0.398
Aradu.INV9V	0.34	164	55.152	38.788	37.576	Araip.F5D2P	0.346
Aradu.INW8Z	0.363	401	50.995	31.841	52.488	Araip.R1BCW	0.373
Aradu.IP5K7	0.399	371	55.645	33.333	45.43	Araip.IL3I2	0.399
Aradu.IPN47	0.377	1164	47.124	37.768	40.258	Araip.D4SGT	0.374
Aradu.IPP1D	0.471	141	48.592	45.07	40.845	Araip.PQA29	0.44
Aradu.IQ8X9	0.387	196	52.792	35.533	46.193	Araip.FU0RV	0.366
Aradu.IQK2Q	0.422	418	58.234	41.289	45.346	Araip.H09FU	0.431
Aradu.IQS3D	0.423	218	49.772	47.945	49.772	Araip.I99X3	0.413
Aradu.IR00B	0.347	371	49.194	39.785	42.473	Araip.U4GR2	0.341
Aradu.IR1JZ	0.355	597	53.679	41.137	41.973	Araip.0U07W	0.358
Aradu.IR2D4	0.373	321	47.826	44.41	45.031	Araip.97FYI	0.369

Aradu.IR41I	0.429	361	56.354	37.569	38.122	Araip.LE6XA	0.438
Aradu.IRG5E	0.421	171	52.907	52.907	55.233	Araip.9R7P2	0.427
Aradu.IRK1D	0.369	729	53.973	40.137	37.808	Araip.FV8HT	0.374
Aradu.IRQ7V	0.4	504	53.861	39.604	50.495	Araip.SYI2E	0.4
Aradu.IS3BT	0.316	580	52.151	43.89	32.53	Araip.Q0RIX	0.327
Aradu.IS5GH	0.436	436	57.208	45.309	62.014	Araip.ISU30	0.46
Aradu.IS5YT	0.45	753	54.111	46.021	40.981	Araip.QYZ6U	0.451
Aradu.IS6MU	0.413	431	50	40.741	42.361	Araip.3Z6CU	0.425
Aradu.IS9ZH	0.395	539	48.148	39.074	42.593	Araip.99NVB	0.387
Aradu.ISD6F	0.409	631	46.203	36.551	45.095	Araip.SCJ6B	0.407
Aradu.ISN1L	0.381	323	44.136	48.765	40.432	Araip.1V5ZI	0.389
Aradu.ISN4P	0.367	711	51.124	40.73	40.73	Araip.IY0UZ	0.364
Aradu.IT4JF	0.403	426	49.415	41.452	33.724	Araip.ER2KN	0.391
Aradu.IT5KP	0.33	277	49.281	35.252	41.007	Araip.KBS8N	0.331
Aradu.ITR9J	0.325	157	46.203	34.177	34.177	Araip.HCQ42	0.331
Aradu.IU0EI	0.423	546	56.307	37.112	42.962	Araip.KG3H5	0.421
Aradu.IU1HH	0.396	695	51.437	39.511	46.264	Araip.GM19P	0.391
Aradu.IUI2U	0.346	200	52.736	37.811	47.761	Araip.FW1RB	0.33
Aradu.IV1W2	0.368	266	41.948	54.307	41.573	Araip.6ZC3D	0.36
Aradu.IV48P	0.368	211	47.642	44.34	36.321	Araip.G72RX	0.347
Aradu.IV87Z	0.446	591	51.52	45.608	50.676	Araip.B0DA9	0.439
Aradu.IV8SJ	0.485	249	47.2	51.2	47.2	Araip.C6CF4	0.468
Aradu.IVB9H	0.353	141	54.93	34.507	43.662	Araip.706JC	0.349
Aradu.IVC5J	0.351	241	51.653	38.843	37.603	Araip.B9DG2	0.352
Aradu.IVE8F	0.349	862	52.26	40.904	32.213	Araip.M2UTN	0.338
Aradu.IW0ES	0.431	376	59.416	51.459	44.562	Araip.W6LGW	0.438
Aradu.IW3DF	0.378	1040	49.472	41.499	45.629	Araip.M7HZK	0.381
Aradu.IW3RC	0.431	580	51.635	43.373	55.077	Araip.K0Q5S	0.43
Aradu.IW8J6	0.375	430	53.132	41.531	38.747	Araip.12A3Y	0.379
Aradu.IW9VR	0.428	373	53.743	43.316	49.198	Araip.781N3	0.43
Aradu.IWK4F	0.453	425	46.009	43.427	52.582	Araip.1C7B4	0.465
Aradu.IWK7R	0.395	884	56.61	42.147	39.774	Araip.5Z0QI	0.401
Aradu.IWR1X	0.425	374	53.6	40.533	49.6	Araip.AE0WJ	0.417
Aradu.IX4YQ	0.389	242	47.325	34.979	43.621	Araip.JQ4TL	0.375
Aradu.IXF4G	0.378	160	55.901	36.646	30.435	Araip.U880P	0.391
Aradu.IXS5D	0.328	253	54.331	37.795	35.039	Araip.K8WAH	0.332
Aradu.IY69R	0.476	322	63.777	40.867	77.09	Araip.T5402	0.481
Aradu.IY93L	0.381	503	58.135	40.873	46.627	Araip.21TG8	0.38
Aradu.IYG99	0.399	441	57.919	49.774	42.308	Araip.ZSL68	0.392
Aradu.IYZ29	0.406	487	50.615	46.516	41.598	Araip.V3SRF	0.418
Aradu.IZ0B7	0.365	1014	44.828	35.369	40.296	Araip.W8764	0.373
Aradu.IZ11Y	0.431	295	55.068	40.878	45.27	Araip.BNQ5K	0.431
Aradu.IZ2A8	0.35	282	51.59	41.696	34.982	Araip.0TX9X	0.343
Aradu.IZ4SH	0.363	792	46.406	39.344	37.831	Araip.33UB6	0.363

Aradu.IZ7V8	0.344	295	44.257	38.176	40.878	Araip.XEF5B	0.341
Aradu.IZQ3Z	0.414	315	53.165	33.861	40.19	Araip.Z0Q6Q	0.444
Aradu.IZX1C	0.507	276	59.928	50.542	73.285	Araip.F8GND	0.511
Aradu.J0689	0.39	164	47.273	40	45.455	Araip.R8A8Y	0.396
Aradu.J06JT	0.433	332	57.658	46.847	42.943	Araip.Z17TF	0.422
Aradu.J08SD	0.442	365	59.29	42.35	57.377	Araip.K4G1H	0.442
Aradu.J090Y	0.389	563	52.305	36.525	38.83	Araip.6C47W	0.392
Aradu.J09BS	0.358	883	42.308	38.235	34.502	Araip.1C776	0.35
Aradu.J0FTC	0.351	237	54.202	36.555	37.815	Araip.UI4QL	0.352
Aradu.J0PRI	0.379	1271	54.481	40.173	34.355	Araip.Z08PZ	0.376
Aradu.J0SGA	0.344	593	52.02	34.68	37.71	Araip.19T2H	0.339
Aradu.J0ZDG	0.359	359	47.5	51.111	41.389	Araip.3W093	0.367
Aradu.J16T3	0.503	328	57.447	47.416	46.809	Araip.E7852	0.461
Aradu.J1AYY	0.517	336	51.632	39.466	56.973	Araip.4K0TW	0.502
Aradu.J1D71	0.386	295	45.27	36.149	33.784	Araip.26W3E	0.374
Aradu.J1G6I	0.342	462	47.3	43.197	44.06	Araip.4B1GU	0.341
Aradu.J1JIJ	0.323	325	42.638	36.196	39.571	Araip.1G19U	0.324
Aradu.J1JQ8	0.554	615	45.13	47.078	67.045	Araip.AB8FX	0.558
Aradu.J1M1P	0.471	785	51.399	43.893	60.305	Araip.UU70G	0.462
Aradu.J1S8L	0.373	798	50.563	39.424	34.043	Araip.92A2E	0.367
Aradu.J1S90	0.375	645	50	40.248	54.025	Araip.94W62	0.374
Aradu.J1Y0V	0.403	262	51.331	42.205	58.935	Araip.I7WTL	0.427
Aradu.J1ZY0	0.414	370	48.518	34.232	41.779	Araip.09YU8	0.403
Aradu.J21EA	0.386	365	51.366	43.989	35.792	Araip.S909G	0.388
Aradu.J25NN	0.377	549	49.091	41.273	41.818	Araip.86EDZ	0.371
Aradu.J296J	0.348	528	47.826	34.026	36.673	Araip.7B1XA	0.335
Aradu.J2BV7	0.36	696	50.359	39.455	36.729	Araip.F77E0	0.359
Aradu.J2H81	0.389	869	45.057	42.874	39.77	Araip.Q1Z2E	0.39
Aradu.J2HBF	0.409	506	59.566	39.448	38.856	Araip.PHT1P	0.4
Aradu.J2RZP	0.422	889	50.112	37.865	44.607	Araip.RT5CP	0.428
Aradu.J2YIY	0.429	444	46.966	38.427	51.91	Araip.A561Y	0.428
Aradu.J31ZQ	0.408	174	50.286	43.429	73.714	Araip.VH0NK	0.406
Aradu.J32IT	0.416	264	56.226	45.283	38.868	Araip.16EI2	0.408
Aradu.J355A	0.361	126	45.669	37.008	40.945	Araip.N9WJ3	0.361
Aradu.J364A	0.416	516	53.578	44.874	38.491	Araip.GZD9C	0.413
Aradu.J3B7D	0.398	255	48.828	46.094	45.703	Araip.5SR4K	0.394
Aradu.J3FIC	0.454	418	52.029	38.425	64.439	Araip.37TH3	0.456
Aradu.J3H3Z	0.404	319	48.125	45.625	50.313	Araip.K8LVP	0.399
Aradu.J3IKA	0.4	136	62.044	43.796	41.606	Araip.26IG1	0.408
Aradu.J3LFY	0.418	139	58.571	31.429	45	Araip.04TGY	0.396
Aradu.J3P01	0.384	1151	55.816	45.052	38.455	Araip.YIX8M	0.383
Aradu.J3SWW	0.456	283	55.634	36.972	61.62	Araip.92A9S	0.449
Aradu.J43S7	0.437	441	49.321	49.774	53.846	Araip.2P2KT	0.441
Aradu.J486B	0.498	333	53.593	41.916	50.599	Araip.LXT0U	0.497

Aradu.J4C77	0.433	337	54.438	37.574	51.183	Araip.14VDU	0.426
Aradu.J4D1T	0.379	581	52.062	43.471	43.127	Araip.6I0UC	0.377
Aradu.J4FDW	0.384	1235	52.023	39.563	33.414	Araip.SMW5E	0.381
Aradu.J4IUU	0.374	215	44.907	42.593	37.5	Araip.64KG6	0.382
Aradu.J4JB7	0.371	454	54.286	36.264	36.923	Araip.02Z8U	0.375
Aradu.J4R4W	0.364	736	53.46	40.299	39.891	Araip.11KLU	0.358
Aradu.J4SMU	0.373	445	42.825	40.135	48.879	Araip.ZEC5D	0.378
Aradu.J4VEH	0.344	265	47.368	60.902	31.203	Araip.553HN	0.332
Aradu.J4XWG	0.364	601	54.485	36.545	42.027	Araip.HJ37G	0.363
Aradu.J4Y5T	0.493	154	49.032	38.71	43.871	Araip.V1FQK	0.486
Aradu.J4Z1M	0.391	342	48.98	44.606	49.854	Araip.2T4WR	0.388
Aradu.J502L	0.347	157	49.367	36.709	43.671	Araip.T4LH3	0.327
Aradu.J51N4	0.397	284	49.123	35.088	38.246	Araip.SJY4S	0.386
Aradu.J5692	0.393	605	44.554	36.964	41.419	Araip.82HK7	0.401
Aradu.J59GH	0.367	961	49.584	38.773	37.11	Araip.P3VNM	0.373
Aradu.J5AA1	0.363	336	54.599	33.531	36.499	Araip.I0EMG	0.362
Aradu.J5AII	0.41	465	57.082	35.622	44.635	Araip.B6VA3	0.413
Aradu.J5BIS	0.4	1138	52.766	47.322	33.977	Araip.689G8	0.394
Aradu.J5HFQ	0.414	460	55.531	42.95	59.219	Araip.K9WKL	0.402
Aradu.J5HIY	0.45	332	57.057	41.141	41.141	Araip.CW34G	0.431
Aradu.J5HSK	0.416	230	60.606	29.004	47.186	Araip.WKJ1H	0.42
Aradu.J5WE7	0.391	323	56.79	34.259	50.926	Araip.84KVA	0.396
Aradu.J5XVH	0.366	335	47.321	32.738	33.631	Araip.CI752	0.38
Aradu.J60UE	0.351	215	48.148	41.667	36.574	Araip.J4ZFW	0.355
Aradu.J67TY	0.4	452	48.124	38.852	46.358	Araip.QK072	0.409
Aradu.J6DQU	0.329	169	52.353	35.882	39.412	Araip.E938G	0.331
Aradu.J6F5H	0.363	389	49.231	40	34.359	Araip.X8YYD	0.357
Aradu.J6GHV	0.406	390	56.266	48.593	40.665	Araip.PKX1N	0.397
Aradu.J6I78	0.369	881	43.878	43.991	35.261	Araip.ZK9JP	0.366
Aradu.J6MLG	0.368	302	51.155	46.205	40.264	Araip.TL7VJ	0.364
Aradu.J6P55	0.422	538	47.495	38.961	47.124	Araip.ML7TB	0.412
Aradu.J72N3	0.369	232	43.348	32.189	20.601	Araip.83T6W	0.327
Aradu.J79ME	0.333	571	46.853	39.51	37.413	Araip.FM1J9	0.343
Aradu.J7CRS	0.338	266	57.303	39.7	41.199	Araip.970Q7	0.376
Aradu.J7CY2	0.364	234	43.404	31.064	38.723	Araip.32X6B	0.358
Aradu.J7DHV	0.387	942	50.053	38.706	42.842	Araip.I4GCK	0.378
Aradu.J7EAE	0.394	472	57.082	42.072	34.672	Araip.KG5RF	0.393
Aradu.J7JS6	0.39	366	58.038	44.687	51.771	Araip.87JV8	0.378
Aradu.J7KPQ	0.391	924	51.459	40	49.081	Araip.HQ9VR	0.387
Aradu.J7LHV	0.36	987	49.899	42.51	38.765	Araip.69TNI	0.351
Aradu.J7XF6	0.358	824	50.303	37.455	33.939	Araip.N118V	0.36
Aradu.J807R	0.366	752	56.972	37.583	42.895	Araip.T4UZH	0.362
Aradu.J80PY	0.36	464	43.656	45.806	37.634	Araip.807VL	0.354
Aradu.J85DC	0.34	514	48.155	34.563	38.252	Araip.K6VWA	0.339

Aradu.J8H2F	0.406	400	48.13	51.621	47.382	Araip.EL37U	0.413
Aradu.J8L3N	0.361	423	47.877	39.623	39.151	Araip.GTG68	0.346
Aradu.J8MWU	0.399	377	51.323	39.947	41.799	Araip.VR4Q2	0.408
Aradu.J8VZJ	0.328	656	44.292	41.705	29.224	Araip.RZ2UN	0.333
Aradu.J967R	0.35	352	42.776	42.493	40.51	Araip.TFG1J	0.35
Aradu.J9ALL	0.362	937	50.64	33.475	41.684	Araip.S8LZD	0.358
Aradu.J9D9F	0.342	479	44.583	31.667	36.667	Araip.3J4UV	0.325
Aradu.J9KV2	0.4	423	50.943	43.868	51.179	Araip.IK2R0	0.411
Aradu.J9V4P	0.396	503	53.968	34.325	55.159	Araip.B1UDK	0.398
Aradu.JA2NX	0.378	616	47.488	39.222	44.408	Araip.K776G	0.379
Aradu.JA3L7	0.353	474	41.684	42.526	33.053	Araip.X2NAH	0.357
Aradu.JAD6J	0.316	498	46.894	39.88	39.679	Araip.I269H	0.32
Aradu.JAF9B	0.457	474	47.158	41.474	62.105	Araip.X26F2	0.46
Aradu.JAT8P	0.379	489	50.816	37.347	36.531	Araip.T0MH3	0.39
Aradu.JAV3X	0.351	443	44.144	52.703	42.342	Araip.J8RG7	0.359
Aradu.JB051	0.395	226	50.22	40.529	39.648	Araip.HY7X8	0.39
Aradu.JB8ME	0.346	362	44.353	29.752	29.752	Araip.Z39M8	0.349
Aradu.JBH10	0.37	266	50.936	41.948	34.457	Araip.MP4SQ	0.359
Aradu.JBU5E	0.384	365	54.918	42.896	38.798	Araip.4P1DQ	0.389
Aradu.JC2LL	0.409	539	48.889	38.519	40.37	Araip.NUV8F	0.4
Aradu.JC32L	0.37	531	49.624	40.038	33.835	Araip.UXF7P	0.371
Aradu.JC4ID	0.431	369	56.486	44.054	48.919	Araip.C58L0	0.456
Aradu.JC6IN	0.435	244	59.592	38.776	63.673	Araip.38XYU	0.441
Aradu.JCH8S	0.345	598	48.581	37.73	40.401	Araip.BT7Z7	0.34
Aradu.JF030	0.402	580	50.947	38.21	47.332	Araip.7G62B	0.407
Aradu.JF3DE	0.41	241	50.413	42.149	39.256	Araip.PJ16E	0.406
Aradu.JF3WA	0.37	948	55.743	34.352	39.41	Araip.K79R5	0.365
Aradu.JF9VE	0.366	375	53.191	42.553	39.628	Araip.PFH2D	0.365
Aradu.JFA7C	0.366	211	45.283	39.623	41.981	Araip.JQ4T7	0.386
Aradu.JFD4U	0.398	459	55.435	33.043	34.783	Araip.QW4F4	0.395
Aradu.JFD76	0.372	498	48.898	35.07	39.078	Araip.V2JWX	0.369
Aradu.JFH3E	0.34	459	54.348	40.652	39.348	Araip.M52X2	0.348
Aradu.JFR2C	0.377	2007	48.805	39.94	34.462	Araip.F9RU1	0.38
Aradu.JG113	0.444	341	51.17	40.936	54.094	Araip.37V98	0.395
Aradu.JG217	0.401	923	50.866	43.831	48.918	Araip.TL0AY	0.403
Aradu.JG252	0.433	686	57.787	40.902	43.523	Araip.SCN43	0.437
Aradu.JG7ZE	0.34	488	50.92	35.174	40.491	Araip.GN34D	0.344
Aradu.JG94E	0.364	478	49.269	38.413	41.336	Araip.JN8FM	0.385
Aradu.JGE04	0.337	360	50.416	44.321	35.18	Araip.QE1HM	0.338
Aradu.JGT0L	0.288	115	47.414	43.103	40.517	Araip.KEX5D	0.28
Aradu.JH3GC	0.322	538	46.568	35.436	32.096	Araip.UA42P	0.327
Aradu.JH5V3	0.424	274	50.545	28.727	37.455	Araip.4V07C	0.424
Aradu.JI08P	0.341	915	44.323	36.681	32.096	Araip.Y4YUV	0.334
Aradu.JI3DJ	0.39	111	52.679	29.464	38.393	Araip.E5BJJ	0.385

Aradu.JI62L	0.345	154	54.839	35.484	35.484	Araip.RM081	0.319
Aradu.JJ0LZ	0.42	359	53.889	43.056	49.722	Araip.G085N	0.423
Aradu.JJ2FT	0.388	323	59.877	42.593	34.568	Araip.NC1F1	0.394
Aradu.JJ88W	0.342	1220	49.386	40.95	32.678	Araip.F0Y7Z	0.351
Aradu.JJA0L	0.496	384	49.351	43.636	52.727	Araip.K3GK4	0.488
Aradu.JJG2Q	0.378	977	46.933	38.037	38.241	Araip.J2KA8	0.363
Aradu.JJQ1T	0.402	494	55.758	45.657	42.828	Araip.62AFJ	0.395
Aradu.JJU8Z	0.336	924	42.595	34.919	37.405	Araip.C9NM5	0.339
Aradu.JJW6C	0.41	265	48.872	40.602	44.737	Araip.X4N9F	0.418
Aradu.JJY9M	0.399	445	52.242	36.996	49.552	Araip.564H9	0.382
Aradu.JK0W0	0.369	449	44	41.778	42.222	Araip.123NR	0.369
Aradu.JK14X	0.549	151	48.026	61.842	54.605	Araip.F7XDB	0.566
Aradu.JK25B	0.349	184	42.703	56.216	31.892	Araip.YD78C	0.385
Aradu.JK5YQ	0.398	1159	54.828	42.414	47.155	Araip.D13ZN	0.398
Aradu.JK78S	0.367	344	44.058	40.29	38.841	Araip.F2E7A	0.376
Aradu.JKI99	0.404	564	53.274	46.903	42.478	Araip.EZE10	0.401
Aradu.JKK33	0.328	204	53.171	35.122	34.634	Araip.Y9WQJ	0.343
Aradu.JKM1G	0.388	138	50.36	45.324	41.727	Araip.X255Z	0.418
Aradu.JL0B3	0.345	393	53.299	37.056	41.117	Araip.ZI6SJ	0.362
Aradu.JL69F	0.307	613	43.811	33.713	32.899	Araip.XLK0H	0.306
Aradu.JL8FJ	0.352	706	46.11	40.877	41.726	Araip.DGW0S	0.35
Aradu.JL9ML	0.351	504	58.812	44.95	51.881	Araip.V2L0E	0.338
Aradu.JLM18	0.42	495	53.831	42.137	47.581	Araip.AK3ZS	0.418
Aradu.JLN1Z	0.364	545	47.07	39.744	32.784	Araip.4A7P4	0.372
Aradu.JLT7Z	0.481	558	54.025	43.113	63.685	Araip.E86GN	0.486
Aradu.JLY8Q	0.382	361	50.276	48.619	46.409	Araip.9V8AS	0.374
Aradu.JM0WC	0.347	123	42.742	32.258	69.355	Araip.L6Z75	0.347
Aradu.JM0ZT	0.362	525	47.148	42.205	39.163	Araip.EI4LW	0.352
Aradu.JM1K2	0.467	392	54.453	53.69	52.672	Araip.06TYZ	0.463
Aradu.JM9X6	0.424	164	49.697	39.394	48.485	Araip.8FI20	0.433
Aradu.JMD12	0.393	442	49.436	36.343	42.664	Araip.U29VB	0.397
Aradu.JMQ30	0.364	419	48.571	34.048	50.476	Araip.J60GN	0.371
Aradu.JMT41	0.483	461	47.619	35.714	61.039	Araip.RLC71	0.476
Aradu.JMV7E	0.436	164	59.394	47.273	42.424	Araip.P7ZIQ	0.429
Aradu.JMZ0B	0.489	242	59.259	46.091	73.251	Araip.WUY3S	0.509
Aradu.JN158	0.371	1245	46.228	33.307	37.56	Araip.I06X7	0.378
Aradu.JN5HH	0.338	380	50.919	40.42	39.633	Araip.1KL0I	0.335
Aradu.JN9TL	0.403	841	56.176	46.437	37.055	Araip.M301B	0.401
Aradu.JP0ZJ	0.382	597	44.649	38.629	44.816	Araip.HUI6H	0.395
Aradu.JP5P4	0.305	515	38.566	33.14	39.535	Araip.TG0L1	0.31
Aradu.JPJ0M	0.423	757	54.617	37.863	51.583	Araip.H3L98	0.447
Aradu.JQ0FN	0.358	113	35.965	55.263	42.105	Araip.DN0QK	0.349
Aradu.JQ4B3	0.3	137	43.478	23.913	47.826	Araip.2H2XV	0.292
Aradu.JQ7DF	0.297	315	38.291	31.329	34.177	Araip.U25LN	0.338

Aradu.JQ7JG	0.351	464	52.903	41.505	38.28	Araip.P8SRT	0.353
Aradu.JQ8WR	0.357	603	45.695	42.384	40.232	Araip.026EM	0.371
Aradu.JQC6T	0.458	159	59.375	48.125	39.375	Araip.9YL2S	0.451
Aradu.JQD1M	0.394	867	48.618	38.594	49.539	Araip.PY0XX	0.377
Aradu.JQY29	0.404	415	64.183	48.558	44.231	Araip.40GWN	0.392
Aradu.JR4VV	0.323	281	48.582	38.652	34.752	Araip.H5UU3	0.323
Aradu.JR8JR	0.341	661	56.193	40.937	42.749	Araip.4SN9W	0.336
Aradu.JR9Q4	0.376	186	56.15	36.898	48.128	Araip.E6H2Y	0.363
Aradu.JR9SL	0.409	772	52.135	44.502	44.502	Araip.XJP9J	0.413
Aradu.JRR3K	0.313	167	50	50	46.429	Araip.X2DNI	0.319
Aradu.JRV68	0.3	227	39.912	35.088	39.912	Araip.S5KEZ	0.3
Aradu.JS0R2	0.389	184	57.838	36.757	44.865	Araip.6ZZ85	0.389
Aradu.JS1AI	0.383	261	40.84	32.061	43.511	Araip.BV9QU	0.383
Aradu.JS1QQ	0.329	1024	43.61	37.171	36.098	Araip.ZY14U	0.33
Aradu.JS2XW	0.474	491	52.033	46.341	46.951	Araip.FJG3C	0.464
Aradu.JSQ7J	0.386	542	50.829	39.411	44.015	Araip.FEQ3S	0.394
Aradu.JT1JK	0.371	556	50.628	43.986	47.576	Araip.22PIW	0.376
Aradu.JT1K4	0.412	526	49.526	39.279	56.167	Araip.D3EYV	0.412
Aradu.JT6Z2	0.496	126	43.307	33.071	67.717	Araip.4UD0W	0.504
Aradu.JT96L	0.406	539	52.778	38.519	53.519	Araip.67K5R	0.41
Aradu.JTA11	0.37	421	48.815	33.175	49.289	Araip.F7384	0.342
Aradu.JTL52	0.354	970	44.593	39.341	36.766	Araip.HVP05	0.343
Aradu.JU5TS	0.416	158	45.912	44.025	37.107	Araip.VY1XN	0.436
Aradu.JU778	0.368	235	44.492	48.729	30.508	Araip.IIL5I	0.386
Aradu.JU8EK	0.409	506	52.465	44.773	46.746	Araip.9W1BE	0.4
Aradu.JU9J9	0.377	309	46.774	38.065	43.548	Araip.9KL4T	0.379
Aradu.JUW7B	0.497	154	52.258	41.935	51.613	Araip.K56MF	0.491
Aradu.JV2C7	0.331	145	50.685	39.041	32.192	Araip.CP6YA	0.324
Aradu.JV441	0.381	337	52.071	38.166	42.899	Araip.1Y87C	0.372
Aradu.JV5EQ	0.478	273	57.664	45.985	68.248	Araip.DU3BQ	0.466
Aradu.JV5YL	0.355	229	53.478	51.739	33.043	Araip.GTR8C	0.357
Aradu.JV6V7	0.353	227	51.754	40.789	35.965	Araip.Y8ZSK	0.367
Aradu.JV8RS	0.468	327	53.354	41.768	55.488	Araip.59ZNY	0.468
Aradu.JVB3U	0.434	429	53.721	38.837	43.023	Araip.JX0VW	0.44
Aradu.JVL5D	0.398	305	51.634	39.869	40.85	Araip.44PA5	0.413
Aradu.JVP4N	0.417	268	53.16	46.468	43.123	Araip.1LL57	0.412
Aradu.JW08T	0.315	484	48.247	41.031	33.402	Araip.V9EKQ	0.308
Aradu.JW82A	0.455	802	56.04	34.994	53.923	Araip.PGB0K	0.452
Aradu.JWG0R	0.36	309	42.258	47.419	39.355	Araip.HRN64	0.36
Aradu.JWM4N	0.375	589	53.39	37.797	40.678	Araip.L153Y	0.366
Aradu.JXB2I	0.44	259	49.615	35.769	57.692	Araip.LC3UN	0.444
Aradu.JY1UA	0.388	351	46.307	40.625	41.477	Araip.Y533V	0.384
Aradu.JY8JV	0.356	458	57.952	34.205	42.484	Araip.RP235	0.364
Aradu.JY9SV	0.348	697	52.292	35.244	49.857	Araip.57ZZX	0.356

Aradu.JYC3Z	0.348	625	57.348	47.444	34.665	Araip.8K2W2	0.339
Aradu.JZ25F	0.413	548	52.277	40.437	47.177	Araip.23QAA	0.413
Aradu.JZ4PA	0.342	782	52.49	42.018	35.121	Araip.BR3MH	0.341
Aradu.JZ8MY	0.405	545	48.535	41.026	44.689	Araip.N74EU	0.408
Aradu.JZI09	0.402	830	47.774	43.442	39.952	Araip.1JL4U	0.399
Aradu.JZR6I	0.469	196	51.777	63.452	49.239	Araip.0K8ZX	0.485
Aradu.JZT13	0.356	853	51.171	38.525	43.208	Araip.GGW4P	0.355
Aradu.JZT16	0.413	253	48.031	51.575	50.394	Araip.E30MW	0.409
Aradu.K00WP	0.374	220	45.249	34.389	46.154	Araip.UVF95	0.394
Aradu.K05BK	0.384	391	52.551	39.796	38.01	Araip.71Z4W	0.395
Aradu.K087N	0.421	407	44.363	46.078	42.402	Araip.78HJ7	0.429
Aradu.K08LS	0.353	501	54.382	44.024	35.458	Araip.JRK98	0.343
Aradu.K0BKL	0.373	263	44.318	34.47	45.833	Araip.7942Z	0.383
Aradu.K0D8U	0.423	720	55.479	45.354	55.34	Araip.29WB6	0.431
Aradu.K0FA0	0.377	502	49.901	38.569	34.195	Araip.MQ9VE	0.393
Aradu.K0HFM	0.332	212	50.704	44.601	39.437	Araip.DI07C	0.327
Aradu.K0L5G	0.349	388	45.501	40.36	36.504	Araip.PN2Q6	0.359
Aradu.K0SK7	0.379	566	48.854	40.212	40.035	Araip.BR9B7	0.378
Aradu.K0SUT	0.362	387	48.454	37.887	41.237	Araip.76PXL	0.358
Aradu.K10TZ	0.394	587	43.537	41.497	42.687	Araip.11N45	0.394
Aradu.K119F	0.403	227	47.368	43.421	47.807	Araip.4WS44	0.412
Aradu.K13LE	0.339	123	54.032	40.323	31.452	Araip.IH553	0.325
Aradu.K14NU	0.353	138	55.396	39.568	40.288	Araip.4J7U0	0.346
Aradu.K16RE	0.351	382	48.564	38.381	38.381	Araip.NV86K	0.34
Aradu.K18SI	0.41	375	52.128	37.234	52.394	Araip.SU28H	0.411
Aradu.K1BYW	0.331	134	61.481	33.333	28.889	Araip.WUV4J	0.338
Aradu.K1JIV	0.393	451	55.31	48.23	42.478	Araip.4F02G	0.402
Aradu.K1MHV	0.404	172	58.382	39.884	45.087	Araip.DY62Y	0.421
Aradu.K1R5H	0.407	369	49.459	45.135	44.595	Araip.G2N6N	0.411
Aradu.K26VM	0.437	294	54.576	34.915	65.085	Araip.WYW75	0.441
Aradu.K285D	0.408	243	56.148	46.311	40.164	Araip.EZ6WD	0.403
Aradu.K2B5B	0.366	543	53.309	38.971	39.338	Araip.VBB27	0.362
Aradu.K2DQ9	0.45	338	47.198	42.773	56.932	Araip.3KN0A	0.45
Aradu.K2M4L	0.381	883	45.023	38.348	42.081	Araip.X7MJQ	0.376
Aradu.K2SIM	0.384	586	46.337	35.264	47.189	Araip.W9TLJ	0.373
Aradu.K2UAZ	0.377	575	55.729	44.097	33.681	Araip.8U122	0.381
Aradu.K2YQU	0.363	230	42.857	31.602	35.065	Araip.KQ0AG	0.372
Aradu.K34WH	0.345	487	48.361	37.91	33.811	Araip.4Y5NV	0.347
Aradu.K358E	0.39	277	51.079	40.288	41.367	Araip.BKJ61	0.391
Aradu.K38DA	0.385	520	48.369	38.964	34.741	Araip.Y7LP9	0.381
Aradu.K39P9	0.374	464	59.355	44.731	55.269	Araip.F1SXZ	0.391
Aradu.K3FG8	0.387	502	54.076	39.96	36.382	Araip.A9FL4	0.392
Aradu.K3GE6	0.451	397	44.724	43.719	45.98	Araip.AA702	0.441
Aradu.K3GZ3	0.395	429	56.744	47.674	38.837	Araip.3E954	0.392

Aradu.K3I53	0.356	154	52.258	40	35.484	Araip.XG2Y6	0.361
Aradu.K3RPT	0.407	525	46.768	41.445	50.951	Araip.F3DK3	0.412
Aradu.K3S1H	0.4	471	53.39	44.703	41.949	Araip.Y1XVN	0.4
Aradu.K3TU7	0.307	333	45.509	38.623	35.928	Araip.Q811U	0.322
Aradu.K3XG1	0.356	397	45.477	39.698	43.719	Araip.0DS8L	0.353
Aradu.K3YJZ	0.38	629	41.905	39.206	33.016	Araip.56WDA	0.377
Aradu.K4111	0.366	470	44.161	33.758	31.635	Araip.C2X2S	0.365
Aradu.K42LN	0.392	129	44.615	38.462	47.692	Araip.BQ2U7	0.416
Aradu.K44L7	0.329	404	46.173	41.481	40	Araip.DML9Y	0.337
Aradu.K45PE	0.436	554	52.072	44.324	43.964	Araip.N346R	0.442
Aradu.K49J7	0.372	223	41.518	52.232	40.179	Araip.13WJ1	0.349
Aradu.K4APN	0.36	313	41.72	42.994	36.943	Araip.4F18W	0.351
Aradu.K4GSH	0.373	115	54.31	36.207	39.655	Araip.4DS9Y	0.341
Aradu.K52EF	0.377	321	45.031	32.609	38.509	Araip.HY6IN	0.405
Aradu.K52XK	0.336	222	43.498	37.668	41.256	Araip.S4KSY	0.336
Aradu.K59XP	0.361	215	48.148	35.648	37.037	Araip.5I1EE	0.375
Aradu.K5HNP	0.417	262	52.091	37.643	63.878	Araip.1ZG04	0.398
Aradu.K5U1F	0.384	498	42.285	40.08	38.677	Araip.UH7KP	0.39
Aradu.K5W59	0.407	596	51.926	36.851	45.059	Araip.7513V	0.401
Aradu.K64M1	0.399	173	45.402	35.632	45.977	Araip.2C3K4	0.388
Aradu.K65B5	0.367	516	54.932	41.006	36.75	Araip.7EN7C	0.369
Aradu.K66VF	0.453	215	50.926	31.019	43.056	Araip.6035D	0.453
Aradu.K6R51	0.473	507	54.134	42.126	67.323	Araip.9D5ER	0.476
Aradu.K6SHY	0.337	528	49.716	38.563	38.752	Araip.QU01Y	0.346
Aradu.K6UHT	0.346	496	51.308	39.235	43.461	Araip.Q34LR	0.349
Aradu.K6X31	0.404	637	47.492	41.223	51.254	Araip.8ER40	0.407
Aradu.K6YI3	0.399	507	50.197	40.748	48.425	Araip.WW0EW	0.405
Aradu.K73MN	0.374	589	47.627	39.492	37.627	Araip.JB4PG	0.353
Aradu.K75LB	0.342	338	50.442	39.823	32.448	Araip.EP35E	0.332
Aradu.K7AFI	0.31	228	44.105	31.004	39.738	Araip.HU8XL	0.313
Aradu.K7CLQ	0.346	245	56.911	37.805	35.366	Araip.2R8F4	0.362
Aradu.K7HN9	0.443	316	50.789	45.426	42.587	Araip.XDY2R	0.423
Aradu.K7I1H	0.409	513	53.307	39.689	51.167	Araip.65AHE	0.404
Aradu.K7JNV	0.356	205	59.223	33.981	38.35	Araip.2ES8K	0.364
Aradu.K7STL	0.289	207	43.75	38.462	27.404	Araip.6T61F	0.37
Aradu.K7TJG	0.379	400	50.873	45.137	46.384	Araip.7MS83	0.391
Aradu.K7USP	0.376	669	54.478	44.328	33.881	Araip.8Q1DE	0.381
Aradu.K7Z0H	0.399	766	45.502	39.505	34.681	Araip.7MT36	0.401
Aradu.K80ZI	0.328	468	50.533	45.842	35.181	Araip.43U91	0.341
Aradu.K81IQ	0.367	376	39.523	35.013	38.727	Araip.K7SM4	0.374
Aradu.K82B1	0.402	278	48.029	34.767	48.029	Araip.RAG9J	0.426
Aradu.K84FP	0.436	342	49.271	38.484	56.56	Araip.A06C0	0.439
Aradu.K85GV	0.374	122	52.846	39.837	52.846	Araip.787IL	0.455
Aradu.K8DY2	0.321	780	50.064	39.052	38.028	Araip.DXM9R	0.32

Aradu.K8EE3	0.438	488	54.192	36.81	50.92	Araip.R6JKD	0.436
Aradu.K8H83	0.341	350	48.433	36.182	40.741	Araip.PK6CY	0.343
Aradu.K8V1Y	0.323	347	45.115	37.356	36.494	Araip.0AG3E	0.318
Aradu.K8WQA	0.435	196	44.162	37.056	45.178	Araip.JN4EG	0.394
Aradu.K8XCN	0.296	367	51.902	37.5	39.674	Araip.V1PYY	0.301
Aradu.K9162	0.34	509	46.275	38.627	39.608	Araip.21S30	0.341
Aradu.K9316	0.402	254	38.431	36.078	46.667	Araip.LN9X0	0.364
Aradu.K93AE	0.414	496	47.887	35.01	42.455	Araip.H41HP	0.422
Aradu.K952B	0.392	351	51.42	34.659	41.477	Araip.E510Q	0.398
Aradu.K955R	0.341	281	53.546	42.553	34.752	Araip.IX4R9	0.341
Aradu.K9JBW	0.402	1035	54.44	51.641	32.239	Araip.P7F38	0.395
Aradu.K9P9T	0.424	137	44.203	52.174	62.319	Araip.1196H	0.424
Aradu.K9R11	0.376	441	44.57	31.9	43.213	Araip.K222Y	0.382
Aradu.K9UV7	0.401	514	53.786	41.748	47.767	Araip.Y1XIM	0.397
Aradu.K9Z81	0.343	420	51.544	34.442	36.105	Araip.9I9CS	0.355
Aradu.K9ZYN	0.474	212	41.784	40.845	61.033	Araip.J95X4	0.48
Aradu.KA3UP	0.39	259	50	40.385	47.308	Araip.S3ICP	0.419
Aradu.KAS7C	0.351	922	50.488	44.42	34.67	Araip.R5NIU	0.35
Aradu.KB518	0.393	1290	51.356	38.962	35.941	Araip.NR5KZ	0.387
Aradu.KB6LK	0.429	308	46.278	40.129	30.744	Araip.ETI9M	0.429
Aradu.KB85J	0.377	518	53.95	38.536	48.362	Araip.K7EHW	0.383
Aradu.KBD84	0.408	425	47.183	38.967	46.714	Araip.T22BQ	0.406
Aradu.KC0GC	0.355	700	48.645	41.655	36.519	Araip.8W4UM	0.355
Aradu.KCC58	0.378	290	46.735	33.677	52.234	Araip.H2SSN	0.38
Aradu.KCP9Z	0.429	423	46.698	39.858	59.198	Araip.YR35J	0.433
Aradu.KD1XS	0.358	392	42.748	37.913	39.695	Araip.A8D0C	0.353
Aradu.KD2FK	0.367	265	43.609	35.338	35.338	Araip.20FU5	0.37
Aradu.KD6QW	0.336	457	46.07	37.773	39.956	Araip.BJA2B	0.348
Aradu.KD75D	0.399	464	43.656	40	43.226	Araip.8M6IT	0.394
Aradu.KDG8K	0.49	424	51.059	43.294	67.059	Araip.420T4	0.487
Aradu.KDH0U	0.404	219	51.364	42.727	53.636	Araip.4R8ZS	0.413
Aradu.KEC4P	0.333	196	48.223	36.041	36.041	Araip.66GM5	0.333
Aradu.KEZ8L	0.366	3880	53.543	36.666	38.444	Araip.0W0BL	0.363
Aradu.KF6LM	0.414	442	55.079	37.02	47.63	Araip.H3PA0	0.419
Aradu.KF8UQ	0.354	184	44.324	35.676	36.757	Araip.9BR1Z	0.357
Aradu.KFD3G	0.344	218	46.575	38.813	44.292	Araip.0EV9Y	0.33
Aradu.KG1H9	0.385	207	50	41.346	46.154	Araip.JK8BE	0.382
Aradu.KG41H	0.397	362	40.496	38.567	42.975	Araip.1E1WQ	0.397
Aradu.KG94L	0.387	366	45.504	37.057	46.049	Araip.27EE4	0.406
Aradu.KGT1D	0.409	404	58.025	40.494	47.407	Araip.ZDT9K	0.398
Aradu.KGX8R	0.387	621	46.945	38.907	42.283	Araip.BV0MU	0.387
Aradu.KH0NV	0.335	697	49.284	36.819	37.679	Araip.Y3MEG	0.337
Aradu.KH1Y4	0.383	529	50.943	35.66	40.377	Araip.S35TY	0.377
Aradu.KH25W	0.418	500	49.701	45.509	45.908	Araip.GL6UI	0.418

Aradu.KH49T	0.382	814	50.061	37.546	41.84	Araip.CK6AG	0.38
Aradu.KH5IZ	0.415	240	45.643	49.378	46.473	Araip.Y0GXG	0.408
Aradu.KH972	0.4	521	48.084	41.379	39.464	Araip.D00MK	0.398
Aradu.KHJ4B	0.363	215	47.222	33.796	37.037	Araip.KVQ8U	0.343
Aradu.KHV94	0.376	183	52.717	34.783	36.413	Araip.B8P7Q	0.376
Aradu.KI06N	0.45	270	52.768	45.756	53.137	Araip.DRP4T	0.453
Aradu.KIS5R	0.37	356	42.297	40.616	46.499	Araip.0PX5T	0.361
Aradu.KIY9T	0.476	222	56.054	44.843	47.982	Araip.JZF59	0.476
Aradu.KJ041	0.349	240	46.058	35.685	32.78	Araip.62MB6	0.338
Aradu.KJ1B9	0.349	456	53.392	40.044	36.105	Araip.TCX3B	0.347
Aradu.KJ6HK	0.339	262	52.852	46.008	49.049	Araip.9C688	0.337
Aradu.KJE7Z	0.379	254	47.059	44.706	36.078	Araip.VF36H	0.383
Aradu.KJH8E	0.425	514	54.369	41.553	60.777	Araip.WI0M1	0.426
Aradu.KJM0J	0.481	192	52.85	37.306	45.596	Araip.PUL8H	0.485
Aradu.KK6CI	0.381	1343	48.289	31.25	34.301	Araip.6WG88	0.379
Aradu.KL3C7	0.379	1194	47.615	40.251	33.556	Araip.3BJ9Q	0.376
Aradu.KL6JZ	0.4	588	50.085	40.238	36.842	Araip.SBB73	0.406
Aradu.KL6KV	0.41	282	55.124	46.643	58.304	Araip.F3CGD	0.428
Aradu.KLJ74	0.354	388	45.501	38.56	35.99	Araip.5J7CQ	0.342
Aradu.KLS6P	0.373	1255	49.761	35.191	39.252	Araip.S4GGT	0.357
Aradu.KLS9W	0.355	375	45.213	41.223	47.606	Araip.H6SDM	0.352
Aradu.KLU2C	0.385	422	57.21	48.463	44.681	Araip.1M393	0.383
Aradu.KM3QT	0.38	353	46.61	40.113	46.893	Araip.VRE44	0.386
Aradu.KMK70	0.374	733	48.365	39.101	33.106	Araip.GS4GH	0.374
Aradu.KNB1Y	0.396	203	50.49	44.608	38.235	Araip.D9UGE	0.396
Aradu.KNH52	0.367	439	50	42.5	38.182	Araip.V5Y6Q	0.357
Aradu.KNK4F	0.391	242	50.617	39.918	60.082	Araip.PGV10	0.398
Aradu.KNN0Y	0.309	489	44.286	36.531	38.367	Araip.FMC77	0.307
Aradu.KNP7I	0.346	404	53.827	34.321	47.901	Araip.KW46U	0.323
Aradu.KNX76	0.386	468	52.239	42.644	41.578	Araip.50XHI	0.399
Aradu.KP13G	0.38	274	49.818	40.364	42.545	Araip.TNU5T	0.363
Aradu.KP7PE	0.413	326	48.93	37.92	43.731	Araip.UM9DW	0.409
Aradu.KPJ13	0.382	320	52.648	41.433	33.645	Araip.KW34A	0.382
Aradu.KQ4LB	0.35	266	46.442	29.588	37.828	Araip.W7ZN3	0.366
Aradu.KR1YF	0.318	603	47.848	36.589	39.073	Araip.K5Y7B	0.317
Aradu.KR2M4	0.414	190	50.262	47.644	49.215	Araip.P1EA5	0.426
Aradu.KR62J	0.348	254	40.784	42.745	40.784	Araip.KT1QG	0.352
Aradu.KR6D1	0.443	149	59.333	28	59.333	Araip.MS1GW	0.45
Aradu.KR6YX	0.372	461	50.433	41.126	29.004	Araip.QL91M	0.37
Aradu.KRJ36	0.358	583	48.116	40.068	37.671	Araip.F4R2Z	0.361
Aradu.KRY7W	0.39	869	50.115	37.816	41.724	Araip.C6PK9	0.38
Aradu.KS2FL	0.575	116	60.684	47.863	58.974	Araip.IIQ50	0.547
Aradu.KS3VF	0.522	285	51.399	33.217	62.937	Araip.F2CNX	0.522
Aradu.KS96H	0.399	145	49.315	40.411	38.356	Araip.Y0ZC4	0.396

Aradu.KSI0H	0.359	1015	52.461	37.992	33.563	Araip.QK1BG	0.356
Aradu.KSK2P	0.441	309	47.419	44.839	50	Araip.HQ0LM	0.429
Aradu.KT4MN	0.398	305	53.595	36.928	42.484	Araip.64GAL	0.405
Aradu.KT81X	0.418	250	48.606	40.637	42.629	Araip.80ZQA	0.414
Aradu.KT95M	0.382	939	49.043	38.511	36.702	Araip.W4U3Q	0.388
Aradu.KTY6M	0.365	374	52.8	42.667	42.133	Araip.BHI10	0.368
Aradu.KU05P	0.337	359	52.5	33.889	45.278	Araip.7K2B7	0.364
Aradu.KU4KX	0.379	852	52.521	38.921	49.355	Araip.9P2KQ	0.378
Aradu.KU7WP	0.339	1126	49.689	43.035	36.469	Araip.4397U	0.339
Aradu.KUN1X	0.415	335	54.464	39.881	41.369	Araip.3R0IT	0.405
Aradu.KUP4F	0.403	510	55.186	43.053	41.487	Araip.1L67X	0.403
Aradu.KUT1Q	0.38	689	50.58	36.522	38.116	Araip.G8BEX	0.39
Aradu.KV07Y	0.371	313	49.045	37.261	42.675	Araip.N9YA2	0.371
Aradu.KV0LC	0.361	1528	50.164	40.746	35.056	Araip.XT6EH	0.364
Aradu.KV87W	0.36	104	44.762	56.19	42.857	Araip.DB9NP	0.336
Aradu.KVD8P	0.359	1155	52.422	40.138	35.208	Araip.82I4N	0.358
Aradu.KVT23	0.405	390	50.384	41.688	38.619	Araip.U985Z	0.423
Aradu.KVX0Z	0.362	631	53.323	43.196	37.342	Araip.BXV13	0.344
Aradu.KW0UC	0.415	365	46.175	46.175	46.995	Araip.3NN3F	0.426
Aradu.KW737	0.383	251	49.206	38.095	47.619	Araip.J7Z63	0.366
Aradu.KWJ2K	0.36	245	49.187	32.52	37.398	Araip.166TL	0.377
Aradu.KWP32	0.375	670	56.334	40.238	32.489	Araip.MP5EW	0.366
Aradu.KWX5Q	0.366	460	45.553	34.49	39.913	Araip.GE4AA	0.366
Aradu.KX0SA	0.367	411	53.155	37.621	37.136	Araip.03MS6	0.362
Aradu.KX34B	0.339	534	52.523	36.449	40.935	Araip.R34I7	0.336
Aradu.KX3FZ	0.418	359	57.222	46.111	60.833	Araip.PDZ35	0.415
Aradu.KX3YY	0.396	1032	53.533	32.333	39.4	Araip.ACZ6E	0.398
Aradu.KY185	0.338	783	51.148	40.689	35.332	Araip.E5LM9	0.339
Aradu.KY2F1	0.374	513	52.724	40.467	62.257	Araip.6DV57	0.376
Aradu.KY6W5	0.432	236	58.228	38.819	40.506	Araip.DBR9L	0.435
Aradu.KYE4X	0.346	450	44.124	42.129	39.69	Araip.E0FPV	0.353
Aradu.KYQ8M	0.416	358	49.025	45.961	37.047	Araip.N3JDF	0.43
Aradu.KZ2JX	0.463	128	58.14	35.659	46.512	Araip.7TY5U	0.415
Aradu.KZ4HC	0.384	303	52.961	41.776	38.487	Araip.VW2EE	0.377
Aradu.KZV99	0.352	135	50	34.559	42.647	Araip.J867Q	0.352
Aradu.KZX0F	0.473	492	56.592	41.988	71.197	Araip.IXQ5W	0.478
Aradu.L02C7	0.367	395	53.283	38.636	37.626	Araip.RZ2KY	0.362
Aradu.L0BQ6	0.339	771	50.259	36.658	39.508	Araip.K7ZBG	0.335
Aradu.L0GAW	0.361	1258	50.516	38.046	34.392	Araip.LUJ64	0.361
Aradu.L0S59	0.39	890	47.811	41.639	36.925	Araip.73WZS	0.386
Aradu.L0TFU	0.394	159	42.5	38.75	39.375	Araip.2GK61	0.403
Aradu.L0YDF	0.397	314	57.143	41.27	42.222	Araip.QLX2I	0.401
Aradu.L1447	0.411	492	48.479	41.785	41.988	Araip.L55CJ	0.412
Aradu.L18YW	0.446	275	46.739	49.275	42.754	Araip.27R9P	0.446

Aradu.L1FUQ	0.466	291	57.534	33.904	60.274	Araip.G1ISM	0.484
Aradu.L1KP9	0.352	427	51.869	43.692	32.009	Araip.L8E8C	0.345
Aradu.L1M1M	0.39	374	54.933	40.267	49.333	Araip.6S389	0.398
Aradu.L1U18	0.313	319	54.063	36.563	46.563	Araip.2LT0K	0.314
Aradu.L1VA7	0.435	302	60.066	47.525	39.274	Araip.6C9XH	0.442
Aradu.L1VEE	0.414	118	47.899	51.261	57.143	Araip.BAC4E	0.495
Aradu.L210Z	0.339	623	49.679	39.103	35.897	Araip.UHC90	0.342
Aradu.L25DZ	0.477	309	56.452	55.161	62.258	Araip.U6PZK	0.448
Aradu.L273D	0.409	352	50.142	41.926	38.244	Araip.CC7UH	0.413
Aradu.L2APB	0.394	133	46.269	29.104	67.91	Araip.41RUB	0.331
Aradu.L2BKM	0.338	236	48.523	41.35	32.068	Araip.PG9PR	0.332
Aradu.L2FW0	0.408	213	48.598	38.785	49.065	Araip.N76B5	0.415
Aradu.L2IAX	0.363	1232	53.041	39.173	32.198	Araip.NP5CT	0.366
Aradu.L2JLA	0.322	439	43.864	34.773	36.136	Araip.1KG8P	0.329
Aradu.L2PP0	0.45	118	53.782	36.975	43.697	Araip.FFF41	0.459
Aradu.L2RSA	0.348	526	46.3	40.228	42.694	Araip.WS2K0	0.348
Aradu.L2S26	0.299	444	44.27	30.112	26.742	Araip.CKV7I	0.337
Aradu.L2TEA	0.442	151	56.579	40.132	37.5	Araip.S4ZEH	0.441
Aradu.L2YDJ	0.359	217	52.752	30.734	36.239	Araip.73QKW	0.364
Aradu.L30M6	0.432	174	41.143	40.571	42.286	Araip.S5WAI	0.438
Aradu.L3677	0.322	355	47.753	42.135	33.708	Araip.EXQ89	0.324
Aradu.L39ME	0.39	468	48.401	33.689	49.467	Araip.98DPV	0.381
Aradu.L3C1J	0.371	819	49.268	32.195	44.024	Araip.TJA70	0.369
Aradu.L3GA1	0.388	255	60.547	41.797	41.797	Araip.HRU9Y	0.358
Aradu.L3I19	0.364	419	49.048	43.095	34.048	Araip.L03B5	0.364
Aradu.L3IFZ	0.368	826	48.851	36.034	42.684	Araip.VFY46	0.375
Aradu.L3K73	0.364	418	54.893	37.709	38.902	Araip.LA07L	0.378
Aradu.L3NRF	0.412	209	51.905	49.524	53.333	Araip.W3HL3	0.412
Aradu.L3NZR	0.354	317	46.541	52.516	40.566	Araip.7869H	0.354
Aradu.L3Q6E	0.374	2175	53.401	42.05	35.432	Araip.V2691	0.377
Aradu.L3QY1	0.344	331	45.181	43.373	36.747	Araip.XK9AB	0.342
Aradu.L3RRG	0.345	947	45.781	33.861	33.861	Araip.C5YQ1	0.341
Aradu.L3UB6	0.37	680	49.633	41.116	37.739	Araip.H1Y7A	0.367
Aradu.L3WM9	0.355	214	53.023	36.279	37.674	Araip.18J4Y	0.356
Aradu.L3X78	0.336	496	43.662	36.016	35.01	Araip.C0T71	0.345
Aradu.L46QG	0.36	487	49.795	40.164	36.475	Araip.61BVJ	0.353
Aradu.L49GZ	0.36	510	49.902	34.638	37.965	Araip.MJR9D	0.35
Aradu.L4AW6	0.369	510	54.795	39.53	35.029	Araip.UR9L3	0.365
Aradu.L4C7Z	0.445	430	42.691	37.355	46.868	Araip.B8J3R	0.434
Aradu.L4D0H	0.433	624	48.96	46.88	44.96	Araip.HKZ7Q	0.437
Aradu.L4E7U	0.371	1252	51.237	39.505	37.111	Araip.H61BH	0.371
Aradu.L4EY2	0.335	810	44.883	38.471	33.539	Araip.3074P	0.332
Aradu.L4IQ2	0.382	564	44.602	41.062	45.841	Araip.Y7S1I	0.384
Aradu.L4J2W	0.395	162	44.785	38.037	35.583	Araip.KV8CW	0.378

Aradu.L4JLT	0.399	706	52.051	43.847	40.877	Araip.RUZ03	0.399
Aradu.L4KP0	0.403	351	55.682	51.42	41.477	Araip.IN0U2	0.405
Aradu.L50NE	0.386	530	55.367	42.373	35.97	Araip.QP7G7	0.384
Aradu.L542Q	0.389	537	51.487	30.669	39.963	Araip.3MW2J	0.393
Aradu.L5CRG	0.382	247	52.016	40.323	34.677	Araip.YCD9D	0.398
Aradu.L5EJ3	0.443	241	44.628	47.934	48.76	Araip.EK4ZS	0.424
Aradu.L5GQQ	0.429	107	59.259	35.185	39.815	Araip.LA7GW	0.431
Aradu.L5IHI	0.367	551	51.63	38.768	38.587	Araip.X4GNJ	0.357
Aradu.L5KF2	0.367	335	59.524	56.845	44.048	Araip.J00PK	0.37
Aradu.L5LVS	0.37	374	47.467	33.867	41.6	Araip.G8PK0	0.398
Aradu.L5NS4	0.428	472	52.431	36.364	37.421	Araip.9D8PC	0.421
Aradu.L5P09	0.348	476	44.444	35.849	39.832	Araip.58VCE	0.354
Aradu.L5Q0P	0.398	411	56.553	41.748	47.33	Araip.5YV6C	0.399
Aradu.L5T1V	0.363	439	52.273	45.682	36.591	Araip.116UB	0.363
Aradu.L5WLB	0.317	148	45.638	36.913	48.322	Araip.57WAC	0.31
Aradu.L5Z6S	0.389	402	49.38	47.891	43.921	Araip.U0CS0	0.383
Aradu.L61KF	0.444	435	51.606	45.183	50.917	Araip.CN5UC	0.444
Aradu.L66NF	0.457	110	42.342	40.541	36.937	Araip.PR3IV	0.47
Aradu.L6791	0.393	479	44.583	32.708	37.917	Araip.32X2R	0.401
Aradu.L6FF7	0.388	556	53.321	35.727	37.882	Araip.MFD08	0.389
Aradu.L6GSR	0.458	296	47.475	46.128	55.219	Araip.TJ04M	0.462
Aradu.L6HBL	0.349	398	52.882	37.093	31.579	Araip.C2ULI	0.341
Aradu.L6IIL	0.371	592	54.637	35.245	47.555	Araip.Q7D64	0.357
Aradu.L6R4W	0.42	701	59.687	46.724	56.268	Araip.DU9DL	0.432
Aradu.L6TW0	0.481	453	53.965	44.493	62.775	Araip.W5IYZ	0.49
Aradu.L6U41	0.382	368	47.967	37.669	36.043	Araip.YNW3R	0.371
Aradu.L701F	0.392	468	48.188	34.115	38.38	Araip.UUS4S	0.402
Aradu.L7120	0.414	464	52.258	46.667	43.441	Araip.4H3RV	0.41
Aradu.L71CJ	0.431	358	42.897	43.175	38.162	Araip.T16GQ	0.426
Aradu.L72JH	0.373	1076	47.075	39.647	41.783	Araip.XF870	0.37
Aradu.L75IP	0.374	911	48.465	45.066	32.018	Araip.DC4LL	0.377
Aradu.L7CNH	0.49	207	57.212	55.288	48.558	Araip.TR541	0.49
Aradu.L7DZT	0.399	351	50.284	44.886	40.909	Araip.BT19G	0.392
Aradu.L7FSS	0.382	477	62.343	50.837	55.858	Araip.I6KEH	0.386
Aradu.L7RAQ	0.378	125	52.381	36.508	42.857	Araip.MTR7T	0.364
Aradu.L7WWU	0.362	487	46.721	36.68	37.09	Araip.91EFH	0.368
Aradu.L7XAF	0.325	278	46.237	30.108	27.24	Araip.10KZ6	0.321
Aradu.L7ZR8	0.371	362	47.383	48.209	40.22	Araip.D5D4T	0.374
Aradu.L7ZWM	0.444	490	53.564	41.955	56.619	Araip.Q0SP8	0.455
Aradu.L86SE	0.368	525	50.951	39.734	37.072	Araip.81A8L	0.379
Aradu.L8BYH	0.404	271	48.529	41.544	34.559	Araip.21F8G	0.396
Aradu.L8EYA	0.348	861	45.128	58.701	37.007	Araip.75MM4	0.351
Aradu.L8GIZ	0.358	682	44.217	36.896	41.142	Araip.F3KB9	0.353
Aradu.L8GUY	0.349	456	48.796	35.667	40.481	Araip.F7L5Y	0.341

Aradu.L8GY0	0.428	548	53.005	45.173	54.645	Araip.Z37FU	0.433
Aradu.L8JCD	0.398	117	51.695	44.068	53.39	Araip.QF0UL	0.367
Aradu.L8QZK	0.398	492	55.172	40.568	56.187	Araip.AQN9K	0.395
Aradu.L8SVN	0.364	286	44.948	37.631	42.509	Araip.T6ICI	0.357
Aradu.L933I	0.369	362	49.036	38.017	39.118	Araip.ZLC7F	0.371
Aradu.L9ISE	0.413	558	47.227	42.934	52.057	Araip.N54TA	0.421
Aradu.L9IYH	0.316	212	57.277	27.23	46.479	Araip.DJU8S	0.32
Aradu.L9J3S	0.355	227	43.421	40.351	46.491	Araip.30SUW	0.366
Aradu.L9JI8	0.463	285	52.098	41.958	43.357	Araip.6AJ5V	0.474
Aradu.L9M2B	0.436	252	55.731	38.735	52.569	Araip.71KEI	0.411
Aradu.L9QRL	0.424	268	51.673	42.379	44.61	Araip.374K5	0.443
Aradu.L9R8I	0.427	233	57.265	41.026	46.154	Araip.X09HZ	0.427
Aradu.L9SJ9	0.346	284	54.737	45.965	41.053	Araip.60QH4	0.379
Aradu.L9VT7	0.347	612	44.046	59.543	39.804	Araip.6EG6G	0.353
Aradu.LA0XT	0.407	362	56.474	43.526	52.066	Araip.TI5D7	0.424
Aradu.LA1WX	0.366	362	50.413	34.16	41.598	Araip.Z8IVG	0.378
Aradu.LA26X	0.383	537	46.84	44.052	37.361	Araip.T4R10	0.361
Aradu.LAA50	0.447	487	45.287	48.566	39.549	Araip.X9JQZ	0.453
Aradu.LAI2T	0.343	695	48.132	41.092	41.523	Araip.1K5KM	0.338
Aradu.LAK4N	0.347	206	57.971	57.005	35.749	Araip.0CF6W	0.312
Aradu.LAX0E	0.375	315	47.785	43.354	39.873	Araip.G7Z83	0.38
Aradu.LBD98	0.407	829	51.446	38.675	44.096	Araip.Z968E	0.398
Aradu.LBJ9G	0.429	210	50.237	42.18	51.185	Araip.JA75T	0.424
Aradu.LBR87	0.41	259	55.385	53.846	39.615	Araip.Q54JE	0.406
Aradu.LBT1F	0.424	412	53.027	42.131	40.92	Araip.3WF3M	0.42
Aradu.LC8HL	0.396	200	39.801	39.801	38.806	Araip.W0DHY	0.396
Aradu.LCC63	0.381	529	56.792	38.868	42.075	Araip.Z865I	0.368
Aradu.LCE5M	0.403	129	48.462	34.615	45.385	Araip.BT890	0.411
Aradu.LCM6E	0.335	276	46.931	36.823	38.628	Araip.ZPS65	0.347
Aradu.LCP0L	0.339	721	51.524	33.38	39.058	Araip.5QZ4M	0.336
Aradu.LD3HZ	0.362	433	46.544	43.318	33.41	Araip.1SH3C	0.357
Aradu.LD7BF	0.438	280	57.651	50.89	62.989	Araip.B5DCI	0.444
Aradu.LDI07	0.404	307	50	41.883	36.039	Araip.1X2V2	0.4
Aradu.LDI0R	0.368	138	53.957	29.496	35.971	Araip.S5AR3	0.376
Aradu.LE6Z2	0.497	160	54.658	41.615	47.826	Araip.B9M77	0.483
Aradu.LEE20	0.414	511	45.898	39.258	48.828	Araip.PM669	0.416
Aradu.LF0Z4	0.388	565	51.413	39.399	38.693	Araip.BGK8H	0.39
Aradu.LF3E5	0.371	386	50.129	41.344	39.793	Araip.Y1R8S	0.382
Aradu.LF5EZ	0.409	451	44.912	36.947	40.929	Araip.LTD41	0.411
Aradu.LF723	0.421	509	57.255	41.176	48.431	Araip.Q71DN	0.421
Aradu.LF76F	0.348	274	53.818	45.455	36	Araip.DQ9PJ	0.356
Aradu.LF9C0	0.342	439	50.227	37.955	35.227	Araip.67C1Y	0.354
Aradu.LFL4V	0.505	321	48.447	54.037	62.422	Araip.UE5VC	0.516
Aradu.LGS6Z	0.343	273	53.285	41.241	37.956	Araip.1T9DH	0.336

Aradu.LH1JG	0.364	248	49.398	38.956	46.185	Araip.7L4MP	0.37
Aradu.LH2UI	0.364	135	54.412	27.941	36.029	Araip.85AW8	0.38
Aradu.LH845	0.398	596	51.759	39.028	39.531	Araip.S24CF	0.408
Aradu.LHL11	0.387	1173	52.044	30.92	37.394	Araip.DHN9V	0.39
Aradu.LHT85	0.415	423	49.292	41.745	42.689	Araip.I12ZR	0.398
Aradu.LI04Q	0.469	362	47.107	39.394	54.27	Araip.WGQ93	0.483
Aradu.LJH4E	0.359	334	59.403	43.582	36.716	Araip.98AKT	0.347
Aradu.LJJ9P	0.404	345	47.688	35.838	46.243	Araip.6N7QF	0.395
Aradu.LJV84	0.498	323	53.086	50.926	52.16	Araip.0A4CX	0.509
Aradu.LK1UG	0.369	255	50.391	33.594	55.078	Araip.CAU9S	0.382
Aradu.LK89V	0.39	157	60.127	40.506	36.709	Araip.W0145	0.39
Aradu.LKB1I	0.358	1792	50.474	42.22	36.308	Araip.HD1YD	0.359
Aradu.LKW4E	0.398	853	47.892	41.92	41.452	Araip.X0K65	0.393
Aradu.LL477	0.343	682	43.924	38.946	34.114	Araip.G9LAW	0.347
Aradu.LL82A	0.391	332	57.357	46.246	34.234	Araip.KX93R	0.394
Aradu.LN5U5	0.401	309	56.774	37.742	46.129	Araip.EV4MA	0.401
Aradu.LN5YX	0.412	530	49.341	42.185	42.185	Araip.9V28V	0.411
Aradu.LNC81	0.389	446	46.756	30.649	46.98	Araip.NPK9J	0.384
Aradu.LNZ6T	0.452	408	52.078	45.232	46.455	Araip.QT8AK	0.441
Aradu.LP1C0	0.372	1639	50.305	37.683	38.902	Araip.Y7GV4	0.373
Aradu.LP8UK	0.357	817	54.768	45.966	31.785	Araip.NZT7X	0.355
Aradu.LP9ZI	0.469	390	55.754	49.872	63.427	Araip.NVV9S	0.477
Aradu.LQA0K	0.395	409	48.78	38.049	48.78	Araip.N6W5R	0.374
Aradu.LQC6W	0.43	192	59.067	38.342	51.295	Araip.3F2A6	0.414
Aradu.LQH8Y	0.393	1575	48.921	33.947	39.848	Araip.ZTF16	0.404
Aradu.LQK8F	0.412	682	50.073	40.264	43.338	Araip.D0AIB	0.406
Aradu.LQY7B	0.4	491	51.423	43.293	38.008	Araip.6Q97S	0.4
Aradu.LR2WR	0.43	405	51.97	41.872	42.365	Araip.HNC7T	0.434
Aradu.LR6SL	0.401	277	44.245	33.094	37.41	Araip.PJ4IX	0.404
Aradu.LRS2W	0.356	582	54.031	37.05	39.451	Araip.0I565	0.359
Aradu.LRZ4G	0.391	801	47.88	41.521	42.145	Araip.YBI64	0.392
Aradu.LS0XC	0.579	249	47.6	53.6	64.8	Araip.AR1XP	0.583
Aradu.LS2KI	0.346	591	46.959	40.203	36.824	Araip.37BUF	0.354
Aradu.LS42S	0.333	184	55.676	32.973	35.676	Araip.P5I6R	0.316
Aradu.LSK5Q	0.389	298	46.488	36.455	41.806	Araip.JKN5G	0.386
Aradu.LSL3G	0.388	539	52.222	39.259	54.815	Araip.HWV62	0.39
Aradu.LSV4Q	0.407	550	51.543	43.194	31.034	Araip.H1403	0.419
Aradu.LT240	0.397	398	47.619	40.852	48.872	Araip.Q779B	0.389
Aradu.LT37C	0.348	426	46.604	42.389	38.407	Araip.7ER0N	0.355
Aradu.LT5WZ	0.43	623	52.244	36.859	41.026	Araip.T9749	0.428
Aradu.LT7X3	0.491	232	48.069	34.335	53.219	Araip.2H1ED	0.478
Aradu.LTN41	0.382	802	49.44	38.232	39.228	Araip.79B36	0.385
Aradu.LUI60	0.395	317	61.635	48.428	53.774	Araip.K5VKJ	0.398
Aradu.LV4I2	0.38	456	50.766	42.67	38.074	Araip.5U7G7	0.387

Aradu.LV8M3	0.385	361	53.867	38.95	44.475	Araip.K3RVL	0.387
Aradu.LVF07	0.368	725	48.623	40.22	38.292	Araip.R19K2	0.366
Aradu.LVM3Z	0.374	144	51.724	35.862	30.345	Araip.FKG2G	0.379
Aradu.LVP8M	0.401	338	52.507	41.888	41.593	Araip.WP2AA	0.401
Aradu.LVQ6D	0.423	179	47.778	36.111	48.333	Araip.35TV0	0.412
Aradu.LVR9T	0.374	210	46.919	45.024	43.602	Araip.P34Y4	0.379
Aradu.LW197	0.359	528	52.93	40.643	38.374	Araip.J5SXF	0.355
Aradu.LW7LA	0.399	741	51.078	44.474	54.043	Araip.HXN3H	0.407
Aradu.LW83G	0.391	241	51.653	41.322	39.669	Araip.15UQN	0.386
Aradu.LW8Y2	0.378	585	56.485	45.222	37.372	Araip.M6R3N	0.39
Aradu.LWE3F	0.473	360	49.861	41.274	46.814	Araip.R46GQ	0.46
Aradu.LWF57	0.452	152	52.941	41.83	54.248	Araip.D1TXC	0.452
Aradu.LX0F3	0.384	330	49.849	41.088	39.879	Araip.1YM3E	0.399
Aradu.LX2KK	0.423	108	50.459	46.789	42.202	Araip.0E3QE	0.443
Aradu.LX505	0.385	504	52.673	38.416	39.406	Araip.9D3HV	0.391
Aradu.LXL9C	0.441	192	40.933	29.534	26.943	Araip.7M8Z5	0.413
Aradu.LXN93	0.42	268	42.007	39.033	43.123	Araip.ZN0SC	0.413
Aradu.LXU7Y	0.359	987	45.85	36.032	42.308	Araip.C8B17	0.366
Aradu.LXV06	0.352	258	49.035	39.768	38.61	Araip.W4GJ9	0.348
Aradu.LY8JJ	0.379	506	54.438	43.59	42.012	Araip.YR3S7	0.37
Aradu.LZ2RQ	0.311	405	42.611	35.714	33.005	Araip.B12DL	0.31
Aradu.LZ48I	0.436	574	51.13	40.348	54.087	Araip.C42Y7	0.437
Aradu.LZ6SK	0.454	124	51.2	56	52.8	Araip.PL9PT	0.445
Aradu.LZ6ZA	0.393	367	51.63	37.228	36.685	Araip.J6HFZ	0.39
Aradu.LZ99D	0.343	463	46.552	41.379	43.319	Araip.FK4XP	0.343
Aradu.LZC3L	0.469	384	47.532	45.714	56.883	Araip.1E43N	0.477
Aradu.LZR7H	0.405	794	66.415	52.075	33.962	Araip.63YUF	0.405
Aradu.M01CD	0.443	112	44.248	41.593	46.903	Araip.529RL	0.453
Aradu.M035D	0.398	678	51.694	37.555	51.399	Araip.UI1ED	0.389
Aradu.M0AKN	0.425	216	50.23	36.406	52.074	Araip.X6PEV	0.425
Aradu.M0DM1	0.376	370	46.361	35.849	42.318	Araip.C60UZ	0.365
Aradu.M0ENQ	0.357	584	43.419	35.897	39.829	Araip.WT898	0.352
Aradu.M0G9T	0.415	163	56.098	42.683	59.756	Araip.7I1D0	0.421
Aradu.M0JAP	0.424	258	47.49	38.61	30.888	Araip.S1S60	0.42
Aradu.M0KDX	0.331	428	46.387	43.823	30.303	Araip.F83BZ	0.337
Aradu.M0Q97	0.359	107	49.074	28.704	29.63	Araip.0S41I	0.355
Aradu.M0QV5	0.432	287	52.778	42.361	50.347	Araip.KHC1F	0.422
Aradu.M0V1K	0.383	272	52.381	54.212	35.165	Araip.AL6IJ	0.38
Aradu.M0VKX	0.382	471	54.873	44.492	39.407	Araip.YIM92	0.389
Aradu.M0XAT	0.378	368	50.949	37.94	36.585	Araip.M3EG0	0.364
Aradu.M0XVK	0.396	145	51.37	34.932	34.932	Araip.F1WJW	0.403
Aradu.M0ZRG	0.326	284	47.719	34.737	40.351	Araip.PQ54V	0.333
Aradu.M1252	0.474	513	48.638	37.938	57.198	Araip.R2UBA	0.459
Aradu.M15HC	0.4	765	46.997	38.642	35.379	Araip.T81Z0	0.394

Aradu.M167P	0.373	903	52.655	38.717	40.708	Araip.B25XR	0.364
Aradu.M18SC	0.437	276	57.401	37.906	49.097	Araip.V4I5J	0.433
Aradu.M1AX1	0.32	353	49.718	37.853	42.938	Araip.Y81SY	0.326
Aradu.M1BLH	0.365	102	46.602	38.835	50.485	Araip.HEJ11	0.354
Aradu.M1DIQ	0.392	348	52.149	41.261	41.834	Araip.NMT69	0.392
Aradu.M1GCR	0.393	334	44.776	40	42.09	Araip.15EJY	0.402
Aradu.M1IAT	0.421	134	56.296	45.926	40.741	Araip.C4K21	0.414
Aradu.M1JE3	0.32	156	43.312	42.675	35.669	Araip.D8IR5	0.308
Aradu.M1UTK	0.425	334	57.612	39.104	57.313	Araip.EYE7R	0.428
Aradu.M1XBW	0.346	981	53.462	35.438	33.198	Araip.4GI7N	0.35
Aradu.M206V	0.391	379	51.053	43.158	36.316	Araip.0T13B	0.388
Aradu.M29FV	0.362	555	52.518	41.727	37.77	Araip.VSW8G	0.354
Aradu.M2A53	0.36	286	43.902	40.418	36.934	Araip.8VG3L	0.363
Aradu.M2B10	0.48	432	49.654	46.651	65.589	Araip.NYT7M	0.468
Aradu.M2EU8	0.291	419	49.048	34.762	26.429	Araip.LPX1M	0.296
Aradu.M2I9A	0.377	134	51.852	31.852	37.778	Araip.7IS7K	0.369
Aradu.M2IMN	0.381	534	47.477	39.626	39.439	Araip.TF5RD	0.379
Aradu.M2JBM	0.377	678	47.717	36.23	38.733	Araip.4V1C3	0.368
Aradu.M2KYK	0.409	1338	53.099	32.487	40.03	Araip.88EKE	0.406
Aradu.M2MY1	0.441	359	51.944	39.444	65.833	Araip.25NFE	0.446
Aradu.M2NRW	0.446	308	51.456	46.602	62.136	Araip.T2627	0.462
Aradu.M35ST	0.389	448	47.884	40.535	39.198	Araip.ZWY76	0.399
Aradu.M35ZU	0.424	285	56.643	43.007	50.699	Araip.KLU9X	0.428
Aradu.M38D2	0.394	898	43.604	43.382	41.824	Araip.53EWD	0.397
Aradu.M3B1E	0.352	343	57.849	40.698	41.86	Araip.Q09LN	0.343
Aradu.M3CK1	0.324	400	42.145	39.152	31.671	Araip.Q42SH	0.313
Aradu.M3FDP	0.468	392	54.707	43.766	62.341	Araip.K28CA	0.475
Aradu.M3KDI	0.397	559	52.143	40.536	42.143	Araip.YE9C6	0.399
Aradu.M3LQ3	0.403	222	50.673	50.673	46.637	Araip.T7GHK	0.403
Aradu.M3Q0G	0.415	141	54.225	44.366	59.155	Araip.87I2H	0.4
Aradu.M3S97	0.286	109	50.909	39.091	35.455	Araip.C3AMC	0.276
Aradu.M3YXV	0.356	426	54.333	43.091	33.958	Araip.MJ6R4	0.35
Aradu.M414E	0.388	561	58.363	39.858	42.527	Araip.044E8	0.357
Aradu.M49FB	0.417	352	52.125	42.493	42.21	Araip.41EGI	0.415
Aradu.M4DGG	0.37	580	53.356	39.587	35.972	Araip.R828T	0.37
Aradu.M4DVD	0.407	506	53.254	41.026	38.067	Araip.2S7LP	0.405
Aradu.M4EHD	0.416	457	51.528	37.336	40.393	Araip.SH4GT	0.405
Aradu.M4GP4	0.382	421	47.867	30.332	39.573	Araip.L92QM	0.376
Aradu.M4JP1	0.436	377	51.852	41.799	42.063	Araip.IJ0FX	0.439
Aradu.M4MQC	0.459	192	52.332	35.233	62.176	Araip.ZQQ7K	0.476
Aradu.M4PQ9	0.364	663	47.741	43.524	40.361	Araip.96LCB	0.36
Aradu.M4TMQ	0.423	494	46.465	34.141	50.101	Araip.J9YKA	0.414
Aradu.M4TPQ	0.376	162	44.172	36.81	34.969	Araip.8AM62	0.384
Aradu.M4U01	0.348	688	50.943	42.525	34.543	Araip.ZDE30	0.348

Aradu.M4V5T	0.368	488	50.511	39.877	37.832	Araip.E5BVS	0.366
Aradu.M4YAD	0.38	553	49.278	38.448	39.17	Araip.EHL7Q	0.387
Aradu.M4ZYN	0.35	432	40.878	38.568	41.109	Araip.TSB8A	0.355
Aradu.M530F	0.455	471	55.932	43.856	56.568	Araip.12PK8	0.447
Aradu.M566R	0.393	129	53.077	39.231	36.923	Araip.YT7PR	0.389
Aradu.M5AZ6	0.426	103	55.769	48.077	59.615	Araip.D72J4	0.426
Aradu.M5I54	0.465	613	50.651	41.857	60.423	Araip.4G5MQ	0.472
Aradu.M5IDE	0.373	148	50.336	55.705	39.597	Araip.YTI0W	0.373
Aradu.M5LK1	0.358	236	50.211	35.865	30.802	Araip.6T0FQ	0.332
Aradu.M5RIF	0.443	325	53.681	40.798	48.466	Araip.H7U4C	0.433
Aradu.M5V2I	0.405	337	52.663	43.491	41.42	Araip.DT2WX	0.391
Aradu.M5W9D	0.371	216	50.691	35.484	43.779	Araip.9NP15	0.371
Aradu.M5WCG	0.457	128	58.915	42.636	64.341	Araip.U5NBJ	0.448
Aradu.M62SP	0.349	509	48.627	45.49	39.02	Araip.G2WFV	0.355
Aradu.M64BQ	0.442	291	52.397	52.055	55.479	Araip.AW9T2	0.456
Aradu.M66BW	0.514	195	57.653	46.429	69.898	Araip.6F7K8	0.519
Aradu.M69JC	0.513	161	50	43.827	69.136	Araip.MSL5F	0.517
Aradu.M6DL9	0.386	480	49.272	43.243	46.778	Araip.N863A	0.393
Aradu.M6HNT	0.386	769	52.208	45.714	44.805	Araip.W6TR0	0.39
Aradu.M6IGU	0.403	434	46.667	41.609	51.954	Araip.8VU6U	0.413
Aradu.M6LMC	0.413	433	54.608	46.083	33.641	Araip.KD7KV	0.384
Aradu.M6THB	0.31	511	50.977	30.273	38.477	Araip.03W6H	0.319
Aradu.M707Y	0.392	301	52.98	41.391	42.715	Araip.IQE81	0.406
Aradu.M70VI	0.472	110	49.55	46.847	52.252	Araip.L8PD6	0.462
Aradu.M7213	0.389	324	46.769	38.154	54.154	Araip.G3ZLR	0.395
Aradu.M72UM	0.393	340	52.493	37.243	36.95	Araip.WI7LP	0.393
Aradu.M73X9	0.403	311	53.526	42.949	36.859	Araip.Q1514	0.403
Aradu.M76UB	0.409	330	48.943	45.921	39.275	Araip.11643	0.408
Aradu.M785W	0.434	364	45.205	40.822	37.534	Araip.VMV0J	0.436
Aradu.M7GVG	0.364	606	44.152	37.068	34.267	Araip.G1HHU	0.344
Aradu.M7K9G	0.338	744	42.416	37.584	38.792	Araip.CU3D7	0.324
Aradu.M7MKB	0.417	213	46.729	44.393	45.794	Araip.K11ZS	0.446
Aradu.M7QN4	0.38	365	51.366	42.077	46.995	Araip.HQ6XR	0.384
Aradu.M7VT5	0.383	2165	55.494	41.09	38.458	Araip.WHL9Y	0.379
Aradu.M89U9	0.36	1022	47.801	40.274	44.184	Araip.EFP5Y	0.362
Aradu.M8B8F	0.373	703	55.824	45.881	44.744	Araip.LVJ4W	0.374
Aradu.M8FIQ	0.411	465	54.506	58.369	37.983	Araip.20WLX	0.415
Aradu.M8PFR	0.382	425	47.887	42.488	49.531	Araip.F8I62	0.387
Aradu.M8RTT	0.388	534	52.15	42.056	34.579	Araip.AQV0I	0.396
Aradu.M8UTW	0.361	749	54.4	34.533	38.133	Araip.M692U	0.365
Aradu.M96PR	0.438	750	49.667	42.21	55.26	Araip.TTR3L	0.442
Aradu.M970R	0.436	380	43.307	41.732	45.669	Araip.LSW6W	0.434
Aradu.M98W9	0.381	570	46.585	46.935	47.811	Araip.WKG50	0.373
Aradu.M9A72	0.384	678	50.221	45.361	39.175	Araip.NM5LZ	0.378

Aradu.M9CLB	0.389	325	56.748	42.638	49.08	Araip.BB6WZ	0.394
Aradu.M9FCY	0.416	411	54.369	45.874	43.204	Araip.50DA0	0.421
Aradu.M9GL4	0.329	308	43.042	38.188	42.718	Araip.64GCN	0.326
Aradu.M9J7Q	0.353	595	51.007	40.772	37.752	Araip.BG7KI	0.357
Aradu.M9L15	0.36	373	55.882	36.364	40.909	Araip.NZ32C	0.38
Aradu.M9QM1	0.382	331	53.614	37.048	41.566	Araip.5T83W	0.378
Aradu.M9TY2	0.398	576	49.567	40.381	41.248	Araip.CC8CA	0.403
Aradu.M9YMG	0.492	406	47.912	44.717	58.968	Araip.8XX1S	0.495
Aradu.MA01V	0.428	175	43.182	38.636	41.477	Araip.L50X7	0.435
Aradu.MA1DE	0.346	420	54.869	35.867	41.33	Araip.B0F5J	0.353
Aradu.MA23R	0.372	239	51.667	47.5	43.333	Araip.40836	0.372
Aradu.MA612	0.412	1040	55.331	44.957	36.888	Araip.M32YC	0.413
Aradu.MA8H9	0.369	273	46.715	36.861	51.46	Araip.FZ51R	0.373
Aradu.MA9YS	0.413	670	51.863	38.599	48.286	Araip.ADA7W	0.419
Aradu.MAS03	0.467	204	44.878	45.854	60	Araip.081EX	0.482
Aradu.MB6LX	0.373	335	48.81	32.143	39.286	Araip.LV58R	0.367
Aradu.MBB01	0.296	542	51.565	37.937	36.28	Araip.YX2BB	0.313
Aradu.MBF39	0.368	569	51.053	34.035	38.772	Araip.7L5BF	0.369
Aradu.MBU55	0.441	300	51.827	37.209	47.841	Araip.D8NFL	0.448
Aradu.MC16W	0.331	463	53.017	39.009	39.871	Araip.D3HJU	0.315
Aradu.MC57M	0.367	255	49.609	46.094	32.813	Araip.T280I	0.369
Aradu.MC6VZ	0.321	283	45.775	34.859	44.366	Araip.A87CL	0.317
Aradu.MCA4R	0.34	353	47.175	43.785	36.723	Araip.E58R7	0.347
Aradu.MD0ZW	0.343	906	46.858	36.384	33.297	Araip.9E6PV	0.342
Aradu.MD14G	0.422	293	58.503	37.755	51.02	Araip.9U01V	0.418
Aradu.MD214	0.392	465	50.215	45.494	34.335	Araip.JY13W	0.399
Aradu.MD5SA	0.414	190	55.497	35.079	46.073	Araip.P97RG	0.431
Aradu.MDC65	0.429	342	50.146	41.691	41.108	Araip.SIY5C	0.447
Aradu.ME00T	0.42	463	57.543	42.026	58.621	Araip.R0VZ6	0.42
Aradu.ME340	0.362	346	49.568	38.04	36.023	Araip.8YV7L	0.376
Aradu.MEC8Q	0.426	350	46.439	44.444	52.991	Araip.5CH0R	0.425
Aradu.MEV99	0.356	837	49.045	41.885	34.487	Araip.8206I	0.371
Aradu.MEY8C	0.386	1071	50.653	33.955	35.914	Araip.SGQ01	0.385
Aradu.MF07S	0.437	534	49.533	38.318	54.393	Araip.J2K2B	0.44
Aradu.MF6JY	0.406	165	47.59	45.181	39.157	Araip.63PMR	0.364
Aradu.MF9WN	0.463	168	48.521	44.379	59.172	Araip.ML4Q2	0.481
Aradu.MFG61	0.447	223	58.036	37.946	49.554	Araip.AE08Z	0.461
Aradu.MG1IB	0.349	248	54.618	35.341	42.972	Araip.2I1EB	0.343
Aradu.MG836	0.378	685	51.603	37.755	41.545	Araip.Q2FZ0	0.384
Aradu.MGD3M	0.413	323	48.457	33.333	39.506	Araip.AYL5E	0.437
Aradu.MH3SJ	0.383	336	46.588	39.763	41.84	Araip.P5W3N	0.376
Aradu.MHM9J	0.357	418	47.255	38.902	33.413	Araip.8Z8G7	0.357
Aradu.MHS46	0.419	298	54.515	44.816	43.478	Araip.CGB26	0.418
Aradu.MI27L	0.39	155	47.436	39.744	59.615	Araip.39P36	0.434

Aradu.MI2LX	0.437	354	53.239	40.563	49.296	Araip.WT1Z7	0.443
Aradu.MI3Z8	0.405	489	43.061	44.898	35.51	Araip.1NJ8N	0.411
Aradu.MI8WP	0.366	805	48.759	38.462	40.819	Araip.9V0W5	0.368
Aradu.MI9SZ	0.419	192	53.368	48.187	35.751	Araip.K8FHN	0.41
Aradu.MIM9N	0.419	253	50	42.126	37.795	Araip.CP6UC	0.417
Aradu.MIQ5P	0.366	402	46.898	38.958	33.499	Araip.MR59S	0.366
Aradu.MIW9U	0.396	256	45.136	41.245	45.914	Araip.S5ATW	0.393
Aradu.MIZ18	0.368	422	42.553	39.48	38.534	Araip.7QP8H	0.368
Aradu.MJ2SD	0.488	357	50.559	39.385	64.525	Araip.GR4Q0	0.49
Aradu.MJ7TV	0.408	371	49.731	43.817	49.194	Araip.Q43KM	0.407
Aradu.MJ83N	0.402	127	41.406	32.813	35.938	Araip.REN75	0.413
Aradu.MJT2G	0.404	203	52.451	50.98	46.078	Araip.VL605	0.38
Aradu.MJZ2W	0.382	183	44.565	38.043	41.848	Araip.9Q1TC	0.382
Aradu.MK4GU	0.402	478	41.962	37.161	49.269	Araip.ND08G	0.404
Aradu.MKD6V	0.373	472	50.106	43.34	36.786	Araip.ACE37	0.367
Aradu.MLQ47	0.358	381	55.497	39.267	35.34	Araip.N0SPZ	0.359
Aradu.MLY6C	0.374	473	50.844	41.983	36.287	Araip.9J4X2	0.379
Aradu.MM40G	0.395	425	49.296	49.765	41.784	Araip.I8FLU	0.395
Aradu.MM6MH	0.376	438	57.403	39.636	40.547	Araip.V3N9B	0.383
Aradu.MM88P	0.493	136	58.394	46.715	51.825	Araip.G1BN4	0.5
Aradu.MM9KG	0.43	266	57.303	54.307	44.944	Araip.GJS6W	0.424
Aradu.MMG1D	0.407	218	43.836	42.922	42.009	Araip.A8L1C	0.411
Aradu.MN6GR	0.474	629	58.413	43.175	67.46	Araip.TQV21	0.464
Aradu.MN7KS	0.404	304	48.852	41.639	38.689	Araip.9T0HK	0.394
Aradu.MNQ43	0.371	428	51.282	42.657	38.695	Araip.JNK1H	0.376
Aradu.MP2DM	0.368	429	50.698	39.07	41.163	Araip.0K6MU	0.373
Aradu.MP2U2	0.382	722	52.282	44.537	38.728	Araip.L02B7	0.369
Aradu.MP583	0.333	817	51.956	40.709	39.487	Araip.NV9EP	0.332
Aradu.MP8TG	0.437	446	52.349	37.808	42.953	Araip.26FIE	0.426
Aradu.MQ1LE	0.411	217	49.541	40.367	54.128	Araip.X04IN	0.415
Aradu.MQ2DW	0.419	249	59.2	42.4	52	Araip.2178J	0.435
Aradu.MQ4WG	0.448	678	55.523	45.066	62.297	Araip.08Z17	0.445
Aradu.MQ8BQ	0.418	479	48.125	38.75	45.208	Araip.S0PQ3	0.426
Aradu.MQK7K	0.44	277	49.64	43.165	69.784	Araip.C1GGI	0.467
Aradu.MQQ90	0.418	295	44.932	28.378	52.703	Araip.QDL0D	0.407
Aradu.MQT1Y	0.409	269	49.63	41.852	42.222	Araip.B6RJA	0.421
Aradu.MR5GS	0.422	305	45.752	35.948	49.02	Araip.G32SV	0.415
Aradu.MRJ6D	0.419	238	42.259	55.649	38.494	Araip.A33WF	0.409
Aradu.MRQ6G	0.425	527	54.167	37.311	59.47	Araip.QX9UN	0.419
Aradu.MRR38	0.388	594	52.437	43.529	47.395	Araip.XPK3R	0.385
Aradu.MRW76	0.422	267	55.224	42.91	38.433	Araip.2MA0U	0.453
Aradu.MS08A	0.389	409	43.415	44.878	35.61	Araip.A18M2	0.397
Aradu.MS2JZ	0.398	336	51.335	45.994	33.531	Araip.601K9	0.395
Aradu.MS406	0.414	231	41.81	51.724	37.931	Araip.Z36KU	0.412

Aradu.MS621	0.397	479	53.958	43.333	39.375	Araip.I2I77	0.395
Aradu.MS73B	0.357	445	55.381	39.238	41.48	Araip.Y8RPI	0.367
Aradu.MS98X	0.389	618	49.758	37.964	39.418	Araip.9XA0M	0.38
Aradu.MSK3Z	0.433	394	54.684	41.519	54.43	Araip.UL14Y	0.432
Aradu.MSQ8X	0.398	216	52.995	42.396	45.161	Araip.CGA2T	0.388
Aradu.MSR4Q	0.447	101	44.118	35.294	46.078	Araip.VR494	0.447
Aradu.MTE62	0.437	453	55.286	46.916	58.811	Araip.C53RL	0.441
Aradu.MTW86	0.376	483	49.793	34.711	42.562	Araip.G881G	0.378
Aradu.MU261	0.351	1036	52.652	38.476	35.487	Araip.S0RCS	0.35
Aradu.MU7BZ	0.382	384	54.286	41.039	47.273	Araip.A9YXZ	0.397
Aradu.MUM0J	0.391	518	54.143	39.884	40.077	Araip.JTL29	0.383
Aradu.MV1EC	0.466	486	43.326	43.121	54.004	Araip.5G9GQ	0.461
Aradu.MV2TG	0.387	297	55.705	38.591	37.584	Araip.S6UEZ	0.387
Aradu.MVB2G	0.387	269	48.519	45.926	48.519	Araip.FHM8U	0.383
Aradu.MVM9Y	0.401	492	51.521	38.337	43.205	Araip.RS9FT	0.401
Aradu.MVP73	0.365	611	54.248	33.987	39.052	Araip.YT0BK	0.355
Aradu.MVW70	0.405	307	54.545	37.987	34.74	Araip.STT9U	0.405
Aradu.MW11Y	0.331	857	48.252	36.713	34.615	Araip.Q2QUM	0.337
Aradu.MW13W	0.343	264	46.792	34.34	33.962	Araip.HP9ZJ	0.351
Aradu.MW1NF	0.377	935	50.855	38.675	36.111	Araip.I2Q6L	0.374
Aradu.MW4MQ	0.41	172	48.555	32.948	52.601	Araip.E3P4S	0.41
Aradu.MW6JM	0.416	329	50.606	40.303	45.758	Araip.YI047	0.423
Aradu.MW8ED	0.433	415	53.846	50.721	40.625	Araip.T0KA2	0.434
Aradu.MX1D6	0.427	237	47.059	34.454	51.681	Araip.AI0JW	0.419
Aradu.MX2L0	0.356	421	53.555	36.73	42.891	Araip.K1JTN	0.373
Aradu.MX7X2	0.376	690	56.585	41.389	39.219	Araip.VJ64H	0.365
Aradu.MX9DT	0.445	491	58.943	45.122	61.585	Araip.SH80B	0.444
Aradu.MY047	0.361	331	54.518	33.133	45.181	Araip.NR4UL	0.34
Aradu.MY1HL	0.367	231	46.552	41.379	37.5	Araip.2AE2F	0.376
Aradu.MY7DS	0.339	523	51.908	36.832	37.977	Araip.C1XY2	0.337
Aradu.MY8MV	0.382	823	45.874	40.534	38.714	Araip.28CQG	0.382
Aradu.MYL46	0.399	586	47.359	35.434	43.271	Araip.IN5DN	0.397
Aradu.MYL9N	0.417	400	51.122	40.898	45.636	Araip.567RF	0.414
Aradu.MYV5A	0.341	647	47.377	37.191	37.654	Araip.QE4BD	0.336
Aradu.MZ49K	0.4	533	52.434	38.015	54.307	Araip.NPU4T	0.406
Aradu.MZ5HW	0.348	570	51.489	40.105	48.862	Araip.GXD6K	0.354
Aradu.MZ5UW	0.387	450	45.898	32.816	45.676	Araip.HUQ8Q	0.387
Aradu.MZ9P5	0.371	531	48.684	36.654	45.301	Araip.BBI30	0.375
Aradu.MZX0S	0.285	260	50.192	35.249	36.398	Araip.H6DP8	0.283
Aradu.N0271	0.344	1270	49.095	36.035	31.55	Araip.TDM9U	0.347
Aradu.N09A5	0.377	1401	47.932	36.234	40.3	Araip.K4VGI	0.362
Aradu.N0CP9	0.37	368	57.182	39.837	39.295	Araip.8W43D	0.373
Aradu.N0F3U	0.361	414	50.361	33.735	39.759	Araip.L7MAN	0.345
Aradu.N0P3W	0.379	788	50.57	37.389	40.431	Araip.72USN	0.384

Aradu.N0QH3	0.484	284	62.456	42.105	74.737	Araip.K0390	0.469
Aradu.N0VBS	0.406	182	52.459	37.158	36.612	Araip.3QQ7J	0.395
Aradu.N0W4C	0.412	340	47.801	40.469	51.906	Araip.GVQ6N	0.408
Aradu.N0WNK	0.387	468	49.041	40.299	40.085	Araip.P1PGD	0.392
Aradu.N190Q	0.4	996	43.129	42.929	37.813	Araip.ADD0N	0.402
Aradu.N1ASM	0.367	102	51.456	47.573	58.252	Araip.PE2BT	0.421
Aradu.N1D5Q	0.381	403	49.505	43.317	39.109	Araip.D98D3	0.376
Aradu.N1DAK	0.431	382	55.091	31.854	51.436	Araip.3P0PJ	0.422
Aradu.N1IDK	0.348	529	55.094	32.075	41.698	Araip.JAR4Q	0.345
Aradu.N1KEX	0.559	210	49.289	38.389	75.829	Araip.XD82V	0.564
Aradu.N1NH0	0.389	1067	47.191	35.019	44.101	Araip.TUV2T	0.374
Aradu.N1NLT	0.343	454	40	40.659	42.637	Araip.6FE6S	0.333
Aradu.N1Q89	0.383	355	48.315	37.921	31.742	Araip.LFM83	0.412
Aradu.N1UVT	0.354	758	48.88	47.694	35.31	Araip.332V3	0.357
Aradu.N1YRP	0.428	458	56.427	49.237	54.902	Araip.SE9XW	0.416
Aradu.N1Z7I	0.319	520	47.409	38.004	36.276	Araip.JSV6Q	0.317
Aradu.N21E2	0.383	315	49.051	44.62	41.772	Araip.ND6Q8	0.397
Aradu.N24L5	0.377	646	43.895	39.104	41.731	Araip.9V3ZQ	0.385
Aradu.N25WS	0.351	330	48.943	42.9	36.254	Araip.T9R11	0.351
Aradu.N289G	0.535	441	51.131	35.068	75.113	Araip.B6GK5	0.521
Aradu.N2D1U	0.487	119	51.667	30.833	36.667	Araip.9D7FV	0.478
Aradu.N2G7A	0.398	403	53.218	43.069	51.98	Araip.NEM0P	0.398
Aradu.N2UA7	0.443	239	43.333	38.75	40.833	Araip.Y79HX	0.443
Aradu.N2VA8	0.326	827	42.271	35.145	39.13	Araip.JU6JI	0.332
Aradu.N2WYB	0.368	169	64.706	36.471	60.588	Araip.9F4Q1	0.356
Aradu.N2X0I	0.373	316	49.211	41.009	46.372	Araip.N95WX	0.379
Aradu.N2X1B	0.378	750	48.868	44.341	40.08	Araip.EVL84	0.387
Aradu.N2YDU	0.396	607	43.914	38.98	39.638	Araip.I1U0Y	0.395
Aradu.N3085	0.344	138	41.007	33.094	38.849	Araip.01BXJ	0.316
Aradu.N39PG	0.388	354	51.831	42.254	43.38	Araip.QT4ZA	0.398
Aradu.N3CWH	0.45	409	51.707	41.22	47.317	Araip.YQI1Y	0.436
Aradu.N3EHS	0.358	258	42.471	41.699	37.452	Araip.UVY31	0.354
Aradu.N3IWP	0.367	279	44.286	32.857	39.286	Araip.9WM3A	0.361
Aradu.N3KHF	0.376	477	49.372	35.983	41.004	Araip.64IP1	0.372
Aradu.N3N49	0.418	493	51.822	38.664	40.081	Araip.U7I8R	0.414
Aradu.N3NL4	0.36	832	54.982	39.256	40.456	Araip.2GN8J	0.353
Aradu.N3SPH	0.352	572	48.342	39.791	46.597	Araip.12MTY	0.362
Aradu.N3TMI	0.381	552	48.282	40.868	36.528	Araip.4Z4V8	0.389
Aradu.N42M1	0.452	147	54.054	35.811	52.027	Araip.TY4ZB	0.475
Aradu.N42W4	0.426	211	49.057	42.453	44.811	Araip.D2S2M	0.39
Aradu.N44D1	0.41	352	48.442	44.759	37.677	Araip.KI3IL	0.395
Aradu.N47AG	0.351	745	47.051	42.091	33.646	Araip.S85UY	0.34
Aradu.N49M2	0.367	708	46.968	40.056	36.812	Araip.Q0MMZ	0.369
Aradu.N4HHX	0.344	1153	42.028	37.262	38.128	Araip.KQ1P6	0.351

Aradu.N4IR4	0.329	331	45.482	44.578	36.446	Araip.TL94E	0.338
Aradu.N4PGS	0.462	217	52.752	38.991	49.083	Araip.AX2SC	0.448
Aradu.N4RHU	0.394	421	47.156	43.365	34.834	Araip.W05R7	0.387
Aradu.N5A68	0.417	546	58.135	38.94	45.704	Araip.R0KZE	0.412
Aradu.N5B8E	0.434	275	50	53.986	54.71	Araip.ZMH2R	0.429
Aradu.N5BC9	0.352	747	52.005	42.647	31.016	Araip.543CV	0.36
Aradu.N5BSE	0.463	134	45.185	36.296	40	Araip.6M6IA	0.488
Aradu.N5F6J	0.352	337	50.592	39.349	44.675	Araip.DHX2R	0.359
Aradu.N5GDJ	0.514	357	52.793	44.413	60.615	Araip.WZG9Z	0.5
Aradu.N60NI	0.395	133	55.97	43.284	52.239	Araip.N4PL2	0.408
Aradu.N62XI	0.399	332	49.55	41.441	42.643	Araip.78DPR	0.403
Aradu.N636R	0.37	509	53.333	47.451	39.804	Araip.TQJ7V	0.365
Aradu.N64VX	0.395	166	60.479	40.12	71.257	Araip.A0AXY	0.401
Aradu.N68GL	0.399	236	50.633	48.945	45.992	Araip.Y9BTG	0.407
Aradu.N69D4	0.369	461	46.753	51.732	36.797	Araip.R4HD6	0.387
Aradu.N725Y	0.366	335	48.512	32.44	44.048	Araip.N6IWK	0.339
Aradu.N76GN	0.393	233	51.709	38.034	45.299	Araip.X45KN	0.388
Aradu.N79JZ	0.37	137	60.145	34.783	33.333	Araip.M15ST	0.363
Aradu.N7C0U	0.367	222	39.013	31.39	37.22	Araip.FZ2I8	0.377
Aradu.N7DXE	0.342	162	47.239	41.104	41.104	Araip.J0PEZ	0.335
Aradu.N7F34	0.377	411	52.67	40.534	43.204	Araip.X8GX1	0.382
Aradu.N7FJP	0.353	481	46.473	36.722	41.079	Araip.H625D	0.351
Aradu.N7H5E	0.422	268	54.647	41.636	51.673	Araip.NL0PP	0.438
Aradu.N7IXY	0.406	671	54.762	40.476	53.869	Araip.N9QES	0.404
Aradu.N7LX6	0.405	436	56.064	37.986	47.368	Araip.BK8GH	0.402
Aradu.N7MUQ	0.425	465	53.433	38.627	51.288	Araip.9RR7M	0.435
Aradu.N7NSR	0.414	286	55.052	45.645	37.979	Araip.1MI2Q	0.423
Aradu.N7TNH	0.319	271	53.676	30.515	35.294	Araip.A1RD2	0.331
Aradu.N7TR4	0.312	363	43.956	38.462	31.593	Araip.R1VPM	0.32
Aradu.N7UWQ	0.353	677	51.032	34.513	38.496	Araip.FMQ8F	0.353
Aradu.N7VLT	0.381	417	48.565	38.756	37.321	Araip.069K4	0.389
Aradu.N7YFC	0.367	572	47.644	41.012	36.475	Araip.7LD9B	0.358
Aradu.N89Q4	0.359	461	44.589	38.312	41.991	Araip.KW3Z9	0.38
Aradu.N8FJN	0.437	259	54.231	37.308	40	Araip.RJ07Z	0.439
Aradu.N8MU8	0.31	362	43.526	38.567	31.405	Araip.609WS	0.315
Aradu.N8P27	0.417	342	46.939	42.566	48.688	Araip.YG292	0.425
Aradu.N8PYS	0.368	789	47.595	34.937	34.557	Araip.NWM0M	0.373
Aradu.N8RFP	0.421	327	57.622	40.549	56.098	Araip.KT3YI	0.431
Aradu.N8S8A	0.361	898	56.841	40.378	36.485	Araip.24FNG	0.371
Aradu.N8T7S	0.33	574	46.435	37.043	38.087	Araip.H2NMQ	0.325
Aradu.N8VJ2	0.471	267	48.134	45.896	57.463	Araip.0MB9K	0.452
Aradu.N906W	0.313	538	51.763	32.653	37.662	Araip.MKC7R	0.318
Aradu.N90WJ	0.429	528	49.527	43.1	52.174	Araip.4T3QB	0.431
Aradu.N98FX	0.402	785	45.802	45.674	34.86	Araip.PYI9U	0.412

Aradu.N9CVY	0.399	209	47.143	27.619	51.429	Araip.4BX2B	0.413
Aradu.N9F03	0.408	589	49.153	40.508	45.424	Araip.Y1FMZ	0.394
Aradu.N9KIE	0.42	775	50.644	36.469	40.077	Araip.VZ9BG	0.417
Aradu.N9KWN	0.406	214	50.233	34.884	40	Araip.572XZ	0.394
Aradu.N9R9J	0.307	204	52.195	44.39	34.634	Araip.03MNI	0.296
Aradu.N9RWR	0.341	883	49.548	39.14	35.973	Araip.WS5NM	0.338
Aradu.NA3JC	0.381	549	52.545	42.909	34.727	Araip.9K6YA	0.38
Aradu.NA9YP	0.362	1082	47.83	42.29	39.243	Araip.RW9UY	0.37
Aradu.NAE9S	0.368	617	48.544	40.615	45.307	Araip.E5XJJ	0.374
Aradu.NAJ8T	0.373	1864	48.257	40.375	37.587	Araip.X4UBD	0.367
Aradu.NB0W9	0.392	380	55.643	47.507	37.795	Araip.D723S	0.371
Aradu.NB41M	0.416	245	47.561	52.033	44.715	Araip.L417Q	0.42
Aradu.NBA2I	0.401	177	44.382	37.079	44.382	Araip.V6XXN	0.409
Aradu.NBA3F	0.372	457	54.148	46.725	34.061	Araip.QW087	0.374
Aradu.NBA8B	0.369	748	46.996	34.446	39.386	Araip.SX347	0.361
Aradu.NBQ7X	0.453	120	42.149	39.669	46.281	Araip.X6DID	0.453
Aradu.NC1XP	0.49	529	52.642	40.566	55.849	Araip.BK4FM	0.456
Aradu.NC3JN	0.47	287	56.944	36.111	39.583	Araip.6A689	0.478
Aradu.NC6JU	0.451	453	52.423	44.053	44.053	Araip.CQ2I9	0.451
Aradu.NCD56	0.427	259	50.769	39.615	41.154	Araip.YFS8J	0.417
Aradu.NCJ0H	0.478	675	50.444	38.757	62.13	Araip.26B5V	0.472
Aradu.NCP7U	0.342	435	51.376	35.092	39.22	Araip.X02G0	0.344
Aradu.ND06J	0.35	330	48.64	42.598	38.369	Araip.SUR5V	0.353
Aradu.ND275	0.425	276	57.762	33.935	36.823	Araip.M0I6P	0.43
Aradu.ND5YL	0.479	325	48.466	45.092	47.853	Araip.M8ZTC	0.44
Aradu.ND96S	0.345	542	52.302	39.779	33.702	Araip.DXJ40	0.333
Aradu.NDR67	0.359	271	49.265	40.441	50	Araip.WC0WL	0.359
Aradu.NE0BE	0.404	571	55.07	39.161	43.357	Araip.Z8IWW	0.4
Aradu.NE42A	0.374	623	47.115	40.705	34.615	Araip.175EE	0.37
Aradu.NE5BN	0.364	716	51.185	38.215	43.375	Araip.72N6Y	0.356
Aradu.NE81M	0.388	704	49.929	43.83	48.227	Araip.RZ5RS	0.39
Aradu.NE8U1	0.374	1550	50.226	43.52	35.397	Araip.AW51S	0.376
Aradu.NE9V5	0.418	598	56.928	36.728	47.746	Araip.21RG8	0.425
Aradu.NEL01	0.348	393	45.939	35.787	39.34	Araip.7EC8V	0.38
Aradu.NEM0A	0.504	463	51.724	44.397	61.207	Araip.I2EVD	0.496
Aradu.NF6XD	0.421	589	47.288	38.136	64.407	Araip.CQ7YT	0.419
Aradu.NF928	0.378	457	47.162	33.624	52.62	Araip.WJD5Q	0.373
Aradu.NFI11	0.351	334	40	35.224	46.567	Araip.X503X	0.357
Aradu.NFL6Z	0.373	388	46.53	39.846	46.015	Araip.KF0DD	0.374
Aradu.NG2JA	0.384	237	50.42	40.756	42.017	Araip.UCT63	0.389
Aradu.NG3QM	0.38	541	50	36.162	40.406	Araip.32MBN	0.383
Aradu.NG5IQ	0.392	619	48.548	42.742	36.129	Araip.E0Q5W	0.393
Aradu.NG8V7	0.417	315	50.633	56.329	45.57	Araip.U6ER1	0.407
Aradu.NGF0Q	0.509	607	50.987	40.461	59.868	Araip.IW8WM	0.492

Aradu.NGG06	0.396	514	52.233	43.883	39.806	Araip.PE9PU	0.408
Aradu.NH34T	0.313	371	46.505	43.011	30.645	Araip.ZD3SC	0.291
Aradu.NH44H	0.522	344	50.435	49.565	64.638	Araip.PY0W6	0.522
Aradu.NH66H	0.459	141	57.746	42.958	47.183	Araip.VB4KC	0.459
Aradu.NHB6E	0.364	361	46.685	38.122	47.514	Araip.SK1X8	0.366
Aradu.NHR1S	0.342	115	50.862	49.138	44.828	Araip.14BL9	0.342
Aradu.NI37K	0.4	571	50.524	38.112	45.629	Araip.GM91W	0.408
Aradu.NI3IK	0.388	955	56.172	39.435	35.46	Araip.NS167	0.394
Aradu.NI3KM	0.448	560	60.963	44.385	46.346	Araip.P0CYG	0.448
Aradu.NI3N5	0.4	509	53.333	40.196	40.392	Araip.AAR28	0.404
Aradu.NI44V	0.414	330	46.828	41.994	38.369	Araip.1M3VN	0.421
Aradu.NI7KT	0.352	831	46.394	39.543	35.216	Araip.MU39B	0.354
Aradu.NIH31	0.398	432	48.037	36.49	44.573	Araip.I19HU	0.4
Aradu.NII42	0.338	877	53.303	37.585	45.786	Araip.39SAQ	0.341
Aradu.NIR19	0.411	575	49.306	36.632	50.347	Araip.MM2M0	0.422
Aradu.NJ1ET	0.41	204	53.171	44.39	41.463	Araip.KE2SI	0.435
Aradu.NJ1GS	0.378	1072	48.089	43.243	34.296	Araip.L1Q1P	0.376
Aradu.NJ2WV	0.372	539	49.259	42.593	39.815	Araip.2P1HN	0.367
Aradu.NJH70	0.371	653	47.248	37.615	39.602	Araip.A35MH	0.367
Aradu.NJI26	0.327	115	54.31	31.034	37.931	Araip.54QEU	0.309
Aradu.NJJ5I	0.378	344	53.913	37.101	50.435	Araip.6D3E7	0.366
Aradu.NK3YY	0.338	200	50.249	33.831	43.781	Araip.SG7HP	0.337
Aradu.NKB4V	0.344	137	43.478	23.188	47.826	Araip.RKZ9H	0.344
Aradu.NKE3U	0.408	815	51.716	40.441	45.956	Araip.XM4LL	0.406
Aradu.NKJ9K	0.379	437	49.772	41.324	35.616	Araip.WB0T6	0.386
Aradu.NKZ2V	0.368	766	52.542	35.332	38.462	Araip.KQF28	0.372
Aradu.NL7A8	0.386	567	52.289	40.845	39.261	Araip.2H61M	0.388
Aradu.NL8HQ	0.385	231	45.259	34.052	45.259	Araip.1ML5W	0.39
Aradu.NM2TY	0.391	694	44.173	34.82	37.842	Araip.E7MNJ	0.407
Aradu.NM8U4	0.383	707	49.576	33.616	44.915	Araip.5CF12	0.354
Aradu.NN536	0.343	372	44.504	42.359	35.121	Araip.Q1LNW	0.344
Aradu.NNA2S	0.393	111	43.75	33.929	44.643	Araip.6QX3D	0.411
Aradu.NND2F	0.424	211	51.415	41.981	48.585	Araip.81PGB	0.431
Aradu.NNH8B	0.417	547	45.62	46.168	45.438	Araip.V9QLL	0.423
Aradu.NP275	0.376	429	45.814	37.907	37.209	Araip.LY9Q9	0.376
Aradu.NP2GP	0.367	628	47.695	39.746	45.628	Araip.U8SW2	0.361
Aradu.NP782	0.392	241	49.174	40.496	42.975	Araip.QNB65	0.392
Aradu.NQ2AE	0.38	720	51.456	38.835	35.09	Araip.CF3QY	0.38
Aradu.NQ3N9	0.376	434	53.103	31.954	50.575	Araip.96E8W	0.387
Aradu.NQ7IA	0.401	245	47.154	43.496	35.772	Araip.H4L5F	0.427
Aradu.NQ8H0	0.494	418	52.745	40.573	63.723	Araip.0E8VY	0.49
Aradu.NQC4B	0.341	833	47.722	38.01	41.607	Araip.0G5JH	0.346
Aradu.NR2Z3	0.369	1117	52.773	39.803	36.315	Araip.3PC35	0.353
Aradu.NRF9P	0.503	158	55.975	32.075	64.151	Araip.N6TB9	0.517

Aradu.NRG58	0.384	227	43.421	42.982	45.175	Araip.GV4V3	0.385
Aradu.NRN2N	0.42	212	49.296	45.54	49.296	Araip.U1KHK	0.397
Aradu.NS08D	0.393	326	46.483	37.309	40.979	Araip.D6MSX	0.393
Aradu.NS576	0.43	460	61.822	44.252	51.193	Araip.UHM0G	0.437
Aradu.NS580	0.418	231	38.362	31.466	37.5	Araip.QS8WY	0.431
Aradu.NS7I7	0.351	204	50.244	39.512	47.317	Araip.6D36H	0.337
Aradu.NS8UC	0.432	336	51.632	38.279	40.356	Araip.B03QY	0.427
Aradu.NSE79	0.353	839	47.857	35	37.857	Araip.0ER3Q	0.378
Aradu.NSL0R	0.365	355	43.258	40.449	33.708	Araip.MJ3JY	0.362
Aradu.NT394	0.393	802	47.572	38.356	34.247	Araip.9LU3E	0.385
Aradu.NT54G	0.326	806	42.627	34.82	34.201	Araip.Z6NL1	0.332
Aradu.NT5AR	0.339	1116	51.388	42.614	34.467	Araip.CEE4L	0.34
Aradu.NTD67	0.418	512	54.971	46.589	35.283	Araip.X4GRJ	0.416
Aradu.NTQ7W	0.455	334	56.119	45.97	52.239	Araip.K80UC	0.443
Aradu.NU5M2	0.423	322	56.037	38.7	52.012	Araip.QDA4J	0.419
Aradu.NUY4D	0.376	442	50.564	41.986	37.472	Araip.QF21H	0.389
Aradu.NVB9V	0.365	216	47.005	30.415	47.005	Araip.Z7H22	0.37
Aradu.NVM07	0.303	445	53.139	42.825	42.377	Araip.6VJ9V	0.3
Aradu.NVM7U	0.387	1010	50.445	31.256	43.126	Araip.YM3WQ	0.387
Aradu.NW9B9	0.384	350	51.282	44.444	45.014	Araip.QT4UB	0.351
Aradu.NX4M4	0.379	418	59.666	40.095	38.663	Araip.X7P83	0.372
Aradu.NXM6F	0.333	124	51.2	46.4	48.8	Araip.NP0MM	0.35
Aradu.NXS8X	0.403	345	59.538	45.087	39.017	Araip.KQ3SG	0.399
Aradu.NY5CH	0.327	330	47.734	37.764	33.233	Araip.3R87M	0.339
Aradu.NYD5R	0.357	418	49.642	38.663	37.47	Araip.4VW3W	0.366
Aradu.NYS45	0.345	422	53.901	42.317	50.591	Araip.C1JFS	0.34
Aradu.NZ3TX	0.377	158	42.767	44.654	27.673	Araip.5WT0G	0.39
Aradu.NZ4WV	0.38	250	52.59	39.442	41.833	Araip.LKP3W	0.38
Aradu.NZ8CP	0.452	301	55.629	42.053	50.662	Araip.LJJ47	0.468
Aradu.NZM5S	0.453	532	55.91	42.402	48.968	Araip.RG980	0.425
Aradu.NZS7L	0.411	381	44.503	41.099	48.429	Araip.IN81X	0.397
Aradu.P04DI	0.377	207	46.154	34.135	38.942	Araip.RG64D	0.373
Aradu.P05N1	0.361	651	50.92	34.509	41.104	Araip.T5WNM	0.363
Aradu.P0CUQ	0.426	639	57.656	41.406	44.531	Araip.5U8GK	0.425
Aradu.P0D43	0.396	484	55.052	38.763	34.227	Araip.HTH9H	0.388
Aradu.P0D7S	0.355	113	48.246	42.105	35.965	Araip.HIX6A	0.325
Aradu.P0G7F	0.367	450	44.124	42.794	38.581	Araip.XFN00	0.376
Aradu.P0IKP	0.394	230	53.247	42.857	38.095	Araip.G27IP	0.385
Aradu.P0J2M	0.376	379	52.105	42.632	41.842	Araip.CG0SE	0.373
Aradu.P0L8F	0.399	602	56.551	40.63	36.65	Araip.F3SVI	0.404
Aradu.P0TWG	0.406	206	45.894	36.715	48.309	Araip.H444F	0.392
Aradu.P0V8P	0.419	158	50.943	39.623	38.365	Araip.WMI4J	0.398
Aradu.P0XL9	0.406	364	59.726	46.301	40	Araip.R00CW	0.406
Aradu.P0ZYD	0.381	386	43.928	40.827	50.388	Araip.VF4EA	0.383

Aradu.P156Z	0.415	122	56.911	43.089	59.35	Araip.Q9JAV	0.398
Aradu.P1KDA	0.36	872	50.401	50.63	35.395	Araip.RA1WW	0.361
Aradu.P1TZQ	0.383	603	52.152	38.576	38.907	Araip.NS4X8	0.385
Aradu.P2130	0.41	105	52.83	37.736	52.83	Araip.X36UP	0.42
Aradu.P24XG	0.415	575	48.264	42.014	46.528	Araip.09AXD	0.408
Aradu.P28IF	0.367	902	54.485	41.86	40.421	Araip.JUS5G	0.362
Aradu.P2C14	0.488	433	58.065	47.235	64.286	Araip.CC2EP	0.486
Aradu.P2KMP	0.355	318	51.724	34.169	36.677	Araip.PC2P2	0.366
Aradu.P2KV0	0.377	571	50	39.685	50.35	Araip.774UX	0.389
Aradu.P2MN3	0.384	635	48.585	45.126	48.27	Araip.MC5NI	0.381
Aradu.P2MZP	0.378	304	47.541	36.066	35.082	Araip.MI54T	0.369
Aradu.P2N1A	0.361	414	48.193	38.072	38.554	Araip.UMG0L	0.363
Aradu.P2TGB	0.405	368	51.22	37.398	40.108	Araip.G5M3E	0.405
Aradu.P2TIC	0.405	707	52.401	43.785	41.102	Araip.TH9KW	0.41
Aradu.P39C1	0.454	334	56.119	36.716	57.612	Araip.M48BY	0.461
Aradu.P3C6F	0.507	281	59.929	38.298	64.539	Araip.AJ99C	0.505
Aradu.P3N99	0.469	133	46.269	35.075	61.94	Araip.1L1V5	0.469
Aradu.P3PDP	0.496	232	48.927	42.06	59.657	Araip.K86BU	0.496
Aradu.P3RU0	0.441	108	50.459	46.789	47.706	Araip.V4W9N	0.472
Aradu.P3SFW	0.449	432	56.351	34.642	47.113	Araip.UN61H	0.462
Aradu.P3VBU	0.404	351	53.977	34.375	45.455	Araip.73614	0.407
Aradu.P3XD1	0.358	518	49.326	42.967	37.38	Araip.X50KN	0.346
Aradu.P3XDF	0.366	568	39.367	39.543	38.84	Araip.GH9X1	0.355
Aradu.P3YYJ	0.441	403	46.04	41.089	42.327	Araip.TGT3S	0.449
Aradu.P432A	0.336	155	50	39.744	41.026	Araip.0P2W0	0.336
Aradu.P477D	0.363	2214	50.655	35.892	35.169	Araip.KK0GD	0.356
Aradu.P47CH	0.383	547	52.19	35.219	48.175	Araip.1G1KY	0.38
Aradu.P4874	0.436	563	51.241	36.525	45.922	Araip.T1IDG	0.432
Aradu.P49BC	0.309	492	46.045	38.742	35.903	Araip.NU3WA	0.313
Aradu.P4HVI	0.377	582	44.082	43.739	41.338	Araip.3B9Y6	0.388
Aradu.P4KG5	0.4	196	57.36	33.503	40.609	Araip.LY7NT	0.396
Aradu.P4R4T	0.589	170	57.895	40.351	64.912	Araip.P91HW	0.595
Aradu.P4XEI	0.337	352	50.708	39.093	38.244	Araip.H36I7	0.344
Aradu.P4XQA	0.406	360	49.307	40.166	42.936	Araip.N8G27	0.404
Aradu.P5027	0.362	931	52.253	38.519	34.442	Araip.YT9K8	0.356
Aradu.P51B9	0.355	634	49.921	40.787	39.055	Araip.V01DZ	0.356
Aradu.P535S	0.391	712	52.174	42.637	43.759	Araip.LW4LX	0.385
Aradu.P54FB	0.44	286	51.22	41.463	57.143	Araip.UPW6L	0.448
Aradu.P56I7	0.429	485	51.44	50.412	41.358	Araip.Z6W5C	0.427
Aradu.P56V8	0.438	461	43.939	34.416	40.693	Araip.WZ7IP	0.432
Aradu.P59ZT	0.398	308	53.722	43.689	35.275	Araip.XKH8U	0.398
Aradu.P5H21	0.321	362	45.455	33.333	39.945	Araip.BYS6X	0.331
Aradu.P5HY6	0.373	450	54.545	41.463	37.029	Araip.0W7EI	0.355
Aradu.P5PIT	0.377	1329	45.338	35.639	39.173	Araip.YI248	0.373

Aradu.P5PU5	0.402	205	53.883	42.718	50	Araip.IVL2L	0.422
Aradu.P5VH9	0.375	1276	52.937	41.817	37.118	Araip.83FL0	0.378
Aradu.P6497	0.37	234	51.064	38.298	31.915	Araip.I17NZ	0.379
Aradu.P6639	0.411	642	55.054	40.591	44.479	Araip.G1WPG	0.426
Aradu.P6DLH	0.368	189	46.316	32.105	42.632	Araip.FCU59	0.437
Aradu.P6GL4	0.376	659	47.273	34.545	48.182	Araip.6IN8N	0.381
Aradu.P6GSC	0.377	572	52.182	36.998	40.314	Araip.6S3BR	0.376
Aradu.P6RI2	0.371	360	45.983	32.964	42.936	Araip.EY5S9	0.372
Aradu.P6UBG	0.427	259	46.538	41.538	32.308	Araip.S3MIZ	0.435
Aradu.P6XBQ	0.41	232	56.223	44.635	38.627	Araip.I2DE4	0.405
Aradu.P6ZM2	0.345	150	53.642	36.424	41.722	Araip.2G1DX	0.331
Aradu.P709D	0.368	355	42.978	39.888	37.079	Araip.RVY5J	0.365
Aradu.P74XB	0.443	226	51.101	40.529	37.445	Araip.LKU3G	0.448
Aradu.P759U	0.424	345	55.491	43.353	42.775	Araip.6U9BD	0.417
Aradu.P778G	0.376	1837	49.565	36.997	34.603	Araip.Q83B6	0.377
Aradu.P79WU	0.414	138	49.64	48.201	31.655	Araip.IMH7R	0.376
Aradu.P7H9H	0.433	623	54.968	38.462	43.109	Araip.ATE50	0.436
Aradu.P7M2S	0.401	443	51.351	44.595	48.649	Araip.GV2B3	0.394
Aradu.P7M49	0.475	184	60.541	43.784	72.432	Araip.J123M	0.458
Aradu.P7PS3	0.407	270	47.601	36.9	39.114	Araip.P6A5Q	0.399
Aradu.P7QDD	0.481	224	44.444	52	52	Araip.23X2M	0.507
Aradu.P7QP3	0.331	161	53.704	43.21	37.654	Araip.A5PDN	0.301
Aradu.P7UBS	0.381	592	46.206	37.605	36.425	Araip.N813Z	0.389
Aradu.P7W5S	0.359	151	55.263	36.184	39.474	Araip.8I8HL	0.359
Aradu.P7XEX	0.402	259	50	32.692	53.846	Araip.M4FLV	0.398
Aradu.P7Y6N	0.404	219	51.364	32.273	47.727	Araip.Y4CF6	0.409
Aradu.P7YVS	0.345	343	54.942	39.535	37.5	Araip.K18VU	0.353
Aradu.P7Z3S	0.389	590	50.254	43.655	38.917	Araip.NEP9J	0.389
Aradu.P81TA	0.346	240	46.888	36.515	42.324	Araip.JM5TE	0.338
Aradu.P82M7	0.346	1566	53.542	35.992	38.864	Araip.R07YY	0.347
Aradu.P82NP	0.311	139	40	37.143	25	Araip.E05X3	0.344
Aradu.P833S	0.383	477	54.184	36.192	41.632	Araip.31SQ6	0.379
Aradu.P86W5	0.38	438	53.531	33.713	37.358	Araip.J1YCX	0.382
Aradu.P8M1S	0.445	237	57.143	31.933	54.202	Araip.SNW0B	0.458
Aradu.P8N7B	0.32	266	45.693	40.824	40.824	Araip.64WT2	0.348
Aradu.P8XB7	0.371	406	51.597	45.209	40.541	Araip.B7ULW	0.368
Aradu.P8Y29	0.437	429	52.791	43.256	62.791	Araip.C4A9H	0.424
Aradu.P8YMU	0.429	168	53.846	42.604	49.112	Araip.09GVV	0.423
Aradu.P98IW	0.335	455	50	42.325	41.009	Araip.0V3N6	0.338
Aradu.P9CN5	0.362	651	49.693	44.939	45.859	Araip.FL59H	0.371
Aradu.P9DB9	0.366	628	54.372	53.895	30.525	Araip.AM36D	0.362
Aradu.P9DZZ	0.364	398	46.867	38.847	39.85	Araip.ZBY9B	0.365
Aradu.P9ETF	0.485	281	53.901	43.617	58.156	Araip.AZ41E	0.481
Aradu.P9IKS	0.339	469	47.66	37.66	32.979	Araip.Z1VW2	0.339

Aradu.P9J09	0.412	289	53.793	46.207	39.31	Araip.U1RD3	0.408
Aradu.P9JVV	0.393	691	47.688	43.064	44.364	Araip.T9719	0.389
Aradu.P9PIK	0.376	1217	46.716	40.312	35.55	Araip.5R5N0	0.378
Aradu.P9PXC	0.395	231	53.017	37.5	46.121	Araip.W1ASW	0.395
Aradu.P9TWB	0.394	648	48.074	44.376	49.769	Araip.3Q94J	0.393
Aradu.P9YG3	0.405	687	50.581	41.134	53.488	Araip.NG0MG	0.409
Aradu.PA2UE	0.386	590	50.085	42.47	32.826	Araip.HB5N9	0.382
Aradu.PA8BP	0.329	620	47.182	38.969	45.089	Araip.A2DFF	0.327
Aradu.PA9WC	0.383	249	54	40.8	34	Araip.Y9NJG	0.37
Aradu.PBM8D	0.452	162	45.399	50.92	33.129	Araip.RQ5W2	0.439
Aradu.PBY44	0.484	357	48.045	41.061	63.687	Araip.160V1	0.47
Aradu.PC4II	0.378	784	48.662	39.49	37.962	Araip.X9FTP	0.379
Aradu.PD37S	0.369	843	48.578	36.848	43.365	Araip.51VQE	0.372
Aradu.PD7X7	0.406	444	52.584	35.281	42.022	Araip.36SP7	0.404
Aradu.PDJ58	0.305	374	45.333	38.667	32	Araip.Q76PE	0.306
Aradu.PDS8K	0.366	235	52.542	51.695	41.949	Araip.YB0D8	0.354
Aradu.PEH3C	0.464	256	56.809	49.416	49.027	Araip.8GW84	0.456
Aradu.PEP5T	0.374	498	50.1	37.675	45.291	Araip.RW6GJ	0.381
Aradu.PF1HF	0.439	434	49.655	44.828	44.598	Araip.P4X64	0.425
Aradu.PF378	0.462	182	48.087	38.251	63.934	Araip.L4IFZ	0.424
Aradu.PFQ1E	0.386	360	48.753	42.659	34.072	Araip.H3DRL	0.399
Aradu.PG3K0	0.351	566	52.734	35.45	35.45	Araip.551RZ	0.357
Aradu.PGH8Z	0.346	425	55.164	42.254	35.446	Araip.FM7NI	0.346
Aradu.PHB5M	0.501	500	51.497	43.912	72.255	Araip.G0MKK	0.493
Aradu.PHE1E	0.426	243	53.279	45.492	49.18	Araip.ZE0AY	0.402
Aradu.PI1VW	0.403	659	51.212	36.515	42.576	Araip.JF7QM	0.401
Aradu.PI5HS	0.476	219	51.818	39.091	53.182	Araip.8133V	0.448
Aradu.PIA7H	0.349	215	55.093	32.407	44.444	Araip.FUS30	0.373
Aradu.PIJ3J	0.418	474	54.737	44.211	39.579	Araip.1A0QT	0.399
Aradu.PIT85	0.354	410	52.068	42.336	40.633	Araip.VN33E	0.359
Aradu.PIU2J	0.396	557	51.792	37.814	43.19	Araip.2B2WU	0.391
Aradu.PJ07U	0.355	355	43.82	39.326	41.573	Araip.788L8	0.351
Aradu.PJ0EM	0.408	521	46.935	42.912	47.701	Araip.Y0BV6	0.402
Aradu.PJ1DH	0.379	289	51.724	35.862	34.138	Araip.WSE1J	0.394
Aradu.PJ77P	0.401	764	52.941	44.052	38.562	Araip.V26H1	0.397
Aradu.PJH5B	0.329	156	46.497	26.115	55.414	Araip.V8ASR	0.336
Aradu.PJM2P	0.42	197	50.505	38.889	51.515	Araip.L434T	0.41
Aradu.PK283	0.501	358	51.532	51.253	57.939	Araip.M90ED	0.503
Aradu.PK4QW	0.382	1233	43.922	33.468	39.546	Araip.A9NYP	0.385
Aradu.PK7XR	0.381	492	56.187	43.205	38.945	Araip.Q8CS4	0.375
Aradu.PKA69	0.383	770	46.822	36.706	40.337	Araip.VA9R0	0.384
Aradu.PL5Z6	0.243	119	25.833	33.333	28.333	Araip.D0SBS	0.316
Aradu.PLL31	0.373	637	53.762	44.671	29.467	Araip.U184X	0.375
Aradu.PLM7U	0.357	947	50.949	37.025	41.983	Araip.ML7EU	0.349

Aradu.PM3IW	0.45	113	57.018	34.211	40.351	Araip.DQ2EG	0.45
Aradu.PM5NX	0.359	257	45.736	42.248	37.984	Araip.05XM7	0.348
Aradu.PMG1D	0.395	451	49.115	37.832	42.699	Araip.6NT76	0.395
Aradu.PP1IT	0.307	584	50.085	37.778	36.752	Araip.ZS6RG	0.308
Aradu.PP2DC	0.386	443	53.829	39.189	36.486	Araip.L9YEX	0.393
Aradu.PP2S4	0.403	482	45.549	35.818	45.135	Araip.W5S9P	0.413
Aradu.PPM14	0.396	330	56.193	41.692	41.39	Araip.FBH15	0.396
Aradu.PQ5HC	0.427	382	55.614	43.603	53.264	Araip.N4GPP	0.429
Aradu.PQF90	0.407	484	48.66	44.33	56.082	Araip.R4PLR	0.408
Aradu.PQW7I	0.345	519	51.538	35.577	32.885	Araip.13H0V	0.347
Aradu.PRD5G	0.364	1517	47.892	37.813	39.46	Araip.30QH3	0.368
Aradu.PRJ6R	0.376	357	50.559	46.648	41.341	Araip.8X38S	0.382
Aradu.PRR6C	0.368	215	54.63	37.963	38.889	Araip.RQ6E9	0.359
Aradu.PRW5G	0.398	332	43.243	40.841	42.643	Araip.1Y2CP	0.375
Aradu.PS0E5	0.423	351	55.398	38.92	39.205	Araip.K2IF4	0.423
Aradu.PS45N	0.396	717	53.203	41.086	43.872	Araip.I5XHJ	0.401
Aradu.PS85R	0.392	306	54.723	38.111	46.58	Araip.K9ABC	0.386
Aradu.PSF4U	0.417	250	54.98	35.458	53.386	Araip.CFK5T	0.415
Aradu.PSU1K	0.409	549	50	38.909	39.455	Araip.VW89Q	0.401
Aradu.PT2CX	0.347	497	50	39.357	38.153	Araip.3PM87	0.347
Aradu.PT44X	0.364	182	40.437	45.902	37.705	Araip.1SL1G	0.364
Aradu.PT6UP	0.391	683	48.246	34.211	49.123	Araip.EQQ7V	0.409
Aradu.PU1U6	0.373	408	54.768	38.631	44.988	Araip.9BQ78	0.386
Aradu.PU456	0.343	565	48.233	32.509	32.155	Araip.76CRM	0.34
Aradu.PU63R	0.415	577	46.54	39.273	45.502	Araip.Z7JB7	0.432
Aradu.PU6SR	0.339	187	52.66	38.298	42.021	Araip.SEZ0Y	0.348
Aradu.PU85I	0.433	624	48.64	48.48	61.12	Araip.F5HYI	0.443
Aradu.PUZ2B	0.376	759	53.684	38.421	45.921	Araip.6VK7W	0.378
Aradu.PV1T4	0.462	310	53.376	36.656	49.518	Araip.G38PQ	0.46
Aradu.PV43B	0.333	310	45.016	36.656	39.55	Araip.LR5ES	0.342
Aradu.PV4QE	0.38	545	50.366	47.985	41.392	Araip.ZH99C	0.385
Aradu.PV79J	0.4	934	56.15	41.818	44.92	Araip.0WD88	0.398
Aradu.PVS6M	0.342	295	48.649	32.77	42.23	Araip.3W28R	0.338
Aradu.PW3YK	0.42	380	55.643	40.682	42.52	Araip.SX5U5	0.411
Aradu.PW4PJ	0.377	392	43.003	36.132	46.565	Araip.DE4JR	0.372
Aradu.PW6QG	0.366	364	44.658	40.822	28.493	Araip.E23GM	0.365
Aradu.PX1XB	0.369	882	53.228	40.997	34.315	Araip.2L6KD	0.366
Aradu.PX3ED	0.327	348	47.564	35.817	40.688	Araip.X9Q77	0.33
Aradu.PXH87	0.421	260	50.958	45.211	52.874	Araip.1ML5Q	0.414
Aradu.PXM5A	0.404	464	51.183	38.065	51.828	Araip.IF23W	0.402
Aradu.PXP20	0.43	981	51.222	38.289	57.332	Araip.06PW6	0.425
Aradu.PXT5K	0.39	585	47.782	42.15	39.59	Araip.DN1XY	0.382
Aradu.PXZ8J	0.379	503	55.754	44.246	35.913	Araip.XS8K8	0.371
Aradu.PY313	0.402	113	50.877	55.263	60.526	Araip.GFH7L	0.421

Aradu.PYG3I	0.438	483	52.893	40.496	50	Araip.7D3J7	0.433
Aradu.PZ18V	0.36	383	51.823	42.969	34.896	Araip.4J20T	0.35
Aradu.PZ2KX	0.448	409	43.171	44.878	49.756	Araip.R8FCB	0.458
Aradu.PZ2PD	0.337	635	55.975	39.937	38.208	Araip.Y45KL	0.34
Aradu.PZ3X7	0.375	407	51.961	42.647	42.157	Araip.KSS1C	0.391
Aradu.PZ509	0.452	498	49.299	46.293	44.89	Araip.1P1MT	0.448
Aradu.PZ5G0	0.407	383	51.563	40.365	43.75	Araip.89MN9	0.413
Aradu.PZB3C	0.381	421	46.445	38.152	38.626	Araip.XVM4V	0.389
Aradu.Q00GR	0.383	679	44.853	36.471	47.206	Araip.H1NU3	0.382
Aradu.Q018E	0.36	1319	45.379	44.545	35.379	Araip.3671W	0.357
Aradu.Q0GRU	0.388	609	46.066	38.033	42.131	Araip.MT85H	0.384
Aradu.Q0H7N	0.392	517	54.826	43.243	46.718	Araip.43WXS	0.402
Aradu.Q0IL1	0.344	368	50.407	34.688	44.715	Araip.AT3RC	0.332
Aradu.Q0MV7	0.373	172	47.399	36.416	32.948	Araip.ZDE4L	0.377
Aradu.Q0NF8	0.375	333	61.677	34.431	47.605	Araip.65APQ	0.387
Aradu.Q0NSJ	0.465	264	53.208	44.528	38.113	Araip.8395H	0.461
Aradu.Q0PGE	0.338	352	46.176	34.844	34.561	Araip.R9REP	0.325
Aradu.Q0T0K	0.398	551	51.63	40.217	44.565	Araip.LDR2S	0.396
Aradu.Q0XNV	0.431	347	50.575	42.241	41.092	Araip.6Q6V5	0.443
Aradu.Q0YMS	0.35	347	48.563	38.793	34.195	Araip.H72KX	0.359
Aradu.Q0Z3H	0.385	112	40.708	36.283	58.407	Araip.AU5HS	0.394
Aradu.Q0ZWM	0.452	338	48.378	37.463	57.522	Araip.SGA37	0.45
Aradu.Q123V	0.353	452	51.876	37.969	43.267	Araip.K1692	0.36
Aradu.Q14N7	0.355	521	47.318	36.782	34.1	Araip.44WZX	0.355
Aradu.Q17ZW	0.37	385	50.259	38.86	39.378	Araip.2L5W7	0.37
Aradu.Q1CYX	0.403	254	53.333	40.784	44.314	Araip.CS4YJ	0.401
Aradu.Q1F6L	0.425	185	54.839	48.387	44.624	Araip.BJ799	0.425
Aradu.Q1NTT	0.422	383	48.698	41.146	52.865	Araip.X2AYN	0.418
Aradu.Q1RYU	0.459	389	49.487	38.974	54.615	Araip.Q73AM	0.475
Aradu.Q1S4K	0.383	465	55.794	39.7	39.7	Araip.P1GI9	0.379
Aradu.Q1VWH	0.368	574	47.304	40.696	44.87	Araip.76V1I	0.384
Aradu.Q26PA	0.397	711	50.421	42.556	41.433	Araip.XFD5U	0.394
Aradu.Q27ZD	0.403	313	54.14	41.083	39.809	Araip.987R9	0.401
Aradu.Q2AQJ	0.346	406	51.843	31.45	49.386	Araip.0R69M	0.344
Aradu.Q2NGG	0.393	756	52.972	38.838	38.838	Araip.EGS0W	0.39
Aradu.Q2QD0	0.401	175	42.614	38.636	45.455	Araip.PIX7S	0.405
Aradu.Q2SWK	0.404	332	55.255	37.538	39.94	Araip.H4PLS	0.42
Aradu.Q3FM7	0.413	430	48.26	35.499	47.332	Araip.BYM6A	0.413
Aradu.Q3FMD	0.333	546	51.92	42.962	36.929	Araip.DYJ2G	0.324
Aradu.Q3HQI	0.409	340	55.132	52.199	35.191	Araip.I5D0R	0.402
Aradu.Q3KFX	0.466	140	44.681	39.716	53.191	Araip.WY6XF	0.457
Aradu.Q3QE4	0.4	206	49.275	42.512	55.072	Araip.AFF6E	0.384
Aradu.Q3RYP	0.442	275	52.899	37.319	56.884	Araip.QBG6Y	0.432
Aradu.Q3XX4	0.293	124	50.4	39.2	38.4	Araip.AH9LW	0.293

Aradu.Q451G	0.364	753	49.072	43.103	38.859	Araip.R7LB3	0.363
Aradu.Q49PC	0.355	148	48.322	42.953	44.295	Araip.S7ANW	0.376
Aradu.Q4G7J	0.365	542	56.906	35.543	44.199	Araip.SNJ74	0.369
Aradu.Q4TAZ	0.468	189	43.158	46.316	48.947	Araip.5XX3F	0.468
Aradu.Q4VIY	0.381	1261	48.97	36.846	42.71	Araip.L270U	0.381
Aradu.Q4Y5D	0.402	327	53.049	37.195	34.146	Araip.6V0UJ	0.413
Aradu.Q4YIY	0.367	314	48.889	48.254	37.143	Araip.TLW99	0.381
Aradu.Q507J	0.434	364	56.986	38.904	63.836	Araip.8B7U9	0.438
Aradu.Q51NH	0.393	983	54.065	36.789	43.293	Araip.XGD3E	0.386
Aradu.Q54JY	0.372	314	57.778	31.429	36.508	Araip.W48V2	0.366
Aradu.Q55UA	0.479	101	45.098	44.118	36.275	Araip.NG0LV	0.469
Aradu.Q566Q	0.427	312	46.645	43.45	48.882	Araip.8M8IP	0.419
Aradu.Q59PD	0.374	127	47.656	33.594	45.313	Araip.FBY21	0.396
Aradu.Q5DZL	0.385	417	55.502	39.952	44.737	Araip.2NV9I	0.382
Aradu.Q5EC8	0.417	205	49.029	53.398	38.835	Araip.3S4CE	0.401
Aradu.Q5FHV	0.407	588	46.01	40.407	35.144	Araip.T8ZMH	0.41
Aradu.Q5JK4	0.353	715	52.654	47.765	35.894	Araip.JYL2X	0.356
Aradu.Q5M0R	0.385	366	56.131	35.695	55.041	Araip.NIV47	0.367
Aradu.Q5NNZ	0.43	279	54.286	39.643	43.214	Araip.UT0YW	0.44
Aradu.Q5PIR	0.362	225	46.018	39.823	32.743	Araip.Z7C6V	0.373
Aradu.Q5Q2B	0.318	184	52.432	31.351	34.595	Araip.BI95U	0.324
Aradu.Q5XDE	0.438	550	50.454	37.931	59.165	Araip.W1LHP	0.431
Aradu.Q6596	0.462	141	42.254	48.592	49.296	Araip.18UE9	0.462
Aradu.Q67RD	0.338	720	50.347	43.273	38.419	Araip.VJ1WN	0.342
Aradu.Q6DD2	0.401	164	50.909	41.212	44.242	Araip.S7E86	0.42
Aradu.Q6P6Q	0.33	296	48.148	39.731	34.007	Araip.NQ67T	0.332
Aradu.Q6RE5	0.388	792	47.667	35.561	47.289	Araip.F73NM	0.383
Aradu.Q6WYU	0.42	222	46.188	33.184	37.668	Araip.5U4XM	0.42
Aradu.Q70DU	0.413	426	56.44	36.3	40.984	Araip.2F6VL	0.418
Aradu.Q71BR	0.445	270	47.232	40.959	56.827	Araip.71CN8	0.453
Aradu.Q7508	0.351	670	54.993	37.109	33.234	Araip.GVN2J	0.35
Aradu.Q77AH	0.53	329	50.303	51.212	50	Araip.J006E	0.518
Aradu.Q78JW	0.457	479	51.875	42.708	58.75	Araip.0ZP55	0.466
Aradu.Q7KU7	0.382	228	56.769	44.978	40.611	Araip.YS2KW	0.386
Aradu.Q7MTE	0.46	164	47.879	44.242	55.758	Araip.47I29	0.472
Aradu.Q7TCS	0.386	760	50.986	40.342	36.137	Araip.XKV3V	0.379
Aradu.Q84QR	0.386	515	51.163	50.194	43.992	Araip.74MNE	0.38
Aradu.Q85V2	0.421	339	49.412	44.118	37.353	Araip.7HD76	0.421
Aradu.Q86YJ	0.373	294	54.237	42.373	34.576	Araip.DH2VM	0.381
Aradu.Q8JL3	0.396	775	53.093	40.335	57.732	Araip.LBU7J	0.395
Aradu.Q8MCV	0.341	522	50.478	35.564	47.036	Araip.T1KRW	0.345
Aradu.Q8N4A	0.437	503	52.381	41.27	50.397	Araip.YV08U	0.439
Aradu.Q8SMK	0.428	159	39.375	35.625	38.75	Araip.3KU1V	0.442
Aradu.Q95MC	0.454	382	56.136	38.12	67.885	Araip.24WNV	0.449

Aradu.Q97KS	0.373	273	53.285	35.401	49.27	Araip.LXC6U	0.375
Aradu.Q9E1W	0.379	500	48.503	34.132	42.515	Araip.A4X5Z	0.378
Aradu.Q9F9Y	0.379	1047	48.76	40.076	35.878	Araip.Z2DN9	0.386
Aradu.Q9JNX	0.376	614	47.967	37.561	44.715	Araip.BZS09	0.36
Aradu.Q9LEZ	0.348	381	54.974	41.623	40.838	Araip.8VK7X	0.344
Aradu.Q9REZ	0.439	194	62.051	61.538	33.333	Araip.S1D0F	0.439
Aradu.Q9UFT	0.389	420	49.644	37.292	51.544	Araip.LTC7U	0.405
Aradu.Q9Y83	0.47	391	56.888	45.663	55.612	Araip.S5AVW	0.458
Aradu.Q9YKL	0.384	780	47.119	40.589	43.406	Araip.AJD1E	0.391
Aradu.Q9ZWS	0.336	306	44.951	46.906	48.86	Araip.XM7CZ	0.337
Aradu.QA0D1	0.38	275	44.203	47.826	37.319	Araip.JQB3R	0.38
Aradu.QA1ML	0.404	162	48.466	41.104	52.761	Araip.UI3CA	0.404
Aradu.QA3D9	0.456	513	56.42	44.553	52.918	Araip.FR0CD	0.456
Aradu.QA44E	0.369	430	61.485	35.267	39.211	Araip.TL9EX	0.376
Aradu.QA9KZ	0.368	604	51.074	32.893	39.008	Araip.7U0RM	0.373
Aradu.QAC2D	0.4	434	49.425	43.218	51.264	Araip.L0CPF	0.394
Aradu.QAZ4U	0.399	324	50.769	41.231	45.538	Araip.LSW71	0.399
Aradu.QBK5E	0.374	483	53.512	45.868	39.876	Araip.G34L7	0.37
Aradu.QC0V1	0.302	555	44.424	39.209	28.417	Araip.HEI1D	0.304
Aradu.QC0XR	0.375	474	48.421	45.684	38.947	Araip.I6LMJ	0.383
Aradu.QC919	0.344	858	46.333	36.671	35.623	Araip.IR8SW	0.353
Aradu.QCD9Z	0.391	266	50.562	38.202	38.951	Araip.2S8VQ	0.4
Aradu.QCF5U	0.441	150	55.629	47.02	49.007	Araip.2D392	0.441
Aradu.QCH6Q	0.445	140	52.482	46.099	54.61	Araip.MGA4W	0.449
Aradu.QCH8L	0.412	547	53.832	47.81	43.978	Araip.UI2R0	0.419
Aradu.QD7TL	0.348	665	45.646	36.637	40.991	Araip.R9EZV	0.344
Aradu.QDB5N	0.383	473	51.477	41.983	41.561	Araip.E1EX9	0.389
Aradu.QDT9L	0.364	547	53.285	34.307	37.774	Araip.FRJ8B	0.367
Aradu.QE1YJ	0.387	364	52.603	40.274	41.096	Araip.3U41R	0.39
Aradu.QE3CA	0.419	528	51.985	40.265	47.07	Araip.F9ULB	0.423
Aradu.QE4AN	0.386	551	53.08	42.935	43.841	Araip.3R01Q	0.384
Aradu.QE5XI	0.511	236	52.321	40.506	51.477	Araip.W5PJ7	0.505
Aradu.QED65	0.469	279	55	61.429	43.929	Araip.S2947	0.477
Aradu.QEK0G	0.377	223	45.536	38.839	43.75	Araip.X98AS	0.395
Aradu.QF91Q	0.426	206	51.691	47.343	58.454	Araip.9B5MM	0.429
Aradu.QFV3H	0.38	384	45.974	35.584	50.649	Araip.95Q23	0.378
Aradu.QG2PF	0.342	369	49.459	36.216	42.703	Araip.1KN42	0.343
Aradu.QGW0X	0.356	429	54.186	38.605	38.372	Araip.YL8HA	0.344
Aradu.QH0IG	0.384	488	47.035	36.81	53.17	Araip.BS4D0	0.4
Aradu.QH23N	0.452	185	53.763	47.312	60.753	Araip.90KYQ	0.452
Aradu.QH2VI	0.456	452	52.539	45.916	49.669	Araip.KJS5P	0.452
Aradu.QH5RZ	0.34	434	46.667	31.954	30.575	Araip.IV7HZ	0.36
Aradu.QH7UZ	0.376	483	46.074	45.455	41.736	Araip.2F21P	0.381
Aradu.QHA6Y	0.328	347	45.977	39.655	36.207	Araip.DER8T	0.328

Aradu.QI4L1	0.46	223	55.357	36.161	55.357	Araip.JM3P0	0.461
Aradu.QI7LX	0.333	315	48.418	40.19	44.62	Araip.L5QI4	0.337
Aradu.QII1E	0.339	428	44.056	34.732	34.732	Araip.D53S9	0.324
Aradu.QIJ36	0.403	543	59.375	46.324	47.978	Araip.H6D5I	0.394
Aradu.QIZ46	0.434	178	56.983	46.369	40.223	Araip.A8S33	0.439
Aradu.QJ7B7	0.39	419	52.619	43.571	40.714	Araip.BC8KL	0.391
Aradu.QJ7XC	0.38	309	48.387	40	40.968	Araip.HX3C4	0.384
Aradu.QJD0U	0.39	482	43.892	39.752	45.963	Araip.LNT1B	0.406
Aradu.QK488	0.384	167	49.405	38.69	42.857	Araip.Q1NLX	0.386
Aradu.QK77V	0.43	538	52.505	45.64	48.052	Araip.CU4DG	0.412
Aradu.QK85I	0.386	605	52.64	39.109	34.818	Araip.T7YD7	0.386
Aradu.QKN8V	0.386	492	49.29	35.091	32.657	Araip.ARE8G	0.384
Aradu.QKQ8N	0.351	1072	55.266	39.795	36.067	Araip.01ZYD	0.351
Aradu.QL41Y	0.364	787	52.284	35.914	34.645	Araip.UR8F6	0.356
Aradu.QL7M2	0.37	497	46.988	36.546	44.779	Araip.BTV23	0.373
Aradu.QM05Z	0.447	253	53.543	45.669	39.764	Araip.FL6S0	0.443
Aradu.QM4PI	0.372	1212	49.711	38.335	32.729	Araip.9DP5A	0.38
Aradu.QMU6X	0.389	295	51.351	38.176	41.892	Araip.IQ4XK	0.398
Aradu.QN5ZJ	0.411	445	51.345	36.996	41.48	Araip.L5ERK	0.394
Aradu.QNE8S	0.355	534	48.785	34.766	37.757	Araip.XLI8D	0.346
Aradu.QNG0R	0.359	595	43.624	35.738	37.248	Araip.WJL2T	0.36
Aradu.QP7DQ	0.394	369	45.405	38.378	36.486	Araip.UIV2U	0.41
Aradu.QPZ5M	0.444	334	50.448	38.209	57.015	Araip.P3SXN	0.409
Aradu.QQ3BK	0.384	326	50.459	38.838	37.309	Araip.75DC1	0.384
Aradu.QQB0D	0.397	435	52.294	44.495	38.991	Araip.DFK5Q	0.395
Aradu.QQF2S	0.372	1543	52.655	45.661	34.456	Araip.J736G	0.366
Aradu.QQX3T	0.336	311	55.769	39.423	39.423	Araip.T1WWY	0.351
Aradu.QQY2K	0.386	964	49.223	40.311	40.415	Araip.G1GCS	0.375
Aradu.QR44H	0.355	628	50.556	45.151	39.746	Araip.VU9PS	0.356
Aradu.QR8QC	0.403	435	41.514	45.183	37.156	Araip.TKX33	0.415
Aradu.QRL86	0.353	380	53.543	39.895	41.47	Araip.QR9S0	0.359
Aradu.QS20K	0.456	495	48.185	40.726	61.492	Araip.DB8NC	0.457
Aradu.QS26M	0.383	1895	54.167	46.677	36.603	Araip.D5S7B	0.386
Aradu.QS5ZN	0.465	169	34.706	43.529	53.529	Araip.BW6Q6	0.468
Aradu.QS8M2	0.4	551	52.899	36.232	41.667	Araip.M8E98	0.394
Aradu.QS9PE	0.407	191	50	44.792	43.75	Araip.AS5XR	0.401
Aradu.QSW68	0.359	383	48.698	38.802	41.146	Araip.E9AXK	0.37
Aradu.QT3H3	0.4	504	45.743	40.198	53.861	Araip.5JT26	0.398
Aradu.QT3HF	0.345	722	45.92	39.419	37.483	Araip.UBX9E	0.353
Aradu.QT6LV	0.41	245	47.967	39.024	47.561	Araip.47CG9	0.429
Aradu.QU0CV	0.445	322	52.012	56.037	50.774	Araip.FB29M	0.447
Aradu.QU3KT	0.408	214	45.116	39.535	56.279	Araip.F18V2	0.403
Aradu.QU580	0.359	235	42.797	38.983	38.559	Araip.HP125	0.36
Aradu.QU5N8	0.369	636	51.177	37.206	38.932	Araip.MIY8R	0.371

Aradu.QU7BE	0.349	858	48.079	47.846	36.088	Araip.7W8RG	0.353
Aradu.QU8L1	0.39	167	44.643	36.31	38.69	Araip.WPV5M	0.402
Aradu.QU90K	0.387	349	49.714	40.857	45.143	Araip.U6WSS	0.379
Aradu.QUJ7R	0.405	227	48.684	35.965	33.772	Araip.53BLN	0.412
Aradu.QV0NG	0.349	582	48.37	43.053	50.086	Araip.LLP3C	0.335
Aradu.QV2N6	0.434	150	62.914	54.305	37.748	Araip.FB3XS	0.434
Aradu.QV5DJ	0.373	732	51.296	45.975	36.562	Araip.2LX3X	0.378
Aradu.QV720	0.349	187	48.936	42.021	60.106	Araip.X6AXT	0.353
Aradu.QV7H5	0.395	367	53.261	33.696	67.663	Araip.HP461	0.394
Aradu.QVD5S	0.402	570	46.41	42.907	41.681	Araip.ID15H	0.407
Aradu.QVF0N	0.359	176	50.847	33.333	46.328	Araip.S11GW	0.353
Aradu.QVN0R	0.476	358	50.696	37.604	56.825	Araip.EMI4R	0.472
Aradu.QVT4C	0.355	689	50	41.594	36.377	Araip.61EW7	0.349
Aradu.QVX65	0.415	474	48.421	36.842	44	Araip.86BCN	0.418
Aradu.QW806	0.384	952	49.213	35.362	41.028	Araip.90Q1T	0.391
Aradu.QWH3Y	0.319	327	47.561	39.939	29.573	Araip.GAR8H	0.325
Aradu.QX6W8	0.525	196	55.33	47.716	77.157	Araip.JK2QJ	0.541
Aradu.QX9N9	0.374	551	53.623	41.667	40.217	Araip.84ICS	0.372
Aradu.QXQ1E	0.358	424	52.941	36.706	51.294	Araip.19YRX	0.358
Aradu.QY2T7	0.365	513	46.304	35.798	43.385	Araip.G0H3V	0.363
Aradu.QY7MG	0.373	688	48.766	36.865	32.946	Araip.I28F8	0.365
Aradu.QYX69	0.383	312	52.396	39.297	46.965	Araip.Q20Y3	0.378
Aradu.QYX7X	0.329	327	42.683	37.805	39.634	Araip.F7YE0	0.319
Aradu.QYZ18	0.423	474	54.105	44.842	44.842	Araip.GFA2F	0.396
Aradu.QZ0ZC	0.358	790	48.04	43.742	39.697	Araip.6R0Q8	0.362
Aradu.QZ3JB	0.34	169	59.412	43.529	45.882	Araip.T2U1L	0.346
Aradu.QZ44H	0.373	733	50.545	41.144	36.512	Araip.D5K21	0.373
Aradu.QZ6DW	0.388	218	43.836	42.922	39.269	Araip.ZR8BC	0.373
Aradu.QZD25	0.374	260	43.295	40.613	45.977	Araip.7D96E	0.378
Aradu.QZS0L	0.356	2639	46.136	31.97	40.417	Araip.MT4D8	0.353
Aradu.QZS0Y	0.441	354	56.338	44.789	54.648	Araip.ATZ24	0.436
Aradu.R07DC	0.424	488	47.648	40.695	47.239	Araip.BV0ZS	0.419
Aradu.R082J	0.353	218	53.425	45.205	50.228	Araip.CL35K	0.357
Aradu.R0BCA	0.397	304	50.164	39.672	50.164	Araip.BIA41	0.401
Aradu.R0BEJ	0.307	129	43.846	40.769	39.231	Araip.B9RXI	0.305
Aradu.R0BLH	0.386	1158	51.941	35.72	40.897	Araip.I3LCA	0.383
Aradu.R0HWP	0.343	625	55.112	37.54	39.776	Araip.50K7X	0.346
Aradu.R0KJF	0.347	247	59.677	45.161	49.194	Araip.6ZS67	0.352
Aradu.R0TCR	0.424	438	49.203	45.786	52.164	Araip.BNI9P	0.426
Aradu.R0TXA	0.464	377	57.407	39.683	55.556	Araip.235AB	0.462
Aradu.R0WSE	0.378	650	53.763	43.011	36.098	Araip.5ZJ5X	0.374
Aradu.R0Z7V	0.374	375	53.457	42.021	37.234	Araip.S9MFB	0.377
Aradu.R12NH	0.438	344	59.71	46.087	56.522	Araip.5T5JE	0.438
Aradu.R17B3	0.386	683	49.854	39.035	47.953	Araip.SP8PR	0.395

Aradu.R18J1	0.347	1071	56.157	49.16	33.116	Araip.19N4V	0.355
Aradu.R1DHB	0.424	458	49.455	44.009	41.612	Araip.K3A6V	0.422
Aradu.R1E3C	0.406	474	54.947	45.474	53.053	Araip.M3RCE	0.403
Aradu.R1FC6	0.357	969	49.897	39.072	41.856	Araip.1JR2Q	0.366
Aradu.R1S51	0.371	834	49.222	37.365	47.545	Araip.T9BHU	0.385
Aradu.R1SGR	0.353	462	48.596	43.413	39.741	Araip.233C9	0.353
Aradu.R1ZAI	0.349	221	58.108	36.486	42.342	Araip.AX32F	0.344
Aradu.R205B	0.355	613	49.349	36.645	33.713	Araip.X9FAS	0.359
Aradu.R2082	0.339	598	45.743	37.396	39.065	Araip.DC3W4	0.337
Aradu.R23AB	0.362	384	54.545	36.883	36.883	Araip.M2HVA	0.347
Aradu.R23DU	0.477	562	53.286	50.622	50.799	Araip.C57WH	0.471
Aradu.R23EZ	0.322	233	49.573	29.487	33.761	Araip.IZ8TF	0.326
Aradu.R27T4	0.49	307	64.61	44.156	62.338	Araip.X44PT	0.495
Aradu.R2B96	0.445	474	49.263	42.947	61.263	Araip.JS7IQ	0.446
Aradu.R2E4D	0.437	298	60.535	50.836	53.512	Araip.1G1M0	0.441
Aradu.R2ERN	0.375	310	53.698	49.839	36.334	Araip.Y41GL	0.378
Aradu.R2K02	0.402	533	52.809	43.071	40.075	Araip.ZQ78E	0.393
Aradu.R2M0E	0.415	131	49.242	34.091	40.152	Araip.21220	0.402
Aradu.R2RMR	0.362	386	45.995	37.984	37.984	Araip.UM7ZN	0.375
Aradu.R2TG0	0.381	777	46.272	39.203	42.931	Araip.A3GFE	0.381
Aradu.R2VBI	0.369	320	56.386	38.318	49.844	Araip.BW8ER	0.371
Aradu.R2W4M	0.359	457	44.323	41.266	46.507	Araip.M0HKY	0.359
Aradu.R2YBN	0.523	401	50.746	35.323	44.527	Araip.47EMD	0.566
Aradu.R3AGE	0.417	173	51.149	37.931	44.828	Araip.LBK80	0.404
Aradu.R3EFV	0.352	1236	49.151	36.378	36.783	Araip.V7EVF	0.354
Aradu.R3QFW	0.363	695	49.282	38.649	40.086	Araip.02IFK	0.361
Aradu.R3T9A	0.315	151	61.184	48.026	37.5	Araip.DE5B2	0.324
Aradu.R3U8P	0.391	388	49.614	42.159	33.419	Araip.A4TEB	0.395
Aradu.R3US5	0.327	337	49.112	42.012	38.462	Araip.KQ9G3	0.326
Aradu.R3YL8	0.387	993	50	36.016	40.443	Araip.Z4BH6	0.392
Aradu.R3Z7V	0.403	305	50.327	35.621	39.869	Araip.Z6K8P	0.386
Aradu.R403Z	0.353	593	48.485	40.067	39.899	Araip.2I7IS	0.354
Aradu.R42GY	0.371	255	45.703	37.109	41.016	Araip.M64Q1	0.367
Aradu.R479P	0.35	303	52.961	42.105	40.132	Araip.ISI3L	0.392
Aradu.R4B3S	0.35	681	51.32	34.751	39.443	Araip.JQ9KH	0.347
Aradu.R4FBZ	0.365	304	42.623	48.197	37.377	Araip.DK1YP	0.375
Aradu.R4H3N	0.426	348	50.43	41.547	51.003	Araip.4M6FA	0.431
Aradu.R4JE4	0.372	445	52.242	41.704	41.48	Araip.03SU2	0.374
Aradu.R4JGP	0.401	162	59.509	49.693	41.718	Araip.04GN0	0.408
Aradu.R4MYV	0.407	937	52.665	42.004	37.633	Araip.ECF7H	0.403
Aradu.R4RRW	0.403	160	54.658	59.006	56.522	Araip.WVB13	0.384
Aradu.R4V51	0.367	957	45.72	33.194	41.441	Araip.S5B0I	0.378
Aradu.R4ZCN	0.363	893	50.895	44.072	37.36	Araip.XX28B	0.366
Aradu.R5E06	0.366	576	53.206	32.582	48.354	Araip.6L5T3	0.363

Aradu.R5FQX	0.348	566	50.265	34.921	36.332	Araip.BVY6Z	0.352
Aradu.R5Y58	0.414	439	46.818	40	45	Araip.IRU7H	0.41
Aradu.R5ZHP	0.396	924	51.784	44.432	47.892	Araip.12IR2	0.384
Aradu.R63W9	0.404	472	49.683	39.958	42.072	Araip.1R84J	0.401
Aradu.R64XC	0.371	492	50.913	40.365	45.233	Araip.ZJJ3R	0.365
Aradu.R659W	0.372	476	54.298	41.929	36.897	Araip.11YG0	0.371
Aradu.R65GQ	0.41	325	55.521	42.025	37.73	Araip.22DFM	0.403
Aradu.R6FI2	0.342	155	48.718	32.051	35.256	Araip.IE1UZ	0.342
Aradu.R6QT2	0.325	517	53.089	40.927	50	Araip.21BTV	0.309
Aradu.R6RRG	0.368	255	57.031	38.281	36.719	Araip.JFS3P	0.364
Aradu.R6UC6	0.351	768	52.016	42.133	34.59	Araip.U8MRA	0.355
Aradu.R6ZKE	0.397	780	57.875	44.558	34.699	Araip.X0E8P	0.4
Aradu.R71CC	0.423	306	52.443	43.648	39.739	Araip.E9VAY	0.416
Aradu.R75XI	0.368	1484	52.323	38.451	34.949	Araip.RY956	0.365
Aradu.R77I5	0.424	174	49.714	34.857	47.429	Araip.U2VM6	0.395
Aradu.R7CTG	0.421	130	45.802	48.092	35.115	Araip.3J01I	0.421
Aradu.R7JCU	0.395	209	45.238	38.095	34.762	Araip.00FG1	0.38
Aradu.R7YY4	0.366	966	53.981	40.951	33.506	Araip.40JWQ	0.367
Aradu.R83G6	0.339	466	49.679	45.182	38.758	Araip.2412K	0.339
Aradu.R8ADM	0.345	1008	55.6	37.661	39.941	Araip.ADD8R	0.343
Aradu.R8CMR	0.441	345	46.821	36.127	45.665	Araip.RD2P3	0.441
Aradu.R8LP2	0.438	739	51.486	47.703	41.081	Araip.M0GMD	0.432
Aradu.R8LXW	0.426	270	58.672	46.494	43.542	Araip.ED9LT	0.423
Aradu.R8RVW	0.394	324	53.846	43.692	40	Araip.71KZF	0.4
Aradu.R8T0J	0.397	209	52.857	37.143	46.19	Araip.13XDD	0.397
Aradu.R8UZU	0.352	901	44.346	40.798	36.475	Araip.NTQ7I	0.353
Aradu.R8YHS	0.394	469	50.851	31.702	42.128	Araip.BN787	0.39
Aradu.R92J4	0.447	409	51.22	50	53.659	Araip.VE3V9	0.442
Aradu.R95HC	0.366	451	46.018	38.053	53.097	Araip.YAQ0L	0.367
Aradu.R9HS1	0.383	1182	49.62	44.21	40.913	Araip.FB0AM	0.378
Aradu.R9NWV	0.387	610	51.227	48.609	34.206	Araip.W7R8X	0.387
Aradu.R9PSM	0.325	383	52.083	39.323	34.635	Araip.4YP5T	0.314
Aradu.R9RNU	0.352	644	49.302	43.721	37.054	Araip.PFF61	0.35
Aradu.R9T1V	0.335	266	55.056	49.438	38.951	Araip.Z4N27	0.335
Aradu.R9TCK	0.362	452	52.539	42.826	38.852	Araip.59E1I	0.376
Aradu.R9TKV	0.399	547	45.62	38.504	38.139	Araip.Y8GBE	0.398
Aradu.R9VGI	0.405	743	50.941	42.339	39.113	Araip.RDY0K	0.411
Aradu.R9XZF	0.476	300	52.824	39.203	63.123	Araip.R3TVX	0.44
Aradu.R9Y5X	0.335	712	50.771	38.569	41.234	Araip.IYS5H	0.332
Aradu.RA5XM	0.503	364	55.89	43.562	67.671	Araip.QF1TG	0.507
Aradu.RA8CW	0.407	291	45.205	39.041	48.288	Araip.LX6XT	0.388
Aradu.RA8II	0.442	252	56.126	41.897	44.664	Araip.A81Z5	0.436
Aradu.RA95M	0.36	120	52.066	41.322	35.537	Araip.N0K65	0.374
Aradu.RA9YT	0.474	142	50.35	30.769	62.937	Araip.4V6RH	0.466

Aradu.RAP3E	0.367	102	51.456	33.01	34.951	Araip.JNX15	0.354
Aradu.RAS9Y	0.358	643	55.745	42.236	55.745	Araip.J3UIZ	0.353
Aradu.RAU4M	0.4	728	47.051	37.723	42.798	Araip.9L7ER	0.399
Aradu.RAX6G	0.393	690	53.546	34.298	41.245	Araip.Z48R4	0.399
Aradu.RAZ0G	0.377	506	47.929	42.998	37.278	Araip.SI5FX	0.371
Aradu.RB14Z	0.387	358	49.304	45.682	37.883	Araip.VE967	0.395
Aradu.RB3TY	0.375	1020	50.833	37.904	34.966	Araip.ZHD9F	0.374
Aradu.RB60U	0.323	433	49.309	39.862	41.014	Araip.8R9R2	0.33
Aradu.RB83Y	0.43	1041	53.839	43.858	45.777	Araip.R4K41	0.432
Aradu.RBF27	0.379	810	51.911	38.224	38.964	Araip.R66IE	0.382
Aradu.RBQ3X	0.339	1017	48.625	39.98	39.096	Araip.9HL1N	0.337
Aradu.RBU21	0.425	511	49.023	37.891	43.555	Araip.YN0DS	0.434
Aradu.RBV6Z	0.368	159	55	43.75	43.75	Araip.66GXB	0.375
Aradu.RC5C1	0.415	109	56.364	48.182	41.818	Araip.J6ZJ6	0.434
Aradu.RC9DQ	0.399	250	51.793	23.904	43.825	Araip.I11J5	0.399
Aradu.RC9UC	0.37	862	51.333	41.367	37.428	Araip.B0B1I	0.37
Aradu.RCQ39	0.364	345	51.734	40.751	40.462	Araip.AEM8T	0.372
Aradu.RCS61	0.357	444	51.685	35.955	39.551	Araip.776JT	0.357
Aradu.RCT4K	0.429	506	53.452	40.434	46.154	Araip.L9GIV	0.429
Aradu.RCY11	0.358	352	54.958	46.459	36.544	Araip.WJ4BG	0.352
Aradu.RDK4X	0.331	502	44.135	42.942	34.195	Araip.X6DZU	0.323
Aradu.RDM6K	0.508	268	56.877	49.814	37.175	Araip.MS1SV	0.502
Aradu.RDQ06	0.432	145	52.74	44.521	54.11	Araip.UY6ZE	0.446
Aradu.RDW4B	0.389	593	46.801	57.576	47.475	Araip.EGG9T	0.393
Aradu.RE1JT	0.336	479	49.792	38.542	48.333	Araip.0P3QP	0.338
Aradu.RE8D2	0.368	279	49.643	37.857	63.929	Araip.DQ5V8	0.377
Aradu.RF2LV	0.389	314	45.079	40	46.349	Araip.BG1FH	0.366
Aradu.RF5XH	0.396	910	49.177	37.541	44.566	Araip.D30ND	0.397
Aradu.RF66L	0.409	344	56.522	42.029	37.391	Araip.0301B	0.412
Aradu.RF6XK	0.354	547	49.453	41.241	33.759	Araip.D37IR	0.359
Aradu.RF6Z3	0.386	610	52.373	38.953	39.444	Araip.KQ1XS	0.378
Aradu.RFH8Y	0.339	113	50	34.211	38.596	Araip.D6VSK	0.316
Aradu.RFQ9Z	0.389	620	53.301	37.198	36.876	Araip.U61TZ	0.399
Aradu.RFT1Y	0.458	268	46.84	44.981	45.353	Araip.CN7HI	0.455
Aradu.RG76L	0.39	642	46.19	37.325	49.3	Araip.Z6IWN	0.387
Aradu.RGH56	0.289	333	58.383	55.689	29.042	Araip.JS37L	0.295
Aradu.RGQ53	0.383	353	57.627	41.525	34.463	Araip.4R6AS	0.374
Aradu.RH8VE	0.338	253	46.457	49.606	30.315	Araip.EY77X	0.342
Aradu.RI1GB	0.44	345	44.22	43.931	36.705	Araip.HDU9M	0.436
Aradu.RI35R	0.495	404	58.765	43.457	58.272	Araip.VQ8DT	0.495
Aradu.RI6SZ	0.351	389	43.333	30.513	42.308	Araip.PGI83	0.351
Aradu.RII5F	0.415	205	57.282	41.748	34.466	Araip.B5KKJ	0.424
Aradu.RIZ04	0.366	334	51.343	37.612	34.328	Araip.K33N8	0.366
Aradu.RJ0UE	0.35	343	42.151	35.465	37.791	Araip.ZP5MJ	0.358

Aradu.RJ2CI	0.398	683	53.801	43.275	36.696	Araip.FXA4L	0.403
Aradu.RJ6JS	0.398	513	46.693	36.576	42.802	Araip.ZQ3Q0	0.395
Aradu.RJA7P	0.369	573	47.909	41.115	39.024	Araip.S0TNW	0.367
Aradu.RKH9D	0.355	223	49.107	50	48.214	Araip.ZG28U	0.374
Aradu.RKM5L	0.402	567	51.408	42.43	38.204	Araip.J6CZK	0.406
Aradu.RL2B3	0.384	225	54.425	39.381	46.018	Araip.6S5NP	0.384
Aradu.RL2YG	0.358	126	37.008	40.157	39.37	Araip.XV7Z4	0.353
Aradu.RLB9U	0.357	468	49.467	43.71	41.791	Araip.J11AY	0.357
Aradu.RLX6T	0.378	413	56.28	51.691	35.507	Araip.QNY9B	0.374
Aradu.RLZ61	0.448	160	57.143	44.72	69.565	Araip.3X35T	0.448
Aradu.RM1RY	0.384	284	46.667	39.649	38.596	Araip.QUZ7L	0.38
Aradu.RM26Y	0.513	162	57.669	44.785	60.123	Araip.D0TT7	0.519
Aradu.RM7GK	0.401	187	46.277	42.021	46.809	Araip.22IQY	0.376
Aradu.RMG12	0.415	204	51.22	46.829	46.829	Araip.T5YYS	0.421
Aradu.RN1PL	0.356	319	56.25	41.875	38.125	Araip.8YC75	0.359
Aradu.RN1V3	0.375	242	57.202	40.741	41.564	Araip.608WW	0.392
Aradu.RP1BS	0.405	157	51.899	34.177	41.139	Araip.0D136	0.392
Aradu.RP5AZ	0.406	414	53.253	40.964	37.59	Araip.C6G0D	0.411
Aradu.RP61F	0.391	306	51.466	41.368	45.928	Araip.PNX61	0.398
Aradu.RP6HI	0.372	169	52.941	48.824	34.118	Araip.Y6RE5	0.372
Aradu.RQ0GF	0.358	1083	48.432	44.004	35.332	Araip.SVN4N	0.355
Aradu.RQ3SQ	0.406	358	49.582	35.097	39.276	Araip.VL5WX	0.388
Aradu.RQ5UT	0.358	1075	53.067	40.056	38.104	Araip.V99DX	0.354
Aradu.RQ7SQ	0.483	217	50	35.78	50	Araip.EDA7H	0.483
Aradu.RQ9JK	0.414	237	46.639	43.697	42.857	Araip.Z4EB4	0.414
Aradu.RQH55	0.4	659	47.424	38.333	36.97	Araip.J2LHI	0.388
Aradu.RQK7Y	0.382	292	52.218	34.13	37.543	Araip.T9C3C	0.398
Aradu.RQQ24	0.39	242	55.967	40.329	50.206	Araip.PF76P	0.386
Aradu.RR75T	0.359	667	47.904	36.527	44.012	Araip.BCQ7T	0.365
Aradu.RRT07	0.238	202	45.32	40.394	41.872	Araip.V4DIY	0.27
Aradu.RS6VM	0.38	428	51.282	41.725	41.958	Araip.L3LJP	0.383
Aradu.RSV1J	0.376	787	51.904	41.878	50.888	Araip.Q0ZQ7	0.405
Aradu.RT9KH	0.423	276	56.318	37.906	43.682	Araip.34S35	0.419
Aradu.RTQ8K	0.393	897	46.659	39.31	39.198	Araip.XKH8G	0.393
Aradu.RU1MG	0.33	764	47.059	39.477	46.667	Araip.9H8RU	0.332
Aradu.RU466	0.361	690	53.401	37.916	40.81	Araip.DW1CY	0.352
Aradu.RU8ZD	0.373	441	52.036	40.498	50.452	Araip.1P6L4	0.372
Aradu.RUD1R	0.414	542	51.197	41.436	53.591	Araip.G5J45	0.411
Aradu.RUK3P	0.451	459	52.391	44.348	60	Araip.3F4LC	0.454
Aradu.RUS0X	0.365	291	55.822	39.384	36.986	Araip.P01W2	0.355
Aradu.RUT6G	0.364	545	51.099	43.407	34.982	Araip.P4TWL	0.362
Aradu.RV5XK	0.465	162	58.282	42.331	61.35	Araip.X7Z10	0.465
Aradu.RV62R	0.462	212	53.052	62.441	47.418	Araip.YJ68E	0.467
Aradu.RV6BG	0.35	467	45.299	43.376	34.615	Araip.IWB76	0.347

Aradu.RV9GW	0.384	941	47.877	36.943	45.011	Araip.M7L7S	0.372
Aradu.RV9UM	0.394	280	51.601	40.569	55.516	Araip.UQ6YY	0.39
Aradu.RVF1V	0.388	403	50.495	38.861	34.901	Araip.2D8LN	0.404
Aradu.RW69W	0.364	603	50.662	41.722	33.113	Araip.6LB90	0.354
Aradu.RW8B0	0.392	229	51.304	36.087	43.913	Araip.HBQ1U	0.397
Aradu.RW91L	0.358	419	46.905	43.095	39.762	Araip.MC3E6	0.35
Aradu.RW9W8	0.412	319	55.937	37.188	39.063	Araip.TY0LX	0.412
Aradu.RWI1K	0.436	636	54.16	43.956	63.422	Araip.RK7QB	0.442
Aradu.RWZ7G	0.369	520	43.378	42.802	41.267	Araip.8C9BN	0.367
Aradu.RWZ7N	0.377	432	54.042	40.647	31.871	Araip.FN9H2	0.372
Aradu.RX5BN	0.379	676	54.653	38.7	36.632	Araip.TYP36	0.384
Aradu.RXW02	0.424	425	55.164	39.437	45.54	Araip.H8UEI	0.43
Aradu.RY2KR	0.402	532	55.722	38.274	42.589	Araip.8AA3H	0.412
Aradu.RY508	0.386	336	51.335	37.685	47.478	Araip.B0RQS	0.38
Aradu.RY6HH	0.345	460	44.902	39.046	35.358	Araip.S0JW5	0.348
Aradu.RY6N7	0.411	202	60.591	37.438	57.143	Araip.WC7UB	0.406
Aradu.RY9YC	0.39	237	57.563	41.176	36.555	Araip.19TXN	0.377
Aradu.RYB7F	0.374	1259	47.778	35.238	38.492	Araip.11Y3M	0.376
Aradu.RYQ8I	0.425	201	53.96	40.594	37.129	Araip.WUR54	0.421
Aradu.RYS1S	0.345	461	49.567	39.177	42.857	Araip.DNQ5K	0.35
Aradu.RZ908	0.367	635	53.145	31.132	37.264	Araip.15C6I	0.365
Aradu.RZY5Q	0.345	1070	49.3	37.162	33.333	Araip.BK6T6	0.343
Aradu.RZZ9E	0.377	573	46.167	40.592	40.244	Araip.6800W	0.368
Aradu.S0307	0.286	153	50	27.273	35.714	Araip.4FT5T	0.322
Aradu.S056J	0.444	257	50.388	46.512	42.636	Araip.L9LM7	0.426
Aradu.S073D	0.36	629	45.873	39.683	48.571	Araip.HTP4Q	0.349
Aradu.S0871	0.409	296	47.138	42.761	53.535	Araip.Q2VVS	0.407
Aradu.S0DLC	0.328	744	41.745	34.094	35.973	Araip.D942R	0.308
Aradu.S0HMQ	0.414	634	47.244	39.528	57.008	Araip.0T4FD	0.414
Aradu.S0JY2	0.383	416	43.165	37.41	48.921	Araip.YP690	0.362
Aradu.S0K8G	0.355	386	57.106	36.693	42.377	Araip.K67IB	0.349
Aradu.S0KU9	0.405	322	49.226	37.461	45.82	Araip.PVV4Q	0.419
Aradu.S0NBF	0.439	538	49.351	45.826	61.039	Araip.S398P	0.42
Aradu.S0S2R	0.389	562	45.115	38.011	34.636	Araip.Y28R2	0.399
Aradu.S0SM3	0.438	267	40.299	39.552	48.134	Araip.EVY0P	0.45
Aradu.S0XAG	0.381	430	58.237	50.812	51.74	Araip.P5CS5	0.402
Aradu.S0XYN	0.375	265	48.496	41.729	27.444	Araip.J8XTW	0.381
Aradu.S0YHI	0.399	288	58.131	43.945	38.062	Araip.P48NJ	0.413
Aradu.S111G	0.412	301	56.291	46.358	43.377	Araip.N3P4J	0.418
Aradu.S13AZ	0.406	223	51.339	42.411	41.964	Araip.GV2U7	0.42
Aradu.S14WF	0.374	481	51.867	43.568	40.664	Araip.826U2	0.389
Aradu.S156I	0.411	394	53.671	44.051	46.076	Araip.PEC40	0.407
Aradu.S166E	0.411	206	49.275	32.85	49.758	Araip.L82PP	0.411
Aradu.S16C5	0.394	321	50	35.093	40.373	Araip.Y8EDR	0.39

Aradu.S1HRU	0.348	531	59.586	43.609	39.098	Araip.FD42N	0.345
Aradu.S1SNW	0.408	474	54.947	42.947	36.632	Araip.U9LFD	0.408
Aradu.S1UZ6	0.335	246	44.939	37.652	37.247	Araip.1H3PC	0.335
Aradu.S20WA	0.388	323	53.395	44.444	36.111	Araip.PCN2L	0.394
Aradu.S226I	0.331	410	44.282	43.066	34.063	Araip.A0AYC	0.335
Aradu.S28UE	0.425	589	47.119	39.831	40.678	Araip.6NP7W	0.448
Aradu.S2HHA	0.347	391	42.857	40.051	40.306	Araip.893D0	0.355
Aradu.S2IZB	0.518	202	49.754	46.305	52.709	Araip.V9N10	0.523
Aradu.S2K0Q	0.354	704	53.333	35.603	40.567	Araip.WN5VI	0.354
Aradu.S2S1T	0.38	792	43.632	38.335	41.866	Araip.EH219	0.383
Aradu.S2UYW	0.404	587	45.918	40.646	40.136	Araip.U91QP	0.404
Aradu.S2VPA	0.427	464	50.108	47.527	37.204	Araip.4B8IV	0.432
Aradu.S37LQ	0.388	156	57.325	37.58	42.675	Araip.T9DPL	0.388
Aradu.S38FI	0.315	342	44.315	39.65	24.781	Araip.NJ5S3	0.321
Aradu.S3AS8	0.417	721	51.524	39.197	52.493	Araip.WU7FZ	0.414
Aradu.S3CLD	0.367	105	48.113	28.302	41.509	Araip.MU3T5	0.406
Aradu.S3DH3	0.327	347	49.713	31.897	42.241	Araip.GY1A9	0.332
Aradu.S3HBK	0.379	588	46.18	38.2	40.917	Araip.Q7WA8	0.384
Aradu.S3MQ8	0.374	275	50.725	47.826	38.406	Araip.5VP72	0.368
Aradu.S3UJF	0.411	219	49.545	42.273	38.636	Araip.W1QI3	0.408
Aradu.S3YJN	0.338	245	36.585	47.154	34.553	Araip.X2SJJ	0.335
Aradu.S44IV	0.474	360	52.909	43.767	52.632	Araip.VKU4A	0.482
Aradu.S46KB	0.398	1100	47.956	36.058	37.965	Araip.965G6	0.398
Aradu.S4DGV	0.445	312	48.882	44.089	56.55	Araip.90YM8	0.447
Aradu.S4LWP	0.453	291	49.658	40.411	43.836	Araip.IXI9R	0.453
Aradu.S4P4Y	0.476	193	57.216	51.031	60.309	Araip.5MF6L	0.471
Aradu.S4UH1	0.467	138	49.64	49.64	38.129	Araip.AD1AP	0.467
Aradu.S4V52	0.463	250	51.793	42.231	67.331	Araip.N2TWA	0.463
Aradu.S4XSK	0.344	538	47.866	39.518	42.301	Araip.HP1ES	0.352
Aradu.S514R	0.395	478	49.687	49.061	37.37	Araip.V8L3R	0.395
Aradu.S54FQ	0.365	713	46.078	37.815	44.538	Araip.2J0CW	0.361
Aradu.S591T	0.399	484	47.01	39.794	42.062	Araip.D29UN	0.379
Aradu.S59LW	0.382	261	47.328	36.641	50.763	Araip.GN6XE	0.386
Aradu.S59W7	0.362	451	53.097	37.389	38.053	Araip.V3RAV	0.365
Aradu.S5K3Z	0.395	527	56.629	37.689	43.182	Araip.J5GX8	0.399
Aradu.S5T1J	0.369	765	51.567	41.253	39.817	Araip.RM73I	0.375
Aradu.S5UQ2	0.391	704	46.667	38.014	35.745	Araip.7EU1J	0.393
Aradu.S5XS2	0.385	768	52.016	42.913	36.541	Araip.22Y09	0.394
Aradu.S6195	0.44	451	52.876	43.142	35.841	Araip.KVK3X	0.425
Aradu.S6366	0.357	466	43.255	42.827	38.116	Araip.SI63P	0.351
Aradu.S63UK	0.444	1231	52.273	31.331	42.451	Araip.7F16U	0.443
Aradu.S66GY	0.379	186	55.08	37.968	47.594	Araip.1S7CN	0.36
Aradu.S68YF	0.454	319	56.875	39.375	61.875	Araip.2A4VP	0.463
Aradu.S69T7	0.402	457	43.668	46.288	38.21	Araip.J0KBV	0.411

Aradu.S6CPD	0.396	205	49.515	47.573	41.262	Araip.K4HRJ	0.396
Aradu.S6EUF	0.365	295	52.027	46.622	33.784	Araip.N59L2	0.349
Aradu.S6F46	0.44	372	46.381	37.534	47.721	Araip.TME8J	0.435
Aradu.S6JX1	0.38	323	50.309	38.272	49.383	Araip.5Y1QQ	0.374
Aradu.S6TIM	0.418	374	50.133	41.333	43.2	Araip.38345	0.4
Aradu.S70AZ	0.38	494	44.646	41.01	38.586	Araip.ADR3S	0.385
Aradu.S7CRC	0.371	1248	51.882	40.753	41.473	Araip.TK8K9	0.371
Aradu.S7JJM	0.423	431	46.296	41.204	40.741	Araip.953XL	0.422
Aradu.S7NAJ	0.362	401	51.244	33.582	46.766	Araip.5Y6S2	0.397
Aradu.S7P2G	0.37	1111	53.507	42.356	39.748	Araip.TIT4D	0.372
Aradu.S7Q9G	0.375	1376	50.109	42.266	35.875	Araip.54FE9	0.378
Aradu.S7WZG	0.43	146	56.463	49.66	43.537	Araip.T7PRK	0.447
Aradu.S7YD6	0.402	540	44.547	45.841	35.49	Araip.RC4R7	0.388
Aradu.S894K	0.415	308	47.249	37.217	32.686	Araip.JR3QJ	0.408
Aradu.S89VI	0.361	602	56.219	40.299	35.821	Araip.S9I8N	0.364
Aradu.S8E6L	0.388	234	51.489	42.979	41.277	Araip.IGH7A	0.396
Aradu.S8IEI	0.374	707	59.04	38.418	37.571	Araip.NLX79	0.378
Aradu.S8IWK	0.392	459	48.913	44.783	37.826	Araip.CD8S3	0.401
Aradu.S8QXC	0.398	636	48.823	47.724	35.165	Araip.1BW4F	0.4
Aradu.S8SZ6	0.471	531	46.992	39.662	52.444	Araip.48P6B	0.462
Aradu.S8XF6	0.357	464	47.742	39.57	43.441	Araip.ZDG59	0.368
Aradu.S8Y3J	0.319	636	51.491	43.014	35.793	Araip.5CL0B	0.331
Aradu.S94H9	0.357	396	50.126	43.325	44.332	Araip.NVV81	0.357
Aradu.S94TG	0.345	647	47.994	37.654	39.352	Araip.QE1LN	0.33
Aradu.S9BZU	0.378	201	51.98	39.604	36.139	Araip.PY45A	0.378
Aradu.S9CWT	0.392	461	49.567	40.693	35.931	Araip.60YE7	0.385
Aradu.S9LIL	0.377	165	59.639	43.373	56.024	Araip.1Q3SQ	0.37
Aradu.S9NLD	0.364	954	50.576	34.66	39.058	Araip.L0EG5	0.365
Aradu.S9P8W	0.396	156	55.414	38.217	51.592	Araip.WG7BV	0.413
Aradu.S9TS8	0.371	334	43.582	40.299	42.985	Araip.RQ3D1	0.353
Aradu.SA3GU	0.332	287	49.306	40.625	37.847	Araip.KR2BY	0.365
Aradu.SA9NZ	0.43	323	54.63	40.741	44.753	Araip.VR8J9	0.42
Aradu.SAM0J	0.365	295	50	38.851	37.162	Araip.W7LWH	0.341
Aradu.SAY01	0.346	595	45.973	32.886	33.221	Araip.5R1U0	0.342
Aradu.SB3IS	0.386	384	51.429	38.182	43.117	Araip.GE5YY	0.381
Aradu.SB5CI	0.415	198	50.754	31.658	53.769	Araip.BF1SB	0.42
Aradu.SB62Q	0.319	100	44.554	47.525	50.495	Araip.LZ646	0.355
Aradu.SB9IZ	0.386	710	50.492	34.318	46.414	Araip.K6VSE	0.385
Aradu.SBA69	0.37	671	50.893	42.708	36.012	Araip.626V8	0.36
Aradu.SBB2J	0.355	461	46.753	33.766	39.177	Araip.IE5FR	0.368
Aradu.SBS1U	0.365	492	56.998	40.974	44.625	Araip.4JZ1Y	0.371
Aradu.SBU2D	0.439	244	53.878	48.163	41.224	Araip.8LI4K	0.439
Aradu.SC3I7	0.564	170	53.801	46.784	47.953	Araip.MA8U3	0.566
Aradu.SC4Y7	0.454	124	54.4	38.4	41.6	Araip.S1N8M	0.429

Aradu.SC5U0	0.358	536	49.907	39.665	38.361	Araip.Y5AAV	0.363
Aradu.SC6RY	0.501	371	60.484	49.194	63.441	Araip.QC09Z	0.501
Aradu.SC9VF	0.296	109	41.818	46.364	40	Araip.Q0WU6	0.323
Aradu.SCA2Z	0.379	482	50.104	35.404	43.685	Araip.A1530	0.381
Aradu.SCF1F	0.346	133	47.761	33.582	38.806	Araip.ZEX5T	0.339
Aradu.SCG5D	0.35	476	44.654	41.509	31.447	Araip.NXU71	0.351
Aradu.SCK30	0.318	209	47.143	36.19	41.905	Araip.37S9E	0.287
Aradu.SCR9A	0.396	357	60.056	41.62	38.268	Araip.RHZ7C	0.4
Aradu.SD45B	0.397	382	48.825	42.82	42.298	Araip.7H6FH	0.409
Aradu.SDL7M	0.39	491	47.967	41.87	37.805	Araip.Z7025	0.39
Aradu.SDR46	0.343	890	51.291	49.383	33.67	Araip.GIN3T	0.34
Aradu.SE3WE	0.365	508	48.33	38.507	39.686	Araip.RT6FK	0.373
Aradu.SE6Y8	0.405	278	51.971	35.842	54.122	Araip.8IH70	0.409
Aradu.SE8E9	0.427	278	45.878	37.276	51.971	Araip.0JU1Y	0.481
Aradu.SEJ3V	0.411	362	50.964	34.435	44.353	Araip.VS7G2	0.409
Aradu.SF38I	0.342	320	46.106	37.695	41.745	Araip.L2AZX	0.353
Aradu.SF9ZV	0.419	111	52.679	38.393	40.179	Araip.BN5L7	0.394
Aradu.SFF9P	0.388	347	52.874	40.23	37.356	Araip.YCH8N	0.379
Aradu.SG4XX	0.411	104	44.762	38.095	42.857	Araip.SU3H2	0.387
Aradu.SG5AP	0.398	202	51.232	41.379	44.828	Araip.JY8IW	0.398
Aradu.SG5CY	0.295	583	49.315	38.87	33.733	Araip.RU3FR	0.295
Aradu.SG5SM	0.401	149	49.333	40.667	34	Araip.0D7QS	0.466
Aradu.SG94C	0.383	125	43.651	34.921	33.333	Araip.7DQ34	0.357
Aradu.SG97P	0.326	504	47.525	34.059	34.257	Araip.9KU8R	0.329
Aradu.SGK85	0.42	121	48.361	35.246	51.639	Araip.W5V9C	0.41
Aradu.SGR1V	0.388	284	57.544	44.561	49.123	Araip.JG35V	0.391
Aradu.SH1J0	0.389	323	51.852	48.148	41.358	Araip.GIZ0F	0.402
Aradu.SH1QV	0.313	337	40.828	43.491	27.515	Araip.BL3I0	0.313
Aradu.SH3UZ	0.449	269	47.037	49.259	54.444	Araip.U8V9W	0.463
Aradu.SH4SS	0.369	291	51.712	47.603	38.356	Araip.K7F8Y	0.387
Aradu.SH4VE	0.402	377	62.963	44.444	34.656	Araip.D5TXM	0.396
Aradu.SH6P7	0.398	323	49.074	34.568	40.432	Araip.8GH2C	0.397
Aradu.SHC8B	0.38	325	46.626	40.491	35.276	Araip.0BB7B	0.38
Aradu.SHF2C	0.332	600	52.08	36.938	41.431	Araip.8K33E	0.342
Aradu.SHH9J	0.468	389	52.821	48.205	52.308	Araip.N2170	0.468
Aradu.SI0X3	0.313	477	48.326	38.075	37.866	Araip.AV5UM	0.315
Aradu.SI3S6	0.336	111	40.179	25.893	47.321	Araip.EDG2M	0.343
Aradu.SJ15T	0.415	454	47.033	45.055	45.055	Araip.NK3C5	0.411
Aradu.SJ2KY	0.328	332	48.649	35.135	33.333	Araip.SBC75	0.332
Aradu.SJ6MW	0.342	198	57.286	36.181	39.698	Araip.1F7P8	0.346
Aradu.SJ7I8	0.368	659	48.333	32.879	43.788	Araip.26QE5	0.37
Aradu.SJ887	0.416	208	44.019	41.627	53.11	Araip.63HRP	0.408
Aradu.SJK47	0.4	578	53.022	39.033	44.732	Araip.Q5RAQ	0.4
Aradu.SJR7G	0.327	445	55.381	34.978	37.892	Araip.FU5J9	0.325

Aradu.SJX08	0.387	621	51.125	29.743	33.28	Araip.8V9X0	0.385
Aradu.SK1HP	0.383	547	49.453	40.146	36.314	Araip.ZH461	0.382
Aradu.SK7RE	0.474	102	43.689	36.893	57.282	Araip.ISZ7W	0.485
Aradu.SK8K0	0.473	152	50.327	49.673	54.902	Araip.HE7HA	0.486
Aradu.SK8YF	0.387	358	52.089	45.404	43.454	Araip.CL7PQ	0.394
Aradu.SK9II	0.37	424	51.765	39.765	43.059	Araip.68UU0	0.371
Aradu.SKC35	0.405	584	56.41	42.051	48.205	Araip.3LL8X	0.403
Aradu.SKW70	0.401	325	57.362	45.706	43.865	Araip.C53Q0	0.385
Aradu.SL05Q	0.362	302	41.584	43.564	39.274	Araip.ZWB49	0.375
Aradu.SL2QQ	0.32	314	49.841	33.016	37.46	Araip.2HD3N	0.335
Aradu.SL3YH	0.378	1095	47.81	43.613	34.489	Araip.ESV2B	0.375
Aradu.SL6TQ	0.351	767	50.781	40.495	33.984	Araip.Y3EN4	0.351
Aradu.SL9AV	0.428	396	47.103	45.844	39.043	Araip.14067	0.447
Aradu.SLM5J	0.358	463	50.862	40.517	39.655	Araip.LV17J	0.362
Aradu.SM6NC	0.381	246	50.607	34.413	42.105	Araip.TG6SS	0.381
Aradu.SM8C1	0.392	843	46.919	34.36	44.076	Araip.TQ2BR	0.387
Aradu.SM8ND	0.411	517	54.44	45.753	43.436	Araip.T2D4X	0.417
Aradu.SMR94	0.448	184	47.568	38.919	45.405	Araip.27R71	0.436
Aradu.SMW23	0.407	593	48.148	43.434	36.027	Araip.SE3MC	0.404
Aradu.SN12S	0.364	762	53.08	36.042	41.284	Araip.D1L9A	0.363
Aradu.SN5BS	0.37	789	47.089	36.709	45.19	Araip.T42NE	0.369
Aradu.SNT27	0.405	378	52.77	39.314	41.425	Araip.3U4DB	0.404
Aradu.SP3D0	0.432	284	49.474	34.386	40.351	Araip.LDM4X	0.44
Aradu.SP6EC	0.333	304	47.869	34.754	32.787	Araip.1H6L7	0.345
Aradu.SP6QR	0.428	310	58.521	46.945	59.807	Araip.FL27H	0.434
Aradu.SP7U9	0.357	286	52.265	43.902	36.934	Araip.34I8K	0.36
Aradu.SP99D	0.492	408	55.257	44.01	64.059	Araip.S7CAX	0.516
Aradu.SPH1K	0.356	779	44.744	36.41	37.308	Araip.7D5HE	0.356
Aradu.SPS87	0.345	374	46.933	39.2	39.733	Araip.Z3KU8	0.345
Aradu.SQ2QT	0.388	390	45.013	42.199	40.665	Araip.0Q5CU	0.385
Aradu.SQD67	0.396	534	48.224	43.178	46.916	Araip.F7NGT	0.4
Aradu.SQK9K	0.387	323	48.457	42.284	40.741	Araip.LXR9M	0.39
Aradu.SR17F	0.348	530	48.588	39.736	34.087	Araip.V2S44	0.345
Aradu.SR2IB	0.351	911	46.491	32.566	33.114	Araip.6MG4Z	0.339
Aradu.SR3CA	0.386	365	57.377	38.798	45.355	Araip.HX4H1	0.384
Aradu.SRM8N	0.426	455	49.561	49.781	34.649	Araip.D6MDF	0.427
Aradu.SRY7A	0.371	235	43.22	46.61	40.254	Araip.Z6CDS	0.377
Aradu.SS76M	0.349	432	51.963	42.263	30.485	Araip.WK203	0.346
Aradu.SS7YL	0.383	607	51.151	37.993	43.586	Araip.RV2U5	0.372
Aradu.SSH0X	0.4	1218	49.713	40.771	33.47	Araip.4WJ5B	0.401
Aradu.SSI0J	0.315	344	45.507	35.652	26.957	Araip.E7C4C	0.318
Aradu.ST3KE	0.42	1071	46.922	40.578	43.19	Araip.0AN9G	0.426
Aradu.ST5ZJ	0.414	425	48.592	40.845	52.113	Araip.X1ZFF	0.412
Aradu.STU80	0.382	513	59.533	38.132	53.113	Araip.0K3NF	0.38

Aradu.SUD1N	0.348	1142	49.431	39.808	34.821	Araip.V76LN	0.346
Aradu.SUN9W	0.381	584	49.573	39.658	47.521	Araip.HY2YI	0.381
Aradu.SUY1C	0.42	430	44.316	41.995	45.244	Araip.Z9UVM	0.41
Aradu.SV33Z	0.418	361	51.934	45.304	37.845	Araip.6T3P4	0.414
Aradu.SW5JR	0.456	524	50.476	43.81	53.143	Araip.K9VA5	0.457
Aradu.SW9YM	0.341	222	54.26	46.188	30.493	Araip.DW1WN	0.346
Aradu.SX4F9	0.324	452	48.344	34.879	40.839	Araip.I6C5W	0.325
Aradu.SX6G4	0.418	191	51.563	33.333	48.438	Araip.5CP68	0.418
Aradu.SXB7Z	0.389	709	49.155	42.958	39.296	Araip.KV8TN	0.381
Aradu.SXB8E	0.368	194	53.333	41.026	55.897	Araip.GTM84	0.357
Aradu.SXL5T	0.388	446	51.007	38.702	40.716	Araip.SJY96	0.39
Aradu.SY0M5	0.365	1065	50	37.523	44.278	Araip.HIR6V	0.367
Aradu.SY356	0.357	449	49.333	44.889	36.889	Araip.TU2YQ	0.351
Aradu.SY6IP	0.361	558	50.626	41.145	34.884	Araip.8PW0Z	0.366
Aradu.SYI1K	0.352	415	56.49	33.413	41.106	Araip.98Q8H	0.357
Aradu.SYY8Y	0.436	318	44.828	40.439	36.364	Araip.9C7ZQ	0.429
Aradu.SZ07F	0.519	132	51.128	33.083	67.669	Araip.B1VRH	0.512
Aradu.SZ9LD	0.423	108	43.119	54.128	37.615	Araip.V6F8I	0.413
Aradu.T01MJ	0.417	552	46.293	45.75	38.879	Araip.14YQZ	0.42
Aradu.T034F	0.441	431	59.491	46.065	40.046	Araip.B0LR2	0.431
Aradu.T08NC	0.384	414	48.434	42.41	40.482	Araip.5J1JM	0.375
Aradu.T09Z3	0.41	276	47.292	38.628	44.404	Araip.2D0HD	0.41
Aradu.T0IJJ	0.36	311	56.41	33.013	37.179	Araip.K2Q3T	0.363
Aradu.T0K7A	0.34	352	51.558	37.394	37.96	Araip.UF0MQ	0.362
Aradu.T0LS0	0.471	494	52.323	42.626	69.091	Araip.XFW7H	0.47
Aradu.T0NTP	0.424	365	50.273	42.077	48.087	Araip.Q8342	0.428
Aradu.T0R6F	0.41	189	37.895	38.421	38.421	Araip.FSA5T	0.395
Aradu.T0VC2	0.39	640	50.39	42.746	37.598	Araip.2W29K	0.385
Aradu.T0VLB	0.367	665	48.949	40.24	36.186	Araip.VX608	0.37
Aradu.T0YTZ	0.369	954	50.262	38.639	39.372	Araip.B73E0	0.377
Aradu.T0ZKV	0.449	216	54.839	72.35	43.318	Araip.2M1DM	0.444
Aradu.T12XR	0.447	1039	47.115	39.423	59.038	Araip.E75HV	0.45
Aradu.T1478	0.396	593	49.832	43.939	43.434	Araip.K2TUF	0.39
Aradu.T19XF	0.424	313	48.408	42.994	41.083	Araip.N8HQ9	0.434
Aradu.T1G5I	0.525	140	53.191	60.993	51.773	Araip.J1I10	0.513
Aradu.T1G99	0.395	682	51.684	42.46	35.432	Araip.YJC1H	0.408
Aradu.T1J1B	0.403	383	55.729	38.802	41.406	Araip.XRY2X	0.403
Aradu.T1JCL	0.395	796	53.45	43.287	45.671	Araip.LQ3KM	0.397
Aradu.T1K5W	0.341	515	47.481	37.791	43.798	Araip.PE948	0.348
Aradu.T1Q4B	0.46	349	55.429	51.143	57.714	Araip.HD55C	0.467
Aradu.T1ZHN	0.387	193	64.948	14.433	23.196	Araip.812WS	0.395
Aradu.T1ZRM	0.493	294	51.525	54.576	53.898	Araip.6L6QM	0.496
Aradu.T25QT	0.384	255	40.234	36.328	43.359	Araip.P6G60	0.397
Aradu.T2BI1	0.382	292	51.195	41.98	37.884	Araip.76R9S	0.385

Aradu.T2I1E	0.432	365	54.372	36.066	51.639	Araip.J3PJ2	0.431
Aradu.T2QCP	0.363	690	48.48	40.81	38.35	Araip.Q00US	0.36
Aradu.T2QMK	0.405	253	42.126	44.882	46.85	Araip.6U899	0.402
Aradu.T2VBJ	0.382	1105	46.835	36.257	38.879	Araip.BNK4F	0.382
Aradu.T2X3V	0.382	966	55.636	42.296	36.505	Araip.5JD7H	0.384
Aradu.T2XD3	0.387	490	52.342	37.271	39.715	Araip.VXN6M	0.382
Aradu.T337U	0.408	400	50.125	35.91	36.908	Araip.6NV0D	0.396
Aradu.T346F	0.404	733	54.36	43.869	36.512	Araip.DY7BJ	0.413
Aradu.T35QD	0.357	280	42.705	35.231	35.587	Araip.9J1EH	0.346
Aradu.T35UC	0.446	580	51.635	43.373	45.611	Araip.S6815	0.448
Aradu.T37GN	0.397	1017	51.375	37.819	42.436	Araip.K7EZ3	0.396
Aradu.T3K2L	0.368	720	52.843	51.456	37.171	Araip.QHS45	0.356
Aradu.T3LBA	0.366	446	47.204	44.072	35.347	Araip.H2PQ2	0.357
Aradu.T3QX0	0.411	640	49.454	36.817	44.774	Araip.J86TR	0.405
Aradu.T3VDH	0.415	287	60.417	39.583	46.528	Araip.QP2XD	0.401
Aradu.T3ZAX	0.46	469	46.383	40.638	32.34	Araip.X95V4	0.453
Aradu.T45BF	0.474	156	53.503	49.045	43.312	Araip.B162Z	0.474
Aradu.T46DH	0.366	296	49.832	34.68	42.424	Araip.MMC70	0.359
Aradu.T4HI5	0.357	437	56.164	36.301	40.639	Araip.2G506	0.36
Aradu.T4PBI	0.366	343	42.442	35.465	40.698	Araip.778BR	0.384
Aradu.T4VLF	0.388	967	50.31	42.252	37.19	Araip.Y4AFN	0.386
Aradu.T5087	0.411	723	55.249	39.641	47.099	Araip.2S2EB	0.412
Aradu.T52CY	0.424	405	45.32	47.044	40.64	Araip.IC9FD	0.424
Aradu.T52PW	0.385	445	46.188	36.323	43.722	Araip.A84G4	0.396
Aradu.T55NY	0.384	779	50.128	36.538	48.077	Araip.M1JS2	0.349
Aradu.T5CVQ	0.345	475	42.857	33.403	36.345	Araip.1F56R	0.338
Aradu.T5NFD	0.381	110	50.45	42.342	54.955	Araip.564FB	0.387
Aradu.T5PPZ	0.431	300	54.485	35.88	46.512	Araip.YQ21T	0.43
Aradu.T5RWU	0.406	414	47.229	39.518	44.819	Araip.Y9H0E	0.407
Aradu.T5UQE	0.429	1427	54.482	51.821	42.297	Araip.K6XKE	0.431
Aradu.T5WNW	0.355	769	43.247	33.896	38.571	Araip.Z9LLX	0.378
Aradu.T65KX	0.421	351	50.852	47.727	48.295	Araip.47NV6	0.432
Aradu.T66QJ	0.367	858	53.783	39.464	45.867	Araip.BH6DK	0.4
Aradu.T693K	0.362	510	50.881	38.943	42.27	Araip.FP06E	0.355
Aradu.T698T	0.429	187	52.66	34.574	42.553	Araip.X25NR	0.431
Aradu.T6EFY	0.371	176	52.542	43.503	46.328	Araip.B1ALG	0.371
Aradu.T6HF1	0.394	827	51.208	38.406	47.947	Araip.T68FX	0.399
Aradu.T6HSI	0.371	521	50.766	42.146	30.46	Araip.C77KQ	0.36
Aradu.T6KM7	0.377	298	53.512	33.779	46.823	Araip.K77RU	0.37
Aradu.T6VPT	0.427	649	48.462	44.923	52.154	Araip.QW1VD	0.421
Aradu.T6XMF	0.315	387	47.938	38.144	30.928	Araip.D0D33	0.313
Aradu.T6XQA	0.385	324	42.462	33.538	36	Araip.Q2MIL	0.383
Aradu.T720W	0.413	404	53.333	43.457	56.296	Araip.8UW3Z	0.435
Aradu.T7BAA	0.345	181	58.791	43.407	33.516	Araip.ZQB6E	0.339

Aradu.T7CCI	0.359	463	48.922	43.534	47.629	Araip.SV4TB	0.361
Aradu.T7E55	0.395	329	55.152	45.152	57.273	Araip.UI4ZB	0.395
Aradu.T7EMW	0.364	909	47.143	35.824	45.165	Araip.4DP2E	0.362
Aradu.T7J8U	0.365	660	50.681	37.216	40.393	Araip.C1Y5Y	0.36
Aradu.T7MUN	0.387	554	47.207	42.162	40.721	Araip.00Q3S	0.382
Aradu.T7TUD	0.436	389	40.513	36.154	41.538	Araip.IDM52	0.43
Aradu.T7YJG	0.418	263	52.273	60.985	46.97	Araip.WT200	0.418
Aradu.T82FQ	0.319	682	47.731	35.286	43.924	Araip.E02KZ	0.314
Aradu.T83RP	0.402	843	48.223	42.654	44.313	Araip.39QP6	0.402
Aradu.T8C1S	0.436	495	48.387	41.734	52.621	Araip.Y6U3P	0.442
Aradu.T8H92	0.402	211	47.17	38.208	47.642	Araip.AB5Q5	0.411
Aradu.T8I2T	0.341	495	55.242	43.75	38.911	Araip.B4RCV	0.369
Aradu.T8IMJ	0.346	606	49.918	38.056	37.397	Araip.ZGM3R	0.349
Aradu.T8IUY	0.38	423	41.509	38.208	48.113	Araip.AHX0C	0.375
Aradu.T8J76	0.376	551	53.986	43.478	39.13	Araip.ZSU41	0.37
Aradu.T8L1U	0.366	665	50.901	46.997	32.733	Araip.7LQ31	0.365
Aradu.T8M37	0.37	584	54.701	35.556	32.991	Araip.V9NIV	0.366
Aradu.T8M8U	0.379	349	41.143	40.857	37.714	Araip.YFS36	0.382
Aradu.T8SW7	0.401	784	55.669	35.796	35.287	Araip.1ZW9V	0.399
Aradu.T8TQR	0.403	628	53.418	46.423	32.75	Araip.SN4N7	0.4
Aradu.T8X2H	0.354	460	49.892	40.564	40.564	Araip.GL9AE	0.36
Aradu.T922Y	0.427	493	40.891	42.308	43.522	Araip.ULA1J	0.421
Aradu.T9MDP	0.396	448	52.116	46.325	37.194	Araip.1I30Q	0.396
Aradu.T9MSY	0.279	171	47.093	36.047	34.302	Araip.G2UJA	0.277
Aradu.T9PKV	0.391	296	50.505	43.434	45.118	Araip.891PE	0.379
Aradu.T9RZI	0.34	851	48.005	44.718	31.455	Araip.2RY51	0.333
Aradu.T9UI2	0.376	754	49.669	40.53	44.106	Araip.PAJ8W	0.375
Aradu.T9UN0	0.397	870	49.254	45.695	36.625	Araip.2A6VZ	0.398
Aradu.T9WAY	0.307	159	51.875	45	36.875	Araip.T6HC3	0.313
Aradu.T9X3T	0.362	390	45.78	38.619	35.038	Araip.ST9EM	0.342
Aradu.T9XRG	0.375	118	57.983	47.059	36.975	Araip.C8Q7I	0.357
Aradu.T9YMT	0.399	1279	46.875	40.078	38.984	Araip.V40G1	0.386
Aradu.TA161	0.414	417	50.957	44.737	38.756	Araip.G4AXZ	0.413
Aradu.TB0L3	0.416	293	51.02	51.361	48.98	Araip.3047C	0.389
Aradu.TB51Z	0.351	378	50.132	43.272	38.522	Araip.IAX0I	0.345
Aradu.TB877	0.424	148	48.993	40.268	45.638	Araip.GDQ5V	0.417
Aradu.TBP46	0.387	463	49.138	40.302	45.905	Araip.JH23Y	0.38
Aradu.TBQ82	0.349	1423	45.646	39.045	34.059	Araip.RDT17	0.35
Aradu.TBT3N	0.355	662	51.584	36.501	41.176	Araip.J05HY	0.353
Aradu.TBX7Z	0.395	977	56.851	37.935	39.162	Araip.9K0LI	0.398
Aradu.TC0KD	0.42	380	52.756	43.832	42.782	Araip.Z68LQ	0.427
Aradu.TC1A2	0.408	679	47.794	37.794	47.941	Araip.D4JH9	0.414
Aradu.TC25V	0.396	341	51.17	38.012	37.135	Araip.K2DDF	0.417
Aradu.TC6LS	0.413	179	38.333	37.222	58.333	Araip.5P4LE	0.407

Aradu.TC75Q	0.477	268	54.647	39.777	47.955	Araip.RW9KD	0.483
Aradu.TCQ2L	0.412	390	56.522	38.619	63.171	Araip.0P3RJ	0.42
Aradu.TD3N5	0.309	417	50.957	36.124	34.211	Araip.Q55MD	0.32
Aradu.TD814	0.445	635	54.245	39.308	44.969	Araip.HGD34	0.441
Aradu.TE0XJ	0.373	509	54.118	40.98	34.314	Araip.HG9T7	0.374
Aradu.TE378	0.352	339	55.294	52.059	40.588	Araip.1E0RS	0.342
Aradu.TE4V4	0.399	634	51.654	42.047	37.48	Araip.63QU3	0.392
Aradu.TE7Z6	0.402	349	46.857	38	44.857	Araip.YBU7P	0.394
Aradu.TEF34	0.345	506	48.915	42.406	41.617	Araip.6ZH5D	0.367
Aradu.TES1U	0.486	301	55.96	37.417	68.212	Araip.H56DJ	0.486
Aradu.TET8U	0.402	667	48.204	35.928	38.473	Araip.MFC9D	0.406
Aradu.TEW52	0.46	418	55.609	42.243	63.962	Araip.BG5YS	0.453
Aradu.TF0TM	0.364	443	53.378	38.063	36.937	Araip.PAE7Y	0.354
Aradu.TF2FR	0.443	539	45.741	44.074	58.148	Araip.EK6VJ	0.448
Aradu.TF48T	0.379	696	57.963	47.92	36.872	Araip.4KW8X	0.374
Aradu.TF4C3	0.425	450	52.772	39.468	50.554	Araip.79KSY	0.417
Aradu.TF9ZM	0.336	288	49.481	40.83	42.215	Araip.B188M	0.332
Aradu.TFD03	0.419	168	43.195	39.645	39.645	Araip.C0P67	0.44
Aradu.TFK2R	0.53	619	47.258	47.258	64.194	Araip.H3C8N	0.51
Aradu.TG162	0.391	586	49.574	39.353	36.286	Araip.3V9HH	0.377
Aradu.TH1E7	0.366	914	46.995	36.503	33.661	Araip.EQA8W	0.359
Aradu.TH5KX	0.358	194	45.128	37.436	38.974	Araip.TUQ7N	0.378
Aradu.THV7P	0.385	479	44.167	33.958	44.792	Araip.66P9D	0.386
Aradu.THY46	0.413	643	45.186	42.547	50.311	Araip.5D9GX	0.416
Aradu.TI2TK	0.377	270	54.982	57.196	33.21	Araip.YIG64	0.374
Aradu.TI81R	0.464	190	57.068	30.89	58.115	Araip.K6C0S	0.464
Aradu.TI9C9	0.404	300	48.173	45.183	37.209	Araip.W4E4J	0.404
Aradu.TJ0ZU	0.369	504	49.703	36.436	41.98	Araip.RXK1S	0.365
Aradu.TJ219	0.462	752	56.175	48.074	56.308	Araip.K4YB2	0.471
Aradu.TJ47H	0.45	338	55.162	44.248	52.802	Araip.B0W5P	0.446
Aradu.TJ8NB	0.322	264	48.302	44.151	48.679	Araip.TNG4W	0.318
Aradu.TJC58	0.427	225	49.115	41.15	45.575	Araip.P3X4Z	0.442
Aradu.TJK3R	0.417	432	45.727	40.185	46.189	Araip.R22ES	0.419
Aradu.TJL9X	0.402	867	51.843	38.479	43.088	Araip.K56RN	0.408
Aradu.TJM76	0.386	462	55.076	38.013	50.108	Araip.QVJ1V	0.393
Aradu.TK1NR	0.357	525	49.24	39.734	45.247	Araip.CW8LJ	0.356
Aradu.TK9TC	0.42	542	51.565	45.856	54.88	Araip.YJ2ZI	0.426
Aradu.TKG0E	0.382	359	47.778	46.389	42.5	Araip.6J7QQ	0.392
Aradu.TKG8T	0.362	395	43.939	35.859	36.364	Araip.EA9WI	0.376
Aradu.TL35C	0.403	664	55.94	42.556	50.827	Araip.23NEF	0.395
Aradu.TL6UB	0.438	571	58.741	44.93	55.769	Araip.YJF57	0.432
Aradu.TL8H2	0.491	394	57.215	45.823	65.063	Araip.D2LW3	0.476
Aradu.TLI73	0.407	394	49.873	43.291	48.861	Araip.Q12S9	0.412
Aradu.TM0DS	0.38	608	49.425	39.573	40.23	Araip.NS24Q	0.385

Aradu.TM2EL	0.381	369	52.432	48.378	45.946	Araip.79M9T	0.375
Aradu.TM4JI	0.336	439	52.727	41.136	31.818	Araip.Q2JJX	0.336
Aradu.TM66X	0.479	308	54.369	47.896	64.078	Araip.SN4PX	0.48
Aradu.TN0QL	0.397	468	53.945	45.203	43.923	Araip.UF36S	0.387
Aradu.TN13P	0.445	334	49.254	35.522	55.522	Araip.Y0VZU	0.436
Aradu.TN75P	0.463	367	55.163	41.848	50.815	Araip.GQB2P	0.475
Aradu.TN7XF	0.356	727	44.368	34.615	39.011	Araip.89I2E	0.359
Aradu.TN9DS	0.398	671	50.595	43.304	37.202	Araip.GZ4IV	0.395
Aradu.TP6EC	0.369	764	53.203	47.19	39.739	Araip.Q5EBR	0.369
Aradu.TP72Z	0.356	1101	47.459	35.753	36.842	Araip.03C48	0.342
Aradu.TP7W2	0.333	123	48.387	29.839	39.516	Araip.CVD1W	0.315
Aradu.TPI8X	0.427	686	56.186	39.592	50.073	Araip.BSJ6S	0.414
Aradu.TQ01E	0.32	354	62.254	53.239	37.183	Araip.11VE0	0.336
Aradu.TQ146	0.362	437	56.164	38.356	36.53	Araip.TK5D4	0.356
Aradu.TQ3RZ	0.409	481	47.925	42.531	34.855	Araip.VVF66	0.417
Aradu.TQ51J	0.4	897	47.884	40.312	39.644	Araip.TP6H4	0.384
Aradu.TR2FS	0.333	289	39.31	37.931	42.759	Araip.4NI21	0.339
Aradu.TR8YP	0.358	403	55.446	46.287	35.891	Araip.Y74Z9	0.374
Aradu.TRI7U	0.381	335	47.024	32.44	38.393	Araip.CF7TW	0.376
Aradu.TS004	0.452	275	47.826	36.957	47.464	Araip.77AM3	0.444
Aradu.TSW1P	0.371	735	46.332	47.418	36.005	Araip.354H4	0.373
Aradu.TSZ7Q	0.398	195	41.837	34.694	53.571	Araip.N7CHL	0.406
Aradu.TT65B	0.339	502	48.509	38.966	42.744	Araip.XJ990	0.337
Aradu.TTI87	0.382	763	50.262	36.518	44.503	Araip.07W64	0.384
Aradu.TTW29	0.477	268	54.647	48.327	40.52	Araip.P7WV8	0.473
Aradu.TU51C	0.421	330	61.934	43.202	43.202	Araip.L9MEB	0.428
Aradu.TUT46	0.493	365	46.721	46.175	46.995	Araip.Y7KTN	0.508
Aradu.TV03P	0.439	168	46.154	39.053	49.704	Araip.68E16	0.446
Aradu.TV1GT	0.386	390	50.128	44.246	40.921	Araip.85X1W	0.397
Aradu.TV3WI	0.414	406	50.86	42.752	39.312	Araip.R07X0	0.409
Aradu.TV4LZ	0.393	113	60.526	61.404	38.596	Araip.5Q25R	0.393
Aradu.TV5GQ	0.403	163	48.78	59.146	43.293	Araip.M2BYP	0.405
Aradu.TVK0N	0.387	1274	52.706	40	38.196	Araip.G7BAX	0.392
Aradu.TVQ04	0.39	344	44.348	40.87	41.739	Araip.LN1Q8	0.374
Aradu.TVX63	0.379	491	47.561	43.902	40.447	Araip.GD8VH	0.371
Aradu.TVX9T	0.335	918	48.531	38.738	36.561	Araip.J98GW	0.334
Aradu.TW1EN	0.466	276	46.931	46.931	45.487	Araip.5J271	0.474
Aradu.TW3FF	0.396	352	44.193	38.81	36.827	Araip.5R4LP	0.393
Aradu.TW9EH	0.344	680	47.137	34.508	41.263	Araip.B0FC3	0.34
Aradu.TWK59	0.342	569	51.404	34.912	39.298	Araip.YXG1J	0.344
Aradu.TX9D6	0.436	325	55.521	38.957	52.147	Araip.R02Z7	0.433
Aradu.TXN9A	0.479	471	50	48.093	59.958	Araip.7PD3K	0.475
Aradu.TY4XK	0.384	386	54.522	32.558	41.86	Araip.6Y1SP	0.384
Aradu.TY7A4	0.474	446	49.888	47.204	55.481	Araip.01ZK6	0.466

Aradu.TY9X1	0.369	397	47.487	41.96	44.472	Araip.HXZ5T	0.369
Aradu.TYF13	0.356	1066	50.328	37.301	35.708	Araip.B832X	0.357
Aradu.TYG98	0.439	805	58.065	45.285	55.459	Araip.45GIV	0.437
Aradu.TYQ4J	0.4	625	49.84	37.22	41.054	Araip.J3YS9	0.403
Aradu.TZ1M5	0.388	157	43.038	40.506	34.177	Araip.YA4KW	0.379
Aradu.TZ5MC	0.336	156	50.318	35.669	37.58	Araip.ITT7M	0.329
Aradu.TZ9RJ	0.39	225	53.097	32.301	40.708	Araip.L2SQL	0.381
Aradu.TZG12	0.432	473	57.595	44.937	52.954	Araip.Y99XU	0.438
Aradu.TZS3T	0.385	429	45.581	37.907	43.256	Araip.SVT52	0.381
Aradu.U012P	0.356	322	58.514	42.415	40.248	Araip.GHK6Z	0.352
Aradu.U033V	0.455	330	56.193	39.577	63.746	Araip.6D6XW	0.455
Aradu.U0DT9	0.386	436	54.691	43.478	44.394	Araip.I6NSD	0.365
Aradu.U0KK7	0.4	192	50.777	35.233	43.005	Araip.E9ALR	0.415
Aradu.U0T6A	0.368	528	53.308	37.429	41.21	Araip.9E8AI	0.378
Aradu.U0V69	0.398	1056	53.359	39.451	39.167	Araip.P67TL	0.406
Aradu.U14K2	0.368	618	52.989	37.48	33.764	Araip.HU9EV	0.363
Aradu.U16MC	0.346	192	47.668	40.415	44.56	Araip.18PWY	0.359
Aradu.U18XV	0.376	1622	49.476	37.708	36.352	Araip.VXU4B	0.372
Aradu.U1A8I	0.407	673	52.077	33.086	43.769	Araip.GP1XI	0.413
Aradu.U1CK3	0.356	438	53.075	40.091	41.686	Araip.C26DA	0.363
Aradu.U1FJC	0.418	891	51.121	41.256	42.152	Araip.FW0V7	0.41
Aradu.U1I3J	0.431	326	40.673	36.391	36.391	Araip.IUW8W	0.401
Aradu.U1JXN	0.471	192	47.668	46.114	36.788	Araip.HGN3R	0.473
Aradu.U1RBP	0.323	333	52.096	47.006	39.82	Araip.313P6	0.33
Aradu.U1S9C	0.382	648	50.539	43.606	42.835	Araip.KKX96	0.39
Aradu.U1T2G	0.362	236	48.945	43.46	38.397	Araip.9JH6T	0.339
Aradu.U20ZR	0.319	368	47.154	38.753	36.043	Araip.6ME1P	0.297
Aradu.U23UQ	0.35	638	43.505	36.15	36.463	Araip.JT07S	0.349
Aradu.U260V	0.358	368	47.425	40.65	28.455	Araip.VN84X	0.352
Aradu.U2NUZ	0.347	560	52.763	41.889	36.364	Araip.KS63Q	0.341
Aradu.U2UEV	0.356	1039	51.154	34.135	38.269	Araip.65HZ4	0.351
Aradu.U35FJ	0.39	334	52.239	34.925	39.104	Araip.BEB3B	0.388
Aradu.U3ACN	0.384	1802	52.413	47.033	33.833	Araip.0XQ95	0.382
Aradu.U3B0I	0.37	193	52.062	45.876	51.031	Araip.S2FNK	0.372
Aradu.U3EK7	0.36	178	48.603	36.872	33.52	Araip.IV1IH	0.355
Aradu.U3GYE	0.392	457	50.218	40.393	38.646	Araip.UB259	0.387
Aradu.U3RTW	0.359	344	51.304	38.551	50.145	Araip.ZEN1Y	0.356
Aradu.U3SNU	0.372	622	53.451	39.165	51.364	Araip.B7KGV	0.378
Aradu.U3UVH	0.342	610	49.591	40.426	37.316	Araip.Z65SS	0.336
Aradu.U3Z1S	0.385	626	45.774	34.45	45.295	Araip.U82NT	0.393
Aradu.U4RIV	0.401	343	52.616	34.884	36.337	Araip.306R5	0.395
Aradu.U4S49	0.392	233	50.427	37.179	37.607	Araip.MP6Y6	0.383
Aradu.U4UF8	0.349	182	45.355	39.891	43.169	Araip.NAY3U	0.355
Aradu.U4XDJ	0.366	637	51.254	42.163	36.834	Araip.LA6RB	0.362

Aradu.U4Z04	0.394	618	51.373	37.964	36.995	Araip.AJL31	0.387
Aradu.U51AH	0.48	227	58.772	44.737	51.316	Araip.AT5YU	0.471
Aradu.U53UJ	0.361	323	50.926	37.037	38.272	Araip.PR57R	0.373
Aradu.U55D3	0.414	475	59.664	46.218	53.151	Araip.M6EG0	0.425
Aradu.U564S	0.36	355	48.315	38.202	48.596	Araip.00G1J	0.351
Aradu.U57WW	0.363	394	44.557	40	35.949	Araip.40X13	0.368
Aradu.U5A8Y	0.365	496	55.734	41.65	32.596	Araip.09CWU	0.371
Aradu.U5BRX	0.461	753	54.244	51.326	52.785	Araip.Y1ZA6	0.469
Aradu.U5BST	0.37	317	41.824	44.34	39.308	Araip.DSZ1Z	0.372
Aradu.U5CVT	0.397	276	54.513	47.653	46.931	Araip.UUG0Y	0.396
Aradu.U5DG8	0.379	368	53.388	40.921	33.875	Araip.W8KP6	0.379
Aradu.U5M8T	0.366	470	54.989	47.983	31.423	Araip.61LLE	0.366
Aradu.U5NU2	0.356	660	50.832	39.486	44.629	Araip.JIL9K	0.349
Aradu.U5UGY	0.363	361	50	33.425	40.055	Araip.5DJ69	0.364
Aradu.U5Z1W	0.384	766	50.456	38.983	37.027	Araip.W00GH	0.379
Aradu.U5ZHH	0.378	269	43.333	46.667	42.593	Araip.A69R7	0.382
Aradu.U61F3	0.375	222	60.987	48.879	41.256	Araip.X69LT	0.37
Aradu.U6514	0.334	326	42.202	38.226	44.343	Araip.J0GUS	0.333
Aradu.U66TG	0.516	291	49.658	55.822	65.068	Araip.BG3YM	0.518
Aradu.U68KB	0.358	226	51.101	44.053	38.767	Araip.9C0A1	0.36
Aradu.U6F8I	0.411	624	46.08	36.16	48.48	Araip.6SM52	0.415
Aradu.U6HMB	0.428	352	54.391	47.875	35.127	Araip.BUM43	0.434
Aradu.U6IZ8	0.36	327	52.439	43.293	36.28	Araip.DJ2ZW	0.374
Aradu.U6RXK	0.401	187	52.128	67.553	38.298	Araip.P20M1	0.383
Aradu.U6RYQ	0.345	1029	47.864	36.699	37.573	Araip.JM4UY	0.333
Aradu.U6V8H	0.427	250	41.833	37.052	37.849	Araip.9X806	0.427
Aradu.U6YR6	0.364	279	46.786	33.929	47.5	Araip.X0SC5	0.366
Aradu.U70TW	0.343	1116	51.298	39.033	35.273	Araip.7UJ60	0.346
Aradu.U75R0	0.423	297	51.342	41.611	49.329	Araip.A6KDQ	0.405
Aradu.U77H9	0.384	693	50.865	37.032	40.058	Araip.P048V	0.38
Aradu.U78VM	0.418	213	47.664	35.514	46.262	Araip.NRD2A	0.418
Aradu.U795Q	0.39	847	55.896	31.368	41.863	Araip.W9BA5	0.385
Aradu.U79FN	0.418	287	52.778	37.5	38.194	Araip.8TI0S	0.422
Aradu.U7A5U	0.417	407	54.412	41.176	43.627	Araip.KF1D0	0.427
Aradu.U7EBV	0.376	541	48.893	33.026	38.192	Araip.Q4MYK	0.367
Aradu.U7FRB	0.37	1081	50	42.421	43.438	Araip.CG564	0.376
Aradu.U7HHN	0.407	403	50.743	47.03	40.842	Araip.Q0CU4	0.408
Aradu.U7IHD	0.426	252	50.988	33.992	43.874	Araip.9E124	0.393
Aradu.U7LA9	0.412	123	54.032	44.355	49.194	Araip.GM32M	0.398
Aradu.U7UH3	0.422	204	60.488	40	41.463	Araip.3SG4B	0.417
Aradu.U7UZ6	0.346	353	46.045	35.593	35.028	Araip.18QQE	0.338
Aradu.U808N	0.394	1264	57.233	43.478	38.182	Araip.U1QS2	0.389
Aradu.U80Z7	0.356	290	56.014	38.832	37.457	Araip.JTN8C	0.371
Aradu.U838H	0.325	354	49.014	37.183	38.873	Araip.DQI4G	0.32

Aradu.U8582	0.398	545	52.015	40.11	38.278	Araip.42EJ9	0.404
Aradu.U8C21	0.499	352	57.79	43.626	63.456	Araip.TWG69	0.497
Aradu.U8CR2	0.394	683	47.661	41.52	50.877	Araip.E2AI3	0.389
Aradu.U8FLG	0.326	743	47.984	45.833	25.941	Araip.09VK0	0.325
Aradu.U8M4U	0.328	725	53.444	33.333	45.592	Araip.6YK9P	0.342
Aradu.U8NZD	0.403	324	45.846	47.385	32.308	Araip.U5BHC	0.406
Aradu.U8PRD	0.364	568	41.828	53.779	33.919	Araip.B6DZJ	0.371
Aradu.U8TGW	0.42	637	44.984	39.028	40.282	Araip.1ZJ86	0.429
Aradu.U8VVP	0.563	127	48.438	36.719	78.906	Araip.56PXP	0.522
Aradu.U8XKU	0.377	491	46.951	42.683	37.805	Araip.I2REN	0.382
Aradu.U8ZNV	0.413	621	50.482	36.977	39.55	Araip.94SGJ	0.417
Aradu.U96FI	0.477	137	42.029	46.377	49.275	Araip.C351P	0.504
Aradu.U974Q	0.386	633	45.899	37.382	44.006	Araip.9W6SR	0.378
Aradu.U987Q	0.441	410	45.985	34.55	44.282	Araip.I4GQV	0.43
Aradu.U999X	0.36	537	48.885	36.989	37.546	Araip.CJ1ZA	0.364
Aradu.U99RK	0.368	234	50.213	42.979	37.021	Araip.2E6XX	0.361
Aradu.U9A7G	0.37	419	54.286	44.524	29.762	Araip.7RD3Q	0.368
Aradu.U9CZ6	0.413	385	44.301	38.86	43.264	Araip.591ZY	0.411
Aradu.U9G17	0.373	394	59.241	38.734	42.785	Araip.AFI6W	0.393
Aradu.U9IPR	0.401	367	51.087	42.391	51.087	Araip.2404G	0.416
Aradu.U9SCT	0.41	298	49.833	39.13	39.13	Araip.0YS5Y	0.406
Aradu.U9WH5	0.428	315	56.962	41.139	46.203	Araip.AP4U8	0.414
Aradu.UA2HU	0.421	527	48.674	41.288	54.735	Araip.WR1Z1	0.427
Aradu.UA393	0.402	1275	52.665	26.724	41.85	Araip.6L8N8	0.405
Aradu.UA4JV	0.391	549	52.182	44.364	40.909	Araip.44RH2	0.394
Aradu.UA6JC	0.394	132	53.383	40.602	60.15	Araip.GU4P9	0.409
Aradu.UA8T0	0.376	446	51.007	38.031	35.794	Araip.RB200	0.364
Aradu.UA9D8	0.393	594	53.782	44.37	41.513	Araip.WVH6X	0.395
Aradu.UAH84	0.333	424	43.765	38.118	33.412	Araip.CLK0K	0.335
Aradu.UAJ8F	0.355	426	51.054	34.66	35.129	Araip.BEJ3Y	0.338
Aradu.UAL0U	0.369	516	43.133	32.302	48.356	Araip.UT9PH	0.365
Aradu.UAQ2R	0.364	369	46.486	54.324	32.973	Araip.X6HL2	0.379
Aradu.UB13R	0.382	866	42.215	42.676	38.293	Araip.6A16L	0.381
Aradu.UB2GG	0.359	231	44.397	43.534	35.776	Araip.BZ0SY	0.362
Aradu.UB339	0.447	455	54.825	36.404	50.658	Araip.12TI6	0.44
Aradu.UC09A	0.364	787	51.015	38.452	40.228	Araip.8508H	0.36
Aradu.UC2R1	0.341	631	50.633	44.778	34.652	Araip.2V2W4	0.345
Aradu.UC6E5	0.365	814	48.344	40.368	37.423	Araip.ZTR01	0.361
Aradu.UD1IS	0.371	441	45.701	40.271	38.462	Araip.BTG7R	0.378
Aradu.UD253	0.36	357	43.296	43.575	40.223	Araip.LRH88	0.393
Aradu.UD2Q8	0.394	315	54.114	39.873	39.241	Araip.ZV5ML	0.394
Aradu.UD5FH	0.443	268	47.955	39.405	49.442	Araip.P4GTJ	0.435
Aradu.UD5NS	0.503	317	46.226	48.428	59.434	Araip.MM1BS	0.506
Aradu.UD5RI	0.393	801	58.229	44.638	41.895	Araip.HF1C0	0.393

Aradu.UDC1Q	0.39	444	43.82	37.978	41.798	Araip.SS4HT	0.39
Aradu.UDE5G	0.398	402	51.613	41.439	36.476	Araip.6WV2C	0.373
Aradu.UDE9J	0.422	532	43.34	42.964	42.777	Araip.H48JL	0.414
Aradu.UDU6Q	0.441	568	52.724	39.895	52.197	Araip.5Z2JS	0.437
Aradu.UE0PI	0.333	431	46.991	30.324	38.657	Araip.K91M4	0.338
Aradu.UE6Z2	0.386	1143	51.923	46.241	38.636	Araip.E8MZN	0.389
Aradu.UEF4U	0.382	893	44.519	38.814	40.604	Araip.PS4N8	0.383
Aradu.UEG36	0.405	342	53.061	40.816	41.108	Araip.2Z354	0.405
Aradu.UEG62	0.409	195	50.51	37.245	41.327	Araip.JAJ4W	0.409
Aradu.UEM5G	0.441	324	53.846	43.077	56.615	Araip.HCG81	0.438
Aradu.UF1FY	0.357	277	47.482	36.691	43.885	Araip.S4CS4	0.35
Aradu.UF4PG	0.376	533	49.064	41.199	36.891	Araip.V4GNP	0.376
Aradu.UF8GX	0.375	520	49.712	37.62	45.106	Araip.3L11M	0.385
Aradu.UFC3R	0.353	204	47.317	41.951	47.805	Araip.87H0B	0.34
Aradu.UFI9S	0.505	101	49.02	43.137	50	Araip.53WJE	0.505
Aradu.UG0RV	0.346	244	53.061	38.776	48.163	Araip.ZCR91	0.35
Aradu.UGC9M	0.441	281	51.064	48.227	58.156	Araip.XR0FV	0.441
Aradu.UH20T	0.392	129	35.385	40.769	35.385	Araip.GCD94	0.366
Aradu.UH61L	0.373	577	43.08	34.775	34.948	Araip.1T9WY	0.371
Aradu.UHQ4E	0.438	178	39.106	35.196	64.804	Araip.6QE8H	0.408
Aradu.UHQ4T	0.403	311	51.282	43.269	36.859	Araip.S75SQ	0.405
Aradu.UI0N2	0.356	893	43.96	37.136	36.13	Araip.T9ZFK	0.36
Aradu.UI0XD	0.397	597	51.839	42.642	49.164	Araip.3N4EX	0.4
Aradu.UI9VM	0.394	444	49.438	37.978	40.449	Araip.7QY88	0.384
Aradu.UIA0J	0.494	170	58.48	40.351	66.667	Araip.HR7AZ	0.485
Aradu.UIA4U	0.402	347	54.598	33.046	42.241	Araip.0B6PB	0.404
Aradu.UIC09	0.367	977	52.761	37.73	42.74	Araip.I90M3	0.369
Aradu.UIF60	0.357	761	53.806	45.276	40.551	Araip.UH3IE	0.355
Aradu.UJ1ZX	0.363	327	50.61	43.293	38.415	Araip.G30W8	0.382
Aradu.UJ5WJ	0.392	432	46.882	43.649	41.339	Araip.R83H8	0.395
Aradu.UJ8BX	0.463	387	52.062	44.33	52.835	Araip.EK901	0.461
Aradu.UJ9WA	0.397	247	50	32.661	44.758	Araip.PB6S5	0.405
Aradu.UJD9G	0.413	469	50.213	36.809	44.255	Araip.417P5	0.416
Aradu.UJJ9I	0.38	147	47.973	40.541	43.919	Araip.YJ489	0.366
Aradu.UK481	0.416	287	50.347	33.681	59.028	Araip.LWR36	0.412
Aradu.UK4BE	0.348	346	47.262	38.905	40.922	Araip.7L4UM	0.344
Aradu.UK58V	0.423	200	53.731	57.214	45.274	Araip.TWB47	0.429
Aradu.UK6QI	0.353	449	51.333	31.333	46.222	Araip.K0ECG	0.353
Aradu.UKP00	0.328	310	46.945	40.193	40.836	Araip.JE0JZ	0.319
Aradu.UL23E	0.38	611	50.49	41.503	33.333	Araip.WJS9Q	0.367
Aradu.ULI8B	0.504	142	49.65	50.35	48.951	Araip.14380	0.504
Aradu.ULX7G	0.332	248	52.209	36.948	36.546	Araip.A98H2	0.344
Aradu.UM1Y9	0.39	160	49.689	36.025	57.143	Araip.U10LF	0.402
Aradu.UM7TW	0.396	721	54.017	40.028	47.784	Araip.UIW35	0.395

Aradu.UM9AF	0.389	304	44.918	38.361	42.295	Araip.7BF1X	0.397
Aradu.UM9US	0.384	407	55.147	44.853	38.235	Araip.Z77CR	0.373
Aradu.UMN0V	0.375	702	44.666	37.838	33.144	Araip.M4K9W	0.36
Aradu.UMP23	0.427	1002	49.252	38.784	51.645	Araip.HL278	0.425
Aradu.UNB9U	0.389	408	49.389	37.897	44.01	Araip.JU13E	0.389
Aradu.UNY97	0.432	353	50.282	36.441	52.825	Araip.1FA5F	0.427
Aradu.UP0S8	0.314	519	48.654	34.615	36.923	Araip.3S762	0.314
Aradu.UPE5A	0.35	567	50.176	45.423	38.38	Araip.X7QQD	0.352
Aradu.UPU3E	0.356	718	47.844	34.214	37.969	Araip.8TY96	0.363
Aradu.UQ2CM	0.352	551	41.667	36.775	37.138	Araip.H7FNG	0.355
Aradu.UQA28	0.349	261	55.725	35.496	34.351	Araip.3H7G0	0.369
Aradu.UQP4Y	0.418	437	55.251	42.694	53.196	Araip.NXK65	0.42
Aradu.UQQ1M	0.479	175	51.136	45.455	56.25	Araip.CCT6I	0.491
Aradu.UQR72	0.44	123	60.484	33.871	62.903	Araip.TD1JT	0.448
Aradu.UR0SD	0.424	617	57.605	43.689	60.194	Araip.U9YQ5	0.421
Aradu.UR64R	0.43	149	49.333	42	52	Araip.IF6H8	0.415
Aradu.UR751	0.325	432	49.654	37.413	37.875	Araip.I6EYC	0.305
Aradu.URD4R	0.416	680	52.717	42.438	48.752	Araip.SV2QM	0.412
Aradu.USH95	0.521	369	49.459	48.649	61.081	Araip.78PTT	0.52
Aradu.UT07K	0.415	406	51.106	39.558	67.322	Araip.E028Y	0.418
Aradu.UT1SE	0.398	547	47.263	36.679	37.591	Araip.RE8JQ	0.394
Aradu.UT3S4	0.355	453	54.846	40.308	41.85	Araip.T87S4	0.387
Aradu.UTB08	0.41	313	47.452	52.866	32.803	Araip.899GP	0.418
Aradu.UTT3P	0.387	457	54.585	47.598	35.59	Araip.FU2FP	0.377
Aradu.UTZ1D	0.381	223	48.214	42.857	45.536	Araip.CF2W6	0.382
Aradu.UU4C2	0.361	1160	49.526	37.812	36.262	Araip.QG6DX	0.36
Aradu.UU910	0.377	643	54.193	42.391	37.888	Araip.AMI35	0.381
Aradu.UUC9E	0.398	197	55.051	48.99	53.535	Araip.N9DIE	0.402
Aradu.UV1C8	0.366	314	45.714	40	38.413	Araip.N517Q	0.36
Aradu.UV8JW	0.397	397	50.754	38.945	39.196	Araip.IIT7G	0.392
Aradu.UVG4U	0.349	223	49.107	34.821	44.643	Araip.5FY0D	0.358
Aradu.UVJ1U	0.4	1168	53.208	39.863	41.061	Araip.C8DTK	0.403
Aradu.UWB5V	0.342	430	43.387	41.067	46.172	Araip.IFL1M	0.354
Aradu.UWX45	0.393	535	55.41	35.261	40.672	Araip.W7JXY	0.393
Aradu.UX579	0.497	186	52.406	47.594	62.032	Araip.AP06R	0.51
Aradu.UX5R5	0.352	589	48.475	41.695	36.949	Araip.KD3S1	0.35
Aradu.UX9CD	0.366	450	44.346	40.355	33.703	Araip.LN55K	0.386
Aradu.UXI37	0.385	437	51.142	38.128	41.781	Araip.6RZ29	0.402
Aradu.UXS7E	0.43	453	57.269	34.361	51.542	Araip.2KR54	0.43
Aradu.UY019	0.38	1175	50.68	42.772	36.735	Araip.7NS74	0.384
Aradu.UY6CA	0.408	418	52.745	41.289	51.79	Araip.PBR2C	0.402
Aradu.UYZ93	0.344	982	50.966	37.03	39.674	Araip.R77E0	0.336
Aradu.UZ3UX	0.495	320	49.844	57.009	59.813	Araip.VX3FG	0.495
Aradu.UZ754	0.403	204	50.732	42.439	43.415	Araip.R86UC	0.408

Aradu.UZJ7G	0.356	275	41.667	40.217	34.42	Araip.IPQ80	0.361
Aradu.UZS9C	0.356	883	49.774	37.217	36.538	Araip.TZC8T	0.347
Aradu.UZX8A	0.429	179	46.667	43.889	47.222	Araip.NPF88	0.418
Aradu.V019K	0.468	328	48.024	42.249	64.134	Araip.51SDF	0.443
Aradu.V05DH	0.437	1128	51.727	39.858	58.282	Araip.KZ67T	0.436
Aradu.V060T	0.352	700	50.785	40.799	35.806	Araip.Y13UG	0.354
Aradu.V07RE	0.374	452	49.007	42.826	38.19	Araip.C4MN8	0.368
Aradu.V0C1K	0.399	323	52.469	38.889	39.506	Araip.J5QMQ	0.397
Aradu.V0CGL	0.398	361	46.961	46.685	34.53	Araip.983RG	0.378
Aradu.V0EF8	0.382	371	49.731	41.398	39.785	Araip.FHB4T	0.38
Aradu.V0JK3	0.299	162	43.558	31.902	29.448	Araip.8KM9E	0.288
Aradu.V0UIC	0.424	567	55.986	41.901	52.465	Araip.3GT2G	0.421
Aradu.V0VX5	0.338	490	52.953	40.937	35.642	Araip.X4EHZ	0.342
Aradu.V1H1X	0.369	785	56.87	37.659	45.42	Araip.L03GB	0.367
Aradu.V1INC	0.34	211	53.774	33.019	45.755	Araip.T13Y8	0.412
Aradu.V1JTR	0.384	416	55.635	40.767	34.053	Araip.E6C5N	0.389
Aradu.V1L1H	0.386	333	53.593	42.216	50	Araip.RB3WS	0.371
Aradu.V1MDS	0.364	570	46.935	38.704	42.732	Araip.S2MVY	0.364
Aradu.V1NXI	0.413	253	53.15	40.551	41.339	Araip.3B3VN	0.417
Aradu.V1PLQ	0.439	163	54.268	40.854	37.805	Araip.917K4	0.439
Aradu.V1TEZ	0.352	643	44.876	38.043	37.888	Araip.9K7D6	0.356
Aradu.V1TZX	0.387	248	53.414	40.161	32.932	Araip.TH0I1	0.387
Aradu.V220X	0.26	383	41.667	35.938	36.458	Araip.0HK7I	0.262
Aradu.V2AUT	0.328	351	48.011	44.318	34.943	Araip.C26XV	0.326
Aradu.V2BW0	0.417	450	44.568	31.486	47.672	Araip.6TJ7D	0.42
Aradu.V2IK2	0.352	341	51.462	36.842	34.211	Araip.X33WA	0.345
Aradu.V2ISN	0.435	348	53.582	34.957	49.57	Araip.HNB3K	0.417
Aradu.V2KKS	0.383	415	51.923	46.154	37.74	Araip.W3TWU	0.388
Aradu.V2RP9	0.389	477	38.703	31.799	38.494	Araip.KFI7C	0.376
Aradu.V2YDB	0.401	357	56.145	40.782	58.659	Araip.ZTF9F	0.401
Aradu.V2YPR	0.431	224	64.889	41.778	67.556	Araip.YPA63	0.435
Aradu.V2Z0U	0.436	360	55.402	42.936	53.74	Araip.7JC4X	0.43
Aradu.V32T3	0.407	446	57.494	44.072	62.192	Araip.D5YYK	0.405
Aradu.V33RZ	0.451	577	55.19	42.388	50.519	Araip.2W24M	0.454
Aradu.V34QS	0.315	692	51.371	35.498	35.209	Araip.PA1WM	0.312
Aradu.V37F6	0.306	168	44.379	35.503	43.195	Araip.A9GHS	0.318
Aradu.V3ATA	0.273	159	52.5	41.875	35.625	Araip.500NU	0.278
Aradu.V3AZX	0.342	550	45.735	35.572	39.02	Araip.QI64Y	0.335
Aradu.V3C05	0.402	255	50	39.844	49.609	Araip.ZG914	0.402
Aradu.V3HYN	0.344	900	48.502	38.846	37.625	Araip.NC47B	0.349
Aradu.V3IQ0	0.405	607	49.507	39.967	49.342	Araip.Y9Y5W	0.409
Aradu.V3JVM	0.359	1113	49.192	41.023	39.408	Araip.WLL04	0.362
Aradu.V3MUV	0.387	249	51.2	35.2	46	Araip.PJ6F5	0.387
Aradu.V4203	0.369	377	46.825	44.18	34.392	Araip.Z0L35	0.382

Aradu.V43LA	0.392	355	51.404	38.764	49.157	Araip.NB0BG	0.381
Aradu.V45Z7	0.388	345	50	37.572	35.26	Araip.PP9AX	0.392
Aradu.V49C9	0.395	357	52.514	40.503	46.369	Araip.66MK2	0.387
Aradu.V4E4H	0.392	591	54.392	37.331	39.189	Araip.U364I	0.393
Aradu.V4IDM	0.37	827	47.826	37.923	41.063	Araip.NVJ9Q	0.381
Aradu.V4L4B	0.354	584	51.282	37.094	42.564	Araip.S3E5L	0.344
Aradu.V4LAJ	0.474	317	57.233	40.252	60.063	Araip.CS3DH	0.474
Aradu.V4R6L	0.368	974	50.769	38.667	36.308	Araip.36D69	0.377
Aradu.V4S8W	0.378	715	48.045	38.827	35.196	Araip.S85MS	0.375
Aradu.V4U2I	0.394	901	47.561	37.916	37.14	Araip.IT046	0.395
Aradu.V4VHC	0.366	978	54.545	41.573	39.122	Araip.I10GN	0.372
Aradu.V56CC	0.336	308	39.482	35.922	43.689	Araip.UKG53	0.339
Aradu.V5FRM	0.391	464	55.054	40.43	43.441	Araip.X4RTI	0.405
Aradu.V5GXH	0.343	1349	46.296	34.963	39.481	Araip.F4BSU	0.347
Aradu.V5HPY	0.379	1109	51.622	39.369	43.063	Araip.P6VI6	0.384
Aradu.V5JUH	0.354	523	53.626	38.74	34.16	Araip.UDC6V	0.359
Aradu.V5LSS	0.4	742	48.048	43.472	43.069	Araip.TY5FK	0.399
Aradu.V5NSB	0.414	284	53.333	41.404	35.088	Araip.N7X0P	0.409
Aradu.V5SB4	0.399	287	52.083	38.194	46.528	Araip.UJ9UQ	0.392
Aradu.V5V1W	0.487	325	58.589	38.037	58.282	Araip.2EA2I	0.474
Aradu.V5XVE	0.366	624	49.76	41.92	36.48	Araip.D0E4F	0.361
Aradu.V5ZS1	0.424	419	52.619	35.714	41.19	Araip.207MI	0.419
Aradu.V638G	0.333	457	48.69	34.716	40.83	Araip.Z7THM	0.342
Aradu.V66GG	0.362	213	56.542	44.393	30.841	Araip.JJ5F2	0.364
Aradu.V66GY	0.412	526	51.992	40.417	51.613	Araip.K8YAU	0.427
Aradu.V68II	0.377	553	55.054	42.96	36.462	Araip.1D5WV	0.365
Aradu.V6900	0.46	439	48.409	45.455	45.909	Araip.NYR45	0.465
Aradu.V69Z9	0.323	424	43.294	37.647	30.353	Araip.NEN8H	0.317
Aradu.V6MI1	0.346	459	50	40.652	38.043	Araip.Y26KW	0.373
Aradu.V6QFH	0.369	237	57.563	34.034	37.815	Araip.03SRG	0.373
Aradu.V6UC8	0.496	121	54.918	44.262	40.984	Araip.3X84U	0.47
Aradu.V6UVG	0.383	323	48.765	40.432	40.123	Araip.T0QWF	0.377
Aradu.V6ZCM	0.422	304	52.459	39.344	41.967	Araip.D7GGC	0.405
Aradu.V6ZMF	0.547	405	55.911	46.305	64.532	Araip.FH6AP	0.544
Aradu.V71C6	0.374	385	50.518	34.715	42.228	Araip.D5TXG	0.379
Aradu.V72BH	0.516	224	50.222	32.889	52.889	Araip.0BL8B	0.525
Aradu.V73WK	0.362	466	47.752	38.116	37.901	Araip.HV7J0	0.376
Aradu.V7EH0	0.456	141	57.042	42.958	57.746	Araip.ZKD8M	0.449
Aradu.V7F48	0.354	316	47.95	42.271	35.962	Araip.80WHH	0.354
Aradu.V7W75	0.396	598	51.586	40.568	39.399	Araip.A5QSA	0.41
Aradu.V7ZTF	0.372	570	49.562	31.699	42.732	Araip.LM2JS	0.392
Aradu.V824B	0.352	1078	48.563	36.979	41.983	Araip.F2IB2	0.344
Aradu.V83DR	0.412	163	47.561	34.146	54.268	Araip.KZF5J	0.405
Aradu.V8FCP	0.421	408	47.677	46.455	41.809	Araip.XV3D7	0.437

Aradu.V8GHA	0.422	286	46.69	42.857	50.523	Araip.H7YH7	0.428
Aradu.V8HSY	0.545	256	50.584	52.918	68.482	Araip.LP8AE	0.551
Aradu.V8J7V	0.445	470	54.777	39.915	56.263	Araip.1H31M	0.445
Aradu.V8MCV	0.39	444	50.562	39.326	35.955	Araip.5N6GI	0.381
Aradu.V8MJ9	0.327	172	61.85	48.555	61.272	Araip.1U5CY	0.327
Aradu.V8XMS	0.415	255	42.969	53.906	42.578	Araip.T7KEI	0.407
Aradu.V92BV	0.374	393	47.208	44.162	40.355	Araip.F6G6S	0.386
Aradu.V94FJ	0.384	541	50.185	35.978	36.162	Araip.PI5NS	0.38
Aradu.V9CJA	0.444	442	48.081	36.343	47.63	Araip.1Y485	0.439
Aradu.V9D23	0.404	378	49.604	39.05	41.161	Araip.94UKG	0.4
Aradu.V9D7S	0.51	365	51.093	48.634	71.311	Araip.E4G9U	0.507
Aradu.V9EPJ	0.349	420	50.831	35.867	37.53	Araip.2HK29	0.344
Aradu.V9MVJ	0.392	895	48.326	40.737	38.393	Araip.WD50G	0.397
Aradu.V9PTU	0.399	1015	55.315	40.354	39.173	Araip.3I7XZ	0.391
Aradu.V9U81	0.394	798	51.439	40.676	46.183	Araip.18474	0.386
Aradu.V9UDT	0.394	290	54.983	34.364	45.361	Araip.V71XV	0.382
Aradu.V9VCU	0.368	293	52.721	47.279	46.599	Araip.WN95W	0.367
Aradu.V9VEN	0.408	1189	46.891	37.143	41.765	Araip.N4RJ5	0.407
Aradu.VA1N9	0.356	614	44.553	63.415	34.309	Araip.5DF3C	0.356
Aradu.VA4EQ	0.348	355	44.944	32.303	38.764	Araip.LM4PJ	0.365
Aradu.VA5ME	0.434	401	57.96	49.005	50.746	Araip.713R1	0.433
Aradu.VA62W	0.386	493	46.964	35.628	34.008	Araip.Y67U3	0.39
Aradu.VA9CU	0.398	404	48.148	35.802	44.444	Araip.N65ZU	0.411
Aradu.VAN9Z	0.424	379	57.105	44.211	39.474	Araip.GJ7LV	0.41
Aradu.VB0PQ	0.374	371	51.882	40.86	34.14	Araip.MJM6V	0.367
Aradu.VB3DF	0.342	227	52.193	40.351	29.386	Araip.RBA5R	0.338
Aradu.VB76D	0.368	595	47.819	42.785	44.631	Araip.Y2F2L	0.379
Aradu.VB99V	0.414	146	55.102	38.776	42.857	Araip.7E55M	0.421
Aradu.VBT9Y	0.383	916	51.254	41.112	40.349	Araip.HMS5U	0.375
Aradu.VBY1Y	0.376	222	51.121	38.117	39.013	Araip.9EA3M	0.376
Aradu.VC25Y	0.389	411	49.029	44.175	42.961	Araip.D61AA	0.401
Aradu.VC6I6	0.372	479	47.708	42.708	46.667	Araip.28BYA	0.384
Aradu.VCF9B	0.316	381	47.382	36.911	30.105	Araip.JEI3K	0.319
Aradu.VD3WH	0.353	231	53.448	39.655	36.207	Araip.LX0AL	0.35
Aradu.VDD5S	0.45	630	53.09	38.352	52.456	Araip.D7KCG	0.445
Aradu.VDW04	0.369	200	47.264	37.811	46.766	Araip.54YKW	0.361
Aradu.VDX8A	0.341	319	50.938	36.25	34.375	Araip.08VK1	0.344
Aradu.VE0KU	0.368	526	48.008	38.33	45.541	Araip.W4DPP	0.357
Aradu.VE1AV	0.319	321	44.41	37.888	38.509	Araip.KA7I0	0.324
Aradu.VE1T0	0.394	321	52.795	38.509	47.205	Araip.3PJ33	0.4
Aradu.VE705	0.38	292	42.321	39.932	34.812	Araip.WHJ14	0.378
Aradu.VF4U3	0.372	328	48.328	41.641	41.337	Araip.433VX	0.382
Aradu.VF877	0.371	258	54.44	45.946	37.452	Araip.HD8AG	0.379
Aradu.VFE8Z	0.365	1230	49.147	43.136	35.5	Araip.9S2X2	0.362

Aradu.VFG89	0.347	160	47.205	47.205	36.646	Araip.CP17Q	0.347
Aradu.VFN3G	0.406	500	52.495	38.723	48.503	Araip.EER3Y	0.415
Aradu.VG3CJ	0.371	518	43.16	49.326	39.306	Araip.PZ3FG	0.367
Aradu.VG4AF	0.399	266	49.813	43.82	47.94	Araip.M00I0	0.381
Aradu.VHJ0W	0.349	345	48.844	40.462	32.948	Araip.VE1A8	0.35
Aradu.VI09T	0.348	752	42.762	42.364	33.997	Araip.YT3RJ	0.341
Aradu.VI2RS	0.398	377	56.085	40.476	42.063	Araip.U0RZI	0.374
Aradu.VI7D5	0.367	715	45.95	35.615	37.57	Araip.3W07G	0.366
Aradu.VI8FD	0.353	440	46.259	36.054	43.764	Araip.4CP0D	0.351
Aradu.VIF27	0.406	564	44.779	43.363	49.381	Araip.U4P9E	0.391
Aradu.VIH8G	0.343	106	51.402	36.449	26.168	Araip.BS9NN	0.354
Aradu.VIL5A	0.261	142	60.14	36.364	37.762	Araip.3ZU66	0.286
Aradu.VJ006	0.436	178	45.81	49.162	41.899	Araip.M5WW0	0.432
Aradu.VJ0SY	0.453	777	53.728	46.272	61.311	Araip.7A7AE	0.441
Aradu.VJ2JF	0.328	268	57.249	44.238	31.599	Araip.4S2KN	0.333
Aradu.VJK1R	0.424	442	46.727	40.181	38.826	Araip.AI81V	0.429
Aradu.VK1JK	0.33	376	49.337	42.44	37.931	Araip.02HSD	0.339
Aradu.VK4A9	0.367	654	43.511	49.466	34.656	Araip.5NJ9K	0.372
Aradu.VK4DU	0.394	236	50.211	42.194	48.523	Araip.V5XRP	0.39
Aradu.VK692	0.35	258	37.838	53.282	30.116	Araip.F9Y3B	0.345
Aradu.VK6NM	0.353	475	46.849	38.445	38.866	Araip.GC0LN	0.353
Aradu.VK93Q	0.354	1252	50.359	40.623	33.759	Araip.6IZ1V	0.352
Aradu.VK984	0.386	930	48.98	37.057	42.105	Araip.X1YIB	0.385
Aradu.VK9S0	0.393	308	47.896	34.951	37.54	Araip.K5MNX	0.399
Aradu.VKB5P	0.368	320	53.894	38.941	36.76	Araip.8K7MC	0.344
Aradu.VKC3S	0.363	579	44.828	38.966	41.034	Araip.NH2DF	0.397
Aradu.VKJ07	0.425	229	45.217	32.174	48.696	Araip.7827F	0.42
Aradu.VKS8C	0.378	622	45.907	35.795	38.523	Araip.W9PNA	0.372
Aradu.VL23N	0.333	558	46.869	48.658	34.168	Araip.D5A51	0.337
Aradu.VL3IZ	0.382	343	47.093	44.477	44.767	Araip.KXH96	0.385
Aradu.VLM3K	0.427	150	60.265	47.02	37.748	Araip.JDU8H	0.448
Aradu.VM1W8	0.347	201	46.535	38.119	50	Araip.EZS4N	0.341
Aradu.VM246	0.397	321	59.006	40.373	36.335	Araip.858RT	0.399
Aradu.VM38K	0.406	163	63.415	45.122	69.512	Araip.308FD	0.419
Aradu.VM6CH	0.359	375	40.957	39.096	41.489	Araip.H9AVS	0.378
Aradu.VM6WK	0.418	147	49.324	40.541	39.189	Araip.Z58RU	0.44
Aradu.VM8XK	0.348	624	45.6	34.08	38.56	Araip.X0ZRE	0.36
Aradu.VM94P	0.389	332	52.252	43.844	37.538	Araip.VD2UK	0.404
Aradu.VMK7V	0.381	459	46.739	41.087	43.043	Araip.E2XDB	0.364
Aradu.VN6S3	0.335	452	44.371	38.852	35.099	Araip.FTB4U	0.332
Aradu.VN8X3	0.399	432	42.494	39.723	40.878	Araip.HGV90	0.394
Aradu.VP5WD	0.357	354	46.479	42.817	39.718	Araip.LA5WR	0.343
Aradu.VPD9Z	0.39	356	49.86	41.737	34.174	Araip.JA8PY	0.386
Aradu.VQ1MT	0.377	817	50.611	38.631	40.22	Araip.BBT8F	0.379

Aradu.VQ1N9	0.367	858	47.031	41.56	37.485	Araip.05E6K	0.366
Aradu.VQ2JX	0.375	2192	54.537	43.046	32.057	Araip.E13P0	0.375
Aradu.VQA02	0.384	1029	44.369	36.311	38.252	Araip.ZW17J	0.387
Aradu.VQB2Q	0.403	197	44.444	48.485	37.374	Araip.V7U9F	0.411
Aradu.VQJ7V	0.359	123	51.613	38.71	43.548	Araip.R2GRG	0.376
Aradu.VR7VT	0.385	762	49.41	39.45	38.27	Araip.6Q6H8	0.387
Aradu.VRG75	0.39	329	43.333	38.485	37.273	Araip.67BDE	0.397
Aradu.VRG8M	0.362	322	43.653	40.248	42.105	Araip.N5RHE	0.374
Aradu.VRW76	0.385	411	47.573	37.864	39.32	Araip.T00RM	0.394
Aradu.VS07W	0.409	582	52.659	41.681	41.166	Araip.79RU1	0.419
Aradu.VS34U	0.414	225	54.425	42.92	45.575	Araip.JS6GC	0.432
Aradu.VS4QJ	0.343	800	45.693	36.454	43.695	Araip.1ZK8J	0.343
Aradu.VS9VK	0.37	327	50.305	39.634	35.061	Araip.7P78Q	0.361
Aradu.VSA8A	0.402	493	52.227	48.583	42.713	Araip.5X628	0.414
Aradu.VSE1D	0.393	426	43.794	37.939	43.56	Araip.V48CN	0.375
Aradu.VSF4G	0.371	696	50.789	41.033	51.793	Araip.UGC3H	0.363
Aradu.VT0DP	0.438	635	52.987	41.509	54.717	Araip.J9D4H	0.44
Aradu.VT3C0	0.382	698	47.353	39.914	42.203	Araip.W7YQ0	0.379
Aradu.VT5GA	0.309	340	48.68	46.921	29.912	Araip.4K0XH	0.309
Aradu.VTB62	0.411	214	52.093	50.233	52.093	Araip.Y561F	0.431
Aradu.VTZ6D	0.377	603	53.146	38.742	35.265	Araip.WW8Y7	0.379
Aradu.VU792	0.385	1297	51.31	40.601	36.133	Araip.YJ40C	0.383
Aradu.VUE49	0.36	955	50.105	34.833	38.285	Araip.99AZK	0.354
Aradu.VUQ4V	0.462	357	54.749	37.989	50.838	Araip.BRP4U	0.451
Aradu.VUW7M	0.357	660	48.109	38.729	38.88	Araip.T371H	0.369
Aradu.VUX24	0.409	326	55.352	45.26	51.376	Araip.8JJ8B	0.394
Aradu.VV1WJ	0.361	475	46.008	37.815	43.277	Araip.JRA2H	0.357
Aradu.VV3RK	0.365	260	39.08	45.977	42.529	Araip.ZJ5LN	0.364
Aradu.VV430	0.353	556	49.91	42.549	42.37	Araip.6W0QH	0.364
Aradu.VV69K	0.428	730	51.984	39.398	42.134	Araip.XLP07	0.43
Aradu.VVM35	0.418	197	46.465	35.859	38.889	Araip.IK2WW	0.418
Aradu.VVR1U	0.378	935	51.923	38.782	42.735	Araip.EKA65	0.393
Aradu.VW26U	0.411	460	54.013	43.818	41.432	Araip.091L0	0.409
Aradu.VW4T4	0.343	918	51.034	41.458	34.385	Araip.UX0Q8	0.343
Aradu.VW5KP	0.392	263	51.136	42.045	39.773	Araip.RLY0Z	0.384
Aradu.VW8XJ	0.456	287	48.264	46.181	41.667	Araip.989UZ	0.457
Aradu.VW946	0.376	564	49.558	40	51.15	Araip.3HJ42	0.375
Aradu.VW9JZ	0.373	397	51.759	37.94	38.693	Araip.L6WEG	0.365
Aradu.VWQ48	0.364	200	50.249	59.701	36.318	Araip.MX9CS	0.36
Aradu.VX0W5	0.329	233	41.026	35.043	39.744	Araip.JLU3W	0.329
Aradu.VX1FR	0.405	1352	52.106	34.664	39.542	Araip.67SLS	0.404
Aradu.VX30A	0.464	116	56.41	37.607	58.974	Araip.JQ4PF	0.5
Aradu.VX595	0.401	802	49.315	37.484	51.806	Araip.VH9FH	0.403
Aradu.VXG8Q	0.426	471	54.025	39.619	51.059	Araip.XE7B2	0.416

Aradu.VXK5T	0.491	305	45.425	49.673	47.712	Araip.4Y3B5	0.488
Aradu.VXS15	0.359	365	48.907	35.519	38.525	Araip.Q9692	0.355
Aradu.VY0ZL	0.424	154	42.581	33.548	64.516	Araip.2UA97	0.43
Aradu.VY6JK	0.451	218	52.968	37.9	62.557	Araip.F8CEG	0.432
Aradu.VY9WF	0.385	1123	52.936	44.128	36.833	Araip.363DR	0.388
Aradu.VZ2PZ	0.428	349	51.714	45.143	41.143	Araip.3B7EF	0.407
Aradu.VZ331	0.444	371	59.14	43.548	53.495	Araip.G7U46	0.441
Aradu.VZ497	0.367	348	54.728	46.132	34.384	Araip.L4V14	0.366
Aradu.VZ4PC	0.354	391	50.765	44.133	37.245	Araip.UDU7A	0.362
Aradu.VZ8XR	0.363	486	46.201	35.729	33.676	Araip.T1BG2	0.356
Aradu.VZQ8X	0.369	443	45.946	47.973	37.162	Araip.R6M68	0.369
Aradu.W01YL	0.363	452	51.656	40.397	35.541	Araip.0MR58	0.354
Aradu.W06MF	0.414	360	50.97	46.26	44.875	Araip.BYF4T	0.414
Aradu.W07KG	0.377	896	50.056	37.904	39.576	Araip.NWR3L	0.377
Aradu.W0B0I	0.375	530	47.458	41.996	39.36	Araip.37594	0.375
Aradu.W0BBS	0.369	382	47.258	40.731	32.637	Araip.IA30F	0.359
Aradu.W0FED	0.368	268	41.636	42.379	47.955	Araip.V4WLJ	0.372
Aradu.W0GZN	0.377	441	54.072	37.33	45.475	Araip.QB1DK	0.386
Aradu.W0ILV	0.414	292	52.218	41.638	40.614	Araip.01CXT	0.399
Aradu.W0IT8	0.384	597	48.662	38.462	44.147	Araip.H3SL4	0.378
Aradu.W0R38	0.4	104	47.619	44.762	46.667	Araip.YBS5U	0.418
Aradu.W0TSV	0.443	221	50	44.144	49.099	Araip.NID0Y	0.452
Aradu.W0WNS	0.376	355	45.225	41.011	39.607	Araip.H47JD	0.402
Aradu.W1519	0.351	817	49.022	39.487	36.797	Araip.9ER14	0.346
Aradu.W162D	0.517	365	55.191	41.53	68.033	Araip.BU3C8	0.525
Aradu.W1AGE	0.401	466	55.246	41.542	40.257	Araip.GNF5N	0.402
Aradu.W1BNF	0.444	555	49.46	41.187	54.137	Araip.BTU8P	0.446
Aradu.W1WQU	0.329	155	41.667	35.897	33.974	Araip.5R38D	0.329
Aradu.W1X0Q	0.397	386	47.804	40.31	44.961	Araip.9TJ4Z	0.377
Aradu.W1XFV	0.374	605	49.67	41.584	36.469	Araip.E2793	0.375
Aradu.W20TC	0.358	413	53.623	39.13	40.821	Araip.KLX3M	0.359
Aradu.W225Y	0.395	750	47.27	39.68	35.819	Araip.YX11Q	0.397
Aradu.W23YF	0.364	178	62.57	37.43	51.955	Araip.1H048	0.37
Aradu.W24J0	0.38	805	50.372	45.409	40.074	Araip.YR3WS	0.376
Aradu.W2A66	0.348	320	52.025	35.826	44.86	Araip.AW13W	0.347
Aradu.W2C81	0.499	905	51.987	41.832	64.349	Araip.YBR8J	0.498
Aradu.W2FE7	0.389	201	59.901	36.139	63.366	Araip.Y1DIL	0.389
Aradu.W2IGQ	0.336	372	42.627	42.091	43.432	Araip.SB79H	0.335
Aradu.W2MG5	0.379	766	51.63	37.158	46.675	Araip.423MS	0.387
Aradu.W2RXK	0.397	636	46.468	40.816	35.008	Araip.08M0G	0.404
Aradu.W2UK2	0.388	527	52.273	38.826	48.485	Araip.79E4D	0.397
Aradu.W2VYJ	0.461	521	53.64	39.464	58.812	Araip.KL8C5	0.459
Aradu.W34NY	0.392	346	57.349	44.092	39.193	Araip.WYG4Z	0.392
Aradu.W3BNI	0.425	601	56.312	37.542	53.488	Araip.9J3MN	0.422

Aradu.W3DTP	0.434	250	56.175	52.59	34.263	Araip.N7IGQ	0.438
Aradu.W3ELN	0.381	496	47.485	42.857	33.602	Araip.L3N8P	0.374
Aradu.W3GLH	0.362	679	44.118	36.765	39.265	Araip.79TDF	0.359
Aradu.W3H15	0.403	485	50.412	39.095	45.473	Araip.T94UV	0.405
Aradu.W3KTW	0.362	681	50.587	39.883	36.51	Araip.R5DP2	0.361
Aradu.W3UAF	0.445	427	56.542	39.019	58.411	Araip.UGL36	0.448
Aradu.W3VUI	0.382	690	54.269	41.679	40.232	Araip.PPD7W	0.38
Aradu.W3YJN	0.382	453	50	36.344	45.374	Araip.69F5Y	0.385
Aradu.W44WH	0.437	238	44.77	43.515	51.883	Araip.I0PWR	0.429
Aradu.W45TC	0.392	205	56.311	33.981	28.155	Araip.W7Q56	0.389
Aradu.W48TA	0.384	165	47.59	37.952	35.542	Araip.X6683	0.382
Aradu.W4I1P	0.391	490	48.88	36.049	45.01	Araip.036V7	0.393
Aradu.W4N58	0.354	786	48.158	37.992	40.152	Araip.B0TCT	0.35
Aradu.W4PG1	0.332	448	44.543	35.635	37.416	Araip.968KT	0.332
Aradu.W4PMX	0.45	190	42.408	49.738	53.927	Araip.Y9RA8	0.458
Aradu.W4RCV	0.392	253	52.362	35.039	41.339	Araip.46XVA	0.414
Aradu.W4S0X	0.388	147	48.649	29.73	42.568	Araip.X1U9E	0.397
Aradu.W4W1A	0.394	1018	49.951	37.782	36.801	Araip.89N01	0.396
Aradu.W4XF0	0.566	269	36.667	27.037	54.815	Araip.IX47H	0.563
Aradu.W4XL4	0.372	427	56.075	40.421	36.682	Araip.R9908	0.372
Aradu.W4ZB9	0.369	422	58.392	48.463	34.515	Araip.67ZY4	0.36
Aradu.W51GD	0.425	445	44.395	45.964	40.583	Araip.XB3PS	0.428
Aradu.W51SD	0.39	694	54.101	41.151	39.281	Araip.EN37W	0.392
Aradu.W56IJ	0.366	970	44.181	39.135	36.869	Araip.1V89E	0.368
Aradu.W56R3	0.409	305	46.732	39.869	35.948	Araip.XY63T	0.405
Aradu.W58GD	0.421	354	49.859	54.93	42.535	Araip.W28KY	0.411
Aradu.W5BH1	0.321	363	48.626	40.385	36.813	Araip.J80SY	0.292
Aradu.W5GQC	0.415	962	53.375	42.264	40.914	Araip.DFU9Y	0.414
Aradu.W5INW	0.387	539	42.407	37.222	38.704	Araip.357WQ	0.378
Aradu.W5JRX	0.376	205	49.029	37.379	44.66	Araip.GZM57	0.337
Aradu.W5KRK	0.369	346	52.161	44.669	44.669	Araip.RD4BX	0.372
Aradu.W5WIK	0.408	605	48.845	48.185	42.079	Araip.N8HHR	0.38
Aradu.W64DR	0.357	580	45.955	39.071	32.186	Araip.ET6IJ	0.357
Aradu.W69K1	0.435	221	48.649	31.532	64.414	Araip.8G9ZI	0.432
Aradu.W6H6W	0.356	372	49.33	36.193	34.048	Araip.GP5B8	0.365
Aradu.W6JL4	0.412	384	47.013	40.779	40.519	Araip.F5941	0.401
Aradu.W6T3N	0.4	620	60.064	44.928	44.605	Araip.RB35J	0.403
Aradu.W6TAQ	0.401	810	54.624	38.964	40.567	Araip.5QY7Z	0.399
Aradu.W71G4	0.495	390	56.01	39.386	57.033	Araip.11BKV	0.483
Aradu.W71HY	0.348	361	52.486	41.436	46.409	Araip.6S6W2	0.357
Aradu.W75H1	0.346	220	49.774	40.724	38.462	Araip.E1WBJ	0.355
Aradu.W7CRW	0.423	585	52.389	46.587	62.287	Araip.J8QA5	0.426
Aradu.W7CVC	0.403	535	54.851	44.963	38.06	Araip.8E97X	0.406
Aradu.W7CVG	0.375	522	43.403	38.241	41.491	Araip.LT700	0.371

Aradu.W7H9E	0.405	884	52.881	44.068	38.644	Araip.RE6G3	0.402
Aradu.W7HND	0.367	724	53.103	38.069	36.552	Araip.S195L	0.366
Aradu.W7JB7	0.381	202	52.217	45.32	30.542	Araip.2SG63	0.397
Aradu.W7KRJ	0.364	271	45.956	44.118	40.809	Araip.TC7E2	0.351
Aradu.W7LFI	0.51	210	51.659	46.445	58.294	Araip.W4DNX	0.487
Aradu.W7MG7	0.358	491	47.764	40.447	39.228	Araip.6C94R	0.359
Aradu.W801G	0.466	163	53.049	44.512	41.463	Araip.8K98A	0.438
Aradu.W87GJ	0.397	149	48.667	40	70	Araip.R1LY9	0.411
Aradu.W88FU	0.373	385	56.218	40.674	35.233	Araip.LJ1YI	0.35
Aradu.W8J78	0.492	192	57.513	41.969	64.767	Araip.X04AD	0.484
Aradu.W8K8D	0.363	506	53.254	38.856	45.365	Araip.9J3NW	0.363
Aradu.W8MVJ	0.504	131	56.061	43.182	56.818	Araip.6T35D	0.504
Aradu.W8SNB	0.403	301	48.344	44.04	41.06	Araip.7WS9N	0.409
Aradu.W903S	0.368	528	46.881	33.459	40.832	Araip.8Y8HZ	0.358
Aradu.W912S	0.418	760	53.482	50.854	34.691	Araip.4KH5L	0.423
Aradu.W98YX	0.408	472	50.74	36.152	43.34	Araip.F66CA	0.402
Aradu.W9DC5	0.525	145	61.644	32.192	63.014	Araip.J92VB	0.511
Aradu.W9ER9	0.415	1087	51.287	47.794	31.526	Araip.D91QG	0.414
Aradu.W9G7C	0.34	727	52.473	40.797	36.676	Araip.2528D	0.34
Aradu.W9GMQ	0.393	381	56.545	37.435	39.267	Araip.Q2M7H	0.389
Aradu.W9IJK	0.449	479	47.708	39.583	46.458	Araip.AB17F	0.45
Aradu.W9Q7M	0.39	203	54.902	38.725	45.098	Araip.JYD7I	0.395
Aradu.W9SJN	0.348	315	46.203	52.215	33.544	Araip.KCM6L	0.343
Aradu.W9TQV	0.444	604	54.876	35.868	48.099	Araip.CU8VC	0.434
Aradu.W9VU7	0.384	471	50.424	35.593	37.712	Araip.VF78K	0.395
Aradu.WA3AU	0.359	1144	53.188	49.694	39.651	Araip.43W52	0.357
Aradu.WAB2C	0.374	562	48.49	42.984	50.266	Araip.XF6W8	0.375
Aradu.WAT9N	0.405	247	50.403	32.258	32.661	Araip.MZJ7P	0.436
Aradu.WB45H	0.312	541	59.779	55.535	33.948	Araip.99J68	0.311
Aradu.WB4GB	0.437	393	55.584	40.609	63.959	Araip.JD11C	0.44
Aradu.WB4HU	0.313	425	43.192	34.742	40.141	Araip.R7MQK	0.323
Aradu.WB5Q0	0.414	263	40.53	32.955	39.773	Araip.5MD11	0.411
Aradu.WB5VJ	0.406	451	51.327	38.274	41.593	Araip.VC3BC	0.408
Aradu.WB7Y4	0.417	229	52.609	37.391	43.478	Araip.CSG3E	0.417
Aradu.WB83L	0.435	182	39.891	36.066	46.448	Araip.J0SL5	0.44
Aradu.WB9GS	0.368	165	55.422	30.723	53.012	Araip.STR9D	0.368
Aradu.WBB7S	0.395	688	49.637	37.881	41.655	Araip.AE3J6	0.395
Aradu.WBJ0E	0.427	302	53.795	46.535	44.554	Araip.I0LNV	0.42
Aradu.WBK0P	0.451	422	51.773	46.336	42.553	Araip.AJ7AK	0.452
Aradu.WBM9H	0.389	476	59.958	43.606	39.832	Araip.C0E46	0.391
Aradu.WC1MX	0.41	711	54.354	42.556	34.551	Araip.337EV	0.419
Aradu.WC37M	0.343	345	46.243	35.26	43.642	Araip.VS4IG	0.346
Aradu.WC9V5	0.365	391	43.112	33.929	35.714	Araip.JIB5P	0.379
Aradu.WDS9Z	0.366	544	45.872	46.422	31.927	Araip.X05NJ	0.369

Aradu.WE6GV	0.352	375	52.394	31.915	43.351	Araip.7KT8S	0.319
Aradu.WEZ9F	0.406	343	50.581	37.5	39.826	Araip.JYT9K	0.412
Aradu.WF5M4	0.447	301	42.715	37.086	48.675	Araip.D814V	0.464
Aradu.WF6VN	0.352	702	49.644	35.277	37.696	Araip.UZ4WB	0.36
Aradu.WFC0U	0.367	777	46.015	37.147	42.159	Araip.HD6GL	0.358
Aradu.WFH0K	0.253	104	46.667	22.857	23.81	Araip.G0JJM	0.325
Aradu.WGG5U	0.412	480	46.57	40.333	45.322	Araip.7WV1U	0.418
Aradu.WGT75	0.343	359	51.944	30.556	42.5	Araip.G2KFT	0.346
Aradu.WH2W0	0.402	750	50.333	42.477	54.328	Araip.FU10G	0.403
Aradu.WH49R	0.37	994	44.523	35.98	41.91	Araip.S4S1Y	0.372
Aradu.WH701	0.375	607	48.849	43.092	38.322	Araip.86XAN	0.371
Aradu.WHH4F	0.361	470	52.229	45.435	33.758	Araip.04SWW	0.361
Aradu.WHS6B	0.44	208	49.761	39.713	44.976	Araip.RHZ53	0.455
Aradu.WI4BU	0.371	820	46.163	30.085	40.804	Araip.MWF2H	0.372
Aradu.WIA05	0.441	228	49.782	38.428	37.555	Araip.JK7V6	0.423
Aradu.WIQ29	0.343	110	46.847	35.135	34.234	Araip.CBB88	0.343
Aradu.WIS65	0.43	684	44.964	44.672	51.679	Araip.H4Q41	0.44
Aradu.WJ1IU	0.395	320	42.056	34.579	39.564	Araip.Z2S9D	0.396
Aradu.WJ2TG	0.419	515	55.233	34.884	46.705	Araip.N0DTV	0.412
Aradu.WJ2ZP	0.375	430	54.292	44.316	44.78	Araip.JXV3W	0.372
Aradu.WJ5JK	0.436	494	55.354	37.778	60.606	Araip.7FJ61	0.438
Aradu.WJ6G7	0.389	321	49.068	38.509	37.578	Araip.0U7A4	0.387
Aradu.WJ709	0.449	462	56.371	39.525	53.564	Araip.IWA7R	0.449
Aradu.WJU1S	0.419	163	50.61	42.073	37.195	Araip.I08KI	0.412
Aradu.WJZ15	0.35	423	50.708	39.623	40.566	Araip.PKN8E	0.35
Aradu.WJZ76	0.329	532	46.154	40.713	36.585	Araip.EK3ME	0.327
Aradu.WKA92	0.381	980	50.051	38.532	34.047	Araip.V59P2	0.392
Aradu.WL13F	0.36	886	46.336	39.91	36.189	Araip.60K6G	0.361
Aradu.WL1AY	0.38	371	51.344	41.129	34.677	Araip.FT330	0.375
Aradu.WL4PN	0.407	124	48	35.2	51.2	Araip.H7PIN	0.415
Aradu.WL7ND	0.348	232	43.777	37.339	39.056	Araip.YNV3J	0.329
Aradu.WL92I	0.46	223	58.929	38.393	48.214	Araip.KA9ES	0.463
Aradu.WLB92	0.377	460	53.796	47.289	38.178	Araip.G0Q9N	0.369
Aradu.WLD4B	0.385	477	49.163	41.213	39.121	Araip.R3KEZ	0.373
Aradu.WLH9X	0.39	1333	52.774	41.379	35.457	Araip.5JH6G	0.387
Aradu.WLL49	0.406	350	52.707	39.031	39.316	Araip.NZ168	0.412
Aradu.WLS5Y	0.398	221	47.297	44.144	34.685	Araip.QT426	0.391
Aradu.WLU86	0.409	230	57.143	35.931	47.619	Araip.F0BPT	0.413
Aradu.WLX6I	0.405	587	56.973	35.034	39.456	Araip.MJ1PB	0.394
Aradu.WLZ4Q	0.39	696	46.198	39.598	47.633	Araip.04VV6	0.389
Aradu.WM0X2	0.453	510	51.859	45.01	62.427	Araip.1S5XZ	0.444
Aradu.WM4V4	0.43	472	51.797	38.266	56.66	Araip.M83DH	0.433
Aradu.WM652	0.386	733	47.275	36.512	34.469	Araip.3Y8R0	0.386
Aradu.WMN78	0.354	574	52	40.522	31.478	Araip.D85BU	0.349

Aradu.WN017	0.369	475	43.487	34.034	45.168	Araip.8VB8P	0.366
Aradu.WNH12	0.357	265	48.12	35.714	42.481	Araip.C4L8E	0.356
Aradu.WNQ8E	0.463	319	57.188	42.813	58.125	Araip.W6M4V	0.47
Aradu.WNV5J	0.394	469	54.681	39.362	36.17	Araip.2LE64	0.39
Aradu.WP07M	0.398	200	51.244	38.806	35.821	Araip.F4TTY	0.392
Aradu.WP30A	0.453	222	46.188	43.498	38.565	Araip.A5AHR	0.435
Aradu.WPA2I	0.361	112	35.398	38.938	38.053	Araip.Z6RXC	0.361
Aradu.WQ0V2	0.4	487	47.131	47.336	34.016	Araip.V3UEW	0.402
Aradu.WQ4BK	0.41	380	49.081	47.244	46.719	Araip.L6DF6	0.42
Aradu.WQI06	0.418	277	50	38.129	46.763	Araip.P0TWG	0.433
Aradu.WQI5W	0.435	1304	49.042	43.372	53.87	Araip.85DUV	0.429
Aradu.WQL62	0.37	351	49.716	38.92	44.034	Araip.X37CH	0.382
Aradu.WQP25	0.319	442	44.695	37.923	32.28	Araip.E60FJ	0.306
Aradu.WR10B	0.369	307	53.896	40.26	44.805	Araip.VR692	0.386
Aradu.WRA79	0.356	434	47.356	33.563	39.77	Araip.E4EEK	0.35
Aradu.WRB0S	0.361	440	49.206	44.898	37.868	Araip.Z9GTK	0.363
Aradu.WS13Y	0.403	261	57.634	41.603	40.076	Araip.2K9WW	0.407
Aradu.WS4CD	0.374	696	50.502	39.885	39.455	Araip.EM233	0.383
Aradu.WSH4V	0.334	381	37.696	35.079	33.246	Araip.R1LM3	0.336
Aradu.WSK5V	0.387	325	55.521	52.147	38.037	Araip.IN8QT	0.402
Aradu.WT2VD	0.37	809	47.531	41.728	38.272	Araip.KX3VJ	0.374
Aradu.WT391	0.399	366	49.046	38.965	40.054	Araip.N1QWX	0.39
Aradu.WT71X	0.423	367	47.554	38.859	57.88	Araip.DDF9G	0.427
Aradu.WU1XZ	0.364	828	51.146	41.858	35.706	Araip.8YJ09	0.368
Aradu.WU2UF	0.473	184	43.243	40.541	52.973	Araip.VYR7U	0.462
Aradu.WU7D1	0.369	825	49.031	39.952	37.409	Araip.162FJ	0.347
Aradu.WUH7T	0.393	345	50.289	39.595	41.908	Araip.FFV1Z	0.399
Aradu.WUP1N	0.378	446	56.152	42.953	46.532	Araip.3I7ZD	0.378
Aradu.WUW36	0.398	223	58.929	42.857	46.875	Araip.0B5Q5	0.394
Aradu.WVC4I	0.495	119	50.833	45	60	Araip.35KW5	0.495
Aradu.WW0P3	0.376	482	46.584	37.474	45.135	Araip.M4X9J	0.371
Aradu.WW2SY	0.427	538	46.939	36.735	41.373	Araip.74PIQ	0.426
Aradu.WW65Q	0.406	258	43.629	41.313	49.807	Araip.6P6UG	0.412
Aradu.WWA28	0.421	137	51.449	44.203	50	Araip.583LJ	0.398
Aradu.WWM41	0.429	262	50.951	42.966	57.414	Araip.F2XI1	0.417
Aradu.WWY9R	0.433	669	51.493	40.149	47.164	Araip.6SM8U	0.436
Aradu.WX32R	0.556	359	58.889	42.222	80.556	Araip.QJ6DW	0.549
Aradu.WXB2Z	0.399	349	51.429	34	38	Araip.L77XA	0.37
Aradu.WYR1J	0.48	574	57.217	39.13	58.609	Araip.C95X7	0.482
Aradu.WYX50	0.409	782	54.151	41.507	48.531	Araip.JRH45	0.397
Aradu.WYZ5E	0.363	323	54.63	34.259	39.198	Araip.0MR1X	0.355
Aradu.WZ2NF	0.402	130	57.252	48.855	37.405	Araip.U7E4D	0.373
Aradu.WZ6G2	0.447	133	46.269	33.582	47.761	Araip.80KYM	0.455
Aradu.X03Q4	0.456	282	48.41	40.283	55.83	Araip.PD52B	0.465

Aradu.X0CJD	0.349	1603	50.561	38.092	35.411	Araip.N3ZNP	0.352
Aradu.X0EAG	0.373	1047	46.469	40.172	34.447	Araip.P4BJL	0.374
Aradu.X0F3K	0.34	355	46.91	39.888	36.517	Araip.X3UTX	0.345
Aradu.X0F96	0.37	452	51.214	40.839	36.424	Araip.3V9D5	0.354
Aradu.X0HV8	0.479	200	52.239	39.801	46.269	Araip.CW1SE	0.482
Aradu.X0I6L	0.371	273	57.299	36.131	47.445	Araip.38ZCB	0.382
Aradu.X0KMT	0.376	1026	54.138	44.888	35.54	Araip.CSV1L	0.374
Aradu.X0LHP	0.35	394	53.671	34.937	36.203	Araip.NPG6G	0.342
Aradu.X0QAS	0.399	333	40.419	38.024	40.12	Araip.FZ3N9	0.391
Aradu.X0SMT	0.436	204	60	45.366	40.488	Araip.K3RKP	0.448
Aradu.X0Z7E	0.39	849	50.824	38	39.765	Araip.67DP4	0.387
Aradu.X10AK	0.398	487	48.566	40.779	54.918	Araip.A87RC	0.398
Aradu.X114S	0.412	414	50.361	42.41	46.265	Araip.N29YP	0.407
Aradu.X1ANV	0.397	289	44.138	40	42.069	Araip.FU667	0.366
Aradu.X1DZR	0.375	301	45.695	29.47	38.079	Araip.4R2IW	0.372
Aradu.X1LTA	0.423	569	53.86	39.474	42.632	Araip.31XHT	0.42
Aradu.X1MH8	0.369	313	48.726	40.446	35.987	Araip.R4JRM	0.366
Aradu.X1PQU	0.432	308	54.369	36.57	49.191	Araip.T9QZJ	0.436
Aradu.X1Y61	0.391	425	56.103	41.315	40.61	Araip.S985N	0.403
Aradu.X22EB	0.376	102	48.544	51.456	44.66	Araip.ZQ9EU	0.376
Aradu.X23JQ	0.485	237	61.345	47.059	62.185	Araip.2G7LE	0.498
Aradu.X23MZ	0.416	346	53.602	38.329	43.804	Araip.QUX0P	0.41
Aradu.X258N	0.405	443	52.027	41.892	39.189	Araip.LUU1Z	0.411
Aradu.X2HBL	0.493	390	59.335	48.849	70.588	Araip.AI3RV	0.488
Aradu.X2I4E	0.449	137	54.348	47.826	48.551	Araip.HPW81	0.441
Aradu.X2LCY	0.347	277	46.043	37.77	34.892	Araip.KC5KI	0.358
Aradu.X2T4X	0.374	341	51.754	44.444	41.52	Araip.R4XYF	0.371
Aradu.X2X3M	0.355	1284	52.374	35.486	41.946	Araip.US77A	0.357
Aradu.X2Y57	0.411	210	54.502	36.019	46.445	Araip.Q1BR2	0.409
Aradu.X33LT	0.452	276	54.874	44.765	38.628	Araip.Y0RK1	0.47
Aradu.X37CM	0.416	173	56.897	41.954	40.805	Araip.4UA5Y	0.414
Aradu.X3A7J	0.358	479	46.875	39.583	43.125	Araip.JGK52	0.353
Aradu.X3DNX	0.357	209	43.333	35.238	47.619	Araip.UGV6F	0.355
Aradu.X3FXV	0.431	264	44.151	53.962	52.83	Araip.K5EKQ	0.42
Aradu.X3GS1	0.487	195	70.408	56.122	49.49	Araip.993UZ	0.492
Aradu.X3GYY	0.33	671	51.637	38.244	38.095	Araip.U32YB	0.334
Aradu.X3LUW	0.466	313	48.726	33.121	59.236	Araip.D2PEY	0.491
Aradu.X3NJ9	0.381	197	50	44.444	41.919	Araip.53HJX	0.386
Aradu.X3R2Q	0.401	493	51.417	42.713	52.429	Araip.SX5JP	0.388
Aradu.X3TFJ	0.379	327	47.561	47.256	37.5	Araip.20IHP	0.38
Aradu.X3U5Y	0.418	238	51.883	44.351	39.749	Araip.84K6K	0.416
Aradu.X3XEZ	0.382	657	51.976	43.161	39.362	Araip.FG09W	0.395
Aradu.X3XXG	0.353	452	44.15	38.19	35.099	Araip.76HFA	0.347
Aradu.X3YIU	0.363	361	49.724	31.492	38.95	Araip.WDY6V	0.377

Aradu.X44N9	0.379	459	50.87	42.391	37.391	Araip.Z3F8H	0.382
Aradu.X49FH	0.345	477	47.071	37.866	33.473	Araip.MN0BK	0.352
Aradu.X4CJ9	0.375	212	42.254	33.803	44.131	Araip.X4V4T	0.37
Aradu.X4DG3	0.437	346	55.043	39.193	39.769	Araip.AEN35	0.431
Aradu.X4G0F	0.382	762	44.037	36.042	41.022	Araip.97W0E	0.378
Aradu.X4GW8	0.458	187	41.489	43.617	42.553	Araip.L5NAQ	0.463
Aradu.X4HEP	0.329	327	43.902	43.902	32.927	Araip.B9NBT	0.332
Aradu.X4JYD	0.381	1009	50.891	45.743	29.208	Araip.YIH0B	0.381
Aradu.X4KI4	0.328	644	44.341	37.519	44.186	Araip.10CZT	0.328
Aradu.X51AF	0.354	257	49.612	33.721	34.109	Araip.7MI21	0.36
Aradu.X53KQ	0.34	661	46.375	35.347	43.353	Araip.L66A6	0.356
Aradu.X5630	0.366	763	51.702	43.325	31.545	Araip.ZI2S3	0.367
Aradu.X57SP	0.393	389	49.744	32.308	41.538	Araip.10PYM	0.382
Aradu.X5BAW	0.5	216	50.23	45.622	44.7	Araip.V2QG1	0.5
Aradu.X5GZZ	0.377	416	50.6	37.41	36.691	Araip.KK7VF	0.375
Aradu.X5JSA	0.42	538	48.052	44.156	55.473	Araip.TZY7L	0.43
Aradu.X5TRC	0.489	135	49.265	35.294	44.118	Araip.B9RM0	0.496
Aradu.X5WR6	0.387	776	55.212	40.154	38.224	Araip.Q6TYI	0.383
Aradu.X66TP	0.368	357	46.369	34.078	37.151	Araip.525WX	0.358
Aradu.X685X	0.373	214	44.651	40.93	33.953	Araip.6G7UA	0.364
Aradu.X689P	0.441	328	48.936	33.131	40.729	Araip.L11FI	0.439
Aradu.X69MW	0.341	126	48.819	48.031	48.819	Araip.T8ZUZ	0.421
Aradu.X6AKD	0.378	175	46.023	46.023	35.227	Araip.AM4LP	0.378
Aradu.X6BWV	0.401	935	50	43.056	53.632	Araip.GGP20	0.402
Aradu.X6DIS	0.427	431	45.139	42.361	35.648	Araip.T29CT	0.419
Aradu.X6FMF	0.402	800	53.933	45.318	47.066	Araip.I5L57	0.401
Aradu.X6KFI	0.346	135	47.794	39.706	49.265	Araip.ATF8R	0.318
Aradu.X6KLL	0.525	325	53.988	40.798	54.908	Araip.B6R33	0.534
Aradu.X6LF1	0.446	394	57.722	40.506	52.658	Araip.XJS45	0.439
Aradu.X6P8N	0.407	100	43.564	34.653	64.356	Araip.M21JP	0.404
Aradu.X6VZP	0.379	474	51.579	39.789	33.053	Araip.164TB	0.383
Aradu.X763N	0.391	448	41.871	38.307	47.439	Araip.R98BP	0.391
Aradu.X7855	0.371	227	59.211	36.842	37.281	Araip.6Y4LC	0.386
Aradu.X7B4K	0.31	471	46.822	36.441	37.712	Araip.FTC1S	0.312
Aradu.X7LBF	0.445	253	46.063	47.244	46.063	Araip.2H669	0.441
Aradu.X7RSP	0.444	401	49.005	39.303	50	Araip.ZT9T9	0.442
Aradu.X7VPA	0.416	143	52.778	48.611	38.194	Araip.4V5MM	0.431
Aradu.X7WGL	0.364	303	38.487	44.079	36.184	Araip.9H56X	0.348
Aradu.X7WYU	0.385	857	47.902	32.867	35.548	Araip.6V57K	0.393
Aradu.X7ZD5	0.384	523	50.763	35.496	44.847	Araip.11WF7	0.363
Aradu.X81G8	0.434	386	51.68	41.86	47.287	Araip.42IVV	0.43
Aradu.X87GF	0.385	366	48.774	34.06	32.425	Araip.W3VW3	0.382
Aradu.X88X0	0.433	569	47.018	37.895	44.912	Araip.T7WB5	0.435
Aradu.X89BI	0.448	578	55.44	41.451	60.276	Araip.5I8PV	0.438

Aradu.X8GPB	0.371	405	47.291	40.64	36.946	Araip.26EKA	0.377
Aradu.X8I5Q	0.328	266	40.824	37.828	35.955	Araip.L0W9L	0.322
Aradu.X8KRI	0.344	319	50	35.625	37.813	Araip.VQH1V	0.34
Aradu.X8LK6	0.453	349	46	37.714	63.143	Araip.XN478	0.436
Aradu.X8MF9	0.336	377	46.561	37.831	30.159	Araip.4I4L7	0.34
Aradu.X8R8H	0.39	1130	52.166	37.754	37.047	Araip.KD41Z	0.387
Aradu.X8R94	0.432	241	51.653	45.041	57.438	Araip.3QG7R	0.419
Aradu.X9447	0.414	325	48.466	48.16	38.037	Araip.N5EXR	0.42
Aradu.X96SI	0.446	495	57.661	38.306	67.339	Araip.U6BJT	0.436
Aradu.X9922	0.386	495	49.597	40.726	39.315	Araip.5J2NZ	0.382
Aradu.X9DDA	0.468	663	56.928	44.88	58.133	Araip.7F2XC	0.472
Aradu.X9GQ3	0.447	108	54.128	52.294	40.367	Araip.1L9GW	0.427
Aradu.X9KA6	0.379	373	47.594	46.524	43.048	Araip.49S7T	0.375
Aradu.X9SEL	0.382	225	55.752	38.053	37.168	Araip.79WPQ	0.369
Aradu.X9U06	0.386	594	55.966	44.37	46.387	Araip.VB5BT	0.384
Aradu.X9YQG	0.262	192	50.259	35.751	26.943	Araip.V6DM9	0.281
Aradu.X9YRF	0.359	877	51.139	42.938	39.408	Araip.8N9UM	0.358
Aradu.X9ZB3	0.344	518	53.372	43.931	37.765	Araip.P5GSM	0.338
Aradu.XA005	0.405	405	48.03	47.291	38.916	Araip.7MJ2M	0.408
Aradu.XA41R	0.37	431	50	36.111	40.046	Araip.8CC6W	0.383
Aradu.XA5F9	0.357	506	48.323	42.209	34.32	Araip.Z8S2H	0.359
Aradu.XA7IS	0.368	450	53.437	39.468	49.667	Araip.49GZ0	0.362
Aradu.XA7KS	0.411	332	51.351	39.039	51.051	Araip.Z8IAR	0.417
Aradu.XAD2K	0.378	152	49.673	35.948	35.948	Araip.K0TZJ	0.381
Aradu.XAE56	0.417	837	49.045	40.931	39.976	Araip.1PU5F	0.423
Aradu.XAX6Y	0.395	383	52.083	39.844	43.49	Araip.Y3JJL	0.392
Aradu.XBE20	0.418	321	50.311	37.267	48.758	Araip.IG5LD	0.408
Aradu.XBM41	0.437	769	54.026	38.052	45.714	Araip.64B54	0.437
Aradu.XBR45	0.493	155	57.692	26.923	61.538	Araip.M2GYW	0.508
Aradu.XBR9P	0.486	181	58.242	38.462	54.945	Araip.CT5HY	0.474
Aradu.XC8VW	0.386	528	49.527	35.539	37.996	Araip.Q3PHC	0.384
Aradu.XCA3M	0.429	340	47.214	38.123	45.748	Araip.J0W46	0.442
Aradu.XD25G	0.463	261	43.511	39.695	48.473	Araip.QL8ZY	0.44
Aradu.XD7VB	0.298	316	53.312	35.962	31.546	Araip.7RK50	0.284
Aradu.XDC1M	0.367	145	55.479	26.712	44.521	Araip.9M0VE	0.367
Aradu.XDC7C	0.407	175	54.545	26.705	34.091	Araip.6YP5U	0.407
Aradu.XDM1U	0.384	265	54.511	36.466	54.135	Araip.101RY	0.394
Aradu.XDM9G	0.332	1201	48.17	37.188	43.594	Araip.3G6B2	0.332
Aradu.XDP02	0.342	431	43.056	34.722	32.87	Araip.K3DD8	0.323
Aradu.XDS84	0.422	435	58.486	38.532	49.541	Araip.LRG7E	0.413
Aradu.XE4CL	0.399	514	57.282	37.476	43.107	Araip.RZ75Q	0.417
Aradu.XE6VM	0.35	506	51.874	40.828	39.645	Araip.98KTC	0.35
Aradu.XEA0F	0.325	302	50.165	37.624	37.954	Araip.PZ75Q	0.334
Aradu.XES29	0.381	657	48.784	42.097	44.833	Araip.FL8FH	0.378

Aradu.XF0PF	0.461	429	57.442	46.047	46.512	Araip.37ILI	0.434
Aradu.XF7S6	0.411	360	59.003	49.861	49.307	Araip.2Z41P	0.408
Aradu.XFR5L	0.376	316	50.789	43.849	41.64	Araip.I71NT	0.384
Aradu.XGC1C	0.385	747	47.193	39.037	45.455	Araip.E0XSF	0.389
Aradu.XGI6B	0.543	145	59.589	41.781	61.644	Araip.8W3B2	0.504
Aradu.XGK7G	0.345	751	44.149	37.633	33.777	Araip.77QS1	0.354
Aradu.XGN4F	0.431	329	49.394	38.182	42.727	Araip.CRU6Z	0.436
Aradu.XH0YF	0.423	956	58.307	35.423	43.469	Araip.TM5WG	0.426
Aradu.XH3MQ	0.472	206	55.072	42.029	47.826	Araip.A1X3Q	0.487
Aradu.XH3ZX	0.383	430	51.276	41.763	40.603	Araip.R9PC3	0.387
Aradu.XHP2L	0.37	340	48.387	39.003	37.537	Araip.I1C79	0.372
Aradu.XHT76	0.435	417	56.22	40.431	43.541	Araip.37P5S	0.425
Aradu.XHT7Q	0.331	825	58.838	41.162	31.719	Araip.U4GJJ	0.333
Aradu.XI961	0.343	644	48.062	40.775	38.295	Araip.SEY9F	0.337
Aradu.XI98N	0.422	236	58.228	52.743	67.932	Araip.VZF8R	0.427
Aradu.XIP04	0.339	995	44.88	37.45	38.855	Araip.TPM3R	0.351
Aradu.XIY30	0.429	587	50.17	41.837	46.088	Araip.RF6HA	0.437
Aradu.XJ1QF	0.339	338	51.917	30.088	38.643	Araip.UI1L0	0.343
Aradu.XJ3JE	0.386	882	50.17	43.828	35.334	Araip.FRI7H	0.385
Aradu.XJ6TW	0.419	578	55.268	33.851	50.604	Araip.9K298	0.438
Aradu.XJF09	0.359	396	53.149	38.791	33.501	Araip.31EFM	0.368
Aradu.XJM2L	0.352	1035	47.973	39.479	42.761	Araip.TW2ID	0.352
Aradu.XK367	0.379	516	54.352	40.232	33.462	Araip.0D2JJ	0.378
Aradu.XK5C3	0.353	2381	51.092	41.226	37.07	Araip.E02MH	0.356
Aradu.XKU5K	0.35	241	47.107	34.711	45.455	Araip.494RG	0.357
Aradu.XL4N2	0.384	489	46.735	33.673	37.959	Araip.VD3IG	0.382
Aradu.XL7AT	0.397	1041	50.672	38.868	37.812	Araip.KA2EW	0.401
Aradu.XLR8N	0.388	607	45.888	39.803	39.145	Araip.J2QMS	0.375
Aradu.XM1M6	0.363	815	50.123	41.544	34.681	Araip.M95YN	0.368
Aradu.XM1WL	0.34	302	50.165	45.875	31.353	Araip.Y2BA4	0.361
Aradu.XM2MR	0.364	433	52.304	38.249	51.382	Araip.RI4NB	0.362
Aradu.XM9I6	0.497	537	54.833	44.238	58.178	Araip.BM7DX	0.498
Aradu.XME24	0.483	123	45.161	32.258	52.419	Araip.H6J0Y	0.475
Aradu.XN3YF	0.391	606	48.105	35.42	30.643	Araip.64V16	0.385
Aradu.XN81L	0.384	364	55.342	50.137	40.822	Araip.74B2T	0.387
Aradu.XN88F	0.318	384	52.208	38.961	33.247	Araip.635QE	0.333
Aradu.XN89N	0.397	196	46.193	41.117	37.563	Araip.K5Y49	0.402
Aradu.XP0GX	0.368	1113	54.668	37.433	40.036	Araip.L3I3U	0.369
Aradu.XP644	0.347	679	52.059	46.618	34.412	Araip.72X39	0.343
Aradu.XPA7G	0.375	405	50	43.596	40.148	Araip.AM8RU	0.378
Aradu.XPZ1I	0.417	411	45.146	36.165	50.971	Araip.0V7N2	0.409
Aradu.XQ1BV	0.341	776	49.936	42.471	40.283	Araip.1F19V	0.337
Aradu.XQ1XQ	0.45	215	51.852	55.093	48.148	Araip.F41IP	0.438
Aradu.XQ3RE	0.493	289	56.552	37.931	61.034	Araip.PRK60	0.493

Aradu.XQ5N2	0.343	686	43.086	42.213	35.662	Araip.AC7TH	0.346
Aradu.XR1NY	0.356	413	43.961	42.754	35.266	Araip.Y3ELL	0.359
Aradu.XR2K7	0.409	314	48.889	43.175	35.873	Araip.C95P2	0.416
Aradu.XR3BY	0.345	509	56.667	40.784	43.333	Araip.7YJ6V	0.36
Aradu.XR3QS	0.39	2146	51.514	42.944	35.026	Araip.4G3PJ	0.394
Aradu.XRA1G	0.428	596	54.439	38.191	40.201	Araip.4P4HG	0.431
Aradu.XRM9M	0.335	203	56.863	28.431	37.745	Araip.G25KD	0.325
Aradu.XRX70	0.401	756	49.406	36.196	46.367	Araip.A8INI	0.393
Aradu.XS31R	0.351	927	45.151	42.78	34.806	Araip.1V6N7	0.354
Aradu.XSB36	0.524	521	51.533	41.571	78.161	Araip.UJ6B2	0.525
Aradu.XSZ7L	0.362	132	35.338	31.579	39.85	Araip.J3G2M	0.389
Aradu.XT2G5	0.427	543	49.632	40.441	50.368	Araip.KZ32L	0.423
Aradu.XT8CD	0.402	624	52.96	42.88	44	Araip.Y7X9R	0.402
Aradu.XTT89	0.409	352	51.558	33.428	50.425	Araip.AQR58	0.408
Aradu.XU2Q6	0.495	325	63.19	48.16	63.497	Araip.V35VM	0.482
Aradu.XU3IM	0.417	266	50.562	45.318	54.682	Araip.AC5JZ	0.435
Aradu.XU97Z	0.348	688	49.347	40.493	35.414	Araip.EHI93	0.345
Aradu.XV2LW	0.342	350	51.567	34.188	36.752	Araip.ARS4V	0.322
Aradu.XV49R	0.407	1189	53.613	43.445	40.756	Araip.HCE40	0.404
Aradu.XV773	0.46	339	50	34.412	53.529	Araip.T0YFD	0.46
Aradu.XVQ80	0.367	357	51.117	37.151	34.916	Araip.06WGU	0.372
Aradu.XVQ98	0.378	1033	46.325	40.039	34.429	Araip.G8VRW	0.378
Aradu.XVT29	0.375	523	46.183	39.122	36.641	Araip.VI7E7	0.378
Aradu.XW1R1	0.4	732	49.523	30.696	44.065	Araip.D5WHX	0.405
Aradu.XW8AZ	0.41	504	48.713	39.406	53.267	Araip.ZD9NB	0.407
Aradu.XW987	0.321	319	47.5	40.625	41.563	Araip.1Z8DE	0.326
Aradu.XWZ7Z	0.405	380	53.281	43.832	51.444	Araip.P6LSC	0.405
Aradu.XX1YI	0.339	129	40	39.231	50	Araip.9B7I5	0.355
Aradu.XX2R4	0.395	664	47.519	36.09	49.624	Araip.D1TZE	0.395
Aradu.XX5Y6	0.522	423	64.623	47.406	58.491	Araip.SRP5E	0.531
Aradu.XY3GQ	0.426	210	59.242	44.076	44.55	Araip.SNP97	0.426
Aradu.XYA4T	0.392	430	46.636	35.731	41.531	Araip.EDH29	0.385
Aradu.XYY0T	0.373	336	52.522	42.73	41.543	Araip.TUV11	0.368
Aradu.XYY87	0.453	731	46.995	42.213	62.022	Araip.90JFZ	0.447
Aradu.XZ0HK	0.353	363	48.901	38.187	43.956	Araip.YF8MJ	0.348
Aradu.XZ1FD	0.38	305	52.614	34.641	40.196	Araip.J4WHX	0.379
Aradu.XZ2EW	0.396	371	52.688	39.785	42.473	Araip.0178A	0.392
Aradu.XZP9V	0.367	537	51.859	39.033	36.989	Araip.Q3CU1	0.369
Aradu.XZS36	0.422	463	54.741	48.276	43.75	Araip.85RP9	0.423
Aradu.Y017B	0.47	339	50.882	39.706	45.588	Araip.S0LKT	0.476
Aradu.Y0AR8	0.472	336	51.335	48.071	41.543	Araip.F8VVQ	0.469
Aradu.Y0VZV	0.364	1013	50.986	36.982	39.448	Araip.LCG1X	0.367
Aradu.Y12MD	0.355	623	50.641	35.256	39.103	Araip.ST976	0.362
Aradu.Y178G	0.387	1311	48.171	38.186	37.805	Araip.MF65Y	0.388

Aradu.Y18FU	0.352	761	54.593	41.864	38.451	Araip.IC54M	0.358
Aradu.Y1B10	0.352	662	47.813	39.065	42.685	Araip.0WL30	0.348
Aradu.Y1DM8	0.398	396	44.836	40.806	51.134	Araip.HYM8C	0.399
Aradu.Y1K8W	0.401	155	44.231	33.974	49.359	Araip.CI6Y7	0.408
Aradu.Y1KM6	0.376	301	46.026	42.053	38.742	Araip.FPC0F	0.383
Aradu.Y1LT0	0.349	741	53.504	40.162	37.062	Araip.J8BZ6	0.347
Aradu.Y1RZK	0.539	301	42.384	53.311	59.934	Araip.S4H30	0.529
Aradu.Y1TID	0.339	293	43.537	46.259	44.218	Araip.66N5R	0.342
Aradu.Y1Z0R	0.37	501	52.191	39.044	35.657	Araip.Y3AYB	0.366
Aradu.Y1Z8I	0.383	408	47.922	39.853	44.499	Araip.RR6QJ	0.363
Aradu.Y28R7	0.341	180	50.829	35.359	49.724	Araip.YA4GL	0.36
Aradu.Y2BEF	0.388	108	48.624	34.862	39.45	Araip.2L0M6	0.408
Aradu.Y2WFH	0.434	183	51.087	38.043	41.304	Araip.C7JYL	0.406
Aradu.Y2Z3L	0.341	127	44.531	37.5	28.125	Araip.T5R90	0.347
Aradu.Y2ZCM	0.377	180	43.646	32.044	35.912	Araip.H04N0	0.405
Aradu.Y31BQ	0.391	875	47.831	37.785	43.265	Araip.T1GN1	0.4
Aradu.Y328N	0.47	308	43.366	37.217	48.544	Araip.7HD9S	0.472
Aradu.Y32L7	0.361	390	54.987	36.573	36.573	Araip.9J27P	0.357
Aradu.Y37CK	0.396	621	51.608	37.299	55.305	Araip.C817Q	0.39
Aradu.Y3BW3	0.359	197	54.545	35.354	31.818	Araip.ZCG46	0.344
Aradu.Y3QBI	0.437	224	47.111	35.111	40	Araip.0W11Z	0.433
Aradu.Y3TY0	0.406	745	50.268	43.834	47.051	Araip.00HCH	0.405
Aradu.Y43HK	0.35	470	51.805	34.183	45.435	Araip.WM0YD	0.356
Aradu.Y478Z	0.39	667	50	35.329	42.515	Araip.A32IS	0.383
Aradu.Y48CE	0.481	592	55.312	44.688	70.826	Araip.14FTC	0.478
Aradu.Y4AJ0	0.327	708	44.852	34.838	35.261	Araip.DI99W	0.327
Aradu.Y4PLR	0.459	150	44.371	39.073	54.967	Araip.WA7Y0	0.445
Aradu.Y4SSQ	0.361	371	46.774	38.978	36.828	Araip.ZDV0R	0.358
Aradu.Y4VNZ	0.435	285	52.797	40.21	45.105	Araip.C0YUJ	0.435
Aradu.Y4W78	0.417	226	51.542	37.445	40.969	Araip.TD7CE	0.405
Aradu.Y54CT	0.442	230	58.874	32.468	38.961	Araip.WBT3S	0.455
Aradu.Y5B7U	0.381	288	52.595	38.408	46.367	Araip.18LXX	0.385
Aradu.Y5G1G	0.38	490	46.436	39.715	45.418	Araip.GP5MB	0.382
Aradu.Y5HX4	0.395	382	47.781	34.465	45.17	Araip.8HM3Z	0.403
Aradu.Y5MLI	0.36	1127	50	39.628	36.348	Araip.KTR75	0.369
Aradu.Y5UE5	0.361	311	40.064	34.615	32.372	Araip.237X1	0.355
Aradu.Y5WFV	0.409	398	51.128	42.105	52.381	Araip.ZUI4X	0.409
Aradu.Y5WGV	0.387	620	50.403	40.419	42.512	Araip.XL0W8	0.387
Aradu.Y5WI6	0.338	320	52.96	37.695	41.745	Araip.82B50	0.34
Aradu.Y5XYT	0.366	247	53.629	41.129	47.581	Araip.W3ZIC	0.37
Aradu.Y62C6	0.404	563	54.61	41.135	40.603	Araip.RT1VC	0.404
Aradu.Y64WA	0.422	313	53.503	45.541	34.395	Araip.TQP7Q	0.419
Aradu.Y65N7	0.424	345	52.023	36.994	37.572	Araip.A4U29	0.43
Aradu.Y66CW	0.379	510	49.902	38.552	45.988	Araip.S4R2R	0.387

Aradu.Y6F9Q	0.376	131	59.091	35.606	38.636	Araip.NBA78	0.408
Aradu.Y6FFI	0.378	1730	55.459	33.102	40.555	Araip.E0G5T	0.378
Aradu.Y6LUX	0.408	154	60.645	47.742	49.032	Araip.84L6B	0.401
Aradu.Y6TV9	0.491	365	49.727	33.88	57.377	Araip.91PPR	0.497
Aradu.Y7357	0.433	410	49.635	34.55	38.929	Araip.LGH1R	0.434
Aradu.Y7A6L	0.427	320	48.91	46.417	49.844	Araip.SR41M	0.418
Aradu.Y7AUY	0.361	1892	55.732	45.061	33.492	Araip.FPJ1M	0.367
Aradu.Y7I9D	0.292	518	47.206	38.15	41.233	Araip.KXX24	0.283
Aradu.Y7NUP	0.361	330	44.109	39.275	37.764	Araip.FD7VR	0.382
Aradu.Y7TD6	0.384	598	49.917	40.234	43.573	Araip.RMF9N	0.388
Aradu.Y82ZL	0.357	365	44.262	42.623	30.874	Araip.TMG8Z	0.356
Aradu.Y85YF	0.38	642	50.233	38.258	35.459	Araip.VS9N0	0.378
Aradu.Y8LHL	0.378	236	58.65	45.992	43.882	Araip.PR7LI	0.357
Aradu.Y8PPD	0.348	563	51.596	39.184	40.78	Araip.I4GZH	0.35
Aradu.Y8PUZ	0.453	179	48.333	42.778	39.444	Araip.BBV0C	0.477
Aradu.Y8Q46	0.429	307	46.753	33.766	48.701	Araip.A6Q3K	0.422
Aradu.Y8XMT	0.365	398	54.386	42.607	34.085	Araip.AW9H3	0.367
Aradu.Y8YHS	0.445	425	46.714	39.202	45.305	Araip.9ML2X	0.443
Aradu.Y92M0	0.476	193	66.495	57.216	45.876	Araip.S5ADV	0.471
Aradu.Y966U	0.453	263	45.455	42.045	42.424	Araip.882KX	0.447
Aradu.Y97H0	0.417	570	46.935	40.105	43.783	Araip.TJ4P5	0.417
Aradu.Y99HX	0.429	123	38.71	32.258	50	Araip.9M4V3	0.445
Aradu.Y9QAN	0.399	488	54.192	34.765	41.104	Araip.IJ811	0.404
Aradu.Y9QIK	0.356	364	47.123	39.178	40.274	Araip.TE2GJ	0.345
Aradu.Y9Y02	0.419	181	51.648	46.154	48.901	Araip.KR2LL	0.413
Aradu.YA6PI	0.386	479	56.458	54.792	39.583	Araip.GVK2U	0.39
Aradu.YA89L	0.428	206	62.802	54.589	25.604	Araip.BP2SC	0.433
Aradu.YA97R	0.334	759	48.947	41.447	37.368	Araip.F7X3N	0.335
Aradu.YAN03	0.374	524	49.905	43.619	40	Araip.J1GUG	0.376
Aradu.YAN6P	0.489	143	54.167	27.083	44.444	Araip.11R1Y	0.489
Aradu.YAX06	0.346	466	44.968	39.4	34.69	Araip.N5MMK	0.33
Aradu.YB7BD	0.436	452	55.629	39.514	45.033	Araip.7B0U4	0.433
Aradu.YBC8E	0.347	436	52.403	39.13	36.384	Araip.YKJ2E	0.345
Aradu.YC13W	0.458	406	53.808	43.735	57.248	Araip.Q2Z6R	0.46
Aradu.YC3GJ	0.41	214	42.791	35.814	46.977	Araip.GI5GJ	0.405
Aradu.YC3N7	0.466	109	52.727	45.455	48.182	Araip.YBY9J	0.466
Aradu.YC3RY	0.37	734	51.429	47.483	38.503	Araip.E2SK1	0.374
Aradu.YC6XN	0.406	450	47.45	39.69	41.685	Araip.AMY99	0.398
Aradu.YC6ZD	0.357	343	51.163	36.337	37.791	Araip.H1BC4	0.337
Aradu.YCB13	0.405	559	50.714	42.5	47.321	Araip.Q69SP	0.419
Aradu.YCI2Q	0.411	408	53.056	36.919	42.054	Araip.6H4KV	0.405
Aradu.YCJ90	0.334	552	50.09	40.868	37.794	Araip.AT55H	0.347
Aradu.YD1VK	0.326	140	51.064	36.17	34.752	Araip.PK7HW	0.348
Aradu.YD7VI	0.367	696	52.654	41.033	41.463	Araip.EC30Y	0.382

Aradu.YDH5Z	0.424	466	53.105	38.758	44.968	Araip.8ZP6J	0.437
Aradu.YDW81	0.381	1139	50.965	43.596	45.175	Araip.HTG9W	0.381
Aradu.YDZ7F	0.358	513	47.665	36.77	42.802	Araip.4EU14	0.358
Aradu.YE0CC	0.417	302	47.525	40.924	40.924	Araip.6I2P8	0.362
Aradu.YE325	0.474	410	48.662	51.825	38.2	Araip.ZX87X	0.469
Aradu.YE41G	0.318	136	44.526	38.686	35.036	Araip.G3D9B	0.321
Aradu.YE5Q3	0.334	322	50.774	37.461	32.817	Araip.K0HCY	0.331
Aradu.YE6PU	0.438	533	45.506	43.82	51.685	Araip.T8YPZ	0.438
Aradu.YEP9N	0.455	323	44.753	37.654	42.284	Araip.55652	0.453
Aradu.YF1F6	0.35	511	48.633	40.82	37.891	Araip.HV00F	0.356
Aradu.YF31D	0.394	884	52.768	42.147	32.316	Araip.24Q48	0.391
Aradu.YF3HA	0.39	368	47.154	36.043	48.238	Araip.HG4BM	0.415
Aradu.YF7CH	0.419	422	50.827	48.463	49.409	Araip.QRI3R	0.421
Aradu.YFQ3P	0.367	260	50.575	39.08	57.471	Araip.8NR3H	0.363
Aradu.YFR3R	0.448	102	54.369	44.66	57.282	Araip.6J64A	0.427
Aradu.YG3U8	0.425	321	44.099	45.031	44.72	Araip.SU78D	0.421
Aradu.YG6N5	0.359	2169	55.115	39.954	35.346	Araip.Y0FEW	0.359
Aradu.YH03P	0.441	388	50.9	38.046	47.044	Araip.C3SNG	0.44
Aradu.YH2KM	0.364	462	45.788	36.717	45.14	Araip.51VIE	0.357
Aradu.YH413	0.405	417	51.435	44.498	53.349	Araip.AZN8Q	0.406
Aradu.YH6P6	0.373	436	46.682	34.554	30.892	Araip.Y2EMI	0.37
Aradu.YH8NY	0.448	423	56.84	45.991	59.198	Araip.6KE2F	0.431
Aradu.YHD0I	0.368	148	47.651	34.899	35.57	Araip.EX3UF	0.38
Aradu.YHW10	0.353	558	45.975	35.063	38.819	Araip.JME6F	0.346
Aradu.YI13V	0.426	379	46.316	46.842	39.737	Araip.2TN3Y	0.383
Aradu.YI7L8	0.398	978	49.438	38.509	43.105	Araip.AEB43	0.4
Aradu.YIG8H	0.349	381	50.785	40.576	35.079	Araip.64EI8	0.358
Aradu.YIH2Y	0.409	271	55.882	49.632	42.279	Araip.3S8EX	0.407
Aradu.YIQ80	0.475	349	54.571	40.286	60	Araip.DL86S	0.49
Aradu.YIV0C	0.376	738	51.556	42.76	33.153	Araip.79FKI	0.379
Aradu.YJ46C	0.413	360	56.51	45.706	34.903	Araip.Z1FA8	0.388
Aradu.YJH4A	0.379	600	54.409	45.757	38.436	Araip.H38UN	0.376
Aradu.YJS53	0.347	242	47.325	36.626	55.144	Araip.YKX26	0.347
Aradu.YK06D	0.426	503	52.579	42.857	55.159	Araip.W2DXP	0.428
Aradu.YL3ZN	0.461	682	49.341	42.753	63.397	Araip.GB07R	0.461
Aradu.YLB4V	0.449	363	52.747	43.132	54.121	Araip.15K52	0.449
Aradu.YLM4Q	0.389	629	51.27	38.73	43.968	Araip.QJT7Y	0.398
Aradu.YLV00	0.412	291	42.123	40.753	40.753	Araip.X7NAU	0.405
Aradu.YM0TI	0.396	332	51.051	39.94	50.15	Araip.IA4XE	0.4
Aradu.YM44Q	0.412	155	47.436	32.051	49.359	Araip.KNZ84	0.416
Aradu.YMC7K	0.448	275	47.101	46.739	43.116	Araip.4P39C	0.436
Aradu.YMD6U	0.439	493	50.405	42.51	61.134	Araip.E00UL	0.436
Aradu.YMP6T	0.409	349	50.571	50.857	41.714	Araip.111QN	0.421
Aradu.YMP6X	0.31	529	48.679	38.113	38.302	Araip.NRD23	0.299

Aradu.YMR36	0.362	262	63.118	52.091	57.034	Araip.GK5JR	0.366
Aradu.YMT2S	0.443	154	58.065	26.452	45.806	Araip.FF9D7	0.447
Aradu.YMZ5A	0.376	564	56.991	42.478	38.407	Araip.T227J	0.378
Aradu.YN1Y7	0.41	186	47.059	40.642	46.524	Araip.LW0C3	0.406
Aradu.YN99B	0.362	215	44.444	39.815	37.963	Araip.F59A9	0.353
Aradu.YNN2E	0.46	352	53.541	48.725	64.589	Araip.YP44K	0.466
Aradu.YP0LD	0.413	489	53.673	40.612	40	Araip.0IL8D	0.416
Aradu.YP0M1	0.39	420	48.219	35.629	35.867	Araip.Y9RQ5	0.389
Aradu.YP8K9	0.541	140	55.319	40.426	61.702	Araip.E43K9	0.517
Aradu.YP97N	0.43	576	46.447	49.913	49.393	Araip.YGP1F	0.434
Aradu.YPI85	0.376	740	49.528	31.579	39.001	Araip.3M1WU	0.374
Aradu.YPK4Q	0.349	192	43.005	47.15	36.788	Araip.V8CIS	0.353
Aradu.YPS83	0.422	570	57.968	44.133	48.862	Araip.LR9VJ	0.418
Aradu.YPV42	0.425	236	54.008	44.304	45.148	Araip.6Q4X9	0.431
Aradu.YPX3B	0.367	544	47.339	39.266	42.752	Araip.ZW2KS	0.373
Aradu.YPY6M	0.36	177	53.933	43.82	34.27	Araip.G0KQK	0.347
Aradu.YQ4IR	0.322	224	48.889	28.444	35.111	Araip.B0NA7	0.327
Aradu.YQ4PR	0.426	389	50.513	41.026	48.718	Araip.K9KUX	0.424
Aradu.YQ4V9	0.326	851	53.991	38.85	37.559	Araip.H0886	0.325
Aradu.YQ9IH	0.371	1107	43.773	35.108	40.523	Araip.SSR3W	0.372
Aradu.YQ9K5	0.322	306	53.746	34.528	27.687	Araip.XM1AF	0.339
Aradu.YQ9M6	0.364	252	45.85	39.526	41.897	Araip.77H8K	0.371
Aradu.YQU8F	0.372	216	45.622	46.083	30.415	Araip.R870C	0.365
Aradu.YQW51	0.34	539	56.481	38.704	37.407	Araip.UM7BZ	0.326
Aradu.YR0IE	0.484	217	47.248	38.991	41.743	Araip.A2CHT	0.488
Aradu.YR4CG	0.364	172	46.243	46.243	38.15	Araip.X7S59	0.316
Aradu.YR762	0.428	469	48.511	46.596	41.064	Araip.A4Y4Y	0.424
Aradu.YR8RM	0.426	123	44.355	44.355	41.129	Araip.YW5U3	0.435
Aradu.YRC2R	0.367	986	50.456	41.033	35.157	Araip.R44NW	0.377
Aradu.YRE5K	0.387	820	47.503	41.048	44.458	Araip.F4XUG	0.395
Aradu.YRI3B	0.384	602	43.781	39.801	41.957	Araip.FT1CW	0.394
Aradu.YRM8T	0.399	513	46.109	36.381	42.218	Araip.G26YQ	0.389
Aradu.YRW97	0.351	873	49.085	39.931	40.618	Araip.BG5WL	0.346
Aradu.YS8K9	0.385	377	56.349	44.974	56.085	Araip.EB7GH	0.388
Aradu.YSG0U	0.455	161	53.086	35.802	50.617	Araip.93JWP	0.391
Aradu.YSN3X	0.348	658	58.574	46.586	37.026	Araip.DXU98	0.356
Aradu.YSR48	0.347	647	47.068	39.66	41.358	Araip.MEW33	0.342
Aradu.YSU0C	0.356	368	40.379	34.417	28.455	Araip.6J0KF	0.349
Aradu.YTF7E	0.472	311	51.282	44.551	65.064	Araip.FZQ92	0.457
Aradu.YTJ16	0.355	334	48.955	32.239	47.761	Araip.E9P76	0.363
Aradu.YTQ46	0.394	290	54.983	48.11	40.206	Araip.727WD	0.397
Aradu.YU6QF	0.455	338	56.932	37.758	55.457	Araip.U1GUY	0.468
Aradu.YU7JM	0.365	1101	47.731	34.936	39.111	Araip.DBL0L	0.366
Aradu.YU82F	0.387	472	57.928	41.86	37.844	Araip.0WM4W	0.391

Aradu.YUD54	0.384	490	52.546	34.012	35.438	Araip.2N3AS	0.377
Aradu.YV0Y1	0.421	336	45.994	35.608	44.214	Araip.M7KCZ	0.409
Aradu.YV75B	0.373	606	54.53	42.669	46.129	Araip.DI7GJ	0.372
Aradu.YV9QI	0.386	345	49.133	40.462	38.439	Araip.M6NPA	0.383
Aradu.YVF29	0.348	783	45.791	38.138	34.821	Araip.70K3K	0.369
Aradu.YVT9V	0.365	1779	49.213	34.101	34.551	Araip.8P06L	0.366
Aradu.YVU9U	0.396	295	58.446	38.514	48.649	Araip.MR96V	0.389
Aradu.YW23C	0.374	842	43.891	38.553	36.299	Araip.J34CB	0.378
Aradu.YW2J0	0.481	440	49.887	43.764	67.12	Araip.U23Q6	0.477
Aradu.YW2M0	0.413	478	53.445	45.929	45.929	Araip.C35A2	0.419
Aradu.YW5TG	0.433	217	49.083	40.367	53.67	Araip.C7AJI	0.44
Aradu.YWC0U	0.387	648	46.379	42.989	34.052	Araip.A1KWI	0.383
Aradu.YWE4J	0.372	227	47.807	31.579	40.351	Araip.QVM9W	0.376
Aradu.YWN5L	0.409	415	53.606	40.144	58.413	Araip.ZIC7C	0.409
Aradu.YX18B	0.437	290	51.89	42.955	45.017	Araip.S0ZCL	0.444
Aradu.YX3TI	0.478	119	50.833	46.667	55	Araip.XF6FZ	0.474
Aradu.YXG3J	0.355	366	49.046	34.06	47.139	Araip.X43U5	0.342
Aradu.YXH0F	0.365	420	47.268	36.58	38.242	Araip.0X2PV	0.367
Aradu.YXJ1Q	0.35	1248	48.679	41.313	39.472	Araip.J0VEU	0.346
Aradu.YXX50	0.436	389	52.051	39.744	48.974	Araip.MXV6P	0.428
Aradu.YXZ49	0.384	1170	51.58	40.905	41.418	Araip.95TR1	0.388
Aradu.YY4G2	0.375	684	54.599	42.336	38.248	Araip.W2Y6S	0.374
Aradu.YY51Q	0.364	342	48.105	39.359	43.149	Araip.N79SI	0.373
Aradu.YY5C4	0.357	364	48.493	42.192	44.932	Araip.ZY9Q1	0.346
Aradu.YYA59	0.391	239	53.333	41.667	50.833	Araip.RW92I	0.391
Aradu.YZ24M	0.371	437	50.457	42.237	36.986	Araip.BJ6HU	0.361
Aradu.YZ2FS	0.406	323	45.988	48.765	37.346	Araip.86DQ7	0.401
Aradu.YZ4F8	0.477	228	41.048	44.541	40.175	Araip.L4AL0	0.467
Aradu.YZ9KD	0.408	1039	45.962	40.192	51.827	Araip.X50TB	0.4
Aradu.YZA37	0.464	400	55.362	42.893	45.137	Araip.4AW6Y	0.473
Aradu.YZR0Z	0.341	512	47.173	40.741	38.012	Araip.0311L	0.351
Aradu.Z030H	0.391	601	55.482	43.355	35.382	Araip.SQ1VV	0.388
Aradu.Z06PE	0.369	308	54.045	31.068	37.217	Araip.DT1LC	0.369
Aradu.Z07JX	0.417	188	56.085	47.09	56.085	Araip.L12RT	0.408
Aradu.Z08PN	0.417	334	50.448	44.478	47.463	Araip.LY9VA	0.401
Aradu.Z0DB5	0.333	420	41.568	36.105	36.105	Araip.7L870	0.338
Aradu.Z0DJ4	0.397	259	51.538	42.308	49.231	Araip.MR96W	0.389
Aradu.Z0E8S	0.356	787	50.254	44.162	36.041	Araip.8WA63	0.361
Aradu.Z0FA2	0.355	128	45.736	43.411	41.085	Araip.Z254D	0.355
Aradu.Z0G82	0.348	732	51.16	34.652	37.653	Araip.77JRH	0.354
Aradu.Z0GNU	0.432	134	60	46.667	54.815	Araip.721GK	0.432
Aradu.Z0JID	0.348	675	51.036	40.976	41.124	Araip.9HJ5A	0.354
Aradu.Z0LGY	0.486	472	48.414	37.421	61.099	Araip.2S9Y0	0.492
Aradu.Z0X17	0.392	393	62.437	47.208	40.609	Araip.F1AX6	0.392

Aradu.Z101N	0.445	410	48.175	45.742	62.53	Araip.R2SZJ	0.454
Aradu.Z156R	0.351	575	51.042	38.021	37.153	Araip.78TK8	0.348
Aradu.Z172U	0.438	259	48.462	37.692	43.846	Araip.60WTQ	0.434
Aradu.Z17B7	0.371	659	47.273	39.545	39.697	Araip.VK9DV	0.365
Aradu.Z194X	0.392	518	54.721	37.572	49.711	Araip.A74FX	0.394
Aradu.Z197N	0.43	465	48.712	39.27	61.159	Araip.H6F7C	0.438
Aradu.Z1BP5	0.362	663	49.849	42.47	41.566	Araip.0L3VD	0.349
Aradu.Z1DBI	0.445	338	51.622	42.773	45.428	Araip.9V1A2	0.442
Aradu.Z1DQ9	0.377	339	53.529	38.529	55.882	Araip.PRI0M	0.374
Aradu.Z1H9W	0.385	186	57.754	44.92	36.898	Araip.JA6AC	0.405
Aradu.Z1N61	0.398	619	49.194	36.774	35.968	Araip.VHZ5J	0.399
Aradu.Z1QR7	0.377	325	44.479	39.877	45.092	Araip.KHB2Z	0.377
Aradu.Z1Y2A	0.386	580	51.291	43.546	39.415	Araip.2HX98	0.384
Aradu.Z2BLJ	0.481	338	51.327	38.643	60.472	Araip.8505Z	0.478
Aradu.Z2H09	0.44	191	47.917	47.396	63.542	Araip.B2B7T	0.434
Aradu.Z2HCJ	0.382	675	59.32	44.231	35.503	Araip.VC43M	0.381
Aradu.Z2J2V	0.378	384	54.805	48.312	33.247	Araip.5F3C7	0.394
Aradu.Z2SQB	0.377	154	50.323	50.323	49.032	Araip.9W150	0.37
Aradu.Z2TGH	0.402	603	47.682	40.397	45.199	Araip.LNC3W	0.398
Aradu.Z2UA0	0.4	437	53.196	35.388	44.292	Araip.QTW3F	0.4
Aradu.Z2UHU	0.36	228	45.852	37.991	54.148	Araip.M86M0	0.364
Aradu.Z31WB	0.436	150	55.629	49.007	51.656	Araip.RIF2S	0.436
Aradu.Z330P	0.369	173	50.575	28.736	31.609	Araip.E6IX0	0.365
Aradu.Z33GV	0.39	129	48.462	34.615	33.846	Araip.2NY3H	0.336
Aradu.Z34FG	0.419	841	51.781	37.886	44.537	Araip.69UD7	0.423
Aradu.Z3EIC	0.37	444	57.528	34.382	34.157	Araip.D1CR7	0.381
Aradu.Z3NVH	0.385	894	49.944	38.659	30.503	Araip.DNI71	0.39
Aradu.Z3R4T	0.419	223	50	41.071	49.554	Araip.R5PNL	0.409
Aradu.Z3UBC	0.318	180	53.039	45.856	46.961	Araip.0FU6X	0.33
Aradu.Z3YHX	0.392	505	56.917	39.13	43.874	Araip.U55ET	0.394
Aradu.Z3Z17	0.397	301	61.258	41.06	58.94	Araip.TTC98	0.394
Aradu.Z4AN4	0.39	335	42.56	39.583	38.69	Araip.M0P25	0.404
Aradu.Z4D1S	0.45	380	49.869	40.945	44.882	Araip.NG6P6	0.455
Aradu.Z4DIZ	0.345	350	48.433	37.322	41.88	Araip.TYL76	0.342
Aradu.Z4EVV	0.36	459	47.826	39.348	33.043	Araip.NBK0L	0.362
Aradu.Z4K97	0.36	493	54.656	36.842	30.972	Araip.E0NQ0	0.372
Aradu.Z4MY7	0.356	684	51.825	41.168	33.869	Araip.3Z2Y1	0.36
Aradu.Z4QII	0.386	903	52.765	40.487	38.274	Araip.HF6E7	0.387
Aradu.Z4QQP	0.343	185	45.161	34.946	45.699	Araip.RAM8P	0.309
Aradu.Z4RIW	0.395	687	53.052	38.372	39.244	Araip.4N0QC	0.39
Aradu.Z5BX3	0.507	383	50	46.354	51.823	Araip.WH7IR	0.509
Aradu.Z5EFE	0.34	219	50.455	41.364	39.091	Araip.CD6XJ	0.359
Aradu.Z5G5W	0.305	427	46.262	36.215	48.598	Araip.S3X4J	0.312
Aradu.Z5H58	0.475	335	48.512	41.964	57.143	Araip.333QY	0.469

Aradu.Z5LJB	0.368	393	52.284	40.863	51.015	Araip.VSP1L	0.368
Aradu.Z5U1L	0.308	797	46.742	36.341	30.326	Araip.K5K1N	0.328
Aradu.Z639Y	0.375	565	49.47	44.17	39.753	Araip.J3QMD	0.384
Aradu.Z65EX	0.363	576	45.754	37.435	40.208	Araip.Z6UY4	0.36
Aradu.Z6652	0.346	367	45.652	37.5	36.141	Araip.A2AJ6	0.335
Aradu.Z67WQ	0.389	508	49.902	38.114	41.847	Araip.2K8HT	0.394
Aradu.Z6I4Q	0.418	293	47.279	41.497	35.034	Araip.L8LRC	0.394
Aradu.Z6UQQ	0.415	385	45.855	41.192	46.891	Araip.IIU3H	0.422
Aradu.Z6X40	0.427	341	56.14	33.333	29.24	Araip.BWV8I	0.46
Aradu.Z6X71	0.373	346	48.991	37.464	34.294	Araip.3AQ0F	0.386
Aradu.Z6Z82	0.363	390	55.243	42.199	36.573	Araip.V7QJP	0.36
Aradu.Z702T	0.332	487	44.467	40.779	35.246	Araip.T97LZ	0.335
Aradu.Z705N	0.336	262	52.091	37.643	36.882	Araip.25CYT	0.343
Aradu.Z753H	0.441	202	47.783	40.887	41.379	Araip.7G11P	0.436
Aradu.Z7FDS	0.375	284	52.281	30.877	42.105	Araip.0TX1W	0.368
Aradu.Z7FSM	0.318	328	48.328	39.21	37.082	Araip.SCT4C	0.313
Aradu.Z7FZQ	0.428	487	50	35.246	47.951	Araip.CSX4W	0.425
Aradu.Z7K8X	0.348	473	42.405	46.624	35.865	Araip.V208D	0.348
Aradu.Z7MCS	0.441	358	57.103	40.39	62.396	Araip.T2Z8Y	0.435
Aradu.Z7SDW	0.372	312	56.23	42.812	41.214	Araip.VZ6R4	0.383
Aradu.Z7W7G	0.349	537	50.929	44.238	37.732	Araip.FC5PA	0.345
Aradu.Z7XZ9	0.345	461	50.216	33.333	56.926	Araip.KS6V8	0.345
Aradu.Z839U	0.381	547	49.453	39.599	38.686	Araip.0BN4Y	0.383
Aradu.Z83RP	0.407	591	53.209	39.189	41.723	Araip.Y7AG2	0.411
Aradu.Z84GQ	0.354	527	53.409	41.288	31.25	Araip.X0SWX	0.358
Aradu.Z84L6	0.387	255	48.047	53.516	35.938	Araip.UQT8V	0.39
Aradu.Z84W9	0.398	494	51.717	34.545	39.596	Araip.L2LE7	0.382
Aradu.Z8A7E	0.359	962	50.987	34.683	38.422	Araip.73UW5	0.362
Aradu.Z8G1Y	0.355	964	51.813	37.927	33.264	Araip.F4PJ9	0.352
Aradu.Z8HLS	0.398	919	55.217	42.174	39.674	Araip.09BNR	0.401
Aradu.Z8JSI	0.424	176	56.497	45.198	49.718	Araip.WF9GZ	0.424
Aradu.Z8K87	0.472	459	55	42.391	70.217	Araip.YN5F9	0.475
Aradu.Z8VFD	0.382	531	50.94	39.098	36.278	Araip.8R4SA	0.379
Aradu.Z8VLV	0.409	794	54.717	43.27	47.044	Araip.216VR	0.408
Aradu.Z8W63	0.315	531	38.158	33.459	39.474	Araip.1F0E9	0.326
Aradu.Z8XIW	0.325	432	51.732	43.418	31.64	Araip.KCK8F	0.337
Aradu.Z922D	0.424	435	45.183	41.972	38.303	Araip.02EM5	0.422
Aradu.Z93ZE	0.43	166	53.892	43.713	42.515	Araip.NVE0S	0.424
Aradu.Z9DZQ	0.33	363	50.824	33.242	30.22	Araip.68F0J	0.324
Aradu.Z9J76	0.338	291	50.685	28.767	34.247	Araip.PH9P5	0.345
Aradu.Z9KCW	0.37	1037	55.299	42.389	47.399	Araip.UF61R	0.372
Aradu.Z9WRN	0.416	455	44.298	40.789	45.833	Araip.9D138	0.414
Aradu.Z9Y3J	0.38	330	42.296	37.462	42.9	Araip.AVV74	0.396
Aradu.Z9YF8	0.372	636	52.433	40.345	36.421	Araip.89AKE	0.366

Aradu.ZA2RQ	0.464	473	49.578	39.451	69.198	Araip.A2PE6	0.468
Aradu.ZA91W	0.42	215	53.241	46.759	52.315	Araip.ING83	0.415
Aradu.ZAA79	0.394	740	49.123	37.922	44.669	Araip.Y8BE0	0.381
Aradu.ZAF9Y	0.366	1403	55.057	51.353	30.271	Araip.DC4BQ	0.371
Aradu.ZAK9S	0.439	276	58.484	38.989	37.545	Araip.B8RZP	0.435
Aradu.ZAY9P	0.317	129	46.923	40	41.538	Araip.Y23N0	0.302
Aradu.ZB2RS	0.387	778	48.395	42.49	46.598	Araip.5ZK6T	0.387
Aradu.ZBZ36	0.535	536	51.955	46.741	69.832	Araip.Z30L7	0.537
Aradu.ZC0SW	0.459	489	48.367	41.429	47.755	Araip.QVW26	0.458
Aradu.ZC5IW	0.356	411	53.883	40.049	35.437	Araip.Q3F5T	0.348
Aradu.ZC6SM	0.406	778	52.888	35.045	37.484	Araip.EQA46	0.403
Aradu.ZC9DU	0.372	432	46.189	39.723	37.413	Araip.C0Q51	0.375
Aradu.ZCP8X	0.391	659	50.152	36.667	38.788	Araip.Q00WT	0.405
Aradu.ZD05E	0.395	132	54.887	38.346	40.602	Araip.E0UG7	0.395
Aradu.ZD4TK	0.427	368	49.864	42.005	46.341	Araip.B24DH	0.424
Aradu.ZD4XA	0.363	444	50.337	44.494	34.157	Araip.XJC3R	0.382
Aradu.ZD5DD	0.347	419	43.571	34.524	54.524	Araip.ST1UP	0.34
Aradu.ZD7QJ	0.307	346	53.314	36.599	39.193	Araip.T0P1U	0.324
Aradu.ZDD46	0.365	802	54.67	39.228	32.13	Araip.Z9JDD	0.369
Aradu.ZDZ6K	0.399	215	51.852	39.815	55.093	Araip.97H71	0.426
Aradu.ZE30N	0.341	218	52.511	28.767	45.662	Araip.69U4D	0.34
Aradu.ZE3IA	0.382	478	56.994	41.336	35.699	Araip.02P6R	0.374
Aradu.ZEB3I	0.453	410	47.689	44.282	59.367	Araip.P77AZ	0.448
Aradu.ZEU97	0.534	494	49.697	43.232	67.475	Araip.PH1BR	0.543
Aradu.ZF1LK	0.395	404	54.074	51.852	46.914	Araip.7PD4P	0.398
Aradu.ZF31Q	0.364	443	51.126	38.514	40.991	Araip.N3W3L	0.368
Aradu.ZF53H	0.383	620	52.174	40.419	43.8	Araip.7C4C2	0.371
Aradu.ZF8NC	0.376	579	44.655	37.069	41.724	Araip.M9SP4	0.374
Aradu.ZFA07	0.364	553	53.43	42.058	40.614	Araip.6I56J	0.366
Aradu.ZFB7W	0.391	497	48.795	38.755	38.554	Araip.02MA2	0.401
Aradu.ZFF1D	0.408	200	56.716	43.284	35.821	Araip.2FX35	0.413
Aradu.ZFI13	0.401	366	49.319	39.51	40.054	Araip.B1TJC	0.392
Aradu.ZFT0U	0.363	593	51.347	36.869	45.455	Araip.JGS8U	0.369
Aradu.ZFW51	0.379	627	51.911	45.382	37.102	Araip.VS416	0.39
Aradu.ZFX80	0.319	864	50.405	33.064	32.832	Araip.7MD3E	0.323
Aradu.ZG4JR	0.397	470	49.894	43.1	49.045	Araip.P10LD	0.395
Aradu.ZG6C0	0.406	423	52.83	45.047	47.17	Araip.SBA9Y	0.409
Aradu.ZG8VV	0.335	283	46.831	47.535	37.324	Araip.SKR9D	0.333
Aradu.ZGD79	0.408	753	54.377	41.114	36.34	Araip.QKJ0T	0.408
Aradu.ZH4KD	0.348	808	48.949	34.611	42.151	Araip.L0F41	0.346
Aradu.ZHM8Y	0.361	370	50.404	38.814	40.97	Araip.M02FS	0.369
Aradu.ZI2XT	0.369	565	48.763	41.166	37.279	Araip.R0C6X	0.38
Aradu.ZI52D	0.401	726	57.497	42.916	48.143	Araip.K461S	0.401
Aradu.ZI9AT	0.336	114	57.391	46.087	41.739	Araip.0S8MN	0.339

Aradu.ZIC37	0.367	877	53.189	38.041	37.927	Araip.G1V85	0.363
Aradu.ZIF6R	0.373	490	51.731	37.882	42.159	Araip.7EF18	0.367
Aradu.ZIW9T	0.383	255	48.828	38.281	37.5	Araip.XN9LA	0.373
Aradu.ZJ042	0.376	462	52.916	34.989	36.069	Araip.5NU5L	0.388
Aradu.ZJ0E3	0.306	581	42.096	35.223	34.021	Araip.P8TR0	0.309
Aradu.ZJ194	0.406	211	46.698	41.038	38.679	Araip.73VEI	0.415
Aradu.ZJ4KX	0.397	460	48.59	43.601	39.262	Araip.T7TLE	0.397
Aradu.ZJ7LY	0.377	709	45.634	40.563	43.662	Araip.A70P4	0.365
Aradu.ZJM47	0.388	547	48.905	49.818	32.117	Araip.DDX3A	0.387
Aradu.ZJM8H	0.353	345	52.023	37.572	37.572	Araip.RV7VH	0.365
Aradu.ZJT5U	0.387	682	51.684	45.681	40.849	Araip.E26HH	0.399
Aradu.ZK0JG	0.387	465	58.584	40.558	52.79	Araip.J63ZS	0.387
Aradu.ZK7ZU	0.503	161	54.321	41.975	45.062	Araip.U324B	0.471
Aradu.ZL56D	0.423	197	58.586	38.384	30.808	Araip.ZRU67	0.418
Aradu.ZL6EF	0.408	376	49.337	39.523	44.297	Araip.R86PR	0.403
Aradu.ZL9WR	0.394	235	48.729	34.746	50.847	Araip.R3S6C	0.403
Aradu.ZLR3W	0.345	1403	43.305	34.33	36.325	Araip.H1VEE	0.334
Aradu.ZM4HW	0.358	615	51.299	44.805	36.851	Araip.QLL2I	0.363
Aradu.ZM5JL	0.387	367	50.272	43.75	38.043	Araip.IH5NI	0.39
Aradu.ZM6VG	0.445	233	45.726	46.154	45.726	Araip.JY10U	0.446
Aradu.ZM868	0.379	581	51.718	41.409	32.99	Araip.H8E01	0.382
Aradu.ZM93S	0.447	408	50.856	44.743	53.545	Araip.14NBL	0.431
Aradu.ZM9CR	0.357	213	48.131	29.907	43.458	Araip.PW5ES	0.353
Aradu.ZMN8D	0.38	873	51.03	37.529	44.622	Araip.ZSM5V	0.378
Aradu.ZN3FN	0.313	352	49.292	32.861	37.394	Araip.I128H	0.314
Aradu.ZN5ZV	0.427	521	58.238	48.084	57.28	Araip.NX2DL	0.435
Aradu.ZN7FN	0.315	398	39.348	36.842	37.093	Araip.87I9S	0.327
Aradu.ZN9Y3	0.377	292	51.877	37.201	47.44	Araip.YRI89	0.399
Aradu.ZNZ7Q	0.414	632	56.24	50.869	34.755	Araip.UIM6D	0.414
Aradu.ZP2GZ	0.389	241	53.719	38.017	43.802	Araip.W54L5	0.412
Aradu.ZP2LZ	0.412	509	51.961	43.529	58.824	Araip.A9CVZ	0.422
Aradu.ZP3W0	0.354	498	46.894	42.285	37.675	Araip.6DK8B	0.356
Aradu.ZP7JG	0.373	155	51.282	51.282	55.769	Araip.4R6C8	0.373
Aradu.ZPB6A	0.34	224	56.444	51.111	45.333	Araip.9A6FH	0.324
Aradu.ZPL5X	0.372	1039	55.673	39.423	40.962	Araip.S5QSK	0.363
Aradu.ZPP4Z	0.375	224	49.778	32	43.556	Araip.5HK6K	0.37
Aradu.ZQ118	0.391	977	49.489	41.82	44.172	Araip.CU4A2	0.396
Aradu.ZQ29G	0.34	102	56.311	42.718	31.068	Araip.45HI9	0.37
Aradu.ZQK52	0.373	891	52.691	43.049	35.09	Araip.31UA1	0.368
Aradu.ZR16S	0.359	922	49.512	39.978	35.428	Araip.E472Z	0.354
Aradu.ZR4H7	0.37	1456	46.397	38.984	38.504	Araip.IAU9S	0.374
Aradu.ZR5CT	0.351	463	50.431	41.81	38.578	Araip.RZ98L	0.356
Aradu.ZR64Z	0.42	440	49.66	35.147	45.805	Araip.TWR85	0.42
Aradu.ZR9ZC	0.425	328	53.495	48.328	42.249	Araip.RV92P	0.422

Aradu.ZRM2P	0.378	957	50.731	37.161	38.205	Araip.WH0MC	0.377
Aradu.ZS0PF	0.357	1048	44.042	37.846	40.61	Araip.Z17SR	0.354
Aradu.ZS4C4	0.391	750	47.537	37.417	43.542	Araip.4YR0R	0.39
Aradu.ZS4VI	0.39	238	51.464	46.444	38.494	Araip.L7KTT	0.383
Aradu.ZS6PI	0.402	371	51.613	45.699	48.925	Araip.RA8N4	0.386
Aradu.ZS8UE	0.353	438	51.936	38.497	34.169	Araip.PD45N	0.353
Aradu.ZS9DU	0.451	257	55.039	58.527	56.202	Araip.87K0L	0.452
Aradu.ZSR47	0.36	939	50.106	33.511	41.915	Araip.C4YLK	0.362
Aradu.ZSZ74	0.384	346	55.908	43.228	39.769	Araip.D92TL	0.39
Aradu.ZT0IB	0.358	381	48.953	43.979	38.482	Araip.81IM1	0.362
Aradu.ZT2KF	0.325	462	49.028	39.093	35.205	Araip.YU18D	0.329
Aradu.ZT2TE	0.378	341	48.246	37.135	32.164	Araip.XQA0A	0.389
Aradu.ZT956	0.424	295	45.27	37.838	35.135	Araip.IMD5Y	0.426
Aradu.ZTH8V	0.436	193	44.845	37.113	45.876	Araip.37TY6	0.441
Aradu.ZTH93	0.418	378	47.23	35.356	43.799	Araip.ZU8P9	0.416
Aradu.ZTY0U	0.406	347	46.839	43.103	43.966	Araip.1T581	0.409
Aradu.ZU0H3	0.431	275	51.087	45.29	44.203	Araip.0P39S	0.408
Aradu.ZU8HW	0.386	1214	51.358	41.564	34.321	Araip.L0LZX	0.384
Aradu.ZUG9I	0.415	375	53.191	38.83	41.223	Araip.UD1ML	0.412
Aradu.ZUH0W	0.429	640	52.262	44.306	57.41	Araip.YU5MB	0.435
Aradu.ZUQ2N	0.378	546	50.823	35.832	38.208	Araip.Y1D91	0.367
Aradu.ZUY8A	0.433	289	52.069	33.793	39.655	Araip.SI9K6	0.443
Aradu.ZV3G2	0.4	653	48.93	39.45	40.979	Araip.Z6KJI	0.402
Aradu.ZV73M	0.403	1353	53.028	38.405	46.972	Araip.PJ399	0.405
Aradu.ZV8PI	0.4	317	43.396	44.654	38.994	Araip.U6RZ5	0.393
Aradu.ZV9WQ	0.371	959	53.646	40.729	45.417	Araip.F5V5K	0.365
Aradu.ZVP8C	0.404	323	46.914	41.358	37.037	Araip.ZK7F1	0.4
Aradu.ZVZ4G	0.402	338	52.802	35.988	38.053	Araip.2WN0Z	0.386
Aradu.ZW165	0.345	886	52.762	37.43	35.626	Araip.P2X4L	0.351
Aradu.ZW38I	0.463	216	44.7	38.71	59.447	Araip.YR061	0.478
Aradu.ZW5X6	0.394	200	50.249	31.841	44.776	Araip.83CVJ	0.402
Aradu.ZWA6F	0.331	521	43.678	35.057	32.95	Araip.642G1	0.341
Aradu.ZWN25	0.365	527	48.674	39.015	46.78	Araip.536TB	0.369
Aradu.ZX42L	0.351	872	56.128	46.392	32.99	Araip.GC2RS	0.349
Aradu.ZX52Y	0.424	274	54.545	45.455	49.091	Araip.US2FW	0.417
Aradu.ZX8II	0.413	400	56.359	44.389	42.394	Araip.LUP74	0.421
Aradu.ZX8WQ	0.387	354	43.662	38.028	40	Araip.1Y3MU	0.396
Aradu.ZY1BM	0.35	549	50.909	45.091	36	Araip.M8CB8	0.358
Aradu.ZY2NN	0.376	200	44.279	40.299	51.741	Araip.ZF9TA	0.393
Aradu.ZY590	0.389	780	51.088	37.772	46.479	Araip.NP2NQ	0.399
Aradu.ZY67R	0.326	371	42.742	42.473	34.677	Araip.11CVG	0.333
Aradu.ZY82G	0.346	345	46.243	44.22	40.751	Araip.XU3BG	0.344
Aradu.ZYE2B	0.396	734	49.66	41.224	40.952	Araip.MI9T9	0.389
Aradu.ZYI0Y	0.35	124	40.8	44.8	43.2	Araip.NMI4A	0.35

Aradu.ZYI4E	0.37	1326	47.777	42.05	32.178	Araip.ZD7CP	0.369
Aradu.ZYN4B	0.376	512	50.097	44.25	40.546	Araip.U9Z5C	0.366
Aradu.ZYW5P	0.387	514	48.35	43.495	44.078	Araip.N95NQ	0.37
Aradu.ZZ215	0.364	496	53.722	33.803	44.869	Araip.79R74	0.371
Aradu.ZZ3IQ	0.463	366	50.681	46.866	55.586	Araip.9Q46C	0.455
Aradu.ZZ3JW	0.317	383	51.823	35.156	35.938	Araip.LWJ5V	0.308
Aradu.ZZ9EE	0.403	629	43.492	35.873	39.683	Araip.E291T	0.394
Aradu.ZZD2I	0.366	296	51.178	38.047	40.067	Araip.HL73L	0.363
Aradu.ZZU4A	0.383	578	53.195	40.76	48.359	Araip.XR2D0	0.385

Table S3 Gene expression level of 22 different tissues in *Arachis duranensis* and *Arachis ipaënsis* one-to-one orthologs

Arachis duranensis	Seedling_Leaves	MainStem_Leaves	LateralStem_Leaves	VegetativeShootTip	ReproductiveShootTip
Aradu.R7JCU	-2.83650127	-3.05889369	-4.32192809	2.526068812	2.498250868
Aradu.U564S	-0.73696559	0.070389328	-0.86249648	0.713695815	0.855989697
Aradu.07V9Z	2.794935663	3.168321116	2.857980995	4.009884589	4.445594291
Aradu.Y3TY0	NA	-5.05889369	-5.64385619	-4.64385619	-4.32192809
Aradu.81LXC	3.065227623	4.464014725	4.475084883	1.232660757	2.914564523
Aradu.IFI3Q	2.211012193	1.981852653	1.883620816	1.448900951	0.925999419
Aradu.BBP4Z	1.831877241	2.247927513	1.937344392	0.411426246	2.025028794
Aradu.T7MUN	-2.64385619	-1.64385619	-3.47393119	-0.59946207	-0.59946207
Aradu.2F2F8	NA	NA	NA	NA	NA
Aradu.IMH3N	2.40599236	2.843983844	2.780310099	3.424922088	3.407352751
Aradu.1Y660	2.901108243	3.29130886	3.132576843	3.744161096	4.019701914
Aradu.XZ2EW	3.892391026	4.292781749	4.138323004	4.904965719	4.978653265
Aradu.0R9W1	3.300123725	3.416839742	3.102658131	2.386810946	3.799087306
Aradu.N3085	NA	NA	NA	NA	-5.64385619

Aradu.W0ILV	0.941106311	1.304511042	1.232660757	2.269033146	2.592158002
Aradu.FZ0BW	3.150559677	2.94673086	2.992768431	2.575312331	2.788685711
Aradu.F8VUJ	2.313245852	2.214124805	2.198494154	2.207892852	2.459431619
Aradu.TY7A4	-4.05889369	-5.64385619	NA	NA	-2.25153877
Aradu.QKQ8N	2.698218478	2.891419187	2.827819025	2.641546029	3.155425432
Aradu.AVN2W	2.150559677	1.967168608	1.925999419	3.382667253	3.49441561
Aradu.JQ8WR	1.510961919	1.718087584	2.166715445	1.871843649	3.493134922
Aradu.C3FIF	-2.94341647	-2.94341647	-3.47393119	-0.73696559	0.555816155
Aradu.Z922D	1.438292852	0.773996325	1.327687364	-3.05889369	-1.94341647
Aradu.VK1JK	0.150559677	0.895302621	0.443606651	1.298658316	1.13093087
Aradu.R3QFW	1.910732662	1.948600847	1.843983844	2.189033824	2.498250868
Aradu.ZFB7W	0.189033824	0.321928095	0.201633861	0.485426827	0.124328135
Aradu.452B2	2.889473543	3.521050737	3.23572706	6.031659854	6.377123749
Aradu.ZE3IA	3.106013238	2.841973119	2.970853654	2.500802053	2.819668183
Aradu.J4JB7	-3.47393119	-4.32192809	-5.05889369	-1.78587519	2.077242999
Aradu.RF66L	0.097610797	0.422233001	0.454175893	1.906890596	2.010779839
Aradu.YZR0Z	-6.64385619	NA	NA	-5.64385619	1.565597176
Aradu.W4I1P	1.10433666	2.077242999	1.124328135	2.805292456	2.750606505
Aradu.TP72Z	-0.13606155	-0.15200309	0	0.713695815	0.669026766
Aradu.0QF6H	1.790772038	2.063502942	1.704871964	2.310340121	2.321928095
Aradu.N9R9J	NA	NA	NA	NA	0.50589093
Aradu.KX0SA	3.393690764	3.673556424	3.203201156	3.454175893	3.648465443

Aradu.74N2Q	-0.83650127	0.695993813	0.739848103	2.166715445	2.555816155
Aradu.577TS	1.815575429	2.22650853	1.731183242	1.550900665	1.871843649
Aradu.V6QFH	3.166715445	3.63691458	3.566815154	3.953265239	4.152994605
Aradu.R4JE4	2.627606838	2.881664619	2.835924074	2.669026766	3.114367025
Aradu.M6THB	-2.94341647	-1.02914635	-2	-1.78587519	1.803227036
Aradu.40078	0.575312331	0.847996907	0.464668267	2.411426246	2.596935142
Aradu.A6WCN	NA	NA	-6.64385619	-3.83650127	NA
Aradu.7WJ9D	3.56315813	4.093391153	4.250203656	3.56924803	3.750606505
Aradu.40VD3	0.22650853	0.98550043	0.622930351	1	1.310340121
Aradu.C4M03	3.62058641	4.06091205	3.892391026	4.742545234	5.082787732
Aradu.M414E	0.613531653	1.327687364	1.049630768	4.374343989	4.488000771
Aradu.E6T6V	NA	NA	NA	NA	NA
Aradu.G4VEH	4.308885057	4.549669171	4.550900665	4.985044962	5.099715999
Aradu.R4JGP	1.981852653	2.124328135	2.060047384	2.550900665	3.173127433
Aradu.3DV9B	1.321928095	1.438292852	1.042644337	2.09423607	2.543495883
Aradu.D1N5P	NA	NA	-6.64385619	-5.64385619	-2.05889369
Aradu.1M9NC	0.956056652	1.117695043	1.042644337	1.028569152	1.339137385
Aradu.WHH4F	1.627606838	2.080657663	1.963474124	1.996388746	2.448900951
Aradu.J3LFY	NA	-6.64385619	NA	NA	-5.05889369
Aradu.WLZ4Q	NA	-6.64385619	-5.05889369	NA	-5.64385619
Aradu.9P432	2.09085343	3.111031312	3.912649865	0.367371066	1.819668183
Aradu.VQ1N9	0.941106311	1.10433666	0.807354922	2.153805336	2.724650272

Aradu.771JD	NA	NA	NA	-4.32192809	-2
Aradu.AH9TD	1.851998837	2.124328135	1.944858446	2.59454855	2.748461233
Aradu.PM5NX	-2.05889369	-1.73696559	-2.73696559	1.257010618	1.580145484
Aradu.A68AJ	-1.18442457	0.097610797	-0.68965988	1.937344392	2.310340121
Aradu.N7VLT	2.707082992	2.967168608	2.811471031	4.420886575	4.059182199
Aradu.GDA41	4.075532631	3.844988157	4.085764554	2.073820233	2.09423607
Aradu.H0XA8	NA	NA	NA	NA	NA
Aradu.PXP20	-0.49410907	0.163498732	-0.04394335	1.40599236	2.358958826
Aradu.JM1K2	2.56315813	2.735522177	2.66448284	3.968090752	3.795974694
Aradu.6N11A	1.599317794	1.967168608	1.580145484	2.98550043	3.13422094
Aradu.XVQ80	-4.32192809	-2.55639335	-1.51457317	5.778208576	4.82374936
Aradu.30RPU	-5.64385619	-4.64385619	-4.05889369	-1.51457317	-1.21759144
Aradu.TTI87	0.516015147	1.731183242	1.10433666	1.531069493	0.748461233
Aradu.34ACD	-0.577767	0.176322773	-0.62148838	1.815575429	1.778208576
Aradu.MAS03	2.40053793	4.7322692	3.781359714	6.008988783	5.650764559
Aradu.C4BMG	-3.05889369	-2.12029423	-2.47393119	-1	-0.35845397
Aradu.W2RXK	5.424586226	5.84899843	5.915042791	4.867402306	4.890933022
Aradu.VDX8A	3.026800059	2.316145742	2.680324357	-0.18442457	0.622930351
Aradu.MQ4WG	0.687060688	0.790772038	-0.01449957	3.890446693	4.13996057
Aradu.VW26U	0.695993813	1.427606173	1.220329955	2.606442228	2.646162657
Aradu.P24XG	0.137503524	-0.07400058	0.137503524	-3.64385619	1.495695163
Aradu.Z8HLS	3.247927513	3.663344619	3.555816155	4.049630768	4.558879853

Aradu.U5A8Y	5.132165525	5.0721058	4.762880293	1.526068812	3.166715445
Aradu.7AP79	NA	-6.64385619	NA	NA	NA
Aradu.I4YNH	2.014355293	2.582556003	2.6622055	1.263034406	2.13093087
Aradu.P8YMU	0.722466024	1.66448284	0.189033824	2.321928095	2.604071324
Aradu.58KSZ	2.427606173	3.078951341	2.584962501	3.532316959	3.609991295
Aradu.G2EQN	1.195347598	1.201633861	1.411426246	2.650764559	2.871843649
Aradu.U8FLG	0.084064265	0.495695163	0.275007047	0.790772038	1.464668267
Aradu.092KC	NA	-4.05889369	-6.64385619	NA	-5.64385619
Aradu.IG70C	0.704871964	1.713695815	1.584962501	0.687060688	-0.16812276
Aradu.J1ZY0	-2.32192809	-1.25153877	-1.55639335	0.815575429	0.214124805
Aradu.LJV84	NA	NA	NA	-0.34007544	-0.07400058
Aradu.G4DWJ	1.220329955	1.871843649	1.443606651	3.13422094	3.22650853
Aradu.K8V1Y	-1.39592868	-0.577767	-1.18442457	-2.83650127	-0.83650127
Aradu.ST3KE	-3.18442457	-2.55639335	-3.32192809	0.286881148	0.344828497
Aradu.6Y1H7	1.700439718	1.263034406	1.244887059	-0.02914635	0.163498732
Aradu.4I70C	2.295723025	2.803227036	2.66448284	1.35614381	1.914564523
Aradu.II4Y3	4.46727948	3.750606505	3.50589093	1.632268215	1.459431619
Aradu.WUW36	1.807354922	1.541019153	1.464668267	-0.18442457	0.82374936
Aradu.UIA4U	-0.18442457	0.871843649	1.819668183	0.757023247	0.321928095
Aradu.SHC8B	2.430285273	2.526068812	2.414135533	2.957914599	3.314696526
Aradu.E515S	4.956521363	5.615004242	4.777682717	6.92919922	6.866537121
Aradu.73PA3	3.930737338	5.11353382	5.325530332	4.634012356	4.527320608

Aradu.V72BH	4.754887502	5.168321116	4.85847839	6.749802402	6.809543067
Aradu.Z839U	3.240314329	2.95419631	2.950468414	2.580145484	2.469885976
Aradu.H1BIK	NA	NA	NA	NA	NA
Aradu.8G81K	6.407692649	6.673415087	6.512542955	6.399854674	7.303963356
Aradu.4DJ5M	-2	-1.78587519	-1.78587519	-0.34007544	-0.4150375
Aradu.LAK4N	2.735522177	3.828834649	3.060047384	4.426264755	4.313245852
Aradu.4YZ2K	3.738767837	3.455491621	4.08236197	1.887525271	1.659924558
Aradu.RP1BS	3.839959587	4.309612772	3.629939409	5.622637567	5.600804955
Aradu.XK367	1.416839742	2.056583528	1.847996907	0.807354922	0.933572638
Aradu.SG5SM	NA	NA	NA	NA	NA
Aradu.5UD3Q	NA	NA	-6.64385619	NA	NA
Aradu.8GW0S	-4.05889369	-2.18442457	NA	2.039138394	2.40053793
Aradu.K3XG1	-1.47393119	-1.83650127	-2.05889369	0.111031312	0.150559677
Aradu.LX2KK	2.570462931	2.821710215	2.472487771	3.09592442	3.545968369
Aradu.03ENG	9.107792469	9.197953828	9.162919968	6.238213641	6.931801228
Aradu.NQ8H0	3.439623138	3.495695163	3.35473424	3.671293372	3.980939266
Aradu.NSE79	-5.64385619	NA	-5.64385619	-5.05889369	-3.47393119
Aradu.KFD3G	-1.83650127	-2.25153877	-2.47393119	-3.05889369	-2.12029423
Aradu.HGJ4G	1.580145484	1.704871964	1.438292852	2.518535139	2.627606838
Aradu.IM5S3	-0.577767	0.669026766	-0.12029423	1.655351829	1.5360529
Aradu.Z3UBC	-2.64385619	-2.39592868	-1.88896869	-3.18442457	-1.68965988
Aradu.C2N0T	5.778734244	5.280213996	5.556122818	0.5360529	0.389566812
Aradu.2RV2	2.375734539	2.687060688	2.910732662	1.111031312	2.176322773

9					
Aradu.NQC4B	-4.64385619	-4.05889369	-3.83650127	-1.83650127	-1.51457317
Aradu.D4F55	NA	NA	NA	NA	-6.64385619
Aradu.76Y0H	2.639232163	3.319039816	2.895302621	3.765534746	3.914564523
Aradu.792L3	-5.64385619	-4.05889369	-5.64385619	-4.32192809	-2.83650127
Aradu.45U0D	-0.64385619	-0.59946207	-0.91593574	-1.21759144	-1.02914635
Aradu.V220X	NA	NA	NA	NA	NA
Aradu.HL03T	NA	NA	NA	NA	NA
Aradu.LRS2W	1.526068812	2.23572706	2.087462841	2.173127433	2.23878686
Aradu.BK9AL	2.44625623	2.599317794	2.40053793	1.526068812	1.372952098
Aradu.F9L0R	-1.83650127	-2.55639335	-1.88896869	-3.83650127	-2.25153877
Aradu.YP0LD	0	0.275007047	0.50589093	0.575312331	0.669026766
Aradu.5V20C	1.333423734	1.655351829	1.250961574	1.875780063	1.695993813
Aradu.0M33I	0.545968369	0.918386234	0.604071324	1.344828497	1.448900951
Aradu.2F99R	0.941106311	1.056583528	0.124328135	1.613531653	2.432959407
Aradu.90B63	-4.05889369	-3.05889369	-6.64385619	-3.05889369	-1.94341647
Aradu.2T85U	0.695993813	1.214124805	1.13093087	1.599317794	1.739848103
Aradu.SE8E9	NA	NA	NA	NA	-4.05889369
Aradu.AF9V9	6.051807107	6.165510018	5.923386916	6.423241996	6.413458688
Aradu.STU80	1.599317794	1.713695815	1.786596362	1.944858446	2.266036894
Aradu.MP2DM	2.007195501	2.627606838	2.508428653	2.066950244	1.959770155
Aradu.JZR6I	4.532940288	4.705425039	4.399171094	4.900625333	5.613531653
Aradu.Z1BP5	2.700439718	4.225737653	3.818646083	2.462052319	3.368768349

Aradu.5RF5F	6.907131025	7.019257119	7.167418146	7.008764745	6.842224613
Aradu.78G42	NA	-3.83650127	-4.64385619	-4.32192809	-5.64385619
Aradu.HE8A8	3.310340121	4.043519494	4.012568674	4.148934105	4.009884589
Aradu.N8VJ2	3.420886575	3.132576843	3.229587923	2.169925001	2.510961919
Aradu.3L41J	2.632268215	2.521050737	2.568032105	0.176322773	0.411426246
Aradu.WYZ5E	NA	NA	NA	NA	NA
Aradu.W01YL	-3.32192809	-3.64385619	-3.83650127	-0.73696559	-0.55639335
Aradu.1RK1Y	-0.49410907	0.50589093	-0.39592868	1.23878686	1.14404637
Aradu.0Z8XN	-3.47393119	0.124328135	-0.68965988	2.176322773	1.50589093
Aradu.54J14	4.116031993	4.554588852	4.343407822	5.126807703	5.480265122
Aradu.ECK51	5.22881869	5.079804755	5.154210531	4.48864354	4.323370069
Aradu.4MN5D	1.757023247	2.204766751	1.794935663	2.510961919	2.528571319
Aradu.P432A	1.867896464	2.060047384	1.90303827	1.799087306	2.195347598
Aradu.ZU0H3	1.925999419	1.646162657	1.526068812	0.757023247	1.111031312
Aradu.RE1JT	NA	NA	NA	-2.94341647	-3.47393119
Aradu.TCQ2L	NA	-4.64385619	-6.64385619	-1.47393119	0.807354922
Aradu.I03LR	2.622930351	3.047887329	2.646162657	4.925049965	4.642123916
Aradu.KIS5R	NA	-4.64385619	NA	2.361768359	4.080657663
Aradu.SQ2QT	NA	NA	NA	NA	0.422233001
Aradu.61ES3	NA	NA	NA	-5.64385619	NA
Aradu.3L1P7	1.250961574	1.799087306	1.389566812	1.298658316	1.560714954
Aradu.D7B0D	2.275007047	2.153805336	2.039138394	1.790772038	1.782408565

Aradu.Q2AQJ	2.028569152	1.541019153	1.659924558	-0.39592868	0.704871964
Aradu.4A7CI	3.390942773	3.264536431	2.889473543	4.021479727	4.72628629
Aradu.65NZB	5.100136671	4.928844037	5.030777472	2.204766751	2.066950244
Aradu.M0Q97	NA	NA	NA	NA	NA
Aradu.DH5V9	3.465974465	1.895302621	0.956056652	-0.88896869	-0.88896869
Aradu.ZI9AT	2.821710215	2.891419187	3	3.229587923	3.522306893
Aradu.6Z34Q	0.23878686	0.555816155	1.10433666	3.241840184	2.523561956
Aradu.M206V	2.989139007	2.835924074	2.482848283	1.959770155	1.794935663
Aradu.S0HMQ	1.49057013	2.673556424	2.510961919	3.854993017	3.783456654
Aradu.DI7YA	1.82374936	2.518535139	1.996388746	3.333423734	3.54225805
Aradu.Z7FDS	3.182692298	3.641546029	4.250961574	3.480265122	2.596935142
Aradu.IZ2A8	-0.20091269	0.056583528	-0.28630419	-2.12029423	-0.62148838
Aradu.IR1JZ	-2.64385619	-1.94341647	-2.55639335	1.316145742	2.032100843
Aradu.H5NQ6	5.862203399	5.997969782	5.518535139	6.11914864	6.109987319
Aradu.26N4W	5.093391153	4.952333566	5.287620044	2.3305584	3.12763328
Aradu.WJ6G7	4.521050737	4.844988157	4.484138131	5.103077947	5.309612772
Aradu.7XA36	1.929790998	1.963474124	1.967168608	2.070389328	2.44625623
Aradu.P98IW	-6.64385619	-6.64385619	-5.64385619	-6.64385619	-4.64385619
Aradu.XPZ1I	9.521168548	9.067515031	9.281466436	6.110196178	6.146288637
Aradu.7JU4H	3.66106548	3.905928478	3.683696454	3.66106548	3.897240426
Aradu.KEZ8L	1.669026766	2.353323291	2.049630768	2.9800253	3.305970521
Aradu.Y3QBI	-1.32192809	-2.47393119	-1.68965988	1.244887059	1.956056652

Aradu.34LNY	0.992768431	1.389566812	0.731183242	2.23878686	2.737686761
Aradu.BCZ93	1.411426246	1.555816155	2.575312331	1.321928095	1.735522177
Aradu.P5HY6	3.744161096	3.82374936	3.529820947	4.189033824	4.586764743
Aradu.PV79J	0.014355293	0.748461233	0.411426246	0.963474124	1.298658316
Aradu.Y1B10	-0.13606155	0.124328135	-0.04394335	0.422233001	1.454175893
Aradu.YU82F	2.879705766	3.560714954	3.189033824	4.63343121	4.605849867
Aradu.64Z0M	NA	NA	NA	NA	NA
Aradu.YXH0F	-3.18442457	-6.64385619	-6.64385619	NA	-3.47393119
Aradu.U3ACN	1.883620816	2.558267634	2.189033824	3.253989266	3.638073837
Aradu.33VFZ	-2.32192809	-2.39592868	-2.83650127	0.150559677	-0.43440282
Aradu.D3REG	2.289834465	2.737686761	2.275007047	3.504620392	3.513490746
Aradu.8GK1W	-1.78587519	-1.12029423	-2.73696559	1.956056652	1.389566812
Aradu.GA8V3	0.411426246	1.15704371	0.722466024	1.682573297	1.735522177
Aradu.04L8A	0.516015147	1.070389328	0.847996907	1.292781749	1.257010618
Aradu.2E3F6	NA	-4.32192809	-6.64385619	-2.55639335	-2.94341647
Aradu.G40TW	-2.47393119	-2.32192809	-2.94341647	0.847996907	0.713695815
Aradu.U9SCT	3.23572706	3.051372102	3.240314329	1.794935663	1.807354922
Aradu.D4CJV	0	0.516015147	1.189033824	0.941106311	1.201633861
Aradu.89ZRN	2.327687364	2.819668183	2.790772038	3.184280294	3.821710215
Aradu.I7M78	0.176322773	0.757023247	0.475084883	0.871843649	0.970853654
Aradu.Q78JW	1.575312331	1.906890596	1.794935663	2.646162657	2.46727948
Aradu.3X1QZ	-1.35845397	-1.47393119	-1.35845397	2.887525271	2.424922088

Aradu.88K72	-0.47393119	-0.02914635	-0.16812276	-2.39592868	0.23878686
Aradu.XDM1U	2.298658316	2.827819025	2.347665656	3.783456654	3.580145484
Aradu.DLR8J	2.948600847	3.300123725	3.028569152	3.428946345	3.469885976
Aradu.X4KI4	NA	NA	NA	NA	-6.64385619
Aradu.H8P6Y	-0.30400619	0.731183242	0.40053793	-0.94341647	2.176322773
Aradu.I5ETD	NA	NA	NA	NA	NA
Aradu.L7XAF	0.584962501	1.604071324	1.321928095	-0.88896869	0.189033824
Aradu.X57SP	-1	-1.05889369	-0.94341647	NA	-1.59946207
Aradu.8A8WP	-1.21759144	-1.83650127	-1.18442457	-2.83650127	-0.34007544
Aradu.7N75D	-3.32192809	-3.83650127	-5.05889369	-4.32192809	-2.64385619
Aradu.9R9X3	8.931328485	8.595369386	9.097926773	2.056583528	2.650764559
Aradu.YMP6T	-0.94341647	-0.28630419	-2.47393119	2.169925001	2.761285273
Aradu.M76UB	4.100977648	4.634012356	4.326968712	5.181500152	5.017476563
Aradu.L5T1V	3.56924803	3.693765712	3.642701572	3.538538164	3.988230223
Aradu.96IA2	-5.64385619	-4.32192809	-3.83650127	1.163498732	3.028569152
Aradu.K9P9T	2.480265122	2.897240426	2.23878686	4.547819957	4.339137385
Aradu.FC1L9	5.01658546	4.23572706	4.402585758	4.471837762	5.273142859
Aradu.W71G4	4.163498732	4.675251386	4.478324711	3.799087306	3.813524689
Aradu.ZY67R	NA	-5.64385619	NA	-4.64385619	-4.64385619
Aradu.J4R4W	2.869871406	2.849999259	2.632268215	1.66448284	2.693765712
Aradu.K10TZ	-0.71311885	0.722466024	0.163498732	1.687060688	1.925999419
Aradu.YAN6P	4.478324711	4.412781525	4.297191417	4.221877081	4.516645558

Aradu.8LE5E	2.266036894	2.720278465	2.40053793	3.337711092	3.642701572
Aradu.TQ01E	-0.08926734	1.163498732	-0.66657627	6.156032499	6.4264325
Aradu.X7ZD5	-5.05889369	-5.05889369	-6.64385619	-2.83650127	-0.10469738
Aradu.RYB7F	-5.05889369	-3.64385619	-4.64385619	-1.59946207	-1.08926734
Aradu.R659W	2.693765712	3.021479727	2.613531653	4.524815928	4.542877099
Aradu.9631L	1.773996325	2.049630768	1.778208576	2.269033146	2.671293372
Aradu.JK0W0	1.111031312	1.541019153	1.23878686	2.060047384	2.435628594
Aradu.I3I8S	3.066950244	3.047887329	3.039138394	3.690417096	4.263785614
Aradu.IW8J6	1.918386234	2.192194165	1.887525271	2.378511623	2.587364991
Aradu.R5ZHP	1.744161096	2.13093087	1.895302621	2.655351829	2.548436625
Aradu.N3SPH	-2.47393119	-1.94341647	-1.68965988	0.367371066	0.042644337
Aradu.M530F	1.389566812	1.678071905	1.292781749	1.560714954	2.042644337
Aradu.92KLI	3.23572706	3.771885579	3.481557281	4.424922088	4.74092756
Aradu.UB339	2.060047384	2.182692298	1.384049807	-1.18442457	-0.23446525
Aradu.425TL	1.704871964	2	1.786596362	2.427606173	2.678071905
Aradu.E34IK	NA	NA	NA	NA	NA
Aradu.16BXD	3.911691582	4.295723025	4.054848477	3.499527024	3.509695842
Aradu.H129B	-2.39592868	0.070389328	0.765534746	-0.577767	0.622930351
Aradu.F1MD3	-1.08926734	-1.51457317	-1.83650127	-2.32192809	-0.68965988
Aradu.12LHJ	-1	0.641546029	0.632268215	1.3950628	1.575312331
Aradu.5Q1VY	1.021479727	2.275007047	3.263034406	4.876271361	4.691534165
Aradu.PQW7I	3.969933275	3.888499736	4.09592442	2.700439718	2.871843649

Aradu.D0RIM	3.787641414	3.845991771	3.835924074	4.09592442	4.408711861
Aradu.8SL2K	3.928844037	4.280213996	4.092545742	3.98458935	4.395748328
Aradu.A22XE	0.650764559	1.910732662	0.650764559	1.827819025	1.815575429
Aradu.GC6L4	3.147306699	3.450221496	3.053111336	3.578938713	4.046141782
Aradu.K49J7	NA	NA	NA	NA	-4.05889369
Aradu.I6I4A	NA	NA	-6.64385619	NA	3.053111336
Aradu.R8T0J	1.735522177	2.319039816	1.944858446	1.906890596	3.272023189
Aradu.SL9AV	2.746312766	3.527320608	3.541019153	2.627606838	2.827819025
Aradu.ULI8B	-0.04394335	0.150559677	-0.13606155	-1.12029423	-0.39592868
Aradu.317LS	-1.21759144	-2.73696559	-2.73696559	-3.05889369	-1.25153877
Aradu.82VY6	-1.05889369	-0.49410907	-1.47393119	2.722466024	2.914564523
Aradu.NHR1S	2.372952098	4.165911939	2.969012308	6.911571752	6.957334247
Aradu.4T5J3	-0.34007544	-0.04394335	-0.05889369	-1.47393119	-1.05889369
Aradu.EP05F	2.604071324	2.780310099	2.680324357	2	2.653060017
Aradu.Y48CE	5.947198584	7.048323387	7.128664587	4.157852169	4.026800059
Aradu.7BV8W	-0.47393119	-0.05889369	-0.37706965	-1.18442457	-0.73696559
Aradu.ZM93S	1.459431619	1.516015147	1.40053793	2.970853654	2.3950628
Aradu.AXU8L	2.500802053	3.679198571	2.565597176	4.695993813	4.588564737
Aradu.J4C77	2.192194165	2.548436625	2.003602237	3.117695043	3.089159132
Aradu.T01MJ	2.811471031	3.19061486	2.987320866	3.122672719	3.203201156
Aradu.25L3E	2.10433666	2.3950628	2.028569152	1.111031312	1.014355293
Aradu.970IC	0.042644337	0.310340121	0.056583528	0.250961574	0.669026766

Aradu.RZ908	3.049630768	3.448900951	3.037382222	4.334139179	4.526694846
Aradu.M1GCR	-1.88896869	1.531069493	1.632268215	-2.05889369	-1.59946207
Aradu.59RNH	5.41987593	5.412442825	4.716990894	6.913966466	7.325710208
Aradu.H03KA	-3.05889369	-2.05889369	-3.47393119	-2.47393119	1.97819563
Aradu.YLB4V	1.339137385	1.895302621	1.695993813	2.801158656	2.711494907
Aradu.9R07T	-1	-0.21759144	-0.97143085	1.432959407	2.107687869
Aradu.DHI4B	NA	-5.64385619	NA	-5.64385619	NA
Aradu.BY7GE	-0.73696559	-0.34007544	-0.64385619	0.014355293	0.739848103
Aradu.LW83G	3.243364426	3.341985747	3.181102551	4.527320608	4.582556003
Aradu.PBY44	1.117695043	2.169925001	1.66448284	2.272023189	1.970853654
Aradu.WU7D1	-5.05889369	-4.64385619	-4.32192809	-5.64385619	-3.05889369
Aradu.X6VZP	3.59812696	3.748461233	3.375734539	2.833902077	3.22342255
Aradu.KWJ2K	-5.05889369	-5.05889369	-4.64385619	-2.47393119	2.010779839
Aradu.475J2	-0.34007544	0.028569152	-0.71311885	1.063502942	1.292781749
Aradu.E4NC1	-1.28630419	-2.73696559	-1.12029423	NA	NA
Aradu.J32IT	2.107687869	2.589763487	2.443606651	2.403267722	2.933572638
Aradu.E90C6	2.003602237	2.353323291	1.744161096	2.937344392	2.871843649
Aradu.AW9HW	1.195347598	1.819668183	1.475084883	1.709290636	2.10433666
Aradu.9R7J6	-1.88896869	-1.59946207	-3.32192809	-0.28630419	-0.55639335
Aradu.D8HZZ	NA	NA	-6.64385619	-6.64385619	-6.64385619
Aradu.NE42A	-2.18442457	-1.21759144	-1.55639335	1.269033146	0.98550043
Aradu.944BF	1.867896464	2.298658316	1.682573297	2.963474124	3.148934105

Aradu.031YC	3.527320608	3.481557281	3.559491813	4.165911939	4.107687869
Aradu.8T38A	1.608809243	0.97819563	1.14404637	1.59454855	1.855989697
Aradu.H0G14	-1.94341647	0.201633861	0.454175893	1.480265122	1.042644337
Aradu.DHT3V	3.393690764	3.578938713	3.751677946	2.61117238	2.201633861
Aradu.DT3LR	-5.05889369	-3.83650127	-4.64385619	-5.64385619	-2.55639335
Aradu.5G6X0	0.014355293	0.659924558	0.485426827	0.310340121	0.298658316
Aradu.CJP5L	2.855989697	2.6622055	2.260025656	3.884597921	4.015247774
Aradu.V9U81	4.425593578	4.66448284	4.814037648	4.046141782	4.211790976
Aradu.63ZUN	-1.64385619	-0.91593574	-1.59946207	0.50589093	0.485426827
Aradu.D03W8	-0.66657627	0.687060688	0.526068812	2.324810603	2.364572432
Aradu.DGW3H	-4.32192809	-6.64385619	-5.64385619	NA	-6.64385619
Aradu.4I5UW	NA	-5.64385619	-6.64385619	-1.25153877	-2.73696559
Aradu.L3WM9	0.632268215	1.137503524	0.713695815	0.963474124	0.669026766
Aradu.4X75W	NA	NA	NA	NA	NA
Aradu.Y5B7U	2.819668183	3.061776198	2.881664619	3.788685711	3.626439137
Aradu.GS270	4.575312331	5.213347282	4.132576843	6.283551423	6.194756854
Aradu.U16MC	2.137503524	2.707082992	2.232660757	3.300123725	3.600507645
Aradu.U7UZ6	1.182692298	0.799087306	0.201633861	-0.94341647	1.454175893
Aradu.0C6YR	-2.25153877	-1.32192809	-2.73696559	1.906890596	1.550900665
Aradu.Q6596	1.618238656	1.599317794	1.432959407	3.075532631	3.171527106
Aradu.BS3NC	2.09423607	2.182692298	2.073820233	2.117695043	2.56315813
Aradu.03X4Q	8.059074015	7.626439137	7.920471958	3.5360529	3.558267634

Aradu.9CT7A	2.053111336	2.247927513	1.989139007	2.344828497	2.831877241
Aradu.C7JRZ	NA	-5.64385619	-3.47393119	-3.64385619	1.500802053
Aradu.F40AG	-3.64385619	-3.83650127	-3.83650127	NA	-2.05889369
Aradu.BYJ1W	-2.25153877	-2	-2.39592868	1.367371066	1.879705766
Aradu.7I0FT	-0.32192809	0.443606651	-0.07400058	0.189033824	0.201633861
Aradu.R18J1	-0.39592868	-0.26881676	-0.26881676	0.895302621	1.550900665
Aradu.J0SGA	NA	NA	NA	NA	NA
Aradu.RY9YC	2.353323291	2.763411574	2.381283373	3.698218478	3.654206378
Aradu.QXQ1E	1.10433666	3.576522138	3.334854269	0.454175893	1.250961574
Aradu.PIJ3J	3.919340082	3.85698569	3.921245889	3.363171077	3.344828497
Aradu.E3GM7	-0.23446525	1.117695043	-0.81096618	2.702657543	2.784503983
Aradu.7R439	-5.64385619	-6.64385619	-6.64385619	-4.64385619	NA
Aradu.8VI8X	1.803227036	1.627606838	0.411426246	1.422233001	2.742006211
Aradu.S8QXC	-4.05889369	-3.64385619	-3.83650127	0.275007047	-0.08926734
Aradu.5E0HP	-0.91593574	0.014355293	-0.97143085	1.589763487	1.790772038
Aradu.272PC	-1.51457317	-1.02914635	-1.35845397	1.831877241	2.195347598
Aradu.8X6ET	-5.05889369	NA	-6.64385619	NA	NA
Aradu.J09BS	0.214124805	-0.34007544	0.443606651	-5.64385619	-5.05889369
Aradu.IWK4F	NA	-6.64385619	-5.64385619	NA	-6.64385619
Aradu.7T5LQ	5.738497644	6.377817853	5.911212202	6.56208975	6.768051961
Aradu.I94L5	2.831877241	2.845991771	2.669026766	1.195347598	2.419538892
Aradu.634DQ	-3.47393119	-2.12029423	-2.05889369	-1.32192809	-2.05889369

Aradu.V68II	3.454175893	3.801158656	3.835924074	3.106013238	3.462052319
Aradu.TE378	2.166715445	2.715893371	2.304511042	3.627606838	3.821710215
Aradu.KG41H	4.491853096	6.718772592	5.70348835	1.097610797	3.504620392
Aradu.LZC3L	NA	NA	NA	NA	-2.64385619
Aradu.E5U8P	-1.39592868	-2.32192809	-1.18442457	-1.43440282	-0.59946207
Aradu.AKB12	3.613531653	4.320484678	3.759155834	3.087462841	3.264536431
Aradu.5Y3I5	1.464668267	2.726831217	2.500802053	3.294253136	3.460742564
Aradu.Z8W63	-1.08926734	-0.97143085	-0.05889369	-4.64385619	-5.05889369
Aradu.XQ1BV	-2.94341647	-3.64385619	-5.05889369	NA	-3.32192809
Aradu.T5CVQ	NA	NA	NA	-2.39592868	-3.18442457
Aradu.SJ6MW	-0.35845397	-0.45403163	-0.88896869	0.5360529	1.097610797
Aradu.UNY97	-4.32192809	-4.64385619	-5.64385619	-5.64385619	-3.47393119
Aradu.GI6IZ	1.925999419	2.500802053	2.166715445	2.819668183	2.875780063
Aradu.J1JIJ	0.948600847	0.704871964	1.09085343	NA	-4.64385619
Aradu.P47CH	NA	-5.05889369	-5.64385619	-3.64385619	-1.25153877
Aradu.R2E4D	6.708187236	5.601696516	6.018144529	-2.47393119	-0.10469738
Aradu.AM8Z8	0.839959587	0.687060688	0.443606651	1.673556424	1.933572638
Aradu.1DV9M	0.98550043	1.263034406	1.111031312	1.469885976	1.89917563
Aradu.09F0B	2.066950244	2.794935663	2.726831217	1.327687364	0.815575429
Aradu.W23YF	1.09085343	1.495695163	1.014355293	2.292781749	2.211012193
Aradu.CT2LH	2.711494907	2.372952098	2.584962501	1.765534746	3.014355293
Aradu.V8J7V	2.073820233	2.386810946	2.23572706	2.763411574	3

Aradu.S1UZ6	NA	-5.05889369	NA	NA	-5.05889369
Aradu.SP6EC	NA	NA	NA	NA	-5.64385619
Aradu.30RBD	1.333423734	1.948600847	1.432959407	2.214124805	2.09085343
Aradu.16HC5	2.629939409	3.386810946	2.500802053	4.579542225	4.465974465
Aradu.T9MDP	1.220329955	1.269033146	1.014355293	0.464668267	1.298658316
Aradu.3B9N0	NA	NA	NA	-4.64385619	-3.83650127
Aradu.BS3D3	NA	-6.64385619	-4.64385619	-4.64385619	-5.05889369
Aradu.88Z5A	1.811471031	2.347665656	1.895302621	3.578938713	3.808385051
Aradu.3X0HY	2.22342255	2.704871964	2.353323291	2.715893371	2.941106311
Aradu.FC1CK	2.780310099	2.575312331	2.35614381	1.316145742	1.731183242
Aradu.60I66	-5.64385619	-4.64385619	-6.64385619	NA	-4.32192809
Aradu.JZI09	-0.25153877	0.389566812	0.097610797	1.480265122	1.941106311
Aradu.4P2F5	8.801126314	8.617063344	9.157927939	3.584962501	3.326249701
Aradu.07AAJ	NA	NA	NA	NA	-4.32192809
Aradu.R1FC6	-3.32192809	-3.32192809	-2.83650127	-0.28630419	0.097610797
Aradu.8L8L4	3.615887074	2.976363636	3.012568674	2.839959587	2.788685711
Aradu.LAI2T	-2.05889369	-2.05889369	-1.78587519	-2.73696559	0.815575429
Aradu.81HAH	-1.35845397	-1.21759144	-2.55639335	0.321928095	-0.49410907
Aradu.L2JLA	0.782408565	-0.94341647	-1.83650127	NA	-2.64385619
Aradu.JN5HH	1.847996907	1.480265122	1.269033146	3.533563348	3.168321116
Aradu.QG2PF	-5.05889369	-4.32192809	-6.64385619	-0.91593574	-1.39592868
Aradu.P3N99	5.39609097	5.729280846	4.87282876	6.843858256	6.765799923

Aradu.KUP4F	-1.28630419	0.176322773	-1.39592868	1.948600847	1.948600847
Aradu.X9GQ3	-3.18442457	-1.78587519	-2.32192809	2.173127433	0.367371066
Aradu.22L4T	0.992768431	1.687060688	1.475084883	2.657640005	2.813524689
Aradu.JVP4N	-4.64385619	-3.83650127	-3.32192809	-4.05889369	3.129283017
Aradu.KLU2C	4.762880293	4.66618849	5.194165869	0.807354922	0.367371066
Aradu.NI44V	2.313245852	1.811471031	1.66448284	2.448900951	3.021479727
Aradu.C9WV0	2.032100843	2.283921772	2.025028794	2.545968369	2.927896454
Aradu.N7NSR	NA	NA	NA	NA	NA
Aradu.PXH87	5.906650126	5.720552092	6.099084761	3.301587647	4.359661722
Aradu.NL8HQ	4.030336078	5.645586386	5.41379715	1.691534165	0.713695815
Aradu.71RRV	3.451540833	3.316145742	3.737686761	-0.62148838	-0.37706965
Aradu.85KJF	2.353323291	2.695993813	2.634593268	2.790772038	3.695993813
Aradu.0XA87	2.969012308	2.477677328	2.575312331	3.206330648	3.228049048
Aradu.8Z0S4	-5.05889369	-5.64385619	-5.05889369	-1.47393119	-2.55639335
Aradu.BC220	1.550900665	1.646162657	1.618238656	2.887525271	3.272023189
Aradu.84XE2	-2.18442457	-1.51457317	-2.55639335	-0.04394335	-0.28630419
Aradu.215DG	-2.32192809	-1.83650127	-1.94341647	-2.73696559	-1.68965988
Aradu.MI3Z8	-2.64385619	-2.32192809	-3.32192809	0.516015147	1.263034406
Aradu.6V6LL	0.443606651	-0.02914635	-0.28630419	-1.83650127	0.176322773
Aradu.PZ509	-1.39592868	-2.18442457	-2.12029423	-0.53533173	0.23878686
Aradu.BG2Q4	-3.05889369	-4.64385619	-3.83650127	-3.64385619	-3.18442457
Aradu.RU8ZD	NA	NA	NA	NA	NA

Aradu.XAE56	2.582556003	2.684818738	2.599317794	2.922197848	2.992768431
Aradu.D4WQD	-2.73696559	-2.39592868	-2.94341647	-0.47393119	-0.45403163
Aradu.S9LIL	NA	NA	NA	NA	NA
Aradu.AGS84	1.389566812	1.269033146	1.304511042	0.310340121	1.021479727
Aradu.R63W9	-0.62148838	0.35614381	-0.39592868	1.03562391	0.941106311
Aradu.257BX	2.304511042	2.680324357	2.454175893	1.570462931	1.765534746
Aradu.3T4GH	3.898208353	4.049630768	4.240314329	3.476381688	3.87774425
Aradu.1EC42	1	0.895302621	0.871843649	0.23878686	-0.16812276
Aradu.WM0X2	2.247927513	2.021479727	1.851998837	1.028569152	2.166715445
Aradu.1QD6E	-1.83650127	-1.15200309	-2.55639335	0.23878686	0.344828497
Aradu.S66GY	-2.55639335	-6.64385619	-3.64385619	0.464668267	0.411426246
Aradu.LD3HZ	1.713695815	2.147306699	1.827819025	3.085764554	3.279471296
Aradu.14D5A	3.336283388	3.657640005	3.224966365	3.733354341	3.850999395
Aradu.PT44X	7.061128135	6.763544364	7.328226119	0.344828497	0.097610797
Aradu.ZTY0U	1.739848103	2.298658316	1.86393845	3.612352499	3.558267634
Aradu.D5GAK	NA	-6.64385619	NA	NA	-2.73696559
Aradu.LGS6Z	4.730639956	4.744699314	4.546585829	5.322649262	5.484138131
Aradu.UH61L	-4.64385619	-3.64385619	-3.32192809	-0.81096618	-1.32192809
Aradu.E2AL2	-0.62148838	0.765534746	0.310340121	1.819668183	2.166715445
Aradu.7MW89	2.341985747	2.560714954	2.392317423	3.005399988	3.56924803
Aradu.V8MJ9	6.688739868	6.443275112	6.570159302	2.084064265	2.510961919
Aradu.6PX5N	-1.78587519	-3.32192809	-2.73696559	-4.64385619	-0.34007544

Aradu.HKL6E	2.873813198	3.35614381	3.241840184	4.289096702	4.571069998
Aradu.EZW4U	6.845866357	7.984133595	7.848685529	6.63531908	6.791162889
Aradu.IL9WG	0.565597176	1.063502942	0.847996907	2	2.516015147
Aradu.ISN1L	-3.32192809	-3.83650127	-5.05889369	-1.51457317	0.333423734
Aradu.FS4N2	NA	NA	NA	NA	4.08236197
Aradu.XS31R	0.35614381	0.632268215	0.286881148	1.641546029	2.204766751
Aradu.W56IJ	3.448900951	3.727920455	3.414135533	3.754887502	4.13422094
Aradu.1FC14	-0.07400058	0.201633861	-0.12029423	1.673556424	1.735522177
Aradu.0KB9D	-5.64385619	-4.32192809	-4.32192809	0.565597176	1.819668183
Aradu.B7Y20	1.298658316	2.028569152	1.752748591	1.464668267	2.080657663
Aradu.8J98F	-0.13606155	-0.39592868	-0.86249648	-2	-0.86249648
Aradu.A68HE	-1.55639335	-1.05889369	-1.68965988	0	-0.12029423
Aradu.LDI07	1.028569152	0.454175893	1.286881148	-0.91593574	2.185866545
Aradu.FU7NG	0.028569152	0.970853654	0.495695163	1.500802053	1.570462931
Aradu.PRW5G	3.642701572	3.121015401	2.72900887	-1.05889369	-0.86249648
Aradu.ZX8WQ	-2.55639335	-4.05889369	-3.64385619	-0.4150375	-0.43440282
Aradu.V9CJA	-4.32192809	-4.64385619	-3.83650127	-2.94341647	-0.66657627
Aradu.JV441	5.385085854	5.312882955	5.698774109	4.422905743	4.14974712
Aradu.LX0F3	1.687060688	2.23572706	1.831877241	2.819668183	2.99095486
Aradu.F8VRL	-1	-1.08926734	-1.02914635	-0.32192809	-0.26881676
Aradu.EG6L6	NA	-6.64385619	NA	-4.32192809	-3.47393119
Aradu.F99CN	1.10433666	0.855989697	2.214124805	0.86393845	0.855989697

Aradu.73HE8	0.23878686	0.82374936	0.839959587	0.82374936	0.422233001
Aradu.XW987	NA	NA	NA	NA	-5.05889369
Aradu.K5HNP	1.989139007	2.025028794	1.827819025	2.319039816	2.819668183
Aradu.U8TGW	-0.35845397	-0.73696559	-0.64385619	1.13093087	1.521050737
Aradu.VS4QJ	-1.39592868	-0.39592868	-1.15200309	0.97819563	0.98550043
Aradu.T8SW7	-0.64385619	-0.26881676	-0.45403163	0.250961574	0.298658316
Aradu.4YQ11	1.718087584	-3.18442457	1.189033824	-0.30400619	-5.05889369
Aradu.V5ZS1	1.09085343	0.963474124	1.321928095	1.40053793	1.691534165
Aradu.9AI6Q	-2.32192809	-0.30400619	-0.59946207	0.847996907	1.163498732
Aradu.CW450	0.286881148	0.475084883	0.298658316	1.15704371	1.63691458
Aradu.KD2FK	-2	-1.43440282	-2.39592868	1.761285273	2.063502942
Aradu.7VQ43	3.174725988	3.375734539	3.001802243	4.157852169	4.44625623
Aradu.X3TFJ	3.75595577	4.169925001	4.086613947	2.851998837	2.59454855
Aradu.7M26B	3.001802243	3.070389328	2.666756592	4.301587647	4.185073638
Aradu.M8FIQ	2.424922088	2.40053793	2.347665656	3.280956314	3.502075956
Aradu.R2M0E	-0.28630419	0.22650853	0.86393845	3.867896464	3.554588852
Aradu.Z8VLV	-0.08926734	0.669026766	0.310340121	1.718087584	2.077242999
Aradu.MQ2DW	4.316869805	4.541638734	4.126807703	5.350850805	5.51916445
Aradu.27F3R	3.264536431	3.353323291	3.080657663	2.606442228	3.037382222
Aradu.R6QT2	2.906890596	2.64385619	1.948600847	1.910732662	2.503348735
Aradu.DG44N	1.778208576	2.17951105	1.275007047	3.424922088	3.255500733
Aradu.BU5Q7	5.949534933	5.571069998	5.836429131	2.384049807	2.232660757

Aradu.L8BYH	NA	-3.64385619	-6.64385619	NA	NA
Aradu.D85KR	2.451540833	2.684818738	2.23572706	2.292781749	2.247927513
Aradu.09HMV	1.906890596	1.735522177	1.851998837	1.879705766	1.918386234
Aradu.B8V9A	0.59454855	1.959770155	2.137503524	0.150559677	1.03562391
Aradu.NE9V5	0.475084883	0.839959587	0.443606651	0.910732662	1.007195501
Aradu.K9162	-3.64385619	-3.83650127	-4.32192809	-2.47393119	-2.83650127
Aradu.IY93L	4.204766751	4.564987801	3.963474124	3.892391026	3.845991771
Aradu.11V2F	1.201633861	1.411426246	0.90303827	3.498250868	4
Aradu.0DL1W	3.368768349	3.246408087	3.075532631	2.384049807	3.298658316
Aradu.9SL8H	0.948600847	1.361768359	1.084064265	1.622930351	0.887525271
Aradu.6R3YG	-4.32192809	-3.47393119	-3.47393119	-0.64385619	-0.26881676
Aradu.22IYP	-5.64385619	-6.64385619	-5.64385619	-5.64385619	-2.12029423
Aradu.F3VDW	1.021479727	1.618238656	1.214124805	2.659924558	3.078951341
Aradu.R65GQ	5.192194165	5.241077458	4.993221467	5.283551423	5.353323291
Aradu.RM7GK	NA	NA	-6.64385619	NA	NA
Aradu.JT1JK	2.097610797	2.965322548	3.195347598	3.218781168	2.857980995
Aradu.131HS	2.739848103	3.500802053	3.288358562	3.9202933	4.075532631
Aradu.S5XS2	1.269033146	0.807354922	0.111031312	4.010779839	3.619413011
Aradu.D7VWN	-0.07400058	1.485426827	0.344828497	2.849999259	2.931683057
Aradu.R1SGR	2.538538164	2.464668267	2.411426246	2.901108243	3.374343989
Aradu.R0TXA	-3.05889369	-1.55639335	-4.64385619	0.956056652	1.427606173
Aradu.Y5UE5	NA	-5.05889369	-5.64385619	NA	NA

Aradu.65H3Y	2.087462841	1.718087584	1.584962501	1.713695815	2.03562391
Aradu.EZ7WI	3.19061486	3.7473874	3.427606173	3.619413011	3.99095486
Aradu.FC7FL	NA	-6.64385619	NA	-0.91593574	-1.64385619
Aradu.0XB6R	0.650764559	0.97819563	0.748461233	1.90303827	2.482848283
Aradu.TL35C	2.680324357	2.897240426	2.538538164	2.639232163	2.488000771
Aradu.JZ25F	0.98550043	1.416839742	0.933572638	1.709290636	1.89917563
Aradu.P7QDD	-3.64385619	-6.64385619	-4.05889369	-0.81096618	-1.43440282
Aradu.U9IPR	1.042644337	1.799087306	2.350497247	2.983677695	2.931683057
Aradu.R83G6	0.163498732	0.782408565	0.82374936	1.13093087	1.98550043
Aradu.C5W7K	0.970853654	1.722466024	0.464668267	2.361768359	2.538538164
Aradu.N8S8A	2.153805336	2.443606651	2.23572706	2.752748591	3.347665656
Aradu.39QNU	-2.83650127	-2.25153877	-4.05889369	NA	-3.05889369
Aradu.16CAR	2.500802053	2.759155834	2.577730931	2.604071324	2.948600847
Aradu.YF31D	2.498250868	3.0721058	2.713695815	4.01346226	4.059182199
Aradu.AT974	-2.73696559	-1.59946207	-3.18442457	-2.55639335	-0.91593574
Aradu.Q95MC	2.292781749	2.63691458	2.361768359	3.491853096	4.526068812
Aradu.8GL4A	NA	-5.05889369	-5.05889369	-5.05889369	-5.64385619
Aradu.W9G7C	1.879705766	1.646162657	1.516015147	2.09423607	2.56315813
Aradu.70NLN	1.887525271	2	1.704871964	1.646162657	2.232660757
Aradu.10YFJ	-6.64385619	-5.05889369	-5.64385619	-4.32192809	-0.15200309
Aradu.Z705N	5.964860664	4.824258697	5.555816155	0.176322773	-0.20091269
Aradu.M2MY1	3.075532631	3.025028794	2.813524689	2.608809243	4.084064265

Aradu.I27F7	NA	NA	NA	NA	NA
Aradu.6CW5S	3.703765179	3.909773104	3.794935663	4.063502942	4.272023189
Aradu.NCJ0H	1.599317794	1.731183242	1.22650853	-0.94341647	-1.73696559
Aradu.G76IS	1.726831217	2.59454855	2.077242999	4.304511042	4.475084883
Aradu.X8GPB	0.782408565	0.918386234	0.443606651	1.910732662	2.440952198
Aradu.MP8TG	2.689299161	2.918386234	2.646162657	4.638073837	4.450881315
Aradu.J3IKA	2.046141782	2.589763487	1.963474124	2.737686761	3.261530815
Aradu.05VAM	2.003602237	2.3950628	1.918386234	2.817623258	3.62058641
Aradu.SJ7I8	-0.83650127	-0.83650127	-1.21759144	0.014355293	1.232660757
Aradu.2SV86	2.472487771	3.240314329	2.17951105	2.653060017	2.500802053
Aradu.J1D71	0.807354922	2.003602237	1.989139007	-1.43440282	-0.53533173
Aradu.DD99U	1.599317794	2.03562391	1.726831217	2.140778656	2.456806149
Aradu.8UL46	1.622930351	1.956056652	1.650764559	2.803227036	2.538538164
Aradu.2HD04	NA	-5.05889369	-6.64385619	0.575312331	1.748461233
Aradu.KG94L	1.23878686	1.350497247	0.879705766	0.757023247	0.910732662
Aradu.76H6A	4.050501697	4.354028938	3.94204526	2.831877241	2.992768431
Aradu.2LH1B	1.137503524	3.7322692	2.9800253	2.503348735	2.23572706
Aradu.SMR94	4.744161096	4.844486088	4.879705766	4.558267634	4.63343121
Aradu.L18YW	2.925999419	5.192194165	5.629065137	3.607626221	4.275007047
Aradu.26288	NA	-6.64385619	NA	-5.64385619	NA
Aradu.34MNK	1.40599236	1.59454855	1.232660757	3.017921908	3.047887329
Aradu.883F2	NA	-6.64385619	NA	NA	NA
Aradu.VC6I6	NA	-6.64385619	NA	NA	-6.64385619

Aradu.MY8MV	-4.32192809	-5.05889369	-5.05889369	-2.73696559	-1.47393119
Aradu.G44I1	2.204766751	1.98550043	1.97819563	0.782408565	1.794935663
Aradu.301P6	NA	NA	NA	-0.21759144	-0.07400058
Aradu.6E81Q	4.628190335	5.049630768	4.687620632	5.455162801	5.426600226
Aradu.7N2H0	6.114575251	4.813524689	5.766065051	-0.73696559	1.286881148
Aradu.44DMI	4.450221496	4.367371066	4.294988268	4.201633861	4.11353382
Aradu.46Z90	1.5360529	-0.37706965	-0.34007544	1.575312331	2.269033146
Aradu.FJZ7Z	-0.12029423	0.263034406	-0.13606155	2.084064265	2.316145742
Aradu.GMN03	0.895302621	0.963474124	0.604071324	2.080657663	1.89917563
Aradu.4SA47	0.632268215	1.744161096	0.50589093	3.326249701	3.583759754
Aradu.K0D8U	1.327687364	1.416839742	1.097610797	1.111031312	1.739848103
Aradu.1G6QS	1.480265122	1.678071905	1.372952098	1.63691458	1.604071324
Aradu.S68YF	-1.94341647	-1.51457317	-2.83650127	-0.32192809	0.137503524
Aradu.HD7PF	-5.64385619	-2.73696559	-4.64385619	-0.91593574	-0.62148838
Aradu.T9UN0	-1.83650127	-0.83650127	-2.12029423	1.996388746	2.214124805
Aradu.FNB9W	2.03562391	2.327687364	2.073820233	2.572889668	3.943921327
Aradu.MY1HL	2.260025656	2.722466024	2.339137385	4.065227623	3.876762491
Aradu.FDG48	3.249445341	2.411426246	1.86393845	3.343407822	3.608809243
Aradu.PIU2J	0.713695815	1.182692298	0.831877241	1.98550043	2.121015401
Aradu.HY9UB	NA	NA	NA	NA	-4.05889369
Aradu.13Y5Y	1.292781749	2.077242999	1.726831217	2.214124805	2.298658316
Aradu.K64M	4.218781168	4.167518504	4.336997417	2.724650272	3.635754391

1					
Aradu.EEF2D	1.070389328	0.86393845	0.422233001	3.137503524	2.821710215
Aradu.D9Q5D	4.008092421	4.559491813	4.698218478	3.407352751	3.622930351
Aradu.926HI	-1.88896869	-0.71311885	-1	0.014355293	0.263034406
Aradu.T09Z3	1.469885976	3.527320608	3.288358562	0.201633861	0.948600847
Aradu.QCF5U	1.207892852	1.641546029	1.521050737	2.831877241	2.989139007
Aradu.RVF1V	2.397802962	4.243364426	3.641546029	0.773996325	2.495695163
Aradu.H4VY0	NA	-6.64385619	NA	-1.59946207	4.18030702
Aradu.U99RK	3.811471031	4.100977648	3.854993017	3.344828497	3.40599236
Aradu.V5V1W	2.46727948	3.201633861	2.375734539	4.923624611	4.923624611
Aradu.9T7BM	3.927896454	4.750070486	4.167518504	4.62935662	4.70763522
Aradu.DG7AI	5.312519967	5.004501392	5.065227623	1.575312331	1.580145484
Aradu.GVQ3S	2.469885976	2.841973119	2.618238656	2.942983598	3.109360559
Aradu.K7JNV	NA	-6.64385619	-5.64385619	NA	NA
Aradu.QH7UZ	3.537296067	3.420886575	3.654206378	-1.83650127	-1.39592868
Aradu.Q70DU	5.036503334	5.377470843	5.063934306	6.131136719	6.196725061
Aradu.ZFF1D	2.289834465	2.386810946	2.23878686	2.477677328	2.680324357
Aradu.P6ZM2	NA	-4.64385619	-4.32192809	-0.43440282	-1.21759144
Aradu.HNN21	2.944858446	2.523561956	2.298658316	2.906890596	3.537296067
Aradu.F6LF0	0.084064265	0.659924558	0.111031312	2.280956314	2.025028794
Aradu.T4HI5	1.86393845	2.229587923	1.918386234	2.435628594	2.50589093
Aradu.X23JQ	-0.59946207	1.891419187	2.003602237	2.871843649	2.748461233
Aradu.B40D	-1.15200309	0.111031312	-0.51457317	-0.88896869	-1.12029423

X					
Aradu.CS27R	-0.53533173	-0.97143085	-0.83650127	0.286881148	1
Aradu.L0TFU	-4.05889369	-4.05889369	NA	-4.32192809	-1.55639335
Aradu.N3NL4	2.572889668	2.601696516	2.269033146	2.684818738	3.117695043
Aradu.AA5UH	-0.88896869	-1.08926734	-0.88896869	5.053545818	4.498889087
Aradu.LT7X3	2.077242999	2.639232163	2.042644337	4.257764969	4.328405659
Aradu.JQ4B3	2.451540833	2.550900665	2.286881148	2.873813198	3.005399988
Aradu.NL7A8	2.324810603	3.198494154	3.03562391	4.058316496	5.515069014
Aradu.X7LBF	-4.32192809	-1.05889369	-1.78587519	-4.05889369	-4.05889369
Aradu.SL2QQ	2.204766751	2.735522177	2.543495883	2.865918815	2.819668183
Aradu.V9EPJ	3.14404637	3.533563348	3.19061486	2.163498732	2.244887059
Aradu.A5XMW	1.748461233	1.963474124	1.839959587	2.653060017	2.965322548
Aradu.Z1Y2A	5.189429246	5.257387843	5.370513052	2.792855352	2.629939409
Aradu.A9F8B	3.276496666	3.220329955	3.025028794	2.641546029	2.895302621
Aradu.3EB7Q	-6.64385619	-2.94341647	-2.64385619	-1.55639335	-2.12029423
Aradu.MG1IB	1.687060688	1.257010618	1.604071324	2.659924558	2.855989697
Aradu.H2G5C	-1.08926734	-1.35845397	-1.35845397	-0.88896869	-0.73696559
Aradu.9R08Q	0.545968369	0.669026766	0.495695163	1.007195501	0.992768431
Aradu.R403Z	0.948600847	1.292781749	1.622930351	-0.4150375	1.485426827
Aradu.90QVH	NA	NA	-6.64385619	NA	-3.05889369
Aradu.I24YI	3.642701572	3.651912745	3.548436625	3.744161096	4.03562391
Aradu.S54FQ	-0.26881676	0.056583528	-0.16812276	1.86393845	1.97819563
Aradu.3DC0	1.214124805	1.35614381	1	2.454175893	2.64385619

K					
Aradu.3J25S	2.229587923	1.887525271	2.039138394	1.163498732	1.86393845
Aradu.8K8TN	7.106536769	6.069530325	6.539313931	2.780310099	3.092545742
Aradu.58K6Q	-4.32192809	-5.64385619	-5.64385619	-3.83650127	-4.05889369
Aradu.Z67WQ	-5.64385619	-6.64385619	NA	-4.64385619	NA
Aradu.WS13Y	3.49057013	3.701549057	3.370164281	4.21800615	4.35473424
Aradu.UXS7E	0.956056652	0.831877241	0.321928095	4.491853096	4.3950628
Aradu.9DJ4L	3.463360886	3.587364991	3.428946345	4.3305584	4.455491621
Aradu.Y2BEF	1.475084883	0.713695815	1.632268215	-1.28630419	-0.97143085
Aradu.Q17ZW	0.604071324	2.207892852	2.733354341	-4.05889369	-3.32192809
Aradu.PX1XB	4.475733431	4.999549087	4.617063344	4.502075956	4.334139179
Aradu.WNV5J	2.935459748	2.817623258	2.904965719	1.59454855	1.757023247
Aradu.2X4WG	-6.64385619	-5.64385619	NA	NA	NA
Aradu.L1U18	6.734980523	7.615887074	7.529040056	8.422905743	7.777879937
Aradu.QV5DJ	1.722466024	1.918386234	1.803227036	2.176322773	2.435628594
Aradu.T0ZKV	0.5360529	1.207892852	-1.59946207	2.707082992	3.340562269
Aradu.CI747	1.195347598	1.761285273	1.269033146	0.189033824	0.443606651
Aradu.72K0W	1.669026766	1.970853654	1.778208576	2.408711861	2.786596362
Aradu.MRW76	1.752748591	1.835924074	1.327687364	2.353323291	2.735522177
Aradu.3LE29	NA	NA	NA	NA	NA
Aradu.YUD54	-0.26881676	-0.12029423	-0.43440282	0.839959587	0.941106311
Aradu.GX5W4	1.485426827	1.310340121	2.160274831	0.411426246	1.372952098
Aradu.I4SX6	3.100977648	3.275007047	3.187451054	1.339137385	1.526068812

Aradu.Q5DZL	5.797272432	5.51916445	5.530445355	2.575312331	3.080657663
Aradu.Z33GV	NA	-5.64385619	NA	NA	NA
Aradu.NJ2WV	2.813524689	2.459431619	2.987320866	-2.73696559	3.003602237
Aradu.J43S7	2.386810946	3.193771743	0.40053793	5.986638472	5.363521544
Aradu.B9U3D	NA	NA	NA	NA	NA
Aradu.237HP	2.007195501	2.44625623	2.66448284	1.655351829	1.786596362
Aradu.234X6	0.59454855	0.422233001	0.124328135	0.879705766	1.124328135
Aradu.B9JN3	-6.64385619	-6.64385619	-6.64385619	-2.32192809	1.98550043
Aradu.C9WAZ	3.040892431	3.264536431	2.704871964	3.628773595	3.701549057
Aradu.764WC	0.321928095	-0.12029423	-1.43440282	0.263034406	0.124328135
Aradu.I977L	1.3950628	2.066950244	1.570462931	2.653060017	2.750606505
Aradu.6MT8W	NA	NA	NA	NA	NA
Aradu.K7CLQ	-1.28630419	-0.32192809	-0.51457317	1.176322773	0.622930351
Aradu.60HCE	9.114132735	8.877284134	9.091117985	5.062639828	5.21916852
Aradu.A8T4C	4.507160349	4.430285273	4.206330648	2.965322548	3.300123725
Aradu.B4Y17	-2	-0.73696559	-1.78587519	0.189033824	0.782408565
Aradu.T9RZI	1.847996907	2.389566812	2.010779839	2.895302621	3.234194723
Aradu.T5087	2.408711861	2.992768431	2.813524689	3.220329955	3.365972428
Aradu.M4DVD	2.195347598	2.726831217	2.389566812	3.442280035	3.622930351
Aradu.QCD9Z	-3.32192809	-1.94341647	-2.73696559	NA	-0.76121314
Aradu.Z0LGY	-4.32192809	-4.64385619	-3.18442457	-4.05889369	-2.05889369
Aradu.W7JB7	NA	NA	NA	NA	NA

Aradu.62HJK	0.748461233	0.807354922	0.056583528	0.963474124	1.021479727
Aradu.BEW9Q	-5.64385619	NA	NA	-3.83650127	-3.47393119
Aradu.D2IBT	0.454175893	1.541019153	0.739848103	2.336283388	2.528571319
Aradu.J4Z1M	-0.02914635	1.207892852	0.464668267	3.310340121	3.121015401
Aradu.HK0FS	0.687060688	0.555816155	0.485426827	0.933572638	1.09085343
Aradu.YI13V	2.056583528	2.13093087	1.66448284	2.871843649	2.82374936
Aradu.81KMB	-6.64385619	-5.05889369	-4.05889369	NA	-5.64385619
Aradu.88125	2.025028794	2.257010618	1.97819563	2.477677328	2.414135533
Aradu.FXP12	3.206330648	3.419538892	3.650764559	2.12763328	2.50589093
Aradu.VY0ZL	2.87774425	3.385431037	2.464668267	4.390942773	4.285402219
Aradu.UC2R1	0.485426827	1.201633861	0.722466024	1.510961919	1.97819563
Aradu.C0NTR	2.056583528	1.895302621	1.646162657	3.35614381	4.051372102
Aradu.4HH0P	-1.73696559	-0.78587519	-0.68965988	-0.18442457	0.028569152
Aradu.V33RZ	2.577730931	3.040892431	3.012568674	2.744161096	2.646162657
Aradu.T0VC2	2.702657543	3.166715445	2.916476644	3.443606651	3.673556424
Aradu.5D5JN	3.084064265	4.479618608	4.199279721	4.642123916	4.741466986
Aradu.2P8EW	-2.12029423	0.070389328	-0.577767	1.757023247	3.340562269
Aradu.DC7TG	2.778208576	2.799087306	2.641546029	3.209453366	3.211012193
Aradu.ZVZ4G	-3.18442457	-1.78587519	-3.64385619	2.339137385	2.861955364
Aradu.14WTD	-1.43440282	-0.78587519	-1.05889369	1.545968369	1.111031312
Aradu.8CG1C	-0.30400619	0.097610797	0.176322773	1.189033824	1.207892852
Aradu.74X9S	4.374343989	4.670727054	4.653060017	2.347665656	1.761285273

Aradu.EI4DF	0.604071324	0.678071905	0.855989697	-0.01449957	0.070389328
Aradu.9D9RN	3.669026766	3.9202933	4.060047384	2.769771739	3.237257771
Aradu.IGJ3I	2.080657663	2.163498732	1.944858446	2.214124805	2.333423734
Aradu.88Z6K	3.424922088	3.679198571	3.372952098	2.580145484	2.584962501
Aradu.UEG36	1.981852653	2.039138394	1.871843649	0.613531653	1.469885976
Aradu.XF7S6	6.17951105	6.205548911	6.137913322	2.835924074	3.608809243
Aradu.8A153	2.169925001	2.275007047	2.147306699	3.030336078	3.357552005
Aradu.TB0L3	9.835655691	9.45920618	9.810041371	6.279285561	6.325350433
Aradu.U4RIV	-1.64385619	2.518535139	2.669026766	1.15704371	1.117695043
Aradu.L4EY2	-2.18442457	-1.35845397	-1.43440282	-0.53533173	-0.66657627
Aradu.VM38K	NA	NA	NA	NA	NA
Aradu.PRD5G	-1.25153877	-0.35845397	-0.55639335	0.367371066	2.140778656
Aradu.7H8MA	NA	NA	NA	NA	NA
Aradu.L9J3S	5.518849829	5.678353654	5.643278996	6.808771159	6.704041955
Aradu.FIU9K	2.263034406	3.975446766	3.612352499	2.897240426	3.368768349
Aradu.U1RBP	NA	NA	NA	NA	NA
Aradu.F6N2R	1.891419187	2.324810603	2.124328135	3.137503524	3.484138131
Aradu.XJF09	-5.05889369	NA	NA	-4.64385619	-5.64385619
Aradu.P833S	1.786596362	2.10433666	1.757023247	2.211012193	2.327687364
Aradu.ZQK52	2.40599236	3.063502942	2.799087306	3.298658316	3.63343121
Aradu.X1LTA	2.336283388	2.684818738	1.778208576	4.040892431	4.130107179
Aradu.GS29Q	-2.05889369	-0.4150375	-0.64385619	-1.05889369	0.59454855

Aradu.HP39D	0.286881148	0.545968369	0.495695163	0.739848103	0.782408565
Aradu.78IK4	NA	-4.32192809	-4.64385619	-4.64385619	-1.32192809
Aradu.IF4XP	3.959770155	5.441616269	5.159064009	6.565597176	6.358607249
Aradu.IHD20	NA	NA	NA	NA	NA
Aradu.G344I	2.63691458	3.249445341	2.680324357	2.289834465	2.13093087
Aradu.CGX1Z	0.887525271	1.584962501	1.35614381	2.353323291	2.40053793
Aradu.NG3QM	1.298658316	1.786596362	1.049630768	1.731183242	1.941106311
Aradu.230WM	-0.62148838	0.344828497	-0.86249648	2.347665656	2.887525271
Aradu.4K089	1.480265122	1.695993813	1.678071905	0.669026766	2.981852653
Aradu.L6791	-0.07400058	0.250961574	0.201633861	-0.08926734	0.632268215
Aradu.J7CY2	NA	-5.64385619	-5.64385619	-2.55639335	-2.83650127
Aradu.FV1SH	2.22650853	2.289834465	1.952333566	2.10433666	2.13422094
Aradu.N1UVT	1.232660757	0.632268215	0.748461233	1.111031312	2.632268215
Aradu.Z5H58	3.871843649	3.554588852	4.553974808	1.778208576	3.596935142
Aradu.CMZ2S	0.669026766	1.117695043	0.378511623	2.682573297	2.918386234
Aradu.WC1MX	NA	NA	NA	-6.64385619	-4.32192809
Aradu.1864T	-4.32192809	-4.64385619	-5.64385619	-5.05889369	-3.83650127
Aradu.15R8P	3.527320608	3.442280035	3.158660175	0.871843649	1.084064265
Aradu.DWZ8T	2.778208576	3.15704371	2.843983844	3.840966704	4.161081482
Aradu.51ELU	2.477677328	3.139142019	2.49057013	5.211012193	5.613826291
Aradu.31VP0	2.972692654	2.865918815	3.246408087	3.137503524	4.201633861
Aradu.IZ4SH	-2.64385619	-2.94341647	-2.73696559	-0.13606155	-0.02914635
Aradu.4L5W	0.659924558	0.464668267	0.056583528	2.869871406	2.961623328

B					
Aradu.F950M	NA	NA	NA	1.070389328	-1.83650127
Aradu.288JJ	2.107687869	2.307428525	2.23878686	3.182692298	4.021479727
Aradu.74KVK	0.82374936	0.739848103	0.704871964	-2.39592868	-1.35845397
Aradu.4S94M	1.021479727	1.23878686	0.871843649	2.185866545	2.843983844
Aradu.SP7U9	3.950468414	3.744161096	3.613531653	4.050501697	3.922197848
Aradu.2CK5F	-3.05889369	-2.39592868	-2.25153877	-4.32192809	-4.64385619
Aradu.RT9KH	4.527946099	4.59275601	4.358958826	4.853496704	5.008092421
Aradu.E8RZK	2.09085343	2.780310099	2.140778656	4.147306699	4.06091205
Aradu.TSW1P	-0.26881676	-0.04394335	-0.76121314	1.650764559	1.316145742
Aradu.W5INW	2.150559677	2.15704371	1.704871964	1.35614381	1.350497247
Aradu.WVC4I	1.042644337	1.682573297	1.22650853	4.028569152	3.943921327
Aradu.HG8ZF	2.518535139	2.521050737	2.503348735	2.137503524	2.241840184
Aradu.LVQ6D	2.587364991	2.957914599	1.718087584	1.438292852	2.411426246
Aradu.5S2PY	NA	-6.64385619	NA	NA	-5.05889369
Aradu.VY9WF	-2.83650127	-2.83650127	-4.64385619	-0.73696559	-0.43440282
Aradu.Q018E	1.448900951	1.622930351	1.531069493	2.204766751	2.451540833
Aradu.V4R6L	-1.12029423	-1.51457317	-1.68965988	-2.64385619	-0.45403163
Aradu.18NVF	-5.64385619	-4.05889369	-4.05889369	-2.39592868	-2.55639335
Aradu.56ZRQ	0.98550043	0.90303827	0.286881148	2.948600847	2.424922088
Aradu.503J9	0.23878686	0.526068812	0.214124805	1.815575429	2.201633861
Aradu.PD7X7	-1.12029423	-0.37706965	-1.08926734	-0.73696559	2.007195501
Aradu.L9QR	5.297191417	5.509062386	5.349082146	2.424922088	2.684818738

L

Aradu.W0B0I	-2.39592868	-1.15200309	-1.02914635	-0.81096618	0.124328135
Aradu.LS2KI	NA	NA	NA	-1.28630419	-0.47393119
Aradu.XF0PF	2.3950628	2.819668183	2.063502942	3.876762491	3.850999395
Aradu.0YY6A	1.438292852	1.298658316	1.778208576	1.66448284	1.304511042
Aradu.XHT76	2.275007047	2.881664619	2.195347598	1.803227036	1.722466024
Aradu.58FPQ	-1.28630419	-1.05889369	-0.97143085	-2	-0.04394335
Aradu.SCK30	3.657640005	4.312519967	3.77925972	-0.47393119	-0.37706965
Aradu.J3FIC	4.757023247	3.824767853	4.206330648	3.298658316	3.171527106
Aradu.ZTH8V	6.299024807	7.643423316	6.726695004	3.726831217	5.349436052
Aradu.JG113	1.049630768	0.526068812	0.367371066	0.475084883	0.084064265
Aradu.H36S4	2.794935663	2.707082992	2.687060688	2.875780063	3.087462841
Aradu.C0GKW	1.851998837	2.462052319	2.275007047	0.014355293	-0.32192809
Aradu.S6TIM	1.580145484	1.411426246	1.22650853	1.925999419	2.336283388
Aradu.3Q3XZ	-2.12029423	-2.55639335	-3.32192809	0.757023247	2.17951105
Aradu.77ZLW	-3.05889369	-2.39592868	-2.12029423	-2.25153877	-2.83650127
Aradu.C3QXP	1.673556424	1.959770155	1.835924074	2.295723025	3.358958826
Aradu.8PE87	-1.43440282	-2.64385619	-1.73696559	-1.39592868	-2
Aradu.BY4US	-2.32192809	-2	-2	0.124328135	0.23878686
Aradu.46M2Y	NA	-5.64385619	-5.64385619	-5.05889369	-5.64385619
Aradu.JC6IN	1.510961919	1.678071905	0.992768431	1.941106311	1.910732662
Aradu.X0I6L	NA	-6.64385619	-5.64385619	-2.83650127	-1.47393119
Aradu.577R9	-5.05889369	-3.83650127	-5.05889369	2.304511042	1.575312331

Aradu.9NR8A	1	1.40599236	1.475084883	2.275007047	2.99095486
Aradu.085LG	-0.15200309	0.201633861	-0.25153877	0.443606651	0.748461233
Aradu.MI27L	NA	-6.64385619	-3.83650127	NA	-4.64385619
Aradu.T83RP	-5.64385619	-4.64385619	NA	-0.97143085	-0.55639335
Aradu.NII42	4.209453366	4.27351589	3.904002316	3.09085343	3.488000771
Aradu.14CRM	4.083213368	4.307428525	4.033863452	2.845991771	2.803227036
Aradu.A205B	-4.05889369	-3.18442457	-4.05889369	1.378511623	0.028569152
Aradu.90MFE	-0.34007544	-0.13606155	-0.73696559	0.475084883	0.790772038
Aradu.I5LRV	NA	NA	NA	NA	-5.64385619
Aradu.6XE07	0.956056652	1.545968369	1.189033824	1.316145742	2.263034406
Aradu.D7HT5	4.743084056	5.997066545	6.18408189	6.302867361	5.875288598
Aradu.724TL	-1.25153877	-0.78587519	-0.4150375	-1.21759144	-0.16812276
Aradu.Z6X71	3.743084056	3.589763487	3.656496371	2.778208576	2.976363636
Aradu.V1NXI	4.661635602	4.894817763	4.564378169	4.856487779	5.242221395
Aradu.VZ2PZ	-1.32192809	-0.94341647	-1.02914635	-0.28630419	-0.15200309
Aradu.24I8J	-0.4150375	-0.73696559	-0.49410907	-0.49410907	1.15704371
Aradu.P4HVI	0.137503524	0.084064265	0.604071324	0.575312331	2.176322773
Aradu.6AZ0N	-5.64385619	-4.05889369	-4.05889369	-5.05889369	-4.64385619
Aradu.KL3C7	-0.34007544	0.163498732	-0.59946207	1.310340121	1.15704371
Aradu.A1BA5	2.773996325	2.815575429	2.759155834	2.885574364	3.211012193
Aradu.0D9UK	1.744161096	2.266036894	2.173127433	2.232660757	2.572889668
Aradu.A0MU	3.155425432	2.372952098	1.859969548	3.267535798	3.150559677

0					
Aradu.BE5FD	NA	NA	NA	NA	NA
Aradu.4AW6R	1.280956314	2.046141782	1.761285273	1.765534746	2.114367025
Aradu.G63Y1	-1.39592868	-1.83650127	-3.32192809	0.948600847	1.169925001
Aradu.EG1H0	3.787641414	4.308885057	4.029452886	0.137503524	-0.01449957
Aradu.CCT0A	-1.59946207	-3.64385619	-2.73696559	-3.83650127	-3.32192809
Aradu.45CR1	-3.64385619	-2.83650127	-4.05889369	0.378511623	0.485426827
Aradu.K3GZ3	-1.88896869	-1.35845397	-1.94341647	-0.04394335	0.014355293
Aradu.GL7L0	0.807354922	1.704871964	1.03562391	2.283921772	2.521050737
Aradu.LQC6W	3.64731451	3.568032105	3.346247774	3.721372659	4.23878686
Aradu.RUK3P	1.298658316	1.117695043	1.169925001	1.201633861	1.316145742
Aradu.3F83L	3.012568674	2.813524689	2.448900951	2.495695163	2.292781749
Aradu.20IMG	2.40053793	2.56315813	2.286881148	2.367371066	3.032100843
Aradu.54URP	NA	NA	NA	NA	NA
Aradu.AT3ZW	1.883620816	2.301587647	2.192194165	2.94673086	3.119356177
Aradu.356M1	0.443606651	-0.12029423	0.604071324	-0.91593574	0.622930351
Aradu.XDM9G	NA	-5.05889369	-6.64385619	-2.39592868	-1.28630419
Aradu.F0ADU	0.23878686	0.839959587	0.669026766	1.718087584	1.839959587
Aradu.EW29C	-0.4150375	-0.04394335	0.189033824	0.22650853	0.773996325
Aradu.V0UIC	0.575312331	1.361768359	1.056583528	1.40599236	1.257010618
Aradu.B6T0L	3.689299161	4.163498732	3.806324057	4.040892431	4.34553831
Aradu.UQA28	2.35614381	1.86393845	1.843983844	0.887525271	2.220329955
Aradu.VW94	1.739848103	1.367371066	1.608809243	-4.32192809	-2.25153877

6					
Aradu.C4MHC	-2.73696559	-2.83650127	-3.64385619	1.310340121	1.124328135
Aradu.V9PTU	-0.64385619	0.807354922	0.056583528	1.432959407	1.709290636
Aradu.WUP1N	0.475084883	1.778208576	0.641546029	3.012568674	2.99095486
Aradu.R7CTG	-4.32192809	-4.64385619	-3.32192809	-0.68965988	-1.25153877
Aradu.AVD8P	2.693765712	2.952333566	2.669026766	3.994579724	4.495695163
Aradu.EGN24	-2.32192809	-1.83650127	-2.25153877	-2.73696559	-1.43440282
Aradu.J9D9F	-1.78587519	-0.43440282	-0.43440282	-2	-0.32192809
Aradu.ADH1A	5.195741293	4.906890596	4.709842019	0.722466024	0.933572638
Aradu.517PS	1.321928095	0.963474124	0.411426246	-0.4150375	-0.39592868
Aradu.1Z7PM	3.368768349	3.430285273	3.828834649	3.033863452	3.22650853
Aradu.GB4U4	-4.05889369	-0.26881676	-2.73696559	-1	-0.91593574
Aradu.INH3Y	0.992768431	1.244887059	1	1.269033146	1.378511623
Aradu.4U7AL	-3.05889369	-5.05889369	-4.32192809	-1	2.726831217
Aradu.EI7Y8	NA	NA	NA	-5.64385619	-6.64385619
Aradu.CC1R3	2.641546029	2.871843649	2.207892852	2.94673086	3.317593505
Aradu.7H178	3.111031312	3.087462841	2.906890596	2.974529312	2.797012978
Aradu.K2DQ9	-1.68965988	-0.15200309	-1.68965988	2.970853654	2.440952198
Aradu.Q8SMK	0.782408565	0.333423734	0.23878686	-2.12029423	-1.05889369
Aradu.UF8GX	2.813524689	3.495695163	3.35614381	2.147306699	2.392317423
Aradu.SKC35	0.150559677	1.304511042	0.389566812	1.967168608	2.295723025
Aradu.5MH0W	3.862947248	3.806324057	3.703765179	5.267161218	5.08491466
Aradu.YPI85	1.214124805	1.718087584	1.713695815	2.545968369	2.691534165

Aradu.EE1XG	2.244887059	2.378511623	2.195347598	2.604071324	2.998195503
Aradu.5GS43	1.773996325	1.996388746	1.678071905	2.757023247	3.257010618
Aradu.9V9BC	-1.55639335	0.097610797	0.23878686	1.480265122	2.066950244
Aradu.353YW	1.669026766	2.211012193	1.948600847	0.695993813	0.918386234
Aradu.A1EFP	1.269033146	1.782408565	1.516015147	2.568032105	2.94673086
Aradu.L542Q	3.310340121	3.605257263	3.544732656	3.910732662	4.357552005
Aradu.UI0XD	-4.32192809	-5.05889369	-5.64385619	-1.39592868	2.253989266
Aradu.BDN4J	-0.43440282	-0.08926734	-0.49410907	-0.49410907	-0.78587519
Aradu.KW0UC	1.416839742	1.422233001	1.570462931	1.176322773	1.815575429
Aradu.N1DAK	1.3950628	2.424922088	2.077242999	2.427606173	2.698218478
Aradu.383XS	2.998195503	4.072963272	3.777156666	3.13422094	3.673556424
Aradu.NR2Z3	0.613531653	1.014355293	0.871843649	1.500802053	1.970853654
Aradu.VE1T0	-0.62148838	0.748461233	1.855989697	-1.15200309	-0.81096618
Aradu.PT2CX	-0.73696559	-0.73696559	-1.25153877	-0.43440282	-0.76121314
Aradu.P9TWB	-4.64385619	-3.47393119	-4.32192809	-1.64385619	-1.73696559
Aradu.E6Z8G	3.017921908	4.178714641	3.527320608	0.941106311	0.389566812
Aradu.X8R94	1.367371066	0.86393845	0.757023247	1.097610797	1.560714954
Aradu.N0VBS	1.09085343	1.339137385	1.070389328	0.201633861	1.084064265
Aradu.QE4AN	4.193771743	3.980939266	3.958842675	2.545968369	3.282439805
Aradu.KUN1X	2.35614381	2.64385619	2.298658316	2.073820233	1.773996325
Aradu.NY5CH	-0.23446525	0.014355293	-0.35845397	-0.97143085	0.495695163
Aradu.CDK6	NA	NA	-3.47393119	NA	NA

C					
Aradu.GR4IB	0.963474124	1.541019153	1.464668267	2.35614381	2.606442228
Aradu.Q5EC8	-1.02914635	-0.73696559	-0.83650127	-1.59946207	-0.4150375
Aradu.UP0S8	-4.64385619	-2.05889369	-1.59946207	-0.81096618	-0.43440282
Aradu.H2K8Y	NA	-6.64385619	NA	-2.25153877	-4.32192809
Aradu.DE4QW	0.275007047	0.887525271	0.757023247	1.941106311	2.292781749
Aradu.YIH2Y	NA	-3.83650127	-4.32192809	-3.83650127	-1.47393119
Aradu.U7UH3	4.961623328	5.544732656	4.851499202	6.565901767	6.592905474
Aradu.640E1	4.700994494	4.689858236	4.56924803	5.515384461	4.808899839
Aradu.2UZ0C	0.739848103	1.411426246	1.117695043	2.124328135	2.080657663
Aradu.25XYL	NA	NA	NA	NA	NA
Aradu.QE1YJ	2.286881148	2.604071324	2.384049807	3.117695043	3.600507645
Aradu.SNT27	2.482848283	3.147306699	2.516015147	4.316869805	4.125155131
Aradu.0HM6U	1.580145484	2	1.815575429	1.618238656	2.321928095
Aradu.2L1WI	1.967168608	2.025028794	2.14404637	1.847996907	2.23572706
Aradu.81RLF	4.169123281	4.231893162	4.073820233	1.794935663	1.98550043
Aradu.90QAV	0.201633861	-0.10469738	1.847996907	1.622930351	1.438292852
Aradu.X0F96	1.269033146	1.361768359	1.169925001	1.292781749	1.50589093
Aradu.TG162	-1.88896869	-1.88896869	-1.25153877	-1.59946207	-3.47393119
Aradu.8UD68	1.327687364	1.682573297	1.275007047	2.137503524	2.247927513
Aradu.VI7D5	-1.28630419	-1.32192809	-1.02914635	-0.25153877	0.250961574
Aradu.J0ZDG	-2.55639335	-1.94341647	-4.32192809	1.189033824	1.367371066
Aradu.431XY	0.097610797	0.495695163	0.176322773	0.298658316	0.411426246

Aradu.PVS6M	-1.35845397	1.761285273	2.14404637	-0.07400058	1.189033824
Aradu.9TL9J	-2.25153877	-0.43440282	-3.64385619	2.3950628	2.704871964
Aradu.LBT1F	1.480265122	3.590961241	3.089159132	0.90303827	1.432959407
Aradu.EZ4BH	3.135863165	3.12763328	2.853995647	2.698218478	3.250961574
Aradu.RLZ61	-2.32192809	-4.32192809	-6.64385619	1.097610797	0.59454855
Aradu.3YH97	NA	-6.64385619	NA	NA	NA
Aradu.I59PP	2.881664619	3.264536431	3.049630768	3.375734539	3.654206378
Aradu.V6UC8	8.536014034	9.442984952	9.304807618	8.940959545	9.143995368
Aradu.CSH6U	1.851998837	2.424922088	1.819668183	3.415488271	3.404630684
Aradu.WM652	-1.68965988	-1.18442457	-2.64385619	0.948600847	1.584962501
Aradu.1A3FB	2.604071324	4.242602506	4.676380255	1.989139007	2.49057013
Aradu.EB16E	3.384049807	4.13422094	3.697106574	5.833143096	6.307064162
Aradu.Z4MY7	-3.05889369	-2.64385619	-3.64385619	0.367371066	0.411426246
Aradu.D3KJ3	NA	-6.64385619	NA	NA	-0.16812276
Aradu.IS6MU	0.5360529	0.970853654	0.687060688	2.229587923	2.204766751
Aradu.VIL5A	NA	NA	NA	NA	NA
Aradu.MA23R	4.760753208	4.488000771	4.288358562	4.203984166	4.559491813
Aradu.IDJ29	-2.12029423	-1.73696559	-1.32192809	-2.83650127	-4.64385619
Aradu.JQY29	2.066950244	2.301587647	2.14404637	2.648465443	2.811471031
Aradu.R7YY4	3.583759754	3.952333566	3.764473551	4.912649865	5.014801602
Aradu.6RC9F	3.844988157	4.545968369	4.66106548	4.152994605	4.159467729
Aradu.U57WW	NA	NA	NA	NA	-5.64385619

Aradu.UJD9G	-5.64385619	NA	-6.64385619	NA	-6.64385619
Aradu.H6MX9	-0.97143085	-0.62148838	-1.32192809	-0.47393119	0.23878686
Aradu.M49FB	-4.64385619	-6.64385619	-4.05889369	-2.83650127	-2.55639335
Aradu.L2APB	3.612352499	4.266786541	3.261530815	5.628481994	5.424250286
Aradu.569XL	2.698218478	2.86393845	2.307428525	3.297191417	3.212569339
Aradu.KDG8K	-1.12029423	-0.78587519	-0.76121314	1.007195501	0.815575429
Aradu.E2DS0	-0.43440282	0.704871964	0.263034406	2.114367025	1.726831217
Aradu.W2MG5	-2.18442457	-0.97143085	-2	0.214124805	0.298658316
Aradu.AI3EU	-0.10469738	1.22650853	0.757023247	-0.13606155	0.516015147
Aradu.U8582	2.087462841	2.124328135	1.970853654	1.757023247	2.229587923
Aradu.9VB1R	-1.12029423	-0.71311885	-1.02914635	0.344828497	-0.53533173
Aradu.3Q7E3	1.09085343	1.286881148	0.839959587	1.778208576	2.077242999
Aradu.X81G8	2.788685711	2.983677695	2.773996325	3.121015401	3.249445341
Aradu.48GKA	3.775050541	4.232660757	3.82374936	4.76394266	5.09085343
Aradu.VF4U3	NA	-3.83650127	-4.32192809	-4.32192809	-1.35845397
Aradu.KU7WP	-4.05889369	-4.05889369	-4.32192809	NA	-5.05889369
Aradu.7PG44	1.35614381	1.996388746	1.613531653	2.875780063	3.073820233
Aradu.FT5QT	-0.02914635	1.996388746	2.257010618	NA	-6.64385619
Aradu.FHW89	NA	-0.16812276	-3.32192809	1.967168608	1.111031312
Aradu.1C8I8	NA	NA	NA	NA	NA
Aradu.16YW2	1.678071905	2.327687364	1.580145484	2.910732662	3.27351589
Aradu.G7CKS	6.784111324	6.397974049	6.184875343	7.269313726	7.5980525
Aradu.5N8I2	-0.81096618	-0.45403163	-0.71311885	1.765534746	2.028569152

Aradu.K80ZI	2.477677328	3.008988783	2.786596362	3.424922088	3.773996325
Aradu.WA3AU	2.780310099	3.241840184	2.99095486	3.414135533	3.74092756
Aradu.Q0H7N	-0.20091269	-0.71311885	-1.43440282	1.150559677	1.070389328
Aradu.EAF6N	NA	NA	NA	NA	NA
Aradu.HD6HL	2.498250868	2.942983598	2.538538164	3.375734539	3.539779192
Aradu.4U54R	2.03562391	1.929790998	1.871843649	2.14404637	1.944858446
Aradu.E516Y	0.722466024	1.220329955	1.201633861	1.691534165	1.929790998
Aradu.9K9AQ	-0.55639335	-0.83650127	-0.68965988	-0.71311885	-0.23446525
Aradu.JVL5D	3.833902077	3.827819025	3.528571319	3.046141782	3.351910961
Aradu.9M8FI	NA	-6.64385619	NA	NA	NA
Aradu.UA4JV	4.700994494	5.364222221	5.185470146	3.720278465	4.014355293
Aradu.EI99W	2.646162657	2.707082992	2.485426827	3.455491621	4.927896454
Aradu.Q14N7	2.195347598	2.543495883	2.283921772	2.805292456	2.657640005
Aradu.0EA4R	NA	-6.64385619	NA	NA	NA
Aradu.IC1L6	NA	NA	-6.64385619	NA	-4.32192809
Aradu.79FBE	0.871843649	1.718087584	1.3950628	2.817623258	3.049630768
Aradu.3C7HF	2.726831217	2.752748591	2.596935142	3.243364426	3.303050085
Aradu.TYG98	-0.25153877	0.526068812	0.214124805	3.257010618	3.270528942
Aradu.ZQ29G	0.22650853	1.757023247	1.933572638	0.263034406	0.454175893
Aradu.05DT4	1.757023247	2.192194165	2.513490746	-0.30400619	1.416839742
Aradu.3ZE3D	2.994579724	3.176322773	3.030336078	3.386810946	3.904002316
Aradu.GUU2	1.321928095	1.891419187	1.50589093	2.275007047	2.250961574

R					
Aradu.HG6NA	2.523561956	2.845991771	2.678071905	3.751677946	3.819668183
Aradu.HVW3A	0.22650853	1.40053793	0.831877241	1.613531653	2.575312331
Aradu.1235N	1.803227036	1.97819563	1.641546029	3.051372102	3.240314329
Aradu.W4RCV	-1.83650127	NA	-1.88896869	-3.05889369	-2.94341647
Aradu.8VP1A	0.815575429	1	0.622930351	2.084064265	2.771885579
Aradu.GHV9J	2.207892852	2.443606651	2.021479727	3.234194723	3.350497247
Aradu.QT6LV	-0.45403163	-0.35845397	-0.91593574	0.23878686	1.432959407
Aradu.DZ5Y1	8.325485359	7.812305682	7.825022364	3.931683057	5.835166157
Aradu.R2YBN	NA	NA	NA	NA	-3.83650127
Aradu.Q7MTE	5.153805336	5.644721547	4.723012396	6.428443926	6.404290064
Aradu.T65KX	4.413458688	5.169524197	4.540399306	6.161484639	6.296273847
Aradu.00CSZ	-1.88896869	-0.83650127	-1.73696559	-0.26881676	0.056583528
Aradu.37C4I	2.601696516	2.742006211	2.304511042	0.855989697	1.713695815
Aradu.CJN7V	-1.08926734	-1.43440282	-1.88896869	-1.94341647	-0.45403163
Aradu.08WSJ	3.08236197	3.443606651	3.699329526	4.891419187	4.911691582
Aradu.5H5FX	-0.20091269	0.042644337	-0.10469738	-2.18442457	-2.47393119
Aradu.S8SZ6	0.963474124	1.339137385	1.23878686	2.046141782	2.324810603
Aradu.2Y43U	-0.39592868	0.070389328	-0.37706965	1.454175893	1.531069493
Aradu.XKU5K	1.827819025	2.15704371	1.992768431	2.657640005	2.440952198
Aradu.XA7IS	NA	-5.64385619	-6.64385619	NA	-5.64385619
Aradu.HHL1H	-3.64385619	-2.94341647	-4.32192809	-1.55639335	-0.71311885
Aradu.4FK3	1.815575429	3.331991778	3.57167681	2.23572706	2.516015147

V					
Aradu.X9KA6	-1.78587519	-2.32192809	-2.55639335	1.459431619	1.189033824
Aradu.H8ZFN	0.970853654	1.137503524	1	0.555816155	0.367371066
Aradu.HAD5C	2.260025656	2.232660757	2.007195501	2.021479727	3.047887329
Aradu.JLN1Z	NA	NA	NA	NA	-6.64385619
Aradu.YZA37	0.545968369	0.895302621	0.687060688	1.014355293	1.411426246
Aradu.J1G6I	-4.05889369	-5.64385619	-6.64385619	-5.05889369	4.98550043
Aradu.ILZ3Q	NA	NA	NA	NA	NA
Aradu.4DA0K	3.988230223	3.589763487	3.933572638	2.341985747	2.555816155
Aradu.2P1NS	-0.04394335	1.195347598	0.871843649	-1.88896869	-1.08926734
Aradu.S2VPA	2.748461233	3.549669171	3.100977648	4.370164281	4.428276414
Aradu.I6JFH	-0.64385619	-0.15200309	-0.62148838	1.169925001	1.704871964
Aradu.N9CVY	3.340562269	4.073820233	3.289834465	5.355439197	5.142821844
Aradu.1CY96	1.432959407	1.691534165	1.298658316	2.987320866	3.046141782
Aradu.01B4C	NA	NA	NA	-1.94341647	-1.32192809
Aradu.HU8I6	3.117695043	3.508428653	3.507160349	3.851998837	4.085764554
Aradu.VI8FD	-5.05889369	-3.83650127	-4.05889369	-3.83650127	-4.05889369
Aradu.1LG94	-0.18442457	0.189033824	-0.10469738	2.35614381	2.709290636
Aradu.F360A	NA	-3.18442457	-5.05889369	-4.32192809	-1.12029423
Aradu.18DC6	4.613531653	3.980939266	3.795974694	4.658782734	4.7382274
Aradu.T7EMW	1.257010618	1.372952098	0.992768431	1.182692298	1.40599236
Aradu.K4GSH	NA	NA	NA	NA	NA
Aradu.H7GIV	NA	NA	NA	NA	NA

Aradu.5R9LY	1.070389328	1.298658316	0.722466024	1.782408565	2.028569152
Aradu.GB1JK	-1.08926734	-1.43440282	-1.83650127	-0.94341647	-0.55639335
Aradu.5926B	-2.12029423	-2.18442457	-1.59946207	-1.73696559	-1
Aradu.YDZ7F	1.531069493	1.726831217	1.839959587	1.855989697	3.12763328
Aradu.K1JIV	1.526068812	2.056583528	1.438292852	4.98458935	4.39848719
Aradu.K4APN	2.545968369	4.46727948	4.817623258	2.121015401	2.451540833
Aradu.DK67P	3.116031993	3.297191417	3.070389328	2.03562391	2.319039816
Aradu.HE28Y	-0.4150375	1.847996907	1.773996325	-0.02914635	0.298658316
Aradu.62BQ5	-0.91593574	-0.94341647	-0.91593574	-2.94341647	-2.32192809
Aradu.S0307	1.077242999	1.250961574	0.948600847	1.269033146	1.03562391
Aradu.XR3QS	0.565597176	0.807354922	0.659924558	1.416839742	1.851998837
Aradu.M5I54	1.063502942	1.350497247	0.678071905	2.100977648	2.40053793
Aradu.BYZ1A	4.831877241	4.975446766	4.94204526	0.757023247	1
Aradu.M1XBW	2.121015401	2.682573297	2.257010618	3.337711092	3.66448284
Aradu.AH9XG	1.257010618	1.500802053	1.275007047	1.682573297	1.933572638
Aradu.L7120	1.344828497	1.748461233	1.500802053	2.558267634	2.577730931
Aradu.X8MF9	-1.15200309	0.23878686	-0.01449957	0.50589093	0.98550043
Aradu.81WTS	1.531069493	1.959770155	1.443606651	1.839959587	1.748461233
Aradu.CG4KR	1.765534746	2.017921908	1.432959407	2.22342255	3.09592442
Aradu.94X53	1.09085343	1.327687364	0.956056652	2.801158656	2.700439718
Aradu.PZ18V	1.244887059	2.056583528	1.372952098	3.286881148	3.349082146
Aradu.C665I	0.214124805	0.910732662	0.575312331	1.875780063	1.807354922

Aradu.K14NU	3.217230716	4.388878339	3.13422094	5.968781974	5.949301468
Aradu.6R624	1.097610797	1.350497247	0.650764559	2.572889668	3.001802243
Aradu.SBS1U	1.713695815	2.861955364	2.790772038	2.336283388	2.214124805
Aradu.J1AYY	8.110300596	8.374430938	8.035293987	9.100846277	8.953323449
Aradu.VT5GA	NA	-6.64385619	NA	NA	NA
Aradu.CDA12	NA	NA	-5.05889369	NA	-5.64385619
Aradu.B2Z42	-0.13606155	-0.05889369	-1.43440282	3.554588852	3.871843649
Aradu.W912S	0.970853654	1.632268215	1.201633861	2.881664619	3.005399988
Aradu.ERC3E	2.097610797	2.503348735	2.250961574	3.314696526	3.642701572
Aradu.CZ3TW	1.070389328	1.304511042	1.350497247	1.731183242	1.944858446
Aradu.TF48T	1.443606651	1.250961574	1.063502942	2.627606838	2.684818738
Aradu.64C9K	2.060047384	0.839959587	0.495695163	0.275007047	0.748461233
Aradu.BB4JP	2.198494154	2.416839742	3.363171077	1.604071324	1.925999419
Aradu.DN87S	-1.88896869	-0.81096618	-2.18442457	0.150559677	0.056583528
Aradu.1Z30Z	1.879705766	2.111031312	1.10433666	NA	-4.32192809
Aradu.4HQ1D	7.697801615	7.277054876	7.881786959	3.94016675	4.140778656
Aradu.78ZXW	-1.35845397	-0.91593574	-1.35845397	-0.76121314	0.22650853
Aradu.42UET	3.168321116	3.019701914	2.821710215	3.087462841	3.807354922
Aradu.08REY	4.638653116	4.739848103	4.833396134	-1.08926734	-0.01449957
Aradu.4RZ6U	-0.71311885	-1	-0.86249648	0.042644337	1.117695043
Aradu.R4H3N	1.835924074	2.503348735	2.111031312	2.813524689	2.937344392
Aradu.Z4RIW	3.743084056	4.124328135	3.804260116	0.731183242	1.700439718

Aradu.I0JQ8	3.83491343	3.890446693	3.64038956	4.366671916	4.495055528
Aradu.10VKJ	0.678071905	0.669026766	0.871843649	1.608809243	1.372952098
Aradu.CXJ5P	5.04220656	4.727920455	4.808385051	1.214124805	1.613531653
Aradu.2E34B	1.50589093	1.682573297	1.195347598	1.97819563	2.381283373
Aradu.36SKU	2.485426827	2.689299161	2.097610797	2.533563348	2.372952098
Aradu.TR2FS	1.673556424	1.726831217	1.361768359	1.773996325	1.847996907
Aradu.JBU5E	5.007195501	5.323730338	5.150965784	2.922197848	3.198494154
Aradu.YMC7K	-5.64385619	-3.05889369	-6.64385619	0.855989697	-0.47393119
Aradu.XRA1G	3.510961919	3.699329526	3.353323291	4.63284983	4.777682717
Aradu.F0FER	-1.32192809	0.5360529	0.310340121	3.200064862	2.381283373
Aradu.82P95	1.937344392	2.289834465	1.794935663	3.037382222	3.469885976
Aradu.4KD1L	0.722466024	0.378511623	0.50589093	-0.66657627	-0.18442457
Aradu.40JMZ	-1.15200309	-0.86249648	0.839959587	-5.64385619	-2.47393119
Aradu.X1DZR	3.62058641	3.842978832	3.695993813	4.262282806	4.540399306
Aradu.RGQ53	4.125155131	3.974529312	4.055716264	2.867896464	2.933572638
Aradu.ZP7JG	-2.18442457	-1.59946207	-1.78587519	0.084064265	-0.86249648
Aradu.KDH0U	1.541019153	1.22650853	1.526068812	1.427606173	1.778208576
Aradu.GXJ7L	3.703765179	4.296457407	3.831877241	5.460414939	6.314515271
Aradu.VJ2JF	-0.21759144	0.495695163	0.432959407	1.454175893	1.575312331
Aradu.CZI9K	0.925999419	1.220329955	0.678071905	0.344828497	1.275007047
Aradu.C3IHK	NA	NA	-5.64385619	NA	NA
Aradu.D15YQ	3.292781749	3.665620164	3.593353771	3.240314329	4.016139703

Aradu.JR8JR	2.375734539	2.722466024	2.548436625	2.687060688	2.769771739
Aradu.74IXL	0.90303827	1.22650853	0.704871964	0.632268215	1.922197848
Aradu.N90WJ	1.189033824	1.269033146	0.887525271	2.403267722	2.392317423
Aradu.6Z2TN	1.316145742	1.722466024	1.361768359	3.099295204	3.451540833
Aradu.66UC2	4.384049807	4.647890091	4.128458384	2.17951105	2.653060017
Aradu.FPU47	0.378511623	-0.78587519	-0.68965988	4.928844037	5.100136671
Aradu.1N9W3	4.828834649	5.110613806	4.790250739	5.56924803	5.579240501
Aradu.BLP4A	-0.01449957	0.176322773	1.070389328	0.084064265	0.925999419
Aradu.X37CM	3.155425432	3.389566812	3.513490746	3.56315813	3.539779192
Aradu.F7E59	4.670727054	5.472162804	4.803743669	2.639232163	3.201633861
Aradu.JT6Z2	5.224194664	5.210622644	4.964860664	5.537606691	5.785550552
Aradu.B0BG6	-2.47393119	1.560714954	0.378511623	-0.59946207	0.176322773
Aradu.JH5V3	1.22650853	1.550900665	1.182692298	1.137503524	1.778208576
Aradu.M2JBM	-0.66657627	-0.53533173	-0.94341647	-0.32192809	-0.55639335
Aradu.7Q9WK	2.176322773	1.944858446	1.731183242	3.13422094	2.488000771
Aradu.X7VPA	2.370164281	3.295723025	2.304511042	4.257010618	4.0721058
Aradu.I79F7	7.156032499	7.182791599	7.087462841	3.631104282	3.838951767
Aradu.RA9YT	4.487357715	4.676380255	4.486070744	4.861459166	5.174725988
Aradu.NYD5R	3.800123353	3.442280035	3.442280035	0.613531653	0.650764559
Aradu.SSH0X	-1.55639335	-1.25153877	-2.55639335	0.344828497	0.831877241
Aradu.K119F	1.704871964	1.5360529	1.350497247	2.881664619	2.794935663
Aradu.ANJ8N	0.584962501	1.10433666	0.807354922	1.97819563	2.40053793

Aradu.VXK5T	0.056583528	0.659924558	-0.05889369	4.810957159	4.699329526
Aradu.K34WH	-3.47393119	-1.64385619	-1.83650127	0.275007047	-0.34007544
Aradu.9P5M8	2.780310099	3.0721058	3.001802243	3.163498732	3.201633861
Aradu.R9PSM	1.722466024	1.989139007	1.700439718	2.280956314	2.570462931
Aradu.ZS4C4	-1.08926734	-1.39592868	-1.78587519	1.03562391	0.807354922
Aradu.BT3SQ	1.748461233	2.09423607	2.182692298	3.047887329	3.13422094
Aradu.A3N3V	9.043710863	8.280677989	8.509735424	4.749534268	4.850999395
Aradu.747VP	1.13093087	1.50589093	1.469885976	0.584962501	1.275007047
Aradu.N3TMI	2.744161096	3.698218478	3.267535798	3.878725341	4.030336078
Aradu.6Q0CD	-1.64385619	-2.32192809	-2.94341647	-1.59946207	-1.51457317
Aradu.V3ATA	NA	NA	NA	1.541019153	1.906890596
Aradu.4VZ9K	3.111031312	3.263034406	3.249445341	3.326249701	3.648465443
Aradu.39CB1	0.895302621	0.367371066	0.056583528	1.86393845	1.464668267
Aradu.M9FCY	1.411426246	3.896271849	3.439623138	2.316145742	2.937344392
Aradu.49WR7	-2.73696559	-1.64385619	-1.21759144	0.948600847	1.070389328
Aradu.E1M4X	2.675815931	2.392317423	2.060047384	0.90303827	0.963474124
Aradu.R0HWP	2.792855352	2.763411574	2.545968369	3.762348816	3.958842675
Aradu.KNX76	3.351910961	4.128458384	3.981852653	5.02103548	4.914086097
Aradu.BJ3DD	2.107687869	2.137503524	1.859969548	2.62058641	2.867896464
Aradu.29A27	-4.05889369	-2	-2.64385619	-4.05889369	-2.94341647
Aradu.3K9KE	-1.05889369	-3.32192809	-3.64385619	0.815575429	-1.47393119
Aradu.3LW1Z	-0.26881676	0.250961574	0.070389328	1.63691458	2.347665656

Aradu.BWE80	0.263034406	0.613531653	-0.04394335	1.007195501	0.887525271
Aradu.E1B1W	2.269033146	2.066950244	2.160274831	1.280956314	1.63691458
Aradu.V019K	-0.02914635	0.5360529	0.286881148	1.608809243	1.819668183
Aradu.20BZ5	2.286881148	2.488000771	2.381283373	3.192194165	3.557042415
Aradu.YH2KM	-0.08926734	0.214124805	0.704871964	-0.04394335	0.695993813
Aradu.PD37S	1.5360529	1.464668267	1.66448284	0.111031312	1.042644337
Aradu.X66TP	0.575312331	0.704871964	0.389566812	-0.23446525	-0.94341647
Aradu.M01CD	5.050936965	5.618532334	4.686500527	6.509854162	6.27668276
Aradu.DV9UJ	-2.25153877	-2.55639335	-2.64385619	-0.86249648	-1.88896869
Aradu.0ZG2X	-5.64385619	-6.64385619	NA	0.678071905	0.35614381
Aradu.B5WG1	-5.64385619	-5.64385619	-4.05889369	0.613531653	1.687060688
Aradu.7T62P	NA	NA	NA	NA	-3.18442457
Aradu.ZWN25	0.925999419	0.070389328	0.411426246	-1.02914635	-1.12029423
Aradu.QUJ7R	0.659924558	0.790772038	0.176322773	1.344828497	2.003602237
Aradu.M38D2	NA	-6.64385619	NA	-6.64385619	-3.64385619
Aradu.X3NJ9	NA	NA	NA	NA	NA
Aradu.5BX1F	-1.73696559	-1.12029423	-2	0.97819563	1.207892852
Aradu.EQ8WP	-3.18442457	-1.78587519	-2.94341647	-0.83650127	-0.83650127
Aradu.UFI9S	NA	NA	NA	NA	NA
Aradu.0514U	NA	-6.64385619	-5.05889369	0.98550043	2.073820233
Aradu.70NX8	2.90303827	3.292781749	3.112700133	2.965322548	3.388189537
Aradu.N5BC9	2.459431619	2.782408565	2.715893371	2.659924558	2.859969548

Aradu.33XI8	3.139142019	3.528571319	3.317593505	4.13422094	4.526068812
Aradu.H42AE	0.454175893	0.687060688	0.632268215	0.422233001	0.90303827
Aradu.235VA	-1.78587519	-2.32192809	-2.32192809	-4.64385619	-0.62148838
Aradu.3Y7QG	NA	NA	NA	NA	-3.32192809
Aradu.S7Q9G	-1.47393119	-0.15200309	-1.51457317	1.275007047	1.350497247
Aradu.NJI26	0.014355293	0.713695815	0.584962501	1.669026766	1.432959407
Aradu.8QI3B	2.169925001	2.488000771	2.389566812	2.204766751	2.381283373
Aradu.VDW04	0.659924558	1.007195501	-0.49410907	-5.64385619	-3.83650127
Aradu.PG3K0	1.40053793	2.655351829	2.422233001	1.580145484	2.028569152
Aradu.J4VEH	2.526068812	2.257010618	2.12763328	1.575312331	1.731183242
Aradu.DF397	-1.39592868	-0.73696559	-1.55639335	3.075532631	1.815575429
Aradu.YEP9N	NA	NA	NA	NA	NA
Aradu.D8H5F	2.397802962	2.704871964	2.709290636	3.044394119	3.711494907
Aradu.F5B1R	-6.64385619	NA	NA	-5.64385619	5.781097381
Aradu.CH84G	-4.05889369	-3.47393119	-3.47393119	-3.47393119	-3.05889369
Aradu.74DEV	2.277984747	2.424922088	2.23878686	3.201633861	3.408711861
Aradu.32X0N	2.601696516	2.596935142	1.989139007	0.214124805	2.220329955
Aradu.0EG1Y	-0.23446525	-0.20091269	-0.73696559	2.333423734	2.657640005
Aradu.T5NFD	5.228433921	5.856736756	5.155020578	6.712458217	6.411934625
Aradu.JJY9M	2.82374936	3.040892431	2.996388746	3.382667253	3.939226578
Aradu.MYL9N	0.454175893	0.948600847	0.815575429	3.430285273	3.364572432
Aradu.C2HZQ	1.843983844	2.799087306	2.319039816	3.266036894	3.463360886

Aradu.I73XP	-5.64385619	-5.05889369	-5.64385619	-2.39592868	2.09423607
Aradu.9Z02R	0.495695163	0.565597176	0.22650853	2.432959407	2.475084883
Aradu.44JZ9	0	0.687060688	-0.25153877	3.731183242	3.71259578
Aradu.U8VVP	0.678071905	0.748461233	1.097610797	1.996388746	2.260025656
Aradu.K3YJZ	-5.64385619	-4.64385619	-5.64385619	-2.39592868	-2.73696559
Aradu.N9KWN	3.310340121	3.334854269	3.564378169	4.003602237	4.642123916
Aradu.3XT84	2.344828497	2.996388746	2.731183242	3.882643049	3.443606651
Aradu.GI6UB	4.117695043	4.117695043	3.916476644	4.356848079	4.581953751
Aradu.DV28N	NA	-6.64385619	-3.83650127	NA	-5.64385619
Aradu.7KK7S	1.201633861	1.469885976	1.560714954	2.163498732	2.5360529
Aradu.BR5RX	0.50589093	4.545350645	3.777156666	2.778208576	3.424922088
Aradu.CQ2HY	0.111031312	0.411426246	0.545968369	-0.20091269	0.150559677
Aradu.82ABY	-0.51457317	-1.15200309	-1.51457317	0.042644337	0.056583528
Aradu.4W183	1.608809243	1.580145484	1.608809243	2.543495883	2.857980995
Aradu.ILV24	5.5360529	6.108942569	5.434628228	7.036173613	6.977394419
Aradu.3L3H2	0.378511623	1.111031312	1.811471031	1.028569152	1.344828497
Aradu.F52BW	0.970853654	1.372952098	1.124328135	1.59454855	2.166715445
Aradu.L5WLB	-0.08926734	1.316145742	0.014355293	2.480265122	2.615887074
Aradu.JY9SV	2.070389328	2.498250868	2.117695043	2.981852653	3.099295204
Aradu.543MU	0.526068812	-0.45403163	-0.71311885	-0.07400058	0.454175893
Aradu.8DJ4R	1.773996325	2.739848103	1.480265122	0.5360529	1.432959407
Aradu.AZ535	NA	NA	NA	NA	NA

Aradu.WWA28	0.150559677	0.5360529	0.333423734	0.485426827	0.613531653
Aradu.HY0D1	1.599317794	1.956056652	1.794935663	2.414135533	3.03562391
Aradu.6CL4K	2.618238656	2.809414444	2.472487771	3.023255352	3.298658316
Aradu.HN39D	1.687060688	2.292781749	1.97819563	2.17951105	2.358958826
Aradu.GI0SK	-3.47393119	-2.83650127	-3.18442457	-5.05889369	-3.83650127
Aradu.9841E	0.98550043	1.22650853	1.169925001	1.803227036	1.682573297
Aradu.L5P09	-2	-1.78587519	-2.05889369	-1.73696559	-1.51457317
Aradu.G4VT9	-1.21759144	-0.81096618	-0.83650127	-0.04394335	0.298658316
Aradu.U9CZ6	2.568032105	1.992768431	2.794935663	0.59454855	0.669026766
Aradu.0Q16W	5.401903472	5.064796646	4.95373085	-0.51457317	-0.45403163
Aradu.51XM7	NA	NA	NA	NA	-5.05889369
Aradu.R9TCK	-0.88896869	-0.86249648	-1.18442457	-1.05889369	-0.51457317
Aradu.DF4RH	2.286881148	2.575312331	2.316145742	3.465974465	3.66106548
Aradu.JV8RS	-2.55639335	-2.32192809	-1.68965988	-0.66657627	0.163498732
Aradu.7TI6W	1.372952098	1.250961574	1.111031312	2.241840184	2.176322773
Aradu.78VGB	-4.64385619	-4.32192809	-4.05889369	-0.45403163	0.042644337
Aradu.EV8G8	10.0468648	9.211863966	9.713987871	3.936402378	4.280213996
Aradu.F70UL	1.10433666	2.066950244	1.752748591	1.495695163	1.790772038
Aradu.6KR5S	-2	-1.39592868	-1.78587519	0.056583528	-0.51457317
Aradu.NM8U4	-6.64385619	-5.64385619	-4.64385619	-3.18442457	-2.83650127
Aradu.MEC8Q	1.286881148	1.292781749	1.15704371	3.801158656	3.884597921
Aradu.S8Y3J	2.324810603	2.659924558	2.831877241	3.283921772	3.485426827

Aradu.SX6G4	4.909293086	5.572586549	4.717539343	6.647890091	6.389050488
Aradu.THY46	NA	-4.64385619	-5.05889369	0.367371066	-3.32192809
Aradu.VA1N9	2.204766751	2.416839742	2.336283388	2.780310099	3.323370069
Aradu.U5UGY	-2.64385619	-0.51457317	-0.16812276	0.35614381	1.495695163
Aradu.80RUZ	-2.83650127	-0.73696559	-1.28630419	-2.05889369	-2.05889369
Aradu.Z2J2V	1.843983844	1.839959587	1.480265122	2.056583528	2.95419631
Aradu.65NT6	2.272023189	2.553360503	2.416839742	2.250961574	2.695993813
Aradu.7X826	5.689299161	5.95489422	5.906169068	5.191799501	5.202417722
Aradu.4EB0K	1.782408565	2.137503524	1.867896464	2.403267722	2.650764559
Aradu.BR6GB	-2.05889369	-1.28630419	-2.55639335	0.22650853	0.799087306
Aradu.UVG4U	1.765534746	2.010779839	1.618238656	2.166715445	2.207892852
Aradu.CMH1Z	-3.64385619	-4.32192809	-4.64385619	-4.32192809	-3.47393119
Aradu.5PB9J	-5.64385619	-6.64385619	-4.32192809	-2.83650127	-3.05889369
Aradu.MV1EC	-4.32192809	-4.05889369	-4.64385619	-0.37706965	-0.02914635
Aradu.G3XBG	3.001802243	3.297191417	2.839959587	4.375039431	4.530445355
Aradu.A0XEY	-0.15200309	-0.25153877	-0.43440282	0.659924558	1.613531653
Aradu.ZPP4Z	-3.47393119	-1.64385619	-2.47393119	1.316145742	1.350497247
Aradu.716Q8	3.958842675	4.203201156	4.063502942	4.47118746	4.856487779
Aradu.K59XP	0.669026766	0.545968369	0.263034406	-0.59946207	-0.12029423
Aradu.10AS3	2.22650853	2.375734539	2.046141782	3.240314329	3.174725988
Aradu.X89BI	-2.73696559	1.521050737	0.23878686	0.613531653	0.545968369
Aradu.H1YN5	1.077242999	1.790772038	1.66448284	2.23572706	2.695993813

Aradu.T08NC	4.930264245	4.460742564	4.682573297	2.03562391	2.786596362
Aradu.TW1EN	-1.94341647	-0.18442457	-0.62148838	2.737686761	2.229587923
Aradu.X9922	0.475084883	0.584962501	0.411426246	-1.51457317	2.347665656
Aradu.GDX34	-3.83650127	-2.94341647	-2.05889369	-3.18442457	3.780310099
Aradu.F6CTF	1.704871964	1.321928095	1.350497247	1.709290636	1.952333566
Aradu.KLJ74	2.673556424	2.89917563	2.765534746	1.735522177	2.073820233
Aradu.T2X3V	0.632268215	1.257010618	1.257010618	2.10433666	2.942983598
Aradu.02GMF	4.404630684	4.46008724	4.401220862	2.981852653	3.255500733
Aradu.WLH9X	-0.62148838	0.526068812	-0.30400619	2.541019153	2.97819563
Aradu.QT3H3	2.869871406	2.560714954	2.582556003	2.207892852	2.682573297
Aradu.2NY77	1.13093087	1.86393845	1.516015147	2.438292852	2.735522177
Aradu.0N518	2.782408565	2.881664619	2.580145484	4.981852653	4.643278996
Aradu.07VYH	2.871843649	1.996388746	1.929790998	2.416839742	2.771885579
Aradu.WB5Q0	-2.64385619	-2.83650127	-2.73696559	-3.47393119	-2.18442457
Aradu.S4P4Y	2.61117238	2.901108243	2.480265122	3.576522138	4.00270252
Aradu.HQ2V8	2.493134922	2.548436625	2.653060017	3.09592442	3.46727948
Aradu.BWM82	1.526068812	1.244887059	1.989139007	-1	-0.577767
Aradu.A1C01	5.203201156	4.837438715	5.018367116	2.488000771	2.759155834
Aradu.V8MCV	NA	-6.64385619	-5.64385619	NA	-5.05889369
Aradu.VK4A9	-2.83650127	-2.05889369	-2.94341647	-0.02914635	0.084064265
Aradu.ZJ042	2.813524689	3.132576843	3.010779839	3.399171094	3.458119481
Aradu.TC6LS	5.498889087	5.801158656	5.098453246	6.159467729	6.165309016

Aradu.8Q0H7	-2.55639335	-1.51457317	-2.25153877	0.275007047	0.35614381
Aradu.TV4LZ	4.952333566	4.626439137	4.687620632	4.780835002	4.616475329
Aradu.W6TAQ	-1.51457317	0.214124805	-0.86249648	2.060047384	1.682573297
Aradu.LCP0L	-0.43440282	0.042644337	-1.12029423	4.027684877	3.718087584
Aradu.SAY01	NA	NA	NA	NA	NA
Aradu.W1WQU	NA	NA	NA	NA	2.347665656
Aradu.TW3FF	2.702657543	2.121015401	2.333423734	-2.12029423	-0.76121314
Aradu.P9PIK	1.250961574	1.641546029	1.3950628	1.15704371	1.40053793
Aradu.2717A	2.742006211	2.627606838	2.472487771	0.263034406	0.432959407
Aradu.AC0Z7	2.989139007	3.947666157	3.010779839	5.578636862	5.371907298
Aradu.657DW	NA	NA	NA	NA	NA
Aradu.J3B7D	3.40599236	4.717539343	4.973152038	4.221103725	4.499527024
Aradu.R12NH	2.485426827	2.782408565	3.10433666	2.17951105	2.498250868
Aradu.M9QM1	2.754887502	3.026800059	2.592158002	3.638073837	3.56924803
Aradu.Q6WYU	-3.64385619	-4.64385619	-2.18442457	NA	NA
Aradu.LV4I2	4.260778432	4.195347598	4.17951105	4.266036894	4.511594542
Aradu.P0CUQ	5.155020578	5.447909749	5.451870479	3.468583317	3.521050737
Aradu.E32C9	4.0721058	4.025028794	4.271276259	3.310340121	4.217230716
Aradu.71LF7	0.992768431	0.948600847	0.855989697	1.526068812	1.98550043
Aradu.1D15U	-1.55639335	-1.94341647	-1.32192809	-3.83650127	-1.43440282
Aradu.I4E8B	5.606738317	5.508428653	5.469560422	1.807354922	2.277984747
Aradu.B3WTX	-4.32192809	-4.64385619	-6.64385619	-3.32192809	-2.73696559

Aradu.S3MQ8	5.653060017	5.771093252	5.77925972	3.420886575	3.736604875
Aradu.3X53E	NA	NA	NA	-0.86249648	-2
Aradu.NZ3TX	NA	NA	NA	NA	NA
Aradu.VSA8A	1.384049807	3	2.513490746	-1.68965988	0.344828497
Aradu.6Y48C	NA	NA	NA	NA	-4.05889369
Aradu.FI39R	-4.32192809	-5.05889369	NA	-1.73696559	1.014355293
Aradu.I5BSN	-0.20091269	0.422233001	0.722466024	0.879705766	0.713695815
Aradu.Q4TAZ	2.841973119	3.089159132	2.771885579	5.035183997	4.918386234
Aradu.S6JX1	1.867896464	2.3950628	1.887525271	1.521050737	1.339137385
Aradu.4A3GU	2.599317794	2.648465443	2.482848283	1.604071324	1.480265122
Aradu.S7NAJ	1.678071905	2.17951105	1.835924074	2.742006211	3.087462841
Aradu.A050J	-0.35845397	0.604071324	-0.53533173	2.350497247	3.065227623
Aradu.L5Q0P	2.508428653	2.700439718	2.408711861	3.575312331	3.875780063
Aradu.2Q1S0	-4.64385619	NA	-5.05889369	NA	-4.32192809
Aradu.IWK7R	1.604071324	2.195347598	2.111031312	4.873813198	4.831877241
Aradu.UDU6Q	-5.64385619	NA	NA	NA	NA
Aradu.G9EXB	1.416839742	2.060047384	1.443606651	2.873813198	2.675815931
Aradu.H88LD	1.803227036	2.526068812	2.910732662	3.802193217	3.775050541
Aradu.R0WSE	1.646162657	2.039138394	1.655351829	1.963474124	2.462052319
Aradu.ZB2RS	1.469885976	1.794935663	0.485426827	3.207892852	3.733354341
Aradu.IBY74	0.933572638	1.803227036	1.250961574	2.198494154	2.632268215
Aradu.MS2JZ	3.390942773	3.812498225	3.357552005	4.625270489	4.724650272

Aradu.K66VF	2.543495883	2.918386234	2.687060688	2.925999419	3.372952098
Aradu.H5I2N	1.350497247	2.117695043	1.799087306	3.310340121	3.189033824
Aradu.RN1V3	3.047887329	3.261530815	3.122672719	4.117695043	4.267535798
Aradu.N8MU8	NA	NA	NA	NA	-4.64385619
Aradu.GPA6U	0.871843649	0.555816155	0.35614381	2.622930351	3.046141782
Aradu.DR5UL	3.651912745	4.203984166	4.161081482	3.263034406	3.092545742
Aradu.H7VTY	-0.71311885	0.042644337	0.189033824	2.107687869	2.03562391
Aradu.6UQ9U	-0.71311885	-0.30400619	-0.53533173	-0.08926734	0.028569152
Aradu.88298	2.843983844	2.648465443	2.680324357	1.422233001	1.757023247
Aradu.WL13F	-1.18442457	-0.577767	-0.49410907	0.678071905	0.555816155
Aradu.I3T1H	2.639232163	3.063502942	2.817623258	3.632268215	3.498250868
Aradu.ANI3L	0.475084883	-0.21759144	0.176322773	2.09085343	1.970853654
Aradu.F8JPN	NA	NA	-6.64385619	NA	-4.32192809
Aradu.L9SJ9	1.956056652	2.713695815	2.316145742	2.869871406	3.026800059
Aradu.53Y6H	-0.39592868	0.176322773	-0.76121314	1.220329955	1.580145484
Aradu.93TCK	1.361768359	1.182692298	0.933572638	2.807354922	2.726831217
Aradu.Z172U	-0.30400619	0.124328135	-0.47393119	1.555816155	1.526068812
Aradu.8ZT7E	3.893362211	4.475733431	4.072963272	5.453517579	5.570462931
Aradu.S9CWT	-2.05889369	-2.64385619	-2.64385619	2.582556003	2.111031312
Aradu.145AN	-2.64385619	-2.12029423	-2.32192809	-1.88896869	-1.83650127
Aradu.HMN77	NA	NA	NA	NA	NA
Aradu.L46QG	1.344828497	1.280956314	0.879705766	2.204766751	2.097610797

Aradu.QVT4C	-3.18442457	-2.39592868	-3.32192809	-1.05889369	0.124328135
Aradu.1JP2B	1.739848103	2.518535139	2.361768359	3.165107985	3.527320608
Aradu.HU7Y9	0.604071324	1.042644337	0.773996325	1.14404637	1.416839742
Aradu.U5M8T	2.295723025	2.257010618	2.007195501	3.316145742	3.414135533
Aradu.7GP5A	1.49057013	2.182692298	1.182692298	3.241840184	3.158660175
Aradu.SBA69	0.014355293	0.575312331	0.214124805	0.622930351	0.782408565
Aradu.I9U5I	1.773996325	1.941106311	1.63691458	1.731183242	2.039138394
Aradu.JJQ1T	2.066950244	2.307428525	2.241840184	2.053111336	2.752748591
Aradu.BX0IC	0	1.304511042	1.201633861	-1.35845397	-0.20091269
Aradu.I9TTZ	-4.05889369	-3.47393119	-4.05889369	0.731183242	0.298658316
Aradu.9LI83	-3.64385619	-2.47393119	-2.64385619	-1.32192809	-0.28630419
Aradu.KJ041	5.251340383	4.733896605	5.167919866	0.286881148	0.201633861
Aradu.3X77Q	1.495695163	1.752748591	1.361768359	1.773996325	1.906890596
Aradu.10HN0	-2	-2.05889369	-2.73696559	0.097610797	1.443606651
Aradu.F05R2	2.538538164	3.13422094	2.545968369	3.187451054	3.514753498
Aradu.73Q6B	0.713695815	1.063502942	0.526068812	1.220329955	1.541019153
Aradu.XN88F	0.124328135	-0.04394335	0.124328135	1.40053793	1.201633861
Aradu.EVB44	1.618238656	1.280956314	1.150559677	1.280956314	1.695993813
Aradu.SJ887	4.587964989	4.855989697	5.110613806	-1.88896869	-0.12029423
Aradu.MF6JY	2.169925001	2.477677328	2.347665656	2.72900887	2.790772038
Aradu.TE4V4	1.257010618	1.550900665	1.560714954	1.933572638	2.386810946
Aradu.LZR7H	3.784503983	4.117695043	3.909773104	4.544732656	4.64731451

Aradu.ZWA6F	-2.05889369	-2.73696559	-1.88896869	0.150559677	-2.05889369
Aradu.4N3HV	-4.64385619	-4.05889369	-4.05889369	-4.05889369	-3.83650127
Aradu.902BX	1.220329955	2.301587647	2.253989266	2.257010618	2.895302621
Aradu.36NL9	-2.83650127	-2.39592868	-2.25153877	-4.32192809	-2.05889369
Aradu.11KMG	2.321928095	2.341985747	1.803227036	3.622930351	3.810443104
Aradu.XBM41	1.007195501	0.773996325	0.5360529	-0.20091269	1.269033146
Aradu.5CN8X	-4.64385619	-3.83650127	-5.64385619	-4.32192809	-2.32192809
Aradu.1Z587	-1.08926734	-0.91593574	-1	-1.78587519	-0.64385619
Aradu.YIG8H	-0.23446525	0.59454855	0.807354922	-1.73696559	-0.59946207
Aradu.KT4MN	1.757023247	2.241840184	1.937344392	2.333423734	2.381283373
Aradu.M9GL4	0.150559677	2.748461233	3.586164246	1.150559677	2.15704371
Aradu.EV17X	3.890446693	3.599317794	3.513490746	1.859969548	2.5360529
Aradu.E4QK5	1.974529312	2.513490746	2.272023189	2.687060688	2.956056652
Aradu.N3KHF	2.550900665	3.165107985	3.109360559	2.266036894	1.811471031
Aradu.J4IUU	3.874796966	5.812498225	6.865671418	6.321026129	6.100136671
Aradu.F6HJK	1.03562391	1.432959407	1.275007047	1.232660757	1.948600847
Aradu.90RE6	3.181102551	3.617063344	3.50589093	4.098453246	4.167518504
Aradu.7I7HI	0.641546029	1.111031312	-0.55639335	2.438292852	1.835924074
Aradu.XN3YF	1.137503524	1.584962501	1.195347598	1.948600847	2.263034406
Aradu.G2YAZ	0.163498732	0.443606651	0.111031312	2.03562391	2.010779839
Aradu.P8N7B	-1.02914635	-0.88896869	-1.51457317	-0.88896869	-0.76121314
Aradu.63K76	2.64385619	2.477677328	2.408711861	2.077242999	1.827819025

Aradu.69JCH	-1.12029423	-2.73696559	-1.78587519	-0.35845397	-0.35845397
Aradu.6UB5R	-4.32192809	-3.64385619	-3.47393119	-2.73696559	-2.47393119
Aradu.4S63Z	5.583759754	4.9795681	5.0725346	5.144862143	3.731183242
Aradu.BDH44	-1.47393119	-1.21759144	-0.64385619	-2.12029423	-2.55639335
Aradu.K7I1H	2.124328135	1.807354922	1.575312331	1.622930351	1.641546029
Aradu.Q0NF8	3.124328135	3.64731451	3.64731451	5.799605422	5.630521963
Aradu.FR1WN	1.097610797	1.744161096	1.257010618	1.925999419	2.121015401
Aradu.I0IKB	5.110196178	4.911212202	4.974529312	2.253989266	2.518535139
Aradu.78C13	-2.12029423	-1.32192809	-3.83650127	1.077242999	1.811471031
Aradu.U2UEV	2.498250868	2.627606838	2.510961919	3.150559677	3.396433531
Aradu.0KH4T	-0.32192809	-0.15200309	-0.62148838	1.443606651	2.192194165
Aradu.5EP67	NA	-5.64385619	NA	NA	NA
Aradu.772NM	NA	-5.64385619	NA	NA	NA
Aradu.AUX6R	0.910732662	0.454175893	0.378511623	0.815575429	1.599317794
Aradu.50IM9	2.364572432	2.613531653	2.111031312	0.910732662	0.956056652
Aradu.KEC4P	0.86393845	1.565597176	1.137503524	2.053111336	2.073820233
Aradu.RBV6Z	4.157852169	4.131754091	4.038260575	4.493775408	4.752213368
Aradu.V49C9	1.89917563	2.080657663	2.422233001	2.03562391	2.50589093
Aradu.Y1TID	-0.28630419	-0.37706965	0.163498732	1.646162657	1.731183242
Aradu.THV7P	-1.43440282	1.056583528	0.070389328	-1.12029423	-0.04394335
Aradu.115J9	0.650764559	1.077242999	0.956056652	1.111031312	1.049630768
Aradu.H2RVW	0.137503524	0.918386234	1.124328135	2.976363636	3.056583528

Aradu.25DC4	-1.35845397	-1	-1.35845397	0.124328135	0.443606651
Aradu.8I7SF	1.275007047	1.169925001	1.097610797	0.97819563	1.244887059
Aradu.A9U3A	2.680324357	3.502075956	2.94673086	4.738767837	4.306699707
Aradu.6C6EU	0.713695815	0.713695815	0.782408565	0.286881148	0.097610797
Aradu.H5END	1.438292852	2.908812908	2.625270489	4.98230913	5.044394119
Aradu.VRG75	-0.20091269	-0.43440282	-0.97143085	-1.39592868	-0.23446525
Aradu.C0B8A	-0.16812276	-0.577767	-0.30400619	-0.26881676	-0.28630419
Aradu.LF9C0	-0.71311885	-0.02914635	-2.18442457	0.98550043	2.182692298
Aradu.X0Z7E	-0.62148838	0.124328135	0.321928095	1.189033824	1.056583528
Aradu.JT96L	1.163498732	1.867896464	1.214124805	2.799087306	3.040892431
Aradu.EMM34	1.86393845	2.003602237	2.12763328	2.280956314	2.307428525
Aradu.3TT1Y	NA	NA	-5.64385619	NA	NA
Aradu.VX1FR	-0.577767	0.028569152	-1.02914635	2.211012193	2.72900887
Aradu.W4ZB9	4.120186028	4.420212891	4.051372102	4.751677946	4.983221651
Aradu.RZZ9E	-0.49410907	-0.25153877	-0.39592868	0.201633861	0.584962501
Aradu.J5BIS	0.40053793	0.855989697	0.782408565	2.066950244	2.22342255
Aradu.TV03P	0.454175893	0.286881148	-0.28630419	1.14404637	1.298658316
Aradu.Z9DZQ	NA	-3.83650127	-3.83650127	1.384049807	-0.26881676
Aradu.54C3S	NA	NA	NA	NA	NA
Aradu.SK9II	1.367371066	1.748461233	1.700439718	3.196921734	3.089159132
Aradu.97ZLB	-0.23446525	-0.13606155	-0.23446525	0.731183242	-0.47393119
Aradu.D2MQU	0.411426246	1.169925001	0.695993813	2.028569152	2.028569152

Aradu.3N4PQ	0.622930351	0.90303827	1.422233001	2.495695163	2.538538164
Aradu.W3YJN	1.214124805	1.992768431	1.40053793	2.629939409	2.9202933
Aradu.GE4T4	0.604071324	0.097610797	0.214124805	-1.83650127	-0.37706965
Aradu.BF125	-3.64385619	-3.18442457	-4.05889369	-2.83650127	-1.08926734
Aradu.B24NU	2.007195501	2.166715445	1.807354922	2.584962501	2.983677695
Aradu.J7LHV	2.790772038	2.867896464	3.139142019	1.695993813	2.925999419
Aradu.ZE30N	0.367371066	0.014355293	-0.21759144	1.765534746	1.691534165
Aradu.Z34FG	-1.73696559	-1.25153877	-1.94341647	-1.59946207	-1.21759144
Aradu.60C6S	NA	-5.05889369	-5.64385619	-6.64385619	-5.64385619
Aradu.2119N	-1.35845397	-1.55639335	-1.68965988	-2.94341647	-1.02914635
Aradu.UB13R	-2.64385619	-3.47393119	-3.47393119	-0.68965988	0.014355293
Aradu.DC5GT	2.220329955	3	2.310340121	3.845991771	3.980939266
Aradu.NC3JN	1.570462931	1.871843649	1.550900665	2.61117238	2.702657543
Aradu.749Z1	1.815575429	1.613531653	1.575312331	3.046141782	3.292781749
Aradu.L9JI8	2.634593268	3.29130886	2.456806149	2.538538164	2.280956314
Aradu.G1RFE	1.709290636	2.049630768	1.722466024	2.469885976	2.693765712
Aradu.E6FTH	1.117695043	1.815575429	1.678071905	2.160274831	2.553360503
Aradu.1SS2C	3.280956314	3.484138131	3.275007047	4.364572432	4.657640005
Aradu.36VCP	1.713695815	2.851998837	1.914564523	4.589763487	4.531069493
Aradu.J090Y	-0.01449957	0.839959587	0.176322773	1.541019153	1.49057013
Aradu.AKR7M	1.257010618	1.384049807	0.59454855	4.10433666	3.955126781
Aradu.W7MG7	-0.08926734	0.056583528	-0.68965988	-0.25153877	-0.05889369

Aradu.L1VA7	0.485426827	1.967168608	0.757023247	2.516015147	2.678071905
Aradu.35P83	2.568032105	3.264536431	2.875780063	3.49057013	3.701549057
Aradu.3AI2Z	3.09423607	3.169925001	2.9800253	3.185866545	3.653060017
Aradu.NS7I7	-1.08926734	-0.78587519	-1.25153877	0.641546029	0.739848103
Aradu.NJJ5I	3.166715445	3.939226578	4.011674533	4.300123725	3.896271849
Aradu.U033V	2.50589093	3.019701914	2.715893371	2.994579724	3.385431037
Aradu.ZP3W0	-0.23446525	-0.12029423	-0.577767	-1.55639335	-0.91593574
Aradu.KY2F1	-5.64385619	-4.32192809	-3.83650127	0.799087306	1.263034406
Aradu.1I015	3.109360559	3.264536431	2.853995647	2.419538892	2.726831217
Aradu.70QGC	2.929790998	3.049630768	2.97819563	3.117695043	3.486714373
Aradu.EI69K	1.843983844	2.456806149	2.056583528	2.558267634	2.944858446
Aradu.L9VT7	1.5360529	1.485426827	1.459431619	2.956056652	3.331991778
Aradu.DA0G4	NA	-5.05889369	NA	NA	-3.64385619
Aradu.M66BW	5.086189313	5.339850003	5.478971805	4.510329019	4.313245852
Aradu.9SB6V	-0.71311885	-0.64385619	-0.71311885	-0.66657627	-0.10469738
Aradu.5B6UX	1.859969548	2	1.669026766	2.277984747	2.526068812
Aradu.N1NLT	2.737686761	2.867896464	2.695993813	3.759155834	3.549669171
Aradu.H3AX1	4.728464765	4.6622055	4.604071324	4.154615611	4.173127433
Aradu.6V1PP	NA	NA	NA	-4.64385619	-2.94341647
Aradu.X685X	NA	NA	NA	NA	NA
Aradu.B0Q1E	3.519793486	4.128458384	3.89917563	1.811471031	2.403267722
Aradu.YCI2Q	2.443606651	3.092545742	3.010779839	3.94016675	3.781359714

Aradu.F2XPJ	NA	NA	NA	NA	NA
Aradu.J4D1T	0.526068812	1.077242999	0.5360529	2.424922088	3.033863452
Aradu.YE0CC	1.541019153	2.09085343	1.786596362	2.493134922	2.773996325
Aradu.1A2XY	1.879705766	1.952333566	1.709290636	2.3950628	2.580145484
Aradu.ZFA07	-1.83650127	-2.18442457	-1.78587519	-0.62148838	1.021479727
Aradu.919C4	2.50589093	2.799087306	2.49057013	2.666756592	3.078951341
Aradu.82KWN	2.275007047	2.10433666	2.163498732	0.956056652	2.289834465
Aradu.P6GL4	1.361768359	1.111031312	0.790772038	-0.30400619	0.124328135
Aradu.7NY4Q	4.618825953	4.893847558	4.637494325	5.066950244	5.299024807
Aradu.VK93Q	-1.25153877	-0.83650127	-1.51457317	0.263034406	0.59454855
Aradu.YSU0C	NA	NA	NA	-3.64385619	-4.32192809
Aradu.772YU	1.163498732	1.500802053	1.169925001	1.464668267	1.726831217
Aradu.YFR3R	2.879705766	2.513490746	2.767654798	NA	NA
Aradu.TKG0E	0.201633861	0.443606651	-0.43440282	0.070389328	-0.66657627
Aradu.92WMB	2.871843649	3.056583528	2.831877241	4.341274184	4.502712486
Aradu.25NPV	3.869871406	2.97819563	3.353323291	2.386810946	2.839959587
Aradu.AR3UR	7.483815777	6.632413641	7.078844629	4.531693361	5.187451054
Aradu.YH8NY	3.468583317	4.063502942	3.958842675	3.699329526	4.253232939
Aradu.315HW	2.72900887	3.375734539	3.416839742	2.957914599	3.508428653
Aradu.CA6PJ	1.700439718	1.560714954	1.541019153	1.89917563	2.09423607
Aradu.R5E06	3.593353771	5.464994927	4.969472865	5.108524457	4.197708158
Aradu.T1ZRM	NA	-4.05889369	-6.64385619	-3.64385619	-2.94341647
Aradu.2M3L	2.983677695	4.663344619	3.845991771	2.752748591	1.827819025

R

Aradu.UA393	2.475084883	2.003602237	2.283921772	1.584962501	1.948600847
Aradu.DS3R5	3.204766751	3.181102551	3.054848477	2.495695163	2.709290636
Aradu.RW69W	3.320484678	3.021479727	2.976363636	0.782408565	1.769771739
Aradu.N5BSE	0.042644337	0.475084883	0.298658316	3.683696454	3.249445341
Aradu.CJQ5M	1.735522177	2.475084883	1.98550043	3.657640005	3.714795011
Aradu.U20ZR	-0.20091269	-0.08926734	-0.577767	-0.78587519	-1.15200309
Aradu.SR2IB	NA	NA	NA	NA	NA
Aradu.492LT	-2.25153877	-1.12029423	-1.94341647	0.545968369	1.063502942
Aradu.85WAE	2.776103988	2.875780063	2.673556424	3.311793718	3.382667253
Aradu.B3Z15	-0.66657627	-0.18442457	-1.21759144	1.169925001	1.137503524
Aradu.LJJ9P	-4.05889369	NA	-4.32192809	-5.64385619	-6.64385619
Aradu.92CPX	2.555816155	2.698218478	2.691534165	3.187451054	3.64385619
Aradu.S28UE	NA	-6.64385619	NA	NA	NA
Aradu.DCD5A	0.839959587	1.207892852	0.887525271	0.687060688	1.201633861
Aradu.PMG1D	2.518535139	2.95419631	2.599317794	3.247927513	3.524815928
Aradu.T337U	2.709290636	3.310340121	2.948600847	3.422233001	3.49057013
Aradu.DS35R	1.50589093	2.263034406	1.956056652	2.451540833	3.010779839
Aradu.WW65Q	-2.25153877	-1.83650127	-2.12029423	1.786596362	1.992768431
Aradu.B03MY	5.319039816	5.059614856	4.992315252	2.250961574	2.671293372
Aradu.D8U6I	-0.02914635	-0.10469738	-0.577767	2.316145742	2.207892852
Aradu.F5KZV	-3.32192809	-2.64385619	-3.32192809	-1.18442457	-0.55639335
Aradu.7N548	5.682573297	5.387500406	5.360364277	2.895302621	2.669026766

Aradu.FF4D5	0.454175893	0.495695163	0.40053793	4.69265037	4.723558561
Aradu.YPV42	4.171527106	4.507160349	4.406672716	4.941575862	4.972692654
Aradu.9J6N2	1.257010618	1.5360529	1.292781749	1.722466024	1.718087584
Aradu.VR7VT	-0.47393119	0.111031312	-0.20091269	1.687060688	2.070389328
Aradu.Q0XNV	NA	NA	NA	NA	NA
Aradu.LQY7B	2.981852653	3.295723025	2.970853654	3.547203025	3.77925972
Aradu.UHQ4E	4.762348816	5.424586226	4.394376945	6.714245518	6.491692788
Aradu.NNA2S	-1.32192809	-0.35845397	-0.62148838	0.739848103	0.475084883
Aradu.QZ0ZC	-4.32192809	NA	NA	-3.83650127	-3.83650127
Aradu.7HE2F	2.301587647	2.147306699	1.744161096	3.371558863	4.023255352
Aradu.I4P64	NA	NA	NA	-6.64385619	-6.64385619
Aradu.UXI37	2.028569152	2.528571319	2.456806149	2.601696516	2.684818738
Aradu.L1M1M	5.575312331	5.642990313	5.422569411	5.6395216	5.702934532
Aradu.P6GSC	1.344828497	1.618238656	1.570462931	0.731183242	1.835924074
Aradu.AT7BU	-3.32192809	-3.18442457	-5.64385619	-4.32192809	-1.83650127
Aradu.RL2B3	1.765534746	2.370164281	1.831877241	2.028569152	2.321928095
Aradu.W71HY	0.963474124	0.042644337	0.189033824	0.22650853	-0.4150375
Aradu.U6F8I	NA	-4.32192809	-4.32192809	0.855989697	1.344828497
Aradu.WWY9R	-1.55639335	-1.35845397	-2.73696559	0.201633861	0.176322773
Aradu.M5LK1	NA	NA	NA	NA	NA
Aradu.W8MVJ	2.678071905	3.019701914	3.046141782	4.08236197	4.192194165
Aradu.SV33	4.623515741	4.42693562	4.061776198	3.021479727	3.078951341

Z					
Aradu.K7STL	NA	NA	NA	NA	NA
Aradu.EH857	1.40053793	1.469885976	1.333423734	1.15704371	0.887525271
Aradu.V2BW0	1.641546029	1.843983844	1.678071905	3.311793718	3.564378169
Aradu.0LC5Q	6.871104374	6.546122759	6.541483864	3.09085343	3.037382222
Aradu.G229R	4.032982417	4.195347598	4.071247819	4.87282876	5.263785614
Aradu.T2QMK	2.198494154	2.367371066	2.09423607	3.906890596	3.787641414
Aradu.P759U	-3.18442457	-3.47393119	-3.32192809	-5.64385619	-2.73696559
Aradu.Q4Y5D	NA	NA	NA	NA	NA
Aradu.X7WYU	2.625270489	2.893362211	2.678071905	2.313245852	2.855989697
Aradu.NVM07	0.565597176	1.137503524	0.344828497	1.042644337	1.82374936
Aradu.PUZ2B	0.695993813	1.427606173	0.871843649	2.316145742	2.49057013
Aradu.A5HRL	6.191207302	6.197904697	5.960233672	5.358255587	6.147713722
Aradu.763L8	0.411426246	1.028569152	0.40053793	1.014355293	1.077242999
Aradu.VV430	0.650764559	0.250961574	-0.43440282	0.111031312	1.275007047
Aradu.73E3B	2.63691458	3.148934105	2.757023247	4.202417722	4.357552005
Aradu.7HR5N	-3.32192809	-1.94341647	-2.18442457	-1.94341647	0.042644337
Aradu.KK6CI	-1.78587519	-2.39592868	-2.64385619	-3.32192809	-1.35845397
Aradu.UDE5G	-1.35845397	-1.18442457	-1.28630419	1.650764559	1.815575429
Aradu.DJ22Q	1.056583528	1.269033146	-0.30400619	3.195347598	4.006298024
Aradu.5M8KA	0.82374936	0.887525271	1.220329955	-0.21759144	-0.01449957
Aradu.BE884	1.867896464	2.114367025	1.782408565	1.63691458	1.475084883
Aradu.89624	4.365972428	4.601696516	4.397802962	4.414812061	4.521678952

Aradu.TY4XK	4.241840184	4.649040566	4.277984747	5.842727469	5.836429131
Aradu.X7855	1.464668267	1.580145484	1.521050737	2.12763328	3.531069493
Aradu.U8M4U	0.59454855	1.049630768	0.98550043	-0.45403163	0
Aradu.75PY6	-0.01449957	0.35614381	-0.91593574	3.253989266	2.891419187
Aradu.XDC7C	3.739848103	2.195347598	3.124328135	-3.64385619	0.565597176
Aradu.IV1W2	NA	NA	NA	NA	NA
Aradu.TEF34	-3.18442457	-1.21759144	-4.05889369	2.961623328	3.176322773
Aradu.9SR9S	0.056583528	0.887525271	0.40053793	3.102658131	3.432959407
Aradu.I1DRB	2.570462931	3.434294618	2.773996325	3.818646083	3.695993813
Aradu.R0KJF	NA	-4.05889369	-3.64385619	-0.10469738	0.411426246
Aradu.D8WVG	1.831877241	2.516015147	2.327687364	2.709290636	2.9800253
Aradu.JS0R2	3.039138394	2.472487771	2.364572432	2.867896464	2.548436625
Aradu.C6S8Z	0.847996907	1.604071324	1.021479727	-1.15200309	-0.04394335
Aradu.IVB9H	NA	NA	NA	NA	NA
Aradu.YVF29	NA	-5.64385619	-5.05889369	-5.05889369	-4.64385619
Aradu.FF6JW	2.117695043	2.6622055	2.464668267	4.486070744	4.369466484
Aradu.A1XBQ	2.682573297	2.778208576	2.771885579	3.915520901	4.119356177
Aradu.VA5ME	0.97819563	0.641546029	0.669026766	1.495695163	1.86393845
Aradu.Q71BR	3.716990894	4.029452886	4.116863758	2.488000771	2.981852653
Aradu.EP425	3.243364426	3.800123353	3.426264755	4.549053029	4.523561956
Aradu.27Y2W	-3.64385619	-2.64385619	-3.83650127	-5.05889369	-2.32192809
Aradu.8S04V	0.137503524	0.485426827	0.214124805	1.275007047	1.673556424

Aradu.L9M2B	1.687060688	2.277984747	2.003602237	3.450221496	3.097610797
Aradu.R8RVW	3.619413011	3.414135533	3.29130886	4.874305166	4.953265239
Aradu.D0XFS	-4.32192809	-5.05889369	-4.32192809	0.124328135	1.687060688
Aradu.CZQ8X	NA	NA	NA	NA	NA
Aradu.ILC9H	-1.94341647	-1.88896869	-2.32192809	-4.32192809	-0.05889369
Aradu.K05BK	2.114367025	2.198494154	1.469885976	3.827819025	3.663344619
Aradu.2448N	1.214124805	1.827819025	1.275007047	2.283921772	2.613531653
Aradu.Z0GNU	4.556429415	5.118110506	4.845490051	5.618238656	6.136683578
Aradu.8D6KF	-1.51457317	-0.83650127	-2	1.310340121	1.555816155
Aradu.YTQ46	3.791814071	3.681449265	4.151371776	1.09085343	1.028569152
Aradu.IJ1L1	0.799087306	1.22650853	1.03562391	1.952333566	2.214124805
Aradu.GY08R	3.549669171	3.657640005	3.552131108	3.82374936	4.262282806
Aradu.NE5BN	3.451540833	3.092545742	3.473786912	3.068670811	3.19061486
Aradu.N0P3W	-0.53533173	0.097610797	-1.68965988	2.386810946	2.869871406
Aradu.0M8VI	0.948600847	1.304511042	0.748461233	2.430285273	2.451540833
Aradu.XP644	1.811471031	1.879705766	1.618238656	2.722466024	2.99095486
Aradu.8I3T0	4.454175893	3.833902077	4.604664415	3.472487771	2.959770155
Aradu.DRN6A	-4.64385619	-3.18442457	-4.05889369	-1.02914635	0.042644337
Aradu.P3VBU	0.464668267	1.765534746	1.070389328	4.542877099	4.258518925
Aradu.BX447	3.375734539	2.895302621	2.765534746	0.98550043	1.286881148
Aradu.52DEQ	1.786596362	2.604071324	2.568032105	1.432959407	3.039138394
Aradu.32ZU	-1.15200309	-1.18442457	-1.55639335	1.367371066	0.98550043

X					
Aradu.L2YDJ	1.748461233	1.922197848	1.560714954	2.220329955	2.347665656
Aradu.7399Q	1.618238656	1.575312331	1.918386234	1.584962501	2.316145742
Aradu.561J2	NA	-5.05889369	-5.05889369	-6.64385619	-4.64385619
Aradu.Z8A7E	1.565597176	2.053111336	1.867896464	2.827819025	3.158660175
Aradu.ZJ194	1.3950628	1.22650853	1.189033824	3.247927513	3.679198571
Aradu.L0S59	0.150559677	0.150559677	-0.18442457	-1.94341647	-0.02914635
Aradu.CH837	-0.01449957	-0.62148838	-1.12029423	1.89917563	1.604071324
Aradu.IB4JW	1.895302621	2.347665656	2.220329955	2.788685711	2.972692654
Aradu.XN81L	0.5360529	1.298658316	0.575312331	2.974529312	3.03562391
Aradu.DU7VI	-5.64385619	-3.05889369	-3.32192809	3.198494154	3.165107985
Aradu.8QE54	-4.32192809	NA	NA	-3.83650127	1.887525271
Aradu.FF3VB	3.411426246	3.228049048	3.275007047	2.15704371	3.053111336
Aradu.Q84QR	2.304511042	2.948600847	2.589763487	2.803227036	3.153805336
Aradu.WW2SY	-2	-1.51457317	-3.18442457	1.03562391	1.550900665
Aradu.K5W59	3.150559677	3.575312331	3.073820233	3.807354922	4.14404637
Aradu.55LZT	NA	NA	NA	NA	NA
Aradu.56PIZ	NA	NA	NA	NA	-6.64385619
Aradu.2H1TE	0.275007047	0.22650853	0.432959407	-1.43440282	0.084064265
Aradu.QQ3BK	2.963474124	2.702657543	2.440952198	2.137503524	1.691534165
Aradu.DH9GD	1.244887059	1.163498732	0.925999419	2.124328135	2.060047384
Aradu.L8EYA	2.974529312	3.547203025	2.95419631	3.94016675	4.318316841
Aradu.PU45	2.914564523	0.5360529	0.565597176	NA	NA

6					
Aradu.BB920	NA	NA	NA	NA	-6.64385619
Aradu.X3XXG	0.831877241	0.565597176	0.575312331	-0.12029423	0.613531653
Aradu.D0L18	4.250203656	4.456806149	4.465321514	4.554588852	4.705425039
Aradu.IE711	-2.12029423	-3.32192809	-1.88896869	-2.12029423	11.12818125
Aradu.K0SUT	-4.32192809	-2.73696559	-4.05889369	-1.25153877	0
Aradu.T2BI1	0.344828497	0.90303827	-0.10469738	2.189033824	2.107687869
Aradu.A9U89	4.658211483	4.770829046	4.596935142	2.541019153	2.684818738
Aradu.9X2TD	1.560714954	2.007195501	1.66448284	2.608809243	2.739848103
Aradu.Q1VWH	2.885574364	3.012568674	3.124328135	1.718087584	2.087462841
Aradu.P2KV0	3.94673086	4.106013238	3.911691582	5.700439718	5.699607154
Aradu.RCS61	5.013908845	5.300855871	5.078951341	6.198297695	6.186857067
Aradu.T4PBI	NA	NA	NA	-4.64385619	-2.32192809
Aradu.TS004	-3.47393119	-5.05889369	NA	-5.05889369	-3.83650127
Aradu.YQ9M6	2.875780063	2.711494907	2.548436625	1.748461233	2.40599236
Aradu.Z0G82	2.275007047	2.432959407	3.475084883	0.464668267	1.13093087
Aradu.GT860	-0.88896869	-1.12029423	-1.47393119	-0.86249648	-0.83650127
Aradu.XGK7G	NA	NA	NA	NA	NA
Aradu.IW9VR	5.829088444	5.568640195	5.625270489	2.070389328	1.839959587
Aradu.VKJ07	NA	-6.64385619	NA	-5.05889369	-5.64385619
Aradu.16P0Z	-0.15200309	-0.25153877	-0.91593574	1.028569152	2.056583528
Aradu.L3NZR	1.5360529	2.244887059	1.859969548	2.695993813	2.925999419
Aradu.K85GV	NA	NA	NA	NA	NA

Aradu.PJ07U	NA	NA	-6.64385619	NA	3.472487771
Aradu.N62XI	3.007195501	3.349082146	3.125981654	4.118525849	4.122672719
Aradu.5QH0B	NA	NA	NA	NA	NA
Aradu.78HGD	NA	NA	NA	NA	-2.64385619
Aradu.K087N	2.416839742	2.361768359	2.244887059	0.632268215	-0.68965988
Aradu.USH95	NA	-6.64385619	-4.05889369	2.220329955	3.054848477
Aradu.72VHJ	0.084064265	-0.21759144	-0.88896869	-0.71311885	-0.25153877
Aradu.Z156R	-0.12029423	0.23878686	-0.55639335	3.411426246	3.455491621
Aradu.1M0CF	NA	NA	NA	NA	NA
Aradu.K0BKL	NA	-3.47393119	-3.47393119	-4.05889369	-2.73696559
Aradu.94T7Q	-0.83650127	0.389566812	-0.88896869	1.704871964	1.761285273
Aradu.F2VIG	6.708187236	6.936873462	6.953032377	3.973611276	5.55059289
Aradu.LTN41	3.63343121	3.841973119	3.503348735	3.768713657	4.200064862
Aradu.G9QEY	0.028569152	0.5360529	0.411426246	1.097610797	1.056583528
Aradu.W2UK2	-2	-1.68965988	-1.59946207	-2.32192809	-1.73696559
Aradu.YIV0C	1.007195501	1.292781749	0.941106311	2.550900665	2.533563348
Aradu.TF4C3	4.7322692	4.974529312	4.092545742	3.365972428	3.22650853
Aradu.TM2EL	0.82374936	0.604071324	0.575312331	1.82374936	2.370164281
Aradu.D464G	1.500802053	2.367371066	1.500802053	2.655351829	2.788685711
Aradu.ZZ215	3.513490746	3.512226887	3.565597176	3.552131108	3.961623328
Aradu.VS07W	1.560714954	1.042644337	0.731183242	-0.23446525	0.111031312
Aradu.W3GLH	-1.12029423	-1.25153877	-1.43440282	-1.68965988	-0.71311885

Aradu.X9SEL	3.608809243	4.745237332	5.171927354	5.824767853	5.512226887
Aradu.D43UE	1.704871964	2.392317423	2.169925001	2.941106311	2.773996325
Aradu.C7AWI	NA	-6.64385619	-6.64385619	-4.64385619	-2.55639335
Aradu.VJ0SY	-0.16812276	-0.47393119	-0.78587519	1.350497247	2.503348735
Aradu.6T4IB	NA	-6.64385619	NA	NA	NA
Aradu.FX47V	0.565597176	0.250961574	1.150559677	5.648465443	5.607626221
Aradu.17ZI4	4.323370069	4.915998852	5.119356177	4.540399306	4.521050737
Aradu.YB7BD	0	1.14404637	-0.78587519	3.211012193	3.935459748
Aradu.J296J	NA	NA	NA	NA	NA
Aradu.D69CU	1.803227036	1.761285273	1.786596362	1.14404637	1.883620816
Aradu.4V2X0	-2.25153877	-3.83650127	NA	-3.83650127	2.153805336
Aradu.UM9AF	4.631686366	4.073820233	4.541019153	0.678071905	1.367371066
Aradu.3V1LI	6.204375511	6.065227623	6.364747506	2.121015401	1.704871964
Aradu.ZF53H	1.839959587	2.107687869	2.386810946	-2.25153877	-1.08926734
Aradu.A3HX3	1.66448284	2.080657663	1.709290636	2.100977648	2.411426246
Aradu.9CU19	NA	NA	NA	NA	NA
Aradu.PYG3I	1.454175893	1.182692298	1.028569152	1.855989697	2.111031312
Aradu.1G4QF	2.687060688	3.361768359	2.987320866	4.418189948	4.360364277
Aradu.SPH1K	-6.64385619	NA	-6.64385619	-5.64385619	-3.18442457
Aradu.QZD25	-3.32192809	-2.47393119	-2.47393119	1.084064265	-0.32192809
Aradu.5QJ48	-1.78587519	-1.59946207	-1.83650127	-0.88896869	0.464668267
Aradu.SG94C	NA	-5.64385619	-3.18442457	NA	NA

Aradu.IL9UG	-2	-1.78587519	-2.47393119	-0.45403163	0.464668267
Aradu.VB99V	NA	NA	NA	NA	-4.05889369
Aradu.50AY2	-1.05889369	-1.47393119	-1.12029423	-3.18442457	-1.51457317
Aradu.2R2WH	-0.55639335	0.150559677	-0.97143085	2.23572706	2.14404637
Aradu.53A4D	NA	-6.64385619	NA	-5.05889369	-1.94341647
Aradu.NEL01	1.5360529	1.782408565	1.40599236	2.260025656	2.742006211
Aradu.ZIF6R	2.032100843	3.775050541	3.587364991	5.490890979	4.607626221
Aradu.K65B5	4.352617299	4.631686366	4.653633311	3.699329526	3.684818738
Aradu.LT37C	-3.18442457	-1.94341647	-1.88896869	-2.94341647	-2.83650127
Aradu.S5UQ2	-1.08926734	-0.45403163	-2.18442457	1.963474124	2.430285273
Aradu.S63UK	1.584962501	1.941106311	1.769771739	2.742006211	2.959770155
Aradu.C5CLY	1.815575429	2.301587647	2.032100843	2.871843649	3.442280035
Aradu.X9DDA	0.344828497	0.275007047	-0.53533173	2.813524689	3.114367025
Aradu.WJ5JK	2.10433666	1.778208576	1.778208576	-1.73696559	-0.35845397
Aradu.Z753H	3.452858965	4.194559886	4.045268215	2.9800253	3.468583317
Aradu.7A2KY	3.241840184	4.017921908	4.015247774	4.429615964	4.374343989
Aradu.JF030	-3.47393119	-1.83650127	-3.05889369	-1	-0.71311885
Aradu.4ER97	-0.66657627	0.084064265	-0.47393119	2.22342255	2.080657663
Aradu.C6EHZ	1.948600847	2.111031312	1.310340121	2.589763487	3.087462841
Aradu.B4US9	NA	-6.64385619	NA	-3.05889369	-4.05889369
Aradu.E91AT	0.056583528	0.731183242	-0.32192809	1.49057013	1.15704371
Aradu.G3TTV	0.389566812	0.695993813	0.565597176	0.925999419	0.948600847

Aradu.SD45B	3.744161096	4.059182199	3.804260116	3.200064862	3.171527106
Aradu.Q85V2	-1	-0.88896869	-1.32192809	0	0.584962501
Aradu.Y328N	-4.32192809	-1.94341647	-2.32192809	1.847996907	1.575312331
Aradu.2K04Y	-0.28630419	4.20867332	2.367371066	3.060047384	2.851998837
Aradu.M0G9T	NA	-6.64385619	-6.64385619	-1.21759144	-2
Aradu.9H4V1	2.23878686	2.754887502	2.432959407	2.869871406	3.212569339
Aradu.DB4LT	0.042644337	-0.30400619	-0.32192809	0.22650853	0
Aradu.73JAV	4.8928767	5.226893814	5.142004916	5.100977648	4.83491343
Aradu.M2I9A	0.40053793	-1.39592868	-0.81096618	-0.08926734	3.010779839
Aradu.61A42	-0.81096618	-1.55639335	-1.55639335	NA	-5.05889369
Aradu.IAV7G	-0.47393119	-0.23446525	-0.76121314	5.485748821	5.142413438
Aradu.V2Z0U	3.267535798	3.509695842	3.777156666	2.887525271	4.213347282
Aradu.0UC7C	-0.20091269	0.516015147	0.298658316	0.485426827	0.739848103
Aradu.99F66	-5.05889369	NA	NA	-1.21759144	-4.32192809
Aradu.EV762	7.827882522	7.808642468	7.845803646	6.857732233	7.103182881
Aradu.KU05P	2.260025656	2.615887074	2.430285273	3.116031993	3.368768349
Aradu.HEE23	4.499527024	4.156234798	4.486714373	0.739848103	1.82374936
Aradu.WE6GV	3.918386234	4.21490191	4.114367025	4.745775149	4.776630423
Aradu.53H6W	NA	-5.64385619	-6.64385619	NA	-1.05889369
Aradu.3E4UZ	NA	NA	NA	NA	NA
Aradu.LH1JG	0.475084883	0.739848103	0.970853654	-0.18442457	-0.20091269
Aradu.UK4BE	3.321928095	3.080657663	3.044394119	1.937344392	2.508428653

Aradu.MBF39	NA	-5.64385619	-6.64385619	-6.64385619	-2.32192809
Aradu.Z0DB5	NA	NA	NA	-0.43440282	-1.64385619
Aradu.N7YFC	0.333423734	1.049630768	0.622930351	1.722466024	1.765534746
Aradu.T8L1U	2.23878686	2.260025656	2.073820233	2.839959587	3.051372102
Aradu.LXL9C	NA	NA	NA	NA	NA
Aradu.ZFX80	1.176322773	1.049630768	1.150559677	2.553360503	2.726831217
Aradu.X51AF	NA	NA	NA	NA	-0.21759144
Aradu.XA005	4.295723025	4.017921908	3.735522177	4.928844037	4.865918815
Aradu.K7TJG	2.9202933	2.669026766	2.639232163	2.097610797	2.014355293
Aradu.K7Z0H	NA	-6.64385619	NA	NA	1.718087584
Aradu.6P19N	-1.32192809	-1.47393119	-1.64385619	-0.12029423	0.176322773
Aradu.68XYR	-1.08926734	-0.91593574	-2	2.189033824	2.039138394
Aradu.32A1K	2.970853654	2.646162657	2.831877241	-0.81096618	0.641546029
Aradu.BTB7U	2.440952198	2.411426246	1.97819563	2.843983844	2.97819563
Aradu.1EH7R	1.411426246	2.541019153	1.914564523	2.604071324	2.464668267
Aradu.H4IQD	1.427606173	1.056583528	1.056583528	1.014355293	1.516015147
Aradu.UY019	0.773996325	1.389566812	1.201633861	1.531069493	1.82374936
Aradu.802R8	-1.78587519	-1.68965988	-2	-1.73696559	0.50589093
Aradu.9S3Z5	1.244887059	0.956056652	1.257010618	-3.47393119	-1.94341647
Aradu.4M2H4	1.691534165	2.220329955	1.867896464	2.543495883	2.987320866
Aradu.B4YLF	2.867896464	2.553360503	2.98550043	1.655351829	2.750606505
Aradu.B0BA3	-1.64385619	-0.53533173	-0.577767	1.207892852	0.879705766

Aradu.VS9VK	NA	NA	-6.64385619	NA	NA
Aradu.TXN9A	-4.64385619	-3.83650127	-3.83650127	-5.05889369	-1.43440282
Aradu.ZF1LK	-0.62148838	0.070389328	-0.32192809	4.371558863	3.963474124
Aradu.G3CH2	-0.43440282	-0.12029423	-0.55639335	0.097610797	-0.01449957
Aradu.6MW2E	1.632268215	2.825785627	2.207892852	2.372952098	3.124328135
Aradu.MIZ18	-1.78587519	-1.25153877	-1.08926734	-3.18442457	-2
Aradu.EH8VM	3.570462931	2.650764559	2.922197848	2.46727948	3.298658316
Aradu.UI9VM	NA	-6.64385619	NA	NA	2.056583528
Aradu.FX5WG	-1.64385619	-1.73696559	-1.68965988	-2	-1.39592868
Aradu.U9A7G	3.835924074	3.935459748	4.023255352	1.411426246	1.875780063
Aradu.CPH08	3.09423607	3.370164281	3.23878686	4.370164281	5.046578367
Aradu.XD7VB	3.589763487	3.720278465	3.969933275	7.064473329	8.82314429
Aradu.4ME26	NA	NA	NA	NA	NA
Aradu.6X9I9	NA	-6.64385619	NA	-4.64385619	-4.64385619
Aradu.KZ2JX	3.364572432	3.687060688	3.548436625	3.914564523	4.277984747
Aradu.QA9KZ	2.82374936	3.182692298	3.026800059	3.092545742	3.830863757
Aradu.55H8J	0.5360529	1.150559677	0.757023247	1.304511042	1.321928095
Aradu.U70TW	1.86393845	2.137503524	2.03562391	2.613531653	3.025028794
Aradu.D89KQ	3.802193217	3.815575429	3.617063344	3.204766751	3.533563348
Aradu.9E473	2.682573297	2.944858446	2.682573297	3.089159132	3.465974465
Aradu.GJ1CE	-1.21759144	-1.68965988	-1.59946207	-2.55639335	-1.35845397
Aradu.90WZG	1.443606651	1.933572638	1.871843649	1.097610797	0.799087306

Aradu.DLS5S	2.805292456	3.03562391	2.744161096	4.403949364	4.720278465
Aradu.QU7BE	3.005399988	3.316145742	3.121015401	3.365972428	3.826802684
Aradu.W8SNB	-0.34007544	0.263034406	0.310340121	0.124328135	0.82374936
Aradu.WGG5U	4.75595577	5.022367813	4.911691582	5.564073256	5.616475329
Aradu.D51RM	1.757023247	2.147306699	1.632268215	2.025028794	2.153805336
Aradu.172F0	2.010779839	2.35614381	2.107687869	2.185866545	2.469885976
Aradu.G8W4L	0.40053793	0.475084883	-0.28630419	1.263034406	0.831877241
Aradu.0MS0A	0.687060688	0.722466024	0.659924558	0.807354922	1.049630768
Aradu.XR3BY	2.56315813	2.378511623	2.650764559	1.580145484	2.424922088
Aradu.1QV2U	1.650764559	2.538538164	2.358958826	2.327687364	2.077242999
Aradu.H44CN	1.718087584	1.650764559	1.550900665	-0.34007544	0.070389328
Aradu.ES65V	3.544732656	3.771885579	3.23878686	4.084064265	4.329841177
Aradu.J80PY	1.510961919	1.244887059	1.14404637	1.03562391	0.084064265
Aradu.CXC2W	-0.59946207	0.286881148	-0.37706965	0.333423734	0.545968369
Aradu.4Z2QP	0.545968369	0.855989697	0.871843649	1.201633861	1.778208576
Aradu.CK902	0.516015147	0.124328135	1.650764559	1.970853654	2.9800253
Aradu.WZ6G2	3.185866545	3.868884273	2.994579724	4.826802684	4.840463234
Aradu.D7PPK	2.472487771	2.983677695	2.627606838	2.419538892	2.389566812
Aradu.V7F48	3.581351247	3.266036894	3.165107985	2.737686761	2.843983844
Aradu.KT81X	2.301587647	2.459431619	2.295723025	3.792855352	3.800123353
Aradu.18BRD	0.070389328	-0.32192809	-0.13606155	0	0.014355293
Aradu.T1ZHN	-4.32192809	-1.88896869	-1.05889369	-3.64385619	-2.94341647

Aradu.PI5HS	0.584962501	0.555816155	0.82374936	-1.39592868	-1.83650127
Aradu.76EJ2	-3.05889369	-2.94341647	-4.05889369	2.277984747	2.307428525
Aradu.L86SE	2.263034406	2.448900951	2.933572638	1.97819563	2.849999259
Aradu.4Q608	1.339137385	1.778208576	1.275007047	1.735522177	2.23572706
Aradu.9V11P	-0.66657627	2.59454855	1.50589093	1.459431619	1.97819563
Aradu.ZT0IB	-2.55639335	-2.39592868	-3.83650127	-1.39592868	-0.28630419
Aradu.NND2F	5.516645558	6.180903709	5.459103696	7.48123435	6.993447932
Aradu.A1KE0	3.13422094	2.726831217	2.733354341	1.748461233	2.908812908
Aradu.MEV99	3.482848283	3.148934105	3.282439805	2.510961919	3.275007047
Aradu.S14WF	-3.64385619	-2.94341647	-3.05889369	-1.12029423	-0.10469738
Aradu.035RQ	4.647890091	4.591559746	4.286141873	4.957450336	4.891419187
Aradu.Y5WI6	-0.73696559	-0.20091269	-0.68965988	0.604071324	0.895302621
Aradu.IH0N2	-0.91593574	-0.577767	-2.18442457	1.739848103	2.495695163
Aradu.J5692	2.748461233	2.049630768	2.364572432	-1.02914635	0.214124805
Aradu.KVD8P	1.344828497	1.843983844	1.550900665	2.704871964	2.845991771
Aradu.CAH9K	4.50589093	4.448240225	4.48864354	2.482848283	2.839959587
Aradu.5P7M7	-4.64385619	0.097610797	0.555816155	-4.64385619	0.782408565
Aradu.4M8RF	3.198494154	3.893362211	3.516015147	5.121429909	5.714245518
Aradu.7VY22	3.033863452	3.286881148	3.148934105	2.89917563	3.121015401
Aradu.Q0NSJ	1.448900951	1.669026766	1.580145484	0.584962501	1.432959407
Aradu.ZW5X6	0.765534746	0.757023247	0.59454855	-0.81096618	2.277984747
Aradu.P5VH9	0.669026766	1.124328135	0.722466024	2.759155834	3.005399988

Aradu.FA8EI	2.845991771	1.918386234	2.147306699	1.541019153	2.321928095
Aradu.G78T6	-3.64385619	-2.83650127	-3.05889369	0.070389328	0.367371066
Aradu.9U2NA	-4.64385619	-6.64385619	-6.64385619	-1.51457317	-2.47393119
Aradu.GA66V	0.201633861	-1.35845397	-1.64385619	-2.94341647	-1.94341647
Aradu.J72N3	NA	NA	NA	NA	NA
Aradu.81PPC	-6.64385619	-4.32192809	-4.05889369	-6.64385619	-3.18442457
Aradu.CQ85M	1.056583528	2.659924558	2.23878686	-0.577767	-0.25153877
Aradu.CDJ2J	0.59454855	1.843983844	0.839959587	2.731183242	2.803227036
Aradu.CH4M9	5.86393845	5.556429415	5.797272432	1.700439718	1.659924558
Aradu.5A0JT	4.946262984	3.080657663	2.875780063	-0.25153877	0.526068812
Aradu.QX9N9	1.906890596	2.99095486	2.541019153	1.22650853	1.992768431
Aradu.5C5XG	-0.49410907	-0.23446525	-0.4150375	-0.18442457	0.042644337
Aradu.X3U5Y	6.216260837	5.918147674	6.20867332	2.321928095	2.283921772
Aradu.J5WE7	3.112700133	3.300123725	3.078951341	3.434294618	3.787641414
Aradu.Y6LUX	-0.88896869	1.981852653	3.203201156	2.560714954	2.722466024
Aradu.612YH	-2.47393119	-1.83650127	-1.88896869	-2.18442457	0.028569152
Aradu.Z2BLJ	5.552131108	5.382667253	5.265286858	6.150762744	6.726831217
Aradu.UC09A	-2.05889369	-0.55639335	-2.73696559	1.22650853	1.361768359
Aradu.085T6	NA	NA	NA	NA	-6.64385619
Aradu.AA8NU	-3.05889369	-2.18442457	-3.18442457	-1.32192809	-1.05889369
Aradu.VM246	2.500802053	2.867896464	2.543495883	3.71918344	3.97819563
Aradu.0M5LW	0.90303827	1.280956314	1.189033824	1.521050737	2.247927513
Aradu.LH2UI	-1.43440282	-0.12029423	-0.59946207	-0.30400619	0.678071905

Aradu.H447Q	8.25502857	6.675392543	6.133810091	6.234961095	5.408032466
Aradu.WQI5W	1.996388746	2.12763328	1.550900665	2.124328135	2.572889668
Aradu.GP6HQ	NA	NA	NA	-5.64385619	-4.64385619
Aradu.XZS36	1.831877241	2.286881148	1.948600847	2.44625623	2.521050737
Aradu.H7KNI	-5.05889369	-6.64385619	-6.64385619	-5.64385619	-3.83650127
Aradu.TV1GT	1.735522177	2.073820233	1.678071905	2.596935142	2.773996325
Aradu.2Z89F	1.15704371	1.232660757	0.970853654	-0.05889369	0.695993813
Aradu.QVX65	1.831877241	2.17951105	1.803227036	2.244887059	2.307428525
Aradu.YZ2FS	0.650764559	1.028569152	0.422233001	3.042644337	3.247927513
Aradu.J25NN	-1.21759144	-1.35845397	-2.18442457	-3.32192809	-1.08926734
Aradu.A87UA	2.182692298	2.025028794	1.879705766	3.941106311	4.058316496
Aradu.FJ441	2.10433666	1.427606173	1.580145484	1.097610797	1.516015147
Aradu.2U1V2	NA	NA	-6.64385619	NA	NA
Aradu.WH701	0.084064265	0.731183242	0.695993813	1.790772038	2.472487771
Aradu.UFC3R	-2.73696559	-1.35845397	-0.86249648	-1.64385619	-1.18442457
Aradu.M3Q0G	5.163498732	5.543186525	4.656496371	4.99004722	4.639810978
Aradu.ZN7FN	4.433627167	4.161081482	4.475733431	0	0.176322773
Aradu.J7JS6	0.070389328	2.392317423	2.553360503	2.570462931	2.687060688
Aradu.ZS9DU	3.142413438	3.308885057	3	3.586164246	3.700439718
Aradu.FQ6XD	-1.68965988	-1.88896869	-2.39592868	-0.32192809	-1.05889369
Aradu.8025N	2.150559677	2.891419187	2.13422094	3.602884409	3.721372659
Aradu.7Y2H	2.372952098	2.403267722	2.673556424	3.019701914	2.887525271

Q					
Aradu.0SV2V	-0.94341647	-1.18442457	-0.47393119	0.50589093	1.937344392
Aradu.H5ED7	2.056583528	2.737686761	2.189033824	2.908812908	2.994579724
Aradu.Y966U	-1.94341647	-2.12029423	-1.39592868	-4.64385619	1.124328135
Aradu.875VG	-3.47393119	-3.47393119	-4.05889369	-2.32192809	0.137503524
Aradu.M2KYK	2.918386234	2.807354922	2.531069493	2.778208576	2.845991771
Aradu.32DSM	0.659924558	0.713695815	0.432959407	NA	NA
Aradu.I0WQG	-5.64385619	-1.94341647	NA	-1.35845397	-0.53533173
Aradu.B4UNP	-1.02914635	-0.71311885	-1.78587519	2.386810946	2.707082992
Aradu.32QV2	NA	NA	-6.64385619	-5.05889369	-3.47393119
Aradu.T9PKV	3.198494154	3.249445341	3.228049048	2.857980995	2.516015147
Aradu.S2HHA	NA	-6.64385619	-6.64385619	NA	-4.05889369
Aradu.UTB08	-1.39592868	-2.05889369	-2	-2.18442457	-1.25153877
Aradu.Z9YF8	1.464668267	1.937344392	2.014355293	1.769771739	2.021479727
Aradu.A8886	-0.62148838	-0.05889369	-0.25153877	0.163498732	0.918386234
Aradu.4C32F	0.35614381	0.443606651	0.250961574	1.599317794	3.440952198
Aradu.TN7XF	-4.05889369	-2.83650127	-3.64385619	-2.05889369	-3.32192809
Aradu.8PJ2J	0.422233001	1.815575429	1.464668267	-0.66657627	0.028569152
Aradu.0V3TN	NA	NA	NA	NA	-5.64385619
Aradu.PZ5G0	4.260778432	4.509695842	4.100136671	2.59454855	2.384049807
Aradu.W4W1A	2.250961574	2.304511042	2.295723025	2.22650853	2.809414444
Aradu.1HX6J	-1.39592868	-0.37706965	-0.71311885	-2.18442457	-0.64385619
Aradu.RY2K	1.454175893	1.895302621	1.718087584	0.992768431	1.803227036

R					
Aradu.M4TPQ	-2.64385619	-5.64385619	-4.64385619	NA	NA
Aradu.29VJC	1.485426827	1.207892852	0.831877241	1.922197848	2.684818738
Aradu.Q507J	1.713695815	1.970853654	1.891419187	2.17951105	2.438292852
Aradu.3BA5T	NA	-5.64385619	-6.64385619	-3.47393119	-2.32192809
Aradu.HZ16A	0.933572638	0.687060688	0.137503524	2.700439718	2.558267634
Aradu.RWZ7G	0.757023247	1.327687364	0.918386234	1.967168608	2.548436625
Aradu.XA41R	2.100977648	2.204766751	2.389566812	2.575312331	2.784503983
Aradu.I4VKL	0.622930351	1.056583528	0.731183242	0.695993813	0.86393845
Aradu.1R4IH	-3.18442457	-1.78587519	-2.64385619	0.150559677	0.815575429
Aradu.GH8LH	2.053111336	2.286881148	1.963474124	3.054848477	3.375734539
Aradu.W7CVC	2.809414444	3.298658316	3.056583528	3.651912745	4.695993813
Aradu.K6X31	0.378511623	0.263034406	-0.30400619	0.344828497	0.678071905
Aradu.2J9A4	0.565597176	1.541019153	1.40599236	2.084064265	2.448900951
Aradu.A336V	0.704871964	1.15704371	0.879705766	1.443606651	1.333423734
Aradu.42JCX	1.59454855	3.282439805	3.215678597	2.488000771	1.632268215
Aradu.JM9X6	3.517275693	2.851998837	3.204766751	1.510961919	1.713695815
Aradu.43YX5	-2.32192809	-3.05889369	-5.05889369	-0.49410907	-0.25153877
Aradu.EG6FJ	3.019701914	3.560714954	3.379898164	3.830863757	3.972692654
Aradu.W69K1	1.831877241	2.46727948	1.700439718	2.629939409	2.935459748
Aradu.SH6P7	NA	NA	NA	-4.64385619	NA
Aradu.PEH3C	-3.32192809	-4.64385619	-3.18442457	-5.05889369	3.246408087
Aradu.00HG	1.659924558	2.025028794	1.895302621	1.843983844	2.150559677

B					
Aradu.I0YKW	2.042644337	0.90303827	1.049630768	2.301587647	2.885574364
Aradu.AL954	0.887525271	0.86393845	0.275007047	2.472487771	3.09085343
Aradu.46T9V	1.344828497	1.618238656	1.40599236	0.475084883	0.545968369
Aradu.Y5HX4	-2.55639335	-1.18442457	-1.64385619	-1.21759144	-0.64385619
Aradu.P7W5S	-2	-1.25153877	-1.47393119	-3.32192809	-2.32192809
Aradu.SE6Y8	1.86393845	2.469885976	2.283921772	2.927896454	3.261530815
Aradu.AET7G	2.606442228	2.771885579	2.62058641	3.82984956	4.199279721
Aradu.VUX24	NA	-5.64385619	NA	0.367371066	-0.45403163
Aradu.AY7EP	3.87774425	3.777156666	3.574101508	4.171527106	4.436961338
Aradu.0NR0Q	2.759155834	3.135863165	3.028569152	3.715893371	4.033863452
Aradu.JYC3Z	1.709290636	2.389566812	1.843983844	2.722466024	3.0721058
Aradu.SHF2C	1.937344392	2.364572432	1.895302621	3.044394119	3.192194165
Aradu.AS44H	1.5360529	2.378511623	1.956056652	3.379898164	3.489286023
Aradu.I5672	2.344828497	2.50589093	2.350497247	2.150559677	2.803227036
Aradu.VKB5P	5.323370069	5.509695842	5.24830712	2.786596362	2.811471031
Aradu.W801G	3.462052319	3.718087584	3.148934105	4.792855352	5.203201156
Aradu.EAI63	1.570462931	2.028569152	1.669026766	1.956056652	1.895302621
Aradu.V0JK3	-1.88896869	-2	-2.39592868	-2.73696559	-2.94341647
Aradu.CV157	1.485426827	0.516015147	0.443606651	-2.83650127	-1.47393119
Aradu.SBU2D	2.885574364	3.343407822	2.950468414	3.560714954	3.521050737
Aradu.7V6T8	-2.73696559	-1.78587519	-2.25153877	1.618238656	0.855989697
Aradu.BBF6	0.526068812	1.13093087	0.855989697	1.427606173	2.411426246

Q					
Aradu.38JU0	-3.05889369	-5.05889369	-2.94341647	0.815575429	1.827819025
Aradu.CZ7XQ	1.275007047	3.420886575	4.391630262	7.387155718	6.176123271
Aradu.3589Z	-0.64385619	-0.35845397	-1.78587519	2.416839742	2.835924074
Aradu.KD75D	2.137503524	2.087462841	2.176322773	-0.32192809	0.411426246
Aradu.Q566Q	NA	-6.64385619	-6.64385619	NA	-4.05889369
Aradu.X9YRF	2.324810603	3.324810603	2.933572638	3.177917792	3.46727948
Aradu.YFQ3P	1.761285273	4.507160349	4.518535139	0.895302621	1.794935663
Aradu.G957G	-2.39592868	-2.47393119	-2.73696559	0.687060688	1.280956314
Aradu.YVT9V	-5.64385619	-3.32192809	-4.05889369	-0.55639335	-0.35845397
Aradu.0MP9W	-2.25153877	-2.12029423	-2.12029423	-3.64385619	-1.15200309
Aradu.SY6IP	2.462052319	2.910732662	2.538538164	3.56193706	3.601696516
Aradu.K7USP	1.367371066	1.887525271	1.669026766	2.056583528	2.386810946
Aradu.7H9F1	2.192194165	2.280956314	2.250961574	2.513490746	2.87774425
Aradu.27WCK	-1.64385619	-1.08926734	-2.05889369	-0.59946207	-0.23446525
Aradu.Z8VFD	NA	-6.64385619	NA	-0.59946207	-0.32192809
Aradu.RB60U	-1.15200309	-0.30400619	-0.59946207	-0.01449957	1.794935663
Aradu.2V69M	1.361768359	1.769771739	1.627606838	2.316145742	2.853995647
Aradu.B33TG	5.528258743	5.081510068	4.003602237	0.028569152	3.305970521
Aradu.97XZI	0.40053793	-0.23446525	-0.28630419	-1.05889369	0.948600847
Aradu.550LU	3.147306699	3.125981654	3.033863452	3.518535139	4.527946099
Aradu.5A70G	2.140778656	2.482848283	2.13093087	2.533563348	2.825785627
Aradu.E8W2	NA	-5.05889369	-5.64385619	-0.30400619	0.389566812

B					
Aradu.A1GM4	2.935459748	3.003602237	2.89917563	3.289834465	3.854993017
Aradu.87YLX	-0.76121314	-1.18442457	-1.39592868	0.632268215	0.495695163
Aradu.II5C0	4.886550147	5.457134594	4.440952198	6.815191137	6.681730355
Aradu.U79FN	-3.47393119	-2.83650127	-3.32192809	-1.83650127	-0.26881676
Aradu.UPU3E	-0.16812276	0.669026766	0.124328135	1.014355293	2.046141782
Aradu.K2UAZ	2.316145742	2.482848283	2.272023189	3.051372102	3.516015147
Aradu.89I67	1.739848103	1.891419187	1.752748591	2.63691458	3.102658131
Aradu.56PKD	NA	-6.64385619	-6.64385619	-2.83650127	-1.59946207
Aradu.T720W	3.789729251	4.19298317	4.006298024	2.916476644	3.548436625
Aradu.IJ0H9	1.575312331	1.599317794	1.59454855	2.906890596	3.049630768
Aradu.SJX08	-1.78587519	-1.25153877	-2.18442457	0.5360529	0.704871964
Aradu.WN017	4.420886575	4.649040566	4.537917249	4.952799478	4.595742339
Aradu.M2A53	-0.62148838	-1.08926734	-0.91593574	-0.07400058	0.344828497
Aradu.Q9LEZ	3.936402378	3.82374936	3.735522177	2.462052319	2.666756592
Aradu.316F6	4.584361253	5.057450272	4.775050541	5.659639187	5.501120634
Aradu.BX9V6	NA	NA	-6.64385619	-3.05889369	-0.43440282
Aradu.M6IGU	NA	NA	-5.64385619	NA	NA
Aradu.XGI6B	0.748461233	1.304511042	0.879705766	2.333423734	2.592158002
Aradu.N0CP9	3.012568674	2.914564523	2.722466024	3.152183419	3.576522138
Aradu.KC0GC	-1.59946207	-1	-2.12029423	1.646162657	2.070389328
Aradu.573UI	1.941106311	2.275007047	2.042644337	2.646162657	2.9800253
Aradu.Z0E8	-1.94341647	-1.94341647	-1.64385619	-3.18442457	-2.05889369

S					
Aradu.D81FK	2.516015147	3.386810946	2.464668267	3.080657663	3.089159132
Aradu.PRJ6R	6.258141996	5.734438666	6.081297014	-1.39592868	-0.43440282
Aradu.BS70J	-0.62148838	-0.25153877	-0.68965988	0.298658316	1.941106311
Aradu.D1QW8	-0.62148838	0.485426827	0.815575429	1.344828497	0.298658316
Aradu.M9YMG	NA	NA	NA	NA	NA
Aradu.H3P3G	-1.39592868	-1.68965988	-2.32192809	1.339137385	1.304511042
Aradu.4M072	-0.47393119	0.86393845	0.604071324	1.063502942	1.959770155
Aradu.D7KSP	NA	-5.05889369	NA	-2.94341647	-2.94341647
Aradu.7XU9R	0.214124805	-0.62148838	-0.28630419	-2.64385619	-2
Aradu.W903S	-4.05889369	-3.18442457	-1.94341647	-1.18442457	-1
Aradu.RN1PL	2.63691458	2.867896464	2.618238656	4.030336078	4.197708158
Aradu.WU1XZ	1.316145742	2.111031312	1.959770155	2.589763487	2.731183242
Aradu.ME340	-3.83650127	-2.39592868	-3.32192809	-1.64385619	-1.08926734
Aradu.GZ5P7	NA	NA	-6.64385619	-3.64385619	-3.18442457
Aradu.I8Q2P	3.139142019	3.10433666	3.201633861	2.339137385	2.553360503
Aradu.0Y99B	NA	NA	NA	NA	-1.15200309
Aradu.G9UB3	1.550900665	1.014355293	0.678071905	-1.32192809	1.232660757
Aradu.MHM9J	3.286881148	4.622344723	4.058316496	3.828834649	4.10433666
Aradu.YDH5Z	NA	-5.64385619	-5.05889369	-6.64385619	NA
Aradu.0SN1Y	-3.83650127	-5.05889369	-6.64385619	-2.32192809	-1.21759144
Aradu.95B31	2.560714954	2.912649865	2.707082992	3.507160349	3.763411574
Aradu.8Y5V	5.618238656	6.070603999	5.673556424	6.698635221	6.505255801

Q					
Aradu.XYY87	NA	NA	NA	NA	NA
Aradu.QH23N	NA	NA	NA	-3.64385619	-5.05889369
Aradu.QW806	1.263034406	1.752748591	1.599317794	2.097610797	1.967168608
Aradu.H92KN	NA	NA	NA	NA	NA
Aradu.S4DGV	0.150559677	1.560714954	0.731183242	-0.10469738	0.176322773
Aradu.F64Z1	5.155425432	5.162290614	5.290940402	1.803227036	2.596935142
Aradu.65TC6	NA	NA	NA	NA	NA
Aradu.V1PLQ	1.875780063	2.137503524	1.851998837	1.786596362	2.192194165
Aradu.DK86D	6.849248703	7.097821455	6.987775616	3.565597176	3.911691582
Aradu.L7WWU	NA	-2.47393119	-2.73696559	-4.05889369	-5.64385619
Aradu.Y6TV9	NA	-4.32192809	-5.64385619	0.014355293	1.948600847
Aradu.02NM4	-1.73696559	-1.15200309	-2	0.163498732	-0.02914635
Aradu.58Q87	-2.32192809	-2.83650127	-1.88896869	-3.64385619	-2.39592868
Aradu.G43SA	-2.94341647	-2.64385619	-3.64385619	-0.91593574	-0.66657627
Aradu.J1S8L	-5.05889369	-2.73696559	-1.78587519	-3.47393119	-2.18442457
Aradu.J3SWW	3.331991778	3.282439805	2.906890596	4.310340121	4.325530332
Aradu.6H4PP	-2.25153877	-1.55639335	-2.55639335	NA	NA
Aradu.1J3FJ	-1	-0.94341647	-0.81096618	1.077242999	1.589763487
Aradu.I609F	1.021479727	2.821710215	2.472487771	1.641546029	1.214124805
Aradu.EL04J	0.333423734	0.59454855	0.250961574	-0.55639335	0.565597176
Aradu.HW921	2.851998837	3.491853096	3.40053793	3.056583528	3.221877081
Aradu.988BI	0.782408565	1.604071324	1.15704371	3.743084056	3.722466024

Aradu.GIY15	-1.78587519	-1.08926734	-2.05889369	-2.83650127	-1.15200309
Aradu.YSG0U	4.197708158	4.478324711	4.188242656	4.963936452	4.986410935
Aradu.9Y9CU	2.367371066	2.89917563	2.307428525	3.607626221	3.782408565
Aradu.D20L9	3.618238656	4.264536431	3.649615459	4.244887059	4.583759754
Aradu.E70M5	0.555816155	1.09085343	0.782408565	1.765534746	1.859969548
Aradu.8V3QK	-5.64385619	NA	NA	NA	-2.18442457
Aradu.2QX0T	-4.05889369	-2.64385619	-2.39592868	-1.83650127	-2.39592868
Aradu.H05SK	2.211012193	2.435628594	2.339137385	2.3950628	1.883620816
Aradu.U8ZNV	3.663344619	3.529820947	3.310340121	3.604071324	3.416839742
Aradu.V9D23	1.97819563	1.659924558	1.879705766	-0.53533173	0.432959407
Aradu.J1S90	0.422233001	0.871843649	0.613531653	1.084064265	1.438292852
Aradu.2PT1E	1.855989697	1.803227036	1.485426827	1.996388746	2.513490746
Aradu.S7JJM	NA	NA	NA	1.618238656	1.97819563
Aradu.329PQ	-1.83650127	-1.28630419	-1.28630419	2.049630768	1.811471031
Aradu.B7XG0	-2.47393119	-1.25153877	-1.28630419	0.731183242	0.632268215
Aradu.2C8QX	0.925999419	0.650764559	0.475084883	2.3950628	2.987320866
Aradu.QFV3H	-1.18442457	-0.97143085	-1.28630419	-2.47393119	-3.05889369
Aradu.G0DW8	0.799087306	0.422233001	-0.04394335	1.480265122	2.039138394
Aradu.YXZ49	2.176322773	2.655351829	2.577730931	3.032100843	3.693765712
Aradu.ACY83	1.669026766	2.12763328	1.929790998	0.584962501	1.5360529
Aradu.1R7R2	2.263034406	1.831877241	2.10433666	6.263597849	5.894817763
Aradu.S46K	-1.05889369	-0.16812276	-1.94341647	2.550900665	3.19061486

B					
Aradu.W4PG1	-1.35845397	-1.64385619	-2.73696559	-1.83650127	-2.25153877
Aradu.NQ3N9	4.161081482	4.316145742	4.191404729	5.221103725	5.436961338
Aradu.GBM3Q	NA	-6.64385619	NA	NA	-1.05889369
Aradu.DXG5L	2.589763487	2.887525271	2.759155834	2.61117238	2.347665656
Aradu.M4PQ9	2.321928095	2.253989266	2.319039816	1.761285273	2
Aradu.G9DQQ	-1.64385619	-0.07400058	-0.94341647	1.298658316	1.298658316
Aradu.J7CRS	4.475084883	4.284662185	4.177120503	3.308885057	3.323370069
Aradu.E6ID0	1.531069493	1.722466024	1.726831217	1.163498732	1.201633861
Aradu.IR2D4	NA	NA	NA	NA	1.361768359
Aradu.ZDZ6K	NA	NA	NA	NA	NA
Aradu.AX3Z0	-3.18442457	-0.97143085	-0.68965988	-1.02914635	0.263034406
Aradu.G09L8	2.918386234	2.464668267	2.063502942	2.042644337	2.087462841
Aradu.X4G0F	3.200064862	2.82984956	2.950468414	-0.66657627	-0.32192809
Aradu.1Z96C	2.790772038	2.715893371	2.419538892	2.82374936	3.459431619
Aradu.V0CGL	-0.86249648	-0.39592868	-0.43440282	-0.37706965	-0.577767
Aradu.HH10J	1.049630768	0.839959587	0.704871964	1.752748591	3.014355293
Aradu.38TXW	-2.55639335	-4.32192809	NA	-4.64385619	-1.35845397
Aradu.Q27ZD	-0.15200309	0.495695163	1.111031312	1.887525271	2.277984747
Aradu.VW8XJ	-0.49410907	-0.23446525	-0.64385619	0.855989697	0.925999419
Aradu.LJH4E	1.280956314	1.207892852	1.40053793	1.244887059	1.794935663
Aradu.L39ME	1.495695163	1.855989697	1.599317794	2.99095486	3.080657663
Aradu.70V2V	1.550900665	1.97819563	1.565597176	2.13093087	2.555816155

Aradu.XE6VM	NA	NA	NA	-5.64385619	-6.64385619
Aradu.SYI1K	3.063502942	2.918386234	2.803227036	2.414135533	2.875780063
Aradu.A6T5Y	-1.64385619	-0.78587519	-1.94341647	0.485426827	0.526068812
Aradu.X3GS1	-1.32192809	-0.91593574	-1.88896869	2.35614381	2.548436625
Aradu.E4G18	5.304511042	4.805292456	5.024142346	1.989139007	2.344828497
Aradu.31H7A	3.549669171	3.434294618	3.897240426	1.15704371	1.883620816
Aradu.6V4TL	-0.81096618	-0.30400619	-0.81096618	1.967168608	2.13093087
Aradu.VUE49	-0.25153877	0.275007047	-0.04394335	0.970853654	1.207892852
Aradu.WB45H	2.702657543	2.875780063	2.596935142	2.881664619	3.543495883
Aradu.IS9ZH	3.576522138	3.897240426	3.821710215	4.44625623	4.441616269
Aradu.61HSA	0.056583528	0.575312331	0.604071324	1.257010618	1.220329955
Aradu.7Q1J6	2.275007047	2.347665656	2.289834465	1.827819025	2.324810603
Aradu.ZPB6A	9.051181746	9.186782802	9.516468283	4.973611276	4.99004722
Aradu.0YE33	-1.43440282	5.527946099	4.3305584	8.463033855	6.751276249
Aradu.QF91Q	3.66448284	3.736604875	4.139142019	0.641546029	0.925999419
Aradu.XX1YI	1.952333566	2.321928095	2.211012193	3.304511042	3.430285273
Aradu.AL6NG	3.682573297	4.143230135	3.838951767	4.436961338	5.221877081
Aradu.PU1U6	-2.64385619	-3.64385619	-3.32192809	-4.05889369	4.578938713
Aradu.KF8UQ	NA	-2.12029423	-3.32192809	-3.64385619	-3.83650127
Aradu.U68KB	-3.05889369	-2.55639335	-3.47393119	-1.73696559	-0.86249648
Aradu.KJ6HK	5.258895755	5.3305584	5.621465834	2.5360529	3.09423607
Aradu.SYY8	5.140369671	5.204766751	5.287620044	3.008988783	3.124328135

Y					
Aradu.ETD4B	NA	-4.64385619	-6.64385619	-2.83650127	0.59454855
Aradu.49BKS	2.066950244	2.117695043	2.070389328	2.702657543	3.272023189
Aradu.DI7FZ	3.116031993	3.270528942	3.177917792	3.974529312	4.165911939
Aradu.Z9WRN	-1.59946207	-2	-1.88896869	-5.05889369	-1.43440282
Aradu.D2EC5	1.709290636	1.344828497	1.327687364	2.198494154	2.220329955
Aradu.LX505	2.253989266	2.94673086	2.797012978	4.720278465	4.033863452
Aradu.00NYG	1.970853654	1.883620816	1.769771739	2.599317794	3.368768349
Aradu.K6R51	-0.73696559	1.678071905	1.659924558	0.263034406	0.333423734
Aradu.N2D1U	2.319039816	2.232660757	2.207892852	1.310340121	1.887525271
Aradu.L5NS4	3.166715445	3.736604875	3.298658316	4.981852653	4.77452353
Aradu.QM4PI	-1.73696559	-0.12029423	0.189033824	1.163498732	0.59454855
Aradu.B6G6H	0.90303827	1.510961919	1.163498732	2.629939409	2.23572706
Aradu.U7IHD	NA	-3.47393119	-5.64385619	-2.83650127	-4.32192809
Aradu.B9T25	1.580145484	2.176322773	1.963474124	2.752748591	2.961623328
Aradu.MD0ZW	-5.64385619	-5.64385619	NA	-1.94341647	-2.25153877
Aradu.U0T6A	1.794935663	2.147306699	1.744161096	2.358958826	2.739848103
Aradu.VBY1Y	-0.16812276	-0.05889369	-0.577767	2.482848283	1.82374936
Aradu.W1519	-3.05889369	-2.83650127	-4.32192809	-2.18442457	-1.02914635
Aradu.FQ240	0.097610797	0.910732662	-0.4150375	3.592158002	3.246408087
Aradu.N2WYB	3.613531653	3.828834649	3.605257263	4.310340121	4.643278996
Aradu.7VL9B	1.22650853	1.59454855	1.422233001	2.301587647	2.541019153
Aradu.1IK7L	NA	NA	NA	-4.64385619	2.855989697

Aradu.H8Z7K	-5.05889369	NA	NA	NA	NA
Aradu.1QN19	3.910732662	4.454175893	4.414812061	2.392317423	2.361768359
Aradu.X7WGL	0.443606651	0.275007047	0.137503524	-1.64385619	0.344828497
Aradu.RU1MG	-2.47393119	-1.88896869	-1.88896869	2.956056652	3.363171077
Aradu.Z0JID	3.439623138	5.052676724	4.176322773	3.169925001	3.857980995
Aradu.RBQ3X	1.298658316	2.153805336	1.50589093	4.255500733	4.257010618
Aradu.E0Q62	4.026800059	3.566815154	4.017031081	5.118110506	5.333423734
Aradu.CG65D	2.13422094	2.482848283	1.867896464	3.924099886	4.071247819
Aradu.8V764	0.214124805	-0.30400619	-1.83650127	2.560714954	2.397802962
Aradu.K9Z81	-5.64385619	-2.55639335	-5.64385619	-3.64385619	-4.32192809
Aradu.T35QD	-2.94341647	-2.83650127	-5.05889369	-2.18442457	-1.59946207
Aradu.Y32L7	-0.81096618	-0.01449957	-0.64385619	0.963474124	1.070389328
Aradu.W3BNI	2.767654798	3.195347598	3.017921908	2.709290636	2.746312766
Aradu.W8K8D	4.937344392	4.539779192	4.695993813	2.13093087	3.510961919
Aradu.MLY6C	1.974529312	1.459431619	1.286881148	1.773996325	2.411426246
Aradu.DN4RU	NA	-4.32192809	-5.05889369	0.250961574	2
Aradu.U1T2G	-1.21759144	-0.12029423	-0.45403163	0.604071324	1.097610797
Aradu.E43JR	1.82374936	2.056583528	1.731183242	2.618238656	2.648465443
Aradu.52L7X	-2.55639335	-2.55639335	-2.73696559	-1.51457317	-1.59946207
Aradu.TBX7Z	1.117695043	2.726831217	1.811471031	2.014355293	2.584962501
Aradu.XJ6TW	0.555816155	1.021479727	0.378511623	1.659924558	2.021479727
Aradu.8W1T	3.092545742	3.214124805	3.09085343	3.246408087	3.614709844

J					
Aradu.NA3JC	2.392317423	2.40053793	2.454175893	1.786596362	2.275007047
Aradu.V1TEZ	-0.08926734	0.411426246	-0.01449957	1.361768359	1.641546029
Aradu.JU9J9	4.425593578	4.265286858	4.040892431	3.617063344	4.583759754
Aradu.SG97P	NA	NA	-6.64385619	-5.05889369	-2.64385619
Aradu.RAU4M	-1.55639335	-1.39592868	-2.39592868	-0.15200309	-0.51457317
Aradu.058CJ	2.718087584	3.423578171	2.780310099	4.207111961	4.300123725
Aradu.ET296	NA	-6.64385619	-5.64385619	-4.32192809	-1
Aradu.7N2UH	3.545968369	4.690975738	5.289834465	5.806066226	6.065873847
Aradu.NT394	1.98550043	1.871843649	1.970853654	0.90303827	1.304511042
Aradu.XDC1M	3.430285273	3.849999259	3.476381688	4.485426827	4.288358562
Aradu.HC4I0	1.86393845	1.59454855	1.111031312	3.124328135	3.346247774
Aradu.GUV6V	NA	NA	NA	NA	NA
Aradu.Y99HX	2.599317794	1.454175893	1.941106311	-0.28630419	1.169925001
Aradu.7NI41	-0.07400058	-0.88896869	-0.78587519	-2.39592868	-1
Aradu.Y8YHS	3.750606505	3.771885579	3.314696526	3.690417096	3.550900665
Aradu.B5DUD	NA	NA	NA	-3.83650127	3.201633861
Aradu.HZ14S	NA	NA	NA	-3.83650127	-1.73696559
Aradu.M5W9D	2.713695815	2.577730931	2.364572432	3.192194165	3.488000771
Aradu.ADH9Y	2.114367025	2.173127433	2.007195501	0.910732662	1.049630768
Aradu.KU4KX	0.678071905	1.411426246	0.831877241	2.693765712	2.653060017
Aradu.B7QXE	3.187451054	3.703765179	3.152183419	2.23878686	2.680324357
Aradu.EK8M	1.687060688	2.147306699	1.575312331	2.411426246	2.485426827

W					
Aradu.6HJ87	0.704871964	-1	0.918386234	4.892391026	4.671293372
Aradu.A2SS4	2.117695043	2.843983844	2.378511623	3.303050085	3.738767837
Aradu.9MQ2E	-0.49410907	-1.47393119	-1.55639335	0.495695163	1.570462931
Aradu.F9182	-1.68965988	-1.39592868	-1.43440282	-0.39592868	0.464668267
Aradu.3SM7F	2.3305584	2.185866545	1.811471031	3.584962501	3.6622055
Aradu.5N3RR	5.312156888	5.612352499	5.395405605	6.18705509	6.140574178
Aradu.MJZ2W	-2.47393119	-1.78587519	-2.12029423	0.22650853	1.056583528
Aradu.ZZ3IQ	-0.94341647	-0.30400619	-4.64385619	-0.20091269	1.214124805
Aradu.IRG5E	1.944858446	1.871843649	1.257010618	3.253989266	3.193771743
Aradu.N7MUQ	NA	NA	NA	NA	-2.39592868
Aradu.VFE8Z	0.056583528	0.286881148	-0.34007544	2.582556003	2.855989697
Aradu.38M3H	2.805292456	3.13422094	2.669026766	3.684818738	3.827819025
Aradu.3DW3S	0.333423734	0.86393845	0.669026766	3.670160514	3.621758857
Aradu.MN7KS	0.855989697	1.232660757	0.773996325	2.347665656	2.63691458
Aradu.E36HP	0.632268215	0.879705766	0.687060688	-0.64385619	-0.37706965
Aradu.W1X0Q	-2.05889369	-1.78587519	-3.64385619	-0.01449957	0.422233001
Aradu.MD14G	1.056583528	1.485426827	1.007195501	2.657640005	2.459431619
Aradu.1GI8T	NA	-5.64385619	NA	NA	NA
Aradu.G4DCM	3.737686761	3.958842675	3.808385051	4.458119481	4.337711092
Aradu.0SE2F	-1.68965988	-0.78587519	-1.73696559	-0.01449957	0.748461233
Aradu.MI8WP	2.386810946	2.742006211	2.528571319	3.841973119	4.020591095
Aradu.Z1DBI	-2.12029423	-2.25153877	-2.55639335	-1.02914635	-0.35845397

Aradu.LN5YX	3.182692298	3.404630684	2.650764559	5.00270252	4.719731057
Aradu.N24L5	NA	-2.47393119	-2.47393119	NA	-3.05889369
Aradu.JLY8Q	4.46727948	4.750606505	4.361768359	6.664198369	6.638653116
Aradu.8Z92W	0.22650853	1.14404637	-0.15200309	2.454175893	2.797012978
Aradu.Z2SQB	2.9202933	2.948600847	2.430285273	4.603477988	4.576522138
Aradu.JU8EK	-5.05889369	-5.05889369	-6.64385619	-5.64385619	-1.39592868
Aradu.U974Q	-1.83650127	-1.94341647	-1.88896869	-3.18442457	-1.55639335
Aradu.1CN3T	1.752748591	2.545968369	2.260025656	3.313245852	3.3305584
Aradu.N3IWP	NA	NA	NA	NA	-5.05889369
Aradu.U6V8H	0.333423734	2.456806149	1.910732662	1.599317794	1.321928095
Aradu.MS98X	-6.64385619	NA	NA	-6.64385619	-5.64385619
Aradu.JQC6T	2.419538892	2.6622055	2.283921772	2.963474124	3.085764554
Aradu.13QYM	3.435628594	3.341985747	3.135863165	3.243364426	3.075532631
Aradu.GP5WA	3.378511623	3.580145484	3.08236197	2.687060688	3.061776198
Aradu.GKD3R	4.058316496	4.448900951	3.981852653	3.258518925	3.493134922
Aradu.EF798	-6.64385619	-3.18442457	-2.94341647	NA	-3.47393119
Aradu.K84FP	-3.83650127	-3.47393119	-5.64385619	1.189033824	1.416839742
Aradu.N64VX	5.344473459	5.952100554	5.078524445	6.933808662	6.787771993
Aradu.S226I	-1.78587519	-2	-2.94341647	-4.32192809	-2.39592868
Aradu.GBG51	1.250961574	2.150559677	3.545968369	2.049630768	2.682573297
Aradu.9BY1S	1.541019153	1.944858446	1.682573297	3.870857864	4.229587923
Aradu.1F5AZ	4.790250739	3.817623258	5.03562391	-0.53533173	0.855989697

Aradu.SCA2Z	-0.47393119	0.411426246	0.014355293	0.963474124	1.367371066
Aradu.9L2RT	2.737686761	2.871843649	3.073820233	2.759155834	2.887525271
Aradu.MS08A	0.565597176	1.014355293	0.555816155	0.871843649	0.933572638
Aradu.2K3I2	1.163498732	1.704871964	1.280956314	1.622930351	1.855989697
Aradu.YWC0U	0.765534746	1.176322773	0.887525271	2.430285273	2.570462931
Aradu.IF5Y6	-0.18442457	0.22650853	-0.45403163	1.310340121	1.220329955
Aradu.N7TNH	6.170926525	5.638363506	5.757023247	2.153805336	2.056583528
Aradu.XH3MQ	1.321928095	2.310340121	1.650764559	2.056583528	2.163498732
Aradu.7I425	3.689299161	3.451540833	3.148934105	3.672425342	3.622930351
Aradu.HLR71	-4.32192809	-4.05889369	-4.64385619	-5.64385619	-2.73696559
Aradu.3SA2N	6.45795538	6.452200049	6.521364878	3.35614381	4.309612772
Aradu.Z6652	2.572889668	1.555816155	1.918386234	-3.47393119	-2
Aradu.YR0IE	3.153805336	3.570462931	3.411426246	4.3652726	3.929790998
Aradu.PA8BP	-6.64385619	-5.64385619	-5.05889369	-6.64385619	-3.83650127
Aradu.EI543	1.599317794	2.097610797	1.748461233	1.974529312	2.263034406
Aradu.0BJ82	NA	NA	NA	NA	NA
Aradu.ZA2RQ	1.89917563	2.327687364	1.790772038	3.864928972	3.974529312
Aradu.Y478Z	-1.28630419	-0.78587519	-1.43440282	0.310340121	0.495695163
Aradu.MRJ6D	0.799087306	0.871843649	0.765534746	2.257010618	2.548436625
Aradu.NJH70	-5.64385619	-4.64385619	NA	-2.47393119	-2.18442457
Aradu.EJW3I	4.968551603	5.48123435	5.076388069	5.625854931	5.784765696
Aradu.DUE4	4.29130886	3.638073837	4.097610797	1.782408565	3.300123725

8					
Aradu.69W3K	3.346247774	3.416839742	3.117695043	3.109360559	3.416839742
Aradu.R2TG0	-1.39592868	-0.78587519	-1.47393119	0.097610797	0.070389328
Aradu.59NQ7	2.726831217	3.635754391	3.390942773	2.675815931	3.654206378
Aradu.6NR6S	-0.05889369	0.263034406	-0.02914635	0.97819563	1.35614381
Aradu.EEP0U	2.121015401	2.13422094	2.204766751	-3.32192809	-2.12029423
Aradu.0Z09C	-4.64385619	NA	NA	-5.05889369	-3.83650127
Aradu.5M687	-0.15200309	0.321928095	-0.28630419	0.86393845	1.650764559
Aradu.096I4	-0.23446525	0.084064265	-0.39592868	-1.32192809	0.310340121
Aradu.R3U8P	1.819668183	3.12763328	2.742006211	2.759155834	2.615887074
Aradu.Y65N7	0.98550043	1.316145742	0.933572638	2.601696516	3.039138394
Aradu.Q9E1W	-5.64385619	-4.64385619	-6.64385619	NA	-3.18442457
Aradu.YR762	1.464668267	2.5360529	2.582556003	2.14404637	2.358958826
Aradu.D0I1J	1.389566812	1.137503524	1.432959407	2.137503524	2.003602237
Aradu.J2YIY	-5.64385619	-4.05889369	-5.64385619	2.117695043	2.914564523
Aradu.WP30A	NA	NA	NA	NA	NA
Aradu.P7QP3	NA	NA	NA	NA	-2
Aradu.V7W75	1.871843649	2.204766751	1.963474124	1.454175893	1.169925001
Aradu.40X7J	3.646162657	4.13996057	3.560714954	5.551516018	5.687060688
Aradu.D0SQ3	-0.97143085	-2.64385619	-2.73696559	-1.83650127	-2.25153877
Aradu.AX7JB	2.695993813	3.529820947	2.895302621	4.667892125	4.451540833
Aradu.GMU6R	-3.47393119	-2.83650127	-4.05889369	-2.73696559	-1.25153877
Aradu.2FM0	2.987320866	2.63691458	2.62058641	0.495695163	0.641546029

G					
Aradu.U5ZHH	1.709290636	1.480265122	1.895302621	-4.64385619	-1.88896869
Aradu.13E7W	NA	NA	NA	-6.64385619	-5.64385619
Aradu.U75R0	4.34978987	4.832383717	3.996388746	2.735522177	2.9800253
Aradu.Y8Q46	-2.39592868	-1.39592868	-2.32192809	-3.83650127	-3.83650127
Aradu.BPK98	-1.28630419	-1.21759144	-1.51457317	0.389566812	0.111031312
Aradu.212W8	0.757023247	1.700439718	1.021479727	0.839959587	1.555816155
Aradu.ZJ7LY	2.350497247	2.641546029	2.693765712	3.27351589	3.424922088
Aradu.Z194X	1.244887059	1.867896464	1.310340121	1.839959587	2.153805336
Aradu.4Y1KN	4.880195729	5.588264894	5.823494625	4.116863758	3.937344392
Aradu.CV497	0.565597176	1.650764559	0.687060688	2.403267722	2.424922088
Aradu.HVZ6R	2.746312766	2.889473543	2.632268215	4.063502942	4.539158811
Aradu.H88U3	2.364572432	2.901108243	2.477677328	2.82374936	2.910732662
Aradu.5M1Q4	1.480265122	2.147306699	1.90303827	3.119356177	3.365972428
Aradu.7J78G	-0.88896869	-2.12029423	-2	-3.05889369	-3.64385619
Aradu.CM89H	-2.64385619	-2.25153877	-3.32192809	0.275007047	0.807354922
Aradu.62PY8	4.015247774	1.622930351	0.695993813	3.670160514	3.454175893
Aradu.RA8II	5.566206293	5.520422249	4.648465443	3.955126781	3.862947248
Aradu.00U6W	0.632268215	1.891419187	1.575312331	-0.10469738	-0.45403163
Aradu.AEH7T	2.904965719	3.160274831	3.025028794	3.448900951	3.83491343
Aradu.G0S92	-2.94341647	-2.83650127	-5.05889369	-3.18442457	-1.78587519
Aradu.T52PW	-2.32192809	-2.64385619	-3.32192809	-5.64385619	-3.18442457
Aradu.MC6V	4.111865964	4.467931546	3.989139007	4.012568674	4.803743669

Z					
Aradu.X10AK	-1.88896869	-2.47393119	-2.39592868	-4.05889369	-3.83650127
Aradu.41I2U	4.115199749	4.091699834	4.09423607	2.653060017	2.996388746
Aradu.KD1XS	NA	-5.05889369	-5.64385619	-4.32192809	-3.32192809
Aradu.CBT6M	NA	-6.64385619	-5.64385619	NA	-5.64385619
Aradu.XRX70	-2.83650127	-2.32192809	-2.39592868	-2.73696559	-1.94341647
Aradu.MMG1D	-6.64385619	-2.55639335	-2.55639335	-1.64385619	-0.71311885
Aradu.QIZ46	-4.64385619	-4.32192809	-5.05889369	NA	-2.64385619
Aradu.F38CM	-3.32192809	NA	NA	NA	-4.32192809
Aradu.BR65G	1	1.560714954	1.269033146	1.895302621	2.15704371
Aradu.9C8PR	-0.71311885	0.250961574	-0.04394335	0.201633861	0.124328135
Aradu.109RJ	3.27351589	3.320484678	3.09592442	4.178714641	4.147306699
Aradu.ULX7G	NA	NA	NA	NA	NA
Aradu.ZP2LZ	0.250961574	0.056583528	1.500802053	0.321928095	1.495695163
Aradu.K3FG8	-0.81096618	0.042644337	0.137503524	0.378511623	0.344828497
Aradu.V37F6	4.010779839	3.71918344	3.86393845	4.384049807	5.163096138
Aradu.PK4QW	-0.47393119	-0.64385619	-1.05889369	-0.97143085	-0.05889369
Aradu.986KM	-5.64385619	-6.64385619	NA	2.301587647	2.427606173
Aradu.MU7BZ	0.201633861	0.941106311	0.704871964	2.358958826	2.321928095
Aradu.18HYX	4.798050515	4.739848103	4.562547724	3.363171077	3.240314329
Aradu.DT0KY	NA	-6.64385619	-6.64385619	NA	-0.68965988
Aradu.G5PJM	-1.28630419	-0.59946207	-0.94341647	1.5360529	1.427606173
Aradu.K3GE	1.641546029	0.97819563	0.97819563	-1.12029423	-0.73696559

6					
Aradu.FV8TW	3.799087306	4.062639828	4.110196178	4.452200049	4.875780063
Aradu.NI3N5	-2.18442457	-1.02914635	-1.35845397	-0.21759144	1.15704371
Aradu.5Y4IB	NA	NA	NA	NA	-4.32192809
Aradu.W9IJK	0.22650853	0.263034406	-0.07400058	0.918386234	0.757023247
Aradu.70NBC	0.443606651	0.59454855	0.464668267	0.389566812	1.384049807
Aradu.T8H92	-1.32192809	-1.18442457	-1.35845397	-0.01449957	-0.577767
Aradu.J1JQ8	4.815575429	5.497931652	5.377817853	5.831117195	6.260590275
Aradu.76UYG	2.851998837	2.825785627	2.731183242	2.914564523	3.538538164
Aradu.41J00	1.790772038	2.244887059	1.879705766	1.786596362	2.66448284
Aradu.XU3IM	2.480265122	2.718087584	2.435628594	2.827819025	3.112700133
Aradu.XQ5N2	-4.05889369	-2.05889369	-2.18442457	-1.94341647	0.014355293
Aradu.MKD6V	2.241840184	2.541019153	2.543495883	2.857980995	3.323370069
Aradu.0N6WQ	1.819668183	1.847996907	1.454175893	1.782408565	1.803227036
Aradu.KX3YY	2.375734539	2.871843649	2.666756592	3.404630684	3.464668267
Aradu.S4UH1	2.422233001	3.298658316	3.896271849	3.40599236	3.042644337
Aradu.MA9YS	1.839959587	2.207892852	1.786596362	3.267535798	3.389566812
Aradu.N190Q	-0.55639335	-0.62148838	-0.53533173	0.831877241	2.244887059
Aradu.R8ADM	2.942983598	3.311793718	2.124328135	3.531069493	3.606442228
Aradu.S70AZ	1.10433666	1.182692298	1.310340121	-1.43440282	2.220329955
Aradu.MFG61	4.130107179	4.578938713	3.999098034	5.418189948	5.346956889
Aradu.IWR1X	1.778208576	2.555816155	2.327687364	1.956056652	2.014355293
Aradu.WBB7S	1.757023247	2.042644337	2.028569152	1.495695163	1.339137385

Aradu.YI7L8	1.757023247	2.066950244	1.700439718	1.959770155	2.22650853
Aradu.RCQ39	-3.32192809	-0.83650127	-3.47393119	0.042644337	3.733354341
Aradu.X4DG3	2.9800253	3.269033146	3.068670811	3.473786912	3.667892125
Aradu.62T99	1.778208576	1.855989697	1.66448284	1.207892852	1.327687364
Aradu.07E2R	-5.05889369	-5.64385619	-5.05889369	-2.73696559	-1.35845397
Aradu.0WU0I	0.124328135	-0.4150375	-0.91593574	-1.68965988	-0.94341647
Aradu.Q3QE4	3.364572432	3.534808661	3.382667253	2.956056652	3.27351589
Aradu.U9G17	NA	NA	NA	NA	NA
Aradu.0P8G3	1.86393845	2.533563348	2.201633861	2.707082992	3.165107985
Aradu.88QB9	3.005399988	4.203984166	4.405311683	4.770300489	4.650190123
Aradu.I0X0M	-0.76121314	-0.23446525	-0.49410907	1.50589093	1.207892852
Aradu.2Q7FP	2.618238656	2.86393845	2.675815931	2.835924074	2.794935663
Aradu.Q3XX4	NA	-3.64385619	-3.47393119	-5.05889369	-2.83650127
Aradu.227K7	1.327687364	1.286881148	1.448900951	2.780310099	3.032100843
Aradu.T8IUY	0.933572638	0.773996325	1.3950628	-2.25153877	-1.25153877
Aradu.MX1D6	-0.97143085	-0.21759144	-1	0.124328135	0.201633861
Aradu.X2HBL	2.887525271	3.063502942	2.707082992	4.339850003	4.199279721
Aradu.VJK1R	-4.32192809	NA	-6.64385619	NA	NA
Aradu.WBK0P	0.454175893	1.269033146	0.5360529	2.414135533	2.295723025
Aradu.P3C6F	-1.51457317	-0.94341647	-1.88896869	-2.83650127	0.310340121
Aradu.72K9J	3.925999419	3.983677695	3.951401292	2.339137385	2.780310099
Aradu.Q9YKL	2.189033824	2.778208576	2.56315813	1.195347598	2.13422094

Aradu.U4Z04	3.09423607	3.310340121	3.13422094	3.010779839	3.384049807
Aradu.C6ILJ	4.186659017	3.881664619	3.793895883	2.160274831	3.053111336
Aradu.JLM18	1.327687364	1.952333566	2.370164281	1.82374936	1.655351829
Aradu.8E8GF	1.339137385	2.150559677	1.608809243	2.653060017	3.025028794
Aradu.6VX3K	-6.64385619	-4.05889369	-6.64385619	3.827819025	3.698218478
Aradu.D1ZWK	-0.26881676	-1.51457317	-2.39592868	2.408711861	2.495695163
Aradu.HI40M	2.201633861	2.650764559	2.432959407	2.493134922	2.289834465
Aradu.E2TII	4.193771743	4.902073579	4.776103988	3.682573297	4.553360503
Aradu.GTJ2E	-4.64385619	NA	NA	-2.18442457	-0.94341647
Aradu.0NE9W	3.846994687	3.939226578	3.950468414	2.931683057	3.243364426
Aradu.1M0CG	6.893968869	6.702657543	6.669593751	3.507160349	3.833902077
Aradu.M0V1K	1.176322773	0.86393845	0.970853654	0.189033824	0.59454855
Aradu.B4HJ4	NA	-5.64385619	NA	NA	NA
Aradu.P9DB9	3.397802962	3.763411574	3.365972428	4.43162296	4.806839582
Aradu.5X3H9	0.757023247	0.963474124	0.575312331	0.918386234	1.321928095
Aradu.X6AKD	3.116031993	3.693765712	3.760220946	-0.13606155	-0.43440282
Aradu.XPA7G	0.918386234	1.327687364	1.021479727	0.956056652	1.669026766
Aradu.UU910	3.053111336	3.544732656	3.201633861	4.393690764	4.553360503
Aradu.YC6XN	-5.64385619	-6.64385619	NA	-1.88896869	-2.39592868
Aradu.EKX2U	3.648465443	2.40599236	2.887525271	1.541019153	2.173127433
Aradu.9AW6M	1.448900951	1.220329955	0.604071324	2.316145742	2.875780063
Aradu.UX579	3.912649865	4.519793486	3.749534268	4.776103988	4.967168608

Aradu.IA5E9	-4.05889369	-2.94341647	-4.05889369	0	0.23878686
Aradu.U9WH5	0.286881148	-0.15200309	0.275007047	0.575312331	0.411426246
Aradu.22ZEB	3.016139703	4.788685711	4.3950628	5.222650022	5.769507291
Aradu.4HB9F	2.046141782	2.508428653	2.283921772	3.139142019	3.513490746
Aradu.200CK	3.317593505	3.586164246	3.168321116	4.6622055	4.621758857
Aradu.L8QZK	-0.26881676	-0.86249648	-1.43440282	-1.83650127	-0.30400619
Aradu.XTT89	-3.83650127	-2	-2.05889369	-0.59946207	0.799087306
Aradu.M8RTT	0.650764559	1.214124805	1.056583528	1.981852653	2.691534165
Aradu.LS0XC	-1.59946207	-2.18442457	-3.64385619	-0.16812276	0.555816155
Aradu.CB26G	NA	NA	NA	NA	-6.64385619
Aradu.2V4EQ	-2.32192809	-0.81096618	-0.71311885	0.22650853	0.189033824
Aradu.58WGM	2.353323291	3.039138394	2.742006211	2.310340121	2.606442228
Aradu.QKN8V	0.565597176	0.895302621	0.855989697	-1	-0.62148838
Aradu.5CY6X	3.826802684	2.384049807	2.13093087	2.257010618	2.173127433
Aradu.XV2LW	NA	NA	NA	NA	-2.94341647
Aradu.87FCC	NA	NA	NA	NA	-0.577767
Aradu.QS9PE	1.495695163	1.10433666	1.117695043	1.269033146	1.304511042
Aradu.306G1	-3.83650127	-6.64385619	NA	-3.47393119	-4.64385619
Aradu.Q0IL1	2.211012193	2.40599236	2.427606173	2.35614381	2.673556424
Aradu.YCJ90	-1.88896869	-2.12029423	-2.05889369	-2.94341647	-1.02914635
Aradu.U51AH	0.641546029	1.700439718	-0.07400058	5.527007761	5.245267467
Aradu.P7H9H	1.459431619	1.207892852	1.084064265	2.998195503	3.255500733

Aradu.X6KFI	NA	-3.83650127	NA	NA	NA
Aradu.97TKQ	3.386810946	3.75595577	3.377123749	4.309612772	4.564378169
Aradu.5T2RZ	2.087462841	1.82374936	2.137503524	1.263034406	1.304511042
Aradu.QZS0Y	1.948600847	2.070389328	2.22650853	1.799087306	1.748461233
Aradu.DF8UA	0.956056652	2.815575429	3.699329526	2.63691458	3.187451054
Aradu.A984U	NA	NA	NA	NA	NA
Aradu.Q0Z3H	4.246408087	4.958378712	3.653060017	6.023920649	5.413458688
Aradu.7M1P4	4.62935662	4.561326136	4.782408565	3.257010618	3.442280035
Aradu.570RS	-0.04394335	0.526068812	0.35614381	1.700439718	1.659924558
Aradu.SI0X3	-3.64385619	-5.64385619	-4.05889369	-0.13606155	1.372952098
Aradu.H33XQ	NA	-6.64385619	NA	NA	NA
Aradu.BG56S	-0.28630419	0.286881148	0.22650853	1.310340121	1.411426246
Aradu.ATP30	-5.64385619	-4.32192809	-5.05889369	-0.86249648	1.748461233
Aradu.8Q4X6	1.298658316	1.989139007	1.432959407	3.046141782	3.300123725
Aradu.Z9Y3J	NA	-4.32192809	-4.64385619	-2.12029423	-1.55639335
Aradu.W2A66	2.201633861	2.283921772	2.046141782	3.339137385	3.648465443
Aradu.NE8U1	0.925999419	1.475084883	1.339137385	1.641546029	2.10433666
Aradu.Y8XMT	1.298658316	1.704871964	1.286881148	1.438292852	1.97819563
Aradu.M64BQ	7.801805344	7.654206378	8.018088877	3.97819563	3.914564523
Aradu.N4PGS	-0.78587519	-0.88896869	-1.28630419	0.298658316	0.695993813
Aradu.R1ZAI	1.137503524	1.195347598	1.042644337	1.163498732	1.485426827
Aradu.6F1MK	2.59454855	2.910732662	2.893362211	4.008988783	3.956056652

Aradu.7091K	2.189033824	2.438292852	2.007195501	2.169925001	2.347665656
Aradu.FH3GQ	1.918386234	2.347665656	2.12763328	2.835924074	3.078951341
Aradu.MGD3M	1.327687364	2.545968369	1.220329955	3.43162296	3.257010618
Aradu.20QKH	2.316145742	2.929790998	2.733354341	3.139142019	3.512226887
Aradu.7VP5C	0.695993813	1.49057013	1.03562391	2.565597176	2.462052319
Aradu.P9ETF	2.969012308	2.967168608	3.252476214	2.855989697	3.056583528
Aradu.33XZT	4.121015401	5.46760555	5.524815928	4.122672719	4.188242656
Aradu.D18PX	2.622930351	2.370164281	2.275007047	1.89917563	2.003602237
Aradu.5E20L	1.298658316	0.298658316	0.124328135	-2.94341647	-2.73696559
Aradu.2IG4B	-3.47393119	-3.47393119	-3.05889369	-1.64385619	-1.64385619
Aradu.YH413	3.117695043	3.769771739	3.481557281	3.966245873	4.116863758
Aradu.D9DNB	NA	-5.64385619	NA	-4.64385619	-2.64385619
Aradu.02DA8	-1.88896869	-2.39592868	-2.18442457	NA	-2.32192809
Aradu.NS8UC	-0.23446525	0.687060688	0.650764559	1.655351829	1.871843649
Aradu.HN7R4	-4.05889369	NA	NA	-1.43440282	0.344828497
Aradu.EAG5M	-5.05889369	-4.32192809	-6.64385619	-0.81096618	-1.43440282
Aradu.2I4G0	-0.20091269	0.847996907	0.443606651	0.124328135	0.411426246
Aradu.I8GKQ	-1.83650127	-1.05889369	-1.64385619	0.678071905	0.956056652
Aradu.RC9UC	0.176322773	0.584962501	0.298658316	1.169925001	1.700439718
Aradu.IV87Z	4.372952098	4.473137488	4.406672716	4.952799478	4.489928219
Aradu.CX437	1.752748591	1.937344392	1.963474124	1.922197848	3.121015401
Aradu.MA1DE	2.691534165	4.503984704	3.82984956	5.853746197	5.227664073

Aradu.TW9EH	1.389566812	1.389566812	1.292781749	2.957914599	3.15704371
Aradu.T034F	0.910732662	0.98550043	0.97819563	1.480265122	2.053111336
Aradu.YQ4IR	-0.43440282	-0.07400058	-0.64385619	0.137503524	-0.4150375
Aradu.RY508	3.663344619	1.807354922	2.759155834	2.22342255	1.66448284
Aradu.4W4JU	-0.23446525	-0.16812276	-0.45403163	0.201633861	0.344828497
Aradu.W4N58	-5.05889369	-6.64385619	-6.64385619	NA	-4.32192809
Aradu.TJ47H	0.555816155	1.124328135	0.799087306	1.752748591	1.851998837
Aradu.1XB3Y	4.457462965	4.412104045	4.268284667	4.123500664	4.49441561
Aradu.LZ2RQ	1.195347598	1.09085343	1.150559677	-0.64385619	-0.43440282
Aradu.T45BF	1.673556424	1.97819563	1.49057013	1.608809243	1.622930351
Aradu.TF9ZM	2.310340121	2.641546029	2.250961574	3.061776198	3.365972428
Aradu.T6EFY	2.691534165	3.061776198	2.580145484	3.621758857	3.564378169
Aradu.FU605	NA	NA	NA	NA	NA
Aradu.5F6FU	-2.47393119	-2.47393119	-2.39592868	-2.47393119	-1.88896869
Aradu.09SAF	1.448900951	1.361768359	1.063502942	-0.26881676	0.97819563
Aradu.A97TU	NA	NA	-3.64385619	NA	NA
Aradu.ZFI13	-1.12029423	-0.86249648	-1.78587519	2.347665656	2.150559677
Aradu.J9V4P	-1.51457317	2.358958826	1.948600847	-0.15200309	-0.10469738
Aradu.SZ07F	2.582556003	2.709290636	2.459431619	2.765534746	3.365972428
Aradu.G68BX	-1.47393119	-1.18442457	-1.51457317	0.333423734	-0.39592868
Aradu.ZD4TK	2.472487771	1.608809243	1.956056652	-3.47393119	-1.02914635
Aradu.M167P	3.59454855	3.751677946	3.339137385	4.631104282	4.845490051

Aradu.Z2H09	1.531069493	1.803227036	1.021479727	1.304511042	0.50589093
Aradu.432N5	4.362469889	4.491853096	4.125155131	4.943452539	5.123914459
Aradu.2Z821	-3.18442457	-5.05889369	-3.83650127	-6.64385619	-3.47393119
Aradu.3P75R	4.51916445	4.923624611	4.591559746	3.075532631	3.517275693
Aradu.9H5DV	-0.71311885	-0.35845397	-0.81096618	1.333423734	1.641546029
Aradu.B74MT	2.10433666	2.731183242	2.307428525	3.574101508	3.555816155
Aradu.1HN9G	0.163498732	-0.34007544	-0.62148838	-0.73696559	-0.577767
Aradu.2EU9J	-5.05889369	-2.94341647	-3.05889369	-2.83650127	-2.05889369
Aradu.1659P	0.250961574	-0.18442457	-0.53533173	1.855989697	2.084064265
Aradu.6R4YC	NA	NA	NA	NA	NA
Aradu.516WS	4.832890014	5.088311236	5.229972385	2.295723025	2.084064265
Aradu.6VU3X	NA	-5.64385619	NA	-4.64385619	-5.64385619
Aradu.0C0S7	2.176322773	2.847996907	2.313245852	2.833902077	3.053111336
Aradu.3MG08	0.687060688	1.389566812	0.933572638	2.386810946	2.577730931
Aradu.AHG3H	-1.18442457	-2.05889369	-1.35845397	-2.73696559	-3.18442457
Aradu.DM7P1	1.327687364	1.117695043	1.15704371	-0.53533173	0.321928095
Aradu.T8I2T	2.386810946	2.659924558	2.485426827	3.75595577	3.759155834
Aradu.33ULW	2.272023189	2.263034406	2.715893371	-1.15200309	-4.64385619
Aradu.C1X9T	0.137503524	0.86393845	0.584962501	2.422233001	2.472487771
Aradu.28WLZ	3.811471031	3.59454855	3.434294618	4.912170803	5.237257771
Aradu.LD7BF	4.496973581	5.833396134	6.166112857	4.29130886	4.828834649
Aradu.RII5F	3.392317423	3.111031312	3.132576843	1.565597176	1.650764559

Aradu.D6DD4	-5.64385619	-6.64385619	-5.64385619	NA	5.043956873
Aradu.0I3GY	-4.64385619	-3.47393119	-4.32192809	-1.02914635	-1.51457317
Aradu.G5NTR	-0.66657627	-1.68965988	-1.35845397	0.411426246	1.084064265
Aradu.IFF56	-5.05889369	NA	-5.05889369	5.643278996	6.607774152
Aradu.DAT06	1.500802053	1.316145742	1.257010618	2.220329955	2.869871406
Aradu.C824H	-2.32192809	-2.94341647	-3.18442457	-2.55639335	-1.08926734
Aradu.0ER06	4.605849867	4.475084883	3.87282876	4.232660757	4.864928972
Aradu.714NZ	-2.73696559	-2.18442457	-3.05889369	0.933572638	0.933572638
Aradu.U8PRD	0.970853654	1.687060688	1.214124805	3.324810603	3.607626221
Aradu.D3JBM	1.10433666	0.970853654	0.678071905	2.049630768	2.493134922
Aradu.N289G	3.481557281	3.681449265	3.503348735	4.408032466	4.675815931
Aradu.4WT7F	3.74092756	4.308156975	4.098453246	5.505255801	5.663913842
Aradu.13SFN	0.321928095	0.432959407	-1.43440282	3.09085343	4.078097423
Aradu.X6KLL	-0.34007544	-1.02914635	-0.97143085	2.912649865	2.724650272
Aradu.MQT1Y	2.22650853	2.3305584	2.185866545	0.799087306	1.042644337
Aradu.6K05J	2.724650272	3.08236197	2.855989697	3.793895883	4.164303583
Aradu.J5AII	3.257010618	3.068670811	2.889473543	3.560714954	3.760220946
Aradu.9D49Q	5.526068812	5.543495883	5.266411766	5.84899843	6.100346961
Aradu.647P3	-2.12029423	-1.43440282	-1.25153877	2.49057013	1.545968369
Aradu.21FR3	2.742006211	3.267535798	2.835924074	4.378511623	4.308885057
Aradu.T0YTZ	-1.25153877	-0.73696559	-1.51457317	0.632268215	0.831877241
Aradu.734NR	2.361768359	2.565597176	2.384049807	2.837943242	2.957914599

Aradu.FQ3N4	-2.64385619	0.124328135	-0.55639335	-3.05889369	-2.64385619
Aradu.U3SNU	4.34269696	4.871350841	5.045705065	5.980939266	5.693765712
Aradu.0Y40Q	5.000901403	5.300855871	5.528571319	2.684818738	3.998195503
Aradu.6V2BQ	1.761285273	1.887525271	1.769771739	1.344828497	2.432959407
Aradu.P8XB7	1.117695043	1.03562391	1.03562391	3.111031312	3.607626221
Aradu.EYV3C	3.724650272	3.777156666	3.077242999	3.975446766	4.182692298
Aradu.4BB0R	5.863195112	5.710117632	5.8008999	2.794935663	3.504620392
Aradu.TYF13	0.555816155	1.333423734	0.757023247	1.843983844	2.283921772
Aradu.81LQN	1.422233001	0.604071324	0.526068812	1.007195501	1.280956314
Aradu.L4C7Z	NA	NA	NA	NA	-4.64385619
Aradu.KHV94	0.298658316	0.22650853	0.214124805	1.454175893	1.761285273
Aradu.ZAK9S	0.5360529	1.13093087	0.687060688	1.98550043	2.46727948
Aradu.4N6LD	0.90303827	1.673556424	0.367371066	2.528571319	2.702657543
Aradu.CJQ6L	6.399854674	6.349082146	6.058316496	6.210038123	6.236301267
Aradu.3ZC2Z	-1.51457317	-1.05889369	-2.18442457	1.97819563	1.613531653
Aradu.D46RH	-2.47393119	-1.35845397	-2.39592868	2.650764559	2.548436625
Aradu.IVC5J	0.659924558	0.887525271	0.422233001	2.358958826	2.247927513
Aradu.61Y7W	4.46139759	4.684257705	4.827310944	4.437627248	4.799605422
Aradu.LE6Z2	NA	NA	NA	NA	NA
Aradu.X4HEP	NA	NA	NA	NA	NA
Aradu.FSK9A	1.23878686	1.59454855	1.5360529	1.883620816	2.100977648
Aradu.X5TRC	-1.35845397	-0.76121314	-0.83650127	NA	-4.05889369

Aradu.R0BEJ	-3.32192809	-4.64385619	NA	NA	NA
Aradu.L1VEE	NA	-2.94341647	-3.47393119	-2.47393119	-2.94341647
Aradu.6W7E4	3.360364277	4.341274184	3.814550423	4.769771739	4.463360886
Aradu.06JB2	2.939226578	3.289834465	2.771885579	5.192588722	5.316507819
Aradu.M9CLB	-1.73696559	-2.05889369	-2.05889369	-1.18442457	-0.18442457
Aradu.3S3UE	4.523561956	4.508428653	4.657068301	3.311793718	3.723558561
Aradu.MZ9P5	-3.18442457	-4.32192809	-4.32192809	-1.43440282	-1.39592868
Aradu.VQ1MT	-3.18442457	-2.64385619	-4.64385619	-1.55639335	-1.47393119
Aradu.Y8PUZ	5.181500152	5.172727518	5.558267634	2.477677328	2.555816155
Aradu.20FIR	-0.39592868	1.14404637	0.815575429	2.482848283	2.336283388
Aradu.QJ7B7	0.5360529	2.124328135	2.09085343	-0.97143085	1.09085343
Aradu.RR75T	2.994579724	2.99095486	3.246408087	-0.30400619	-0.13606155
Aradu.9AB3B	3.423578171	3.649615459	3.097610797	5.084064265	5.086189313
Aradu.CQN7Q	2.541019153	2.518535139	2.855989697	1.220329955	1.580145484
Aradu.U35FJ	0.839959587	0.35614381	-0.47393119	1.263034406	0.622930351
Aradu.UAJ8F	2.40599236	2.472487771	2.427606173	1.843983844	2.44625623
Aradu.CZW32	-2.18442457	-2.64385619	-3.47393119	-0.39592868	-0.26881676
Aradu.SB5CI	NA	-1.83650127	-0.73696559	-1.47393119	4.95419631
Aradu.79876	-0.02914635	-0.08926734	-2.25153877	-3.64385619	1.438292852
Aradu.B925F	-2.25153877	-1.68965988	-1.83650127	-1.08926734	-0.66657627
Aradu.RF2LV	NA	NA	NA	NA	-4.32192809
Aradu.65T0K	1.014355293	1.384049807	1.310340121	1.10433666	1.361768359

Aradu.U66TG	1.40599236	0.773996325	1.007195501	3.08236197	2.541019153
Aradu.YRW97	2.613531653	2.459431619	2.424922088	1.3950628	1.269033146
Aradu.TEW52	1.257010618	1.646162657	1.570462931	2.956056652	3.759155834
Aradu.M9J7Q	1.232660757	1.687060688	1.613531653	1.678071905	1.618238656
Aradu.LF0Z4	2.066950244	1.925999419	2.042644337	1.967168608	2.327687364
Aradu.2Y48Q	-4.64385619	-3.64385619	-3.47393119	-4.32192809	-2.83650127
Aradu.T66QJ	2.737686761	2.82374936	2.904965719	2.698218478	2.867896464
Aradu.2J2S3	NA	-4.05889369	-5.64385619	NA	NA
Aradu.53CQM	4.263034406	2.831877241	3.310340121	4.233427944	4.118525849
Aradu.KTY6M	0.815575429	0.321928095	-0.01449957	-0.26881676	0.918386234
Aradu.F0EGY	3.593353771	3.386810946	3.438292852	0.565597176	0.286881148
Aradu.6PF06	-3.64385619	2.565597176	1.09085343	1.956056652	0.887525271
Aradu.C64A0	2.124328135	1.819668183	2.169925001	-0.97143085	-0.34007544
Aradu.Q5Q2B	2.310340121	2.480265122	2.313245852	2.763411574	3.051372102
Aradu.R0BCA	3.145677455	3.502075956	3.241840184	4.512226887	4.272769732
Aradu.29ERF	-3.83650127	-2.39592868	-3.32192809	-1.59946207	-0.55639335
Aradu.B8U65	0.82374936	0.22650853	-0.13606155	0.565597176	0.641546029
Aradu.074JV	-4.64385619	-3.64385619	-3.64385619	-2.73696559	-3.64385619
Aradu.CI9FK	NA	-4.32192809	-4.64385619	0.687060688	1.769771739
Aradu.YZ24M	-1.12029423	-1.64385619	-2.05889369	0.790772038	0.925999419
Aradu.Q1F6L	-2.47393119	-2.64385619	-1.78587519	0.163498732	0.084064265
Aradu.KD6QW	NA	NA	NA	NA	-6.64385619

Aradu.008V1	-3.83650127	NA	-5.05889369	NA	-5.05889369
Aradu.301B3	2.622930351	3.50589093	3.001802243	2.014355293	2.44625623
Aradu.NC1XP	1.815575429	2.516015147	2.22342255	2.843983844	3.039138394
Aradu.RZY5Q	0.956056652	0.748461233	0.575312331	-1.02914635	-0.18442457
Aradu.HS2F0	NA	NA	NA	NA	NA
Aradu.N7LX6	3.90014226	4.516645558	4.160274831	4.948133578	4.903520374
Aradu.2BI47	0.275007047	1.438292852	1.695993813	0.773996325	1.361768359
Aradu.K358E	2.298658316	2.684818738	2.14404637	2.704871964	2.462052319
Aradu.SH1QV	NA	NA	NA	NA	NA
Aradu.6X9VQ	-3.18442457	-3.18442457	-2.05889369	-2.05889369	-1.28630419
Aradu.D2U3D	NA	-4.05889369	-5.64385619	2.66448284	1.700439718
Aradu.G8EKB	NA	-5.05889369	-5.05889369	NA	-4.32192809
Aradu.XM9I6	2.250961574	2.192194165	1.695993813	3.62058641	3.473786912
Aradu.E1Q5N	-2.55639335	-1.68965988	-2.64385619	-1.64385619	-1.21759144
Aradu.SF9ZV	1.589763487	1.914564523	1.599317794	1.280956314	1.339137385
Aradu.041TI	5.477029653	5.197314999	5.521050737	3.122672719	2.684818738
Aradu.R8YHS	3.548436625	3.871843649	3.378511623	4.852498299	5.050501697
Aradu.5W065	2.185866545	2.568032105	2.220329955	2.937344392	3.270528942
Aradu.R0TCR	0.948600847	0.454175893	0.321928095	-1.08926734	1.782408565
Aradu.T2VBJ	-1.51457317	-0.53533173	-2.12029423	1.659924558	2.124328135
Aradu.IZ11Y	4.963474124	6.834660658	6.640678764	4.040892431	3.988230223
Aradu.8Q6IV	-1.15200309	-3.05889369	-3.05889369	0.485426827	0.948600847

Aradu.YA89L	4.654779217	5.100557221	4.705425039	6.179311989	6.208868371
Aradu.3MD78	NA	NA	NA	NA	NA
Aradu.D6N4B	NA	NA	NA	NA	NA
Aradu.I9PC5	0.871843649	0.704871964	0.86393845	0.124328135	0.704871964
Aradu.7GG37	1.220329955	1.339137385	1.014355293	1.232660757	1.367371066
Aradu.K42LN	-0.34007544	-0.21759144	-0.49410907	2.910732662	2.495695163
Aradu.8AC2D	8.460169172	7.956811732	8.058208246	4.299391206	4.257764969
Aradu.7L12D	0.124328135	-0.47393119	-0.59946207	-1.88896869	-1.39592868
Aradu.20KIH	NA	-4.64385619	-5.05889369	-5.05889369	-5.05889369
Aradu.I4MAR	-0.01449957	1.378511623	1.682573297	1.887525271	-0.02914635
Aradu.DLI1Q	0.86393845	1.182692298	0.50589093	1.464668267	1.555816155
Aradu.JZ4PA	-4.05889369	-3.47393119	-4.32192809	-1.64385619	-2.47393119
Aradu.K0SK7	0.367371066	-0.30400619	-0.04394335	-2.47393119	-1.64385619
Aradu.VUQ4V	5.255500733	5.397118409	5.107687869	5.01346226	5.112283109
Aradu.QH0IG	-5.64385619	-1.35845397	-0.18442457	-6.64385619	-1.78587519
Aradu.CG33N	-4.32192809	-6.64385619	-4.32192809	-2.94341647	-2.39592868
Aradu.VIH8G	-2.47393119	-0.83650127	-0.71311885	0.411426246	0.839959587
Aradu.TPI8X	1.98550043	2.570462931	2.541019153	3.200064862	4.217230716
Aradu.L7DZT	3.420886575	3.381283373	3.378511623	3.952333566	3.966245873
Aradu.9JQ87	2.784503983	2.673556424	2.419538892	2.15704371	2.560714954
Aradu.3DX6E	1.757023247	2.217230716	1.963474124	2.715893371	2.893362211
Aradu.G7CZU	-1.08926734	0.250961574	0.333423734	1.50589093	1.495695163

Aradu.C89XH	3.15704371	3.531069493	3.300123725	3.875780063	3.928844037
Aradu.JCH8S	-2.25153877	-0.59946207	-1.47393119	1.389566812	1.959770155
Aradu.LCE5M	1.778208576	2.217230716	1.731183242	1.925999419	2.464668267
Aradu.UD1IS	3.837943242	3.9800253	3.386810946	5.049195106	5.511278265
Aradu.W1BNF	-4.64385619	-3.32192809	-3.64385619	-3.47393119	-1.59946207
Aradu.QL7M2	-5.64385619	-4.64385619	-4.05889369	-3.47393119	-2.12029423
Aradu.CI6AA	-4.64385619	NA	-6.64385619	2.14404637	2.459431619
Aradu.W162D	-0.68965988	0.298658316	-1.05889369	4.394376945	4.196921734
Aradu.50RNX	-1.28630419	-0.73696559	-1.43440282	2.831877241	2.553360503
Aradu.U6HMB	2.833902077	2.807354922	2.857980995	2.411426246	2.819668183
Aradu.E4IE7	0.250961574	1.286881148	1.722466024	1.372952098	1.117695043
Aradu.KGX8R	0.790772038	1.182692298	1.042644337	2.260025656	2.416839742
Aradu.R07DC	1.970853654	3.544732656	2.14404637	1.941106311	3.794935663
Aradu.6S4B3	-4.05889369	-6.64385619	NA	-3.32192809	1.021479727
Aradu.2YF2R	5.607330314	6.09592442	6.153400028	6.780835002	7.48050749
Aradu.JS1AI	-5.05889369	-6.64385619	-4.05889369	NA	-1.43440282
Aradu.HN2EG	-3.64385619	-2.83650127	-3.18442457	-1.02914635	-0.13606155
Aradu.R5FQX	1.195347598	1.361768359	0.918386234	0.137503524	0.176322773
Aradu.6J2SN	-1.43440282	-0.53533173	-0.83650127	-2.64385619	-2.12029423
Aradu.C1ZP7	1.298658316	1.773996325	1.269033146	2.650764559	2.718087584
Aradu.QS5ZN	4.196134881	4.053980168	4.185866545	2.528571319	3.629939409
Aradu.R2VBI	2.769771739	3.203201156	2.861955364	4.10349764	4.138323004

Aradu.Z6X40	-1.59946207	-0.88896869	-1.68965988	0.956056652	1.589763487
Aradu.47VKV	3.058316496	4.339850003	4.022367813	3.196921734	3.350497247
Aradu.KVX0Z	4.275752049	4.437627248	4.239550797	5.096346199	5.403949364
Aradu.8HB43	-0.39592868	-0.13606155	-0.47393119	1.811471031	1.925999419
Aradu.W06MF	-0.05889369	-0.37706965	-0.76121314	1.827819025	2.014355293
Aradu.Q3FM7	3.283921772	2.841973119	2.702657543	1.469885976	-0.21759144
Aradu.366M2	1.98550043	2.010779839	2.687060688	2.316145742	2.713695815
Aradu.2AQ5X	NA	NA	NA	0.555816155	-1.21759144
Aradu.P5H21	0.831877241	0.555816155	0.925999419	1.599317794	1.952333566
Aradu.7YM1I	-2.32192809	-0.83650127	-0.86249648	-3.18442457	-1.51457317
Aradu.DXP84	2.039138394	2.503348735	2.12763328	3.109360559	3.419538892
Aradu.UB2GG	NA	NA	NA	NA	-4.64385619
Aradu.Q9JNX	-5.64385619	-3.47393119	NA	-2.55639335	-2.32192809
Aradu.CYS3J	5.040015679	4.808385051	4.909773104	2.244887059	2.480265122
Aradu.WBM9H	3.914564523	3.836934011	3.9800253	4.040892431	4.283921772
Aradu.7G9TQ	NA	NA	-6.64385619	NA	NA
Aradu.CS1KJ	-0.01449957	0.722466024	0.084064265	2.430285273	2.575312331
Aradu.TFD03	-0.25153877	-0.26881676	-0.68965988	1.422233001	1.580145484
Aradu.ZC9DU	1.350497247	3.021479727	3.601696516	2.448900951	2.462052319
Aradu.L3X78	NA	NA	NA	NA	-5.05889369
Aradu.Y4VNZ	1.298658316	1.835924074	1.316145742	3.14404637	3.100977648
Aradu.068JX	2.935459748	3.028569152	2.875780063	3.71259578	4.200064862

Aradu.7LR28	2.319039816	2.82374936	2.495695163	3.489286023	3.350497247
Aradu.FU7AU	NA	NA	NA	NA	NA
Aradu.41JSL	3.311793718	3.061776198	3.294253136	0.432959407	0.879705766
Aradu.F1E13	0.137503524	1.731183242	0.201633861	2.014355293	2.021479727
Aradu.MQK7K	3.252476214	3.153805336	2.97819563	3.229587923	3.754887502
Aradu.NYS45	-4.64385619	-2.39592868	-3.32192809	-2.73696559	-3.83650127
Aradu.G2GUD	1.014355293	1.189033824	1.070389328	1.049630768	1.021479727
Aradu.F0544	-3.83650127	-3.18442457	-3.32192809	1.443606651	1.40599236
Aradu.75AY8	-1.73696559	-1.59946207	-1.88896869	-0.66657627	0.526068812
Aradu.MY7DS	2.560714954	2.959770155	2.653060017	3.645009884	3.730096466
Aradu.T7J8U	1.361768359	1.790772038	1.613531653	1.887525271	1.944858446
Aradu.U1CK3	3.725741157	3.795974694	3.584962501	1.411426246	1.480265122
Aradu.V2AUT	2.459431619	2.873813198	2.533563348	2.295723025	2.553360503
Aradu.92NW5	-4.05889369	-4.05889369	-3.18442457	NA	-6.64385619
Aradu.GZZ2X	3.622930351	3.576522138	3.538538164	4.74092756	5.146492307
Aradu.L6HBL	2.070389328	2.622930351	2.063502942	3.211012193	3.177917792
Aradu.K4111	0.298658316	0.310340121	-0.25153877	0.632268215	0.895302621
Aradu.3UN15	NA	-3.64385619	-4.64385619	-3.05889369	-2.73696559
Aradu.U96FI	-0.12029423	1.056583528	-0.16812276	2.837943242	3.382667253
Aradu.YW2M0	1.86393845	2.286881148	1.773996325	1.843983844	2.182692298
Aradu.M3S97	4.137503524	4.324090516	5.270528942	NA	-5.05889369
Aradu.YH03P	-2.25153877	-3.18442457	-3.83650127	NA	-3.05889369

Aradu.9J6QD	3.241840184	2.077242999	2.967168608	-5.05889369	-2.64385619
Aradu.LZ48I	1.475084883	1.49057013	1.599317794	0.565597176	1.722466024
Aradu.80AKC	3.548436625	3.87282876	3.780310099	2.9202933	3.434294618
Aradu.P8Y29	4.081510068	5.707359132	4.72955277	2.117695043	3
Aradu.3IM1W	1.244887059	1.510961919	1.220329955	3.019701914	3.016139703
Aradu.8Z4L7	-0.88896869	-0.47393119	-0.51457317	0.097610797	3.122672719
Aradu.M1IAT	2.295723025	2.469885976	2.35614381	2.994579724	3.181102551
Aradu.WNH12	0.659924558	1.304511042	1.042644337	1.411426246	1.659924558
Aradu.V07RE	-0.62148838	-0.47393119	-0.88896869	-0.07400058	0.070389328
Aradu.8567N	3.344828497	3.365972428	3.030336078	2.09085343	2.257010618
Aradu.DQM3T	3.455491621	3.005399988	3.0721058	3.981852653	3.557042415
Aradu.9M2XX	-0.64385619	-0.25153877	-0.91593574	1.859969548	1.682573297
Aradu.ZSR47	-2.05889369	-1.39592868	-1.39592868	-1.64385619	-0.59946207
Aradu.SKW70	3.758089934	3.969012308	4.234194723	3.727920455	4.025914699
Aradu.MTE62	1.778208576	2.007195501	1.97819563	2.438292852	2.970853654
Aradu.G307L	NA	-4.32192809	-6.64385619	-3.47393119	-0.13606155
Aradu.R23DU	2.744161096	2.908812908	2.847996907	3.659924558	3.74092756
Aradu.JC4ID	2.584962501	2.965322548	2.761285273	3.264536431	3.397802962
Aradu.8G7WX	0.526068812	1.214124805	0.895302621	0.941106311	1.613531653
Aradu.HG68U	NA	NA	NA	NA	NA
Aradu.AA5JL	4.14404637	4.314696526	3.83491343	2.59454855	2.912649865
Aradu.L3RRG	1.250961574	1.084064265	0.765534746	0.464668267	0.443606651

Aradu.M0DM1	0.422233001	1.344828497	0.879705766	1.570462931	0.201633861
Aradu.HG1BY	4.891419187	4.438958148	5.278356528	0.333423734	1.879705766
Aradu.C8Q2A	NA	NA	NA	NA	-2.25153877
Aradu.IV8SJ	3.224966365	4.04176865	2.378511623	5.362119167	5.600804955
Aradu.D400V	2.693765712	3.142413438	2.769771739	3.945794957	3.797012978
Aradu.RP5AZ	1.687060688	2.49057013	1.748461233	2.495695163	2.301587647
Aradu.3E55M	0.632268215	1.438292852	0.687060688	1.560714954	1.859969548
Aradu.KRY7W	1.560714954	1.807354922	2.277984747	1.790772038	2.622930351
Aradu.HUW75	3.257010618	3.102658131	2.901108243	2.378511623	2.950468414
Aradu.82C4A	-1.12029423	-2.55639335	-1.35845397	1.310340121	1.124328135
Aradu.3CF99	1.713695815	2.733354341	1.925999419	2.241840184	2.344828497
Aradu.GT1TJ	4.56924803	4.744161096	4.550900665	5.993221467	6.230548887
Aradu.T6HSI	2.541019153	2.939226578	2.780310099	3.582556003	3.812498225
Aradu.YW5TG	NA	NA	NA	NA	-3.64385619
Aradu.Y2WFH	NA	NA	NA	NA	-3.83650127
Aradu.61FU3	-0.39592868	0.189033824	-0.23446525	0.847996907	1.361768359
Aradu.Y37CK	2.720278465	2.935459748	2.553360503	3.463360886	3.813524689
Aradu.C4J7U	-2.83650127	-2.12029423	-2.05889369	-0.73696559	-0.02914635
Aradu.86KS5	5.18030702	4.983677695	5.179112901	3.901108243	3.838951767
Aradu.1L0P8	-1.78587519	-1	-1	0.5360529	0.014355293
Aradu.5X8VR	-1.55639335	-1.55639335	-2	0.214124805	1.070389328
Aradu.LXU7Y	0.90303827	1.744161096	1.13093087	1.555816155	1.765534746

Aradu.114L5	NA	NA	NA	NA	NA
Aradu.UVJ1U	3.656496371	4.09085343	3.794935663	5.04176865	5.329841177
Aradu.0BM64	3.111031312	3.695993813	3.267535798	4.207111961	4.509062386
Aradu.8MB2V	-6.64385619	-6.64385619	-6.64385619	0.411426246	0.82374936
Aradu.T9XRG	NA	-3.83650127	-5.64385619	-3.83650127	-3.83650127
Aradu.XR2K7	6.701133155	6.735792928	6.672283894	3.758089934	5.130107179
Aradu.WYR1J	5.055716264	4.313971371	4.083213368	3.078951341	3.727920455
Aradu.412P9	5.67468662	5.372255648	6.799216853	4.424250286	4.078097423
Aradu.2X0TU	4.026800059	6.717128026	5.706530553	2.073820233	2.718087584
Aradu.E84ZM	NA	NA	NA	NA	NA
Aradu.E8NYC	3.613531653	3.399171094	3.581351247	1.298658316	1.526068812
Aradu.2AU2Z	-4.32192809	-5.05889369	-4.64385619	-2.47393119	-1.83650127
Aradu.19EUZ	0.650764559	1.042644337	0.584962501	2.117695043	1.835924074
Aradu.C8KKZ	-1.78587519	-1.47393119	-2.32192809	-0.47393119	0.124328135
Aradu.JJU8Z	1.678071905	2.998195503	2.247927513	-0.32192809	1.516015147
Aradu.4ZD6C	NA	-4.64385619	-5.05889369	-3.83650127	-5.05889369
Aradu.4RN5Y	NA	NA	NA	NA	NA
Aradu.4V3YY	-5.64385619	-4.64385619	-5.64385619	NA	-4.05889369
Aradu.HA5JM	-0.12029423	0.163498732	-0.13606155	1.007195501	1.23878686
Aradu.LK1UG	5.572889668	5.634302842	5.586764743	5.416501993	5.787118983
Aradu.F0D6Z	-3.64385619	-6.64385619	NA	-5.64385619	-4.32192809
Aradu.8T260	1.86393845	2.140778656	1.952333566	3.282439805	3.780310099
Aradu.I1U3H	0.790772038	1.163498732	0.713695815	0.584962501	0.704871964

Aradu.83MPA	1.150559677	1.422233001	0.575312331	-0.47393119	1.922197848
Aradu.WIQ29	NA	NA	NA	NA	-2.39592868
Aradu.24BNI	1.673556424	1.752748591	1.646162657	1.344828497	1.659924558
Aradu.73ZLX	3.09085343	3.560714954	3.232660757	4.253989266	4.250203656
Aradu.P2C14	-2.47393119	0.722466024	0.669026766	2.693765712	3.791814071
Aradu.L273D	0.378511623	0.82374936	0.464668267	0.275007047	0.378511623
Aradu.M9TY2	-2.32192809	-1.32192809	-1.55639335	-2.47393119	-2.32192809
Aradu.47ZJ4	-2.39592868	-1.55639335	-1.94341647	0.286881148	1.59454855
Aradu.UQQ1M	7.111865964	6.667892125	6.506684448	3.601696516	3.791814071
Aradu.49VWN	2.097610797	1.937344392	1.469885976	2.257010618	2.879705766
Aradu.5D1VA	1.704871964	1.992768431	1.521050737	2.269033146	2.767654798
Aradu.Z5EFE	1.618238656	1.464668267	1.327687364	1.687060688	2.283921772
Aradu.S8IWK	5.841721581	5.690975738	5.388533979	4.418864577	4.76394266
Aradu.D2JYY	-0.28630419	0.201633861	-0.66657627	2.283921772	2.430285273
Aradu.NT5AR	-0.55639335	0.214124805	0.189033824	1.378511623	1.560714954
Aradu.EZW7V	0.432959407	1.526068812	0.933572638	1.819668183	1.933572638
Aradu.CR9NG	2.469885976	2.5360529	2.40599236	2.3305584	2.107687869
Aradu.UTZ1D	1.448900951	1.691534165	1.691534165	1.378511623	1.263034406
Aradu.NQ2AE	1.250961574	2.060047384	1.632268215	2.198494154	2.327687364
Aradu.7GX01	NA	NA	NA	NA	NA
Aradu.DVL50	1.827819025	2.204766751	2.003602237	2.648465443	3.08236197
Aradu.8Q9T	-0.66657627	-0.55639335	-1.28630419	1.790772038	2.169925001

1					
Aradu.TRI7U	-0.86249648	-0.68965988	-0.32192809	-3.05889369	-0.23446525
Aradu.I3E1J	NA	-6.64385619	-3.83650127	-3.32192809	-3.64385619
Aradu.PSF4U	3.418189948	2.865918815	3.198494154	3.634593268	3.602884409
Aradu.35HVS	4.358255587	4.51412226	4.608809243	4.037382222	3.847996907
Aradu.1ZR6L	3.35473424	1.989139007	2.500802053	-1.32192809	0.454175893
Aradu.P0J2M	3.632268215	3.916476644	3.589763487	3.9800253	4.310340121
Aradu.6D72R	3.393690764	3.766595162	3.64731451	4.080657663	4.412781525
Aradu.U7FRB	0.378511623	0.97819563	0.86393845	1.321928095	1.992768431
Aradu.MSQ8X	8.706185171	8.291032526	8.665264847	5.871843649	6.03760186
Aradu.CD22H	NA	-6.64385619	NA	-2.47393119	-4.05889369
Aradu.MHS46	-0.91593574	-0.45403163	-1	1.443606651	1.650764559
Aradu.I67U0	-1.12029423	-1.83650127	-5.05889369	2.634593268	1.316145742
Aradu.89TY1	2.669026766	2.944858446	2.513490746	4.430285273	4.600507645
Aradu.Y1K8W	4.470536865	5.044394119	4.186659017	5.669026766	5.557961427
Aradu.50HDP	0.948600847	1.063502942	0.933572638	1.891419187	2.182692298
Aradu.J5XVH	NA	-5.05889369	NA	-0.577767	-0.51457317
Aradu.U999X	-0.04394335	-0.81096618	-0.68965988	-0.04394335	-0.91593574
Aradu.CVS4L	-2.64385619	-2.18442457	-1.94341647	-2.32192809	-1.28630419
Aradu.2DM5J	-3.83650127	-0.62148838	-1.55639335	-2.83650127	-1.08926734
Aradu.FK4M3	-1.47393119	-0.68965988	-1.02914635	-0.86249648	-0.83650127
Aradu.KH49T	-2.94341647	-2.12029423	-2.94341647	-0.47393119	-0.45403163
Aradu.L2S26	NA	NA	NA	NA	NA

Aradu.DPF9Q	2.575312331	2.669026766	2.767654798	2.773996325	3.003602237
Aradu.R082J	-2.94341647	-4.32192809	-3.64385619	-1.68965988	-1.08926734
Aradu.SK8YF	-4.32192809	-5.05889369	-6.64385619	-3.18442457	-2.83650127
Aradu.BK3J1	3.12763328	2.845991771	2.669026766	1.459431619	2.3305584
Aradu.UAH84	-1.78587519	-1.02914635	-1.28630419	-2.39592868	-0.81096618
Aradu.98K4P	0.839959587	1.427606173	1.049630768	2.568032105	2.963474124
Aradu.GR9T1	-4.32192809	-1.88896869	-3.18442457	1.416839742	2.763411574
Aradu.L61KF	-3.32192809	-3.05889369	-2.39592868	-1.39592868	-0.28630419
Aradu.RFT1Y	8.756122615	8.216406361	8.251624424	4.517905554	4.690975738
Aradu.5H0S7	-3.47393119	-6.64385619	-4.32192809	NA	-5.64385619
Aradu.72KXP	1.641546029	1.298658316	1.244887059	0.464668267	1.321928095
Aradu.VFG89	1.176322773	1.327687364	0.757023247	2.5360529	2.432959407
Aradu.MIM9N	-3.32192809	-0.78587519	-2.32192809	NA	-3.83650127
Aradu.JV2C7	2.924099886	3.010779839	2.599317794	3.240314329	3.460742564
Aradu.5474V	-5.05889369	0.695993813	-1	-0.26881676	-1.12029423
Aradu.6LS7N	2.627606838	2.942983598	2.684818738	3.739848103	3.956056652
Aradu.BV2Q9	NA	NA	NA	2.13422094	3.263034406
Aradu.NC6JU	0.887525271	1.646162657	1.584962501	1.989139007	2.408711861
Aradu.NF6XD	-1.18442457	-1.08926734	-1.05889369	-3.64385619	-1.02914635
Aradu.EXV8Z	-0.25153877	-4.05889369	1.03562391	-4.64385619	-4.05889369
Aradu.FP3TI	0.344828497	-0.45403163	-0.4150375	-1.55639335	-0.59946207
Aradu.DK5WH	1.952333566	2.568032105	2.207892852	2.541019153	2.372952098

Aradu.XGN4F	0.275007047	-0.26881676	-0.07400058	2.592158002	2.87774425
Aradu.V4LAJ	0.86393845	1.49057013	1.35614381	1.811471031	1.469885976
Aradu.Q1CYX	-0.21759144	0.815575429	0.443606651	2.771885579	2.813524689
Aradu.WB7Y4	0.150559677	2.608809243	0.918386234	2.15704371	2.321928095
Aradu.X0KMT	2.003602237	1.545968369	1.871843649	1.66448284	1.794935663
Aradu.Z7FZQ	-2.18442457	-2.94341647	-2.73696559	0.970853654	0.713695815
Aradu.XBR9P	3.626439137	4.437627248	4.650764559	4.564378169	4.231893162
Aradu.G7BFP	-2	-1.47393119	-2.05889369	0.782408565	0.722466024
Aradu.GKD84	3.965322548	4.444269502	4.239550797	4.989139007	5.003152449
Aradu.M7K9G	0.422233001	0.526068812	0.526068812	2.59454855	2.10433666
Aradu.ZQ118	1.855989697	1.819668183	0.910732662	4.000901403	4.173926932
Aradu.QK77V	-0.88896869	-0.04394335	-0.55639335	-0.25153877	-0.30400619
Aradu.W9TQV	1.09085343	1.98550043	0.86393845	3.699329526	4.051372102
Aradu.26IB4	-2.47393119	-1.18442457	-2	0.650764559	1.687060688
Aradu.HR3NA	2.087462841	2.160274831	2.304511042	0.650764559	0.632268215
Aradu.2BE4F	2.897240426	2.914564523	3.033863452	3.516015147	3.897240426
Aradu.8C0WH	2.375734539	2.959770155	2.794935663	2.759155834	2.974529312
Aradu.1M9DY	NA	NA	NA	NA	-5.64385619
Aradu.TP7W2	NA	NA	NA	NA	NA
Aradu.X0HV8	0.807354922	0.90303827	0.970853654	0.5360529	1.292781749
Aradu.J5HIY	5.488964817	5.526381863	5.131754091	6.595443985	6.607478275
Aradu.95REC	2.169925001	2.124328135	1.859969548	1.879705766	2.462052319

Aradu.1M38D	NA	NA	NA	NA	-3.64385619
Aradu.B4FEF	4.220329955	5.116863758	5.505255801	2.10433666	3.017921908
Aradu.AW1PY	-0.86249648	1.201633861	-1.94341647	4.469234794	4.488000771
Aradu.TK1NR	-5.64385619	-1.78587519	-4.05889369	-1.83650127	-2.05889369
Aradu.D7Y0Z	0.799087306	1.316145742	1.021479727	1.59454855	2.869871406
Aradu.4L5V2	3.040892431	3.049630768	2.553360503	4.152183419	3.937344392
Aradu.I7LA7	3.075532631	3.241840184	3.250961574	2.232660757	2.367371066
Aradu.BR0NQ	2.666756592	3.065227623	2.761285273	3.049630768	3.240314329
Aradu.H40D4	-5.64385619	-1.83650127	-2.94341647	-1.64385619	-1.51457317
Aradu.FNM4G	-0.45403163	-0.4150375	-0.91593574	0.389566812	0.790772038
Aradu.B71RC	-1.83650127	-2	-2.94341647	-1.15200309	2.13422094
Aradu.G5P3T	1.922197848	2.572889668	2.589763487	1.195347598	1.618238656
Aradu.792W7	NA	-6.64385619	-6.64385619	-0.59946207	-1.39592868
Aradu.KH972	5.323370069	6.369466484	5.838951767	7.777090896	7.640678764
Aradu.LQK8F	5.105175192	5.096767855	5.153400028	3.316145742	3.436961338
Aradu.T6XMF	-4.32192809	-3.83650127	-3.83650127	-4.32192809	-2.73696559
Aradu.V5XVE	-5.64385619	-5.64385619	-5.05889369	NA	-3.64385619
Aradu.0CM87	3.876762491	4.696550301	4.482202926	2.070389328	2.97819563
Aradu.3SD99	3.196921734	3.249445341	3.50589093	3.288358562	3.609991295
Aradu.PL5Z6	NA	NA	NA	NA	NA
Aradu.B1C6F	NA	-5.64385619	NA	NA	NA
Aradu.RM26Y	5.116031993	4.888012585	4.477677328	6.118733476	6.067810784
Aradu.FFY3	1.448900951	1.097610797	1	-0.39592868	2.748461233

F					
Aradu.6CQ40	1.201633861	1.007195501	1.124328135	1.500802053	1.944858446
Aradu.JK5YQ	1.49057013	1.89917563	1.485426827	2.269033146	2.767654798
Aradu.94BIF	-0.05889369	1.531069493	1.321928095	2.459431619	2.959770155
Aradu.FZ0BJ	1.735522177	1.757023247	1.735522177	1.321928095	1.604071324
Aradu.Z3EIC	2.384049807	2.942983598	2.669026766	2.901108243	3.234194723
Aradu.HS8FV	1.163498732	1.641546029	1.521050737	3.604071324	3.415488271
Aradu.CX1PI	2.23878686	3.070389328	3.206330648	2.634593268	2.673556424
Aradu.03ESY	3.220329955	3.272023189	3.314696526	3.384049807	3.852997588
Aradu.14U3F	0.344828497	1.859969548	0.014355293	2.817623258	2.912649865
Aradu.SN12S	-0.35845397	0.847996907	0.014355293	2.025028794	2.14404637
Aradu.AT44H	0.622930351	0.933572638	0.59454855	2.408711861	2.389566812
Aradu.LWF57	4.887525271	5.406672716	4.597531174	6.700717133	6.477191599
Aradu.XX2R4	-5.64385619	-5.05889369	-4.64385619	-3.83650127	-3.83650127
Aradu.89XIU	-0.43440282	-0.23446525	-0.30400619	0.5360529	0.855989697
Aradu.S591T	NA	NA	NA	NA	NA
Aradu.TL8H2	3.950468414	4.146492307	4.054848477	4.441616269	4.934044647
Aradu.DWS1F	-0.94341647	-0.43440282	-0.62148838	-1.28630419	-0.59946207
Aradu.X3LUW	1.521050737	1.773996325	1.560714954	-0.43440282	-0.28630419
Aradu.N42W4	2.659924558	2.042644337	2.304511042	2.715893371	3.165107985
Aradu.C3YLC	-3.32192809	-2.83650127	-3.64385619	0.150559677	-0.37706965
Aradu.9ID7S	2.592158002	2.904965719	2.50589093	0.286881148	0.201633861
Aradu.RF5XH	1.232660757	1.959770155	1.5360529	2.584962501	2.833902077

Aradu.B34AL	1.827819025	2.269033146	1.941106311	3.292781749	3.258518925
Aradu.RF6XK	0.807354922	1.007195501	1.10433666	1.646162657	1.992768431
Aradu.1R5WG	2.64385619	3.193771743	3.158660175	2.23572706	2.580145484
Aradu.JT1K4	1.752748591	2.14404637	1.887525271	1.867896464	2.007195501
Aradu.MC16W	1.220329955	1.422233001	1.063502942	1.786596362	1.90303827
Aradu.0TS71	0.333423734	0.214124805	-0.05889369	2.46727948	2.229587923
Aradu.39755	NA	-0.73696559	-0.76121314	-3.32192809	NA
Aradu.CBB8Q	-5.64385619	NA	NA	-3.18442457	-0.97143085
Aradu.ER4SD	3.564378169	3.804260116	3.806324057	4.066950244	4.524189078
Aradu.TC1A2	-3.83650127	NA	-4.32192809	NA	-4.64385619
Aradu.IPN47	0.298658316	0.839959587	0.669026766	0.565597176	1.084064265
Aradu.FWL77	-0.23446525	0.575312331	0.014355293	2.319039816	2.482848283
Aradu.QII1E	NA	-4.64385619	-4.32192809	-4.64385619	-5.05889369
Aradu.HUV25	-0.94341647	-0.35845397	-1.64385619	0.516015147	0.933572638
Aradu.G0FGB	4.583158004	4.19061486	3.992768431	2.397802962	2.475084883
Aradu.VL23N	2.944858446	3.171527106	3.231125158	1.495695163	2.100977648
Aradu.L7ZR8	0.321928095	1.111031312	-0.62148838	3.876762491	4.06608919
Aradu.QZ44H	2.650764559	3.026800059	2.869871406	3.411426246	3.54225805
Aradu.GUH8G	1.432959407	2.017921908	1.925999419	3.054848477	3.19061486
Aradu.QS26M	3.22650853	3.649615459	3.502075956	4.186659017	4.694880193
Aradu.V71C6	3.699329526	3.363171077	3.386810946	3.443606651	3.121015401
Aradu.SH4VE	3.631104282	4.152994605	3.95419631	4.722466024	4.795974694

Aradu.XW1R1	2.548436625	2.632268215	2.454175893	2.427606173	2.718087584
Aradu.483RE	1.378511623	1.933572638	1.682573297	2.411426246	2.666756592
Aradu.V32T3	-1.18442457	-1.51457317	-1.64385619	0.137503524	-0.68965988
Aradu.VC25Y	-5.64385619	-5.05889369	-4.64385619	-1.73696559	-0.37706965
Aradu.6S06R	1.09085343	1	1.189033824	3.23878686	3.340562269
Aradu.6YX1J	-1.88896869	-1.08926734	-2.32192809	1.459431619	2.03562391
Aradu.SRM8N	0.028569152	0.422233001	0.028569152	1.855989697	1.803227036
Aradu.NS08D	-0.76121314	0.298658316	0.056583528	2.871843649	2.803227036
Aradu.RFH8Y	4.106013238	4.105175192	3.667892125	4.46727948	4.217230716
Aradu.7Y76M	2.879705766	3.017921908	2.22650853	4.425593578	4.984133595
Aradu.NB0W9	2.392317423	2.438292852	2.060047384	3.881664619	3.689299161
Aradu.M5AZ6	5.69849632	6.257953495	5.881419908	8.34881666	8.925643447
Aradu.V6ZCM	1.22650853	2.010779839	1.967168608	2.3305584	2.523561956
Aradu.1L0UP	2.680324357	2.797012978	2.634593268	2.477677328	2.761285273
Aradu.VDD5S	1.422233001	1.673556424	1.63691458	2.835924074	3.220329955
Aradu.EX30Z	4.937344392	6.174126737	6.002027365	5.6622055	5.905206469
Aradu.2LE9C	2.62058641	3.389566812	2.815575429	4.155425432	4.265286858
Aradu.WF5M4	3.627606838	3.523561956	3.411426246	4.315421316	4.526694846
Aradu.WMN78	2.319039816	2.678071905	2.321928095	3.12763328	2.925999419
Aradu.M1JE3	NA	NA	NA	NA	NA
Aradu.49JIJ	5.466300829	5.289465631	5.020146573	5.630813152	5.734167661
Aradu.MBU55	3.910732662	4.529196268	3.87774425	5.465321514	5.542567608

Aradu.W9ER9	1.941106311	2.232660757	2.247927513	2.861955364	3.232660757
Aradu.ZSZ74	4.10349764	3.777156666	3.817623258	1.443606651	1.49057013
Aradu.S0DLC	-4.64385619	-4.64385619	-3.64385619	-4.32192809	-5.05889369
Aradu.69H8E	3.117695043	3.321928095	3.423578171	3.368768349	3.699329526
Aradu.N1D5Q	NA	-4.64385619	-5.64385619	-3.32192809	-3.83650127
Aradu.6Y81Q	3.812498225	4.115199749	3.793895883	4.385431037	4.82984956
Aradu.GL73Z	1.176322773	1.169925001	1.070389328	1.007195501	0.963474124
Aradu.94WDB	-2.05889369	-2.25153877	-1.73696559	-2.83650127	-2.47393119
Aradu.473E6	1.304511042	1.286881148	1.298658316	1.475084883	1.941106311
Aradu.KNB1Y	NA	-6.64385619	NA	NA	0.111031312
Aradu.7M5RR	1.292781749	1.922197848	1.59454855	2.124328135	2.23878686
Aradu.D7HWB	2.229587923	2.10433666	2.169925001	2.100977648	2.669026766
Aradu.0RQ1E	-5.64385619	-6.64385619	NA	-4.32192809	-5.05889369
Aradu.70U4X	NA	NA	NA	NA	NA
Aradu.50M8D	3.384049807	4.069530325	3.853995647	2.737686761	2.807354922
Aradu.QS20K	NA	NA	NA	-5.05889369	-3.32192809
Aradu.KV87W	-2	-1.68965988	-2.55639335	-1.43440282	-0.68965988
Aradu.28IY3	-3.47393119	-2.94341647	-2.94341647	-2.12029423	-2.73696559
Aradu.YU7JM	-1.18442457	-0.39592868	-0.577767	1.14404637	0.895302621
Aradu.KY6W5	3.707082992	3.969933275	3.56924803	4.383358695	4.00270252
Aradu.D6JKJ	-3.05889369	-3.32192809	-4.32192809	-0.15200309	0.622930351
Aradu.R2082	-1.25153877	-2.05889369	-0.37706965	1.427606173	3.201633861

Aradu.ZAF9Y	2.526068812	2.313245852	2.260025656	2.097610797	2.713695815
Aradu.L75IP	-1.73696559	-1.28630419	-2.18442457	1.580145484	1.618238656
Aradu.23E3L	0.5360529	0.22650853	0.59454855	-4.64385619	-1.02914635
Aradu.D0JN5	-3.64385619	-2.39592868	-2.55639335	-4.64385619	-2.32192809
Aradu.8G93R	3.502075956	3.857980995	3.622930351	1.063502942	1.891419187
Aradu.WT71X	2.606442228	3.09085343	2.726831217	1.794935663	1.967168608
Aradu.ZJM47	0.454175893	0.40053793	0.163498732	2.319039816	2.722466024
Aradu.PW4PJ	-4.64385619	-5.05889369	-5.64385619	-3.32192809	-0.64385619
Aradu.24DGD	2.861955364	2.889473543	2.742006211	3.171527106	3.743084056
Aradu.R3T9A	2.347665656	2.510961919	2.493134922	1.944858446	2.241840184
Aradu.1E0KB	4.409390936	4.74899785	4.310340121	3.289834465	3.427606173
Aradu.QHA6Y	NA	NA	NA	NA	-5.64385619
Aradu.8S28F	NA	NA	NA	NA	-2.25153877
Aradu.I4L9J	5.399854674	5.026357447	5.077670274	2.23878686	2.666756592
Aradu.QQB0D	1.49057013	2.275007047	2.017921908	2.702657543	2.887525271
Aradu.W5GQC	0.189033824	0.485426827	0.056583528	2.792855352	4.276496666
Aradu.IDN32	3.995484519	3.853995647	3.765534746	3.595742339	3.450221496
Aradu.C31L4	-0.86249648	-0.02914635	-0.39592868	-0.07400058	0.464668267
Aradu.JL8FJ	-0.26881676	-0.62148838	-1.05889369	0.722466024	1.077242999
Aradu.Q86YJ	-0.55639335	0.422233001	0.411426246	4.054848477	3.671293372
Aradu.LHL11	2.232660757	3.054848477	2.752748591	3.444932049	3.528571319
Aradu.N5F6J	-0.78587519	-0.83650127	-0.88896869	3.29130886	3.683696454

Aradu.K0HFM	1.937344392	2.333423734	2.056583528	3.319039816	3.388189537
Aradu.A43U5	-5.64385619	-4.32192809	-4.32192809	0.378511623	1.580145484
Aradu.YV75B	1.244887059	1.843983844	1.63691458	2.214124805	2.364572432
Aradu.Y4AJ0	NA	-6.64385619	NA	NA	NA
Aradu.D48W8	-0.15200309	0.014355293	-0.16812276	-1.05889369	-0.76121314
Aradu.U6IZ8	1.232660757	1.339137385	1.250961574	1.722466024	2.182692298
Aradu.L9IYH	1.454175893	0.895302621	1.021479727	1.875780063	2.195347598
Aradu.R4FBZ	0.454175893	0.704871964	0.871843649	-3.64385619	-3.47393119
Aradu.HX36X	-3.32192809	0	0.948600847	-1.32192809	-1.88896869
Aradu.FJ6K3	-0.21759144	0.40053793	0.028569152	0.669026766	0.963474124
Aradu.70QSY	2.653060017	3.343407822	3.766595162	2.577730931	2.411426246
Aradu.DVQ9A	NA	-6.64385619	NA	-5.64385619	0
Aradu.FFW2J	6.141800612	5.996388746	5.807354922	3.270528942	3.155425432
Aradu.YIQ80	1.310340121	4.116863758	4.532940288	0.584962501	2.871843649
Aradu.F06JI	1.443606651	1.608809243	1.3950628	2.350497247	2.59454855
Aradu.88CYL	8.141238626	7.282903083	7.566130168	1.883620816	2.182692298
Aradu.41EZW	1.963474124	1.952333566	1.944858446	2.201633861	2.464668267
Aradu.K44L7	2.901108243	2.443606651	2.792855352	3.078951341	3.106013238
Aradu.ECS2U	NA	NA	NA	NA	NA
Aradu.JQ0FN	-0.18442457	0.22650853	-0.12029423	-0.76121314	0.028569152
Aradu.PXT5K	-0.51457317	-0.4150375	-0.76121314	0.669026766	1.097610797
Aradu.9G825	-2.18442457	-0.55639335	-1.21759144	5.243364426	4.286141873

Aradu.1BC5C	NA	-4.32192809	-3.83650127	-0.45403163	-0.20091269
Aradu.EYQ2A	3.549669171	3.99004722	3.463360886	3.727920455	4.289834465
Aradu.Z3NVH	-3.83650127	-1.28630419	-1.28630419	-0.47393119	0.422233001
Aradu.RYS1S	-4.32192809	0.014355293	0.321928095	-2.39592868	-1.83650127
Aradu.G6N6R	3.496973581	3.347665656	3.234194723	3.129283017	3.436961338
Aradu.C6SJS	4.934044647	5.13422094	4.668459557	5.76420813	5.606146078
Aradu.97X4A	1.389566812	1.344828497	1.007195501	1.967168608	2.244887059
Aradu.PM3IW	1.851998837	2.344828497	1.967168608	3.350497247	3.760220946
Aradu.RE8D2	-3.18442457	-0.88896869	-1.64385619	-2	-0.78587519
Aradu.LF76F	4.685379552	4.66618849	4.465321514	2.198494154	2.488000771
Aradu.3JN5I	0.739848103	1.722466024	0.189033824	3.012568674	2.981852653
Aradu.U838H	NA	-5.64385619	NA	NA	-4.64385619
Aradu.H5024	6.019034669	5.809157165	5.660209873	3.140778656	3.3950628
Aradu.B1W2E	3.964398632	4.642123916	4.382667253	3.785550552	3.473786912
Aradu.KI06N	5.210622644	5.396433531	5.089582893	5.41040895	5.252476214
Aradu.5R3CV	2.580145484	3.63691458	2.217230716	5.296457407	5.217230716
Aradu.C0ZT2	0.956056652	1.98550043	1.735522177	2.397802962	2.604071324
Aradu.U5BST	1.550900665	2.648465443	2.538538164	2.63691458	2.788685711
Aradu.Z06PE	-3.18442457	-3.32192809	-5.64385619	-2.18442457	0.22650853
Aradu.M5V2I	5.833649128	5.892148128	5.820178962	2.950468414	2.875780063
Aradu.HH0H8	-1.02914635	0.028569152	-0.76121314	1.673556424	1.827819025
Aradu.E1NAR	1.959770155	2.443606651	2.073820233	2.304511042	2.570462931

Aradu.44Z6R	0.782408565	0.757023247	0.704871964	0.495695163	0.782408565
Aradu.6T9HL	0.695993813	1.286881148	0.933572638	1.86393845	1.111031312
Aradu.EAQ74	-0.05889369	0.604071324	-0.12029423	2.207892852	2.324810603
Aradu.JV5EQ	-0.68965988	0.757023247	-0.07400058	0.731183242	1.23878686
Aradu.DV3N6	1.682573297	2.538538164	2.185866545	3.382667253	3.458119481
Aradu.7Z2RH	1.124328135	1.367371066	1.10433666	2.253989266	3.381283373
Aradu.L6R4W	NA	-6.64385619	-6.64385619	-5.64385619	-5.05889369
Aradu.B1FJV	-2.64385619	-3.05889369	-4.05889369	-2.73696559	1.98550043
Aradu.RU466	1.384049807	1.952333566	2.066950244	2.485426827	3.862947248
Aradu.SW9YM	-3.83650127	NA	-6.64385619	NA	-0.81096618
Aradu.CL5YM	-0.35845397	-0.05889369	-1.15200309	2.339137385	2.765534746
Aradu.I4X46	6.189626916	3.997292408	4.257764969	2.477677328	3.150559677
Aradu.70H43	4.789207575	4.566815154	4.662775172	2.918386234	3.122672719
Aradu.AP9TH	NA	NA	NA	NA	-5.64385619
Aradu.G5WQA	1.678071905	2.64385619	1.925999419	4.052241981	4.319039816
Aradu.46IYL	1.15704371	1.3950628	1.438292852	2.580145484	2.797012978
Aradu.ND96S	1.280956314	1.298658316	0.799087306	0.82374936	0.669026766
Aradu.K8DY2	-2.55639335	-1.35845397	-4.32192809	1.23878686	1.63691458
Aradu.YSN3X	3.119356177	3.688180359	3.317593505	4.231893162	4.792334806
Aradu.K1MHV	-0.4150375	-0.71311885	-1	0.137503524	0.731183242
Aradu.T346F	3.349082146	3.350497247	2.959770155	1.765534746	2.150559677
Aradu.H83MI	5.809414444	5.879950768	5.798309782	5.477677328	5.5055734

Aradu.3RP7R	3.063502942	2.883620816	3.017921908	2.160274831	2.46727948
Aradu.Q3FMD	2.408711861	2.64385619	2.283921772	3.62058641	4.092545742
Aradu.YMD6U	-2.32192809	-1.88896869	-1.68965988	-0.32192809	0.298658316
Aradu.UT07K	-0.13606155	0.35614381	-0.97143085	1.545968369	1.316145742
Aradu.T82FQ	-2.47393119	-2	-2.64385619	-0.94341647	-0.81096618
Aradu.XK5C3	2.469885976	2.855989697	2.726831217	2.879705766	3.481557281
Aradu.P82NP	-4.32192809	-3.83650127	-2.64385619	NA	-2.64385619
Aradu.KYE4X	-3.32192809	-4.32192809	-3.64385619	-3.83650127	1.350497247
Aradu.Y6FFI	4.001802243	4.402585758	4.123500664	5.010332283	5.467931546
Aradu.83CAT	-0.55639335	-0.76121314	-1.35845397	1.03562391	1.794935663
Aradu.72HG2	2.650764559	4.200064862	4.982765463	4.151371776	3.66448284
Aradu.Z4K97	0.555816155	0.632268215	0.722466024	2.646162657	2.689299161
Aradu.09JHP	-4.64385619	-4.32192809	-3.64385619	-2.32192809	-1.15200309
Aradu.NG5IQ	-0.25153877	0.084064265	-0.83650127	0.871843649	1.50589093
Aradu.8F49F	-0.68965988	-0.16812276	-0.51457317	2.419538892	2.560714954
Aradu.ZD05E	NA	-4.32192809	-6.64385619	NA	-3.64385619
Aradu.E4N51	3.137503524	3.430285273	3.297191417	4.181102551	5.025471815
Aradu.212BQ	2.963474124	2.722466024	2.675815931	3.263034406	3.672425342
Aradu.XGC1C	-3.05889369	-1.78587519	-3.32192809	-0.86249648	-0.81096618
Aradu.VQ2JX	2.503348735	2.510961919	2.49057013	2.272023189	2.384049807
Aradu.D9IA6	2.780310099	3.023255352	2.765534746	3.063502942	3.261530815
Aradu.QDB5N	-0.15200309	0.028569152	-0.53533173	2.992768431	4.566815154

Aradu.W75H1	1.855989697	1.650764559	1.790772038	2.014355293	2.580145484
Aradu.PW6QG	NA	-5.05889369	NA	-4.32192809	-4.32192809
Aradu.910RR	1.480265122	1.580145484	1.316145742	3.264536431	3.658782734
Aradu.ZJT5U	-6.64385619	-5.05889369	-5.05889369	-2.64385619	-1.47393119
Aradu.W1XFV	1.713695815	2.250961574	1.879705766	1.765534746	1.765534746
Aradu.F08JS	2.289834465	2.608809243	2.704871964	2.432959407	2.039138394
Aradu.ZZ9EE	-0.39592868	0.443606651	0.176322773	-2.12029423	-1.94341647
Aradu.U8CR2	-1.68965988	-1.88896869	-1.28630419	-2.32192809	-2.05889369
Aradu.00XL9	3.906890596	4.217230716	3.99004722	5.457134594	5.690137693
Aradu.YC3RY	1.769771739	2.182692298	2.114367025	3.751677946	3.991861931
Aradu.0924G	NA	NA	NA	NA	NA
Aradu.VMK7V	-1.39592868	-2.25153877	-2.94341647	-1.21759144	1.3950628
Aradu.JZT16	4.5987225	4.004501392	4.098453246	-0.28630419	1.475084883
Aradu.FP3UD	3.003602237	2.914564523	2.82374936	3.527320608	4
Aradu.5SD5G	2.23572706	2.361768359	1.967168608	2.843983844	3.608809243
Aradu.A9CNL	4.00270252	3.925049965	3.816599707	3.023255352	3.193771743
Aradu.BB0Q9	0.98550043	1.361768359	1.14404637	2.23572706	2.275007047
Aradu.MW4MQ	-4.32192809	-3.83650127	-1.78587519	-4.32192809	-2.83650127
Aradu.15QSN	-0.18442457	-0.18442457	-0.15200309	1.077242999	1.195347598
Aradu.8N3KD	1.321928095	1.220329955	1.028569152	2.292781749	2.601696516
Aradu.YP8K9	-3.47393119	-4.05889369	-2.94341647	-1.83650127	1.480265122
Aradu.ZR16S	-6.64385619	-5.64385619	NA	NA	-4.32192809
Aradu.B9ZH	1.847996907	2.869871406	2.500802053	3.046141782	2.98550043

B					
Aradu.WRA79	-0.94341647	0	0.678071905	-1.43440282	-0.43440282
Aradu.V9D7S	6.6395216	6.459759467	6.735522177	4.775577361	4.305240966
Aradu.K952B	0	1.137503524	0.201633861	2.13093087	2.35614381
Aradu.5U8HG	5.177917792	5.197314999	5.024142346	5.740657772	5.864681406
Aradu.AV6RW	0.526068812	1.207892852	0.5360529	1.739848103	1.875780063
Aradu.MCA4R	1.14404637	2.773996325	0.970853654	3.182692298	3.021479727
Aradu.JI3DJ	-1.55639335	-1.28630419	-1.08926734	3.853995647	3.62058641
Aradu.M4V5T	0.757023247	1.263034406	1.09085343	1.883620816	2.021479727
Aradu.9A69L	4.952333566	4.581953751	4.725195817	1.82374936	2.560714954
Aradu.KY185	-3.32192809	-4.32192809	-3.64385619	-2.83650127	-0.59946207
Aradu.31RFE	2.715893371	2.582556003	2.550900665	1.500802053	1.304511042
Aradu.7V00N	0.111031312	1.137503524	0.565597176	1.778208576	2.295723025
Aradu.E8PJN	-3.47393119	-2.25153877	-1.83650127	-0.49410907	0.214124805
Aradu.I32AI	-2.32192809	-3.18442457	-2.47393119	-0.71311885	-1.02914635
Aradu.NAE9S	2.538538164	2.803227036	2.46727948	2.629939409	2.87774425
Aradu.WQP25	NA	-6.64385619	NA	NA	NA
Aradu.02I1H	-3.05889369	-2.55639335	-4.32192809	0.367371066	0.516015147
Aradu.F2CY5	0.40053793	0.713695815	0.475084883	0.250961574	0.622930351
Aradu.V1JTR	3.989139007	4.378511623	4.13422094	4.913607512	5.02103548
Aradu.JR9Q4	2.695993813	3.112700133	2.618238656	3.327687364	3.678071905
Aradu.Z330P	-1.39592868	-3.18442457	-4.05889369	-1.18442457	-1.73696559
Aradu.T12X	0.575312331	0.956056652	0.22650853	2.937344392	3.277984747

R					
Aradu.J16T3	3.09423607	3.155425432	2.786596362	3.654206378	4.292045492
Aradu.80C88	1.974529312	2.378511623	2	2.61117238	2.965322548
Aradu.SSI0J	NA	-6.64385619	NA	-5.64385619	-3.18442457
Aradu.9DJ84	3.558267634	3.615887074	3.308885057	1.981852653	2.59454855
Aradu.FN83T	-4.64385619	-6.64385619	NA	-5.64385619	-6.64385619
Aradu.NM2TY	NA	NA	NA	NA	-5.64385619
Aradu.5L39Q	0.526068812	0.516015147	0.310340121	0.757023247	1.117695043
Aradu.H0BW9	-4.05889369	-4.32192809	-5.05889369	-6.64385619	-5.05889369
Aradu.4P1MR	-3.05889369	-3.05889369	-3.83650127	-0.51457317	0.014355293
Aradu.7KC0A	0.464668267	0.389566812	-0.94341647	1.232660757	1
Aradu.JLT7Z	-0.35845397	-0.39592868	-0.78587519	0.344828497	0.815575429
Aradu.71U9H	3.568032105	3.981852653	3.632268215	4.579542225	4.772941338
Aradu.6GD1V	3.017921908	3.447579197	2.916476644	4.21023299	4.135042286
Aradu.FW1J1	2.386810946	2.702657543	2.508428653	2.797012978	3.122672719
Aradu.UE6Z2	1.201633861	1.49057013	1.232660757	2.250961574	2.680324357
Aradu.GN3K0	-5.05889369	NA	NA	-5.05889369	-1.18442457
Aradu.CN1WR	1.195347598	1.678071905	1.384049807	2.182692298	2.44625623
Aradu.105GZ	1.695993813	2.053111336	2.039138394	1.883620816	2.266036894
Aradu.J6DQU	NA	-5.64385619	NA	-1.21759144	-2.39592868
Aradu.29QNA	2.456806149	2.675815931	1.914564523	3.016139703	2.998195503
Aradu.93CHA	1.232660757	0.659924558	0.722466024	0.526068812	1.03562391
Aradu.U0KK	0.275007047	2.718087584	1.803227036	1.807354922	2.053111336

7					
Aradu.QSW68	3.357552005	3.046141782	2.480265122	2.861955364	2.82984956
Aradu.E26HT	-0.97143085	0.124328135	-2.32192809	1.310340121	2.427606173
Aradu.EFX4S	4.241077458	4.178714641	4.0721058	3.56924803	3.428946345
Aradu.4AK3M	1	-0.59946207	0.23878686	NA	-4.32192809
Aradu.YTJ16	2.906890596	3.192194165	2.722466024	3.99095486	4.159467729
Aradu.R71CC	3.310340121	3.163498732	3.08236197	2.845991771	2.538538164
Aradu.91876	NA	NA	NA	NA	NA
Aradu.TKG8T	-0.28630419	-0.15200309	-0.59946207	2.304511042	1.786596362
Aradu.H0NY1	3.47118746	3.361768359	3.10433666	3.177917792	3.267535798
Aradu.HL6TS	4.328405659	4.169123281	4.406672716	2.724650272	3.070389328
Aradu.7J6XP	6.629648044	6.987434567	6.924693758	2.073820233	3.19061486
Aradu.YS8K9	2.070389328	2.646162657	2.046141782	2.163498732	2.498250868
Aradu.E9HU8	2.367371066	2.759155834	2.558267634	2.879705766	3.454175893
Aradu.8479S	1.531069493	1.384049807	1.182692298	1.744161096	2.375734539
Aradu.3MG6J	1.500802053	1.974529312	1.584962501	2.788685711	2.698218478
Aradu.2QR5U	1.361768359	2.080657663	1.207892852	1.970853654	2.166715445
Aradu.YD7VI	1.182692298	1.744161096	1.782408565	2.737686761	3.384049807
Aradu.R4MYV	2.042644337	2.568032105	1.992768431	3.679198571	3.967168608
Aradu.DKR1D	2.23878686	2.347665656	1.867896464	2.12763328	1.584962501
Aradu.1Y370	NA	NA	NA	NA	NA
Aradu.AA0QS	NA	-4.64385619	-3.32192809	-0.81096618	-1.39592868
Aradu.R8LXW	4.300855871	4.567423758	4.555202634	4.553974808	4.557042415

Aradu.RQ7SQ	3.679198571	3.772941338	3.261530815	3.084064265	3.379898164
Aradu.200KX	3.59812696	3.889473543	3.426264755	4.518535139	4.477029653
Aradu.9MZ2X	NA	-6.64385619	-4.64385619	NA	-5.05889369
Aradu.SI3S6	-0.64385619	-0.88896869	-0.86249648	1.718087584	0.879705766
Aradu.4M66F	-0.49410907	0	-0.12029423	0.50589093	1.111031312
Aradu.XYA4T	NA	NA	NA	NA	NA
Aradu.64JV5	-1.39592868	-1.73696559	-2.73696559	-4.64385619	-1.94341647
Aradu.VFN3G	-1.35845397	-0.81096618	-1.55639335	0.378511623	0.650764559
Aradu.2JQ32	1.286881148	1.3950628	1.584962501	2.247927513	2.40053793
Aradu.7P38Z	1.632268215	1.815575429	1.448900951	2.797012978	2.726831217
Aradu.HJ2Y0	-5.64385619	NA	NA	0.263034406	1.608809243
Aradu.M89U9	-4.05889369	-4.32192809	-5.05889369	-1.28630419	-1.12029423
Aradu.I4TMG	3.909773104	3.264536431	3.114367025	3.572889668	4.018812186
Aradu.RDW4B	1.50589093	1.859969548	1.378511623	3.495695163	3.508428653
Aradu.Q2NGG	NA	NA	NA	-6.64385619	-6.64385619
Aradu.S2S1T	-2.55639335	-2.64385619	-3.47393119	-1.21759144	-0.91593574
Aradu.XU97Z	0.765534746	1.176322773	0.97819563	1.5360529	2.244887059
Aradu.M4YAD	NA	-6.64385619	-5.05889369	NA	-4.64385619
Aradu.IHP1V	3.609991295	3.764473551	3.588564737	4.207892852	4.682573297
Aradu.JM0ZT	1.232660757	0.613531653	1.097610797	0.176322773	1.021479727
Aradu.11960	2.596935142	2.811471031	2.40053793	3.320484678	3.596935142
Aradu.7MF12	NA	-5.05889369	-3.64385619	NA	NA

Aradu.0FH1C	2.304511042	2.61117238	2.408711861	3.367371066	3.482848283
Aradu.WJZ76	-0.51457317	-0.64385619	-0.97143085	-0.10469738	0.454175893
Aradu.L5EJ3	4.019701914	4.015247774	3.804260116	0.847996907	1.014355293
Aradu.D24R3	-0.86249648	-0.07400058	-0.30400619	1.250961574	1.03562391
Aradu.TF2FR	-3.83650127	-2.94341647	-4.64385619	-2.55639335	-2.94341647
Aradu.UJ8BX	2.353323291	2.516015147	2.22650853	3.0721058	3.493134922
Aradu.5ZT49	NA	NA	NA	NA	NA
Aradu.VVR1U	1.269033146	0.879705766	0.650764559	2.765534746	2.950468414
Aradu.8I2Q1	0.250961574	0.97819563	0.545968369	1.641546029	2.063502942
Aradu.G7UXI	-2.73696559	-2.18442457	-2.47393119	-1.28630419	0.704871964
Aradu.AM9WK	3.150559677	2.970853654	2.765534746	2.250961574	2.577730931
Aradu.AG70H	NA	NA	NA	NA	NA
Aradu.J8H2F	1.575312331	1.469885976	1.510961919	0.687060688	0.411426246
Aradu.A90PK	-5.64385619	-6.64385619	-5.05889369	-2.94341647	2.480265122
Aradu.WS4CD	NA	-4.05889369	-4.64385619	-2.32192809	-0.02914635
Aradu.9E2AM	1.207892852	1.049630768	0.887525271	1.769771739	1.90303827
Aradu.QVN0R	1.269033146	1.589763487	0.871843649	4.872336288	4.770829046
Aradu.IFP6S	1.443606651	1.807354922	1.967168608	1.244887059	1.333423734
Aradu.EZK5X	-0.20091269	-0.4150375	-0.577767	0.622930351	0.831877241
Aradu.232FJ	-4.64385619	-5.05889369	-5.64385619	NA	-6.64385619
Aradu.W51SD	2.889473543	3.070389328	2.857980995	3.894332742	3.994579724
Aradu.87LE7	2.750606505	2.908812908	2.572889668	2.933572638	2.799087306

Aradu.AA5CG	2.891419187	2.95419631	2.833902077	2.558267634	3.234194723
Aradu.4T0IX	4.735522177	5.264911693	4.489286023	6.422737587	6.276310547
Aradu.K75LB	4.891419187	4.910732662	4.819157224	3.316145742	3.279471296
Aradu.GJB7M	-2.94341647	-1.35845397	-2.83650127	1.526068812	1.14404637
Aradu.463D4	4.031218731	4.377817853	4.233427944	4.778208576	4.705425039
Aradu.BB6TM	-0.28630419	0.201633861	-1	2.851998837	3.150559677
Aradu.EB4A5	-2.73696559	-3.83650127	-4.05889369	-4.32192809	0.815575429
Aradu.ZC6SM	2.748461233	3.427606173	3.166715445	3.698218478	3.929790998
Aradu.TH1E7	1.748461233	1.570462931	1.769771739	-2.47393119	-1.15200309
Aradu.PT6UP	-2.83650127	-3.32192809	-2.83650127	-3.64385619	-3.32192809
Aradu.I69M4	1.859969548	2.702657543	2.097610797	3.472487771	3.482848283
Aradu.IT4JF	-2.25153877	-1.59946207	-2.83650127	0.50589093	0.782408565
Aradu.BJL71	-5.05889369	-6.64385619	-6.64385619	NA	-6.64385619
Aradu.5CF9R	-2.32192809	-4.32192809	-3.18442457	-1.83650127	-1.47393119
Aradu.SL3YH	2.286881148	2.032100843	2.070389328	0.855989697	1.40599236
Aradu.DZY4R	-0.47393119	-0.15200309	-0.34007544	-0.08926734	-0.25153877
Aradu.W64DR	1.275007047	1.618238656	1.286881148	0.516015147	0.839959587
Aradu.DXW6X	0.495695163	-0.577767	1.275007047	5.001802243	4.856487779
Aradu.KB6LK	-1.47393119	-1.47393119	-2	-0.88896869	-0.86249648
Aradu.4IR6I	0.35614381	1.292781749	0.565597176	2.269033146	2.510961919
Aradu.3II03	NA	NA	NA	-4.64385619	-1.08926734
Aradu.13H1D	3.832890014	3.723558561	4.106850796	0.286881148	0.042644337

Aradu.I567U	-0.04394335	1.042644337	0.286881148	2.63691458	2.618238656
Aradu.B7486	-0.07400058	0.575312331	0.111031312	1.189033824	1.50589093
Aradu.LN5U5	-1.12029423	-0.91593574	-1.73696559	2.150559677	2.748461233
Aradu.254Z6	0.739848103	1.269033146	1.589763487	-1.08926734	-0.64385619
Aradu.7W9JR	-1.02914635	0.831877241	1.10433666	0.584962501	0.925999419
Aradu.GGI7I	4.099295204	4.299391206	4.48864354	3.198494154	4.869377924
Aradu.E1VP1	-0.66657627	-0.64385619	-1.08926734	1.244887059	1.378511623
Aradu.N2X1B	1.761285273	2.263034406	1.970853654	2.516015147	3.145677455
Aradu.S0SM3	0.150559677	0.584962501	0.422233001	0.344828497	1.22650853
Aradu.H9GMI	NA	NA	-6.64385619	NA	-6.64385619
Aradu.EC406	2.851998837	3.119356177	2.937344392	3.85698569	4.37920506
Aradu.070N1	NA	NA	NA	NA	-3.05889369
Aradu.YHD0I	-4.32192809	-2.83650127	-2.47393119	5.855740592	4.803227036
Aradu.3X2EP	4.266786541	4.203984166	4.065227623	4.189033824	4.323370069
Aradu.1T2VN	-3.64385619	-1.94341647	-2.94341647	-1.21759144	-0.12029423
Aradu.L3677	5.459431619	5.044831233	5.635754391	3.528571319	4.415488271
Aradu.24X5V	2.841973119	3.304511042	3.206330648	3.493134922	3.628773595
Aradu.P6RI2	NA	-6.64385619	NA	NA	NA
Aradu.RH8VE	NA	NA	NA	NA	NA
Aradu.1P8DE	-3.83650127	-4.05889369	NA	-2.73696559	1
Aradu.M1UTK	1.613531653	1.650764559	1.815575429	1.887525271	2.182692298
Aradu.8I12T	-0.4150375	-0.73696559	-0.25153877	-0.51457317	0.214124805

Aradu.K285D	6.557655155	6.367371066	6.890689878	2.813524689	2.503348735
Aradu.JKI99	3.626439137	3.833902077	3.672425342	4.271276259	4.657640005
Aradu.1V1PL	2.50589093	2.250961574	1.66448284	3.618238656	3.508428653
Aradu.VM1W8	3.384049807	3.35473424	3.063502942	2.967168608	3.148934105
Aradu.83Q97	-0.53533173	-0.25153877	-1.18442457	1.98550043	1.891419187
Aradu.DL5AX	2.596935142	2.40599236	2.232660757	2.247927513	2.319039816
Aradu.WLU86	4.221103725	4.478324711	4.360364277	4.773996325	4.765534746
Aradu.CUQ8J	-0.88896869	-1.51457317	-1.35845397	1.627606838	1.678071905
Aradu.C04VQ	-5.05889369	NA	-6.64385619	-4.64385619	-1.51457317
Aradu.8KH6E	1.263034406	4.361066489	2.700439718	0.111031312	1.974529312
Aradu.8KW68	-0.34007544	-0.51457317	-1.25153877	0.98550043	1.416839742
Aradu.JJ88W	-0.97143085	-0.53533173	-0.66657627	0.22650853	0.422233001
Aradu.H50Y7	1.90303827	2.275007047	2.039138394	2.575312331	2.378511623
Aradu.CNT80	2.912649865	2.82984956	2.432959407	1.963474124	2.386810946
Aradu.QU3KT	-5.05889369	-2.73696559	-3.83650127	3.13093087	2.974529312
Aradu.Z0X17	2.851998837	3.381283373	2.935459748	4.098453246	4.225737653
Aradu.1E1V6	0.201633861	0.933572638	0.632268215	1.691534165	1.910732662
Aradu.IG7LI	1.963474124	1.952333566	1.565597176	3.089159132	3.176322773
Aradu.D2I4Y	2.277984747	2.553360503	2.485426827	2.675815931	3.060047384
Aradu.K39P9	3.331991778	3.303050085	3.063502942	3.966245873	3.529820947
Aradu.M0XVK	NA	NA	NA	NA	NA
Aradu.FV6YV	-4.64385619	-4.64385619	-6.64385619	-4.05889369	-3.64385619

Aradu.7J9TI	NA	NA	NA	NA	-5.64385619
Aradu.6NR0H	-0.76121314	-1.15200309	-0.71311885	-5.64385619	-1.68965988
Aradu.KS3VF	5.095080492	5.095502518	4.960697039	4.154615611	4.781884235
Aradu.JK78S	1.752748591	2.40599236	2.140778656	2.553360503	2.689299161
Aradu.54M2C	NA	-5.64385619	-6.64385619	-5.64385619	-3.47393119
Aradu.V824B	0.526068812	0.250961574	0.485426827	0.871843649	1.09085343
Aradu.A2ZB2	0	-0.18442457	-0.16812276	-5.05889369	-1.39592868
Aradu.3B2AS	1.495695163	2.432959407	2.521050737	1.871843649	2.17951105
Aradu.WWM41	2.90303827	2.286881148	2.769771739	2.117695043	2.709290636
Aradu.KL6KV	4.241840184	4.06436554	4.188242656	5.193771743	5.234194723
Aradu.K3RPT	2.414135533	2.543495883	2.99095486	1.316145742	1.500802053
Aradu.F623P	0.970853654	1.021479727	0.310340121	3.221877081	3.196921734
Aradu.L8GIZ	NA	NA	NA	-3.47393119	-4.64385619
Aradu.3UE4N	4.189824559	4.516645558	4.638073837	6.445759804	6.041330607
Aradu.8Q40H	2.981852653	2.927896454	2.855989697	2.50589093	2.893362211
Aradu.P0L8F	-3.64385619	-4.32192809	-4.64385619	NA	-3.64385619
Aradu.AC9ZE	4.689858236	4.723012396	4.72900887	4.024142346	3.808385051
Aradu.1VB35	1.443606651	1.952333566	1.495695163	2.327687364	2.480265122
Aradu.XQ1XQ	3.802193217	3.504620392	3.723558561	1.613531653	1.704871964
Aradu.GWN14	-5.05889369	-3.47393119	-4.05889369	-3.32192809	-3.83650127
Aradu.V5GXH	1.084064265	1.150559677	1.056583528	0.895302621	0.941106311
Aradu.FD8VQ	3.106013238	3.464668267	3.336283388	4.375734539	4.410069692

Aradu.Z8G1Y	2.531069493	2.778208576	2.625270489	2.771885579	2.86393845
Aradu.KRJ36	-3.64385619	-1.73696559	-2.73696559	-3.64385619	4.06091205
Aradu.WP07M	4.015247774	4.336997417	4.187451054	5.773468928	5.870611313
Aradu.YRE5K	2.277984747	3.147306699	2.922197848	3.666756592	3.871843649
Aradu.4I166	0	0.378511623	-0.02914635	2.49057013	2.786596362
Aradu.W6JL4	NA	-5.64385619	-2.73696559	NA	NA
Aradu.YN99B	NA	NA	NA	NA	-3.83650127
Aradu.F6Z4G	-1.59946207	-1.55639335	-2.05889369	-0.21759144	1.150559677
Aradu.INV9V	4.659353759	4.220329955	4.408711861	1.214124805	1.659924558
Aradu.FUK65	-1.64385619	0	-0.21759144	-0.76121314	-1.05889369
Aradu.PU85I	5.14404637	4.438292852	4.866413481	0.056583528	1.731183242
Aradu.0G5T2	-1.47393119	-0.51457317	-1.28630419	2.464668267	1.922197848
Aradu.ZV9WQ	1.327687364	1.879705766	1.691534165	1.855989697	2.277984747
Aradu.8C561	-3.05889369	-2.55639335	-3.32192809	-0.30400619	0.575312331
Aradu.W98YX	2.881664619	2.572889668	2.531069493	1.959770155	2.316145742
Aradu.V92BV	-0.97143085	-0.53533173	-1.18442457	0.757023247	0.22650853
Aradu.268F1	NA	-6.64385619	NA	-4.32192809	-4.05889369
Aradu.D05FL	-2.83650127	-3.47393119	-2.47393119	-2.73696559	-2.25153877
Aradu.G04HZ	1.555816155	1.891419187	1.704871964	2.422233001	2.944858446
Aradu.JTA11	NA	-5.05889369	-5.05889369	NA	-5.05889369
Aradu.Q6RE5	-0.97143085	-0.25153877	-0.86249648	0.59454855	0.378511623
Aradu.J2BV7	-0.30400619	1.150559677	0.176322773	-1.35845397	0.056583528

Aradu.CV6FA	4.583158004	3.537296067	4.029452886	0.124328135	1.070389328
Aradu.L8GUY	2.220329955	2.475084883	2.316145742	2.280956314	2.341985747
Aradu.DEY30	1.599317794	1.782408565	1.545968369	2.160274831	2.807354922
Aradu.SQD67	-3.83650127	-6.64385619	-6.64385619	NA	-1
Aradu.YA97R	-0.05889369	0.545968369	0.389566812	1.757023247	2.185866545
Aradu.JK14X	-0.05889369	0.422233001	-1.05889369	3.854993017	3.884597921
Aradu.QYX7X	-2.55639335	-2.32192809	-3.47393119	0.201633861	0.782408565
Aradu.D0AKP	-0.86249648	-1.43440282	-0.35845397	-5.64385619	-4.05889369
Aradu.M0KDX	-4.05889369	-2.39592868	-5.05889369	0.765534746	1.028569152
Aradu.418KR	1.350497247	1.344828497	1.176322773	0.847996907	1.726831217
Aradu.2J3VX	-0.76121314	-0.12029423	-0.88896869	1.13093087	1.589763487
Aradu.5M48C	-1.25153877	-0.88896869	-2.12029423	1.327687364	1.879705766
Aradu.VY6JK	-1.02914635	0.137503524	-0.34007544	-0.13606155	1.744161096
Aradu.FA88A	1.66448284	1.438292852	1.495695163	1.280956314	1.49057013
Aradu.IZX1C	-0.71311885	-0.88896869	-1.18442457	0.604071324	0.545968369
Aradu.M8PFR	2.214124805	2.448900951	2.347665656	2.922197848	3.046141782
Aradu.02NZN	-3.05889369	-1.59946207	-6.64385619	2.925999419	2.821710215
Aradu.40GCA	-4.64385619	-2.64385619	-1.02914635	0.189033824	0.042644337
Aradu.48DYG	-2.39592868	-2.47393119	-2.47393119	-2.83650127	-2.55639335
Aradu.Y0AR8	2.182692298	2.49057013	2.060047384	3.039138394	3.275007047
Aradu.267PI	4.14974712	3.716990894	3.750606505	2.918386234	3.434294618
Aradu.AKP9L	0.722466024	0.722466024	0.516015147	1.89917563	3.037382222

Aradu.AQJ6I	0.941106311	1.263034406	0.895302621	2.528571319	2.948600847
Aradu.707Z5	-3.47393119	-3.83650127	-2.64385619	NA	2.477677328
Aradu.GPN3U	1.82374936	1.560714954	1.855989697	-0.78587519	-0.577767
Aradu.523T3	2.976363636	2.634593268	2.627606838	2.010779839	2.469885976
Aradu.DG903	1.189033824	1.704871964	1.63691458	1.35614381	1.028569152
Aradu.JFR2C	1.722466024	2.17951105	2.021479727	2.733354341	3.122672719
Aradu.QE3CA	4.059182199	4.257764969	4.105175192	4.215678597	4.366671916
Aradu.9CU4Z	-0.83650127	-0.91593574	-0.78587519	0.250961574	0.799087306
Aradu.VK692	-0.4150375	-0.20091269	-0.97143085	1.59454855	2.107687869
Aradu.EGG67	1.117695043	1.250961574	0.887525271	2.384049807	2.010779839
Aradu.R9HS1	2.974529312	3.29130886	3.089159132	3.609991295	4.12763328
Aradu.QU0CV	1.367371066	1.344828497	1.608809243	3.887525271	4.3950628
Aradu.QV2N6	2.98550043	3.173127433	2.849999259	3.969012308	3.989139007
Aradu.EM688	3.534808661	4.814037648	4.043519494	1.646162657	2.867896464
Aradu.AU35U	0.432959407	0.97819563	0.367371066	2.737686761	2.480265122
Aradu.GX867	0.555816155	0.815575429	1.14404637	1.752748591	1.790772038
Aradu.GS1LC	1.82374936	1.622930351	1.220329955	1.761285273	2.176322773
Aradu.PPM14	3.246408087	3.215678597	3.058316496	3.203201156	3.171527106
Aradu.2H1GD	2.799087306	3.390942773	4.948133578	3.744161096	2.720278465
Aradu.Q59PD	NA	NA	NA	NA	NA
Aradu.Z7W7G	-2.64385619	-2.64385619	-2.55639335	3.153805336	3.228049048
Aradu.295V7	3.746312766	5.654492826	5.999774561	2.17951105	2.414135533

Aradu.096TH	NA	NA	NA	NA	-5.05889369
Aradu.P6DLH	NA	-3.47393119	-2.83650127	-0.97143085	-1.35845397
Aradu.2Q2R1	-0.91593574	-1	-2.05889369	-1.21759144	-0.55639335
Aradu.S1HRU	2.150559677	2.803227036	2.416839742	2.871843649	2.9202933
Aradu.90S6X	NA	-6.64385619	-6.64385619	NA	NA
Aradu.Y7NUP	0.713695815	0.40053793	0.070389328	1.748461233	1.220329955
Aradu.D24Y8	2.675815931	2.392317423	3.058316496	1.443606651	1.384049807
Aradu.0QN5D	1.014355293	1.23878686	0.839959587	3.600507645	3.629939409
Aradu.JSQ7J	-0.86249648	-0.10469738	0.495695163	-0.39592868	0.475084883
Aradu.YMT2S	4.124328135	3.577730931	3.582556003	2.773996325	3.215678597
Aradu.L2PP0	2.969012308	3.75595577	3.065227623	4.205548911	4.055716264
Aradu.WUH7T	3.798050515	3.744161096	3.654206378	3.454175893	3.975446766
Aradu.X3XEZ	0.722466024	1.097610797	0.59454855	1.63691458	2.13422094
Aradu.AJ5ZZ	-2.94341647	-5.05889369	-3.18442457	-1.88896869	-1.12029423
Aradu.GC05Y	1.495695163	1.757023247	1.613531653	2.498250868	2.548436625
Aradu.V6ZMF	1.66448284	2.448900951	2.14404637	4.599912842	4.595742339
Aradu.531ZK	1.89917563	1.839959587	1.835924074	2.147306699	2.435628594
Aradu.DN2D2	2	2.976363636	2.678071905	3.634593268	4.028569152
Aradu.V0EF8	-2.64385619	-2.47393119	-2.55639335	-0.26881676	-0.47393119
Aradu.MVB2G	0.632268215	3.010779839	1.687060688	2.438292852	2
Aradu.C9I9A	-5.05889369	-3.47393119	-4.05889369	-1.12029423	-1
Aradu.18FWJ	-4.32192809	-2.94341647	-4.64385619	5.058316496	5.294253136

Aradu.0XX6X	2.786596362	3.401903472	2.914564523	3.100977648	3.286881148
Aradu.1EI01	1.757023247	2.367371066	1.879705766	2.733354341	2.9202933
Aradu.B99AB	-4.05889369	-4.32192809	-5.64385619	-0.97143085	-1.73696559
Aradu.JS2XW	1.250961574	1.843983844	1.464668267	2.825785627	3.161887682
Aradu.APR5V	1.769771739	1.695993813	1.480265122	2.454175893	2.805292456
Aradu.LZ99D	NA	NA	NA	NA	NA
Aradu.H2I13	2.032100843	2.813524689	2.528571319	3.347665656	3.263034406
Aradu.LVM3Z	NA	NA	NA	NA	-6.64385619
Aradu.4S7IS	-1.02914635	-0.53533173	-1.15200309	-0.43440282	-0.23446525
Aradu.0Z207	NA	-5.64385619	NA	NA	-5.64385619
Aradu.SP6QR	1.773996325	2.22342255	1.786596362	2.541019153	3.129283017
Aradu.P9CN5	2.09423607	2.364572432	2.150559677	3.703765179	3.972692654
Aradu.0YP72	1.22650853	1.438292852	1.207892852	2.6622055	3.454175893
Aradu.G8KNY	1.49057013	1.831877241	1.851998837	3.044394119	3.243364426
Aradu.QM05Z	-2	-2.64385619	-2.83650127	NA	-3.47393119
Aradu.XES29	2.448900951	2.541019153	2.419538892	3.014355293	3.044394119
Aradu.6L7FC	1.709290636	2.430285273	2.003602237	2.639232163	2.974529312
Aradu.FV4D1	NA	-5.64385619	NA	NA	NA
Aradu.E26DL	1.922197848	2.173127433	2.416839742	2.211012193	2.432959407
Aradu.J79ME	2.207892852	2.724650272	2.558267634	2.526068812	3.087462841
Aradu.98KHN	1.952333566	2.03562391	1.769771739	2.440952198	3.030336078
Aradu.PGH8Z	5.175524601	5.28169825	5.296824459	4.789729251	5.124741692

Aradu.3I9MJ	-2.47393119	-1.21759144	-1.83650127	-0.53533173	-0.05889369
Aradu.KNN0Y	3.241840184	3.308885057	2.5360529	-0.64385619	1.970853654
Aradu.70A8U	2.414135533	2.397802962	2.263034406	1.883620816	2.032100843
Aradu.N7UWQ	-1.73696559	-1.25153877	-2.55639335	1.13093087	1.526068812
Aradu.9WH4P	NA	NA	NA	-3.32192809	-3.47393119
Aradu.EUP1G	2.782408565	2.937344392	2.733354341	2.6622055	2.883620816
Aradu.RWZ7N	4.846994687	4.246408087	4.473137488	3.746312766	3.519793486
Aradu.A5UR9	NA	-5.64385619	NA	NA	NA
Aradu.T693K	-0.86249648	-0.21759144	-0.88896869	0.641546029	0.641546029
Aradu.2BI4W	-1.21759144	-0.28630419	-1.28630419	-0.37706965	0.485426827
Aradu.9ZP9L	-2.64385619	-1.78587519	-1.21759144	-0.71311885	0.310340121
Aradu.8UL0Z	1.650764559	1.304511042	1.372952098	-0.15200309	1.292781749
Aradu.D7ZWV	2.207892852	2.813524689	2.353323291	2.944858446	3.044394119
Aradu.Y1KM6	0.042644337	-0.37706965	-0.45403163	1.23878686	1.941106311
Aradu.Y7AUY	1.195347598	2.039138394	1.5360529	1.941106311	2.622930351
Aradu.A32HC	2.137503524	2.244887059	2.150559677	3.061776198	3.552131108
Aradu.QA3D9	2.757023247	2.634593268	2.324810603	2.924099886	3.007195501
Aradu.BV4I8	-3.32192809	-0.20091269	-2.12029423	3.517275693	3.488000771
Aradu.XJ3JE	2.752748591	2.744161096	2.956056652	1.883620816	2.430285273
Aradu.QDT9L	5.62935662	5.253232939	5.277240898	-0.07400058	0.097610797
Aradu.0H4SB	1.257010618	1.963474124	1.15704371	1.372952098	1.344828497
Aradu.2GH9Y	4.211012193	3.221877081	3.613531653	3.176322773	2.438292852

Aradu.DE1N1	0.176322773	-0.577767	0.070389328	-2.18442457	-0.71311885
Aradu.T0R6F	NA	-3.83650127	NA	NA	NA
Aradu.YRI3B	-0.21759144	0.298658316	-0.07400058	0.40053793	1.063502942
Aradu.WL1AY	0.613531653	-0.37706965	-0.37706965	-1.02914635	-0.64385619
Aradu.VN6S3	1.361768359	0.773996325	0.82374936	0.042644337	-0.23446525
Aradu.X7B4K	NA	NA	NA	NA	NA
Aradu.IQ8X9	2.298658316	3.060047384	2.403267722	4.033863452	4.129283017
Aradu.WH2W0	1.641546029	1.879705766	1.632268215	2.241840184	2.587364991
Aradu.UTT3P	2.560714954	2.503348735	2.462052319	2.592158002	2.813524689
Aradu.43K0P	-0.91593574	0.495695163	-1.59946207	2.03562391	2.843983844
Aradu.SJR7G	0.765534746	0.879705766	1.042644337	0.286881148	0.163498732
Aradu.X1ANV	NA	NA	NA	NA	-5.64385619
Aradu.3D87V	-0.04394335	0.516015147	0.344828497	0.298658316	1.09085343
Aradu.PIA7H	1.427606173	2.032100843	1.871843649	2.657640005	3.017921908
Aradu.AR9AM	1.867896464	2.185866545	2.207892852	3.727920455	4.025028794
Aradu.HU672	NA	-6.64385619	NA	-2.39592868	-4.64385619
Aradu.IRK1D	2.869871406	3.246408087	2.86393845	3.499527024	3.766595162
Aradu.U1FJC	-0.43440282	1.333423734	0.298658316	2.488000771	2.750606505
Aradu.IUI2U	NA	NA	NA	NA	NA
Aradu.73S33	-3.64385619	-2.73696559	-5.64385619	-2.05889369	-1.64385619
Aradu.0291B	1.384049807	1.500802053	1.438292852	1.794935663	2.17951105
Aradu.9BC7H	3.107687869	5.14974712	4.815063017	7.363433935	6.320123598
Aradu.2447B	2.111031312	1.859969548	1.815575429	1.14404637	2.646162657

Aradu.RJ2CI	1.918386234	2.548436625	1.839959587	2.763411574	3.161887682
Aradu.H9EEY	6.570159302	6.105803772	6.497133304	3.781359714	3.97819563
Aradu.H7AQV	2.572889668	2.682573297	2.538538164	2.066950244	2.160274831
Aradu.250G2	2.722466024	2.959770155	2.560714954	3.526068812	3.837943242
Aradu.24YRC	-0.59946207	-1.18442457	-0.78587519	-0.81096618	-0.51457317
Aradu.BZA24	-0.86249648	0.137503524	-0.62148838	1.713695815	2.244887059
Aradu.N7C0U	-0.39592868	-0.78587519	-1.02914635	-3.05889369	1.464668267
Aradu.EY5U3	1.021479727	0.82374936	1.176322773	1.778208576	1.49057013
Aradu.X0QAS	NA	NA	NA	-4.05889369	0.042644337
Aradu.MA8H9	NA	-5.64385619	-6.64385619	NA	-3.18442457
Aradu.YTF7E	3.486714373	3.460742564	3.065227623	2.691534165	3.030336078
Aradu.137AN	-1.02914635	0.163498732	-2.05889369	2.596935142	3.084064265
Aradu.6PG6R	4.678071905	5.375039431	5.447248569	3.988230223	4.684257705
Aradu.5PW6G	NA	-4.64385619	-6.64385619	-3.18442457	-1.08926734
Aradu.A0FDR	NA	NA	NA	NA	NA
Aradu.JJ0LZ	1.815575429	2.010779839	1.757023247	2.761285273	3.030336078
Aradu.3J95M	3.64385619	4.050501697	3.695993813	4.605849867	4.932155684
Aradu.18V7U	3.454175893	3.908812908	3.822730148	2.475084883	2.742006211
Aradu.79MUY	4.324810603	4.634593268	4.026800059	5.678635348	5.505255801
Aradu.QY2T7	NA	NA	NA	NA	NA
Aradu.WFH0K	NA	NA	NA	-3.64385619	-2.39592868
Aradu.YPY6	5.781621998	5.364222221	5.726831217	1.937344392	1.929790998

M					
Aradu.PHB5M	1.757023247	1.906890596	1.416839742	3.802193217	3.840966704
Aradu.WLB92	-0.18442457	1.014355293	0.871843649	1.761285273	1.427606173
Aradu.DU4CZ	3.568032105	4.974070367	4.704318678	6.851873945	6.369291982
Aradu.CTQ6E	0.992768431	1.378511623	1.049630768	1.726831217	1.695993813
Aradu.MM88P	7.324720612	7.502075956	7.314243347	7.142311318	7.68699068
Aradu.54BXB	4.989593185	5.623223076	5.131342539	6.282439805	6.240123484
Aradu.QQY2K	3.016139703	3.463360886	3.22650853	3.255500733	3.614709844
Aradu.15MGK	2.669026766	2.859969548	2.98550043	2.98550043	3.564378169
Aradu.M7GVG	NA	-4.64385619	NA	-1.25153877	-0.64385619
Aradu.2R9BM	4.721372659	4.568032105	4.751677946	2.241840184	1.570462931
Aradu.L1FUQ	2.59454855	3.049630768	2.780310099	3.658782734	3.625270489
Aradu.AT0C1	0.35614381	0.584962501	0.35614381	0.40053793	1.195347598
Aradu.ZIC37	1.510961919	2.121015401	2.060047384	2.66448284	2.944858446
Aradu.P6639	5.785027362	5.983905663	5.96208625	5.211790976	5.393347551
Aradu.B21Z9	-2.39592868	-3.47393119	-3.05889369	-2.12029423	0.475084883
Aradu.XRM9M	1.550900665	2.40599236	1.769771739	2.56315813	2.794935663
Aradu.CZ5NE	1.531069493	1.827819025	1.659924558	1.86393845	2.22342255
Aradu.YRM8T	-5.64385619	-6.64385619	-3.64385619	-5.05889369	NA
Aradu.P0IKP	6.437127844	5.538538164	6.054848477	-0.30400619	0.150559677
Aradu.07NMY	-2.55639335	-2.83650127	-3.47393119	-5.05889369	-3.05889369
Aradu.6U3S2	1.163498732	1.646162657	1.632268215	0.925999419	1.782408565
Aradu.E0I63	-0.30400619	0.23878686	0.389566812	-1.47393119	0.028569152

Aradu.3MI4M	3.478971805	3.665620164	3.726831217	3.503348735	3.56193706
Aradu.WGT75	1.831877241	2.17951105	2.042644337	3.152183419	3.489286023
Aradu.C718S	-2.73696559	-1.55639335	-2.73696559	1.475084883	1.321928095
Aradu.K1R5H	5.261154673	4.923624611	4.894332742	6.401220862	6.725604841
Aradu.T9MSY	-4.32192809	-2	-2.25153877	-0.88896869	-2.83650127
Aradu.M62SP	-0.07400058	1.879705766	1.599317794	1.292781749	2.378511623
Aradu.9TY2N	0.933572638	0.475084883	0.389566812	-0.73696559	-0.47393119
Aradu.26MHM	0.286881148	0.831877241	0.910732662	1.042644337	0.97819563
Aradu.UJ1ZX	NA	-5.64385619	-5.05889369	-3.32192809	-3.18442457
Aradu.MR5GS	2.039138394	2.384049807	2.341985747	2.750606505	2.987320866
Aradu.QBK5E	4.347665656	4.207892852	3.993674362	3.932628157	4.303050085
Aradu.83UZ1	-0.577767	-1.88896869	-0.91593574	-3.64385619	-2.55639335
Aradu.24BEK	3.046141782	2.90303827	2.942983598	2.992768431	2.080657663
Aradu.PV1T4	-0.39592868	0.411426246	0.286881148	1.599317794	1.891419187
Aradu.YE41G	NA	NA	NA	NA	NA
Aradu.EH0QA	1.5360529	2.13093087	1.867896464	2.03562391	2.606442228
Aradu.59CIU	0.189033824	0.669026766	0.137503524	1.63691458	2.060047384
Aradu.FU1XB	1.464668267	1.584962501	1.056583528	3.554588852	4.053111336
Aradu.3IZ11	-1.94341647	-1.73696559	-1.88896869	-2.73696559	1.021479727
Aradu.F3745	-0.73696559	-0.15200309	-0.49410907	0.526068812	0.82374936
Aradu.99AQ5	3.521050737	3.353323291	3.422233001	2.12763328	2.488000771
Aradu.M721	0.632268215	0.659924558	0.545968369	1.028569152	1.550900665

3					
Aradu.19ZIS	-1.73696559	-1.28630419	-2.32192809	0.839959587	2.358958826
Aradu.296RJ	4.121015401	4.06608919	3.617063344	2.992768431	3.153805336
Aradu.TA161	NA	NA	NA	NA	-3.18442457
Aradu.CE0RB	3.340562269	3.82984956	2.98550043	5.007644031	5.121429909
Aradu.9VQ7B	2.952333566	3.295723025	3.040892431	2.937344392	3.582556003
Aradu.0K11B	-2.55639335	-2.05889369	-3.32192809	-0.12029423	0.310340121
Aradu.292D3	-0.37706965	-0.20091269	-0.35845397	0.669026766	0.669026766
Aradu.FTI5H	1.124328135	1.63691458	1.244887059	1.028569152	1.485426827
Aradu.BW310	-3.05889369	-2.25153877	-2.47393119	1.726831217	2.195347598
Aradu.RUD1R	NA	NA	NA	NA	-4.64385619
Aradu.426TW	-0.47393119	0.275007047	0.097610797	2.523561956	1.887525271
Aradu.C4EEM	3.381283373	4.027684877	3.862947248	4.715344295	4.706530553
Aradu.P2TGB	1.14404637	1.641546029	1.333423734	1.929790998	2.066950244
Aradu.D1XLL	1.604071324	1.070389328	1.500802053	1.056583528	1.438292852
Aradu.B0NST	-0.577767	-0.71311885	-2	3.112700133	2.657640005
Aradu.20EC4	-0.16812276	-0.86249648	-2	NA	NA
Aradu.IV48P	-2.18442457	-5.64385619	-5.64385619	NA	-4.64385619
Aradu.94E63	1.333423734	1.646162657	1.521050737	2.427606173	2.769771739
Aradu.TVK0N	-1.05889369	0.545968369	-0.30400619	2.173127433	2.550900665
Aradu.999EQ	3.404630684	3.970853654	3.66448284	4.927896454	4.870857864
Aradu.A3TK2	2.13093087	9.397653244	5.961623328	2.039138394	0.839959587
Aradu.VZ331	3.66106548	3.165107985	3.404630684	3.631104282	3.945794957

Aradu.2KV4N	3.033863452	3.13093087	2.931683057	6.216454865	5.865918815
Aradu.LAX0E	2.042644337	3.554588852	3.364572432	2.304511042	2.521050737
Aradu.AM9MC	-0.28630419	-0.43440282	-0.49410907	-0.94341647	0.275007047
Aradu.MTW86	2.244887059	2.718087584	2.324810603	3.323370069	3.49057013
Aradu.085I9	9.884933648	9.458057945	9.849467655	6.743622676	6.964167561
Aradu.KUT1Q	NA	NA	NA	NA	-2.05889369
Aradu.64HJW	NA	NA	NA	NA	NA
Aradu.B7233	1.773996325	2.62058641	1.773996325	3.189033824	3.324810603
Aradu.AP5EP	-3.32192809	-3.05889369	-2.64385619	-3.83650127	-1
Aradu.L5LVS	2.89917563	3.249445341	2.817623258	3.735522177	4.065227623
Aradu.5NS8M	2.678071905	3.010779839	2.918386234	2.726831217	3.109360559
Aradu.D1PNR	NA	NA	NA	NA	NA
Aradu.XVQ98	1.807354922	2.22342255	2.084064265	-0.04394335	0.070389328
Aradu.F9AQV	4.324810603	4.848497755	4.316869805	5.82374936	5.765799923
Aradu.EAT9T	0.622930351	1.464668267	0.790772038	2.403267722	2.14404637
Aradu.5C8VM	-2.32192809	-0.81096618	-1.68965988	0.201633861	-0.28630419
Aradu.I572Z	0.918386234	1.350497247	1.275007047	1.778208576	2.869871406
Aradu.DY43A	3.124328135	3.558267634	2.989139007	3.624100895	3.802193217
Aradu.1JK2E	3.424922088	3.232660757	3.415488271	2.673556424	2.754887502
Aradu.LL477	-3.83650127	-5.64385619	-6.64385619	-4.64385619	-3.64385619
Aradu.1IM7V	1.310340121	-0.25153877	0	0.35614381	2.634593268
Aradu.HK2IG	0.464668267	0.765534746	0.084064265	3.116031993	2.528571319

Aradu.6M9LZ	8.746480731	8.438750276	8.788685711	6.142413438	6.319039816
Aradu.7UX0U	3.139142019	3.430285273	3.058316496	3.793895883	4.188242656
Aradu.FK3YK	NA	NA	NA	-2.73696559	0.176322773
Aradu.1P5GD	2.541019153	2.972692654	2.849999259	2.589763487	3.039138394
Aradu.QWH3Y	NA	-6.64385619	-5.05889369	NA	-5.64385619
Aradu.YL3ZN	-0.04394335	0.678071905	0.5360529	2.538538164	3.169925001
Aradu.82P2N	0.50589093	0.970853654	0.839959587	1.35614381	2.03562391
Aradu.A9FZ7	1.97819563	2.310340121	1.815575429	2.794935663	3.282439805
Aradu.VK6NM	-1.59946207	-2	-0.53533173	0.475084883	0.790772038
Aradu.ZX42L	1.967168608	2.220329955	2.003602237	2.488000771	2.989139007
Aradu.UH20T	NA	NA	NA	NA	NA
Aradu.C0GMC	0.35614381	0.286881148	0.137503524	1.748461233	1.50589093
Aradu.A49KD	4.078951341	3.859969548	4.055716264	1.718087584	2.080657663
Aradu.A5MHD	3.23572706	3.558267634	3.171527106	4.509695842	4.47118746
Aradu.TVX63	3.932628157	3.397802962	3.761285273	2.508428653	2.887525271
Aradu.TB877	2.50589093	4.157852169	2.969012308	4.49441561	5.244887059
Aradu.C87QT	-0.88896869	-0.91593574	-1.32192809	1.937344392	2.007195501
Aradu.ELH7L	-2.32192809	-2.05889369	-3.18442457	-1.47393119	-1.83650127
Aradu.G28T8	2.289834465	3.039138394	2.584962501	3.549669171	3.481557281
Aradu.KWX5Q	NA	NA	NA	NA	-1.73696559
Aradu.33G7Z	-0.35845397	0.310340121	-0.21759144	1.201633861	1.220329955
Aradu.SB3IS	7.925940096	7.454587187	7.809221489	1.839959587	2.541019153

Aradu.B4I2A	2.066950244	2.432959407	2.23572706	2.572889668	3.229587923
Aradu.F3I3T	-0.71311885	-0.53533173	-1.32192809	-0.76121314	-0.34007544
Aradu.8F3EB	0.056583528	-0.51457317	-0.30400619	NA	NA
Aradu.DJ1H2	1.35614381	1.575312331	1.438292852	1.604071324	2.117695043
Aradu.QYZ18	-6.64385619	-3.18442457	-3.47393119	1.14404637	0.214124805
Aradu.69HMZ	NA	-4.32192809	-4.05889369	NA	-4.64385619
Aradu.PY313	0.475084883	3.046141782	3.896271849	3.220329955	2.462052319
Aradu.CW6KA	1.799087306	2.124328135	1.86393845	2.408711861	2.548436625
Aradu.46TFL	4.974070367	4.837438715	4.66732447	3.078951341	3.486714373
Aradu.X6BWV	0.86393845	1.454175893	1.40599236	1.070389328	1.867896464
Aradu.JZT13	1	1.914564523	2.275007047	2.327687364	2.367371066
Aradu.P3XDF	-3.64385619	-4.32192809	-4.64385619	-1	-1.88896869
Aradu.U012P	2.056583528	2.160274831	1.992768431	1.933572638	2.813524689
Aradu.CWG39	1.655351829	1.883620816	2.021479727	1.827819025	1.952333566
Aradu.YC3GJ	4.975446766	4.343407822	4.318316841	3.323370069	2.998195503
Aradu.BWJ69	-0.35845397	-0.37706965	-1.18442457	3.895302621	3.736604875
Aradu.SDR46	-0.12029423	0.042644337	-0.02914635	1.769771739	1.937344392
Aradu.SH1J0	NA	-5.05889369	-5.64385619	-1.68965988	-1.83650127
Aradu.GY0R3	5.925762114	5.5360529	5.598424761	2.687060688	2.691534165
Aradu.VAN9Z	4.500802053	4.394376945	4.27351589	4.57167681	4.778208576
Aradu.MM9KG	3.314696526	3.246408087	3.09085343	4.244887059	4.516645558
Aradu.D53Y	-1.94341647	-3.47393119	-4.64385619	-1.12029423	-1.47393119

G					
Aradu.YMR36	2.163498732	2.639232163	2.969012308	2.160274831	3.147306699
Aradu.08Y2X	-2	-2.18442457	-2.47393119	0.443606651	2.23572706
Aradu.413J1	-2.73696559	-3.05889369	-2.73696559	-6.64385619	-2.18442457
Aradu.0US4M	1.269033146	1.604071324	0.956056652	0.333423734	0.790772038
Aradu.KH25W	-0.88896869	-2.73696559	-2.39592868	-4.32192809	-3.83650127
Aradu.T8X2H	-1.47393119	-0.81096618	-2.25153877	2.080657663	2.321928095
Aradu.883SN	-1.12029423	-1.15200309	-0.88896869	-2.94341647	-2.83650127
Aradu.D1ZPU	-0.16812276	0.411426246	-0.51457317	1.028569152	1.669026766
Aradu.IU1HH	-1.05889369	-1.43440282	-1.73696559	0.22650853	0.59454855
Aradu.U7LA9	1.646162657	1.98550043	1.682573297	1.608809243	2.010779839
Aradu.9C32Y	0.298658316	0.485426827	0.378511623	1.028569152	1.655351829
Aradu.8I8L4	4.574101508	4.277240898	3.90014226	4.475733431	4.386810946
Aradu.NI37K	2.364572432	2.657640005	2.292781749	3.061776198	3.392317423
Aradu.034KP	1.851998837	1.82374936	1.659924558	2.263034406	4.109360559
Aradu.H128Z	-0.86249648	0.150559677	0.097610797	1.10433666	1.726831217
Aradu.69PE5	1.811471031	2.769771739	2.339137385	2.833902077	3.750606505
Aradu.JG7ZE	2.678071905	2.939226578	2.748461233	3.750606505	4.089159132
Aradu.0D317	0.948600847	1.50589093	1.220329955	1.709290636	1.432959407
Aradu.S59LW	4.448240225	4.867896464	4.097610797	5.85698569	5.827565007
Aradu.W1AGE	1.910732662	1.974529312	2.211012193	1.176322773	1.321928095
Aradu.U1A8I	-1.78587519	-2.12029423	-2.83650127	-4.32192809	-2.73696559
Aradu.W6H6	-5.05889369	-3.18442457	-4.64385619	0.150559677	0.214124805

W					
Aradu.I0VP0	1.339137385	1.63691458	1.35614381	2.739848103	3.016139703
Aradu.Y5G1G	1.485426827	1.422233001	1.933572638	3.408711861	3.896271849
Aradu.BA701	-3.47393119	-5.05889369	-4.05889369	-0.97143085	5.788685711
Aradu.32ETH	2.422233001	2.5360529	2.722466024	2.916476644	3.228049048
Aradu.B78K2	-2.12029423	-2.83650127	-2.47393119	-0.30400619	-0.86249648
Aradu.TN75P	0.286881148	0.310340121	-0.02914635	0	0.042644337
Aradu.B3611	3.703765179	3.565597176	3.544732656	0.163498732	0.722466024
Aradu.MJ2SD	NA	-6.64385619	NA	-4.64385619	1.510961919
Aradu.87VJS	4.353323291	4.375039431	4.06091205	5.225737653	5.491532462
Aradu.2N8AH	1.794935663	2.14404637	2.087462841	2.861955364	2.887525271
Aradu.073NL	0.887525271	1.23878686	1.070389328	0.871843649	1.22650853
Aradu.KMK70	-3.05889369	-2.39592868	-2.55639335	-1.64385619	-1.94341647
Aradu.E79Y1	-2.55639335	-0.66657627	-1.68965988	-1.15200309	-0.12029423
Aradu.805L3	2.370164281	4.469885976	3.781359714	2.599317794	3.032100843
Aradu.J8L3N	-0.76121314	-1	-1.18442457	-1.28630419	-0.91593574
Aradu.SXB8E	NA	NA	NA	NA	NA
Aradu.JV5YL	-2	-0.55639335	-1.35845397	1.989139007	2.189033824
Aradu.5D1Z0	1.286881148	1.589763487	1.580145484	1.63691458	1.996388746
Aradu.BNM3L	1.655351829	1.799087306	1.778208576	0.344828497	0.722466024
Aradu.UA6JC	3.444932049	3.92314918	3.531069493	3.806324057	4.201633861
Aradu.P7M2S	2.344828497	2.673556424	2.3305584	3.896271849	3.893362211
Aradu.S13A	-4.32192809	-2.05889369	-2	-5.05889369	-5.64385619

Z					
Aradu.A0SRT	1.35614381	1.35614381	1.555816155	1.769771739	2.042644337
Aradu.FM0YX	-5.64385619	NA	NA	NA	-6.64385619
Aradu.NRG58	0.584962501	0.475084883	0.22650853	-3.18442457	-2
Aradu.FP0C1	0.86393845	1.40053793	1.469885976	1.726831217	2.007195501
Aradu.HY1A1	0.739848103	2.049630768	3.112700133	0.604071324	0.250961574
Aradu.F9XLT	2.192194165	2.070389328	1.744161096	2.784503983	3.021479727
Aradu.DB6JJ	3.861955364	3.751677946	3.626439137	4.125155131	4.055716264
Aradu.1I2B8	6.826802684	6.851124363	6.486714373	3.304511042	3.485426827
Aradu.G31YK	0.286881148	1.384049807	0.678071905	1.650764559	1.292781749
Aradu.CJN5E	1.713695815	2.007195501	1.963474124	2.646162657	2.541019153
Aradu.YA6PI	2.839959587	2.046141782	2.289834465	1.438292852	2.198494154
Aradu.Q7508	2.565597176	2.780310099	2.702657543	3.43162296	3.619413011
Aradu.N0W4C	4.514753498	4.010779839	5.121429909	-0.45403163	-0.01449957
Aradu.CYF2K	-1.59946207	-1	-1.35845397	2.289834465	2.627606838
Aradu.FBB2P	3.988230223	3.893362211	3.63691458	2.6622055	2.752748591
Aradu.CQ0KB	2.575312331	3.481557281	3.512226887	1.687060688	2.229587923
Aradu.7BG9P	1.070389328	1.201633861	0.879705766	0.807354922	1.459431619
Aradu.MZ5HW	-2.47393119	-1.64385619	-2.94341647	-0.577767	-0.25153877
Aradu.B7FB5	1.992768431	2.313245852	1.618238656	3.745237332	3.69265037
Aradu.S3DH3	0.604071324	1.344828497	0.432959407	1.014355293	1.459431619
Aradu.DL83H	4.683134985	4.102658131	4.166715445	-5.05889369	-3.18442457
Aradu.EDD1	1.580145484	2.017921908	1.3950628	2.372952098	2.50589093

N					
Aradu.22C31	2.653060017	2.759155834	2.577730931	3.193771743	3.545968369
Aradu.2C9WA	NA	-6.64385619	NA	-0.43440282	4.695993813
Aradu.TN9DS	2.969012308	3.169925001	3.125981654	1.700439718	1.989139007
Aradu.B4Z82	-1.64385619	-2.05889369	-3.18442457	0.98550043	1.632268215
Aradu.55SXN	-0.20091269	0.799087306	-0.04394335	3.89917563	4.10433666
Aradu.J364A	2.021479727	2.014355293	1.773996325	3.129283017	2.922197848
Aradu.D7NJ0	1.565597176	2.080657663	1.641546029	6.317774373	5.719457275
Aradu.1J1TW	-0.02914635	0.925999419	0.443606651	1.250961574	1.718087584
Aradu.W5JRX	NA	NA	NA	NA	NA
Aradu.5I6E9	0.925999419	0.790772038	0.918386234	-0.34007544	0.22650853
Aradu.Y2ZCM	-4.64385619	NA	NA	-4.64385619	-2.25153877
Aradu.YQ4V9	1.526068812	1.97819563	1.773996325	2.229587923	2.709290636
Aradu.IQK2Q	-0.68965988	0.084064265	-0.23446525	0.475084883	0.545968369
Aradu.1J1DY	0.475084883	1.014355293	-0.16812276	2.269033146	2.771885579
Aradu.AFB92	NA	-5.05889369	-5.05889369	NA	NA
Aradu.HSB55	1.028569152	1.23878686	1.03562391	1.761285273	1.807354922
Aradu.LSV4Q	5.308521062	6.083213368	5.736334277	3.914564523	3.583759754
Aradu.1ZL4G	-3.05889369	-2.18442457	-4.64385619	-0.88896869	-1.18442457
Aradu.A723B	3.850999395	4.033863452	4.100136671	0.910732662	0.641546029
Aradu.A6BTS	0.495695163	0.526068812	0.201633861	1.40599236	1.967168608
Aradu.YC6ZD	0.82374936	1.432959407	0.98550043	1.691534165	2.032100843
Aradu.Q00G	1.327687364	1.891419187	1.361768359	1.09085343	1.182692298

R					
Aradu.7ZK0R	-4.05889369	-2	-2.73696559	1.786596362	3.244887059
Aradu.ZLR3W	-3.18442457	-1.88896869	-2	-0.97143085	-1.32192809
Aradu.L3UB6	0.948600847	0.90303827	0.516015147	2.176322773	2.733354341
Aradu.GH8FF	4.90303827	4.565597176	4.589164237	3.524815928	4.032100843
Aradu.3EF6P	0.713695815	0.910732662	0.847996907	1.748461233	1.895302621
Aradu.7JW9M	2.767654798	3.121015401	3.012568674	3.161887682	3.797012978
Aradu.N8T7S	-1.83650127	0.070389328	-2.18442457	0.50589093	1.150559677
Aradu.HM4L0	-3.64385619	-3.83650127	-3.18442457	-1.83650127	-2.73696559
Aradu.T3LBA	2.702657543	3.049630768	2.550900665	3.769771739	3.893362211
Aradu.KCC58	0.90303827	1.416839742	1.15704371	2.713695815	2.604071324
Aradu.P4XEI	-5.64385619	-4.32192809	-6.64385619	0.831877241	-0.66657627
Aradu.YJH4A	1.650764559	1.871843649	1.744161096	2.111031312	2.477677328
Aradu.0AW7C	-1.43440282	-0.68965988	-1.25153877	1.956056652	1.510961919
Aradu.TFK2R	3.850999395	3.881664619	4.092545742	3.522306893	4.170726276
Aradu.PFQ1E	-0.76121314	-0.53533173	-1.28630419	0.687060688	0.495695163
Aradu.53YGT	NA	NA	NA	NA	-4.05889369
Aradu.JPJ0M	0.773996325	1.304511042	0.815575429	1.604071324	1.922197848
Aradu.A2ZJG	9.252286971	8.909263079	9.385991785	5.229587923	5.249445341
Aradu.KF6LM	-1.83650127	-1.55639335	-2.39592868	-1.55639335	-2.83650127
Aradu.W0IT8	0.555816155	0.82374936	0.604071324	-0.64385619	-0.49410907
Aradu.64B3S	2.250961574	2.531069493	1.855989697	2.811471031	2.916476644
Aradu.K93A	6.167719199	5.998195503	5.798828178	-2.39592868	-1.51457317

E

Aradu.014NJ	NA	NA	-6.64385619	NA	-6.64385619
Aradu.P0TWG	3.528571319	3.572889668	3.524815928	3.147306699	3.588564737
Aradu.W0WNS	0.575312331	1.028569152	0.90303827	0.831877241	1.163498732
Aradu.0B375	1.275007047	2.629939409	1.646162657	3.56315813	3.439623138
Aradu.UDE9J	-3.47393119	-3.18442457	-4.05889369	-0.577767	-0.07400058
Aradu.31IVL	1.89917563	2.039138394	1.944858446	2.5360529	2.389566812
Aradu.HBT8X	-2.18442457	-3.47393119	-3.47393119	-0.32192809	0.014355293
Aradu.NQ7IA	NA	NA	NA	NA	-3.64385619
Aradu.Q2SWK	2.64385619	2.724650272	2.361768359	2.733354341	2.901108243
Aradu.WIS65	0.650764559	0.50589093	0.495695163	2.09085343	2.166715445
Aradu.I01VW	-1.51457317	-1.73696559	-3.05889369	0.871843649	0.97819563
Aradu.495R5	0.275007047	0.516015147	0.250961574	1.257010618	1.182692298
Aradu.TES1U	8.293977366	8.505255801	8.582066692	3.819668183	3.650764559
Aradu.E4WB9	1.655351829	2.488000771	2.298658316	2.709290636	3.040892431
Aradu.AP7WX	-3.83650127	-1.51457317	-2.47393119	-0.59946207	0.056583528
Aradu.286YF	5.02103548	4.838447593	4.54225805	6.309794644	6.578485913
Aradu.JR4VV	NA	NA	NA	NA	NA
Aradu.I2DKG	2.107687869	3.301587647	2.724650272	2.03562391	2.731183242
Aradu.L4E7U	-2.32192809	-1.59946207	-1.88896869	-0.68965988	0.189033824
Aradu.N7FJP	-2.94341647	-3.32192809	-3.32192809	-2.64385619	-1.64385619
Aradu.C8CZA	1.859969548	2.451540833	2.053111336	3.161887682	3.541019153
Aradu.AR7X	-6.64385619	NA	NA	-4.05889369	-2.73696559

M					
Aradu.EYD2G	3.865918815	5.075104722	3.564378169	3.121015401	3.211012193
Aradu.QIJ36	-3.47393119	-0.47393119	-3.18442457	-4.32192809	-1.51457317
Aradu.MZX0S	0.35614381	1.389566812	1.691534165	3.904965719	3.743084056
Aradu.Z197N	-0.21759144	0.124328135	-0.21759144	0.59454855	2.182692298
Aradu.XME24	4.677508242	4.59454855	4.634593268	4.17951105	3.844988157
Aradu.51KU5	1.14404637	1.378511623	0.111031312	3.600507645	3.749534268
Aradu.KLS9W	NA	NA	NA	NA	-4.05889369
Aradu.IN2S6	1.169925001	1.327687364	0.831877241	1.646162657	1.550900665
Aradu.54WNJ	0.956056652	2.207892852	1.839959587	4.132576843	4.537296067
Aradu.92ZAJ	-5.05889369	-3.64385619	-5.05889369	-2.12029423	-3.18442457
Aradu.Q0YMS	-2	-2	-2.64385619	1.15704371	0.970853654
Aradu.12EER	4.382667253	3.862947248	3.801158656	0.201633861	0.82374936
Aradu.5Y1RP	0.895302621	0.59454855	0.526068812	-0.49410907	-0.39592868
Aradu.64BNM	-4.64385619	-4.32192809	-3.64385619	-0.73696559	-0.35845397
Aradu.7X64R	-1.12029423	-0.30400619	-0.55639335	0.042644337	0.622930351
Aradu.UQ2CM	-1	-0.97143085	-1.47393119	2.518535139	1.555816155
Aradu.0DS3V	0.070389328	0.40053793	0.137503524	1.195347598	1.292781749
Aradu.WL4PN	NA	-5.64385619	-6.64385619	-2.64385619	0.687060688
Aradu.M5RIF	2.733354341	2.901108243	2.673556424	2.765534746	2.629939409
Aradu.V8GHA	3.404630684	5.260025656	5.014801602	4.795455272	5.336640446
Aradu.63TDV	3.460742564	3.529820947	3.742006211	3.374343989	3.526068812
Aradu.HTX3	1.111031312	2.742006211	2.381283373	-0.30400619	1.182692298

0					
Aradu.ZM868	2.339137385	2.40599236	2.389566812	1.996388746	2.386810946
Aradu.0Q24I	-3.64385619	-1.25153877	-1.55639335	-0.23446525	0
Aradu.0N1XE	5.566510756	5.873321063	5.343763122	7.028790136	7.01189812
Aradu.RXW02	-0.81096618	-0.43440282	-1.18442457	-0.39592868	-0.23446525
Aradu.BE6XZ	2.788685711	1.963474124	2.440952198	3.486714373	3.725741157
Aradu.3QR5Y	1.263034406	1.650764559	1.622930351	2.137503524	2.580145484
Aradu.33VZ6	5.338780944	5.521364878	5.689299161	5.537606691	5.283551423
Aradu.VM6CH	0.201633861	-0.25153877	-0.34007544	-0.39592868	1.028569152
Aradu.6FR0W	-0.577767	-0.53533173	-0.76121314	-0.83650127	0.565597176
Aradu.E4IDB	4.563768278	4.763411574	4.503348735	4.9202933	5.282069075
Aradu.7LZ09	0.070389328	0.432959407	-0.07400058	1.89917563	2.424922088
Aradu.PA2UE	2.5360529	3.033863452	2.809414444	3.455491621	3.75595577
Aradu.H0HJA	2.140778656	2.386810946	1.589763487	1.687060688	2.298658316
Aradu.0252U	-0.577767	-0.45403163	-0.28630419	1.195347598	2.266036894
Aradu.RW8B0	4.607626221	4.841973119	5.071676874	4.397802962	4.761285273
Aradu.XV49R	1.691534165	1.887525271	1.847996907	2.518535139	2.94673086
Aradu.28KTI	3.416839742	3.544732656	3.526068812	2.049630768	2.339137385
Aradu.UEM5G	7.58165253	8.048868273	8.160678213	8.131599773	8.060533822
Aradu.ITR9J	-2.83650127	-2.05889369	-3.47393119	0.35614381	0.757023247
Aradu.CW391	NA	NA	NA	NA	NA
Aradu.LKB1I	3.228049048	3.500802053	3.526068812	2.887525271	3.465974465
Aradu.9JB4Z	-1.68965988	-1.55639335	-1.59946207	-0.49410907	-0.04394335

Aradu.T1Q4B	3.684818738	5.57500972	3.947666157	2.176322773	3.473786912
Aradu.WFC0U	2.028569152	2.073820233	2.10433666	2.448900951	2.570462931
Aradu.3L5NK	2.003602237	2.711494907	2.448900951	3.972692654	4.321928095
Aradu.VF877	2.933572638	2.891419187	2.666756592	4.266786541	4.514753498
Aradu.71DP5	1.40599236	1.718087584	1.350497247	2.560714954	2.680324357
Aradu.9EZ7Z	3.085764554	2.974529312	2.994579724	3.552131108	3.816599707
Aradu.RI1GB	1.304511042	0.815575429	0.895302621	-4.32192809	3.962549023
Aradu.SK8K0	NA	-4.32192809	-6.64385619	-2.73696559	-1.94341647
Aradu.QC0V1	-4.64385619	-5.05889369	-5.05889369	-3.47393119	-3.32192809
Aradu.M1BLH	1.599317794	1.3950628	0.565597176	NA	0.201633861
Aradu.C2PQ3	NA	NA	NA	NA	NA
Aradu.9EK2R	NA	NA	NA	NA	NA
Aradu.UD5RI	3.498250868	4.148120631	3.554588852	5.0721058	5.016139703
Aradu.GGH8Z	2.422233001	3.089159132	2.833902077	4.146492307	4.312519967
Aradu.I957I	-0.59946207	0.263034406	-0.32192809	0.124328135	-0.08926734
Aradu.EG568	4.851499202	4.855989697	4.659924558	1.292781749	1.422233001
Aradu.42P16	-0.01449957	0.23878686	-0.26881676	-2.25153877	-3.47393119
Aradu.Z4QII	2.788685711	3.097610797	2.733354341	4.005399988	4.424250286
Aradu.6E59T	2.459431619	2.548436625	2.042644337	2.803227036	3.005399988
Aradu.YF3HA	1.632268215	2.056583528	1.700439718	2.596935142	2.687060688
Aradu.61Y68	0.992768431	0.575312331	0.765534746	0.925999419	1.718087584
Aradu.TE0XJ	-4.64385619	-2.94341647	-2.94341647	-0.08926734	-0.04394335

Aradu.TD814	-0.30400619	-0.02914635	-0.34007544	-0.91593574	0.014355293
Aradu.C00W8	-1	-1.68965988	-2.55639335	0.50589093	1.321928095
Aradu.U1JXN	NA	NA	NA	-0.55639335	-0.86249648
Aradu.VN8X3	2.901108243	3.283921772	3.017921908	1.989139007	2.62058641
Aradu.14261	0.82374936	1.304511042	0.839959587	1.835924074	2.166715445
Aradu.8WJ4P	NA	NA	NA	NA	NA
Aradu.3WJ36	4.382667253	4.214124805	3.931683057	3.56315813	4.051372102
Aradu.149WS	2.244887059	2.220329955	1.891419187	3.295723025	3.710393192
Aradu.SY0M5	0.464668267	1.09085343	0.871843649	1.799087306	2.077242999
Aradu.P0D7S	NA	NA	NA	NA	NA
Aradu.J4XWG	3.340562269	4.624685811	4.759155834	2.641546029	2.99095486
Aradu.199XC	0.042644337	0.097610797	0.056583528	-0.45403163	0.704871964
Aradu.5DL1E	-0.97143085	1.042644337	-0.55639335	3.030336078	2.819668183
Aradu.C6P70	6.659068275	6.476543706	6.646018611	4.09423607	3.993674362
Aradu.9602J	-1.73696559	-2.47393119	-2.64385619	-4.32192809	-3.64385619
Aradu.L4D0H	-0.78587519	-0.73696559	-0.94341647	-0.91593574	-0.88896869
Aradu.1FQ73	2.963474124	3.528571319	3.125981654	4.308885057	4.546585829
Aradu.A4737	1.948600847	2.341985747	2.100977648	2.650764559	3.030336078
Aradu.UMP23	-1.47393119	-0.73696559	-1.32192809	0.669026766	1.3950628
Aradu.ZZD2I	2.049630768	2.448900951	2.408711861	2.298658316	2.797012978
Aradu.6V13C	-5.05889369	-4.64385619	-4.32192809	-1.88896869	-0.20091269
Aradu.VBT9	1.678071905	2.176322773	1.82374936	2.472487771	2.89917563

Y					
Aradu.92ILA	3.393690764	2.831877241	2.908812908	5.660780334	4.966245873
Aradu.V2ISN	1.799087306	1.765534746	1.695993813	2.9800253	3.695993813
Aradu.LR2WR	-0.08926734	0.807354922	-0.05889369	1.292781749	1.632268215
Aradu.QU580	1.910732662	1.298658316	1.59454855	-2.18442457	-0.88896869
Aradu.S4XSK	-1.47393119	-2.18442457	-1.73696559	-1.05889369	-0.10469738
Aradu.QV7H5	0.992768431	1.432959407	1.14404637	1.963474124	2.021479727
Aradu.MW13W	NA	NA	NA	NA	NA
Aradu.9E2KY	-0.45403163	-0.47393119	-0.73696559	-3.05889369	-0.30400619
Aradu.X2I4E	4.805292456	4.821199978	4.545350645	5.376776572	5.319401167
Aradu.KSK2P	-0.05889369	0.575312331	0	1.275007047	1.794935663
Aradu.GB0MR	1.790772038	1.981852653	1.807354922	1.914564523	2.521050737
Aradu.AH39E	-3.32192809	-3.47393119	-2.12029423	-0.59946207	0.378511623
Aradu.M7QN4	3.957914599	4.303780748	4.270528942	5.294620749	4.739308071
Aradu.CZD45	-1.43440282	-1.25153877	-1.32192809	-0.12029423	0.137503524
Aradu.J7KPQ	-1.15200309	-0.26881676	-0.47393119	0.111031312	1.286881148
Aradu.UIA0J	-2.55639335	-1.59946207	-1.39592868	1.23878686	1.531069493
Aradu.JWG0R	4.422905743	4.34269696	4.43629512	4.325530332	4.254745198
Aradu.EU0BW	-4.64385619	-6.64385619	-4.64385619	0.526068812	1.669026766
Aradu.L3GA1	2.805292456	2.9202933	3.288358562	2.95419631	2.927896454
Aradu.724NR	1.232660757	1.220329955	1.367371066	1.195347598	1.422233001
Aradu.YDW81	0.378511623	0.933572638	1.03562391	2.451540833	3.042644337
Aradu.P0D4	1.244887059	1.646162657	1.14404637	2.217230716	2.060047384

3					
Aradu.S073D	1.570462931	0.970853654	1.321928095	1.384049807	0.773996325
Aradu.20C0X	1.286881148	1.803227036	1.333423734	4.368069877	4.749534268
Aradu.B6TUW	-1	-1.28630419	-1.55639335	-0.16812276	-0.45403163
Aradu.38IMA	0.443606651	-0.64385619	0.214124805	-0.37706965	-0.39592868
Aradu.K7AFI	-4.32192809	-2	-3.05889369	1.280956314	1.23878686
Aradu.U14K2	-1.64385619	-1.43440282	-2.25153877	1.650764559	1.687060688
Aradu.7Y1E8	2.523561956	3.097610797	2.996388746	4.244125943	3.63691458
Aradu.GZ5DT	0.59454855	0.992768431	0.713695815	0.871843649	1
Aradu.72QL5	-5.64385619	-4.05889369	-4.32192809	-2.94341647	-2
Aradu.JP0ZJ	3.044394119	2.746312766	2.733354341	1.189033824	2.375734539
Aradu.MZ5UW	NA	-3.18442457	-6.64385619	NA	-3.83650127
Aradu.YF1F6	5.250961574	5.110196178	5.257387843	2.204766751	2.347665656
Aradu.V73WK	NA	-4.64385619	-4.05889369	-3.18442457	-2.64385619
Aradu.JTL52	-4.32192809	-5.64385619	-6.64385619	NA	-5.05889369
Aradu.HU3I3	1.007195501	1.214124805	1.049630768	1.744161096	1.933572638
Aradu.69YXI	0.757023247	0.895302621	0.739848103	4.704871964	4.717539343
Aradu.1J0IW	1.695993813	1.695993813	1.526068812	1.521050737	1.815575429
Aradu.LSL3G	1.799087306	1.40599236	0.641546029	1.695993813	2.912649865
Aradu.QJ7XC	2.124328135	2.641546029	2.014355293	2.972692654	3.12763328
Aradu.SR3CA	-5.05889369	NA	NA	NA	-3.83650127
Aradu.LW7LA	1.13093087	1.189033824	0.773996325	3.150559677	3.117695043
Aradu.HK5U	NA	NA	NA	-5.64385619	-5.05889369

D					
Aradu.TY9X1	0.963474124	2.634593268	1.778208576	2.972692654	4.083213368
Aradu.SUN9W	3.053111336	3.073820233	3.14404637	2.895302621	3.116031993
Aradu.E3ZED	2.063502942	2.03562391	2.121015401	1.028569152	0.739848103
Aradu.K52EF	NA	-6.64385619	-5.64385619	NA	NA
Aradu.JB051	0.411426246	-0.02914635	0.275007047	0.782408565	-1.05889369
Aradu.Y1DM8	1.232660757	1.879705766	1.189033824	1.137503524	1.695993813
Aradu.I66GQ	NA	-6.64385619	NA	-6.64385619	-3.83650127
Aradu.JN158	-3.83650127	-5.05889369	-5.64385619	-1.68965988	-2
Aradu.WJU1S	1.028569152	0.933572638	1.124328135	0.604071324	0.678071905
Aradu.IC19J	2.843983844	3.634593268	3.289834465	4.857980995	4.976821852
Aradu.J5AA1	3.918386234	3.964398632	3.859969548	3.875780063	4.023255352
Aradu.WBJ0E	6.288912203	6.104546339	5.934280594	5.311793718	5.541638734
Aradu.4X7WG	NA	-6.64385619	-5.64385619	-6.64385619	-3.83650127
Aradu.W44WH	-2.83650127	-2.83650127	-2.32192809	1.98550043	2.204766751
Aradu.G39DJ	NA	NA	NA	NA	NA
Aradu.V4VHC	1.769771739	2.516015147	2.17951105	3.697106574	3.737686761
Aradu.RC9DQ	2.918386234	4.313245852	2.813524689	6.293333695	6.087887101
Aradu.ZN3FN	0.286881148	-0.07400058	-0.76121314	-0.47393119	0.201633861
Aradu.LHT85	2.147306699	2.495695163	2.14404637	3.304511042	3.61117238
Aradu.AAT7R	NA	NA	NA	NA	NA
Aradu.FH10G	3.642701572	3.307428525	3.370164281	2.748461233	3.680324357
Aradu.P6497	2.09085343	2.220329955	1.914564523	2.084064265	2.301587647

Aradu.NIH31	NA	NA	NA	NA	NA
Aradu.1E1I2	NA	-5.05889369	-5.05889369	-2.39592868	-2.12029423
Aradu.XHP2L	-3.32192809	-1.28630419	-1.78587519	-1.68965988	-1.15200309
Aradu.8GS2N	1.327687364	0.526068812	-0.34007544	1.10433666	1.23878686
Aradu.1DT27	6.327866971	5.997066545	6.612647378	0.411426246	0.748461233
Aradu.GX9A4	2.241840184	2.424922088	2.09085343	3.82374936	4.001802243
Aradu.BC6IG	2.244887059	2.084064265	2.077242999	1.691534165	2.538538164
Aradu.320AL	-5.64385619	NA	NA	NA	NA
Aradu.I83P6	NA	NA	NA	NA	NA
Aradu.77UYS	3.539779192	3.6622055	3.512226887	4.445594291	4.478971805
Aradu.N2YDU	-3.05889369	-4.64385619	-3.64385619	-4.64385619	-3.47393119
Aradu.DRU5H	5.037821465	4.822730148	4.822730148	2.851998837	3.007195501
Aradu.JAD6J	1.500802053	2.454175893	2.111031312	2.375734539	2.659924558
Aradu.QY7MG	0.956056652	1.739848103	1.469885976	2.469885976	2.592158002
Aradu.P6XBQ	1.589763487	2.039138394	1.879705766	2.10433666	2.513490746
Aradu.NEM0A	-4.32192809	-4.05889369	-5.64385619	0.411426246	1.189033824
Aradu.DWJ8H	-2	-1.18442457	-1.02914635	-1.12029423	-1.25153877
Aradu.GU21D	4.410748128	4.997744026	4.854993017	6.087462841	5.991861931
Aradu.MS621	2.263034406	2.678071905	2.568032105	3.450221496	3.811471031
Aradu.MW1NF	3.214124805	3.360364277	3.075532631	4.493134922	4.625854931
Aradu.U8XKU	-2	-1.78587519	-3.18442457	0.432959407	0.871843649
Aradu.10BG	2.657640005	3.03562391	2.883620816	3.775050541	4.06608919

T					
Aradu.6JU5H	1.443606651	1.632268215	1.40053793	1.739848103	2.381283373
Aradu.FI298	6.541174073	6.972922364	6.722329399	5.708463165	4.421559945
Aradu.EM2UC	0.042644337	-0.15200309	-0.25153877	-1.55639335	-0.64385619
Aradu.C51SS	-0.83650127	0.454175893	-0.97143085	1.739848103	2.257010618
Aradu.R0BLH	2.163498732	1.778208576	2.070389328	1.063502942	1.867896464
Aradu.AW46C	-4.05889369	-5.05889369	-4.32192809	NA	NA
Aradu.07ZWB	3.220329955	3.258518925	3.135863165	2.998195503	2.319039816
Aradu.35B90	NA	NA	NA	NA	NA
Aradu.12ETV	3.581351247	1.765534746	2.107687869	0.871843649	2.837943242
Aradu.4X1GI	1.411426246	2.49057013	2.498250868	3.426264755	3.548436625
Aradu.J7DHV	2.454175893	2.89917563	2.906890596	3.337711092	3.867896464
Aradu.U987Q	0.333423734	0.389566812	-0.05889369	0.799087306	0.722466024
Aradu.Y8PPD	2.22650853	2.214124805	2.182692298	2.599317794	2.889473543
Aradu.E2KJV	3.158660175	3.288358562	3.419538892	2.327687364	3.397802962
Aradu.4TM7E	0.641546029	0.799087306	0.765534746	0.871843649	1.063502942
Aradu.HY7QC	0.137503524	1.257010618	-0.37706965	4.012568674	3.583759754
Aradu.98KS9	0.389566812	1.469885976	0.604071324	1.803227036	1.722466024
Aradu.0HA70	4.379898164	4.19061486	4.266036894	3.772941338	4.317593505
Aradu.88VQK	3.215678597	2.998195503	3.032100843	1.584962501	1.739848103
Aradu.IBQ9G	1.59454855	1.959770155	1.608809243	1.82374936	2.17951105
Aradu.3TD2D	-1.78587519	-0.76121314	-0.78587519	0.389566812	1.056583528
Aradu.EJA5	-2.55639335	-6.64385619	-1.51457317	-4.32192809	1.189033824

A					
Aradu.Q3HQI	1.891419187	2.687060688	2.737686761	2.98550043	3.148934105
Aradu.G0AVK	-2.12029423	-1.94341647	-2.25153877	-1.94341647	-2.18442457
Aradu.X6FMF	2.408711861	3.476381688	3.392317423	3.959770155	4.482202926
Aradu.78FH9	3.524815928	1.855989697	2.010779839	-2.83650127	-0.55639335
Aradu.1LI5S	3.539779192	3.708187236	3.504620392	4.521050737	4.787641414
Aradu.15W25	-0.51457317	-0.76121314	0.765534746	-1	-0.25153877
Aradu.I9N63	3.132576843	3.336283388	2.837943242	4.060047384	4.301587647
Aradu.PS45N	1.739848103	1.704871964	1.831877241	2.422233001	2.232660757
Aradu.SX4F9	5.502075956	5.619413011	5.358958826	3.349082146	3.114367025
Aradu.UR751	NA	NA	-6.64385619	-3.05889369	-1.88896869
Aradu.CP1HR	-1.94341647	-4.05889369	-3.32192809	-3.47393119	-2.73696559
Aradu.L7FSS	NA	NA	NA	NA	NA
Aradu.QC0XR	-5.64385619	-5.05889369	-6.64385619	NA	-2.55639335
Aradu.U0DT9	3.408711861	3.280956314	3.046141782	5.138323004	4.867896464
Aradu.A9V55	0.622930351	0.189033824	-0.05889369	1.584962501	1.956056652
Aradu.5UI2Y	-0.20091269	0.028569152	-0.51457317	1.443606651	2.084064265
Aradu.XFR5L	3.435628594	2.744161096	2.684818738	1.855989697	2.601696516
Aradu.5GH6D	4.250961574	4.128458384	4.15704371	4.49057013	4.59275601
Aradu.4E6H2	2.324810603	2.99095486	2.720278465	5.057450272	5.301953395
Aradu.5T6PH	5.223036338	5.483493351	4.424250286	6.939226578	6.418021241
Aradu.6FR5V	3.065227623	2.992768431	2.788685711	2.835924074	2.933572638
Aradu.ACG3	-5.05889369	-5.64385619	-4.64385619	-2.73696559	1.5360529

Y					
Aradu.J1Y0V	5.768713657	5.301587647	5.142413438	2.533563348	2.495695163
Aradu.EJE3Z	4.572889668	4.401903472	4.129283017	3.372952098	3.701549057
Aradu.2RC64	2.916476644	3.379898164	3.304511042	2.883620816	2.885574364
Aradu.MM40G	2.788685711	2.970853654	2.189033824	4.678071905	5.160678213
Aradu.39CFF	-2.05889369	-4.64385619	-5.05889369	-3.83650127	-2.47393119
Aradu.C1C0K	NA	NA	NA	NA	NA
Aradu.FT8DK	-3.47393119	-3.05889369	-3.64385619	-2.47393119	5.824767853
Aradu.UIC09	1.959770155	2.46727948	1.90303827	3.624100895	3.733354341
Aradu.7S6GM	2.247927513	1.89917563	1.859969548	1.883620816	2.367371066
Aradu.54E80	0.014355293	-0.37706965	-0.4150375	0	1.269033146
Aradu.IQS3D	0.731183242	1.555816155	0.992768431	2.673556424	2.548436625
Aradu.FSY43	1.195347598	1.516015147	1.344828497	1.163498732	1.682573297
Aradu.06HG0	-1.21759144	-0.10469738	0.831877241	-1	0.286881148
Aradu.7KU2A	2.837943242	3.132576843	2.996388746	3.545968369	3.767654798
Aradu.W0BBS	-0.83650127	-0.43440282	-1.02914635	2.063502942	2.23878686
Aradu.GG5EE	NA	NA	NA	-4.64385619	-4.05889369
Aradu.YM0TI	2.451540833	2.12763328	1.773996325	0.201633861	0.310340121
Aradu.BB9NS	-0.86249648	-0.15200309	-0.32192809	0.286881148	0.275007047
Aradu.ZR4H7	-1.08926734	-1.02914635	-1.35845397	-0.4150375	0.367371066
Aradu.TB51Z	1.09085343	1.469885976	1.40053793	2.250961574	2.908812908
Aradu.0UW7J	9.647746217	9.160098316	9.342474744	5.63343121	5.665335917
Aradu.DB78	3.367371066	3.455491621	3.493134922	1.887525271	2.419538892

U					
Aradu.68AHZ	-2.64385619	-0.64385619	-3.05889369	-0.97143085	-0.12029423
Aradu.B5ML3	-0.21759144	0.286881148	0.298658316	1.056583528	1
Aradu.Y18FU	1.948600847	2.124328135	2.014355293	1.555816155	2.211012193
Aradu.T52CY	2.653060017	4.151371776	3.744161096	3.187451054	3.5360529
Aradu.CR12A	-1.02914635	-1	-0.78587519	1.599317794	1.59454855
Aradu.QVD5S	3.051372102	2.859969548	2.601696516	3.365972428	3.911691582
Aradu.2ZT9L	2.759155834	3.114367025	2.933572638	2.965322548	3.285402219
Aradu.T7TUD	-4.32192809	-2.73696559	-2.94341647	1.15704371	1.207892852
Aradu.ID8H5	-0.81096618	-0.68965988	-1.02914635	-0.97143085	0.704871964
Aradu.R6FI2	NA	-4.64385619	-6.64385619	NA	-3.18442457
Aradu.SBB2J	1.655351829	1.700439718	1.545968369	2.3305584	2.895302621
Aradu.22ZWX	2.702657543	3.044394119	2.704871964	4.516015147	4.658211483
Aradu.9FZ57	1.989139007	2.837943242	1.989139007	3.361768359	3.321928095
Aradu.IF1JM	0.35614381	0.659924558	0.584962501	1.66448284	2.080657663
Aradu.PXM5A	0.22650853	2.606442228	3.340562269	1.82374936	2.521050737
Aradu.6MH23	3.614709844	3.986410935	3.844988157	3.286881148	3.975446766
Aradu.UR64R	5.025914699	5.47118746	4.887525271	6.316688823	6.311793718
Aradu.F510W	4.584361253	3.648465443	3.766595162	2.09423607	2.865918815
Aradu.UWB5V	0.23878686	0.799087306	0.748461233	1.937344392	1.992768431
Aradu.XBE20	-5.05889369	-5.64385619	-6.64385619	-4.05889369	-3.64385619
Aradu.S8E6L	1.10433666	0.956056652	0.887525271	2.247927513	1.726831217
Aradu.K13LE	NA	NA	NA	NA	NA

Aradu.EA9CU	2.448900951	2.704871964	2.22650853	1.150559677	1.070389328
Aradu.ZM5JL	1.510961919	2.347665656	1.613531653	2.35614381	2.62058641
Aradu.14QL4	3.09592442	2.9202933	3.317593505	0.454175893	1.682573297
Aradu.HD19V	1.769771739	1.847996907	1.632268215	1.952333566	2.482848283
Aradu.5DE0L	2.459431619	2.974529312	2.3950628	1.887525271	1.673556424
Aradu.8005S	2.195347598	1.855989697	1.959770155	2.333423734	2.709290636
Aradu.0GS9T	0.443606651	1.063502942	0.739848103	0.263034406	1.15704371
Aradu.DZ2GN	-1.78587519	-0.49410907	-1.15200309	2.014355293	2.577730931
Aradu.H76RB	2.849999259	2.879705766	2.606442228	1.464668267	1.752748591
Aradu.H2W20	0.748461233	1.22650853	0.815575429	1.752748591	1.700439718
Aradu.747H9	0.545968369	1.339137385	1.232660757	2.698218478	2.695993813
Aradu.JU778	4.063502942	4.007195501	4.025028794	1.655351829	2
Aradu.KS2FL	3.830863757	3.924099886	3.9202933	3.13422094	3.965322548
Aradu.UV8JW	0.189033824	0.879705766	0.799087306	1.691534165	2.283921772
Aradu.Z6UQQ	NA	NA	NA	NA	-4.05889369
Aradu.M4JP1	3.826802684	3.871843649	3.422233001	1.137503524	2.025028794
Aradu.Y9QAN	2.477677328	2.87774425	2.344828497	3.698218478	3.631104282
Aradu.1UI8P	NA	-6.64385619	-5.64385619	-4.64385619	-3.64385619
Aradu.4UY6C	2.107687869	2.137503524	2.117695043	1.350497247	1.722466024
Aradu.J9KV2	0.970853654	0.432959407	1.811471031	-0.26881676	-0.08926734
Aradu.VVM35	1.13093087	0.970853654	0.790772038	1.691534165	1.879705766
Aradu.50TKD	2.266036894	2.837943242	1.521050737	4.649040566	4.595742339

Aradu.IP5K7	1.516015147	5.155830172	4.447579197	3.040892431	3.282439805
Aradu.572L7	6.144250356	5.388533979	5.343763122	1.819668183	1.944858446
Aradu.7NM14	-3.32192809	-6.64385619	-5.05889369	NA	-1.18442457
Aradu.ZT956	NA	-3.64385619	-6.64385619	-3.47393119	-4.05889369
Aradu.P79WU	NA	NA	NA	NA	NA
Aradu.24PXZ	2.260025656	2.704871964	2.3950628	2.684818738	2.989139007
Aradu.HP3G1	-1.73696559	-0.86249648	-1.88896869	0	0.201633861
Aradu.DSS3T	-2.18442457	-2.18442457	-3.18442457	-0.51457317	1.049630768
Aradu.483P6	6.310158318	6.297007949	6.404800964	5.92243574	6.402244655
Aradu.3Q82R	NA	-6.64385619	NA	-5.64385619	NA
Aradu.L4KP0	-1.73696559	-1.25153877	-2.47393119	3.037382222	3.481557281
Aradu.GVV0Z	1.613531653	1.98550043	1.918386234	1.996388746	2.344828497
Aradu.MYL46	3.148934105	3.196921734	3.221877081	4.042644337	4.091699834
Aradu.NZS7L	-1.21759144	-0.07400058	-0.51457317	1.070389328	1.169925001
Aradu.WSK5V	3.478971805	3.7322692	3.683696454	4.484138131	4.505255801
Aradu.ZA91W	2.3950628	2.301587647	2.014355293	3.543495883	3.746312766
Aradu.10ZFH	1.687060688	2.464668267	2.010779839	1.807354922	1.794935663
Aradu.UZJ7G	-1.35845397	-1.05889369	-1.35845397	0.722466024	0.831877241
Aradu.GJN8J	2.831877241	2.950468414	2.659924558	-0.78587519	1.485426827
Aradu.QMU6X	2.198494154	2.572889668	2.289834465	2.769771739	3.014355293
Aradu.M707Y	1.59454855	2.424922088	2.321928095	3.75595577	3.792855352
Aradu.QC919	-5.05889369	NA	NA	-1.73696559	-0.55639335

Aradu.9YL2F	-5.05889369	-3.18442457	-2.32192809	-3.83650127	-4.05889369
Aradu.R5Y58	-2.47393119	-2.94341647	-2.83650127	-4.64385619	-3.32192809
Aradu.2GB7A	1.761285273	1.584962501	1.469885976	0.739848103	1.948600847
Aradu.R479P	4.814550423	3.148934105	2.9800253	1.695993813	2.003602237
Aradu.IS5GH	-1.88896869	-1	-1.68965988	1.731183242	1.3950628
Aradu.SK7RE	2.316145742	2.650764559	2.752748591	3.500802053	3.587364991
Aradu.V4U2I	0.526068812	1.464668267	0.613531653	3.365972428	3.3950628
Aradu.9T8AF	-0.78587519	-0.16812276	-0.32192809	0.545968369	1.244887059
Aradu.DA6I6	NA	NA	NA	NA	-3.18442457
Aradu.AB724	-5.64385619	-2.47393119	-3.18442457	0.214124805	-0.32192809
Aradu.C2W69	-2.73696559	-2.18442457	-2.83650127	NA	-2.73696559
Aradu.TZ5MC	NA	NA	NA	NA	NA
Aradu.8EU70	0.250961574	1.389566812	-0.53533173	3.63343121	4.275007047
Aradu.U1I3J	NA	NA	NA	NA	NA
Aradu.GP6IB	-5.05889369	-3.05889369	-4.05889369	-3.47393119	-3.32192809
Aradu.EM2BW	2.028569152	3.310340121	2.12763328	3.74092756	3.410069692
Aradu.U3EK7	0.613531653	1.384049807	0.555816155	0.90303827	1.176322773
Aradu.QH5RZ	NA	NA	NA	NA	NA
Aradu.9T1SG	-0.86249648	4.358958826	1.922197848	6.263597849	5.336997417
Aradu.P5PU5	5.071247819	4.401903472	4.926473911	2.310340121	2.275007047
Aradu.51BBB	5.91910168	6.658925511	6.169925001	2.618238656	2.897240426
Aradu.NGF0Q	-2	-4.32192809	-3.18442457	-0.45403163	-0.10469738
Aradu.47F3C	5.327687364	5.510012465	5.464341533	-0.01449957	0.432959407

Aradu.WJ709	-2.73696559	-1.43440282	-1.68965988	0.056583528	1.389566812
Aradu.RV6BG	0.22650853	1.214124805	1.333423734	0.790772038	1.201633861
Aradu.63RP0	-2.18442457	-1.55639335	-1.68965988	1.469885976	-0.20091269
Aradu.0ZB6D	-1.25153877	0.367371066	-1.59946207	0.40053793	0.765534746
Aradu.18R1L	2.49057013	2.910732662	2.794935663	2.657640005	3.201633861
Aradu.W4XF0	-0.59946207	0.22650853	-0.97143085	1.3950628	1.565597176
Aradu.K955R	NA	NA	-6.64385619	-4.64385619	4.589164237
Aradu.S4LWP	7.008764745	6.794935663	6.912290583	3.963474124	4.081510068
Aradu.KZX0F	5.971773447	5.303780748	5.302684614	3.881664619	4.70763522
Aradu.ISN4P	-0.07400058	0.250961574	-0.23446525	2.648465443	3.109360559
Aradu.AM65N	-3.18442457	-6.64385619	NA	NA	-6.64385619
Aradu.2I1UD	3.014355293	3.121015401	3.876762491	3.042644337	1.580145484
Aradu.R9Y5X	0.855989697	0.97819563	1.207892852	-0.30400619	0.86393845
Aradu.90TGE	1.40053793	1.859969548	1.604071324	2.063502942	2.392317423
Aradu.R23EZ	-2.64385619	-2.25153877	-2.73696559	-0.59946207	-0.21759144
Aradu.B2YUE	1.748461233	1.077242999	1.13093087	1.454175893	1.422233001
Aradu.DCZ6C	2.912649865	3.184280294	2.550900665	3.714795011	3.915520901
Aradu.Q77AH	1.589763487	2.304511042	1.66448284	4.934044647	4.313245852
Aradu.L5KF2	-4.64385619	-3.47393119	-2.39592868	0.678071905	1.042644337
Aradu.TBT3N	4.04701482	4.777156666	4.099295204	2.622930351	2.372952098
Aradu.U6514	-0.28630419	0.321928095	0.475084883	-0.4150375	0.378511623
Aradu.4094	3.168321116	3.295723025	3.117695043	4.04701482	4.61176256

W					
Aradu.S69T7	1.214124805	1.632268215	1.454175893	1.906890596	2.182692298
Aradu.N7DXE	2.752748591	2.698218478	2.698218478	2.682573297	2.941106311
Aradu.WB83L	1.769771739	2.241840184	1.981852653	3.109360559	3.008988783
Aradu.3TE64	0.333423734	0.23878686	0.163498732	0.545968369	0.575312331
Aradu.YXJ1Q	0.298658316	0.678071905	0.641546029	1.189033824	1.350497247
Aradu.B6TJT	-2.73696559	-2.64385619	-2	-2	-1.51457317
Aradu.XCA3M	NA	-5.64385619	NA	NA	-4.05889369
Aradu.C73IQ	6.956869798	7.135042286	6.414812061	5.447909749	5.383013015
Aradu.RLB9U	1.150559677	1.15704371	0.790772038	0.475084883	0.411426246
Aradu.P7M49	-0.05889369	-0.73696559	-1.68965988	-0.73696559	-0.23446525
Aradu.F2KAM	4.237257771	4.207892852	4.13422094	3.955126781	4.263785614
Aradu.YAN03	-1.35845397	-0.76121314	-1.51457317	2.910732662	3.267535798
Aradu.T1G5I	2.541019153	3.300123725	3.160274831	5.049195106	5.549977143
Aradu.755RJ	3.947666157	4.112700133	3.839959587	4.366671916	4.46727948
Aradu.5F8QQ	-1.47393119	-2.83650127	-3.47393119	-3.83650127	-3.18442457
Aradu.P86W5	2.794935663	2.969012308	2.702657543	3.023255352	3.22342255
Aradu.H77WY	3.193771743	3.061776198	2.994579724	4.065227623	4.19298317
Aradu.MF07S	NA	NA	NA	NA	2.752748591
Aradu.JJG2Q	-5.64385619	-4.05889369	-3.83650127	NA	-3.18442457
Aradu.89S1X	-1.05889369	-0.94341647	-1.68965988	-1.55639335	-0.47393119
Aradu.RQH55	1.084064265	1.263034406	1.298658316	1.541019153	2.080657663
Aradu.XLR8	-1.64385619	-1.15200309	-2.32192809	0.5360529	0.871843649

N					
Aradu.3G1RX	1.906890596	2.627606838	2.408711861	2.735522177	3.351910961
Aradu.AM0CF	NA	-6.64385619	NA	NA	-5.64385619
Aradu.YW23C	-1.78587519	-1.39592868	-1.83650127	-1.43440282	-0.66657627
Aradu.HXK3I	1.580145484	1.981852653	1.922197848	2	2.028569152
Aradu.945F4	-5.64385619	-6.64385619	-5.64385619	-4.64385619	-4.05889369
Aradu.XSZ7L	NA	-2.47393119	-2.55639335	-1.64385619	-2.32192809
Aradu.D66VA	3.560714954	3.46727948	3.595742339	1.959770155	2.09085343
Aradu.T2I1E	4.692092375	4.335569006	4.265286858	4.105175192	4.440952198
Aradu.Z639Y	NA	NA	NA	-4.64385619	NA
Aradu.RAS9Y	3.13093087	3.77925972	3.486714373	3.583759754	3.907852072
Aradu.TYQ4J	2.007195501	2.003602237	2.241840184	0.604071324	0.575312331
Aradu.HK2E0	NA	-5.64385619	NA	-4.05889369	-5.05889369
Aradu.F3XDM	4.060047384	4.492494152	4.170726276	4.336283388	4.522306893
Aradu.01YLW	-1.94341647	-1.18442457	-2.25153877	-0.08926734	0.454175893
Aradu.64LXI	-1.28630419	-0.94341647	-1.88896869	0.910732662	0.86393845
Aradu.7C8N3	3.464668267	3.389566812	3.305970521	3.075532631	3.361768359
Aradu.23FX2	3.13422094	3.480265122	3.150559677	3.847996907	3.943921327
Aradu.8QB2V	0.070389328	0.042644337	0.495695163	1.90303827	1.941106311
Aradu.0SM3T	-0.51457317	0.333423734	0.35614381	2.316145742	2.056583528
Aradu.XZ1FD	2.659924558	3.285402219	3.037382222	3.817623258	3.797012978
Aradu.J60UE	6.079164742	5.545350645	5.665904355	3.283921772	3.320484678
Aradu.40CK	NA	NA	NA	-5.64385619	-5.05889369

U					
Aradu.BE7PX	1.855989697	2.063502942	1.974529312	2.077242999	2.666756592
Aradu.2088W	1.40599236	0.773996325	0.90303827	-0.4150375	0.298658316
Aradu.S5K3Z	3.570462931	4.173926932	4.337711092	5.841721581	5.108942569
Aradu.V0C1K	-0.43440282	1.713695815	0.176322773	1.286881148	1.063502942
Aradu.LW197	5.875780063	5.584661908	5.780310099	3.410069692	3.715893371
Aradu.JMQ30	1.137503524	1.339137385	1.367371066	1.687060688	1.855989697
Aradu.ZK0JG	6.234003066	5.516015147	6.555509427	4.332707934	4.111865964
Aradu.B2BUH	0.214124805	-0.01449957	-0.08926734	-0.64385619	0.042644337
Aradu.RKM5L	3.276496666	3.945794957	3.334854269	3.678071905	3.943921327
Aradu.LZ6ZA	4.694880193	4.917431755	4.638073837	5.319039816	5.443606651
Aradu.C0E4H	NA	-5.05889369	NA	-4.05889369	NA
Aradu.RC5C1	NA	-1.51457317	-4.05889369	NA	-2.55639335
Aradu.QQF2S	0.464668267	0.807354922	0.584962501	0.90303827	1.807354922
Aradu.9Q62W	NA	NA	NA	2.070389328	1.327687364
Aradu.8T3RP	1.575312331	2.09085343	1.883620816	3.231125158	3.340562269
Aradu.196ZM	2.72900887	2.849999259	2.693765712	1.739848103	1.744161096
Aradu.0D7Q2	0.432959407	1.49057013	-0.08926734	0.622930351	0.871843649
Aradu.KW737	1.367371066	1.169925001	1.021479727	1.650764559	2.166715445
Aradu.0JV1U	1.541019153	2.438292852	1.744161096	3.066950244	3.137503524
Aradu.W5BH1	-3.64385619	-3.05889369	-2.39592868	-2.25153877	-1.88896869
Aradu.KZV99	2.173127433	1.996388746	1.735522177	2.408711861	2.761285273
Aradu.T3QX	-1.59946207	-2	-2.64385619	-0.76121314	-0.25153877

0

Aradu.Y1LT0	2.853995647	3.347665656	3.201633861	3.813524689	3.845991771
Aradu.8K2VK	3.272023189	3.757023247	3.384049807	4.547819957	4.427606173
Aradu.HCB3B	NA	NA	NA	NA	NA
Aradu.I2L2L	4.714245518	5.077242999	4.789207575	5.427941333	5.552438555
Aradu.W7CRW	-1.35845397	-0.68965988	-1.47393119	-3.05889369	0.042644337
Aradu.JAV3X	0.970853654	1.22650853	0.641546029	3.240314329	3.334854269
Aradu.CX8YW	NA	-6.64385619	NA	NA	-0.02914635
Aradu.D588D	-0.02914635	-0.68965988	-0.47393119	-2.94341647	-4.05889369
Aradu.S0XYN	4.300123725	4.705977902	4.472487771	0.695993813	0.855989697
Aradu.W9DC5	6.6441447	7.010332283	6.703073006	7.270155139	7.441699257
Aradu.BS3JU	-0.05889369	-0.05889369	-0.25153877	-1.12029423	-0.49410907
Aradu.K9ZYN	4.196921734	3.912649865	3.843983844	4.615298579	4.59275601
Aradu.B3V05	-2.83650127	-1.73696559	-3.32192809	1.084064265	1.59454855
Aradu.TVX9T	2.014355293	2.503348735	2.63691458	1.895302621	2.257010618
Aradu.VT0DP	-0.18442457	0.286881148	-1.02914635	3.726831217	3.844988157
Aradu.0B0D2	1.321928095	1.687060688	0.815575429	2.313245852	2.316145742
Aradu.0MJ0X	NA	NA	NA	NA	NA
Aradu.4XL4D	3.397802962	3.482848283	3.488000771	3.503348735	3.578938713
Aradu.M4TMQ	0.565597176	0.632268215	0.443606651	0.790772038	0.97819563
Aradu.Z1H9W	2.821710215	3.389566812	3.042644337	3.730096466	4.515384461
Aradu.LBJ9G	-1.05889369	-1.59946207	-0.76121314	0.286881148	0.22650853
Aradu.VPD9	2.518535139	2.845991771	2.503348735	2.070389328	2.776103988

Z					
Aradu.EG0P5	NA	-3.64385619	-6.64385619	-1.94341647	7.277519887
Aradu.UEG62	2.543495883	2.956056652	2.757023247	3.267535798	3.294253136
Aradu.4H56Q	0.411426246	0.669026766	0.137503524	0.790772038	1.726831217
Aradu.N1IDK	1.769771739	2.46727948	1.726831217	3.331991778	3.378511623
Aradu.AXH5G	1.485426827	1.883620816	1.803227036	2.283921772	2.10433666
Aradu.K73MN	2.361768359	2.689299161	2.560714954	2	2.435628594
Aradu.6011J	-4.05889369	-5.64385619	-3.05889369	NA	-4.05889369
Aradu.55M8P	NA	-5.05889369	NA	-6.64385619	-5.64385619
Aradu.5TM0N	0.82374936	1.948600847	1.056583528	2.56315813	3.132576843
Aradu.6F2AZ	-5.05889369	-6.64385619	-6.64385619	-3.64385619	-4.64385619
Aradu.WB4GB	2.039138394	2.95419631	2.298658316	-0.12029423	-0.35845397
Aradu.VLM3K	NA	-5.05889369	-5.05889369	NA	NA
Aradu.UKP00	0.678071905	0.97819563	0.443606651	1.944858446	2.10433666
Aradu.83KXV	-3.83650127	-6.64385619	NA	NA	NA
Aradu.1ES3P	2.553360503	2.859969548	2.680324357	2.757023247	3.171527106
Aradu.VCF9B	-0.53533173	-0.64385619	-0.4150375	-0.94341647	-1.51457317
Aradu.F3FT3	2.833902077	2.272023189	2.241840184	3.056583528	3.390942773
Aradu.70AAJ	NA	-5.05889369	NA	NA	-4.32192809
Aradu.BNJ62	5.262658655	5.390254956	5.28503225	1.627606838	1.673556424
Aradu.PI1VW	1.989139007	2.435628594	2.070389328	2.859969548	3.153805336
Aradu.R6RRG	-1	-0.86249648	-1.21759144	-0.05889369	0.214124805
Aradu.88EG	1.757023247	1.459431619	1.189033824	1.922197848	2.269033146

A					
Aradu.46VDC	-1.47393119	-0.97143085	-1.05889369	1	1.480265122
Aradu.SGR1V	11.3658467	10.6953066	11.20244221	7.014132087	7.033973543
Aradu.16E7C	0.613531653	0.970853654	0.704871964	0.98550043	1.531069493
Aradu.X3A7J	-0.97143085	0.464668267	0.432959407	1.10433666	0.659924558
Aradu.ZFT0U	-0.37706965	0.321928095	-0.12029423	1.914564523	1.996388746
Aradu.G2R4U	2.304511042	2.711494907	2.298658316	2.908812908	3.407352751
Aradu.TBP46	0.014355293	-0.13606155	0.014355293	0.748461233	1.182692298
Aradu.34QFB	-0.20091269	0.584962501	1.182692298	0.748461233	1.263034406
Aradu.8LR63	0.748461233	1.843983844	0.731183242	2.513490746	2.935459748
Aradu.DVH8N	2.14404637	1.333423734	1.580145484	2.09085343	2.62058641
Aradu.32310	3.09592442	3.294253136	2.849999259	2.40599236	2.937344392
Aradu.H3050	-0.05889369	-1.21759144	-0.64385619	-2.73696559	-3.32192809
Aradu.WC9V5	NA	NA	-5.05889369	-2.18442457	-2.05889369
Aradu.U5NU2	-6.64385619	NA	NA	-5.05889369	5.155425432
Aradu.CD8RE	0.344828497	0.722466024	0.176322773	1.201633861	1.042644337
Aradu.2DG1V	3.786596362	4.386121157	3.996388746	5.108942569	5.04745114
Aradu.V66GG	4.516015147	4.464014725	4.316869805	2.799087306	3.234194723
Aradu.QX6W8	4.434294618	4.370164281	4.238022518	3.304511042	3.513490746
Aradu.WIA05	-0.51457317	-0.05889369	-1.21759144	2.462052319	2.477677328
Aradu.KG1H9	0.411426246	0.137503524	-0.26881676	-1	-0.86249648
Aradu.B1CQK	4.080657663	4.350497247	4.324810603	4.099295204	4.327687364
Aradu.LSK5	-3.32192809	-3.05889369	-2.94341647	-1.55639335	-2.47393119

Q					
Aradu.0PC6S	0.82374936	1.459431619	0.367371066	1.63691458	1.748461233
Aradu.E1KPT	0.678071905	1.275007047	0.731183242	3.648465443	3.621758857
Aradu.8T892	NA	-0.83650127	-6.64385619	-5.05889369	-3.32192809
Aradu.6E0QJ	2.776103988	3.485426827	3.313245852	3.462052319	3.552131108
Aradu.G4M3I	4.299391206	4.350497247	4.206330648	3.808385051	4.186659017
Aradu.VX0W5	-0.45403163	0.150559677	0.98550043	-1.25153877	-1.15200309
Aradu.0RA4R	0.918386234	0.98550043	0.632268215	2.422233001	2.493134922
Aradu.69GC2	NA	-5.64385619	NA	-1.15200309	-0.94341647
Aradu.QI4L1	-0.577767	-1.59946207	-1.39592868	NA	-0.15200309
Aradu.U6RYQ	-0.4150375	0.613531653	-0.30400619	2.15704371	2.09085343
Aradu.P81TA	1.432959407	1.618238656	1.967168608	1.604071324	2.13422094
Aradu.8W1FQ	0.59454855	1.292781749	0.604071324	0.925999419	0.948600847
Aradu.YHW10	-5.64385619	-5.64385619	NA	-3.64385619	1.275007047
Aradu.934TY	0.992768431	-0.35845397	-0.23446525	-3.05889369	-1.25153877
Aradu.K8WQA	0.765534746	1.316145742	0.782408565	2.477677328	2.207892852
Aradu.JG94E	-6.64385619	-5.05889369	-5.05889369	NA	-0.97143085
Aradu.51DJW	NA	NA	NA	NA	-5.64385619
Aradu.MNQ43	2.821710215	2.786596362	2.613531653	2.737686761	3.100977648
Aradu.RAP3E	0.807354922	1.63691458	1.333423734	2.480265122	2.924099886
Aradu.4Q38F	NA	NA	NA	-4.32192809	-1.32192809
Aradu.580FR	1.521050737	1.646162657	1.641546029	-1.25153877	-0.39592868
Aradu.G0IJA	-3.47393119	-3.18442457	-3.64385619	-4.32192809	-3.47393119

Aradu.02LQD	-1.59946207	0.137503524	0.214124805	-1.83650127	-2.25153877
Aradu.C014T	-1.68965988	-2.32192809	-2	-0.59946207	0.137503524
Aradu.32EAS	1.13093087	1.333423734	0.948600847	2.347665656	2.675815931
Aradu.82T8W	0.084064265	0.622930351	-0.15200309	3.317593505	3.349082146
Aradu.CI3I2	-1.51457317	-0.47393119	-1.47393119	-2.73696559	1.570462931
Aradu.VX30A	2.443606651	3.224966365	3.307428525	3.161887682	3.381283373
Aradu.JFA7C	5.519479002	4.844486088	5.096767855	1.40053793	1.195347598
Aradu.IX4YQ	NA	NA	NA	NA	-1.21759144
Aradu.2P8HG	4.289096702	4.100136671	4.404630684	2.833902077	3.397802962
Aradu.GS1TU	NA	-3.47393119	-3.64385619	-2.12029423	-3.18442457
Aradu.1G3YF	-2.47393119	-1.39592868	-1.21759144	3.070389328	2.927896454
Aradu.R4B3S	3.708187236	3.485426827	3.627606838	3.106013238	3.203201156
Aradu.QA0D1	0.815575429	1.169925001	0.555816155	1.189033824	1.531069493
Aradu.BD9UN	5.21490191	5.132988043	5.098032083	2.709290636	3.142413438
Aradu.S894K	0.895302621	1.378511623	0.839959587	1.807354922	1.929790998
Aradu.6I8N8	2.553360503	2.032100843	2.62058641	-1.02914635	0.298658316
Aradu.6M72C	0.565597176	2.117695043	1.735522177	-0.07400058	-0.07400058
Aradu.VV1WJ	-1.59946207	-1.94341647	-1.21759144	-1.08926734	-3.18442457
Aradu.WYX50	3.260025656	2.448900951	1.883620816	2.021479727	2.625270489
Aradu.K08LS	2.097610797	2.5360529	2.070389328	3.66106548	3.675815931
Aradu.3D2PD	2.289834465	2.577730931	2.107687869	2.983677695	2.941106311
Aradu.RGH5	2.944858446	2.733354341	2.435628594	2.885574364	3.464668267

6					
Aradu.VS34U	4.823239844	4.90303827	4.757556689	5.203592714	5.362820526
Aradu.0Q5NS	NA	NA	-6.64385619	-4.32192809	-3.47393119
Aradu.R2B96	-0.15200309	-0.51457317	-0.07400058	NA	-3.64385619
Aradu.N1Z7I	NA	NA	NA	NA	NA
Aradu.DA1ZW	NA	NA	NA	NA	-5.64385619
Aradu.12T0R	NA	NA	-6.64385619	NA	-3.64385619
Aradu.U23UQ	0.189033824	-0.04394335	0.097610797	0.650764559	1.757023247
Aradu.64V56	1.584962501	1.782408565	1.560714954	1.941106311	2.23878686
Aradu.H46EZ	NA	NA	NA	NA	1.918386234
Aradu.MUM0J	7.41379715	7.57500972	7.782343034	3.890446693	4.040015679
Aradu.3G22S	NA	NA	NA	NA	-3.64385619
Aradu.U80Z7	2.169925001	2.565597176	2.250961574	2.422233001	2.821710215
Aradu.UNB9U	1.541019153	0.695993813	0.970853654	-1.55639335	-1.05889369
Aradu.HX0A2	3.171527106	3.54225805	3.232660757	1.895302621	2.295723025
Aradu.N2VA8	NA	NA	NA	-6.64385619	-5.64385619
Aradu.P28IF	1.389566812	2.100977648	0.839959587	3.203201156	3.297191417
Aradu.29IAA	3.365972428	3.140778656	3.189033824	3.189033824	3.349082146
Aradu.C4HNC	5.267161218	5.282069075	5.128458384	5.647890091	5.877007993
Aradu.45ENN	1.613531653	2.22342255	2	2.761285273	3.073820233
Aradu.AU7A2	2.596935142	2.815575429	2.801158656	3.240314329	3.50589093
Aradu.2X0ZQ	3.148934105	3.460742564	3.241840184	4.384049807	4.651912745
Aradu.25H68	-0.4150375	0.150559677	0.214124805	0.613531653	1.560714954

Aradu.AN6BF	1.843983844	2.416839742	2.361768359	3.608809243	3.876762491
Aradu.14EED	-4.05889369	-5.05889369	-5.64385619	NA	-6.64385619
Aradu.JVB3U	6.218587452	6.601845057	6.043519494	7.908932967	7.824004051
Aradu.A13V2	1.956056652	2.408711861	2.241840184	2.851998837	3.234194723
Aradu.WJ2ZP	4.363171077	4.627606838	5.20867332	-0.26881676	0.464668267
Aradu.4S6WR	1.316145742	1.641546029	1.372952098	2.370164281	2.40053793
Aradu.305W9	-1.25153877	-1.08926734	-1.47393119	-0.81096618	2.107687869
Aradu.ZM6VG	2.084064265	2.765534746	2.372952098	3.641546029	3.468583317
Aradu.MD214	2.622930351	2.613531653	2.622930351	3.851998837	3.979110755
Aradu.C65VQ	1.378511623	1.718087584	1.627606838	2.22650853	3.09085343
Aradu.SG5AP	NA	-6.64385619	NA	NA	1.321928095
Aradu.W9Q7M	1.757023247	1.956056652	1.786596362	2.480265122	2.803227036
Aradu.Q5JK4	0.097610797	0.111031312	0.056583528	1.599317794	1.59454855
Aradu.1H4CB	3.631104282	4.068670811	3.855989697	4.739308071	5.017921908
Aradu.WEZ9F	1.042644337	1.432959407	1.500802053	1.350497247	1.687060688
Aradu.CG16W	1.23878686	1.786596362	1.726831217	1.521050737	2.386810946
Aradu.0C9TU	3.987320866	4.185073638	4.09085343	1.207892852	0.807354922
Aradu.45ASZ	0.367371066	0.871843649	0.713695815	1.510961919	1.722466024
Aradu.KIY9T	NA	-5.64385619	-6.64385619	NA	0.925999419
Aradu.HRR2H	NA	NA	NA	NA	NA
Aradu.8VT0P	0.831877241	1.831877241	1.438292852	2.053111336	2.339137385
Aradu.52XV	0.933572638	0.713695815	0.298658316	-0.4150375	0.739848103

D					
Aradu.ATW70	2.916476644	3.129283017	2.87774425	3.54225805	3.63691458
Aradu.N0QH3	2.220329955	2.839959587	1.459431619	3.921245889	4.192194165
Aradu.0AW6N	2.22650853	2.925999419	2.94673086	2.042644337	2.23878686
Aradu.UK6QI	NA	-6.64385619	NA	-3.64385619	-1.39592868
Aradu.YE5Q3	1.269033146	1.555816155	1.327687364	1.731183242	1.713695815
Aradu.IW3RC	2.669026766	6.656639374	5.296090263	1.718087584	1.918386234
Aradu.XAD2K	-4.64385619	-3.47393119	-6.64385619	NA	-1.88896869
Aradu.9M4ZC	3.439623138	3.459431619	3.102658131	1.454175893	1.89917563
Aradu.M7MKB	NA	-2.39592868	-2.94341647	-3.18442457	-2.64385619
Aradu.Q123V	2.039138394	2.347665656	2.201633861	2.443606651	2.392317423
Aradu.P7YVS	-1.43440282	-1.15200309	-0.81096618	-3.32192809	-1.68965988
Aradu.3J7Z6	-1.43440282	-0.88896869	-0.97143085	0.201633861	0.189033824
Aradu.MX2L0	2.498250868	2.587364991	2.495695163	1.589763487	1.827819025
Aradu.A9RVD	5.667892125	5.502394256	5.141187525	5.904965719	6.374517881
Aradu.K9R11	-2.32192809	-1.59946207	-3.47393119	0.5360529	0.526068812
Aradu.IC00B	2.053111336	2.650764559	2.277984747	3.313245852	3.707082992
Aradu.B3VS2	5.46662712	5.706254254	5.570159302	6.495855027	6.747253114
Aradu.EX9SJ	0.367371066	0.432959407	-0.04394335	0.176322773	0.565597176
Aradu.65ZNS	2.039138394	2.244887059	2.09423607	2.937344392	3.142413438
Aradu.M3FDP	1.782408565	1.464668267	0.887525271	0.014355293	0.150559677
Aradu.TC25V	1.695993813	2.364572432	1.86393845	2.737686761	3.007195501
Aradu.PS0E	3.422233001	3.316145742	3.3305584	3.738767837	4.019701914

5					
Aradu.HL1PG	4.374343989	4.807870078	4.700439718	5.513174885	5.6622055
Aradu.T0IJJ	-0.88896869	-1.15200309	-0.94341647	-2.83650127	2.220329955
Aradu.T1478	-2.94341647	-2.55639335	-5.05889369	2.752748591	2.941106311
Aradu.4Q3MP	2.550900665	2.61117238	2.488000771	2.378511623	3.35473424
Aradu.RIZ04	0.014355293	0.695993813	0.124328135	1.526068812	1.63691458
Aradu.R1DHB	-0.47393119	-0.20091269	-0.73696559	1.280956314	1.427606173
Aradu.XDP02	NA	NA	NA	NA	-6.64385619
Aradu.JJA0L	NA	-4.64385619	-5.05889369	-1.59946207	0.333423734
Aradu.CF5Z2	NA	NA	NA	NA	-5.05889369
Aradu.X0SMT	3	3.358958826	2.950468414	3.442280035	3.605257263
Aradu.LV8M3	4.501439145	5.084489525	5.144862143	4.34978987	4.299391206
Aradu.6K81G	4.788685711	4.814037648	4.831370588	4.933572638	5.151777655
Aradu.ZI52D	2.411426246	2.821710215	4.752748591	1.584962501	2.140778656
Aradu.A3E1X	NA	NA	NA	2.498250868	1.344828497
Aradu.J08SD	3.016139703	3.304511042	3.654206378	3.538538164	3.819668183
Aradu.S6CPD	1.989139007	2.060047384	2.324810603	1.214124805	0.918386234
Aradu.N09A5	-2.94341647	-2.94341647	-3.05889369	-3.05889369	-2.18442457
Aradu.TJ219	1.372952098	1.448900951	1.292781749	1.970853654	2.361768359
Aradu.8W40E	-2.55639335	-0.34007544	-0.55639335	-0.59946207	-0.30400619
Aradu.EW7BI	0.35614381	1.339137385	1.555816155	1.367371066	2.691534165
Aradu.JUW7B	2.931683057	2.673556424	2.277984747	2.163498732	2.56315813
Aradu.TJL9X	4.672990994	5.822730148	5.299391206	7.325170512	8.120186028

Aradu.304FM	-5.05889369	-4.32192809	-5.64385619	NA	-4.32192809
Aradu.92FHF	1.077242999	1.819668183	1.454175893	1.622930351	1.992768431
Aradu.X3FXV	5.906169068	5.934516502	5.913128768	0.941106311	1.843983844
Aradu.Z5U1L	1.761285273	1.176322773	2.014355293	-2.12029423	-1.08926734
Aradu.VK9S0	3.478971805	3.845991771	4.078951341	2.754887502	3.510961919
Aradu.0X9JH	0.97819563	1.280956314	1.195347598	2.189033824	2.397802962
Aradu.6DL9M	0.678071905	1.316145742	1.201633861	1.722466024	1.608809243
Aradu.HYT44	1.989139007	2.09423607	2.10433666	2.851998837	2.989139007
Aradu.LUI60	-0.55639335	-0.26881676	-1.21759144	0.82374936	1.550900665
Aradu.XN89N	NA	NA	-6.64385619	NA	0.137503524
Aradu.KR1YF	0.659924558	1.049630768	0.910732662	1.333423734	2.010779839
Aradu.S0K8G	2.010779839	1.646162657	1.422233001	4.168321116	4.433627167
Aradu.CU20J	3.270528942	3.793895883	3.244887059	5.013908845	4.943921327
Aradu.TI81R	4.294988268	4.791293149	4.292781749	5.365622556	5.553360503
Aradu.69TMW	2.744161096	3.634593268	2.769771739	4.556429415	4.361768359
Aradu.HH2HB	-4.64385619	-2.64385619	-3.05889369	-2.25153877	-0.577767
Aradu.B219P	-2.47393119	-4.05889369	-2.05889369	-2.05889369	-2.94341647
Aradu.SB9IZ	1.361768359	1.070389328	1.220329955	1.748461233	1.765534746
Aradu.J85DC	NA	NA	NA	NA	-6.64385619
Aradu.T5UQE	3.264536431	3.75595577	3.601696516	4.043519494	4.672990994
Aradu.7K1LW	3.310340121	2.992768431	3.253989266	3.247927513	3.671293372
Aradu.I4Z8N	-2.18442457	-1.64385619	-2	-1.02914635	-1.08926734

Aradu.AKG36	-0.05889369	0.565597176	0.333423734	0.344828497	2.100977648
Aradu.6V9D4	2.974529312	3.464668267	2.495695163	4.308156975	4.29130886
Aradu.6X61Q	-1.21759144	-1.21759144	-1.25153877	3.396433531	3.814550423
Aradu.JA2NX	0.879705766	0.650764559	0.739848103	1.117695043	1.765534746
Aradu.T6KM7	-5.05889369	-6.64385619	-6.64385619	-3.83650127	-5.05889369
Aradu.52U3G	-0.66657627	1.15704371	2.641546029	-3.64385619	-2.94341647
Aradu.JF3WA	0.82374936	1.545968369	0.042644337	4.325530332	4.011674533
Aradu.KB85J	1.10433666	1.480265122	1.137503524	0.815575429	0.82374936
Aradu.F4EXD	0.422233001	0.839959587	0.855989697	0.59454855	0.432959407
Aradu.T37GN	1.028569152	2.339137385	2.485426827	2.289834465	2.9800253
Aradu.SH4SS	2.64385619	2.761285273	2.500802053	3.416839742	3.533563348
Aradu.K81IQ	-1.21759144	-1.43440282	-1.73696559	-2.05889369	-0.86249648
Aradu.06CIQ	NA	NA	NA	NA	-5.64385619
Aradu.L0BQ6	1.189033824	1.992768431	1.59454855	2.22650853	2.90303827
Aradu.NTQ7W	0.604071324	0.641546029	0.704871964	-0.78587519	-0.21759144
Aradu.P3PDP	3.007195501	3.738767837	3.450221496	3.498250868	3.443606651
Aradu.3GC8H	0.35614381	1.042644337	0.50589093	1.137503524	1.49057013
Aradu.4E354	2.577730931	3.044394119	2.541019153	4.300123725	4.359661722
Aradu.MI9SZ	4.171527106	4.798569004	3.944858446	5.809671678	5.661635602
Aradu.88AFG	NA	NA	NA	NA	NA
Aradu.18DQZ	4.255500733	4.229587923	4.263785614	2.22650853	2.430285273
Aradu.J3H3Z	3.097610797	2.657640005	2.969012308	2.053111336	1.952333566

Aradu.GL6NL	4.194559886	3.97819563	4.009884589	3.904002316	4.14404637
Aradu.GT5D9	2.488000771	3.283921772	3.042644337	0.056583528	1.659924558
Aradu.IXS5D	3.153805336	2.897240426	2.678071905	3.102658131	3.385431037
Aradu.V66GY	-2	-0.76121314	-1.83650127	0.163498732	-0.53533173
Aradu.CT5EJ	2.333423734	4.773996325	3.986410935	1.189033824	2.646162657
Aradu.UE0PI	NA	NA	NA	NA	-3.47393119
Aradu.443KM	1.521050737	1.627606838	1.541019153	0.40053793	1.214124805
Aradu.PS85R	2.560714954	2.845991771	2.577730931	3.693765712	3.961623328
Aradu.D77GP	-0.47393119	0.275007047	-1.51457317	1.704871964	2.50589093
Aradu.YQ4PR	1.5360529	1.944858446	1.992768431	1.565597176	1.292781749
Aradu.94ETY	-2.12029423	-2.94341647	-3.64385619	-2.94341647	-0.08926734
Aradu.AJZ98	2.280956314	2.711494907	2.3950628	5.060479781	5.315421316
Aradu.SW5JR	-4.05889369	-2.64385619	-4.64385619	3.727920455	4.94673086
Aradu.J5HFQ	1.695993813	2.430285273	1.735522177	2.843983844	3.246408087
Aradu.HRJ8J	1.63691458	1.339137385	1.454175893	0.722466024	1.339137385
Aradu.XL7AT	0.516015147	1.014355293	0.555816155	1.084064265	1.438292852
Aradu.BR4VK	1.03562391	1.250961574	0.963474124	-0.62148838	-1.64385619
Aradu.VE1AV	NA	-6.64385619	NA	-2.94341647	-1.25153877
Aradu.WL92I	5.118110506	5.567423758	4.780835002	6.980825052	6.682011391
Aradu.14Z9Y	NA	NA	NA	-2.12029423	-1.25153877
Aradu.57NVY	1.799087306	2.207892852	2.070389328	2.925999419	3.107687869
Aradu.FG20	-1.64385619	-0.21759144	-0.73696559	1.604071324	-0.43440282

F					
Aradu.0EZ1S	3.951401292	4.13093087	4.274261661	1.678071905	1.778208576
Aradu.IT5KP	NA	-5.05889369	-5.64385619	-1.35845397	0.275007047
Aradu.I022X	2.835924074	3.085764554	3.025028794	3.892391026	4.378511623
Aradu.X2LCY	4.227278994	4.290571851	4.048759312	2.438292852	2.841973119
Aradu.EF2HR	0.782408565	0.773996325	0.454175893	3.295723025	3.327687364
Aradu.Z8XIW	4.142413438	4.458119481	4.726831217	2.09085343	2.107687869
Aradu.W9SJN	-5.05889369	NA	NA	3.815575429	2.659924558
Aradu.FA889	-2.39592868	-1.68965988	-3.05889369	1.565597176	1.650764559
Aradu.UX5R5	-0.25153877	0.790772038	1.014355293	-0.26881676	1.298658316
Aradu.X8R8H	0.879705766	1.344828497	1.056583528	2.378511623	2.503348735
Aradu.M6LMC	3.017921908	3.026800059	2.841973119	4.147306699	4.173926932
Aradu.NJ1ET	4.575917361	4.627606838	4.462706751	3.150559677	2.748461233
Aradu.1ZQ3K	-1.68965988	-1.47393119	-1.55639335	-0.66657627	-0.88896869
Aradu.JGT0L	-0.10469738	0.650764559	-0.21759144	1.263034406	1.748461233
Aradu.NFL6Z	-3.18442457	-3.64385619	-2.94341647	-2	-1.51457317
Aradu.U7A5U	8.380807357	7.699468346	8.108785791	4.004501392	4.050501697
Aradu.40AX1	-3.32192809	-3.05889369	-3.47393119	-0.28630419	0.286881148
Aradu.77XML	-5.05889369	-6.64385619	NA	-4.64385619	-4.64385619
Aradu.V2RP9	-1.59946207	-1.25153877	-1.08926734	-2.73696559	-2.12029423
Aradu.IU0EI	3.928844037	4.724650272	3.838951767	5.792074462	5.894817763
Aradu.J7EAE	1.944858446	2.691534165	2.084064265	3.667892125	3.772941338
Aradu.57TM	NA	-4.64385619	-5.05889369	-0.81096618	-2.18442457

Q					
Aradu.IM4U5	1.875780063	2.114367025	1.713695815	2.485426827	2.87774425
Aradu.GB23E	3.234194723	3.518535139	3.351910961	3.336283388	4.036503334
Aradu.Z1QR7	-4.64385619	-6.64385619	-4.64385619	-0.25153877	-0.53533173
Aradu.M0QV5	4.215678597	4.503348735	4.182692298	5.551208374	5.581953751
Aradu.EQB6S	4.525442506	4.780835002	4.435628594	5.206721357	5.787641414
Aradu.3T5V1	-3.64385619	-3.64385619	-4.64385619	-2	-2
Aradu.N44D1	5.857980995	6.190022175	5.969242604	0.731183242	0.98550043
Aradu.44KEY	2.646162657	2.707082992	2.528571319	3.927896454	4.272769732
Aradu.QH2VI	2.893362211	3.337711092	3.264536431	3.679198571	3.785550552
Aradu.P477D	-1.08926734	-0.76121314	-1.35845397	2.310340121	2.021479727
Aradu.X5GZZ	2.56315813	2.435628594	2.22650853	2.389566812	2.687060688
Aradu.U1S9C	2.737686761	2.963474124	2.657640005	4.125155131	4.055716264
Aradu.862GA	2.313245852	2.757023247	2.456806149	2.422233001	2.691534165
Aradu.HVD76	1.176322773	1.124328135	1	1.918386234	2.575312331
Aradu.E9IFL	4.90014226	4.957914599	4.711494907	3.99095486	4.155425432
Aradu.W2VYJ	2.776103988	3.166715445	2.889473543	3.670160514	3.919340082
Aradu.6S44C	1.879705766	1.937344392	1.90303827	2.464668267	2.650764559
Aradu.M35ZU	1.811471031	1.555816155	1.292781749	1.516015147	1.952333566
Aradu.5K9HV	2.111031312	1.673556424	1.531069493	3.403267722	3.819668183
Aradu.W20TC	1.361768359	1.589763487	1.195347598	0.40053793	0.176322773
Aradu.HB1CW	NA	NA	NA	NA	NA
Aradu.G0P4	-5.05889369	-4.32192809	-3.83650127	-4.64385619	-4.64385619

Z					
Aradu.0QQ3Z	1.584962501	2.292781749	1.906890596	3.422233001	3.679198571
Aradu.ANN7C	4.873813198	5.469234794	5.252097703	3.595742339	3.046141782
Aradu.4B6K6	5.158660175	5.319039816	4.954661621	5.699051844	5.817879032
Aradu.G4PX6	-1.78587519	0.263034406	-0.62148838	5.033863452	3.718087584
Aradu.YM44Q	-0.66657627	-1.12029423	-2.05889369	0.263034406	0.855989697
Aradu.C4WL2	2.321928095	2.523561956	2.599317794	-0.26881676	-0.62148838
Aradu.06C7F	4.234961095	5.18030702	4.315421316	6.806839582	6.46662712
Aradu.1W7NB	1.49057013	2.028569152	1.731183242	2.575312331	2.873813198
Aradu.K2YQU	-0.26881676	0.622930351	1.459431619	NA	-3.47393119
Aradu.N4HHX	-3.18442457	-3.05889369	-1.88896869	-6.64385619	NA
Aradu.RF6Z3	0.014355293	0.782408565	0.871843649	2.140778656	1.867896464
Aradu.NXS8X	1.09085343	1.292781749	1.028569152	2.464668267	2.66448284
Aradu.HIV9Z	0.137503524	0.918386234	0.150559677	0.443606651	0.432959407
Aradu.R3US5	2.99095486	2.759155834	2.925999419	2.700439718	2.895302621
Aradu.NKZ2V	0.918386234	1.765534746	1.575312331	1.298658316	1.286881148
Aradu.SA3GU	1.049630768	1.09085343	1.117695043	2.769771739	3.010779839
Aradu.Y9Y02	2.908812908	3.326249701	3.068670811	4.473786912	4.572283367
Aradu.IN3UA	2.166715445	2.82984956	1.550900665	5.845740933	6.483815777
Aradu.34FHG	4.539158811	4.54225805	4.449561375	2.9800253	3.187451054
Aradu.I23XS	-1.35845397	-1.64385619	-0.86249648	0.333423734	0.137503524
Aradu.8WJ1L	-0.04394335	0.454175893	-0.07400058	1.589763487	1.541019153
Aradu.U2NU	3.037382222	3.111031312	3.196921734	2.857980995	3.214124805

Z					
Aradu.Z7XZ9	6.252854626	6.621319301	6.899538193	4.900625333	4.828326926
Aradu.0RW41	1.214124805	1.3950628	1.427606173	0.992768431	1.339137385
Aradu.PZ3X7	0.925999419	1.786596362	1.555816155	1.40599236	2.748461233
Aradu.KR62J	-1.25153877	-0.37706965	-2.18442457	-2.18442457	0.378511623
Aradu.N8RFP	2.358958826	1.839959587	1.895302621	0.056583528	1.220329955
Aradu.1A2PM	3.904002316	3.893362211	3.737686761	2.087462841	2.211012193
Aradu.117B0	NA	NA	NA	NA	1.604071324
Aradu.I9X8G	-3.83650127	-2.47393119	-3.64385619	-0.34007544	0.214124805
Aradu.Y5MLI	1.641546029	1.82374936	1.691534165	2.375734539	2.769771739
Aradu.5Z6H3	1.704871964	2.137503524	2.003602237	2.232660757	2.493134922
Aradu.4P8D3	0.250961574	0.070389328	0.35614381	-0.10469738	0.275007047
Aradu.FN4CD	1.35614381	1.944858446	1.500802053	2.124328135	2.86393845
Aradu.CDX53	3.056583528	3.212569339	3.200064862	3.025028794	3.671293372
Aradu.0WP1T	0.748461233	1.384049807	0.839959587	1.15704371	1.811471031
Aradu.GVH4D	2.765534746	2.735522177	2.726831217	2.750606505	3.111031312
Aradu.3N57P	-5.05889369	-6.64385619	-5.64385619	NA	-5.05889369
Aradu.L4J2W	-1.28630419	-1.35845397	-1.47393119	-3.32192809	-1.83650127
Aradu.SXB7Z	1.111031312	1.682573297	1.077242999	2.942983598	4.193771743
Aradu.S6195	3.976363636	3.974529312	3.800123353	1.859969548	1.700439718
Aradu.7SN56	-0.71311885	-1.55639335	-0.04394335	2.066950244	1.773996325
Aradu.6I2MF	2.543495883	2.865918815	2.678071905	2.414135533	3.313245852
Aradu.KHJ4	3.001802243	2.715893371	3.153805336	2.257010618	1.827819025

B					
Aradu.2T909	1.077242999	0.910732662	0.641546029	2.59454855	3.327687364
Aradu.G14M4	-0.81096618	-0.81096618	-0.43440282	0.622930351	1.887525271
Aradu.KPJ13	4.7382274	4.280956314	4.070389328	-0.12029423	0.23878686
Aradu.N89Q4	-5.64385619	-3.83650127	-4.32192809	-1.32192809	-1.35845397
Aradu.KNP7I	-1.12029423	0.604071324	-0.62148838	2.275007047	2.073820233
Aradu.D3RAM	NA	NA	NA	NA	NA
Aradu.2IP37	-1	-0.81096618	-2.25153877	1.176322773	1.049630768
Aradu.WT2VD	-1.02914635	0.070389328	-2.64385619	2.748461233	3.427606173
Aradu.EQ5J6	1.361768359	2.989139007	2.495695163	2.592158002	3.09085343
Aradu.88Z5T	2.316145742	2.333423734	2.344828497	1.682573297	2.066950244
Aradu.LL82A	2.771885579	3.061776198	2.733354341	3.7322692	3.821710215
Aradu.VL3IZ	NA	-2.55639335	-3.05889369	-4.32192809	-2.32192809
Aradu.Y7I9D	NA	-6.64385619	-5.64385619	-3.32192809	-5.64385619
Aradu.83F0C	3.203201156	3.935459748	3.44625623	4.700439718	4.771885579
Aradu.HZ9T9	3.987320866	3.715893371	3.764473551	3.244887059	3.699329526
Aradu.50ZYK	-0.05889369	-0.68965988	-0.76121314	0.757023247	0.722466024
Aradu.E9AVR	3.428946345	3.451540833	3.642701572	2.869871406	3.326249701
Aradu.629Q2	1.214124805	1.480265122	1.269033146	1.739848103	2.427606173
Aradu.XT2G5	-0.13606155	0.163498732	-0.20091269	0.910732662	0.454175893
Aradu.V05DH	0.799087306	0.713695815	0.879705766	1.599317794	2.070389328
Aradu.7AZ8Z	3.032100843	2.974529312	2.695993813	3.822730148	3.999098034
Aradu.V83D	1.269033146	1.042644337	0.5360529	1.3950628	1.687060688

R					
Aradu.MP2U2	1.3950628	1.963474124	1.790772038	3.102658131	3.379898164
Aradu.L3I19	1.718087584	2.117695043	2.153805336	3.03562391	2.873813198
Aradu.V1H1X	2.375734539	3.572889668	2.629939409	2.513490746	2.498250868
Aradu.D0URU	2.587364991	3.305970521	3.005399988	3.46727948	3.485426827
Aradu.QAC2D	0.250961574	0.773996325	0.516015147	1.298658316	1.545968369
Aradu.903PW	1.13093087	1.500802053	0.910732662	3.181102551	3.762348816
Aradu.S9NLD	-1.43440282	-1.55639335	-1.51457317	1.327687364	1.232660757
Aradu.ZH4KD	-0.15200309	0.50589093	0.028569152	1.570462931	1.575312331
Aradu.ZU8HW	-0.37706965	0.575312331	0.575312331	0.286881148	0.748461233
Aradu.X8I5Q	NA	NA	NA	-2.18442457	-2.05889369
Aradu.DD5QZ	2.61117238	2.488000771	2.44625623	0.695993813	0.98550043
Aradu.870BG	2.176322773	2.456806149	2.195347598	2.849999259	3.552131108
Aradu.X689P	-0.66657627	-0.23446525	-1.05889369	1.049630768	1.333423734
Aradu.I940M	NA	-5.64385619	NA	2.718087584	2.853995647
Aradu.Z07JX	2.310340121	3.859969548	4.426264755	7.852747965	7.093919284
Aradu.H9EKZ	0.526068812	0.545968369	0.124328135	-0.16812276	-1.08926734
Aradu.JWM4N	3.099295204	4.176322773	3.893362211	3.890446693	3.945794957
Aradu.8J00F	NA	-5.05889369	-5.05889369	-2.73696559	-1.02914635
Aradu.68E2R	2.5360529	2.996388746	2.545968369	3.659924558	3.524815928
Aradu.3IY7B	1.480265122	1.835924074	1.480265122	2.548436625	2.867896464
Aradu.NJ1GS	0.50589093	0.918386234	0.695993813	1.310340121	2.010779839
Aradu.E6N9	0.831877241	1.292781749	1.416839742	2.12763328	2.286881148

S					
Aradu.G44IN	-0.88896869	-0.20091269	-1.68965988	-1.08926734	4.61176256
Aradu.Q4VIY	-1.88896869	-0.78587519	-1.88896869	0.464668267	0.82374936
Aradu.SF38I	-1.21759144	-1.55639335	-1.83650127	-1.43440282	-1.51457317
Aradu.HZI51	-4.64385619	-6.64385619	NA	-4.64385619	-2.94341647
Aradu.509WJ	1.545968369	2.634593268	1.98550043	3.295723025	3.187451054
Aradu.Z84W9	1.555816155	1.744161096	1.575312331	1.726831217	1.948600847
Aradu.TZ9RJ	3.90014226	3.874796966	3.211012193	2.639232163	2.918386234
Aradu.9MF3N	5.19061486	5.123914459	5.365622556	2.414135533	2.021479727
Aradu.32A9T	2.989139007	3.310340121	3.171527106	3.440952198	3.925999419
Aradu.85GH9	2.025028794	2.684818738	2.347665656	3.240314329	3.407352751
Aradu.XP0GX	-1.25153877	-0.83650127	-1.28630419	1.014355293	0.941106311
Aradu.RS6VM	1.655351829	1.819668183	1.545968369	2.625270489	2.742006211
Aradu.W3ELN	-3.47393119	-2.05889369	-1.94341647	-1.15200309	-1.18442457
Aradu.EQ3HU	2.23572706	3.981852653	3.267535798	3.824767853	4.458775699
Aradu.752JK	3.489286023	3.439623138	3.240314329	2.883620816	3.142413438
Aradu.NB41M	NA	-5.05889369	-3.83650127	0.722466024	0.35614381
Aradu.PJM2P	1.819668183	2.042644337	1.879705766	2.189033824	2.189033824
Aradu.C7AZ0	-4.05889369	-6.64385619	-6.64385619	NA	-0.59946207
Aradu.AE6VJ	0.189033824	-1.18442457	-1.51457317	1.575312331	1.298658316
Aradu.YZ4F8	-1.55639335	-1.47393119	-1.94341647	-1.05889369	-0.97143085
Aradu.I92X3	4.339850003	4.459431619	4.452858965	1.63691458	1.304511042
Aradu.PF378	-0.83650127	-0.53533173	-1.28630419	-2.83650127	-2.05889369

Aradu.91Y21	1.372952098	1.59454855	1.286881148	1.321928095	1.275007047
Aradu.47ARL	-5.64385619	-5.64385619	-5.05889369	NA	-2.83650127
Aradu.38DG2	2.769771739	1.650764559	1.411426246	2.879705766	3.124328135
Aradu.VZ497	1.925999419	2.592158002	2.408711861	2.482848283	2.918386234
Aradu.61JFB	3.001802243	3.370164281	3.454175893	2.937344392	3.341985747
Aradu.MA01V	1.761285273	2.039138394	1.722466024	3.337711092	3.799087306
Aradu.E2TJL	0.545968369	1.070389328	0.650764559	2.042644337	2.150559677
Aradu.L1447	NA	NA	NA	NA	-6.64385619
Aradu.ER3NJ	1.604071324	2.709290636	2.754887502	1.66448284	2.867896464
Aradu.QN5ZJ	0.722466024	1.361768359	-0.76121314	4.357552005	5.122258568
Aradu.X4GW8	7.433794059	7.662846365	7.748930784	5.5055734	5.116447936
Aradu.4SA7Z	1.835924074	2.361768359	2.23878686	2.241840184	2.769771739
Aradu.QI7LX	-5.05889369	-4.32192809	-4.32192809	-2.83650127	-3.32192809
Aradu.D8TZD	2.933572638	3.135863165	3.077242999	3.955126781	3.874796966
Aradu.69820	3.800123353	3.618238656	3.577730931	0.555816155	0.669026766
Aradu.C924Y	3.529820947	3.731183242	3.518535139	2.776103988	2.794935663
Aradu.B151U	4.126807703	4.349082146	3.945794957	5.096767855	5.327328083
Aradu.X53KQ	-3.64385619	-2.39592868	-2.47393119	-2.55639335	-1.78587519
Aradu.5W8P5	0.028569152	0.333423734	0.124328135	0.526068812	0.422233001
Aradu.WQ4BK	1.765534746	2.028569152	1.641546029	2.375734539	2.914564523
Aradu.IJM7H	3.943921327	4.492494152	3.649615459	4.984133595	5.100557221
Aradu.VW9J	1.3950628	1.526068812	1.257010618	1.807354922	1.731183242

Z					
Aradu.JM0WC	5.09085343	5.755154643	4.763411574	6.803614528	6.722875823
Aradu.WXB2Z	-2.55639335	-4.32192809	-3.32192809	-5.64385619	-2.73696559
Aradu.270YY	7.012903833	6.577126661	6.575463612	3.386810946	3.439623138
Aradu.D0ZYM	4.412104045	4.239550797	4.207111961	3.919340082	4.567423758
Aradu.ZS4VI	3.148934105	3.7473874	3.060047384	1.575312331	1.327687364
Aradu.N0F3U	1.182692298	2.510961919	2.629939409	-0.35845397	0.042644337
Aradu.8IN6N	2.422233001	2.266036894	1.910732662	3.09423607	3.350497247
Aradu.D3M3F	-5.64385619	-4.32192809	-3.64385619	-1.73696559	-0.83650127
Aradu.GT2DG	2.780310099	3.196921734	2.275007047	3.969933275	3.766595162
Aradu.5P6B7	6.531225485	6.115823977	6.858229714	-0.78587519	-0.64385619
Aradu.0PI99	1.13093087	3.005399988	2.715893371	4.667892125	3.808385051
Aradu.E5KCW	3.326249701	4.145677455	3.691534165	5.04745114	5.220716892
Aradu.I1RXA	-5.05889369	-6.64385619	-5.05889369	-5.64385619	-5.05889369
Aradu.S166E	NA	-2.94341647	-4.32192809	-2.83650127	-1.88896869
Aradu.L1KP9	-0.64385619	-1.08926734	-1.39592868	-2.12029423	0.042644337
Aradu.5K7DH	NA	-6.64385619	NA	NA	NA
Aradu.Z6I4Q	4.821199978	3.999098034	4.19298317	3.614709844	3.49057013
Aradu.FW3IU	-0.73696559	-0.12029423	-1.15200309	2.682573297	3.112700133
Aradu.M70VI	NA	NA	NA	NA	NA
Aradu.7F3TN	NA	-4.64385619	-6.64385619	-5.05889369	5.073391816
Aradu.27T3V	0.042644337	0.799087306	0.773996325	1.214124805	1.485426827
Aradu.CP7C	-0.59946207	0.286881148	-0.62148838	0.704871964	0.739848103

1					
Aradu.M4GP4	3.177917792	4.275752049	3.629939409	3.518535139	3.528571319
Aradu.I2Z9Y	NA	-6.64385619	-5.64385619	NA	3.886550147
Aradu.BIW4R	0.97819563	2.247927513	1.084064265	2.983677695	3.13093087
Aradu.HAL7W	0.713695815	0.613531653	0.773996325	1.111031312	1.521050737
Aradu.G3X0F	3.185866545	3.392317423	3.435628594	3.370164281	3.605257263
Aradu.E6FD3	1.475084883	2.408711861	1.22650853	3.503348735	3.495695163
Aradu.RCT4K	2.622930351	2.748461233	2.475084883	3.211012193	2.523561956
Aradu.S056J	1.344828497	1.327687364	1.495695163	1.974529312	1.207892852
Aradu.65YXV	NA	NA	NA	NA	-2.32192809
Aradu.TU51C	0.35614381	1.042644337	0.150559677	2.077242999	2.424922088
Aradu.206DU	1.941106311	2.700439718	2.464668267	1.560714954	2.073820233
Aradu.PP2DC	2.424922088	2.627606838	2.543495883	3.289834465	3.592158002
Aradu.3VR2T	1.150559677	1.541019153	1.981852653	2.066950244	1.859969548
Aradu.L3K73	-2.47393119	-2.05889369	-2.12029423	-2.05889369	-1.43440282
Aradu.8I79H	4.707082992	4.255500733	4.726831217	4.239550797	4.244125943
Aradu.EZU9P	1.007195501	1.280956314	1.286881148	-1.47393119	-0.78587519
Aradu.VP5WD	-1.39592868	-1.05889369	-1.47393119	-0.64385619	-0.45403163
Aradu.U4XDJ	NA	-6.64385619	NA	-5.64385619	-5.05889369
Aradu.L5GQQ	3.889473543	3.604071324	3.464668267	3.323370069	4.09000653
Aradu.9E8FC	5.541638734	5.863442934	5.590661896	0.124328135	0.097610797
Aradu.AQ0Z8	NA	NA	NA	NA	NA
Aradu.R3AG	1.59454855	2.353323291	2.13422094	2.720278465	2.925999419

E					
Aradu.DES3N	-0.53533173	-0.23446525	-0.53533173	0.298658316	1.50589093
Aradu.Q8JL3	NA	-1.32192809	-6.64385619	NA	-4.64385619
Aradu.BFS1P	3.599317794	4.521050737	4.053111336	4.933100475	4.921245889
Aradu.DTW5Z	3.228049048	2.887525271	2.803227036	3.275007047	3.198494154
Aradu.JXB2I	4.22650853	4.949067966	3.87774425	5.958378712	5.732811872
Aradu.5G7S4	1.14404637	1.275007047	0.765534746	2.927896454	2.675815931
Aradu.DP2D5	0.310340121	0.739848103	0.5360529	4.319039816	4.587964989
Aradu.B98C6	0.650764559	0.765534746	0.310340121	3.629939409	3.572889668
Aradu.1WV86	NA	NA	NA	NA	NA
Aradu.Y0VZV	0.622930351	1.516015147	1.03562391	0.526068812	0.014355293
Aradu.3TJ8Z	3.415488271	3.605257263	3.295723025	3.397802962	3.577730931
Aradu.CG9KF	0.970853654	1.257010618	1.077242999	2.257010618	2.634593268
Aradu.6Z59M	0.992768431	1.03562391	0.933572638	1.448900951	2
Aradu.CT0FI	-2	-1.88896869	-2.18442457	0.632268215	0.948600847
Aradu.ES74T	2.339137385	2.992768431	2.809414444	2.195347598	2.3950628
Aradu.SP3D0	1.182692298	1.49057013	0.925999419	2.353323291	2.451540833
Aradu.Q0T0K	1.799087306	2.232660757	1.970853654	2.3950628	2.526068812
Aradu.IR41I	-1.28630419	-0.59946207	-0.68965988	-0.71311885	-0.34007544
Aradu.DR03T	-0.39592868	-0.59946207	-0.47393119	0.704871964	1.021479727
Aradu.4206R	0.659924558	0.86393845	0.687060688	0.495695163	2.40599236
Aradu.HNV5C	-2.39592868	-2.18442457	-2.39592868	-1.39592868	-0.37706965
Aradu.5I03J	NA	-6.64385619	NA	2.10433666	2.857980995

Aradu.DJ2MU	3.399171094	2.976363636	2.869871406	1.042644337	1.007195501
Aradu.N1Q89	NA	NA	NA	NA	NA
Aradu.D31R9	-0.15200309	0	-0.20091269	1.042644337	1.765534746
Aradu.3Q3ML	2.298658316	2.572889668	2.469885976	2.726831217	3.378511623
Aradu.Y7357	2.422233001	2.646162657	2.381283373	2.35614381	2.493134922
Aradu.B7RDX	NA	-2.64385619	-2.64385619	1.389566812	-0.55639335
Aradu.64PSN	3.021479727	3.263034406	3.0721058	2.790772038	3.066950244
Aradu.W88FU	0.879705766	1.124328135	0.604071324	2.386810946	2.321928095
Aradu.NZ8CP	-0.53533173	0.82374936	0.641546029	0.687060688	1.448900951
Aradu.DWL7L	NA	NA	-6.64385619	-3.83650127	-4.05889369
Aradu.A252K	2.263034406	2.757023247	2.358958826	3.010779839	3.247927513
Aradu.NZ4WV	3.053111336	3.264536431	3.353323291	4.031218731	4.250203656
Aradu.P74XB	6.922197848	6.78633498	6.816343705	3.793895883	3.935459748
Aradu.D3LC1	-1	-1.12029423	-1	-0.4150375	-0.30400619
Aradu.HFI9K	5.402244655	5.328046556	5.672990994	5.071247819	5.630230716
Aradu.BM68M	3.023255352	3.559491813	3.580145484	4.203201156	4.215678597
Aradu.2B9FT	3.693765712	4.284662185	4.123500664	3.570462931	3.349082146
Aradu.DM4CY	4.579542225	5.240314329	5.081510068	6.522463835	6.525285887
Aradu.QV0NG	4.868884273	5.374691752	5.13996057	4.642123916	5.020146573
Aradu.0JT6M	3.272023189	2.752748591	2.66448284	1.86393845	2.440952198
Aradu.V7ZTF	4.386810946	4.267535798	4.067810784	2.257010618	3.189033824
Aradu.GB4V	1.86393845	4.965784285	2.526068812	4.420886575	4.344118335

7					
Aradu.VA4EQ	0.5360529	0.632268215	0.35614381	0.495695163	0.443606651
Aradu.GH6V7	NA	-3.64385619	NA	-3.83650127	-3.18442457
Aradu.0NC5M	-5.64385619	-4.05889369	-4.32192809	NA	-3.64385619
Aradu.DJW6E	-2.25153877	-2.25153877	-2.47393119	0.028569152	0.084064265
Aradu.A68MB	1.847996907	2.472487771	2.327687364	3.390942773	3.596935142
Aradu.TVQ04	2.493134922	2.353323291	2.443606651	1.929790998	1.918386234
Aradu.UX9CD	NA	NA	NA	-2.73696559	-3.83650127
Aradu.K9316	NA	NA	NA	NA	NA
Aradu.Z2TGH	-3.05889369	-1.64385619	-1.59946207	-1.88896869	-1.39592868
Aradu.QJD0U	-3.83650127	-2.83650127	-3.47393119	-0.30400619	0.22650853
Aradu.752ZV	NA	NA	NA	NA	-5.64385619
Aradu.V8HSY	4.498889087	4.248686627	3.878725341	3.968090752	4.175524601
Aradu.CP61L	1.769771739	2.169925001	2.100977648	3.014355293	3.150559677
Aradu.M2EU8	-2.05889369	-2.73696559	-2.94341647	NA	1.286881148
Aradu.T1JCL	-0.23446525	0.545968369	0.189033824	0.475084883	0.963474124
Aradu.970W1	1.280956314	0.298658316	0.40053793	-3.05889369	-0.4150375
Aradu.BBW0R	NA	NA	NA	NA	NA
Aradu.5PD1B	0.23878686	0.782408565	0.5360529	2.124328135	2.44625623
Aradu.E9CVQ	-4.32192809	-3.47393119	-3.18442457	-2.25153877	-0.28630419
Aradu.PV43B	NA	-6.64385619	NA	NA	NA
Aradu.YPS83	-2.94341647	-1.68965988	-2.55639335	-1.21759144	-0.78587519
Aradu.XDS84	NA	NA	NA	-2.55639335	-0.62148838

Aradu.UD253	0.516015147	0.214124805	-1.12029423	-0.43440282	0.201633861
Aradu.44R1C	-4.05889369	-3.83650127	-5.05889369	-1.25153877	0.150559677
Aradu.12WE9	NA	NA	NA	NA	NA
Aradu.66MNC	-2.94341647	-1.12029423	-1.88896869	0.757023247	0.871843649
Aradu.28KIR	2.250961574	2.599317794	2.97819563	1.049630768	1.384049807
Aradu.M970R	4.597531174	4.593951284	4.063502942	2.873813198	4.14974712
Aradu.QAZ4U	-1.47393119	-2.05889369	-1.18442457	-0.91593574	3.777156666
Aradu.CX05K	NA	-6.64385619	NA	-3.47393119	-3.32192809
Aradu.W7CVG	-1.39592868	-0.81096618	-0.37706965	3.028569152	3.070389328
Aradu.DAN0Y	1.695993813	1.339137385	1.056583528	1.03562391	1.063502942
Aradu.Q9UFT	-5.64385619	-4.32192809	-3.18442457	-2.39592868	-1.21759144
Aradu.LF5EZ	-5.64385619	NA	NA	NA	NA
Aradu.D4D3B	NA	NA	NA	-3.05889369	0.475084883
Aradu.L0GAW	-1.55639335	-1.64385619	-2.55639335	-0.35845397	0.137503524
Aradu.ZX8II	3.23878686	3.805292456	3.518535139	2.702657543	3.764473551
Aradu.X258N	2.861955364	3.140778656	2.922197848	4.627023106	4.364572432
Aradu.SLM5J	1.560714954	2.275007047	2.163498732	2.817623258	3.384049807
Aradu.MB6LX	NA	NA	NA	NA	NA
Aradu.003TN	3.454175893	3.697106574	3.320484678	5.259649121	5.360013042
Aradu.M8B8F	2.634593268	3.522306893	3.0721058	1.545968369	2.724650272
Aradu.YN1Y7	0.097610797	0.40053793	0.298658316	4.587364991	3.883620816
Aradu.P535S	0.748461233	1.422233001	0.879705766	3.534808661	3.731183242

Aradu.C7CT2	2.5360529	1.448900951	2.748461233	-1.32192809	-0.47393119
Aradu.2Q562	3.725741157	3.849999259	3.752748591	3.447579197	3.438292852
Aradu.ZZ3JW	-4.05889369	-3.64385619	-3.83650127	2.310340121	2.389566812
Aradu.UK481	2.994579724	3.277984747	3.27351589	2.09085343	3.71259578
Aradu.63D6A	0.014355293	-0.12029423	-0.32192809	1.464668267	2.641546029
Aradu.10XKC	2.056583528	2.370164281	2.032100843	2.12763328	2.538538164
Aradu.VD3WH	1.350497247	1.691534165	1.709290636	2.263034406	2.575312331
Aradu.RA8CW	0.678071905	0.98550043	0.704871964	2.702657543	2.500802053
Aradu.Q97KS	-1.12029423	1.5360529	0.757023247	3.658782734	3.731183242
Aradu.SQK9K	3.941106311	4.750070486	4.663913842	3.19061486	3.298658316
Aradu.J486B	-1.78587519	-0.83650127	-5.64385619	0.263034406	2.307428525
Aradu.54E1H	0.799087306	1.22650853	2.214124805	0.722466024	0.765534746
Aradu.ED8I5	NA	-5.64385619	NA	-5.64385619	-3.47393119
Aradu.GN6RI	1.748461233	2.053111336	1.910732662	3.047887329	3.327687364
Aradu.P4KG5	1.432959407	2.185866545	1.310340121	2.803227036	3.192194165
Aradu.NP275	1.580145484	1.847996907	1.438292852	2.137503524	2.321928095
Aradu.Z08PN	NA	-6.64385619	NA	NA	NA
Aradu.8LY9S	0.070389328	0.632268215	-1.18442457	2.771885579	3.23878686
Aradu.73DHW	0.731183242	1.422233001	1.207892852	1.49057013	1.970853654
Aradu.SB62Q	1.922197848	2.327687364	1.704871964	2.853995647	3.116031993
Aradu.DYP0A	-3.64385619	-1.59946207	-1.55639335	-0.51457317	0.056583528
Aradu.EJ72Y	1.454175893	1.454175893	1.150559677	1.765534746	2.12763328

Aradu.VG4AF	NA	NA	-6.64385619	NA	2.983677695
Aradu.ZHM8Y	2.140778656	2.176322773	1.948600847	1.831877241	2.459431619
Aradu.1H2UR	-1.21759144	-1.43440282	-0.76121314	1.014355293	1.875780063
Aradu.R8LP2	4.464014725	4.742545234	4.474436043	5.98208091	5.840211433
Aradu.R2W4M	-3.05889369	-2.18442457	-3.64385619	-1.47393119	-1.12029423
Aradu.ND275	3.300123725	4.414812061	3.177917792	5.704595348	5.58044702
Aradu.4HV8T	2.472487771	2.570462931	2.698218478	3.408711861	3.739848103
Aradu.9UB80	-5.64385619	-4.64385619	-4.64385619	-5.64385619	-4.32192809
Aradu.Z4AN4	2.641546029	2.767654798	2.341985747	3.297191417	3.100977648
Aradu.88HY6	-4.64385619	-3.18442457	-3.83650127	-1.32192809	-1.51457317
Aradu.10I3J	NA	-5.64385619	NA	NA	-5.64385619
Aradu.N79JZ	1.427606173	1.773996325	1.475084883	2.464668267	2.545968369
Aradu.B73B3	2.111031312	2.508428653	1.803227036	2.752748591	2.933572638
Aradu.GTZ9I	0.90303827	1.599317794	1.250961574	1.464668267	1.952333566
Aradu.EG2K3	3.772941338	3.699329526	3.7322692	4.14974712	5.123086751
Aradu.B7P36	4.859472667	4.605257263	4.467931546	1.584962501	1.550900665
Aradu.T55NY	-0.23446525	0.333423734	-0.32192809	0.713695815	1.286881148
Aradu.HR4SC	5.145269857	6.371210343	5.819923596	7.117902789	6.850874416
Aradu.X6P8N	NA	NA	NA	NA	NA
Aradu.TV5GQ	0.443606651	0.956056652	0.367371066	1.526068812	1.526068812
Aradu.175L3	0.963474124	0.650764559	-0.53533173	3.033863452	2.604071324
Aradu.XBR45	3.936402378	3.981852653	4.012568674	-4.32192809	-2.12029423

Aradu.FN25A	5.946262984	5.890446693	6.025914699	2.032100843	2.063502942
Aradu.R23AB	-0.73696559	-0.23446525	0	-0.16812276	-0.55639335
Aradu.IVE8F	-6.64385619	-5.64385619	-6.64385619	-1.51457317	-0.71311885
Aradu.JN9TL	1.531069493	1.933572638	1.464668267	2.324810603	2.641546029
Aradu.MA612	1.613531653	2.381283373	1.90303827	2.480265122	3.019701914
Aradu.7N01K	3.250961574	3.798050515	2.885574364	4.178714641	4.28169825
Aradu.70NQH	1.871843649	1.339137385	1.304511042	3.155425432	2.843983844
Aradu.A6HVN	0.070389328	0.516015147	0.454175893	1.056583528	1.575312331
Aradu.H49AM	-1.02914635	-0.43440282	-1.51457317	2.260025656	2.053111336
Aradu.M0XAT	0.014355293	0.111031312	0.070389328	0.748461233	0.632268215
Aradu.R1E3C	5.408032466	5.218393711	5.206721357	4.271276259	4.46008724
Aradu.5Q6ZX	6.044831233	6.089794727	5.791814071	3.267535798	3.080657663
Aradu.9QT6G	1	1.292781749	0.970853654	1.084064265	2.241840184
Aradu.79JEZ	1.731183242	2.232660757	2.017921908	2.622930351	2.965322548
Aradu.P39C1	-4.64385619	-0.23446525	-2.39592868	-1.73696559	-1.68965988
Aradu.GSM33	2.959770155	3.15704371	3.010779839	1.918386234	2.169925001
Aradu.08LLU	1.117695043	1.970853654	1.500802053	2.195347598	2.443606651
Aradu.1I5W4	1.98550043	2.014355293	1.859969548	2.414135533	2.632268215
Aradu.P7XEX	3.181102551	4.263034406	3.963474124	3.454175893	3.601696516
Aradu.UMN0V	NA	NA	NA	NA	-4.64385619
Aradu.6C2JR	2.526068812	2.592158002	2.438292852	3.166715445	3.527320608
Aradu.D4FDN	0.731183242	0.443606651	0.464668267	0.344828497	0.871843649

Aradu.D5Z0P	2.713695815	2.601696516	2.788685711	1.773996325	1.03562391
Aradu.WW0P3	-1.39592868	-1.25153877	-1.39592868	-0.35845397	0.028569152
Aradu.1NK9R	2.807354922	2.13422094	2.160274831	0.367371066	2.007195501
Aradu.JFH3E	2.269033146	2.56315813	2.22342255	1.879705766	1.929790998
Aradu.IM9AT	4.195347598	4.977279923	4.645586386	2.994579724	3.364572432
Aradu.0U1AX	NA	NA	NA	-3.18442457	-1.78587519
Aradu.VJ006	-2.83650127	-5.05889369	-5.05889369	-1.39592868	-1.94341647
Aradu.R42GY	2.825785627	2.843983844	2.319039816	2.659924558	3.099295204
Aradu.M8UTW	2.648465443	2.528571319	1.704871964	0.879705766	1.827819025
Aradu.U55D3	2.782408565	3.109360559	2.825785627	3.928844037	4.005399988
Aradu.6PP4L	0.263034406	0.432959407	0.124328135	0.773996325	1.495695163
Aradu.YV9QI	3.23878686	3.723558561	3.527320608	0.773996325	1.275007047
Aradu.LW8Y2	0.367371066	0.678071905	0.411426246	1.464668267	1.937344392
Aradu.9DS5H	1.925999419	2.241840184	2.10433666	2.963474124	3.276496666
Aradu.GGF3N	1.269033146	2.107687869	1.510961919	2.987320866	3.237257771
Aradu.45I6B	-2.73696559	-1.73696559	-3.32192809	-2.39592868	-0.91593574
Aradu.ED9QM	0.070389328	0.275007047	-0.18442457	0.739848103	1.427606173
Aradu.939VA	-4.05889369	-4.64385619	-4.05889369	-5.64385619	-6.64385619
Aradu.IW3DF	-5.64385619	-5.64385619	-4.64385619	-5.64385619	-4.05889369
Aradu.YV0Y1	-3.47393119	-2.18442457	-2.25153877	0.176322773	0.286881148
Aradu.RV9GW	-4.32192809	-3.83650127	-4.64385619	-2.47393119	0.124328135
Aradu.E01TZ	-1.32192809	1.286881148	-0.20091269	-1.05889369	-1.21759144

Aradu.3C582	2.350497247	2.709290636	2.392317423	3.165107985	3.252476214
Aradu.4S1TT	-0.43440282	-0.05889369	-0.16812276	1.169925001	1.163498732
Aradu.A8KM2	-4.05889369	-1.88896869	-3.18442457	-0.26881676	-0.43440282
Aradu.WM4V4	1.286881148	1.03562391	0.847996907	-3.64385619	-3.32192809
Aradu.Z2UHU	0.584962501	0.678071905	0.422233001	2.14404637	2.353323291
Aradu.31PM4	0.695993813	0.847996907	0.669026766	1.632268215	2.241840184
Aradu.EZ6B1	1.765534746	1.056583528	0.98550043	0.731183242	0.925999419
Aradu.ZY1BM	1.384049807	1.948600847	1.718087584	2.72900887	3.333423734
Aradu.QS8M2	1.871843649	2.367371066	1.925999419	3.553360503	4.040015679
Aradu.A0DL1	4.726831217	4.412104045	4.597531174	2.182692298	2.375734539
Aradu.BY27U	1.438292852	1.739848103	1.182692298	1.286881148	1.819668183
Aradu.1IC89	1.201633861	1.63691458	1.378511623	2.039138394	2.358958826
Aradu.ND5YL	4.758622982	4.939226578	5.007644031	1.819668183	1.516015147
Aradu.PK283	1.480265122	3.252476214	3.454175893	3.825785627	4.070389328
Aradu.3KI43	0.5360529	1.97819563	1.646162657	0.933572638	0.150559677
Aradu.FL0YZ	2.440952198	2.189033824	2.49057013	0.687060688	0.056583528
Aradu.XM1M6	-0.4150375	0.687060688	-0.02914635	1.641546029	2.23878686
Aradu.632MM	NA	-6.64385619	-5.64385619	-5.05889369	-4.64385619
Aradu.50C9M	5.294620749	5.902556005	5.480911346	5.744699314	5.5987225
Aradu.86SPU	2.761285273	3.003602237	2.767654798	1.709290636	1.941106311
Aradu.898CQ	2.372952098	2.925999419	2.601696516	2.618238656	2.893362211
Aradu.B0TIL	4.161081482	3.94673086	3.843983844	3.592158002	3.82984956

Aradu.ZF8NC	-0.16812276	0.014355293	-0.18442457	1.475084883	1.298658316
Aradu.SC3I7	NA	NA	NA	NA	NA
Aradu.G8GYX	4.168321116	4.689299161	3.838951767	5.344473459	5.241840184
Aradu.5M2IF	4.614709844	5.185866545	4.102658131	6.678071905	6.344118335
Aradu.CST5R	2.03562391	2.160274831	2.028569152	2.10433666	2.550900665
Aradu.47JD2	1.117695043	1.427606173	1.372952098	2.722466024	3.408711861
Aradu.RW91L	1.232660757	2.211012193	2.049630768	0.918386234	1.182692298
Aradu.P2MN3	-0.47393119	-0.4150375	0.028569152	1.432959407	2.217230716
Aradu.CD8IH	1.967168608	2.010779839	1.989139007	1.275007047	2.341985747
Aradu.FW5R5	1.575312331	2.269033146	2.107687869	2.700439718	2.538538164
Aradu.51556	3.942983598	4.219555769	3.482848283	4.245647774	3.893362211
Aradu.E4PH8	3.051372102	3.934516502	4.032100843	1.819668183	2.195347598
Aradu.5K7BE	-2.47393119	-0.30400619	-0.76121314	0.40053793	1.650764559
Aradu.56X7I	-3.32192809	-2.64385619	-2.94341647	0.214124805	0.22650853
Aradu.475M7	0.807354922	1.344828497	1.286881148	1.641546029	1.803227036
Aradu.YSR48	NA	-5.05889369	-4.64385619	-6.64385619	0.214124805
Aradu.FA8DG	NA	NA	NA	NA	-5.05889369
Aradu.2U2Q6	3.746312766	4.397802962	3.54225805	5.043519494	5.161484639
Aradu.Y178G	-6.64385619	NA	NA	-5.05889369	-3.32192809
Aradu.TET8U	NA	NA	NA	NA	-4.64385619
Aradu.L6FF7	1.475084883	1.622930351	1.5360529	2.173127433	2.280956314
Aradu.81XX0	NA	NA	NA	NA	NA

Aradu.QCH6Q	0.263034406	1.316145742	0.871843649	0.411426246	1.15704371
Aradu.0V2BM	NA	NA	NA	NA	NA
Aradu.44VUH	1.555816155	1.97819563	1.438292852	2.150559677	2.531069493
Aradu.DB65S	-0.91593574	-0.62148838	-1.59946207	0.310340121	0.678071905
Aradu.H9N2F	NA	NA	NA	NA	NA
Aradu.D5VFR	NA	NA	NA	NA	-5.64385619
Aradu.P2MZP	NA	NA	NA	-3.64385619	NA
Aradu.ZYE2B	-6.64385619	-4.64385619	-4.64385619	NA	1.443606651
Aradu.QU5N8	-0.68965988	0.432959407	0.042644337	2.718087584	2.378511623
Aradu.WLX6I	-2.32192809	-2.64385619	-2.55639335	0.056583528	-0.45403163
Aradu.NSL0R	2.724650272	2.720278465	2.849999259	0.887525271	1.807354922
Aradu.M3YXV	0	0.871843649	-0.18442457	2.140778656	2.097610797
Aradu.G1Z6G	1.263034406	1.739848103	1.117695043	1.580145484	1.996388746
Aradu.VB0PQ	0.097610797	0.056583528	-0.07400058	1.765534746	1.726831217
Aradu.L49GZ	0.918386234	1.541019153	1.49057013	2.488000771	1.731183242
Aradu.N906W	4.259272487	4.346956889	4.3950628	2.851998837	2.448900951
Aradu.7065G	-4.05889369	-0.35845397	-0.51457317	0.90303827	0.790772038
Aradu.97PVW	-1.64385619	-1.18442457	-0.94341647	-2.25153877	-2.12029423
Aradu.MF9WN	-4.64385619	-5.05889369	-6.64385619	-4.32192809	-2.39592868
Aradu.PLM7U	1.604071324	2.451540833	1.815575429	3.772941338	3.507160349
Aradu.J6P55	2.250961574	2.375734539	2.121015401	3.526068812	3.832890014
Aradu.F0W17	2.140778656	2.477677328	1.803227036	1.650764559	1.40599236

Aradu.HX9PW	0.59454855	1.220329955	0.970853654	2.548436625	2.40599236
Aradu.HC4GG	2.601696516	2.392317423	2.13422094	2.648465443	2.313245852
Aradu.91M2N	3.63343121	4.006298024	3.670160514	4.445594291	4.675815931
Aradu.UD5NS	0.485426827	1.545968369	1.599317794	0.526068812	1.361768359
Aradu.NIR19	2.23878686	3.303050085	4.188242656	2.367371066	2.283921772
Aradu.9RZ6U	3.773996325	3.6622055	3.502075956	2.750606505	3.17951105
Aradu.FUM3Y	1.580145484	2.879705766	2.482848283	1.195347598	1.550900665
Aradu.T46DH	-2	-2.94341647	-2.12029423	-3.05889369	-2.47393119
Aradu.X49FH	-1.59946207	-2.39592868	-2.05889369	-0.4150375	-2.12029423
Aradu.JBH10	3.578938713	3.613531653	3.379898164	4.211790976	4.529820947
Aradu.KWP32	3.286881148	3.683696454	3.509695842	4.025914699	4.175524601
Aradu.U4S49	1.700439718	2.017921908	1.827819025	2.961623328	3.427606173
Aradu.6T5AB	-0.01449957	0.378511623	-0.37706965	0.584962501	1.111031312
Aradu.K0FA0	0.86393845	1.280956314	1.070389328	1.007195501	1.378511623
Aradu.AK4LW	-5.05889369	-5.64385619	-6.64385619	-3.32192809	-3.05889369
Aradu.D12DR	NA	NA	-5.64385619	NA	-5.64385619
Aradu.50IFA	1.077242999	0.839959587	0.59454855	-0.86249648	0.344828497
Aradu.MIQ5P	-5.05889369	-6.64385619	NA	NA	-5.05889369
Aradu.YVU9U	4.247927513	4.192194165	4.047887329	3.23572706	3.243364426
Aradu.Z0DJ4	4.840463234	4.712045449	4.865918815	4.622930351	5.007644031
Aradu.KR6D1	5.032541697	5.156639311	4.914564523	6.324090516	6.335032987
Aradu.RDM6K	-0.23446525	1.464668267	0.687060688	0.23878686	0.475084883

Aradu.6BH4S	1.195347598	1.250961574	1.201633861	1.769771739	2.641546029
Aradu.398CK	-6.64385619	-6.64385619	-6.64385619	-2	-2.73696559
Aradu.EG96G	-4.32192809	-5.64385619	-6.64385619	NA	-1.51457317
Aradu.A7GFW	4.266036894	4.509695842	4.473137488	5.395405605	5.161887682
Aradu.M69JC	0.367371066	0.887525271	1.220329955	5.533563348	3.724650272
Aradu.F2D3W	NA	NA	NA	NA	NA
Aradu.QZS0L	-0.64385619	0.056583528	-0.23446525	1.275007047	1.704871964
Aradu.Q0GRU	3.385431037	3.304511042	3.427606173	2.247927513	2
Aradu.L7RAQ	2.372952098	2.010779839	1.669026766	2.017921908	2.40053793
Aradu.NI7KT	-5.64385619	-5.64385619	-6.64385619	-1.88896869	-0.577767
Aradu.49CW6	-0.81096618	0.028569152	-0.71311885	1	1.163498732
Aradu.S3CLD	-3.64385619	-2.32192809	-3.47393119	-3.05889369	-1.78587519
Aradu.2A8ZQ	-5.05889369	-5.64385619	NA	-6.64385619	-5.64385619
Aradu.9YK5E	1.480265122	1.545968369	1.438292852	4.344828497	3.885574364
Aradu.3C2YM	-0.25153877	-0.08926734	-1.21759144	0.137503524	-0.18442457
Aradu.F7BLG	2.82374936	2.397802962	2.22342255	2.350497247	2.587364991
Aradu.22C3R	1.176322773	1.895302621	1.765534746	2.301587647	2.150559677
Aradu.3V0DC	NA	NA	-5.64385619	-3.05889369	-1.47393119
Aradu.A77EC	1.839959587	2.675815931	1.859969548	3.484138131	3.749534268
Aradu.WI4BU	0.443606651	0.422233001	0.695993813	0.163498732	-0.30400619
Aradu.09HBR	10.29197645	9.894726834	10.36502102	6.207502459	6.34553831
Aradu.AE9WN	NA	NA	NA	NA	-3.47393119

Aradu.VWQ48	NA	-5.05889369	-5.05889369	-3.32192809	-1.18442457
Aradu.YXX50	-0.47393119	0.422233001	0.298658316	1.49057013	1.304511042
Aradu.07167	NA	-5.64385619	-6.64385619	NA	-2.25153877
Aradu.2Y4H2	0.632268215	0.678071905	0.286881148	0.659924558	0.765534746
Aradu.2A606	0.86393845	0.50589093	1.207892852	NA	-3.83650127
Aradu.77XDI	4.040892431	3.43162296	3.629939409	2.657640005	2.485426827
Aradu.EX4VM	-5.64385619	-3.18442457	-1.68965988	-2.94341647	-0.20091269
Aradu.DW9IA	0	1.244887059	1	-1.05889369	-0.32192809
Aradu.8W59U	NA	NA	NA	NA	-4.05889369
Aradu.FS7LG	-5.64385619	-4.32192809	-5.64385619	-1.51457317	3.211012193
Aradu.FD81T	NA	NA	NA	NA	NA
Aradu.8M02Z	NA	NA	NA	-1.68965988	1.13093087
Aradu.77WYF	-4.32192809	-4.05889369	-3.83650127	-0.62148838	-0.53533173
Aradu.WAT9N	0.678071905	1.316145742	1.124328135	1.15704371	0.847996907
Aradu.GH35A	1	1.333423734	0.565597176	2.925999419	4.736063628
Aradu.8F945	-5.05889369	-6.64385619	-4.05889369	-5.64385619	NA
Aradu.WJ2TG	4.156234798	4.294253136	3.956056652	4.386121157	4.40053793
Aradu.RA95M	NA	NA	NA	NA	NA
Aradu.8ND9A	4.263034406	4.136683578	3.806324057	2.657640005	3.051372102
Aradu.MLQ47	0.970853654	0.918386234	0.632268215	1.695993813	1.811471031
Aradu.EZ25P	1.700439718	2.280956314	1.981852653	3.121015401	3.57167681
Aradu.68YSI	3.773996325	4.236492618	3.800123353	3.828834649	3.828834649

Aradu.J7XF6	-3.83650127	-2	-2.18442457	-3.32192809	-4.32192809
Aradu.E2YZD	5.765534746	5.700717133	5.113117036	5.963242904	5.541019153
Aradu.WT391	1.310340121	1.650764559	1.321928095	2.392317423	3.100977648
Aradu.SHH9J	2.849999259	2.843983844	2.72900887	0.879705766	2.066950244
Aradu.X114S	2.451540833	2.364572432	2.485426827	1.021479727	0.871843649
Aradu.35NHM	2.124328135	2.784503983	2.207892852	1.959770155	1.918386234
Aradu.GKG4B	-0.04394335	0.389566812	0.014355293	0.739848103	0.622930351
Aradu.35Z4P	6.539624122	3.982765463	3.687060688	5.368768349	5.851998837
Aradu.44QYZ	-1.83650127	-0.88896869	-1	0.722466024	0.847996907
Aradu.4IN7K	-0.94341647	0.310340121	0.070389328	-2.47393119	-0.64385619
Aradu.C674J	4.356848079	5.036942845	4.538538164	4.435628594	4.765534746
Aradu.S4V52	10.31600994	10.09862431	10.53256048	6.59559317	6.674827832
Aradu.K45PE	2.956056652	3.218781168	2.901108243	2.364572432	2.518535139
Aradu.3EX3F	2.15704371	2.733354341	2.397802962	2.64385619	2.797012978
Aradu.251QA	-2.73696559	-2.94341647	-3.32192809	-3.18442457	-0.18442457
Aradu.GP04Q	0.695993813	1.584962501	1.220329955	3.74092756	2.767654798
Aradu.KYQ8M	0.799087306	1	0.831877241	1.613531653	1.974529312
Aradu.D3HZI	2.09423607	2.416839742	2.176322773	2.397802962	3.054848477
Aradu.EJ00N	-3.05889369	-3.05889369	-2.94341647	-2	2.260025656
Aradu.S111G	0.925999419	1.49057013	1.269033146	2.805292456	2.925999419
Aradu.ZF31Q	1.906890596	2.17951105	2.232660757	2.759155834	2.82984956
Aradu.X0CJD	0.495695163	0.97819563	0.956056652	1.510961919	1.996388746

Aradu.C7NWK	2.625270489	2.759155834	2.543495883	4.679198571	4.573495715
Aradu.7K62W	NA	-5.05889369	NA	NA	-3.64385619
Aradu.PQ5HC	2.263034406	2.950468414	2.879705766	4.491853096	4.599912842
Aradu.N60NI	2.087462841	2.23572706	1.937344392	1.831877241	1.929790998
Aradu.CDI4M	0	-0.86249648	-0.71311885	-1.35845397	-2.32192809
Aradu.B5E0M	3.025028794	3.119356177	2.89917563	3.27351589	3.544732656
Aradu.V9VEN	-0.64385619	1.257010618	1.367371066	-0.76121314	0.432959407
Aradu.D4TC3	NA	NA	NA	NA	NA
Aradu.UV1C8	-1.88896869	-1.15200309	-1.32192809	-0.577767	0.948600847
Aradu.H8AL3	5.176322773	5.29130886	4.910732662	3.371558863	3.261530815
Aradu.L9ISE	-2.73696559	-3.18442457	-2.83650127	0.201633861	0.070389328
Aradu.FMP57	2.803227036	2.555816155	2.500802053	3.564378169	3.722466024
Aradu.S6EUF	2.121015401	2.22342255	2.13093087	3.364572432	3.3950628
Aradu.X9447	6.354557947	6.969472865	6.887037791	3.605257263	3.748461233
Aradu.0M3HI	-0.81096618	-1.12029423	-1.39592868	-0.49410907	0.879705766
Aradu.EKZ3T	-5.05889369	-5.64385619	-6.64385619	-3.64385619	-3.64385619
Aradu.YAX06	-1.43440282	-1.51457317	-1.35845397	1.427606173	0.941106311
Aradu.10YFM	1.316145742	2.277984747	1.952333566	1.843983844	2.229587923
Aradu.VRG8M	2.618238656	3.275007047	3.112700133	-0.05889369	1.361768359
Aradu.A8KIV	1.531069493	2.257010618	1.722466024	2.935459748	3.026800059
Aradu.2CJ0B	1.575312331	1.339137385	1.007195501	0.933572638	1.59454855
Aradu.CD4CM	-0.78587519	-1.39592868	-1.78587519	1.469885976	3.250961574

Aradu.VA9CU	NA	NA	NA	NA	NA
Aradu.8KB8X	NA	NA	NA	NA	NA
Aradu.92KA6	3.950468414	4.416839742	4.150559677	5.053545818	4.98458935
Aradu.EN58B	1.182692298	1.097610797	1.244887059	0.124328135	-0.08926734
Aradu.I03VB	NA	-5.64385619	NA	NA	NA
Aradu.N725Y	1.378511623	1.851998837	1.691534165	2.931683057	2.948600847
Aradu.470B5	4.71918344	4.476381688	4.40053793	3.350497247	3.94673086
Aradu.NRF9P	NA	-5.64385619	NA	NA	-5.64385619
Aradu.9B6LA	5.253611152	5.489928219	5.13996057	5.661920579	5.968781974
Aradu.LQA0K	NA	NA	NA	NA	-5.05889369
Aradu.B353U	7.212763864	7.526460115	7.521443403	4.3305584	3.895302621
Aradu.JZ8MY	-0.21759144	0.575312331	1.189033824	1.189033824	2.295723025
Aradu.L2FW0	-0.13606155	-0.62148838	-0.88896869	3.331991778	2.053111336
Aradu.YY51Q	-3.47393119	-5.05889369	-6.64385619	-2.39592868	-2.18442457
Aradu.TSZ7Q	4.482202926	4.085764554	3.593353771	1.851998837	2.596935142
Aradu.B81RW	-4.32192809	-3.83650127	-3.83650127	-1.51457317	-2.18442457
Aradu.4DR9N	0.970853654	1.996388746	1.641546029	2.257010618	2.618238656
Aradu.65JMT	0.056583528	0.695993813	0.565597176	2.589763487	2.493134922
Aradu.W3DTP	4.116031993	4.915998852	3.820689561	6.265099288	6.129489102
Aradu.EA3WK	NA	NA	NA	NA	-5.05889369
Aradu.8FB72	0.731183242	1.575312331	1.914564523	2.375734539	2.565597176
Aradu.16QCN	1.269033146	1.883620816	1.189033824	1.731183242	1.765534746
Aradu.V5NS	3.895302621	3.631104282	3.64731451	2.66448284	2.695993813

B					
Aradu.HZ8ES	2.324810603	0.871843649	0.669026766	0.831877241	0.475084883
Aradu.P7UBS	2.568032105	3.502075956	3.609991295	0.925999419	0.333423734
Aradu.80TQM	-3.18442457	-2.83650127	-4.32192809	-1.94341647	-1.51457317
Aradu.M6DL9	NA	-5.05889369	-6.64385619	NA	-6.64385619
Aradu.AU4WK	-1.02914635	1.673556424	0.545968369	2.587364991	3.770829046
Aradu.P4XQA	1.480265122	2.073820233	1.40053793	2.459431619	2.87774425
Aradu.W5WIK	-0.47393119	-1.02914635	-0.86249648	-1.68965988	0.150559677
Aradu.T19XF	4.684818738	4.901590992	5.073820233	3.521050737	3.67468662
Aradu.5Y3NY	2.737686761	2.82984956	2.687060688	3.327687364	3.513490746
Aradu.2CB86	-3.47393119	-2.83650127	-2	-2	-1.18442457
Aradu.ZYW5P	NA	NA	NA	NA	NA
Aradu.N2X0I	NA	-5.64385619	-6.64385619	NA	-3.83650127
Aradu.UUC9E	0.367371066	1.875780063	1.599317794	1.350497247	4.508428653
Aradu.43TNC	-3.18442457	-2.18442457	-3.18442457	-1.21759144	-1.08926734
Aradu.N7IXY	NA	NA	NA	NA	-4.05889369
Aradu.J355A	-1.47393119	-0.88896869	-1.73696559	0.150559677	-0.30400619
Aradu.KV07Y	4.20867332	4.391630262	3.882643049	3.392317423	3.410069692
Aradu.36PEF	1.731183242	1.10433666	0.887525271	0.111031312	0.695993813
Aradu.47FME	-3.47393119	-4.64385619	-1.94341647	-0.15200309	-0.51457317
Aradu.31ZWP	-0.10469738	0.333423734	0.028569152	0.687060688	0.887525271
Aradu.U4UF8	-5.05889369	-4.64385619	-6.64385619	-3.32192809	0.90303827
Aradu.V43LA	1.827819025	2.15704371	1.906890596	2.801158656	3.132576843

Aradu.Y6F9Q	1.03562391	-0.12029423	0.189033824	0.925999419	1.778208576
Aradu.Z4EVV	NA	NA	-6.64385619	-0.37706965	0.895302621
Aradu.JJ2FT	0.042644337	0.575312331	0.23878686	1.189033824	1.438292852
Aradu.V3HYN	1.790772038	2.121015401	1.819668183	1.786596362	2.09423607
Aradu.AW21H	1.454175893	2.060047384	1.839959587	2.5360529	2.983677695
Aradu.H1RA6	2.053111336	2.454175893	1.344828497	2.713695815	1.752748591
Aradu.MK4GU	2.283921772	1.89917563	2.080657663	1.948600847	2.558267634
Aradu.N21E2	1.613531653	2.304511042	1.839959587	3	3.207892852
Aradu.2L1YZ	-0.83650127	0.014355293	-0.94341647	0.298658316	-1.94341647
Aradu.553J0	-0.08926734	-1	-1.21759144	-0.78587519	-0.07400058
Aradu.EW82S	-1.51457317	-1.94341647	-2.83650127	NA	-2.94341647
Aradu.N2G7A	2.735522177	2.438292852	1.933572638	2.341985747	2.780310099
Aradu.V69Z9	NA	NA	NA	NA	NA
Aradu.P7Z3S	2.910732662	3.317593505	3.187451054	3.596935142	3.881664619
Aradu.H9ZXM	0.925999419	1.321928095	1.339137385	1.925999419	2.424922088
Aradu.5B3LS	0.790772038	0.831877241	0.443606651	1.250961574	1.937344392
Aradu.1T3UD	5.747655933	5.580145484	4.277240898	6.774918807	7.008652713
Aradu.Q55UA	0.443606651	1.372952098	0.855989697	3.252476214	2.97819563
Aradu.P9YG3	4.119356177	4.052241981	3.969933275	2.107687869	2.313245852
Aradu.Z4D1S	NA	NA	NA	NA	NA
Aradu.6Y8G9	-1.05889369	2.673556424	2.289834465	-2.25153877	0.35614381
Aradu.D1G1F	0.084064265	-0.39592868	-1.39592868	0.176322773	0.871843649

Aradu.6C4RF	NA	NA	NA	NA	NA
Aradu.VKC3S	-1.51457317	-2.25153877	-2.12029423	-1.55639335	-2.47393119
Aradu.7H3UM	-4.05889369	-4.05889369	-6.64385619	-4.05889369	-2.83650127
Aradu.8B0HV	4.324090516	4.422905743	4.489928219	4.384740587	4.267535798
Aradu.I71PE	-0.53533173	-0.01449957	0.084064265	1.03562391	1.678071905
Aradu.EAE80	2	2.702657543	2.488000771	3.174725988	3.456806149
Aradu.DCQ49	1.933572638	1.622930351	1.361768359	1.49057013	1.480265122
Aradu.CX99Z	1.049630768	1.996388746	1.875780063	1.98550043	2.693765712
Aradu.EXQ60	-1.25153877	-0.94341647	-1.08926734	1.327687364	1.469885976
Aradu.9T44T	-1.78587519	-0.71311885	-1.64385619	-1.28630419	-0.64385619
Aradu.W0TSV	NA	NA	NA	NA	NA
Aradu.Q5M0R	3.839959587	4.152183419	3.921245889	4.536674618	4.566815154
Aradu.B14QA	2.010779839	2.295723025	1.941106311	3.289834465	3.334854269
Aradu.13TYF	0.495695163	1.280956314	0.97819563	1.952333566	2.117695043
Aradu.S38FI	NA	NA	NA	NA	NA
Aradu.SJ15T	2.976363636	3.297191417	3.017921908	2.733354341	3.129283017
Aradu.06NY9	NA	NA	NA	NA	NA
Aradu.N7H5E	NA	NA	NA	NA	NA
Aradu.6FW8M	1.604071324	2.182692298	1.794935663	2.671293372	2.759155834
Aradu.8CB01	NA	NA	-5.64385619	0.056583528	-1.59946207
Aradu.CP5FF	1.378511623	3.139142019	1.819668183	2.23878686	2.147306699
Aradu.00WGF	-0.25153877	-0.91593574	-3.32192809	1.941106311	1.86393845

Aradu.S8IEI	3.694880193	3.801158656	3.933572638	2.253989266	2.422233001
Aradu.D5WZF	1.843983844	2.935459748	2.912649865	1.298658316	0.669026766
Aradu.61NAP	2.195347598	2.462052319	2.257010618	2.813524689	3.381283373
Aradu.M9A72	NA	-5.64385619	NA	-5.64385619	-5.05889369
Aradu.ZYI0Y	3.682573297	3.961623328	3.9800253	-0.76121314	0.871843649
Aradu.M1DIQ	-0.68965988	-0.23446525	-1	0.378511623	0.028569152
Aradu.245YW	-4.05889369	-3.32192809	-4.64385619	-5.64385619	-0.91593574
Aradu.4J3R3	-2.12029423	-1.51457317	-1.83650127	0.970853654	1.510961919
Aradu.BP7BM	-4.32192809	-4.64385619	-4.64385619	-1.47393119	-3.05889369
Aradu.NXM6F	-1.43440282	-0.83650127	-0.59946207	0.887525271	3.075532631
Aradu.ZY590	-0.73696559	0.443606651	0.275007047	0.807354922	0.773996325
Aradu.L2IAX	2.056583528	2.283921772	2.263034406	2.316145742	2.582556003
Aradu.A0Z5X	2.925999419	4.170726276	4.266036894	5.513806537	5.384395238
Aradu.UZX8A	7.403182494	7.061668207	7.061992154	3.13093087	3.15704371
Aradu.X0LHP	3.919340082	4.275007047	3.705977902	4.61999983	4.734438666
Aradu.LNC81	0.575312331	1.286881148	0.956056652	1.5360529	1.627606838
Aradu.95TME	1.427606173	2.277984747	2.257010618	2.117695043	2.381283373
Aradu.MZ49K	-4.32192809	-2.94341647	-4.32192809	0.879705766	0.678071905
Aradu.D8K1Y	1.941106311	1.918386234	1.687060688	0.948600847	2.350497247
Aradu.Q6P6Q	1.339137385	1.298658316	1.372952098	1.416839742	2.596935142
Aradu.MY047	NA	NA	NA	NA	NA
Aradu.79NAD	3.43162296	3.454175893	3.317593505	3.275007047	3.378511623

Aradu.KB518	-0.49410907	0.014355293	-1.73696559	2.286881148	2.883620816
Aradu.35WGP	1.608809243	1.803227036	1.659924558	2.464668267	2.682573297
Aradu.YMP6X	2.700439718	2.570462931	2.584962501	2.560714954	2.941106311
Aradu.U78VM	2.339137385	3.244887059	2.875780063	4.222650022	4.57167681
Aradu.97AME	NA	NA	NA	NA	-5.64385619
Aradu.5DD09	8.183188734	7.483896373	7.527711572	2.495695163	2.641546029
Aradu.NI3IK	2.883620816	3.443606651	3.286881148	3.486714373	3.711494907
Aradu.TM0DS	0.641546029	1.752748591	1.014355293	2.684818738	2.744161096
Aradu.P1TZQ	-3.47393119	-3.05889369	-6.64385619	-2.05889369	-1.12029423
Aradu.60DAC	-2.64385619	-1.47393119	-1.59946207	-1.39592868	-0.73696559
Aradu.3R84Q	4.59812696	4.672425342	4.787641414	5.102238194	5.739308071
Aradu.R8UZU	-3.47393119	-1.88896869	-2.94341647	-1.47393119	-1.83650127
Aradu.IFZ2Q	3.030336078	3.012568674	2.724650272	1.933572638	2.003602237
Aradu.2HJ80	2.827819025	3.339137385	2.906890596	4.275752049	4.277984747
Aradu.P49BC	2.5360529	3.106013238	2.851998837	2.430285273	2.485426827
Aradu.7WG5V	-1.05889369	-1.43440282	-2	-0.26881676	-0.76121314
Aradu.JC2LL	3.99095486	4.207892852	4.355439197	3.763411574	3.979110755
Aradu.810XL	5.355439197	5.142821844	5.159467729	5.323730338	5.485748821
Aradu.K16RE	2.731183242	3.545968369	3.311793718	3	3.040892431
Aradu.MP583	NA	-5.05889369	-6.64385619	-4.32192809	-2.39592868
Aradu.Z93ZE	5.415150205	5.438958148	5.289465631	3.710393192	3.980939266
Aradu.V4IDM	-1.78587519	-2.55639335	-2.73696559	-4.05889369	-2.83650127

Aradu.S94H9	2.739848103	2.673556424	2.704871964	2.523561956	3.529820947
Aradu.LP9ZI	3.792855352	3.738767837	3.704871964	2.752748591	3.261530815
Aradu.G05M1	0.575312331	1.922197848	1.906890596	4.365972428	3.392317423
Aradu.8542F	NA	NA	NA	NA	NA
Aradu.N8PYS	-1.68965988	-1.28630419	-1.64385619	1.40599236	0.575312331
Aradu.W07KG	-0.47393119	-0.25153877	-0.02914635	4.169123281	3.326249701
Aradu.7B9HI	4.072963272	4.904484098	4.341985747	5.45779126	5.471837762
Aradu.ZN5ZV	4.731726323	4.542877099	4.73714592	3.684818738	4.584361253
Aradu.C8ZMR	-0.71311885	-1.02914635	-1	-0.49410907	-0.91593574
Aradu.56DPU	3.217230716	3.933572638	3.308885057	4.926948248	4.934044647
Aradu.76BXR	1.411426246	2.459431619	1.49057013	3.030336078	2.981852653
Aradu.UQP4Y	2.114367025	2.277984747	2.3950628	2.392317423	2.922197848
Aradu.SCG5D	NA	NA	NA	-2.64385619	0.35614381
Aradu.1B2LG	NA	-5.64385619	NA	NA	NA
Aradu.9W64L	2.298658316	2.438292852	3.815575429	0.189033824	0.40053793
Aradu.V6900	2.292781749	2.742006211	2.498250868	2.748461233	3.455491621
Aradu.M2B10	-0.83650127	-1.05889369	-1.32192809	-3.47393119	-0.18442457
Aradu.WLL49	2.384049807	3.13422094	2.192194165	3.145677455	2.976363636
Aradu.M9L15	-0.07400058	0.5360529	0.310340121	-0.25153877	1.063502942
Aradu.01E53	2.358958826	2.773996325	2.201633861	3.67468662	3.809414444
Aradu.LP8UK	3.303050085	3.367371066	3.125981654	3.776103988	4.011674533
Aradu.RUS0X	0.887525271	1.516015147	0.918386234	3.111031312	3.250961574

Aradu.71MQE	5.216842843	6.434127784	6.402585758	5.361417467	6.422569411
Aradu.U77H9	-0.49410907	-0.21759144	-1.02914635	1.589763487	1.90303827
Aradu.FTT59	1.310340121	1.891419187	1.422233001	5.015247774	5.21023299
Aradu.DYF0M	-5.05889369	NA	NA	NA	-2.39592868
Aradu.NI3KM	5.857483428	6.063287212	5.694044413	6.658639942	6.818518269
Aradu.DA7MW	-0.51457317	0.298658316	0.815575429	-1.47393119	-0.78587519
Aradu.B26BB	NA	NA	NA	-5.05889369	-6.64385619
Aradu.A3D3N	0.910732662	0.90303827	0.695993813	0.925999419	1.63691458
Aradu.CV6Z5	NA	NA	NA	NA	NA
Aradu.BUW4Z	-5.64385619	-6.64385619	NA	NA	-5.64385619
Aradu.WQI06	2.443606651	2.66448284	2.448900951	1.565597176	1.807354922
Aradu.ZG4JR	1.280956314	1.443606651	1.23878686	2.153805336	2.797012978
Aradu.C3ARN	-0.13606155	0.495695163	0.275007047	1.056583528	0.807354922
Aradu.5S25K	3.857980995	4.014355293	3.66106548	2.89917563	3.220329955
Aradu.KR2M4	-3.18442457	-2.55639335	-2	-3.64385619	-0.59946207
Aradu.Q1S4K	0.98550043	0.933572638	1.13093087	0.871843649	0.918386234
Aradu.E1ZU5	3.171527106	3.553360503	3.566815154	4.282439805	4.320484678
Aradu.N0WNK	1.895302621	2.416839742	2.053111336	3.343407822	3.510961919
Aradu.I118N	NA	NA	NA	NA	NA
Aradu.54FG7	-0.02914635	1.673556424	1.459431619	3.514753498	3.859969548
Aradu.U6RXK	NA	NA	NA	NA	NA
Aradu.D9ISK	0.799087306	1.070389328	0.632268215	2.150559677	2.263034406

Aradu.0NY8Y	3.390942773	3.7473874	3.49057013	2.904965719	3.03562391
Aradu.ZW165	1.3950628	1.726831217	1.5360529	2.414135533	2.788685711
Aradu.DT20E	1.851998837	2.344828497	2.007195501	3.456806149	3.434294618
Aradu.GJ5P4	-4.32192809	-3.05889369	-5.05889369	-3.83650127	-3.64385619
Aradu.LVR9T	4.474436043	4.092545742	4.249445341	2.14404637	2.671293372
Aradu.36UYV	0.799087306	1.416839742	0.90303827	1.618238656	1.89917563
Aradu.DH828	7.284569655	7.40752271	7.075211711	3.609991295	4.029452886
Aradu.QPZ5M	NA	NA	NA	-4.64385619	NA
Aradu.J59GH	2.066950244	2.353323291	2.032100843	2.384049807	2.865918815
Aradu.TJC58	3.047887329	3.053111336	2.893362211	2.98550043	3.456806149
Aradu.S0YHI	3.548436625	4.076388069	3.746312766	4.61176256	4.926948248
Aradu.X0EAG	1.63691458	2.007195501	1.803227036	1.839959587	2.097610797
Aradu.C5DXV	4.117695043	4.192194165	3.837943242	5.647602329	5.819668183
Aradu.142E0	-1.47393119	-2.32192809	-2.39592868	-0.76121314	0.298658316
Aradu.UD5FH	3.021479727	2.263034406	2.49057013	3.450221496	3.267535798
Aradu.A61EY	-5.64385619	-4.64385619	-5.64385619	-1.68965988	-1.88896869
Aradu.RUT6G	-5.05889369	-3.05889369	-4.32192809	-2.47393119	-1.59946207
Aradu.PF1HF	1.361768359	1.333423734	1.422233001	0.50589093	0.871843649
Aradu.CM6S6	NA	-6.64385619	-6.64385619	NA	NA
Aradu.3N314	3.163498732	3.970853654	3.163498732	1.704871964	2.364572432
Aradu.S0XAG	4.125155131	4.04176865	4.169925001	2.742006211	2.580145484
Aradu.X9ZB3	-0.71311885	-0.28630419	-0.73696559	0.084064265	0.464668267

Aradu.LS42S	2.44625623	2.204766751	2.523561956	3.892391026	3.392317423
Aradu.EV49X	8.711976642	8.374387464	8.541522583	3.95419631	3.881664619
Aradu.MH3SJ	NA	NA	NA	NA	NA
Aradu.68MP6	3.111031312	2.972692654	2.987320866	2.272023189	3.370164281
Aradu.D5C91	4.816087659	5.649040566	5.422905743	4.368069877	4.09000653
Aradu.U0V69	2.867896464	3.189033824	2.879705766	3.270528942	3.695993813
Aradu.P7PS3	1.389566812	1.735522177	2.286881148	1.98550043	2.807354922
Aradu.T25QT	-1.21759144	-1.18442457	-1.68965988	0.475084883	0.50589093
Aradu.ILB9Z	5.241077458	5.374343989	5.470536865	3.642701572	4.358958826
Aradu.XWZ7Z	2.914564523	2.969012308	2.752748591	3.026800059	3.493134922
Aradu.V5HPY	-2.39592868	-1.47393119	-3.18442457	1.201633861	1.526068812
Aradu.ZEB3I	3.459431619	3.879705766	3.443606651	4.647890091	4.66448284
Aradu.7ZG0H	2.851998837	3.337711092	3.410069692	2.482848283	2.397802962
Aradu.D4CLJ	1.847996907	2.087462841	1.757023247	3.224966365	3.426264755
Aradu.K9JBW	1.070389328	1.40053793	1.014355293	2.150559677	2.304511042
Aradu.TTW29	7.627971551	8.031494449	7.529118164	7.367720513	7.136683578
Aradu.JMV7E	6.842350343	7.304419775	6.968897145	7.613752637	7.206526016
Aradu.001N3	-0.35845397	-0.4150375	-0.97143085	1.339137385	1.372952098
Aradu.5IN4K	NA	NA	NA	NA	NA
Aradu.C96YL	0.475084883	2.451540833	1.959770155	1.22650853	1.678071905
Aradu.6K5DV	3.099295204	3.272023189	3.155425432	3.469885976	3.557042415
Aradu.BM2KZ	3.751677946	2.64385619	3.357552005	-5.05889369	-3.83650127

Aradu.83Z01	1.163498732	2.835924074	2.087462841	3.09423607	3.211012193
Aradu.CLE55	-3.83650127	-2.05889369	-2.47393119	-0.88896869	-1
Aradu.D1CF5	NA	-6.64385619	-6.64385619	-5.64385619	-1.51457317
Aradu.JQ7JG	2.709290636	2.815575429	2.629939409	3.22342255	3.655351829
Aradu.ZJ0E3	-3.83650127	-2.83650127	-3.32192809	-1	-0.78587519
Aradu.P4R4T	NA	-4.32192809	-4.64385619	1.23878686	1.757023247
Aradu.MD5SA	1.250961574	2.347665656	1.867896464	2.906890596	2.801158656
Aradu.66KCD	2.682573297	3.075532631	2.634593268	3.184280294	3.323370069
Aradu.V34QS	2.543495883	3.007195501	2.707082992	3.646162657	3.987320866
Aradu.3L4RS	NA	NA	-6.64385619	1.718087584	-0.43440282
Aradu.TF0TM	2.189033824	2.173127433	2.087462841	0.545968369	0.604071324
Aradu.T9UI2	2.007195501	2.14404637	2.111031312	3.550900665	3.54225805
Aradu.4V3WP	NA	NA	NA	NA	NA
Aradu.3P5HB	2.307428525	2.364572432	2.659924558	3.3305584	3.184280294
Aradu.H5EIL	0.879705766	0.879705766	0.659924558	0.799087306	1.310340121
Aradu.UJ9WA	NA	NA	NA	-4.64385619	3.249445341
Aradu.40DSM	3.039138394	3.66448284	3.229587923	4.060047384	4.069530325
Aradu.4U4LS	3.334854269	3.403267722	3.10433666	3.326249701	3.416839742
Aradu.UY6CA	0.687060688	2.150559677	2.13093087	1.627606838	1.757023247
Aradu.7Y3DJ	5.813524689	4.258518925	5.10349764	2.080657663	2.13093087
Aradu.A9H9T	NA	NA	NA	NA	NA
Aradu.P2KMP	1.263034406	1.422233001	1.150559677	1.691534165	2.017921908

Aradu.6SC3T	4.259272487	4.635754391	3.559491813	6.050501697	6.574858391
Aradu.C1S3R	-0.59946207	-0.37706965	-0.81096618	-0.07400058	0.613531653
Aradu.S20WA	2.114367025	2.451540833	2.339137385	2.40053793	2.669026766
Aradu.ZS8UE	2.253989266	2.646162657	2.5360529	3.313245852	4.009884589
Aradu.X03Q4	-0.02914635	0	0.50589093	-0.64385619	-0.28630419
Aradu.F19SY	1.815575429	5.407012774	4.85698569	5.152589069	5.170325694
Aradu.KX3FZ	4	4.159467729	4.369466484	1.220329955	2.003602237
Aradu.N1ASM	NA	NA	NA	NA	NA
Aradu.EG5ES	1.378511623	1.327687364	1.214124805	3.070389328	3.165107985
Aradu.7JY58	1.687060688	1.555816155	1.485426827	1.361768359	1.459431619
Aradu.B4CLV	0.389566812	1.15704371	0.201633861	2.204766751	1.700439718
Aradu.T1K5W	NA	NA	NA	-4.05889369	-4.64385619
Aradu.NGG06	2.893362211	3.326249701	3.017921908	3.701549057	3.952333566
Aradu.S156I	-5.64385619	-4.64385619	-4.64385619	NA	NA
Aradu.RQQ24	1.875780063	2.032100843	1.843983844	2.757023247	3.116031993
Aradu.R9RNU	NA	-6.64385619	NA	-6.64385619	-4.05889369
Aradu.JF9VE	3.056583528	3.176322773	2.931683057	2.918386234	3.364572432
Aradu.I83LC	2.313245852	2.324810603	1.895302621	3.851998837	3.762348816
Aradu.00SPE	1.632268215	2.12763328	1.855989697	2.007195501	2.625270489
Aradu.L2BKM	-5.05889369	-3.64385619	-5.64385619	-5.64385619	1.726831217
Aradu.JW82A	3.244887059	3.49057013	3.250961574	5.651051691	5.405311683
Aradu.RI6SZ	-5.64385619	NA	NA	-2.25153877	-2.64385619

Aradu.KNK4F	2.166715445	2.419538892	1.871843649	2.684818738	2.918386234
Aradu.ZEU97	2.565597176	2.90303827	1.531069493	1.137503524	0.678071905
Aradu.EB27U	2.906890596	3.228049048	3.119356177	3.303050085	3.918386234
Aradu.82FNR	NA	NA	NA	NA	NA
Aradu.Z9J76	NA	NA	NA	NA	NA
Aradu.J2HBF	0.23878686	0.411426246	0.367371066	-0.20091269	1.007195501
Aradu.ERN5M	-2	-2.47393119	-2.32192809	-1.32192809	-0.18442457
Aradu.V94FJ	1.220329955	1.803227036	1.599317794	1.895302621	1.956056652
Aradu.0Q24B	3.087462841	3.548436625	3.516015147	3.443606651	3.632268215
Aradu.Q2QD0	-3.18442457	-3.47393119	-2.25153877	-0.81096618	0.250961574
Aradu.JF3DE	6.956521363	6.75033852	6.638653116	4.543495883	4.678635348
Aradu.ZV73M	7.815575429	7.580070091	7.693068724	4.023255352	4.495055528
Aradu.LR6SL	NA	-5.64385619	NA	-0.32192809	0.286881148
Aradu.63Q7N	4.759155834	5.170726276	5.242602506	2.301587647	2.632268215
Aradu.V3MUV	0.495695163	1.646162657	1.097610797	2.295723025	2.350497247
Aradu.6L9XG	3.047887329	3.308885057	3.112700133	3.61117238	3.715893371
Aradu.1K1MI	-1	-0.71311885	-0.68965988	-1.28630419	-1.08926734
Aradu.GTI49	2.815575429	3.343407822	2.533563348	4.378511623	4.332707934
Aradu.K8H83	1.263034406	1.613531653	1.084064265	2.09085343	2.451540833
Aradu.YD1VK	-3.05889369	-3.32192809	-4.64385619	0.443606651	-0.05889369
Aradu.C8SSW	NA	-2.18442457	-1.35845397	-3.64385619	-1.64385619
Aradu.WJZ15	1.10433666	1.50589093	1.257010618	1.495695163	1.480265122

Aradu.G47J2	-3.32192809	-4.64385619	-3.47393119	NA	-4.64385619
Aradu.J6GHV	1.372952098	3.016139703	2.853995647	2.541019153	3.294253136
Aradu.AFL8M	1.722466024	3.488000771	3.016139703	1.678071905	2.381283373
Aradu.LZ6SK	6.524189078	7.941165013	8.353455626	12.60507434	11.33118008
Aradu.1E0WP	1.914564523	1.910732662	2.182692298	-0.32192809	0.042644337
Aradu.FL7H6	0.956056652	1.713695815	1.575312331	2.017921908	2.601696516
Aradu.A2VXS	1.3950628	1.922197848	1.922197848	1.550900665	2.166715445
Aradu.LEE20	-0.30400619	-0.26881676	-0.91593574	-0.66657627	-0.12029423
Aradu.99GQI	1.316145742	1.575312331	1.5360529	2.23878686	2.577730931
Aradu.908DD	1.618238656	2.084064265	1.773996325	3.005399988	2.893362211
Aradu.K0L5G	NA	NA	-6.64385619	-4.05889369	-3.64385619
Aradu.2L2SE	0.632268215	0.137503524	0.137503524	-2.47393119	-1.05889369
Aradu.I88HR	1.378511623	0.918386234	0.831877241	NA	-1.39592868
Aradu.C8KRG	-2.55639335	-2.94341647	-4.64385619	-3.18442457	-3.18442457
Aradu.RP61F	-1.35845397	-0.78587519	-1.68965988	-0.13606155	-0.53533173
Aradu.F0Y0D	3.285402219	3.030336078	2.622930351	3.414135533	3.410069692
Aradu.947SP	-5.05889369	-4.64385619	-3.64385619	-3.83650127	-3.47393119
Aradu.ESL6S	-3.05889369	-6.64385619	NA	NA	-4.64385619
Aradu.0Y2ZI	-1.83650127	-0.71311885	-1.08926734	0.111031312	0.163498732
Aradu.1LZ8T	0.90303827	1.500802053	1.925999419	0.516015147	0.757023247
Aradu.35TX9	2.22650853	2.304511042	1.895302621	2.229587923	2.419538892
Aradu.V45Z7	-2.18442457	-0.64385619	-0.91593574	-0.97143085	-1.18442457

Aradu.W3VUI	3.854993017	4.017031081	3.66106548	2.809414444	2.944858446
Aradu.98WRB	2.09423607	1.835924074	1.678071905	0.056583528	1.111031312
Aradu.M0ZRG	-1.78587519	-0.04394335	-0.91593574	1.448900951	1.321928095
Aradu.IPP1D	6.626877135	6.139551352	7.034853974	3.971773447	3.360364277
Aradu.07WQA	0.757023247	1.056583528	0.59454855	3.941106311	3.601696516
Aradu.ANS8T	1.941106311	2.114367025	1.944858446	2.912649865	3.805292456
Aradu.L66NF	NA	NA	NA	NA	0.516015147
Aradu.U53UJ	NA	-5.05889369	-5.64385619	-3.47393119	-4.64385619
Aradu.Y8LHL	10.66340689	10.17939908	10.82423483	7.22650853	7.256350238
Aradu.G2CUK	NA	-4.32192809	-3.83650127	-4.05889369	-2.32192809
Aradu.G07IW	1.327687364	1.769771739	1.650764559	1.937344392	2.09423607
Aradu.Z1DQ9	-1.28630419	-1.18442457	-1.08926734	-2.83650127	-0.12029423
Aradu.XQ3RE	4.18030702	6.321567376	6.529977074	6.215678597	5.010779839
Aradu.40QFE	NA	NA	NA	-0.88896869	-0.05889369
Aradu.4KE9F	NA	NA	NA	NA	NA
Aradu.4D9C8	0.831877241	0.584962501	0.731183242	2.657640005	3.163498732
Aradu.C1KJF	2.786596362	2.709290636	2.604071324	1.807354922	2.553360503
Aradu.I5YUI	0.669026766	1.070389328	1.15704371	-0.71311885	0.042644337
Aradu.UEF4U	NA	NA	NA	-6.64385619	-4.32192809
Aradu.826D1	3.361768359	3.085764554	2.82374936	3.422233001	3.456806149
Aradu.BD2SQ	-2.64385619	-1.47393119	-1.94341647	0.695993813	0.773996325
Aradu.8203M	4.539158811	5.259272487	4.7382274	4.18030702	4.178714641

Aradu.7HS2D	-4.64385619	-5.05889369	-6.64385619	-1.28630419	-1.35845397
Aradu.1347E	1.028569152	1.438292852	1.201633861	1.726831217	1.970853654
Aradu.HQ5WT	NA	NA	NA	-2.47393119	-2.32192809
Aradu.KJM0J	-2.64385619	-2.73696559	-4.64385619	-2.83650127	-0.59946207
Aradu.G26I2	4.475733431	3.298658316	3.341985747	3.537296067	3.622930351
Aradu.H3XI7	0.773996325	0.555816155	0.163498732	2.307428525	2.813524689
Aradu.8P3R8	0.687060688	1.304511042	0.839959587	2.049630768	1.895302621
Aradu.67PUF	0.918386234	1.815575429	1.021479727	2.160274831	2.301587647
Aradu.S0KU9	-2.64385619	-1.68965988	-1.47393119	-1.68965988	-0.55639335
Aradu.27DYI	0.310340121	0.111031312	-0.16812276	1.565597176	1.819668183
Aradu.ZM9CR	0.124328135	0.839959587	0.704871964	1.03562391	1.459431619
Aradu.HER1A	1.469885976	1.510961919	1	3.538538164	3.575312331
Aradu.5JM2L	5.013908845	4.457462965	4.973152038	1.339137385	1.550900665
Aradu.2HT1T	-1.18442457	-0.88896869	-1.78587519	-0.53533173	0.214124805
Aradu.3W0JX	-5.64385619	-5.05889369	-5.64385619	-1.78587519	1.263034406
Aradu.G2VQL	1.443606651	1.169925001	1.280956314	2.533563348	2.13093087
Aradu.0PL1F	2.526068812	2.724650272	1.914564523	2.207892852	2.182692298
Aradu.NH44H	1.867896464	3.153805336	2.748461233	3.09423607	3.97819563
Aradu.JQD1M	-2.94341647	-2.94341647	-3.32192809	-1.55639335	-1.18442457
Aradu.S9BZU	0.028569152	0.748461233	0.124328135	1.695993813	1.589763487
Aradu.N98FX	0.678071905	0.070389328	-0.15200309	-2.12029423	0.773996325
Aradu.298AN	-2.25153877	-1.68965988	-3.05889369	-1.68965988	-1.78587519

Aradu.7P55W	0.748461233	1.565597176	0.887525271	1.608809243	1.485426827
Aradu.VG3CJ	-0.37706965	-1.21759144	-1.25153877	1.480265122	2.769771739
Aradu.XEA0F	NA	NA	-6.64385619	-4.32192809	-2.32192809
Aradu.7RC99	1.316145742	1.150559677	1.339137385	2.358958826	2.324810603
Aradu.T2QCP	2.087462841	2.301587647	2.495695163	2.5360529	2.935459748
Aradu.ZCP8X	-0.51457317	-1	-0.64385619	-0.07400058	0.137503524
Aradu.F4S3N	-1.59946207	-1.59946207	-1.68965988	0.176322773	0.084064265
Aradu.M3B1E	1.531069493	1.700439718	1.584962501	1.117695043	1.475084883
Aradu.7W6T8	2.632268215	2.669026766	2.217230716	3.171527106	3.675815931
Aradu.U7HHN	4.700994494	3.998195503	3.689299161	4.313971371	3.397802962
Aradu.5B2EV	0.799087306	1.189033824	0.790772038	2.358958826	2.007195501
Aradu.N49M2	1.117695043	1.195347598	0.739848103	2.687060688	2.459431619
Aradu.1HQ4L	-5.64385619	-6.64385619	-6.64385619	-2.94341647	-4.05889369
Aradu.IS3BT	-1.73696559	-1.32192809	-2.64385619	-1.21759144	-1.18442457
Aradu.L7ZWM	1.90303827	1.948600847	1.910732662	1.641546029	2.046141782
Aradu.7I9FX	-5.05889369	-6.64385619	-6.64385619	-4.32192809	-5.64385619
Aradu.SC9VF	2.475084883	2.448900951	4.622344723	2.066950244	0.831877241
Aradu.RSV1J	-3.83650127	-3.83650127	-4.05889369	-2.55639335	2.477677328
Aradu.F2R8P	1.718087584	2.042644337	1.871843649	2.416839742	2.845991771
Aradu.BSR1D	0.432959407	1.570462931	1.007195501	2.443606651	2.788685711
Aradu.TLI73	4.19298317	4.442943496	4.634593268	2.867896464	2.835924074
Aradu.7GM9W	2.895302621	3.3950628	3.252476214	4.008988783	4.401220862

Aradu.M73X9	3.526068812	3.523561956	3.249445341	3.606442228	3.621758857
Aradu.X2Y57	1.963474124	2.648465443	2.735522177	2.803227036	4.194559886
Aradu.4707K	-0.43440282	-0.73696559	-0.91593574	-0.25153877	0.910732662
Aradu.NN536	NA	NA	NA	NA	NA
Aradu.QK488	0.956056652	0.879705766	0.970853654	-0.88896869	-0.05889369
Aradu.CVT5N	NA	NA	NA	NA	NA
Aradu.8X203	0.526068812	1.111031312	0.815575429	1.521050737	1.650764559
Aradu.J2H81	-4.05889369	-4.64385619	-4.32192809	-6.64385619	-2.47393119
Aradu.QYX69	NA	NA	-5.64385619	-4.32192809	6.689578726
Aradu.73MTJ	NA	-6.64385619	NA	-5.05889369	-5.64385619
Aradu.GN2JM	-3.64385619	-6.64385619	NA	-0.43440282	-0.47393119
Aradu.7K02I	1.627606838	1.627606838	1.655351829	1.819668183	2.021479727
Aradu.MG836	3.771885579	4.141596278	3.897240426	4.657068301	4.811984719
Aradu.TM4JI	0.970853654	1.475084883	1.189033824	2.313245852	2.495695163
Aradu.1W5FD	-0.76121314	2.538538164	2.017921908	-1.83650127	0.201633861
Aradu.FWD57	2.875780063	3.137503524	2.918386234	4.209453366	4.268284667
Aradu.W9GMQ	2.384049807	2.99095486	2.675815931	3.430285273	3.572889668
Aradu.T6XQA	NA	NA	-4.64385619	NA	NA
Aradu.4TU5K	2.604071324	3.047887329	2.653060017	4.057450272	4.046141782
Aradu.MW11Y	-1.05889369	-0.47393119	-0.94341647	2.204766751	2.759155834
Aradu.S0871	4.159467729	4.084064265	3.87774425	2.659924558	2.904965719
Aradu.YC13W	-3.64385619	-2.73696559	-3.05889369	-4.64385619	-0.71311885

Aradu.K6UHT	1.207892852	1.292781749	1.207892852	-1.39592868	-2
Aradu.B7H15	-4.64385619	-2.55639335	-2.73696559	-1.18442457	-1.47393119
Aradu.XZP9V	1.182692298	1.163498732	1.891419187	0.879705766	1.063502942
Aradu.G85IC	-4.64385619	-4.05889369	-5.64385619	-5.05889369	NA
Aradu.ZC5IW	2.053111336	2.375734539	2.411426246	2.881664619	4.13422094
Aradu.50FPJ	1.097610797	1.646162657	1.480265122	2.014355293	2.553360503
Aradu.XC8VW	0.773996325	1.678071905	0.604071324	2.813524689	2.908812908
Aradu.F2DT2	-4.64385619	-4.05889369	-5.05889369	-3.47393119	-2.25153877
Aradu.G9RLQ	-1.28630419	-2.47393119	-1.83650127	-3.64385619	0.773996325
Aradu.HM0P2	4.419538892	4.468583317	4.609991295	2.513490746	2.13093087
Aradu.1JK1L	1.835924074	1.895302621	1.790772038	2.771885579	2.912649865
Aradu.M3CK1	NA	NA	NA	NA	NA
Aradu.MJ7TV	5.630230716	5.867402306	5.923862268	3.765534746	3.854993017
Aradu.BF8QQ	0.475084883	2.12763328	1.786596362	2.500802053	2.272023189
Aradu.BNM8A	1.855989697	2.063502942	1.959770155	2.298658316	2.358958826
Aradu.GNV5R	NA	-5.64385619	-6.64385619	NA	-2.94341647
Aradu.G6ZH0	2.722466024	3.145677455	3.187451054	4.10349764	4.363871925
Aradu.H1FFF	0.250961574	0.757023247	0.432959407	1.748461233	1.40599236
Aradu.U7EBV	1.244887059	1.835924074	1.49057013	2.731183242	2.827819025
Aradu.ESU02	2.87774425	1.952333566	2.427606173	0.545968369	1.959770155
Aradu.0X9JV	2.080657663	2.801158656	2.049630768	3.74092756	3.734438666
Aradu.CKD1V	2.147306699	2.773996325	2.454175893	3.198494154	3.439623138

Aradu.D72GI	-1.21759144	-3.18442457	0.214124805	-3.05889369	-1.21759144
Aradu.LBR87	-3.05889369	-2.73696559	-1.88896869	-0.71311885	-0.12029423
Aradu.TD3N5	3.092545742	3.140778656	3.198494154	3.27351589	3.458119481
Aradu.0VP21	-3.47393119	-4.64385619	-4.64385619	-3.05889369	-4.32192809
Aradu.GC1Z5	-0.55639335	-0.37706965	-0.81096618	-0.23446525	-0.18442457
Aradu.TP6EC	-0.39592868	0.097610797	-1.25153877	2.689299161	2.724650272
Aradu.0FB9R	NA	-5.64385619	-6.64385619	NA	-5.64385619
Aradu.SJK47	2.839959587	3.436961338	3.155425432	4.372952098	4.526694846
Aradu.B1NGL	NA	NA	NA	NA	-2.32192809
Aradu.3N6NA	0.333423734	0.790772038	0.695993813	2.538538164	2.49057013
Aradu.YCB13	-0.39592868	-1.25153877	-0.86249648	-3.47393119	-2
Aradu.1DJ56	0.584962501	1.560714954	1.367371066	2.666756592	2.989139007
Aradu.BH9NG	1.879705766	2.09085343	1.959770155	2.742006211	3.019701914
Aradu.B83GZ	-1.25153877	-0.59946207	-1	-1.32192809	-0.53533173
Aradu.X5WR6	0.028569152	0.839959587	-0.64385619	3.832890014	3.581351247
Aradu.LF723	5.960697039	5.659639187	5.877253454	3.655351829	4.163498732
Aradu.Q1RYU	5.128870759	5.899900664	4.72955277	7.380850638	7.327956767
Aradu.HK1JB	3.532316959	3.469885976	3.326249701	2.521050737	3.003602237
Aradu.PDJ58	-3.47393119	-3.83650127	-5.64385619	-3.47393119	NA
Aradu.209EV	-3.83650127	-4.05889369	-5.64385619	NA	-5.05889369
Aradu.LT240	-1.25153877	-1.15200309	-1.35845397	-5.64385619	-3.05889369
Aradu.L6IIL	-2.94341647	-5.64385619	-3.64385619	-0.51457317	1.925999419

Aradu.F297C	-1.21759144	-2.55639335	-2.32192809	-2	-2.18442457
Aradu.S3HBK	5.0725346	4.710393192	4.854494418	2.124328135	3.173127433
Aradu.H49WY	2.384049807	3.334854269	3.221877081	3.615887074	3.634593268
Aradu.K3TU7	NA	-5.05889369	-5.05889369	NA	-4.64385619
Aradu.H306E	2.857980995	2.827819025	2.845991771	3.314696526	3.749534268
Aradu.DU86V	2.166715445	1.875780063	2.140778656	0.748461233	0.613531653
Aradu.T0NTP	-0.21759144	0.464668267	0.189033824	2.280956314	2.726831217
Aradu.P778G	-1.64385619	-0.73696559	-1.43440282	1.150559677	1.13093087
Aradu.AA5ZE	3.169925001	3.418189948	3.260025656	3.735522177	4.045268215
Aradu.BF9MN	2.46727948	2.901108243	2.358958826	3.669026766	3.736604875
Aradu.23ZKM	0.807354922	1.195347598	0.887525271	1.859969548	2.042644337
Aradu.PK7XR	3.148934105	3.489286023	3.14404637	3.621758857	3.7473874
Aradu.951UC	3.220329955	3.92314918	3.363171077	6.621905346	7.251624424
Aradu.4PC58	-5.05889369	NA	NA	-5.05889369	-1.05889369
Aradu.B04QQ	-3.83650127	-5.64385619	-4.64385619	-2.73696559	-3.18442457
Aradu.VXS15	-4.05889369	-4.64385619	-4.64385619	2.61117238	1.981852653
Aradu.0G2ML	2.13093087	2.150559677	2.204766751	2.718087584	3.070389328
Aradu.D80L9	1.448900951	1.726831217	1.555816155	1.613531653	1.799087306
Aradu.AN363	3.469885976	3.731183242	3.370164281	4.234961095	4.069530325
Aradu.P156Z	4.64731451	5.554895775	5.390942773	6.363521544	6.028348134
Aradu.88KLW	0.124328135	0.250961574	-0.08926734	2.286881148	2.324810603
Aradu.1DA34	2.301587647	3.13422094	2.469885976	3.502075956	3.314696526

Aradu.H1ZYW	3.862947248	4.886550147	4.161081482	6.198690586	6.124741692
Aradu.W0GZN	3.845991771	4.006298024	3.837943242	3.065227623	3.182692298
Aradu.A36AV	-0.86249648	-0.02914635	-2	-3.47393119	-2.39592868
Aradu.Q3RYP	3.921245889	4.151371776	3.858975614	5.128045891	5.335211682
Aradu.SC6RY	2.121015401	2.13422094	1.23878686	3.177917792	2.788685711
Aradu.8SH4F	NA	NA	NA	NA	NA
Aradu.15KNS	1.344828497	1.169925001	1.269033146	1.704871964	2.124328135
Aradu.88IFJ	-3.18442457	-2.64385619	-3.32192809	-0.20091269	0.831877241
Aradu.ASE53	0.275007047	0.722466024	1.316145742	-3.05889369	-1.94341647
Aradu.NU5M2	1.541019153	2.140778656	1.718087584	3.153805336	3.382667253
Aradu.MQQ90	3.13422094	3.440952198	3.439623138	4.129283017	4.055716264
Aradu.JGE04	3.129283017	3.135863165	2.912649865	1.86393845	2.289834465
Aradu.S94TG	-4.05889369	-5.64385619	-5.05889369	NA	-0.68965988
Aradu.7MV2V	-1	-1.47393119	-1.78587519	-1.35845397	-0.78587519
Aradu.MYV5A	-1.15200309	-1.18442457	-1.43440282	-3.32192809	1.028569152
Aradu.E9DCA	2.422233001	2.847996907	2.408711861	3.665620164	3.815575429
Aradu.L8JCD	1.748461233	1.655351829	1.422233001	3.351910961	3.153805336
Aradu.RA5XM	4.137503524	4.43629512	4.154615611	5.152994605	5.300489844
Aradu.NUY4D	3.037382222	3.622930351	3.121015401	2.742006211	2.847996907
Aradu.GJY15	-5.64385619	-3.64385619	-6.64385619	-2.94341647	-2.55639335
Aradu.CU60X	3.840966704	4.112700133	4.331275267	4.083213368	4.221103725
Aradu.UU4C2	-1.02914635	-0.53533173	-1.32192809	1.731183242	2.313245852

Aradu.I99RK	1.182692298	1.786596362	2.007195501	1.819668183	2.289834465
Aradu.75JKD	-1.88896869	0.150559677	0.516015147	2.14404637	2.03562391
Aradu.GC3CE	NA	NA	NA	NA	NA
Aradu.2YY9U	1.189033824	2.367371066	1.659924558	3.129283017	3.412781525
Aradu.T3K2L	0.970853654	1.316145742	1.163498732	1.678071905	2.010779839
Aradu.1D399	0.90303827	1.526068812	1.137503524	2.748461233	2.560714954
Aradu.V3AZX	1.298658316	0.948600847	1.411426246	-4.32192809	-3.47393119
Aradu.DE67Z	-0.97143085	-2.32192809	-1.68965988	-3.05889369	-1.51457317
Aradu.4LY04	1.014355293	-0.23446525	-0.577767	-1.47393119	-0.37706965
Aradu.7FW6D	3.12763328	3.72900887	3.161887682	4.637494325	4.843481425
Aradu.WX32R	3.261530815	3.675815931	3.522306893	3.916476644	4.196921734
Aradu.YLM4Q	3.634593268	3.331991778	3.507160349	3.303050085	2.799087306
Aradu.J67TY	3.333423734	2.117695043	1.843983844	0.411426246	0.948600847
Aradu.KSI0H	-3.32192809	-1.55639335	-1.78587519	-1.73696559	-0.28630419
Aradu.49T9W	-0.10469738	-1.43440282	-0.83650127	0.23878686	0.622930351
Aradu.0P9WS	4.033863452	4.078951341	3.936402378	4.62058641	4.780835002
Aradu.ZGD79	0.713695815	1.443606651	0.855989697	1.50589093	1.883620816
Aradu.DF5I2	1.827819025	2.09085343	1.769771739	2.98550043	3.220329955
Aradu.GZ0CM	2.493134922	2.711494907	2.403267722	3.565597176	3.800123353
Aradu.CC9MM	0	0.454175893	0.22650853	1.056583528	1.570462931
Aradu.XD25G	-1.59946207	-1.32192809	-0.26881676	-1.28630419	-1.43440282
Aradu.KR6YX	0.263034406	0.411426246	0.333423734	-0.23446525	0.150559677

Aradu.ZM4HW	1.521050737	1.906890596	1.66448284	2.304511042	2.435628594
Aradu.L0YDF	0.941106311	0.815575429	0.757023247	0.782408565	1.761285273
Aradu.FZ0TC	2.451540833	2.839959587	2.735522177	2.516015147	2.531069493
Aradu.7Q4MS	0.23878686	1.286881148	0.084064265	0.992768431	1.111031312
Aradu.NP782	0.056583528	0.97819563	0.807354922	0.704871964	1.150559677
Aradu.RLX6T	1.807354922	2.298658316	2.017921908	0.176322773	1.384049807
Aradu.0LB5P	-0.04394335	0.773996325	0.111031312	4.512858954	4.715344295
Aradu.T3VDH	6.106013238	6.45795538	6.291124643	3.831877241	4.925999419
Aradu.L50NE	5.551823596	5.258518925	5.34020618	2.307428525	2.526068812
Aradu.A8ITS	-0.91593574	-0.97143085	-1.15200309	0.432959407	0.565597176
Aradu.C8SIT	2.618238656	3.065227623	2.50589093	3.626439137	3.763411574
Aradu.720N0	3.488000771	3.841973119	3.663344619	4.552745937	4.639810978
Aradu.0LP8B	0.847996907	0.545968369	0.687060688	-1	1.201633861
Aradu.CED81	-2.94341647	-3.32192809	-3.32192809	-3.47393119	-3.64385619
Aradu.AZC7U	4.666756592	4.755421735	4.390254956	5.596338864	5.757823336
Aradu.1Q100	1.150559677	1.941106311	1.641546029	2.080657663	1.989139007
Aradu.QRL86	2.298658316	3.032100843	2.592158002	3.424922088	3.324810603
Aradu.YF7CH	NA	NA	NA	NA	NA
Aradu.B45K6	1.333423734	1.883620816	1.516015147	2.528571319	3.030336078
Aradu.NS580	NA	NA	NA	NA	NA
Aradu.WLS5Y	0.475084883	0.765534746	0.35614381	0.90303827	2.392317423
Aradu.NW9B9	2.66448284	3.63343121	3.065227623	2.198494154	2.339137385

Aradu.N39PG	NA	-5.05889369	NA	-4.32192809	-1.68965988
Aradu.AU8EP	NA	NA	NA	NA	2.702657543
Aradu.C7X7Y	NA	NA	NA	NA	NA
Aradu.LNZ6T	-0.05889369	-0.07400058	-0.26881676	4.247168	4.253989266
Aradu.Z2UA0	0.933572638	1.310340121	0.748461233	3.385431037	3.606442228
Aradu.K6SHY	0.739848103	1.327687364	1.189033824	2.014355293	2.253989266
Aradu.8A5JB	4.496334513	4.492494152	4.46008724	3.746312766	4.141596278
Aradu.F433U	-3.05889369	-3.83650127	-4.64385619	-0.25153877	-1.35845397
Aradu.32TSS	-1.94341647	-1.78587519	-1.59946207	-3.05889369	-1.59946207
Aradu.X23MZ	2.675815931	2.580145484	2.414135533	2.545968369	3.0721058
Aradu.RM1RY	1.316145742	1.521050737	1.448900951	2.163498732	2.339137385
Aradu.TJM76	2.9202933	1.480265122	1.469885976	-4.05889369	-0.30400619
Aradu.YWE4J	-1	-0.51457317	-1.59946207	1.269033146	1.275007047
Aradu.ZC0SW	NA	NA	NA	NA	-5.64385619
Aradu.NBA3F	0.790772038	1.422233001	0.378511623	2.153805336	2.370164281
Aradu.A8RS9	NA	-5.64385619	-5.64385619	NA	-5.64385619
Aradu.T6VPT	0.310340121	1.298658316	1.263034406	1.207892852	1.989139007
Aradu.JFD4U	3.933572638	4.305240966	3.804260116	2.147306699	2.049630768
Aradu.MRQ6G	3.689299161	3.481557281	3.534808661	0.584962501	0.526068812
Aradu.6YC2Q	NA	NA	NA	NA	-6.64385619
Aradu.5K5P7	0.111031312	0.925999419	1.555816155	0.790772038	0.545968369
Aradu.IS5YT	8.525755693	8.410196923	8.578372691	5.089582893	5.181897643

Aradu.56ZVJ	3.070389328	3.653060017	3.008988783	4.838951767	4.757023247
Aradu.F7D9L	1.077242999	1.070389328	1.782408565	NA	-2.94341647
Aradu.FCK70	1.13093087	1.608809243	0.748461233	1.310340121	1.646162657
Aradu.P0XL9	3.363171077	3.64731451	3.234194723	2.448900951	2.996388746
Aradu.TX9D6	-1.47393119	-1.28630419	-1.68965988	-1.39592868	-1.18442457
Aradu.79PL2	-1.47393119	-1.12029423	-1.25153877	-0.577767	-0.71311885
Aradu.1T3XU	1.176322773	1.244887059	1.111031312	1.042644337	2.217230716
Aradu.TV3WI	-0.97143085	-0.64385619	-0.86249648	0.454175893	1.22650853
Aradu.P82M7	1.570462931	2.397802962	1.922197848	3.044394119	3.17951105
Aradu.ZI2XT	-3.32192809	-2.55639335	-3.64385619	-0.73696559	-0.62148838
Aradu.D42IC	0.941106311	1.056583528	1.097610797	2.214124805	2.469885976
Aradu.AMA0U	2.543495883	1.839959587	1.678071905	1.752748591	1.726831217
Aradu.BL1S9	-2	-1.32192809	-1.39592868	-0.71311885	-0.04394335
Aradu.N5A68	2.370164281	2.939226578	2.204766751	3.932628157	3.994579724
Aradu.D7MSN	2.64385619	2.464668267	2.475084883	1.541019153	1.541019153
Aradu.2X54C	-0.13606155	-0.78587519	-1	-2.18442457	-0.07400058
Aradu.ME00T	1.815575429	1.432959407	1.350497247	3.124328135	2.718087584
Aradu.3E5G3	-5.05889369	-5.05889369	-6.64385619	-2.39592868	1.195347598
Aradu.E4AIC	3.051372102	3.060047384	3.337711092	1.22650853	1.682573297
Aradu.LVF07	-0.47393119	1.03562391	-0.86249648	1.622930351	1.555816155
Aradu.INW8Z	3.13093087	3.237257771	3.060047384	3.6622055	3.921245889
Aradu.03IBL	-0.13606155	1.063502942	0.584962501	1	1

Aradu.WSH4V	0.40053793	-0.55639335	-0.83650127	0.815575429	1.459431619
Aradu.W87GJ	6.761019265	6.90303827	6.639955645	8.190367938	8.998731449
Aradu.N7TR4	-1.05889369	0.678071905	-1.78587519	-3.18442457	-3.47393119
Aradu.TJK3R	NA	NA	NA	NA	NA
Aradu.C114V	1.176322773	0.97819563	0.887525271	1.500802053	1.956056652
Aradu.VQJ7V	-2.64385619	-2.47393119	-2.18442457	-2.32192809	-2.55639335
Aradu.BA406	1.989139007	1.948600847	1.887525271	1.678071905	1.883620816
Aradu.Z101N	-0.49410907	-0.26881676	-0.94341647	0.831877241	1.545968369
Aradu.M1252	1.416839742	1.214124805	1.15704371	-2.64385619	-1.64385619
Aradu.A6VDY	1.726831217	1.5360529	1.250961574	0.831877241	1.480265122
Aradu.KX34B	1.275007047	1.333423734	1.063502942	1.063502942	1.608809243
Aradu.E2BAC	3.392317423	3.169925001	3.311793718	3.49441561	3.464668267
Aradu.76148	1.500802053	2.22650853	1.867896464	2.942983598	3.671293372
Aradu.WLD4B	-0.21759144	0.495695163	-0.62148838	2.5360529	2.545968369
Aradu.B3YY0	3.032100843	2.967168608	2.970853654	3.109360559	3.527320608
Aradu.68CEE	1.709290636	2.22342255	1.655351829	2.861955364	3.049630768
Aradu.ZL9WR	3.650764559	5.165911939	4.881664619	6.798309782	6.664198369
Aradu.R9XZF	3.472487771	3.871843649	3.592158002	3.419538892	3.64038956
Aradu.2K88G	6.866907978	6.586464526	6.570614722	4.92552477	4.735522177
Aradu.YRC2R	3.073820233	2.912649865	2.99095486	1.443606651	1.718087584
Aradu.C4XJQ	-3.47393119	-4.32192809	-2.73696559	-1.47393119	-2.94341647
Aradu.1Z4K3	2.438292852	2.927896454	2.767654798	3.90303827	4.053111336

Aradu.LWE3F	-0.49410907	-0.20091269	-0.39592868	1.480265122	1.280956314
Aradu.40JGL	2.137503524	2.241840184	2.201633861	0.367371066	0.659924558
Aradu.05H52	-1.08926734	-1.15200309	-1.51457317	-3.47393119	-2.18442457
Aradu.N69D4	2.757023247	2.580145484	2.693765712	3.703765179	3.837943242
Aradu.HL95H	1.084064265	1.111031312	1.250961574	1.150559677	1.700439718
Aradu.X1MH8	-0.88896869	0.014355293	-0.35845397	-1	0.333423734
Aradu.RB83Y	8.900232849	8.552400128	8.780539767	3.142413438	3.634593268
Aradu.PQF90	NA	-6.64385619	NA	-0.49410907	-0.62148838
Aradu.X2T4X	3.066950244	3.388189537	3.165107985	3.703765179	4.060047384
Aradu.8A22P	2.22342255	1.411426246	1.411426246	0.879705766	1.495695163
Aradu.W3KTW	0.545968369	-0.18442457	-0.35845397	-3.18442457	-2.32192809
Aradu.KAS7C	0.495695163	1.244887059	1.35614381	1.111031312	2.414135533
Aradu.Z3R4T	4.20867332	4.510329019	3.867896464	5.553053253	5.600507645
Aradu.94ZD7	-4.64385619	-3.64385619	-5.64385619	-2.73696559	-4.05889369
Aradu.I72RE	-3.64385619	-5.64385619	NA	0.028569152	0.5360529
Aradu.RBF27	2.757023247	3.147306699	2.680324357	3.381283373	3.94204526
Aradu.K8EE3	3.651912745	3.635754391	3.465974465	3.976363636	4.084064265
Aradu.VZQ8X	3.404630684	3.486714373	3.641546029	3.578938713	3.727920455
Aradu.B92XB	0.807354922	1.097610797	0.815575429	2.392317423	2.229587923
Aradu.UYZ93	2.419538892	1.970853654	1.811471031	2.761285273	3.089159132
Aradu.6LZ7W	2.754887502	3.023255352	2.845991771	3.904965719	4.348374075
Aradu.Q451G	-0.86249648	-0.86249648	-1.64385619	3.145677455	3.415488271

Aradu.WB4HU	-3.83650127	-2.94341647	-2.64385619	-4.32192809	-3.18442457
Aradu.H802Y	0.201633861	0.765534746	-1.68965988	3.641546029	4.065227623
Aradu.I1SW1	2.010779839	2.117695043	2.211012193	0.98550043	1.63691458
Aradu.EQT8P	-0.26881676	0.333423734	-0.18442457	0.704871964	1.275007047
Aradu.5RE8S	0.98550043	1.427606173	1.014355293	2.283921772	2.641546029
Aradu.I7KI9	-4.32192809	-3.32192809	-5.64385619	-6.64385619	-4.64385619
Aradu.M4DGG	4.351910961	4.372952098	4.318316841	2.269033146	4.012568674
Aradu.UJ5WJ	NA	NA	NA	NA	-6.64385619
Aradu.ZL6EF	6.981396032	7.0061858	6.96474517	6.553821257	6.547357282
Aradu.UZ754	-1.32192809	-1.32192809	-1.55639335	-1.55639335	-0.47393119
Aradu.YQU8F	-0.86249648	0.298658316	-0.47393119	1.891419187	1.063502942
Aradu.674AT	NA	NA	NA	NA	NA
Aradu.J0689	-3.64385619	-4.32192809	-5.64385619	-1.59946207	-0.34007544
Aradu.PZ2KX	-2.12029423	-3.47393119	-4.32192809	2.572889668	3.447579197
Aradu.464FL	-0.15200309	0.275007047	-0.05889369	2.13422094	2.025028794
Aradu.71EUZ	1.448900951	1.773996325	1.632268215	0.40053793	0.613531653
Aradu.7Y15X	NA	-5.05889369	-6.64385619	-3.83650127	-0.59946207
Aradu.E8YAE	0.22650853	-0.83650127	-0.78587519	-1.18442457	-1
Aradu.62QCX	0.084064265	0.687060688	0.056583528	1.097610797	0.782408565
Aradu.X763N	NA	NA	NA	-5.64385619	-6.64385619
Aradu.W4XL4	2.508428653	2.976363636	2.533563348	2.596935142	2.671293372
Aradu.GN16C	2.970853654	3.303050085	3.107687869	2.925999419	3.727920455
Aradu.QD7T	-0.577767	0.545968369	0.084064265	2.286881148	2.480265122

L					
Aradu.XH3ZX	-1.25153877	-0.10469738	-2.12029423	1.879705766	2.577730931
Aradu.Q0PGE	2.516015147	1.049630768	2.060047384	NA	NA
Aradu.P1KDA	2.15704371	2.443606651	2.304511042	3.229587923	3.589763487
Aradu.ZS6PI	0.526068812	0.35614381	0.150559677	1.475084883	1.531069493
Aradu.C0HL8	4.59812696	4.749534268	4.619413011	5.178316272	4.95930649
Aradu.K82B1	1.292781749	0.815575429	0.847996907	3.220329955	2.720278465
Aradu.Z4QQP	NA	-5.64385619	-5.05889369	NA	-1.68965988
Aradu.I6GSC	NA	NA	NA	-3.64385619	-2.83650127
Aradu.UA8T0	0.495695163	0.895302621	0.526068812	0.839959587	0.790772038
Aradu.W6T3N	NA	NA	NA	NA	NA
Aradu.B8HUR	3.867896464	4.042644337	4.152994605	2.831877241	3.148934105
Aradu.V1L1H	1.286881148	1.469885976	1.257010618	1.189033824	1.875780063
Aradu.23D9S	-5.64385619	-5.05889369	-6.64385619	-3.83650127	-3.18442457
Aradu.VB3DF	5.056583528	5.727648222	5.64731451	5.03562391	4.547203025
Aradu.88KAK	-0.73696559	-1.51457317	-2.05889369	-4.64385619	0.378511623
Aradu.3CD7I	NA	-6.64385619	-5.64385619	-3.18442457	-2.55639335
Aradu.S7YD6	-0.23446525	3.028569152	1.952333566	0	1.13093087
Aradu.98FLM	NA	NA	NA	NA	NA
Aradu.R8CMR	3.460742564	3.35473424	3.026800059	4.100136671	4.363871925
Aradu.W5KRK	1.111031312	1.316145742	1.66448284	2.695993813	2.750606505
Aradu.BNK96	0.86393845	1.339137385	0.831877241	2.319039816	2.350497247
Aradu.TBQ8	-4.05889369	-3.64385619	-3.47393119	-0.78587519	-0.18442457

2					
Aradu.R9VGI	1.925999419	2.084064265	2.039138394	2.260025656	2.803227036
Aradu.87IV3	-0.37706965	1.545968369	1.244887059	1.03562391	1.422233001
Aradu.22X1P	-2.25153877	-1.94341647	-1.83650127	-0.07400058	4.679761574
Aradu.W7H9E	1.807354922	2.166715445	1.948600847	2.922197848	3.209453366
Aradu.UT1SE	NA	NA	NA	-3.47393119	-2.83650127
Aradu.0C27S	-2.55639335	-3.18442457	-1.83650127	-0.71311885	2.137503524
Aradu.MJ83N	-3.47393119	-5.05889369	-5.05889369	NA	-4.64385619
Aradu.31VER	1.298658316	1.769771739	1.646162657	2.22650853	2.477677328
Aradu.XIY30	1.220329955	1.700439718	1.448900951	2.204766751	2.411426246
Aradu.1I5UA	2.482848283	2.608809243	2.545968369	2.042644337	2.700439718
Aradu.1TX9B	NA	NA	NA	-5.05889369	-4.64385619
Aradu.P04DI	1.117695043	2.687060688	2.021479727	1.014355293	-2.83650127
Aradu.NZM5S	2.353323291	2.003602237	2.310340121	1.280956314	2.432959407
Aradu.WHS6B	-1.43440282	-1.32192809	-1.68965988	0.641546029	0.40053793
Aradu.SCR9A	5.12722055	5.148120631	5.316869805	3.682573297	3.516015147
Aradu.XM2MR	-2.73696559	-2.64385619	-2.73696559	1.641546029	-0.37706965
Aradu.Z31WB	4.556429415	5.025028794	4.498250868	6.873936206	7.67645078
Aradu.N8FJN	3.638073837	1.49057013	2.50589093	2.358958826	4.018812186
Aradu.GC5S7	3.781359714	4.148934105	3.770829046	4.780310099	5.13996057
Aradu.I2J7R	-1.55639335	-1.51457317	-2	-0.64385619	-1.08926734
Aradu.RWI1K	NA	-6.64385619	NA	-4.32192809	-2.47393119
Aradu.3X54	0.084064265	0.704871964	0.641546029	0.722466024	1.150559677

U					
Aradu.9Q1AJ	-1.32192809	1.042644337	0.495695163	-0.10469738	1.182692298
Aradu.NKB4V	-3.47393119	-6.64385619	-4.32192809	NA	-4.64385619
Aradu.22650	1.432959407	1.831877241	0.632268215	0.464668267	2.572889668
Aradu.A7CW2	2.176322773	2.89917563	2.641546029	4.031218731	3.882643049
Aradu.7X8FI	-3.32192809	-5.05889369	-5.05889369	-2.47393119	-2.64385619
Aradu.88UWV	-3.18442457	-1.55639335	-1.73696559	-0.78587519	-0.76121314
Aradu.JMT41	1.214124805	1.321928095	1.169925001	0.575312331	1.09085343
Aradu.H6JXR	1.163498732	1.35614381	1.201633861	0.333423734	-0.20091269
Aradu.VW5KP	-4.64385619	-6.64385619	-5.64385619	NA	-2.12029423
Aradu.JI62L	2.13422094	3.410069692	2.381283373	4.631686366	4.067810784
Aradu.F6LZ6	2.680324357	2.321928095	2.550900665	3.047887329	3.174725988
Aradu.AAH6J	0.545968369	0.443606651	0.464668267	0.807354922	0.765534746
Aradu.S5T1J	-2.18442457	-2.47393119	-2.32192809	-1.64385619	-0.71311885
Aradu.A8B4S	1.443606651	1.773996325	1.565597176	2.438292852	2.897240426
Aradu.Y7TD6	2.364572432	2.695993813	2.538538164	3.524815928	3.35614381
Aradu.8U0A6	NA	NA	NA	NA	NA
Aradu.4196P	1.333423734	2.533563348	1.163498732	3.23572706	3.129283017
Aradu.28PRF	4.138323004	3.933572638	3.92314918	1.839959587	2.56315813
Aradu.1D3EG	4.607034346	4.969012308	4.808385051	5.46139759	5.523561956
Aradu.HG75V	-4.64385619	-5.05889369	-5.05889369	-1.08926734	-0.577767
Aradu.88CDZ	0.678071905	1.070389328	0.526068812	0.925999419	0.855989697
Aradu.0VN2	-5.05889369	NA	-6.64385619	NA	2.217230716

L					
Aradu.BU1C4	NA	NA	-5.64385619	NA	0.378511623
Aradu.JY8JV	4.077242999	4.218781168	4.08236197	3.955126781	4.071247819
Aradu.16U3K	3.368768349	3.59454855	3.40053793	3.854993017	4.040015679
Aradu.8UZ9S	1.843983844	2.280956314	1.50589093	2.786596362	2.895302621
Aradu.FA1DK	1.937344392	2.163498732	1.996388746	1.82374936	2.257010618
Aradu.S9TS8	-1.15200309	0.765534746	0.084064265	-0.16812276	-0.62148838
Aradu.PBM8D	NA	NA	NA	NA	-2.47393119
Aradu.PRR6C	5.495375381	5.417852515	5.491532462	2.879705766	3.019701914
Aradu.FT1FE	NA	-6.64385619	-5.05889369	1.929790998	1.275007047
Aradu.3D1TM	3.857980995	4.677508242	3.726831217	5.06608919	5.149340669
Aradu.Y1Z8I	3.363171077	3.69265037	3.545968369	3.543495883	3.820689561
Aradu.8Y02T	0.389566812	0.739848103	0.584962501	1.622930351	1.944858446
Aradu.A4DEK	2.217230716	2.510961919	2.039138394	3.005399988	3.423578171
Aradu.AXB4D	1.959770155	2.263034406	2.073820233	2.887525271	2.94673086
Aradu.50KFD	NA	-6.64385619	NA	NA	-3.47393119
Aradu.MVM9Y	2.63691458	3.063502942	3.12763328	3.289834465	3.618238656
Aradu.CI3JS	11.0295012	10.36499914	10.9039722	6.789859641	7.12649799
Aradu.DJF16	1.678071905	2.336283388	1.641546029	3.294253136	3.407352751
Aradu.I625R	-0.21759144	0.378511623	-0.12029423	1.189033824	1.757023247
Aradu.DM3FR	0.757023247	1.269033146	1.269033146	0.831877241	1.063502942
Aradu.Y62C6	3.042644337	3.896271849	3.037382222	5.217618485	5.207892852
Aradu.J2RZ	0.790772038	1.469885976	1.378511623	2.077242999	2.61117238

P					
Aradu.SE3WE	1.604071324	1.443606651	1.835924074	-1.51457317	0.475084883
Aradu.SG5CY	-0.45403163	-0.4150375	-0.68965988	0.765534746	1.028569152
Aradu.CA6YX	-4.05889369	NA	-6.64385619	-5.05889369	-3.18442457
Aradu.L4JLT	1.545968369	1.933572638	1.316145742	2.587364991	2.655351829
Aradu.SS7YL	0.298658316	0.86393845	0.992768431	0.933572638	1.887525271
Aradu.ZJM8H	1.769771739	2.241840184	1.867896464	2.821710215	3.085764554
Aradu.E3I48	1.063502942	1.735522177	1.384049807	2.687060688	2.726831217
Aradu.ZR9ZC	NA	-3.32192809	-1.59946207	-4.05889369	-3.64385619
Aradu.GA7ZA	2.847996907	3.412781525	3.215678597	4.460742564	4.626439137
Aradu.CN7V5	-6.64385619	NA	NA	-4.64385619	-6.64385619
Aradu.A9NSU	NA	-3.05889369	-3.83650127	-2.05889369	-1.68965988
Aradu.I9RLC	5.134631671	4.8928767	4.639810978	4.571069998	5.008092421
Aradu.P709D	6.194165869	6.01368557	5.859472667	4.584361253	5.441284272
Aradu.PEP5T	4.387500406	4.065227623	4.039138394	2.217230716	2.347665656
Aradu.YYA59	-0.43440282	-0.577767	-3.05889369	2.639232163	3.054848477
Aradu.TC75Q	0.713695815	1.304511042	1.014355293	2.324810603	2.577730931
Aradu.9MA52	2.526068812	2.901108243	2.485426827	3.927896454	4.304511042
Aradu.NA9YP	-5.64385619	-6.64385619	-6.64385619	-3.47393119	-4.05889369
Aradu.H3LPD	3.266036894	3.220329955	2.827819025	4.045268215	4.638073837
Aradu.C6I2X	3.215678597	3.185866545	2.912649865	2.350497247	2.629939409
Aradu.GH2K1	-5.05889369	NA	NA	-5.05889369	NA
Aradu.04QL	0.757023247	1.367371066	1.070389328	2.12763328	2.23878686

M					
Aradu.5JX5D	-0.55639335	0.070389328	-0.53533173	0.310340121	0.695993813
Aradu.GX58B	1.475084883	0.879705766	0.90303827	1.827819025	2.046141782
Aradu.01TNR	NA	NA	-4.05889369	-3.18442457	-3.64385619
Aradu.TJ0ZU	-1.64385619	-0.13606155	-0.13606155	0.790772038	1.427606173
Aradu.R75XI	-2.55639335	-1.88896869	-2.18442457	0.176322773	0.956056652
Aradu.GN4F8	-0.49410907	-0.07400058	-0.86249648	2.114367025	2.558267634
Aradu.DGR2N	3.650764559	3.758089934	3.804260116	0.669026766	1.565597176
Aradu.L02C7	-1.51457317	-1.15200309	-1.94341647	-1	-0.78587519
Aradu.J8VZJ	NA	-5.64385619	-5.64385619	-6.64385619	-1.94341647
Aradu.NE81M	-0.28630419	0.757023247	0.613531653	1.918386234	2.137503524
Aradu.XE4CL	1.981852653	2.648465443	1.851998837	4.845490051	4.823239844
Aradu.E52Z4	2.671293372	3.007195501	2.845991771	4.135863165	4.292045492
Aradu.0K49H	2.17951105	2.220329955	2.100977648	2.671293372	2.625270489
Aradu.ZR5CT	-0.4150375	-0.18442457	-0.55639335	-1.39592868	-0.21759144
Aradu.767UQ	3.132576843	3.424922088	3.246408087	3.75595577	3.944858446
Aradu.CAM8W	-3.83650127	-2.25153877	-1.64385619	0.014355293	0.286881148
Aradu.RY6HH	-5.05889369	-5.05889369	-6.64385619	-0.76121314	-1.78587519
Aradu.Y017B	0.695993813	0.963474124	-0.34007544	3.231125158	2.925999419
Aradu.MQ8BQ	1.378511623	1.49057013	1.613531653	3.689299161	3.762348816
Aradu.MU261	0.807354922	1.070389328	0.687060688	1.575312331	1.963474124
Aradu.1C5Z6	2.956056652	2.996388746	3.715893371	2.763411574	3.003602237
Aradu.RJA7	3.9800253	3.451540833	3.472487771	2.232660757	2.659924558

P					
Aradu.YX18B	2.769771739	3.12763328	2.918386234	3.913607512	4.114367025
Aradu.B0MFP	0.137503524	1.111031312	0.344828497	1.021479727	0.790772038
Aradu.QVF0N	-4.32192809	0.422233001	1.50589093	-2.83650127	0.956056652
Aradu.BAA8F	4.032100843	4.245647774	4.043519494	4.815063017	4.818646083
Aradu.GT6LC	1.989139007	2.147306699	1.98550043	3.298658316	3.892391026
Aradu.9ND07	2.935459748	3.150559677	3.169925001	2.339137385	2.653060017
Aradu.W7HND	4.764473551	4.933100475	4.673556424	2.72900887	3.370164281
Aradu.Q9REZ	2.22342255	3.27351589	1.485426827	4.784503983	4.82527683
Aradu.D0NVI	-3.83650127	-2.55639335	-3.83650127	-2.64385619	-3.05889369
Aradu.SC4Y7	2.847996907	3.107687869	2.914564523	2.871843649	2.56315813
Aradu.M0JAP	2.336283388	2.639232163	2.381283373	3.418189948	3.489286023
Aradu.B3QR0	NA	NA	NA	NA	NA
Aradu.1TF3W	-6.64385619	NA	NA	NA	0.111031312
Aradu.669IL	2.070389328	2.691534165	2.077242999	3.298658316	3.258518925
Aradu.LH845	2.948600847	3.001802243	2.948600847	2.198494154	2.565597176
Aradu.QED65	1.070389328	3.690417096	2.702657543	2.879705766	2.63691458
Aradu.B4GBB	3.240314329	3.381283373	3.721372659	-0.21759144	0.321928095
Aradu.U3B0I	4.157852169	4.151371776	4.089159132	3.27351589	3.22342255
Aradu.FEU0W	-1.35845397	-0.91593574	-1.12029423	-1.94341647	-1.55639335
Aradu.V1MDS	-3.64385619	-4.32192809	-5.05889369	NA	-5.05889369
Aradu.D8A1M	3.907852072	3.791814071	3.968090752	2.459431619	2.5360529
Aradu.3GX3	0.22650853	0.23878686	-0.08926734	0.604071324	0.855989697

5

Aradu.46J5J	-0.4150375	-0.91593574	-1.55639335	-2.25153877	-1.59946207
Aradu.665TV	1.516015147	2.073820233	1.007195501	4.258518925	4.857483428
Aradu.HJZ2Z	-0.91593574	-1.78587519	-0.91593574	-1.28630419	-0.59946207
Aradu.KH1Y4	1.731183242	2.241840184	1.773996325	2.646162657	2.912649865
Aradu.S0NBF	NA	NA	NA	NA	NA
Aradu.0S3RJ	NA	NA	NA	NA	-6.64385619
Aradu.V4L4B	0.59454855	1.063502942	0.669026766	2.503348735	2.475084883
Aradu.G1YNF	2.691534165	2.22342255	2.548436625	0.084064265	0.757023247
Aradu.KA3UP	0.839959587	0.613531653	0.831877241	1.384049807	1.704871964
Aradu.P6UBG	1.925999419	1.627606838	1.811471031	-0.35845397	1.650764559
Aradu.7JU28	6.657354182	5.866907978	6.335032987	-1.51457317	0.925999419
Aradu.Z5G5W	1.169925001	0.575312331	0.722466024	-2.05889369	-1.12029423
Aradu.AA7LT	-1.02914635	-1	-0.78587519	0.298658316	0.378511623
Aradu.UF1FY	1.263034406	1.910732662	1.790772038	0.275007047	-0.37706965
Aradu.8U64A	NA	-6.64385619	-3.64385619	-4.32192809	2.440952198
Aradu.KLS6P	0.263034406	0.298658316	0.250961574	0.526068812	0.50589093
Aradu.Y1RZK	1.292781749	1.722466024	1.310340121	-0.05889369	1.339137385
Aradu.0EM6K	1.269033146	1.298658316	0.773996325	2.070389328	2.883620816
Aradu.1UD93	2.901108243	3.56315813	2.873813198	3.865918815	4.158660175
Aradu.K52XK	2.049630768	2.163498732	1.3950628	3.707082992	4.806839582
Aradu.EHC6P	3.23878686	3.063502942	2.857980995	3.496973581	3.473786912
Aradu.Y66C	1.855989697	2.247927513	2.22650853	2.166715445	2.5360529

W					
Aradu.WH49R	-0.35845397	0.879705766	0.765534746	0.704871964	1.007195501
Aradu.L2TEA	0.86393845	1.843983844	1.641546029	3.35473424	4.08236197
Aradu.C5AKB	0.124328135	0.422233001	0	0.687060688	0.163498732
Aradu.BJ55N	4.268284667	4.642701572	4.367371066	5.22650853	5.322288724
Aradu.Y92M0	3.557042415	3.468583317	3.553360503	3.534808661	3.777156666
Aradu.LDI0R	4.725195817	4.980939266	4.644433152	5.501439145	5.266786541
Aradu.MIW9U	1.40053793	1.713695815	1.292781749	0.887525271	1.286881148
Aradu.Q9Y83	2.689299161	3.791814071	3.557042415	4.137503524	4.277240898
Aradu.R4V51	-5.05889369	-3.83650127	-0.47393119	-2.47393119	-1.18442457
Aradu.DB13D	-1.94341647	-1.35845397	-1.94341647	-0.577767	-0.35845397
Aradu.H6T1L	0.59454855	1	0.739848103	0.847996907	1.521050737
Aradu.JRV68	-0.64385619	-0.53533173	-0.43440282	-0.18442457	0.056583528
Aradu.ZPL5X	-1.83650127	-1.64385619	-2.83650127	1.411426246	2.028569152
Aradu.L30M6	-0.68965988	-2.83650127	-2.73696559	NA	-3.32192809
Aradu.T35UC	3.582556003	4.124328135	3.364572432	4.14404637	4.301587647
Aradu.IIT7E	0.839959587	1.5360529	1.169925001	1.811471031	2.972692654
Aradu.68MCI	1.422233001	1.056583528	1.007195501	-2.39592868	2.028569152
Aradu.HA4YC	-2.83650127	-3.64385619	-3.32192809	-3.64385619	-2.25153877
Aradu.2UI08	2.803227036	4.454833907	4.119356177	2.629939409	3.046141782
Aradu.8A403	3.54225805	3.632268215	3.473786912	3.691534165	3.56193706
Aradu.MV2TG	2.49057013	2.809414444	2.659924558	2.66448284	3.047887329
Aradu.UHQ4	6.073606041	6.040673293	6.399342019	3.657640005	3.843983844

T					
Aradu.Q49PC	3.173127433	2.731183242	2.983677695	0.454175893	2.003602237
Aradu.SP99D	1.526068812	1.316145742	1.316145742	1.469885976	1.963474124
Aradu.Q6DD2	2.324810603	3.357552005	3.053111336	4.480265122	3.793895883
Aradu.A5EC7	4.289834465	3.927896454	4.093391153	3.078951341	3.846994687
Aradu.AN6JJ	6.130724991	6.273142859	5.683696454	4.158660175	4.178714641
Aradu.38ZXG	0.333423734	0.739848103	-0.10469738	1.879705766	1.918386234
Aradu.3SS5K	0.622930351	1.835924074	0.956056652	3.087462841	3.182692298
Aradu.9H3L9	1.925999419	2.304511042	2.063502942	2.361768359	2.548436625
Aradu.8P3KF	-1.02914635	-0.59946207	-0.81096618	0.545968369	1.028569152
Aradu.V4S8W	0.389566812	1.042644337	1.182692298	2.350497247	2.857980995
Aradu.N47AG	-6.64385619	-5.64385619	-6.64385619	-5.64385619	NA
Aradu.58JJ3	0.847996907	0.970853654	0.214124805	-1.39592868	0.163498732
Aradu.206YZ	2.361768359	2.871843649	2.722466024	2.622930351	3.158660175
Aradu.F8T28	2.077242999	2.503348735	2.321928095	2.528571319	2.891419187
Aradu.8482Y	-1.43440282	-0.53533173	-1.78587519	0.22650853	-0.26881676
Aradu.J9ALL	-1.25153877	-0.91593574	-1.15200309	0.250961574	0.411426246
Aradu.FE1BY	NA	NA	-6.64385619	NA	NA
Aradu.7F8A1	1.13093087	1.608809243	1.427606173	3.688180359	3.292781749
Aradu.14TMT	-0.37706965	1.189033824	0.097610797	1.867896464	1.622930351
Aradu.1LM0G	NA	NA	NA	NA	NA
Aradu.J21EA	3.166715445	4.198494154	4.068670811	5.790511412	5.723558561
Aradu.X1Y61	2.166715445	3.08236197	2.765534746	1.339137385	2.003602237

Aradu.70WHV	0.555816155	-0.12029423	-0.73696559	1.906890596	1.855989697
Aradu.AAN3U	1.321928095	0.678071905	1.214124805	-3.83650127	-3.83650127
Aradu.S89VI	2.794935663	2.653060017	2.869871406	2.488000771	2.675815931
Aradu.R0Z7V	2.914564523	3.29130886	2.825785627	3.435628594	3.910732662
Aradu.FJQ80	-0.43440282	-0.30400619	-0.71311885	1.350497247	1.137503524
Aradu.EL0FF	1.646162657	2.080657663	0.948600847	3.64385619	3.914564523
Aradu.7YJ00	2.485426827	2.987320866	2.666756592	3.454175893	3.625270489
Aradu.8M51F	1.189033824	2.028569152	1.580145484	1.922197848	2.358958826
Aradu.H1R88	0.367371066	0.321928095	0.070389328	1.367371066	1.304511042
Aradu.EX904	-2	-1.32192809	-2.47393119	1.469885976	1.659924558
Aradu.8P6BN	2.269033146	2.545968369	1.98550043	4.283921772	4.50779464
Aradu.W2IGQ	-3.83650127	-4.64385619	-4.32192809	1.815575429	0.650764559
Aradu.ZG6C0	5.211012193	5.024585638	5.454175893	3.439623138	4.062639828
Aradu.KL6JZ	1.042644337	1.3950628	1.250961574	2.269033146	2.070389328
Aradu.SJ2KY	-0.97143085	-1	-0.78587519	-1.73696559	-1.25153877
Aradu.9P6VM	3.286881148	3.657640005	3.6622055	2.897240426	3.27351589
Aradu.ISD6F	-1.94341647	-0.53533173	-1.08926734	0.956056652	1.042644337
Aradu.JG252	6.205939832	6.56285296	5.942514505	7.256067125	7.467931546
Aradu.EMN7F	0.963474124	1.941106311	1.761285273	2.821710215	3.17951105
Aradu.Z7FSM	0.389566812	0.871843649	0.333423734	1.786596362	1.827819025
Aradu.6LK51	2.865918815	2.582556003	2.650764559	3.181102551	3.412781525
Aradu.857YF	0.464668267	1.14404637	0.565597176	0.650764559	0.748461233

Aradu.7X679	3.669026766	3.367371066	3.635754391	3.783456654	4.46727948
Aradu.0YR51	-1.47393119	-0.73696559	-1.47393119	-6.64385619	-2.32192809
Aradu.2JA77	-0.35845397	-0.34007544	-0.13606155	0	0.485426827
Aradu.D4D16	2.477677328	2.269033146	2.392317423	-0.47393119	-0.01449957
Aradu.SMW23	-0.30400619	0.584962501	0.084064265	2.17951105	2.10433666
Aradu.N1YRP	-0.25153877	0.411426246	-0.28630419	3.361768359	3.390942773
Aradu.XI961	3.512226887	3.689299161	3.396433531	2.44625623	2.397802962
Aradu.PU6SR	2.408711861	2.972692654	2.604071324	3.288358562	3.297191417
Aradu.NK3YY	0.201633861	1.163498732	1.070389328	1.963474124	2.715893371
Aradu.Q0ZWM	-3.32192809	-1.73696559	-1.32192809	2.09085343	2.90303827
Aradu.MEY8C	2.298658316	2.389566812	1.981852653	3.7322692	3.343407822
Aradu.6BK0V	1.761285273	2.121015401	1.918386234	2.548436625	3.161887682
Aradu.C19JT	0.014355293	1.906890596	1.207892852	2.548436625	2.247927513
Aradu.I1DFV	4.532940288	4.632268215	4.691534165	5.028569152	5.057016965
Aradu.M4EHD	2.307428525	5.214124805	4.432291338	1.992768431	3.039138394
Aradu.MX9DT	-3.47393119	-3.05889369	-1.94341647	1.042644337	0.124328135
Aradu.22AJD	4.514753498	4.454833907	4.531693361	0.367371066	1.111031312
Aradu.RAZ0G	1.967168608	2.319039816	2.185866545	2.825785627	3.054848477
Aradu.S6366	-1.68965988	-3.47393119	-3.32192809	NA	NA
Aradu.ZUY8A	NA	-6.64385619	-5.05889369	-5.64385619	-4.64385619
Aradu.BI3QN	1.063502942	1.526068812	1.655351829	2.114367025	1.970853654
Aradu.MDC6	NA	-5.05889369	NA	-3.83650127	-4.64385619

5					
Aradu.79Z90	0.632268215	1.531069493	0.815575429	2.543495883	2.389566812
Aradu.199G1	-0.02914635	-0.81096618	-0.12029423	-0.13606155	-1.28630419
Aradu.751D4	-1.32192809	-4.64385619	-0.68965988	NA	-5.64385619
Aradu.J51N4	-0.34007544	-0.55639335	-0.53533173	1.855989697	1.344828497
Aradu.SXL5T	-2.83650127	-2.64385619	-4.32192809	-0.39592868	-0.12029423
Aradu.NHB6E	1.275007047	1.097610797	1.042644337	-0.25153877	0.799087306
Aradu.0KX1J	0.765534746	0.298658316	0.056583528	0.50589093	0.641546029
Aradu.ZG8VV	0.070389328	0.584962501	0.097610797	1.82374936	1.718087584
Aradu.30TWS	1.220329955	1.5360529	1.097610797	3.365972428	3.434294618
Aradu.9K72K	0.695993813	0.695993813	0.201633861	1.263034406	1.367371066
Aradu.97C3D	-4.05889369	-3.83650127	-4.05889369	-4.05889369	-4.32192809
Aradu.95F2Q	2.304511042	2.493134922	2.606442228	1.922197848	2.295723025
Aradu.J4FDW	0.641546029	0.815575429	0.575312331	0.739848103	1.389566812
Aradu.T8TQR	2.021479727	2.46727948	2.241840184	2.839959587	3.171527106
Aradu.TM66X	-1.59946207	-0.97143085	-2.32192809	1.992768431	1.887525271
Aradu.Q4G7J	4.203201156	4.313971371	3.830863757	5.394719913	5.629939409
Aradu.XY3GQ	-1.39592868	-0.97143085	-2.12029423	-0.30400619	0.124328135
Aradu.P8M1S	5.656496371	6.283366213	5.958842675	5.34269696	5.5055734
Aradu.F3KB2	-4.64385619	-0.83650127	-1.88896869	-1	1.10433666
Aradu.HU94F	2.50589093	3.458119481	3.155425432	3.40053793	3.311793718
Aradu.CU9HL	2.935459748	2.855989697	2.765534746	3.435628594	3.676944359
Aradu.D11T	2.432959407	2.459431619	1.918386234	1.03562391	2.448900951

E					
Aradu.R17B3	1.803227036	1.475084883	1.13093087	1	1.22650853
Aradu.8IQ81	-0.08926734	-0.20091269	-0.39592868	-3.05889369	-3.64385619
Aradu.Z030H	-1	-0.15200309	-0.64385619	-0.08926734	1.378511623
Aradu.CLY5P	-0.45403163	-0.18442457	-0.73696559	1.310340121	1.475084883
Aradu.FWZ0U	-4.32192809	-4.05889369	NA	NA	3.198494154
Aradu.95XXR	2.786596362	2.924099886	2.837943242	2.217230716	2.837943242
Aradu.Y7A6L	-4.64385619	-5.05889369	-6.64385619	NA	-1.88896869
Aradu.2Z4NE	NA	NA	NA	NA	NA
Aradu.D3RA8	3.634593268	3.250961574	3.513490746	4.37920506	4.558879853
Aradu.XX5Y6	2.301587647	2.10433666	2.989139007	3.212569339	2.10433666
Aradu.725QH	0.895302621	1.744161096	1.443606651	2.767654798	2.769771739
Aradu.4D16J	2.678071905	2.653060017	2.869871406	3.396433531	3.010779839
Aradu.UDC1Q	2.066950244	2.324810603	2.370164281	1.3950628	2.411426246
Aradu.A7934	-1.68965988	-3.18442457	-2.73696559	0.411426246	-0.35845397
Aradu.F9JAF	1.799087306	2.545968369	2.010779839	3.727920455	3.527320608
Aradu.FL9HS	NA	NA	-6.64385619	NA	-6.64385619
Aradu.YQ9IH	0.622930351	0.831877241	0.555816155	0.163498732	0.731183242
Aradu.ZD5DD	2.160274831	2.416839742	2.077242999	2.952333566	3.19061486
Aradu.Y12MD	3.484138131	3.416839742	3.310340121	3.673556424	3.759155834
Aradu.T9X3T	-0.02914635	0.422233001	0.070389328	0.678071905	0.632268215
Aradu.GR7RL	-1.68965988	NA	-4.05889369	NA	NA
Aradu.WB9G	3.622930351	3.82984956	4.088311236	1.500802053	2.087462841

S					
Aradu.MVW70	1.422233001	1.910732662	1.589763487	2.715893371	3.016139703
Aradu.K18SI	3.464668267	4.186659017	3.150559677	2.375734539	2.292781749
Aradu.SG4XX	NA	NA	-5.64385619	-3.18442457	-2.18442457
Aradu.YG3U8	0.22650853	1.201633861	0.443606651	3.260025656	3.538538164
Aradu.ND06J	-2.94341647	-3.64385619	-4.05889369	-2.55639335	-0.91593574
Aradu.URD4R	2.606442228	3.021479727	3.308885057	2.673556424	3.344828497
Aradu.T7CCI	-0.4150375	0.565597176	-2.12029423	1.906890596	2.541019153
Aradu.7P7FQ	NA	-4.64385619	NA	NA	-6.64385619
Aradu.8R2K8	3.484138131	3.604071324	3.595742339	3.971773447	4.010779839
Aradu.RQ0GF	0.176322773	0.389566812	-0.30400619	2.021479727	1.989139007
Aradu.TZS3T	3.624100895	3.559491813	2.98550043	2.295723025	2.046141782
Aradu.0KG04	NA	-6.64385619	-5.64385619	-4.32192809	-2.39592868
Aradu.1TB02	1.565597176	1.485426827	1.263034406	2.577730931	2.711494907
Aradu.CC8V7	2.746312766	2.912649865	2.731183242	3.529820947	3.754887502
Aradu.1LQ1Q	NA	NA	NA	-4.64385619	-5.64385619
Aradu.1QU40	2.063502942	2.214124805	1.887525271	3.304511042	3.329123596
Aradu.7GQ9E	8.389910925	7.835797782	8.117695043	3.782408565	3.865918815
Aradu.NBA8B	1.589763487	3.054848477	3.040892431	4.368768349	4.244887059
Aradu.EL4CE	3.249445341	3.196921734	3.023255352	2.432959407	2.528571319
Aradu.X3R2Q	-1.39592868	-0.88896869	-1.51457317	-0.01449957	0.124328135
Aradu.PW3YK	3.012568674	3.326249701	3.385431037	3.087462841	3.911691582
Aradu.6896	-2.12029423	-1.94341647	-2.05889369	-2.18442457	-1.35845397

W					
Aradu.8R77K	2.09085343	2.650764559	2.615887074	2.825785627	3.046141782
Aradu.G3TP5	-4.05889369	-6.64385619	NA	NA	-4.64385619
Aradu.IRQ7V	3.044394119	3.427606173	3.292781749	3.357552005	3.244887059
Aradu.AZF5J	-5.05889369	NA	-5.05889369	-3.05889369	-2.94341647
Aradu.7G8QU	0.695993813	0.485426827	0.50589093	1.214124805	1.485426827
Aradu.VRW76	-0.81096618	-0.43440282	-0.45403163	-0.45403163	-0.20091269
Aradu.4FA0W	4.994127114	5.119356177	5.126807703	5.810957159	6.243935602
Aradu.BZU3P	NA	-6.64385619	NA	-0.43440282	1.464668267
Aradu.545W2	-3.18442457	-3.47393119	-3.83650127	-5.05889369	-2.73696559
Aradu.MW8ED	2.794935663	3.051372102	2.811471031	3.370164281	3.646162657
Aradu.1PQ4F	0.176322773	0.59454855	0.669026766	0.22650853	1.613531653
Aradu.6W1XZ	3.122672719	3.770829046	3.411426246	4.772413555	4.685379552
Aradu.JAT8P	NA	NA	NA	NA	NA
Aradu.ZD7QJ	5.088311236	4.808385051	4.957914599	3.99004722	4.325530332
Aradu.V6UVG	5.64038956	5.533874777	5.146084939	3.334854269	5.116031993
Aradu.XV773	NA	NA	NA	NA	-5.64385619
Aradu.V1INC	NA	-4.64385619	-6.64385619	-1.35845397	-0.86249648
Aradu.HAF4V	3.875780063	4.538538164	2.742006211	5.325530332	5.499527024
Aradu.GFT3R	NA	-6.64385619	NA	NA	1.150559677
Aradu.1U41E	0.070389328	0.5360529	0.189033824	0.411426246	0.286881148
Aradu.L71CJ	-1.83650127	-2.47393119	-3.83650127	-0.94341647	0.111031312
Aradu.VZ8X	-2	-0.78587519	-1.25153877	-1.78587519	-2.55639335

R					
Aradu.FX57K	-3.64385619	-4.32192809	-6.64385619	-5.64385619	4.001802243
Aradu.CSN5D	1.521050737	1.372952098	1.646162657	2.087462841	2.837943242
Aradu.Y31BQ	4.195347598	4.092545742	3.846994687	3.948600847	4.708187236
Aradu.P4874	3.09592442	4.116863758	3.873813198	4.423578171	4.391630262
Aradu.Q8MCV	0.879705766	0.622930351	1.163498732	-2.32192809	-3.32192809
Aradu.1I7E9	NA	NA	-6.64385619	NA	NA
Aradu.QQX3T	1.263034406	1.448900951	1.09085343	1.361768359	1.726831217
Aradu.YMZ5A	3.486714373	3.512226887	3.519793486	2.782408565	3.147306699
Aradu.KBD84	3.776103988	4.218781168	4.322649262	4.727920455	4.43162296
Aradu.M2NRW	7.032762074	7.42315794	7.354469792	3.198494154	6.200457271
Aradu.CCC04	NA	-6.64385619	NA	-2.83650127	-3.18442457
Aradu.MC57M	3.63691458	3.694880193	3.66106548	1.361768359	1.316145742
Aradu.EX52R	1.195347598	1.86393845	1.655351829	2.475084883	2.904965719
Aradu.X6DIS	-5.64385619	-4.05889369	-3.47393119	NA	-2.55639335
Aradu.EJ09V	3.016139703	3.537296067	3.244887059	4.542877099	4.702103407
Aradu.B240I	1.292781749	1.516015147	1.350497247	1.575312331	2.214124805
Aradu.SM8ND	-1.64385619	-1.55639335	-1.88896869	-1.55639335	-1.68965988
Aradu.82TF8	4.059182199	4.258518925	3.821710215	5.364922559	5.496654083
Aradu.CBZ5I	3.140778656	3.361768359	3.263034406	4.467931546	4.42693562
Aradu.G5F0C	2.44625623	2.837943242	2.257010618	3.346247774	3.57167681
Aradu.QZ3JB	2.495695163	3.016139703	2.61117238	3.656496371	3.83491343
Aradu.7A20D	NA	NA	NA	NA	NA

Aradu.99PKX	NA	NA	NA	NA	NA
Aradu.Z7MCS	3.99004722	3.307428525	3.40053793	0.150559677	1.111031312
Aradu.4IL2F	-4.32192809	-1.59946207	-1.94341647	-5.05889369	-1.08926734
Aradu.VUW7M	-4.05889369	-4.05889369	-5.05889369	-6.64385619	-5.05889369
Aradu.B90GQ	-0.71311885	2.731183242	2.232660757	-2.12029423	-0.97143085
Aradu.934D7	-0.55639335	-0.35845397	-0.76121314	0.50589093	0.565597176
Aradu.BIZ8F	2.087462841	2.166715445	1.570462931	1.250961574	1.475084883
Aradu.CRB6R	3.439623138	3.555816155	3.436961338	4.856487779	5.010779839
Aradu.67X2R	-0.47393119	-0.01449957	-0.02914635	2.704871964	2.809414444
Aradu.SN5BS	-6.64385619	-4.64385619	-6.64385619	-4.32192809	-3.47393119
Aradu.G8ESS	4.219555769	4.689858236	3.917431755	5.684818738	5.573192724
Aradu.3KC68	5.276124405	5.371907298	5.618238656	2.956056652	3.789729251
Aradu.C08W6	2.543495883	2.931683057	2.715893371	4.112700133	4.384740587
Aradu.A2MQ0	2.794935663	2.9202933	2.788685711	3.434294618	3.651912745
Aradu.J502L	-0.01449957	1.726831217	2.295723025	1.98550043	1.137503524
Aradu.1R3I6	NA	NA	NA	NA	NA
Aradu.LA26X	2.23878686	2.950468414	2.650764559	2.855989697	3.119356177
Aradu.J807R	2.49057013	3.192194165	2.695993813	3.959770155	4.044394119
Aradu.6IB2A	1.13093087	1.682573297	1.280956314	2.983677695	3.163498732
Aradu.22RTM	2.895302621	3.321928095	2.952333566	1.831877241	1.98550043
Aradu.IY69R	7.440620048	6.653490009	6.70763522	4.682011391	4.59454855
Aradu.65QW9	2.711494907	3.244887059	3.033863452	4.08491466	4.718635616

Aradu.GD6AN	-2.47393119	0.367371066	-1.18442457	-0.66657627	-0.39592868
Aradu.BP0GU	0.722466024	2.169925001	1.275007047	1.682573297	1.049630768
Aradu.6A2D1	1.292781749	1.761285273	1.275007047	2.72900887	3.026800059
Aradu.Y2Z3L	NA	NA	NA	NA	NA
Aradu.0QA18	NA	-5.64385619	NA	0.669026766	0.895302621
Aradu.I29MY	4.052241981	4.596935142	3.320484678	6.848748115	7.522228415
Aradu.P05N1	1.485426827	1.974529312	1.59454855	3.016139703	3.323370069
Aradu.RMG12	-1.55639335	0.687060688	0.275007047	-0.62148838	0.485426827
Aradu.1QU0K	3.566815154	3.327687364	3.117695043	2.272023189	2.327687364
Aradu.A24RB	-0.43440282	-2.39592868	-0.86249648	1.843983844	0.5360529
Aradu.47LJL	NA	NA	-6.64385619	NA	-4.64385619
Aradu.T6HF1	NA	-5.64385619	-5.64385619	-2.25153877	-1.78587519
Aradu.0NY83	-2.05889369	-1.73696559	-2.25153877	0.344828497	0.641546029
Aradu.H4MY7	6.076601849	6.3172317	6.332528928	6.547974148	6.740522858
Aradu.H102I	-0.02914635	0.310340121	0.163498732	0.632268215	0.632268215
Aradu.T9WAY	1.195347598	1.799087306	1.480265122	1.678071905	2.111031312
Aradu.L8SVN	-1.88896869	1.176322773	2.3950628	-1.12029423	-2.64385619
Aradu.G2TPD	NA	-6.64385619	-4.32192809	-3.64385619	-2.18442457
Aradu.EWZ0P	NA	NA	NA	NA	NA
Aradu.B2A4G	1.201633861	1.232660757	1.15704371	1.275007047	1.555816155
Aradu.4XP3R	4.241077458	4.450881315	4.401220862	4.11353382	3.860962798
Aradu.M3LQ3	4.297191417	3.882643049	3.822730148	2.025028794	3.241840184

Aradu.HJ002	0.545968369	-0.10469738	0.40053793	-2.39592868	2.341985747
Aradu.V8XMS	-3.32192809	-5.05889369	NA	-3.83650127	-0.78587519
Aradu.S7WZG	-4.64385619	-1.02914635	-1.83650127	0.765534746	-0.26881676
Aradu.BS31B	1.992768431	2.313245852	2.17951105	2.845991771	3.112700133
Aradu.9C9UT	0.422233001	0	-0.26881676	2.889473543	2.472487771
Aradu.3K2CT	NA	-4.32192809	-5.05889369	-3.83650127	-2.64385619
Aradu.ZJ4KX	1.35614381	1.40599236	1.232660757	1.974529312	2.513490746
Aradu.X88X0	3.757023247	3.919340082	3.56193706	4.422233001	4.602884409
Aradu.QK85I	4.294253136	3.761285273	4.121844298	0.847996907	1.310340121
Aradu.M15HC	0.35614381	0.773996325	0.613531653	-1.39592868	-1.12029423
Aradu.AQB31	NA	NA	NA	NA	NA
Aradu.1X6Z1	1.748461233	-2.94341647	3.129283017	NA	NA
Aradu.00MP0	6.876025733	6.469885976	6.717676423	1.070389328	2.650764559
Aradu.UT3S4	NA	-5.64385619	NA	NA	NA
Aradu.EPY5P	0.925999419	2.553360503	1.063502942	3.761285273	3.40053793
Aradu.524PX	0.443606651	0.687060688	0.367371066	1.084064265	1.803227036
Aradu.YE6PU	-5.05889369	-5.64385619	NA	-5.05889369	-5.64385619
Aradu.Q5FHV	5.826548487	5.703765179	5.821455119	4.375039431	4.073820233
Aradu.X69MW	6.46139759	6.144454314	6.513964407	1.526068812	2.835924074
Aradu.AS0KA	-0.18442457	0.933572638	0.90303827	3.264536431	3.396433531
Aradu.W3H15	1.992768431	2.438292852	2.364572432	3.042644337	3.17951105
Aradu.P9JVV	3.388189537	3.231125158	3.253989266	2.817623258	2.780310099

Aradu.LT5WZ	-0.21759144	0.454175893	-0.55639335	1.867896464	2.3305584
Aradu.9RQ53	NA	-6.64385619	-6.64385619	NA	-2.32192809
Aradu.Z702T	NA	NA	NA	-3.18442457	-1.83650127
Aradu.R1S51	1.422233001	1.855989697	1.422233001	1.22650853	1.575312331
Aradu.RQK7Y	-3.83650127	-5.05889369	-6.64385619	NA	-6.64385619
Aradu.S37LQ	3.465974465	4.025028794	3.764473551	4.287620044	4.606442228
Aradu.7B3CD	0.748461233	1.815575429	-0.78587519	4.351910961	4.963474124
Aradu.1X6G8	-5.64385619	-4.32192809	-4.32192809	-0.78587519	0.956056652
Aradu.X1PQU	NA	NA	NA	NA	-4.32192809
Aradu.N25WS	1.555816155	3.060047384	3.13422094	2.682573297	2.618238656
Aradu.51WYZ	NA	-6.64385619	-5.05889369	-5.64385619	-1.68965988
Aradu.UI0N2	NA	NA	NA	NA	-6.64385619
Aradu.E0QTY	-3.83650127	-4.32192809	-5.64385619	-3.32192809	-2.55639335
Aradu.E9QIB	1.807354922	1.815575429	1.765534746	0.86393845	1.232660757
Aradu.A8AWS	0.584962501	1.070389328	0.925999419	-0.64385619	0.137503524
Aradu.63RCW	5.509062386	5.156639311	5.383013015	2.733354341	2.584962501
Aradu.F7Z5Y	0.887525271	1.967168608	1.263034406	2.700439718	2.639232163
Aradu.W7KRJ	0.454175893	0.5360529	0.150559677	-1.05889369	0.176322773
Aradu.677BP	-3.32192809	-1.88896869	-2.32192809	-1.47393119	-1.08926734
Aradu.E37Q4	3.327687364	3.43162296	3.23878686	4.286141873	4.246408087
Aradu.96DV9	4.465321514	4.441616269	4.061776198	2.459431619	2.961623328
Aradu.KJ1B9	-1.47393119	-2.32192809	-2.73696559	-2.55639335	2.757023247

Aradu.UQR72	5.569551851	5.686500527	5.438292852	4.617651119	4.871843649
Aradu.7C9V0	1.650764559	2.472487771	1.765534746	3.217230716	3.261530815
Aradu.3B8NW	-4.32192809	-2.73696559	-2.94341647	NA	-4.64385619
Aradu.Y4W78	2.924099886	3.241840184	3.336283388	4.005399988	4.100977648
Aradu.05WJ3	-2.32192809	-2.55639335	-2.47393119	-2.73696559	-0.53533173
Aradu.N0271	2.321928095	2.166715445	1.835924074	2.604071324	3.051372102
Aradu.B2STS	1.15704371	1.941106311	1.084064265	3.578938713	3.478971805
Aradu.Y9QIK	0.948600847	0.669026766	0.137503524	1.411426246	2.82984956
Aradu.IIC03	0.910732662	0.992768431	0.516015147	2.967168608	3.337711092
Aradu.M2IMN	2.555816155	2.189033824	2.3305584	2.570462931	2.543495883
Aradu.H5GIM	1.13093087	1.815575429	1.713695815	2.553360503	3.249445341
Aradu.J967R	-4.32192809	-3.18442457	-4.05889369	-2.83650127	-2.25153877
Aradu.JP5P4	NA	NA	NA	NA	NA
Aradu.4L72B	-2.39592868	-1.25153877	-2.39592868	-0.04394335	-0.12029423
Aradu.SM6NC	2.646162657	3.008988783	2.786596362	3.462052319	3.66106548
Aradu.P3YYJ	0.432959407	0.839959587	0.389566812	2.773996325	2.673556424
Aradu.V1TZX	4.339137385	3.736604875	3.915520901	1.761285273	2.615887074
Aradu.P2TIC	1.875780063	2.761285273	2.972692654	3.957914599	4.214124805
Aradu.B3CRQ	3.087462841	3.320484678	3.152183419	3.152183419	3.334854269
Aradu.EV2Z9	2.757023247	2.956056652	2.752748591	2.66448284	3.14404637
Aradu.LA0XT	0.90303827	1.124328135	0.831877241	0.855989697	1.263034406
Aradu.I05PE	2.914564523	3.107687869	2.935459748	3.840966704	3.992768431

Aradu.4XQ5D	-0.47393119	0.704871964	-0.10469738	1.895302621	1.803227036
Aradu.S7P2G	3.858975614	3.90014226	4.111865964	3.403267722	4.021479727
Aradu.L6GSR	-0.53533173	0.23878686	0.992768431	2.805292456	3.539779192
Aradu.2Z52F	-1.68965988	-4.32192809	NA	NA	-5.64385619
Aradu.CWT7S	1.608809243	1.851998837	1.500802053	2.765534746	2.967168608
Aradu.1573F	-0.91593574	-0.34007544	-0.81096618	-0.21759144	0.201633861
Aradu.Y97H0	-3.47393119	-1.55639335	-3.18442457	-4.32192809	-2.55639335
Aradu.1ED5Z	-1.25153877	-0.21759144	-1.83650127	3.042644337	3.364572432
Aradu.GRE2M	NA	NA	NA	NA	NA
Aradu.L3C1J	-0.62148838	-0.51457317	-0.78587519	0.298658316	0.35614381
Aradu.CD2E5	-2.47393119	-3.47393119	-3.83650127	-1.94341647	-2.05889369
Aradu.I9SPE	-0.71311885	-0.04394335	-0.39592868	1.22650853	1.464668267
Aradu.TQ146	2.855989697	3.033863452	2.963474124	3.381283373	3.295723025
Aradu.AY7XX	1.659924558	1.084064265	1.49057013	1.372952098	1.021479727
Aradu.B5JI0	0.575312331	0.028569152	-0.4150375	0.782408565	0.475084883
Aradu.S7CRC	-2	-1.73696559	-2.94341647	0.321928095	1.207892852
Aradu.31IUC	-1.28630419	-2.39592868	-2.18442457	-0.94341647	0.669026766
Aradu.QR8QC	NA	NA	NA	-5.64385619	NA
Aradu.JG217	0.90303827	1.13093087	0.669026766	1.933572638	2.185866545
Aradu.C37MP	4.250961574	3.9202933	3.986410935	3.241840184	3.653060017
Aradu.2V0TP	NA	NA	NA	NA	NA
Aradu.J6MLG	1.713695815	1.811471031	1.545968369	1.794935663	2.147306699

Aradu.N4IR4	-0.18442457	0.5360529	0.454175893	1.516015147	1.803227036
Aradu.QA44E	2.375734539	2.914564523	2.901108243	3.171527106	3.685940148
Aradu.18TLF	-2.94341647	-3.64385619	-4.05889369	-1.88896869	-2.64385619
Aradu.5E5UI	2.763411574	2.865918815	2.381283373	2.759155834	3.826802684
Aradu.Q4YIY	2.992768431	2.871843649	2.809414444	2.937344392	3.121015401
Aradu.I9JU3	0.422233001	0.422233001	-0.04394335	3.6622055	3.952333566
Aradu.XH0YF	4.088311236	4.270528942	4.044394119	2.744161096	3.100977648
Aradu.S6F46	NA	NA	-6.64385619	-4.05889369	-0.66657627
Aradu.Y82ZL	-0.02914635	-0.73696559	-0.02914635	-2.55639335	-1.78587519
Aradu.7M4Z2	0.82374936	2.403267722	1.232660757	3.62058641	3.462052319
Aradu.TJ8NB	NA	-4.64385619	NA	1.516015147	1.03562391
Aradu.KP13G	3.026800059	2.881664619	3.163498732	2.526068812	2.811471031
Aradu.TQ51J	0.411426246	0.50589093	0.111031312	0.669026766	0.790772038
Aradu.14ANW	NA	NA	NA	-5.64385619	0.641546029
Aradu.XIP04	NA	-5.64385619	-6.64385619	-4.32192809	-2.73696559
Aradu.SM8C1	-2.55639335	-1.25153877	-2.32192809	0.432959407	0.443606651
Aradu.898PR	5.929080835	5.732811872	5.439955517	6.807097275	7.171627179
Aradu.94UHF	0.925999419	0.641546029	0.687060688	2.063502942	2.448900951
Aradu.N636R	7.475165967	6.903761365	7.269407241	3.568032105	3.937344392
Aradu.Y64WA	2.9202933	2.969012308	2.861955364	2.861955364	3.427606173
Aradu.MN6GR	1.981852653	2.488000771	1.855989697	4.217230716	4.373648211
Aradu.G6GR7	-3.47393119	-4.64385619	-5.05889369	-5.64385619	-2.12029423

Aradu.L7CNH	NA	0.275007047	-2.05889369	-2.25153877	-4.05889369
Aradu.352P0	3.212569339	3.160274831	3.061776198	1.704871964	1.992768431
Aradu.6WC85	2.480265122	2.950468414	2.847996907	3.135863165	3.604071324
Aradu.6AL72	NA	NA	NA	NA	NA
Aradu.0G8DQ	0.565597176	0.782408565	0.422233001	1.111031312	1.411426246
Aradu.M4ZYN	2.117695043	2.046141782	1.765534746	-1.39592868	-1
Aradu.73L3W	0.464668267	0.918386234	0.584962501	1.82374936	2.09423607
Aradu.DW9XI	NA	-6.64385619	-4.64385619	-4.64385619	-3.18442457
Aradu.7Q4DG	NA	NA	NA	-5.05889369	-4.32192809
Aradu.Z3Z17	1.843983844	2.292781749	1.735522177	2.648465443	2.809414444
Aradu.62WGK	2.021479727	2.12763328	1.963474124	4.300123725	4.277984747
Aradu.M96PR	2.367371066	2.521050737	2.014355293	2.987320866	2.853995647
Aradu.DME0M	0.464668267	1.669026766	0.871843649	2.414135533	2.831877241
Aradu.SY356	2.613531653	3.046141782	3.153805336	4.275752049	4.13996057
Aradu.F77GY	2.891419187	3.493134922	3.032100843	3.304511042	3.588564737
Aradu.61W2G	-1.55639335	-2.94341647	-4.64385619	NA	-0.04394335
Aradu.TH5KX	2.114367025	2.327687364	2.09423607	3.085764554	3.201633861
Aradu.XYY0T	0.555816155	1.195347598	0.956056652	1.687060688	2.17951105
Aradu.N1NH0	1.739848103	2.124328135	1.815575429	1.851998837	2.301587647
Aradu.04EM8	3.379898164	3.145677455	3.27351589	2.22342255	2.523561956
Aradu.F00XS	4.038260575	3.361768359	3.114367025	1.150559677	0.839959587
Aradu.33DTZ	NA	-5.05889369	NA	-3.18442457	-2.25153877

Aradu.B8ABT	3.295723025	3.718087584	3.357552005	4.108524457	4.297191417
Aradu.0A17C	0.485426827	0.82374936	0.422233001	1.883620816	2.042644337
Aradu.24LGE	NA	-4.64385619	NA	-0.53533173	-0.88896869
Aradu.668QS	2.22650853	3.969933275	4.324810603	1.541019153	1.871843649
Aradu.AU181	1.176322773	1.778208576	1.66448284	2.063502942	2.063502942
Aradu.XJM2L	-6.64385619	-5.05889369	NA	-6.64385619	-6.64385619
Aradu.UK58V	4.157852169	3.639232163	3.385431037	2.956056652	3.787641414
Aradu.U8C21	0.028569152	-0.71311885	0.389566812	0.378511623	0.992768431
Aradu.B4D1B	NA	-4.05889369	NA	NA	NA
Aradu.1N8DM	1.967168608	1.761285273	1.937344392	2.843983844	3.385431037
Aradu.ZR64Z	0.545968369	0.5360529	0.378511623	-2.18442457	-0.21759144
Aradu.ATH33	3.070389328	2.56315813	2.752748591	1.333423734	1.948600847
Aradu.RW9W8	1.794935663	2.056583528	2.053111336	2.275007047	2.831877241
Aradu.N42M1	3.991861931	3.298658316	3.298658316	3.657640005	3.909773104
Aradu.V5LSS	3.581351247	3.051372102	3.67468662	0.86393845	2.333423734
Aradu.B3U8E	2.608809243	3.111031312	2.780310099	3.3305584	3.550900665
Aradu.Z4DIZ	-3.32192809	-4.32192809	-6.64385619	-2.39592868	-0.94341647
Aradu.AF0JE	-4.32192809	-5.64385619	-4.64385619	-1.68965988	-1.94341647
Aradu.RX5BN	0.124328135	0.963474124	0.275007047	2.336283388	2.430285273
Aradu.0Z7KI	NA	-4.05889369	-5.05889369	NA	-3.64385619
Aradu.UZS9C	1.726831217	2.482848283	2.14404637	3.129283017	3.339137385
Aradu.HKL0U	-2.64385619	-1.55639335	-1.73696559	1.014355293	1.40053793

Aradu.9YL2E	-4.64385619	-6.64385619	NA	NA	NA
Aradu.X5JSA	1.464668267	1.09085343	0.90303827	-0.45403163	0.432959407
Aradu.4T8J3	1.232660757	0.98550043	0.948600847	0	1.23878686
Aradu.L5Z6S	7.155425432	6.383013015	6.732540561	2.998195503	3.068670811
Aradu.86I8Y	4.655924213	5.720278465	6.150559677	6.214707673	5.624685811
Aradu.16GQU	6.631104282	7.158862106	6.305423389	8.238595812	8.016306878
Aradu.VI2RS	1.35614381	1.495695163	0.948600847	3.153805336	3.301587647
Aradu.51L6N	1.790772038	2.384049807	1.996388746	-0.08926734	-0.02914635
Aradu.BV95P	1.3950628	1.40053793	1.028569152	2.220329955	2.15704371
Aradu.FPC2C	-1	-2.55639335	-1.94341647	-1.55639335	-2.25153877
Aradu.0060C	3.301587647	2.464668267	2.250961574	-2.25153877	5.375734539
Aradu.UM1Y9	0.321928095	2.560714954	2.827819025	3.799087306	3.001802243
Aradu.BV67Y	-0.43440282	0.545968369	-0.43440282	2.62058641	2.95419631
Aradu.E9P6L	0.98550043	1.454175893	1.35614381	2.763411574	2.541019153
Aradu.PLL31	2.629939409	3.066950244	2.929790998	3.869871406	4.025914699
Aradu.YU6QF	0.941106311	1.22650853	1.15704371	2.22650853	2.124328135
Aradu.C99J0	1.361768359	1.659924558	1.422233001	1.13093087	2.782408565
Aradu.NRN2N	5.027684877	5.680605666	4.675251386	7.017921908	6.773073253
Aradu.U808N	3.19061486	3.568032105	3.46727948	4.198494154	4.48864354
Aradu.P9J09	2.40053793	2.485426827	2.117695043	0.263034406	0.895302621
Aradu.F84AW	-3.64385619	-3.05889369	-3.18442457	2.111031312	2.032100843
Aradu.YW2J0	2.523561956	2.845991771	3.037382222	3.426264755	2.963474124

Aradu.JQ7DF	NA	NA	NA	NA	NA
Aradu.1195M	1.757023247	1.769771739	1.799087306	1.914564523	2.929790998
Aradu.98BQH	-1.25153877	-1.08926734	-0.91593574	0.298658316	0.485426827
Aradu.DI3D2	0.704871964	0.855989697	0.070389328	0.411426246	1.827819025
Aradu.JMD12	1.333423734	1.608809243	1.13093087	1.13093087	2.007195501
Aradu.D17LB	1.367371066	1.757023247	1.163498732	1.86393845	2.007195501
Aradu.A5IW3	NA	-4.64385619	-3.64385619	NA	-4.05889369
Aradu.ECB76	1.59454855	2.289834465	1.682573297	3.185866545	3.139142019
Aradu.R77I5	NA	NA	NA	NA	NA
Aradu.ZK7ZU	2.046141782	2.121015401	2.100977648	3.10433666	2.908812908
Aradu.X3GYY	2.3950628	2.516015147	2.14404637	2.835924074	2.746312766
Aradu.24SCU	-1.21759144	0.070389328	0.298658316	0.773996325	0.014355293
Aradu.D3TM0	0.82374936	1.416839742	1.084064265	1.608809243	1.570462931
Aradu.V4E4H	1.124328135	1.682573297	1.097610797	1.819668183	1.959770155
Aradu.FLZ7V	2.641546029	2.032100843	2.100977648	-0.35845397	0.014355293
Aradu.7YA73	-1.15200309	-0.577767	-0.68965988	1	1.799087306
Aradu.E92C1	2.797012978	2.622930351	2.100977648	3.958842675	4.026800059
Aradu.G6PP5	2.784503983	2.737686761	2.375734539	1.937344392	2.724650272
Aradu.XHT7Q	3.564378169	3.917431755	3.808385051	4.125981654	4.167518504
Aradu.IR00B	2.700439718	2.847996907	2.684818738	3.443606651	3.635754391
Aradu.VIF27	NA	-6.64385619	-6.64385619	-5.64385619	-3.64385619
Aradu.8S4TL	1.906890596	3.772941338	3.460742564	2.107687869	2.967168608
Aradu.E0NR	1.722466024	1.15704371	0.807354922	-4.32192809	-4.64385619

M					
Aradu.CN01H	0.641546029	1.316145742	0.871843649	2.384049807	3.986410935
Aradu.93Y1F	3.944858446	3.92314918	3.865918815	3.922197848	4.325530332
Aradu.88DVA	1.871843649	2.147306699	2.14404637	3.112700133	2.82374936
Aradu.Z3YHX	1.042644337	1.443606651	1.333423734	2.09085343	2.742006211
Aradu.63N31	1.891419187	4.023255352	2.981852653	3.368768349	1.82374936
Aradu.U8NZD	-5.05889369	-3.47393119	-4.05889369	-5.64385619	NA
Aradu.7MF1E	6.046360091	5.530133184	5.673273737	7.247073033	7.029563314
Aradu.54DRJ	3.014355293	2.944858446	2.799087306	1.970853654	2.286881148
Aradu.M5WCG	-2.25153877	0.389566812	-1.05889369	1.3950628	1.257010618
Aradu.FGV5A	1.811471031	2.477677328	2.192194165	2.827819025	3.171527106
Aradu.RFQ9Z	3.645009884	3.943921327	3.350497247	3.778208576	4.021479727
Aradu.AX5BM	5.063502942	5.092545742	5.533251852	1.709290636	2.192194165
Aradu.ARZ2Q	-1.47393119	-1.25153877	-1.68965988	0.111031312	-0.05889369
Aradu.5VL3J	-1.88896869	-0.51457317	-1.18442457	1.14404637	0.992768431
Aradu.0QC7R	NA	NA	NA	NA	NA
Aradu.A4FGM	-5.64385619	-3.47393119	-4.64385619	0.275007047	0.263034406
Aradu.X96SI	3.639232163	3.370164281	3.500802053	3.042644337	2.976363636
Aradu.NG8V7	2.803227036	2.780310099	2.424922088	3.140778656	3.803227036
Aradu.45HXA	1.063502942	1.454175893	1.257010618	2.182692298	2.298658316
Aradu.G0ZCH	2.952333566	3.010779839	3.169925001	1.280956314	1.550900665
Aradu.L25DZ	2.521050737	3.200064862	3.289834465	3.435628594	4.165911939
Aradu.ZV8PI	-1.15200309	-0.97143085	0.545968369	-5.05889369	-1.64385619

Aradu.3S60E	11.05967569	10.39794197	10.75827319	6.413627929	6.340562269
Aradu.QU90K	NA	-6.64385619	NA	-2.47393119	4.932628157
Aradu.EAZ6K	NA	NA	NA	NA	-5.64385619
Aradu.WZ2NF	2.95419631	3.207892852	3.206330648	1.704871964	1.327687364
Aradu.ID7XN	-4.64385619	-4.32192809	-4.64385619	-1.94341647	-2.32192809
Aradu.F9KJM	4.116863758	4.311793718	4.140778656	5.175923742	5.276496666
Aradu.0ME59	1.389566812	1.967168608	1.5360529	2.967168608	3.121015401
Aradu.N3N49	1.422233001	2.140778656	1.871843649	3.949534933	4.053980168
Aradu.5TE5X	-2.64385619	-3.18442457	-3.47393119	-3.83650127	-2.18442457
Aradu.04VAI	2.084064265	2.263034406	2.080657663	2.711494907	3.401903472
Aradu.AJ236	2.169925001	2.298658316	2.266036894	1.944858446	1.974529312
Aradu.0NA24	0.695993813	0.887525271	0.575312331	1.726831217	1.298658316
Aradu.U3Z1S	0.097610797	0.084064265	-0.55639335	1.201633861	1.627606838
Aradu.IXF4G	NA	-3.05889369	-3.47393119	-1.68965988	-0.71311885
Aradu.R6UC6	1.773996325	2.12763328	1.86393845	2.403267722	2.702657543
Aradu.NP2GP	2.641546029	3.841973119	3.649615459	4.860962798	5.037382222
Aradu.4H615	NA	NA	NA	-5.64385619	-6.64385619
Aradu.GGI7X	1.257010618	1.66448284	1.432959407	2.298658316	2.587364991
Aradu.SH3UZ	1.735522177	2.601696516	1.765534746	0.757023247	1.15704371
Aradu.9LK7Y	1.851998837	2.049630768	1.63691458	3.482848283	3.465974465
Aradu.S2UYW	1.372952098	1.378511623	1.150559677	2.032100843	2.90303827
Aradu.CW3N	NA	-3.64385619	-3.83650127	-4.32192809	0.895302621

A					
Aradu.KVT23	-2.64385619	-2.12029423	-2.55639335	-0.49410907	0.201633861
Aradu.A4ISL	NA	NA	NA	NA	NA
Aradu.S1SNW	7.480426705	6.812626573	7.212082911	3.344828497	3.450221496
Aradu.BGR5Z	2.584962501	3.165107985	2.462052319	3.769771739	3.921245889
Aradu.13D06	1.97819563	2.336283388	2.169925001	0.250961574	0.111031312
Aradu.UR0SD	NA	NA	NA	NA	NA
Aradu.ZYN4B	1.744161096	1.691534165	1.752748591	1.641546029	1.682573297
Aradu.22647	-3.05889369	-2.47393119	-2	-3.64385619	-2.25153877
Aradu.A1MN7	-3.05889369	-2.64385619	-3.47393119	-3.18442457	1.220329955
Aradu.JH3GC	-1.78587519	-2.18442457	-1.73696559	-3.47393119	-4.32192809
Aradu.ERR96	NA	NA	NA	NA	NA
Aradu.U3GYE	3.13422094	3.533563348	3.377123749	4.297925053	4.476381688
Aradu.642R3	-0.64385619	-0.66657627	-0.45403163	-0.25153877	-0.4150375
Aradu.HT46D	0.310340121	2.280956314	1.941106311	0.695993813	1.669026766
Aradu.C881Z	0.344828497	2.861955364	2.298658316	3.261530815	2.5360529
Aradu.QT3HF	-5.64385619	NA	NA	NA	-3.32192809
Aradu.666C5	-5.64385619	-6.64385619	NA	-5.05889369	-1.47393119
Aradu.NG2JA	0.411426246	0.50589093	0.516015147	1.516015147	1.310340121
Aradu.DDG3L	1.632268215	2.776103988	2.545968369	1.263034406	1.485426827
Aradu.ZUG9I	1.933572638	2.341985747	1.565597176	2.657640005	2.324810603
Aradu.V5JUH	-5.64385619	-3.47393119	-4.32192809	-1.94341647	0.516015147
Aradu.VZ4PC	2.857980995	2.422233001	2.657640005	2.485426827	2.575312331

Aradu.28N0X	4.491853096	4.488000771	4.567423758	0.344828497	1.613531653
Aradu.FK3YI	0.422233001	1.310340121	1	1.618238656	1.941106311
Aradu.81UZ4	-3.18442457	-3.83650127	-4.32192809	-1.88896869	-2.05889369
Aradu.LFL4V	0.411426246	0.748461233	0.847996907	0.286881148	1.327687364
Aradu.3Z6PL	1.13093087	1.526068812	1.372952098	2.017921908	1.989139007
Aradu.T0K7A	-5.64385619	-6.64385619	NA	-5.64385619	NA
Aradu.A65JI	1.918386234	2.384049807	2.153805336	2.657640005	3.112700133
Aradu.TN0QL	6.206916673	5.986183363	5.529508641	3.980939266	4.097610797
Aradu.27VHF	2.263034406	2.589763487	2.493134922	2.813524689	3.22342255
Aradu.Z9KCW	2.653060017	3.168321116	2.837943242	3.382667253	3.691534165
Aradu.BAW60	6.191207302	6.193377511	5.921483937	5.919340082	5.839959587
Aradu.BH653	0.545968369	1.244887059	1.521050737	4.550285049	3.721372659
Aradu.EWB3L	7.426684082	7.44178224	7.477353527	4.224966365	4.807354922
Aradu.VSF4G	NA	-6.64385619	NA	-5.05889369	3.119356177
Aradu.2S5CB	-4.64385619	-4.05889369	-4.05889369	-0.88896869	-1.15200309
Aradu.W3UAF	0.659924558	1	0.871843649	2.767654798	2.927896454
Aradu.X3DNX	NA	-6.64385619	NA	NA	-1.15200309
Aradu.UIF60	0.963474124	1.232660757	0.782408565	1.887525271	2.260025656
Aradu.K5U1F	NA	-3.83650127	-4.64385619	-1.94341647	-2.55639335
Aradu.L210Z	-1.51457317	-0.88896869	-1.51457317	-2.73696559	-1.68965988
Aradu.G21TT	0.333423734	0.014355293	0.176322773	-0.34007544	0.310340121
Aradu.NS576	3.941106311	4.810957159	4.234194723	5.163498732	5.035183997

Aradu.3A2M2	1.454175893	3.478971805	3.030336078	1.510961919	1.709290636
Aradu.M035D	0.782408565	1.150559677	0.799087306	0.847996907	0.933572638
Aradu.XJ1QF	NA	-5.05889369	-6.64385619	NA	-3.18442457
Aradu.QCH8L	3.625270489	3.90014226	3.802193217	4.222650022	4.517905554
Aradu.QA1ML	1	1.201633861	1.124328135	1.687060688	1.867896464
Aradu.J0FTC	3.584962501	3.3950628	3.769771739	1.350497247	0.790772038
Aradu.T7E55	5.442280035	5.040892431	5.152994605	2.09085343	2.344828497
Aradu.ZNZ7Q	1.948600847	3.283921772	2.176322773	4.631686366	4.587964989
Aradu.QP7DQ	2.94673086	2.381283373	2.353323291	1.895302621	2.073820233
Aradu.UM7TW	2.44625623	2.485426827	2.153805336	2.12763328	3.443606651
Aradu.4TY89	4.13996057	4.418864577	3.904965719	3.407352751	3.71918344
Aradu.48GRH	2.761285273	2.95419631	2.709290636	4.028569152	4.14974712
Aradu.102BT	1.182692298	1.50589093	2.117695043	-1.47393119	-1.59946207
Aradu.16Z19	2.553360503	2.887525271	2.711494907	2.59454855	2.778208576
Aradu.XSB36	3.152183419	3.381283373	3.324810603	2.35614381	2.680324357
Aradu.384WQ	4.692092375	4.969472865	4.703765179	5.762348816	5.778208576
Aradu.7R8IR	-3.83650127	-3.18442457	-5.64385619	-4.64385619	-2.64385619
Aradu.V5SB4	1.550900665	3.513490746	3.08236197	3.386810946	3.228049048
Aradu.8ET3H	-0.71311885	0.389566812	0.150559677	1.726831217	1.521050737
Aradu.8L9C6	3.991861931	4.21800615	3.962549023	4.930737338	5.132576843
Aradu.CHD0U	2.3305584	2.589763487	2.283921772	4.108524457	4.529820947
Aradu.D1CUJ	4.283921772	4.012568674	3.912649865	2.295723025	2.124328135

Aradu.V56CC	NA	-5.05889369	-4.05889369	-0.81096618	-3.18442457
Aradu.4QT4U	0.650764559	0.59454855	0.298658316	1.263034406	1.339137385
Aradu.1WT6A	5.198886991	5.769507291	4.874305166	6.974644026	6.901228945
Aradu.MSK3Z	1.97819563	2.220329955	2.060047384	1.748461233	1.981852653
Aradu.T922Y	NA	NA	NA	NA	-6.64385619
Aradu.G7DFD	-1.18442457	-0.73696559	-3.05889369	1.286881148	1.718087584
Aradu.FJ32V	3.678071905	3.833902077	3.648465443	4.543495883	4.836429131
Aradu.YQW51	0.879705766	1.339137385	0.773996325	1.454175893	1.875780063
Aradu.R2RMR	-4.05889369	-6.64385619	-5.05889369	-4.64385619	-4.64385619
Aradu.KP7PE	2.809414444	3.349082146	3.140778656	3.688180359	3.450221496
Aradu.P2N1A	-0.34007544	-0.21759144	-0.28630419	0.475084883	0.575312331
Aradu.AE07K	3.500802053	3.67468662	3.368768349	4.121015401	4.331991778
Aradu.P3SFW	NA	NA	NA	NA	NA
Aradu.I5BER	-3.83650127	-3.18442457	-2.64385619	NA	-5.64385619
Aradu.D252Q	3.361768359	3.349082146	3.152183419	4.032100843	3.868884273
Aradu.G5W2I	3.689299161	3.83491343	3.518535139	4.776103988	4.978653265
Aradu.EWR3P	0.070389328	1.765534746	2.959770155	-0.07400058	0.263034406
Aradu.P54FB	NA	-3.32192809	-4.64385619	-4.05889369	-5.64385619
Aradu.RV9UM	4.939696741	4.555202634	4.753818443	2.707082992	2.941106311
Aradu.Z84L6	0.201633861	0.86393845	0.310340121	1.794935663	2.163498732
Aradu.BV9FL	NA	NA	NA	NA	NA
Aradu.QL41Y	-1.25153877	-0.21759144	-1.02914635	1.526068812	1.310340121

Aradu.L4AW6	4.609991295	4.861955364	4.563768278	2.916476644	3.272023189
Aradu.27XB8	2.070389328	2.627606838	2.333423734	3.280956314	3.349082146
Aradu.ZX52Y	7.556352772	7.081936082	7.338959175	4.286141873	4.403949364
Aradu.A7Z3C	3.042644337	2.545968369	2.424922088	2.906890596	3.648465443
Aradu.X2X3M	3.214124805	3.559491813	3.017921908	3.114367025	3.384049807
Aradu.04DD4	1.327687364	1.871843649	1.207892852	2.211012193	2.46727948
Aradu.Q5NNZ	1.526068812	1.98550043	1.269033146	2.565597176	2.841973119
Aradu.2QS2R	-0.53533173	-0.73696559	-0.66657627	-1.32192809	1.097610797
Aradu.UAL0U	-5.64385619	-2.64385619	-4.32192809	-0.13606155	-0.73696559
Aradu.7673S	1.752748591	0.948600847	1.344828497	5.290571851	5.03562391
Aradu.7Z549	NA	-5.64385619	-5.64385619	-6.64385619	-4.32192809
Aradu.G4U2S	2.948600847	3.260025656	3.13093087	4.206330648	4.61176256
Aradu.6C1Z1	NA	NA	NA	NA	NA
Aradu.J1M1P	-1.15200309	-0.47393119	-1.59946207	3.014355293	3.500802053
Aradu.FI2RL	-0.81096618	-0.45403163	-1.21759144	0.847996907	0.910732662
Aradu.U5CVT	1.910732662	1.992768431	1.632268215	1.618238656	2
Aradu.L701F	NA	NA	NA	NA	-1.78587519
Aradu.8T3G1	NA	NA	-6.64385619	1.189033824	1.925999419
Aradu.I0353	3.116031993	3.145677455	3.10433666	2.869871406	3.279471296
Aradu.DGG35	1.028569152	1.316145742	1.042644337	1.752748591	2.266036894
Aradu.K00WP	-4.64385619	-5.64385619	NA	NA	NA
Aradu.G44X8	2.970853654	2.974529312	2.959770155	-0.83650127	-0.02914635

Aradu.N3EHS	3.803227036	3.521050737	3.651912745	1.700439718	0.545968369
Aradu.E1MX8	1.613531653	2.107687869	1.35614381	2.875780063	2.939226578
Aradu.VW4T4	-0.01449957	0.847996907	0.422233001	1.384049807	1.646162657
Aradu.JC32L	1.618238656	1.956056652	1.757023247	2.811471031	2.963474124
Aradu.RDQ06	0.40053793	1.883620816	1.23878686	5.22881869	4.801676029
Aradu.8L7DK	2.606442228	3.176322773	3.008988783	4.447579197	4.756489607
Aradu.576P8	2.422233001	2.901108243	2.339137385	4.171527106	4.310340121
Aradu.D2NW0	5.293149737	4.753283616	4.881664619	5.220329955	5.148120631
Aradu.WF6VN	5.336640446	4.802710219	5.209063395	0.084064265	-0.02914635
Aradu.P51B9	3.945794957	3.534808661	3.439623138	1.459431619	1.516015147
Aradu.23179	1.695993813	3.269033146	2.875780063	0.815575429	0.35614381
Aradu.ED9IS	3.201633861	3.625270489	3.46727948	4.351204277	4.448240225
Aradu.1SD0Q	0.411426246	1.604071324	1.117695043	3.008988783	2.548436625
Aradu.J4Y5T	NA	NA	NA	NA	-4.64385619
Aradu.E0AA1	-0.88896869	-0.21759144	-1.28630419	0.855989697	1.111031312
Aradu.K8XCN	2.718087584	2.627606838	2.50589093	2.897240426	3.145677455
Aradu.7N1F7	-2.18442457	-0.71311885	0.263034406	0.59454855	1.704871964
Aradu.959KY	0.50589093	0.713695815	-0.02914635	2.462052319	2.364572432
Aradu.BUA5R	-0.23446525	0.367371066	0.163498732	-1.78587519	0
Aradu.Z7K8X	2.263034406	2.280956314	1.891419187	3.919340082	3.650764559
Aradu.L3Q6E	0.925999419	1.731183242	1.448900951	2.244887059	2.62058641
Aradu.PJ77P	0.163498732	0.669026766	0.097610797	0.847996907	0.831877241

Aradu.HR1QJ	-2.12029423	-1.68965988	-2.64385619	0.516015147	0.782408565
Aradu.I1T9D	0.214124805	1.117695043	1.176322773	-1.28630419	-0.39592868
Aradu.JFD76	2.837943242	3.77925972	3.627606838	2.289834465	1.757023247
Aradu.JL9ML	NA	NA	NA	NA	NA
Aradu.69EQ4	1.333423734	1.220329955	1.344828497	3.087462841	2.922197848
Aradu.X5BAW	7.574479999	7.16158541	6.989933724	4.484138131	4.46139759
Aradu.5G8Q4	-1.43440282	-0.20091269	-0.39592868	0.516015147	0.807354922
Aradu.SR17F	-1.68965988	-2	-2	-3.05889369	-2.47393119
Aradu.BE2IC	4.72900887	4.313971371	4.202417722	3.607626221	3.880685525
Aradu.XU2Q6	6.551054528	6.687340687	6.22881869	4.51412226	5.881175155
Aradu.5T4PS	1.189033824	1.3950628	1.207892852	2.056583528	2.392317423
Aradu.02IGP	-4.64385619	-2.73696559	-5.05889369	-1.88896869	-3.18442457
Aradu.H976G	-0.71311885	-1.18442457	-1.02914635	0.575312331	1.659924558
Aradu.F6VM1	1.22650853	1.454175893	1.117695043	1.608809243	1.937344392
Aradu.F2B57	2.498250868	2.634593268	2.411426246	3.080657663	3.423578171
Aradu.MM6MH	2.007195501	2.201633861	1.803227036	3.733354341	3.651912745
Aradu.32V7X	4.428946345	4.602884409	4.307428525	2.726831217	3.005399988
Aradu.S59W7	1.580145484	1.827819025	1.752748591	2.618238656	2.805292456
Aradu.IYZ29	2.049630768	2.835924074	2.435628594	2.211012193	2.160274831
Aradu.WQ0V2	4.249445341	4.038260575	4.154615611	2.480265122	2.462052319
Aradu.A42TE	4.003602237	3.997292408	3.587364991	3.781359714	3.390942773
Aradu.069GY	3.478971805	3.797012978	3.523561956	4.831877241	5.169123281
Aradu.T9YM	0.070389328	1.891419187	0.321928095	1.659924558	2.313245852

T					
Aradu.A9E1A	3.503348735	2.528571319	2.62058641	3.119356177	2.733354341
Aradu.I7X6T	1.752748591	2.565597176	2.584962501	1.201633861	1.195347598
Aradu.VSE1D	2.480265122	2.792855352	2.627606838	3.304511042	3.218781168
Aradu.65WG6	-1.59946207	-1.12029423	-1.51457317	2.280956314	1.521050737
Aradu.RRT07	NA	NA	NA	NA	NA
Aradu.UF4PG	-2.25153877	-2.12029423	-1.94341647	-3.05889369	-1.51457317
Aradu.M18SC	2.283921772	2.572889668	2.403267722	3.508428653	3.531069493
Aradu.IC2R8	2.140778656	2.073820233	1.709290636	1.807354922	2.263034406
Aradu.P3RU0	2.124328135	2.464668267	2.22650853	3.073820233	3.592158002
Aradu.W0FED	2.711494907	2.192194165	1.367371066	2.353323291	2.773996325
Aradu.HR13D	-1.18442457	-0.577767	-1.43440282	1.891419187	2.414135533
Aradu.B4AI9	2.66448284	2.709290636	2.397802962	4.150559677	3.765534746
Aradu.HS2H7	2.247927513	2.847996907	2.599317794	3.187451054	3.850999395
Aradu.WKA92	-0.88896869	-0.4150375	-0.88896869	0.432959407	0.704871964
Aradu.0FI0R	0.40053793	0.632268215	0.310340121	0.887525271	1.469885976
Aradu.0U5ND	3.102658131	3.17951105	2.757023247	4.442943496	4.590362488
Aradu.VK4DU	5.804001915	5.773205157	5.846493316	4.66106548	5.009436756
Aradu.KNH52	3.218781168	3.902073579	3.786596362	3.44625623	3.507160349
Aradu.9I2P6	0.790772038	1.275007047	0.669026766	2.121015401	2.339137385
Aradu.65BY0	-1.32192809	2.89917563	1.150559677	-0.25153877	-0.08926734
Aradu.B78C7	4.184280294	3.558267634	3.509695842	2.746312766	2.039138394
Aradu.X9YQ	-3.05889369	-3.18442457	-4.64385619	-3.47393119	-3.18442457

G					
Aradu.SZ9LD	-4.64385619	-4.64385619	-5.05889369	-5.05889369	-5.64385619
Aradu.93KPX	3.116031993	3.477677328	3.365972428	4.257764969	4.452200049
Aradu.B80V6	1.220329955	0.815575429	1.389566812	-0.47393119	0.622930351
Aradu.3127Y	2.702657543	3.364572432	2.533563348	3.977279923	4.155425432
Aradu.NBA2I	NA	-6.64385619	NA	NA	-4.05889369
Aradu.V9UDT	5.099295204	5.452529545	5.021479727	5.904243227	6.203005338
Aradu.808LA	2.10433666	2.367371066	2.459431619	3.292781749	3.930737338
Aradu.19W8X	4.040015679	3.897240426	4.087462841	2.46727948	2.538538164
Aradu.20TYF	-1.83650127	0.98550043	-0.25153877	0.014355293	-0.15200309
Aradu.SUD1N	-1.83650127	-0.28630419	-1.28630419	1.22650853	0.757023247
Aradu.C7P1W	-2.47393119	-4.32192809	-3.05889369	2.568032105	1.10433666
Aradu.42SQT	1.859969548	2.124328135	2.042644337	1.974529312	2.073820233
Aradu.B3V7U	-0.86249648	-0.39592868	-0.37706965	0.443606651	0.748461233
Aradu.R3EFV	0	0.454175893	0.250961574	1.443606651	1.839959587
Aradu.A42Y8	-0.45403163	-0.23446525	-0.88896869	3.624100895	3.061776198
Aradu.ICY54	NA	NA	-3.64385619	NA	-4.32192809
Aradu.G0BMM	-0.39592868	0.464668267	0.613531653	1.500802053	2.266036894
Aradu.Z6Z82	2.046141782	2.160274831	1.63691458	1.14404637	1.744161096
Aradu.4H70X	3.317593505	3.460742564	3.0721058	2.843983844	2.687060688
Aradu.VQB2Q	5.326609251	5.465321514	5.247168	1.867896464	1.952333566
Aradu.7QE0L	3.357552005	2.443606651	2.989139007	0.782408565	0.028569152
Aradu.H8Q0	NA	NA	NA	NA	NA

3					
Aradu.4XE1V	NA	NA	NA	NA	NA
Aradu.PJH5B	2.435628594	2.364572432	1.416839742	0.632268215	1.321928095
Aradu.YPK4Q	2.153805336	2.424922088	2.14404637	2.049630768	2.070389328
Aradu.EYN6M	NA	NA	-6.64385619	NA	-5.64385619
Aradu.9DD9N	-0.91593574	-0.78587519	-0.39592868	-0.20091269	0.070389328
Aradu.712AL	NA	-6.64385619	-5.64385619	NA	-5.05889369
Aradu.12CJQ	-0.62148838	-0.35845397	-0.43440282	1.933572638	1.40599236
Aradu.S514R	3.40053793	3.329123596	3.224966365	4.09423607	4.301587647
Aradu.IG2QJ	1.82374936	2.263034406	2.053111336	2.3305584	2.650764559
Aradu.RQ5UT	0.014355293	0.963474124	-0.20091269	1.891419187	2.22650853
Aradu.7DV9M	1.070389328	1.655351829	1.485426827	2.15704371	2.443606651
Aradu.5BW4X	3.137503524	3.249445341	3.220329955	3.415488271	3.934516502
Aradu.JW08T	-5.64385619	-5.64385619	NA	-3.47393119	-2.94341647
Aradu.EU9MI	-3.64385619	-2.32192809	-2.94341647	-2.25153877	-1.83650127
Aradu.1P1D6	-5.05889369	-5.05889369	NA	-0.86249648	0.807354922
Aradu.S2IZB	-0.12029423	-0.07400058	0.176322773	1.713695815	1.316145742
Aradu.T8M37	2.908812908	3.200064862	3.016139703	3.371558863	3.745237332
Aradu.NNH8B	-3.64385619	-3.32192809	-5.64385619	-1.02914635	1.232660757
Aradu.E3SP0	3.19061486	2.861955364	3.028569152	1.691534165	1.485426827
Aradu.1FD3D	-3.18442457	-2.39592868	-3.32192809	-2.47393119	-2.73696559
Aradu.G7F8E	3.286881148	3.129283017	3.327687364	1.469885976	2.153805336
Aradu.H48T8	7.060695932	6.684397984	7.172727518	1.23878686	1.454175893

Aradu.IM707	-2.64385619	-3.18442457	-3.18442457	-4.05889369	-1.59946207
Aradu.67UXU	1.411426246	1.952333566	1.560714954	2.89917563	2.925999419
Aradu.G0Z55	NA	NA	NA	-0.02914635	-1.08926734
Aradu.PKA69	-1.39592868	-1.08926734	-1.05889369	2.03562391	2.99095486
Aradu.I67GN	2.448900951	2.518535139	2.584962501	2.50589093	2.09085343
Aradu.1J279	1.86393845	2.028569152	1.765534746	1.682573297	1.786596362
Aradu.AQ9N2	1.831877241	2.763411574	2.010779839	3.488000771	3.340562269
Aradu.NH66H	2.003602237	2.49057013	1.887525271	2.786596362	2.904965719
Aradu.X9U06	1.469885976	2.073820233	2.049630768	2.367371066	2.286881148
Aradu.26N74	NA	NA	NA	NA	NA
Aradu.K2B5B	3.549669171	3.527320608	3.707082992	3.868884273	4.051372102
Aradu.8X6B9	2.292781749	2.046141782	1.516015147	0.464668267	1.831877241
Aradu.BLA9Y	2.596935142	2.976363636	3.073820233	3.631104282	3.767654798
Aradu.HD203	NA	-6.64385619	NA	NA	NA
Aradu.CI09B	4.095080492	4.442943496	4.639232163	4.628773595	4.970393538
Aradu.WB5VJ	3.891419187	3.997292408	3.799087306	1.555816155	1.761285273
Aradu.Z2HCJ	2.333423734	2.678071905	2.533563348	3.207892852	3.473786912
Aradu.0768L	-1.47393119	-1.39592868	-2.05889369	0.378511623	1.618238656
Aradu.FRD61	4.383358695	5.518535139	5.078524445	5.30633516	5.159467729
Aradu.GF1M7	3.344828497	3.187451054	3.025028794	4.266036894	4.62935662
Aradu.4NW8B	-4.05889369	-3.47393119	-2.05889369	-3.64385619	-3.18442457
Aradu.1X2P	2.493134922	0.545968369	0.50589093	0.344828497	1.589763487

C					
Aradu.65CE7	NA	-5.64385619	-5.64385619	-5.05889369	-3.18442457
Aradu.VM94P	7.106536769	6.644433152	6.7822775	1.014355293	1.799087306
Aradu.XL4N2	3.602884409	3.22650853	2.66448284	4.906890596	4.564378169
Aradu.CMI46	3.456806149	3.22650853	3.250961574	0.275007047	0.40053793
Aradu.9S9RY	1.855989697	2.807354922	2.500802053	2.22650853	2.17951105
Aradu.VHJ0W	-5.05889369	-5.05889369	-4.64385619	-4.05889369	-2.47393119
Aradu.R92J4	2.375734539	2.443606651	2.475084883	1.655351829	2.049630768
Aradu.H56YA	1.304511042	2.841973119	2.077242999	-3.05889369	-1.39592868
Aradu.RB14Z	-2.55639335	-1.18442457	-1.78587519	-2.18442457	-0.59946207
Aradu.BM2UV	3.261530815	3.870857864	3.625270489	4.658211483	4.890933022
Aradu.CTE87	2.691534165	3.019701914	2.797012978	3.303050085	3.575312331
Aradu.F8MV2	-6.64385619	-2.32192809	-3.18442457	-0.577767	-0.12029423
Aradu.KJE7Z	-5.64385619	-4.32192809	-5.64385619	-3.64385619	-3.05889369
Aradu.P0ZYD	1.778208576	0.084064265	-1.12029423	3.658782734	3.102658131
Aradu.W9VU7	2.324810603	2.765534746	2.266036894	3.085764554	3.209453366
Aradu.L3IFZ	-1.78587519	-1.28630419	-1.55639335	-0.37706965	-0.76121314
Aradu.58BVX	3.59812696	3.447579197	3.578938713	1.959770155	2.032100843
Aradu.7P5YA	1.981852653	2.613531653	2.440952198	2.669026766	3.270528942
Aradu.2503N	4.952799478	5.526694846	4.536674618	6.359134582	6.348905161
Aradu.5R97S	2	1.879705766	1.97819563	1.40053793	1.604071324
Aradu.CML1P	1.195347598	1.286881148	1.15704371	1.996388746	2.137503524
Aradu.0H9W	5.657925772	5.726013749	5.374343989	2.381283373	2.419538892

K					
Aradu.EU3KX	0.516015147	0.250961574	0.23878686	0.333423734	1.427606173
Aradu.J31ZQ	3.080657663	4.917431755	4.684257705	4.449561375	4.996388746
Aradu.CT448	-2.18442457	-5.64385619	-4.05889369	0.214124805	-0.20091269
Aradu.B4P3F	NA	NA	NA	NA	-5.64385619
Aradu.8Z9JB	0.150559677	0.765534746	0.59454855	1.056583528	2.3950628
Aradu.VX595	2.587364991	3.619413011	3.512226887	3.15704371	2.792855352
Aradu.3D1M6	1.257010618	1.59454855	1.201633861	0.650764559	1.475084883
Aradu.HU8ZS	3.132576843	3.388189537	3.173127433	3.830863757	3.993674362
Aradu.Z1N61	-5.05889369	-3.64385619	-4.32192809	-3.83650127	-1.15200309
Aradu.9S16L	NA	-6.64385619	NA	-6.64385619	-4.32192809
Aradu.XVT29	2.03562391	3.049630768	2.750606505	3.375734539	5.254745198
Aradu.ET2UI	1.13093087	1.182692298	0.895302621	2.160274831	2.211012193
Aradu.Q67RD	0.918386234	-0.16812276	0.214124805	2.780310099	3.454175893
Aradu.0IR2T	0.333423734	0.321928095	-1.78587519	4.185073638	4.370164281
Aradu.MX7X2	2.15704371	2.432959407	2.176322773	1.835924074	2.389566812
Aradu.B3D9E	0.298658316	1.14404637	-0.30400619	3.653060017	3.593353771
Aradu.Z17B7	-0.86249648	-0.68965988	-1.32192809	0.963474124	0.839959587
Aradu.S44IV	1.220329955	2.14404637	2.214124805	4.185866545	4.666756592
Aradu.55W9R	3.298658316	3.439623138	3.307428525	3.782408565	4.058316496
Aradu.75ICF	-5.64385619	-3.83650127	-3.18442457	-0.88896869	0.713695815
Aradu.RQ3SQ	NA	NA	NA	NA	NA
Aradu.MJT2	4.218781168	4.254745198	4.095080492	4.272023189	4.735522177

G					
Aradu.0SC6H	0.097610797	2.195347598	2.121015401	3.757023247	3.459431619
Aradu.AP5ZW	1.941106311	2.189033824	2.070389328	2.759155834	2.939226578
Aradu.8Y4W7	4.698218478	4.6622055	4.709290636	2.90303827	4.379898164
Aradu.BLA0I	-0.78587519	-0.34007544	0.432959407	-1.32192809	0.321928095
Aradu.M785W	1.10433666	1.176322773	0.98550043	1.201633861	1.761285273
Aradu.PIT85	4.121844298	4.060047384	3.781359714	1.933572638	2.813524689
Aradu.C42LD	1.15704371	1.659924558	1.411426246	2.298658316	2.244887059
Aradu.U260V	3.329123596	2.615887074	3.09423607	4.448240225	3.683696454
Aradu.C4Y83	2.211012193	2.780310099	2.448900951	3.412781525	3.607626221
Aradu.0IN20	1.575312331	1.673556424	1.599317794	1.687060688	2.232660757
Aradu.0GK1P	1.339137385	1.887525271	1.618238656	2.084064265	2.114367025
Aradu.5D3K5	0.5360529	0.887525271	0.432959407	2.14404637	2.321928095
Aradu.0E8DM	-0.4150375	0.22650853	-0.64385619	5.152994605	4.347665656
Aradu.FNH9F	2.464668267	3.017921908	2.761285273	3.56924803	3.669026766
Aradu.RI35R	4.258518925	4.048759312	3.814550423	3.584962501	3.893362211
Aradu.X8KRI	1.86393845	1.526068812	1.599317794	0.86393845	1.344828497
Aradu.MSR4Q	NA	NA	-5.05889369	NA	-2.83650127
Aradu.J8MWU	3.165107985	3.349082146	2.87774425	3.638073837	3.495695163
Aradu.WR10B	4.424250286	4.244125943	4.124328135	3.739848103	3.690417096
Aradu.SA9NZ	-0.73696559	-0.76121314	-1.64385619	1.63691458	1.867896464
Aradu.KM3QT	-5.05889369	-0.78587519	-2	1.580145484	0.910732662
Aradu.ZFW5	3.270528942	3.545968369	3.363171077	3.857980995	3.803227036

1					
Aradu.WC37M	0.367371066	1.565597176	0.970853654	1.427606173	1.713695815
Aradu.SEJ3V	3.321928095	2.895302621	3.137503524	3.908812908	4.045268215
Aradu.0M9X8	5.516645558	4.9800253	5.283180979	2.687060688	2.778208576
Aradu.Y85YF	1.084064265	1.232660757	0.970853654	1.618238656	1.871843649
Aradu.Z5LJB	-5.64385619	-4.32192809	-4.64385619	-3.47393119	-3.32192809
Aradu.M29FV	2.017921908	1.970853654	1.782408565	2.817623258	3.324810603
Aradu.457AG	2.427606173	2.786596362	2.469885976	3.97819563	3.810443104
Aradu.2TK0M	-3.18442457	-2.94341647	NA	-0.23446525	3.516015147
Aradu.87SDL	3.22650853	3.341985747	3.061776198	3.825785627	4.233427944
Aradu.QR44H	-0.76121314	-0.71311885	-1.15200309	1.773996325	1.807354922
Aradu.DM13Q	NA	NA	-6.64385619	NA	NA
Aradu.B4N91	1.618238656	2.472487771	2.201633861	3.117695043	3.420886575
Aradu.A2F1I	-4.32192809	-2.18442457	-3.83650127	-2.05889369	-1.55639335
Aradu.E7L5G	-0.86249648	-0.62148838	-1.47393119	1.182692298	1.516015147
Aradu.5XQ31	-4.64385619	-3.83650127	-5.05889369	-3.32192809	-0.76121314
Aradu.TQ3RZ	2.807354922	2.577730931	2.6622055	-1.59946207	-0.47393119
Aradu.E58SH	1.028569152	2.646162657	1.659924558	2.462052319	2.462052319
Aradu.CV82P	-0.13606155	-0.10469738	0.124328135	-0.21759144	1.718087584
Aradu.KZ4HC	1.10433666	1.220329955	0.782408565	2.09085343	1.726831217
Aradu.G5CNQ	3.607626221	4.100977648	3.669026766	4.470536865	4.830356747
Aradu.PSU1K	0.432959407	1.50589093	1.257010618	1.545968369	1.843983844
Aradu.UZ3U	0.422233001	0.86393845	0.910732662	1.944858446	2.613531653

X					
Aradu.T0VLB	1.117695043	1.448900951	1.03562391	2.160274831	2.528571319
Aradu.366AT	2.341985747	2.659924558	2.500802053	3.005399988	3.44625623
Aradu.DBJ1I	2.060047384	1.59454855	1.510961919	0.59454855	1.007195501
Aradu.93VCV	-0.68965988	0.704871964	0.333423734	1.263034406	1.839959587
Aradu.T2XD3	0.855989697	1.22650853	1.137503524	1.176322773	2.310340121
Aradu.13MQ9	2.983677695	3.140778656	2.653060017	2.813524689	2.963474124
Aradu.U18XV	-0.55639335	0.584962501	-0.34007544	1.516015147	1.531069493
Aradu.4V6Q9	NA	NA	NA	-6.64385619	-6.64385619
Aradu.JU5TS	0.575312331	0.669026766	0.765534746	1.695993813	2.09423607
Aradu.WU2UF	-0.88896869	-2.12029423	-2.73696559	-3.64385619	-0.86249648
Aradu.Z7SDW	3.781359714	3.221877081	3.491853096	2.629939409	2.500802053
Aradu.N9KIE	-0.39592868	-0.43440282	-0.81096618	-0.02914635	0.485426827
Aradu.XI98N	2.275007047	3.935459748	3.303050085	3.310340121	4.491853096
Aradu.04GWS	1.124328135	1.678071905	1.3950628	2.46727948	2.831877241
Aradu.U5Z1W	3.220329955	3.559491813	3.378511623	3.285402219	3.201633861
Aradu.LK89V	1.63691458	1.632268215	1.773996325	2.039138394	2.169925001
Aradu.72C6M	0.367371066	0.485426827	0.526068812	0.263034406	0.86393845
Aradu.N4RHU	0.948600847	0.790772038	1.007195501	0.887525271	1.604071324
Aradu.2C28W	0.321928095	0.613531653	0.310340121	0.782408565	1.084064265
Aradu.1U59X	2.589763487	2.472487771	2.070389328	3.414135533	3.655351829
Aradu.LC8HL	-1.05889369	-0.68965988	-1.28630419	0.389566812	0.887525271
Aradu.C2BM	-0.15200309	-0.51457317	-0.97143085	-0.28630419	0.411426246

8					
Aradu.99FEF	1.03562391	1.726831217	1.495695163	1.469885976	1.646162657
Aradu.3Y26A	NA	NA	NA	NA	-5.64385619
Aradu.2W9TG	-3.05889369	-4.64385619	-5.64385619	-4.32192809	-1.55639335
Aradu.60GK5	-2.83650127	-4.64385619	-4.32192809	-0.78587519	1.13093087
Aradu.98PQS	-2.73696559	-2.73696559	-3.47393119	-0.59946207	-0.76121314
Aradu.9IB22	-0.73696559	-1.05889369	-1.32192809	-2.32192809	-0.94341647
Aradu.P9PXC	-5.64385619	-5.64385619	NA	-2.25153877	-0.64385619
Aradu.1B3IN	8.883498632	8.241840184	8.449189924	5.197314999	6.455491621
Aradu.065GY	-5.64385619	-4.05889369	-5.64385619	-5.64385619	-3.05889369
Aradu.GY8D2	4.295723025	4.545350645	4.382667253	5.109778429	5.206330648
Aradu.Q5XDE	2.032100843	2.250961574	2.077242999	1.887525271	2.23572706
Aradu.S3UJF	-1.68965988	-0.59946207	-0.88896869	1.464668267	1.974529312
Aradu.94JTX	-4.05889369	NA	NA	-2.18442457	7.684327846
Aradu.B11ZS	-0.16812276	3.075532631	2.568032105	-0.4150375	1.292781749
Aradu.W58GD	-1.73696559	-1.68965988	-1.78587519	0.344828497	0.495695163
Aradu.YK06D	3.99095486	2.364572432	2.739848103	2.510961919	3.255500733
Aradu.623Y7	3.176322773	3.388189537	3.283921772	1.855989697	2.077242999
Aradu.YY4G2	2.289834465	2.680324357	2.301587647	3.564378169	3.943921327
Aradu.G31GN	-2.94341647	-0.83650127	-1.28630419	-1.21759144	-1.15200309
Aradu.L3NRF	2.976363636	2.386810946	3.220329955	-0.23446525	1.485426827
Aradu.A64FA	NA	NA	NA	NA	NA
Aradu.DL7C	3.680324357	3.277984747	3.381283373	1.687060688	2.042644337

8					
Aradu.AR630	-1.43440282	-1.68965988	-1.73696559	-1.94341647	-1.39592868
Aradu.BQ4IU	1.718087584	2.014355293	1.895302621	2.970853654	3.047887329
Aradu.V2KKS	2.634593268	2.922197848	2.639232163	1.622930351	2.056583528
Aradu.12XXA	-0.18442457	0.23878686	-0.07400058	0.042644337	1.007195501
Aradu.X87GF	-3.83650127	-2.64385619	-4.05889369	0.150559677	-0.47393119
Aradu.Y5XYT	2.952333566	3.017921908	2.711494907	2.778208576	3.192194165
Aradu.0FG6K	-4.64385619	-3.83650127	-4.05889369	-3.64385619	-3.64385619
Aradu.11XZB	NA	NA	NA	NA	NA
Aradu.Q54JY	2.244887059	2.516015147	2.316145742	2.533563348	2.931683057
Aradu.W7LFI	-2.94341647	-3.83650127	-1.64385619	-2.64385619	-2
Aradu.VE0KU	0.5360529	1.604071324	0.333423734	2.182692298	2.435628594
Aradu.TI9C9	-0.43440282	0.014355293	-0.07400058	1.316145742	1.831877241
Aradu.94J1A	NA	NA	NA	-2.64385619	-0.37706965
Aradu.LXV06	-3.64385619	-5.64385619	-5.05889369	-4.64385619	-3.47393119
Aradu.648HW	2.341985747	2.22650853	2.241840184	2.545968369	2.592158002
Aradu.98BJD	NA	NA	NA	NA	NA
Aradu.KT95M	2.042644337	2.849999259	2.548436625	3.209453366	3.576522138
Aradu.7X3HU	NA	-6.64385619	-5.64385619	-1.94341647	-2.73696559
Aradu.ZP2GZ	NA	NA	NA	NA	NA
Aradu.CGD8Q	-0.91593574	-1.15200309	-2.73696559	3.260025656	0.839959587
Aradu.5Y6Q8	0	0.695993813	-0.10469738	3.323370069	3.485426827
Aradu.G99G	4.174725988	4.64731451	4.577730931	4.676944359	5.106013238

B					
Aradu.GFS4B	NA	NA	NA	NA	NA
Aradu.L6TW0	-1.94341647	-1.05889369	-1.51457317	2.378511623	2.260025656
Aradu.3P3LL	2.147306699	2.528571319	2.358958826	3.053111336	3.316145742
Aradu.QE5XI	2.994579724	2.827819025	2.533563348	4.177120503	4.234194723
Aradu.A2UTQ	NA	NA	NA	NA	NA
Aradu.PP2S4	-0.78587519	-1.21759144	-1.64385619	1.40053793	1.257010618
Aradu.SGK85	4.454833907	4.412781525	4.424922088	3.282439805	3.450221496
Aradu.2F17T	1.443606651	1.700439718	1.669026766	2.639232163	2.879705766
Aradu.2P97F	3.577730931	4.0721058	3.764473551	4.692092375	4.700439718
Aradu.BV2ZB	2.35614381	2.482848283	2.392317423	3.058316496	3.198494154
Aradu.93R3L	2.198494154	2.275007047	2.073820233	1.244887059	1.541019153
Aradu.CKV49	-0.05889369	0.056583528	-0.97143085	2.121015401	2.618238656
Aradu.IW0ES	1.22650853	2.456806149	2.176322773	4.212569339	3.836934011
Aradu.WNQ8E	2.742006211	2.948600847	2.901108243	3.232660757	3.614709844
Aradu.IHZ0W	5.045268215	5.09423607	4.890933022	4.802193217	5.231893162
Aradu.IFW71	-3.32192809	-3.05889369	-2.83650127	-1.68965988	-0.16812276
Aradu.91ENH	NA	NA	NA	NA	-4.64385619
Aradu.EFI11	-1.02914635	-0.49410907	-2.32192809	1.769771739	2.839959587
Aradu.5L8S5	-3.32192809	-4.32192809	-5.64385619	-5.64385619	0.650764559
Aradu.G99LQ	-0.66657627	-0.34007544	-0.43440282	0.367371066	1.049630768
Aradu.M6HNT	-0.62148838	-0.05889369	-2.83650127	2.201633861	3.097610797
Aradu.0KB9L	-0.88896869	-1.51457317	-1.28630419	-0.23446525	0.669026766

Aradu.23Z4K	2.353323291	3.148934105	2.987320866	2.731183242	3.66106548
Aradu.0WA6T	3.114367025	3.544732656	2.776103988	5.215678597	4.853496704
Aradu.UWX45	0.807354922	1.952333566	0.895302621	3.234194723	3.266036894
Aradu.SAM0J	0.815575429	1.13093087	0.650764559	1.510961919	1.526068812
Aradu.W45TC	0.831877241	1.220329955	0.970853654	1.056583528	1.263034406
Aradu.R9NWV	1.722466024	2.541019153	2.229587923	2.482848283	2.786596362
Aradu.66DLZ	2.23878686	2.885574364	2.929790998	3.249445341	3.480265122
Aradu.E0JEI	NA	-4.64385619	-4.64385619	NA	-3.83650127
Aradu.3GX0M	-3.47393119	-3.05889369	-2.39592868	NA	-6.64385619
Aradu.VT3C0	-0.55639335	1.063502942	-0.86249648	-1.15200309	-0.51457317
Aradu.KQ4LB	NA	NA	NA	NA	NA
Aradu.ANT5S	-6.64385619	-5.05889369	-5.64385619	-4.32192809	-4.32192809
Aradu.5ME2Z	3.904002316	4.387500406	3.493134922	5.414473836	5.435295216
Aradu.IZ0B7	-6.64385619	-4.05889369	-3.83650127	-3.05889369	-0.83650127
Aradu.96IDX	-2.73696559	-2.55639335	-2.94341647	-1.32192809	-0.97143085
Aradu.U5DG8	-0.97143085	-1.05889369	-1.21759144	-0.91593574	0.378511623
Aradu.HQI6K	NA	NA	NA	NA	NA
Aradu.G676K	1.66448284	2.384049807	1.952333566	3.269033146	3.962549023
Aradu.CMZ60	-1.43440282	-2.18442457	-1.28630419	0.575312331	1.555816155
Aradu.F591F	-2.32192809	-0.68965988	0.443606651	NA	-2.55639335
Aradu.94Q3R	-5.64385619	-2.73696559	-2.64385619	0.650764559	1.244887059
Aradu.U795	3.43162296	3.403267722	3.195347598	3.228049048	3.992768431

Q					
Aradu.AZ3DS	1.778208576	2	1.859969548	3.042644337	3.189033824
Aradu.17HPD	2.137503524	3.374343989	2.063502942	4.427606173	4.305970521
Aradu.7E3FL	-0.81096618	-0.13606155	-0.10469738	0.516015147	0.695993813
Aradu.E5ATX	-5.64385619	-4.05889369	-6.64385619	-3.18442457	-4.64385619
Aradu.VKS8C	-5.64385619	NA	NA	-5.64385619	-3.18442457
Aradu.K2SIM	NA	-6.64385619	-5.64385619	NA	2.169925001
Aradu.7I20U	6.759954742	6.835924074	6.529820947	7.153906645	6.942397208
Aradu.Y4PLR	4.140778656	4.713145902	3.627606838	5.7465815	5.61117238
Aradu.4N3TL	-3.47393119	-2.55639335	-2.73696559	-4.05889369	1.195347598
Aradu.NKJ9K	3.107687869	3.099295204	2.959770155	3.324810603	3.575312331
Aradu.889PJ	NA	NA	NA	NA	NA
Aradu.FB7VW	2.969012308	5.113117036	4.203984166	2.039138394	2.35614381
Aradu.3KJ9A	3.718087584	3.687060688	3.865918815	2.23572706	2.361768359
Aradu.Y54CT	0.948600847	0.941106311	0.604071324	2.675815931	3.344828497
Aradu.NDR67	1.195347598	1.967168608	1.761285273	1.627606838	1.992768431
Aradu.RY6N7	2.865918815	3.212569339	2.970853654	4.340562269	4.313245852
Aradu.6Q6K7	1.608809243	2.021479727	1.735522177	2.292781749	2.691534165
Aradu.V9MVJ	1.378511623	1.469885976	1.23878686	0.963474124	1.214124805
Aradu.586VX	2.316145742	2.963474124	2.456806149	2.572889668	3.016139703
Aradu.X3YIU	-5.64385619	-4.05889369	-4.32192809	-0.53533173	0.411426246
Aradu.DZ6IG	2.166715445	0.641546029	0.411426246	-0.10469738	0.084064265
Aradu.01VA	-1.68965988	1.084064265	1.084064265	1.14404637	1.384049807

S

Aradu.HKL1A	1.799087306	2.198494154	1.970853654	2.6622055	2.976363636
Aradu.C90G3	4.125981654	4.533563348	4.318316841	4.032982417	4.61117238
Aradu.I5BGE	-5.64385619	-4.64385619	-2.94341647	0.485426827	0.485426827
Aradu.2W21G	0.070389328	0.35614381	0.263034406	0.454175893	0.5360529
Aradu.Z8JSI	-1.21759144	-3.47393119	-4.64385619	0.028569152	0.150559677
Aradu.S9P8W	NA	-5.05889369	NA	NA	NA
Aradu.15RGK	0.941106311	1.257010618	1.13093087	1.580145484	1.650764559
Aradu.LI04Q	5.623515741	7.401818163	7.149848714	4.757556689	5.613826291
Aradu.36LCY	3.061776198	3.683696454	3.397802962	3.82374936	3.875780063
Aradu.ZRM2P	-0.53533173	-0.16812276	-0.86249648	2.269033146	2.389566812
Aradu.3ZR52	3.374343989	3.237257771	3.324810603	1.22650853	1.735522177
Aradu.Z5BX3	-1.47393119	-1.68965988	-1.32192809	1.042644337	1.169925001
Aradu.VE705	-1.35845397	0.310340121	-1.05889369	-2.05889369	-1.88896869
Aradu.4K5XY	8.226074963	7.997235945	8.285540933	3.032100843	3.368768349
Aradu.M7VT5	2.22342255	2.782408565	2.666756592	3.295723025	3.713695815
Aradu.KJH8E	-4.05889369	-3.83650127	-2.83650127	-4.64385619	-3.47393119
Aradu.00WD1	3.724650272	4.177917792	4.229587923	3.962549023	4.367371066
Aradu.M72UM	4.808899839	4.512226887	4.477677328	3.924099886	4.244125943
Aradu.9GN4P	1.427606173	1.726831217	1.622930351	3.240314329	3.94204526
Aradu.3ZF9R	-1.88896869	-1.18442457	-1.78587519	-0.39592868	-0.577767
Aradu.RCY11	3.839959587	3.566815154	3.552131108	3.140778656	3.119356177
Aradu.270H	0.176322773	0.731183242	0.650764559	1.042644337	1.500802053

A					
Aradu.NF928	NA	-5.64385619	-5.05889369	-4.32192809	1.422233001
Aradu.CG1AI	-0.71311885	-1.55639335	-1.51457317	-2.94341647	-3.18442457
Aradu.QNG0R	0.565597176	1.049630768	0.50589093	1.695993813	1.622930351
Aradu.UL23E	2.475084883	1.992768431	2.316145742	2.422233001	2.229587923
Aradu.SS76M	NA	-6.64385619	NA	NA	-4.64385619
Aradu.E3BEW	0.35614381	0.910732662	0.722466024	3.215678597	2.801158656
Aradu.M98W9	2.46727948	2.925999419	2.63691458	4.062639828	4.13093087
Aradu.J5HSK	4.914564523	5.106850796	4.756489607	4.751677946	4.867402306
Aradu.HNS4U	-3.05889369	-2.25153877	-4.64385619	0.516015147	0.584962501
Aradu.V3JVM	0.98550043	1.427606173	1.298658316	1.570462931	1.925999419
Aradu.Y43HK	-5.05889369	-3.83650127	-4.32192809	-5.64385619	-3.18442457
Aradu.5KS9C	NA	-4.05889369	-4.64385619	-3.64385619	-1.94341647
Aradu.P0V8P	1.748461233	1.974529312	1.560714954	2.61117238	2.192194165
Aradu.1Y87X	NA	-6.64385619	NA	NA	-3.64385619
Aradu.E101D	NA	NA	NA	-5.64385619	-4.32192809
Aradu.CPF02	NA	NA	NA	NA	-5.64385619
Aradu.S2K0Q	1.786596362	2.066950244	2.017921908	3.102658131	3.220329955
Aradu.V9VCU	-2	-3.32192809	-5.05889369	1.304511042	1.432959407
Aradu.84U8V	-1.73696559	-2.05889369	-2.64385619	-0.25153877	-0.21759144
Aradu.CBY05	1.739848103	2.073820233	1.831877241	3.286881148	3.592158002
Aradu.61CW5	0.495695163	-0.26881676	-0.07400058	-1.32192809	-0.45403163
Aradu.LVP8M	-0.21759144	0.432959407	0.137503524	2.353323291	2.5360529

Aradu.95DDC	2.746312766	2.629939409	2.565597176	1.521050737	2.244887059
Aradu.BWC86	1.250961574	1.361768359	0.97819563	1.967168608	2.427606173
Aradu.7PT52	NA	-5.64385619	-5.05889369	NA	-3.18442457
Aradu.QU8L1	NA	-6.64385619	NA	NA	-5.64385619
Aradu.D9N4K	1.807354922	2.260025656	1.839959587	1.992768431	2.107687869
Aradu.D4KTP	-4.05889369	-2.83650127	-2.18442457	-3.83650127	-2.83650127
Aradu.UA2HU	-4.32192809	NA	NA	-4.05889369	-2.12029423
Aradu.C9LG8	5.121015401	5.510645504	5.340562269	6.920055055	6.964052011
Aradu.63EET	0.22650853	-0.21759144	-0.51457317	0.641546029	0.748461233
Aradu.0T5YP	0.464668267	2.375734539	2.100977648	1.5360529	1.847996907
Aradu.9NC59	NA	NA	NA	NA	-6.64385619
Aradu.L2RSA	-0.32192809	-1.25153877	-1.47393119	-0.76121314	-2.18442457
Aradu.N9RWR	0.773996325	0.526068812	0.575312331	0.5360529	0.82374936
Aradu.B80WC	-3.05889369	-1.55639335	-1.51457317	-0.51457317	-0.08926734
Aradu.PJ1DH	2.386810946	2.939226578	2.726831217	3.025028794	2.553360503
Aradu.3V07T	-4.32192809	-1	-0.78587519	0.310340121	0.163498732
Aradu.MI2LX	3.575312331	3.486714373	3.077242999	5.417177412	6
Aradu.T7YJG	-0.18442457	0.070389328	-0.34007544	3.005399988	2.87774425
Aradu.D77VM	NA	NA	NA	NA	NA
Aradu.81WR9	0.201633861	0.963474124	0.59454855	1.427606173	1.580145484
Aradu.4NJ9R	1.879705766	2.464668267	2.182692298	2.961623328	3.125981654
Aradu.M0ENQ	-5.64385619	-3.18442457	-2.73696559	-2.55639335	-2.18442457

Aradu.5R9X5	NA	NA	NA	NA	NA
Aradu.43TRE	4.991861931	4.99004722	4.811984719	5.046141782	5.171527106
Aradu.S3AS8	-1.21759144	-1.47393119	-1.73696559	-4.64385619	-1.88896869
Aradu.RYQ8I	6.832383717	7.008204497	7.104965605	2.386810946	2.543495883
Aradu.K1BYW	NA	-4.32192809	-5.64385619	0.895302621	-1.12029423
Aradu.JMZ0B	1.286881148	1.416839742	1.378511623	2.301587647	2.392317423
Aradu.F5XX7	2.916476644	3.116031993	2.849999259	1.40053793	1.847996907
Aradu.R4RRW	NA	NA	NA	NA	-4.64385619
Aradu.UA9D8	4.011674533	3.849999259	3.949534933	1.555816155	1.956056652
Aradu.B18E9	NA	NA	-6.64385619	-3.83650127	-6.64385619
Aradu.FL84V	0.070389328	1.03562391	0.389566812	2.260025656	2.107687869
Aradu.K6YI3	3.514753498	3.92314918	3.638073837	4.375734539	4.969012308
Aradu.C7C1R	4.175524601	4.754887502	4.3305584	5.802968651	5.742006211
Aradu.VTZ6D	1.735522177	2.021479727	1.757023247	2.361768359	2.634593268
Aradu.1ZJ1C	-0.35845397	-0.49410907	-1.68965988	-2.05889369	-1
Aradu.5HX5K	6.033643244	7.037711667	6.89056829	3.751677946	4.074676686
Aradu.9TG4T	1.3950628	1.622930351	1.350497247	1.63691458	2.414135533
Aradu.49PAS	3.282439805	3.861955364	3.694880193	1.618238656	1.659924558
Aradu.27SYD	NA	-6.64385619	NA	NA	-4.64385619
Aradu.Q3KFX	NA	-5.05889369	NA	NA	NA
Aradu.W34NY	4.15704371	4.489286023	4.184280294	3.472487771	3.703765179
Aradu.G6ZA5	-5.64385619	-0.47393119	-3.64385619	-4.32192809	-1

Aradu.K26VM	-0.30400619	0.189033824	0.070389328	-2.55639335	-0.94341647
Aradu.46FZZ	2.6622055	3.217230716	3.243364426	2.843983844	3.139142019
Aradu.P56V8	NA	NA	NA	NA	NA
Aradu.N5GDJ	-1.55639335	-1.02914635	-1.25153877	-3.64385619	1.952333566
Aradu.8C9N3	0.150559677	1.416839742	0.831877241	1.22650853	1.150559677
Aradu.DK6DD	0.704871964	0.86393845	-0.18442457	2.310340121	1.855989697
Aradu.NCP7U	1.887525271	2.107687869	1.655351829	2.639232163	2.861955364
Aradu.W8J78	-1.21759144	1.014355293	1.97819563	NA	NA
Aradu.MQ1LE	3.016139703	3.382667253	3.078951341	3.705977902	3.814550423
Aradu.WDS9Z	-0.86249648	-1.15200309	-1.35845397	-1.05889369	0.097610797
Aradu.4R8IN	NA	NA	NA	-3.83650127	-0.97143085
Aradu.L9R8I	4.110196178	4.399854674	4.072963272	5.348374075	5.615004242
Aradu.97VNG	NA	-5.64385619	NA	NA	NA
Aradu.R6ZKE	1.895302621	2.247927513	2.121015401	2.901108243	3.152183419
Aradu.LKW4E	2.313245852	1.263034406	1.531069493	1.214124805	1.575312331
Aradu.C716W	-5.05889369	-5.05889369	-6.64385619	0.659924558	1.831877241
Aradu.U6YR6	3.056583528	3.933572638	4.817111573	0.070389328	1.22650853
Aradu.Z84GQ	2.825785627	3.049630768	2.754887502	1.86393845	2.100977648
Aradu.93WPA	2.341985747	2.918386234	2.629939409	3.439623138	3.71918344
Aradu.9W61Z	0.084064265	0.263034406	0.097610797	1.195347598	1.480265122
Aradu.VM8XK	1.82374936	1.875780063	2.080657663	-2.94341647	-1.64385619
Aradu.G0QWJ	0.097610797	0.014355293	-0.28630419	-0.34007544	2.028569152

Aradu.1EH3D	-0.32192809	-0.88896869	-0.35845397	NA	-1.78587519
Aradu.22BDB	2.859969548	3.447579197	3.106013238	4.019701914	3.963474124
Aradu.69JL5	-1.32192809	-1.25153877	-1.73696559	-2.64385619	-1.32192809
Aradu.I2A4P	2.82984956	1.163498732	1.713695815	3.412781525	2.572889668
Aradu.F66UW	2.084064265	2.666756592	1.948600847	2.558267634	2.386810946
Aradu.67MY8	NA	NA	NA	NA	-5.64385619
Aradu.W4S0X	2.438292852	2.737686761	2.307428525	3.145677455	3.381283373
Aradu.48ATB	1.613531653	1.5360529	1.937344392	4.56315813	4.91981677
Aradu.7FN1T	-1.88896869	-0.91593574	-0.94341647	0.137503524	0.773996325
Aradu.VK984	1.13093087	1.541019153	1.298658316	1.269033146	1.782408565
Aradu.ST5ZJ	-4.32192809	-5.64385619	-5.05889369	-2.32192809	-1.18442457
Aradu.JKM1G	NA	NA	NA	NA	NA
Aradu.T698T	2.845991771	3.200064862	2.897240426	4.511594542	4.659924558
Aradu.JAF9B	0.411426246	0.35614381	-1.35845397	1.646162657	2.608809243
Aradu.E0HSZ	-0.16812276	0.163498732	0.150559677	-1.73696559	-2.18442457
Aradu.CA5TC	0	0.286881148	0.443606651	1.40599236	1.427606173
Aradu.2V3YU	2.244887059	2.634593268	2.304511042	3.314696526	3.56193706
Aradu.Q1NTT	-3.32192809	-3.47393119	-3.05889369	-4.05889369	2.914564523
Aradu.22KJ4	-2.94341647	-1.83650127	-2.32192809	-4.05889369	-1.28630419
Aradu.JRR3K	5.906890596	5.060047384	5.246027981	2.771885579	2.709290636
Aradu.FWS4A	6.188242656	5.813524689	5.29351763	6.150965784	6.717676423
Aradu.IEX64	-3.83650127	-3.64385619	-3.64385619	-4.32192809	NA

Aradu.ETZ8K	-0.81096618	-0.39592868	-0.26881676	1.883620816	2.389566812
Aradu.JA3L7	NA	NA	NA	NA	NA
Aradu.S3YJN	2.298658316	1.589763487	1.50589093	-3.64385619	-0.64385619
Aradu.V2IK2	1.361768359	2.124328135	1.575312331	2.709290636	2.776103988
Aradu.P2130	NA	-1.94341647	-3.05889369	-4.64385619	-1.59946207
Aradu.WQL62	NA	NA	NA	-0.66657627	1.3950628
Aradu.F6ABZ	-4.05889369	-6.64385619	-6.64385619	-2.47393119	-0.13606155
Aradu.CW4J0	1.521050737	1.516015147	1.327687364	1.3950628	2.066950244
Aradu.7GC8S	0.879705766	0.847996907	0.659924558	0.855989697	1.584962501
Aradu.3MT4E	1.416839742	1.599317794	1.333423734	1.752748591	2.3950628
Aradu.X0F3K	0.565597176	0.333423734	-0.02914635	-1.51457317	-1.64385619
Aradu.DDQ1E	NA	NA	NA	NA	0.443606651
Aradu.80WBV	2.280956314	1.669026766	2.361768359	NA	-3.64385619
Aradu.YXG3J	4.873321063	4.862947248	4.902073579	1.782408565	2.176322773
Aradu.DNJ9W	-0.07400058	1.263034406	0.111031312	2.948600847	2.904965719
Aradu.R27T4	-3.05889369	-3.32192809	-5.64385619	-0.08926734	-0.07400058
Aradu.N76GN	2.827819025	3.410069692	2.639232163	4.495695163	4.442943496
Aradu.V0VX5	3.440952198	3.621758857	3.473786912	3.745237332	4.150559677
Aradu.L5IHI	2.301587647	1.937344392	1.929790998	2.025028794	2.414135533
Aradu.NTD67	0.333423734	1.565597176	0.941106311	3.418189948	3.596935142
Aradu.JJW6C	4.373648211	4.680886921	4.376429311	4.833902077	5.215678597
Aradu.83I6G	4.315421316	4.503348735	4.186659017	3.19061486	3.220329955

Aradu.V5FRM	2.166715445	4.125981654	4.341274184	2.419538892	3.592158002
Aradu.NAJ8T	2.358958826	2.411426246	2.150559677	2.94673086	3.71918344
Aradu.X4CJ9	-0.62148838	-0.86249648	-0.55639335	-2.18442457	-0.81096618
Aradu.90H62	2.166715445	2.678071905	2.498250868	2.389566812	2.498250868
Aradu.NFI11	-0.68965988	-0.25153877	0.250961574	-0.83650127	-0.45403163
Aradu.P3XD1	-0.21759144	-0.07400058	-0.577767	1.718087584	1.879705766
Aradu.YZ9KD	-6.64385619	-3.83650127	-5.05889369	0.333423734	0.137503524
Aradu.4R7ZC	2.632268215	2.543495883	2.634593268	1.500802053	2.017921908
Aradu.GG6GL	NA	-6.64385619	NA	-5.64385619	-3.18442457
Aradu.H9NK1	-0.34007544	-0.37706965	-0.20091269	-0.66657627	-0.577767
Aradu.DH7CT	-1.39592868	-1	-1.25153877	0.50589093	0.565597176
Aradu.4U6IV	NA	NA	NA	-6.64385619	-6.64385619
Aradu.W48TA	3.407352751	3.837943242	3.246408087	3.673556424	3.839959587
Aradu.EU5HZ	-0.91593574	-0.97143085	-1.15200309	0.176322773	0.443606651
Aradu.U61F3	2.283921772	3.454175893	3.260025656	4.267535798	4.510961919
Aradu.4118A	3.344828497	4.292045492	3.341985747	4.621758857	4.666756592
Aradu.QV720	-3.83650127	-4.05889369	-5.64385619	NA	-0.97143085
Aradu.NBQ7X	0.545968369	1.608809243	0.722466024	1.744161096	1.731183242
Aradu.RDK4X	NA	-6.64385619	NA	NA	-2.05889369
Aradu.A16D8	NA	NA	-5.64385619	NA	-3.83650127
Aradu.UAQ2R	1.713695815	1.63691458	1.275007047	2.869871406	3.389566812
Aradu.G1DZV	1.084064265	1.851998837	1.709290636	1.790772038	2.114367025

Aradu.M0AKN	3.600507645	3.634593268	3.292781749	4.459431619	4.714795011
Aradu.F54T9	-2.47393119	-1.94341647	-2.55639335	-0.47393119	-0.4150375
Aradu.E8BQZ	2.475084883	2.925999419	2.974529312	2.140778656	2.435628594
Aradu.F4Y7W	2.518535139	2.809414444	2.538538164	2.580145484	3.19061486
Aradu.7I7Y0	5.395405605	5.401220862	5.047887329	5.762083004	5.995032192
Aradu.EI8LI	1.541019153	2.403267722	2.50589093	0.495695163	1.372952098
Aradu.DAC70	1.726831217	1.604071324	1.480265122	2.857980995	3.358958826
Aradu.K2M4L	-0.97143085	0.5360529	-0.73696559	-0.59946207	-0.68965988
Aradu.YLV00	2.49057013	3.86393845	4.475084883	5.006746832	4.496334513
Aradu.NX4M4	2.565597176	2.752748591	2.510961919	2.974529312	3.189033824
Aradu.UPE5A	NA	NA	NA	NA	-6.64385619
Aradu.671CI	-3.47393119	-4.32192809	-5.05889369	NA	-6.64385619
Aradu.YR4CG	1.560714954	1.811471031	1.744161096	2.358958826	2.713695815
Aradu.BUC40	6.807998839	6.509695842	7.00337736	1.280956314	1.195347598
Aradu.IM8LC	2.615887074	3.09592442	2.675815931	3.631104282	3.905928478
Aradu.RV5XK	3.915520901	3.67468662	3.703765179	3.888499736	4.08236197
Aradu.N7F34	8.065712318	7.43721109	7.729348832	2.961623328	3.084064265
Aradu.5Q0IP	1.875780063	2.007195501	2.003602237	1.843983844	2.166715445
Aradu.25F61	2.301587647	2.40599236	2.117695043	2.482848283	2.849999259
Aradu.J6F5H	-1.64385619	-1.25153877	-1.59946207	-0.53533173	-0.45403163
Aradu.T3ZAX	0.084064265	0.40053793	0.411426246	3.269033146	1.748461233
Aradu.QEK0G	NA	NA	NA	NA	NA

Aradu.R205B	0.613531653	1.13093087	0.855989697	1.516015147	1.752748591
Aradu.PC4II	2.805292456	2.859969548	2.950468414	2.709290636	2.869871406
Aradu.HLK79	1.875780063	2.289834465	1.959770155	2.790772038	3.327687364
Aradu.LAA50	NA	-6.64385619	-4.64385619	0.622930351	1.169925001
Aradu.3NG75	2.817623258	3.375734539	2.841973119	2.292781749	2.419538892
Aradu.PX3ED	3.631104282	3.488000771	3.326249701	3.155425432	3.478971805
Aradu.F2ZNU	-1.78587519	-1.25153877	-1.35845397	-0.32192809	0.176322773
Aradu.9HV89	-1.88896869	-0.62148838	-0.23446525	0.98550043	0.411426246
Aradu.W51GD	3.193771743	4.782408565	4.217230716	5.971083658	5.487679279
Aradu.E1TLU	2.124328135	2.794935663	2.456806149	2.042644337	2.339137385
Aradu.N1KEX	0.604071324	0.367371066	-0.25153877	-0.83650127	1.731183242
Aradu.33Q41	3.367371066	4.12763328	3.739848103	4.173127433	3.549669171
Aradu.C5KI2	3.060047384	3.439623138	3.414135533	2.803227036	3.472487771
Aradu.3VD4A	-0.43440282	0.056583528	-0.71311885	-1.28630419	-0.64385619
Aradu.K7HN9	-0.59946207	-0.4150375	-1.25153877	-4.64385619	-1.73696559
Aradu.VXG8Q	1.40599236	2.189033824	1.124328135	3.323370069	3.423578171
Aradu.6WI58	4.745775149	4.782408565	4.552131108	5.671859468	5.803743669
Aradu.IZ7V8	-1.73696559	-2.25153877	-1.51457317	-2.32192809	-1.47393119
Aradu.E8VVL	NA	NA	NA	NA	NA
Aradu.YX3TI	-0.45403163	2.266036894	1.786596362	4.500802053	3.313245852
Aradu.WAB2C	-0.39592868	-0.64385619	-1.39592868	-2.73696559	-1.55639335
Aradu.8C9LT	2.967168608	2.989139007	2.845991771	3.051372102	3.768713657

Aradu.L72JH	-4.32192809	-5.05889369	-5.05889369	0.50589093	0.713695815
Aradu.95EP3	NA	NA	NA	NA	NA
Aradu.Q26PA	1.304511042	1.232660757	0.799087306	1.799087306	2.121015401
Aradu.P0G7F	NA	-4.32192809	-4.64385619	-5.64385619	-5.05889369
Aradu.T0LS0	2.100977648	2.687060688	3.367371066	-0.577767	0.070389328
Aradu.K3I53	NA	NA	NA	NA	NA
Aradu.EUM6N	1.887525271	1.851998837	1.580145484	2.805292456	2.778208576
Aradu.Q51NH	1.952333566	2.503348735	2.028569152	2.835924074	3.246408087
Aradu.47F2N	3.420886575	3.814550423	3.49057013	4.272023189	4.621758857
Aradu.9L879	0.35614381	0.807354922	0.310340121	1.40599236	1.790772038
Aradu.E2VTX	-4.64385619	-3.83650127	-4.64385619	-3.83650127	-3.83650127
Aradu.56GAP	NA	NA	NA	NA	NA
Aradu.B049P	1.773996325	1.918386234	1.321928095	0.516015147	1.871843649
Aradu.BK33F	1.769771739	1.937344392	2.063502942	1.786596362	1.90303827
Aradu.8Y6AX	-1.12029423	-0.34007544	-0.71311885	1.891419187	1.970853654
Aradu.6GP3J	6.108106224	6.356672044	5.869377924	7.291953434	7.163498732
Aradu.TT65B	-0.05889369	0.815575429	0.464668267	2.003602237	1.875780063
Aradu.2123V	1.570462931	1.726831217	1.389566812	4.026800059	3.390942773
Aradu.ZD4XA	-1.47393119	-0.51457317	-1.32192809	1.257010618	1.722466024
Aradu.JR9SL	2.9800253	3.489286023	2.948600847	4.954661621	5.033423002
Aradu.X6LF1	6.981281854	7.158357226	7.048323387	7.98230913	7.646090636
Aradu.901R7	7.102028179	6.253043795	6.786727035	-0.43440282	-0.28630419
Aradu.L3QY	NA	NA	-6.64385619	-5.64385619	-6.64385619

1

Aradu.RTQ8K	0.201633861	0.475084883	-1.08926734	3.026800059	3.324810603
Aradu.P59ZT	1.778208576	2	1.895302621	2.23878686	2.639232163
Aradu.Q7TCS	4.257010618	4.465321514	4.509062386	5.329482431	5.040015679
Aradu.Y5WGV	3.495695163	4.723012396	4.622344723	4.340562269	4.378511623
Aradu.598J8	0.933572638	0.286881148	-0.12029423	-2.55639335	-3.18442457
Aradu.83RRW	-0.43440282	0.084064265	-0.43440282	2.010779839	2.077242999
Aradu.0QJ0H	2.313245852	2.408711861	2.336283388	1.007195501	1.077242999
Aradu.QNE8S	-1.68965988	-0.68965988	-1	-1.59946207	-0.71311885
Aradu.JL69F	-4.64385619	-2.25153877	-3.64385619	-2.55639335	-3.83650127
Aradu.VV69K	-4.05889369	-2	-2.94341647	0.150559677	0.50589093
Aradu.YQ9K5	-4.64385619	-4.05889369	-3.32192809	-3.64385619	-2.55639335
Aradu.NKE3U	1.207892852	1.23878686	0.831877241	1.021479727	1.695993813
Aradu.9G7GM	3.534808661	3.537296067	3.152183419	4.277984747	3.866907978
Aradu.Q9ZWS	-2.73696559	-2.25153877	-3.18442457	-1.78587519	-1
Aradu.7W7QL	5.029011087	5.034743949	5.032100843	4.887525271	5.00270252
Aradu.D814J	2.430285273	1.839959587	2.44625623	2.266036894	2.250961574
Aradu.X8LK6	NA	-6.64385619	NA	-4.32192809	-2
Aradu.1FM9H	-5.05889369	-4.05889369	-5.64385619	-2.18442457	-3.47393119
Aradu.ZIW9T	-0.02914635	-0.34007544	-0.49410907	-0.47393119	-0.34007544
Aradu.BLK03	NA	-6.64385619	NA	-4.32192809	-2.55639335
Aradu.8E0AS	-4.64385619	-4.05889369	-4.64385619	-4.05889369	-5.05889369
Aradu.188J4	1.150559677	2.247927513	1.475084883	3.590961241	3.684818738

Aradu.MRR38	2.733354341	3.137503524	3.025028794	2.970853654	3.148934105
Aradu.ZT2TE	-1.35845397	-1.15200309	-1.73696559	-4.05889369	-1.59946207
Aradu.UGC9M	-1.73696559	-1.28630419	-1.78587519	0.22650853	-0.25153877
Aradu.ZZU4A	0.86393845	1.163498732	0.773996325	0.464668267	1.263034406
Aradu.5LG80	4.581953751	4.492494152	4.6622055	1.851998837	1.59454855
Aradu.E9QI4	1.618238656	2.03562391	1.906890596	2.731183242	2.937344392
Aradu.T1J1B	3.343407822	3.324810603	3.326249701	2.693765712	3.160274831
Aradu.AH5QJ	6.12763328	5.652773285	6.563615766	-0.34007544	-0.34007544
Aradu.I7IYL	-2.73696559	-2.39592868	-4.64385619	0.250961574	0.831877241
Aradu.PXZ8J	1.944858446	2.427606173	2.269033146	2.754887502	3.137503524
Aradu.AU74Y	-4.05889369	-3.18442457	-4.32192809	-4.64385619	-1.64385619
Aradu.KV0LC	1.367371066	1.655351829	1.650764559	1.480265122	1.744161096
Aradu.I38Z6	-0.71311885	-0.30400619	-0.68965988	0.389566812	0.454175893
Aradu.ZY82G	1.580145484	1.691534165	1.150559677	2.912649865	2.548436625
Aradu.V8FCP	1.459431619	2.056583528	1.86393845	2.46727948	2.750606505
Aradu.RL2YG	NA	NA	NA	NA	NA
Aradu.GIN82	3.859969548	3.304511042	3.250961574	3.066950244	2.121015401
Aradu.PZB3C	3.49057013	3.887525271	4.029452886	2.565597176	2.073820233
Aradu.35U3T	6.291124643	5.850999395	5.839707698	2.344828497	2.853995647
Aradu.FHT7B	2.40599236	1.89917563	1.867896464	-0.37706965	2.182692298
Aradu.GV115	2.107687869	3.608809243	3.295723025	2.875780063	2.963474124
Aradu.81GP	-2	-1.39592868	-1.59946207	-2.64385619	-0.13606155

B					
Aradu.EQ6UY	NA	-5.64385619	-6.64385619	-3.47393119	-5.64385619
Aradu.1WD61	0.422233001	1.646162657	1.182692298	2.150559677	2.430285273
Aradu.R4ZCN	0.757023247	1.15704371	0.970853654	2.364572432	2.198494154
Aradu.19YSM	NA	NA	NA	-3.64385619	-4.05889369
Aradu.W56R3	2.150559677	2.077242999	1.879705766	1.03562391	1.13093087
Aradu.ILS90	4.067810784	4.114367025	3.94016675	3.3305584	3.381283373
Aradu.EZ30H	1.454175893	1.722466024	1.516015147	3.066950244	3.279471296
Aradu.7AK58	1.682573297	1.739848103	1.565597176	1.163498732	1.883620816
Aradu.9B60Q	0.150559677	0.028569152	-0.23446525	0.807354922	1.372952098
Aradu.7C3KS	3.625270489	3.937344392	3.478971805	4.699884729	4.860466259
Aradu.GAW9B	2.582556003	2.748461233	2.761285273	2.263034406	4.084064265
Aradu.90SID	NA	NA	NA	NA	NA
Aradu.PJ0EM	NA	-6.64385619	-4.32192809	-6.64385619	0.163498732
Aradu.YG6N5	1.163498732	1.748461233	1.608809243	2.711494907	2.9800253
Aradu.KH5IZ	-4.32192809	-5.05889369	-3.32192809	-2.83650127	-3.47393119
Aradu.BHH9A	0.622930351	1.111031312	0.918386234	1.608809243	1.90303827
Aradu.54IZY	-0.16812276	1.111031312	0.042644337	2.03562391	1.974529312
Aradu.X33LT	0.669026766	0.275007047	0.59454855	-1.32192809	1.214124805
Aradu.D45R1	NA	-5.05889369	-6.64385619	NA	-4.05889369
Aradu.TN13P	NA	NA	NA	NA	-3.83650127
Aradu.KS96H	NA	NA	NA	NA	NA
Aradu.7Z02Q	1.163498732	1.480265122	0.516015147	3.768713657	3.814550423

Aradu.V060T	0.378511623	0.526068812	0.344828497	1.560714954	2.007195501
Aradu.C2VRV	-2.25153877	-1.39592868	-1.83650127	0.622930351	0.910732662
Aradu.AX4BJ	NA	NA	-6.64385619	NA	-5.05889369
Aradu.ZUQ2N	3.734438666	3.738767837	3.370164281	2.286881148	2.414135533
Aradu.W2FE7	2.629939409	2.952333566	2.63691458	3.749534268	4.001802243
Aradu.N9F03	3.403267722	3.448900951	3.63691458	3.364572432	4.116031993
Aradu.AF2FZ	2.201633861	2.475084883	2.070389328	2.462052319	2.918386234
Aradu.8G40J	3.724650272	3.814550423	3.935459748	3.386810946	3.237257771
Aradu.LF3E5	5.525129251	5.211401637	5.462052319	1.560714954	2.124328135
Aradu.K9UV7	2.454175893	3.334854269	3.303050085	4.199279721	4.160274831
Aradu.K3S1H	-0.88896869	-0.76121314	-1.39592868	1.150559677	1.516015147
Aradu.FD1ZK	2.761285273	2.757023247	2.811471031	2.117695043	2.553360503
Aradu.U5BRX	3.66448284	3.757023247	3.934516502	4.720825666	5.647026633
Aradu.HSJ3A	NA	NA	NA	-4.64385619	0.526068812
Aradu.ZAY9P	4.405311683	4.294988268	4.474436043	2.582556003	2.702657543
Aradu.FRY2U	1.077242999	1.176322773	1.321928095	0.411426246	0.847996907
Aradu.V6MI1	0.214124805	0.82374936	-0.47393119	0.59454855	0.443606651
Aradu.S0S2R	2.648465443	3.124328135	2.881664619	1.389566812	1.914564523
Aradu.XM1WL	-0.73696559	-0.55639335	-0.59946207	-0.10469738	-0.07400058
Aradu.YH6P6	NA	-6.64385619	-6.64385619	-3.05889369	-1.94341647
Aradu.VB76D	4.929790998	4.803227036	4.954661621	3.778208576	4.548436625
Aradu.1M2X1	11.59324638	10.87446656	11.29508013	8.368986552	8.628008017

Aradu.1WP35	-0.64385619	-1.47393119	-1.68965988	-3.18442457	-1.64385619
Aradu.GM25T	3.632268215	3.957914599	3.424922088	5.091699834	5.121015401
Aradu.Y1Z0R	1.13093087	1.914564523	2.010779839	2.659924558	2.994579724
Aradu.52M2A	0.713695815	0.918386234	0.495695163	-0.15200309	0.263034406
Aradu.XR1NY	1.782408565	2.6622055	1.632268215	3.351910961	3.443606651
Aradu.SL6TQ	-2.73696559	-2	-2.94341647	1.014355293	0.678071905
Aradu.3L1J2	5.023698917	5.240695944	5.025471815	6.003152449	6.11914864
Aradu.XAX6Y	1.097610797	1.111031312	0.925999419	2.195347598	1.794935663
Aradu.KH0NV	-0.01449957	0.584962501	0.555816155	0.321928095	0.389566812
Aradu.9VB4E	2.44625623	2.475084883	2.575312331	2.693765712	3.166715445
Aradu.694KT	6.131959823	5.966707314	6.045486656	2.44625623	2.63691458
Aradu.R2ERN	2.859969548	2.469885976	2.204766751	2.364572432	3.675815931
Aradu.PZ2PD	1.510961919	1.09085343	0.722466024	3.192194165	3.23572706
Aradu.GH306	0.97819563	0.23878686	0.124328135	-2.83650127	-1.47393119
Aradu.T4VLF	3.656496371	3.477677328	3.764473551	2.733354341	2.817623258
Aradu.P7Y6N	3.54225805	4.050501697	3.965322548	3.289834465	4.018812186
Aradu.942ZP	8.440329353	8.294391002	7.299024807	1.608809243	1.555816155
Aradu.6ZR5R	4.765004246	4.733354341	4.749534268	4.026800059	4.393690764
Aradu.JI08P	-6.64385619	-6.64385619	-5.05889369	-4.64385619	-4.05889369
Aradu.JY1UA	1.286881148	1.014355293	0.526068812	0.298658316	0.650764559
Aradu.VTB62	9.161308272	8.796526375	9.450654536	5.128458384	5.271649772
Aradu.5IY98	4.82374936	7.817047599	7.023588039	4.915520901	4.680886921

Aradu.SC5U0	1.956056652	2.247927513	1.887525271	2.301587647	2.575312331
Aradu.6M9JS	3.260025656	3.174725988	3.080657663	3.889473543	3.671293372
Aradu.F9BJN	3.422233001	3.258518925	3.215678597	1.735522177	1.790772038
Aradu.B1CPC	2.310340121	2.748461233	2.521050737	2.40599236	2.601696516
Aradu.EK3X2	1.021479727	2.508428653	1.35614381	3.13093087	2.998195503
Aradu.GXP8K	-0.62148838	-0.23446525	-0.76121314	-0.4150375	0.389566812
Aradu.GNT02	2.286881148	2.784503983	2.341985747	2.280956314	2.773996325
Aradu.HNU76	2.370164281	2.742006211	2.704871964	3.622930351	3.697106574
Aradu.2597P	2.477677328	3.028569152	3.377123749	2.056583528	2.23878686
Aradu.VA62W	8.804001915	8.361549062	8.787053666	5.253232939	5.346602375
Aradu.8IX7E	0.933572638	1.933572638	1.627606838	2.080657663	2.077242999
Aradu.76BI5	-1.18442457	-0.68965988	-3.64385619	1.555816155	1.883620816
Aradu.H9FC2	2.521050737	3.371558863	3.032100843	3.532316959	3.528571319
Aradu.HQF0H	NA	NA	NA	-5.64385619	NA
Aradu.RP6HI	1.673556424	2.087462841	1.82374936	2.032100843	2.403267722
Aradu.D9QX6	-2.94341647	-3.47393119	-4.05889369	-2.47393119	-1.59946207
Aradu.I9SXC	2.344828497	2.22650853	2.17951105	2.922197848	3.122672719
Aradu.T8C1S	-2.39592868	-3.05889369	-3.05889369	-4.32192809	0.887525271
Aradu.617EV	NA	-5.64385619	NA	NA	-5.05889369
Aradu.162IL	3.713695815	3.655351829	3.602884409	3.195347598	3.209453366
Aradu.TR8YP	3.282439805	3.601696516	3.177917792	3.87774425	4.017031081
Aradu.N2UA7	NA	NA	NA	NA	-5.64385619

Aradu.Z83RP	-2	-1.59946207	-2.18442457	-1.83650127	-0.71311885
Aradu.LP1C0	1.269033146	1.819668183	1.531069493	2.44625623	2.769771739
Aradu.TUT46	-1.83650127	-1.47393119	-3.18442457	0.918386234	1.565597176
Aradu.K38DA	0.124328135	0.454175893	0.214124805	1.056583528	2.13093087
Aradu.D8UDW	-3.64385619	-3.32192809	-3.83650127	1.49057013	1.744161096
Aradu.L4IQ2	-2	-1.94341647	-1.83650127	0.084064265	1.070389328
Aradu.XT8CD	3.713695815	3.888499736	3.671293372	3.3950628	3.84899843
Aradu.M1AX1	5.521364878	6.471350063	6.092968509	5.284292026	5.241077458
Aradu.1E9H5	0.622930351	0.748461233	0.432959407	1.948600847	2.060047384
Aradu.ZAA79	2.195347598	2.286881148	2.392317423	1.485426827	1.650764559
Aradu.HS65R	1.516015147	1.704871964	1.565597176	1.90303827	1.608809243
Aradu.S16C5	NA	NA	NA	NA	-2.73696559
Aradu.6KC8S	NA	NA	NA	-2.64385619	-3.05889369
Aradu.R9TKV	2.448900951	2.935459748	2.952333566	-0.62148838	1.40053793
Aradu.GFE68	-3.05889369	-2.32192809	-3.18442457	0.298658316	0.150559677
Aradu.EZJ0T	-2.55639335	-0.81096618	-0.64385619	NA	-4.32192809
Aradu.83253	-0.88896869	-1.12029423	-0.91593574	2.257010618	1.871843649
Aradu.DB14S	5.045705065	4.998646839	4.873813198	1.448900951	2.097610797
Aradu.7U8XS	NA	-5.64385619	-5.64385619	-5.64385619	-4.32192809
Aradu.MS73B	0.367371066	0.933572638	0.485426827	2.44625623	2.634593268
Aradu.9Y73E	1.967168608	3.87774425	3.606442228	1.378511623	1.339137385
Aradu.C5UHL	-1.78587519	-1.05889369	-2.73696559	1.627606838	2.5360529

Aradu.4V0GE	-3.47393119	-2.47393119	-3.18442457	0.90303827	1.03562391
Aradu.JV6V7	-0.07400058	0.941106311	0.056583528	1.910732662	1.769771739
Aradu.FU781	-5.64385619	-6.64385619	-5.64385619	-3.64385619	-5.05889369
Aradu.6AI81	4.137503524	4.489928219	5.248686627	5.22342255	5.200849575
Aradu.8J5I0	-0.43440282	0.189033824	-1.08926734	1.941106311	3.046141782
Aradu.TZG12	0.847996907	1.063502942	0.475084883	2.456806149	2.411426246
Aradu.N68GL	-2.12029423	-1.59946207	-3.05889369	0.286881148	1.757023247
Aradu.T5RWU	-3.32192809	NA	NA	-0.51457317	-0.49410907
Aradu.PA9WC	2.080657663	2.726831217	2.456806149	3.142413438	3.277984747
Aradu.W4PMX	NA	NA	NA	NA	0.895302621
Aradu.YP0M1	0.454175893	0.815575429	0.028569152	0.622930351	0.839959587
Aradu.80TC2	-3.83650127	-3.32192809	-3.83650127	-3.05889369	-2.39592868
Aradu.24ZSJ	-1.05889369	-1.51457317	-0.59946207	-0.12029423	1.13093087
Aradu.JKK33	-3.64385619	-5.05889369	NA	-2.47393119	-4.64385619
Aradu.HG6CP	1.709290636	2.140778656	1.819668183	2.508428653	2.632268215
Aradu.4YA8F	-0.577767	0.782408565	-0.34007544	0.963474124	1.042644337
Aradu.V3IQ0	NA	-6.64385619	-6.64385619	-5.64385619	-4.64385619
Aradu.ER2GU	1.948600847	2.09085343	1.570462931	3.430285273	3.422233001
Aradu.Y28R7	0.659924558	2.454175893	1.454175893	1.963474124	1.084064265
Aradu.TZ1M5	1.007195501	0.933572638	0.82374936	5.960928667	4.139142019
Aradu.DN8FJ	1.480265122	1.744161096	1.50589093	1.531069493	1.811471031
Aradu.9Z8LC	-1.73696559	-2	-2.32192809	-5.64385619	-2.25153877

Aradu.R95HC	NA	NA	NA	-0.21759144	-0.51457317
Aradu.PDS8K	-4.64385619	-2.18442457	-3.05889369	0.214124805	-1.15200309
Aradu.9L539	NA	NA	NA	NA	-4.05889369
Aradu.C7JM0	-5.64385619	-5.64385619	-6.64385619	-3.64385619	-2.32192809
Aradu.LRZ4G	-2.25153877	-1.73696559	-2.12029423	-4.64385619	-2.94341647
Aradu.296X5	0.565597176	0.669026766	0.23878686	3.565597176	3.933572638
Aradu.W2C81	6.17851547	4.680324357	4.931683057	5.025471815	3.152183419
Aradu.W0R38	3.168321116	3.493134922	4.112700133	2.89917563	2.541019153
Aradu.G9W58	-1.43440282	-2.12029423	-3.47393119	-2.83650127	1.646162657
Aradu.TE7Z6	NA	NA	NA	NA	NA
Aradu.YC3N7	3.639232163	4.493134922	3.832890014	5.466300829	5.424250286
Aradu.58DAR	9.726831217	9.664020546	10.07761687	6.613236955	6.50191678
Aradu.CZ128	1.910732662	1.49057013	1.632268215	-2.73696559	-1.47393119
Aradu.4BV7T	3.468583317	3.802193217	3.3305584	2.64385619	2.769771739
Aradu.L5CRG	9.793473258	9.697158714	9.817543319	5.065658471	5.476705707
Aradu.SFF9P	-1.47393119	-1.55639335	-2.12029423	0.298658316	1.250961574
Aradu.HM1JQ	2.15704371	2.124328135	2.049630768	2.260025656	2.077242999
Aradu.JK25B	1.40053793	1.182692298	0.82374936	1.526068812	2.655351829
Aradu.CJ510	0.970853654	1.189033824	0.963474124	1.384049807	1.709290636
Aradu.M3KDI	2.327687364	2.589763487	2.386810946	2.319039816	2.773996325
Aradu.9TU4F	2.625270489	2.809414444	2.965322548	2.347665656	3.608809243
Aradu.6B67D	2.906890596	2.671293372	2.464668267	2.606442228	2.618238656
Aradu.XZ0H	-4.32192809	-5.05889369	-4.32192809	NA	-2.18442457

K					
Aradu.T8M8U	1.14404637	1.35614381	1.150559677	2.23572706	2.528571319
Aradu.NCD56	5.260778432	4.922197848	4.82374936	2.493134922	2.577730931
Aradu.55F3D	NA	NA	NA	-5.64385619	-4.32192809
Aradu.N8P27	3.375734539	3.465974465	3.152183419	3.83491343	4.011674533
Aradu.YP97N	-4.64385619	-6.64385619	-6.64385619	-1.88896869	-2
Aradu.464FR	-4.32192809	-6.64385619	-4.05889369	-2.25153877	-3.18442457
Aradu.MW6JM	1.769771739	2.364572432	2.214124805	3.665620164	3.566815154
Aradu.P5PIT	-5.05889369	-4.64385619	-5.05889369	-1.94341647	-1.88896869
Aradu.99LXR	1.014355293	1.622930351	1.361768359	1.042644337	1.344828497
Aradu.5I3G5	-1.55639335	-2	-1.88896869	-1.83650127	-1.15200309
Aradu.TI2TK	-3.83650127	-1.28630419	-2.94341647	1.367371066	1.49057013
Aradu.X4JYD	1.432959407	1.5360529	1.049630768	2.941106311	3.427606173
Aradu.M0VKX	2.908812908	3.333423734	2.841973119	4.056583528	4.454833907
Aradu.J3P01	2.169925001	2.765534746	2.397802962	2.89917563	3.187451054
Aradu.TK9TC	NA	NA	NA	NA	NA
Aradu.VU792	-3.47393119	-3.47393119	-2.83650127	-2.94341647	0.124328135
Aradu.UJJ9I	4.238022518	4.115199749	3.951401292	3.09085343	2.691534165
Aradu.C6W6J	3.195347598	3.228049048	3.182692298	2.897240426	3.360364277
Aradu.RV62R	-4.05889369	-4.05889369	-3.32192809	-5.64385619	-3.18442457
Aradu.D6W9N	1.90303827	1.855989697	2.414135533	3.849999259	4.075532631
Aradu.75D3M	3.839959587	3.714795011	3.820689561	0.545968369	0.704871964
Aradu.F8DL0	NA	-6.64385619	-4.64385619	-4.05889369	-2.32192809

Aradu.T1G99	-1.25153877	-0.43440282	-1.28630419	1.691534165	1.510961919
Aradu.0E5MF	4.318316841	4.232660757	4.296457407	2.049630768	2.114367025
Aradu.TL6UB	0.545968369	0.622930351	0.250961574	0.807354922	1.169925001
Aradu.86IQQ	-5.64385619	NA	NA	2.693765712	2.403267722
Aradu.YBC8E	-0.37706965	0.201633861	-0.28630419	0.432959407	0.815575429
Aradu.5U0XL	-4.32192809	-5.64385619	NA	NA	NA
Aradu.YJS53	2.408711861	2.22342255	1.627606838	2.280956314	1.875780063
Aradu.F4UZI	2.22650853	2.09085343	2.232660757	-0.30400619	1.560714954
Aradu.QGW0X	-0.577767	-0.15200309	-1.15200309	0.422233001	1.831877241
Aradu.C61GI	1.214124805	1.731183242	0	3.547203025	4.338424415
Aradu.NVM7U	0.084064265	-0.08926734	-0.35845397	-1.32192809	1.084064265
Aradu.RBU21	2.456806149	2.845991771	2.541019153	2.289834465	2.531069493
Aradu.BGJ4E	2.622930351	2.731183242	2.381283373	3.368768349	3.554588852
Aradu.E710C	1.137503524	0.871843649	0.389566812	3.377123749	3.401903472
Aradu.Z8K87	0.815575429	0.432959407	0.097610797	1.811471031	2.077242999
Aradu.461JS	NA	NA	NA	NA	NA
Aradu.WL7ND	2.545968369	2.784503983	2.498250868	3.220329955	3.22650853
Aradu.L6U41	NA	NA	NA	NA	-0.37706965
Aradu.YNN2E	3.971773447	4.838447593	4.311793718	4.19061486	4.271276259
Aradu.S0JY2	1.169925001	1.722466024	1.545968369	2.23572706	1.925999419
Aradu.V2YPR	1.86393845	2.339137385	2.173127433	3.092545742	3.484138131
Aradu.80EY	2.722466024	2.608809243	2.528571319	1.137503524	0.941106311

C					
Aradu.95FMB	-1.15200309	0.782408565	-0.94341647	2.456806149	2.671293372
Aradu.T5PPZ	NA	-6.64385619	-5.64385619	NA	NA
Aradu.124FF	NA	NA	NA	NA	NA
Aradu.BI5JF	NA	-6.64385619	NA	NA	NA
Aradu.EA94B	0.757023247	1.310340121	0.815575429	1.070389328	1.286881148
Aradu.N3CWH	-1.18442457	-0.78587519	-1.83650127	-2.32192809	-0.73696559
Aradu.ZW38I	NA	NA	NA	NA	-3.32192809
Aradu.03GCY	-2.55639335	-2	-3.18442457	0.933572638	0.757023247
Aradu.KCP9Z	3.280956314	3.691534165	3.124328135	5.577126661	5.5360529
Aradu.LY8JJ	2.704871964	3.014355293	2.541019153	3.666756592	3.803227036
Aradu.W24J0	0.831877241	1.5360529	1.163498732	3.523561956	3.364572432
Aradu.0YT2V	-0.04394335	0.669026766	0.367371066	0.641546029	0.687060688
Aradu.ZN9Y3	-0.94341647	-1.59946207	-0.88896869	-0.76121314	0.23878686
Aradu.GEA65	NA	NA	NA	-3.32192809	-4.32192809
Aradu.0KQ9H	0.613531653	2.077242999	1.678071905	0.895302621	1.189033824
Aradu.Q7KU7	4.207892852	4.671859468	4.50779464	2.283921772	3.145677455
Aradu.DIB1B	-1.55639335	-1.25153877	-1.78587519	-1.08926734	-0.68965988
Aradu.6JB9C	4.628773595	4.94016675	4.750606505	1.989139007	1.851998837
Aradu.MVP73	2.3950628	2.680324357	2.526068812	2.759155834	3.181102551
Aradu.VI09T	-2.47393119	-2.25153877	-1.83650127	-2.73696559	-0.88896869
Aradu.M566R	-0.88896869	-0.64385619	-2	2.260025656	2.887525271
Aradu.P5027	0.815575429	1.378511623	1.111031312	2.336283388	2.432959407

Aradu.3AT2D	-1.51457317	-0.16812276	-1	1.941106311	1.819668183
Aradu.M5IDE	4.71259578	4.561326136	4.396433531	3.615887074	3.451540833
Aradu.ZT2KF	-1.43440282	-1.51457317	-2.12029423	-2.25153877	-1.59946207
Aradu.6FP0C	NA	NA	NA	NA	-5.64385619
Aradu.I9SZF	1.803227036	2.381283373	1.906890596	2.757023247	3.056583528
Aradu.ZUH0W	0.070389328	0.545968369	0.411426246	-0.81096618	2.543495883
Aradu.DD097	1.269033146	2.09423607	-0.30400619	3.851998837	4.638653116
Aradu.71T87	NA	NA	-6.64385619	NA	-2.12029423
Aradu.H926S	3.279471296	3.798050515	3.407352751	3.818646083	4.160274831
Aradu.Q8N4A	0.189033824	-0.15200309	0.042644337	0.782408565	1.292781749
Aradu.YR8RM	-1.18442457	-0.83650127	-0.34007544	-2.47393119	-3.64385619
Aradu.1D7KB	-3.83650127	NA	-3.05889369	NA	NA
Aradu.W225Y	-2.32192809	-1.94341647	-2.39592868	1.22650853	0.669026766
Aradu.MBB01	1.416839742	1.941106311	2.025028794	2.192194165	2.313245852
Aradu.C2F9N	NA	NA	NA	NA	NA
Aradu.TWK59	-1.08926734	-1.73696559	-1.25153877	-3.83650127	-2.25153877
Aradu.1D83F	NA	NA	NA	NA	-4.64385619
Aradu.5D1Y3	3.480265122	3.63691458	3.673556424	3.008988783	3.935459748
Aradu.EH245	-2.47393119	-2.18442457	-2.83650127	-2.39592868	-3.47393119
Aradu.4D7HL	-0.4150375	-0.08926734	-0.30400619	0.23878686	0.773996325
Aradu.DQQ0U	0.22650853	0.344828497	0.056583528	1.077242999	2.17951105
Aradu.J0PRI	0.748461233	1.292781749	1.097610797	2.100977648	1.50589093

Aradu.V4203	-1.02914635	0.263034406	-0.34007544	1.713695815	1.941106311
Aradu.IZQ3Z	-3.47393119	-2.12029423	-2.83650127	3.09592442	2.207892852
Aradu.57C0B	-1.59946207	-1.35845397	-2.12029423	0.056583528	-0.08926734
Aradu.75AIB	-3.64385619	-1.25153877	-2.05889369	-3.32192809	-3.05889369
Aradu.7E2JG	-0.07400058	0.321928095	-0.66657627	1.207892852	1.989139007
Aradu.ZS0PF	0.389566812	0.333423734	0.516015147	-3.64385619	-3.64385619
Aradu.J06JT	3.010779839	3.119356177	2.974529312	3.552131108	3.772941338
Aradu.86C6D	3.099295204	3.489286023	3.234194723	5.702657543	5.454833907
Aradu.DAV01	8.211839636	8.801740688	9.081723091	9.392768195	9.510368584
Aradu.YJ46C	3.021479727	3.169925001	2.97819563	3.272023189	3.638073837
Aradu.4A33P	2.381283373	2.59454855	2.353323291	2.347665656	1.378511623
Aradu.44QR7	4.146492307	4.523561956	4.591559746	6.659211024	6.771753554
Aradu.0572C	-3.83650127	-4.05889369	-4.05889369	-1.51457317	0.014355293
Aradu.P9IKS	-5.05889369	-3.18442457	-4.64385619	0.948600847	0.713695815
Aradu.DE8ET	1.63691458	1.952333566	1.952333566	2.23572706	2.584962501
Aradu.Z0FA2	0.475084883	0.773996325	0.526068812	2.689299161	2.510961919
Aradu.3F84N	NA	-6.64385619	-6.64385619	NA	-2.18442457
Aradu.2MS2X	NA	NA	NA	NA	-4.05889369
Aradu.IAE5L	-1.28630419	-1.08926734	-2.12029423	0.545968369	1.014355293
Aradu.Q9F9Y	0.765534746	1.344828497	1.275007047	1.510961919	2.121015401
Aradu.H15D7	-0.83650127	-0.25153877	-0.39592868	2.229587923	2.427606173
Aradu.WJ1IU	-3.83650127	-2.83650127	-2.32192809	0.286881148	1.201633861

Aradu.GHE18	2.908812908	3.422233001	3.107687869	4.177917792	4.007195501
Aradu.ZBZ36	4.445594291	5.027684877	5.163498732	2.845991771	2.761285273
Aradu.MS406	0.970853654	0.956056652	1.163498732	2.169925001	1.765534746
Aradu.FK0J4	2	2.516015147	2.304511042	3.021479727	2.992768431
Aradu.L8GY0	3.250961574	3.125981654	3.021479727	1.744161096	4.600507645
Aradu.71JL3	-0.23446525	0.150559677	-0.26881676	-2.64385619	-1.08926734
Aradu.JB8ME	NA	NA	NA	NA	NA
Aradu.DRK7I	1.891419187	1.550900665	1.63691458	2.253989266	2.910732662
Aradu.X44N9	1.871843649	2.510961919	1.646162657	2.625270489	2.700439718
Aradu.8GE4T	-2.32192809	-2.47393119	-2.32192809	-3.32192809	-2.64385619
Aradu.9F1L9	4.09592442	3.386810946	3.565597176	0.963474124	1.137503524
Aradu.254QE	3.418189948	3.909773104	3.809414444	4.305970521	4.56193706
Aradu.SPS87	1.361768359	2.100977648	1.565597176	2.845991771	3.061776198
Aradu.64RBA	1.124328135	1.427606173	1.257010618	2.23572706	2.451540833
Aradu.RAX6G	0.443606651	1.584962501	0.176322773	2.347665656	2.440952198
Aradu.25DKA	0.411426246	5.591260525	4.802710219	4.615298579	3.621758857
Aradu.R3YL8	-2.73696559	-2.05889369	-2.83650127	-0.91593574	-0.12029423
Aradu.RQ9JK	1.731183242	2.411426246	1.622930351	2.538538164	2.970853654
Aradu.80U52	1.292781749	1.521050737	1.752748591	1.718087584	2.17951105
Aradu.FN1D7	1.411426246	1.304511042	1.056583528	-2.25153877	-1.39592868
Aradu.R9T1V	2.983677695	3.519793486	2.901108243	4.656496371	4.723012396
Aradu.02ZTY	4.088311236	3.967168608	4.060047384	3.988230223	3.833902077

Aradu.70YAF	1.695993813	2.021479727	2.028569152	2.053111336	1.98550043
Aradu.623GS	-5.64385619	NA	NA	-4.32192809	-4.64385619
Aradu.HG92V	-0.62148838	-0.20091269	-4.05889369	0.333423734	4.731726323
Aradu.6VN02	-2.18442457	-1.78587519	-1.88896869	-0.21759144	0.748461233
Aradu.VM6WK	1.077242999	-0.81096618	1.480265122	1.469885976	2.060047384
Aradu.H365I	NA	NA	NA	-4.05889369	-2.25153877
Aradu.G013M	0.815575429	1.580145484	0.925999419	1.948600847	2.049630768
Aradu.U3UVH	-2	-1.73696559	-2.18442457	-0.59946207	0.084064265
Aradu.TC0KD	-0.01449957	0.632268215	0.298658316	1.195347598	2.286881148
Aradu.C1UGC	-5.64385619	-5.05889369	-4.64385619	-3.47393119	-0.10469738
Aradu.SRY7A	1.974529312	1.906890596	1.835924074	1.704871964	1.97819563
Aradu.RG76L	0.286881148	1.117695043	0.321928095	1.929790998	2.220329955
Aradu.R3Z7V	-2.73696559	-0.76121314	-1.39592868	-3.32192809	-3.32192809
Aradu.ZV3G2	3.691534165	4.079804755	3.926948248	3.675815931	3.980939266
Aradu.NT54G	-2.47393119	-2.12029423	-2.83650127	NA	NA
Aradu.WPA2I	1.89917563	1.974529312	2.046141782	1.321928095	2.046141782
Aradu.HP9LA	2.443606651	3.078951341	2.931683057	3.244887059	3.537296067
Aradu.Z65EX	-2.32192809	-1.59946207	-2.39592868	-0.83650127	-0.55639335
Aradu.P56I7	2.653060017	3.132576843	2.799087306	4.143230135	4.295723025
Aradu.SDL7M	2.555816155	2.666756592	2.792855352	2.22342255	2.782408565
Aradu.GUH6T	-6.64385619	-4.64385619	-6.64385619	-5.64385619	-2.47393119
Aradu.9TU3	0	0.176322773	-0.02914635	2.14404637	2.341985747

R					
Aradu.BMJ8R	-5.64385619	-4.05889369	-5.05889369	-3.47393119	-3.83650127
Aradu.UM9US	3.688180359	3.850999395	3.327687364	2.707082992	2.722466024
Aradu.8II80	1.176322773	2.17951105	1.718087584	2.416839742	3.097610797
Aradu.1C7BQ	-5.64385619	NA	NA	-3.05889369	-3.18442457
Aradu.Q5PIR	NA	NA	NA	NA	NA
Aradu.NVB9V	-2	-2.05889369	-2.94341647	-2.18442457	2.10433666
Aradu.PU63R	-1.18442457	-0.32192809	-0.43440282	2.244887059	1.827819025
Aradu.2RS8H	3.244887059	3.424922088	3.017921908	2.698218478	3.025028794
Aradu.D2UEN	3.364572432	1.922197848	2.14404637	-0.4150375	1.565597176
Aradu.V638G	3.653060017	3.56193706	3.791814071	2.23572706	2.957914599
Aradu.03THN	1.66448284	2.121015401	1.981852653	1.86393845	2.432959407
Aradu.LCC63	0.941106311	1.794935663	1.232660757	3.789729251	3.925049965
Aradu.B6CZV	3.147306699	3.285402219	2.232660757	-0.49410907	-2.12029423
Aradu.XA7KS	-2.94341647	-5.64385619	-1.55639335	-4.05889369	-1.28630419
Aradu.LA1WX	-4.64385619	-2.83650127	-3.18442457	-2.64385619	-1.83650127
Aradu.NE0BE	-1.51457317	1.782408565	0.731183242	0.895302621	0.687060688
Aradu.DGK47	NA	NA	NA	NA	NA
Aradu.XA5F9	-2.83650127	-1.83650127	-2	-0.23446525	-0.25153877
Aradu.LBD98	-1.64385619	-1.55639335	-2.12029423	-3.83650127	-2.83650127
Aradu.HVG4L	1.790772038	2.124328135	1.618238656	1.765534746	2.3305584
Aradu.HPG6B	-3.47393119	-2.64385619	-2.39592868	-1.21759144	-0.76121314
Aradu.WRB0	1.570462931	1.459431619	1.700439718	1.056583528	0.659924558

S					
Aradu.ZDD46	1.350497247	1.744161096	1.416839742	1.726831217	2.277984747
Aradu.T5WNW	-5.05889369	-3.64385619	-3.83650127	-4.64385619	-4.64385619
Aradu.SUY1C	0.214124805	-0.05889369	0.189033824	-0.88896869	0.5360529
Aradu.08MEK	-2.32192809	-1.28630419	-2.73696559	-1.94341647	-0.23446525
Aradu.GHK20	3.973611276	4.01346226	3.942983598	2.889473543	3.826802684
Aradu.60S2U	0.790772038	2.488000771	2.742006211	2.994579724	3.066950244
Aradu.3GX6J	4.302319051	4.754887502	4.498250868	5.482848283	6.319401167
Aradu.P9DZZ	0.82374936	0.97819563	0.495695163	3.410069692	4.09000653
Aradu.93BUT	-0.23446525	0.097610797	-0.88896869	0.604071324	0.790772038
Aradu.Y3BW3	NA	-2.94341647	-3.32192809	NA	NA
Aradu.UG0RV	-2.73696559	-6.64385619	-6.64385619	-5.64385619	4.958842675
Aradu.NH34T	NA	NA	-5.64385619	NA	-4.32192809
Aradu.0BT33	NA	NA	NA	-1.68965988	-1.94341647
Aradu.ZYI4E	1.565597176	1.622930351	1.454175893	1.49057013	2.025028794
Aradu.0GZ0B	-0.04394335	0.23878686	-0.53533173	3.485426827	3.690417096
Aradu.G6CRU	NA	NA	NA	NA	NA
Aradu.XW8AZ	-0.81096618	2.260025656	3.388189537	0.298658316	1.063502942
Aradu.4C314	3.13422094	2.811471031	2.801158656	1.722466024	2.121015401
Aradu.M4U01	5.185470146	5.201241771	5.358607249	2.63691458	3.198494154
Aradu.Q0MV7	NA	NA	NA	-3.64385619	NA
Aradu.S8XF6	1.028569152	1.541019153	1.384049807	1.835924074	2.182692298
Aradu.BHV1	4.163498732	4.460742564	3.701549057	5.094658343	5.292781749

0

Aradu.E8471	3.161887682	3.486714373	3.12763328	2.22342255	2.807354922
Aradu.KGT1D	2.797012978	3.727920455	3.09085343	3.404630684	3.468583317
Aradu.Y4SSQ	2.253989266	2.09085343	2.063502942	1.176322773	0.485426827
Aradu.6W9YP	0.799087306	0.613531653	0.344828497	1.339137385	1.843983844
Aradu.PHE1E	5.440952198	5.594249948	6.001126665	2.257010618	2.17951105
Aradu.41DJI	3.770829046	3.609991295	4.06091205	2.543495883	2.861955364
Aradu.J4SMU	NA	NA	NA	NA	NA
Aradu.U3RTW	0.516015147	0.613531653	0.321928095	3.436961338	3.878725341
Aradu.9PD7U	NA	-6.64385619	NA	NA	-5.64385619
Aradu.SCF1F	-3.05889369	-3.64385619	-3.18442457	-0.34007544	-0.01449957
Aradu.I8NTZ	3.382667253	3.861955364	3.531069493	4.500802053	4.95419631
Aradu.88E60	3.514753498	3.560714954	3.444932049	3.472487771	3.62058641
Aradu.ZY2NN	NA	NA	NA	NA	NA
Aradu.RKH9D	4.454833907	4.116031993	4.46727948	1.948600847	1.056583528
Aradu.V3C05	1.531069493	1.111031312	1.124328135	1.761285273	2.283921772
Aradu.BM5FL	3.902073579	3.64731451	3.721372659	1.815575429	2.189033824
Aradu.346IW	1.063502942	1.333423734	1.469885976	1.389566812	2.09085343
Aradu.3D0ZZ	NA	NA	-5.64385619	-3.47393119	-2.64385619
Aradu.T8IMJ	-0.68965988	-0.43440282	-0.55639335	1.432959407	1.735522177
Aradu.CJT43	3.401903472	4.393690764	4.724650272	5.50589093	5.583158004
Aradu.SK1HP	-1.73696559	-2.47393119	-3.18442457	1.835924074	2.214124805
Aradu.PV4Q	0.956056652	1.269033146	1.028569152	1.773996325	2.339137385

E					
Aradu.RB3TY	NA	NA	NA	NA	NA
Aradu.FF8NM	-0.86249648	-0.68965988	-0.64385619	-3.83650127	-2.39592868
Aradu.X5630	0.956056652	1.718087584	1.275007047	2.472487771	2.831877241
Aradu.2QG6G	NA	NA	-5.64385619	NA	5.358607249
Aradu.JL0B3	2.03562391	2.283921772	1.97819563	2.321928095	2.372952098
Aradu.1F4NN	NA	-6.64385619	NA	NA	1.327687364
Aradu.YWN5L	2.056583528	2.641546029	1.835924074	6.320484678	6.448570626
Aradu.289R9	1.887525271	1.765534746	1.769771739	-0.26881676	0.839959587
Aradu.VV3RK	-0.83650127	0.799087306	0.263034406	0.565597176	1.819668183
Aradu.G8908	2.324810603	2.695993813	2.625270489	2.879705766	3.184280294
Aradu.4IC4Q	3.635754391	4.01346226	3.837943242	4.440287821	4.516645558
Aradu.H1X77	2.533563348	3.025028794	2.66448284	4.850999395	4.62058641
Aradu.8NK92	0.250961574	0.565597176	0.422233001	1.176322773	1.427606173
Aradu.FL7HA	3.392317423	3.760220946	3.389566812	3.817623258	4.070389328
Aradu.R64XC	-0.59946207	0.084064265	-0.20091269	1.333423734	1.748461233
Aradu.ZVP8C	0.704871964	1.176322773	0.992768431	1.827819025	2.289834465
Aradu.J6I78	1.918386234	2.310340121	2.14404637	2.976363636	3.023255352
Aradu.V7EH0	-3.83650127	-2.83650127	-3.18442457	0.22650853	-0.04394335
Aradu.73KGG	2.587364991	2.336283388	2.201633861	3.053111336	3.50589093
Aradu.FB522	1.914564523	2.752748591	2.689299161	3.475084883	3.608809243
Aradu.L933I	1.967168608	2.625270489	2.147306699	2.922197848	3.09085343
Aradu.DWF9	2.053111336	2.010779839	2.025028794	0.925999419	1.735522177

5					
Aradu.AF8HB	0.50589093	1.03562391	0.659924558	1.555816155	2.053111336
Aradu.N5B8E	0.526068812	1.443606651	0.50589093	3.032100843	3.257010618
Aradu.7H2PW	NA	NA	NA	2.397802962	1.967168608
Aradu.LXN93	6.528883827	5.859472667	6.185470146	3.267535798	3.189033824
Aradu.9AT7R	1.263034406	1.695993813	1.097610797	2.384049807	2.604071324
Aradu.HK079	-0.07400058	0.86393845	1.056583528	-0.83650127	-1.68965988
Aradu.85E8I	NA	NA	-5.64385619	-4.05889369	-6.64385619
Aradu.95964	1.819668183	2.014355293	1.709290636	3.078951341	2.961623328
Aradu.0V01P	5.730096466	5.877253454	5.928133408	-0.10469738	0.454175893
Aradu.4635I	-1.68965988	-1.18442457	-0.86249648	-1.94341647	-2.47393119
Aradu.RJ0UE	-2.94341647	-2.32192809	-2.94341647	-2.32192809	-1.88896869
Aradu.86BB6	-3.32192809	-1.64385619	-2.05889369	-0.83650127	-0.577767
Aradu.9XX4W	0.622930351	0.250961574	0.613531653	-5.64385619	-0.66657627
Aradu.LCM6E	NA	-6.64385619	NA	0.042644337	0.933572638
Aradu.ETS8A	NA	-5.05889369	-3.64385619	-1.12029423	4.727920455
Aradu.7WF2V	-0.68965988	-1.39592868	-1.78587519	-1.47393119	-1.32192809
Aradu.RJ6JS	-1.15200309	-1.12029423	-1.39592868	-1.12029423	-0.45403163
Aradu.EQY7L	0.137503524	0.422233001	0.731183242	1.070389328	1.718087584
Aradu.8X20T	0.847996907	1.292781749	1.021479727	1.718087584	1.815575429
Aradu.R2K02	3.781359714	4.3652726	5.081083929	4.051372102	4.155425432
Aradu.X22EB	-3.83650127	-1.78587519	-1.73696559	NA	NA
Aradu.T7BA	4.343407822	4.496973581	4.420212891	2.998195503	2.970853654

A					
Aradu.M4MQC	3.106013238	2.629939409	3.021479727	3.09423607	3.653060017
Aradu.2JF44	5.28503225	4.766595162	4.737686761	4.751142325	5.537606691
Aradu.QZ6DW	0.790772038	1.207892852	1.021479727	1.111031312	1.769771739
Aradu.2W1AJ	0.622930351	-0.18442457	-0.23446525	-3.64385619	-3.83650127
Aradu.ZL56D	7.612499946	6.577881959	7.12287975	2.347665656	3.365972428
Aradu.CKU4P	-1.83650127	-1.68965988	-0.88896869	2.618238656	2.807354922
Aradu.E79CC	-3.18442457	-2.47393119	-2.83650127	-0.91593574	1.448900951
Aradu.PP1IT	-6.64385619	NA	NA	-4.64385619	-3.83650127
Aradu.IYG99	-0.08926734	0.879705766	1.189033824	2.671293372	2.459431619
Aradu.ZMN8D	0.887525271	1.773996325	0.895302621	2.608809243	2.942983598
Aradu.6JP4A	1.269033146	1.531069493	1.475084883	1.748461233	2.201633861
Aradu.CP8NA	1.761285273	2.260025656	2.381283373	1.761285273	2.097610797
Aradu.T8J76	1.66448284	2.100977648	1.765534746	2.752748591	2.942983598
Aradu.X7RSP	-0.76121314	-0.91593574	-1.08926734	-0.68965988	0.111031312
Aradu.LQH8Y	-4.64385619	-6.64385619	-5.05889369	-4.05889369	-3.32192809
Aradu.V2YDB	-3.05889369	-1.25153877	-2.64385619	1.007195501	1.895302621
Aradu.UC6E5	2.680324357	3.249445341	3.09423607	3.377123749	3.663344619
Aradu.76V5M	-1.12029423	-2.47393119	-1.73696559	-3.18442457	-3.18442457
Aradu.ZTH93	1.867896464	3.730096466	3.828834649	3.019701914	3.807354922
Aradu.Y5WFV	0	1.292781749	0.056583528	2.163498732	1.959770155
Aradu.1AJ4F	NA	-5.64385619	-6.64385619	NA	-5.64385619
Aradu.3GT7	-5.05889369	-3.32192809	-4.05889369	-1.73696559	-1.55639335

T					
Aradu.8V7CS	1.757023247	1.111031312	0.189033824	2.560714954	2.724650272
Aradu.UD2Q8	-0.10469738	0.028569152	-0.62148838	1.117695043	0.956056652
Aradu.0VP5J	-0.12029423	0.028569152	-0.43440282	2.443606651	2.801158656
Aradu.GA7JT	-1.02914635	-0.73696559	-0.64385619	-1.51457317	-0.97143085
Aradu.VQA02	0.722466024	1.201633861	0.831877241	1.280956314	1.608809243
Aradu.YPX3B	-0.68965988	-0.47393119	-0.47393119	-0.71311885	-1.08926734
Aradu.9SY6W	0.124328135	0.565597176	0.286881148	0.40053793	0.773996325
Aradu.HP9IR	-3.64385619	-1.78587519	-1.78587519	-0.01449957	0.367371066
Aradu.B1ZZT	-2.73696559	-2.83650127	-3.32192809	0.214124805	1.480265122
Aradu.EQE9W	2.904965719	2.754887502	2.659924558	3.434294618	3.480265122
Aradu.74JTE	-0.49410907	3.576522138	2.885574364	0.516015147	-0.64385619
Aradu.8QL4Z	NA	-4.32192809	-5.05889369	-2.83650127	-1.59946207
Aradu.SL05Q	-0.94341647	-1	-1.12029423	0.739848103	0.5360529
Aradu.G1DD8	3.196921734	3.750606505	3.970853654	3.087462841	3.042644337
Aradu.M35ST	-0.23446525	0.495695163	0.042644337	1.333423734	1.280956314
Aradu.46U1T	NA	NA	NA	NA	-5.64385619
Aradu.YE325	-1.64385619	-1.15200309	-1.78587519	0.014355293	-0.39592868
Aradu.04RDY	4.556429415	4.64096791	5.130519083	4.732811872	4.348374075
Aradu.70JJH	2.14404637	2.384049807	1.996388746	2.014355293	2.266036894
Aradu.4XJ32	0.887525271	1.726831217	0.871843649	3.412781525	3.389566812
Aradu.8H45I	2.994579724	3.206330648	3.0721058	3.626439137	4.024142346
Aradu.1AM05	0.111031312	-0.13606155	-0.83650127	1.98550043	2.100977648

Aradu.JS1QQ	-6.64385619	-5.64385619	-5.64385619	-3.83650127	-4.05889369
Aradu.5LR1R	-1.18442457	-0.78587519	-1.18442457	0.056583528	-0.45403163
Aradu.GA8EY	NA	NA	NA	NA	-1.83650127
Aradu.YY5C4	-0.10469738	0.263034406	0.495695163	3.053111336	0.731183242
Aradu.54T4X	0.713695815	2.124328135	2.364572432	0.970853654	1.207892852
Aradu.DKE60	-0.34007544	1.384049807	0.815575429	-2.83650127	-1.21759144
Aradu.6M56A	2.718087584	2.927896454	2.59454855	4.225737653	3.825785627
Aradu.22C6J	1.077242999	1.843983844	0.695993813	3.56315813	3.392317423
Aradu.C7QG4	NA	-1.59946207	-2.94341647	-2.83650127	-1.78587519
Aradu.GQT7W	0.584962501	0.722466024	0.871843649	3.596935142	3.277984747
Aradu.GM5S2	-4.05889369	-4.64385619	-4.32192809	NA	-6.64385619
Aradu.FW65S	2.485426827	2.901108243	2.503348735	2.744161096	2.702657543
Arachis ipaënsis					
Araip.00FG1	NA	-6.64385619	NA	1.150559677	1.475084883
Araip.00G1J	-0.26881676	0.150559677	-0.20091269	1.049630768	1.298658316
Araip.00H39	4.117695043	4.321206567	4.032100843	5.367021533	5.557042415
Araip.00HCH	-3.47393119	-2.18442457	-3.83650127	-1.94341647	-2.12029423
Araip.00JXM	3.378511623	4.780835002	4.670160514	1.799087306	3.418189948
Araip.00K86	1.618238656	1.321928095	1.40599236	0.918386234	0.35614381
Araip.00P1B	1.82374936	1.989139007	1.709290636	0.887525271	2.021479727
Araip.00Q3S	-2.47393119	-1.64385619	-2.83650127	-0.25153877	-0.28630419
Araip.00QX5	NA	NA	NA	NA	NA
Araip.00WV	3.220329955	3.601696516	3.528571319	4.303050085	4.196134881

B

Araip.00X39	2.996388746	3.22342255	3.161887682	3.748461233	4.010779839
Araip.0178A	4.105175192	4.409390936	4.275752049	4.853995647	4.922673593
Araip.01AT6	4.625270489	4.537917249	4.310340121	3.477677328	4.966707314
Araip.01BXJ	NA	NA	NA	NA	-5.05889369
Araip.01CXT	1.967168608	2.232660757	2.087462841	2.707082992	2.715893371
Araip.01H2N	3.432959407	3.087462841	3.17951105	2.477677328	2.89917563
Araip.01T7B	1.713695815	1.803227036	1.454175893	1.575312331	1.480265122
Araip.01ZK6	NA	NA	NA	NA	-1.88896869
Araip.01ZYD	2.767654798	2.970853654	2.937344392	2.693765712	3.266036894
Araip.0214S	3.053111336	2.875780063	2.82984956	4.495695163	4.61117238
Araip.026EM	1.589763487	2.10433666	2.59454855	2.046141782	3.557042415
Araip.028PN	-1.78587519	-1.64385619	-3.83650127	-0.97143085	0.632268215
Araip.02EM5	2.269033146	1.691534165	2.837943242	-2.64385619	-0.86249648
Araip.02HSD	0.516015147	1.280956314	0.831877241	1.416839742	1.722466024
Araip.02IFK	2.584962501	2.367371066	2.553360503	2.895302621	2.9800253
Araip.02MA2	-2.94341647	-2.12029423	-1.83650127	-0.68965988	-1.35845397
Araip.02N4R	3.148934105	3.715893371	3.353323291	6.204962331	6.594996337
Araip.02P6R	2.821710215	2.790772038	2.942983598	2.482848283	2.742006211
Araip.02Z8U	-3.64385619	-5.05889369	-5.64385619	-1.73696559	1.731183242
Araip.0301B	-0.20091269	0.475084883	0.545968369	2.014355293	2.13093087
Araip.0311L	NA	NA	NA	NA	0.956056652
Araip.036V7	1.580145484	2.286881148	1.3950628	2.983677695	3.106013238

Araip.03C48	0.389566812	0.526068812	0.722466024	0.704871964	0.722466024
Araip.03GF9	1.82374936	1.735522177	1.89917563	1.959770155	2.039138394
Araip.03MNI	NA	NA	NA	NA	1.316145742
Araip.03MS6	1.367371066	1.669026766	1.389566812	1.97819563	2.244887059
Araip.03Q00	-0.73696559	0.815575429	0.713695815	2.63691458	2.841973119
Araip.03RBH	1.778208576	2.13422094	1.650764559	1.292781749	1.739848103
Araip.03SRG	3.107687869	3.634593268	3.498250868	3.831877241	4.058316496
Araip.03SU2	2.673556424	3.039138394	2.895302621	2.746312766	3.152183419
Araip.03W6H	-2.18442457	0.137503524	-1	-1.21759144	2.3950628
Araip.03WH7	0.650764559	1.007195501	0.454175893	2.560714954	2.853995647
Araip.03WUR	NA	NA	NA	NA	NA
Araip.041RZ	3.682573297	4.213347282	4.318316841	3.566815154	3.758089934
Araip.0431E	1.117695043	1.618238656	1.063502942	1.761285273	1.891419187
Araip.0448C	3.711494907	4.125155131	3.957914599	4.830356747	5.17512535
Araip.044E8	1.232660757	1.835924074	1.432959407	4.439623138	4.653633311
Araip.0475C	NA	NA	NA	NA	NA
Araip.049JK	4.498250868	4.800123353	4.706530553	5.432959407	5.564683017
Araip.04GN0	0.970853654	1.286881148	0.941106311	1.622930351	2.207892852
Araip.04K1E	1.286881148	1.599317794	1.207892852	2.17951105	2.66448284
Araip.04KBR	-6.64385619	NA	-6.64385619	-3.18442457	-0.83650127
Araip.04KZI	1.516015147	1.599317794	1.521050737	1.263034406	1.778208576
Araip.04SW	2.080657663	2.295723025	2.195347598	2.060047384	2.422233001

W					
Araip.04TGY	NA	-4.05889369	-6.64385619	-5.64385619	-3.18442457
Araip.04VV6	-5.64385619	-4.64385619	-6.64385619	-5.05889369	-5.05889369
Araip.04X9B	2.498250868	3.966245873	4.483493351	0.545968369	1.799087306
Araip.05E6K	0.941106311	0.90303827	0.687060688	1.992768431	2.669026766
Araip.05I70	NA	-6.64385619	-6.64385619	NA	-2.94341647
Araip.05VR1	1.948600847	2.253989266	2.114367025	2.769771739	2.957914599
Araip.05XM7	-2.39592868	-1.43440282	-1.88896869	1.63691458	1.310340121
Araip.0675S	-1.47393119	-0.91593574	-2.55639335	1.627606838	2.121015401
Araip.069K4	2.859969548	3.258518925	3.056583528	4.451540833	4.059182199
Araip.06FC6	4.56315813	4.296457407	4.596338864	2.776103988	2.713695815
Araip.06FXT	NA	NA	NA	NA	NA
Araip.06PW6	0.23878686	0.641546029	0.464668267	1.98550043	2.873813198
Araip.06TYZ	1.007195501	1.718087584	1.613531653	2.678071905	2.72900887
Araip.06WA5	2.063502942	2.451540833	2.042644337	3.63691458	4.018812186
Araip.06WGU	-1.21759144	-0.12029423	0.613531653	7.166112857	5.889230152
Araip.07BW1	NA	-6.64385619	-5.64385619	-1.78587519	-1.88896869
Araip.07W64	0.35614381	1.361768359	0.963474124	1.956056652	0.925999419
Araip.07XD6	-1.21759144	0.070389328	-0.577767	1.575312331	1.613531653
Araip.081EX	3.874796966	5.902556005	5.069100632	7.072427412	6.6441447
Araip.08H2V	-2.94341647	-1.18442457	-1.55639335	-0.23446525	0.422233001
Araip.08M0G	5.970393538	6.283921772	6.467768557	4.998195503	5.19298317
Araip.08VK1	2.582556003	2.166715445	2.260025656	0.565597176	0.948600847

Araip.08Z17	0.201633861	0.286881148	-0.49410907	2.996388746	3.09085343
Araip.091L0	0.879705766	1.378511623	1.117695043	2.72900887	2.761285273
Araip.09AXD	1.298658316	0.847996907	1.014355293	-3.83650127	2.269033146
Araip.09BNR	3.182692298	3.619413011	3.46727948	4.18030702	4.59275601
Araip.09CWU	5.054414388	4.958842675	4.684818738	1.510961919	3.184280294
Araip.09ESU	NA	NA	NA	NA	-6.64385619
Araip.09EYJ	2.720278465	3.247927513	3.263034406	2.107687869	3.016139703
Araip.09GVV	0.454175893	1.929790998	0	2.49057013	2.726831217
Araip.09N6L	2.475084883	3.13422094	2.558267634	3.74092756	3.780310099
Araip.09PBC	1.372952098	1.555816155	1.584962501	2.87774425	3.097610797
Araip.09VK0	0.275007047	0.622930351	0.475084883	0.933572638	1.794935663
Araip.09VK1	NA	NA	NA	NA	NA
Araip.09WK1	-0.25153877	0.622930351	0.575312331	-0.62148838	-1.15200309
Araip.09YU8	-4.64385619	-5.64385619	-3.47393119	3.974529312	2.277984747
Araip.0A4CX	NA	NA	NA	0.263034406	0.443606651
Araip.0A5UL	2.007195501	2.613531653	2.121015401	3.873813198	3.924099886
Araip.0AG3E	-0.78587519	0.163498732	-1.18442457	-4.32192809	-0.20091269
Araip.0AN9G	-3.05889369	-2.83650127	-2.83650127	0.632268215	0.90303827
Araip.0AR95	0.98550043	0.704871964	0.695993813	-0.62148838	-0.37706965
Araip.0B2S6	1.411426246	2.430285273	2.100977648	1.021479727	1.5360529
Araip.0B3H2	4.680324357	3.964398632	3.750606505	1.195347598	1.459431619
Araip.0B5Q5	4.636334602	4.346956889	4.280213996	1.50589093	1.895302621
Araip.0B6PB	1.464668267	2.582556003	3.337711092	2.375734539	2.063502942

Araip.0BB7B	2.956056652	2.9800253	2.957914599	3.379898164	3.731183242
Araip.0BH7D	4.707082992	5.163096138	4.363871925	6.323910438	6.306517445
Araip.0BL53	2.608809243	4.200849575	4.28169825	4.218781168	3.956056652
Araip.0BL8B	4.23572706	4.737686761	4.287620044	6.454998363	6.557808299
Araip.0BN4Y	3.280956314	3.070389328	3.070389328	2.650764559	2.761285273
Araip.0C096	-5.64385619	-3.83650127	-3.32192809	-4.32192809	-3.64385619
Araip.0C8GZ	5.259272487	5.45779126	5.283551423	5.458775699	6.374170076
Araip.0C951	-2.39592868	-1.55639335	-1.94341647	0.367371066	0.214124805
Araip.0CF6W	2.313245852	3.363171077	2.629939409	3.956056652	3.828834649
Araip.0CL7X	3.859969548	3.682573297	4.270528942	2.22342255	1.843983844
Araip.0D136	3.767654798	4.267535798	3.631104282	5.618825953	5.573495715
Araip.0D2JJ	1.827819025	2.217230716	2.140778656	1.438292852	1.66448284
Araip.0D7QS	NA	NA	NA	NA	NA
Araip.0DK96	NA	NA	-5.64385619	NA	-5.05889369
Araip.0DM6L	-4.05889369	-0.88896869	-3.83650127	3.628773595	4.04701482
Araip.0DS8L	-1.35845397	-1.83650127	-1.59946207	0.097610797	0.250961574
Araip.0E3QE	2.59454855	2.790772038	2.438292852	2.998195503	3.482848283
Araip.0E4ZE	8.500403728	8.441740749	8.289004456	5.766065051	6.47037417
Araip.0E8VY	3.824767853	3.904965719	3.754887502	4.080657663	4.279471296
Araip.0ER3Q	-2.64385619	-2.55639335	-2.39592868	0.40053793	0.422233001
Araip.0EV9Y	-3.64385619	-2.47393119	-2.25153877	-2.94341647	-1.39592868
Araip.0F3YM	1.485426827	2.046141782	1.735522177	3.142413438	3.347665656

Araip.0FB59	0.201633861	0.713695815	-0.01449957	1.521050737	1.40599236
Araip.0FU6X	-1.55639335	-0.94341647	-0.88896869	-1	0.150559677
Araip.0G24M	6.483009577	5.985955755	6.211012193	1.292781749	1.3950628
Araip.0G32G	2.565597176	2.733354341	2.684818738	1.182692298	1.941106311
Araip.0G5JH	-2.25153877	-1.88896869	-1.64385619	0.014355293	0.584962501
Araip.0H2X8	NA	NA	NA	NA	-4.32192809
Araip.0H44R	3.09423607	3.682573297	3.289834465	4.147306699	4.317593505
Araip.0HE2H	-2.55639335	-1.21759144	-1.94341647	-1.25153877	-0.78587519
Araip.0HI1A	-0.37706965	-0.4150375	-0.71311885	-0.26881676	-0.15200309
Araip.0HK7I	NA	NA	NA	NA	NA
Araip.0I39J	NA	NA	NA	NA	-6.64385619
Araip.0I565	1.575312331	2.14404637	1.981852653	2.304511042	2.150559677
Araip.0I7VH	3.454175893	3.967168608	4.014355293	3.750606505	3.272023189
Araip.0I82K	-2.94341647	-4.32192809	-1.59946207	-5.05889369	-2.32192809
Araip.0IL8D	0.90303827	1.028569152	1.232660757	0.584962501	0.713695815
Araip.0IQ14	2.163498732	2.333423734	1.937344392	1.887525271	1.831877241
Araip.0J2TC	0.545968369	0.367371066	0.344828497	1.580145484	1.40599236
Araip.0J4UH	1.495695163	1.704871964	0.659924558	2.014355293	3.032100843
Araip.0J6W3	-3.05889369	-2.73696559	-4.64385619	-1.55639335	-1.35845397
Araip.0J7GI	0.124328135	0.879705766	0.918386234	1.521050737	1.555816155
Araip.0JU1Y	NA	NA	NA	NA	-5.05889369
Araip.0JY6V	6.383704292	6.498729559	6.283180979	6.819540461	6.744833837

Araip.0K3NF	1.98550043	2.107687869	2.22650853	2.3950628	2.493134922
Araip.0K6MU	3.030336078	3.390942773	3.250961574	2.375734539	2.553360503
Araip.0K8ZX	4.618825953	4.767125077	4.498889087	4.94016675	5.678353654
Araip.0L3VD	2.865918815	3.725741157	3.426264755	2.459431619	3.0721058
Araip.0L5SE	6.463524373	6.585413272	6.788816194	6.604664415	6.545350645
Araip.0L8ZM	NA	-2.64385619	-2.64385619	-3.05889369	-2.83650127
Araip.0LC57	4.270528942	4.949534933	4.87774425	4.765534746	4.780310099
Araip.0MB9K	3.340562269	3.051372102	3.019701914	2.347665656	2.59454855
Araip.0MK8M	3.367371066	3.434294618	3.337711092	1.09085343	1.411426246
Araip.0MR1X	NA	NA	NA	NA	NA
Araip.0MR58	NA	-4.64385619	-3.83650127	-1.51457317	-0.88896869
Araip.0N0ZT	-0.15200309	0.782408565	-0.05889369	1.367371066	1.275007047
Araip.0N6RB	-4.32192809	0.214124805	-0.94341647	3.005399988	2.163498732
Araip.0NG6C	4.138323004	4.462052319	4.315421316	5.310703657	5.668175869
Araip.0NL51	4.796493929	4.69543711	4.810957159	4.125155131	3.814550423
Araip.0NQ3E	2.03562391	2.510961919	2.107687869	2.86393845	2.95419631
Araip.0P2W0	2.275007047	2.601696516	2.419538892	2.430285273	2.845991771
Araip.0P39S	2.214124805	1.875780063	1.859969548	1.063502942	1.084064265
Araip.0P3QP	NA	-6.64385619	NA	-2	-1.55639335
Araip.0P3RJ	NA	-3.64385619	-6.64385619	0.59454855	2.893362211
Araip.0PV7W	1.632268215	2.403267722	1.843983844	4.884597921	4.713695815
Araip.0PX5T	NA	-4.64385619	NA	1.678071905	3.713695815

Araip.0Q5CU	NA	NA	NA	NA	2.147306699
Araip.0QG56	NA	NA	NA	NA	NA
Araip.0QI44	1.495695163	2.201633861	1.704871964	1.659924558	1.761285273
Araip.0QI46	0.263034406	0.545968369	0.124328135	1.632268215	1.748461233
Araip.0R69M	1.14404637	1.280956314	1.286881148	0.042644337	0.713695815
Araip.0RS02	3.163498732	3.270528942	2.528571319	4.329123596	4.832890014
Araip.0RS31	5.504938132	5.278728213	5.343052435	2.648465443	2.488000771
Araip.0S41I	NA	NA	NA	NA	NA
Araip.0S5VX	5.966937979	5.000450772	4.078951341	-0.02914635	0.321928095
Araip.0S8MN	2.9800253	2.757023247	2.869871406	3.339137385	3.592158002
Araip.0ST2B	1.063502942	1.790772038	2.073820233	4.968090752	4.030336078
Araip.0T13B	2.711494907	2.702657543	2.516015147	2.114367025	2.039138394
Araip.0T4FD	1.933572638	3.09592442	2.981852653	3.534808661	3.675815931
Araip.0TB3B	-1.15200309	-0.78587519	-0.86249648	0.263034406	0.50589093
Araip.0TX1W	3.116031993	3.645009884	3.982765463	4.245647774	3.484138131
Araip.0TX9X	-0.34007544	-0.21759144	-0.53533173	-1.94341647	0.097610797
Araip.0U07W	-1.25153877	-0.81096618	-1.08926734	-0.15200309	1.541019153
Araip.0U2QD	4.600507645	4.716990894	4.066950244	5.670727054	5.715069679
Araip.0U4IT	5.351204277	5.301953395	5.659924558	2.849999259	3.292781749
Araip.0U7A4	4.489928219	4.831370588	4.484782623	5.095080492	5.338424415
Araip.0V0EF	2.247927513	2.283921772	2.289834465	2.14404637	2.503348735
Araip.0V3N6	-5.64385619	-5.05889369	-5.64385619	-3.64385619	-4.32192809
Araip.0V7N2	9.578580258	9.187945856	9.36756764	6.335569006	6.307974897

Araip.0W082	3.435628594	3.759155834	3.590961241	3.622930351	3.888499736
Araip.0W0BL	1.627606838	2.350497247	2.056583528	2.937344392	3.307428525
Araip.0W11Z	-0.51457317	-0.45403163	-1	1.014355293	1.495695163
Araip.0W3FE	-1.08926734	-0.71311885	-2	1.344828497	2.207892852
Araip.0W448	1.565597176	1.510961919	2.613531653	1.448900951	2.003602237
Araip.0W7EI	3.521050737	3.648465443	3.346247774	4.085764554	4.49057013
Araip.0WD88	2.682573297	3.185866545	2.891419187	3.568032105	3.798050515
Araip.0WL30	0.831877241	0.790772038	0.748461233	0.97819563	1.811471031
Araip.0WM4W	2.678071905	3.709290636	3.473786912	4.522934561	4.474436043
Araip.0WR1B	NA	NA	NA	NA	NA
Araip.0X2PV	-4.05889369	-5.64385619	-6.64385619	-5.64385619	-3.05889369
Araip.0XQ95	2.124328135	2.680324357	2.341985747	3.316145742	3.742006211
Araip.0XT2W	1.687060688	2.080657663	1.63691458	3.003602237	3.058316496
Araip.0Y08C	2.582556003	3.137503524	2.784503983	3.651912745	3.710393192
Araip.0Y2KA	-1.64385619	-1.15200309	-2	2.217230716	1.510961919
Araip.0Y4CE	0.847996907	1.560714954	1.137503524	2.077242999	2.137503524
Araip.0Y7IG	2.014355293	2.204766751	2.124328135	2.40053793	2.503348735
Araip.0Y9HM	-3.83650127	-3.05889369	-4.64385619	-1.68965988	-2.64385619
Araip.0YN4A	-1.55639335	-1.73696559	-2.05889369	1.35614381	1.111031312
Araip.0YS5Y	3.35473424	3.612352499	3.736604875	2.367371066	2.475084883
Araip.0Z0CW	1.485426827	1.722466024	2.313245852	1.910732662	2.272023189
Araip.0Z5TL	2.912649865	3.132576843	3.073820233	3.392317423	3.956985925

Araip.0Z6G1	0.50589093	0.97819563	0.704871964	1.275007047	1.316145742
Araip.0ZP55	1.49057013	1.726831217	1.669026766	2.582556003	2.622930351
Araip.0ZR5Y	-1.51457317	-1.18442457	-1.64385619	2.904965719	2.166715445
Araip.0ZS3I	0.070389328	0.344828497	0.150559677	-1.88896869	-0.20091269
Araip.101RY	1.163498732	1.910732662	1.618238656	2.914564523	2.807354922
Araip.10A0F	3.353323291	3.589763487	3.364572432	3.787641414	3.565597176
Araip.10CZT	-5.05889369	-6.64385619	NA	NA	-5.05889369
Araip.10EBD	1.361768359	2.137503524	2.021479727	0.855989697	2.62058641
Araip.10KHS	NA	NA	NA	NA	NA
Araip.10KZ6	-0.49410907	0.485426827	0.575312331	-0.88896869	0.189033824
Araip.10PYM	-1.68965988	-2.25153877	-1.88896869	-5.64385619	-2.83650127
Araip.10QHS	-0.83650127	0.150559677	-0.10469738	-2.18442457	-2.55639335
Araip.10YD3	-3.47393119	-3.47393119	-4.05889369	-2.55639335	-2.12029423
Araip.1117E	9.025637912	8.776498832	9.295654157	2.560714954	3.17951105
Araip.111QN	-1.05889369	-0.32192809	-2.73696559	2.49057013	2.910732662
Araip.11643	4.891905188	5.388878339	5.08491466	5.737686761	5.437294331
Araip.116UB	3.795974694	3.865918815	3.82374936	3.653060017	4.086613947
Araip.118R7	-5.64385619	-5.05889369	-4.64385619	1.782408565	3.253989266
Araip.1196H	2.176322773	2.908812908	2.192194165	4.61999983	4.363871925
Araip.11AMG	4.414812061	3.795974694	3.885574364	4.007195501	5.100557221
Araip.11BKV	3.930737338	4.478324711	4.331275267	3.276496666	3.403267722
Araip.11CVG	-5.05889369	-2.83650127	-5.64385619	-2.47393119	-1.83650127

Araip.11KLU	3.218781168	3.073820233	2.881664619	2.117695043	2.847996907
Araip.11N45	-1.08926734	-0.04394335	-0.55639335	1.485426827	1.608809243
Araip.11R1Y	4.450221496	4.34269696	4.165911939	4.263785614	4.46139759
Araip.11SIA	2.606442228	3.152183419	2.857980995	3.968090752	4.19298317
Araip.11VE0	0.963474124	1.735522177	-0.07400058	6.388533979	6.683275373
Araip.11WF7	NA	-6.64385619	-5.64385619	-5.05889369	-4.64385619
Araip.11Y3M	-2.64385619	-2.64385619	-2.94341647	-0.94341647	-0.39592868
Araip.11YG0	3.419538892	3.66106548	3.319039816	4.941106311	4.991861931
Araip.122Z7	2	2.584962501	2.201633861	2.827819025	2.989139007
Araip.123NR	1.432959407	1.933572638	1.550900665	2.414135533	2.767654798
Araip.125MX	3.22342255	3.260025656	3.27351589	3.882643049	4.473137488
Araip.12A3Y	1.66448284	2.03562391	1.718087584	2.372952098	2.469885976
Araip.12IR2	1.803227036	2.292781749	2.03562391	2.99095486	2.746312766
Araip.12MTY	-1.83650127	-1.73696559	-1.55639335	0.713695815	0.250961574
Araip.12PK8	1.757023247	2.298658316	1.803227036	2.275007047	2.739848103
Araip.12RXW	3.410069692	3.861955364	3.539779192	4.517275693	4.786596362
Araip.12TI6	1.86393845	2.140778656	1.077242999	-1.39592868	-0.16812276
Araip.12XQ6	-2.39592868	-2.12029423	-2.64385619	-2.47393119	-2.12029423
Araip.12ZFL	NA	NA	NA	NA	NA
Araip.1309E	4.29130886	4.234961095	4.040015679	3.09592442	3.372952098
Araip.135BH	-3.18442457	-0.28630419	0.748461233	-0.64385619	0.344828497
Araip.136S0	-1.39592868	-1.73696559	-2.47393119	-1.64385619	-0.10469738

Araip.137YQ	-1.59946207	0.310340121	0.35614381	1.344828497	1.361768359
Araip.13D8C	-4.32192809	-1.78587519	-1.32192809	0.310340121	0.454175893
Araip.13H0V	3.15704371	3.016139703	3.435628594	1.895302621	3.331991778
Araip.13HZD	4.087462841	3.854993017	3.969012308	3.885574364	4.344828497
Araip.13I5K	4.129283017	4.577126661	4.272023189	4.320484678	4.751142325
Araip.13N2J	0.613531653	1.859969548	0.687060688	1.782408565	1.867896464
Araip.13VTA	3.203201156	3.517275693	3.100977648	3.588564737	4.089159132
Araip.13WJ1	NA	NA	NA	NA	-3.05889369
Araip.13WQP	NA	-5.05889369	-5.64385619	-3.83650127	3.181102551
Araip.13XDD	2.22342255	2.678071905	2.211012193	2.084064265	3.699329526
Araip.14067	2.713695815	3.785550552	3.663344619	2.201633861	2.435628594
Araip.14380	4.327687364	4.239550797	4.186659017	2.124328135	1.86393845
Araip.149YW	-2.39592868	-3.64385619	-3.47393119	-3.83650127	-2.18442457
Araip.14A0C	-1.12029423	-0.21759144	-1.08926734	3.056583528	3.155425432
Araip.14BL9	2.163498732	3.82374936	2.50589093	6.630230716	6.65692534
Araip.14ENN	-1.47393119	-0.53533173	-0.86249648	-0.64385619	-0.20091269
Araip.14ESI	2.906890596	3.087462841	2.994579724	2.275007047	2.89917563
Araip.14FTC	4.445594291	5.740657772	5.815063017	2.819668183	2.754887502
Araip.14HQ9	2.944858446	3.119356177	3.003602237	2.321928095	2.482848283
Araip.14NBL	2.419538892	2.386810946	2.09423607	3.721372659	3.264536431
Araip.14RNX	1.967168608	2.916476644	1.815575429	3.957914599	3.880685525
Araip.14VDU	2.375734539	2.752748591	2.160274831	3.40053793	3.460742564

Araip.14YQZ	2.15704371	2.339137385	2.201633861	2.301587647	2.601696516
Araip.154H4	2.114367025	2.319039816	1.827819025	0.992768431	1.007195501
Araip.15B33	0.887525271	1	0.485426827	0.464668267	0.790772038
Araip.15C6I	3.021479727	3.47118746	3.005399988	4.44625623	4.568640195
Araip.15EJY	-1.51457317	1.014355293	1.042644337	-3.32192809	-1.47393119
Araip.15F3V	5.668459557	5.630813152	4.98458935	7.309794644	7.636407112
Araip.15H8S	-2.55639335	-2.47393119	-4.05889369	-1.78587519	2.039138394
Araip.15K52	1.049630768	1.831877241	1.618238656	2.797012978	2.648465443
Araip.15KTJ	-0.88896869	-0.10469738	-0.73696559	1.778208576	2.358958826
Araip.15N6Y	-5.05889369	-4.05889369	-6.64385619	-4.64385619	-3.83650127
Araip.15RTX	0.632268215	0.613531653	0.59454855	0.970853654	1.422233001
Araip.15UQN	3.217230716	3.269033146	3.152183419	4.390254956	4.372255648
Araip.160V1	1.952333566	2.746312766	2.275007047	2.275007047	2.166715445
Araip.162FJ	NA	-2.94341647	-3.83650127	-3.47393119	-2.47393119
Araip.164TB	3.037382222	3.40053793	3.030336078	2.553360503	3.010779839
Araip.166TL	-2.83650127	-3.18442457	-1.88896869	-2.94341647	1.735522177
Araip.167J0	0.263034406	1.028569152	0.432959407	1.163498732	1.389566812
Araip.167MA	-0.73696559	-2.64385619	-0.73696559	NA	NA
Araip.16EI2	2.769771739	2.792855352	2.748461233	2.744161096	3.341985747
Araip.16F3N	2.498250868	2.871843649	2.23878686	3.604071324	3.590961241
Araip.16L7J	1.589763487	2.053111336	1.718087584	1.98550043	2.344828497
Araip.171V2	-2.47393119	-0.68965988	-2.94341647	-0.16812276	-0.49410907

Araip.172DZ	-5.64385619	-6.64385619	-6.64385619	-6.64385619	NA
Araip.175EE	-1.83650127	-0.78587519	-0.97143085	1.516015147	1.443606651
Araip.175SR	2.456806149	2.678071905	2.367371066	4.114367025	4.032982417
Araip.176AR	3.529820947	3.324810603	3.365972428	2.746312766	2.704871964
Araip.177Y0	1.316145742	0.613531653	0.86393845	2.073820233	2.250961574
Araip.17941	-0.49410907	0.344828497	0.40053793	2.073820233	1.22650853
Araip.17GEB	3.462052319	3.762348816	3.972692654	2.722466024	2.324810603
Araip.17Z7K	-3.83650127	-2.47393119	-3.47393119	-3.47393119	-2.05889369
Araip.17ZYU	0.432959407	1	0.815575429	0.432959407	0.650764559
Araip.1842Y	2.100977648	2.13093087	1.843983844	3.177917792	3.410069692
Araip.18474	4.287620044	4.704318678	4.82984956	4.121844298	4.268284667
Araip.186SX	-3.47393119	-2.18442457	-2.64385619	0.443606651	0.298658316
Araip.1897A	0.250961574	1.13093087	1.007195501	2.584962501	2.599317794
Araip.18BT4	NA	-5.05889369	-4.05889369	-4.32192809	-3.47393119
Araip.18CW0	-2.55639335	-0.43440282	-1.73696559	2.570462931	2.277984747
Araip.18J4Y	0.422233001	0.59454855	0.495695163	0.678071905	0.432959407
Araip.18K24	NA	NA	NA	NA	NA
Araip.18LXX	2.659924558	3.073820233	2.891419187	3.776103988	3.650764559
Araip.18N2T	4.308885057	4.987775616	4.152183419	6.151168794	6.043300754
Araip.18PWY	2.659924558	2.994579724	2.526068812	3.833902077	3.988230223
Araip.18QQE	2.416839742	2.114367025	1.632268215	-0.62148838	1.669026766
Araip.18S7T	-2.55639335	-1.55639335	-3.32192809	0.790772038	0.739848103

Araip.18UE9	2.582556003	2.289834465	2.324810603	3.544732656	3.495695163
Araip.19369	2.327687364	2.247927513	2.182692298	2.046141782	2.604071324
Araip.1942F	8.149543909	7.823621998	8.083851588	3.894332742	3.890446693
Araip.19472	2.44625623	2.613531653	2.370164281	2.799087306	3.13422094
Araip.194YI	-2.73696559	-3.18442457	-2.64385619	-4.64385619	3.468583317
Araip.19CBQ	NA	NA	-6.64385619	NA	-2.55639335
Araip.19H18	-3.05889369	-2.25153877	-2.83650127	-0.28630419	1.182692298
Araip.19HBE	-0.28630419	0.378511623	-0.10469738	0	0.344828497
Araip.19N4V	0.475084883	0.695993813	0.443606651	1.86393845	2.358958826
Araip.19T2H	-5.64385619	NA	NA	NA	NA
Araip.19TXN	2.565597176	2.718087584	2.558267634	3.106013238	3.109360559
Araip.19YRX	1.59454855	3.945794957	3.508428653	0.584962501	1.510961919
Araip.1A0QT	4.04701482	3.973611276	3.929790998	3.488000771	3.370164281
Araip.1A4HT	-0.43440282	1.201633861	-0.97143085	2.845991771	2.944858446
Araip.1A9I8	-5.05889369	-4.64385619	-5.64385619	NA	NA
Araip.1BL7S	1.627606838	1.361768359	-0.01449957	1.207892852	2.659924558
Araip.1BW4F	NA	-3.05889369	-3.47393119	0.333423734	0.464668267
Araip.1C050	0.124328135	0.855989697	0.422233001	2.283921772	2.495695163
Araip.1C20S	-0.76121314	-0.97143085	-1.43440282	1.263034406	1.250961574
Araip.1C4CW	NA	-5.64385619	-5.64385619	NA	NA
Araip.1C776	0.422233001	-0.08926734	0.475084883	-2.47393119	-2.05889369
Araip.1C7B4	NA	-4.64385619	-4.05889369	NA	-4.05889369

Araip.1CJ82	6.09212285	6.629648044	6.217812331	6.979796718	7.205548911
Araip.1CV02	2.881664619	2.859969548	2.761285273	1.367371066	2.648465443
Araip.1D0FW	NA	-2.25153877	-2.47393119	-0.05889369	-1.21759144
Araip.1D5WV	4.007195501	4.275007047	4.244887059	3.493134922	3.907852072
Araip.1E0RS	1.871843649	2.560714954	2.173127433	3.418189948	3.658782734
Araip.1E1WQ	4.412104045	6.844234988	6.272209861	1.150559677	3.575312331
Araip.1E43N	NA	NA	NA	NA	-0.62148838
Araip.1E680	-1.32192809	-1.51457317	-0.81096618	-1.25153877	-0.20091269
Araip.1E7ZD	3.84899843	4.700994494	4.129283017	3.693765712	3.841973119
Araip.1EQ95	1.480265122	2.792855352	2.424922088	3.139142019	2.970853654
Araip.1F0E9	-1.08926734	-1.21759144	-0.66657627	-2.55639335	-2.94341647
Araip.1F19V	-3.18442457	-2.94341647	-3.18442457	-6.64385619	-3.18442457
Araip.1F56R	NA	-4.32192809	NA	-1.18442457	-4.64385619
Araip.1F7P8	1.378511623	1.427606173	1.15704371	0.378511623	0.831877241
Araip.1FA5F	-5.05889369	-3.32192809	-3.83650127	-4.32192809	-2.05889369
Araip.1FV4W	1.584962501	2.307428525	1.891419187	2.809414444	2.782408565
Araip.1G19U	1.659924558	1.678071905	2.117695043	-3.18442457	-4.64385619
Araip.1G1KY	-4.05889369	-4.32192809	-4.32192809	-2.83650127	-0.88896869
Araip.1G1M0	6.783456654	5.720278465	6.16711703	-1.83650127	-0.45403163
Araip.1G2UE	0.23878686	0.176322773	-0.02914635	1.117695043	1.608809243
Araip.1G7Y9	1.10433666	1.580145484	1.13093087	1.641546029	1.952333566
Araip.1GQ6A	2.61117238	3.231125158	3.114367025	1.713695815	1.49057013

Araip.1H048	1.589763487	1.851998837	1.632268215	2.570462931	2.454175893
Araip.1H1H0	2.495695163	2.107687869	2.140778656	1.432959407	3.12763328
Araip.1H31M	2.163498732	2.531069493	2.341985747	2.959770155	3.106013238
Araip.1H3PC	NA	-5.05889369	NA	NA	NA
Araip.1H6L7	NA	NA	NA	NA	-4.05889369
Araip.1HR72	2.266036894	2.815575429	2.301587647	3.460742564	3.03562391
Araip.1I1JH	2.599317794	3.314696526	2.541019153	4.548436625	4.414135533
Araip.1I30Q	1.765534746	2.032100843	1.811471031	1.14404637	1.918386234
Araip.1I49F	NA	NA	NA	NA	-6.64385619
Araip.1IV9L	NA	NA	-4.32192809	NA	-3.32192809
Araip.1J28K	1.970853654	2.596935142	2.176322773	3.578938713	3.90014226
Araip.1J2S6	2.173127433	2.604071324	2.250961574	2.641546029	2.918386234
Araip.1J91U	2.735522177	2.575312331	2.397802962	1.280956314	1.673556424
Araip.1JG8Y	-2.47393119	-4.05889369	-4.32192809	-5.64385619	-3.47393119
Araip.1JL4U	0	0.526068812	0.163498732	1.835924074	2.13422094
Araip.1JL7K	9.596525228	9.340384235	9.853059987	4.47118746	4.336997417
Araip.1JP91	NA	NA	NA	NA	-3.47393119
Araip.1JR2Q	-1.88896869	-2.64385619	-2.25153877	0.378511623	1.22650853
Araip.1K0LY	3.707082992	2.989139007	3.22650853	3.336283388	3.319039816
Araip.1K5KM	-1.12029423	-1.35845397	-0.94341647	-1.73696559	1.695993813
Araip.1KE3S	-0.94341647	-1.08926734	-2.18442457	0.757023247	0.150559677
Araip.1KG8P	2.889473543	0.933572638	-0.35845397	-4.64385619	-3.05889369

Araip.1KL0I	1.646162657	1.709290636	1.269033146	3.894332742	3.371558863
Araip.1KN42	NA	-6.64385619	-6.64385619	-1.83650127	-2.73696559
Araip.1L1V5	5.551208374	5.865176497	4.964860664	6.943452539	6.746984506
Araip.1L67X	-0.51457317	0.855989697	-0.88896869	2.735522177	2.63691458
Araip.1L9GW	-0.45403163	-0.13606155	-1.08926734	1.516015147	0.565597176
Araip.1LA66	1.541019153	2.014355293	1.914564523	2.897240426	2.992768431
Araip.1LL57	-2.47393119	-3.18442457	-2.94341647	-2.18442457	3.742006211
Araip.1M393	5.113950482	4.880195729	5.393690764	1.097610797	0.90303827
Araip.1M3VN	2.713695815	2.173127433	1.914564523	2.754887502	3.343407822
Araip.1M7FH	1.748461233	2.137503524	1.891419187	2.548436625	2.897240426
Araip.1MI2Q	NA	NA	NA	NA	NA
Araip.1ML5Q	5.943452539	5.850749426	6.150965784	3.40599236	4.430285273
Araip.1ML5W	3.701549057	5.094658343	5.304145941	1.778208576	0.604071324
Araip.1MM96	3.558267634	3.378511623	3.722466024	-0.51457317	-0.39592868
Araip.1MS7W	2.582556003	2.957914599	2.843983844	2.689299161	3.496973581
Araip.1MT39	3.430285273	3.119356177	3.408711861	3.682573297	3.423578171
Araip.1N36Z	-4.32192809	-4.32192809	-5.05889369	-2	-2.73696559
Araip.1N4XS	2.275007047	2.40599236	2.475084883	3.804260116	4.157852169
Araip.1N6HI	-2.47393119	-1.73696559	-2.73696559	-0.64385619	-0.577767
Araip.1N7IP	-1.32192809	-2	-1.78587519	-3.18442457	-1.73696559
Araip.1NJ8N	-2.94341647	-2.32192809	-2.73696559	-0.28630419	0.250961574
Araip.1P0XB	-0.01449957	-0.49410907	-0.53533173	-0.64385619	0.124328135

Araip.1P1MT	-4.64385619	-3.64385619	-5.64385619	-4.05889369	-3.47393119
Araip.1P50V	-0.73696559	-1.73696559	-1.18442457	-0.73696559	-2.25153877
Araip.1P6L4	NA	NA	NA	NA	NA
Araip.1PU5F	2.384049807	2.553360503	2.427606173	2.914564523	2.959770155
Araip.1Q1XN	-2.83650127	-2.18442457	-2.64385619	-0.91593574	-0.23446525
Araip.1Q3SQ	NA	NA	NA	NA	-4.64385619
Araip.1Q8PX	2.182692298	2.350497247	2.247927513	1.584962501	1.835924074
Araip.1R84J	-0.55639335	0.378511623	-0.30400619	1.056583528	0.855989697
Araip.1R8Y6	2.214124805	2.601696516	2.298658316	1.769771739	1.883620816
Araip.1RB5V	3.528571319	3.666756592	3.771885579	2.976363636	3.277984747
Araip.1S1ZZ	0.722466024	0.86393845	0.807354922	0.40053793	-0.10469738
Araip.1S5XZ	2.333423734	2.40599236	1.89917563	1.182692298	2.384049807
Araip.1S76R	-1.55639335	0.056583528	-1.12029423	1.35614381	1.176322773
Araip.1S7CN	-3.18442457	-3.32192809	-2.55639335	-0.01449957	0.084064265
Araip.1SH3C	1.575312331	2.003602237	1.887525271	3.089159132	3.307428525
Araip.1SI6Z	3.244887059	3.602884409	3.246408087	3.634593268	3.94016675
Araip.1SL1G	7.005624549	6.805679393	7.321657564	0.650764559	0.310340121
Araip.1T581	1.981852653	2.553360503	2.182692298	3.944858446	3.971773447
Araip.1T874	NA	NA	NA	NA	NA
Araip.1T9DH	4.801676029	4.872336288	4.64038956	5.305970521	5.550285049
Araip.1T9WY	-3.47393119	-3.05889369	-2.64385619	-1.51457317	-1.78587519
Araip.1U1PJ	-0.20091269	0.575312331	0.056583528	1.475084883	1.608809243

Araip.1U5BB	1.82374936	2.137503524	2.042644337	2.924099886	3.077242999
Araip.1U5CY	7.670372993	7.525833979	7.655709596	3.23878686	3.557042415
Araip.1U8VF	-0.51457317	-0.76121314	-0.73696559	-0.64385619	1.378511623
Araip.1UK4V	2.803227036	3.19061486	3.192194165	4.221103725	4.402585758
Araip.1UW0G	9.60912332	10.74406857	10.74690895	9.751677946	10.30101598
Araip.1UW8I	2.776103988	2.316145742	2.014355293	3.212569339	3.295723025
Araip.1V5ZI	-3.18442457	-2.83650127	-3.47393119	0.286881148	1.361768359
Araip.1V67D	NA	NA	NA	NA	-4.64385619
Araip.1V6N7	1.304511042	1.327687364	0.98550043	2.361768359	2.950468414
Araip.1V89E	3.329123596	3.629939409	3.313245852	3.624100895	4.096767855
Araip.1VK73	-0.32192809	0.137503524	-0.45403163	1.432959407	1.416839742
Araip.1VN1Z	-5.64385619	-4.32192809	-5.05889369	-0.43440282	0.799087306
Araip.1W5SS	1.510961919	2.169925001	1.922197848	1.807354922	2.498250868
Araip.1WE6F	-0.76121314	-1.15200309	-0.83650127	-0.81096618	-0.01449957
Araip.1X10D	-0.88896869	0.124328135	-0.71311885	1.070389328	1.097610797
Araip.1X2V2	1.111031312	0.604071324	1.220329955	-0.83650127	2.22342255
Araip.1Y0FU	0.659924558	1.731183242	1.077242999	2.289834465	2.301587647
Araip.1Y2CP	0.650764559	0.565597176	0.250961574	-3.83650127	-4.05889369
Araip.1Y3MU	-0.35845397	-0.35845397	-0.71311885	0.839959587	0.422233001
Araip.1Y485	-2.83650127	-2.32192809	-2.73696559	-2.64385619	-1.59946207
Araip.1Y87C	3.917431755	4.051372102	4.643278996	3.731183242	3.838951767
Araip.1YM3E	1.495695163	1.799087306	1.459431619	2.722466024	2.737686761
Araip.1Z0SD	-0.73696559	-0.81096618	-1.21759144	-0.43440282	0.275007047

Araip.1Z5U3	NA	NA	NA	-4.32192809	-2.39592868
Araip.1Z65W	2.381283373	2.615887074	2.286881148	2.150559677	2.584962501
Araip.1Z76L	-0.59946207	-0.68965988	-0.78587519	1.09085343	0.90303827
Araip.1Z8DE	NA	-5.64385619	NA	NA	-3.32192809
Araip.1ZG04	1.189033824	1.464668267	1.316145742	1.63691458	2.321928095
Araip.1ZJ86	-0.64385619	-0.59946207	-0.577767	0.757023247	1.541019153
Araip.1ZK8J	-0.66657627	-0.23446525	-0.23446525	0.970853654	1.250961574
Araip.1ZW9V	0.454175893	0.286881148	0.575312331	0.613531653	0.799087306
Araip.206QL	-0.18442457	-5.05889369	-0.73696559	-5.05889369	-6.64385619
Araip.207MI	-0.28630419	-0.39592868	-0.07400058	1.014355293	1.257010618
Araip.20CID	-2.32192809	0.056583528	-0.08926734	0.948600847	1.432959407
Araip.20CWB	1.13093087	1.10433666	0.963474124	1.678071905	2.084064265
Araip.20FU5	-3.32192809	-1.43440282	-2.39592868	1.485426827	1.673556424
Araip.20HUP	3.527320608	3.792855352	3.374343989	4.663913842	4.862947248
Araip.20IHP	3.472487771	3.770829046	3.646162657	2.560714954	2.166715445
Araip.20V0X	0.82374936	1.163498732	0.604071324	2.003602237	2.182692298
Araip.20WLX	2.23878686	2.341985747	2.298658316	3.109360559	3.401903472
Araip.21220	0.22650853	1.700439718	1.411426246	4.37086174	3.303050085
Araip.216VR	-0.04394335	0.855989697	0.443606651	2.056583528	2.286881148
Araip.2178J	4.47118746	4.614709844	4.228049048	5.381283373	5.657068301
Araip.217TR	3.010779839	3.054848477	2.773996325	2.451540833	2.700439718
Araip.21BTV	3.124328135	3.618238656	2.827819025	2.372952098	2.6622055

Araip.21DP5	3.292781749	3.553360503	2.6622055	4.836429131	4.677508242
Araip.21F2H	6.036503334	5.672708196	5.966937979	2.543495883	2.503348735
Araip.21F8G	NA	0.687060688	NA	NA	-5.64385619
Araip.21M98	2.35614381	2.653060017	2.367371066	2.375734539	2.480265122
Araip.21QMU	2.077242999	2.046141782	1.97819563	2.403267722	2.521050737
Araip.21REB	0.526068812	2.44625623	2.324810603	0.286881148	0.98550043
Araip.21RG8	0.214124805	1.084064265	0.411426246	1.384049807	1.35614381
Araip.21S30	-1.78587519	-1.39592868	-1.15200309	-0.81096618	-0.30400619
Araip.21TG8	4.805292456	5.003602237	4.486714373	4.371558863	4.271276259
Araip.21TTJ	-1.28630419	-0.51457317	-0.97143085	2.056583528	3.200064862
Araip.21V2Q	3.71259578	3.464668267	3.377123749	3.023255352	3.658782734
Araip.220UP	1.389566812	1.416839742	1.232660757	1.855989697	1.007195501
Araip.224ZW	-1.94341647	-1.55639335	-1.35845397	0.941106311	1.111031312
Araip.225U5	-6.64385619	-6.64385619	-4.64385619	-2.55639335	-2.12029423
Araip.2290W	2.025028794	2.498250868	2.066950244	3.472487771	3.607626221
Araip.22DFM	4.949534933	5.019701914	4.769771739	5.132988043	5.141596278
Araip.22IQY	NA	NA	NA	NA	NA
Araip.22PIW	1.778208576	2.746312766	2.61117238	2.711494907	2.289834465
Araip.22U9L	2.550900665	3.404630684	3.246408087	4.067810784	4.063502942
Araip.22Y09	1.250961574	0.970853654	0.014355293	3.656496371	3.340562269
Araip.22Y3L	-0.32192809	1.432959407	0.275007047	2.987320866	3.100977648
Araip.233C9	2.693765712	2.803227036	2.797012978	3.381283373	3.680324357

Araip.235AB	-2.32192809	-1.64385619	-3.05889369	1.063502942	1.673556424
Araip.237X1	-1.59946207	-2.12029423	-2.12029423	-2.32192809	-2.39592868
Araip.23JR9	2.891419187	2.639232163	2.627606838	2.10433666	2.500802053
Araip.23LH0	-1.28630419	-0.91593574	-1.12029423	-1.12029423	-1.08926734
Araip.23M3Y	-3.32192809	NA	NA	-0.83650127	-2.39592868
Araip.23MFU	-0.28630419	0.014355293	-0.13606155	1.15704371	1.891419187
Araip.23NEF	3.224966365	3.578938713	3.196921734	3.252476214	3.196921734
Araip.23QAA	1.269033146	1.713695815	1.214124805	1.731183242	1.891419187
Araip.23X2M	-3.47393119	-3.32192809	-4.32192809	-0.39592868	-0.55639335
Araip.2404G	1.632268215	1.773996325	2.111031312	2.565597176	2.849999259
Araip.2412K	0.475084883	1.111031312	0.970853654	1.469885976	2.182692298
Araip.246YY	0.411426246	1.550900665	0.584962501	2.545968369	2.384049807
Araip.24FNG	2.182692298	2.560714954	2.350497247	3.010779839	3.651912745
Araip.24GT1	-1.02914635	-1.05889369	-1.68965988	-1.39592868	-2
Araip.24N70	2.40599236	2.786596362	2.548436625	3.042644337	3.228049048
Araip.24Q48	2.427606173	3.145677455	2.817623258	4.000901403	4.125155131
Araip.24TKU	-0.577767	-0.59946207	-1.55639335	-0.81096618	0.124328135
Araip.24WNV	0.871843649	0.90303827	0.622930351	1.799087306	2.956056652
Araip.24ZZQ	NA	-5.64385619	-5.64385619	-5.64385619	-3.47393119
Araip.2528D	1.843983844	1.526068812	1.485426827	2.007195501	2.560714954
Araip.2539H	3.428946345	3.518535139	3.187451054	3.622930351	3.775050541
Araip.256VQ	-5.64385619	-6.64385619	NA	-5.05889369	-0.73696559

Araip.25CYT	6.586614642	5.689578726	6.430117975	0.757023247	0.584962501
Araip.25NFE	3.427606173	3.432959407	3.124328135	2.967168608	4.498250868
Araip.263ND	NA	NA	NA	NA	NA
Araip.2685R	3.878725341	4.012568674	3.9800253	4.216454865	4.356848079
Araip.26B5V	3.849999259	3.89917563	3.311793718	2.14404637	0.933572638
Araip.26B8A	1.757023247	2.687060688	2.100977648	4.411426246	4.581351247
Araip.26EKA	0.790772038	1.021479727	0.613531653	2.066950244	2.580145484
Araip.26FIE	2.513490746	2.691534165	2.372952098	4.470536865	4.253989266
Araip.26IG1	2.381283373	2.680324357	2.201633861	2.639232163	3.206330648
Araip.26Q67	1.641546029	2	1.875780063	2.22650853	3.250961574
Araip.26QE5	-0.34007544	-0.51457317	-1.02914635	-0.43440282	0.839959587
Araip.26UP8	3.148934105	3.881664619	3.148934105	2.526068812	2.482848283
Araip.26W3E	-1.68965988	-0.78587519	-0.66657627	-2.47393119	-1.39592868
Araip.26WUC	1.765534746	2.253989266	1.879705766	2.232660757	2.689299161
Araip.270IQ	1.5360529	2.10433666	1.89917563	2.82984956	2.56315813
Araip.274S3	NA	-4.32192809	-3.83650127	1.232660757	2.592158002
Araip.27EE4	0.344828497	0.584962501	0.344828497	0.650764559	1.13093087
Araip.27JTJ	4.661635602	5.015247774	4.669593751	3.10433666	3.196921734
Araip.27Q72	1.03562391	4.132576843	3.365972428	3.008988783	2.344828497
Araip.27R71	4.943921327	5.09212285	5.118525849	4.637494325	4.741466986
Araip.27R9P	3.581351247	5.533563348	6.05115455	4.00270252	4.678635348
Araip.27UQJ	NA	-6.64385619	NA	-5.64385619	-4.32192809

Araip.280PC	1.244887059	1.339137385	0.739848103	2.702657543	2.827819025
Araip.287MP	NA	-6.64385619	NA	-4.05889369	NA
Araip.28BYA	NA	NA	NA	NA	NA
Araip.28CQG	-6.64385619	-5.64385619	-5.64385619	-4.05889369	-3.18442457
Araip.28HGC	2.408711861	2.424922088	2.648465443	1.232660757	2.066950244
Araip.28HGD	NA	NA	NA	0.831877241	2.032100843
Araip.28KXR	4.904002316	5.23227701	4.925999419	5.612942196	5.512858954
Araip.28YBL	5.769771739	4.280213996	5.087887101	-0.49410907	1.422233001
Araip.290GK	3.624100895	3.82984956	3.531069493	3.743084056	3.930737338
Araip.291LW	2.060047384	0.214124805	0.584962501	2.053111336	2.14404637
Araip.29BDM	-0.43440282	-0.18442457	-0.32192809	2.066950244	2.080657663
Araip.29BWP	0.275007047	0.765534746	0.650764559	2.370164281	2.03562391
Araip.29FLU	0.855989697	2.042644337	0.678071905	3.612352499	3.82984956
Araip.29WB6	1.526068812	1.454175893	1.275007047	1	1.794935663
Araip.2A3ZP	2.283921772	2.403267722	2.17951105	2.533563348	2.589763487
Araip.2A4VP	-1.05889369	-0.68965988	-1.47393119	0.669026766	0.887525271
Araip.2A5U1	-2.39592868	-2.25153877	-3.32192809	-0.21759144	-0.51457317
Araip.2A6VZ	-3.05889369	-1.94341647	-4.05889369	0.678071905	0.918386234
Araip.2A9KZ	1.475084883	2.073820233	1.761285273	2.3305584	2.508428653
Araip.2AE2F	2.35614381	2.715893371	2.367371066	3.852997588	3.845991771
Araip.2AZ3I	3.621758857	2.891419187	2.50589093	3.476381688	3.960697039
Araip.2B2W	1.339137385	1.608809243	1.427606173	2.358958826	2.516015147

U

Araip.2B884	NA	NA	NA	NA	-5.64385619
Araip.2BU9K	1.613531653	2.10433666	1.726831217	2.319039816	2.568032105
Araip.2C3K4	3.270528942	3.553360503	3.498250868	2.488000771	3.087462841
Araip.2C5G8	0.321928095	0.90303827	-0.04394335	2.698218478	2.46727948
Araip.2CH00	3.881664619	4.482202926	4.634012356	3.592158002	3.722466024
Araip.2CJ7I	-2.83650127	-1.64385619	-2.05889369	-1.35845397	-0.39592868
Araip.2D0HD	1.589763487	3.392317423	3.266036894	0.23878686	1.304511042
Araip.2D392	0.422233001	0.411426246	0.613531653	1.691534165	1.480265122
Araip.2D8LN	2.59454855	4.297925053	3.893362211	1.298658316	2.3950628
Araip.2E2K8	NA	-5.64385619	-6.64385619	-1.02914635	4.435628594
Araip.2E6XX	2.95419631	3.215678597	2.935459748	2.657640005	2.266036894
Araip.2EA2I	2.831877241	3.364572432	2.545968369	5.052241981	5.061776198
Araip.2EA83	5.524502537	6.247168	5.699884729	6.18705509	6.216648867
Araip.2EE1I	6.691394579	6.347311316	6.400196342	3.058316496	2.987320866
Araip.2ES43	2.44625623	2.931683057	2.577730931	2.944858446	3.168321116
Araip.2ES8K	NA	NA	NA	NA	NA
Araip.2F21P	3.624100895	3.589763487	3.816599707	-2.39592868	-1.51457317
Araip.2F6VL	5.048759312	5.38024459	5.076388069	6.089159132	6.193771743
Araip.2FX35	2.998195503	3.304511042	3.206330648	3.367371066	3.541019153
Araip.2G1DX	NA	NA	NA	0.97819563	0.176322773
Araip.2G27W	3.351910961	2.849999259	2.659924558	3.40599236	3.87774425
Araip.2G28P	-0.37706965	0.028569152	-0.51457317	2.010779839	1.709290636

Araip.2G506	1.97819563	2.150559677	1.914564523	2.414135533	2.61117238
Araip.2G7LE	0.201633861	1.914564523	2.010779839	3.053111336	2.761285273
Araip.2GB7B	1.827819025	1.941106311	1.541019153	1.372952098	1.40053793
Araip.2GH89	-0.32192809	-0.37706965	-1.35845397	0.367371066	0.731183242
Araip.2GK61	NA	NA	NA	NA	-4.64385619
Araip.2GN8J	2.817623258	2.924099886	2.634593268	2.974529312	3.365972428
Araip.2H071	-0.30400619	-0.64385619	-0.45403163	5.303415463	4.723012396
Araip.2H1ED	2.275007047	2.648465443	2.241840184	4.372952098	4.331275267
Araip.2H2XV	2.163498732	2.392317423	2.025028794	2.82984956	2.765534746
Araip.2H61M	3.266036894	3.953265239	3.745237332	4.828834649	6.141800612
Araip.2H669	-3.47393119	-1.68965988	-1.94341647	-1.83650127	-2.32192809
Araip.2HD3N	3.418189948	3.987320866	3.738767837	4.204766751	4.203201156
Araip.2HK29	2.625270489	2.841973119	2.443606651	1.646162657	1.794935663
Araip.2HU43	1.839959587	2.389566812	2.324810603	3.063502942	3.244887059
Araip.2HX98	5.449891473	5.569855608	5.628190335	2.981852653	2.835924074
Araip.2I121	3.007195501	2.970853654	2.803227036	2.477677328	2.780310099
Araip.2I18H	-1.78587519	-2.39592868	-1.64385619	-1.51457317	-1.73696559
Araip.2I1EB	2.341985747	2.032100843	2.347665656	3.340562269	3.454175893
Araip.2I21U	0.163498732	-0.4150375	-0.30400619	-0.53533173	-0.01449957
Araip.2I50M	1.622930351	1.933572638	2.003602237	3.10433666	2.969012308
Araip.2I7IS	1.367371066	2.080657663	1.891419187	1.042644337	2.275007047
Araip.2I7WK	NA	NA	NA	NA	-4.05889369

Araip.2I89L	3.735522177	3.709290636	3.435628594	3.676944359	4.040892431
Araip.2J0CW	0.214124805	0.5360529	0.137503524	1.66448284	2.087462841
Araip.2JH3B	0.815575429	1.495695163	1	2.513490746	2.503348735
Araip.2JM6B	2.577730931	2.344828497	2.488000771	1.500802053	2.269033146
Araip.2JP01	7.719388821	6.587064898	7.086401646	3.850999395	4.001802243
Araip.2K79X	-5.64385619	-4.32192809	-5.64385619	-1.83650127	-2.64385619
Araip.2K8HT	NA	-6.64385619	-6.64385619	-5.05889369	-6.64385619
Araip.2K9WW	3.613531653	3.691534165	3.323370069	4.196921734	4.337711092
Araip.2KR54	0.765534746	0.641546029	0.275007047	4.234961095	4.168321116
Araip.2KU4C	4.025914699	3.986410935	3.854993017	4.780835002	4.882153917
Araip.2L0M6	2.007195501	1.350497247	1.327687364	-0.47393119	-0.49410907
Araip.2L5W7	1.550900665	3.148934105	3.566815154	-2.47393119	-2.32192809
Araip.2L6KD	4.579542225	5.09423607	4.713695815	4.831370588	4.660495132
Araip.2LE64	3.23878686	3.10433666	3.142413438	1.847996907	2.03562391
Araip.2LP4Y	-5.64385619	-4.64385619	-4.32192809	0.704871964	-1.05889369
Araip.2LT0K	5.686220365	6.538072503	6.390770849	8.654743421	7.938109326
Araip.2LX3X	1.691534165	1.98550043	1.941106311	2.17951105	2.350497247
Araip.2M1DM	0.056583528	1.007195501	-0.66657627	2.887525271	3.390942773
Araip.2M3GS	3.198494154	3.493134922	3.166715445	2.10433666	2.778208576
Araip.2M8UJ	1.765534746	2.185866545	1.855989697	2.64385619	2.922197848
Araip.2MA0U	2.956056652	2.815575429	2.375734539	3.295723025	3.786596362
Araip.2MY4B	NA	NA	NA	NA	NA

Araip.2N3AS	1.09085343	1.344828497	1.021479727	2.09423607	2.250961574
Araip.2N7ZX	1.269033146	1.339137385	2.17951105	0.367371066	0.847996907
Araip.2NP4Y	2.40599236	2.715893371	2.704871964	-0.49410907	0.495695163
Araip.2NV9I	6.277798821	6.096135325	6.015470808	3.448900951	3.71918344
Araip.2NY3H	NA	NA	NA	NA	NA
Araip.2P1HN	2.744161096	2.613531653	3.092545742	-4.05889369	3.340562269
Araip.2P2KT	1.726831217	2.707082992	-0.32192809	5.633721813	4.984133595
Araip.2Q11V	NA	-4.64385619	-6.64385619	-4.64385619	-5.05889369
Araip.2Q2JE	2.767654798	3.255500733	3.203201156	2.250961574	2.056583528
Araip.2Q3AI	0.275007047	-0.01449957	0.042644337	0.773996325	1.485426827
Araip.2Q9MF	-6.64385619	-5.64385619	-4.64385619	-4.32192809	2.080657663
Araip.2QM99	3.124328135	3.263034406	2.62058641	3.690417096	3.71259578
Araip.2QS8R	0.790772038	0.111031312	-0.07400058	0.40053793	-0.02914635
Araip.2R3UE	1.827819025	2.416839742	1.90303827	2.629939409	2.778208576
Araip.2R6PA	NA	NA	NA	NA	NA
Araip.2R8F4	1.608809243	2.176322773	1.713695815	3.314696526	3.286881148
Araip.2RJ39	7.855429149	7.606886339	7.807741306	4.227278994	4.333423734
Araip.2RQ0L	4.370164281	4.360364277	4.061776198	2.987320866	3.29130886
Araip.2RW57	-1.94341647	-0.73696559	-1.88896869	0.464668267	0.695993813
Araip.2RY51	1.914564523	2.456806149	2.124328135	2.969012308	3.314696526
Araip.2S2EB	4.207111961	4.638073837	4.414812061	4.854494418	4.999098034
Araip.2S7LP	2.289834465	2.813524689	2.61117238	3.427606173	3.502075956
Araip.2S8VQ	NA	-5.05889369	-5.05889369	NA	0.879705766

Araip.2S9Y0	-3.32192809	NA	-4.05889369	-6.64385619	-1.73696559
Araip.2SG63	NA	NA	NA	NA	-5.05889369
Araip.2SH4C	0.176322773	1	0.176322773	0.773996325	0.678071905
Araip.2T05W	NA	-6.64385619	NA	-6.64385619	-3.32192809
Araip.2T40G	0.669026766	1.627606838	0.704871964	2.655351829	2.737686761
Araip.2T4WR	1.163498732	2.046141782	1.700439718	3.201633861	3.297191417
Araip.2T5Q6	1.709290636	2.080657663	1.722466024	2.260025656	2.498250868
Araip.2TN3Y	1.189033824	1.464668267	1.443606651	2.324810603	2.698218478
Araip.2TV5Y	-3.47393119	-3.18442457	-3.47393119	NA	-6.64385619
Araip.2U0KZ	2.821710215	2.851998837	2.533563348	3.147306699	3.173127433
Araip.2U0RL	4.681449265	4.690417096	5.036063689	3.010779839	3.326249701
Araip.2UA97	4.21800615	4.780835002	3.866907978	5.692929286	5.875042803
Araip.2V2W4	1.475084883	1.906890596	1.3950628	2.327687364	2.632268215
Araip.2V326	1.855989697	1.646162657	1.250961574	3.701549057	4.17951105
Araip.2V74Q	-0.35845397	1.521050737	1.704871964	1.475084883	0.757023247
Araip.2W24M	3.517275693	4.081510068	4.313971371	3.722466024	3.528571319
Araip.2W29K	2.918386234	3.488000771	3.184280294	3.800123353	4.044394119
Araip.2W5R5	3.077242999	4.679198571	4.399171094	4.759688488	4.890933022
Araip.2W8EJ	-3.05889369	-1.25153877	-2	0.495695163	2.641546029
Araip.2WB4K	3.135863165	3.112700133	2.969012308	3.560714954	3.613531653
Araip.2WN0Z	-2.64385619	-2.73696559	-2.64385619	4.502712486	4.879705766
Araip.2X2JD	-1.21759144	-0.86249648	-1.21759144	0.782408565	0.545968369

Araip.2X3C3	1.480265122	1.992768431	1.963474124	2.589763487	2.62058641
Araip.2XW30	4.588564737	4.826802684	4.843983844	2.443606651	1.739848103
Araip.2Y3EX	1.049630768	1.042644337	1.150559677	0.250961574	0.310340121
Araip.2Y6XY	3.942983598	4.148120631	4.295723025	2.809414444	3.189033824
Araip.2Y8GA	2.298658316	2.416839742	2.250961574	2.553360503	2.765534746
Araip.2YE37	3.384049807	3.708187236	3.404630684	2.678071905	2.416839742
Araip.2Z354	0.839959587	1.182692298	1.275007047	1.195347598	2.313245852
Araip.2Z41P	3.938285792	4.195347598	4.161887682	0.757023247	1.59454855
Araip.2ZN3C	2.111031312	2.295723025	2.117695043	3.301587647	3.276496666
Araip.3047C	9.73753467	9.323234945	9.685099172	6.164303583	6.171527106
Araip.306R5	-1.78587519	2.653060017	2.49057013	-0.05889369	0.263034406
Araip.3074P	-1.78587519	-1.02914635	-1.32192809	-0.34007544	-0.12029423
Araip.308FD	NA	NA	NA	NA	NA
Araip.30QH3	-1.55639335	-0.88896869	-1	0.097610797	2.169925001
Araip.30QVU	NA	-6.64385619	NA	-3.32192809	-4.64385619
Araip.30SUW	5.091699834	5.280956314	5.23457796	6.541019153	6.403097262
Araip.30XFU	2.220329955	3.929790998	3.638073837	2.895302621	3.307428525
Araip.313P6	NA	NA	NA	NA	-5.64385619
Araip.313YK	1.879705766	2.295723025	1.98550043	3.124328135	3.484138131
Araip.31EFM	NA	NA	NA	-5.05889369	-5.05889369
Araip.31SQ6	1.959770155	2.232660757	1.769771739	3.114367025	3.106013238
Araip.31UA1	2.310340121	2.989139007	2.761285273	3.392317423	3.602884409

Araip.31XHT	2.518535139	2.577730931	1.794935663	4.303050085	4.423578171
Araip.31ZB6	-3.64385619	-2.12029423	-2.39592868	0.432959407	1.40599236
Araip.3230M	0.918386234	1.333423734	1.257010618	1.40599236	1.310340121
Araip.3266N	NA	-5.64385619	-6.64385619	-4.32192809	-1.12029423
Araip.3269G	4.776630423	6.432959407	6.144658243	7.506446438	7.129695157
Araip.3280G	NA	NA	NA	NA	NA
Araip.32BSU	2.14404637	2.989139007	1.956056652	2.411426246	2.498250868
Araip.32FLV	1.333423734	1.859969548	1.82374936	2.292781749	2.397802962
Araip.32MBN	1.49057013	2.025028794	1.321928095	2.049630768	2.111031312
Araip.32U31	-0.18442457	1.189033824	0.084064265	2.819668183	3.030336078
Araip.32W9F	1.613531653	2.066950244	2.283921772	0.765534746	2.887525271
Araip.32X2R	0.40053793	0.895302621	0.90303827	1	1.3950628
Araip.32X6B	NA	-3.32192809	-3.83650127	-3.05889369	-2.39592868
Araip.32YK0	2.518535139	2.50589093	2.117695043	2.250961574	2.361768359
Araip.332V3	0.124328135	-0.28630419	-0.23446525	-0.30400619	1.575312331
Araip.333QY	3.527320608	3.346247774	4.339137385	1.752748591	3.502075956
Araip.333ZF	0.659924558	1.195347598	0.555816155	2.835924074	2.925999419
Araip.337EV	NA	NA	NA	NA	-6.64385619
Araip.33CRB	-4.32192809	-3.18442457	-4.05889369	-3.32192809	-5.05889369
Araip.33H23	4.837438715	4.515384461	4.23572706	1.859969548	2.066950244
Araip.33PYF	2.937344392	3.276496666	2.914564523	4.075532631	4.321206567
Araip.33RQP	2.550900665	3.319039816	2.673556424	5.431957188	5.780835002
Araip.33SF4	3.070389328	3.077242999	3.434294618	3.295723025	4.420212891

Araip.33UB6	-2.94341647	-2.55639335	-3.47393119	-0.4150375	-0.43440282
Araip.33YRY	1.469885976	0.526068812	-0.13606155	2.451540833	1.914564523
Araip.344D4	NA	NA	NA	-3.32192809	NA
Araip.3485Y	2.032100843	2.12763328	2.070389328	3.13422094	3.713695815
Araip.348XC	1.23878686	1.35614381	1.350497247	-0.35845397	0.344828497
Araip.34D6V	1.416839742	1.650764559	1.189033824	2.929790998	3.277984747
Araip.34I8K	3.881664619	3.631104282	3.529820947	4.030336078	3.862947248
Araip.34N7T	-3.05889369	-3.83650127	-2.64385619	NA	NA
Araip.34S35	4.100977648	4.253232939	3.933572638	4.512858954	4.721372659
Araip.35416	2.680324357	3.266036894	2.922197848	4.654206378	4.780310099
Araip.354H4	0.250961574	0.782408565	0.201633861	2.198494154	1.941106311
Araip.357WQ	3.085764554	2.825785627	2.361768359	2.03562391	1.752748591
Araip.35KW5	0.50589093	1.03562391	0.367371066	2.776103988	2.821710215
Araip.35NZV	3.075532631	2.805292456	2.908812908	2.150559677	2.229587923
Araip.35TV0	3.870857864	4.19298317	2.659924558	2.790772038	3.368768349
Araip.3630D	-4.64385619	NA	-6.64385619	NA	-3.64385619
Araip.363DR	-2.94341647	-3.05889369	-4.32192809	-1.25153877	-0.64385619
Araip.3671W	0.22650853	0.622930351	0.584962501	1.50589093	1.521050737
Araip.36D69	-1.43440282	-0.97143085	-1.32192809	-1.15200309	-0.18442457
Araip.36HY2	NA	-3.32192809	-3.47393119	-1.32192809	-1.68965988
Araip.36PI6	2.771885579	2.304511042	2.22342255	4.388189537	4.005399988
Araip.36RIF	0.344828497	0.659924558	0.422233001	2.010779839	2.370164281

Araip.36SP7	-1.94341647	-1.59946207	-2.18442457	-1.78587519	1.124328135
Araip.374K5	3.821710215	4.447579197	4.196134881	3.626439137	4.117695043
Araip.37594	-3.18442457	-1.12029423	-0.86249648	-1.05889369	-0.07400058
Araip.37BUF	NA	-6.64385619	NA	-0.35845397	-0.32192809
Araip.37ILI	2.263034406	2.713695815	1.669026766	3.973611276	3.77925972
Araip.37KVD	2.925999419	2.86393845	3.220329955	3.051372102	2.543495883
Araip.37P5S	2.739848103	3.531069493	2.641546029	2.847996907	2.765534746
Araip.37R5L	-2.05889369	-1.35845397	-1.55639335	-1.32192809	0.757023247
Araip.37S9E	2.435628594	3.142413438	2.691534165	-0.34007544	-0.97143085
Araip.37TH3	4.698774109	4.194559886	4.457462965	3.56193706	2.952333566
Araip.37TY6	6.213541702	7.783194703	6.845490051	4.201633861	5.576824431
Araip.37V98	1.757023247	1.495695163	1.510961919	1.13093087	1.137503524
Araip.37WSW	2.835924074	2.671293372	2.648465443	2.906890596	3.109360559
Araip.37ZE6	3.112700133	3.320484678	3.160274831	0.839959587	0.084064265
Araip.38345	1.627606838	1.510961919	0.948600847	2.087462841	2.333423734
Araip.386YD	-1.78587519	-1.83650127	-2.64385619	1.117695043	2.533563348
Araip.387J2	0.82374936	0.90303827	1.014355293	1.232660757	1.049630768
Araip.38J51	1.344828497	1.641546029	1.448900951	2.166715445	3.001802243
Araip.38JBI	1.584962501	1.59454855	1.786596362	2.070389328	2.010779839
Araip.38QJB	-2	-2	-1.88896869	-0.04394335	0.056583528
Araip.38X68	-5.05889369	-5.64385619	-6.64385619	-4.64385619	-3.47393119
Araip.38XYU	1.807354922	2.217230716	1.565597176	2.720278465	3.14404637

Araip.38ZCB	NA	-4.32192809	-6.64385619	-2.83650127	-1.68965988
Araip.390JY	NA	-5.64385619	-6.64385619	-0.28630419	-0.577767
Araip.39CBS	1.575312331	2.017921908	1.90303827	2.843983844	3.195347598
Araip.39IGX	1.555816155	1.778208576	1.411426246	1.847996907	2.192194165
Araip.39P36	-1.88896869	-3.05889369	-3.47393119	-4.64385619	-4.05889369
Araip.39QP6	-4.32192809	-4.32192809	-6.64385619	0.321928095	1.220329955
Araip.39SAQ	4.487357715	4.553974808	4.222650022	3.207892852	3.572889668
Araip.39SY8	5.121015401	5.303415463	5.025028794	3.811471031	3.691534165
Araip.3A1VT	-3.83650127	-3.64385619	NA	1.056583528	-0.30400619
Araip.3A27H	2.185866545	2.053111336	1.59454855	3.073820233	3.121015401
Araip.3A5RC	NA	NA	NA	NA	-3.64385619
Araip.3A6UX	1.575312331	2.250961574	1.722466024	2.459431619	2.695993813
Araip.3A81Q	6.467116418	7.64508196	7.904122777	7.972175672	7.17512535
Araip.3A86R	0.201633861	0.22650853	0.815575429	-0.25153877	0.176322773
Araip.3AQ0F	3.217230716	3.27351589	3.305970521	2.700439718	2.901108243
Araip.3B3VN	4.866907978	5.050501697	4.695993813	5.065658471	5.329123596
Araip.3B7EF	0.757023247	1.263034406	1.042644337	1.851998837	2
Araip.3B8VN	-1.39592868	-1.08926734	-0.78587519	-0.64385619	1.367371066
Araip.3B9Y6	0.454175893	1.014355293	1.063502942	1.480265122	3.675815931
Araip.3BD5Q	-4.64385619	-4.05889369	-5.05889369	-6.64385619	-3.05889369
Araip.3BJ9Q	-0.16812276	0.545968369	-0.20091269	1.361768359	1.422233001
Araip.3C14J	2.763411574	2.841973119	2.86393845	2.805292456	3.160274831

Araip.3C7F6	1.879705766	2.40053793	2.416839742	2.321928095	2.713695815
Araip.3C7MV	2.813524689	2.192194165	2.014355293	3.252476214	3.64731451
Araip.3CP6N	NA	NA	NA	NA	NA
Araip.3D0I5	1.321928095	2.15704371	1.948600847	1.89917563	2.163498732
Araip.3D13X	-2.32192809	-2.94341647	-3.64385619	-0.16812276	0.163498732
Araip.3D6BD	3.833902077	4.617651119	4.353323291	0.584962501	0.321928095
Araip.3E86P	-1.94341647	-3.32192809	-3.18442457	-4.32192809	-2.12029423
Araip.3E8GX	-2.94341647	-2.55639335	-3.18442457	0.070389328	0.097610797
Araip.3E954	-1.51457317	-1.08926734	-1.59946207	-0.32192809	-0.26881676
Araip.3EZ7B	1.007195501	2.185866545	1.176322773	3.241840184	3.100977648
Araip.3F2A6	3.116031993	3.203201156	2.942983598	3.43162296	3.69265037
Araip.3F4LC	2.653060017	2.485426827	2.596935142	2.430285273	2.62058641
Araip.3F57L	3.054848477	3.304511042	3.037382222	3.087462841	3.042644337
Araip.3F9PG	2.049630768	2.173127433	2.021479727	1.891419187	2.244887059
Araip.3F9W2	NA	NA	NA	NA	NA
Araip.3FA1A	2.440952198	2.815575429	2.669026766	3.321928095	3.622930351
Araip.3FK8B	1.782408565	1.150559677	2.084064265	-0.64385619	1.560714954
Araip.3G6B2	-5.05889369	-3.47393119	-5.05889369	-1.51457317	-0.64385619
Araip.3GM3S	1.941106311	2.100977648	1.879705766	2.831877241	2.961623328
Araip.3GQ6X	1.021479727	1.35614381	1.232660757	1.811471031	2.087462841
Araip.3GT2G	1.014355293	1.550900665	1.117695043	1.678071905	1.448900951
Araip.3H4X4	3.890446693	4.220329955	3.938285792	4.229587923	4.391630262

Araip.3H7G0	4.011674533	3.303050085	3.097610797	3.058316496	4.078097423
Araip.3HJ42	1.59454855	1.117695043	1.464668267	-3.18442457	-2
Araip.3HN76	-4.64385619	-3.47393119	-3.64385619	1.427606173	1.292781749
Araip.3I7XZ	0.739848103	1.843983844	1.195347598	2.419538892	2.560714954
Araip.3I7ZD	0.678071905	1.819668183	0.722466024	3.243364426	3.275007047
Araip.3J01I	-2.39592868	-2.32192809	-1.73696559	-0.35845397	-0.86249648
Araip.3J27X	3.161887682	3.476381688	3.23878686	4.284662185	4.67468662
Araip.3J3Q4	2.389566812	2.438292852	1.769771739	2.811471031	2.901108243
Araip.3J4UV	0.097610797	0.5360529	0.495695163	-0.62148838	0.782408565
Araip.3JF99	5.716990894	5.361768359	5.14689956	1.608809243	1.339137385
Araip.3JG6V	0.925999419	0.757023247	0.604071324	-0.25153877	-0.08926734
Araip.3JH87	3.478971805	3.596935142	3.996388746	3.077242999	3.313245852
Araip.3JJ8N	-2.83650127	0.555816155	-2.39592868	-1.21759144	-0.35845397
Araip.3JN5Z	1.726831217	1.974529312	1.735522177	1.560714954	1.992768431
Araip.3JS6L	-3.64385619	-4.32192809	-3.32192809	-0.47393119	2.675815931
Araip.3K0PR	NA	NA	NA	NA	NA
Araip.3K2R6	2.480265122	2.63691458	2.077242999	2.827819025	3.109360559
Araip.3K50N	3.203201156	3.353323291	3.03562391	3.289834465	3.058316496
Araip.3KN0A	-1.15200309	0.411426246	-1.05889369	3.503348735	2.95419631
Araip.3KU1V	-0.86249648	-1	-0.81096618	-0.35845397	-0.97143085
Araip.3L11M	3.59454855	3.797012978	3.879705766	2.347665656	2.606442228
Araip.3LL8X	-0.10469738	1.169925001	0.411426246	1.782408565	2.283921772

Araip.3LR0U	4.148120631	4.196134881	4.089159132	5.711219557	5.508428653
Araip.3M1WU	2.510961919	2.678071905	2.59454855	3.555816155	3.62058641
Araip.3M2RS	2.526068812	2.618238656	2.510961919	2.843983844	3.297191417
Araip.3M2TW	1.981852653	2.392317423	2.09085343	3.121015401	3.531069493
Araip.3M36F	-3.32192809	-0.78587519	-1.59946207	0.263034406	0.176322773
Araip.3M5WT	3.795974694	3.736604875	4.075532631	2.229587923	2.304511042
Araip.3MN07	0.782408565	1.350497247	0.992768431	2.304511042	2.722466024
Araip.3MW2J	3.375734539	3.657640005	3.577730931	3.967168608	4.340562269
Araip.3N4EX	-5.64385619	-6.64385619	-6.64385619	-0.73696559	2.950468414
Araip.3NM2I	1.014355293	1.321928095	1.03562391	0.565597176	-0.02914635
Araip.3NN3F	2.025028794	1.700439718	2.241840184	2.017921908	2.319039816
Araip.3P0PJ	1.411426246	2.495695163	2.03562391	2.416839742	2.887525271
Araip.3P5TL	3.658782734	4.742545234	4.414812061	3.727920455	4.200064862
Araip.3PC35	1.269033146	1.646162657	1.555816155	2.137503524	2.82374936
Araip.3PJ33	-4.64385619	-2.12029423	-0.30400619	-3.47393119	-4.64385619
Araip.3PM87	-0.39592868	-0.66657627	-0.94341647	-0.21759144	-0.73696559
Araip.3Q94J	-5.05889369	-3.47393119	-3.32192809	-0.94341647	-1.73696559
Araip.3Q9LP	3.607626221	4.451540833	3.905928478	0.963474124	0.773996325
Araip.3QG7R	1.250961574	1.049630768	1.10433666	1.220329955	1.565597176
Araip.3QQ7J	1.22650853	1.201633861	1.077242999	1.182692298	1.773996325
Araip.3R01Q	4.838447593	4.843481425	4.704871964	2.485426827	2.799087306
Araip.3R0IT	2.46727948	2.693765712	2.40053793	1.933572638	1.839959587

Araip.3R87M	-0.59946207	-0.30400619	-0.10469738	-0.86249648	0.5360529
Araip.3RS0Y	NA	-4.05889369	-3.64385619	-2.94341647	-4.05889369
Araip.3S1SV	1.321928095	2.100977648	1.769771739	2.925999419	3.065227623
Araip.3S4CE	-0.16812276	-0.71311885	-0.15200309	-1.18442457	0.321928095
Araip.3S762	-1.94341647	-1	-0.68965988	-0.07400058	0.275007047
Araip.3S7QC	NA	-6.64385619	NA	-2.64385619	-5.05889369
Araip.3S8CW	0.765534746	1.350497247	1.257010618	1.82374936	2.438292852
Araip.3S8EX	-3.83650127	-4.05889369	-3.64385619	-1.02914635	-0.51457317
Araip.3SG4B	4.973152038	5.523875551	4.782408565	6.619853147	6.634883637
Araip.3SL7S	4.521050737	4.672990994	4.514753498	5.766860144	4.796493929
Araip.3TF2U	-0.51457317	0.687060688	0.189033824	1.150559677	1.049630768
Araip.3TN6J	NA	NA	NA	NA	-6.64385619
Araip.3U41R	2.508428653	2.801158656	2.49057013	3.29130886	3.953265239
Araip.3U4DB	2.272023189	2.855989697	2.272023189	4.004501392	3.901108243
Araip.3UC9S	2.153805336	2.543495883	2.304511042	1.875780063	2.589763487
Araip.3UE1W	2.304511042	2.336283388	2.414135533	2.060047384	2.485426827
Araip.3UW0X	4.996840648	4.910732662	4.75595577	2.220329955	2.528571319
Araip.3V1RA	1.925999419	1.516015147	2.521050737	2.364572432	2.375734539
Araip.3V9D5	1.214124805	1.545968369	1.275007047	1.63691458	1.835924074
Araip.3V9HH	-4.64385619	-5.64385619	-4.64385619	NA	-5.64385619
Araip.3VW7G	0.555816155	1.718087584	0.992768431	1.879705766	2.189033824
Araip.3W07G	-0.91593574	-0.81096618	-0.71311885	-0.10469738	0.263034406

Araip.3W093	-1.73696559	-1.15200309	-3.32192809	2.189033824	2.364572432
Araip.3W1KE	2.704871964	3.29130886	3.176322773	3.078951341	3.285402219
Araip.3W28R	-2.05889369	1.550900665	1.906890596	0.042644337	1.275007047
Araip.3W2E6	-1.47393119	-0.66657627	-3.47393119	2.430285273	2.689299161
Araip.3WF3M	1.195347598	3.524815928	3.008988783	1.163498732	1.604071324
Araip.3X1VK	3.372952098	3.568032105	3.106013238	3.176322773	3.559491813
Araip.3X35T	NA	-1.68965988	-2.64385619	2.776103988	2.543495883
Araip.3X74X	NA	-5.05889369	-6.64385619	NA	NA
Araip.3X7VM	2.827819025	3.323370069	3.08236197	3.218781168	3.624100895
Araip.3X84U	7.833396134	8.790706886	8.657818616	7.740455397	8.035183997
Araip.3Y5DK	1.220329955	2.100977648	1.5360529	3.252476214	2.983677695
Araip.3Y8R0	-2.64385619	-1.59946207	-3.32192809	0.722466024	1.459431619
Araip.3YS8U	1.66448284	3.66106548	4.050501697	1.257010618	2.464668267
Araip.3Z0P1	3.498250868	4.339850003	3.84899843	6.222456826	6.608661418
Araip.3Z2Y1	-2.25153877	-1.94341647	-2.39592868	0.765534746	0.669026766
Araip.3Z4V7	NA	NA	NA	NA	-1.68965988
Araip.3Z6CU	0.333423734	0.887525271	0.669026766	2.411426246	2.364572432
Araip.3ZU66	NA	NA	NA	NA	NA
Araip.40836	4.86393845	4.554588852	4.427606173	4.231893162	4.523561956
Araip.40FIW	-3.32192809	-0.64385619	-0.97143085	-0.91593574	-2
Araip.40GWN	2.653060017	2.95419631	2.855989697	3.077242999	3.440952198
Araip.40JWQ	3.778208576	4.101818134	3.926948248	5.019701914	5.102658131
Araip.40P7B	3.958842675	4.697106574	4.673556424	4.283180979	4.34553831

Araip.40X13	NA	-6.64385619	NA	NA	-5.05889369
Araip.417P5	-1.12029423	-0.83650127	-2	-4.05889369	-2.39592868
Araip.41DGV	-2.83650127	-3.64385619	-2.94341647	-2.12029423	-0.16812276
Araip.41EGI	-0.71311885	-3.18442457	-2.64385619	NA	-2.55639335
Araip.41RUB	4.973611276	5.534808661	4.597531174	6.321747747	6.20143783
Araip.41YFB	3.026800059	3.097610797	2.46727948	3.788685711	3.600507645
Araip.420T4	0.604071324	0.925999419	0.855989697	2.577730931	2.397802962
Araip.422GS	1.891419187	1.214124805	1.182692298	2.403267722	2.510961919
Araip.423MS	-2.25153877	-1.08926734	-2.05889369	0.23878686	-0.21759144
Araip.4263U	-0.10469738	1	0.941106311	0.214124805	0.613531653
Araip.42EJ9	2.927896454	2.881664619	2.767654798	2.6622055	2.910732662
Araip.42ERT	-0.76121314	2.618238656	2.582556003	1.389566812	1.545968369
Araip.42FFR	1.021479727	1.555816155	1.163498732	1.521050737	2.070389328
Araip.42IVV	2.713695815	2.906890596	2.786596362	2.981852653	3.163498732
Araip.42YVV	4.206330648	4.662775172	4.274261661	4.955126781	5.291677223
Araip.433VX	-5.05889369	-5.64385619	NA	-1.64385619	-1.51457317
Araip.4397U	-2.25153877	-2.55639335	-2.55639335	-6.64385619	-6.64385619
Araip.43FKD	1.851998837	2.40053793	2.22650853	3.102658131	3.344828497
Araip.43I77	-3.05889369	-0.81096618	-0.35845397	-5.64385619	-6.64385619
Araip.43JFQ	-2.73696559	-1.51457317	NA	1.531069493	-0.35845397
Araip.43KWH	NA	NA	NA	NA	NA
Araip.43MG	2.277984747	2.851998837	2.176322773	2.976363636	3.244887059

U					
Araip.43P31	6.920412408	6.527320608	6.28077077	7.42508999	7.705701497
Araip.43TDN	-0.81096618	0.084064265	-0.62148838	2.416839742	2.711494907
Araip.43U91	3.119356177	3.375734539	3.23878686	3.87282876	4.287620044
Araip.43W52	3.472487771	3.803227036	3.580145484	3.654206378	3.912649865
Araip.43WXS	-2.18442457	-2.64385619	-2.12029423	1.739848103	0.176322773
Araip.43ZG0	NA	NA	-6.64385619	NA	NA
Araip.445G9	2.893362211	3.158660175	2.825785627	3.687060688	3.782408565
Araip.44BHE	1.803227036	1.304511042	1.411426246	1.761285273	1.646162657
Araip.44DMF	1.189033824	1.575312331	1.23878686	2.014355293	2.163498732
Araip.44M02	-0.94341647	-1.12029423	-1.15200309	-1.35845397	-0.51457317
Araip.44PA5	3.559491813	3.587364991	3.477677328	2.784503983	3.193771743
Araip.44Q3I	NA	-6.64385619	NA	NA	NA
Araip.44RH2	2.370164281	3.280956314	3.112700133	1.59454855	2.107687869
Araip.44UYF	2.722466024	2.916476644	2.543495883	3.451540833	5.041330607
Araip.44WZX	0.713695815	1.565597176	1.15704371	2.169925001	2.629939409
Araip.4509F	NA	NA	NA	NA	NA
Araip.45253	NA	NA	NA	NA	NA
Araip.45C6Q	1.372952098	1.970853654	1.646162657	2.655351829	2.819668183
Araip.45CYE	2.550900665	2.713695815	2.516015147	3.092545742	3.187451054
Araip.45GIV	0.070389328	0.847996907	0.545968369	3.341985747	3.317593505
Araip.45HI9	1.137503524	2.44625623	2.419538892	1.316145742	1.604071324
Araip.45RHI	-0.88896869	0.163498732	0.321928095	-4.32192809	-0.34007544

Araip.45X5I	2.865918815	3.168321116	3.14404637	3.313245852	3.833902077
Araip.461HE	1.871843649	2.416839742	2.042644337	2.531069493	2.500802053
Araip.46604	2.837943242	3.089159132	2.935459748	3.921245889	4.08491466
Araip.467WI	-0.47393119	0.933572638	0.555816155	2.063502942	3.112700133
Araip.46LQV	1.839959587	2.060047384	1.739848103	3.040892431	3.200064862
Araip.46XVA	1.803227036	-3.32192809	1.859969548	-2.83650127	-4.05889369
Araip.4786J	0.124328135	0.713695815	0.22650853	2.173127433	2.746312766
Araip.47BGG	2.438292852	2.480265122	2.13422094	3.893362211	3.953265239
Araip.47CG9	-4.64385619	-2.47393119	-1.51457317	-0.64385619	1.589763487
Araip.47DVE	9.087568918	8.411722822	8.446752485	4.547819957	6.447083226
Araip.47EMD	NA	NA	NA	NA	-3.32192809
Araip.47I29	4.996388746	5.537606691	4.639810978	6.403778984	6.292965755
Araip.47NV6	5.114367025	5.737416366	5.178316272	6.541483864	6.770300489
Araip.483YA	-1.05889369	-0.51457317	-1.21759144	0.367371066	0.286881148
Araip.485LJ	2.528571319	2.653060017	2.124328135	0.678071905	1.673556424
Araip.488BQ	-0.94341647	-1.05889369	-1.32192809	-1.73696559	-0.21759144
Araip.48FDM	6.169323753	6.288912203	6.432625411	7.691952843	7.603032826
Araip.48FMM	3.209453366	3.303050085	3.021479727	1.077242999	1.028569152
Araip.48P6B	1.673556424	1.811471031	1.819668183	2.698218478	2.914564523
Araip.493E4	-0.62148838	-0.577767	-0.83650127	1.077242999	1.097610797
Araip.494RG	1.815575429	2.370164281	2.056583528	2.622930351	2.744161096
Araip.49GZ0	-5.64385619	-6.64385619	-6.64385619	NA	-5.64385619

Araip.49HT7	-2.83650127	-2.32192809	-3.18442457	-0.73696559	0.028569152
Araip.49NYC	3.836934011	4.266036894	4.042644337	3.173127433	3.646162657
Araip.49S7T	-2.32192809	-2.47393119	-2.55639335	2.508428653	1.700439718
Araip.4A18K	1.40599236	1.695993813	1.50589093	1	0.970853654
Araip.4A1JG	2.039138394	1.974529312	1.673556424	1.526068812	2.788685711
Araip.4A7P4	NA	NA	NA	NA	-6.64385619
Araip.4AW6Y	0.748461233	0.782408565	0.641546029	0.839959587	1.40053793
Araip.4B1GU	-3.64385619	-5.05889369	-3.83650127	-2.32192809	4.759155834
Araip.4B1V4	NA	NA	NA	NA	NA
Araip.4B2QS	3.958842675	3.671293372	3.95419631	2.508428653	2.875780063
Araip.4B6XP	-1.05889369	-0.12029423	-0.59946207	-2.64385619	-3.64385619
Araip.4B8IV	2.792855352	3.510961919	3.135863165	4.338424415	4.377817853
Araip.4BU2D	-0.49410907	0.014355293	-0.64385619	1.09085343	1.531069493
Araip.4BX2B	4.331275267	5.037821465	4.091699834	6.374691752	6.148934105
Araip.4C08I	2.454175893	2.648465443	2.44625623	4.065227623	3.996388746
Araip.4C1AU	-2.64385619	-4.32192809	-5.64385619	0.485426827	0.275007047
Araip.4C49Y	2.733354341	2.9800253	2.879705766	3.384049807	3.527320608
Araip.4CP0D	-3.64385619	-2.73696559	-3.18442457	-3.47393119	-1.94341647
Araip.4CU64	-0.45403163	-0.28630419	-0.73696559	1.847996907	2.372952098
Araip.4CZ22	-4.32192809	-4.32192809	-5.64385619	-0.66657627	-0.53533173
Araip.4DN6E	4.631686366	4.044394119	3.927896454	4.710393192	4.69432306
Araip.4DP2E	-0.26881676	-0.01449957	-0.55639335	-0.20091269	-0.08926734

Araip.4DS9Y	-1.05889369	-0.32192809	0.485426827	-2.83650127	-2.12029423
Araip.4DY64	NA	NA	NA	NA	NA
Araip.4E6BS	1.232660757	1.799087306	1.23878686	2.09423607	2.358958826
Araip.4E6MH	-0.32192809	-0.23446525	-0.66657627	0.713695815	0.650764559
Araip.4E9YI	-1.55639335	-1.83650127	-1.39592868	-0.76121314	-0.71311885
Araip.4EU14	0.748461233	1.565597176	1.510961919	1.89917563	3.147306699
Araip.4F02G	1.189033824	1.584962501	1.10433666	4.845490051	4.319762428
Araip.4F18W	0.970853654	3.550900665	3.938285792	1.475084883	1.839959587
Araip.4F1IC	3.153805336	3.545968369	3.097610797	2.372952098	2.733354341
Araip.4F7TS	2.414135533	4.904002316	4.708739041	2.10433666	1.350497247
Araip.4FJ07	3.326249701	2.780310099	3.001802243	-0.39592868	0.310340121
Araip.4FT5T	1.257010618	1.220329955	0.970853654	0.887525271	0.344828497
Araip.4G3PJ	0.526068812	0.831877241	0.748461233	1.411426246	1.929790998
Araip.4G5MQ	1.232660757	1.646162657	0.97819563	2.060047384	2.386810946
Araip.4G5WD	4.543495883	4.810443104	4.778208576	0.310340121	0.963474124
Araip.4GI7N	2.283921772	2.733354341	2.448900951	3.412781525	3.72900887
Araip.4GL4N	1.622930351	1.704871964	1.704871964	1.280956314	1.627606838
Araip.4H3RV	1.622930351	1.933572638	1.5360529	2.786596362	2.739848103
Araip.4I4L7	-5.64385619	-2.73696559	-2.94341647	-1.64385619	-1.12029423
Araip.4I67D	1.839959587	2.327687364	1.641546029	2.3950628	2.263034406
Araip.4I8QR	1.918386234	2.253989266	1.545968369	2.788685711	3.436961338
Araip.4IT76	1.464668267	1.700439718	1.250961574	2.865918815	2.837943242

Araip.4J20T	0.014355293	1.13093087	0.545968369	2.23572706	2.411426246
Araip.4J570	0.731183242	1.495695163	1.214124805	1.765534746	1.790772038
Araip.4J7U0	4.221877081	5.357903839	4.084064265	7.171627179	6.9800253
Araip.4JD4I	1.163498732	1.438292852	0.925999419	2.700439718	3.116031993
Araip.4JZ1Y	1.344828497	2.893362211	2.746312766	2.435628594	2.350497247
Araip.4K0TW	7.790641731	8.017810585	7.656353352	8.575198859	8.565292521
Araip.4K0XH	-5.05889369	-6.64385619	NA	-4.64385619	-3.32192809
Araip.4K162	NA	NA	-6.64385619	NA	-6.64385619
Araip.4KB77	0.042644337	0	-1.21759144	3.627606838	4.088311236
Araip.4KH5L	1.344828497	2.107687869	1.722466024	3.578938713	3.550900665
Araip.4KS9Y	2.622930351	2.97819563	2.792855352	3.294253136	3.804260116
Araip.4KU76	1.570462931	1.722466024	1.673556424	2.007195501	2.454175893
Araip.4KW8X	1.839959587	1.731183242	1.66448284	3.10433666	3.298658316
Araip.4L18R	2.495695163	1.10433666	1.028569152	0.815575429	1.014355293
Araip.4L5AH	2.117695043	2.414135533	3.459431619	1.910732662	2.084064265
Araip.4L5CB	-2	-0.81096618	-1.83650127	-0.05889369	0.150559677
Araip.4L730	1.687060688	2.286881148	1.257010618	-2.64385619	-1.02914635
Araip.4L98G	7.66831772	7.327956767	7.9202933	3.976363636	4.161081482
Araip.4LG3M	0.214124805	0.097610797	-0.32192809	-0.73696559	-0.23446525
Araip.4LL3W	3.260025656	2.970853654	2.778208576	3.297191417	3.659924558
Araip.4LL7A	3.846994687	4.021479727	4.06608919	-3.83650127	-2.39592868
Araip.4LV9F	-1.39592868	-1.51457317	-1.25153877	0.014355293	1.250961574

Araip.4M6FA	1.778208576	2.244887059	1.691534165	2.482848283	2.720278465
Araip.4N0QC	4.300123725	4.477677328	4.228049048	1.500802053	2.232660757
Araip.4N45L	2.883620816	2.910732662	2.720278465	3.659924558	3.693765712
Araip.4N5EI	1.40599236	1.350497247	1.560714954	2.169925001	1.422233001
Araip.4N7WF	6.017253839	5.642990313	5.744968348	1.914564523	2.182692298
Araip.4NC09	1.90303827	2.247927513	1.786596362	2.508428653	2.86393845
Araip.4NG5J	2.853995647	3.007195501	2.389566812	2.189033824	2.09085343
Araip.4NI21	1.570462931	1.786596362	1.59454855	1.422233001	1.411426246
Araip.4P1DQ	4.985044962	5.292045492	5.145677455	3.028569152	3.246408087
Araip.4P39C	-5.64385619	-2.47393119	-6.64385619	1.49057013	0.275007047
Araip.4P4HG	3.257010618	3.310340121	3.023255352	3.588564737	3.945794957
Araip.4P78F	-1.43440282	0.411426246	0.50589093	3.169925001	2.456806149
Araip.4PT6K	1.918386234	2.344828497	1.90303827	2.809414444	3.40053793
Araip.4PY6A	1	0.748461233	0.831877241	-0.97143085	0.40053793
Araip.4QE8A	-4.05889369	-3.83650127	-1.43440282	NA	-3.05889369
Araip.4R2IW	3.552131108	3.773996325	3.614709844	4.213347282	4.443606651
Araip.4R6AS	4.363171077	4.112700133	4.250961574	2.735522177	2.809414444
Araip.4R6C8	-2.18442457	-1.47393119	-1.88896869	0.028569152	-0.51457317
Araip.4R8ZS	0.367371066	0.097610797	0.137503524	0.59454855	1.310340121
Araip.4RI8H	5.442943496	5.622930351	5.442611804	6.559338848	7.173527238
Araip.4S2KN	0.678071905	1.049630768	0.695993813	1.704871964	1.700439718
Araip.4S4ZY	1.22650853	1.372952098	0.604071324	0.765534746	1.526068812

Araip.4S7ZN	NA	-5.64385619	-4.64385619	NA	NA
Araip.4SA3R	3.890446693	4.109360559	4.14404637	3.350497247	4.059182199
Araip.4SN9W	2.13422094	2.622930351	2.358958826	2.40599236	2.742006211
Araip.4SP4E	1.752748591	2.028569152	1.510961919	1.411426246	2.277984747
Araip.4T3QB	1.839959587	1.851998837	1.384049807	2.618238656	2.601696516
Araip.4U3W0	1.63691458	2.084064265	1.669026766	3.150559677	3.393690764
Araip.4U431	3.184280294	3.547203025	2.974529312	2.548436625	2.587364991
Araip.4U4XR	-0.37706965	-1.55639335	-1.51457317	4.135042286	4.27351589
Araip.4U5VW	5.215290306	5.490249211	5.155425432	6.055282436	6.017031081
Araip.4U9WI	-0.73696559	0.464668267	0.632268215	-0.04394335	0.097610797
Araip.4UA5Y	2.392317423	2.503348735	2.580145484	2.627606838	2.813524689
Araip.4UB04	4.928370323	5.656782364	4.806324057	2.272023189	2.615887074
Araip.4UD0W	5.360013042	5.147713722	4.861955364	5.495375381	5.784765696
Araip.4UX45	-1.18442457	1.879705766	1.042644337	-0.12029423	-0.13606155
Araip.4V07C	1.773996325	2.13422094	1.859969548	2.634593268	2.731183242
Araip.4V1C3	-2.18442457	-1.94341647	-2.05889369	-1.35845397	-1.64385619
Araip.4V3JQ	2.773996325	2.711494907	2.495695163	3.842978832	3.410069692
Araip.4V5MM	3.301587647	4.145677455	3.109360559	5.324450605	5.268284667
Araip.4V6B3	7.2244841	7.331902234	7.196626714	3.592158002	3.852997588
Araip.4V6RH	4.368069877	4.435628594	4.20867332	4.853496704	5.050936965
Araip.4VW3W	3.139142019	2.933572638	2.996388746	1.516015147	1.443606651
Araip.4WJ5B	-1.35845397	-1.35845397	-2.39592868	0.941106311	1.021479727

Araip.4WS44	1.835924074	1.867896464	1.389566812	2.875780063	2.963474124
Araip.4X1DQ	0.622930351	0.815575429	0.575312331	1.416839742	2.039138394
Araip.4Y3B5	0.111031312	0.782408565	-0.15200309	4.869871406	4.690417096
Araip.4Y5NV	-5.05889369	-5.05889369	-5.64385619	-4.05889369	-3.83650127
Araip.4YN76	3.122672719	3.415488271	3.317593505	3.559491813	3.565597176
Araip.4YP5T	1.929790998	2.23572706	2.014355293	2.508428653	2.695993813
Araip.4YR0R	-0.30400619	-0.35845397	-0.45403163	1.35614381	1.438292852
Araip.4YS5X	1.871843649	2.17951105	2.032100843	2.895302621	3.054848477
Araip.4Z02U	8.623332832	7.961739073	8.148883276	4.148934105	4.511594542
Araip.4Z07Z	1.207892852	1.327687364	1.344828497	0.871843649	1.475084883
Araip.4Z4V8	3.784503983	4.477029653	4.117695043	4.608217853	4.708739041
Araip.4Z79G	-1.51457317	-2.05889369	-2.83650127	-1.32192809	-1.28630419
Araip.500NU	NA	NA	NA	0.731183242	1.589763487
Araip.506UP	3.153805336	3.176322773	3.119356177	3.005399988	3.252476214
Araip.50B92	1.475084883	0.895302621	1.042644337	3.039138394	2.046141782
Araip.50DA0	1.22650853	3.590961241	3.187451054	2.608809243	3.026800059
Araip.50H5N	-2.94341647	-0.83650127	-0.55639335	1.3950628	1.40053793
Araip.50JTJ	4.491211756	4.08491466	3.886550147	2.879705766	2.908812908
Araip.50K7X	2.757023247	2.687060688	2.498250868	3.601696516	3.841973119
Araip.50XHI	0.028569152	1	0.941106311	1.811471031	1.89917563
Araip.50ZEB	1.773996325	2.232660757	1.819668183	2.15704371	2.584962501
Araip.510K0	-4.05889369	-3.47393119	-4.32192809	NA	-4.05889369

Araip.51AE7	0.176322773	-2	-2.32192809	-0.28630419	-2.73696559
Araip.51ELU	0.163498732	0.847996907	0.632268215	2.339137385	2.792855352
Araip.51HAH	0.807354922	1.454175893	0.910732662	1.655351829	1.803227036
Araip.51M35	2.759155834	2.495695163	2.56315813	1.480265122	1.959770155
Araip.51SDF	-0.94341647	-0.07400058	-0.47393119	1.560714954	1.752748591
Araip.51T0U	2.13422094	2.411426246	2.275007047	2.904965719	3.326249701
Araip.51VIE	0.918386234	0.799087306	1.731183242	-0.10469738	0.575312331
Araip.51VQE	1.510961919	1.263034406	1.469885976	0.321928095	1.063502942
Araip.525WX	1.618238656	1.304511042	1.361768359	1.263034406	0.22650853
Araip.529RL	4.885086225	5.511278265	4.575312331	6.498889087	6.238595812
Araip.52W96	0.641546029	0.799087306	0.176322773	2.448900951	2.493134922
Araip.52WME	0.201633861	0.321928095	0.310340121	1.22650853	1.220329955
Araip.52XKK	-4.64385619	-3.83650127	-5.64385619	1.992768431	2.263034406
Araip.52Z96	NA	NA	-6.64385619	NA	-3.18442457
Araip.536TB	1.40053793	0.650764559	1.09085343	-0.94341647	-1.02914635
Araip.53BLN	0.321928095	0.545968369	0.150559677	1.963474124	2.269033146
Araip.53EWD	-4.64385619	-6.64385619	-5.64385619	NA	-2.73696559
Araip.53HJX	NA	NA	NA	NA	NA
Araip.53MRH	-1.88896869	-1.05889369	-2.18442457	1.163498732	1.35614381
Araip.53NP1	-1.08926734	-1.35845397	-2.12029423	-0.21759144	0.23878686
Araip.53WJE	NA	NA	NA	NA	NA
Araip.541ME	NA	-5.64385619	-4.05889369	0.815575429	1.739848103

Araip.5435Q	3.564378169	3.858975614	3.666756592	3.550900665	3.905928478
Araip.543CV	2.198494154	2.575312331	2.397802962	2.655351829	2.861955364
Araip.545DL	3.116031993	3.565597176	3.283921772	4.130107179	4.587364991
Araip.54D0C	1.333423734	1.475084883	1.443606651	1	1.744161096
Araip.54D8D	-2.39592868	-3.32192809	-2.94341647	-3.32192809	-1.43440282
Araip.54EQT	NA	-6.64385619	NA	NA	-2.25153877
Araip.54FE9	-1.39592868	-0.16812276	-1.35845397	1.454175893	1.49057013
Araip.54QEU	2.286881148	2.66448284	2.472487771	3.416839742	3.207892852
Araip.54Y6W	2.367371066	3.087462841	2.99095486	2.935459748	3.122672719
Araip.54YKW	2.10433666	2.754887502	1.718087584	-3.83650127	-1.83650127
Araip.551RZ	-0.18442457	1.427606173	1.292781749	1.327687364	1.475084883
Araip.553HN	1.765534746	1.521050737	1.389566812	0.713695815	0.918386234
Araip.555KP	0.941106311	0.669026766	-0.34007544	2.965322548	1.613531653
Araip.55652	NA	NA	NA	NA	NA
Araip.5582Q	2.275007047	2.613531653	2.765534746	3.001802243	3.788685711
Araip.55A1G	-6.64385619	NA	-6.64385619	-5.64385619	5.97384084
Araip.55F8V	-1.83650127	-2.05889369	-2.55639335	-2.64385619	-1.08926734
Araip.55L6R	2.289834465	2.541019153	2.367371066	3.249445341	3.512226887
Araip.560IW	1.111031312	1.163498732	0.555816155	-0.83650127	0.831877241
Araip.563L5	-0.86249648	-0.45403163	-1.18442457	2.63691458	2.942983598
Araip.564FB	5.099295204	5.781621998	5.113117036	6.669735463	6.325530332
Araip.564H9	2.464668267	2.821710215	2.726831217	3.165107985	3.820689561
Araip.567RF	1.137503524	1.516015147	1.40053793	3.939226578	3.814550423

Araip.568TN	2.028569152	2.752748591	2.49057013	3.276496666	3.553360503
Araip.569RT	-5.64385619	-5.05889369	-6.64385619	-2.32192809	2.214124805
Araip.56C70	2.472487771	1.799087306	1.66448284	3.558267634	3.675815931
Araip.56MFZ	-0.45403163	0.925999419	0.014355293	3.957914599	3.777156666
Araip.56PXP	0.97819563	1.176322773	0.575312331	2.608809243	2.819668183
Araip.56WDA	-6.64385619	-3.64385619	-5.05889369	-0.88896869	-1.28630419
Araip.572XZ	2.443606651	2.629939409	2.811471031	4.464668267	5.155830172
Araip.5735B	2.871843649	3.23572706	2.99095486	3.845991771	3.850999395
Araip.576WD	4.278728213	4.332707934	4.114367025	4.7322692	4.951867504
Araip.57AWT	-3.83650127	-6.64385619	-4.32192809	NA	-5.64385619
Araip.57HXG	2.891419187	3.028569152	2.989139007	3.818646083	4.152994605
Araip.57L7Q	1.545968369	5.111448698	4.973152038	3.374343989	3.860962798
Araip.57MS8	0.575312331	0.575312331	0.871843649	-1.12029423	-0.10469738
Araip.57QUV	0.757023247	0.713695815	0.163498732	1.10433666	0.137503524
Araip.57R3H	1.650764559	1.641546029	1.570462931	2.596935142	2.916476644
Araip.57S0G	5.218393711	5.6794801	4.946262984	6.62935662	6.529977074
Araip.57TGT	0.124328135	1.292781749	1.925999419	1.316145742	1.704871964
Araip.57VD8	2.063502942	2.313245852	2.201633861	2.64385619	3.148934105
Araip.57WAC	0.344828497	1.59454855	0.443606651	3.003602237	3.037382222
Araip.57ZZX	2.957914599	3.305970521	3.063502942	3.615887074	3.750606505
Araip.5804B	0.659924558	-0.25153877	-0.20091269	0.056583528	0.464668267
Araip.580BK	1.378511623	2.097610797	0.389566812	-0.12029423	1.207892852

Araip.5836D	-3.18442457	-2.18442457	-4.05889369	NA	-5.05889369
Araip.583LJ	2.229587923	2.182692298	2.046141782	2.392317423	2.577730931
Araip.58I5B	1.839959587	2.23878686	1.910732662	2.809414444	3.285402219
Araip.58L9N	1.98550043	2.528571319	2.23878686	3.016139703	3.211012193
Araip.58NSN	1.526068812	2.263034406	1.807354922	2.370164281	2.66448284
Araip.58R6B	-0.49410907	-1.25153877	-0.81096618	-2.73696559	-1.51457317
Araip.58SA1	1.752748591	1.89917563	1.86393845	2.260025656	2.176322773
Araip.58VCE	-0.34007544	-0.71311885	-1.08926734	-1.88896869	-0.73696559
Araip.58W0E	-0.94341647	-0.64385619	-0.35845397	0.189033824	0.485426827
Araip.591ZY	1.895302621	1.66448284	2.378511623	0.992768431	1.195347598
Araip.59D2H	6.459923363	6.28503225	6.252476214	1.117695043	1.427606173
Araip.59D3Z	-3.64385619	NA	-5.05889369	NA	NA
Araip.59E1I	-1.43440282	-1.59946207	-1.55639335	-2.73696559	-1.78587519
Araip.59LK4	2.82374936	3.207892852	2.992768431	3.976363636	4.272023189
Araip.59ZNY	-1.88896869	-0.97143085	-0.55639335	0.163498732	0.659924558
Araip.59ZT3	1.883620816	2.028569152	1.967168608	2.797012978	3.153805336
Araip.5AX8I	-4.64385619	-5.05889369	-4.32192809	-0.21759144	0.23878686
Araip.5BR6I	10.39196315	9.552457769	10.00887677	4.346247774	4.771357409
Araip.5C457	1.077242999	2.23572706	1.883620816	1.372952098	1.646162657
Araip.5C9RI	1.150559677	0.641546029	0.948600847	1.350497247	0.790772038
Araip.5CF12	NA	-6.64385619	-5.64385619	-3.83650127	-2
Araip.5CH0R	1.565597176	1.361768359	1.416839742	4.167518504	4.08236197

Araip.5CL0B	1.608809243	2.09423607	2.12763328	3.153805336	3.319039816
Araip.5CP68	3.765534746	4.401903472	3.519793486	5.423241996	5.203201156
Araip.5D9GX	NA	NA	NA	NA	-5.05889369
Araip.5DF3C	2.053111336	2.247927513	2.207892852	2.778208576	3.334854269
Araip.5DJ69	-1.28630419	-0.34007544	0.641546029	1.13093087	1.464668267
Araip.5E8U9	-2.05889369	-1.39592868	-2.47393119	-2.12029423	-1.32192809
Araip.5F3C7	1.722466024	1.819668183	1.459431619	1.963474124	3.142413438
Araip.5FB8R	2.869871406	3.215678597	2.976363636	2.9202933	3.217230716
Araip.5FG6Z	5.409730354	5.805550425	5.720004787	5.014801602	4.98458935
Araip.5FM9T	2.283921772	2.518535139	2.321928095	2.924099886	3.241840184
Araip.5FP0F	-0.86249648	-0.34007544	-1.08926734	0.992768431	1.207892852
Araip.5FY0D	1.790772038	1.996388746	1.895302621	1.847996907	2.192194165
Araip.5G3Z8	-3.83650127	-3.83650127	-5.64385619	-4.32192809	-3.47393119
Araip.5G7EV	-1.39592868	-1.43440282	-1.64385619	1.292781749	0.687060688
Araip.5G9GQ	-2.73696559	-3.64385619	-3.83650127	-0.20091269	-0.577767
Araip.5GT5G	3.333423734	3.537296067	3.200064862	4.639810978	4.869871406
Araip.5H0GE	-1.88896869	-1.47393119	-2.32192809	0.622930351	1.23878686
Araip.5HK6K	-3.83650127	-2.05889369	-3.18442457	1.66448284	1.464668267
Araip.5HV78	4.008988783	4.183486514	4.10349764	4.479618608	4.919340082
Araip.5I1EE	-0.02914635	-0.05889369	-0.62148838	-2	-0.37706965
Araip.5I7AM	2.381283373	2.22342255	1.580145484	3.340562269	3.317593505
Araip.5I8PV	-2.64385619	2.61117238	1.310340121	0.90303827	1.14404637

Araip.5IP7M	0.516015147	1.014355293	1.070389328	2.996388746	2.784503983
Araip.5J1JM	5.068240861	4.61176256	4.898692073	2.266036894	2.885574364
Araip.5J271	0.847996907	1.269033146	1.150559677	1.608809243	2.114367025
Araip.5J2NZ	-1.43440282	-1.32192809	-1.51457317	-2.47393119	1.344828497
Araip.5J60P	-4.32192809	-3.47393119	-2.64385619	-3.64385619	4.308885057
Araip.5J63B	1.422233001	1.40053793	1.448900951	1.589763487	2.295723025
Araip.5J7CQ	1.944858446	1.989139007	1.704871964	0.622930351	1.275007047
Araip.5JD7H	1.280956314	1.59454855	1.722466024	2.021479727	2.765534746
Araip.5JH12	4.794935663	4.77452353	4.698218478	3.22650853	3.491853096
Araip.5JH6G	0	1.042644337	0.263034406	3.09592442	3.460742564
Araip.5JT26	3.12763328	2.837943242	2.803227036	2.558267634	2.827819025
Araip.5L4N3	2.017921908	2.613531653	2.173127433	3.158660175	3.423578171
Araip.5M5DL	2.737686761	2.881664619	2.580145484	4.589763487	4.628773595
Araip.5MC2N	1.411426246	0.432959407	0.056583528	0.963474124	1.169925001
Araip.5MD11	-1.21759144	-2.18442457	-1.94341647	-2.18442457	-2.47393119
Araip.5MF6L	3.012568674	3.097610797	2.883620816	3.715893371	4.047887329
Araip.5MK79	3.246408087	3.214124805	3.307428525	3.608809243	4.022367813
Araip.5MT98	-0.01449957	-0.66657627	-0.01449957	-0.05889369	-0.81096618
Araip.5N24I	5.22881869	5.004501392	5.123086751	2.3305584	2.435628594
Araip.5N6GI	NA	-6.64385619	-5.05889369	-3.64385619	-2.83650127
Araip.5NJ9K	-1.12029423	-1.05889369	-1.51457317	0.070389328	0.298658316
Araip.5NU5L	2.053111336	2.44625623	2.22342255	2.813524689	2.865918815

Araip.5P4LE	5.838195439	6.234386354	5.579240501	6.616622355	6.594399256
Araip.5P589	-2	-1.05889369	-1.55639335	0.189033824	0.855989697
Araip.5Q25R	0.35614381	1.03562391	1.14404637	0.669026766	0.815575429
Araip.5QY7Z	-1.64385619	0.189033824	-1.32192809	1.182692298	0.722466024
Araip.5QZ4M	-0.64385619	0.333423734	-1.18442457	4.363171077	4
Araip.5R1U0	NA	NA	NA	NA	NA
Araip.5R38D	NA	NA	NA	NA	3.249445341
Araip.5R4LP	4.185866545	3.518535139	3.691534165	-1.25153877	-0.21759144
Araip.5R5N0	1.40053793	1.761285273	1.485426827	1.333423734	1.485426827
Araip.5RN6F	2.454175893	2.518535139	2.472487771	0.137503524	0.641546029
Araip.5S1QP	3.588564737	4.409390936	3.575312331	5.768449015	5.733083132
Araip.5S35P	NA	NA	NA	NA	-3.83650127
Araip.5SR4K	3.810443104	5.050501697	5.343052435	4.683134985	4.964860664
Araip.5T5JE	3.388189537	3.454175893	3.777156666	3.407352751	3.528571319
Araip.5T83W	3.124328135	3.341985747	2.987320866	3.983677695	3.977279923
Araip.5U4XM	NA	-4.64385619	-3.18442457	NA	NA
Araip.5U7G7	4.229587923	4.125155131	4.150559677	4.161081482	4.486070744
Araip.5U8GK	4.997744026	5.255123015	5.298658316	3.112700133	3.385431037
Araip.5UJ5P	4.922197848	4.686500527	4.937815169	4.329841177	5.21490191
Araip.5V540	0.807354922	0.86393845	0.669026766	1.835924074	1.871843649
Araip.5V6AL	1.084064265	0.669026766	0.963474124	-0.18442457	0.790772038
Araip.5V8J3	3.351910961	3.378511623	3.375734539	-0.08926734	0.137503524
Araip.5VE9R	-4.32192809	-3.32192809	-3.47393119	-1.21759144	-0.76121314

Araip.5VP72	5.638653116	5.725741157	5.766595162	3.298658316	3.486714373
Araip.5VX8G	NA	-5.64385619	NA	-3.64385619	0.137503524
Araip.5WT0G	NA	NA	NA	-4.64385619	NA
Araip.5X628	3.427606173	4.699329526	4.381283373	-0.10469738	1.819668183
Araip.5XB02	NA	NA	NA	-5.05889369	0.575312331
Araip.5XD8H	-4.05889369	-4.32192809	NA	-1.39592868	0.765534746
Araip.5XR69	-2.64385619	-2.05889369	-1.78587519	-2.39592868	-2.32192809
Araip.5XX3F	2.855989697	3.023255352	2.72900887	5.009436756	4.852997588
Araip.5Y1QQ	3.320484678	3.428946345	3.033863452	2.582556003	2.253989266
Araip.5Y2PN	1.521050737	1.575312331	1.448900951	1.111031312	0.871843649
Araip.5Y6S2	1.910732662	2.3305584	2	3.09423607	3.358958826
Araip.5Y8KI	-0.07400058	0.847996907	-0.51457317	2.451540833	3.22342255
Araip.5YV6C	1.952333566	2.3950628	2.121015401	3.454175893	3.64385619
Araip.5YY4M	NA	NA	NA	NA	NA
Araip.5Z0QI	2.185866545	2.744161096	2.608809243	5.358958826	5.323730338
Araip.5Z2JS	NA	NA	NA	NA	-6.64385619
Araip.5Z5WT	1.367371066	1.327687364	0.97819563	1.608809243	1.526068812
Araip.5ZJ1R	1.655351829	2.983677695	3.403267722	3.821710215	3.841973119
Araip.5ZJ5X	1.803227036	2.260025656	1.722466024	2.049630768	2.62058641
Araip.5ZK6T	2.339137385	2.56315813	1.541019153	3.782408565	4.333423734
Araip.5ZR88	1.584962501	2.056583528	1.66448284	2.411426246	2.659924558
Araip.601K9	3.765534746	4.221877081	3.772941338	5.026800059	5.111865964

Araip.6035D	2.889473543	3.078951341	2.799087306	3.08236197	3.503348735
Araip.60749	1.22650853	2.053111336	1.613531653	3.689299161	3.249445341
Araip.608WW	2.765534746	3.196921734	3.063502942	4.06608919	4.253989266
Araip.609WS	NA	NA	NA	NA	-3.18442457
Araip.60EAB	1.443606651	0.90303827	0.622930351	2.901108243	3.137503524
Araip.60F3J	3.7322692	4.200064862	4.054848477	3.155425432	3.068670811
Araip.60GBF	1.014355293	0.970853654	0.871843649	3.434294618	3.350497247
Araip.60HFL	-0.51457317	-0.30400619	-0.37706965	-0.16812276	0.163498732
Araip.60IH6	2.301587647	2.185866545	2.13093087	2.49057013	2.493134922
Araip.60K6G	-1.68965988	-1	-0.97143085	0.669026766	0.298658316
Araip.60LL8	2.568032105	3.148934105	2.704871964	3.331991778	3.382667253
Araip.60Q0E	0.855989697	0.378511623	0.464668267	1.831877241	2.160274831
Araip.60Q87	NA	NA	NA	NA	-5.64385619
Araip.60QH4	2.370164281	2.771885579	2.592158002	3.234194723	3.485426827
Araip.60SDM	-0.16812276	0.378511623	-0.577767	1.316145742	1.944858446
Araip.60WGZ	1.687060688	1.794935663	1.333423734	2.811471031	2.957914599
Araip.60WTQ	1.59454855	2.176322773	1.937344392	3.211012193	3.066950244
Araip.60Y07	3.991861931	4.513490746	4.06091205	5.460414939	5.528571319
Araip.60YE7	-1.94341647	-1.94341647	-2.94341647	2.707082992	2.173127433
Araip.613HG	-2.25153877	-0.81096618	-1.73696559	-0.73696559	-1.25153877
Araip.615VA	NA	NA	NA	NA	NA
Araip.61BVJ	1.432959407	1.201633861	0.86393845	2.23572706	2.039138394

Araip.61EW7	-2.73696559	-2.73696559	-3.83650127	-1.35845397	-0.53533173
Araip.61FFX	1.608809243	2.361768359	2.195347598	3.03562391	3.411426246
Araip.61HR0	1.5360529	1.835924074	1.584962501	1.831877241	2.10433666
Araip.61LLE	1.769771739	2.060047384	1.565597176	2.35614381	2.448900951
Araip.61SU1	1.752748591	2.220329955	1.432959407	3.121015401	3.165107985
Araip.626V8	1.182692298	1.59454855	1.286881148	1.82374936	1.851998837
Araip.627JR	1.35614381	1.910732662	1.50589093	2.192194165	2.477677328
Araip.62AFJ	1.769771739	1.941106311	1.887525271	2.121015401	2.819668183
Araip.62GEG	0.263034406	1.731183242	1.63691458	-1.78587519	0.070389328
Araip.62IDN	-3.05889369	-3.05889369	-3.64385619	0.713695815	0.367371066
Araip.62J85	-5.05889369	-3.32192809	-3.83650127	-2.64385619	-1.47393119
Araip.62MB6	5.489928219	5.016139703	5.393004257	0.526068812	0.613531653
Araip.62MQ Q	1.786596362	2.327687364	2.014355293	2.655351829	2.545968369
Araip.62ZD7	-1.55639335	-2.73696559	-2.73696559	-0.20091269	1.286881148
Araip.62ZJ7	-2.39592868	-1.39592868	-2.05889369	0.545968369	-0.25153877
Araip.6328V	0.82374936	1.189033824	0.765534746	1.521050737	1.709290636
Araip.635QE	-2.05889369	-1.68965988	-1.51457317	1.704871964	1.871843649
Araip.638DS	2.526068812	1.799087306	1.922197848	1.757023247	2.13422094
Araip.63HRP	4.815063017	5.239168879	5.549053029	-0.76121314	-0.28630419
Araip.63PMR	2.229587923	2.553360503	2.553360503	2.956056652	2.845991771
Araip.63QU3	1.731183242	2.073820233	2.073820233	2.353323291	2.918386234
Araip.63YUF	3.760220946	4.06091205	3.87282876	4.516645558	4.672990994

Araip.642G1	-3.83650127	-4.64385619	-4.64385619	-2.39592868	-2.64385619
Araip.64470	-2.73696559	-2.94341647	-2.83650127	-1.51457317	-2
Araip.645I6	1.757023247	2.61117238	2.565597176	2.307428525	3.0721058
Araip.645JG	-1.32192809	-1.25153877	-1.25153877	-1.32192809	0.014355293
Araip.647BE	2.82374936	3.005399988	2.618238656	3.89917563	3.982765463
Araip.64B54	1.077242999	1.097610797	0.575312331	-0.16812276	1.333423734
Araip.64DCF	-2.55639335	-3.83650127	-3.83650127	-3.18442457	-1.83650127
Araip.64E4D	-0.66657627	-1.02914635	-1.15200309	-1.83650127	-0.47393119
Araip.64EI8	-1.43440282	0.604071324	0.565597176	-1.39592868	-0.16812276
Araip.64GAL	2.744161096	2.956056652	2.833902077	2.974529312	3.196921734
Araip.64GCN	0.201633861	2.726831217	3.522306893	1.384049807	2.204766751
Araip.64GPS	4.414812061	4.01346226	3.994579724	0.847996907	1.40599236
Araip.64I9W	2.414135533	2.976363636	2.765534746	3.153805336	3.440952198
Araip.64IP1	3.382667253	3.666756592	3.798050515	3.35614381	2.776103988
Araip.64KG6	-2.47393119	-0.07400058	0.948600847	-0.13606155	-0.35845397
Araip.64L2N	2.121015401	2.495695163	2.408711861	2.500802053	2.99095486
Araip.64P0C	3.377123749	3.778208576	3.578938713	4.312519967	4.356848079
Araip.64TPH	0.695993813	0.855989697	-0.25153877	2.121015401	1.952333566
Araip.64V16	1.40053793	1.650764559	1.378511623	2.017921908	2.687060688
Araip.64VMF	0.176322773	0.464668267	0.084064265	1.59454855	1.773996325
Araip.64WT2	-2.39592868	-2.94341647	-3.47393119	-2.05889369	-2.94341647
Araip.653FM	2.867896464	3.005399988	2.817623258	1.803227036	1.59454855

Araip.6544P	-1.94341647	-1.73696559	-1.78587519	-0.37706965	-0.26881676
Araip.654N3	-3.18442457	-2.39592868	-2.83650127	-1.83650127	-2.05889369
Araip.655BS	5.620879611	5.073820233	5.182692298	5.603477988	3.977279923
Araip.658MF	0.214124805	0.056583528	0.622930351	-1.43440282	-1.68965988
Araip.65AHE	1.731183242	1.257010618	1.232660757	1.286881148	1.389566812
Araip.65APQ	3.846994687	4.294988268	4.374343989	5.894090171	5.521364878
Araip.65AV8	1.021479727	1.744161096	1.150559677	1.773996325	1.970853654
Araip.65H6H	5.389910925	5.124328135	5.220716892	2.40053793	2.752748591
Araip.65HIY	-1.88896869	-0.97143085	-3.18442457	1.350497247	2.220329955
Araip.65HZ4	2.548436625	2.726831217	2.606442228	3.351910961	3.486714373
Araip.65II6	-0.05889369	0.443606651	0.23878686	2.060047384	2.807354922
Araip.65QZN	NA	NA	NA	NA	NA
Araip.65SLH	NA	-6.64385619	NA	NA	NA
Araip.666Q3	0.659924558	0.475084883	0.641546029	1.014355293	1.89917563
Araip.66A5Z	4.057450272	4.169123281	3.671293372	1.765534746	1.5360529
Araip.66GM5	1.250961574	1.86393845	1.531069493	2.10433666	2.3305584
Araip.66GXB	4.647890091	4.495695163	4.420886575	4.977279923	5.009436756
Araip.66MK2	2.010779839	2.198494154	2.482848283	2.070389328	2.648465443
Araip.66N5R	-1.25153877	-1.32192809	-0.59946207	1.526068812	1.713695815
Araip.66P9D	1.03562391	2.350497247	1.263034406	-0.78587519	-0.4150375
Araip.671MH	-0.23446525	0.056583528	-0.08926734	0.014355293	-0.18442457
Araip.673NH	-0.53533173	0.687060688	0.565597176	2.592158002	2.558267634

Araip.6749X	-0.26881676	-0.12029423	-0.45403163	1.111031312	1.304511042
Araip.6759X	2.173127433	1.851998837	1.963474124	1.646162657	1.929790998
Araip.6787A	3.195347598	3.762348816	3.294253136	4.989139007	4.544732656
Araip.678UJ	0.90303827	0.82374936	1.007195501	0.263034406	-0.08926734
Araip.679B1	1.443606651	3.003602237	2.577730931	5.09423607	5.080657663
Araip.67BDE	-0.68965988	-0.05889369	-0.59946207	-0.68965988	-0.01449957
Araip.67BIA	0.475084883	-0.12029423	0.056583528	0.150559677	0.344828497
Araip.67C1Y	-0.21759144	0.014355293	-0.64385619	1.35614381	2.22342255
Araip.67DP4	-0.78587519	0.042644337	-0.25153877	0.933572638	0.855989697
Araip.67K5R	1.372952098	1.963474124	1.469885976	2.702657543	2.959770155
Araip.67QDW	-0.94341647	-0.55639335	0.028569152	2.182692298	1.769771739
Araip.67S5I	NA	-5.05889369	-3.64385619	NA	NA
Araip.67SLS	-0.88896869	-0.39592868	-2.18442457	1.989139007	2.584962501
Araip.67ZY4	4.183486514	4.520422249	4.121015401	4.844988157	5.091699834
Araip.6800W	-0.18442457	0	0	0.5360529	0.632268215
Araip.689G8	1.263034406	1.580145484	1.389566812	2.771885579	2.867896464
Araip.68E16	0.687060688	1.111031312	0.622930351	2.189033824	2.003602237
Araip.68F0J	-5.64385619	-2.55639335	-2.47393119	0.748461233	-0.32192809
Araip.68IEK	NA	NA	-6.64385619	NA	-5.64385619
Araip.68UU0	1.575312331	1.807354922	1.722466024	3.249445341	3.15704371
Araip.68YDH	0.992768431	0.773996325	0.5360529	0.286881148	-0.47393119
Araip.6983Z	0.887525271	1.700439718	1.169925001	2.780310099	2.462052319

Araip.69A15	0.333423734	0.757023247	1.526068812	2.769771739	2.641546029
Araip.69F5Y	2.150559677	2.577730931	2.039138394	3.346247774	3.547203025
Araip.69H3W	1.389566812	1.220329955	0.948600847	-0.34007544	0.23878686
Araip.69K1T	-2.55639335	-1.94341647	-3.64385619	-1	0
Araip.69PE3	2.080657663	2.114367025	1.815575429	2.378511623	2.669026766
Araip.69TNI	1.232660757	1.531069493	1.765534746	0.23878686	1.531069493
Araip.69U4D	0.555816155	0.40053793	0.189033824	2.14404637	1.974529312
Araip.69UD7	-1.94341647	-1.28630419	-2.18442457	-1.39592868	-1.39592868
Araip.69WKH	-5.64385619	-4.05889369	-3.83650127	-3.83650127	-2.83650127
Araip.69YUJ	0.344828497	-0.10469738	0.070389328	-2.25153877	-0.28630419
Araip.6A16L	-4.32192809	-2.94341647	-2	-1.08926734	-1.05889369
Araip.6A4J6	2.469885976	3.218781168	2.589763487	4.046141782	4.159467729
Araip.6A689	1.13093087	1.799087306	1.480265122	2.028569152	2.316145742
Araip.6AB1Y	1.778208576	1.933572638	1.632268215	3.749534268	3.871843649
Araip.6AJ5V	1.66448284	2.344828497	1.704871964	2.364572432	2.531069493
Araip.6AX31	1.970853654	2.117695043	2.097610797	2.700439718	2.889473543
Araip.6B24B	1.232660757	1.867896464	1.718087584	2.140778656	2.560714954
Araip.6B9LC	2.759155834	3.116031993	2.961623328	3.615887074	3.999098034
Araip.6BC4J	2.13422094	3.382667253	2.454175893	5.240314329	5.102658131
Araip.6C47W	1.432959407	2.153805336	1.811471031	2.750606505	2.794935663
Araip.6C7DA	0.50589093	0.963474124	0.454175893	3.869871406	3.588564737
Araip.6C94R	-0.25153877	-0.01449957	-0.62148838	-0.30400619	-0.4150375

Araip.6C9XH	0.782408565	1.682573297	0.59454855	1.815575429	1.937344392
Araip.6CP6H	2.063502942	2.87774425	2.565597176	3.206330648	3.456806149
Araip.6D2E5	2.969012308	2.924099886	2.801158656	3.140778656	3.455491621
Araip.6D36H	-0.43440282	0.014355293	-0.30400619	1.137503524	1.124328135
Araip.6D3E7	3.085764554	4.156234798	4.314696526	4.731183242	4.131754091
Araip.6D6XW	3.173127433	3.509695842	3.102658131	3.559491813	3.951401292
Araip.6DK8B	0.084064265	-0.18442457	-0.47393119	-1.73696559	-0.43440282
Araip.6DV57	-3.47393119	-3.83650127	-3.64385619	0.925999419	1.550900665
Araip.6E6I2	2.498250868	2.867896464	2.568032105	2.558267634	2.744161096
Araip.6E7KQ	3.117695043	3.367371066	3.214124805	3.408711861	3.794935663
Araip.6E9J8	1.709290636	2.40599236	1.970853654	2.648465443	2.733354341
Araip.6EG6G	1.333423734	1.250961574	1.137503524	2.773996325	3.165107985
Araip.6F52Y	NA	-4.32192809	NA	NA	-2.73696559
Araip.6F7K8	5.034743949	5.165107985	5.345893086	4.244125943	4.221103725
Araip.6F88V	-0.23446525	0.070389328	0.286881148	0.956056652	0.454175893
Araip.6FE68	1.257010618	1.464668267	1.189033824	1.618238656	1.811471031
Araip.6FE6S	2.543495883	2.680324357	2.443606651	3.655351829	3.255500733
Araip.6FW03	4.040892431	4.005399988	3.969933275	3.607626221	3.604071324
Araip.6G6P9	NA	NA	NA	NA	-1.78587519
Araip.6G7UA	NA	NA	NA	NA	NA
Araip.6GF41	3.601696516	4.384740587	4.052241981	1.735522177	2.353323291
Araip.6H4KV	2.289834465	3.147306699	2.897240426	3.547203025	3.22342255

Araip.6H93L	NA	NA	NA	NA	NA
Araip.6I0UC	0.613531653	1.244887059	0.659924558	2.477677328	3.192194165
Araip.6I2P8	2.599317794	2.731183242	2.451540833	3.420886575	3.570462931
Araip.6I3DS	2.761285273	2.646162657	2.488000771	3.224966365	3.393690764
Araip.6I56J	-1.25153877	-1.68965988	-1.78587519	-0.43440282	1.007195501
Araip.6IE3Z	2.56315813	2.916476644	2.666756592	2.761285273	2.861955364
Araip.6IK4X	2.498250868	2.339137385	2.389566812	1.475084883	2.6622055
Araip.6IN8N	2.419538892	2.286881148	2.12763328	1.117695043	1.344828497
Araip.6IS7T	4.843983844	5.091699834	4.82984956	5.241077458	5.477353527
Araip.6IZ1V	-0.66657627	-0.73696559	-1.21759144	0.432959407	0.687060688
Araip.6J0KF	NA	NA	NA	-1.05889369	-0.78587519
Araip.6J4W8	0.176322773	0.807354922	0.23878686	1.922197848	2.204766751
Araip.6J64A	0.722466024	1.704871964	1.40599236	NA	NA
Araip.6J7QQ	-0.83650127	-0.81096618	-2.39592868	-1.43440282	-1.68965988
Araip.6JD6V	3.13422094	3.237257771	3.019701914	4.367371066	4.708187236
Araip.6K1VA	4.247168	3.255500733	3.663344619	3.017921908	3.173127433
Araip.6K53R	7.88563537	6.904122777	7.392231546	4.474436043	5.198101209
Araip.6KE2F	3.532316959	4.04176865	4.096767855	3.787641414	4.410748128
Araip.6L19K	3.294253136	3.7473874	3.820689561	3.261530815	3.875780063
Araip.6L1FE	2.03562391	1.835924074	1.769771739	1.86393845	2.229587923
Araip.6L5T3	3.748461233	5.905928478	5.282069075	5.060047384	4.308885057
Araip.6L6QM	NA	-4.64385619	-5.64385619	-5.64385619	-5.05889369

Araip.6L6ZR	-3.32192809	-2.39592868	-3.64385619	0.970853654	2.889473543
Araip.6L8N8	2.908812908	2.333423734	2.577730931	1.66448284	1.906890596
Araip.6L8TP	3.482848283	3.317593505	3.212569339	2.776103988	3.030336078
Araip.6LB90	4.300855871	3.947666157	3.913607512	1.937344392	2.533563348
Araip.6M6IA	0.941106311	1.021479727	0.748461233	4.375039431	4.312519967
Araip.6M87Y	1.974529312	2.59454855	2.22650853	3.775050541	3.847996907
Araip.6ME1P	-0.43440282	-0.23446525	-0.45403163	-1.02914635	-0.83650127
Araip.6MG4Z	NA	NA	NA	NA	-5.64385619
Araip.6MZ14	-1.47393119	0.855989697	-0.43440282	0.5360529	1.416839742
Araip.6N0Z9	3.166715445	3.22342255	3.106013238	3.586164246	3.713695815
Araip.6N7BK	-0.51457317	0.526068812	-0.43440282	1.599317794	1.599317794
Araip.6N7QF	NA	NA	NA	NA	-3.05889369
Araip.6NN2A	2.370164281	2.443606651	2.422233001	2.948600847	3.220329955
Araip.6NP7W	NA	-5.64385619	-6.64385619	NA	NA
Araip.6NR98	1.469885976	1.531069493	1.280956314	1.269033146	1.773996325
Araip.6NT76	2.503348735	2.961623328	2.646162657	3.415488271	3.857980995
Araip.6NV0D	2.739848103	3.152183419	2.780310099	3.442280035	3.407352751
Araip.6NZ59	1.495695163	2.185866545	1.859969548	2.464668267	2.939226578
Araip.6P6UG	NA	-6.64385619	-5.05889369	-1.55639335	-1.55639335
Araip.6P9G9	5.951168129	5.617357262	5.606146078	2.6622055	3.061776198
Araip.6P9JS	5.478001056	5.600804955	5.298658316	6.783980414	5.822730148
Araip.6PA6C	-4.64385619	-2.94341647	-3.83650127	-0.62148838	-0.21759144

Araip.6PA9N	5.689019541	5.345893086	5.360013042	2.748461233	2.526068812
Araip.6PJ8B	1.448900951	1.871843649	1.827819025	5.689578726	5.313971371
Araip.6Q4X9	4.44625623	4.743622676	4.665051614	5.02989455	5.125981654
Araip.6Q5LU	1.956056652	2.229587923	1.887525271	2.080657663	2.372952098
Araip.6Q6H8	-0.68965988	0.042644337	-0.30400619	1.422233001	1.914564523
Araip.6Q6V5	NA	NA	NA	NA	NA
Araip.6Q97S	2.972692654	3.337711092	2.974529312	3.602884409	3.789729251
Araip.6QE8H	4.995936704	5.595145568	4.602884409	6.722192761	6.533563348
Araip.6QX3D	4.023255352	4.697662633	3.800123353	6.254745198	5.934516502
Araip.6R0Q8	-5.05889369	NA	-6.64385619	-5.64385619	-2.94341647
Araip.6R5A3	2.17951105	2.260025656	1.761285273	3.687060688	4.155425432
Araip.6RW05	NA	NA	NA	-3.64385619	-5.05889369
Araip.6RZ29	2.5360529	2.87774425	2.722466024	2.942983598	3.085764554
Araip.6S389	5.521678952	5.615004242	5.373996142	5.601993581	5.838195439
Araip.6S3BR	2.014355293	2.192194165	2.087462841	1.803227036	2.372952098
Araip.6S3M7	-2.64385619	-3.32192809	-3.83650127	-3.05889369	-2.73696559
Araip.6S5NP	1.992768431	2.599317794	2.046141782	2.327687364	2.472487771
Araip.6S6W2	1.056583528	0.622930351	0.422233001	0.722466024	-0.88896869
Araip.6SM52	-5.05889369	-3.47393119	-3.47393119	0.333423734	0.739848103
Araip.6SM8U	-3.32192809	-1.51457317	-3.64385619	0.687060688	0.389566812
Araip.6T0FQ	NA	NA	NA	NA	NA
Araip.6T35D	4.089159132	4.231125158	4.173926932	5.326609251	5.259649121

Araip.6T3P4	2.715893371	2.718087584	2.319039816	1.063502942	1.344828497
Araip.6T61F	NA	NA	NA	NA	NA
Araip.6TB90	2.580145484	2.480265122	2.292781749	2.316145742	1.929790998
Araip.6TJ7D	1.967168608	2.319039816	1.967168608	3.607626221	4.111031312
Araip.6TL19	7.430536184	6.954428984	6.836807808	3.430285273	3.379898164
Araip.6TW9A	3.967168608	4.253989266	4.095080492	4.92314918	5.289465631
Araip.6U899	2.684818738	2.731183242	2.541019153	4.335569006	4.29351763
Araip.6U9BD	-6.64385619	-4.32192809	-4.64385619	-3.64385619	-0.59946207
Araip.6V0UJ	-3.47393119	-3.83650127	-5.64385619	-0.76121314	-0.577767
Araip.6V57K	3.404630684	3.537296067	3.388189537	2.778208576	3.346247774
Araip.6VJ9V	1.321928095	1.82374936	1.367371066	1.339137385	2.039138394
Araip.6VK7W	1.327687364	1.956056652	1.422233001	2.879705766	2.967168608
Araip.6VN5N	6.394548439	6.16651461	5.917431755	5.899900664	6.376950171
Araip.6VR42	1.201633861	1.659924558	1.22650853	1.855989697	1.970853654
Araip.6W0QH	1.10433666	0.86393845	0.310340121	0.50589093	1.516015147
Araip.6W639	3.058316496	3.604071324	3.165107985	4.945326777	4.999549087
Araip.6W75R	-2	-1.59946207	-1.83650127	-2.25153877	0.014355293
Araip.6WG88	-2.94341647	-3.32192809	-2.94341647	-4.64385619	-0.97143085
Araip.6WV2C	-1.02914635	-0.94341647	-1.28630419	1.807354922	1.914564523
Araip.6WY95	0.659924558	1.056583528	-0.71311885	2.99095486	3.657640005
Araip.6X4HD	0.887525271	0.839959587	1.169925001	-0.30400619	-0.12029423
Araip.6XF23	1.959770155	2.386810946	1.989139007	1.778208576	1.3950628

Araip.6Y0I9	5.042644337	5.237640195	5.10433666	4.604664415	4.59812696
Araip.6Y1SP	3.500802053	3.906890596	3.553360503	5.15704371	5.132576843
Araip.6Y4LC	1.941106311	2.114367025	1.992768431	2.639232163	4.021479727
Araip.6YK9P	0.464668267	0.847996907	0.687060688	-0.49410907	0.042644337
Araip.6YN77	-0.35845397	-0.01449957	-0.62148838	3.49057013	3.454175893
Araip.6YP5U	3.655351829	2.440952198	2.82984956	-0.97143085	0.014355293
Araip.6ZC3D	NA	NA	NA	NA	NA
Araip.6ZH5D	-5.05889369	-2.12029423	-4.32192809	2.220329955	2.521050737
Araip.6ZJ3N	0.815575429	1.176322773	0.59454855	3.403267722	3.605257263
Araip.6ZN4Z	2.533563348	3.294253136	2.689299161	3.975446766	3.853995647
Araip.6ZS67	-3.18442457	-4.32192809	-3.18442457	0.855989697	0.82374936
Araip.6ZX2I	2.776103988	2.931683057	2.632268215	2.855989697	3.140778656
Araip.6ZZ85	3.961623328	3.198494154	2.839959587	3.840966704	3.232660757
Araip.702H5	1.550900665	2.260025656	1.713695815	0.526068812	0.584962501
Araip.706JC	NA	NA	NA	NA	NA
Araip.70K3K	-3.32192809	-5.64385619	-3.83650127	-6.64385619	-4.32192809
Araip.70UIF	1.580145484	2.277984747	2.185866545	4.589164237	4.701549057
Araip.712IZ	3.073820233	3.058316496	3.039138394	4.286881148	4.486070744
Araip.713R1	0.970853654	0.871843649	0.526068812	1.981852653	2.310340121
Araip.71CN8	4.087462841	4.408032466	4.498250868	2.811471031	3.371558863
Araip.71CS3	3.40053793	3.822730148	3.459431619	4.493134922	4.577730931
Araip.71FLX	-3.05889369	-2.55639335	-3.32192809	-4.05889369	-2.73696559

Araip.71J30	0.028569152	0.464668267	-0.30400619	1.59454855	1.691534165
Araip.71KEI	1.257010618	1.944858446	1.263034406	3.008988783	2.967168608
Araip.71KZF	2.344828497	2.22342255	2.111031312	3.622930351	3.750606505
Araip.71U6H	-5.64385619	NA	-5.05889369	-0.15200309	1.895302621
Araip.71W8G	-4.32192809	NA	NA	NA	NA
Araip.71XEY	-0.35845397	-0.86249648	-1.21759144	-2.83650127	1.232660757
Araip.71Z4W	0.613531653	0.895302621	0.097610797	2.40599236	2.477677328
Araip.71Z7T	1.669026766	1.967168608	1.695993813	2.684818738	2.87774425
Araip.721GK	4.547819957	5.006298024	4.704318678	5.551516018	6.057233635
Araip.7274A	-0.23446525	-0.30400619	-2.12029423	1.66448284	1.765534746
Araip.727WD	3.797012978	4.036503334	4.272769732	2.182692298	1.922197848
Araip.72BD2	0.669026766	1.028569152	0.910732662	1.933572638	2.189033824
Araip.72FUN	3.600507645	3.629939409	3.568032105	3.901108243	4.304511042
Araip.72N6Y	1.432959407	1.459431619	1.782408565	1.182692298	1.232660757
Araip.72USN	-0.23446525	0.250961574	-1.47393119	2.720278465	3.198494154
Araip.72WPA	1.66448284	2.084064265	1.550900665	3.320484678	3.448900951
Araip.72X39	2.173127433	1.761285273	1.459431619	2.720278465	3.215678597
Araip.72YG4	-0.26881676	-0.35845397	0.669026766	2.883620816	2.782408565
Araip.73385	-5.05889369	-2.73696559	-3.64385619	-0.68965988	0.163498732
Araip.73614	1.422233001	2.792855352	2.327687364	5.289465631	4.789729251
Araip.736QB	2.931683057	2.746312766	2.675815931	1.042644337	1.438292852
Araip.738JR	2.344828497	2.46727948	2.257010618	2.313245852	3.605257263

Araip.73IW9	1.028569152	1.150559677	0.704871964	3.657640005	3.204766751
Araip.73QKW	1.887525271	2.021479727	1.669026766	2.185866545	2.386810946
Araip.73ST3	1.790772038	1.914564523	2.10433666	1.937344392	2.918386234
Araip.73UEX	NA	-6.64385619	NA	-6.64385619	-6.64385619
Araip.73UW5	1.819668183	2.280956314	2.077242999	2.927896454	3.303050085
Araip.73VEI	1.10433666	1.427606173	1.35614381	3.43162296	3.589763487
Araip.73WZS	0.925999419	0.963474124	0.650764559	-1.18442457	0.485426827
Araip.73ZM2	-0.05889369	-0.43440282	-1.08926734	1.992768431	1.847996907
Araip.7408P	2.097610797	2.548436625	2.313245852	2.931683057	3.169925001
Araip.74B2T	1.169925001	1.722466024	1.070389328	3.468583317	3.502075956
Araip.74F08	-4.64385619	-2.64385619	-3.18442457	3.399171094	3.412781525
Araip.74FNF	NA	NA	-6.64385619	NA	2.378511623
Araip.74IBX	3.531069493	3.670160514	3.698218478	2.321928095	3.099295204
Araip.74MNE	2.757023247	3.331991778	2.961623328	3.077242999	3.580145484
Araip.74PIQ	-1.94341647	-1.18442457	-2.83650127	1.195347598	1.655351829
Araip.7513V	2.283921772	2.655351829	2.333423734	2.965322548	3.304511042
Araip.75757	NA	NA	NA	NA	NA
Araip.758EY	NA	NA	NA	NA	NA
Araip.75CVM	0.659924558	0.40053793	0.773996325	-1.15200309	0.189033824
Araip.75DC1	2.969012308	2.657640005	2.370164281	2.748461233	2.201633861
Araip.75JSE	0.970853654	1.207892852	1.007195501	2.319039816	2.344828497
Araip.75MM4	3.140778656	3.83491343	3.288358562	4.201633861	4.635173947

Araip.76CRM	2.15704371	0.59454855	0.799087306	NA	NA
Araip.76E63	NA	NA	NA	NA	NA
Araip.76HFA	1.10433666	0.773996325	0.82374936	-0.07400058	0.613531653
Araip.76IDI	3.430285273	3.468583317	3.489286023	3.679198571	3.772941338
Araip.76JTT	-2.55639335	-2.25153877	-2.55639335	-2.47393119	11.21646093
Araip.76PXL	-2.64385619	-2.64385619	-3.47393119	-1.25153877	0.137503524
Araip.76R9S	-0.51457317	0	-0.4150375	1.189033824	1.550900665
Araip.76SLC	5.29130886	5.38024459	5.229972385	2.987320866	3.267535798
Araip.76TXX	1.778208576	2.007195501	1.811471031	2.659924558	2.847996907
Araip.76V1I	2.124328135	3.003602237	2.803227036	1.618238656	2.073820233
Araip.774UX	3.955126781	4.102658131	3.931683057	5.634593268	5.609695873
Araip.776JT	5.09000653	5.429615964	5.216066783	6.262658655	6.286696365
Araip.778BR	NA	-6.64385619	NA	-3.83650127	-2.32192809
Araip.77AM3	NA	NA	-5.64385619	NA	NA
Araip.77H8K	2.82374936	2.608809243	2.521050737	1.944858446	2.201633861
Araip.77JRH	3.021479727	3.125981654	4.001802243	1.214124805	1.974529312
Araip.77NJ9	3.317593505	3.232660757	3.092545742	2.327687364	2.513490746
Araip.77QS1	NA	NA	-6.64385619	NA	-6.64385619
Araip.781N3	6.114991613	5.752748591	5.82374936	2.438292852	2.232660757
Araip.7827F	NA	-6.64385619	NA	NA	-5.64385619
Araip.785V6	0.097610797	0.070389328	-0.577767	1.333423734	2.13093087
Araip.7869H	2	2.570462931	2.217230716	3.450221496	3.43162296

Araip.787IL	NA	NA	NA	NA	NA
Araip.788L8	NA	NA	NA	NA	3.514753498
Araip.78DPR	2.929790998	3.165107985	2.931683057	4.12763328	4.300123725
Araip.78FT4	NA	NA	NA	NA	NA
Araip.78H4F	-4.64385619	NA	NA	NA	-3.64385619
Araip.78HJ7	2.794935663	2.735522177	2.700439718	1.565597176	-0.32192809
Araip.78PTT	NA	NA	-6.64385619	2.025028794	2.873813198
Araip.78Q2V	0.298658316	0.084064265	-0.30400619	-0.07400058	0.333423734
Araip.78TK8	-0.26881676	0.056583528	-0.94341647	3.757023247	3.548436625
Araip.78XNL	NA	-5.64385619	NA	NA	-4.64385619
Araip.7942Z	-2.55639335	-2.18442457	-3.47393119	-0.94341647	-1.02914635
Araip.7944J	-0.71311885	-0.20091269	-1.28630419	1.565597176	1.835924074
Araip.798H5	6.695576306	6.948133578	6.970508581	4.062639828	5.581953751
Araip.79B36	3.532316959	3.792855352	3.458119481	3.808385051	4.224966365
Araip.79CH7	1.459431619	1.627606838	1.516015147	1.782408565	1.914564523
Araip.79E4D	-1.78587519	-2.47393119	-2.47393119	-1	-1.59946207
Araip.79FKI	1.03562391	1.604071324	1.189033824	2.419538892	2.580145484
Araip.79KSY	2.811471031	3.15704371	2.521050737	0.807354922	1.077242999
Araip.79M9T	0.584962501	0.604071324	0.263034406	1.659924558	2.03562391
Araip.79PEZ	0.713695815	1.618238656	0.678071905	2.397802962	2.319039816
Araip.79R74	3.71918344	3.56924803	3.61117238	3.592158002	3.910732662
Araip.79RU1	1.786596362	1.3950628	1.124328135	-0.10469738	0.286881148

Araip.79TDF	-1	-1.32192809	-1.21759144	-1.73696559	-0.64385619
Araip.79WPQ	3.572889668	4.977737849	5.50747753	5.799087306	5.409051439
Araip.7A0S5	3.070389328	3.606442228	3.288358562	3.799087306	3.693765712
Araip.7A4AR	-6.64385619	-5.64385619	NA	-4.05889369	-2
Araip.7A7AE	0.214124805	0.298658316	-0.13606155	1.555816155	2.693765712
Araip.7A9DT	-3.32192809	-5.05889369	-4.05889369	-2.39592868	-2.83650127
Araip.7A9UM	2.625270489	2.195347598	2.498250868	6.297558281	6.187253086
Araip.7AQ3E	4.277240898	4.763411574	4.86393845	4.532316959	4.589164237
Araip.7B0U4	0.887525271	1.500802053	-0.30400619	3.691534165	4.340562269
Araip.7B1XA	-5.64385619	-5.64385619	NA	-5.64385619	-6.64385619
Araip.7B3CC	1.981852653	1.875780063	1.90303827	1.097610797	1.411426246
Araip.7BB5W	NA	-6.64385619	NA	NA	0.250961574
Araip.7BF1X	4.092545742	3.595742339	3.935459748	0.659924558	1.310340121
Araip.7C03S	6.317412614	6.160678213	6.469234794	2.687060688	2.307428525
Araip.7C4C2	1.257010618	1.545968369	2.042644337	-2	-0.97143085
Araip.7C73C	2.327687364	2.64385619	2.295723025	2.786596362	3.049630768
Araip.7D071	NA	-6.64385619	NA	NA	NA
Araip.7D3J7	1.384049807	1.063502942	1.077242999	1.807354922	2.176322773
Araip.7D543	5.6114675	5.553360503	5.593054922	4.287620044	4.313245852
Araip.7D5HE	NA	NA	-6.64385619	-3.18442457	-1.43440282
Araip.7D96E	-3.18442457	-2.55639335	-2.25153877	1.604071324	0.765534746
Araip.7D99U	-1.15200309	-0.83650127	-1.28630419	0.070389328	0.757023247

Araip.7DQ34	NA	NA	NA	NA	NA
Araip.7E4DK	-0.23446525	0.189033824	-0.71311885	1.40053793	2.204766751
Araip.7E55M	NA	-5.05889369	NA	NA	-3.32192809
Araip.7E6JE	-1.15200309	-0.577767	-1	-1.88896869	-1.25153877
Araip.7E7XY	-0.45403163	0.333423734	-0.55639335	2.211012193	2.060047384
Araip.7E8G5	-3.64385619	-4.32192809	-3.47393119	-1.88896869	0.070389328
Araip.7EC8V	1.056583528	1.207892852	1.049630768	2.427606173	2.797012978
Araip.7EF18	1.459431619	3.237257771	3.037382222	5.734167661	4.632268215
Araip.7EN7C	4.209453366	4.254745198	4.388189537	3.452858965	3.646162657
Araip.7ER0N	NA	NA	NA	NA	-6.64385619
Araip.7EU1J	-0.64385619	-0.16812276	-1.39592868	2.386810946	2.707082992
Araip.7F16U	2.217230716	2.653060017	2.381283373	3.218781168	3.578938713
Araip.7F1V2	1.906890596	2.430285273	2.13422094	2.869871406	3.47118746
Araip.7F2XC	0.782408565	0.847996907	0.250961574	3.263034406	3.537296067
Araip.7FJ61	2.608809243	2.339137385	2.232660757	-1.51457317	-0.21759144
Araip.7G11P	4.438958148	5.063934306	4.872336288	3.862947248	4.402585758
Araip.7G39W	3.269033146	4.078097423	4.09592442	4.605849867	4.43496176
Araip.7G62B	-2.39592868	-0.94341647	-1.94341647	0.367371066	0.59454855
Araip.7G8YS	0.056583528	0.722466024	0.097610797	2.629939409	2.435628594
Araip.7G9YB	2.790772038	2.691534165	1.948600847	3.560714954	3.898208353
Araip.7H0TY	-4.32192809	-3.47393119	-3.64385619	-2.94341647	-2.25153877
Araip.7H21U	-0.49410907	-0.4150375	-1.47393119	1.097610797	0.641546029
Araip.7H2KA	0.871843649	0.98550043	0.722466024	1.687060688	1.541019153

Araip.7H6FH	3.75595577	3.904965719	3.646162657	2.750606505	2.883620816
Araip.7HD76	-0.64385619	-0.21759144	-0.45403163	0.59454855	0.970853654
Araip.7HD9S	NA	-2.94341647	-2.94341647	1.169925001	1.214124805
Araip.7HP1R	-0.49410907	5.214124805	3.237257771	3.193771743	2.451540833
Araip.7I1D0	-5.05889369	-3.47393119	-4.64385619	-0.49410907	-1.73696559
Araip.7I2ZN	2	2.715893371	2.295723025	2.853995647	3.077242999
Araip.7IA97	0.344828497	0.097610797	0.097610797	0.807354922	0.378511623
Araip.7IS5A	5.241840184	5.617651119	5.604664415	5.464668267	5.103917211
Araip.7IS7K	2.070389328	0.042644337	0.344828497	1.189033824	4.015247774
Araip.7J81A	-1.02914635	-1.08926734	-1.12029423	-4.64385619	-5.64385619
Araip.7JC2C	0.298658316	0.333423734	-0.47393119	5.963705307	5.557961427
Araip.7JC4X	3.949534933	3.906890596	4.229587923	3.326249701	4.589763487
Araip.7JE55	0.454175893	0.799087306	0.687060688	0.669026766	0.97819563
Araip.7JJ4U	-5.64385619	NA	-5.64385619	-1.12029423	-2.73696559
Araip.7JN11	1.937344392	1.922197848	1.811471031	2.50589093	1.992768431
Araip.7K2B7	-0.01449957	0.555816155	0.150559677	0.98550043	1.422233001
Araip.7KS0U	4.713145902	4.304511042	4.670160514	1.049630768	1.992768431
Araip.7KT8S	4.327687364	4.599317794	4.498889087	5.140778656	5.111031312
Araip.7KZ5D	NA	-5.64385619	-6.64385619	NA	-1.28630419
Araip.7L3C8	NA	NA	NA	NA	NA
Araip.7L4MP	0.286881148	0.695993813	0.871843649	-0.26881676	-0.28630419
Araip.7L4UM	2.837943242	2.518535139	2.592158002	1.510961919	2.253989266

Araip.7L5BF	NA	-4.32192809	-6.64385619	-5.64385619	-1.94341647
Araip.7L870	NA	NA	NA	0.214124805	-0.91593574
Araip.7LD9B	1.700439718	1.937344392	1.765534746	2.229587923	2.253989266
Araip.7LQ31	2.403267722	2.289834465	2.201633861	2.82374936	3.0721058
Araip.7M8Z5	NA	NA	NA	NA	NA
Araip.7MD3E	1.550900665	1.427606173	1.40053793	2.910732662	3.039138394
Araip.7MI21	NA	NA	NA	NA	0.790772038
Araip.7MJ2M	2.678071905	2.565597176	2.257010618	3.343407822	3.361768359
Araip.7MS83	3.632268215	3.073820233	3.258518925	2.22650853	2.13422094
Araip.7MT36	NA	-6.64385619	-6.64385619	-6.64385619	2.063502942
Araip.7MX4C	-2.73696559	-1.83650127	-2.94341647	-0.15200309	0.056583528
Araip.7N26D	-1.35845397	-1.43440282	-2.73696559	1.555816155	1.459431619
Araip.7N2TS	2.63691458	2.244887059	2.456806149	-1.64385619	0.275007047
Araip.7N6M4	2.277984747	2.217230716	1.722466024	2.867896464	3.007195501
Araip.7N70L	-0.35845397	1.063502942	0.176322773	1.111031312	0.879705766
Araip.7N7X7	1.910732662	1.516015147	1.531069493	1.416839742	1.97819563
Araip.7NS74	1.03562391	1.618238656	1.432959407	1.627606838	2.014355293
Araip.7P115	-4.64385619	-2.25153877	-2.94341647	-3.83650127	-1.02914635
Araip.7P2V7	2.283921772	1.678071905	2.244887059	-3.64385619	-3.18442457
Araip.7P4BA	1.485426827	2.137503524	1.794935663	2.606442228	3.012568674
Araip.7P4U9	2.935459748	2.629939409	3.09423607	2.010779839	2.957914599
Araip.7P6A7	-2.83650127	-1.59946207	-1.83650127	1.10433666	1.150559677

Araip.7P78Q	NA	NA	-6.64385619	NA	NA
Araip.7PD3K	NA	-6.64385619	NA	NA	-5.05889369
Araip.7PD4P	-0.02914635	0.298658316	-0.577767	4.39848719	3.961623328
Araip.7PF1U	-0.83650127	-0.23446525	-0.51457317	-0.35845397	-0.12029423
Araip.7QP65	1.269033146	2.367371066	1.867896464	1.855989697	2.897240426
Araip.7QP8H	-0.577767	-0.53533173	-0.59946207	-1.94341647	-1.15200309
Araip.7QT5G	4.251719093	3.339137385	3.731183242	2.739848103	3.361768359
Araip.7QY88	NA	NA	NA	NA	0.731183242
Araip.7R33Q	1.89917563	1.799087306	1.839959587	1.480265122	1.739848103
Araip.7RD3Q	4.390254956	4.43162296	4.498250868	2.23572706	2.625270489
Araip.7RJ08	3.916476644	4.163498732	4.137503524	4.703765179	5.292413668
Araip.7RK50	4.0721058	4.255500733	4.479618608	7.521678952	9.139193192
Araip.7RN1R	NA	NA	NA	NA	NA
Araip.7S61E	-2.94341647	-2.94341647	-5.05889369	NA	-3.47393119
Araip.7TY5U	5.133810091	5.369117459	5.341274184	5.263410059	5.579542225
Araip.7U0RM	2.792855352	3.363171077	3.122672719	3.255500733	3.917431755
Araip.7UJ37	0.632268215	1	0.555816155	1.906890596	1.843983844
Araip.7UJ60	1.996388746	2.381283373	2.277984747	2.805292456	3.122672719
Araip.7V77F	3.742006211	3.801158656	3.700439718	3.10433666	3.509695842
Araip.7V7BF	2.531069493	2.859969548	2.629939409	3.388189537	3.510961919
Araip.7V9BH	-1.55639335	-1.83650127	-1.55639335	-2.47393119	-1.32192809
Araip.7VZ5Q	1.925999419	2.498250868	2.333423734	1.855989697	1.531069493

Araip.7W817	-0.34007544	0.722466024	0.344828497	2.09423607	2.427606173
Araip.7W8RG	2.867896464	3.234194723	3.049630768	3.47118746	3.862947248
Araip.7WS9N	-2.18442457	-1.32192809	-1.21759144	-1.64385619	-1.02914635
Araip.7WV1U	4.85698569	5.187846909	5.069100632	5.593951284	5.663344619
Araip.7X70M	1.339137385	1.765534746	1.49057013	1.627606838	1.700439718
Araip.7Y0PA	2.182692298	2.3950628	2.09423607	2.13422094	2.528571319
Araip.7Y21E	0.378511623	0.575312331	-0.16812276	0.807354922	0.650764559
Araip.7Y7KI	0.526068812	0.454175893	0.344828497	0.50589093	0.933572638
Araip.7YJ6V	2.269033146	2.310340121	2.521050737	1.469885976	2.301587647
Araip.7Z8D2	1.778208576	2.87774425	2.257010618	2.347665656	2.147306699
Araip.802QV	1.207892852	1.550900665	1.580145484	0.23878686	0.839959587
Araip.8074I	4.084064265	4.159467729	3.59812696	4.181897643	4.316145742
Araip.807VL	2.15704371	2.358958826	2.053111336	1.214124805	0.613531653
Araip.80D4Q	0.263034406	0.584962501	0.042644337	0.575312331	0.948600847
Araip.80E2A	0.613531653	1.014355293	1.150559677	1.344828497	1.967168608
Araip.80FVV	-1.64385619	-1.88896869	0.214124805	1.691534165	2.801158656
Araip.80KYM	3.553360503	4.300855871	3.384049807	5.292781749	5.254745198
Araip.80R5B	2.166715445	2.604071324	2.295723025	1.895302621	1.910732662
Araip.80WHH	2.347665656	2.021479727	1.815575429	1.678071905	1.632268215
Araip.80ZQA	2.257010618	2.577730931	2.289834465	3.840966704	3.771885579
Araip.81209	1.669026766	1.275007047	1.40599236	1.40599236	1.782408565
Araip.812WS	-1.47393119	0.286881148	1.084064265	-2.55639335	-1.94341647

Araip.8133V	1.655351829	1.944858446	1.769771739	0.485426827	0.042644337
Araip.819C3	-2.55639335	-2.64385619	-2.25153877	2.014355293	2.039138394
Araip.81A8L	2.72900887	2.560714954	3.13093087	1.963474124	2.904965719
Araip.81EVU	2.523561956	2.910732662	2.35614381	2.82374936	3.061776198
Araip.81HNQ	0.948600847	3.6622055	2.748461233	3.097610797	3.070389328
Araip.81IM1	-2	-1.32192809	-1.59946207	-0.51457317	0.659924558
Araip.81PGB	6.362469889	7.089265084	6.418358635	8.460660665	7.870611313
Araip.81TFR	2.316145742	2.053111336	2.025028794	1.03562391	2.298658316
Araip.8206I	0.333423734	0.201633861	0.389566812	-0.34007544	0.137503524
Araip.826U2	-5.64385619	-1.25153877	-1.51457317	0.097610797	1.13093087
Araip.8276P	5.198101209	5.116863758	4.891419187	5.462706751	5.451540833
Araip.82B50	1.176322773	1.286881148	1.124328135	0.799087306	1.599317794
Araip.82GLE	-0.35845397	0.056583528	-1.43440282	2.229587923	2.963474124
Araip.82HK7	1.350497247	0.722466024	1.163498732	-3.18442457	-1.32192809
Araip.82I4N	1.555816155	2.073820233	1.786596362	2.924099886	3.003602237
Araip.82QS5	4.189033824	4.052241981	4.049630768	1.815575429	2.17951105
Araip.82VVP	-5.05889369	0.201633861	0.678071905	-4.05889369	0.35614381
Araip.82XGB	3.163498732	3.944858446	3.577730931	5.173926932	5.8008999
Araip.83671	3.019701914	3.329123596	3.232660757	2.582556003	3.010779839
Araip.8395H	2.372952098	2.516015147	2.62058641	1.82374936	2.080657663
Araip.83CVJ	0.189033824	-0.12029423	-0.62148838	-0.23446525	2.9202933
Araip.83FL0	1.042644337	1.263034406	0.855989697	2.713695815	3.023255352

Araip.83IVK	2.618238656	1.608809243	1.974529312	1.361768359	2.063502942
Araip.83LYV	-3.64385619	-3.05889369	-2.94341647	0.028569152	0.214124805
Araip.83N9U	-4.64385619	NA	NA	-1.21759144	-1.83650127
Araip.83S01	-0.4150375	-2.83650127	-3.32192809	-5.64385619	-2.47393119
[truncated: 337,080 more chars]
